# Comprehensive Expression Profiling of Tumor Cell Lines Identifies Molecular Signatures of Melanoma Progression

**DOI:** 10.1371/journal.pone.0000594

**Published:** 2007-07-04

**Authors:** Byungwoo Ryu, Dave S. Kim, Amena M. DeLuca, Rhoda M. Alani

**Affiliations:** The Sidney Kimmel Comprehensive Cancer Center at Johns Hopkins, Department of Oncology, Johns Hopkins University School of Medicine, Baltimore, Maryland, United States of America; City of Hope Medical Center, United States of America

## Abstract

**Background:**

Gene expression profiling has revolutionized our ability to molecularly classify primary human tumors and significantly enhanced the development of novel tumor markers and therapies; however, progress in the diagnosis and treatment of melanoma over the past 3 decades has been limited, and there is currently no approved therapy that significantly extends lifespan in patients with advanced disease. Profiling studies of melanoma to date have been inconsistent due to the heterogeneous nature of this malignancy and the limited availability of informative tissue specimens from early stages of disease.

**Methodology/Principle Findings:**

In order to gain an improved understanding of the molecular basis of melanoma progression, we have compared gene expression profiles from a series of melanoma cell lines representing discrete stages of malignant progression that recapitulate critical characteristics of the primary lesions from which they were derived. Here we describe the unsupervised hierarchical clustering of profiling data from melanoma cell lines and melanocytes. This clustering identifies two distinctive molecular subclasses of melanoma segregating aggressive metastatic tumor cell lines from less-aggressive primary tumor cell lines. Further analysis of expression signatures associated with melanoma progression using functional annotations categorized these transcripts into three classes of genes: 1) Upregulation of activators of cell cycle progression, DNA replication and repair (*CDCA2, NCAPH, NCAPG, NCAPG2, PBK, NUSAP1, BIRC5, ESCO2, HELLS, MELK, GINS1, GINS4, RAD54L, TYMS*, and *DHFR*), 2) Loss of genes associated with cellular adhesion and melanocyte differentiation (*CDH3, CDH1, c-KIT, PAX3, CITED1/MSG-1, TYR, MELANA, MC1R,* and *OCA2)*, 3) Upregulation of genes associated with resistance to apoptosis (BIRC5/survivin). While these broad classes of transcripts have previously been implicated in the progression of melanoma and other malignancies, the specific genes identified within each class of transcripts are novel. In addition, the transcription factor NF-KB was specifically identified as being a potential “master regulator” of melanoma invasion since NF-KB binding sites were identified as consistent consensus sequences within promoters of progression-associated genes.

**Conclusions/Significance:**

We conclude that tumor cell lines are a valuable resource for the early identification of gene signatures associated with malignant progression in tumors with significant heterogeneity like melanoma. We further conclude that the development of novel data reduction algorithms for analysis of microarray studies is critical to allow for optimized mining of important, clinically-relevant datasets. It is expected that subsequent validation studies in primary human tissues using such an approach will lead to more rapid translation of such studies to the identification of novel tumor biomarkers and therapeutic targets.

## Introduction

The incidence of melanoma is increasing at one of the highest rates for any form of cancer in the United States [1]. At present, there are no systemic agents available that significantly extend the lifespan of patients with advanced disease, and the key to improved survival in all affected individuals remains early diagnosis and treatment. While early stage disease may result in occasional deaths, there are no available tests to predict which early stage tumors have a high likelihood of progression and therefore a worse prognosis. Thus, an urgent need exists for the identification of molecular signatures of melanoma progression which can be used to develop accurate prognostic markers and effective targeted therapies. High-throughput gene expression profiling technologies offer an opportunity to uncover critical molecular events in the development and progression of human melanoma and can be used to design improved prognostic testing and effective treatment strategies. Previous transcriptome analyses in other malignancies have provided valuable information for the assessment of patient group classifications such as subgroups of patients that are likely to respond to a particular therapy [2]. Expression profiling of metastatic melanomas was able to identify previously unrecognized subtypes of disease and predict phenotypic characteristics which may be of importance to melanoma progression [3]. Further studies using serial analysis of gene expression (SAGE) and cDNA arrays have yielded the identification of additional novel molecules and pathways which may be involved in melanoma development[4]–[7]. Such studies have been limited in utility due to the lack of concordance from one study to the next suggesting tumor heterogeneity [8]. In addition, the limited availability of primary tissue from early stages of disease has hindered the ability to identify serial molecular events that lead to melanoma onset. This shortcoming of tissue availability has largely restricted gene expression profiling studies in melanoma to the use of small numbers of established tumor cell lines and cases of metastatic disease.

Previous studies of primary human melanomas have identified gene signatures associated with tumor progression [9]–[11]. These signatures included upregulation of cell cycle regulatory proteins, mitotic checkpoint genes, genes involved in DNA replication and repair, and cellular stress response genes in addition to loss of genes promoting apoptosis; however, few of the genes identified in these studies were concordant suggesting limitations due to tumor variability. Since better knowledge of gene expression signatures associated with melanoma progression may identify improved screening tools and therapeutic strategies, we used high density cDNA microarrays for gene expression profiling of genetically well-defined melanoma cell lines isolated from distinctive stages of tumor progression. Novel data reduction algorithms were used to identify gene signatures associated with tumor invasion and metastasis. Here we report unique sets of gene expression signatures that are associated with melanoma progression. Many of these pathways have previously been implicated in melanoma progression; however, the specific signature genes identified are novel. These particular progression-associated genes may reflect the underlying molecular mechanisms of the various phases in the known tumor progression pathways of melanoma. As such, the pathways and molecules identified in this study have the potential to be utilized as therapeutic targets for melanoma as well as novel molecular markers for melanoma progression. Moreover, these studies support the use of renewable sources of tumor cells, such as informative tumor cell lines, for the early identification of genes associated with malignant progression which can be subsequently validated using more precious primary tissue specimens.

## Results

In order to define gene expression patterns during the course of melanoma development and progression, we evaluated a series of primary and metastatic melanoma cells derived from lesions of discrete phases of melanoma progression as well as pools of primary human melanocytes. Tumor cell lines derived from three radial growth phase (RGP) melanomas (WM35, SBC12, and WM1552C), four vertical growth phase (VGP) melanomas (WM902B, WM278, WM983A, and WM793), and three metastatic melanomas (WM852, WM983B, 1205Lu) were evaluated. These cell lines possess a notable ability to recapitulate the clinical stages of disease from which they were derived [12], [13] and have been characterized with respect to tumorigenicity and metastasis [14]–[17]; cellular growth characteristics including life span, growth factor dependency, anchorage-independent growth [12]; and pigmentation and morphology [18]. In addition, cytogenetic analyses in these cell lines including non-random abnormalities such as deletions, translocations, and amplifications have been well-documented and suggest high relevance to the primary tumor of origin (reviewed in [12]).

**Figure 1:**
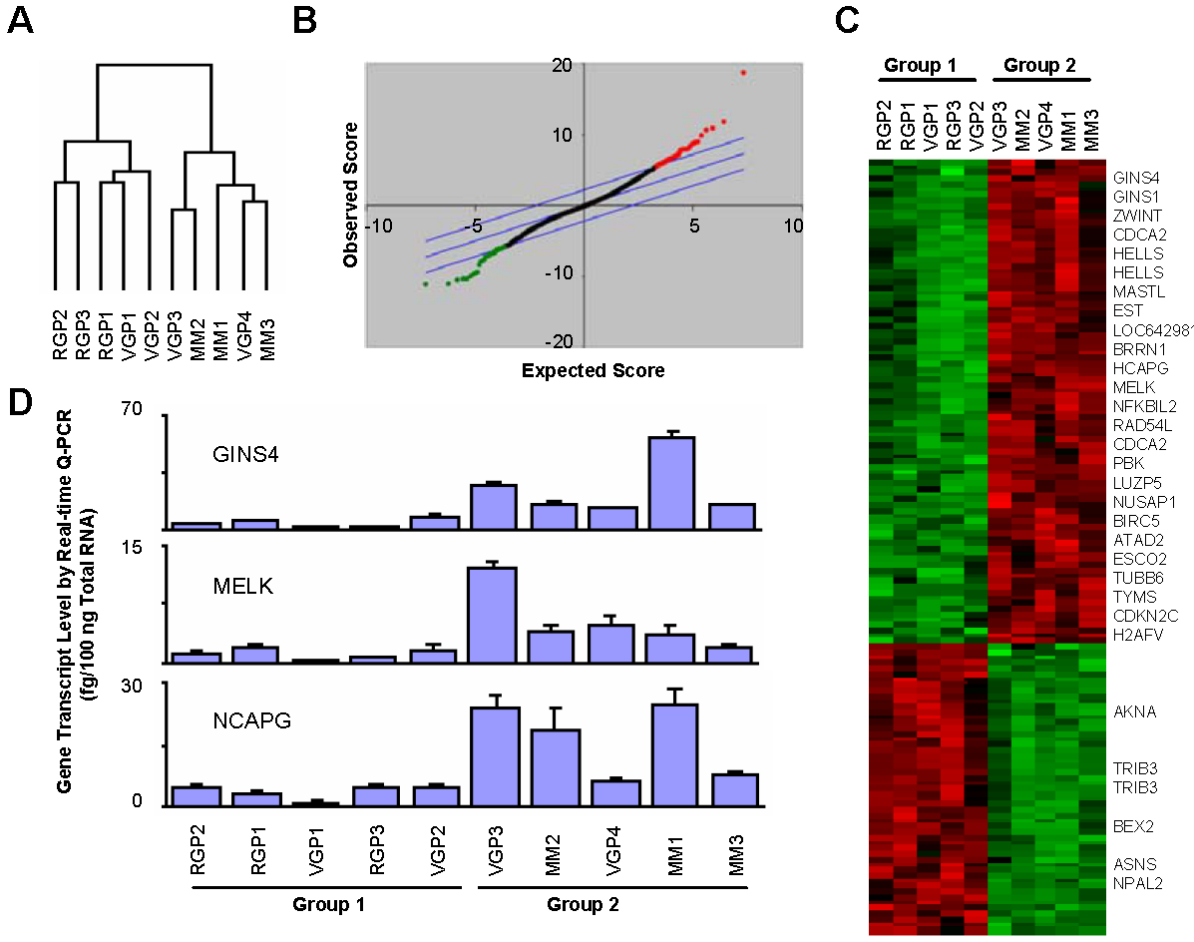
Evaluation of gene expression profiles from melanoma cells lines of varying stages of progression identifies a signature for aggressive melanomas. A) Unsupervised hierarchical clustering of melanoma cells indicates the existence of two distinct groups of melanoma cells based on global gene expression patterns (Group 1: RGP2, RGP3, RGP1, VGP1, and VGP2; Group 2: VGP3, MM2, MM1, VGP4, and MM3). B) SAM plot sheet illustrating a signature for differentially expressed genes in aggressive melanomas. Gene expression profiles from the two groups of melanomas were compared (Group1 vs. Group 2) and a differentially expressed gene signature was identified by SAM. Red and green dots represent gene probesets upregulated and downregulated respectively in Group 2. C) The melanoma gene signature was visualized using Java TreeView. Genes over four-fold differentially expressed are indicated on the right side of the image. D) Validation of select differentially expressed genes by real-time RT-PCR. Three genes upregulated in aggressive melanomas (Group 2) were selected for analysis and their differential expression was verified. 3.0 µg of total RNA was subjected to cDNA synthesis reaction as described in the materials and methods. 1.0 µl of the final cDNA samples (100 µl) were used for real-time Q-PCR reaction. For the measurement of gene transcript level, standard curves were generated for each gene using known amount of PCR amplified product from the corresponding genes. Error bars are SD of three independent experiments.

Global gene expression patterns were obtained using Affymetrix gene chips and comparison of gene expression profiles was performed using hierarchical clustering analysis. This clustering analysis identified two distinct groups of melanoma cell lines based on the similarity of their expression patterns, separating radial growth phase (RGP) and metastatic melanomas (MM) (Figure 1); however, vertical growth phase (VGP) melanomas failed to form a distinctive cluster (Figure 1A). The first group, which we characterized as “less-aggressive” primary melanomas (designated as Group1), included all three RGP melanomas (WM35, Sbcl2, and WM1552C) and two VGP melanomas (WM902B and WM278). The second group, which we characterized as “more-aggressive” melanomas (designated as Group2), included all three metastatic melanomas (WM853, WM983B, and 1205Lu) and two VGP melanomas (WM983B and WM793). Additional cluster analyses with different linkage matrices produced similar results (data not shown). Of note, only 2 of the 10 cell lines (Sbcl2 and WM853) were found to be wildtype for BRAF kinase and this genotype failed to demonstrate a notable cluster in our hierarchical analyses.

In order to identify a cohort of genes differentially expressed between our defined groups of melanomas, the gene expression array dataset was subjected to the microarray data analysis program Significance Analysis of Microarray (SAM) [19]. This analysis resulted in 142 differentially expressed probesets with a 3.5 % false discovery rate (Figure 1B and C). In total, 89 probe sets representing 65 well-defined genes were found to be upregulated in more-aggressive (group 2) versus less-aggressive (group 1) melanomas and 53 probe sets representing 37 well-defined genes were found to be downregulated (Table S1). When more stringent criteria were applied (well-characterized genes which are differentially expressed greater than 4-fold) to this signature, we identified 21 upregulated and 5 downregulated genes in the more-aggressive melanoma cells (Figure 1C). Of note, the set of genes highly expressed in more-aggressive melanomas includes many novel genes with reported functional roles in cell cycle regulation and proliferation such as *ZWINT, CDCA2, NCAPH, NCAPG, NCAPG2, PBK, NUSAP1, BIRC5, ESCO2, HELLS, MELK, and CDKN2C* [20]–[31] as well as genes that are involved DNA replication and repair processes including *GINS1, GINS4, RAD54L, TYMS*, and *DHFR* [32]–[34] (Table 1). Differential expression of these genes was validated by quantitative real-time RT-PCR (Figure 1D).

**Table 1:** Genes with altered expression in aggressive melanoma cells are involved in cell cycle control, cell proliferation, DNA repair and replication^a^.

Probe Set ID	Gene Title	Gene Symbol	Fold b	Function
227350_at	Helicase, lymphoid-specific	HELLS^c^	8.5	cell proliferation
202589_at	thymidylate synthetase	TYMS	7.2	DNA replication, DNA repair
204558_at	RAD54-like (S. cerevisiae)	RAD54L	6.2	DNA repair, response to DNA damage
204825_at	maternal embryonic leucine zipper kinase	MELK	5.9	Mitosis, protein phosphorylation
204159_at	cyclin-dependent kinase inhibitor 2C (p18)	CDKN2C	5.8	cell cycle
212949_at	barren homolog 1 (Drosophila)	NCAPH	5.8	cell cycle, chromosome condensation
219588_s_at	leucine zipper protein 5	NCAPG2	4.6	cell cycle, chromosome condensation
218663_at	chromosome condensation protein G	HCAPG1	4.5	cell cycle, chromosome condensation
211767_at	GINS complex subunit 4 (Sld5 homolog)	GINS4	5.6	DNA replication, cell proliferation
206102_at	GINS complex subunit 1 (Psf1 homolog)	GINS1	4.7	DNA replication, cell proliferation
202095_s_at	baculoviral IAP repeat-containing 5	BIRC5	5.1	G2/M transition cell cycle
219148_at	PDZ binding kinase	PBK	5.1	mitosis, protein phosphorylation
235178_x_at	establishment of cohesion 1 homolog 2	ESCO2	5.1	cell cycle
226661_at	cell division cycle associated 2	CDCA2^c^	4.9	cell cycle
204026_s_at	ZW10 interactor	ZWINT	4.7	cell cycle, spindle organization
202534_x_at	dihydrofolate reductase	DHFR	4.5	DNA replication, nucleotide metab olism
218039_at	nucleolar and spindle associated protein 1	NUSAP1	4.1	cell cycle, chromosome condensation
1555788_a_at	tribbles homolog 3 (Drosophila)	TRIB3^c^	−6.7	anti-proliferation, apoptosis

a Genes with greater than four-fold differential expression are shown.

b Fold represents average expression ratio of group 2 over Group1.

c Genes with multiple probesets are shown with data from a single representative probeset.

**Figure 2:**
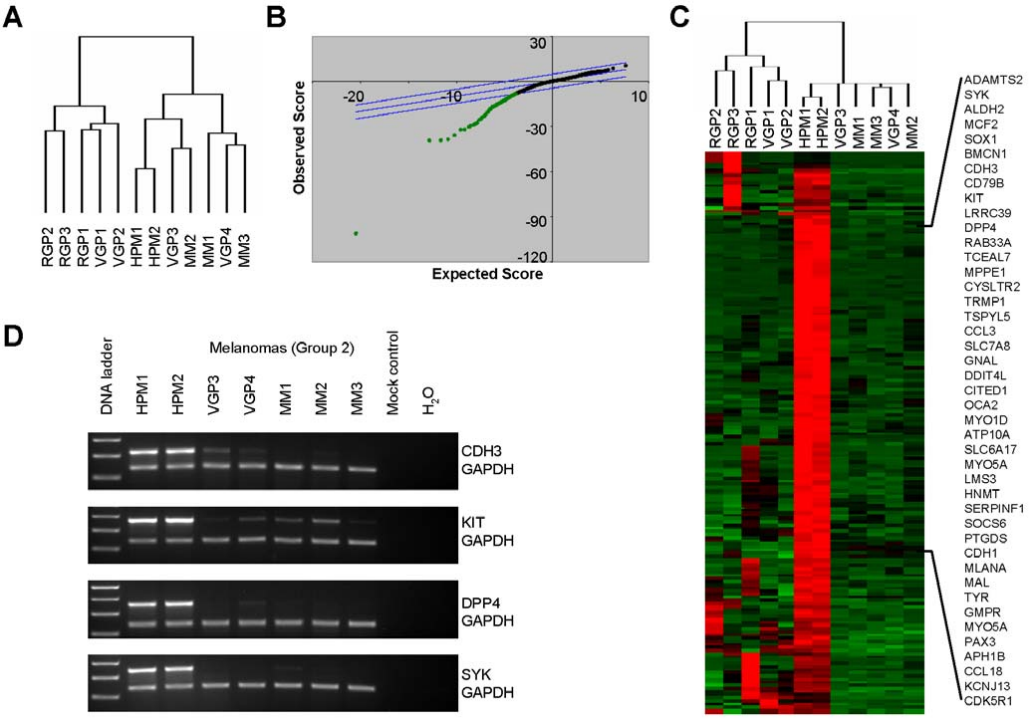
Evaluation of differential gene expression from aggressive melanomas (Group 2) vs. primary human melanocytes identifies a signature characterized by loss of differentiation-associated genes. A) Java TreeView analysis of melanoma cell lines and primary human melanocytes clusters two pools of human primary melanocytes (HPM1 and HPM2) with the Group 2 melanomas. B) SAM plot sheet illustrating a signature of down-regulated genes in group 2 melanomas compared to HPMs. Gene expression profiles of two pools of human primary melanocytes (HPM1 and HPM2) were compared to those of aggressive melanomas (Group 2) and a differentially expressed gene signature was identified by SAM. C) The melanoma gene signature was visualized using Java TreeView. Genes over five-fold downregulated are indicated on the right. D) Validation of differential expression for selected genes by semi-quantitative duplex RT-PCR. Four genes (CDH3, KIT, DPP4, SYK) downregulated in the aggressive melanoma cells (Group 2) were selected for analysis and their differential expression was verified.

Since we were interested in defining melanoma progression signatures, and all melanomas are initiated in primary human melanocytes, we evaluated our expression profiling data in the context of cultured neonatal primary human melanocytes (Figure 2). Surprisingly, when two pools of short-term cultured primary human melanocytes (HPM1 and HPM2) were included in the previously employed hierarchical clustering protocol, the global gene expression pattern of the normal melanocytes was found to be more similar to that of the more-aggressive melanomas (Group 2) than the less-aggressive melanomas (Group 1) (Figure 2A). Since early cultures of primary human melanocytes derived from neonatal foreskins divide rapidly yet possess a normal differentiation program, we reasoned that the similarities of these cells to more aggressive tumors was likely due to their proliferative potential. In order to test this hypothesis we compared gene expression profiles of more-aggressive melanoma cells (Group 2) to those of short-term cultured primary human melanocytes. Expression profiles were subjected to SAM analysis which identified a cohort of differentially expressed genes with a 0.85% false discovery rate. Remarkably, all differentially expressed genes were found to be down-regulated genes in aggressive melanoma cells versus primary human melanocytes suggesting that loss of specific gene signatures may be a key event in the development of advanced melanomas (Figure 2B). Further assessment of all melanoma expression profiles using TreeView revealed that the majority of these melanoma-associated genes are also down-regulated in the less-aggressive primary melanomas (Group1) (Figure 2C). This gene signature is comprised of critical mediators of cellular adhesion and melanocyte development and differentiation and includes: *CDH3, CDH1, c-KIT, PAX3, CITED1/MSG-1, TYR, MELANA, MC1R,* and *OCA2* [35]–[42] (Table 2). While such a loss of cellular adhesion by E-cadherin and P-cadherin has been extensively documented in melanoma (reviewed in [43]), and the loss of differentiation-associated genes is not wholly surprising, this signature does notably identify specific defects in the intrinsic melanocyte development program that may contribute to melanoma development. In addition, genes with tumor suppressor and metastasis suppressor functions (DPP4, SYK) are included in this melanoma signature [44], [45]. Significant down-regulation of these genes in the aggressive metastatic melanoma cells was validated using semi-quantitative duplex RT-PCR (Figure 2D). Furthermore, this differentially expressed “melanoma signature” contains many genes whose functional roles in melanoma progression have not been well characterized and may provide novel insights into the early development of melanoma from primary melanocytes.

**Table 2:** Differential expression of genes that are downregulated in aggressive melanoma cells (Group2) compared to primary human melanocytes. Genes with greater than five-fold differential expression are shown a.

Probe Set ID	Gene Title	Gene Symbol	Fold b	Function
203256_at	cadherin 3, type 1, P-cadherin (placental)	CDH3	−161.9	cell adhesion
201131_s_at	cadherin 1, type 1, E-cadherin (epithelial)	CDH1^c^	−35.1	cell adhesion
205051_s_at	v-kit viral oncogenehomolog	KIT	−94.0	signal transduction
1565162_s_at	microsomal glutathione S-transferase 1	MGST1^c^	−92.5	—
203716_s_at	dipeptidyl-peptidase 4 (CD26)	DPP4^c^	−74.0	proteolysis
206039_at	RAB33A, member RAS oncogene family	RAB33A	−70.4	signal transduction
226311_at	CDNA clone IMAGE: 30924414	—	−64.3	—
236901_at	ADAM metallopeptidase	ADAMTS2	−62.1	proteolysis
229095_s_at	similar to LIM	LOC440895	−54.6	—
227705_at	transcription elongation factor A (SII)-like 7	TCEAL7	−49.1	—
1556698_a_at	hypothetical protein LOC285513	LOC285513	−35.0	—
213924_at	metallophosphoesterase 1	MPPE1	−33.5	—
209569_x_at	DNA segment on chromosome 4	D4S234E^c^	−31.6	dopamine receptor signaling
202283_at	serpin peptidase inhibitor, clade F, member 1	SERPINF1	−29.2	development/proliferation
204570_at	cytochrome c oxidase subunit VIIa polypeptide 1	COX7A1	−28.8	electron transport
202546_at	vesicle-associated membrane protein 8	VAMP8	−27.9	vesicle-mediated transport
208017_s_at	MCF.2 cell line derived transforming sequence	MCF2^c^	−27.8	intracellular signaling cascade
217248_s_at	solute carrier family 7, member 8	SLC7A8^c^	−27.1	amino acid transport
206479_at	transient receptor potential cation channel	TRPM1	−25.8	cation transport
205114_s_at	chemokine (C-C motif) ligand 3	CCL3	−25.6	chemotaxis/inflammatory
220813_at	cysteinyl leukotriene receptor 2	CYSLTR2	−25.3	signal transduction
206355_at	G protein, alpha activating activity polypeptide	GNAL^c^	−23.5	signal transduction
237472_at	SRY (sex determining region Y)-box 1	SOX1	−23.4	regulation of transcription
203815_at	glutathione S-transferase theta 1	GSTT1	−22.1	response to stress
212599_at	autism susceptibility candidate 2	AUTS2	−20.3	—
206426_at	melan-A	MLANA	−20.3	—
225407_at	myelin basic protein	MBP^c^	−19.7	CNS development
219436_s_at	endomucin	EMCN	−19.4	—
1556427_s_at	similar to hypothetical protein	LOC221091	−17.9	—
201425_at	aldehyde dehydrogenase 2 family (mitochondrial)	ALDH2	−17.8	carbohydrate metabolism
207144_s_at	Cbp/p300-interacting transactivator 1	CITED1	−17.7	regulation of transcription
212338_at	myosin ID	MYO1D	−17.7	—
219315_s_at	chromosome 16 open reading frame 30	C16orf30	−17.0	—
227532_at	leucine rich repeat containing 39	LRRC39	−16.5	—
206569_at	interleukin 24	IL24	−16.1	apoptosis/immune response
226068_at	spleen tyrosine kinase	SYK^c^	−15.9	signal transduction
213122_at	TSPY-like 5	TSPYL5	−15.8	nucleosome assembly
204187_at	guanosine monophosphate reductase	GMPR	−14.7	nucleic acid metabolism
204880_at	O-6-methylguanine-DNA methyltransferase	MGMT	−14.3	DNA repair
204777_s_at	mal, T-cell differentiation protein	MAL	−14.2	cell differentiation
1555505_a_at	tyrosinase (oculocutaneous albinism IA)	TYR	−13.6	melanin biosynthesis
226778_at	chromosome 8 open reading frame 42	C8orf42^c^	−13.1	—
225792_at	hook homolog 1 (Drosophila)	HOOK1	−12.5	cytoskeleton organization
209924_at	chemokine (C-C motif) ligand 18	CCL18	−11.8	chemotaxis/inflammatory
211427_s_at	potassium inwardly-rectifying channel,	KCNJ13	−11.7	ion transport
213556_at	similar to R28379-1	LOC390940	−11.7	—
240173_at	transcribed locus	—	−11.5	—
206498_at	oculocutaneous albinism II	OCA2	−11.4	eye pigment biosynthesis
241600_at	transcribed locus	—	−11.0	—
205297_s_at	CD79b molecule, immunoglobulin-associated	CD79B	−10.9	immune response
228256_s_at	erythrocyte membrane protein band 4.1 like 4A	EPB41L4A	−10.9	—
223693_s_at	hypothetical protein FLJ10324	FLJ10324	−10.3	signal transduction
243727_at	copine VIII	CPNE8	−10.1	—
232687_at	CDNA FLJ33091 fis, clone TRACH2000660	—	−9.9	—
232443_at	hypothetical gene supported by AF131741	LOC441052	−9.9	—
236377_at	transmembrane protein 132D	TMEM132D	−9.1	—
211748_x_at	prostaglandin D2 synthase 21kDa (brain)	PTGDS	−9.0	lipid biosynthesis
204112_s_at	histamine N-methyltransferase	HNMT	−8.7	respiratory gaseous exchange
228057_at	DNA-damage-inducible transcript 4-like	DDIT4L	−8.6	—
51158_at	hypothetical gene	LOC400451	−8.3	—
209550_at	necdin homolog (mouse)	NDN	−8.3	regulation of cell cycle
214255_at	ATPase, Class V, type 10A	ATP10A	−7.9	cation transport
1553485_at	hypothetical protein LOC151278	FLJ32447	−7.8	—
204273_at	endothelin receptor type B	EDNRB	−7.6	G-protein signaling
235758_at	paraneoplastic antigen like 6A	PNMA6A	−7.6	—
213816_s_at	met proto-oncogene	MET	−7.2	cell proliferation
227704_at	full-length cDNA clone CS0CAP008YI07	—	−7.1	—
224566_at	trophoblast-derived noncoding RNA	TncRNA	−6.9	—
207610_s_at	EGF-like module containing, mucin-like	EMR2	−6.8	signal transduction
232504_at	hypothetical protein LOC285628	LOC285628	−6.8	—
241966_at	myosin VA (heavy polypeptide 12, myoxin)	MYO5A^c^	−6.7	actin-based movement
229925_at	solute carrier family 6, member 17	SLC6A17	−6.6	neurotransmitter transport
229251_s_at	two pore segment channel 2	TPCN2^c^	−6.2	cation transport
216059_at	paired box gene 3 (Waardenburg syndrome 1)	PAX3^c^	−6.2	development
227949_at	phosphatase and actin regulator 3	PHACTR3	−5.7	—
209685_s_at	protein kinase C, beta 1	PRKCB1	−5.6	intracellular signaling cascade
204995_at	cyclin-dependent kinase 5, regulatory subunit 1	CDK5R1	−5.3	neuron differentiation
206020_at	suppressor of cytokine signaling 6	SOCS6	−5.0	regulation of cell growth
221036_s_at	anterior pharynx defective 1 homolog B	APH1B	−5.0	Notch signaling pathway
205458_at	melanocortin 1 receptor	MC1R	−5.0	G-protein signaling

a Genes with greater than five-fold differential expression are shown.

b Fold represents average expression ratio of aggressive metastatic melanoma samples (group2) over normal primary human melanocyte samples.

c Gene with multiple probesets are shown with a representative probeset.

**Figure 3:**
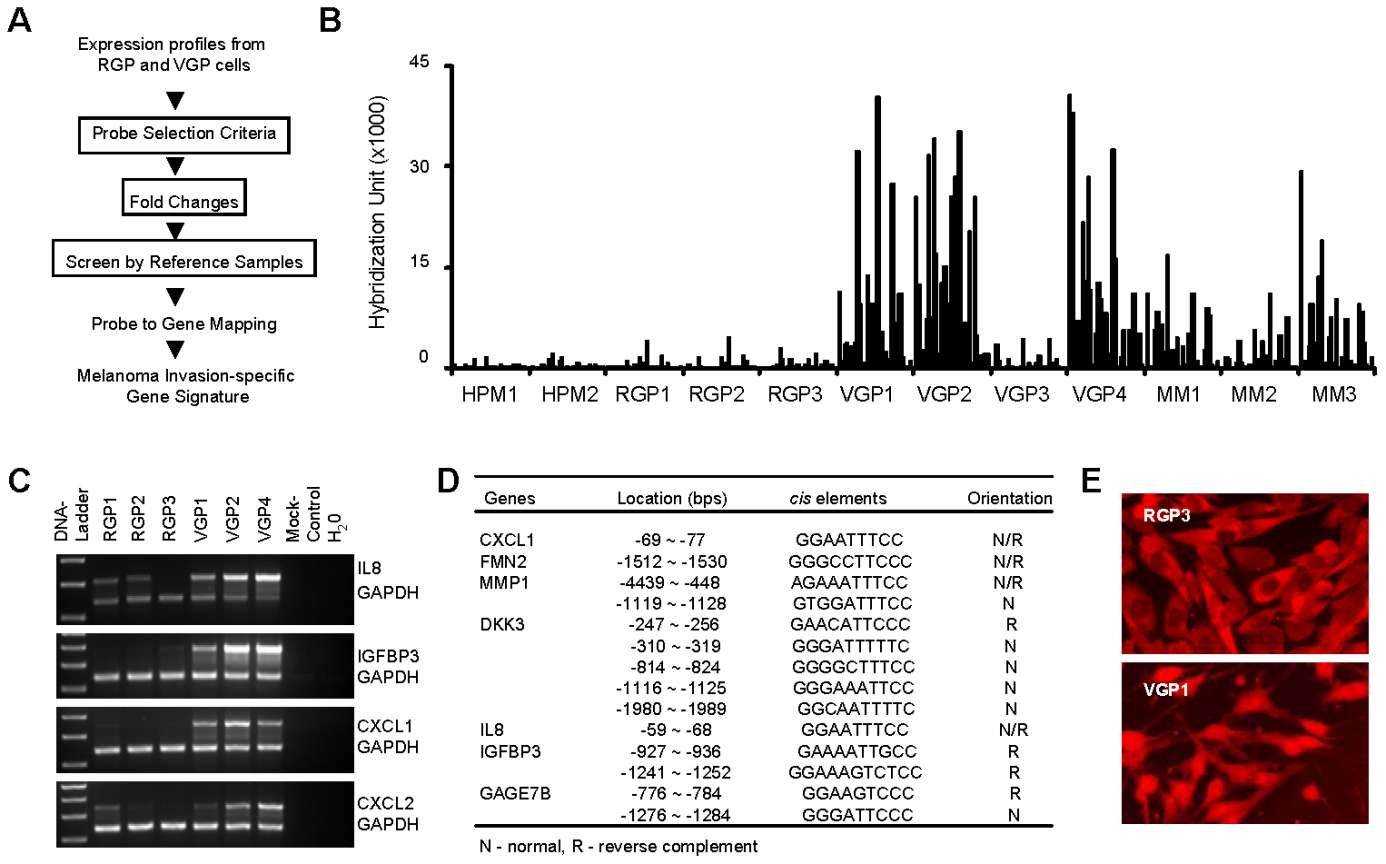
Identification of an invasion-specific gene signature for melanoma. A) The three-step data reduction algorithm used for identification of a melanoma invasion-specific signature. (see detailed description in Data Extraction and Statistical Analysis section of Methods). B) Relative expression levels of melanoma invasion-specific signature genes in all cells analyzed including human primary melanocytes (HPM1, HPM2). C) Validation of differential expression for selected genes by semi-quantitative duplex RT-PCR. Four genes (IL-8, IGFBP3, CXCL1, CXCL2) that are upregulated in invasive melanomas were selected and their differential expression was verified. D) Promoter analysis of selected genes from the melanoma invasion-specific signature identifies putative NF-κB binding *cis* elements. E) Immunofluorescence staining of NF-κB in invasive (WM902B) vs. non-invasive (WM1552C) melanoma cells demonstrates constitutive activation and nuclear trafficking of NF-κB in invasive melanomas.

A current melanoma progression model suggests the sequential evolution of primary *in situ* tumors and minimally invasive tumors which are termed “radial growth phase”, followed by a subsequent conversion to a more aggressive “vertical growth phase”, in which tumor cells are programmed to cross the epidermal basement membrane and invade vertically into the dermis. It has been postulated that the VGP is the critical stage in which a tumor gains metastatic capacity. We therefore compared the gene expression profiles of RGP and VGP melanomas using a uniquely designed data reduction algorithm in order to identify genes that are likely to be relevant to this critical invasive phenotype (Figure 3A). Our melanoma invasion-specific signature is notably characterized by the inclusion of several genes involved in chemotaxis and the inflammatory response (*CXCL1, CXCL2, IL8,* and *IL6*), cell adhesion (*HNT, ITGA4, ITGB8, CSPG2, ZP4,* and *FLRT3*), and extracellular matrix organization (*MMP1, COL4A1, COL4A2,* and *COL5A2*) (Table 3). These genes and their relative expression profiles are depicted in Figure 3B. These cellular processes have previously been implicated in tumor progression for a wide variety of malignancies including melanoma and are felt to be essential components of tumor invasion and metastasis. In addition, many of these invasion-specific signature genes are also upregulated in metastatic melanomas (Figure 3B). The differential expressions were validated on the selected genes using semi-quantitative duplex-PCR analysis (Figure 3C).

**Table 3:** Melanoma invasion-specific signature genes that are upregulated in VGP compared to RGP melanoma cells a.

Probe Set ID	Gene Title	Gene Symbol	Fold b	Biological Process
204470_at	chemokine (C-X-C motif) ligand 1	CXCL1	62.3	chemotaxis/inflammatory
1555471_a_at	formin 2	FMN2	38.8	development
204475_at	matrix metallopeptidase 1	MMP1	34.2	proteolysis/ECM organization
202196_s_at	dickkopf homolog 3 (Xenopus laevis)	DKK3^c^	24.5	Wnt receptor signaling
208894_at	major histocompatibility complex, class II, DRα	HLA-DRA	21.6	immune response
211506_s_at	interleukin 8	IL8^c^	19.9	chemotaxis/inflammatory
212730_at	desmuslin	DMN	17.6	—
210095_s_at	insulin-like growth factor binding protein 3	IGFBP3^c^	17.5	regulation of cell growth
206640_x_at	G antigens	GAGEs	15.5	cellular defense response
227566_at	neurotrimin	HNT	15.2	cell adhesion
212327_at	hypothetical protein DKFZP686A01247^c^	—	14.8	actomyosin structure
211776_s_at	erythrocyte membrane protein band 4.1-like 3	EPB41L3	13.3	cytoskeleton organization
221729_at	collagen, type V, alpha 2	COL5A2	12.7	ECM organization
226189_at	integrin, beta 8	ITGB8	12.4	cell-matrix adhesion
226847_at	follistatin	FST^c^	11.4	development
222450_at	transmembrane, prostate androgen induced RNA	TMEPAI^c^	11.3	androgen receptor signaling
212942_s_at	KIAA1199	KIAA1199	11.2	sensory perception of sound
205207_at	interleukin 6 (interferon, beta 2)	IL6	11.0	immune response/inflammatory
209619_at	CD74 molecule, major histocompatibility complex	CD74	10.7	immune response
211571_s_at	chondroitin sulfate proteoglycan 2 (versican)	CSPG2^c^	10.3	cell adhesion/development
223614_at	chromosome 8 open reading frame 57	C8orf57	10.3	—
207030_s_at	cysteine and glycine-rich protein 2	CSRP2^c^	10.0	muscle development
229800_at	Doublecortin and CaM kinase-like 1	DCAMKL1	10.0	nervous system development
211538_s_at	heat shock 70kDa protein 2	HSPA2	9.9	protein folding
223638_at	neuroblastoma breakpoint family, member 3	NBPF3	9.9	—
209312_x_at	major histocompatibility complex, class II, DR β1	HLA-DRB1^c^	9.6	immune response
1561691_at	hypothetical protein LOC285735	LOC285735	9.0	—
209774_x_at	chemokine (C-X-C motif) ligand 2	CXCL2	8.6	chemotaxis/inflammatory
209392_at	pyrophosphatase/phosphodiesterase 2	ENPP2^c^	8.3	cell motility/chemotaxis
231756_at	zona pellucida glycoprotein 4	ZP4	7.8	fertilization/cell adhesion
209309_at	alpha-2-glycoprotein 1, zinc	AZGP1	7.7	cell adhesion
241803_s_at	—	—	7.4	—
204469_at	protein tyrosine phosphatase, receptor-type, Z	PTPRZ1	7.2	nervous system development
205885_s_at	integrin, alpha 4	ITGA4	7.2	cell adhesions
228293_at	DEP domain containing 7	DEPDC7	7.0	regulation of transcription
213075_at	olfactomedin-like 2A	OLFML2A	7.0	—
219230_at	transmembrane protein 100	TMEM100	7.0	—
204681_s_at	Rap guanine nucleotide exchange factor 5	RAPGEF5	6.6	signal transduction
211980_at	collagen, type IV, alpha 1	COL4A1	6.4	ECM organization
211991_s_at	major histocompatibility complex, class II, DP α1	HLA-DPA1	6.3	immune response
216959_x_at	neuronal cell adhesion molecule	NRCAM	6.3	neuron cell adhesion
209967_s_at	cAMP responsive element modulator	CREM	6.2	signal transduction
222162_s_at	ADAM metallopeptidase	ADAMTS1	6.1	proteolysis
233903_s_at	Src domain-containing guanine exchange factor	SGEF^c^	6.1	Rho protein signaling
207034_s_at	GLI-Kruppel family member GLI2	GLI2	6.1	morphogenesis
222853_at	fibronectin leucine rich transmembrane protein 3	FLRT3	6.1	cell adhesion
226436_at	Ras association (RalGDS/AF-6) domain family 4	RASSF4	6.0	signal transduction
219872_at	chromosome 4 open reading frame 18	C4orf18	5.9	—
211966_at	collagen, type IV, alpha 2	COL4A2	5.8	ECM organization
209859_at	tripartite motif-containing 9	TRIM9	5.8	—
214023_x_at	tubulin, beta 2B	TUBB2B	5.7	microtubule-based movement
204702_s_at	nuclear factor (erythroid-derived 2)-like 3	NFE2L3	5.7	regulation of transcription
205599_at	TNF receptor-associated factor 1	TRAF1	5.6	signal transduction
212325_at	hypothetical protein, DKFZP686A01247	—	5.6	actomyosin organization
1554474_a_at	monooxygenase, DBH-like 1	MOXD1	5.6	catecholamine metabolism
235116_at	TNF receptor-associated factor 1	TRAF1	5.2	signal transduction
210139_s_at	peripheral myelin protein 22	PMP22	5.1	peripheral nervous develop
203680_at	protein kinase, cAMP-dependent, type II, beta	PRKAR2B	5.0	signal transduction

a Genes with greater than five-fold upregulation are shown.

b Fold represents average expression ratio of VGP melanoma cells over RGP melanoma cells.

c Gene with multiple probesets are shown with a representative probeset.

Since the melanoma invasion-specific signature was associated with common functions of matrix invasion/inflammation/cell migration we sought to determine whether a common upstream regulatory pathway might link these signature genes. The eight most highly up-regulated genes from our melanoma invasion-specific signature were selected for further evaluation and gene promoter sequences were analyzed to identify transcription factor binding *cis* elements. This promoter analysis yielded a profile of transcription factors with common sequence elements in the signature genes. The most ubiquitous *cis* elements among the gene promoters evaluated were E12, E47, GCN4, GR, HES-1, IL-6, MEF-2, NF-κB, N-Oct-3, PU.1, RAR-alpha1, SRF, and the basal gene transcriptional complex components of TFIID, TBP, and TBF1. We notably identified the NF-κB binding sequence in 7 out of 8 of the most upregulated invasion-specific signature genes (Figure 3D). We were particularly interested in the NF-κB pathway as a mediator of melanoma invasion since our most highly upregulated invasion-specific genes, *CXCL-1* and *IL-8*, have previously been reported to be activated by NF-κB and had previously been implicated in melanoma progression (reviewed in [46]). Given the consequences of NF-κB activation in a cell, NF-κB function is highly regulated by specific cytosolic inhibitory activities which prevent inappropriate NF-κB activation and shuttling to the nucleus. Thus, only nuclear NF-κB is considered to be functionally activated. In order to evaluate NF-κB function in our tumor cell lines, we used NF-κB cellular localization as a surrogate marker for NF-κB activity. We find that invasive (VGP) melanomas posses both cytosolic (inactive) and nuclear (active) localization of NF-κB, while non-invasive (RGP) melanomas possess NF-κB confined to the cytosolic compartment suggesting specific activation of NF-κB during melanoma progression (Figure 3E).

## Discussion

Molecular profiling studies of melanoma to date have been variably successful and often inconsistent. Much of this inconsistency has been attributed to the heterogeneous nature of this malignancy and the lack of significant sources of meaningful archived tissue specimens for analysis. In addition, variable sample preparation techniques are also likely to lead to disparate results between investigators. Here we have used a series of well-defined melanoma cell lines from varying stages of malignant progression to assess molecular signatures associated with disease progression. These cell lines have undergone extensive characterization of their tumorigenic potential and invasion capacity [14], [47], and have been shown to possess a remarkable ability to recapitulate the clinical stages of disease from which they were derived. We show that unsupervised hierarchical clustering of global gene expression profiles of melanoma cell lines allows for the classification of tumor cells into 2 groups (Figure 1A) that we have defined as less-aggressive (Group 1) and more-aggressive (Group 2) melanomas. While all radial growth phase melanomas clustered in Group 1, and all metastatic melanomas clustered in Group 2, vertical growth phase melanomas failed to form a distinctive cluster suggesting that vertical growth phase melanomas may be considered to be a transient or transition phase within the current melanoma progression model [48]. Aggressive (Group 2) melanomas were characterized by upregulation of genes associated with cell cycle progression, DNA replication and repair, and altered expression of apoptosis-related genes including upregulation of the antiapoptotic gene BIRC5/survivin [49] and downregulation of the novel stress-associated apoptosis inducer TRIB3 [50] (Table 1). These signature genes for melanoma progression are remarkably similar to those obtained from recent large-scale studies using primary human melanomas and suggest high correlation with alterations seen in primary tumor specimens [10]. Notably, we did not identify a dominant signature associated with BRAF kinase mutations which may be reflective of the relative infrequency of wildtype BRAF in these cell lines. As a whole, this gene signature suggests a series of molecular alterations occur in aggressive melanomas that promote melanoma cell growth, survival and apoptotic resistance which contribute to the unresponsiveness of melanomas to traditional chemotherapeutic agents [51].

While gene signatures associated with aggressive melanomas provide insights into molecular pathways important for tumor progression, further analysis of these tumor cell lines in conjunction with expression profiles from primary human melanocytes using SAM analysis revealed a striking signature characterized exclusively by gene loss in melanomas and primarily by loss of cellular adhesion and melanocyte differentiation-associated genes (Figures 2B, 2C). We suggest that this melanoma-associated signature defines critical molecular mechanisms involved in melanocyte development and differentiation which distinguish these tumor cells from their primary cell of origin. In fact, the identification of several genes in this signature with established functional roles in melanocyte differentiation and melanin biosynthesis such as *CDH3, CDH1, c-KIT, PAX3, CITED1/MSG-1, TYR, MELANA, MC1R,* and *OCA2* [35]–[42], supports this notion. Moreover, this signature has identified two tumor suppressor genes, DPP4 and SYK, whose downregulation has previously been implicated in melanoma development [52]–[54].

Finally, evaluation of an invasion-specific signature for melanoma identified dominant gene activation by the transcription factor, NF-κB. Constitutive activation of NF-κB and an inflammatory response is an emerging hallmark of various tumor types [55]. In addition, NF-κB has specifically been implicated in the development of invasive aggressive melanomas through autocrine and paracrine mechanisms (reviewed in [46]). Our melanoma invasion signature is associated with upregulation of critical NF-κB effectors including CXCL1, FMN2, MMP1, IL-8, IGFBP3 which have been implicated in the regulation of tumor cell proliferation, motility, migration, and/or invasion (Figure 3D). In addition, the putative NF-κB target gene GAGE7B which we identified in our melanoma invasion-specific signature, has been associated with apoptotic resistance and worse prognosis in other tumors [56]. In addition, several of our invasion-specific signature genes are chemokines including CXCL1, CXCL2, and IL-8 which have been implicated in the promotion of tumor-associated angiogenesis, a critical feature of invasive tumors [57].

In summary, our gene expression profiling studies of melanoma cell lines from varying stages of malignant progression and primary human melanocytes have identified several important melanoma signatures including: 1) Aggressive melanomas are characterized by upregulation of genes associated with cell cycle progression, DNA replication and repair and apoptotic resistance as well as loss of genes associated with apoptotic susceptibility, 2) Melanomas notably differ from their cell of origin, primary human melanocytes, due to a loss of cellular adhesion and differentiation-associated genes, and 3) Invasive melanomas are characterized by a signature indicative of global activation of NF-κB and downstream effector genes associated with tumor cell migration, invasion, chemotaxis, and proliferation. Since pathways associated with tumor progression may have clinical utility as prognostic tumor markers and therapeutic targets, we expect novel melanoma signature genes identified in this study will be further developed for such translational endpoints. Moreover, the important information regarding melanoma biology gleaned from these studies on renewable cell resources cannot be understated. A major roadblock to advances in melanoma therapy has been the relative paucity of informative tissue specimens available for analysis in profiling studies as well as the notoriously heterogeneous nature of this malignancy. The use of surrogate tissue resources including tumor cell lines for the early discovery phases in melanoma, as used in this study, will undoubtedly allow for the conservation of precious tissue specimens for use in more advanced validation studies. It is expected that the novel melanoma progression-associated genes identified in this study will provide new insights into the molecular defects associated with this malignancy and ultimately pave the way for the development of new melanoma biomarkers and novel targeted therapies.

## Materials and Methods

### Cells

Ten melanoma cell lines (WM35, SBC12, and WM1552C, WM902B, WM278, WM983A, and WM793, WM852, WM983B, 1205Lu) were obtained from M. Herlyn (The Wistar Institute, Philadelphia, PA). These cell lines were maintained in modified complete melanocyte growth medium (Cell Application Inc., San Diego, CA) which lacked 12-*O* -tetradecanoyl phorbol-13-acetate and was supplemented with 2 % fetal bovine serum. Normal human primary melanocytes were isolated from neonatal foreskins and grown in complete melanocyte growth medium (Cell Applications, Cat. No., 135–500). The complete melanocyte growth medium is consisted of the melanocyte basal medium (Cell Applications, Cat. No. 134-500) and growth supplement cocktails containing hydrocortisone (0.5 µg/ml), insulin (5 µg/ml), 12-O-tetradecanoylphorbol-13-acetate (10 ng/ml), bovine pituitary extract (21 µg/ml), bFGF (1 ng/ml), heparin (1 µg/ml), FBS (0.5 %), gentamycin sulfate (50 µg/ml), amphotericin B (5 ng/ml), and NaCl (45 mM).

### Gene Expression Profiling

Total RNA was isolated from exponentially growing melanoma cell lines using RNeasy column purification per manufacturer's protocol (Qiagen). Two sets of short-term cultured (2 to 3 passage numbers) normal human melanocytes were prepared from neonatal foreskins. In order to minimize genetic variability melanocytes from 4–5 individuals were pooled for each culture. Total RNA from normal melanocytes was extracted and purified by a combination of phase extraction and chromatography using TRIzol reagent (Invitrogen Life Technologies Inc.) and RNeasy columns (Qiagen) in order to remove melanin. In brief, exponentially growing melanocytes were lysed with TRIzol reagent and lysate was incubated at 65°C for 2 minutes to inactivate melanin. Lysate was then subjected to phase extraction and RNeasy column purification. RNA quality checks, double strand complementary DNA synthesis, hybridization with Human Genome U133 Plus 2.0 Array Chips (Affymetrix Inc. Santa Clara, CA), and initial data extraction were performed at The Gene Array Core Facility in the Malaria Research Institute (JHMRI) at The Johns Hopkins Bloomberg School of Public Health (http://malaria.jhsph.edu/jhmri/resources_education/gene_array_core).

### Data Extraction and Statistical Analysis

SAM [19], Gene Cluster 3.0 [58] and TreeView (http://bonsai.ims.u-tokyo.ac.jp/∼mdehoon/software/cluster/index.html), Access, and Excel (Microsoft, Seattle, WA) programs were used. For all of the statistical analysis beyond the initial description of datasets, microarray data were normalized (Dataset S1) and a subset of the 12 microarray data (10 from melanoma cell lines and 2 from normal human melanocytes) was obtained by filtering to require each gene probe to have at least one observation in the expression intensity resulting in a ‘present’ call from all 12 samples. This produced a subset of data containing a total of 32,632 affymetrix gene probes (Dataset S2). This filtered subset of data was used for all of the additional analysis. *Cluster Analysis.* Unsupervised hierarchical clustering analysis was performed on the subset of data (without log transformation) with Gene Cluster 3.0 (http://bonsai.ims.u-tokyo.ac.jp/∼mdehoon/software/cluster/index.html) by using the correlation (uncentered) similarity metric and centeroid linkage clustering method. The resulting tree-images were visualized using Java TreeView. *Statistical Analysis of Microarray (SAM).* SAM was performed on the subset of array data without log transformation using SAM software package. Groups are defined based on the hierarchical clustering; for example, group 1  =  less-aggressive primary melanomas (RGP melanomas: WM35, Sbcl2, WM1552C and VGP melanomas: WM902B and WM278), and group 2  =  aggressive metastatic melanomas (Metastatic melanomas: WM852, WM983B, and 1205Lu; and VGP melanomas: WM983A and WM793) as seen Figure 1. Delta was chosen to limit the output gene list so that minimum predicted false-positives would be included. *Three-step Data Reduction Algorithm.* In order to identify melanoma invasion-specific gene signature, uniquely designed three-step data reduction algorithm was applied to the subset of expression data. First step is that the proveset should be called as ‘present’ in three samples out of four VGP melanoma cells and two samples out of three RGP melanoma cells. Second step is that the candidate proveset should be expressed five folds or more in VGP melanoma cell lines than RGP melanoma cell lines. The last step is that the gene probesets were retained only when the expression level is greater than three folds in VGP melanoma cell lines compared to that of primary human melanocytes. The last step is implemented because the candidate gene expression level should be higher if the gene products have certain degree of functional roles in the invasion processes of malignant melanoma. The probesets, those that pass through three-step filtration criteria are subjected to probeset to gene mapping using NetAffx, a web interface program from Affymetrix Inc. Gene annotation for the gene mane, gene symbol, and GO Biological Procession Analysis also performed by the NetAffx.

### Quantitative Real-time PCR

cDNA was generated by using the SuperScript™ First-Strand Synthesis System for RT-PCR according to manufacturers instructions (Invitrogen, Carlsbad, CA). Quantitative real-time PCR was performed with an Applied Biosystems Prism 7900 HT Sequence Detection System using SYBR Green PCR Master Mix (Applied Biosystems, Foster City, CA). The thermal cycling conditions for quantitative real-time RT-PCR analysis to validate gene expression changes were as follows: hold for 10 minutes at 95°C, followed by three-step PCR for 40 cycles of 95°C for 15 seconds, 55°C to 60°C for 25 seconds, and 72°C for 30 seconds. Optimal annealing temperatures were predetermined to ensure single amplified product. All samples were performed in triplicate. Amplification data were analyzed with an Applied Biosystems Prism Sequencer Detection Software Version 2.3 (Applied Biosystems, Forster City, CA). Human *GAPDH* gene was used as endogenous control. To normalize the relative expression of the genes of interest to the *GAPDH* control, standard curves were prepared for each gene and *GAPDH* in each experiment.

### Semi-quantitative Duplex PCR

Semi-quantitative duplex RT-PCR was performed by an MJ Research Programmable Thermal Controller (PTC-100, Inc., Watertown, MA) and the amplified products were separated on an agarose gel. Our duplex PCR utilized 20 bp oligonucleotides to amplify regions of 300–400 bp from the genes of interest. Intitial optimization experiments were conducted to establish the most favorable primer concentrations between the genes of interest and internal control GAPDH, yielding 0.8 µM and 0.04 µM, respectively. The PCR was carried out in a total volume of 25 µL, containing 2.5 µL of 10X PCR Buffer (containing 15 mM MgCl_2_), 0.2 mM dNTPs, and 0.3 ul AmpliTaq Gold DNA Polymerase (Applied Biosystems, Foster City, CA). Thirty to thirty-five amplification cycles were performed by an MJ Research Programmable Thermal Controller (PTC-100, Inc., Watertown, MA), using a denaturing temperature of 95°C for 25 seconds, an annealing temperature varying between 55°C−60°C (depending on gene) for 30 seconds, and primer extension at 72°C for 30 seconds. Each amplification experiment also included two negative PCR controls, a no-RNA control from reverse transcription procedures and a no-cDNA water control. Following amplification, 25 µL of the samples were separated via electrophoresis on a 3% agarose gel. The primer sequences were designed by using Primer3, primer analysis software (http://frodo.wi.mit.edu/cgi-bin/primer3/primer3_www.cgi), yielded only one amplified product and had the following sequences:[Table pone-0000594-t004]

**Table pone-0000594-t004:** 

MELK	Forward: 5′-TGGCTCTCTCCCAGTAGCAT-3′
	Reverse: 5′-TAGCACTGGCTTGTCCACAG-3′
GINS4	Forward: 5′-CAGAGAGTTCATGGCGAACA-3′
	Reverse: 5′-CTCCCAAAGTGCTGGGATTA-3′
NCAPG	Forward: 5′-TATTGGTGTGCCCTTTGTGA-3′
	Reverse: 5′-CAGGGATATTGGGATTGTGG-3′
CDH3	Forward: 5′-ACGACACCCTCTTGGTGTTC-3′
	Reverse: 5′-GTCAAACTGCCCACATTCCT-3′
KIT	Forward: 5′-TGACTTACGACAGGCTCGTG-3′
	Reverse: 5′-AAGGAGTGAACAGGGTGTGG-3′
DPP4	Forward: 5′-CAAATTGAAGCAGCCAGACA-3′
	Reverse: 5′-CAGGGCTTTGGAGATCTGAG-3′
SYR	Forward: 5′-GAAGCCATATCGAGGGATGA-3′
	Reverse: 5′-TGACAAGTTGTGGGCATGTT-3′
CXCL1	Forward: 5′-TGTTTGAGCATCGCTTAGGA-3′
	Reverse: 5′-GATCTCATTGGCCATTTGCT-3′
CXCL2	Forward: 5′-TTGCGCCTAATGTGTTTGAG-3′
	Reverse: 5′-ATACATTTCCCTGCCGTCAC-3′
IL8	Forward: 5′-AGGGTTGCCAGATGCAATAC-3′
	Reverse: 5′-AGCAGACTAGGGTTGCCAGA-3′
IGFBP3	Forward: 5′-GCTACAGCATGCAGAGCAAG-3′
	Reverse: 5′-AACATGTGGTGAGCATTCCA-3′
GAPDH	Forward: 5′-GATCATCAGCAATGCCTCCT-3′
	Reverse: 5′-TTCAGCTCAGGGATGACCTT-3′

### Immunofluorescence Labeling

RGP and VGP Cells were plated on glass slides and cultured in melanocyte growth media overnight without any stimulation. Cells were fixed at room temperature for 15 minutes using 3.5% para-formaldehyde solution. Cells were washed briefly with PBS and then permeabilized with either 0.5% Triton X-100 for 10 minutes or −20°C cooled methanol for 15 minutes. Slides were blocked with 16% normal goat serum (Santa Cruz Biotech., Santa Cruz, CA) for 1 hour and then incubated with rabbit polyclonal IgG p65 antibody (Santa Cruz Biotech., Santa Cruz, CA) at 1:100 dilution. Subsequent to overnight incubation at 4°C, the slides were washed with PBS and incubated with goat anti-rabbit IgG-Alexa 594 (Molecular Probes Eugene, OR) at 1:200 dilution at room temperature for 1 hour. Stained slides were washed with PBS and viewed under a fluorescence microscope (Eclipse TS100, Nikon, Tokyo, Japan).

### Gene Transcription Promoter Analysis

Transcription factor binding *cis* element sequence profiling in the selected gene promoter was performed by using a web tool known as TESS (Transcription Element Search System, http://www.cbil.upenn.edu/tess). Each genes promoter sequences from the transcription start site up to 2.0 kb of upstream of the genes were subjected to the TESS and screened by TRANSFAC database to identify matched consensus sequences of known DNA binding transcription factors.

## Supporting Information

Dataset S1 A combined and normalized raw dataset from the 12 sets of microarray data (two sets of primary human melanocytes and ten melanoma cell lines).
(18.02 MB DOC)

Dataset S2 A subset of the 12 microarray data (10 from melanoma cell lines and 2 from human primary melanocytes) was obtained by filtering to require each Affymetrix probe to have at least one observation in the expression intensity resulting in a present call from all 12 samples.
(4.50 MB DOC)

Table S1 Results of SAM analysis between Group 1 and Group 2 melanoma cells. The results include the SAM Output showing the list of Affymetrix probesets and associated gene symbols that are differentially expressed in the two groups of melanoma cell lines.
(3.20 MB DOC)

Table S2 Results of SAM analysis between HPMs and Group 2 melanoma cells. The results include the SAM Output showing the list of Affymetrix probesets and associated gene symbols that are downregulated in Group 2 melanoma cell lines.
(3.05 MB DOC)
