# Supplementary material for: Comprehensive Expression Profiling of Tumor Cell Lines Identifies Molecular Signatures of Melanoma Progression
Source: PLoS One. 2007 Jul 4;2(7):e594. doi: 10.1371/journal.pone.0000594 (PMC1895889; doi:10.1371/journal.pone.0000594)
Supplement: Supplementary file 1 [file pone.0000594.s003.doc]

Table S1. Results of SAM analysis between Group 1 and Group 2 melanoma cells. Th

Current settings

Input parameters

Data type?
Arrays centered?
Delta
Minimum fold change
Test statistic
Are data are log scale?
Number of permutations
Input percentile for exchangeability factor s0
Number of neighbors for KNN
Seed for Random number generator

Computed values

Estimate of pi0 (proportion of null genes)
Exchangibility factor s0
s0 percentile
False Discovery Rate (%)

List of Significant Genes for Delta =

Positive genes (89)

Row	Gene ID	Gene Name	Score(d)	Numerator(r)
27884		235793_at	18.7602492728	347.14
24536		228955_at	11.8836140621	675.32
31456		243840_at	10.91816328	311.84
10690		209507_at	10.6398915061	6188.5
3850	RAD21	200608_s_at	9.90961291258	3022.88
7492	CDC7L1	204510_at	9.00532218246	1107.6
6508	RPS6KA1	203379_at	8.86490614226	634.9
7621	RNF24	204668_at	8.73040757701	313.7
12405	PURB	212205_at	8.44119501671	5215.88
23338		227349_at	8.08427250008	385.7
12256	DKFZP586M1523	212055_at	8.0157736683	1874.4
25502		230522_s_at	7.97811375019	161.64
27467		235178_x_at	7.93006302217	166.5
14767	MGC3077	215380_s_at	7.91327622426	6180.04
11684		211042_x_at	7.91084425671	1162.1
7016	LOC51668	203960_s_at	7.84628486666	899.3
4346	CSE1L	201112_s_at	7.73948729105	6861.04
16135	LOC51203	218039_at	7.52249178164	4634.64
6670	GGH	203560_at	7.17591298479	5357.72
5503	MTHFD1	202309_at	7.14035720132	3708.22
12406	PURB	212206_s_at	7.11341237962	2148.44
22591	KIAA1357	226490_at	7.0023736424	1541.84
7703	CHAF1B	204775_at	6.96602233612	521.48
10398		209191_at	6.86672582133	4953.12
17550	FLJ20311	219588_s_at	6.79576133425	2618.42

17314	FLJ10545	219294_at	6.78362116143	465.58
6232	CUL2	203079_s_at	6.77074235407	568.7
17872	FLJ20641	220060_s_at	6.7418943039	431.76
22402	LOC81023	226287_at	6.72351367948	967.72
21617		225447_at	6.69146435798	1846.6
29285		238964_at	6.66265806666	99.1
16573	FLJ22940	218493_at	6.63838459824	2450.78
7741	KIAA0175	204825_at	6.63038468708	5597.62
22989		226936_at	6.60227429076	2368.28
18692		221677_s_at	6.55123846483	1627.84
17974	SBBI26	220239_at	6.53853787641	1729.38
21472		225297_at	6.52133194519	1261.3
27614		235391_at	6.48245021489	1190.04
28454		236957_at	6.46784895418	807.5
24419	KIAA0203	228774_at	6.46275775439	643.02
1919		1558330_x_at	6.45178310806	403.56
13112	HCAP-H	212949_at	6.38178962937	1265.88
20925		224734_at	6.33748246712	1453.28
27528		235266_at	6.2344703727	621.84
7532	RAD54L	204558_at	6.20713904649	929.9
17973	SBBI26	220238_s_at	6.19423747377	1017.44
12071		211792_s_at	6.18350449732	459.06
4547	PSMA2	201317_s_at	6.17799179261	7351.5
26848		233456_at	6.14231013534	97.3
15155	AND-1	216228_s_at	6.11298914505	373.12
16735	HCAP-G	218663_at	6.11084339404	1523
8521	POLE2	205909_at	6.06021825213	891.92
13921	MLX	213951_s_at	5.99720391422	696.24
31033		242890_at	5.98707233981	346.12
16112	FLJ12549	218014_at	5.97404315737	2246.3
7031	CHAF1A	203975_s_at	5.97288782416	431.84
8099	HNRPA2B1	205292_s_at	5.95928623307	10087.04
13680	DKFZP586M1523	213617_s_at	5.95038384422	1461.76
5298	BIRC5	202095_s_at	5.90441496305	3067.32
15066	DKFZp434F222	216026_s_at	5.90326859721	553.32
10700	ASH2L	209517_s_at	5.90033757309	1633.52
5770	TYMS	202589_at	5.8959787499	20131.92
17184	TOPK	219148_at	5.88335215176	5771.44
7073	ZWINT	204026_s_at	5.87841278789	5819.8
4931	TUBG1	201714_at	5.8633486875	2402.12
26056		231832_at	5.84278811371	203.62
9960	SMT3H2	208738_x_at	5.83782415049	8709.92
3963	ACTR1A	200721_s_at	5.83293197233	1140.68
10067		208845_at	5.81730653779	4613.62
7189	CDKN2C	204159_at	5.80071137988	2309.78
25204		230021_at	5.7953150343	355.88
3807		200072_s_at	5.78477472546	3932.48
23339		227350_at	5.78263784429	679
10938		209811_at	5.76064094307	165.32
22745		226661_at	5.74045404683	1464.18
20443		223945_x_at	5.71113968483	766
8089	LRP8	205282_at	5.70179705865	966.28
8636	KIAA0186	206102_at	5.6872925259	2122.74

24200		228468_at	5.67940274524	750.54
7694	NUDT1	204766_s_at	5.67257373488	981.8
24089	AF15Q14	228323_at	5.64915821518	823.5
12055		211767_at	5.6334329373	538.7
22171		226032_at	5.62533093237	1352.74
7864	NF2	204991_s_at	5.61885182771	149.66
18926	MPHOSPH9	221965_at	5.59479560144	465.42
21844		225686_at	5.58546717794	1515.38
27640		235425_at	5.57551730515	486.44
5717	DHFR	202534_x_at	5.57471528853	3490.1
28458		236970_s_at	5.57248354785	92.78

Negative genes (53)

Row	Gene ID	Gene Name	Score(d)	Numerator(r)
11709		211073_x_at	-11.099417125	-22822.6
11981	TARBP-b	211666_x_at	-11.058246753	-18784.68
12240	RPL3	212039_x_at	-10.514131183	-21597.02
15041		215963_x_at	-10.374842365	-21641.5
1227		1555788_a_at	-10.344093462	-5107.8
17909	FLJ13955	220128_s_at	-9.9835711228	-246.4
1988		1558620_at	-9.7172087017	-585.54
3771	RPL10A	200036_s_at	-9.5855208023	-10322.42
4449	RPL3	201217_x_at	-9.4031648379	-22920.78
13931	RPL29	213969_x_at	-8.4474941009	-20778.48
13462	RPS4X	213347_x_at	-7.9393871397	-11700.9
3760	RPL27	200025_s_at	-7.8369182317	-12265.5
21272	C11ORF4	225090_at	-7.7512619046	-2134.96
18203	G8	220755_s_at	-7.6598655681	-12062.74
4172	RPS4X	200933_x_at	-7.2872431791	-12583.58
16238	LOC57761	218145_at	-7.2056385012	-15305.16
19891		223195_s_at	-7.1783307662	-1830
4128	RPL23	200888_s_at	-7.0784148584	-12961.92
25387		230304_at	-7.0517705731	-107.62
5518	GCP60	202324_s_at	-6.9185296441	-1317.8
12140		211936_at	-6.865353085	-23074.8
6020	PCK2	202847_at	-6.8252058545	-1706.36
20643		224367_at	-6.8164275824	-2606.52
7910	ASNS	205047_s_at	-6.8064080487	-10877.96
16346	HUGT1	218257_s_at	-6.7501413109	-336.56
21092		224905_at	-6.680254941	-1243.82
12961	KIAA0350	212786_at	-6.5959882785	-293.16
4718	USP7	201498_at	-6.5292775722	-381.74
9438	XAP4	207713_s_at	-6.4493537376	-1144.2
30087		240983_s_at	-6.44838588	-1472.88
3753	RPS13	200018_at	-6.4192810877	-15440.7
22180		226042_at	-6.2388339244	-436.12
13111	KIAA0909	212948_at	-6.2345654567	-1041.52
13656	RPL14	213588_x_at	-6.2242046084	-9070.2
21858	FLJ00020	225701_at	-6.2151236323	-707.3
4109	RPL18A	200869_at	-6.2078963779	-21139.66
12160	SUI1	211956_s_at	-6.1936960785	-21126.88
21028	GAS5	224841_x_at	-6.157769047	-21948.94

19892	DKFZp761M02121	223196_s_at	-6.1120592641	-1373.44
14163	TPT1	214327_x_at	-6.1064510987	-9194.66
3767	RPL9	200032_s_at	-6.0864675896	-15183.6
23660	RPL21	227755_at	-6.0843744304	-962.12
12481	FLJ20030	212284_x_at	-6.0842694642	-15679
20932	GAS5	224741_x_at	-6.0638864791	-23942.36
12991	CBS	212816_s_at	-6.0388640977	-1327.66
3834		200099_s_at	-6.0299203187	-14848.56
13041	FLJ20030	212869_x_at	-5.986905358	-17899.92
4023	RPS15A	200781_s_at	-5.957752059	-16131.7
17563	MGC4645	219609_at	-5.9573414351	-246
24933	HSPB1	229563_s_at	-5.9561717709	-14933.22
4149	RPLP2	200909_s_at	-5.9170624857	-19855.22
12396		212196_at	-5.9092056309	-3298.26
27476		235191_at	-5.897135428	-879.82

Estimated Miss rates for Delta=2.215

Quantiles	Cutpoints	Miss Rate(%)
0 -> 0.05	-5.875 -> -2.277	67.34
0.05 -> 0.1	-2.277 -> -1.706	42.45
0.1 -> 0.15	-1.706 -> -1.375	31.45
0.15 -> 0.2	-1.375 -> -1.144	28.66
0.2 -> 0.25	-1.144 -> -0.948	20.11
0.25 -> 0.75	-0.948 -> 0.814	1.52
0.75 -> 0.8	0.814 -> 1.041	0
0.8 -> 0.85	1.041 -> 1.334	1.02
0.85 -> 0.9	1.334 -> 1.706	21.44
0.9 -> 0.95	1.706 -> 2.372	38.61
0.95 -> 1	2.372 -> 5.546	73.21

e results include the SAM Output showing the list of Affymetrix probesets and associated gene sym

Two class unpaired
0
2.215
0
standard
0
100
Automatic choice
10
1234567

0.827760954
1.475466028
0
3.497581497

: 2.215

Denominator(s+s0)	Fold Change	q-value(%)
18.5040185208725	3.8486788118	0
56.8278300245645	2.2415339927	0
28.5615805519401	8.9551020408	0
581.631870629454	3.5079227421	0
305.045214850317	1.7375900369	0
122.993933760349	3.6	0
71.6194835919403	2.6211316515	0
35.931884878558	2.2111033897	0
617.907771313902	4.0246103173	0
47.7099207128649	4.4492935074	0
233.838937770084	2.7581840353	0
20.2604280988367	2.3427479648	0
20.996050035731	5.0570175439	0
780.971095265339	2.8573626823	0
146.899618079925	2.5780825638	0
114.614752750238	2.391329909	0
886.497999413524	2.1025647618	0
616.104362029555	4.1290187553	2.178318301
746.625552923325	3.8344425517	2.178318301
519.332562146298	2.9457346444	2.178318301
302.026634383813	3.2998137404	2.178318301
220.188193138318	3.0896671365	2.178318301
74.8605121887189	3.3439410284	2.178318301
721.3219413268	4.20308337	2.178318301
385.301936193955	4.6395251863	2.178318301

68.6329600253933	2.9542478173	2.399307114
83.99374400332	2.0506965229	2.399307114
64.0413481044899	3.6009638554	2.399307114
143.930695486315	2.8721609596	2.399307114
275.963511305095	2.1939738782	2.399307114
14.8739435535243	3.2918593895	2.399307114
369.183189634745	3.115038749	2.399307114
844.237591660212	5.9011645215	2.399307114
358.706696465728	3.8005770777	2.399307114
248.478208927824	3.0778106811	2.399307114
264.490323783297	3.5131953729	2.399307114
193.411408988219	2.2512152054	2.399307114
183.578733434235	2.8290298783	2.399307114
124.848308258445	4.3362254173	2.399307114
99.4962250539047	2.9010761589	2.399307114
62.5501498176769	4.6109520401	2.399307114
198.35815241771	5.7560865645	2.399307114
229.315032828926	2.9414342204	2.399307114
99.7422335540284	5.7432494279	2.399307114
149.811369301691	6.2288573999	3.143396029
164.255891755494	2.7093511643	3.143396029
74.2394543739582	2.7591201717	3.143396029
1189.94978413512	1.9793094812	3.143396029
15.8409454840475	2.9267326733	3.143396029
61.037242361558	3.5796460177	3.143396029
249.229100108384	4.4998621197	3.143396029
147.176217570405	3.9260547208	3.143396029
116.09410151114	3.0733770101	3.143396029
57.8112273169745	4.1835908756	3.143396029
376.010005423308	3.2775941435	3.143396029
72.3000352113735	3.0038979118	3.143396029
1692.65908793316	2.1283017264	3.143396029
245.658101774353	2.3220939908	3.143396029
519.496007512676	5.1360841424	3.183695978
93.7311238491528	3.049485147	3.183695978
276.851956310015	1.9702657432	3.183695978
3414.51705543187	7.1638641569	3.183695978
980.97816536075	5.0978117323	3.183695978
990.029147322115	4.6692053565	3.183695978
409.683975493518	3.013782234	3.183695978
34.8498004783666	2.8157660068	3.183695978
1491.98053512378	2.3937165369	3.183695978
195.558598216319	1.9556955662	3.183695978
793.085248307197	1.9723979783	3.183695978
398.189092464071	5.7849270799	3.183695978
61.4082233482573	3.7712194362	3.183695978
679.798295807523	1.8126469798	3.183695978
117.420460745397	8.487869431	3.183695978
28.6981955018059	2.5756767061	3.183695978
255.063447604488	4.900319659	3.183695978
134.123842572983	1.858628884	3.183695978
169.46937782252	2.3911315865	3.183695978
373.242626492793	4.6846727999	3.183695978

132.151219708701	4.9711111111	3.183695978
173.078402483136	3.6013459806	3.497581497
145.773931023374	3.5122025625	3.497581497
95.6255281629445	5.5590724441	3.497581497
240.472963504512	2.0089201808	3.497581497
26.6353348671596	1.6499609137	3.497581497
83.1880256502029	2.1438191202	3.497581497
271.307654619428	3.1487436901	3.497581497
87.2457161151524	3.1078083023	3.497581497
626.05887823176	4.470250169	3.497581497
16.649667819272	2.3407514451	3.497581497

Denominator(s+s0)	Fold Change	q-value(%)
2056.19806360664	0.6001520721	0
1698.70327725437	0.5931290157	0
2054.09459168193	0.6215082384	0
2085.95940436869	0.5993438136	0
493.789041912388	0.1489978508	0
24.6805473681597	0.2027953928	0
60.2580450800345	0.2922619479	0
1076.87628172425	0.6078093002	0
2437.5601613948	0.6143604456	0
2459.72116131567	0.5579797303	0
1473.77874314186	0.7290625452	2.670196626
1565.09225149694	0.7510830945	2.670196626
275.433861774581	0.2958575198	2.670196626
1574.79787246612	0.4102128325	2.670196626
1726.7956743952	0.7383494673	2.670196626
2124.05326709921	0.2168870242	2.670196626
254.933919822774	0.2665683414	2.670196626
1831.1896461666	0.6805495311	3.123626242
15.2614153969059	0.4906767629	3.123626242
190.473997768402	0.6116006956	3.123626242
3361.05073028396	0.4098145305	3.123626242
250.008576498289	0.3772726941	3.123626242
382.387983806106	0.0810334372	3.123626242
1598.19392580738	0.2205073672	3.123626242
49.8596969303006	0.687525532	3.123626242
186.193492760713	0.4385066811	3.123626242
44.4451972350525	0.5845120327	3.143396029
58.4658862756909	0.7047321442	3.143396029
177.413124873473	0.3703777073	3.183695978
228.410648404011	0.3588256804	3.183695978
2405.36281072624	0.6869180671	3.183695978
69.9040886943237	0.6511095822	3.183695978
167.055748670664	0.3749429868	3.183695978
1457.24643881191	0.6499041214	3.183695978
113.803045900517	0.1691140192	3.183695978
3405.28557715355	0.622307783	3.183695978
3411.02949390327	0.5876114472	3.183695978
3564.43053198105	0.3659575255	3.497581497

224.709863019219	0.2746477386	3.497581497
1505.72891707946	0.776194271	3.497581497
2494.64895301208	0.719719328	3.497581497
158.129650140724	0.2700151745	3.497581497
2576.97330667947	0.7452477055	3.497581497
3948.35227910446	0.3587978511	3.497581497
219.852604483545	0.3874694348	3.497581497
2462.4803007653	0.7326221774	3.497581497
2989.8451586866	0.7215375923	3.497581497
2707.6823339153	0.6456862398	3.497581497
41.2935875307264	0.2945629732	3.497581497
2507.184240886	0.7259870139	3.497581497
3355.58734555744	0.6552188915	3.497581497
558.15624061976	0.3360963611	3.497581497
149.194470898808	0.3337725276	3.497581497

ıbols that are differentially expressed in the two groups of melanoma cell lines.

Significant: 142
Median number of false positives: 4.97
False Discovery Rate (%): 3.5

Tail strength (%): 27.3
se (%): 10.3

SAM Plotsheet

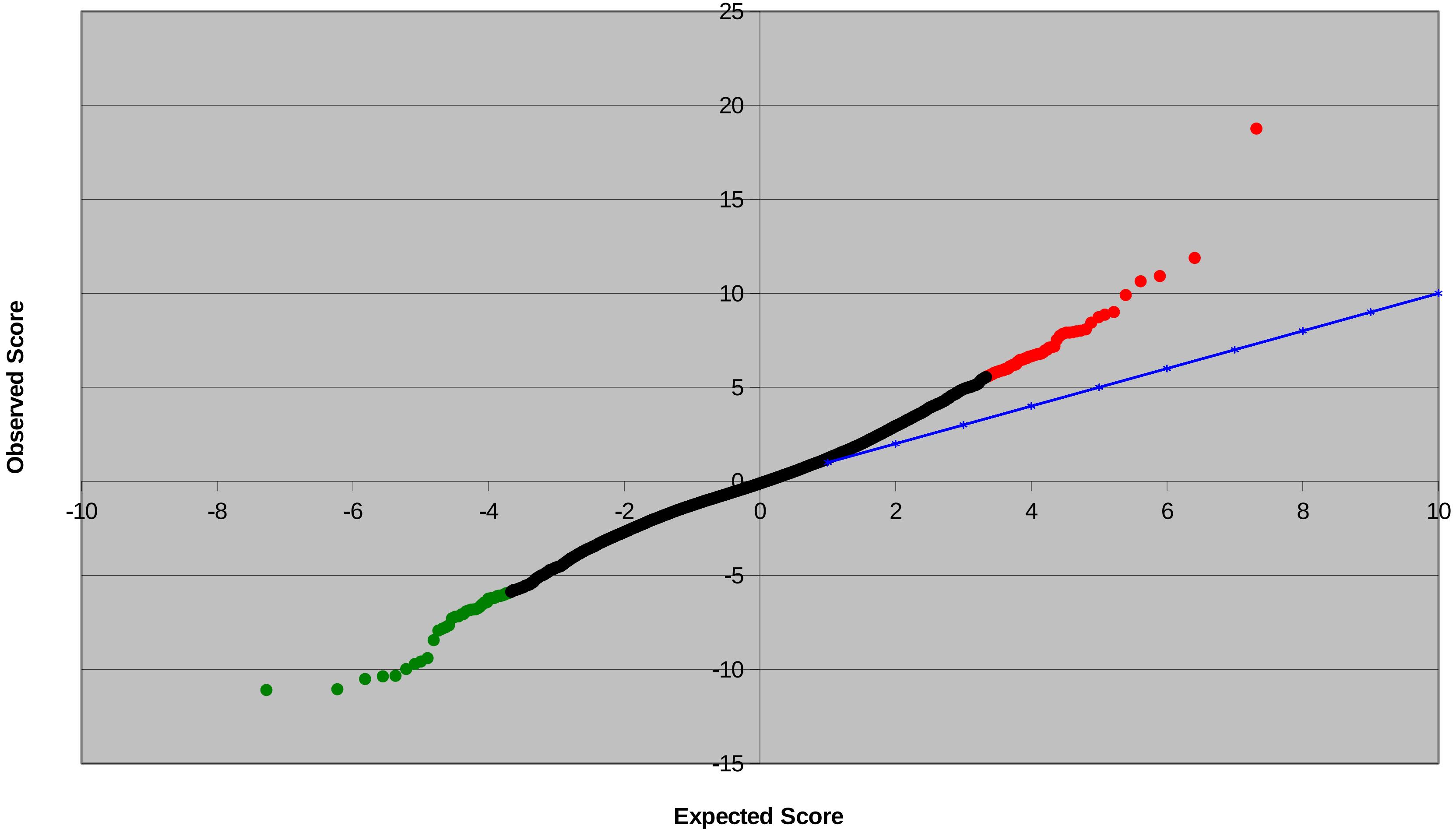

		Positive X	Positive Y	Negative X	Negative Y
-3.667295935993	-5.875334934532	3.3388701	5.5724835	-7.2745812	-11.099417
-3.657626464799	-5.868289317223	3.3457543	5.5747153	-6.2291853	-11.058247
-3.645580555033	-5.810127385666	3.351136	5.5755173	-5.8203205	-10.514131
-3.634246400995	-5.787671241788	3.3576187	5.5854672	-5.5582447	-10.374842
-3.623605939807	-5.786455199291	3.3648525	5.5947956	-5.3719189	-10.344093
-3.614402370674	-5.785841801685	3.3725559	5.6188518	-5.2145314	-9.9835711
-3.604782758822	-5.784542615303	3.3803063	5.6253309	-5.0866806	-9.7172087
-3.595026743306	-5.771427710355	3.387007	5.6334329	-4.9985759	-9.5855208
-3.586096083679	-5.747887971665	3.3941438	5.6491582	-4.8993672	-9.4031648
-3.575026615806	-5.745544924114	3.4012798	5.6725737	-4.8091587	-8.4474941
-3.565713102187	-5.733609007631	3.4073894	5.6794027	-4.7402715	-7.9393871
-3.556697642566	-5.716620876122	3.4143099	5.6872925	-4.6806702	-7.8369182
-3.549162260521	-5.706104110323	3.4217058	5.7017971	-4.6278028	-7.7512619
-3.539044757441	-5.695781869265	3.4304579	5.7111397	-4.5836508	-7.6598656
-3.529553904948	-5.676998443978	3.4398508	5.740454	-4.5384332	-7.2872432
-3.52149851963	-5.661819728992	3.4489803	5.7606409	-4.4919659	-7.2056385
-3.512205745434	-5.655926297703	3.4581991	5.7826378	-4.4422924	-7.1783308
-3.503368788672	-5.645115110412	3.4660559	5.7847747	-4.3986653	-7.0784149
-3.494772409985	-5.643351120675	3.4746158	5.795315	-4.3674916	-7.0517706
-3.487108467634	-5.626772098883	3.4837245	5.8007114	-4.3302681	-6.9185296
-3.47730606953	-5.60963932384	3.4927858	5.8173065	-4.2883622	-6.8653531
-3.469463522713	-5.572990921017	3.5019293	5.832932	-4.2575295	-6.8252059
-3.461545356938	-5.558049745802	3.510382	5.8378242	-4.2258901	-6.8164276
-3.451121449272	-5.553357377215	3.5212669	5.8427881	-4.1897105	-6.806408
-3.443453132279	-5.551772374214	3.5301052	5.8633487	-4.1578686	-6.7501413
-3.434670859828	-5.536032667511	3.5387986	5.8784128	-4.1301015	-6.6802549
-3.427685956835	-5.533737134111	3.5474907	5.8833522	-4.1082701	-6.5959883
-3.420102910245	-5.513524646595	3.5589796	5.8959787	-4.0889783	-6.5292776
-3.412990297728	-5.497637378948	3.5699221	5.9003376	-4.0657928	-6.4493537
-3.40621900961	-5.489470067794	3.5796877	5.9032686	-4.0387319	-6.4483859
-3.398662968444	-5.482331724323	3.5875485	5.904415	-4.0193202	-6.4192811
-3.390556235618	-5.480537119492	3.5974211	5.9503838	-4.0017939	-6.2388339
-3.384194160736	-5.438131916309	3.6070238	5.9592862	-3.9846993	-6.2345655
-3.377353344166	-5.437772071788	3.6187198	5.9728878	-3.9658636	-6.2242046
-3.371213189444	-5.408460695928	3.6322399	5.9740432	-3.9460058	-6.2151236
-3.363386841479	-5.398920044142	3.6430129	5.9870723	-3.9238829	-6.2078964
-3.35651522136	-5.376027453719	3.6572195	5.9972039	-3.9079016	-6.1936961
-3.34947857306	-5.369951332952	3.6693082	6.0602183	-3.8908334	-6.157769
-3.343213798054	-5.34961556641	3.6794848	6.1108434	-3.8760688	-6.1120593
-3.336684742922	-5.344940726369	3.6919854	6.1129891	-3.8586219	-6.1064511
-3.331024494773	-5.329383119952	3.7046213	6.1423101	-3.8448622	-6.0864676
-3.324058035571	-5.261250661482	3.7182504	6.1779918	-3.8290871	-6.0843744
-3.316983750291	-5.246618636601	3.732799	6.1835045	-3.8142618	-6.0842695
-3.311301896114	-5.220633948118	3.7477903	6.1942375	-3.8005711	-6.0638865
-3.304855090361	-5.210674146458	3.7631672	6.207139	-3.7838011	-6.0388641
-3.298394832284	-5.171242448709	3.7784175	6.2344704	-3.76851	-6.0299203
-3.293076439792	-5.170210559818	3.7913709	6.3374825	-3.7543523	-5.9869054
-3.287840655534	-5.159572911705	3.8094459	6.3817896	-3.7405525	-5.9577521
-3.281671759605	-5.149151351843	3.8285998	6.4517831	-3.7259217	-5.9573414
-3.277245006419	-5.129368414255	3.8445465	6.4627578	-3.7127825	-5.9561718
-3.271582553521	-5.119397303596	3.8620377	6.467849	-3.7017967	-5.9170625
-3.26686216533	-5.098415092478	3.8800433	6.4824502	-3.6903375	-5.9092056
-3.261127705203	-5.092504190507	3.8978209	6.5213319	-3.679913	-5.8971354

-3.256335944407	-5.090853654071	3.9105633	6.5385379
-3.250952804246	-5.071727828615	3.9306964	6.5512385
-3.24522245805	-5.049740648425	3.9481469	6.6022743
-3.240677787989	-5.047848586646	3.9643241	6.6303847
-3.236781655374	-5.038910373948	3.9838842	6.6383846
-3.232520013496	-5.034240337699	4.0047505	6.6626581
-3.22706467055	-5.016201175193	4.0293905	6.6914644
-3.222214286064	-5.015454168096	4.0528746	6.7235137
-3.216791765245	-5.001188024331	4.0727884	6.7418943
-3.212060417162	-4.996432127549	4.0944726	6.7707424
-3.206354694511	-4.988119645085	4.1159313	6.7836212
-3.200922617997	-4.982770323086	4.145873	6.7957613
-3.19633024611	-4.982503266196	4.1765214	6.8667258
-3.191052699909	-4.971642247671	4.2018425	6.9660223
-3.185709531153	-4.963719187586	4.2323248	7.0023736
-3.180940506834	-4.950386862242	4.2615268	7.1134124
-3.176305826216	-4.943429432484	4.3062346	7.1403572
-3.172606479285	-4.926285211693	4.339921	7.175913
-3.167819460843	-4.906912309714	4.3740421	7.5224918
-3.162700258481	-4.894953640313	4.4198415	7.7394873
-3.158437749548	-4.887407318294	4.4633297	7.8462849
-3.153415479807	-4.885723820178	4.5132912	7.9108443
-3.150003576014	-4.884216617477	4.5614726	7.9132762
-3.145225197587	-4.87531270573	4.6076336	7.930063
-3.140173731242	-4.853958611782	4.6643522	7.9781138
-3.136389535267	-4.842581680878	4.7282257	8.0157737
-3.132314645454	-4.821289188184	4.8029139	8.0842725
-3.1274307268	-4.8108464649	4.8827635	8.441195
-3.123900851959	-4.810404783132	4.9909427	8.7304076
-3.119534427611	-4.804141357642	5.0825054	8.8649061
-3.114817357376	-4.800862623543	5.2162117	9.0053222
-3.110777329496	-4.776693738613	5.3907767	9.9096129
-3.106421257841	-4.75174026693	5.6104542	10.639892
-3.102531024617	-4.730845374899	5.8935282	10.918163
-3.098425940369	-4.723329862821	6.4068302	11.883614
-3.093246423936	-4.714883820979	7.3163084	18.760249
-3.089076112114	-4.711382885449		
-3.085408772637	-4.710031112927		
-3.080937487482	-4.707101203334		
-3.076671076257	-4.695028384691		
-3.073041590189	-4.694354679014		
-3.068327127803	-4.690951403903		
-3.064765452582	-4.690729037499		
-3.061220446534	-4.687867091334		
-3.057762018382	-4.683443795628		
-3.053586276229	-4.681298342844		
-3.049062423806	-4.668704330429		
-3.045361063531	-4.66792345126		
-3.040907471977	-4.659981101731		
-3.036364196726	-4.655618832323		
-3.032631484606	-4.648221595724		
-3.028641670672	-4.646376655149		
-3.024778631366	-4.632676211247		
-3.02097566299	-4.6199630854		

-3.017022404027	-4.596227502104
-3.012564404566	-4.596094563812
-3.008536244787	-4.592514608692
-3.005180313324	-4.59015649635
-3.001796576464	-4.586803812067
-2.998557794004	-4.586477469297
-2.995016154294	-4.579926067587
-2.991716616161	-4.576555638832
-2.98866695556	-4.574828850182
-2.985170984791	-4.573479108823
-2.981533077838	-4.561435410231
-2.978051474182	-4.554522988674
-2.975020362781	-4.552754801129
-2.971605807775	-4.551061789161
-2.967550807996	-4.544363136747
-2.963742766737	-4.54313082715
-2.961155500105	-4.539493707466
-2.957926709822	-4.532268543686
-2.954617106844	-4.528335596663
-2.951832413685	-4.527419284873
-2.949391079273	-4.522562099034
-2.945706893956	-4.521379618282
-2.943188890186	-4.515604767375
-2.940403754057	-4.513493281419
-2.937470750383	-4.509019639609
-2.93438726388	-4.498757555919
-2.931625834085	-4.495042149618
-2.928139201765	-4.48328946444
-2.925478537418	-4.473943328239
-2.922064909116	-4.460327655896
-2.919398802022	-4.45074312955
-2.916341072743	-4.447685042262
-2.913241098787	-4.424987313284
-2.910219402052	-4.416795566464
-2.907864665057	-4.414806708269
-2.904558906675	-4.413778209652
-2.901756533362	-4.412613358889
-2.89838349664	-4.409776798857
-2.895286654272	-4.404902884477
-2.892494903832	-4.402855054753
-2.890067853911	-4.400761521372
-2.887219116838	-4.400449798041
-2.884405604177	-4.371347341956
-2.881243724638	-4.354024088401
-2.878684021847	-4.349558497689
-2.875329920092	-4.346186992356
-2.872620147244	-4.337732392372
-2.870081373044	-4.332911536899
-2.867637161106	-4.314397620308
-2.865364000153	-4.310609848262
-2.862929125803	-4.30802277996
-2.860345494537	-4.3063768407
-2.857935672441	-4.299536870963
-2.855730708632	-4.298052743569

-2.853230541118	-4.280827205909
-2.850144736144	-4.277153572575
-2.847145810401	-4.269854289202
-2.844521620962	-4.258694548949
-2.84182563777	-4.248919628682
-2.839459366167	-4.246520454012
-2.837122903385	-4.244197849243
-2.834345752164	-4.238912452901
-2.832200243948	-4.223064394209
-2.829806531851	-4.22222005642
-2.826900958533	-4.219983341258
-2.824317227376	-4.216929864325
-2.821805546349	-4.207110023297
-2.818729094441	-4.19756589568
-2.815537461365	-4.186832072956
-2.812832964844	-4.17803935547
-2.810529396018	-4.17431236984
-2.808016209389	-4.171756192248
-2.8060253737	-4.166786787899
-2.803732111397	-4.164569384018
-2.801074149569	-4.155076779248
-2.798841820333	-4.128379408161
-2.796575486313	-4.12469880403
-2.794027767833	-4.122929170216
-2.791406667273	-4.118027826919
-2.788826917946	-4.10917060413
-2.786252679347	-4.099557657929
-2.783762242534	-4.098589591033
-2.781085263905	-4.09606056139
-2.778984294463	-4.092053767933
-2.777082858721	-4.085875858684
-2.775511735676	-4.085427657911
-2.773020453178	-4.085388792872
-2.770764183211	-4.075620336164
-2.768151957293	-4.073816539832
-2.765904756277	-4.073390812205
-2.763589180837	-4.07231867132
-2.761359908739	-4.057928945036
-2.759620125252	-4.056393336445
-2.757632288182	-4.055560420766
-2.755844414041	-4.048652014341
-2.753861392121	-4.033112538359
-2.751261124072	-4.032926820434
-2.748857333392	-4.031347406933
-2.747071932612	-4.02755963838
-2.744998211985	-4.023270155947
-2.742718897494	-4.018002742591
-2.740618389894	-4.008789787892
-2.738298027256	-4.008213537312
-2.735959059188	-4.007012329053
-2.733819961334	-4.00692814498
-2.731504411504	-3.99997019552
-2.729670796096	-3.991107451779
-2.727811748149	-3.980563677006

-2.725597126971	-3.98020531934
-2.723618849551	-3.979494016961
-2.721763885296	-3.979347903027
-2.720204713368	-3.978429495484
-2.717816659955	-3.96506168704
-2.715730884978	-3.958086931405
-2.713784839579	-3.949481161903
-2.711987400155	-3.946897789501
-2.710169386441	-3.94320238478
-2.70821386786	-3.942378313001
-2.706095950835	-3.938149546835
-2.70391214	-3.933991155108
-2.701810973758	-3.932588358472
-2.700226338036	-3.9227345717
-2.698305699182	-3.918371964237
-2.696521737061	-3.91338439011
-2.694355195722	-3.907980824656
-2.692359383049	-3.895354965093
-2.690667418963	-3.891076358278
-2.688217120231	-3.883802816271
-2.68608210074	-3.88255004471
-2.683654106552	-3.879932210176
-2.68179889506	-3.878940568178
-2.679784328992	-3.877125901882
-2.678254498742	-3.87298546977
-2.676583079718	-3.870138697543
-2.67463893691	-3.866142260871
-2.672783664231	-3.861394676859
-2.670858047415	-3.859802398779
-2.669239707924	-3.856662650714
-2.667518263055	-3.854178823968
-2.665626262637	-3.853486989317
-2.664068160222	-3.851776484606
-2.662163074346	-3.848122570023
-2.660619757166	-3.845879258968
-2.658537466788	-3.844136089571
-2.656585360087	-3.84149527617
-2.654824356523	-3.839428248915
-2.65350470517	-3.834896937952
-2.651847498726	-3.830617582857
-2.650048841367	-3.824950826714
-2.647985376051	-3.823899103035
-2.646282054258	-3.822805094462
-2.644544206788	-3.811136308038
-2.642591089967	-3.800376700222
-2.64085631429	-3.799870594909
-2.639351335659	-3.798896733221
-2.637485851902	-3.798857225482
-2.636053128911	-3.794332648482
-2.634467450194	-3.779293780873
-2.632659350858	-3.777217355193
-2.631194127296	-3.776996769307
-2.629613825882	-3.773022588522
-2.62805492305	-3.772661168216

-2.626378369153	-3.762684026834
-2.624492691518	-3.759615180634
-2.622622559888	-3.758444337111
-2.620777836635	-3.757156849591
-2.619289708397	-3.753204998048
-2.617886731755	-3.748681532096
-2.616263187385	-3.747805874076
-2.614722292521	-3.747462561702
-2.612976009877	-3.746798494157
-2.611241536322	-3.745380607559
-2.609752009662	-3.740312220741
-2.607889614885	-3.739853015287
-2.606492060124	-3.738457833445
-2.605165816265	-3.738236804587
-2.603756098681	-3.73392734222
-2.601992283768	-3.732664510077
-2.600359203326	-3.731102843734
-2.599000749272	-3.721346092248
-2.597364976975	-3.721004553988
-2.595700843949	-3.714227986466
-2.594168916781	-3.710453142615
-2.592370676388	-3.707958824001
-2.590949528122	-3.702734521634
-2.58927867168	-3.699332260572
-2.587536588949	-3.698296337728
-2.586030532182	-3.694565998117
-2.584476081215	-3.693767967926
-2.583076722783	-3.693186029808
-2.58152797516	-3.686408831012
-2.580167267018	-3.684417968872
-2.578767517509	-3.67582646479
-2.577325901938	-3.671579946654
-2.575939323114	-3.671412459769
-2.574734076807	-3.670707564805
-2.573281398482	-3.658456868063
-2.571893612592	-3.656040870415
-2.570359371895	-3.655337368071
-2.568816553449	-3.654234365631
-2.567334106111	-3.651622897894
-2.565911220999	-3.649659172112
-2.564512601655	-3.648849300706
-2.562800973456	-3.647248162363
-2.561430442017	-3.645969985504
-2.560021803382	-3.643347588016
-2.558600052492	-3.640205982944
-2.557059391664	-3.637514768924
-2.555183801118	-3.631668728349
-2.553469955069	-3.630998299494
-2.551925135477	-3.629147415976
-2.550271863124	-3.625172323782
-2.548854067851	-3.623136483986
-2.547464678544	-3.621730845779
-2.545808628607	-3.621420054793
-2.544237882043	-3.619607325858

-2.542637406794	-3.618402521501
-2.541104597553	-3.614126501944
-2.539535902916	-3.611012537674
-2.537894608627	-3.608293778564
-2.536066996196	-3.599828564881
-2.534486002337	-3.599168696125
-2.532995651056	-3.595367679111
-2.531465127398	-3.594824104725
-2.529994419721	-3.592708387453
-2.528689655329	-3.591518334016
-2.527200144139	-3.591431991774
-2.525769066981	-3.589817594946
-2.524607526663	-3.587566595457
-2.52332444313	-3.587224083869
-2.521855928325	-3.583097703173
-2.520609916423	-3.579858160266
-2.519197347878	-3.576882472597
-2.518036522649	-3.575349718827
-2.516455227612	-3.574461760723
-2.514959306509	-3.57245832831
-2.513592904358	-3.570591282024
-2.512340751667	-3.569299469498
-2.510950468279	-3.567399228381
-2.509452145036	-3.565638261616
-2.508288577393	-3.560981651283
-2.506994613546	-3.556927448999
-2.50530865776	-3.556189504293
-2.503990100866	-3.555982073576
-2.502891403389	-3.553940623539
-2.501619441529	-3.551052364678
-2.500394805293	-3.549040053289
-2.498972118423	-3.546621150154
-2.497656233234	-3.544313120054
-2.49648330251	-3.543468660904
-2.495228667523	-3.540556508255
-2.493922459895	-3.536466255084
-2.492352235901	-3.535058188222
-2.490947060515	-3.531245004345
-2.489782589761	-3.530202702474
-2.488369827821	-3.525420415456
-2.48678780268	-3.525241696818
-2.48511578883	-3.518550058672
-2.48378306111	-3.517262112244
-2.482695885678	-3.516749634105
-2.481344006732	-3.5164168576
-2.480161037359	-3.514605854628
-2.478770692634	-3.513077935363
-2.477510718623	-3.506055205283
-2.476227649795	-3.502489024464
-2.475029362873	-3.501698263666
-2.473789477636	-3.498043058186
-2.472400218391	-3.497109211736
-2.471331437139	-3.494482155379
-2.470018121497	-3.49393996535

-2.46858670877	-3.491703025908
-2.467311848763	-3.486710107654
-2.466143429887	-3.483643530001
-2.464699613231	-3.480936526613
-2.463330002329	-3.479003946717
-2.462085734257	-3.475264266488
-2.460953775826	-3.474765563091
-2.4596482825	-3.470526313144
-2.458484758272	-3.469722185232
-2.456983099069	-3.467633426579
-2.455741661358	-3.465929694871
-2.454478281701	-3.464740600467
-2.452977013825	-3.464697661914
-2.45185284906	-3.463547270398
-2.450719823149	-3.459329477258
-2.449532796425	-3.45884455579
-2.44832092892	-3.457976040578
-2.447091986725	-3.454717544037
-2.445642796713	-3.454004734348
-2.444546728578	-3.453462446747
-2.443273423992	-3.452869978295
-2.441964018714	-3.451428411647
-2.44063860818	-3.449038394623
-2.439605260028	-3.448171814692
-2.43843536511	-3.447621274892
-2.437074360756	-3.441646823516
-2.435907510304	-3.439062268659
-2.434545929831	-3.438945482738
-2.433192197445	-3.438909300201
-2.432239336033	-3.438399698412
-2.430998712844	-3.437918055759
-2.429697399896	-3.432860959699
-2.428521122202	-3.430735562548
-2.427285868824	-3.430431983005
-2.426121303313	-3.42674855855
-2.425163941875	-3.417662244676
-2.42417309996	-3.415798118919
-2.42290957214	-3.410006807775
-2.421665255042	-3.40915831768
-2.420651192252	-3.408879530976
-2.419597289906	-3.407805119788
-2.41847036811	-3.407090308236
-2.417388166302	-3.404809712968
-2.416271181541	-3.40286991578
-2.415134850192	-3.402230529613
-2.413993848253	-3.399990418204
-2.412874572041	-3.395554852111
-2.411876679942	-3.395101495621
-2.410660896256	-3.386661855838
-2.409814148692	-3.384007561518
-2.408794413043	-3.379877606752
-2.407388544583	-3.379774551726
-2.406264929748	-3.375573567702
-2.405098349182	-3.368482401478

-2.403903187729	-3.366404161468
-2.402726252619	-3.365728130656
-2.401717786896	-3.364687121109
-2.400591866193	-3.364455351526
-2.399459878997	-3.364226725157
-2.398422642198	-3.358146952632
-2.397300778014	-3.354181033584
-2.395762850734	-3.353652222886
-2.394568752415	-3.351901941575
-2.393662401823	-3.351565945781
-2.392579001394	-3.350842147546
-2.391440972794	-3.346495725531
-2.390359086773	-3.343234491729
-2.389189121673	-3.342991007922
-2.388309266334	-3.341560315465
-2.387110313227	-3.338408535188
-2.385940921302	-3.337855336924
-2.384907777444	-3.337549742048
-2.38387221045	-3.336780052567
-2.382843314404	-3.33394537369
-2.381783012476	-3.330350603298
-2.380581847745	-3.328948167825
-2.379519920338	-3.324812909373
-2.378447349517	-3.323140679718
-2.377460467983	-3.32066205929
-2.376520555276	-3.319488344957
-2.375278798991	-3.313496131866
-2.374375386631	-3.310093281759
-2.372984118555	-3.306089160709
-2.37173208083	-3.304665279091
-2.370793402456	-3.300649831846
-2.36992433692	-3.298738318899
-2.368807132684	-3.296879050088
-2.367444141154	-3.291428925393
-2.366387139431	-3.290660844953
-2.365458775445	-3.288755865421
-2.364491436564	-3.287382458359
-2.363260266076	-3.28664799483
-2.362267309374	-3.286190436842
-2.36135744175	-3.285826213233
-2.360495737972	-3.285655261409
-2.359539491048	-3.283272954916
-2.358428294015	-3.28321254794
-2.357291938066	-3.280541953776
-2.356129674872	-3.277650709364
-2.355129311861	-3.277619897142
-2.354189178999	-3.276105118043
-2.35306534997	-3.27367360617
-2.352023914818	-3.272252539861
-2.351120107305	-3.271851929786
-2.350066380422	-3.27167672309
-2.349014762253	-3.26595871723
-2.348102104809	-3.26550453494
-2.347018945543	-3.263065815389

-2.345945600817	-3.261901221131
-2.345000899349	-3.260367072318
-2.343766242788	-3.259976317854
-2.342806975716	-3.258151057039
-2.341766446624	-3.254777391463
-2.340639849048	-3.254122467537
-2.339641022342	-3.253542692474
-2.338543737144	-3.25017413234
-2.337285816021	-3.249772522736
-2.336404489743	-3.249752730352
-2.335489621379	-3.248725499894
-2.334430936586	-3.247294891202
-2.333445495003	-3.247274448019
-2.332481203237	-3.242340630279
-2.33149453867	-3.239221308445
-2.330491790472	-3.238714011877
-2.329299092339	-3.238375068517
-2.328277231378	-3.235667943368
-2.327274954247	-3.232855188273
-2.326228066287	-3.23268479485
-2.325410906284	-3.229763187079
-2.324473487783	-3.229080257419
-2.323359309845	-3.225014067755
-2.322475547142	-3.22499505964
-2.32178273542	-3.224874609244
-2.320729420152	-3.223973380523
-2.319759729534	-3.219550895472
-2.318801643995	-3.215480741734
-2.317872324985	-3.21501548064
-2.316835092446	-3.205315963065
-2.315945834968	-3.20475991715
-2.314977103061	-3.199829178032
-2.314150912795	-3.199076715131
-2.313250344085	-3.197104672176
-2.312255781714	-3.195033819764
-2.311385955188	-3.194314601275
-2.310422143624	-3.193261143553
-2.309586238328	-3.19302457913
-2.308505257451	-3.191588960409
-2.307561174934	-3.189956374458
-2.306515826306	-3.186714287974
-2.305542576162	-3.186134832252
-2.304555016287	-3.184693740841
-2.303902420208	-3.184134063759
-2.302999576462	-3.184015495801
-2.302071532307	-3.183886384575
-2.30096653704	-3.182393185634
-2.30021203261	-3.179000968469
-2.299374208419	-3.178156461763
-2.298515305098	-3.177655978756
-2.297437742573	-3.177455982237
-2.296494014235	-3.176755746625
-2.295677813671	-3.176480183393
-2.294682869992	-3.175895942361

-2.293703190406	-3.175154200132
-2.292628038231	-3.173934284107
-2.291905978102	-3.168601471062
-2.291032810878	-3.168034625175
-2.290059488881	-3.167814934978
-2.289028887883	-3.167459674044
-2.288070926726	-3.160758626625
-2.287050528994	-3.157124241357
-2.2863212232	-3.155578090827
-2.28542038929	-3.155440028287
-2.284566010028	-3.151863946173
-2.283784962939	-3.147470396644
-2.282829237882	-3.145573777541
-2.281929031645	-3.144578915655
-2.281113207192	-3.144321166258
-2.280264890387	-3.143216408032
-2.279386807951	-3.140253743168
-2.278630814129	-3.139760424405
-2.27757827943	-3.139340093911
-2.276761163741	-3.138925331202
-2.275912212158	-3.138166424513
-2.275063760923	-3.136900414481
-2.2741412004	-3.136385053779
-2.273431819917	-3.135441894018
-2.272670845926	-3.134763484542
-2.271662224633	-3.133776883354
-2.270654355006	-3.133648652423
-2.269748166133	-3.133456248456
-2.268654562883	-3.131749275771
-2.267876835161	-3.125640440819
-2.267017218256	-3.124306735296
-2.266322587047	-3.123540128774
-2.265452211849	-3.122285039642
-2.264605278704	-3.121546409315
-2.263738550557	-3.120752274044
-2.262857487094	-3.11907344584
-2.262078090011	-3.117603301001
-2.261225436044	-3.113879441792
-2.260355427419	-3.111800614855
-2.259440092438	-3.111753225972
-2.258612828174	-3.109525141384
-2.25776608738	-3.108388138405
-2.256968143698	-3.107448646131
-2.256097540298	-3.105192652049
-2.25517529271	-3.100107233229
-2.254339864551	-3.098033610867
-2.253430232783	-3.097626353498
-2.252685194317	-3.0960979464
-2.251713948701	-3.095894792005
-2.250778757047	-3.09577408852
-2.250003285068	-3.094351484265
-2.24924863047	-3.094233235914
-2.248482282137	-3.094154206805
-2.247700273127	-3.089512364326

-2.246777762795	-3.089455520566
-2.246047535974	-3.088020210469
-2.245040087298	-3.086470044427
-2.24418246142	-3.086051186604
-2.243369363189	-3.084571044802
-2.242570122833	-3.083595521838
-2.241799248438	-3.08294583205
-2.240929457101	-3.082759936707
-2.239961570876	-3.080991221624
-2.239019103225	-3.079688631177
-2.23822074113	-3.079027461163
-2.237313756789	-3.078580422787
-2.236464365054	-3.076267200357
-2.235689581379	-3.075792974273
-2.234788768193	-3.074866107016
-2.233889659382	-3.073809310155
-2.232908571479	-3.071698112984
-2.232150787492	-3.070708084995
-2.231332429242	-3.070402825506
-2.230576950441	-3.066514047165
-2.229923576492	-3.064494029578
-2.229271912732	-3.060729979944
-2.228464136494	-3.057082016157
-2.227573356914	-3.053945506624
-2.226847524352	-3.053378866213
-2.22578714263	-3.051041535385
-2.225275283775	-3.049618979492
-2.224466211701	-3.04916508939
-2.223775829046	-3.049031435105
-2.222936553242	-3.048704707475
-2.222019260822	-3.048499041847
-2.221074727643	-3.047961369533
-2.220298886867	-3.04788418582
-2.219410549196	-3.047140037088
-2.218606936463	-3.044402485129
-2.217764165439	-3.043243864582
-2.216944720434	-3.042933903141
-2.216188153526	-3.042341458941
-2.215437511529	-3.039931726587
-2.21471816748	-3.039904958954
-2.213804863108	-3.037077198533
-2.213000938404	-3.036394101337
-2.212009081588	-3.036277045744
-2.211006517404	-3.035451095455
-2.210268315106	-3.033609198896
-2.209581525562	-3.030867175263
-2.208859464669	-3.029061557174
-2.208105165119	-3.027017542444
-2.207331191311	-3.02696371797
-2.206618265409	-3.02666708034
-2.205836156888	-3.02312055153
-2.205150761311	-3.021692332833
-2.204372995006	-3.021468038131
-2.203668726546	-3.021192807652

-2.202779692447	-3.02097823722
-2.201939947016	-3.019711770207
-2.200994133256	-3.017371551109
-2.200129617207	-3.017142993729
-2.199472226738	-3.015758902594
-2.198856034684	-3.015656603431
-2.198081905337	-3.013413710164
-2.197181441013	-3.011249690144
-2.196257408293	-3.008754916515
-2.195331032974	-3.006569679725
-2.19459618561	-3.00474406596
-2.193782245211	-3.00448855744
-2.192964683261	-3.004121586074
-2.192144604317	-3.003220268227
-2.191425211535	-3.002611056922
-2.190529847049	-3.002127396616
-2.189959856185	-2.999350281637
-2.189248299666	-2.99856196458
-2.188534209623	-2.996956754073
-2.187682277557	-2.995455270182
-2.186936467368	-2.992501961247
-2.186456846941	-2.99202767992
-2.185803858773	-2.989470462862
-2.185104429444	-2.989030325915
-2.184336275569	-2.988557910288
-2.183599441725	-2.988065791185
-2.182753934011	-2.987180003689
-2.182047581894	-2.986806303612
-2.181262965507	-2.985970288137
-2.180439471413	-2.985301822234
-2.179836684086	-2.983748311845
-2.179053516017	-2.983088245372
-2.178351425103	-2.980973628993
-2.177710077589	-2.980927781122
-2.176976079743	-2.979694735838
-2.176281379032	-2.979526827347
-2.175644357129	-2.979124579308
-2.174764798811	-2.978306174621
-2.174167814034	-2.977764243728
-2.173517429734	-2.977340774254
-2.172863502161	-2.976809082813
-2.172214941331	-2.976698426296
-2.171457866552	-2.975436697966
-2.170727131156	-2.974414375268
-2.170075329639	-2.973904267226
-2.169194346925	-2.972235237071
-2.168419285984	-2.971821306844
-2.167827965785	-2.971391740451
-2.167222783857	-2.971208544072
-2.166505399428	-2.96257214565
-2.165846249811	-2.962009994985
-2.164988121895	-2.961109025215
-2.16426253125	-2.961005282171
-2.163592804812	-2.959326895441

-2.162771026097	-2.956585951515
-2.162023049731	-2.95418405694
-2.161454669348	-2.953586841328
-2.160710421392	-2.953154602424
-2.159973490902	-2.951940524845
-2.159117714568	-2.951620240738
-2.158453119518	-2.95128326688
-2.157772754089	-2.95127429441
-2.157214170537	-2.951209529954
-2.156503669149	-2.949135691778
-2.155923961104	-2.948830519487
-2.15534383859	-2.947563581774
-2.15460864151	-2.946782330486
-2.153776916722	-2.9457601474
-2.152889191438	-2.944932504322
-2.152190738417	-2.941794594801
-2.151630716449	-2.940611213874
-2.150834246071	-2.940241665717
-2.150132546212	-2.938958254856
-2.149410894459	-2.938721441397
-2.148660731916	-2.938486583991
-2.147972659951	-2.937839757268
-2.147275193369	-2.935747684355
-2.146565671996	-2.93517656263
-2.145870781618	-2.934259671159
-2.145245665995	-2.933504644133
-2.144541399094	-2.92987378955
-2.143983229454	-2.928174827938
-2.14335599634	-2.927599977678
-2.142678520003	-2.92731379459
-2.142087090021	-2.922306167901
-2.141259570525	-2.921722154316
-2.140598218325	-2.918848137528
-2.140019087053	-2.918706280868
-2.139349131222	-2.916466981341
-2.138807850864	-2.916381529408
-2.138261792651	-2.912722727094
-2.137448497326	-2.912638797152
-2.136864183599	-2.909828885895
-2.136204466911	-2.909523170081
-2.135500955303	-2.909248751947
-2.134850375664	-2.908260155898
-2.134225697378	-2.907216488046
-2.133534781267	-2.905687690921
-2.13274589814	-2.905185124513
-2.132132133024	-2.903022213285
-2.131559700572	-2.902953929101
-2.130868424749	-2.898059401962
-2.130110196391	-2.896371262309
-2.129524303691	-2.896184618273
-2.128797937851	-2.895573943291
-2.127867719734	-2.895428006986
-2.127133265764	-2.891391282509
-2.126425762431	-2.891379765704

-2.125673521484	-2.891137319009
-2.124930599227	-2.890965654764
-2.124256351525	-2.885298574018
-2.123575892293	-2.884814446152
-2.122932816963	-2.884599406859
-2.122309454274	-2.883890889468
-2.121758081427	-2.883134968009
-2.121031573513	-2.882507926851
-2.120142738874	-2.881751517968
-2.119489336153	-2.88137220271
-2.118877124414	-2.88135811291
-2.11818267006	-2.880663000012
-2.11757928365	-2.878966759038
-2.116946343125	-2.878639797105
-2.116386310367	-2.878188540406
-2.11573216947	-2.876588495592
-2.115171554257	-2.876263340261
-2.114375816894	-2.875702954065
-2.113724698889	-2.873763433721
-2.112991052895	-2.873256110866
-2.112332735275	-2.872623901858
-2.111673757285	-2.872532698163
-2.111043807276	-2.872033265855
-2.110463285516	-2.871995486386
-2.109782695939	-2.871813992945
-2.109158575787	-2.870294595038
-2.108632362073	-2.867872980391
-2.107974126678	-2.86543310821
-2.107329411925	-2.864126301413
-2.10655841793	-2.862232073448
-2.106000425299	-2.862081537088
-2.105340963843	-2.861616112124
-2.104583618334	-2.861558765894
-2.103749096514	-2.858927582169
-2.10313991207	-2.858781890869
-2.102553380217	-2.855338064812
-2.101875831444	-2.855222466035
-2.101146147018	-2.852737852744
-2.100530700937	-2.851502161104
-2.099729021551	-2.850032438193
-2.099215809581	-2.849712952054
-2.098590012469	-2.849213428987
-2.097881628339	-2.848763725385
-2.097282630807	-2.848416941317
-2.096612778938	-2.848105996258
-2.096045268623	-2.84726024844
-2.095433672546	-2.846657905047
-2.094715242417	-2.846601660334
-2.094119857208	-2.846418753511
-2.093491642352	-2.845784275826
-2.092791892893	-2.845015557491
-2.092213009454	-2.843403638749
-2.091568921123	-2.842550058208
-2.090887848235	-2.841082343868

-2.090205690097	-2.84000899474
-2.089619329465	-2.838580140277
-2.088949506695	-2.835913713912
-2.08832308157	-2.835772467832
-2.087751567002	-2.832471848895
-2.086957899496	-2.831626948874
-2.08638613795	-2.830340932041
-2.085848226441	-2.829513066495
-2.085111522291	-2.828281698455
-2.084457473573	-2.828142436213
-2.083993833681	-2.827296995107
-2.083347968356	-2.826944729553
-2.082732075998	-2.826927770872
-2.082117052515	-2.826757942468
-2.081495708927	-2.826430425442
-2.080911197832	-2.825736057421
-2.080302515713	-2.825518366421
-2.079695432274	-2.82332031813
-2.079098833719	-2.82224909167
-2.078413140244	-2.82140156558
-2.077790984067	-2.821248538484
-2.077238455349	-2.820121829285
-2.076556703852	-2.820063817601
-2.076070544852	-2.819961818841
-2.075497178479	-2.819096492
-2.074861754474	-2.819085788895
-2.074199143974	-2.81847621826
-2.073688734168	-2.818255006444
-2.073087033697	-2.81781618643
-2.072518214931	-2.817644231619
-2.071966820863	-2.816297662515
-2.071248885768	-2.813992648289
-2.07067535031	-2.813044640813
-2.070105343063	-2.812252687583
-2.069318712135	-2.811934728558
-2.068630303085	-2.811548383706
-2.06799654097	-2.809840588256
-2.067339188735	-2.809011895946
-2.066738317618	-2.807353154698
-2.06617240279	-2.805426746836
-2.065484333093	-2.804864985016
-2.065027926539	-2.804567267479
-2.064346986056	-2.803269223296
-2.063803002213	-2.802936452562
-2.063165580018	-2.802440690579
-2.062559995335	-2.802197372326
-2.061974018782	-2.800624676295
-2.061447860048	-2.800603372868
-2.060933961775	-2.800207742523
-2.060439068039	-2.799876751586
-2.059900667822	-2.798468858888
-2.059242530685	-2.796335783371
-2.058574272449	-2.795940008466
-2.057898136252	-2.795904682287

-2.057245652469	-2.795542288125
-2.056620077455	-2.791223225727
-2.056014050159	-2.790039036547
-2.0553961505	-2.789434422761
-2.054815897064	-2.787787246436
-2.05427369922	-2.786415302892
-2.053743020724	-2.786256388776
-2.053260448374	-2.785958054883
-2.052590856609	-2.785781721892
-2.05199791125	-2.784469531
-2.051401529456	-2.783050853781
-2.050900640333	-2.782959714141
-2.050231843766	-2.782851281911
-2.049474017923	-2.782631635333
-2.048844606885	-2.782136933161
-2.048239687985	-2.780424871128
-2.047723465467	-2.780413811142
-2.047030961773	-2.780100258498
-2.046423302454	-2.779942110517
-2.045739071389	-2.779073456508
-2.045186895953	-2.778311231563
-2.044539202423	-2.778114349011
-2.044036347385	-2.776905023748
-2.043406915103	-2.776257535957
-2.042882684196	-2.776140715171
-2.042216722744	-2.7754791178
-2.041635129682	-2.775089509567
-2.041005027543	-2.77303193776
-2.040373506749	-2.772852808546
-2.039810350674	-2.771721529421
-2.039258805517	-2.769990354119
-2.038700261265	-2.769087581515
-2.038167844738	-2.768704222863
-2.037513211733	-2.768391919488
-2.036891735125	-2.76582881618
-2.0364193608	-2.765773460994
-2.035781724859	-2.763201600643
-2.035208982654	-2.758920723679
-2.034652340373	-2.758137882087
-2.033935809273	-2.757589205604
-2.033231054099	-2.756931347768
-2.032599087282	-2.756738253005
-2.032008180143	-2.756362954863
-2.031462051534	-2.750010902486
-2.030913031452	-2.74937655839
-2.030419914431	-2.749205457716
-2.029920619913	-2.746641947551
-2.029445623173	-2.746277642598
-2.028843346876	-2.745963135745
-2.028239799889	-2.745101565227
-2.027756744922	-2.744735391193
-2.027227361108	-2.742967079246
-2.026697271232	-2.740651219389
-2.02616536029	-2.73933076175

-2.025673982136	-2.737888007271
-2.025197200754	-2.736157671834
-2.024700517187	-2.734887809361
-2.024137651837	-2.734848215523
-2.023529553775	-2.734705566956
-2.023092096862	-2.732094885132
-2.022603293187	-2.730010873636
-2.022115225142	-2.729951451704
-2.02163862898	-2.729419375026
-2.021070905746	-2.729089588817
-2.020391497359	-2.72829482178
-2.019832528057	-2.728022369699
-2.01913241686	-2.727701351067
-2.018593506315	-2.726923866336
-2.017966984638	-2.725966538707
-2.017482671583	-2.725891271927
-2.016953760843	-2.725640073818
-2.016379339518	-2.724825537024
-2.015793270113	-2.724719893496
-2.015253715405	-2.724667477792
-2.014790156321	-2.724085010365
-2.014194220205	-2.721053189823
-2.013527484315	-2.720777815558
-2.013022454397	-2.718030145224
-2.012505738996	-2.717802021927
-2.011949796866	-2.717767237085
-2.011422847057	-2.717713736311
-2.010914129117	-2.717288338282
-2.010356335859	-2.716225721263
-2.009778545969	-2.715143440313
-2.009279890642	-2.713971627675
-2.008690110095	-2.713542043665
-2.008166934276	-2.710972812332
-2.007590574423	-2.710589589511
-2.006959517153	-2.710421987176
-2.006509619978	-2.71036334219
-2.006024584958	-2.708448985576
-2.005488927801	-2.708356694528
-2.004960595097	-2.707305972864
-2.004366918488	-2.706146786642
-2.003866483214	-2.705787527634
-2.003349496327	-2.705741839483
-2.002779562106	-2.704860919358
-2.00223605058	-2.703319728592
-2.001662977086	-2.702692523219
-2.001153314384	-2.700546217149
-2.000665663669	-2.700145689165
-2.000005996205	-2.699345898216
-1.99947459037	-2.69742647402
-1.998826125377	-2.697085189178
-1.998343203304	-2.696692714748
-1.997806842811	-2.696353786115
-1.997343868406	-2.694319884679
-1.996871630555	-2.693824207681

-1.996318663085	-2.693728742087
-1.995783997006	-2.691367887435
-1.995231500842	-2.690983995996
-1.99467603734	-2.690615376126
-1.994164392323	-2.690220374459
-1.993569642837	-2.689944353132
-1.993094108399	-2.689783497325
-1.992528946845	-2.68919382725
-1.991999079466	-2.688917301421
-1.991602175232	-2.688661879868
-1.991090055949	-2.68776172985
-1.990626332315	-2.684539091934
-1.990080857046	-2.683942089106
-1.98956411701	-2.68376085644
-1.989019013463	-2.682669585939
-1.98856449473	-2.682567712774
-1.987977377187	-2.681162645251
-1.987366544716	-2.680619919488
-1.986929861424	-2.680036073421
-1.986403037451	-2.679460080135
-1.985868593128	-2.678306949759
-1.985375947431	-2.678174068308
-1.984843629695	-2.67802494674
-1.984322517087	-2.677312679341
-1.983863159271	-2.677125693962
-1.983297296747	-2.676690554038
-1.982883810691	-2.67537926576
-1.982303057011	-2.675284550178
-1.981804921472	-2.673094211019
-1.981376383408	-2.672739788881
-1.980843067478	-2.672664592148
-1.980322664842	-2.671707792116
-1.979773739295	-2.669460692151
-1.979219143412	-2.665669816915
-1.978577987963	-2.665350649681
-1.97806504162	-2.663886057145
-1.977615338904	-2.66313085971
-1.97718550447	-2.662355202113
-1.976679881846	-2.662036887028
-1.976103371753	-2.661580890719
-1.975652528682	-2.661408575822
-1.975191160079	-2.660568780525
-1.974612460535	-2.660313484473
-1.974172842748	-2.658788192288
-1.973575750981	-2.658755251843
-1.973064891622	-2.657684578184
-1.972591508308	-2.656647830579
-1.972095763651	-2.655431993303
-1.971682320812	-2.653718379984
-1.971184454096	-2.652735450025
-1.97070530721	-2.652403933067
-1.970156423449	-2.651507986923
-1.969595365705	-2.650141508029
-1.96902388589	-2.649406170139

-1.968512189684	-2.648931755262
-1.967985656641	-2.648567423361
-1.967413131408	-2.648119133986
-1.967025256037	-2.647899448793
-1.966470284058	-2.647787164775
-1.966061055357	-2.647736872046
-1.96558066947	-2.647155950435
-1.965103868258	-2.646463287331
-1.964541486715	-2.645621853435
-1.964011903387	-2.644659259603
-1.963518848663	-2.641991292486
-1.96301803768	-2.640840583001
-1.962448418548	-2.639230983574
-1.961900333693	-2.638320134358
-1.961478171931	-2.637315100398
-1.961030588222	-2.637295777142
-1.960524910007	-2.6337854759
-1.959976705912	-2.632777361829
-1.959489781359	-2.632624988231
-1.959024151226	-2.631765753513
-1.958472311256	-2.631338919545
-1.957928570352	-2.630613781607
-1.957483873681	-2.630236648888
-1.95700394716	-2.628497047337
-1.956596453231	-2.627989212586
-1.956088387554	-2.62497004854
-1.955538370047	-2.624321112163
-1.955028503662	-2.622499301949
-1.954580135336	-2.621879232759
-1.954132526149	-2.621312036923
-1.953697183221	-2.619060488248
-1.953049322148	-2.618889519257
-1.952669618683	-2.618844406097
-1.952109981364	-2.618680431236
-1.951525867093	-2.617152146093
-1.951008735698	-2.615435229167
-1.950552048729	-2.615274137466
-1.949893938353	-2.615246759032
-1.949476516081	-2.614937159283
-1.949034242729	-2.614909051315
-1.948584409163	-2.614323782665
-1.94807890983	-2.614217987797
-1.947582465765	-2.614171023149
-1.947020364151	-2.61338862942
-1.946578095255	-2.613206239392
-1.946060465262	-2.613181336377
-1.945529792853	-2.611760586012
-1.945028803393	-2.610956679268
-1.944575808032	-2.61056100174
-1.944133052351	-2.610343053816
-1.943562242205	-2.610274830947
-1.94310917652	-2.608391005463
-1.942679227249	-2.608140647577
-1.942211432556	-2.606988847512

-1.941745126179	-2.606278757547
-1.941197050837	-2.60560430396
-1.940657735242	-2.60460860926
-1.940157660201	-2.603405959437
-1.939661411855	-2.603242155955
-1.939151465742	-2.603047765085
-1.938666258197	-2.601661822232
-1.938257039016	-2.60122577344
-1.93778336939	-2.60122100493
-1.937226392095	-2.601110657989
-1.936770470628	-2.60090232294
-1.936279867776	-2.600749507012
-1.935784702881	-2.600746862909
-1.935281003077	-2.600547082452
-1.934763758238	-2.600433645535
-1.934189820799	-2.599140388163
-1.933736185751	-2.596059860592
-1.933277514757	-2.595419088459
-1.932828151584	-2.595263132216
-1.932338014431	-2.593951584544
-1.931872870021	-2.593772632755
-1.931394584735	-2.592694063302
-1.93092112091	-2.592019543382
-1.930492690243	-2.591841541093
-1.930020959141	-2.591448833389
-1.92962913509	-2.591371993405
-1.929059750969	-2.588817047282
-1.928615611732	-2.588666608009
-1.92803810406	-2.588215276335
-1.927484627385	-2.587381674266
-1.927027036352	-2.586049786472
-1.926620529165	-2.585976109745
-1.926136894939	-2.584548283134
-1.925681595543	-2.582748443527
-1.925146283232	-2.582511888999
-1.924779229527	-2.582305529393
-1.924426280405	-2.581985422784
-1.923890745785	-2.581816772591
-1.923386764723	-2.580928916114
-1.922883264617	-2.579788344421
-1.922254537534	-2.579333024513
-1.921790450097	-2.579086239877
-1.921364468054	-2.578859655361
-1.920922072789	-2.57618324069
-1.920433433193	-2.57573285696
-1.919894922089	-2.574330964068
-1.9195070865	-2.572636857657
-1.919028378144	-2.571553996177
-1.918591378296	-2.569139541279
-1.918146507841	-2.568847973972
-1.917740381525	-2.568725607395
-1.917302877363	-2.568632105299
-1.916836570328	-2.568562341292
-1.916417699413	-2.568066782271

-1.915977861623	-2.567314095212
-1.915544343852	-2.567270088066
-1.915035576838	-2.564544540597
-1.914519624605	-2.560792268993
-1.914150172104	-2.558985813939
-1.913728087067	-2.558127617462
-1.913169432053	-2.554950761761
-1.912638279944	-2.554478451551
-1.912186389671	-2.554237582257
-1.911740142964	-2.553932350915
-1.911300592426	-2.553910050742
-1.910813220411	-2.553180282698
-1.910321980633	-2.552833581543
-1.909906854999	-2.552407728788
-1.909456958781	-2.550588829285
-1.908968043823	-2.549912242144
-1.908459563303	-2.5484444833
-1.908016684877	-2.548414687039
-1.907491986151	-2.547901150098
-1.907064964384	-2.547415521411
-1.90655403029	-2.547191968898
-1.906109609304	-2.547184722797
-1.90567977489	-2.547112966132
-1.9052622662	-2.544764374331
-1.904856060013	-2.544758179976
-1.904341175332	-2.543623325117
-1.903836926857	-2.543414436394
-1.903408203621	-2.538335801825
-1.902966509499	-2.537847715704
-1.902438863398	-2.537600650496
-1.901894099959	-2.535402141776
-1.901406796543	-2.534899600418
-1.900984140821	-2.533022557972
-1.900570873229	-2.532924940149
-1.900028180195	-2.532709573959
-1.899581932405	-2.53229086634
-1.899092932937	-2.531681631629
-1.898579571995	-2.530079382152
-1.898041214998	-2.529932020888
-1.897651494241	-2.529881175308
-1.897250394027	-2.529845751699
-1.896755386469	-2.529213179123
-1.896323173808	-2.529100776371
-1.895798196731	-2.528341284591
-1.895381754751	-2.527449242802
-1.894945062099	-2.526799265244
-1.89458837466	-2.526302742435
-1.89403968342	-2.526288932928
-1.893601561563	-2.526272474085
-1.89321453802	-2.526081158992
-1.89278794707	-2.525949016469
-1.892277972097	-2.525824961798
-1.89182233901	-2.525079533855
-1.891381065483	-2.525019933707

-1.890949272162	-2.524621853327
-1.890544997202	-2.524516587131
-1.890134091519	-2.521975566508
-1.889689811697	-2.521551224171
-1.889284294924	-2.52045499238
-1.888857415793	-2.518399934099
-1.888422557077	-2.516548734179
-1.888046267384	-2.515829947588
-1.887532864827	-2.515571186669
-1.887079956266	-2.514553294621
-1.886603122896	-2.514492829623
-1.886138791446	-2.514023178639
-1.885665526609	-2.512084750325
-1.885190564826	-2.511836249587
-1.884660878988	-2.511817968548
-1.88418666063	-2.509931120434
-1.883729455487	-2.509590323543
-1.88326796173	-2.509364021111
-1.88289053569	-2.509216768409
-1.882428276696	-2.507901849879
-1.881921949143	-2.507090375853
-1.881479086553	-2.505868259932
-1.880974695471	-2.505847681046
-1.880498902579	-2.505376704568
-1.880007768256	-2.504079280896
-1.879611991	-2.503722331398
-1.879086307217	-2.503586013263
-1.878669282057	-2.503072422054
-1.878271079943	-2.502926478219
-1.877830938474	-2.501325637714
-1.877434136713	-2.50120314433
-1.876982058698	-2.501042800401
-1.87664396498	-2.500863832742
-1.876223115575	-2.500730262373
-1.87578372574	-2.500721013804
-1.875347925118	-2.500528445977
-1.874969670265	-2.498273068402
-1.874544483493	-2.496462491046
-1.874112726152	-2.495196629112
-1.873690039586	-2.494807029953
-1.873327162466	-2.494382727521
-1.872853600722	-2.493324242072
-1.872421729664	-2.492995239149
-1.872067443297	-2.492622510546
-1.871728261759	-2.492390773458
-1.871283129584	-2.492312358197
-1.870982499335	-2.490756177263
-1.870634247203	-2.489900157288
-1.870149909839	-2.488973257099
-1.869685136312	-2.488250919721
-1.869285110509	-2.486831303261
-1.868826217065	-2.485794415497
-1.868348480964	-2.484953272136
-1.86797431045	-2.483618644208

-1.867521173103	-2.4832747042
-1.867011953514	-2.483246742576
-1.866590222262	-2.482282649028
-1.866172037538	-2.482259737049
-1.865750710374	-2.481725251579
-1.865374046389	-2.479960874001
-1.864954035411	-2.479803500873
-1.864507972106	-2.478779828509
-1.864021662996	-2.477566717711
-1.863633110312	-2.477499580942
-1.863190985155	-2.477436273101
-1.862831629171	-2.476660489162
-1.862466553189	-2.475803187802
-1.862037801531	-2.472781627871
-1.861630553552	-2.4726662186
-1.861245540001	-2.472565107572
-1.860817493641	-2.471374213652
-1.860442074065	-2.471087646216
-1.860033533055	-2.470666857887
-1.859613638299	-2.470376242281
-1.859074761201	-2.469769813665
-1.858725282964	-2.469160433193
-1.858307952802	-2.468535222339
-1.857863124646	-2.466603408986
-1.857467140246	-2.466148943742
-1.856975239531	-2.465870505515
-1.856612521975	-2.465648004217
-1.856201227289	-2.464510187539
-1.85575551086	-2.463774332081
-1.855388849364	-2.462237302715
-1.854956646935	-2.46219027798
-1.85459568134	-2.461542099785
-1.854184843308	-2.460822902255
-1.853689146365	-2.460807971992
-1.853261227087	-2.459915724191
-1.852908107382	-2.45863880732
-1.852484398922	-2.45851459741
-1.852018804703	-2.458303469205
-1.851584775701	-2.458035958619
-1.851120734855	-2.457914294288
-1.850622259885	-2.457471904426
-1.850166203326	-2.457225457536
-1.849777367765	-2.456876737101
-1.849355258215	-2.456781395257
-1.84895907577	-2.455394162407
-1.848593125513	-2.455078489251
-1.848109987094	-2.454798200397
-1.847671310938	-2.454722851253
-1.847200644832	-2.453820659522
-1.846803970167	-2.453629737188
-1.846324118424	-2.45294755908
-1.845906188009	-2.452914096397
-1.845535194545	-2.452626726901
-1.845027769983	-2.451998403148

-1.844617919753	-2.450558139511
-1.844142780804	-2.450471583647
-1.843820026028	-2.450324498641
-1.843413045905	-2.449350734926
-1.842959771821	-2.448192793958
-1.842675270678	-2.447853493188
-1.842247925994	-2.446637513034
-1.841718271494	-2.446590577354
-1.841299729718	-2.446367469025
-1.840862172011	-2.445951821521
-1.840433143749	-2.445615131487
-1.839975387694	-2.445004086669
-1.839570794515	-2.444363039142
-1.839162101182	-2.444155962629
-1.838608542485	-2.443453795221
-1.838261887868	-2.44339896639
-1.837795335011	-2.443265361935
-1.83740421401	-2.443135608986
-1.836956901515	-2.442671936716
-1.836560599907	-2.441898581157
-1.836152201656	-2.441503382765
-1.835711722027	-2.440717426908
-1.8353612168	-2.440273832278
-1.834946144439	-2.440226706692
-1.834533730996	-2.438334476989
-1.834186741671	-2.437686885501
-1.833765811529	-2.437234214515
-1.833383433033	-2.436758469894
-1.83303416552	-2.436580528221
-1.832630148621	-2.436492278172
-1.832278615861	-2.436373282323
-1.831897114005	-2.436254259284
-1.831511028788	-2.436105590236
-1.831091318798	-2.435835743062
-1.83071719032	-2.431303888324
-1.830276681461	-2.430914731992
-1.829897576717	-2.430331765059
-1.829536309176	-2.42925399572
-1.829189271576	-2.427118357371
-1.82884470833	-2.425700853106
-1.828432492647	-2.425619996334
-1.828014670289	-2.425169683332
-1.827601182295	-2.425119395744
-1.827290562811	-2.424744055617
-1.826876067713	-2.424054599551
-1.826420092809	-2.420688430461
-1.825905844106	-2.4205243745
-1.825549223862	-2.419256382855
-1.825090986808	-2.418810028267
-1.824722284543	-2.418634511584
-1.824364884564	-2.418581395857
-1.824012185041	-2.418091324162
-1.823638687009	-2.418020491491
-1.823268004467	-2.417834090574

-1.822860272488	-2.416076177244
-1.822477603095	-2.415460489675
-1.822093775739	-2.413540689696
-1.821616018263	-2.413009565471
-1.821275303981	-2.412449516549
-1.820879748619	-2.412127520417
-1.820462461849	-2.412105309
-1.820125801259	-2.41201602175
-1.819705393127	-2.411158524
-1.819381740641	-2.410663487253
-1.818990331412	-2.41056456676
-1.818618240859	-2.409805431891
-1.818159491984	-2.40979022024
-1.817743850719	-2.409109127925
-1.817334048988	-2.409096510354
-1.816846217212	-2.407984320799
-1.816527948819	-2.407931109493
-1.816134420199	-2.407592497141
-1.815791879804	-2.406397410546
-1.815366599407	-2.4061552805
-1.814965452015	-2.406141117156
-1.814637595922	-2.40540332292
-1.814239929574	-2.404768288569
-1.813846107964	-2.404244736754
-1.813499924525	-2.40375892183
-1.813164587211	-2.403739104606
-1.81271693995	-2.403584696538
-1.812367156656	-2.402806402033
-1.81197028468	-2.401984773205
-1.811436732383	-2.401040901065
-1.810930462159	-2.40045744762
-1.810539016365	-2.400433388551
-1.810141332734	-2.400138505832
-1.809787040214	-2.400067228082
-1.809386746373	-2.399919425727
-1.809040216338	-2.399515426785
-1.808611009822	-2.399248186893
-1.808211371542	-2.399008656043
-1.807868112316	-2.396894103813
-1.807379728294	-2.396484588011
-1.807050878384	-2.395318312392
-1.806682223042	-2.39449233877
-1.806397287934	-2.392630590645
-1.806027831608	-2.392478652761
-1.80559437308	-2.392040639616
-1.805227888846	-2.391339425851
-1.804876630702	-2.39025450935
-1.804481141557	-2.389253304779
-1.804096808056	-2.389147356781
-1.803734976414	-2.388306660657
-1.803335791704	-2.387344119633
-1.802917295265	-2.386492989998
-1.802539953293	-2.386353814733
-1.802188678472	-2.385820785242

-1.801789587613	-2.385490941168
-1.801411483166	-2.385106289555
-1.801083714606	-2.384792568967
-1.800761617969	-2.384073805545
-1.800371241315	-2.383581655901
-1.799934410753	-2.381308717088
-1.799554742099	-2.381098551449
-1.799177801543	-2.380043915255
-1.798730613401	-2.379988016224
-1.798363337161	-2.379668149383
-1.79805641762	-2.379561335103
-1.797674658385	-2.378398602694
-1.797292048902	-2.378006266905
-1.79698395241	-2.377201616672
-1.796643005946	-2.376538889719
-1.796250212367	-2.37634195337
-1.795937458441	-2.375901014461
-1.795547663181	-2.375727817111
-1.795153554776	-2.375358461576
-1.794767274217	-2.373175976954
-1.79439800142	-2.372599619291
-1.794057647647	-2.372420726063
-1.793688664339	-2.371699307635
-1.793298161123	-2.370031373626
-1.792815278338	-2.369801926864
-1.792472136231	-2.36959246147
-1.7920515695	-2.369568368385
-1.791713443399	-2.369303161566
-1.791286597262	-2.36916288833
-1.7909620669	-2.366892841816
-1.790561718602	-2.365570807838
-1.790199245018	-2.365463700398
-1.789852797803	-2.365017461557
-1.789470759181	-2.363701208902
-1.789197485005	-2.363522123805
-1.788833778461	-2.362088051135
-1.7884323791	-2.362065282932
-1.787980860094	-2.362061199126
-1.787612555785	-2.361982142783
-1.78720741832	-2.361833205015
-1.786804995503	-2.361129808584
-1.786461667483	-2.360751189608
-1.786126083769	-2.360062573888
-1.785780296835	-2.359891973881
-1.785434900974	-2.359492830188
-1.785026348075	-2.358839518838
-1.784680726106	-2.358246389453
-1.784378796817	-2.357803087873
-1.784081922981	-2.357514387734
-1.783728263105	-2.357236701213
-1.783429716924	-2.357201082854
-1.783054566905	-2.356243083597
-1.782648953074	-2.356018837698
-1.782301134635	-2.355003187712

-1.781982778896	-2.354296291877
-1.781644300788	-2.353718715101
-1.781273971433	-2.353476865682
-1.780926130875	-2.350872660769
-1.780559695756	-2.350323403815
-1.7802627574	-2.350217638418
-1.77988849047	-2.349388875715
-1.779471848618	-2.349153754029
-1.779080669965	-2.348767560702
-1.778717470803	-2.347741025224
-1.778341898343	-2.347512136463
-1.777991974653	-2.34746535929
-1.77767807492	-2.347137280243
-1.777363077418	-2.346433784116
-1.77699049323	-2.345713341326
-1.77660764478	-2.345083908872
-1.776294223083	-2.34446912161
-1.775949570451	-2.344274678726
-1.775635694084	-2.342917671136
-1.775277802053	-2.342566274746
-1.7749639509	-2.342196876629
-1.77465936863	-2.340842091857
-1.774231137817	-2.339397580707
-1.773860563014	-2.339277839878
-1.773469755799	-2.339071121693
-1.77310564455	-2.338707810205
-1.772772778164	-2.337603595148
-1.77231080128	-2.336721048472
-1.771956723964	-2.336512830007
-1.771609626544	-2.335836814198
-1.771254888737	-2.335139930151
-1.770955379504	-2.334763759599
-1.770578801158	-2.334071022667
-1.7701548144	-2.333232889242
-1.769815510856	-2.333226335893
-1.769456998889	-2.332897624897
-1.769053712979	-2.332546510043
-1.768660676012	-2.332501475778
-1.768299513122	-2.332072707374
-1.767971051199	-2.331824744026
-1.767566518259	-2.331575508481
-1.767202317325	-2.331362479413
-1.766908372041	-2.331091776412
-1.766554526883	-2.328906264548
-1.766213509178	-2.327501423533
-1.765785138734	-2.32737226961
-1.765485064855	-2.327248759355
-1.76513916444	-2.32683395456
-1.764847786518	-2.326541513457
-1.764506815138	-2.325587636674
-1.764180378518	-2.324977512413
-1.763839450338	-2.324874503787
-1.763522938781	-2.323644027981
-1.763179162839	-2.323192028356

-1.762769586922	-2.322891899467
-1.762454483133	-2.32266602612
-1.762130126121	-2.321980712474
-1.761763310562	-2.320980200176
-1.761408217761	-2.320499532479
-1.76106070749	-2.319534232868
-1.760650057011	-2.31916747365
-1.760360462844	-2.318694570376
-1.76010897005	-2.31850575423
-1.75974082459	-2.318363498734
-1.75946898837	-2.317526203206
-1.759120081494	-2.317479128103
-1.758837619041	-2.316824648589
-1.758461361732	-2.31672361216
-1.758160561408	-2.315810251729
-1.757793902963	-2.315616239043
-1.757447254954	-2.315080678743
-1.757097827985	-2.314957707426
-1.756780529544	-2.314788600156
-1.756455100332	-2.314784832294
-1.756187078451	-2.314510429344
-1.755848084281	-2.314348947683
-1.755459331957	-2.313144808668
-1.755153318424	-2.312904563887
-1.754772604919	-2.31258074481
-1.754417496634	-2.3099676128
-1.754064829281	-2.309963064953
-1.753654644198	-2.309681347098
-1.753273352574	-2.309471638239
-1.752950923858	-2.309136087527
-1.752592096417	-2.308473528896
-1.752185620807	-2.3082316638
-1.751850086824	-2.308200742827
-1.751508042506	-2.308065634009
-1.751173399133	-2.30762724904
-1.750795344312	-2.307613356003
-1.750445702705	-2.307585538554
-1.7500546944	-2.306339216256
-1.749690941409	-2.305581675669
-1.749388226332	-2.304682073389
-1.749063024708	-2.304458458255
-1.748681338595	-2.302813820311
-1.748356888637	-2.302575088883
-1.747966138831	-2.302386116044
-1.747597587202	-2.301346228916
-1.747228316377	-2.300719764478
-1.746917192692	-2.300531957064
-1.746644957524	-2.299825591635
-1.746308695006	-2.299164980196
-1.746000712151	-2.298879040999
-1.745626813335	-2.298815968065
-1.745341613375	-2.29859347873
-1.745045212688	-2.29848573658
-1.744665278948	-2.298203004862

-1.744232344811	-2.297856758924
-1.743806653568	-2.297772973562
-1.743356992639	-2.297767994787
-1.743038840617	-2.297178750574
-1.742693577311	-2.296500257131
-1.742363115521	-2.296271530641
-1.741943598628	-2.295440800437
-1.741593960002	-2.295350419185
-1.741275667335	-2.295261737088
-1.740921377014	-2.295180329764
-1.740529949625	-2.29460888794
-1.740197956781	-2.29451153176
-1.739800422245	-2.294386228868
-1.739517666863	-2.294095440654
-1.739159934529	-2.293900489089
-1.738831366021	-2.293627612477
-1.738499180348	-2.292716758247
-1.738150721912	-2.292658302866
-1.737818752525	-2.292537114736
-1.737520443113	-2.292101974442
-1.737182485969	-2.290803200961
-1.736838491462	-2.29022198666
-1.73650706314	-2.290080784779
-1.73617291595	-2.289845124045
-1.735859577228	-2.287892912539
-1.735495904707	-2.287638943764
-1.735085043208	-2.287488880577
-1.734741731748	-2.287303996123
-1.734377947149	-2.286751468319
-1.733974076938	-2.285900132568
-1.733616089218	-2.284830326333
-1.733303997818	-2.284525453269
-1.732990943945	-2.284067551624
-1.732627997196	-2.283974691011
-1.732299116972	-2.283100361171
-1.731955332818	-2.282548856341
-1.731599252782	-2.280453260691
-1.731243847336	-2.279045608282
-1.730848755595	-2.278775013104
-1.730530573443	-2.278194150831
-1.73014395999	-2.277993746915
-1.729839666605	-2.277483914743
-1.729538343275	-2.277360410308
-1.729223247929	-2.277236342154
-1.728899390843	-2.277225704924
-1.728507135097	-2.27713726215
-1.728184249881	-2.276944792334
-1.727792192309	-2.276768815906
-1.727487015349	-2.276757718921
-1.727134319454	-2.275811872702
-1.726820946211	-2.275763135119
-1.726521732315	-2.273784393072
-1.726161088242	-2.27378258078
-1.725787704499	-2.273740367968

-1.725391507024	-2.272921141319
-1.725042996987	-2.272808028987
-1.724707403768	-2.272085773681
-1.72434232643	-2.271665849514
-1.724046408002	-2.271033915
-1.723667466964	-2.269804762663
-1.723303930094	-2.269575516979
-1.722901297825	-2.268534175004
-1.722540899629	-2.268272342399
-1.722202983384	-2.2681388106
-1.721878815985	-2.267895505909
-1.721632817731	-2.267631680041
-1.7212184349	-2.266927853807
-1.720885724078	-2.266889210194
-1.720534739974	-2.266601251112
-1.720237388822	-2.266124778714
-1.719871531463	-2.265952178119
-1.719577748413	-2.265874010933
-1.719245780704	-2.26552815017
-1.718923690233	-2.265468552785
-1.718587451322	-2.264907107645
-1.718262403815	-2.264096712312
-1.717910778155	-2.263942083839
-1.717563814227	-2.262376853243
-1.717308259887	-2.261944769171
-1.716981368569	-2.261327599076
-1.71665055306	-2.260758326291
-1.716309217638	-2.259426918295
-1.715976390659	-2.258914317318
-1.715681746494	-2.258537470148
-1.715238223567	-2.256978511642
-1.714836168626	-2.256712227804
-1.714463680815	-2.256001918786
-1.714109531032	-2.255805849796
-1.713768482126	-2.255581296951
-1.713388998359	-2.255378550364
-1.713077116068	-2.254874792549
-1.712706665088	-2.253739715295
-1.712340077901	-2.251786058961
-1.712049415221	-2.251093050105
-1.711773716586	-2.249832109866
-1.711440020575	-2.249251242476
-1.711127197587	-2.24829989013
-1.71086280382	-2.247620294685
-1.710577322384	-2.247432141415
-1.710205106414	-2.247265272749
-1.70989128771	-2.246513943558
-1.709560813759	-2.24636863733
-1.709300679921	-2.246156291438
-1.708952199464	-2.246110559538
-1.708669832546	-2.244536946439
-1.708329953671	-2.243436868331
-1.708053403429	-2.241981712318
-1.707723410849	-2.241762080595

-1.707452089344	-2.241670653729
-1.707139802009	-2.240973621242
-1.706789917625	-2.240276686608
-1.706474987507	-2.240273781759
-1.706118623169	-2.239941581212
-1.705839324253	-2.238129893244
-1.705497735209	-2.238054810524
-1.705206473007	-2.237127466532
-1.704921389182	-2.236370411713
-1.704645862971	-2.236144834182
-1.704317090162	-2.235757536236
-1.70397156715	-2.235167315622
-1.703592129686	-2.234789837105
-1.703295867681	-2.234651577663
-1.702948951001	-2.234381911753
-1.702585247749	-2.234092332174
-1.702251055519	-2.233562456536
-1.701846462644	-2.233200852011
-1.701455451969	-2.23275337657
-1.701181089595	-2.232517253102
-1.700863549576	-2.232288987509
-1.700556128044	-2.23184162992
-1.700157935848	-2.231477751099
-1.699876555128	-2.230953687561
-1.699585466042	-2.230227126471
-1.699201627008	-2.230196103386
-1.698861517583	-2.229947030324
-1.698556479081	-2.229039002401
-1.698272194994	-2.228724055623
-1.697980316541	-2.228485184198
-1.697600814997	-2.228080961188
-1.697338803197	-2.228021573169
-1.697040071457	-2.227525362976
-1.696693530688	-2.227341392221
-1.69638460072	-2.227168421217
-1.696043654142	-2.226762758518
-1.695680393868	-2.225695154138
-1.695412836126	-2.224684809787
-1.695141138493	-2.224635618072
-1.694881634326	-2.224483235425
-1.69454331154	-2.223827430182
-1.69421729066	-2.223778118431
-1.693860529264	-2.223569829768
-1.693569331692	-2.222936526516
-1.693180355517	-2.222723799497
-1.692865310023	-2.221480593852
-1.692530347562	-2.219839432694
-1.692266897371	-2.21927805353
-1.691989846764	-2.219245068693
-1.691705755273	-2.216801392394
-1.69135676843	-2.216007103383
-1.690984804433	-2.215325842551
-1.690657077083	-2.215164771366
-1.690361370859	-2.215132547023

-1.690024816479	-2.213503789308
-1.689807025743	-2.213329879019
-1.689513312163	-2.212953415311
-1.689221939119	-2.212051854095
-1.688860506162	-2.211729610295
-1.688547127814	-2.211629543836
-1.688263529243	-2.210839721788
-1.687922172751	-2.210763160076
-1.687609183458	-2.209254219832
-1.68731893592	-2.209074957694
-1.687025447462	-2.209043297343
-1.686720286239	-2.208471150839
-1.686379586804	-2.206649092154
-1.686063012261	-2.206220236229
-1.685801579081	-2.204903627665
-1.685508416533	-2.203169157471
-1.685223179644	-2.202450942636
-1.684888497953	-2.20218654108
-1.684610720797	-2.201978330068
-1.684246936661	-2.201789668111
-1.683881973238	-2.201343481772
-1.683643798722	-2.200894805644
-1.68333399573	-2.199827304359
-1.683038537153	-2.19971622966
-1.682738538071	-2.199649928532
-1.68247069832	-2.198881376688
-1.682162556917	-2.198879435605
-1.681849053588	-2.198568448691
-1.681566419304	-2.198301305454
-1.681262820193	-2.198242335735
-1.680934668636	-2.198078322236
-1.680589984863	-2.197637044663
-1.680312428375	-2.197484372939
-1.680048817071	-2.195277369021
-1.679710185175	-2.195263414012
-1.679411377519	-2.194575037311
-1.67913307576	-2.194211886959
-1.678838521172	-2.194199138317
-1.678587350975	-2.193779203326
-1.67826418956	-2.193608517195
-1.677925139263	-2.193454593857
-1.677623315185	-2.192477943418
-1.677364305927	-2.190852403862
-1.677090453306	-2.190643214307
-1.676757435458	-2.189210859333
-1.676469021585	-2.188803345334
-1.676196436251	-2.188706860182
-1.675957175904	-2.187556698671
-1.675649332764	-2.187106465326
-1.67533937838	-2.186650667974
-1.675037467807	-2.186498439198
-1.674770366821	-2.186424530926
-1.674478863898	-2.185603968056
-1.674158083224	-2.185577117959

-1.673901602752	-2.184373635633
-1.673567351458	-2.184254020685
-1.673287045351	-2.183291695509
-1.67296183113	-2.183114003102
-1.672567480427	-2.182845721686
-1.672235187129	-2.181384922952
-1.671859293876	-2.181163490768
-1.671539720334	-2.181085478483
-1.671237432029	-2.181010032373
-1.670954773602	-2.180671673967
-1.670652605643	-2.17952303808
-1.670374890868	-2.179362993233
-1.67005992743	-2.179086080854
-1.669732899357	-2.17798205404
-1.669365410412	-2.176693162914
-1.669024870155	-2.175353292125
-1.668656968259	-2.175183482732
-1.668382641557	-2.17516411223
-1.668069734301	-2.175000679849
-1.667780579573	-2.174966577569
-1.667451207248	-2.174723323229
-1.667179226008	-2.174575203222
-1.666920700493	-2.174238174275
-1.666621813449	-2.173720149941
-1.666379176876	-2.172274402124
-1.666088848047	-2.172035391975
-1.665749829415	-2.171733364475
-1.665401858787	-2.171049252142
-1.66510497629	-2.170490405781
-1.664847602531	-2.170080681115
-1.664503590914	-2.169899498516
-1.664168889941	-2.169679530699
-1.66387392248	-2.169304222798
-1.663622809215	-2.169113511365
-1.66325772655	-2.168430929389
-1.662980845645	-2.16839053916
-1.662715332344	-2.168085592489
-1.662437876727	-2.167428117576
-1.662147856606	-2.166469025493
-1.661805380627	-2.166366544281
-1.661486130943	-2.166326091053
-1.661200354204	-2.166147701195
-1.660884558438	-2.166058713807
-1.660535791741	-2.165413243607
-1.660212622178	-2.165213578535
-1.659896363479	-2.164743646957
-1.659604695238	-2.16451497306
-1.659225013646	-2.163965990694
-1.658977604558	-2.163563832102
-1.658700668452	-2.163114033486
-1.658397639383	-2.162664499858
-1.658137626602	-2.162602968531
-1.657865118404	-2.162147203777
-1.65757276362	-2.162023264111

-1.657192408032	-2.160501630742
-1.656937862699	-2.160122547836
-1.656642347053	-2.159948332013
-1.656288032674	-2.159681602628
-1.65597869358	-2.159421545608
-1.655705058821	-2.158967011694
-1.655375395752	-2.15877932802
-1.655154996631	-2.15716167494
-1.654863359803	-2.157101780497
-1.65455230838	-2.157033054887
-1.654203293105	-2.156838997144
-1.653890795636	-2.156630015476
-1.653594821462	-2.155476086661
-1.653266541194	-2.155233435055
-1.652985604155	-2.154198915846
-1.652697746099	-2.154154439986
-1.652359254388	-2.153397169548
-1.652120888364	-2.152921716839
-1.651846919946	-2.152906232358
-1.651586067453	-2.152034897617
-1.651278914729	-2.151534957776
-1.650997333178	-2.151095420438
-1.650723138377	-2.150907586599
-1.650432802795	-2.15048385236
-1.650180547796	-2.150099311436
-1.649883572552	-2.149409507958
-1.649546874828	-2.149134836016
-1.649259051304	-2.148294787189
-1.64893788662	-2.146727812931
-1.648610831172	-2.145794555565
-1.648319585141	-2.144273494288
-1.64801507686	-2.142952331857
-1.647739778684	-2.142551022105
-1.64739077374	-2.142270107328
-1.647116462858	-2.142015961666
-1.646749838676	-2.140911484479
-1.646451947409	-2.140354251507
-1.646171389903	-2.140243198156
-1.645847665327	-2.139301981193
-1.645569426996	-2.138925885735
-1.64519758613	-2.138555626311
-1.644829266591	-2.137920894044
-1.644527275387	-2.137755366027
-1.644172325202	-2.137096998134
-1.64384997232	-2.136913676219
-1.643544781293	-2.135233323504
-1.643265394623	-2.134624384788
-1.642905322772	-2.134050794888
-1.642609877404	-2.134013450603
-1.642326056997	-2.13363872954
-1.64196497794	-2.132465303326
-1.641623450785	-2.131592093567
-1.641334245419	-2.131121454123
-1.640986014029	-2.129575532641

-1.640569793825	-2.129278162009
-1.640250356692	-2.128954264258
-1.639997107991	-2.12881696933
-1.639709526588	-2.12854286169
-1.639410594968	-2.128493228455
-1.639175371115	-2.128068141453
-1.638887267918	-2.127998850858
-1.638579006432	-2.127846222418
-1.638304003998	-2.127144356122
-1.638061221254	-2.126617605159
-1.637804054573	-2.126436591341
-1.637525021139	-2.125928713299
-1.637204339319	-2.125876131094
-1.636926634642	-2.124212343102
-1.636650272384	-2.12361220898
-1.636349276608	-2.123599663521
-1.636081801461	-2.123536325095
-1.635787232784	-2.12353104349
-1.635503719836	-2.123333508485
-1.635238046965	-2.123075716511
-1.63494881213	-2.122786630776
-1.634611154982	-2.121480075861
-1.634294867049	-2.120646918208
-1.634019806889	-2.120507383035
-1.633760142705	-2.119527490742
-1.633450263249	-2.119144273197
-1.633200977786	-2.119078875523
-1.632936522106	-2.119063133234
-1.632636980793	-2.116968149733
-1.632402129233	-2.116430119126
-1.63215280176	-2.116063194878
-1.631824418232	-2.115477082904
-1.631588992664	-2.114666992595
-1.631267330225	-2.114233514073
-1.630959866629	-2.113910990391
-1.630705750769	-2.113869959218
-1.630365149428	-2.113399617239
-1.630089654552	-2.113356933306
-1.629831717081	-2.113258800604
-1.629539692991	-2.112777228754
-1.629274857389	-2.112519672486
-1.629000180464	-2.112000941385
-1.628723578407	-2.111877166603
-1.628396557054	-2.111493749669
-1.628111373685	-2.110843320236
-1.627729157249	-2.110252279898
-1.627440861292	-2.109995806574
-1.627120393882	-2.109663751267
-1.626855244944	-2.109623626169
-1.62660574328	-2.109307598495
-1.626316185775	-2.108253008591
-1.62604264106	-2.107396616214
-1.62576423022	-2.107133153145
-1.625515646432	-2.105359468946

-1.625231880717	-2.104288770877
-1.624885039133	-2.102814221165
-1.624572279009	-2.102778567271
-1.624325003662	-2.10277513992
-1.624066429455	-2.102341437678
-1.623772568663	-2.101966572914
-1.623475461496	-2.101870053462
-1.623226859972	-2.10169705449
-1.622921678443	-2.101153683264
-1.622615973708	-2.101127138082
-1.622298947086	-2.101081623532
-1.622045637938	-2.100590435819
-1.621788443131	-2.099493173574
-1.621520996373	-2.099198331638
-1.621248531264	-2.099005606304
-1.620968175719	-2.098321426573
-1.620689353399	-2.098282442695
-1.62043964724	-2.098112605056
-1.620162791833	-2.09796681098
-1.619936521592	-2.097747393329
-1.61964006225	-2.096433921921
-1.619431297154	-2.096226723789
-1.619165644222	-2.096068981792
-1.618886433671	-2.095441577248
-1.618576309629	-2.094692377408
-1.618307389409	-2.0944891797
-1.617998797785	-2.094404688337
-1.617754151262	-2.094266932949
-1.617519937504	-2.094056832149
-1.617202990907	-2.093403318711
-1.616912624226	-2.093317503035
-1.616609871274	-2.092831727295
-1.616358536923	-2.092180991604
-1.616117094266	-2.091927713637
-1.61580155241	-2.091032918157
-1.615458009936	-2.090100496509
-1.615210238404	-2.089418757924
-1.614974238089	-2.08922645862
-1.614662242383	-2.089200599694
-1.614420125092	-2.08873324894
-1.614175671693	-2.088433714189
-1.613881227862	-2.088393563311
-1.613576682531	-2.088211922059
-1.613290274971	-2.088071233325
-1.613031966119	-2.087603072278
-1.612780972787	-2.087202495205
-1.612483714345	-2.086549578331
-1.612195033774	-2.085546632014
-1.611909601238	-2.085285175686
-1.611622143153	-2.085260587511
-1.611411469329	-2.084288771783
-1.611169139448	-2.083951501023
-1.610921645286	-2.083040158806
-1.610576070585	-2.082922820109

-1.610307688378	-2.082395599321
-1.610034385747	-2.082357595363
-1.609793694137	-2.08217190529
-1.609520745869	-2.081614125828
-1.609302777354	-2.081233288529
-1.608968885106	-2.081131638651
-1.608674581641	-2.081118824732
-1.608423494549	-2.080516067715
-1.608122629857	-2.07948623776
-1.607836745165	-2.07929273254
-1.607604860737	-2.079203174145
-1.607300615617	-2.078659462076
-1.6070397647	-2.078593431147
-1.606727687767	-2.077753813396
-1.606492330494	-2.077750655079
-1.606218184652	-2.077623119771
-1.605917215802	-2.077279110235
-1.605669974686	-2.077067745347
-1.605386307091	-2.076488275583
-1.60509382552	-2.07638291854
-1.604810602084	-2.07601132055
-1.604502565973	-2.075543702839
-1.604234305923	-2.075295049615
-1.60397273227	-2.075173967447
-1.603695058612	-2.074770159524
-1.603410957926	-2.074345231385
-1.603119084447	-2.074206998453
-1.602863790705	-2.073735881661
-1.6025936177	-2.073406086427
-1.60233833059	-2.073106132567
-1.602042837216	-2.073063827025
-1.601763557295	-2.073007591276
-1.601468720295	-2.072686556534
-1.601202403551	-2.072277728298
-1.600933063609	-2.072152042786
-1.600684966644	-2.071240706998
-1.600436141121	-2.071158650174
-1.600159119836	-2.071148464362
-1.599900592759	-2.07105498135
-1.599601725759	-2.069446191077
-1.599317704764	-2.06884710687
-1.599074112798	-2.068462473137
-1.598833202497	-2.068407535021
-1.59854825864	-2.068256007382
-1.598267936298	-2.067774668319
-1.597982366966	-2.067773359271
-1.597720695432	-2.066895739255
-1.597493941223	-2.06674507518
-1.597212142478	-2.066529394428
-1.596972950108	-2.066003008718
-1.596684372863	-2.065946302075
-1.596425430601	-2.06577826901
-1.59620279713	-2.065734925926
-1.595947395961	-2.064994185253

-1.595618108002	-2.064715034621
-1.595374371244	-2.064634963195
-1.595092351358	-2.064514051785
-1.594824934095	-2.064216845343
-1.594539896968	-2.064019778434
-1.594270264603	-2.063981862222
-1.593959191789	-2.063844194235
-1.59364018811	-2.063511464333
-1.593346627615	-2.063149502629
-1.593015867525	-2.06244737042
-1.592724473311	-2.062270058567
-1.592481108961	-2.062073372918
-1.592240690665	-2.061505108712
-1.591937615525	-2.060637382897
-1.591682431195	-2.060213583045
-1.591422275395	-2.059000249898
-1.59117283121	-2.058120640485
-1.590907678448	-2.05803012358
-1.590690435377	-2.057069882238
-1.590404624668	-2.056988110023
-1.590075404466	-2.05677949177
-1.589806241934	-2.056437968575
-1.589570894934	-2.054661359889
-1.589260639035	-2.054600626233
-1.589040315679	-2.054563622733
-1.588765508584	-2.054096135402
-1.588487172776	-2.054037153253
-1.588197034334	-2.053853464077
-1.587903217085	-2.053395483705
-1.587628209874	-2.05308043756
-1.587357671471	-2.052381719359
-1.587113889598	-2.051634156232
-1.586839863387	-2.051112626621
-1.586589043089	-2.051092317363
-1.58633536816	-2.05064051428
-1.586062093037	-2.050538914874
-1.585822110692	-2.049253918554
-1.585544915807	-2.048828063353
-1.585243790861	-2.047233893963
-1.584968462894	-2.047070013071
-1.584708868201	-2.046903614647
-1.584491739438	-2.045770345853
-1.584245803274	-2.045556341952
-1.583954702264	-2.044995760135
-1.583728579959	-2.044717653705
-1.583466414888	-2.044231133699
-1.583204369326	-2.043737630898
-1.582926042371	-2.043319662992
-1.582616600071	-2.043074746657
-1.582336211081	-2.042835150775
-1.582003066021	-2.042709268606
-1.581762340907	-2.042665786566
-1.581524952587	-2.042560616321
-1.581253140973	-2.042147798413

-1.580993360944	-2.042114650204
-1.580728802508	-2.041836529924
-1.580495648404	-2.041728044914
-1.580236311785	-2.040959592024
-1.580002325485	-2.04061237057
-1.579730474863	-2.040543086707
-1.57945347713	-2.040424093705
-1.57922675304	-2.040260093234
-1.578966346899	-2.039847756552
-1.578644438375	-2.039751043472
-1.578383767047	-2.039552624654
-1.578140783498	-2.039223665314
-1.577860558808	-2.038806946681
-1.577590482525	-2.038368727296
-1.577357650777	-2.038353628286
-1.57706926776	-2.037844108129
-1.576811838199	-2.037138254974
-1.576569951555	-2.036972651203
-1.57631745735	-2.036290729862
-1.57602604683	-2.036215545624
-1.575727101708	-2.035783386684
-1.575481382855	-2.035747313621
-1.575216245912	-2.035072901931
-1.574971058947	-2.035053537663
-1.574723259166	-2.034854667746
-1.574466951641	-2.03457236572
-1.574207856606	-2.034154016857
-1.573953631232	-2.033871611626
-1.57369675198	-2.033565568559
-1.57344165624	-2.03339302643
-1.5732113331	-2.033274942211
-1.572932281323	-2.032565585219
-1.572635909973	-2.032338951911
-1.57238220671	-2.032235258884
-1.572111200392	-2.031645341739
-1.571877670409	-2.030901611046
-1.571615874394	-2.03070021951
-1.571354352893	-2.030517939588
-1.571072310318	-2.030009758881
-1.570839085073	-2.029844203435
-1.570554139173	-2.029372039581
-1.570310701608	-2.029176290304
-1.570049369549	-2.028894382291
-1.569801879813	-2.028244883725
-1.569517548831	-2.028113910122
-1.569222636512	-2.028046425116
-1.568971531019	-2.027889511628
-1.568777901382	-2.026687796664
-1.56848432913	-2.025858549809
-1.568180501346	-2.025194269001
-1.567949217293	-2.024985293324
-1.567738434186	-2.023789709039
-1.567478409783	-2.02376869627
-1.567230649253	-2.022840890593

-1.566974198807	-2.02278896691
-1.566765627775	-2.022556987534
-1.566467953131	-2.022427933943
-1.566210736653	-2.022385473231
-1.566044495088	-2.021397575396
-1.56581378958	-2.020651605046
-1.565590115624	-2.020365697447
-1.565354801935	-2.019695915436
-1.565110594963	-2.019047879742
-1.564868042428	-2.018166747584
-1.564607412605	-2.018119718636
-1.564349192282	-2.018099666094
-1.564064960274	-2.017918309394
-1.563802988888	-2.01774197739
-1.563535650978	-2.017320595975
-1.563277474295	-2.017092373289
-1.563003775143	-2.01708116592
-1.562666586772	-2.01557378018
-1.562433199976	-2.01556360646
-1.562161285275	-2.015525169409
-1.561882187542	-2.0153245424
-1.561636174676	-2.014957811263
-1.561379237842	-2.014636900804
-1.561084880798	-2.014050745824
-1.56082853071	-2.013797073156
-1.560571553778	-2.013773079953
-1.560366741934	-2.013312021787
-1.560127462232	-2.013171080613
-1.559893033762	-2.012848494649
-1.559659737351	-2.010895827745
-1.559394473099	-2.010861489032
-1.559173023111	-2.01080635396
-1.558903961705	-2.010729767676
-1.558631055825	-2.010320750775
-1.558352278721	-2.010312106827
-1.55811992664	-2.010189323859
-1.55784876438	-2.009845020108
-1.55755568609	-2.009637488596
-1.557290615161	-2.009358098463
-1.557048764542	-2.009273214045
-1.556824439952	-2.009249906832
-1.556605888018	-2.008103582252
-1.556382263086	-2.007434527797
-1.556103027913	-2.007264425862
-1.555874235328	-2.007156446117
-1.555614636795	-2.007040316712
-1.555337037818	-2.006863473366
-1.55509787915	-2.006243113015
-1.554881258006	-2.006019065669
-1.554636267799	-2.005808154663
-1.554384154655	-2.005771406467
-1.554156042656	-2.005122397961
-1.553911931907	-2.005100388293
-1.553674790817	-2.004938901214

-1.553400347545	-2.004756270145
-1.553144269712	-2.004603013553
-1.552878575619	-2.004448017604
-1.552587612092	-2.003978203743
-1.552298784276	-2.003852260029
-1.552082709712	-2.003435238995
-1.551785769032	-2.003076758756
-1.55153548515	-2.003010808223
-1.551295235523	-2.00300784886
-1.551052359312	-2.003002504058
-1.550812373689	-2.002929402196
-1.550523371222	-2.0026437005
-1.550250345693	-2.002251584751
-1.549998255554	-2.001909209134
-1.549777222437	-2.001768917182
-1.549474649198	-2.001680364997
-1.549265588957	-2.001564818477
-1.548997336874	-2.001501110846
-1.54873392013	-2.001482796677
-1.548441453815	-2.001306214451
-1.548229369066	-2.001000394164
-1.547979780655	-2.000934739481
-1.547718100687	-2.000459992155
-1.547465276038	-2.000402012656
-1.547236406648	-1.999487323086
-1.547033963784	-1.999377013154
-1.546795779255	-1.99867828685
-1.546536969475	-1.998415476043
-1.546298538041	-1.998214941817
-1.546094980104	-1.998123296529
-1.545885584331	-1.997422559727
-1.545605884445	-1.99739009575
-1.545369984745	-1.997257184277
-1.545133132786	-1.997082193144
-1.544859335976	-1.996584237988
-1.544628854137	-1.996469617316
-1.544362627209	-1.996303245941
-1.544147963787	-1.996220223327
-1.543884274083	-1.996071014534
-1.543555995854	-1.995677204786
-1.543268033308	-1.995269103933
-1.543050533315	-1.995203423238
-1.542825707369	-1.9940223321
-1.542573357377	-1.993968467936
-1.542316472746	-1.99330039023
-1.542134856401	-1.99239173337
-1.541905599112	-1.991804020506
-1.54164161588	-1.99041986194
-1.541403284097	-1.990272851094
-1.5411631939	-1.989280739995
-1.540970043079	-1.989189491121
-1.540736387716	-1.988886015585
-1.540481296899	-1.988233262073
-1.540221733433	-1.987784538679

-1.539968084503	-1.987748820098
-1.539701223489	-1.987334422677
-1.539435164764	-1.987065970066
-1.539147224353	-1.986768927762
-1.538931885003	-1.986751269516
-1.538703689228	-1.986493170484
-1.538483709833	-1.985745029081
-1.538217889922	-1.985181515968
-1.538003350942	-1.985029366911
-1.537779817409	-1.984897539755
-1.537535525569	-1.984702579542
-1.537242442986	-1.984696389067
-1.537005642029	-1.984641111194
-1.536797660909	-1.984081038488
-1.536590464429	-1.983883202351
-1.53628481598	-1.983811304633
-1.53604444595	-1.983025957502
-1.535773534378	-1.982731788715
-1.535537495935	-1.982633161655
-1.535266697576	-1.982520506535
-1.535022433757	-1.981843086485
-1.534818578743	-1.98181367696
-1.534572018763	-1.981154602389
-1.534293351015	-1.978833518183
-1.53403693282	-1.978807119217
-1.533774229208	-1.978336559234
-1.533482075771	-1.976800777252
-1.533243320839	-1.976766821806
-1.533005363225	-1.97654189403
-1.532752845994	-1.976117430946
-1.532574635452	-1.976077123441
-1.532319206336	-1.976006186978
-1.532061795362	-1.975532135737
-1.531794243252	-1.975256921792
-1.53159865543	-1.974299530173
-1.531347718525	-1.974228842892
-1.53108438321	-1.974191975202
-1.530876962381	-1.973995310329
-1.530642949122	-1.973812786586
-1.530395274243	-1.973638160335
-1.530121139878	-1.973612660208
-1.529852355433	-1.972292864939
-1.529644763142	-1.971638299102
-1.529428342359	-1.971076755405
-1.529147894953	-1.970950095852
-1.528888369741	-1.970615021614
-1.528646133624	-1.970412401431
-1.528403984351	-1.970356462377
-1.52814949064	-1.970296049486
-1.527922081165	-1.970273997483
-1.527673529713	-1.970226246039
-1.527438827205	-1.970054914918
-1.527216104962	-1.969137788164
-1.527006264196	-1.968556750151

-1.526808958014	-1.968265828787
-1.526564553941	-1.967896070163
-1.526342134093	-1.967894815289
-1.526053190735	-1.967859635932
-1.525835061121	-1.967479243873
-1.525586473015	-1.967338922321
-1.525291283936	-1.967145738754
-1.525069330218	-1.966780158324
-1.524794955092	-1.965970943246
-1.524566017545	-1.965925629799
-1.524343057449	-1.965806798242
-1.524053864621	-1.965079587914
-1.523798300213	-1.96492419843
-1.523586990635	-1.964796014296
-1.5233609855	-1.9647882086
-1.523080729682	-1.964602515094
-1.522841547979	-1.964315414831
-1.522570497552	-1.963967550545
-1.522344053898	-1.963731110484
-1.52210336636	-1.963683921438
-1.521865754577	-1.963367638528
-1.521670165481	-1.96259014064
-1.521435754914	-1.962310471611
-1.521207287559	-1.962183908098
-1.520916877308	-1.961867306949
-1.520677371017	-1.961685182559
-1.520489957838	-1.96105482691
-1.520230818592	-1.960619796499
-1.520030384617	-1.959964895147
-1.519799231685	-1.959903408005
-1.51954866758	-1.959743299951
-1.519298042868	-1.959663086461
-1.519065261594	-1.959082186576
-1.518847475827	-1.959063230257
-1.518648309597	-1.959040036708
-1.518441618048	-1.958757928278
-1.518191765287	-1.95874597897
-1.517988709579	-1.958733543788
-1.517748128771	-1.958450144038
-1.517525684676	-1.958107568006
-1.517327116029	-1.957671379301
-1.517056913394	-1.957372330075
-1.516829674683	-1.957123736888
-1.516598080713	-1.956618753147
-1.516332492179	-1.956524759872
-1.516062355884	-1.956044092095
-1.515827904676	-1.955906541792
-1.515570768942	-1.955888072754
-1.515370011832	-1.955653834656
-1.515143257708	-1.95385764531
-1.514913827729	-1.953463917956
-1.514670143425	-1.953237084476
-1.514463318698	-1.952123935775
-1.514255453142	-1.951895340646

-1.513971907241	-1.951409926242
-1.513734988779	-1.951293692449
-1.513503039751	-1.951053476954
-1.513339972102	-1.950787262241
-1.513112067878	-1.95072793175
-1.51288671362	-1.950661623895
-1.512657905	-1.950180119684
-1.512402835179	-1.949917915397
-1.512190682029	-1.949839368361
-1.511929640855	-1.949404877381
-1.511722998831	-1.949197621333
-1.51149448808	-1.948623264628
-1.511280570054	-1.948511586687
-1.511026316683	-1.948467926119
-1.510807678287	-1.948248580167
-1.510573923641	-1.948245661835
-1.510317238577	-1.94806563705
-1.510067839482	-1.946832669677
-1.509898259925	-1.94591723868
-1.509654647357	-1.945666220172
-1.50941384127	-1.945323251704
-1.509194788574	-1.945052512441
-1.508947466023	-1.94480416026
-1.508698245761	-1.944666828284
-1.508451395233	-1.943964057654
-1.508241473546	-1.94346871076
-1.507944974137	-1.943410236856
-1.507721965904	-1.942897663537
-1.507502482467	-1.942697174072
-1.507260376507	-1.942565064091
-1.507027257246	-1.942325406158
-1.506798190072	-1.94222394456
-1.506571332788	-1.941212628646
-1.506316061904	-1.94086751737
-1.506052017839	-1.94027020421
-1.505810613308	-1.939735206345
-1.50555633138	-1.939639958272
-1.505270861233	-1.939564387879
-1.505043219253	-1.939510524621
-1.504786701836	-1.939252622359
-1.504553412093	-1.938892539833
-1.504308938373	-1.938694240671
-1.504070447602	-1.938227461451
-1.503834226546	-1.937588829456
-1.503591093914	-1.937520915233
-1.50331006196	-1.936619043887
-1.503060305388	-1.936505927935
-1.502831771938	-1.93646756465
-1.502586537108	-1.936426578654
-1.502369982982	-1.935941326805
-1.50213777414	-1.93496358202
-1.501867345576	-1.934632638809
-1.501691747884	-1.934574346535
-1.50147598779	-1.934532066369

-1.501268834536	-1.934523923955
-1.50102451021	-1.93450905117
-1.500785956332	-1.933831927704
-1.500509099055	-1.933802274959
-1.500298712362	-1.932963398326
-1.500087722147	-1.932879749289
-1.499850787012	-1.932698456287
-1.49965736291	-1.932548795091
-1.499447174115	-1.93228437287
-1.499213703062	-1.930986493524
-1.499008528386	-1.930938165617
-1.498784491016	-1.930878735977
-1.498552563281	-1.930123388223
-1.49831389843	-1.930102638043
-1.498060279828	-1.929669820209
-1.497818307289	-1.92928843172
-1.497630987368	-1.929250744654
-1.497427473558	-1.928234460365
-1.497139261189	-1.928032840091
-1.496905265306	-1.927952702138
-1.496660425651	-1.927802569829
-1.496461882266	-1.927636371275
-1.496262864451	-1.92669961708
-1.496043121307	-1.926160999903
-1.4958377556	-1.925992322495
-1.495621166938	-1.924956090798
-1.495395837612	-1.924694214315
-1.495148751475	-1.924690844149
-1.49495143985	-1.924138856185
-1.494737506709	-1.923811361051
-1.494506853527	-1.923635727969
-1.494276123654	-1.923355950252
-1.494038597176	-1.923160379488
-1.493813987983	-1.922849386938
-1.49353800979	-1.922649856348
-1.493308811849	-1.922213177765
-1.493087673274	-1.922062870336
-1.492871812333	-1.922058179084
-1.492627180147	-1.921872010627
-1.492329098591	-1.921464758355
-1.49204910702	-1.920925577514
-1.491802459697	-1.920896716521
-1.491621909332	-1.920736521246
-1.491385282432	-1.920601146716
-1.491192651701	-1.920274190578
-1.490966899378	-1.919337959817
-1.490756022098	-1.91850234177
-1.490561725018	-1.918272143182
-1.49032151191	-1.918259674596
-1.490072427826	-1.918250725071
-1.489846730455	-1.91754149766
-1.489646643349	-1.917518554244
-1.489427454624	-1.917315965308
-1.489191103886	-1.917176810408

-1.488965714819	-1.916954009345
-1.488745630462	-1.916553584264
-1.488497496548	-1.916477397406
-1.488194276405	-1.916060550876
-1.487960373756	-1.915410603122
-1.487686772215	-1.914642071321
-1.487471110506	-1.913929768576
-1.487186812299	-1.913914905337
-1.486953719474	-1.913458889369
-1.486724634394	-1.912996835656
-1.48650633345	-1.912950378131
-1.486279135147	-1.912924508031
-1.486034611611	-1.912641468885
-1.485804048396	-1.912472089968
-1.485579396679	-1.912457490604
-1.485383015101	-1.912337633719
-1.485181978694	-1.912046258385
-1.484937072489	-1.911621154068
-1.484719246276	-1.911533952203
-1.484535274541	-1.911518591893
-1.484332071561	-1.911486208116
-1.484137099916	-1.911482666482
-1.483935803443	-1.910992777281
-1.483719121209	-1.910649829201
-1.483537882559	-1.910537277932
-1.483311452992	-1.909926873199
-1.483068727875	-1.909076496768
-1.48281884384	-1.908526972361
-1.48261229293	-1.908349256089
-1.482363539277	-1.908237502767
-1.482185585653	-1.908185755019
-1.481943107935	-1.907923127398
-1.481688028064	-1.907466505022
-1.481478176633	-1.907358203277
-1.481286367382	-1.907267529412
-1.481066295296	-1.90726248883
-1.480890734164	-1.90547780162
-1.48062056646	-1.905105759133
-1.480351480563	-1.904767718468
-1.480135331194	-1.904045692959
-1.479939866453	-1.904028979798
-1.479733114993	-1.903858125342
-1.479553151362	-1.903591056162
-1.479345203821	-1.902850135966
-1.479163274335	-1.902707170209
-1.47893860275	-1.902704847044
-1.478735310793	-1.902700681646
-1.478525073133	-1.90269046341
-1.478315381035	-1.902480586207
-1.478027520319	-1.902361961716
-1.477807680832	-1.902324419882
-1.477551044334	-1.901857418571
-1.477357944713	-1.900320378192
-1.477143090363	-1.900227588106

-1.476927711083	-1.899704965826
-1.476687919805	-1.899467309588
-1.476460005652	-1.899195407593
-1.476252950248	-1.899089438681
-1.476047027233	-1.89907633253
-1.475830447132	-1.898533097706
-1.475584043839	-1.898247914983
-1.475427292085	-1.898053255462
-1.475232357651	-1.897376607856
-1.475008380874	-1.897353928318
-1.474774093126	-1.89721770195
-1.474511237166	-1.896986043128
-1.474292240742	-1.896872690095
-1.474064562157	-1.896686085688
-1.473838492073	-1.896635853501
-1.473609484209	-1.896044342962
-1.473389978794	-1.895689434037
-1.473147358588	-1.895679107767
-1.472928483721	-1.895429246942
-1.472670393412	-1.894898096337
-1.472463534484	-1.894728643219
-1.4722456858	-1.894379958023
-1.472003319323	-1.893849723455
-1.471768195846	-1.893129839019
-1.471518262382	-1.892368561871
-1.471301515094	-1.891703317123
-1.471067221282	-1.891554193568
-1.470861771062	-1.891535507142
-1.470582883085	-1.8910805163
-1.470406544699	-1.890556390378
-1.47017751951	-1.890205557098
-1.469948962983	-1.88970184789
-1.469681495979	-1.889682666423
-1.469468620323	-1.889643370586
-1.469232470315	-1.888955234305
-1.469014749818	-1.888799694429
-1.46883212132	-1.88860079577
-1.468573416364	-1.888236220981
-1.468353403197	-1.888208316605
-1.468140937529	-1.887916333453
-1.467947749827	-1.887823439146
-1.467734413244	-1.886693037796
-1.467472006309	-1.885968308824
-1.467249875439	-1.88559212839
-1.467054348965	-1.885270141776
-1.466854033094	-1.885209008542
-1.466644490519	-1.885149791462
-1.466430153715	-1.884788620281
-1.466226834896	-1.884313303221
-1.466002597991	-1.883977913368
-1.465783373339	-1.883857972209
-1.465535146997	-1.88368886452
-1.465329397147	-1.882957022684
-1.465102400572	-1.882909927129

-1.464904866268	-1.882826559131
-1.464713196976	-1.882812547868
-1.464510181144	-1.882760724125
-1.464299263027	-1.882149243697
-1.464109521218	-1.881890427557
-1.463886252844	-1.881700140209
-1.46365943054	-1.88158831522
-1.463386271248	-1.88151212314
-1.463188338977	-1.881230674066
-1.462985446224	-1.880887236718
-1.462784920852	-1.879715537459
-1.462568807392	-1.879486159859
-1.462347296729	-1.87928721599
-1.462115451027	-1.879234957329
-1.4619183048	-1.879081950127
-1.461709543172	-1.879002108569
-1.461409384532	-1.878926546836
-1.461204883821	-1.878759459519
-1.461009463444	-1.878663370403
-1.460766526692	-1.878268963075
-1.460486462977	-1.877353818753
-1.460253001725	-1.876451628162
-1.46006954229	-1.876143281097
-1.459887659783	-1.875737568186
-1.459696497072	-1.875303345786
-1.459487467203	-1.874631639606
-1.459263548006	-1.872163021332
-1.459061009351	-1.872148956075
-1.458865833142	-1.871973587158
-1.458639994076	-1.871966003862
-1.458438259051	-1.871435581834
-1.458235277971	-1.870807545506
-1.457987725459	-1.870767253448
-1.457783776355	-1.870758540542
-1.457555754657	-1.870338461868
-1.457349378317	-1.870098161331
-1.457087027309	-1.870036021831
-1.45687111065	-1.869093437764
-1.456596161802	-1.868945420731
-1.456399272556	-1.86887724623
-1.456153726264	-1.86824329923
-1.455897930927	-1.867945210713
-1.45569778038	-1.867603229397
-1.455517496285	-1.867543203004
-1.455297517749	-1.866861031837
-1.455089951649	-1.866547033704
-1.454864810465	-1.866437307935
-1.454672161988	-1.866282669155
-1.454452828485	-1.866263384932
-1.454260306635	-1.866076322202
-1.454074883205	-1.865798699115
-1.453817217746	-1.865727964561
-1.453587687649	-1.865111119171
-1.453406142524	-1.864792082831

-1.453193333445	-1.864462704085
-1.453000708319	-1.864427630143
-1.452762936823	-1.864007622858
-1.452560033076	-1.863119497327
-1.452376068752	-1.86296945463
-1.452178757394	-1.862852647848
-1.451920958115	-1.862628381231
-1.451719788522	-1.861969542643
-1.451466079722	-1.861759119052
-1.451164207783	-1.861099851792
-1.450975923789	-1.86048569326
-1.450795274441	-1.859826000836
-1.450544421167	-1.859629704863
-1.450311347556	-1.859382272454
-1.450093445289	-1.858317500264
-1.449858461536	-1.857619280878
-1.44964098695	-1.857350484716
-1.449410542542	-1.857094678961
-1.44920109373	-1.856420265922
-1.449005227258	-1.856412479109
-1.448790987134	-1.85621689345
-1.448630572935	-1.856182491209
-1.448410076787	-1.856025207735
-1.448177115738	-1.855663061822
-1.447979115392	-1.855555880156
-1.447746730488	-1.855358351828
-1.447560693942	-1.855307605312
-1.447356130738	-1.85509951245
-1.447090207606	-1.854672327588
-1.446908098349	-1.854632418542
-1.4466820838	-1.854171569965
-1.446444304237	-1.853898150972
-1.446225445088	-1.852525029419
-1.446005670162	-1.852035794532
-1.445769056546	-1.851994689008
-1.445528742313	-1.851564228302
-1.445328793122	-1.851538397844
-1.445112967655	-1.850681797007
-1.444886517363	-1.850633633479
-1.444662365549	-1.850417746709
-1.444478272477	-1.850307382999
-1.444231365809	-1.8498305549
-1.444019379657	-1.84982842928
-1.443807640029	-1.849720622111
-1.443600084812	-1.849599422638
-1.443398137998	-1.849176247002
-1.443172609977	-1.849004805611
-1.442948082792	-1.848895713244
-1.442751698921	-1.848749911927
-1.44252912663	-1.848561530926
-1.442263067029	-1.848287987802
-1.44207820016	-1.848111676779
-1.441840614964	-1.847894266176
-1.441613582713	-1.847330322825

-1.441381779652	-1.846684470378
-1.441181712589	-1.846073052282
-1.440986625081	-1.845290724873
-1.44078235626	-1.845027694094
-1.440582092143	-1.844764839434
-1.440374480588	-1.84473662006
-1.440166098158	-1.844565411757
-1.439944982786	-1.844218724083
-1.43970629548	-1.843982962081
-1.439499385996	-1.843815002088
-1.439301377696	-1.843704821509
-1.439137810787	-1.843626548135
-1.438904283449	-1.843387985715
-1.438690614378	-1.842537699649
-1.438500426469	-1.842299562798
-1.438263607473	-1.842270388651
-1.438012089945	-1.842262970115
-1.437809061766	-1.842125566963
-1.437598660503	-1.842004931707
-1.437371371718	-1.841726079152
-1.437147395421	-1.84164788141
-1.436927262857	-1.841626677145
-1.436728620824	-1.841390161747
-1.436532474441	-1.840896181802
-1.436333025942	-1.840817983962
-1.436089942464	-1.840557903571
-1.435891312449	-1.840507469916
-1.435688286658	-1.83980463694
-1.435511896701	-1.839391659231
-1.435272480073	-1.838892572237
-1.435076239994	-1.837574510394
-1.434855357241	-1.83713895428
-1.434654624797	-1.83663609015
-1.434445229237	-1.836217181875
-1.434216258178	-1.83599672725
-1.434006275692	-1.835868294842
-1.4338256202	-1.835868010495
-1.433616039819	-1.83577900523
-1.433387376282	-1.835575350778
-1.433170390619	-1.834520257139
-1.432982927536	-1.834491622099
-1.432807976913	-1.834324499561
-1.432573373776	-1.833115352418
-1.432378403967	-1.832769974921
-1.432123040259	-1.832686500846
-1.431900712557	-1.832649650028
-1.43168768507	-1.832473268009
-1.431500444967	-1.832404692614
-1.431338114471	-1.832274515241
-1.431159643961	-1.8321798352
-1.430939830808	-1.831944590848
-1.430717844949	-1.831848040659
-1.430509213279	-1.831605264454
-1.430340531356	-1.831542395332

-1.430136991029	-1.831262732811
-1.429899746202	-1.831194456596
-1.429699934317	-1.831029712236
-1.429516661809	-1.830900889881
-1.429317922705	-1.830458582075
-1.429135813832	-1.829697799619
-1.428901589915	-1.828802207076
-1.428703600397	-1.828649296003
-1.428504641938	-1.828512367681
-1.428283816784	-1.828099215513
-1.428089888128	-1.827778644526
-1.427879653249	-1.827744406353
-1.427649338516	-1.827512195921
-1.427447110077	-1.827011466635
-1.427283310282	-1.826758798444
-1.42708370531	-1.826449503134
-1.426885249599	-1.825874354473
-1.426623987401	-1.825775441729
-1.426398349643	-1.825667678592
-1.426188550763	-1.824377201058
-1.425963084477	-1.824355288741
-1.425739618787	-1.824231587611
-1.425532820907	-1.823608195821
-1.425298387701	-1.823474808383
-1.425128883442	-1.823369674744
-1.42493965639	-1.823216215881
-1.424729859343	-1.823126969246
-1.424531853694	-1.821551116886
-1.424311178075	-1.82083383382
-1.424099543775	-1.820747946393
-1.423855778433	-1.820242111059
-1.423622584147	-1.820049121033
-1.423426838626	-1.819844836281
-1.423207549542	-1.819740738442
-1.423021434367	-1.819305991635
-1.422835549055	-1.81924974969
-1.422655798939	-1.819202916294
-1.422429886626	-1.819177958032
-1.422235628379	-1.819115112167
-1.422040542046	-1.818942236617
-1.421809751722	-1.818708746208
-1.421631524337	-1.818680003695
-1.421423440139	-1.8184976882
-1.421170344867	-1.818028896375
-1.42097281262	-1.817941883254
-1.420766156111	-1.817840291477
-1.420612208091	-1.817250391574
-1.420414830176	-1.817199666051
-1.420214074962	-1.816699649613
-1.420008091598	-1.816578414137
-1.419822727611	-1.816296231914
-1.419593801458	-1.816204876099
-1.419362850033	-1.815758685957
-1.419112368248	-1.815723320458

-1.418870539309	-1.815176285246
-1.418627128973	-1.814453110545
-1.418400518367	-1.814225508025
-1.418217595235	-1.814155185894
-1.418030773017	-1.81386682322
-1.417818472184	-1.813808287236
-1.417613629944	-1.81334262153
-1.417425208895	-1.813049460352
-1.417246246541	-1.812562131699
-1.417092594697	-1.811709805109
-1.416855025651	-1.811443133636
-1.416662409087	-1.810955650048
-1.416455847244	-1.810806938172
-1.416219629307	-1.810727673425
-1.416000123994	-1.810365947861
-1.415797333631	-1.810062808482
-1.415582361684	-1.809649773965
-1.415353992379	-1.809012000156
-1.415126808097	-1.808924323678
-1.414951999106	-1.808820381583
-1.414749156011	-1.808405526987
-1.414533648431	-1.808064879815
-1.414283259005	-1.808002784713
-1.414049033649	-1.807997101394
-1.413839497981	-1.807975682267
-1.41363897705	-1.807142918836
-1.413433446055	-1.806536046775
-1.413201632084	-1.80632860967
-1.412987471732	-1.80620492206
-1.412777099925	-1.806009768912
-1.412589900283	-1.80582449417
-1.412374060226	-1.80570789191
-1.412176266442	-1.805569735619
-1.411961900413	-1.805284714747
-1.411725526134	-1.804872117374
-1.41149033856	-1.804650348173
-1.411268397031	-1.804506878126
-1.411063967145	-1.804323657173
-1.410859637963	-1.804305845012
-1.410669799432	-1.803028009854
-1.410485152587	-1.802992053615
-1.410224350971	-1.802596869945
-1.410025417204	-1.802133171834
-1.409820505726	-1.802099901749
-1.409604714797	-1.801985464953
-1.409394184205	-1.801978908897
-1.409177492905	-1.801584655904
-1.408945248791	-1.801286725868
-1.408758317455	-1.801150920197
-1.408570000293	-1.800614127758
-1.408379206725	-1.800559103195
-1.408160730382	-1.799881875113
-1.407935468466	-1.799862348829
-1.407713530834	-1.799259135422

-1.407495375581	-1.798865428878
-1.407308863395	-1.798569697745
-1.407114419547	-1.798550420041
-1.406911971591	-1.798453393006
-1.406748799314	-1.798295141981
-1.406528256497	-1.798277427656
-1.406343419848	-1.797880500211
-1.406117647303	-1.797636789655
-1.405927087947	-1.797595844278
-1.405752821312	-1.797184705952
-1.405516924058	-1.797173386194
-1.405287548933	-1.797002387878
-1.405090366639	-1.7969719993
-1.404889984422	-1.796599868703
-1.404706987104	-1.796372861702
-1.40451638849	-1.79629717187
-1.404287743533	-1.7961303112
-1.40411270436	-1.796051303409
-1.403940244662	-1.795955541565
-1.403773290575	-1.795849447699
-1.40356378263	-1.795126278415
-1.403358657979	-1.794957378408
-1.403173513116	-1.794880782035
-1.402956311669	-1.794622872249
-1.402739894442	-1.794508824327
-1.402550942434	-1.794195818574
-1.402384130928	-1.794083047574
-1.402241674103	-1.793491816942
-1.402031682064	-1.793395597092
-1.401873536795	-1.792798503392
-1.401661696839	-1.792613625867
-1.401477434844	-1.792440706604
-1.401253883676	-1.79191531679
-1.401031304734	-1.791681728915
-1.400823513845	-1.79165420857
-1.400634602013	-1.79155721626
-1.400419793035	-1.790576715372
-1.400226308491	-1.790560861719
-1.400079527803	-1.788326843323
-1.399868255131	-1.788098335271
-1.399666287981	-1.788051131555
-1.399429509428	-1.787767005488
-1.399253460661	-1.787707810819
-1.399057606506	-1.787634096431
-1.398875729564	-1.787551750628
-1.398693638056	-1.78750290036
-1.398498746017	-1.787370876555
-1.398278414911	-1.787126486603
-1.398113878887	-1.787116260819
-1.397938268744	-1.787058690243
-1.397767185818	-1.786938068359
-1.397578015155	-1.786905129897
-1.397355649275	-1.786644947833
-1.397163393951	-1.786613805725

-1.396962810962	-1.786607657083
-1.396747339927	-1.785795909519
-1.396523100153	-1.785590324445
-1.396315011829	-1.783914299315
-1.396098975822	-1.783369372321
-1.395845124924	-1.783343877674
-1.39562911504	-1.783109554531
-1.39539895737	-1.782109034678
-1.395187417896	-1.781426060902
-1.395008903101	-1.781351485194
-1.39477715891	-1.781276376115
-1.394604403732	-1.780568959398
-1.394415629905	-1.780438526264
-1.394216002021	-1.780035951297
-1.394011747831	-1.779952691977
-1.393811077731	-1.779743258911
-1.39362295243	-1.779645608195
-1.393448990717	-1.779548783704
-1.393280658164	-1.779380347362
-1.393088808669	-1.779060866003
-1.39287521926	-1.778649220802
-1.392691116392	-1.778489896811
-1.392494781833	-1.778372044059
-1.392330055353	-1.777787177032
-1.392140827291	-1.77770129364
-1.391930672721	-1.777627044595
-1.391754587831	-1.777322263515
-1.391543554323	-1.777238747196
-1.391318739331	-1.777091727942
-1.391157003543	-1.776974518157
-1.390950903422	-1.776238302688
-1.390753338286	-1.775790805871
-1.390559213816	-1.775779374528
-1.390356663644	-1.775728898501
-1.390168997526	-1.775575658165
-1.389937441987	-1.775157002837
-1.389754227428	-1.775090986597
-1.389578658236	-1.774388348682
-1.389383849638	-1.774293614202
-1.38921182611	-1.77420791328
-1.389017374015	-1.774148964084
-1.38880631174	-1.77362239377
-1.388618695618	-1.773383670888
-1.388440982813	-1.773228633303
-1.3882184034	-1.772912876181
-1.387999113299	-1.772848914887
-1.387853180052	-1.772633124096
-1.387670527234	-1.772600597161
-1.387460387111	-1.772193890533
-1.387224807201	-1.772134445158
-1.387039279227	-1.771452468749
-1.386853135008	-1.771304709034
-1.386680066997	-1.771175797592
-1.386506091025	-1.770325330666

-1.386287477119	-1.770256676183
-1.386109148827	-1.769836639667
-1.385927122088	-1.769608264991
-1.385701754674	-1.769370641948
-1.385490733064	-1.769164823935
-1.385290515753	-1.768961004852
-1.385092889204	-1.768326618199
-1.384901932183	-1.768169410432
-1.384669570879	-1.768107393005
-1.384444827934	-1.768049385898
-1.384194536876	-1.767995657675
-1.38400742015	-1.767935768042
-1.383792483659	-1.767775909587
-1.3835802684	-1.767331584269
-1.38339729958	-1.767234904541
-1.383197603175	-1.767140187653
-1.382981772677	-1.767010596992
-1.382807702157	-1.766508437438
-1.382602148595	-1.766277926795
-1.382359365363	-1.766258917385
-1.382212405637	-1.765825083364
-1.381986477289	-1.765331859685
-1.381803096232	-1.764632666685
-1.381598443969	-1.764440238859
-1.381400414098	-1.764198158012
-1.381192037296	-1.764175143831
-1.380977430244	-1.763260358073
-1.380769532314	-1.763114936325
-1.380623555869	-1.762737651644
-1.380385183925	-1.76199549544
-1.38016690547	-1.76193524699
-1.379966639155	-1.761488506951
-1.379770595783	-1.76146466832
-1.379563479145	-1.761430778018
-1.379373085756	-1.761029981612
-1.379146006853	-1.760746585582
-1.378949948757	-1.760669354795
-1.378761905105	-1.760650255513
-1.378573662548	-1.760585957845
-1.378393741495	-1.760309072209
-1.378223578205	-1.760225591249
-1.378058777755	-1.759949530568
-1.377899309875	-1.759933374463
-1.377654111074	-1.759442098467
-1.377469692649	-1.758261215198
-1.377263306409	-1.757596555326
-1.377069708859	-1.757562117525
-1.376854630813	-1.757314672567
-1.376668992077	-1.757306696744
-1.376496533112	-1.756864923643
-1.376309172232	-1.756758752142
-1.376111947452	-1.75665228195
-1.375923520207	-1.756626611489
-1.375731113166	-1.756532609651

-1.375564674773	-1.756395509472
-1.375354337199	-1.755997293692
-1.375152786946	-1.75584182219
-1.374976477612	-1.755766056352
-1.374822284425	-1.755629423772
-1.374632631512	-1.7553209912
-1.37449735234	-1.754492210556
-1.374285505602	-1.754429287238
-1.374090434386	-1.754294160517
-1.373902607219	-1.754246888973
-1.373700531945	-1.754218121916
-1.373500713295	-1.753753191573
-1.373277884277	-1.753421981527
-1.373065079885	-1.753367403712
-1.372837461314	-1.7533057684
-1.372667740125	-1.753167206329
-1.372441119886	-1.753091332679
-1.372230282905	-1.752873967677
-1.372054713168	-1.752812324328
-1.371867161605	-1.752585223044
-1.371688007966	-1.752544148166
-1.371514312768	-1.75242622959
-1.371298012821	-1.752373650603
-1.371087210867	-1.751205555958
-1.3709292561	-1.751060631644
-1.370718247252	-1.750685736434
-1.370514391163	-1.750133259711
-1.370303500509	-1.749608946388
-1.370099498652	-1.749458177124
-1.369919433676	-1.749425849753
-1.369737291941	-1.748997360284
-1.369538848759	-1.748927019378
-1.36933663043	-1.748892288842
-1.369167914338	-1.748779121756
-1.368962091525	-1.748688659532
-1.368763068143	-1.748572870104
-1.368557659326	-1.748466721794
-1.368368096515	-1.74798559332
-1.368175764814	-1.747971606369
-1.367977058131	-1.74771573961
-1.367786986118	-1.74768690187
-1.367580542402	-1.747616494979
-1.36736852854	-1.747447237192
-1.367209144208	-1.747314861551
-1.367005575188	-1.747053357532
-1.366841013811	-1.746425849454
-1.366665573484	-1.746350979784
-1.366491587533	-1.745590561036
-1.366292363759	-1.745484659888
-1.366055707846	-1.745439967068
-1.36588627225	-1.745385069236
-1.365684777921	-1.745163311358
-1.365488237735	-1.745030275256
-1.365295276286	-1.744682973104

-1.365089084017	-1.744492565072
-1.364862250391	-1.744299185195
-1.364659958307	-1.744288895498
-1.36448186312	-1.74426700859
-1.36427851447	-1.744166664999
-1.364099934856	-1.744045412848
-1.36387448532	-1.74389565995
-1.363666219423	-1.743549736196
-1.363486297337	-1.743503620524
-1.363324130934	-1.743383863394
-1.363137107656	-1.743231802279
-1.362923100019	-1.743075173415
-1.362731888663	-1.742782618479
-1.362552444541	-1.742321901755
-1.362337587949	-1.742181972336
-1.362174116969	-1.742159381548
-1.362006382688	-1.742058146575
-1.361810490715	-1.741638941561
-1.361629524835	-1.741146575997
-1.361477857022	-1.740915720838
-1.361282486395	-1.740075377042
-1.361130606857	-1.740022491351
-1.360933929197	-1.739571399119
-1.360752264743	-1.739571281597
-1.360580408018	-1.73934940825
-1.360355834553	-1.739311161927
-1.360202229996	-1.73928769946
-1.360012706318	-1.738875244035
-1.359794937074	-1.738754432045
-1.359595302635	-1.738399749789
-1.359421871779	-1.738172137979
-1.359249454166	-1.737915037776
-1.359043286212	-1.737607879239
-1.358839429638	-1.737537030575
-1.35862517518	-1.737112338105
-1.358413631735	-1.737082253843
-1.358230805043	-1.736802614269
-1.35804866788	-1.736273940767
-1.357852476976	-1.735956156446
-1.357660684559	-1.735926342514
-1.357448639005	-1.735778661996
-1.357270520589	-1.735575383949
-1.357111146711	-1.735536952543
-1.35697248377	-1.734782052883
-1.356783188111	-1.734765875244
-1.356580662647	-1.734703325012
-1.356427708678	-1.734701112236
-1.356233117548	-1.734246909384
-1.356059478799	-1.733806128415
-1.355861173879	-1.733693309165
-1.355665942311	-1.733577320631
-1.355486021567	-1.733479762403
-1.355305635193	-1.733289829953
-1.355124540256	-1.733245059717

-1.354944112664	-1.732833289962
-1.354770489812	-1.732338080556
-1.354595043424	-1.731146085489
-1.354366097339	-1.731106458246
-1.354192672742	-1.730862331181
-1.353987567151	-1.729985354012
-1.353811034929	-1.729577788884
-1.353617768334	-1.729292184346
-1.353427085075	-1.729288840598
-1.353223712097	-1.728904261834
-1.353054012196	-1.727675870436
-1.352840908361	-1.727076027678
-1.352651000876	-1.726923102569
-1.352485561299	-1.726121170694
-1.352300871877	-1.725939412071
-1.352147989069	-1.725897170686
-1.351965466224	-1.725611403928
-1.351790582185	-1.725549503404
-1.351603281242	-1.724999008934
-1.351439363809	-1.724512538504
-1.351230753667	-1.724446173118
-1.351035268392	-1.724390126443
-1.350837236279	-1.724324980077
-1.350669355136	-1.724266810893
-1.35045773156	-1.724189603394
-1.350280310047	-1.724019540117
-1.350083630302	-1.723845741535
-1.349894693309	-1.723761418416
-1.349682155877	-1.723435163556
-1.34948184674	-1.723334806548
-1.349239119264	-1.72305841383
-1.349080941088	-1.723022628305
-1.348899643434	-1.722726643724
-1.348686299015	-1.722308742289
-1.348480413072	-1.72178622009
-1.348285131976	-1.721516918925
-1.34809658935	-1.720891121092
-1.347895052831	-1.720860888299
-1.347721256803	-1.720750574084
-1.347549999625	-1.72074565529
-1.347379971325	-1.720475371928
-1.347190329471	-1.720096114646
-1.347040078011	-1.719904708961
-1.346874681075	-1.719649299242
-1.346695454645	-1.719604351212
-1.346534691049	-1.719356152477
-1.346375776996	-1.718995789674
-1.346221653638	-1.718898951371
-1.346023493157	-1.718720294864
-1.345820792409	-1.718707802794
-1.345655927819	-1.718305539234
-1.345447469969	-1.71775464992
-1.345264088869	-1.717053487927
-1.345055215856	-1.716474931363

-1.344894940993	-1.716080583016
-1.344744495621	-1.715473847967
-1.344537124219	-1.714998830221
-1.344352719714	-1.714830315966
-1.344173853227	-1.714829148385
-1.343979680715	-1.714745453478
-1.343760226404	-1.714326495363
-1.343519182196	-1.714314598377
-1.343296277246	-1.714299749298
-1.343102019469	-1.714251716065
-1.342918091491	-1.713986052758
-1.342727560754	-1.713740134508
-1.342534850579	-1.713311604683
-1.342360546948	-1.712971663467
-1.342162610724	-1.712132075722
-1.341968785707	-1.712033634662
-1.341827400112	-1.71166555554
-1.341628918676	-1.711598762599
-1.341392289145	-1.711423897164
-1.341200624144	-1.711164425792
-1.341006637705	-1.710971192858
-1.340806522895	-1.710883859829
-1.340580943327	-1.710506518044
-1.34039771407	-1.71049210992
-1.340223350833	-1.709873097271
-1.340052585342	-1.709296220894
-1.339863878099	-1.709279122305
-1.339675385224	-1.709057477829
-1.339504603236	-1.708137587617
-1.3393103998	-1.708132874707
-1.339142257486	-1.707324180381
-1.338971482755	-1.707321389766
-1.338795808781	-1.706834775871
-1.338616084882	-1.706820756008
-1.338468935067	-1.706682495853
-1.338298008073	-1.70660370078
-1.338146932452	-1.706419223017
-1.337949219367	-1.706052099978
-1.337745044354	-1.705898027769
-1.337557803671	-1.705346053355
-1.337405740502	-1.705217752628
-1.337239539029	-1.705018767908
-1.337023186244	-1.704984577909
-1.336802716362	-1.704976554699
-1.336605042893	-1.704491690102
-1.336424139835	-1.704402984965
-1.336245858753	-1.704265109854
-1.336040990197	-1.704152657308
-1.335901110538	-1.704028271342
-1.335688413771	-1.703825464643
-1.335497917813	-1.702828820811
-1.335308488542	-1.702793620406
-1.335155458855	-1.702722344249
-1.334972834835	-1.702257430154

-1.334782791777	-1.702248974074
-1.334594164556	-1.70190012197
-1.334439161307	-1.701878084517
-1.33426583491	-1.701615818372
-1.334055070756	-1.701535921032
-1.333838390365	-1.701433770044
-1.333665640549	-1.70123642376
-1.333498732046	-1.701210018275
-1.333322148904	-1.700988931746
-1.333134825738	-1.700973860807
-1.332903558349	-1.700377888035
-1.332699484904	-1.700271078073
-1.332538980661	-1.699699541606
-1.332361146929	-1.699600132673
-1.332183084607	-1.699538146092
-1.332025533536	-1.699429715508
-1.331883228884	-1.699412616167
-1.331726285386	-1.699082064891
-1.331510932648	-1.698797535251
-1.331335351755	-1.698725688548
-1.331139527706	-1.698554347064
-1.330979750839	-1.698257103449
-1.330815222215	-1.697885608779
-1.330643703264	-1.697885487141
-1.330446626306	-1.697730038867
-1.330269756886	-1.697372329228
-1.330113613571	-1.697186895877
-1.32991642837	-1.697094850112
-1.329755517675	-1.696850177973
-1.329550842772	-1.696778554954
-1.329387528611	-1.696674246378
-1.32923711425	-1.696520704199
-1.329036315017	-1.696307661647
-1.328844005197	-1.696051522138
-1.328659246865	-1.695583151724
-1.328502777574	-1.695464275252
-1.32833179423	-1.695169457024
-1.328112036759	-1.694549923579
-1.32793901439	-1.694435089662
-1.327791987797	-1.694235049057
-1.327596840404	-1.694151876192
-1.32739574932	-1.693495787673
-1.327195705185	-1.692968970856
-1.327014317056	-1.692611036646
-1.326837446372	-1.692604234614
-1.326639532549	-1.692143858747
-1.326450311775	-1.691669255289
-1.326248865912	-1.690697895009
-1.326059669751	-1.690569888675
-1.325868032081	-1.690380999808
-1.325708634463	-1.690326268504
-1.325544443934	-1.690022451376
-1.325358520298	-1.689317725801
-1.325167731947	-1.689031412013

-1.325023029895	-1.689010618103
-1.324859580997	-1.68892980274
-1.324690226651	-1.688089696276
-1.324506024839	-1.687482277072
-1.324327622651	-1.687306604081
-1.324184348254	-1.68676937698
-1.324000500573	-1.68666018948
-1.323840419172	-1.686333660981
-1.323677763878	-1.686034227998
-1.323514167031	-1.68576000005
-1.323325118166	-1.685624192979
-1.323168524865	-1.685546884951
-1.322974364159	-1.685339669401
-1.322788929106	-1.684947983063
-1.322556526007	-1.68462078306
-1.322358124289	-1.68459573899
-1.32214337373	-1.684576032045
-1.321960413453	-1.684147242691
-1.321719349183	-1.684084012241
-1.321560940307	-1.684021897531
-1.321376590776	-1.683918609027
-1.321210989153	-1.683389449346
-1.321017209313	-1.683093746028
-1.320834171812	-1.683071634079
-1.320628207848	-1.682675698516
-1.3204559839	-1.682398987875
-1.320273881961	-1.681854820624
-1.320083074791	-1.681759696416
-1.319866356232	-1.681466528414
-1.319696561756	-1.680671340429
-1.319461457067	-1.680555912007
-1.319266999118	-1.679916527197
-1.319088788531	-1.6793755309
-1.318892978821	-1.67922974409
-1.318728003376	-1.678902278736
-1.318563370488	-1.678633041013
-1.318398190195	-1.678297087539
-1.318200381152	-1.677845207039
-1.318023329431	-1.677830035569
-1.317859605401	-1.677689870584
-1.317702803743	-1.677551629582
-1.317515125146	-1.677407814421
-1.317358240026	-1.674949371022
-1.317178807643	-1.67443145527
-1.317001838616	-1.674390847022
-1.316823589067	-1.674009049349
-1.316684816576	-1.673829097104
-1.316508791824	-1.673773554072
-1.316317777515	-1.673608433062
-1.316164996037	-1.673493076776
-1.31600696471	-1.673321985974
-1.315810679653	-1.672830218592
-1.315633732366	-1.671881777235
-1.315450042719	-1.671871760436

-1.315276762714	-1.67177557726
-1.315096015488	-1.671678749752
-1.314925932789	-1.671603988055
-1.31470704221	-1.671494969041
-1.314522087719	-1.671481008335
-1.31434840065	-1.671342362871
-1.314188913137	-1.671201192216
-1.314028484152	-1.671197980686
-1.313825777228	-1.67041032816
-1.313633424505	-1.670304721305
-1.313468824284	-1.670289256148
-1.313288907068	-1.670146372808
-1.313109636613	-1.66984190201
-1.31295587176	-1.669839928241
-1.312770491305	-1.669752044078
-1.312583560239	-1.669690241151
-1.31238768709	-1.669630193161
-1.312211653165	-1.669461914021
-1.312057943867	-1.66888500803
-1.311873377375	-1.668820123392
-1.311694298911	-1.668172557281
-1.311512047914	-1.667870914007
-1.311319029321	-1.667339586689
-1.311170509542	-1.667321174277
-1.311007005043	-1.667315503585
-1.310800202271	-1.666896781513
-1.310637936589	-1.666714672592
-1.310479243039	-1.666607835658
-1.310333792482	-1.666424534114
-1.310117153708	-1.666103023093
-1.309918672094	-1.666073314766
-1.309725873666	-1.665696943789
-1.309528333253	-1.665587595167
-1.309371618729	-1.665493529815
-1.309196422841	-1.665458700916
-1.308982065257	-1.665385696899
-1.308835898028	-1.665097758234
-1.308647241129	-1.664803599218
-1.30846174577	-1.664738903394
-1.308298979729	-1.663501521826
-1.308110791966	-1.663484258968
-1.307904611945	-1.663229875081
-1.307735801957	-1.663142845914
-1.3075692214	-1.663128728574
-1.307397184599	-1.663118398115
-1.307248040611	-1.662897341438
-1.307073634708	-1.662642917203
-1.306940386162	-1.662626058006
-1.306768033933	-1.661276588629
-1.306619594145	-1.661258524138
-1.306475091018	-1.661246884701
-1.306307991809	-1.660973729743
-1.306141842703	-1.660886961786
-1.305959890726	-1.660669855209

-1.305808811295	-1.660555011373
-1.305637376858	-1.660543761496
-1.305451244082	-1.659893861686
-1.305316085779	-1.658929499302
-1.305150381404	-1.658826109376
-1.304969718253	-1.658783570955
-1.304778829538	-1.658757690504
-1.304625748966	-1.658303455107
-1.304455196736	-1.658286979445
-1.304305037972	-1.658285014678
-1.304108428951	-1.65794602366
-1.30393809515	-1.65786379081
-1.303746349648	-1.657756774309
-1.303596390827	-1.657719038901
-1.303431825762	-1.657249046196
-1.303251532451	-1.657091919887
-1.303052141189	-1.656882138173
-1.302864443138	-1.65635789581
-1.302694247364	-1.655913769614
-1.302497567454	-1.655852111773
-1.302323437627	-1.655850841092
-1.302151975226	-1.655117541039
-1.301961689866	-1.654691219046
-1.301784501683	-1.654359094936
-1.301628315245	-1.654344265914
-1.301425638538	-1.654213034645
-1.301265787544	-1.654205376708
-1.301094064408	-1.654190107344
-1.300924599355	-1.653537028072
-1.300751649037	-1.653506283171
-1.300558052863	-1.653502454044
-1.300387705092	-1.653473488946
-1.300213343651	-1.653150158339
-1.300065045048	-1.652872284819
-1.299906634214	-1.652065398126
-1.299716867565	-1.651990197136
-1.299513758962	-1.651786403642
-1.29936034076	-1.651090126274
-1.299210848149	-1.650757086254
-1.29904791057	-1.650321522151
-1.298875748303	-1.650005091779
-1.2987205585	-1.649796268358
-1.2985697475	-1.649445473969
-1.298370632978	-1.64935476137
-1.298184715989	-1.649258306565
-1.298028457957	-1.647953386205
-1.297850940569	-1.647719416053
-1.297706289393	-1.647631426977
-1.29753718925	-1.647200786306
-1.297349854069	-1.647170701023
-1.297154177444	-1.647056000466
-1.296989991877	-1.647020800866
-1.296815232018	-1.646975234118
-1.296631497101	-1.646665556232

-1.296441958922	-1.646640984245
-1.296237471228	-1.646479572731
-1.296063468291	-1.645452115257
-1.295866652682	-1.645093951876
-1.295677258833	-1.644752624839
-1.29550668125	-1.644713474613
-1.295312949784	-1.644487369401
-1.295139713696	-1.644329571138
-1.294966236329	-1.643799026221
-1.294781345197	-1.643510301292
-1.294595141446	-1.642786757845
-1.294427701933	-1.642464319339
-1.294264964311	-1.642368096593
-1.29413229923	-1.64204611248
-1.293976094945	-1.642026334277
-1.29381615054	-1.641803593245
-1.293661036005	-1.641740604548
-1.293500506834	-1.64172247747
-1.293313940509	-1.641491200788
-1.293137545331	-1.641220337371
-1.292981522795	-1.64101856468
-1.29280150023	-1.64050749197
-1.292623678718	-1.640388874035
-1.292476015146	-1.640152645632
-1.292298451098	-1.639541369361
-1.292115226118	-1.639415416456
-1.291953553326	-1.639378234499
-1.291789329433	-1.639362807827
-1.291638812533	-1.63876017839
-1.291498157112	-1.638670655277
-1.291334553033	-1.638261401555
-1.291171003291	-1.638235137053
-1.290991017335	-1.637915232586
-1.290799956258	-1.637632008213
-1.290636385961	-1.636963127062
-1.290462887514	-1.636440769104
-1.290289266291	-1.636178399968
-1.290113572266	-1.635996879788
-1.2899478251	-1.635984446219
-1.289757212686	-1.635895536712
-1.289586613704	-1.63560135551
-1.289404971849	-1.634412189906
-1.289240049557	-1.634296760673
-1.289083417231	-1.633513392456
-1.288877493769	-1.632885550285
-1.288709871757	-1.63284864778
-1.288520463982	-1.632690760057
-1.288332143349	-1.632690616317
-1.288188366525	-1.632580192135
-1.288003945804	-1.632438614313
-1.28784229852	-1.632080198447
-1.287675333538	-1.632037839688
-1.287504232427	-1.631757413042
-1.287334073728	-1.631752246562

-1.287144341986	-1.631017533846
-1.286996603666	-1.630661057754
-1.286860982498	-1.630622788287
-1.286708556037	-1.630549272423
-1.286532752681	-1.630220785548
-1.286341384937	-1.630218997792
-1.286199741471	-1.630101629906
-1.286052215515	-1.629907996061
-1.285865411257	-1.629750004888
-1.285663354895	-1.629638851509
-1.285488102983	-1.629471165298
-1.285336605448	-1.629261648282
-1.285200315811	-1.62926020044
-1.285020553236	-1.629027962405
-1.284842896624	-1.629025002202
-1.284693947148	-1.62884797639
-1.284509114665	-1.628648962581
-1.284280595634	-1.628241479399
-1.284105239165	-1.627969096238
-1.283958646324	-1.627916401283
-1.283786345679	-1.627912720564
-1.283590514801	-1.62787311579
-1.2833703394	-1.627694308366
-1.283178836536	-1.627647251856
-1.283024416442	-1.6273354158
-1.282858004273	-1.62689915917
-1.282690870185	-1.626590637122
-1.282535279619	-1.626412469998
-1.282360820613	-1.6261133172
-1.282172457677	-1.625803899811
-1.282003470425	-1.625736544996
-1.281833596443	-1.625686399447
-1.281680880982	-1.625274391452
-1.28151249302	-1.624717343708
-1.281372300316	-1.624698727862
-1.281197144979	-1.62464304086
-1.281026255925	-1.624511958732
-1.280851929324	-1.624022987372
-1.280640741211	-1.623877037467
-1.280448738216	-1.623845153944
-1.280305282248	-1.623666921351
-1.280113180662	-1.623565506183
-1.27995407166	-1.62352188991
-1.279783260862	-1.623446337576
-1.279632040725	-1.623413246665
-1.279475173325	-1.623160362464
-1.279271373045	-1.622668578205
-1.279095332062	-1.622634283894
-1.278934972295	-1.622609593094
-1.278787937801	-1.622232104781
-1.278652872131	-1.621854274265
-1.278461938495	-1.621758418858
-1.278298921529	-1.620888603975
-1.278101848	-1.620625577638

-1.277918016001	-1.620392945347
-1.277783105759	-1.619566498115
-1.277609763665	-1.619402187187
-1.277444684006	-1.619323066193
-1.277264952627	-1.619312661122
-1.277093928923	-1.61910025942
-1.276898408346	-1.618861489839
-1.276745574599	-1.618757428877
-1.276542089725	-1.618436238721
-1.276356782538	-1.618386271244
-1.276189799055	-1.618003798232
-1.276050096634	-1.617981032672
-1.275887365983	-1.617103071617
-1.27572308366	-1.616763486043
-1.275545889931	-1.616706170789
-1.275373955671	-1.616254661563
-1.275187187431	-1.616220927749
-1.275029925309	-1.615601081721
-1.27486561673	-1.615472686
-1.274671816304	-1.61539092329
-1.274522165622	-1.614736343513
-1.274361187633	-1.614292621577
-1.274185918015	-1.614215265828
-1.274029485944	-1.614194208606
-1.273854386529	-1.61416161509
-1.273675866157	-1.614090784422
-1.273512931965	-1.613852542454
-1.273345118827	-1.61374516407
-1.273209373512	-1.61317138007
-1.273048305318	-1.612502658619
-1.272860569258	-1.612269501503
-1.272707662697	-1.612223559591
-1.272543534133	-1.612134022368
-1.272393008629	-1.611636908743
-1.272200742922	-1.611500934644
-1.272051641422	-1.610837658308
-1.271863158028	-1.610820158184
-1.271691424388	-1.61049303016
-1.271555619858	-1.610307904476
-1.271370658634	-1.610251088567
-1.271223608734	-1.609849272774
-1.271044718235	-1.609491208662
-1.27090975016	-1.60947541832
-1.270712958023	-1.609394214609
-1.270533668935	-1.609318577652
-1.270334151184	-1.609259749068
-1.270142703071	-1.609149198904
-1.269981421732	-1.608970549399
-1.269813958524	-1.608933865603
-1.269643078345	-1.608726698028
-1.269495000634	-1.608712488075
-1.269319712886	-1.608476627059
-1.269153403663	-1.607978523849
-1.269016305005	-1.607752165021

-1.268876409709	-1.607481641891
-1.268709350488	-1.607253623718
-1.268560042371	-1.60664140405
-1.268405389649	-1.606331130106
-1.268250004974	-1.606309689877
-1.268114009625	-1.606143104405
-1.267938834252	-1.606052796495
-1.267759896393	-1.60550060915
-1.267579995608	-1.605452424946
-1.267421264915	-1.605451406652
-1.26724805809	-1.605126145864
-1.267050999679	-1.60511699475
-1.266911869584	-1.605115682964
-1.266715413954	-1.605064216473
-1.266545545799	-1.604615745754
-1.266388934894	-1.604502904275
-1.266218469703	-1.60362780063
-1.266044373395	-1.603589956066
-1.265875852216	-1.603415702778
-1.265727119922	-1.602721601369
-1.265554865384	-1.602486506319
-1.265400194128	-1.602410627121
-1.265235193233	-1.602357804506
-1.265089097336	-1.602321527638
-1.264912182688	-1.602019133226
-1.264745377773	-1.60194374253
-1.264593460518	-1.601773187491
-1.264456227862	-1.601766777801
-1.264292530828	-1.601614505412
-1.264143944069	-1.601312054901
-1.263961207166	-1.600847512145
-1.263780717945	-1.600765757897
-1.263596270382	-1.6006905573
-1.263416309452	-1.600512808918
-1.263245804292	-1.600353758046
-1.263109407777	-1.599899316552
-1.262969937749	-1.599567352026
-1.262813474538	-1.599363515441
-1.262640612231	-1.599070576482
-1.262497208247	-1.598860246426
-1.262356197899	-1.598833143844
-1.262212065194	-1.598702788663
-1.262011285073	-1.59855510397
-1.261840793442	-1.598540506923
-1.261686586746	-1.598194641567
-1.261505854188	-1.59818620315
-1.261328994238	-1.598099208469
-1.261123881449	-1.597998338131
-1.260982444492	-1.597550325863
-1.260814115227	-1.597065035588
-1.26066201283	-1.596793923082
-1.260475612424	-1.596433099223
-1.26031144522	-1.596191742292
-1.260128869755	-1.596189149082

-1.259971831916	-1.596022959898
-1.259807476147	-1.595857196449
-1.259634899241	-1.595831496838
-1.259523075063	-1.595773705807
-1.259365226904	-1.595628061895
-1.259165014976	-1.595616470109
-1.258990270409	-1.595387828181
-1.258810195934	-1.595276545665
-1.258654600648	-1.595213323172
-1.258502756638	-1.595078405369
-1.258351841829	-1.594727186681
-1.258168292948	-1.594575296025
-1.258007969746	-1.594549794099
-1.257823745089	-1.594137977168
-1.257674605967	-1.594136662858
-1.257497232586	-1.593725348469
-1.257334957246	-1.593579930176
-1.257185539705	-1.593386982454
-1.25701064091	-1.593044547215
-1.256859361884	-1.593037589877
-1.256669794039	-1.592352686741
-1.256502387101	-1.592202833893
-1.256333549445	-1.591959610693
-1.256163815437	-1.59161464062
-1.255980912917	-1.591021555672
-1.255797619692	-1.590981682732
-1.255614014651	-1.590288308124
-1.255430900309	-1.590283863872
-1.255277783463	-1.590153632492
-1.255115487266	-1.590024269065
-1.254954472279	-1.589804616836
-1.254783618928	-1.589547557436
-1.254661268634	-1.5892091122
-1.254510754679	-1.589044519877
-1.2543611665	-1.589016297548
-1.25422811421	-1.588996237832
-1.254063101707	-1.588444605494
-1.253903860735	-1.58843923245
-1.253772301928	-1.588239911772
-1.253594027707	-1.588204061467
-1.253406003676	-1.587914974725
-1.253238802086	-1.587892142874
-1.253063581134	-1.587663741706
-1.252878518023	-1.587412157641
-1.252706628946	-1.587259115149
-1.252524637629	-1.587220698995
-1.252336193158	-1.587109001959
-1.252170382368	-1.586976763911
-1.251985246431	-1.586975780907
-1.251820228663	-1.586726157591
-1.251611766601	-1.586678801364
-1.251447288532	-1.586548436988
-1.251251421904	-1.586390103948
-1.251112569162	-1.585438200419

-1.25095767264	-1.585365778283
-1.250745842037	-1.585306890237
-1.250597225508	-1.58507190098
-1.250424489077	-1.584850906205
-1.250249043043	-1.58441885123
-1.250051658396	-1.584269900186
-1.249900074808	-1.584162559475
-1.249755236308	-1.583851848865
-1.249596761651	-1.583630259559
-1.249440025466	-1.583394135101
-1.249249964149	-1.583064550315
-1.249102401851	-1.581937406578
-1.248936735322	-1.581766120997
-1.248728741899	-1.581526280741
-1.248583659401	-1.581319528778
-1.248414343003	-1.580961491446
-1.248275218505	-1.580937992539
-1.248118467711	-1.580724994828
-1.247943719756	-1.580714474678
-1.247769140674	-1.580616447853
-1.247616054923	-1.580145831799
-1.24741843222	-1.580106953516
-1.24729347315	-1.579905210363
-1.247135345708	-1.578726568693
-1.246977031885	-1.578511806207
-1.24679737734	-1.578274200896
-1.246657813516	-1.578260508718
-1.246472653403	-1.578018316278
-1.246327613969	-1.577772684289
-1.246170426733	-1.577507711476
-1.245960558496	-1.577467366096
-1.245764335117	-1.5773922486
-1.245601700757	-1.577360032451
-1.245442035575	-1.577245857223
-1.245270859268	-1.5771323733
-1.245080455735	-1.576405712039
-1.244903185581	-1.576205936572
-1.244706480216	-1.575948412182
-1.244543741525	-1.575477913153
-1.244391127279	-1.574935997613
-1.24424541672	-1.574824085224
-1.244052189919	-1.574754597136
-1.243914634704	-1.574499030654
-1.24374883091	-1.574086836568
-1.243587053236	-1.573833591557
-1.243373087837	-1.573267414725
-1.243223668207	-1.572864257363
-1.243093584093	-1.572759217162
-1.242948795401	-1.572620779736
-1.242809873477	-1.572557194704
-1.24268090833	-1.572343615249
-1.242526989396	-1.571777430437
-1.242385124719	-1.57151964696
-1.242245232415	-1.571306767977

-1.242103501372	-1.571035799585
-1.241955297154	-1.570932645656
-1.241800295342	-1.570634965914
-1.241640965994	-1.570621366626
-1.241453660897	-1.570493380893
-1.241258232105	-1.5702710058
-1.241072782307	-1.569834074087
-1.240929182263	-1.569757735056
-1.240772256084	-1.569742239134
-1.240576813221	-1.569360774612
-1.240407672571	-1.568883370207
-1.240208324898	-1.568725646081
-1.240035980089	-1.568439215054
-1.239836738097	-1.568436691695
-1.239704738305	-1.568223014632
-1.239550207803	-1.568091478586
-1.239382169529	-1.568018113103
-1.23919615242	-1.567255894801
-1.239022103599	-1.567176279029
-1.238898828613	-1.567135758555
-1.238727564926	-1.567034810172
-1.238553910032	-1.566977505978
-1.238383287349	-1.566879169951
-1.238201632205	-1.566632668667
-1.238056627258	-1.566468198891
-1.237882001867	-1.565860157792
-1.237702176831	-1.565667035777
-1.23752984212	-1.56531983873
-1.237368403941	-1.564375998239
-1.237218741204	-1.564273970114
-1.237061349113	-1.564253082327
-1.236894850981	-1.564061319786
-1.236756950402	-1.563743532745
-1.236610165288	-1.563623967649
-1.236413769932	-1.563564453672
-1.236231674001	-1.563436837343
-1.236064705109	-1.563077513745
-1.235917897328	-1.562637789906
-1.235750853664	-1.562520917734
-1.235612873129	-1.562413851125
-1.235483605771	-1.562322011914
-1.235323515518	-1.562190630183
-1.235142675095	-1.562039728701
-1.234971447565	-1.56202507818
-1.234813604002	-1.561947982293
-1.234691180705	-1.561677286835
-1.234535555441	-1.561441705295
-1.234361872328	-1.561322626442
-1.234176417773	-1.561190409724
-1.234014519793	-1.561033630453
-1.233847281337	-1.560810001209
-1.233711157275	-1.560574823724
-1.233513756169	-1.559689196157
-1.233399339817	-1.559479384336

-1.233235813927	-1.55908255422
-1.233071966836	-1.558979244075
-1.232895144915	-1.558886393673
-1.232735970094	-1.558854937551
-1.232571857455	-1.558727279683
-1.232415589707	-1.558604109059
-1.232279085329	-1.558517368326
-1.232128179659	-1.558162100993
-1.23197863014	-1.558106176216
-1.231820705772	-1.557922591281
-1.231663135994	-1.557786788483
-1.231475001492	-1.55759292264
-1.231311841484	-1.557528900052
-1.231185713831	-1.557246854352
-1.230991672533	-1.55722601415
-1.230801396669	-1.55719597364
-1.230642005687	-1.556981149714
-1.230518402668	-1.556798587889
-1.23034990481	-1.556662353403
-1.230176618816	-1.556556231174
-1.230026159599	-1.556182637764
-1.229865610011	-1.556118089098
-1.229713261421	-1.556056134485
-1.229573832716	-1.55604482862
-1.229424655157	-1.556001742419
-1.229242734231	-1.555942089232
-1.229097544447	-1.555779146358
-1.228939669144	-1.555733897778
-1.228771338094	-1.555659996908
-1.228628948826	-1.555356613011
-1.228484799254	-1.555351333319
-1.228363178128	-1.554956272539
-1.228218470087	-1.554884129934
-1.228054806412	-1.554753132631
-1.227872284671	-1.554669606416
-1.227712504794	-1.554531204526
-1.227578053285	-1.554339970293
-1.227417513682	-1.554210069139
-1.227226540685	-1.554200557761
-1.227065154069	-1.553810451693
-1.226909903198	-1.552880033811
-1.226751451695	-1.55226794105
-1.226615838943	-1.552215848471
-1.226465709881	-1.552140568278
-1.226323425918	-1.552019468493
-1.226172326687	-1.551123300301
-1.226014040986	-1.551120565981
-1.225843166261	-1.550545544212
-1.225678460996	-1.550538684649
-1.225508057872	-1.550475806073
-1.225365557536	-1.549566319796
-1.225191063182	-1.549511184849
-1.225029640856	-1.549296329807
-1.224852542456	-1.549087110016

-1.224688409179	-1.548817093041
-1.224520535725	-1.548736315178
-1.224371291385	-1.548632051959
-1.224230784127	-1.548311802368
-1.224064869587	-1.548245692589
-1.223888786204	-1.547696335751
-1.223727732087	-1.547341903136
-1.223555056527	-1.547304482499
-1.223417379282	-1.546887888444
-1.223269079903	-1.54636589424
-1.223080438223	-1.545964015545
-1.222938496875	-1.545282126202
-1.222771170042	-1.545266661392
-1.222599115124	-1.545144594456
-1.222430556702	-1.545043510192
-1.222261987921	-1.54487382066
-1.222118518482	-1.544333513035
-1.221947288517	-1.543971069433
-1.221789285182	-1.543919602349
-1.221628437288	-1.543727320373
-1.221483191037	-1.543569125065
-1.22131737942	-1.543483231614
-1.221165401235	-1.543422517679
-1.220991471395	-1.543321652288
-1.220842975659	-1.543289361783
-1.220681355716	-1.543118652988
-1.220519908388	-1.542849017728
-1.220352063559	-1.542534787058
-1.220180232383	-1.542509673864
-1.220024760361	-1.542366592573
-1.219880425765	-1.5423185918
-1.219694753109	-1.541585871037
-1.219532756377	-1.541244383466
-1.219357595834	-1.540252570884
-1.219174074843	-1.540153666356
-1.219020846327	-1.539801878311
-1.218868911206	-1.539783693143
-1.218732371536	-1.539673598766
-1.218580936999	-1.53925274628
-1.218430667187	-1.539157536061
-1.218278007613	-1.538926019425
-1.218147864178	-1.538614526464
-1.218012472183	-1.538401630505
-1.217868027581	-1.538288576007
-1.21772484951	-1.53827316934
-1.217578170328	-1.537868240244
-1.217390674671	-1.537692947942
-1.217233059096	-1.537509362443
-1.217068222783	-1.537353363626
-1.216891635627	-1.537311359328
-1.216763902938	-1.537245924524
-1.216621783123	-1.537162398835
-1.216478939647	-1.537097858016
-1.216324388415	-1.537043267494

-1.216181242871	-1.536920405793
-1.216009916335	-1.536757945545
-1.215881364883	-1.536336192469
-1.21568072617	-1.536277578189
-1.215529830129	-1.536250322499
-1.215352308324	-1.536231554329
-1.215175646568	-1.536038706744
-1.21503723723	-1.535629133755
-1.214875084612	-1.535473742821
-1.214736853461	-1.535338063916
-1.214573633378	-1.535057440888
-1.214392152997	-1.534892904507
-1.214239089869	-1.534880099029
-1.214116328053	-1.534736958599
-1.213964212632	-1.534626650793
-1.213830710328	-1.534413985266
-1.213691181883	-1.534366698913
-1.213549815264	-1.533960471811
-1.21338574767	-1.533931086297
-1.21324286786	-1.533924252445
-1.213084945956	-1.533738002081
-1.212935927237	-1.533561075392
-1.212762755411	-1.533350877774
-1.212606012768	-1.533038836337
-1.212455689444	-1.532608264849
-1.212297831361	-1.532580354249
-1.212128428217	-1.531936828257
-1.211963160412	-1.531768938641
-1.211789571582	-1.531684435397
-1.211624661347	-1.531288773336
-1.211460053832	-1.531230912262
-1.211324201289	-1.531178236671
-1.211179417446	-1.531173981528
-1.211018833174	-1.53104736727
-1.210858869299	-1.530757247932
-1.210712252012	-1.530620127739
-1.210587713135	-1.530383050985
-1.210442641215	-1.530306707853
-1.21029065069	-1.530291773132
-1.210140160371	-1.530270767595
-1.209974170086	-1.530161631912
-1.209803237613	-1.529967837412
-1.209675889743	-1.529635155999
-1.209543223406	-1.529586566222
-1.209378413068	-1.529531789272
-1.209230355519	-1.529435853788
-1.209097113669	-1.52934578481
-1.208928744941	-1.529313223168
-1.208774536289	-1.52904641481
-1.208605235558	-1.52837219111
-1.208458232588	-1.528267358817
-1.208339080545	-1.528256823517
-1.208175807073	-1.5281100814
-1.208036282179	-1.527889054868

-1.207886511439	-1.52778866386
-1.207739754666	-1.527734691605
-1.207569710599	-1.527732329246
-1.207398453521	-1.527469598047
-1.207227317136	-1.527022650316
-1.207067156646	-1.526971527014
-1.206931170184	-1.526757984016
-1.206780805152	-1.526755847432
-1.206647625084	-1.526591839467
-1.206422276669	-1.526564821303
-1.206258103131	-1.525983599608
-1.20609540232	-1.525956484794
-1.205920218598	-1.525955490067
-1.205757106515	-1.525651405835
-1.205612529509	-1.525408754582
-1.205450448527	-1.525262810798
-1.205272111969	-1.524957032548
-1.205126116888	-1.524756132446
-1.205016139467	-1.524508194062
-1.204866902372	-1.524079610809
-1.204718175245	-1.523550869443
-1.204590109569	-1.523436980445
-1.204417512144	-1.523359346277
-1.204228908422	-1.523132635636
-1.204053343464	-1.522913687273
-1.203932373581	-1.522705342838
-1.20375312324	-1.522615955404
-1.203633679287	-1.522501149367
-1.203494342759	-1.522497844137
-1.203344831349	-1.52233497685
-1.203152931025	-1.522302374593
-1.203020588689	-1.522251301388
-1.202888522929	-1.521545908263
-1.202749488079	-1.520765531455
-1.202591523637	-1.520067029847
-1.202439378518	-1.519839412912
-1.202288113617	-1.519684402731
-1.202143937025	-1.519456603033
-1.201978523911	-1.519156893672
-1.201832000749	-1.518818522424
-1.201662332959	-1.518808992028
-1.201486714909	-1.518651298118
-1.201310290937	-1.518568088927
-1.201163547407	-1.518359207077
-1.200990158249	-1.517686915846
-1.200852296343	-1.517542388352
-1.20069968849	-1.517535415146
-1.20053136701	-1.517437887928
-1.200367714057	-1.517416496936
-1.200229436722	-1.51725913818
-1.200087300631	-1.516566494504
-1.199935601527	-1.516551347398
-1.199774376287	-1.516045144902
-1.199601687415	-1.515680021746

-1.199462782441	-1.515089851679
-1.199303370714	-1.515036128754
-1.19916973862	-1.514698270388
-1.19899464866	-1.514639849827
-1.198846275087	-1.514549580621
-1.198668308939	-1.513860118961
-1.198526071794	-1.513734424544
-1.198396339929	-1.513437933067
-1.198248198937	-1.513413060128
-1.198087891268	-1.513376149458
-1.197943231326	-1.513213165059
-1.197773174987	-1.512923498724
-1.197622737012	-1.512684675374
-1.197490457829	-1.512593918263
-1.197337052508	-1.512554777333
-1.197194933558	-1.512436160322
-1.197063372302	-1.512312685493
-1.196915048417	-1.512107658822
-1.19674526179	-1.512030307308
-1.196583664193	-1.512027335372
-1.196414425129	-1.511392093523
-1.196271438743	-1.51129715406
-1.196105875364	-1.511282560049
-1.195970420719	-1.510991883237
-1.195802813326	-1.510858094969
-1.195646270474	-1.510531169242
-1.195479317409	-1.510295352743
-1.195342072257	-1.510230037762
-1.195203739673	-1.510039495307
-1.195084361512	-1.510001037606
-1.194919473038	-1.509845757389
-1.194757557726	-1.509703657242
-1.194623515024	-1.509681252483
-1.194473434416	-1.50911359031
-1.194340118945	-1.508677994646
-1.194199541419	-1.508581023942
-1.194040878585	-1.508349624628
-1.193882670241	-1.508325110327
-1.193742909554	-1.508099682116
-1.193597411445	-1.508096851151
-1.193436090625	-1.507847553042
-1.193275258206	-1.507812831306
-1.193087128791	-1.507427784518
-1.192942074818	-1.507396231875
-1.19280560438	-1.50726094208
-1.192672155599	-1.507231562018
-1.192528611153	-1.5070438579
-1.192354811488	-1.506842078662
-1.192229353392	-1.506435596615
-1.192078611297	-1.506235339694
-1.191944523626	-1.506154783529
-1.191804397263	-1.50592368386
-1.191678352993	-1.505832866941
-1.191515362701	-1.505737875876

-1.191393537292	-1.505254855564
-1.191249946982	-1.505167630377
-1.191115956446	-1.505018057396
-1.190946974611	-1.504704231623
-1.190798835549	-1.50445050592
-1.190635024055	-1.504450211554
-1.190488838109	-1.50437389996
-1.19033325839	-1.504214805473
-1.190160943423	-1.503979668215
-1.190029164223	-1.503952827561
-1.189873450124	-1.503923849655
-1.189720142362	-1.503895258215
-1.189559491017	-1.50368663138
-1.189402800848	-1.503551897789
-1.189250564961	-1.503370278457
-1.189101800005	-1.503229397799
-1.188954148199	-1.503209105334
-1.18878498285	-1.503154566775
-1.188648080956	-1.503113276298
-1.188507754665	-1.503027660179
-1.188337236901	-1.502874611245
-1.188190442447	-1.502721367116
-1.188037091739	-1.50222541876
-1.187890195593	-1.502056228963
-1.18769634459	-1.501061937804
-1.187520665716	-1.500750879604
-1.187342567941	-1.500657333286
-1.187197539607	-1.500650535187
-1.187053266418	-1.500147733729
-1.186911154505	-1.500099068198
-1.186772156431	-1.500091287288
-1.186634614212	-1.500086866941
-1.186519991488	-1.500023202607
-1.186380605538	-1.499912522132
-1.186215437254	-1.499830171782
-1.186049554241	-1.499649814067
-1.185909097906	-1.499597451399
-1.18577924722	-1.49907920229
-1.185627726581	-1.498984589183
-1.185488628977	-1.49867172507
-1.18536048347	-1.498652966575
-1.185223488297	-1.49863964542
-1.185063785505	-1.498451415099
-1.184879141423	-1.498439461442
-1.184732599375	-1.498435538138
-1.184569035585	-1.498042881003
-1.184433214303	-1.497917996745
-1.184277179705	-1.497131545714
-1.184125548974	-1.497034422402
-1.183955426569	-1.496970648787
-1.183783093657	-1.496937169862
-1.183616846013	-1.496918701351
-1.183502501335	-1.496622422699
-1.183365764779	-1.49659843955

-1.18324091619	-1.496565814614
-1.183070096474	-1.496441051766
-1.182938910833	-1.496411512016
-1.182830193494	-1.496142733518
-1.182685256321	-1.496138669645
-1.182538363459	-1.495942909319
-1.182405669459	-1.495917984126
-1.182281272834	-1.495916316769
-1.182113607502	-1.495552034768
-1.181976641668	-1.494792800245
-1.181837376299	-1.494597838859
-1.181690918139	-1.494508357656
-1.181531229341	-1.493964465473
-1.181382288722	-1.493951240753
-1.181252470623	-1.49342207247
-1.181064675282	-1.49332252481
-1.180923927257	-1.493132432795
-1.180755901484	-1.493069176523
-1.180602727385	-1.49283197897
-1.180462706011	-1.492687558214
-1.180309881256	-1.492670137691
-1.180148288622	-1.492621241968
-1.179998580706	-1.492615724702
-1.179848167454	-1.492388710571
-1.17968420282	-1.492245609473
-1.179547250483	-1.492166266614
-1.179395927298	-1.492074784051
-1.179255994246	-1.491924192183
-1.179082239127	-1.491631526628
-1.178925494636	-1.491562353823
-1.178781617892	-1.491176713547
-1.178625859587	-1.490818023336
-1.178453683095	-1.490487158995
-1.17829583097	-1.490346210083
-1.17815609228	-1.490242531328
-1.177980330754	-1.489843545873
-1.177843859397	-1.489414095463
-1.1777014139	-1.489206180931
-1.177537825204	-1.489112708524
-1.177402528382	-1.488540674899
-1.1772692407	-1.488456011796
-1.177110152909	-1.488428596746
-1.176944985511	-1.487863213937
-1.176775834378	-1.487710554885
-1.176652694838	-1.487611243606
-1.176541897896	-1.487188372795
-1.17640701241	-1.486488622924
-1.176274896992	-1.486454288348
-1.176134887548	-1.486442537577
-1.175978035549	-1.486389600449
-1.175848236734	-1.486220738617
-1.175690397119	-1.486213317648
-1.175544830827	-1.486117512243
-1.175383827534	-1.485967267552

-1.175243744694	-1.485713282451
-1.17511487152	-1.485275101997
-1.174967619541	-1.48495090613
-1.174797241017	-1.484863142212
-1.174639798261	-1.484240566443
-1.174505526433	-1.484056672443
-1.174369449183	-1.483881102074
-1.174224829363	-1.483543138131
-1.174065273993	-1.483511643795
-1.173915103281	-1.483501643217
-1.173764858948	-1.483306480085
-1.173588374806	-1.483244809959
-1.173433301249	-1.483180965948
-1.173292074874	-1.483015691931
-1.173147584892	-1.482817189346
-1.173046251277	-1.482810121296
-1.172909867601	-1.482740788676
-1.17274547841	-1.482721645756
-1.172606500571	-1.482700966903
-1.172458593994	-1.482364942009
-1.172306138637	-1.482205105235
-1.172170470378	-1.482101920826
-1.172011848207	-1.48200958601
-1.171887892372	-1.48178789952
-1.171754172042	-1.481500370047
-1.171606792459	-1.481418182504
-1.171477218954	-1.481176103128
-1.171311630903	-1.480870283215
-1.171177695699	-1.480748442314
-1.171034422388	-1.480459331339
-1.17087456325	-1.48042490688
-1.170727087471	-1.480307206786
-1.170589872932	-1.480187558458
-1.170446353296	-1.480175869697
-1.170321437012	-1.480142316409
-1.17017291082	-1.479926851882
-1.170044797007	-1.479618799905
-1.169911350448	-1.47949755179
-1.169774898055	-1.479487206062
-1.169605145294	-1.479076677668
-1.16946263269	-1.478913786395
-1.169305411649	-1.478909260368
-1.169157951354	-1.478886989107
-1.169035874828	-1.478728033518
-1.168913802617	-1.478635498138
-1.16879359596	-1.478213247146
-1.168644580662	-1.478177138198
-1.16850343223	-1.477973340265
-1.168373258615	-1.477605407531
-1.168216759742	-1.477510734309
-1.168089701236	-1.477228021983
-1.167921485413	-1.477165941346
-1.16776784107	-1.477093641894
-1.167645862312	-1.477045831387

-1.16751709602	-1.476959261599
-1.167380206579	-1.476815638146
-1.167255207615	-1.47644785996
-1.167114931625	-1.476383720513
-1.166984394882	-1.476331096129
-1.16685410288	-1.476224173153
-1.166711179621	-1.476187303587
-1.16658206628	-1.476095148929
-1.166455241064	-1.475863644232
-1.166304206318	-1.475672122907
-1.166184046785	-1.475613367308
-1.166050202486	-1.475555670863
-1.165910485632	-1.475358734089
-1.165787301519	-1.47497084771
-1.165634893293	-1.474828142297
-1.165502777967	-1.474623938105
-1.165374984012	-1.474595305198
-1.165217083698	-1.474250864947
-1.165070267112	-1.474053126123
-1.164952369015	-1.474016007344
-1.164809917887	-1.473918748458
-1.164691562208	-1.473877189069
-1.164535037822	-1.473512469187
-1.164376315795	-1.473428541693
-1.164234498126	-1.473380074729
-1.164101823211	-1.473365709978
-1.163953544276	-1.473282212257
-1.163791885709	-1.473214946126
-1.163650842769	-1.473058946426
-1.163514592916	-1.472947489386
-1.163379976348	-1.472904377451
-1.163213612756	-1.472870490663
-1.163087297376	-1.472711524213
-1.162950953281	-1.472542035772
-1.162815353458	-1.472344702741
-1.16264919133	-1.472299688605
-1.162484790569	-1.472284168384
-1.162339712359	-1.472057662208
-1.162183439359	-1.471949275015
-1.162069777096	-1.471904461712
-1.161940223138	-1.471880210576
-1.161787702559	-1.47177607697
-1.161644895722	-1.471612012084
-1.161479960497	-1.471063057582
-1.161342887416	-1.470977652129
-1.161201201594	-1.470865289379
-1.161052320526	-1.470734529963
-1.160927898021	-1.470732742328
-1.160764268594	-1.470379069393
-1.160627383738	-1.470203245726
-1.160485412533	-1.46955473286
-1.160365512299	-1.469433863434
-1.160260087186	-1.469326841671
-1.160115362061	-1.468926352694

-1.159963682201	-1.468896518117
-1.159822935675	-1.468791465626
-1.159694365966	-1.468597338862
-1.159545239711	-1.468519892091
-1.159404026502	-1.468444978648
-1.159244728876	-1.46844495979
-1.159131088641	-1.46801210887
-1.158981706922	-1.467910879742
-1.158845378432	-1.467569919806
-1.158707415622	-1.46740812297
-1.158571186458	-1.466972950523
-1.158416685955	-1.466900614712
-1.158289092025	-1.466829642416
-1.158151166661	-1.466705396775
-1.157998756984	-1.466570004089
-1.157889008531	-1.466134950305
-1.157767717172	-1.466091383199
-1.157622878147	-1.465821279613
-1.157493696675	-1.465556467402
-1.157375568142	-1.465293049447
-1.157237861076	-1.465137274702
-1.157092910613	-1.464914495768
-1.156955253656	-1.464437848724
-1.156807069928	-1.464222781535
-1.156677781705	-1.46396579456
-1.156498248467	-1.463778805894
-1.15637165242	-1.463434923468
-1.156231717304	-1.463331206074
-1.15605993222	-1.463224862248
-1.155915019166	-1.462694552659
-1.155743462887	-1.462461737478
-1.155585043902	-1.462441415947
-1.1554378044	-1.4621822063
-1.155299757219	-1.461790257483
-1.155164038685	-1.461552546398
-1.155011470118	-1.461344741972
-1.15488012919	-1.461323734072
-1.15472958775	-1.461240599801
-1.15455254463	-1.460830191372
-1.15439710802	-1.46050557819
-1.154236079008	-1.460339353475
-1.15407248605	-1.460169753579
-1.153940853103	-1.460148676449
-1.153767320434	-1.459938270127
-1.153650687514	-1.459411131614
-1.153516658442	-1.459386283279
-1.153384399437	-1.459222799254
-1.153238809993	-1.459067479458
-1.153103871093	-1.459008617006
-1.15295637932	-1.458348037781
-1.152798888628	-1.458326096194
-1.152670867154	-1.45813360911
-1.152559963648	-1.458024410635
-1.152417660603	-1.458007332823

-1.152281850789	-1.457919225195
-1.152147352176	-1.457837608575
-1.152024012373	-1.457783460372
-1.151859186092	-1.457402720104
-1.151729292672	-1.456890463405
-1.151593180748	-1.456603275998
-1.151433872174	-1.456567288478
-1.151319326539	-1.456536555809
-1.151148003522	-1.456359324149
-1.150988967764	-1.456312247282
-1.150848695817	-1.456266785958
-1.150717669397	-1.456131816316
-1.150570236358	-1.455930861777
-1.150425415886	-1.455912846562
-1.150312505417	-1.455904359251
-1.150198200168	-1.455576001351
-1.15005742549	-1.455527050837
-1.149925875572	-1.455493308984
-1.149783039145	-1.455414462729
-1.149626217886	-1.454951395825
-1.149493284011	-1.454803231098
-1.149330236752	-1.454758590562
-1.149180476715	-1.454663940982
-1.149028132906	-1.454269096086
-1.14886657729	-1.453924042814
-1.148714598778	-1.453639853983
-1.148550429524	-1.453265779155
-1.148348651823	-1.453054185871
-1.148206585235	-1.45294928337
-1.148065509113	-1.452872552811
-1.147934004706	-1.45280073099
-1.147806568979	-1.452602825842
-1.147673500011	-1.452342544984
-1.147519560997	-1.452322096117
-1.147382385604	-1.452205679335
-1.147233056777	-1.452106831947
-1.147080331797	-1.451948984417
-1.146929143785	-1.451913195297
-1.146781126382	-1.451686748002
-1.146651048822	-1.451618927407
-1.14647889528	-1.451465598557
-1.146345574434	-1.451300232285
-1.146180259469	-1.451170991328
-1.146039298744	-1.450860976747
-1.145909196983	-1.45026238801
-1.145774936816	-1.450080214944
-1.145679236597	-1.449558831119
-1.145522105605	-1.449510035102
-1.145379288947	-1.449256617477
-1.145227393002	-1.449227420571
-1.145083874655	-1.449132511032
-1.144946713198	-1.448972581039
-1.144774336072	-1.448681816597
-1.144643956648	-1.44863559782

-1.144522423161	-1.448501904748
-1.14437347253	-1.448406414565
-1.144218336091	-1.448273862598
-1.144081567794	-1.44812332045
-1.143937691052	-1.448104839793
-1.143836856153	-1.448042246266
-1.143689590791	-1.446973236121
-1.143580933754	-1.446696426609
-1.143461623267	-1.445801575319
-1.143331737261	-1.44575406328
-1.143179573479	-1.445639217209
-1.14302678036	-1.445550770267
-1.142898198271	-1.445226374936
-1.142757137091	-1.445170946404
-1.142605825074	-1.444933436942
-1.142446399266	-1.444904514806
-1.142326534616	-1.444879217778
-1.142168702766	-1.444652649671
-1.142025395599	-1.444534471281
-1.141881007111	-1.443710279331
-1.141742295411	-1.443665967932
-1.141605472569	-1.44347425648
-1.141494064162	-1.443324488382
-1.141332391672	-1.443137270645
-1.141195049369	-1.442879814609
-1.141040729339	-1.442726396722
-1.140889468577	-1.442549715126
-1.140760206463	-1.441605803864
-1.14063328663	-1.441524026273
-1.140492536983	-1.441475733237
-1.14032267845	-1.441314013993
-1.140160309771	-1.441274564099
-1.140019817309	-1.440961894306
-1.139881997178	-1.440749674664
-1.139742764267	-1.440473919717
-1.139606142432	-1.439993570634
-1.139474532306	-1.439906293097
-1.139356108861	-1.439742321494
-1.139202522444	-1.439689168588
-1.13906198127	-1.439400878164
-1.138936012294	-1.439192088291
-1.138791847497	-1.438841816762
-1.138654384117	-1.438743792491
-1.138516928553	-1.43872256307
-1.138371125328	-1.438612941908
-1.138238830692	-1.438611273984
-1.138093127125	-1.438508899137
-1.137966070809	-1.438438688494
-1.137842113864	-1.437962505343
-1.137696870047	-1.43788539143
-1.13752029433	-1.437806375517
-1.137364887514	-1.437803114987
-1.137222966912	-1.437747111893
-1.137072187393	-1.437406724112

-1.136933139055	-1.437395057468
-1.136760336033	-1.437128286715
-1.136626189623	-1.436877275472
-1.136505960614	-1.43682313608
-1.136350527108	-1.436783055738
-1.136224964291	-1.436606618772
-1.136083865017	-1.436314318196
-1.135937480121	-1.435931916637
-1.135821580204	-1.435826545351
-1.135655055732	-1.435785364449
-1.135502264964	-1.43538531181
-1.135355491095	-1.435250864727
-1.135223183205	-1.434750715411
-1.135104022608	-1.434505286105
-1.134937916045	-1.434430987882
-1.134819764069	-1.434364832462
-1.13466491159	-1.43417122771
-1.134554578358	-1.434124083868
-1.134415743389	-1.434105933569
-1.13426686715	-1.434075654117
-1.13412914195	-1.433968187971
-1.133962645693	-1.433546446757
-1.133778444185	-1.433461801537
-1.133645178982	-1.433437942958
-1.133525447291	-1.433242558574
-1.133371610291	-1.432897010351
-1.133260859107	-1.43289405026
-1.133125885572	-1.432501821118
-1.132964947184	-1.43242407751
-1.132812903883	-1.432312290871
-1.132668386183	-1.432257219749
-1.132506730729	-1.432244710644
-1.132384003595	-1.431947658858
-1.132250754003	-1.431933944027
-1.13213665257	-1.431763876436
-1.131992013545	-1.431135632286
-1.131805952614	-1.431051455765
-1.131667214381	-1.430903873576
-1.131522661399	-1.430859530445
-1.131380402911	-1.430697312043
-1.131241007811	-1.430435037316
-1.131121478249	-1.430067183412
-1.130974079152	-1.430057661136
-1.130819499958	-1.429544646921
-1.130681584024	-1.429330713619
-1.130542970435	-1.429110441267
-1.130373303048	-1.429108981768
-1.130220951199	-1.429088253282
-1.130076567292	-1.428536828572
-1.129966492105	-1.428376132679
-1.129829554008	-1.428336728925
-1.12966735318	-1.428132157233
-1.129540867304	-1.428076885374
-1.129411055715	-1.428007421082

-1.129280001683	-1.427281221467
-1.129118331369	-1.427004043833
-1.12897315264	-1.426951401745
-1.128830489292	-1.426925993587
-1.128703978825	-1.426917612192
-1.128558904444	-1.426850082482
-1.128420092616	-1.42650381342
-1.128312853738	-1.426482772655
-1.128189771201	-1.426462901897
-1.128058937125	-1.426446204686
-1.127907813067	-1.426253890489
-1.127767464662	-1.425622840655
-1.127639425834	-1.425562160391
-1.127498135856	-1.425490122977
-1.127368224632	-1.425418572212
-1.127226585226	-1.425395708079
-1.127073240334	-1.425324214743
-1.12692316651	-1.425159355728
-1.126774360535	-1.42502189578
-1.126634172656	-1.424966488819
-1.126524592451	-1.424912724335
-1.126375523971	-1.42487844568
-1.126218584522	-1.424173048096
-1.126078514555	-1.42406507443
-1.125932294028	-1.423583128941
-1.125788139292	-1.423526010387
-1.125656338377	-1.423510998367
-1.125515956583	-1.423325283046
-1.125396133408	-1.423169416717
-1.1252581676	-1.423131107816
-1.125115701699	-1.423046405529
-1.124976281715	-1.422907559437
-1.124839527758	-1.422637640708
-1.124707441833	-1.422533557897
-1.12457257826	-1.422350567802
-1.124437309589	-1.422275555385
-1.124310652185	-1.422233702182
-1.124175034335	-1.422093674171
-1.124021015269	-1.421886008691
-1.12390032002	-1.421378599248
-1.123769077393	-1.421307502121
-1.123642036829	-1.421185107165
-1.123514349224	-1.421083483618
-1.123387936493	-1.42086190588
-1.123259713722	-1.420823351359
-1.123094614347	-1.420514136597
-1.122951158438	-1.420307260057
-1.122816682649	-1.419971609779
-1.122654917883	-1.419677219615
-1.12252343752	-1.41936364052
-1.122385081308	-1.419225930372
-1.122236524215	-1.419189070312
-1.122084508064	-1.418745772811
-1.121947794314	-1.418729347844

-1.121819012166	-1.418630079226
-1.121673725132	-1.418628048873
-1.121524050999	-1.418564117262
-1.121381644623	-1.418561681692
-1.121247318446	-1.418465759876
-1.121109008723	-1.417898712606
-1.120975188167	-1.417681137452
-1.120834918282	-1.41742439347
-1.120677914958	-1.417354023348
-1.120542438437	-1.417142934392
-1.120392708918	-1.417083712917
-1.120264667816	-1.417071209888
-1.120128909255	-1.417023278999
-1.119979745722	-1.4166868136
-1.119851183624	-1.416681677274
-1.119702816911	-1.416575169457
-1.119571724288	-1.41650117128
-1.119442775592	-1.416425072342
-1.119325309086	-1.416334749798
-1.119202039991	-1.416331755409
-1.119063598548	-1.416174389282
-1.118918386899	-1.416148308507
-1.11879066558	-1.415848871924
-1.118656461144	-1.415633183928
-1.11852562452	-1.415148467635
-1.118388920466	-1.414995064956
-1.118280181967	-1.414830543605
-1.118178206208	-1.414549643193
-1.118044851891	-1.414540530915
-1.117893557986	-1.414338352277
-1.117750379955	-1.414282585056
-1.117601754705	-1.41422285723
-1.117473735629	-1.414027914373
-1.11733186381	-1.413800743707
-1.11719735486	-1.413752638954
-1.117063107578	-1.413478800864
-1.116930890879	-1.413168745124
-1.116778586726	-1.413110531055
-1.116657981478	-1.412678459828
-1.116504915098	-1.41259572042
-1.116367138906	-1.412244543255
-1.116225878656	-1.412080709253
-1.116083270793	-1.411858572556
-1.115919103681	-1.411833905445
-1.115773739376	-1.411652773678
-1.115647193894	-1.411477840823
-1.115526613736	-1.411186548295
-1.11536719745	-1.410922299669
-1.115213959999	-1.410832004263
-1.115060537002	-1.410708766399
-1.114903635023	-1.410471311965
-1.114760682831	-1.41038295015
-1.114643112199	-1.410164042752
-1.114508714616	-1.409780463015

-1.114345323475	-1.409748506219
-1.114237676124	-1.409678769955
-1.114114617664	-1.409610138578
-1.113988587804	-1.409561851259
-1.113845539176	-1.409432765606
-1.113686004395	-1.409189945421
-1.113543145063	-1.408906844076
-1.113417607289	-1.408821416508
-1.113273139004	-1.408801199168
-1.113172243258	-1.408762391531
-1.113049171799	-1.408725653529
-1.112914754525	-1.408241575146
-1.112785991371	-1.408204335115
-1.112653482195	-1.408083670649
-1.11251023468	-1.408013653015
-1.112383598472	-1.407925199536
-1.112269628004	-1.40781905598
-1.112123304338	-1.407346864904
-1.111996337397	-1.407342229448
-1.11183946115	-1.407336951445
-1.111722429256	-1.407113387175
-1.111572250029	-1.407040569757
-1.111435526386	-1.406669746371
-1.111297704046	-1.406617420778
-1.111164965611	-1.406532612061
-1.111021798714	-1.405893429953
-1.110895315987	-1.405849355906
-1.110760262008	-1.405804815737
-1.110642002551	-1.405553131798
-1.110518361251	-1.404936225696
-1.110343522573	-1.404844838445
-1.110213404389	-1.404764076412
-1.110071881801	-1.404740591172
-1.109935162166	-1.404369624239
-1.109820136827	-1.404312902226
-1.109664932804	-1.404129768232
-1.10953094379	-1.403897033459
-1.109410028985	-1.403705458827
-1.109291917641	-1.403446171848
-1.109180160348	-1.403337260857
-1.109051688704	-1.403034391591
-1.108915591196	-1.402902410879
-1.108771436509	-1.402882516477
-1.108652875552	-1.402831664587
-1.108501540006	-1.402830344937
-1.108355812734	-1.402656068682
-1.108226866405	-1.402464207241
-1.108096410329	-1.402316728267
-1.107960730955	-1.40221158134
-1.107834025517	-1.402167550517
-1.107695429882	-1.402132724972
-1.10756488695	-1.401986979628
-1.107422553298	-1.401624283522
-1.107274907265	-1.401547812014

-1.107126805862	-1.401524137958
-1.10701261922	-1.4013865307
-1.10690390999	-1.40094308585
-1.106781159677	-1.400735204272
-1.106639734052	-1.400634399487
-1.106517388991	-1.400545152851
-1.106372510205	-1.400267145924
-1.10623804628	-1.400227749055
-1.10610350929	-1.399754968296
-1.105961850381	-1.399592200023
-1.105838804803	-1.399005730309
-1.105726914802	-1.398474875392
-1.105597350964	-1.398473813159
-1.105441330604	-1.398413322982
-1.10531282843	-1.398366084087
-1.105173930763	-1.397265353861
-1.105041076989	-1.397061727187
-1.104924144764	-1.396932620577
-1.104786567033	-1.396762731092
-1.104668738565	-1.396753082112
-1.104527255032	-1.39658491362
-1.104406385973	-1.396575549463
-1.104286440406	-1.396571710221
-1.104149882663	-1.396081802362
-1.104016886683	-1.395843652414
-1.103884365443	-1.395842175478
-1.103785671128	-1.395839005097
-1.103657377385	-1.395635304319
-1.103516572812	-1.395443373974
-1.10337689494	-1.39488344583
-1.103234422551	-1.394788874152
-1.103117684845	-1.394754680613
-1.102975389343	-1.393665336961
-1.10283243833	-1.393596604135
-1.102691505115	-1.393391445328
-1.102574186943	-1.393246953258
-1.102443784937	-1.393164994494
-1.102320726696	-1.393060944018
-1.102170658541	-1.392974644248
-1.102047627297	-1.392766690285
-1.10190768709	-1.39271557917
-1.101771628134	-1.392695523722
-1.101626638051	-1.392677340136
-1.101485079869	-1.392562994077
-1.101343722207	-1.392549119417
-1.101238351489	-1.392514636077
-1.101105663577	-1.392201158898
-1.100958382709	-1.391954168623
-1.100829670225	-1.391717941805
-1.100689844472	-1.391552330828
-1.100559226521	-1.391488160754
-1.100400283677	-1.391346784978
-1.10027798841	-1.391196450089
-1.100161231462	-1.391044110942

-1.100027836974	-1.390356616417
-1.099903300674	-1.390191141573
-1.099763333	-1.390126534552
-1.099630006621	-1.390093445358
-1.09949130865	-1.389959803042
-1.099370210552	-1.389868338934
-1.099237808291	-1.389798729717
-1.099091518035	-1.389738421623
-1.098973028168	-1.389645588896
-1.098848619229	-1.38944603668
-1.098702053547	-1.389422674907
-1.098567661858	-1.389251240146
-1.098430500581	-1.388928627139
-1.098304137759	-1.388848251703
-1.098171878559	-1.388676445461
-1.098039763091	-1.388273283644
-1.097903599479	-1.388231615056
-1.09777360316	-1.388174220177
-1.097661095097	-1.387824522155
-1.097517960303	-1.387797944433
-1.097368791035	-1.387247397037
-1.097228549426	-1.387240023134
-1.097094679315	-1.386990949796
-1.096955519984	-1.386862077779
-1.096814060785	-1.386657737378
-1.096721771495	-1.38648143643
-1.096577855114	-1.386438415747
-1.09644216732	-1.386383963418
-1.096307071953	-1.386353007998
-1.096173458001	-1.386180793966
-1.096042162698	-1.38596345762
-1.095888546555	-1.385866460365
-1.095761972578	-1.385619257705
-1.095641765513	-1.38538388378
-1.09553064656	-1.385253005888
-1.095411753462	-1.385198404877
-1.09527273183	-1.384979770639
-1.095127713414	-1.384690037801
-1.094979442822	-1.384570908297
-1.094832460877	-1.38447180948
-1.094686082465	-1.384419158957
-1.094538178801	-1.383812083739
-1.094421674839	-1.383632180497
-1.094252691737	-1.383306079283
-1.094107665916	-1.383292578453
-1.093974737693	-1.383270369757
-1.093837815697	-1.383143542971
-1.093713409374	-1.383041756808
-1.093584891804	-1.382938972748
-1.093452194232	-1.382827123983
-1.093299256017	-1.382588537755
-1.093171374165	-1.382374290849
-1.093035721104	-1.382372526924
-1.092881044142	-1.381709974383

-1.09273492293	-1.381491538141
-1.092596775044	-1.381367366353
-1.092486168921	-1.381225253952
-1.092362797179	-1.381040731693
-1.092238944824	-1.380867387324
-1.092086141092	-1.380744781532
-1.091939392493	-1.38040816086
-1.091814655163	-1.38010430767
-1.091695494541	-1.379547246662
-1.091581897753	-1.379442947275
-1.091453979149	-1.37927117035
-1.091322352187	-1.379150278813
-1.091186542127	-1.378729313323
-1.091045868994	-1.37831118977
-1.090918698912	-1.378206874698
-1.090792661693	-1.377755360058
-1.090672414342	-1.376824272022
-1.090545182838	-1.376796535867
-1.090402568576	-1.376760038234
-1.090274194471	-1.376623681768
-1.090156628761	-1.376438380193
-1.09003877674	-1.375960501366
-1.08990156778	-1.375804156459
-1.089778242252	-1.375663921576
-1.089650747128	-1.37500533392
-1.089521996217	-1.374806796592
-1.08938249647	-1.374673160361
-1.089233735031	-1.374575465906
-1.089083575819	-1.374563167488
-1.088953262177	-1.37440159157
-1.088807588667	-1.374212101029
-1.088666575778	-1.374030343941
-1.088533822537	-1.37400752361
-1.0883936907	-1.373855917297
-1.088243993055	-1.373768574288
-1.088110964372	-1.373688833017
-1.087974059384	-1.372807241722
-1.087851622238	-1.3726649838
-1.087732785208	-1.37265853313
-1.087602751361	-1.37262790781
-1.087453970326	-1.372585533849
-1.087320456422	-1.372581579361
-1.087215587254	-1.372487845613
-1.087098809842	-1.372373984839
-1.086965853995	-1.372244631138
-1.086805956206	-1.372015248232
-1.086665094729	-1.37198692916
-1.086526061734	-1.371901826993
-1.086400999453	-1.371874675011
-1.086243670195	-1.37185803194
-1.086095545208	-1.371475031304
-1.08597549988	-1.371376997282
-1.085853868782	-1.371310622026
-1.085704900371	-1.371302594839

-1.085595094935	-1.371071613094
-1.085459028606	-1.370639095584
-1.085367369477	-1.370478226008
-1.085236939333	-1.370286544052
-1.085104222918	-1.370102368491
-1.084961017846	-1.369847399298
-1.084852706859	-1.36966631279
-1.084746140433	-1.369439733227
-1.084631907405	-1.369177130499
-1.084494665226	-1.369157055628
-1.084375500006	-1.368949207397
-1.084257593377	-1.368873107183
-1.084121331769	-1.368847990371
-1.083999004108	-1.368577907641
-1.083884941021	-1.368534778051
-1.083765704069	-1.368451203261
-1.083636939439	-1.368322081259
-1.083505803531	-1.367698085469
-1.083388488414	-1.367361025676
-1.083231205038	-1.367318912028
-1.083119252758	-1.367304458899
-1.08298670044	-1.36727380419
-1.082866476501	-1.366985478691
-1.082760226682	-1.366952206583
-1.082655920665	-1.366946356092
-1.082517534267	-1.366908880484
-1.082370559265	-1.366884497231
-1.082245595373	-1.366608404824
-1.082105535997	-1.366564065053
-1.081971331889	-1.366435055189
-1.081847991151	-1.366356958479
-1.081693482795	-1.366221368465
-1.081554288213	-1.366211088927
-1.081428761615	-1.366180562131
-1.081302752706	-1.366160974924
-1.081173815323	-1.366075407231
-1.081038670093	-1.365948386867
-1.080919221486	-1.365903645056
-1.080790808543	-1.365894622859
-1.080673681713	-1.365871966665
-1.080549230568	-1.365869621455
-1.080412140533	-1.365827118256
-1.080300052369	-1.365312803522
-1.080140436162	-1.365153000516
-1.079998428178	-1.364991383857
-1.079887360583	-1.364969673761
-1.079760826064	-1.36451452553
-1.079619727568	-1.364509023833
-1.079497603947	-1.364296490371
-1.079365581302	-1.364243788498
-1.079240006429	-1.364029844177
-1.079116587658	-1.36397320551
-1.078977368251	-1.36377840666
-1.078844958229	-1.363616266219

-1.078735108907	-1.363559352509
-1.07861018595	-1.363528938919
-1.078480258382	-1.363482326682
-1.078334018614	-1.363472439551
-1.078192769341	-1.362984337353
-1.078064593447	-1.362741846837
-1.077930128076	-1.362713773447
-1.077797094843	-1.362633407611
-1.077660589714	-1.362574323923
-1.077521845394	-1.362377341783
-1.077374392577	-1.362364316427
-1.077243637838	-1.362353970285
-1.07711185996	-1.362113177741
-1.076971478909	-1.361829579997
-1.076833295268	-1.361787028882
-1.076711080015	-1.361778332972
-1.076603567981	-1.36173217678
-1.076490427419	-1.361706413099
-1.076368945708	-1.361486425844
-1.07623350591	-1.361414154308
-1.076090013405	-1.361387464669
-1.075945111964	-1.36132638216
-1.075823893796	-1.361245674713
-1.075675843286	-1.361144731867
-1.075545860633	-1.360950637518
-1.075415868663	-1.360865935596
-1.075291891027	-1.360851873219
-1.075171789051	-1.360712246097
-1.07504976171	-1.360316141794
-1.074915971464	-1.360265787922
-1.074784330551	-1.360138753273
-1.07465487647	-1.360135108626
-1.074521584667	-1.360055528335
-1.074395113343	-1.359946238234
-1.074274141912	-1.359918350814
-1.074137337776	-1.35924328975
-1.074009622442	-1.359127269896
-1.073854053172	-1.35909977563
-1.073723395143	-1.359094282432
-1.073589395044	-1.358851141472
-1.073436345793	-1.358724202062
-1.073271960388	-1.358204810037
-1.07314585223	-1.358068029701
-1.073010374608	-1.358054019962
-1.072887802631	-1.357954997242
-1.072756408062	-1.357949324157
-1.072649265651	-1.357917293472
-1.072508138524	-1.357866995666
-1.072379221324	-1.357792070317
-1.072232886216	-1.357727164463
-1.072108552784	-1.357684732267
-1.071975729008	-1.357671958027
-1.071837930046	-1.35758675639
-1.07169014376	-1.357347036276

-1.071573680053	-1.356946185901
-1.071441775944	-1.356939617695
-1.071303060374	-1.356820715539
-1.071175153436	-1.356392029465
-1.071042351159	-1.356259369685
-1.070911772588	-1.355735077964
-1.070786738031	-1.35556946482
-1.070656361033	-1.355540577213
-1.070540629453	-1.355268672603
-1.070389667453	-1.355173960708
-1.070262152906	-1.355160738494
-1.070143572564	-1.355000426843
-1.070003801632	-1.35496025216
-1.069878260428	-1.354887591493
-1.069746673788	-1.35485543056
-1.069614315838	-1.354816529317
-1.0694834329	-1.354710537098
-1.069331046194	-1.354681621955
-1.069210726164	-1.35444828565
-1.069072283615	-1.354319537315
-1.068920836099	-1.354264070174
-1.068806025487	-1.35416079213
-1.068674965697	-1.353824588745
-1.068524900122	-1.353722438898
-1.06840017201	-1.353434078567
-1.068264456704	-1.353200423042
-1.068131131995	-1.353095162218
-1.068018046318	-1.352823397889
-1.067893783185	-1.352692637993
-1.067763436483	-1.352533173791
-1.067622179596	-1.352478465974
-1.067506812157	-1.352202233514
-1.067347628814	-1.352129020103
-1.067242885115	-1.351952847533
-1.067111079181	-1.351941080575
-1.066958477066	-1.35170971564
-1.066813756591	-1.351655735534
-1.066674374083	-1.351561184306
-1.066522770915	-1.351425520271
-1.066357989394	-1.351390609511
-1.066228451097	-1.351343494006
-1.066095706262	-1.351317955915
-1.065964531442	-1.350955239852
-1.065843486712	-1.350832916337
-1.065719936191	-1.350307347035
-1.065591664749	-1.350218927976
-1.065463762068	-1.350172373271
-1.065350979921	-1.350136548355
-1.065237275239	-1.350027579916
-1.065107584769	-1.349965322402
-1.064986004715	-1.349931190903
-1.064858438516	-1.349739163157
-1.064727922791	-1.349601483171
-1.064589978057	-1.349513664467

-1.06447186407	-1.34945994831
-1.064320322492	-1.349436288639
-1.064181079642	-1.349257768874
-1.064039856723	-1.349059560965
-1.063908659637	-1.348913754378
-1.06379040772	-1.348769470741
-1.063652078778	-1.348673163382
-1.063503755613	-1.34853309752
-1.063367337553	-1.348490520594
-1.063248453018	-1.348317423425
-1.06314071204	-1.348115330514
-1.063024020446	-1.348103196682
-1.062895765416	-1.347874012557
-1.062793215284	-1.347801615926
-1.062666340204	-1.347772862107
-1.062545498499	-1.347762221255
-1.062415246433	-1.347731255399
-1.06225586678	-1.347386223676
-1.062114636219	-1.347246314926
-1.061981652508	-1.347121635896
-1.061854799341	-1.347113577093
-1.061722854102	-1.34698883829
-1.061611022921	-1.346828119484
-1.061502058012	-1.346733706863
-1.061379588897	-1.34671654956
-1.061243631967	-1.346535647933
-1.061124386242	-1.346410997996
-1.061004379358	-1.346375798051
-1.060871667136	-1.346281021686
-1.060718353971	-1.346252467438
-1.060579975373	-1.346177613033
-1.060458430976	-1.345887876746
-1.060342553444	-1.345744148952
-1.060219526117	-1.34554452273
-1.060105398072	-1.34551396814
-1.059982420722	-1.345469166372
-1.059834337358	-1.345354678984
-1.059719372445	-1.345086361587
-1.059605194285	-1.344908150511
-1.05947133725	-1.344881500917
-1.05933130342	-1.344763122392
-1.059206529414	-1.344592067477
-1.059068466725	-1.344572044638
-1.058933783664	-1.344571311447
-1.0587948282	-1.344529829404
-1.058674343926	-1.344529512724
-1.058553569063	-1.344101535526
-1.058411628849	-1.344035455087
-1.058306791879	-1.343684495399
-1.05820070314	-1.343667748155
-1.058071284144	-1.343265309296
-1.057921268543	-1.343201431037
-1.057780634656	-1.343130941955
-1.05764098131	-1.342702529109

-1.057495590959	-1.342695994166
-1.057371523238	-1.342343179224
-1.057233219999	-1.34204392559
-1.057116526334	-1.341807642418
-1.0569981976	-1.341732462203
-1.056881107585	-1.341502376584
-1.056748311029	-1.341389738097
-1.05662480112	-1.341099197349
-1.056485883964	-1.341087978251
-1.056367043242	-1.340924102555
-1.056240402901	-1.340845350398
-1.056097644636	-1.340634028934
-1.055972502	-1.340538304231
-1.055847997503	-1.340347601753
-1.05574339191	-1.340261686607
-1.055584052839	-1.340225868539
-1.055453212061	-1.340212376082
-1.055325375551	-1.340085271052
-1.055216150077	-1.340021188293
-1.055099754899	-1.339988828345
-1.054988436052	-1.339985325262
-1.054855341913	-1.339801647664
-1.054717939081	-1.339559439345
-1.054593645331	-1.339508761352
-1.054464901837	-1.339313903902
-1.054317026912	-1.339216516984
-1.054190237907	-1.339159789357
-1.054079007715	-1.339050352144
-1.053933868373	-1.338947944074
-1.053765654629	-1.338688017129
-1.053661853897	-1.338659595275
-1.053520068914	-1.338643254413
-1.053383787595	-1.338625241645
-1.05324421074	-1.338421294426
-1.053127022689	-1.338337707324
-1.052987469298	-1.338100743586
-1.05284091164	-1.337834096947
-1.052706586369	-1.337574932023
-1.05258544404	-1.33738768477
-1.052463949937	-1.33737674238
-1.052363589125	-1.337370911761
-1.052234781706	-1.337045975232
-1.052109303527	-1.336978787693
-1.051965809884	-1.33688526479
-1.051829922541	-1.336724287685
-1.051693411406	-1.33664203453
-1.051594327051	-1.336526698555
-1.051471156931	-1.336493646571
-1.051333326912	-1.336242705815
-1.0511898287	-1.336001734509
-1.051074569106	-1.335922577867
-1.05093253972	-1.335804006769
-1.050795830165	-1.335737543211
-1.050645418692	-1.33563598803

-1.050523753405	-1.335324109117
-1.050401920448	-1.335145539207
-1.050285894042	-1.335118320355
-1.050163141355	-1.334869874571
-1.050051412265	-1.334794684285
-1.049916910161	-1.334748954757
-1.049789209464	-1.334740169559
-1.049650433619	-1.334557586794
-1.049515450594	-1.334207456042
-1.049387707789	-1.334062470465
-1.049244432731	-1.333698202374
-1.049129147645	-1.333644469004
-1.049007337592	-1.33363266517
-1.048886470796	-1.333410879187
-1.048744113367	-1.333233778841
-1.04861426609	-1.333233432435
-1.048497661622	-1.332911348927
-1.048379781832	-1.332826127328
-1.048260269545	-1.332782435373
-1.048117771225	-1.332680801253
-1.047995657392	-1.332363058548
-1.047835376962	-1.332311583544
-1.047720383933	-1.33210401595
-1.04759955182	-1.331964316594
-1.047486812007	-1.331927022613
-1.047381571179	-1.33166813584
-1.047239063661	-1.331601148482
-1.047124427459	-1.331593750844
-1.047002635475	-1.331252585821
-1.046879454705	-1.331241935584
-1.046756334057	-1.33109449975
-1.046628793563	-1.331049101303
-1.046528435886	-1.330934432917
-1.046406769509	-1.330763705311
-1.046282631324	-1.33061141035
-1.046158292304	-1.330570219585
-1.046039127496	-1.330553556893
-1.045910950545	-1.330530973498
-1.04579215615	-1.330527867203
-1.045656772661	-1.330451906095
-1.045542633902	-1.329787459356
-1.045435741055	-1.329706138989
-1.045321647605	-1.329668310693
-1.045186174765	-1.3295166546
-1.045031088632	-1.329240885461
-1.044911495365	-1.329227544014
-1.04479419332	-1.32883117693
-1.0446689233	-1.328615295278
-1.044545575582	-1.328451486951
-1.044421155731	-1.328360837532
-1.044305638707	-1.328283883289
-1.044168030175	-1.328198978111
-1.044046956581	-1.3281583886
-1.043941793497	-1.328123529953

-1.043839398312	-1.328100553258
-1.043690052339	-1.327872242594
-1.043554420552	-1.327797315944
-1.043432560169	-1.327765857214
-1.043299375386	-1.327713393287
-1.043192326484	-1.327468626314
-1.043048561859	-1.327386934276
-1.042902521704	-1.327282342751
-1.042767549025	-1.327261656825
-1.042631215626	-1.327009698628
-1.042497355815	-1.326759992154
-1.042366080968	-1.326290073487
-1.042238599802	-1.326247988506
-1.042121590769	-1.326122460201
-1.041981902763	-1.325830277278
-1.041870896187	-1.325798745953
-1.041741930368	-1.325670100927
-1.041621171686	-1.325312284323
-1.041504380507	-1.325215627562
-1.041373618657	-1.325141018924
-1.041239332577	-1.324973221713
-1.041114504076	-1.32480552362
-1.040985145337	-1.324736649001
-1.040861411912	-1.324616157662
-1.040742173126	-1.32454418128
-1.040623206984	-1.324368175019
-1.040503398104	-1.323911728083
-1.040387895306	-1.323755606412
-1.040256693705	-1.323683869803
-1.040112483707	-1.323612495116
-1.03998165563	-1.323601480272
-1.039873012074	-1.323438293504
-1.039730464853	-1.323229358897
-1.039589925992	-1.323217860959
-1.039457838647	-1.323201201597
-1.039331581919	-1.323017089522
-1.039209536425	-1.32281143405
-1.039103224852	-1.322724010544
-1.038973435426	-1.322478098576
-1.038851445202	-1.322465484065
-1.038732346649	-1.32246171834
-1.038623631318	-1.322418189025
-1.038470703922	-1.322382030288
-1.038359876561	-1.322368835497
-1.038240332756	-1.322317357618
-1.038087547482	-1.322055734864
-1.037956390318	-1.321886192686
-1.037821638222	-1.321785247603
-1.037711507536	-1.321721544276
-1.037582754191	-1.321393989522
-1.037476468895	-1.32124785726
-1.037364904778	-1.32111954852
-1.037238328366	-1.321037925528
-1.037130768202	-1.320767229549

-1.037005219421	-1.320754870979
-1.036860518474	-1.320550046378
-1.036727205699	-1.32038874502
-1.0366056195	-1.32032834033
-1.036446204066	-1.320326850982
-1.036331846889	-1.320277573357
-1.036210102411	-1.320204904416
-1.036094273791	-1.320197189867
-1.035997635618	-1.320140587264
-1.035888150501	-1.320128355209
-1.035753879807	-1.319952158181
-1.035634452975	-1.319772747889
-1.035516667263	-1.319478013271
-1.035395793668	-1.319438620182
-1.035277418142	-1.318914714531
-1.035134431976	-1.318785720691
-1.035002810919	-1.318418906552
-1.034885555605	-1.318410143345
-1.034753903985	-1.318207283055
-1.034630544953	-1.318056278443
-1.034503507455	-1.318039258869
-1.034381756067	-1.31797265115
-1.034258767945	-1.317767587355
-1.034140836683	-1.317746587997
-1.033992179386	-1.317742982298
-1.033891506422	-1.316925629985
-1.033764925091	-1.31682512179
-1.033634326081	-1.316788240591
-1.033504147163	-1.316553701106
-1.033376724369	-1.316238418848
-1.033248404232	-1.316077411795
-1.033122948613	-1.316072970203
-1.032984875979	-1.315822772969
-1.03281903927	-1.314587898188
-1.032709996296	-1.314526724392
-1.0325774302	-1.31448869584
-1.032455524015	-1.314344715684
-1.032321735785	-1.314227358963
-1.03217996834	-1.314225753319
-1.032066324802	-1.314177675745
-1.031955402052	-1.314174114407
-1.031832948635	-1.314164636065
-1.031693469321	-1.314056466967
-1.031567503597	-1.313862925339
-1.031456065519	-1.31374735474
-1.031348902044	-1.313730688007
-1.031209145106	-1.313641400378
-1.031101870837	-1.313192884355
-1.030958848456	-1.313092670859
-1.030840089008	-1.313004590524
-1.030740801548	-1.312889092215
-1.030620154309	-1.312306556899
-1.030501282519	-1.312274272855
-1.030368332537	-1.312060700613

-1.030248837678	-1.311954160871
-1.030127014031	-1.311842546185
-1.03000423904	-1.311680379125
-1.029877262527	-1.311598094427
-1.029770129442	-1.311389415143
-1.029667856587	-1.311303772074
-1.029536501227	-1.31115004251
-1.029433590627	-1.310545584837
-1.029322927188	-1.310383254228
-1.029179433913	-1.310180235615
-1.029063859186	-1.310062642305
-1.028943377032	-1.310038025169
-1.0288347095	-1.309878176983
-1.028709406366	-1.309809828821
-1.028601039925	-1.309793573051
-1.028481528314	-1.309428377028
-1.028378244124	-1.309287575025
-1.028248860235	-1.309270456178
-1.028134863612	-1.308071219863
-1.028011866695	-1.307598450687
-1.027881231319	-1.307191297575
-1.027783456703	-1.307172635718
-1.027641677414	-1.306911847939
-1.027537107248	-1.30680275462
-1.027426434254	-1.306786180805
-1.027307982426	-1.306693541871
-1.027193680351	-1.306540281215
-1.027060478747	-1.30646200596
-1.026938570187	-1.30638938386
-1.026814169927	-1.306130896204
-1.026684618214	-1.305984069667
-1.026563339968	-1.30593104598
-1.026435418742	-1.305895562704
-1.026331034941	-1.305381050272
-1.026213689047	-1.305288009976
-1.026065918789	-1.305242746236
-1.025923377657	-1.30473873693
-1.025810563101	-1.304589607555
-1.025696472995	-1.304381208128
-1.025506780708	-1.30431707296
-1.025383964577	-1.304190164747
-1.025246987458	-1.303815891667
-1.025133950696	-1.303763092818
-1.024995710414	-1.303590124527
-1.024843502752	-1.303416564505
-1.024717315042	-1.303258335214
-1.024599254956	-1.303042911458
-1.024473594476	-1.302988261245
-1.024351183281	-1.302987515352
-1.024207882408	-1.302518041749
-1.024085278774	-1.302478121489
-1.023977239387	-1.302453007999
-1.023863456811	-1.302156528139
-1.023739708198	-1.301938568065

-1.02363473647	-1.301735931562
-1.023501258576	-1.301529452898
-1.023381847707	-1.301485205609
-1.023271490469	-1.301370927035
-1.023153625083	-1.301295171695
-1.023034106383	-1.301228173628
-1.022915126082	-1.301226131864
-1.022792428231	-1.301016953662
-1.02268808242	-1.300064017548
-1.022564326894	-1.299560676834
-1.022441818725	-1.299534906812
-1.022292966434	-1.299458602658
-1.022158420348	-1.299036117921
-1.022050437418	-1.298891843799
-1.021922422979	-1.298750760109
-1.021773782388	-1.298716758888
-1.021646845535	-1.298696871009
-1.021519079067	-1.298254426931
-1.021418866003	-1.298042237756
-1.021320018484	-1.297911428104
-1.021207696836	-1.297835502685
-1.021091106239	-1.297793994633
-1.020988980805	-1.297631384729
-1.020869187211	-1.297294871719
-1.020739671769	-1.297267961928
-1.020619429458	-1.297245946753
-1.020505733442	-1.297223416857
-1.020408324369	-1.297064803457
-1.02027742341	-1.296924884465
-1.020163506464	-1.296835783465
-1.020030230674	-1.296747046808
-1.019899505367	-1.296564076536
-1.019773437448	-1.296286764957
-1.019645843493	-1.296180025828
-1.019510831606	-1.296036828462
-1.01939781027	-1.295965240579
-1.019261724896	-1.29575292738
-1.019146284238	-1.295179017781
-1.019019443017	-1.295031615024
-1.01889540551	-1.294939363792
-1.018779828319	-1.294570583237
-1.018663558976	-1.294127177664
-1.018534626855	-1.294040708977
-1.018403767332	-1.294031892636
-1.018272838965	-1.293868571817
-1.018149425712	-1.293416288569
-1.018023109736	-1.293065127809
-1.01791082808	-1.293022392687
-1.017804423915	-1.29289104228
-1.017663737712	-1.292823995802
-1.017560638793	-1.292760622288
-1.017436959751	-1.292571619234
-1.017314426942	-1.292529032037
-1.017180530858	-1.29250016778

-1.0170796128	-1.292276590493
-1.016967536001	-1.292047276573
-1.016861349811	-1.292017238329
-1.016736386333	-1.291969308636
-1.016604892779	-1.291798466697
-1.016481687642	-1.291425647401
-1.0163568899	-1.290968129592
-1.016252623534	-1.29078614051
-1.016124586469	-1.290486997255
-1.016019178514	-1.290096075593
-1.015916338158	-1.289801478471
-1.015805870951	-1.289345302389
-1.015697022081	-1.289333287167
-1.015560694498	-1.289310202261
-1.015452303137	-1.289249831133
-1.01533885336	-1.289063849598
-1.015222038978	-1.288951404468
-1.015103957831	-1.288695207067
-1.014980350429	-1.288681955929
-1.014854413556	-1.288664432165
-1.014738760927	-1.288641274836
-1.014639098677	-1.288316040735
-1.014519011358	-1.287992391882
-1.014376922962	-1.287827015166
-1.014224683685	-1.287757292842
-1.014112149597	-1.287552027406
-1.013994912568	-1.287537740717
-1.013853477069	-1.287521434251
-1.013709976904	-1.287478905984
-1.013609352085	-1.287215753873
-1.013479123203	-1.287092790715
-1.013361055275	-1.287089415959
-1.013241605143	-1.28700064489
-1.013126885126	-1.2868805464
-1.012970218022	-1.286619044673
-1.012845989906	-1.286582236782
-1.012698464537	-1.286526806675
-1.012587796581	-1.28648384087
-1.012438657218	-1.286353655629
-1.012326575037	-1.286350324452
-1.012190976221	-1.286090951152
-1.012064010999	-1.285947122753
-1.011948183576	-1.285862047992
-1.01181642492	-1.285725398816
-1.011698121563	-1.285715038488
-1.011579990186	-1.28558817953
-1.011449882669	-1.285501211164
-1.011334239123	-1.285453470442
-1.011214517868	-1.285452969918
-1.011103402724	-1.285210374529
-1.010985952667	-1.285106769245
-1.010870449791	-1.285103617551
-1.010758406828	-1.28496182118
-1.01063980118	-1.284733470232

-1.010514795311	-1.284489102616
-1.010387443758	-1.28437436443
-1.010263615584	-1.284265510359
-1.01013239216	-1.283393061819
-1.009994856062	-1.283142792012
-1.009867312056	-1.283030027421
-1.009748385459	-1.282987176359
-1.009621258746	-1.282934200312
-1.009476218374	-1.282852260924
-1.009339310654	-1.282760629108
-1.009175658911	-1.282673536356
-1.009063942617	-1.282533783432
-1.008959978428	-1.282226877613
-1.008831716387	-1.282089691859
-1.008703928488	-1.281819091927
-1.008598945284	-1.28166816777
-1.008461315097	-1.281452598203
-1.008322253008	-1.281410454743
-1.008217063558	-1.281326493304
-1.00808113127	-1.281273282263
-1.007978662062	-1.28121560033
-1.007844145175	-1.280914043304
-1.007702989876	-1.280468055242
-1.007594765359	-1.280456820964
-1.007490634481	-1.280365924836
-1.007379482153	-1.280252367017
-1.007271162361	-1.280102936403
-1.007167265273	-1.279814327649
-1.00704423431	-1.279669205624
-1.006919106001	-1.279510145534
-1.006806422153	-1.279462276086
-1.006664814794	-1.279168671422
-1.006545932896	-1.279033113594
-1.006430797131	-1.27902710008
-1.00629755198	-1.279004873041
-1.006177884394	-1.278579574559
-1.006054925036	-1.277866327218
-1.005926111347	-1.277634840012
-1.005801054978	-1.277527049369
-1.005666356002	-1.277167286349
-1.0055458605	-1.277109048286
-1.005426034924	-1.277090693273
-1.005300715117	-1.277065914707
-1.005167816339	-1.276451933432
-1.005024620929	-1.276379531949
-1.004895251205	-1.27619426179
-1.004753942465	-1.27608695944
-1.004638606851	-1.275846568847
-1.004521714732	-1.275827208019
-1.004404746995	-1.275575981249
-1.00428731719	-1.2755742131
-1.004167283738	-1.275557903553
-1.004047972002	-1.275310828682
-1.003905510137	-1.275041004802

-1.003803727096	-1.274743635719
-1.003703192694	-1.274649952864
-1.003605171653	-1.274601112579
-1.003496699001	-1.27459639694
-1.003346720806	-1.274589063187
-1.003203571622	-1.274582807493
-1.003081854187	-1.274580526139
-1.002956594991	-1.274368530294
-1.002837724281	-1.274322466821
-1.002723077503	-1.273781164018
-1.002597089428	-1.273722322283
-1.002488698859	-1.273492654044
-1.002378854031	-1.273206390473
-1.00224897601	-1.273161431018
-1.00210671054	-1.27305848457
-1.001989548556	-1.273039280156
-1.001880229106	-1.272894505625
-1.001755783485	-1.272669476103
-1.001621856412	-1.272587488955
-1.001530176694	-1.272570248723
-1.001426242198	-1.272418886537
-1.001293143874	-1.272318366375
-1.001169120101	-1.272205671284
-1.00104055888	-1.272183211852
-1.00092504104	-1.271827563258
-1.000794359395	-1.271799585043
-1.000666224717	-1.271724135748
-1.000555324796	-1.271555610985
-1.000414118027	-1.27099597278
-1.000295979148	-1.270867651318
-1.000171039295	-1.270586456193
-1.00007095204	-1.270420875822
-0.999948510641	-1.270323289854
-0.999831090845	-1.270257699423
-0.999695159473	-1.270229595352
-0.999586406687	-1.270005992747
-0.999454494734	-1.269855139751
-0.999330556996	-1.269714464699
-0.999207678608	-1.269661323242
-0.999087305957	-1.269375769353
-0.998959684178	-1.269272435713
-0.998854785777	-1.269152189747
-0.998748304625	-1.269143580342
-0.998631718577	-1.26877955654
-0.998511053333	-1.268474276395
-0.998372743912	-1.268445903905
-0.998264244705	-1.26843147386
-0.998122176141	-1.268388530075
-0.997995763601	-1.268228216705
-0.997867893471	-1.268065463833
-0.997748687628	-1.268029582628
-0.997647703878	-1.267960068724
-0.997518239291	-1.267782294299
-0.997383698617	-1.267685647216

-0.997258866945	-1.267513069891
-0.997141121621	-1.267120061963
-0.997012773907	-1.267016836776
-0.996905400888	-1.266883499781
-0.996798687545	-1.266762871315
-0.996663421054	-1.266588401559
-0.996552902688	-1.266511946827
-0.996441329937	-1.2661409778
-0.996324162341	-1.265989849924
-0.99620798255	-1.265789708901
-0.996070357254	-1.265572387213
-0.995944558719	-1.265538268028
-0.995836445962	-1.265509834368
-0.99571635813	-1.26485869431
-0.995591161243	-1.264747028848
-0.995457385794	-1.264697096005
-0.99532951194	-1.264570830974
-0.995221868225	-1.264394637848
-0.995105715454	-1.264376781355
-0.994984979336	-1.264119753879
-0.994846473074	-1.263607823395
-0.994727889354	-1.263060536788
-0.994612685388	-1.262820101055
-0.994499369768	-1.262290709817
-0.99439051321	-1.262031382706
-0.994254028037	-1.261947298158
-0.994132673179	-1.261491488075
-0.993985239332	-1.261196523699
-0.99386312536	-1.261153845806
-0.9937491804	-1.260831086805
-0.993630779179	-1.260479271809
-0.993491725698	-1.260445632964
-0.993363376093	-1.260388039372
-0.993233710176	-1.260027460141
-0.993130368777	-1.260014536986
-0.993009097629	-1.259874620053
-0.992907001491	-1.259870861264
-0.992778106431	-1.259753179991
-0.992662015237	-1.259607947455
-0.992531490583	-1.259541380888
-0.992408685729	-1.259420687121
-0.992275193462	-1.259372024298
-0.992155811242	-1.259368210323
-0.992067247457	-1.259277121811
-0.991914703518	-1.259276845504
-0.991809660428	-1.259274510561
-0.991684494718	-1.259270333087
-0.991583644334	-1.259268277479
-0.991437556768	-1.259030534101
-0.991343256837	-1.258829318348
-0.991231571444	-1.258823873486
-0.991087229217	-1.258787278641
-0.990971492858	-1.258737986864
-0.990825318955	-1.258672168275

-0.990723975406	-1.25854816465
-0.990596351613	-1.258316397233
-0.990477699753	-1.258090176595
-0.990356119075	-1.257772745552
-0.990198977792	-1.257722865057
-0.990060752518	-1.257671568995
-0.989946648527	-1.257667487036
-0.989823322527	-1.257542177523
-0.989694107935	-1.257468066745
-0.98958938253	-1.257455769089
-0.989470935097	-1.257357826981
-0.989348337248	-1.257337501418
-0.989239342653	-1.257304796269
-0.989133769306	-1.257223986214
-0.989009328594	-1.257182700799
-0.988894689945	-1.257070828108
-0.988788290183	-1.257015970452
-0.988648988141	-1.256890054392
-0.988530296296	-1.256823868557
-0.988410453572	-1.256696158971
-0.988277104211	-1.256679233293
-0.988179009304	-1.256347054809
-0.988063701738	-1.256013428367
-0.987944497771	-1.255674957023
-0.987815129882	-1.255269447037
-0.987709134035	-1.25489014453
-0.987595765498	-1.254880754823
-0.987481194679	-1.254577635376
-0.987328757529	-1.254335183169
-0.987207898186	-1.254275395479
-0.987083481841	-1.254179805515
-0.986955743919	-1.254080454523
-0.986796114285	-1.253625523691
-0.986698177562	-1.253561752247
-0.986578625329	-1.253497955643
-0.986461008834	-1.253384470424
-0.986328591764	-1.253268886087
-0.986235415642	-1.253264775216
-0.986128434636	-1.253230441669
-0.986035023832	-1.253184509874
-0.985928035009	-1.253139884497
-0.985827022025	-1.252793047784
-0.985721271975	-1.252789150583
-0.985620234491	-1.252575249574
-0.985488456188	-1.252487505528
-0.985370510593	-1.252184167013
-0.985252937241	-1.251968008016
-0.985141874217	-1.251954865214
-0.985018948222	-1.2519287428
-0.984883707672	-1.251765362432
-0.984753416177	-1.251645401757
-0.984643513792	-1.251563068296
-0.984518790873	-1.251349411781
-0.984403950717	-1.251198737279

-0.984290090977	-1.251014294799
-0.984170185327	-1.250880315882
-0.984063780298	-1.250845078482
-0.983945692832	-1.25073228782
-0.983818657196	-1.250529008017
-0.983724050993	-1.250516123912
-0.98362479623	-1.250415712891
-0.983501120919	-1.250215907652
-0.983360616878	-1.250104833836
-0.983252558375	-1.250030894659
-0.98313952731	-1.249996442658
-0.98302753775	-1.249924968022
-0.982922710846	-1.249757486808
-0.982795133099	-1.249727651472
-0.982667274737	-1.249441591988
-0.982559101464	-1.249439260281
-0.982412859994	-1.249098156261
-0.982275129622	-1.249076354427
-0.982147492026	-1.249057392207
-0.982014241282	-1.248956252266
-0.981910872272	-1.248926130136
-0.981767740488	-1.248901411158
-0.981666246167	-1.248786043746
-0.981550890284	-1.248490059949
-0.981400097619	-1.248348505876
-0.98125062208	-1.248109351727
-0.981132289261	-1.248088966425
-0.981025638748	-1.247683885692
-0.980931204376	-1.247657113362
-0.980795845804	-1.247627752132
-0.980682761809	-1.247554931273
-0.980561681907	-1.247494515915
-0.98041556045	-1.247440853959
-0.980315936285	-1.247245528902
-0.980183589369	-1.247228367212
-0.980064366246	-1.247117115716
-0.979936923579	-1.246752466313
-0.979796867779	-1.246599025004
-0.979690483246	-1.246558795491
-0.979569822043	-1.24637603643
-0.979442201028	-1.246184559214
-0.97931147759	-1.245884958662
-0.979193148252	-1.245852354521
-0.979078832576	-1.24576634187
-0.978954268014	-1.24576358452
-0.978836317033	-1.245748461332
-0.978692038788	-1.245692889276
-0.978577627223	-1.245632778949
-0.978471110445	-1.245534446234
-0.978371966597	-1.245512997148
-0.978238863844	-1.245278479447
-0.978128227663	-1.245237453751
-0.978016578923	-1.244915319638
-0.977889757364	-1.24489793725

-0.977793344802	-1.244888702259
-0.977661807404	-1.24460599161
-0.97754115467	-1.244344331457
-0.977394757002	-1.244340055291
-0.977285910744	-1.244244419282
-0.977172388243	-1.244183538131
-0.97705983523	-1.244105754824
-0.976953355002	-1.24402531744
-0.976841137462	-1.244012653814
-0.97672818569	-1.243772421328
-0.976603623219	-1.243745010139
-0.976466629042	-1.243735626945
-0.976351646516	-1.243555293659
-0.976213318319	-1.243241232532
-0.976104683718	-1.243154762385
-0.975980398003	-1.243143175996
-0.975870206482	-1.243102548109
-0.97575394253	-1.242984140614
-0.975629143556	-1.242626653516
-0.975521407926	-1.242318159357
-0.975399193532	-1.242206607301
-0.975285467516	-1.242193198035
-0.975170083709	-1.241827265202
-0.975055200049	-1.241735234302
-0.974945967864	-1.241663498102
-0.974813926549	-1.241655880934
-0.974685269995	-1.241596971416
-0.97455363125	-1.24156912746
-0.974449317557	-1.241339423321
-0.974337650613	-1.24120595285
-0.97420065886	-1.241189291421
-0.974101012774	-1.241064221146
-0.973974597924	-1.24096139776
-0.973861542258	-1.24089847764
-0.973751440656	-1.240748547601
-0.973649303294	-1.24052373055
-0.973522589869	-1.240080925592
-0.973393915236	-1.239900634883
-0.973288850007	-1.239790080395
-0.973179176314	-1.239300517171
-0.97305628419	-1.239209026471
-0.972934585682	-1.239154143209
-0.972818588924	-1.239096007754
-0.972716433373	-1.239012331155
-0.972592229126	-1.238976007426
-0.972476758638	-1.238826185583
-0.972354194307	-1.23872224773
-0.972221848376	-1.238540928596
-0.972124513578	-1.238311132829
-0.971988065251	-1.238031161255
-0.971878235763	-1.237899251246
-0.971775261402	-1.237799817593
-0.971632365405	-1.23766688724
-0.9714897149	-1.237284189805

-0.971381644645	-1.23716023439
-0.971262960969	-1.236818992437
-0.971163624907	-1.236684629878
-0.971059467801	-1.236563512399
-0.970929036957	-1.236414103762
-0.97079859045	-1.236276358703
-0.97068526225	-1.236265324231
-0.970543165142	-1.236239920736
-0.970433037341	-1.236120717443
-0.970293936965	-1.236114057656
-0.970179554976	-1.236017119305
-0.970080603294	-1.235859566799
-0.969976594606	-1.235830781631
-0.969859510949	-1.235807444045
-0.969725903897	-1.23580447804
-0.969606423857	-1.235650580072
-0.969484288552	-1.235534597478
-0.969327843788	-1.235237350796
-0.969190702745	-1.235204627001
-0.969075879687	-1.235072577313
-0.968946445559	-1.234774932812
-0.968869971388	-1.234729369288
-0.968775102758	-1.234699133418
-0.968635768855	-1.234482588919
-0.968520933158	-1.234437787185
-0.968386543678	-1.234307177666
-0.968224707717	-1.234301730803
-0.96808920659	-1.234215625633
-0.967947577417	-1.234089632279
-0.967802116183	-1.234030331304
-0.967686554878	-1.233953755393
-0.967577187577	-1.233817439342
-0.967449434764	-1.233816602874
-0.967362576732	-1.233677190319
-0.967250508951	-1.233515339985
-0.967121345332	-1.233435640629
-0.966989479473	-1.233427145293
-0.966894676912	-1.233152662736
-0.966772629036	-1.233100502949
-0.966651632037	-1.232941638087
-0.966528045902	-1.232927619609
-0.966408503952	-1.232851042367
-0.96627967375	-1.23269818517
-0.96615491747	-1.232564953234
-0.966050172162	-1.231988184152
-0.965923857716	-1.231842433189
-0.96580766555	-1.231725546836
-0.965674166474	-1.231713269844
-0.965573776816	-1.231615323787
-0.965458405122	-1.231469161301
-0.965355251962	-1.231395130858
-0.965251974366	-1.231350430435
-0.965122955694	-1.231264794339
-0.965021400873	-1.231146578374

-0.964910408902	-1.230924473333
-0.964782601396	-1.230873023941
-0.964667842273	-1.230663388765
-0.964551237332	-1.230460451809
-0.964430622368	-1.230143181318
-0.964333253009	-1.229977549078
-0.964207629488	-1.229732475599
-0.964089436865	-1.229479548432
-0.963989776354	-1.229207432888
-0.963863613304	-1.22913213207
-0.963744957455	-1.229088989191
-0.963640955513	-1.22901295707
-0.963550571642	-1.228967403701
-0.963420359557	-1.228953513047
-0.963289859246	-1.228927722465
-0.963186312989	-1.228700173147
-0.963062126591	-1.228623328036
-0.962946959993	-1.228512746906
-0.962833801395	-1.228091958888
-0.962697988712	-1.228044964551
-0.9625921638	-1.227903107396
-0.962489569268	-1.227476242839
-0.96236970558	-1.227428495755
-0.962264189377	-1.227059530111
-0.96216295536	-1.227028364804
-0.962056679319	-1.226780810901
-0.961958688295	-1.226449175906
-0.961841661237	-1.226412821478
-0.961711679265	-1.226346543784
-0.961615451337	-1.226339404713
-0.961479275122	-1.225900751723
-0.961364622097	-1.225802472014
-0.961239292151	-1.225624009325
-0.961118408731	-1.225607226565
-0.961013486268	-1.225563149101
-0.960899149266	-1.225520871664
-0.960753048627	-1.22544932374
-0.960660478465	-1.225328036626
-0.960528151624	-1.225277113714
-0.960400792772	-1.225052244127
-0.960291682507	-1.22488715227
-0.960163895294	-1.224828944966
-0.960029251671	-1.224643439259
-0.959932880925	-1.22440597588
-0.959810698027	-1.224327527533
-0.95966774233	-1.224321645222
-0.959548915526	-1.224315281819
-0.959414924123	-1.224094436627
-0.959296947539	-1.223894124458
-0.959172066346	-1.223863678061
-0.959069930546	-1.223675426927
-0.958951941151	-1.223516852793
-0.958858162608	-1.223302035402
-0.958750398716	-1.22295759443

-0.958629701759	-1.222880176526
-0.958506721828	-1.222771317357
-0.958377957998	-1.222658911553
-0.958262629234	-1.222658010517
-0.958151732426	-1.222555028901
-0.958020153954	-1.222543791902
-0.957858020357	-1.222490838384
-0.957750937727	-1.222316249753
-0.957639647886	-1.222215175621
-0.957541369683	-1.222155996459
-0.957398577326	-1.222149733634
-0.957289050521	-1.222146453773
-0.957184802718	-1.222134466659
-0.957069379537	-1.221913534672
-0.956956760011	-1.22168751786
-0.956848459751	-1.221563299768
-0.956740385642	-1.221377136047
-0.956641833758	-1.221371184972
-0.956536823324	-1.221326666552
-0.956420233804	-1.221234347124
-0.956308819676	-1.221128690306
-0.956193588536	-1.221028953351
-0.956090778019	-1.221009241392
-0.955966985059	-1.220991652614
-0.955850572759	-1.220602225231
-0.955734374304	-1.22044482785
-0.955602791881	-1.220419479853
-0.955496685901	-1.220244980388
-0.955392206672	-1.220226618885
-0.955265011179	-1.220188539511
-0.955149913642	-1.220025443089
-0.955027236947	-1.219971183992
-0.95490422005	-1.219323976406
-0.95477525598	-1.219134022126
-0.954658236026	-1.219119427948
-0.954534116214	-1.219068259157
-0.95440987654	-1.218972956867
-0.954297666412	-1.218963596648
-0.954201183258	-1.21894955181
-0.954075849096	-1.218548177554
-0.953945900322	-1.218358781482
-0.95383316988	-1.218309359087
-0.953733490438	-1.218226327943
-0.953589707592	-1.217927902913
-0.953462015658	-1.217920459792
-0.953349137718	-1.217637503479
-0.953208397809	-1.21747655347
-0.953104025257	-1.217277368859
-0.953002602405	-1.216887163689
-0.952871009075	-1.216756807011
-0.952777577503	-1.216720670793
-0.952647433411	-1.216657731288
-0.952543499367	-1.216501924225
-0.952428733438	-1.216486242525

-0.952320657746	-1.216474426902
-0.952212945949	-1.216462818682
-0.952109419705	-1.216400633157
-0.951990737003	-1.216306948988
-0.951871593193	-1.216230581427
-0.951767797258	-1.216093005538
-0.951654559089	-1.21603784987
-0.951549197679	-1.215927574085
-0.951422896829	-1.215704789535
-0.951316610073	-1.215559571542
-0.951211411392	-1.215484745195
-0.951101078565	-1.215400292107
-0.950994155261	-1.214905014382
-0.950868078289	-1.214884481904
-0.950756561048	-1.214309700964
-0.950636026745	-1.214252125985
-0.950529946144	-1.214194710414
-0.95043111883	-1.214173860487
-0.950299356186	-1.213972042015
-0.950185226174	-1.213944932237
-0.950064255092	-1.213874711422
-0.949958358146	-1.213817998199
-0.949835177005	-1.213288248563
-0.949716605988	-1.21318051294
-0.94958070115	-1.212801194677
-0.949452783406	-1.212437186036
-0.949329819107	-1.212130444465
-0.949215250333	-1.212073084142
-0.94907535232	-1.211873731206
-0.948950029622	-1.211783114607
-0.948841400164	-1.21148936284
-0.948752765721	-1.210905840339
-0.948655723924	-1.210869555804
-0.948539401392	-1.210790170869
-0.948419365736	-1.210450502227
-0.948312122457	-1.210430946374
-0.94819265204	-1.21040795323
-0.948075672233	-1.21037424506
-0.947962327285	-1.21032600424
-0.94785621058	-1.20978172441
-0.947743316405	-1.209674731701
-0.94762171785	-1.209505520976
-0.947519070369	-1.209323530344
-0.947391806166	-1.209283658562
-0.947260872656	-1.208983591111
-0.947148226979	-1.208891162553
-0.947013890465	-1.208405884156
-0.946887216925	-1.208213047349
-0.946764059057	-1.208001240902
-0.946665294498	-1.207879551602
-0.946539044766	-1.207851145183
-0.946429515655	-1.20771004155
-0.946290689426	-1.207660428518
-0.946180806744	-1.207348704527

-0.946055037573	-1.207325022282
-0.945941206142	-1.207304393595
-0.945820801045	-1.207282462228
-0.945702253123	-1.207222820276
-0.945572232623	-1.20720747889
-0.945452774936	-1.206933312779
-0.945351143868	-1.206877696656
-0.945233694407	-1.206780685735
-0.94509591718	-1.206673324785
-0.944981644764	-1.206330846532
-0.944872169676	-1.206074346202
-0.944739613935	-1.20602412394
-0.944619944254	-1.205988771472
-0.944499071316	-1.205849909232
-0.944392369574	-1.205708301756
-0.944282808844	-1.205669172192
-0.944176445952	-1.205624679536
-0.94407317964	-1.2051375093
-0.943957480863	-1.205071532746
-0.943849867058	-1.204935208445
-0.943722509234	-1.204730329416
-0.943617188588	-1.20472936443
-0.943509149139	-1.204398108833
-0.943405720474	-1.20438522686
-0.943288673626	-1.20432861699
-0.943183869083	-1.204211250974
-0.943048137128	-1.204135238744
-0.942939372292	-1.204105397407
-0.942816610276	-1.203972830197
-0.942708835545	-1.203875294439
-0.942598421854	-1.203830546465
-0.942495331383	-1.203559948977
-0.942360506908	-1.20346676181
-0.942257080436	-1.203062750424
-0.942166868998	-1.202941785779
-0.942042993817	-1.202865322512
-0.941943742185	-1.202853076634
-0.941825432595	-1.202732753521
-0.941718853199	-1.202720739153
-0.941606170783	-1.202556869605
-0.941484960314	-1.202532399128
-0.941382054544	-1.202436574922
-0.941249036178	-1.202411269291
-0.941120721553	-1.201535925884
-0.941008124091	-1.201331797389
-0.940908290353	-1.201294104495
-0.940780798576	-1.200917275516
-0.940659081426	-1.200854056705
-0.940542413472	-1.200685504975
-0.940441880134	-1.2006607661
-0.940324166169	-1.200364659724
-0.940221183675	-1.200292998206
-0.940104717651	-1.200249076408
-0.940005891732	-1.200193765176

-0.93988371619	-1.200028362279
-0.939788040342	-1.199962469162
-0.939680671796	-1.1998054705
-0.93957450288	-1.199791988868
-0.939471691811	-1.199724313949
-0.939355555887	-1.199671640954
-0.939253546708	-1.198985459796
-0.939125111752	-1.198723384628
-0.93901886957	-1.1986560098
-0.938901365787	-1.198601875051
-0.938793042925	-1.198566825266
-0.938683586539	-1.198406199023
-0.938564917082	-1.198264353421
-0.938474543677	-1.198214365763
-0.938372130985	-1.198183131917
-0.938258490546	-1.19723524954
-0.938145194422	-1.197123266262
-0.938018247853	-1.197121094823
-0.937892610116	-1.196401171364
-0.937771989986	-1.196328315662
-0.937652123114	-1.196235255524
-0.937539807402	-1.196175853585
-0.93743860952	-1.19609065637
-0.937334310101	-1.195918356149
-0.937212636451	-1.195865403937
-0.937073319398	-1.195578766335
-0.936951634401	-1.195178884641
-0.936827605234	-1.195000906151
-0.936721683443	-1.194976540519
-0.936596675371	-1.194783575591
-0.936478921619	-1.194648816019
-0.936363838687	-1.194642989544
-0.936243720267	-1.194629706804
-0.936126504826	-1.19418515056
-0.936012175409	-1.194087476467
-0.935883607232	-1.193985267507
-0.935785380458	-1.193830747844
-0.935689054426	-1.193673804867
-0.935559651401	-1.193525509618
-0.935439337619	-1.193209707018
-0.935323357698	-1.193071156632
-0.935214207977	-1.192991062635
-0.935087741947	-1.192965591784
-0.934984281265	-1.192854972662
-0.934871521757	-1.192792345779
-0.934762752681	-1.192674081935
-0.934660561447	-1.192476677903
-0.934571403002	-1.192417659364
-0.934469902708	-1.192114417407
-0.934338261779	-1.191996874961
-0.934236738489	-1.191996749798
-0.934129675976	-1.191945635771
-0.934028616018	-1.191812898152
-0.933920241442	-1.191566921656

-0.933824736456	-1.191337705627
-0.933700117547	-1.191031277675
-0.933581139263	-1.190796191039
-0.933472212877	-1.190557483948
-0.933345260752	-1.190370177972
-0.933258266165	-1.19028797579
-0.933157437862	-1.190118829618
-0.933075442238	-1.189734487861
-0.932970406442	-1.189459634848
-0.932832204591	-1.189444264909
-0.932709660083	-1.18921791832
-0.932593780371	-1.18920373392
-0.932483078879	-1.189018872043
-0.932366423029	-1.18880563552
-0.932246332748	-1.188672911976
-0.932138761074	-1.188608467154
-0.932025659036	-1.188304648024
-0.931899355029	-1.188217923741
-0.931777950869	-1.188120411467
-0.931649884923	-1.188109806046
-0.931512390742	-1.188107059393
-0.931392332567	-1.188051049747
-0.931292944297	-1.187788326983
-0.931195880255	-1.187739773447
-0.931073064167	-1.187657338767
-0.930950589559	-1.18752219076
-0.93082987367	-1.187379942234
-0.930723322192	-1.187322574077
-0.930605613121	-1.187265933631
-0.930492964049	-1.187199761318
-0.930382268242	-1.18695584976
-0.930270327559	-1.186953946596
-0.930152614217	-1.186845418524
-0.930041715399	-1.186625830569
-0.929940122662	-1.186611619924
-0.929841557421	-1.186597029427
-0.929728002548	-1.18646111166
-0.929605838976	-1.186417453638
-0.929485702193	-1.186286335499
-0.929360294221	-1.18600812911
-0.929244400881	-1.18586315053
-0.929133256123	-1.185853268813
-0.929021499889	-1.185790652166
-0.928904720113	-1.185735399516
-0.928814124609	-1.185718589849
-0.928696834422	-1.185611653288
-0.928579451845	-1.185538080346
-0.92845038837	-1.185435865364
-0.928334498743	-1.185375520295
-0.928209751404	-1.185333420906
-0.928068377537	-1.185228126367
-0.927927559821	-1.185171336788
-0.927811951734	-1.185017291002
-0.927693438365	-1.18497061067

-0.927588665927	-1.184725885938
-0.927478397332	-1.184535538321
-0.927387356355	-1.18444892071
-0.927276402543	-1.184238563274
-0.92715761484	-1.18397125282
-0.927062039748	-1.183936552963
-0.926938646332	-1.183826483419
-0.926840165661	-1.18368461292
-0.926721984764	-1.183469130446
-0.926598617807	-1.183386767882
-0.926487158036	-1.182804919827
-0.926359658285	-1.182757979007
-0.926243250172	-1.182755208415
-0.92613832754	-1.182682265572
-0.925995609681	-1.182476083424
-0.92587680947	-1.18239828401
-0.925759366776	-1.182279194202
-0.925635744444	-1.182155531417
-0.925520822534	-1.182144226411
-0.925419529092	-1.181950705062
-0.92530873845	-1.181905459359
-0.925214296049	-1.181839568315
-0.925095613478	-1.181670353974
-0.92500209165	-1.18163442354
-0.924904485968	-1.181568137862
-0.924767860854	-1.181480043181
-0.924664080256	-1.181449695125
-0.924558216327	-1.181392536758
-0.924460350728	-1.181285367799
-0.92434568611	-1.181270120865
-0.924204675394	-1.181162109892
-0.924097886889	-1.181144536834
-0.923980516891	-1.180667510489
-0.923850778947	-1.180577980871
-0.923753208589	-1.180419155944
-0.923647250169	-1.179928200743
-0.923544698843	-1.179838737223
-0.923428585929	-1.179835702256
-0.923328912864	-1.179757148451
-0.923228541204	-1.179524430505
-0.923099358302	-1.179338075605
-0.922973582533	-1.179226277883
-0.922867201837	-1.179124040985
-0.922760080264	-1.179111645743
-0.922642726034	-1.179037804366
-0.922530330913	-1.179024050519
-0.922400869057	-1.178965936999
-0.922282356841	-1.178737542614
-0.922169367071	-1.178698917309
-0.922045833476	-1.178658944451
-0.921944367846	-1.178413156581
-0.921834177522	-1.178315447343
-0.921710449585	-1.178313047179
-0.921602262271	-1.178069823109

-0.921484505114	-1.178040224364
-0.921370827149	-1.178025978388
-0.921275396897	-1.177992413376
-0.921153644689	-1.17776226187
-0.921027126821	-1.177362838522
-0.920898558316	-1.17731115974
-0.920802639802	-1.177226238372
-0.920688597102	-1.177188703356
-0.920584931885	-1.177075823149
-0.920477517853	-1.176865910445
-0.920383956909	-1.176593957419
-0.92025838218	-1.176583225348
-0.920133061142	-1.176475509406
-0.920009606374	-1.176423516987
-0.919892135487	-1.17630131362
-0.919761335931	-1.176080571333
-0.919659588581	-1.176030497572
-0.919532126382	-1.176024821497
-0.919418578848	-1.175928802854
-0.919311834848	-1.175912357205
-0.919186567935	-1.175713660708
-0.919072312483	-1.175534448718
-0.918974023314	-1.175366653459
-0.918849580446	-1.17528835272
-0.918716133911	-1.175260829624
-0.918588187929	-1.175252801551
-0.918459650464	-1.175227182532
-0.918355979034	-1.174779433973
-0.918264478211	-1.174714744456
-0.918116835924	-1.174569119534
-0.918025254316	-1.174198372006
-0.91792524382	-1.173925934849
-0.917816214988	-1.173835831688
-0.917709919925	-1.173825845708
-0.917597730646	-1.173777443587
-0.917467946772	-1.173534707963
-0.917378358968	-1.173406297926
-0.917262091743	-1.173368659956
-0.917139636079	-1.173353429256
-0.917033115695	-1.173311360851
-0.916953846581	-1.173143458875
-0.916844055448	-1.173011092475
-0.916733556074	-1.172912549338
-0.916619645988	-1.172578845022
-0.916513338834	-1.172498443514
-0.916387662416	-1.172494857921
-0.916266657093	-1.172302011647
-0.916143265827	-1.172289308751
-0.916022921469	-1.172276691246
-0.915897603606	-1.172241862729
-0.915790153159	-1.172019071586
-0.915669273338	-1.171938300041
-0.915567743202	-1.171666567067
-0.915451198899	-1.171193251261

-0.915368359558	-1.171163890623
-0.915252445362	-1.171003283352
-0.915123786389	-1.17087972179
-0.915024922179	-1.170808095125
-0.914929098097	-1.170790286695
-0.914816111685	-1.170695156327
-0.914692483968	-1.170652317122
-0.91458135028	-1.170630316786
-0.91446650575	-1.170545668965
-0.914337951523	-1.170459838283
-0.914217181421	-1.170129155682
-0.9141209365	-1.169655672921
-0.913991808431	-1.169600017514
-0.913883840607	-1.16946189694
-0.913780585875	-1.169461589926
-0.91366694151	-1.169269189698
-0.913542765672	-1.16926159812
-0.913417603834	-1.16912645366
-0.91331514166	-1.168994979989
-0.913189206746	-1.168785535019
-0.913073763693	-1.168755998512
-0.912951852195	-1.168551111728
-0.912825247552	-1.168372827433
-0.912708175646	-1.168280953592
-0.912592338798	-1.16800952247
-0.912468346266	-1.167985818851
-0.912375535175	-1.167879207259
-0.91226597865	-1.167842203182
-0.912166001082	-1.167717395235
-0.912073522018	-1.167273509183
-0.911929311551	-1.167115971909
-0.911805790994	-1.167013457378
-0.911693959761	-1.166950559604
-0.911561877543	-1.166884933319
-0.91143871418	-1.166029978464
-0.911339694723	-1.165973570159
-0.911239376661	-1.165870106082
-0.911122996684	-1.165756681111
-0.911016753417	-1.165410351862
-0.910924026879	-1.165394435391
-0.910810520475	-1.165248781955
-0.910708433247	-1.16523399722
-0.910593101065	-1.165164241407
-0.910493628614	-1.165091784643
-0.910386630371	-1.164985977279
-0.910282316797	-1.164799343272
-0.910151509294	-1.164744070919
-0.910054527632	-1.164638154937
-0.909945375765	-1.164476148533
-0.90984103569	-1.164465479881
-0.909724940049	-1.16427169822
-0.909611030758	-1.164142446916
-0.909510368461	-1.164085101684
-0.909398490569	-1.163825509017

-0.909285576969	-1.16358770974
-0.909168064539	-1.16352688875
-0.90907212904	-1.163218770098
-0.908958657223	-1.163192083964
-0.908840270477	-1.163157708374
-0.908722930914	-1.163136351482
-0.908622471025	-1.163135376982
-0.908514838327	-1.162840722507
-0.908403038798	-1.162679812758
-0.908310907095	-1.162616300276
-0.908200893089	-1.162377443242
-0.908092726085	-1.162253349276
-0.907988565352	-1.16206578795
-0.907866003157	-1.16176034827
-0.90773903226	-1.161311229722
-0.907625945559	-1.161190963163
-0.907493872937	-1.161156780823
-0.907391630402	-1.16112744349
-0.907281515926	-1.161071033522
-0.907157627442	-1.160630962381
-0.907062638059	-1.160215165857
-0.9069461225	-1.160184070423
-0.906811949593	-1.160120522555
-0.906678777595	-1.159803305274
-0.906563657114	-1.159750780765
-0.906462623776	-1.159693745801
-0.906337129768	-1.159418509347
-0.906220359824	-1.159415471579
-0.906111808693	-1.159197612551
-0.905984866108	-1.15915806917
-0.905859645586	-1.159056512559
-0.905741684709	-1.159040260554
-0.905641761876	-1.158983728704
-0.905526743327	-1.158911353753
-0.905408816914	-1.158811879712
-0.905319041145	-1.158789477032
-0.905217933344	-1.158498934091
-0.905110998703	-1.158374782141
-0.904974638751	-1.158353857541
-0.904860924865	-1.158274617635
-0.904743183807	-1.158137211354
-0.904631777228	-1.157518847175
-0.904503452064	-1.15673760741
-0.904405511216	-1.156661179024
-0.904299136511	-1.156526663247
-0.904177984546	-1.156272858497
-0.904082467662	-1.156117259792
-0.903976243515	-1.156097520224
-0.903860821447	-1.155934026273
-0.903744674951	-1.155925393341
-0.903652796949	-1.155790058193
-0.903531500157	-1.155741152784
-0.903404502521	-1.155568410796
-0.903286719749	-1.155083289586

-0.903173164725	-1.154805620233
-0.903048126361	-1.154770837103
-0.90294039626	-1.154710463651
-0.902831579901	-1.154192485672
-0.902710614769	-1.15414442608
-0.902599156476	-1.154055145585
-0.902474493507	-1.15389067714
-0.902363332315	-1.153797908431
-0.902256701679	-1.153685345152
-0.902170335919	-1.153578333464
-0.902072633884	-1.153541145139
-0.901966804269	-1.153446288598
-0.901859707179	-1.153279067818
-0.901728906231	-1.153267378274
-0.901625607917	-1.153198740888
-0.90148901488	-1.153195960336
-0.901361868965	-1.153165004457
-0.901258763826	-1.153027656958
-0.901155748869	-1.152853537272
-0.901048655738	-1.152843087557
-0.900933931852	-1.152570464581
-0.900830075309	-1.152373245422
-0.900730938327	-1.15229087029
-0.900602521507	-1.152259816837
-0.900492210955	-1.152208116284
-0.900379056712	-1.151818115469
-0.900263885487	-1.151736049863
-0.900145022961	-1.151529318756
-0.900038755038	-1.151522538502
-0.899942925923	-1.151153267353
-0.899837900374	-1.151093506444
-0.899730172178	-1.151069691406
-0.899624095655	-1.15074069255
-0.899521195968	-1.150691825051
-0.89940448819	-1.150675788892
-0.8992677365	-1.1500410484
-0.899153502819	-1.149986698448
-0.899040895776	-1.14990048496
-0.898939181681	-1.14984487459
-0.898840379569	-1.149588509286
-0.898723516632	-1.149580057472
-0.89859963742	-1.14957327545
-0.898494133942	-1.149043531898
-0.898381943388	-1.149018986816
-0.898275520415	-1.148976589222
-0.89816070132	-1.14895935372
-0.898050229633	-1.148151602487
-0.897959443032	-1.14811772786
-0.897853181693	-1.148003045081
-0.897720473398	-1.147731834542
-0.897608220983	-1.147495197969
-0.897507043853	-1.147483319996
-0.897406626309	-1.147480346711
-0.897305551611	-1.147267965084

-0.897192344049	-1.147253292155
-0.897080484719	-1.147147342555
-0.89697056724	-1.147116225822
-0.896857270219	-1.146891182522
-0.896754510638	-1.146717597842
-0.896635042631	-1.146499996041
-0.896533834092	-1.146444772898
-0.896421531286	-1.146418532316
-0.896314489587	-1.145741465397
-0.896214572324	-1.145607464505
-0.896087783611	-1.145568104197
-0.896003936686	-1.145545211094
-0.895879844669	-1.145359810997
-0.895779755275	-1.145320581406
-0.895645633875	-1.145032239083
-0.89553878925	-1.144819426235
-0.895402569107	-1.144437952419
-0.895294697726	-1.144434400237
-0.895168433171	-1.144325701776
-0.895064775483	-1.144312173572
-0.894953911321	-1.144262890133
-0.894826384956	-1.144179647452
-0.894709711964	-1.144031398524
-0.89458369367	-1.143422919233
-0.894476103285	-1.143405190614
-0.894371467382	-1.143333022922
-0.894256528319	-1.143269161389
-0.894146190612	-1.143023530596
-0.894057609789	-1.142857302661
-0.893960723534	-1.142749193851
-0.893834887617	-1.142567267954
-0.893731667922	-1.142105589
-0.893630885076	-1.142061382903
-0.893537261785	-1.141975210601
-0.893423128729	-1.1418795463
-0.893318602819	-1.141715505158
-0.893201418823	-1.141692301498
-0.893070931372	-1.14144711485
-0.892936561935	-1.141384356622
-0.892831106246	-1.141109876849
-0.892713599913	-1.141067219331
-0.892617464784	-1.140989881872
-0.892499120707	-1.140818656574
-0.892380775553	-1.14075535697
-0.892268173859	-1.140548503407
-0.892161871284	-1.140494572744
-0.892038475662	-1.140346354753
-0.891939386365	-1.140167035295
-0.891821848594	-1.140014350223
-0.891708666911	-1.139345678509
-0.891587652721	-1.13914948857
-0.891487895771	-1.139130459997
-0.89137995168	-1.13911278719
-0.891280385093	-1.139022172651

-0.891174039804	-1.138897919727
-0.891054276873	-1.138806661105
-0.890951514921	-1.138704784478
-0.890845499982	-1.13860108579
-0.890746713612	-1.138337010158
-0.890638486267	-1.137942116095
-0.89052712767	-1.137887572978
-0.890415756573	-1.137736269298
-0.89031744272	-1.137722666199
-0.890209840922	-1.137639976323
-0.890090511728	-1.13745517254
-0.889982883101	-1.137333772808
-0.889894749656	-1.137148549531
-0.889777889917	-1.13713376558
-0.889646167825	-1.137073766905
-0.889525756186	-1.137003848549
-0.889415262912	-1.136914983829
-0.889317526311	-1.136860669069
-0.889212885461	-1.136836501402
-0.889091438784	-1.13683221358
-0.888999209499	-1.136479394923
-0.888887459399	-1.136378361148
-0.888758801285	-1.136202545951
-0.888652991425	-1.135887124621
-0.888525860663	-1.135876522705
-0.888412819524	-1.13552931781
-0.888314549629	-1.135472381157
-0.888202704435	-1.135379957461
-0.888102816749	-1.135162749095
-0.888011250909	-1.135139006071
-0.887918706039	-1.134883513278
-0.88778138387	-1.134808520983
-0.887655314486	-1.134657203881
-0.887549938029	-1.1346497718
-0.887433354435	-1.13452080546
-0.887326771068	-1.134409852655
-0.887207377806	-1.13435366274
-0.887091969793	-1.134237656032
-0.886960856021	-1.134214026735
-0.886849891487	-1.134076316924
-0.886743793775	-1.134071341129
-0.886631948188	-1.134049720925
-0.886513850969	-1.133989614764
-0.886407005114	-1.133828804327
-0.886299670852	-1.133769364949
-0.886196170397	-1.133769295515
-0.886077382303	-1.133701270753
-0.885969046477	-1.133318623705
-0.885864232859	-1.133308122385
-0.88575062273	-1.133292028215
-0.885657223783	-1.13324390141
-0.885552130564	-1.132947228023
-0.885445905624	-1.132915222082
-0.885342349572	-1.132811913937

-0.885238985607	-1.132619157916
-0.885139306337	-1.132582031143
-0.885009853359	-1.132458629357
-0.884906544916	-1.132398158003
-0.88479846472	-1.132225060271
-0.884698166155	-1.13185922126
-0.884590135694	-1.131489976528
-0.884479585812	-1.131300944917
-0.884360806847	-1.131210552099
-0.884254834999	-1.131150417283
-0.884133406262	-1.131136418566
-0.884008691453	-1.13094014735
-0.88390972056	-1.130809857013
-0.883769165968	-1.130665353778
-0.88363812855	-1.130507976335
-0.883540556325	-1.130370985462
-0.883428708281	-1.130302671833
-0.883317180181	-1.130287290403
-0.883212623516	-1.130062425634
-0.883113490681	-1.130015011434
-0.883003355909	-1.130000849538
-0.882894645786	-1.129861977806
-0.882792470226	-1.12977487193
-0.882673942703	-1.129664351196
-0.882570367333	-1.129605253854
-0.882456539575	-1.129457592863
-0.88234385866	-1.129333506641
-0.882241162837	-1.129129570058
-0.882143591771	-1.129085787188
-0.882025363641	-1.129057181511
-0.881929917383	-1.128741762427
-0.881814555674	-1.128701812036
-0.881716257445	-1.128522829588
-0.881608860144	-1.128052016532
-0.881506994795	-1.127943854679
-0.881434617766	-1.127761788781
-0.881305350833	-1.12736491991
-0.881181449516	-1.127260419329
-0.881063497569	-1.127004082102
-0.880936330681	-1.126857508918
-0.880819719131	-1.126849270208
-0.88071107643	-1.126778795343
-0.880594803328	-1.126775370333
-0.88048326775	-1.126752502645
-0.880368631122	-1.126530159359
-0.880268717173	-1.126431700161
-0.880153051793	-1.126022378088
-0.88002955876	-1.125978602146
-0.879924458177	-1.12586075906
-0.879839180637	-1.12534277314
-0.879729365445	-1.125169814505
-0.87958592922	-1.124664310511
-0.879487904233	-1.124598715122
-0.879378228445	-1.12448485216

-0.879270669132	-1.124365132335
-0.879166405573	-1.124183530375
-0.879043461542	-1.12412070056
-0.878919056059	-1.123998253305
-0.878794024117	-1.123967070812
-0.878667690362	-1.123864092204
-0.878552878718	-1.123864067893
-0.878458351013	-1.123664708749
-0.878366277059	-1.123492049742
-0.878273140318	-1.123310720342
-0.878184830726	-1.123251423048
-0.878061009302	-1.123041075702
-0.877940355694	-1.12301576471
-0.877843054606	-1.122983493696
-0.877723806675	-1.122970679709
-0.877600859298	-1.12291647974
-0.877495810821	-1.12286967132
-0.877387116658	-1.122583850407
-0.877280723254	-1.122465606884
-0.877183933838	-1.122290160417
-0.877095423502	-1.122035649249
-0.876975420683	-1.121984357963
-0.87686676998	-1.121823734844
-0.876765526146	-1.121807662786
-0.876654085004	-1.121712657454
-0.876554765942	-1.12161176353
-0.876470229105	-1.121465616299
-0.876372500192	-1.12138460529
-0.876252578961	-1.121364804863
-0.876154932815	-1.121055056381
-0.876032800802	-1.120913618361
-0.875947288557	-1.120454135198
-0.875838980448	-1.120317215927
-0.875723853285	-1.120241761005
-0.875625801268	-1.119847358946
-0.875525393597	-1.119745150913
-0.87540802097	-1.119649339544
-0.875287839388	-1.119581223396
-0.875191829836	-1.11955562247
-0.875073871244	-1.119349055526
-0.874966192248	-1.119336921406
-0.874875196213	-1.119136739017
-0.874758465734	-1.119014717389
-0.874646183371	-1.118532650439
-0.874524729131	-1.118358503242
-0.874403502817	-1.118342032708
-0.874294181225	-1.118300259605
-0.874182156292	-1.118223011624
-0.874097939388	-1.118102143755
-0.874007110077	-1.118095913222
-0.873894881326	-1.117985968715
-0.87378250477	-1.117788534684
-0.873671567126	-1.11778810314
-0.873583085462	-1.117787424258

-0.873475701957	-1.117740397945
-0.873381281342	-1.117697264812
-0.873271647539	-1.117570192029
-0.873171965259	-1.11754622902
-0.87307674017	-1.117544304702
-0.872962801782	-1.11734595251
-0.872856982641	-1.117276005544
-0.87276214818	-1.117233533999
-0.872645903402	-1.116963412063
-0.872545898835	-1.11691887802
-0.87245269384	-1.116912550634
-0.872353342104	-1.116879122215
-0.872215813019	-1.116068946406
-0.872093929786	-1.116045844843
-0.871986850724	-1.115837102894
-0.871872575747	-1.115810643148
-0.871770594957	-1.115805613813
-0.871642214204	-1.115782089506
-0.871537013408	-1.115749234133
-0.871400511378	-1.115384739927
-0.871304105892	-1.115235775717
-0.871206394911	-1.115219210197
-0.871109018166	-1.114902108332
-0.870980014617	-1.114762557853
-0.870859920003	-1.114675908662
-0.870751170013	-1.114662626993
-0.870645389567	-1.114624158131
-0.870537923121	-1.11455202365
-0.870430205358	-1.114529539194
-0.870327979462	-1.114447100689
-0.870223368038	-1.114442719734
-0.870111439054	-1.11443237141
-0.870007534337	-1.114263512942
-0.8699089648	-1.113882434607
-0.869810002392	-1.113874690311
-0.869710007904	-1.113864598083
-0.86959690542	-1.113543646187
-0.869521445317	-1.113535185623
-0.869393889285	-1.113529966589
-0.869290927147	-1.113325434045
-0.869169029726	-1.113297861971
-0.86906446779	-1.113262793893
-0.868963457105	-1.113109018347
-0.86884471273	-1.112843221941
-0.86872535552	-1.112799481485
-0.868638651	-1.112695450105
-0.868528997838	-1.112564657038
-0.868433224668	-1.112311871061
-0.868309394243	-1.112217497695
-0.868214971322	-1.112184136169
-0.86809677757	-1.112171263768
-0.867989652037	-1.112024904957
-0.867897874	-1.112005272154
-0.867785190516	-1.111964618858

-0.867679763921	-1.111927412387
-0.867555302777	-1.11170348103
-0.867441011639	-1.111667159479
-0.867349704948	-1.111381707077
-0.86723054076	-1.111283322074
-0.867106755318	-1.111226721718
-0.86700027151	-1.110948553809
-0.866875040917	-1.110586309139
-0.866759072646	-1.11058154315
-0.866633965347	-1.11052011637
-0.866520129662	-1.110458215447
-0.866425016149	-1.110451867293
-0.86632329653	-1.110406848307
-0.866211889326	-1.110400162803
-0.866101924638	-1.110380444528
-0.865990466682	-1.110332721015
-0.865878529032	-1.11030578771
-0.865771732716	-1.11009783732
-0.865617020193	-1.110077315089
-0.86553179709	-1.109879363042
-0.865401874933	-1.109478688751
-0.865317647989	-1.109344510665
-0.865230522411	-1.109249727626
-0.865130841865	-1.109116333124
-0.865026902871	-1.109051689106
-0.864932495504	-1.10887928194
-0.864817287132	-1.108838768664
-0.864726378577	-1.108781350385
-0.864597809633	-1.108738113919
-0.864512973647	-1.108621178356
-0.864404154878	-1.108567140299
-0.864295324219	-1.108501183347
-0.864194362171	-1.107671382101
-0.864085181059	-1.107573357801
-0.863989354437	-1.1068601569
-0.863884812103	-1.106776848103
-0.863774881075	-1.106689582261
-0.863684761266	-1.106621310103
-0.863589314847	-1.106376600264
-0.86346771316	-1.106354562914
-0.863370445179	-1.106342253089
-0.863229042956	-1.106300433521
-0.863113853458	-1.106251074207
-0.863011288953	-1.10621198323
-0.862893649274	-1.106204431401
-0.862785740077	-1.105977600309
-0.862683901601	-1.105806207099
-0.862569310446	-1.105790562152
-0.862469674377	-1.105769708752
-0.862359204216	-1.105753566192
-0.862253790676	-1.105722294773
-0.862161399797	-1.10571106136
-0.862052667941	-1.10562729812
-0.861934221912	-1.105604491064

-0.861839159971	-1.105558950386
-0.86174052302	-1.105557626451
-0.861641802991	-1.105546224243
-0.861543692026	-1.105303447375
-0.861415665149	-1.105300694293
-0.861311036601	-1.105249029029
-0.861183192778	-1.105104151953
-0.861083034917	-1.104848667268
-0.860962190945	-1.104609596945
-0.860843404121	-1.104575534675
-0.860733554014	-1.104407133107
-0.860632940029	-1.104366297935
-0.86052729393	-1.104153752888
-0.860429272418	-1.1039411
-0.860311621325	-1.103920106581
-0.860219473312	-1.103781066292
-0.860121616199	-1.103714890852
-0.859995058581	-1.103683027401
-0.85988841868	-1.103544122671
-0.85978451981	-1.103436917996
-0.85968203497	-1.103376340433
-0.859558513696	-1.102964853061
-0.859446155507	-1.102910967978
-0.859333837748	-1.102855656127
-0.859225896853	-1.102752339512
-0.859092860869	-1.102635201877
-0.858988709502	-1.102606389461
-0.858874515044	-1.102412957465
-0.858768560679	-1.102305377983
-0.858664039747	-1.102029551533
-0.858531555759	-1.101704891499
-0.858408341649	-1.10168646725
-0.858316148117	-1.101672263747
-0.85821286776	-1.101657272133
-0.858075125909	-1.101616754477
-0.857965674052	-1.101601906366
-0.857849485586	-1.101191589267
-0.857732366275	-1.101140785838
-0.857637597302	-1.101110073389
-0.85751837767	-1.101076735581
-0.857407423202	-1.101068437842
-0.857307726573	-1.101023618033
-0.857216947296	-1.100989209048
-0.857095882495	-1.100836806217
-0.857005175489	-1.100813797254
-0.856902216645	-1.100677926813
-0.856817886334	-1.100381151285
-0.856683542688	-1.100197987023
-0.856567067001	-1.100000276398
-0.85644981257	-1.099907848389
-0.856346822788	-1.099585976013
-0.856228930601	-1.099532885939
-0.85611717096	-1.099426457642
-0.855980691235	-1.098575461607

-0.855866064896	-1.098471087383
-0.85573390767	-1.098405604648
-0.855632439955	-1.098312309527
-0.855528541	-1.098270900782
-0.855431680319	-1.098248224513
-0.855320223164	-1.09793630182
-0.855189273884	-1.097846732976
-0.855086080468	-1.097814710943
-0.854970416213	-1.097765822881
-0.854866757081	-1.097698780422
-0.854786949298	-1.097626594812
-0.854663015312	-1.097504273304
-0.854574068199	-1.096848670666
-0.854463004453	-1.096756006025
-0.854362703936	-1.096713138131
-0.854247967487	-1.096472700287
-0.854132090524	-1.09645898892
-0.853979707793	-1.096279356814
-0.853853489614	-1.09610890969
-0.853743138745	-1.09599075508
-0.853617440936	-1.095909378462
-0.853493917751	-1.095863226299
-0.853380390314	-1.095854202821
-0.853291004407	-1.095646020859
-0.853194408746	-1.095341223033
-0.853080751197	-1.095248345689
-0.852963758554	-1.09520758569
-0.852847091607	-1.095180889005
-0.852749586409	-1.09509879142
-0.852649384962	-1.094955278595
-0.852545818533	-1.094827402845
-0.852438066945	-1.09438040028
-0.852319914621	-1.094283281346
-0.852201652704	-1.094274804235
-0.852091601645	-1.093974326879
-0.851981932216	-1.093836196537
-0.851876424068	-1.093822179204
-0.851744057725	-1.093539470331
-0.851646764723	-1.093538621333
-0.851536949287	-1.093399832829
-0.851425598118	-1.093137555966
-0.851322014391	-1.093008424492
-0.851207893573	-1.092896746502
-0.851115863738	-1.092653205318
-0.851003739113	-1.09263099568
-0.850881139548	-1.092607604366
-0.850774828621	-1.092502097803
-0.850681102618	-1.092438840474
-0.850572958834	-1.092374226925
-0.850466672924	-1.092271117282
-0.850352594111	-1.092201246087
-0.850254541174	-1.09202683583
-0.850132093276	-1.091690457344
-0.850031820546	-1.091525483938

-0.849899661693	-1.091494195569
-0.849784356131	-1.091455429542
-0.849678416154	-1.091336332378
-0.849590849462	-1.091269863596
-0.849479370973	-1.091040444744
-0.849380452108	-1.090974862313
-0.849259731747	-1.090581373754
-0.849144913416	-1.09019951058
-0.849037741366	-1.089656513519
-0.848909843514	-1.089378603771
-0.848810367407	-1.089167378423
-0.848719738874	-1.089126710313
-0.848588127302	-1.089066626555
-0.84846377371	-1.08905318336
-0.848359862526	-1.088986828242
-0.848253808084	-1.088481421069
-0.848132872078	-1.088458823667
-0.848023698661	-1.088229645398
-0.847906955382	-1.08809459108
-0.847798005655	-1.088069280038
-0.847678535271	-1.087893410418
-0.8475642321	-1.087774847558
-0.847483091481	-1.087714450569
-0.847373765705	-1.087660456461
-0.847270935895	-1.08760822663
-0.847154043845	-1.087467039256
-0.847042199872	-1.08704395585
-0.84693802779	-1.086817848788
-0.846839951971	-1.086654573607
-0.846725788888	-1.086561132993
-0.84660313077	-1.086526279046
-0.846483184135	-1.086421133709
-0.846400630923	-1.08626010666
-0.8462893277	-1.086119895065
-0.846155345608	-1.085973013484
-0.846061378496	-1.085892336075
-0.845942664202	-1.085870833648
-0.845831105969	-1.085819649085
-0.845716999666	-1.085778921155
-0.845605064627	-1.085772884231
-0.845498622173	-1.085536895987
-0.84540041999	-1.085473574388
-0.845307717431	-1.085430613382
-0.845201636243	-1.085292352184
-0.845083486615	-1.085178463362
-0.844957769719	-1.084981947717
-0.844823914433	-1.084669120395
-0.844707597448	-1.084660924122
-0.844600779644	-1.084559776208
-0.844478784536	-1.084537108851
-0.844389077991	-1.084280041388
-0.844282636136	-1.08406335313
-0.844176121392	-1.084036114891
-0.844058654136	-1.08401838047

-0.843958349999	-1.083979428331
-0.843821000239	-1.083856002627
-0.843709172453	-1.083854221513
-0.84360112347	-1.083822260042
-0.843506779903	-1.083691207201
-0.843400914937	-1.083658260002
-0.843298095673	-1.083375003199
-0.843190989656	-1.083255359401
-0.843085279794	-1.08322084551
-0.842971318042	-1.083150493104
-0.842886873536	-1.083057203639
-0.842794540887	-1.083042037299
-0.84267938694	-1.082834746254
-0.842584647272	-1.082744908467
-0.842476259486	-1.082702611691
-0.842385114546	-1.082126379918
-0.842290165209	-1.081956152204
-0.842182206474	-1.081608529846
-0.842063780371	-1.081170615232
-0.8419708496	-1.081165694394
-0.84183106984	-1.081037517367
-0.84173904015	-1.080997561745
-0.841638949401	-1.080698181688
-0.841527258495	-1.08054924464
-0.841426360139	-1.080234874095
-0.841309077775	-1.080118546458
-0.841198633781	-1.079994751069
-0.841062444436	-1.079883385781
-0.840961684155	-1.079581478176
-0.840862304937	-1.079544127096
-0.84073279583	-1.079514820938
-0.840639341206	-1.07899957891
-0.840538701708	-1.078510862816
-0.840426962557	-1.078366580246
-0.840321189362	-1.078212554484
-0.840218555937	-1.078072759656
-0.84011988672	-1.077987863107
-0.840010875603	-1.077947954218
-0.839901801017	-1.077836809346
-0.839778692801	-1.077786987453
-0.839671329266	-1.07777547348
-0.839556946722	-1.077698637086
-0.839453089373	-1.077529390771
-0.83936006068	-1.077451644995
-0.839240600273	-1.077445587677
-0.839144204423	-1.077184577391
-0.839041053895	-1.077159298986
-0.838926152269	-1.077026670773
-0.838813420548	-1.077007906506
-0.838719991786	-1.07700701402
-0.838617848775	-1.076855842944
-0.838507444043	-1.076646770033
-0.838415137456	-1.07651102215
-0.838297216555	-1.076413859102

-0.838177934886	-1.076312009806
-0.838072735405	-1.076290369332
-0.837966420938	-1.076237359808
-0.837877992767	-1.07605599705
-0.837778919499	-1.075921903521
-0.837665933108	-1.075711412289
-0.837560558028	-1.075707168529
-0.837461207732	-1.075604981068
-0.837367391691	-1.075396656256
-0.837263449319	-1.075348143886
-0.837129770439	-1.075229748036
-0.837012692462	-1.075154779911
-0.836885421752	-1.075062621803
-0.836771660672	-1.074957406895
-0.836660077673	-1.074903687842
-0.836556447806	-1.074812026245
-0.836464540864	-1.074681600669
-0.836381197377	-1.074455427051
-0.836262193437	-1.074417754637
-0.836177961013	-1.074302698974
-0.836067196041	-1.073854309155
-0.835922951109	-1.073833664382
-0.835804830094	-1.073705611545
-0.835705371572	-1.073445199554
-0.835599437174	-1.073387404311
-0.835467991798	-1.073367899079
-0.835369330398	-1.073101759088
-0.835267509275	-1.073097707287
-0.835166681649	-1.072704750319
-0.835067438921	-1.072694425862
-0.834948774526	-1.072643388571
-0.834836482022	-1.07256414266
-0.834730768588	-1.072314402595
-0.83461922173	-1.07124313155
-0.834484047815	-1.070889164376
-0.834369397676	-1.070885760181
-0.834274356941	-1.070714862466
-0.834169850575	-1.070644777338
-0.834057943996	-1.070474379375
-0.833963841673	-1.070402642275
-0.833865470449	-1.070172959608
-0.833766091977	-1.069851678884
-0.833644952783	-1.069647039401
-0.833540283755	-1.069574766657
-0.83343918225	-1.069494933673
-0.833339906251	-1.069457885933
-0.833233830578	-1.06902759061
-0.833132010679	-1.068961982314
-0.833030228647	-1.068640806663
-0.832898466057	-1.0685236491
-0.83279889176	-1.068415257561
-0.832697620834	-1.067849711919
-0.832604872717	-1.067846933965
-0.832477074673	-1.067743043171

-0.832365418967	-1.067706541869
-0.832268824315	-1.067630018323
-0.832158853662	-1.067519956081
-0.832063948336	-1.067447546121
-0.83193140617	-1.067321771064
-0.831842070845	-1.067269910538
-0.831731101665	-1.067211455628
-0.831647643466	-1.066780166779
-0.831557456031	-1.066768691348
-0.831461340549	-1.066755929566
-0.831351283116	-1.066513190656
-0.831267097914	-1.066481827219
-0.83114741444	-1.066393609209
-0.831044937927	-1.066324448539
-0.830948062734	-1.06625960323
-0.830837239009	-1.066222511093
-0.83074987631	-1.065978079066
-0.830635580992	-1.065921354949
-0.830536508501	-1.065814542288
-0.830444210326	-1.065742877259
-0.830346591553	-1.065642269146
-0.830220649844	-1.065587597901
-0.830124231565	-1.065446476157
-0.829997575363	-1.065097037376
-0.829882482576	-1.064931377351
-0.829774377541	-1.064897228034
-0.829667230133	-1.064867728506
-0.829551672783	-1.064759407588
-0.829455515475	-1.064506280246
-0.829358077742	-1.06437602453
-0.829264407815	-1.064190421077
-0.829164429807	-1.064161529284
-0.829069080885	-1.064151270754
-0.82898055859	-1.063627364003
-0.828863133157	-1.063567605295
-0.828754120115	-1.06342290321
-0.828647243761	-1.06322324668
-0.828549191844	-1.063211796495
-0.828436681234	-1.06320433699
-0.828324283731	-1.063131049072
-0.828218936557	-1.062791410207
-0.828100249924	-1.062750631453
-0.828010772525	-1.062739165826
-0.827903183337	-1.06263295005
-0.827795740229	-1.062562937541
-0.827684994977	-1.062330343065
-0.827579241361	-1.062110559052
-0.827484043497	-1.061930438301
-0.827365325286	-1.061891390576
-0.82727303577	-1.061666403579
-0.827189589511	-1.061512844611
-0.827087394367	-1.061337477506
-0.826980984585	-1.061336705319
-0.82685895506	-1.061274988634

-0.826759811528	-1.061239054975
-0.826635691697	-1.061190680359
-0.82655800126	-1.061170822984
-0.826451379955	-1.061163538262
-0.826349227945	-1.061090410512
-0.826232913741	-1.061074029838
-0.826106470197	-1.060974638422
-0.825993635419	-1.060942156611
-0.825892971534	-1.060936570487
-0.825771941893	-1.060745948811
-0.825661371568	-1.060365521412
-0.825552060851	-1.060271594613
-0.825437318746	-1.060235401666
-0.825335930321	-1.059685515462
-0.825218708261	-1.059663918734
-0.825122895676	-1.059284976171
-0.825005096608	-1.059276954197
-0.824903054521	-1.059061222502
-0.824793991482	-1.05899806835
-0.824683696361	-1.05884245576
-0.824587905391	-1.058292633754
-0.824484511755	-1.05810684742
-0.824359550604	-1.05806318571
-0.824260157779	-1.057949006689
-0.82416288153	-1.057820883605
-0.824063879107	-1.057696043805
-0.823954344955	-1.057639579153
-0.823836886192	-1.057365447485
-0.823714877122	-1.057136112963
-0.823618002091	-1.056909996064
-0.82351717371	-1.05690418466
-0.823416841075	-1.056848504483
-0.823319524859	-1.056785206034
-0.823213809451	-1.056487091566
-0.823084191789	-1.056381528907
-0.822968091427	-1.056073027256
-0.822887387206	-1.055810136373
-0.822773378329	-1.055508015098
-0.822666269994	-1.055429219951
-0.822564043773	-1.054973323488
-0.822461974482	-1.054815945826
-0.822351786999	-1.054789819078
-0.822258374993	-1.054743360882
-0.822175253327	-1.054704783055
-0.822061914137	-1.054250369393
-0.82195508775	-1.054150280135
-0.821838569479	-1.053905853155
-0.821733861612	-1.053657902419
-0.82161361992	-1.053418722524
-0.821502279916	-1.053234837862
-0.821404650969	-1.053199435964
-0.821313581937	-1.053091578998
-0.821199179669	-1.052993879747
-0.821084793713	-1.052899173897

-0.820966624014	-1.052846690155
-0.820871585227	-1.052815790478
-0.820759910793	-1.052799857408
-0.820656634411	-1.052455002832
-0.820561966323	-1.05236380749
-0.820441289341	-1.052312551921
-0.820343532328	-1.052153284937
-0.8202309509	-1.052069722185
-0.820143789947	-1.051403539705
-0.820017371022	-1.051374958457
-0.819911625262	-1.051310198174
-0.819806581856	-1.051298790619
-0.81968685537	-1.051288423465
-0.819582839226	-1.051191343477
-0.819478536336	-1.051114041366
-0.819365065879	-1.051101997776
-0.819282528186	-1.050822145973
-0.819167305604	-1.050770934806
-0.819066051041	-1.050528591212
-0.818950294373	-1.050500716309
-0.818843948421	-1.050481066865
-0.818735593203	-1.050336820913
-0.818637097804	-1.05031665639
-0.818529990267	-1.050305241877
-0.818439914884	-1.050290724804
-0.8183459711	-1.050133493142
-0.818232020968	-1.049696466827
-0.818154226379	-1.049609650862
-0.818030539398	-1.049427192681
-0.817907403633	-1.049084904138
-0.817780577644	-1.049009392516
-0.817691050161	-1.048970722356
-0.817574889806	-1.048923500832
-0.817470039093	-1.048833628632
-0.817365599641	-1.048734569529
-0.817244934082	-1.048689788037
-0.817115434549	-1.048558889476
-0.817018466754	-1.048403510186
-0.8169154849	-1.048377387642
-0.816797831213	-1.0481174318
-0.816688657288	-1.048057615197
-0.81659255699	-1.047980408211
-0.816472443955	-1.047925344259
-0.81635972982	-1.047629759332
-0.816266488965	-1.047621315038
-0.816153955256	-1.047385982096
-0.81605178569	-1.047319495141
-0.81592203603	-1.047189910194
-0.815808620247	-1.047035584802
-0.815709927685	-1.046846906802
-0.815604937249	-1.046725416334
-0.815468695027	-1.046656039692
-0.815358873916	-1.04663391982
-0.81525023691	-1.046627488544

-0.815138030627	-1.046585177589
-0.815030953235	-1.046517347285
-0.814928061477	-1.046499470619
-0.814817171594	-1.046398042526
-0.814726527723	-1.046366520489
-0.814604833638	-1.046353410419
-0.814498712834	-1.04628704752
-0.814389854794	-1.046069557252
-0.814275453943	-1.046068597814
-0.814153130668	-1.045759290288
-0.81406003706	-1.045447304298
-0.813958884439	-1.045362271624
-0.813864258566	-1.045272383942
-0.813759177679	-1.044922080473
-0.813670811849	-1.044802656608
-0.813565384561	-1.044675600341
-0.813456829853	-1.044637199069
-0.813359322439	-1.044605430973
-0.813233836517	-1.044520651572
-0.813135980826	-1.044452238747
-0.813031756086	-1.044281430341
-0.812917531945	-1.044245250053
-0.812802245779	-1.044228297286
-0.812685745062	-1.04415769628
-0.812577540609	-1.043958471046
-0.812460968504	-1.043954189399
-0.812340689338	-1.04393637813
-0.81222510029	-1.043861161616
-0.812118481288	-1.043777731448
-0.81197880985	-1.043643939049
-0.811882051382	-1.043529786058
-0.811776924752	-1.043335159928
-0.811690278105	-1.043153083061
-0.811572179136	-1.042952516519
-0.811468117748	-1.042725140083
-0.811362458107	-1.04270646284
-0.811277065187	-1.04256473592
-0.811164591197	-1.042560487423
-0.811059391417	-1.042484690795
-0.810960856477	-1.042082355826
-0.810853948752	-1.042019521095
-0.810728665314	-1.042010802993
-0.810634245298	-1.041967988915
-0.810499247491	-1.041917442404
-0.810389031171	-1.041896852502
-0.810300395825	-1.041860772838
-0.810199442426	-1.041549156808
-0.810092534967	-1.041341591384
-0.809983511809	-1.041330727684
-0.809889500098	-1.04127602344
-0.809783783385	-1.041203054371
-0.809684240721	-1.041184775332
-0.809599671823	-1.041046306628
-0.809470842865	-1.041037029766

-0.809380482082	-1.041015858356
-0.809257523431	-1.040851908376
-0.809147756997	-1.040698093452
-0.809051274875	-1.040589195135
-0.808944823346	-1.040474308405
-0.808834802106	-1.040407429356
-0.808740099388	-1.040317030635
-0.808632377791	-1.040314224182
-0.808522574511	-1.040132460916
-0.808401158454	-1.040038427027
-0.808293644825	-1.03993362991
-0.80817253108	-1.039743736332
-0.808047104656	-1.039526989148
-0.807952011165	-1.039436433924
-0.807850998455	-1.039315408657
-0.807724204076	-1.038971546041
-0.807640897069	-1.038806827575
-0.807526981288	-1.038799525168
-0.807419249799	-1.038690507658
-0.807288160229	-1.038633688288
-0.807195994719	-1.038514664322
-0.807094070518	-1.038508301483
-0.806987412803	-1.038204311068
-0.806893980367	-1.038057572371
-0.806779200189	-1.038026231125
-0.806683600255	-1.038000014238
-0.806565495576	-1.037977370188
-0.806475091949	-1.037858732456
-0.806384435998	-1.037715338181
-0.806295518685	-1.037374576838
-0.806180469064	-1.03737148939
-0.806070894134	-1.037072422344
-0.805956382673	-1.037052742444
-0.805835573549	-1.03698011676
-0.805721804228	-1.036835578519
-0.805638003383	-1.03665566372
-0.805510076104	-1.036063809364
-0.805415932099	-1.035977775894
-0.805287870948	-1.035918242917
-0.805180807236	-1.035807159079
-0.805055031354	-1.035659223907
-0.804928062549	-1.035582998696
-0.804822356506	-1.035559669507
-0.80472058229	-1.035514751312
-0.804617309102	-1.0353207493
-0.804511795785	-1.035277191005
-0.804403959603	-1.035260598533
-0.804300924325	-1.035185514648
-0.804198279916	-1.035085661336
-0.804100962825	-1.034981255975
-0.804000748083	-1.034928942502
-0.803893360088	-1.03491950074
-0.803760302463	-1.034846375736
-0.803677589043	-1.034833862887

-0.803576784599	-1.034768956064
-0.80348544687	-1.034713866079
-0.803395445324	-1.03467513574
-0.80329016835	-1.034636692761
-0.803163653845	-1.034587988278
-0.803033177034	-1.034582379819
-0.802904839904	-1.034498063669
-0.802813386071	-1.034315466471
-0.802692963127	-1.034273246097
-0.802583889733	-1.034205722705
-0.802506301292	-1.034078578949
-0.80240489265	-1.034066313288
-0.802306895414	-1.033998941782
-0.80220376158	-1.033805087152
-0.802099178927	-1.033750374575
-0.801981232178	-1.033440345873
-0.801868121428	-1.033395897755
-0.801767798998	-1.033279161298
-0.801657950744	-1.033171525701
-0.801545378031	-1.033130645961
-0.801422173057	-1.033104432036
-0.801319279246	-1.032748887044
-0.801221181948	-1.03267390848
-0.801093080388	-1.032496204379
-0.800997531873	-1.032428426223
-0.800875609675	-1.032421567917
-0.800754222471	-1.032361123291
-0.800650226766	-1.032295367742
-0.800553788501	-1.03229521148
-0.800415982112	-1.032194926404
-0.800319252162	-1.03213340927
-0.800206386919	-1.032075856191
-0.800108713282	-1.032026342913
-0.79999397805	-1.031960257553
-0.799881927011	-1.031853328562
-0.799761921512	-1.031414305185
-0.799659602689	-1.031234749137
-0.799552456875	-1.031093401324
-0.799458134411	-1.030913124975
-0.799351351564	-1.030529234045
-0.799251301295	-1.030349345382
-0.799153839028	-1.030076808358
-0.79904072597	-1.030055150647
-0.798958268823	-1.030011018802
-0.798861946553	-1.029935511764
-0.798729965382	-1.0298565585
-0.79861286811	-1.029765670597
-0.798512950647	-1.029619786339
-0.798402069905	-1.029574343314
-0.798279301617	-1.029565308119
-0.798180287507	-1.029443320466
-0.798080376907	-1.029396329996
-0.797971701974	-1.029300053579
-0.797857443508	-1.029163891017

-0.797746364256	-1.02908684118
-0.797637578433	-1.029042101769
-0.797528079692	-1.028844373011
-0.797420552643	-1.028725647378
-0.797335916076	-1.028722387005
-0.797251351094	-1.028701121532
-0.797138894709	-1.028589827707
-0.797036471386	-1.028577855236
-0.796937448176	-1.028569367582
-0.79683948526	-1.028552870853
-0.796736088769	-1.028524173873
-0.796650298996	-1.028361889294
-0.796531460535	-1.028311752974
-0.796415957346	-1.02806079258
-0.796317942371	-1.028005978061
-0.796222213371	-1.028000124829
-0.796115596527	-1.027918946951
-0.796020691677	-1.027368331851
-0.795912373718	-1.027357223058
-0.79580217317	-1.027351722087
-0.795696222044	-1.027295093185
-0.795598227103	-1.027253513514
-0.795497228639	-1.027226549234
-0.795390610822	-1.027185790194
-0.795301932683	-1.027148948049
-0.795212304764	-1.027066535669
-0.795078580494	-1.02702256858
-0.794976326172	-1.026832471558
-0.794887992669	-1.02679112278
-0.79477857616	-1.026786132448
-0.794685504906	-1.026529478521
-0.794572624151	-1.02638914332
-0.794475428225	-1.026388864217
-0.794353288341	-1.026175622901
-0.794269282809	-1.026022852194
-0.794138928508	-1.025947511715
-0.79402760775	-1.025466409556
-0.793913758685	-1.025451392671
-0.79381618156	-1.024860719861
-0.793711481785	-1.02477116265
-0.793613353143	-1.024705406696
-0.793505825349	-1.024702285975
-0.793411467451	-1.024497625536
-0.793297754017	-1.024228454014
-0.793187608458	-1.024093863789
-0.793093702568	-1.023972077641
-0.792999313426	-1.023784109239
-0.792881111919	-1.023679751108
-0.792774214283	-1.023470413624
-0.792673167761	-1.023318653522
-0.792572074238	-1.023274119829
-0.792470658824	-1.023097236094
-0.792363705891	-1.023013535821
-0.792259582382	-1.022959086164

-0.79215283106	-1.022852413814
-0.792045371846	-1.022823824117
-0.791933245578	-1.022748614781
-0.791823607886	-1.022519598874
-0.791711358848	-1.022455292674
-0.791602066372	-1.022277312057
-0.791519009324	-1.022264985847
-0.791416979195	-1.021836323075
-0.791305703972	-1.021723855659
-0.791194442859	-1.021606405841
-0.79107274842	-1.021574441302
-0.791004429034	-1.02156737547
-0.790898638653	-1.021532905049
-0.790797961147	-1.021497612252
-0.790688484446	-1.021230919279
-0.790566142315	-1.021150015666
-0.790476578481	-1.021134674389
-0.790395258365	-1.021016043713
-0.790303379939	-1.020921611264
-0.790199811857	-1.020537451804
-0.790090833927	-1.020493240869
-0.789975096055	-1.019898897419
-0.789877741466	-1.01985806606
-0.789765457502	-1.019662803662
-0.789660788508	-1.019632199139
-0.789576197516	-1.019585344032
-0.789461864045	-1.019534772459
-0.789351259426	-1.019359840652
-0.789244994495	-1.019333674703
-0.789142569152	-1.018896986735
-0.789036119588	-1.018886478515
-0.788917916702	-1.018589813647
-0.788811290582	-1.018508013198
-0.788725048827	-1.018452474217
-0.788614447472	-1.018424595827
-0.788503036229	-1.018384420973
-0.788398189996	-1.018382559241
-0.78828900014	-1.018309660525
-0.78819553577	-1.018150440098
-0.788097588747	-1.017943463512
-0.787989828128	-1.017918633993
-0.787898494479	-1.017842630529
-0.787784948073	-1.017722544582
-0.787677900291	-1.017661833497
-0.787594406789	-1.017657099945
-0.787471957914	-1.017557951832
-0.787388933794	-1.017412931321
-0.787281317015	-1.017247782331
-0.787179250834	-1.017105740349
-0.787081631517	-1.016964523883
-0.786971792697	-1.016794337647
-0.786868376718	-1.016630698629
-0.786767719875	-1.016598140744
-0.786658174728	-1.016560875909

-0.786560508711	-1.016459898014
-0.786458865915	-1.016457266462
-0.786339260844	-1.01613922701
-0.786233008762	-1.015578055767
-0.78612267605	-1.015473093952
-0.786015559596	-1.015326642196
-0.785931244104	-1.015261678712
-0.78582740876	-1.01511646627
-0.78570003595	-1.014751994365
-0.785590251338	-1.014521692003
-0.78546349985	-1.014371839984
-0.785337579858	-1.014291077636
-0.785224213274	-1.01409785388
-0.785119929456	-1.014001652043
-0.785026924331	-1.013870661217
-0.784901706422	-1.013822906507
-0.784794128129	-1.013398839606
-0.784689462753	-1.013377443686
-0.784572384106	-1.013095936718
-0.784489744949	-1.012923716749
-0.784390545076	-1.012570240546
-0.784289802801	-1.012471811215
-0.784172599129	-1.012137335374
-0.784076452025	-1.012084090198
-0.783975683951	-1.011564277848
-0.783874193401	-1.011410974979
-0.783778175123	-1.011149736738
-0.78365907067	-1.01097811364
-0.783555608734	-1.010899192661
-0.78343892312	-1.010797828133
-0.783318243714	-1.010608324476
-0.783229571958	-1.010461783363
-0.783135416208	-1.010365320268
-0.783032508263	-1.010321715934
-0.782942313994	-1.010242795018
-0.782833533692	-1.010178744856
-0.782731839968	-1.010041458845
-0.782605004307	-1.010030232989
-0.78250126704	-1.010017293799
-0.782412099225	-1.009982399782
-0.782309038116	-1.009948446895
-0.782209639827	-1.009858224205
-0.782107309309	-1.009543644227
-0.781994470203	-1.009432961104
-0.781885662675	-1.009391895262
-0.781784056964	-1.009181838856
-0.78166891086	-1.009140459008
-0.781571299644	-1.008884503394
-0.781468307887	-1.008851953826
-0.781369154619	-1.008638841965
-0.781275116392	-1.008539921586
-0.781172669911	-1.008209257471
-0.78108981752	-1.00819558368
-0.780989727669	-1.008163685213

-0.78088717091	-1.008069568682
-0.780783974752	-1.007999197366
-0.780681578899	-1.007834956896
-0.780564620382	-1.00768947641
-0.780462895195	-1.007678167056
-0.78035544404	-1.007622244349
-0.780249469838	-1.007457477523
-0.780135574183	-1.007318682358
-0.780033740356	-1.007280633224
-0.779946910468	-1.007117511622
-0.779835329331	-1.007044095543
-0.77972884766	-1.006975046303
-0.779634505121	-1.006829364398
-0.779526985005	-1.006789736779
-0.779415552839	-1.006780563506
-0.779295494037	-1.006742490771
-0.779182822455	-1.006727062209
-0.779073626833	-1.006694656398
-0.778959486302	-1.006536765239
-0.77884261737	-1.006508421697
-0.778723658052	-1.006429323462
-0.778615222319	-1.006399386453
-0.778497355143	-1.006355710337
-0.778397336429	-1.006300258335
-0.778307587455	-1.006278779488
-0.778208777888	-1.006272523153
-0.778096710176	-1.006113485126
-0.778010760006	-1.006035405249
-0.777905026906	-1.005887743166
-0.77781730029	-1.00581885101
-0.777725441157	-1.005686345294
-0.777622774786	-1.00564149279
-0.777524375239	-1.005553338863
-0.777398952977	-1.005124489224
-0.777300565916	-1.004899126803
-0.777191478924	-1.004884124811
-0.777082344064	-1.004881285223
-0.776987020319	-1.004862838557
-0.776883793962	-1.00475807846
-0.776773687364	-1.004697665492
-0.776655153748	-1.004532157146
-0.776543790977	-1.004464963135
-0.776442710142	-1.004345858598
-0.776337540306	-1.004318798383
-0.776230553148	-1.004257370031
-0.776136558066	-1.003976479202
-0.776031384703	-1.003591444978
-0.775944707581	-1.003563253935
-0.775855126316	-1.003481521687
-0.775763692033	-1.003421977459
-0.775657034504	-1.003383348195
-0.775561743446	-1.00327047329
-0.775460419478	-1.003197280706
-0.775349255883	-1.003077485614

-0.775253167276	-1.002823030337
-0.775145144149	-1.00281537246
-0.775020975924	-1.002787285234
-0.774911808872	-1.002770792725
-0.774819338309	-1.002555020499
-0.774746211777	-1.002406526858
-0.774637091959	-1.002305185888
-0.774537516259	-1.002137869058
-0.774442507512	-1.001619945774
-0.774348898704	-1.001607787917
-0.7742440973	-1.001574369879
-0.774140312225	-1.001511920591
-0.774047231696	-1.001442556818
-0.77393206497	-1.001390879482
-0.773825547356	-1.001343614273
-0.773723330112	-1.001265491444
-0.773599908301	-1.00111869697
-0.77347419139	-1.000944189462
-0.773361672096	-1.000811214746
-0.773256396598	-1.000690367083
-0.773140479063	-1.000381094148
-0.773036919352	-1.000055379215
-0.772944935576	-0.999934049637
-0.77284812677	-0.999786199155
-0.772736844689	-0.999619862744
-0.772630761702	-0.999599677584
-0.772542498741	-0.999449455473
-0.772416665862	-0.999408430071
-0.772317915903	-0.999148639284
-0.77222944672	-0.99872977492
-0.772117714594	-0.998577132357
-0.772028788446	-0.998329897886
-0.771918604799	-0.998066676172
-0.771837430061	-0.997921529811
-0.771748942584	-0.997907952135
-0.771652131005	-0.997592331202
-0.771548327734	-0.997491628294
-0.771444311508	-0.99733940413
-0.77132427521	-0.997317756384
-0.771220760872	-0.997256621602
-0.771130831614	-0.997206544464
-0.771031547528	-0.997195085275
-0.77091886373	-0.997015497357
-0.770798278046	-0.996997916703
-0.770687996379	-0.996916816304
-0.770584341126	-0.996745017195
-0.770477297162	-0.996529476125
-0.770349341515	-0.996472688458
-0.770235618933	-0.996347476187
-0.77010022361	-0.996131159153
-0.76999220374	-0.996078748824
-0.76986393707	-0.996023875842
-0.769759005383	-0.995635368642
-0.769654244332	-0.995460154061

-0.769558018774	-0.995442767
-0.769446128037	-0.995419649952
-0.769335250337	-0.995406741685
-0.769250242751	-0.995385478921
-0.76914065584	-0.995245240988
-0.769051237617	-0.995238745268
-0.768954666287	-0.995177066695
-0.768842585674	-0.995105376343
-0.768749107824	-0.994934517272
-0.76865336704	-0.994922185668
-0.768551459042	-0.994578471029
-0.768472757347	-0.99453007202
-0.768371710716	-0.994518315093
-0.768267495916	-0.994498527076
-0.768169344574	-0.994387107607
-0.768059855329	-0.994272171919
-0.767933892836	-0.994253920285
-0.76781202041	-0.994200552662
-0.767710514028	-0.994148585866
-0.767608183825	-0.993985416562
-0.767505705381	-0.993910409694
-0.767408664382	-0.993779169606
-0.767303405798	-0.993746086961
-0.767186643161	-0.993681012298
-0.767082566911	-0.992846887315
-0.76696492946	-0.992782928009
-0.766867214533	-0.992758874042
-0.766747566137	-0.992727089251
-0.766651949278	-0.992549345242
-0.76653924332	-0.992428084793
-0.766439970035	-0.992363138084
-0.76632993509	-0.992265745078
-0.766234227565	-0.992034216367
-0.766132811267	-0.992007663185
-0.766010414893	-0.991555408214
-0.765911450821	-0.991510794294
-0.765785039544	-0.991152291903
-0.76569378001	-0.991128048986
-0.765572056814	-0.991038757944
-0.76545694732	-0.990999376533
-0.765365695829	-0.990958525994
-0.765258624197	-0.990938685377
-0.765171938495	-0.990920712037
-0.765078257113	-0.990882742432
-0.764959308025	-0.990851531917
-0.764868871159	-0.990539034882
-0.764764097043	-0.990508271097
-0.764669528858	-0.990375415365
-0.764569143478	-0.990303470728
-0.764462956745	-0.990261537479
-0.764328157827	-0.990245635336
-0.764222008276	-0.990123570544
-0.764101384769	-0.98993348485
-0.764005062778	-0.989918384614

-0.76388825217	-0.989676964595
-0.763762727781	-0.989609084791
-0.763671254341	-0.989515924652
-0.763576061898	-0.989501484205
-0.763497363601	-0.989274137414
-0.763393361285	-0.989246605678
-0.763282069864	-0.98923582938
-0.763168740627	-0.989060709143
-0.7630666932	-0.988995174972
-0.762940916285	-0.988476862978
-0.762838234402	-0.988405147716
-0.762738143412	-0.988374297163
-0.762647853723	-0.988245683382
-0.762557985031	-0.98818280028
-0.762452676862	-0.988137651848
-0.762351771035	-0.988094657329
-0.762255188579	-0.987849766377
-0.762157613104	-0.987762094266
-0.762048818124	-0.987706843355
-0.761948425005	-0.987612582772
-0.76183607293	-0.987518464767
-0.761721258799	-0.987417777142
-0.76163782381	-0.987294590101
-0.761544768478	-0.987134555449
-0.761438570896	-0.987125155557
-0.761336496308	-0.987059474284
-0.761256812407	-0.986904649945
-0.761131719741	-0.98683892706
-0.761006389523	-0.986634091056
-0.760905249252	-0.98654931895
-0.760768504382	-0.986543298822
-0.760657185656	-0.98643872625
-0.760547316583	-0.986393544157
-0.760444387212	-0.986154601721
-0.760344310556	-0.986015778122
-0.760241395592	-0.985793907386
-0.760158189696	-0.985473246194
-0.760057910486	-0.985435466332
-0.759958331528	-0.985317375931
-0.759866544595	-0.985049018817
-0.759772338463	-0.985043364123
-0.759671990126	-0.984988551282
-0.759556096228	-0.984869136116
-0.759447191207	-0.98472190812
-0.759347318839	-0.984675206286
-0.759255627367	-0.984674881729
-0.759154134539	-0.984616957594
-0.759062110341	-0.984536266482
-0.758963436884	-0.984518235188
-0.758871213445	-0.984512352829
-0.758779502007	-0.984170672873
-0.758675824886	-0.984124159985
-0.758587046284	-0.983934088938
-0.758456732881	-0.983901339077

-0.758353408303	-0.983796503309
-0.758235431943	-0.983545246555
-0.758115168644	-0.983264731585
-0.758012072828	-0.983192674741
-0.757894339906	-0.9831609444
-0.757792467292	-0.982955384175
-0.757677819269	-0.982954050908
-0.757573448181	-0.982926176708
-0.757479669362	-0.982871760486
-0.757379433779	-0.982844047352
-0.757299680293	-0.982462042747
-0.757213400567	-0.982330833307
-0.757069849688	-0.982249632947
-0.756960794358	-0.982225041945
-0.756859092903	-0.982191809376
-0.756754066947	-0.982164466742
-0.756666228708	-0.982079404043
-0.756573235224	-0.982029710718
-0.756480820128	-0.98190508828
-0.756364162983	-0.981856034303
-0.756273902969	-0.981774647148
-0.756164927737	-0.981543573345
-0.756057444446	-0.981460840823
-0.755942756479	-0.981116738319
-0.755833978008	-0.981085239069
-0.755746611881	-0.98106642101
-0.75563353103	-0.980988769881
-0.75553580033	-0.980970088876
-0.755436005699	-0.980950365847
-0.755323984229	-0.980881620987
-0.755211909221	-0.980606688498
-0.75511783713	-0.980549257857
-0.755027389423	-0.980512216358
-0.754911037422	-0.980428909192
-0.754818179699	-0.980170467242
-0.754720934872	-0.980132909874
-0.754624320802	-0.979984532816
-0.7545111605	-0.979947630608
-0.754408725136	-0.979787440195
-0.754335095856	-0.979551473503
-0.754218646149	-0.97943832664
-0.754090591032	-0.979199964828
-0.753979304241	-0.979080292559
-0.753889992522	-0.979033249899
-0.753771001538	-0.978852729446
-0.75365879103	-0.978774181342
-0.753530166435	-0.978750881975
-0.753423584285	-0.978653954965
-0.753319268132	-0.978556000285
-0.753218318194	-0.978505276952
-0.753111099172	-0.978306262557
-0.753009873027	-0.978146860042
-0.752897766493	-0.977573826935
-0.752798914631	-0.977513740495

-0.752709295548	-0.977444919368
-0.752598469058	-0.97703081374
-0.752496742709	-0.976846737997
-0.752384400606	-0.976741497562
-0.752277788486	-0.976704170637
-0.752183865819	-0.976396628569
-0.752066382264	-0.976317196066
-0.751980380013	-0.976272666869
-0.75188569607	-0.975939604634
-0.751754048965	-0.975932242523
-0.751656134681	-0.975920872157
-0.751564652004	-0.975850538074
-0.751463980062	-0.975323301824
-0.751370202366	-0.975202589361
-0.751263174917	-0.975101009692
-0.751162950289	-0.974897584696
-0.751064747781	-0.974822987953
-0.750961492463	-0.974586679474
-0.750871092155	-0.974550028389
-0.750766076222	-0.974530898351
-0.750667964949	-0.97446090362
-0.750576127299	-0.974452770063
-0.75048349103	-0.974224606689
-0.750389585801	-0.974165770912
-0.750282781057	-0.974133270589
-0.750189308809	-0.974121137464
-0.750098488135	-0.973981895264
-0.750006677199	-0.973977692461
-0.749900295134	-0.973940096406
-0.749803481361	-0.973875997727
-0.749701699122	-0.973703189659
-0.749605812115	-0.973633947468
-0.749502047889	-0.9736211453
-0.749402785734	-0.973488466521
-0.749287978896	-0.973294155528
-0.74918831338	-0.973214240462
-0.749083843261	-0.973166128789
-0.748977052583	-0.972945435105
-0.748900640164	-0.972656036341
-0.748794262464	-0.972348621085
-0.748702271333	-0.972267716539
-0.748616789771	-0.972174352718
-0.748491123554	-0.972136929934
-0.748397282415	-0.972058130365
-0.748284995898	-0.972045593718
-0.748187293297	-0.971929537079
-0.748086312805	-0.971805415443
-0.747989646436	-0.971715090669
-0.74789337213	-0.971605140013
-0.747790340435	-0.971402741206
-0.747682070777	-0.971387915041
-0.747566366177	-0.971318462409
-0.747454361549	-0.971165915103
-0.747367914503	-0.971107155634

-0.747267029203	-0.970839628961
-0.747154971847	-0.970651404752
-0.747027499369	-0.97064971778
-0.746912693277	-0.970486045148
-0.746815912087	-0.970478969435
-0.746694686732	-0.970434051094
-0.746592914485	-0.970400447122
-0.746484079334	-0.970388567747
-0.746375546773	-0.970369852095
-0.746268649015	-0.970319706544
-0.746186619305	-0.970233491154
-0.746060640418	-0.970199979178
-0.745973734745	-0.970178307077
-0.745888612212	-0.97009314665
-0.745768323791	-0.970080041285
-0.745683871121	-0.969948631284
-0.745586311929	-0.969915121847
-0.745480575254	-0.969844384695
-0.745372585659	-0.969813474914
-0.745283044728	-0.969786384278
-0.745175263867	-0.969657589651
-0.745068438843	-0.969530445134
-0.74497247686	-0.969528929189
-0.744872188671	-0.969435988199
-0.744786381632	-0.96941697066
-0.744685375403	-0.969370446815
-0.744569573907	-0.969305332828
-0.74445196218	-0.969179517624
-0.744337186172	-0.969164148495
-0.744231426485	-0.968906431235
-0.74409997038	-0.968863539351
-0.743999282522	-0.968598376209
-0.743898299295	-0.968272056428
-0.743801446156	-0.968200578805
-0.743696577685	-0.968114170266
-0.74360229807	-0.968039642052
-0.743510395509	-0.967906069841
-0.74342389787	-0.967521495843
-0.743318248426	-0.96747959722
-0.743227716643	-0.967110597831
-0.743127734635	-0.967066310928
-0.74304126607	-0.966972339848
-0.74295101406	-0.966718482434
-0.742843484405	-0.966605958043
-0.742763409595	-0.966572587596
-0.742669419323	-0.966431255644
-0.742566374385	-0.96642081661
-0.742461318529	-0.966343384386
-0.742381847951	-0.966115182175
-0.742268392329	-0.966026074792
-0.742165421133	-0.965924577333
-0.742056310911	-0.965728492091
-0.741971825209	-0.965701791655
-0.741871035087	-0.965324428969

-0.741758414729	-0.965304711219
-0.741678475686	-0.965022109567
-0.741587511386	-0.965011656319
-0.741484576335	-0.964905306433
-0.741386585419	-0.964848630504
-0.74129604914	-0.964687402616
-0.741185407829	-0.964651724309
-0.741079520974	-0.964560415664
-0.740963738962	-0.964526622924
-0.740870145294	-0.964495058663
-0.740772595304	-0.964463389133
-0.740665588392	-0.964311878808
-0.74055283489	-0.964282238233
-0.740456489512	-0.964248766656
-0.740354325708	-0.96422725846
-0.740266149032	-0.964152862236
-0.740168057863	-0.964104186971
-0.74006691512	-0.964014747304
-0.739973030102	-0.963737073007
-0.739880028725	-0.963435081844
-0.739778924633	-0.963426475726
-0.739667535867	-0.963295895835
-0.739572979796	-0.963114499615
-0.739493936601	-0.963000937393
-0.739373468704	-0.962953825256
-0.739272992729	-0.962882315545
-0.739167891586	-0.962795671444
-0.739063021019	-0.962717952411
-0.738962718247	-0.962684793685
-0.738863626747	-0.962652086758
-0.738763552726	-0.962599748256
-0.738649978573	-0.962502486255
-0.738549487504	-0.962472128237
-0.738463958576	-0.962462922873
-0.738363533823	-0.962454428059
-0.738280436665	-0.962370896431
-0.738185991418	-0.962320584204
-0.738096729312	-0.962293354534
-0.737982420257	-0.962250980835
-0.737879849929	-0.962200371284
-0.737780512043	-0.962152464863
-0.737673046035	-0.962139762112
-0.737573609053	-0.962100771121
-0.737450145149	-0.962099884365
-0.737330418843	-0.962072102319
-0.737220462056	-0.961910333152
-0.737108636101	-0.961803777309
-0.737014538113	-0.961766521696
-0.736917260933	-0.961708229954
-0.736815204509	-0.961569431103
-0.736694366404	-0.961541290972
-0.736571869694	-0.961157308
-0.736468607104	-0.961135640161
-0.736373096451	-0.961135561995

-0.736284377861	-0.961053375541
-0.736184886944	-0.960836372604
-0.736090930816	-0.960700248617
-0.735992106719	-0.960665744489
-0.73590947227	-0.960585748005
-0.735803744147	-0.960575073664
-0.735711613684	-0.960528540643
-0.73559969843	-0.960411807403
-0.735500768519	-0.960057454973
-0.735408216101	-0.960046631706
-0.735287726606	-0.959703785191
-0.735171809036	-0.959624241493
-0.735064297637	-0.959413168551
-0.734947466736	-0.959193223237
-0.734855676926	-0.959027514201
-0.73474682616	-0.958954713324
-0.734637240442	-0.958753556784
-0.73454930875	-0.95872397933
-0.734443411341	-0.958660159019
-0.734356483389	-0.958644668857
-0.734265107709	-0.958523636029
-0.73415261863	-0.958396008751
-0.734030997907	-0.95824287702
-0.733940444816	-0.957948678557
-0.733848720727	-0.957904357612
-0.733724478054	-0.957595051814
-0.733627954855	-0.957464244546
-0.733531333908	-0.957447074081
-0.733432766895	-0.957435929496
-0.733324457228	-0.957268074825
-0.733220620945	-0.957188188131
-0.733115138602	-0.956950252519
-0.733003394028	-0.956837626522
-0.732899101069	-0.956823943229
-0.732801496325	-0.956771236809
-0.732712537464	-0.95661978155
-0.732623770389	-0.956348401894
-0.732510058777	-0.956276807151
-0.732405340347	-0.956263752037
-0.7322792471	-0.956109975742
-0.73219409535	-0.95608480257
-0.732093527148	-0.956079048612
-0.731970324944	-0.955947457288
-0.731888403125	-0.955946138241
-0.731762826665	-0.955686602934
-0.731661021502	-0.955632419025
-0.731549066582	-0.955587965101
-0.731443086139	-0.955515228044
-0.731314012902	-0.9553937567
-0.73118972761	-0.95535026579
-0.731068690729	-0.955117319462
-0.730961335975	-0.955060955031
-0.730857722256	-0.954876456961
-0.730740188189	-0.954689375995

-0.730633019548	-0.954619024767
-0.730533224054	-0.954424046401
-0.7304311492	-0.954242738248
-0.730346561449	-0.954212312252
-0.730244322976	-0.954173915321
-0.730130800051	-0.954081222383
-0.730035487251	-0.954013114613
-0.729926912653	-0.953971689362
-0.729837938375	-0.953920910724
-0.729746416631	-0.953882235881
-0.729629585863	-0.953546172725
-0.729518310782	-0.953544658712
-0.729412256927	-0.953399140192
-0.72930111025	-0.953262923054
-0.72920503292	-0.953233952458
-0.729108491605	-0.953115717665
-0.729010159726	-0.953091618533
-0.72889498	-0.953072604897
-0.728791497613	-0.95306140455
-0.728696654025	-0.9530162881
-0.728594065527	-0.952919244535
-0.728476692668	-0.952903972883
-0.728381063858	-0.952709720472
-0.728271529554	-0.952565347311
-0.728146252399	-0.952549160381
-0.728031948862	-0.95247086933
-0.727924320727	-0.952454912029
-0.727833064192	-0.952381207303
-0.727743790583	-0.952244182474
-0.727637024399	-0.952052409123
-0.727548577833	-0.951992734298
-0.727465666217	-0.951929444627
-0.727375858997	-0.951854713888
-0.727283895535	-0.951784568039
-0.727185065039	-0.951739317041
-0.727076160503	-0.951424028302
-0.72697068947	-0.951412100286
-0.7268566906	-0.951005329451
-0.726749333952	-0.951004419516
-0.726678666947	-0.950900489533
-0.72657127621	-0.950755907165
-0.726486362641	-0.9507434921
-0.726374130055	-0.950668381908
-0.726260305412	-0.950651267912
-0.726164144132	-0.950590382347
-0.726025548823	-0.950511025495
-0.725916687919	-0.950417089963
-0.725821863464	-0.950246574618
-0.725712247108	-0.9502192917
-0.725613688955	-0.950178833343
-0.725512686313	-0.95003359864
-0.725424681562	-0.949864863255
-0.72530833482	-0.949679056569
-0.725218850554	-0.949622445245

-0.725134196382	-0.949597920425
-0.725028832781	-0.949572412441
-0.72492824055	-0.949553445634
-0.72484539365	-0.949452887899
-0.72474546354	-0.9493588205
-0.724648237118	-0.949354579637
-0.724545332223	-0.948924018581
-0.724442808561	-0.948815164613
-0.724323331214	-0.948598137162
-0.724235810255	-0.948411094717
-0.724143146614	-0.948318224541
-0.724043748265	-0.948188563
-0.723934479508	-0.947997675858
-0.723823866691	-0.947914789404
-0.723729859389	-0.947863574896
-0.723614235014	-0.947824305536
-0.723494414487	-0.947729203532
-0.723409279931	-0.947716937861
-0.723290575034	-0.947689456321
-0.723187509351	-0.947527397288
-0.72308946007	-0.947510827815
-0.722997067899	-0.947408386077
-0.722907650828	-0.947024104242
-0.722796648182	-0.946964341793
-0.72269886812	-0.946886346256
-0.722607954372	-0.946841890312
-0.722504524612	-0.946770795397
-0.722403404352	-0.9467175773
-0.722304659065	-0.946715777146
-0.722216434133	-0.946518084707
-0.72211972564	-0.946157341382
-0.722012809902	-0.946101990507
-0.721924491318	-0.946089570294
-0.721814846108	-0.945907320598
-0.721736681788	-0.945844823151
-0.721633532198	-0.945801030487
-0.721543371326	-0.945739246374
-0.721451464181	-0.945705134089
-0.721358124205	-0.945535245788
-0.721252357582	-0.945406208674
-0.721151238561	-0.945295529547
-0.721051881688	-0.945232523168
-0.720942513166	-0.944885931008
-0.720832044594	-0.944855512051
-0.720738293174	-0.944723662906
-0.720621639619	-0.944677808791
-0.720524488123	-0.9446589298
-0.720419685313	-0.944635183159
-0.720315131639	-0.944629621681
-0.720213866462	-0.944076170445
-0.720105324368	-0.94406040924
-0.719996816146	-0.944052240869
-0.71990146243	-0.943909727663
-0.719789852917	-0.943599114214

-0.719695635156	-0.943582854396
-0.719614426213	-0.943411663935
-0.71951731109	-0.94308451816
-0.719425390264	-0.942980046874
-0.719324970363	-0.942923614217
-0.719239834951	-0.942627895131
-0.719133633241	-0.94261096989
-0.719010161375	-0.942526543204
-0.718914649282	-0.942518549731
-0.718822199555	-0.941969736814
-0.718702191543	-0.941577007616
-0.718608979731	-0.941531024214
-0.718504026863	-0.941443232004
-0.718397624928	-0.9412546469
-0.718312006832	-0.941157260829
-0.718201957964	-0.940976356544
-0.718095187526	-0.940926705451
-0.718014898657	-0.94092003845
-0.717927837879	-0.940895809634
-0.717823976679	-0.94088085276
-0.717738717218	-0.940466554867
-0.71765147149	-0.94029663818
-0.717559074876	-0.940289588448
-0.71744755078	-0.940048472188
-0.717334424494	-0.940040489351
-0.717218592552	-0.940002489433
-0.717107598125	-0.939971744237
-0.717011464141	-0.939888132611
-0.716894212223	-0.939800526608
-0.716819243984	-0.939779062562
-0.716712720422	-0.939704510943
-0.71661836789	-0.939580123046
-0.716513766095	-0.939552581148
-0.716375293682	-0.939416024111
-0.716284234091	-0.939398510401
-0.716165604792	-0.939319801095
-0.716060511407	-0.939283214898
-0.715933147017	-0.939264849442
-0.715832185692	-0.939053359266
-0.715740138245	-0.93898850698
-0.715628012699	-0.938906820335
-0.715540162028	-0.938869950996
-0.715440181636	-0.938827096948
-0.715339157154	-0.938780613263
-0.715225487498	-0.93863177177
-0.71511960362	-0.938563325872
-0.715019011339	-0.938303480615
-0.714926202306	-0.938285421675
-0.714833733088	-0.938131059546
-0.714730707937	-0.937956335476
-0.714634736124	-0.937817161842
-0.714534255718	-0.93760814727
-0.714437234662	-0.937593197856
-0.714335665617	-0.93753170459

-0.714226824248	-0.937416211734
-0.714126919111	-0.937369450887
-0.71404411865	-0.937342138813
-0.713917720938	-0.937273341338
-0.713797915284	-0.937214149701
-0.71369848255	-0.937020243795
-0.713596548755	-0.936766833155
-0.713499037113	-0.936617213103
-0.71340134672	-0.936571910382
-0.713301082583	-0.936451286926
-0.713204601971	-0.936399242499
-0.713096934119	-0.936114919796
-0.713009658241	-0.936017599107
-0.712918798152	-0.93548284927
-0.712811435637	-0.935432445389
-0.71270508269	-0.935313972705
-0.712600485961	-0.935271135237
-0.712498422105	-0.934961306
-0.712406922729	-0.934788476293
-0.712312879522	-0.934779333937
-0.712217999064	-0.934672345893
-0.712111030908	-0.934637835691
-0.712020958851	-0.934631146005
-0.711919432439	-0.934605223437
-0.711805026568	-0.93453457153
-0.711706378783	-0.93446697175
-0.711622023413	-0.934218491846
-0.711517580593	-0.934068829128
-0.711412294282	-0.934037834334
-0.71131057173	-0.933925315942
-0.711208984991	-0.933766631054
-0.711101050674	-0.933747856021
-0.711006914939	-0.933694517266
-0.710903519816	-0.933694270771
-0.710786905633	-0.933368740167
-0.710667970174	-0.933367813361
-0.710554611669	-0.933348455418
-0.710477656946	-0.9332626403
-0.710330724232	-0.93321968776
-0.710202053393	-0.933218939457
-0.71008222374	-0.933066322254
-0.709989591645	-0.932906946618
-0.709908818181	-0.932893876385
-0.709832645093	-0.932666124541
-0.70973322178	-0.932611508523
-0.709636768911	-0.932597660707
-0.709530188708	-0.931731205795
-0.709424826164	-0.931667113414
-0.709320885514	-0.931551923624
-0.70923091879	-0.931309285458
-0.709125511473	-0.931164907087
-0.709022971217	-0.931058807363
-0.708915098776	-0.93105495028
-0.708811461846	-0.930795268849

-0.708718575065	-0.930493978544
-0.708634113952	-0.93034387647
-0.708532062723	-0.930290171742
-0.708430182213	-0.930258523261
-0.708325237865	-0.930150347136
-0.708220077551	-0.929880343531
-0.708135021941	-0.929858712378
-0.708028573534	-0.929851392776
-0.707937246593	-0.929804025059
-0.70783255894	-0.929728282554
-0.707732684323	-0.929694870342
-0.707634771993	-0.929630649434
-0.707530135502	-0.929609930772
-0.707421371472	-0.929549132618
-0.707291077881	-0.929470739411
-0.707184770898	-0.929425656114
-0.707089549342	-0.929382954337
-0.70698301033	-0.929260923925
-0.706888921254	-0.929127487523
-0.706774400907	-0.928997145505
-0.70666963313	-0.928959796308
-0.706546878912	-0.928811046429
-0.706449570043	-0.928496747714
-0.706349750846	-0.928423421643
-0.706248218964	-0.928296820167
-0.706137263513	-0.928219839632
-0.706022565379	-0.928095062406
-0.705921030599	-0.928062164829
-0.705825790011	-0.927932968678
-0.705726487136	-0.927874804654
-0.70563086524	-0.927705694737
-0.705540439874	-0.92767632125
-0.705438019401	-0.927518548817
-0.705344411705	-0.927302929115
-0.70525137829	-0.927212059641
-0.705155771459	-0.927209902289
-0.705052660336	-0.927200099851
-0.704930291432	-0.927193651348
-0.704824970375	-0.927099323574
-0.704726914219	-0.926898706389
-0.704639926664	-0.926896667094
-0.704550640393	-0.926809770693
-0.704439298143	-0.926774953645
-0.704347903873	-0.926710357516
-0.704224469848	-0.926642904825
-0.704145510777	-0.926623191319
-0.704069059563	-0.926596085666
-0.703968992542	-0.926456134723
-0.70386867026	-0.926437870135
-0.703758877698	-0.926248943503
-0.703636615233	-0.926235530004
-0.703552514944	-0.926172934981
-0.703476634055	-0.926127144315
-0.703372622529	-0.926125034694

-0.703267931758	-0.92610511117
-0.703170983749	-0.926032367742
-0.703067638484	-0.925760928087
-0.702981148298	-0.925678312762
-0.702875459553	-0.92564553817
-0.702773105326	-0.925588611569
-0.702687703775	-0.925440607324
-0.702598188467	-0.925262843598
-0.702488716159	-0.925104714744
-0.702397637205	-0.924995574058
-0.702310598339	-0.92492934167
-0.702217348129	-0.924840371124
-0.70211117837	-0.92483725254
-0.702009345831	-0.924710147167
-0.701916048888	-0.924527623725
-0.701809125279	-0.924491133972
-0.7016965865	-0.924484684887
-0.701600409551	-0.924393462953
-0.701511024613	-0.924220526884
-0.701393975029	-0.924177031088
-0.701281626309	-0.924156857459
-0.701162832913	-0.924030778278
-0.701061775867	-0.923847469687
-0.700970469737	-0.923796955579
-0.700869883936	-0.92379093555
-0.700787586392	-0.923710782191
-0.700671124439	-0.923682250897
-0.700582156826	-0.923588015946
-0.700499843164	-0.923551663632
-0.700394716135	-0.923445397395
-0.700291470872	-0.923367546195
-0.700201972019	-0.92306001506
-0.700095934532	-0.922917116682
-0.699998328168	-0.922818990223
-0.699912519422	-0.922747553537
-0.699808907377	-0.92259461525
-0.699691070189	-0.922574877236
-0.699598799509	-0.922412965923
-0.699463568246	-0.922412131869
-0.699358569949	-0.922381616076
-0.699267655655	-0.922040124859
-0.699187787346	-0.921798325584
-0.699079582481	-0.921352393964
-0.69898887072	-0.921110206739
-0.698874186817	-0.920913602321
-0.698779107026	-0.920703372964
-0.698693318059	-0.920517186685
-0.698576947696	-0.9202772192
-0.698479056615	-0.920254782093
-0.698397122402	-0.920213107286
-0.698301241817	-0.919963004082
-0.698208500785	-0.919845612846
-0.698104990554	-0.919839315486
-0.69797412193	-0.919724139359

-0.697883385812	-0.919641554853
-0.697790772062	-0.91931921569
-0.697693235888	-0.919306867348
-0.697586853682	-0.919214152336
-0.697487282125	-0.919069248927
-0.697390358637	-0.919066774948
-0.697293286279	-0.918900436437
-0.69720263336	-0.918665357804
-0.697100604138	-0.91864766945
-0.697010161771	-0.918605037327
-0.696919608655	-0.918379697261
-0.696809773855	-0.91833594949
-0.696731880654	-0.91800663335
-0.696597572039	-0.917995522172
-0.696483609378	-0.917907232554
-0.696374731492	-0.917592499159
-0.696270641854	-0.917540079781
-0.696152758498	-0.917488362442
-0.696050759785	-0.917386534721
-0.69596253048	-0.917350578122
-0.695855937268	-0.917231917931
-0.695758243682	-0.917105120047
-0.695657913172	-0.916959441656
-0.695574018671	-0.916824691635
-0.695482970239	-0.916815359802
-0.695366608961	-0.916768287648
-0.695271761067	-0.916637760597
-0.695148259613	-0.916609537326
-0.695028416853	-0.916471498019
-0.694939930542	-0.916416031784
-0.694865944222	-0.916365586706
-0.6947697826	-0.916267571483
-0.694677514904	-0.916254540535
-0.69459142887	-0.916216522826
-0.694501752293	-0.91596988196
-0.694402115931	-0.915905136002
-0.694286729225	-0.915779358614
-0.694187603323	-0.91575799119
-0.694089951223	-0.915518945757
-0.693992174987	-0.915505077593
-0.693896698742	-0.915376763598
-0.693805636548	-0.915271590371
-0.693684412224	-0.915209219655
-0.693582122845	-0.915161136758
-0.693469244749	-0.915102007518
-0.693365114427	-0.915026414667
-0.693243320971	-0.914929779593
-0.693131502085	-0.914921056611
-0.693020263159	-0.914763786861
-0.692942785581	-0.914683320941
-0.692843764735	-0.914666245251
-0.692742555627	-0.914490293404
-0.692657572093	-0.914380779813
-0.692556614267	-0.914351498044

-0.692453555916	-0.914347563679
-0.692350424704	-0.914305943282
-0.692250706597	-0.914200148247
-0.692153519336	-0.914137924217
-0.692063562431	-0.913901032498
-0.691960966298	-0.913898200251
-0.691863649973	-0.913868120521
-0.69176896885	-0.913808043261
-0.691660812555	-0.913787066427
-0.691563547883	-0.913703758344
-0.691459273448	-0.9136809806
-0.691360251722	-0.913269170498
-0.691254597143	-0.913218261723
-0.691166956103	-0.912955221248
-0.691081246904	-0.912809631376
-0.690972177507	-0.912779398752
-0.690880276407	-0.912756390385
-0.690776290641	-0.912521117368
-0.690679158701	-0.91248757422
-0.69057611541	-0.912169248655
-0.690458786522	-0.911936663137
-0.690336697467	-0.911880112394
-0.690228040591	-0.911835459567
-0.690126982989	-0.911744526941
-0.690019621733	-0.911647281721
-0.689918945692	-0.91157493164
-0.689804935354	-0.91150927442
-0.68971107604	-0.911504442451
-0.689619119991	-0.911466665498
-0.689540002289	-0.911461536021
-0.689436429487	-0.911435172061
-0.6893354416	-0.911422844079
-0.689248588339	-0.911398227061
-0.68914723838	-0.911308388783
-0.689042196016	-0.911253579559
-0.688933082815	-0.910881097073
-0.688840992164	-0.910788042985
-0.688739675283	-0.910771449374
-0.688634434303	-0.910611403683
-0.688543051278	-0.91042346942
-0.688449617044	-0.910418228937
-0.688349014367	-0.909937192911
-0.688241570398	-0.909682080797
-0.688138300172	-0.909643471718
-0.688032887046	-0.909587682508
-0.687931111774	-0.909512151367
-0.687802950296	-0.909496443932
-0.687699272968	-0.909479998256
-0.687608544723	-0.909458697493
-0.687505622625	-0.909424056585
-0.687417311549	-0.909402710788
-0.687332187671	-0.909297927247
-0.687243614686	-0.90908105752
-0.687153959419	-0.909053244671

-0.687043523338	-0.908974128375
-0.686963183347	-0.908940757311
-0.686867144397	-0.908872786831
-0.686782741736	-0.908858805457
-0.686681997869	-0.908554899768
-0.686570956512	-0.908501223841
-0.686480269455	-0.908249807099
-0.686385931878	-0.908156902776
-0.686300465419	-0.90806227394
-0.686197232966	-0.908011581584
-0.686093126711	-0.907926482527
-0.68598784371	-0.907843300589
-0.685881647498	-0.907841776527
-0.685778568932	-0.907760515831
-0.685682912604	-0.90757350239
-0.685599917446	-0.90697818717
-0.685497277673	-0.906628744953
-0.685396133915	-0.906492858243
-0.685290009288	-0.906405957181
-0.685183556254	-0.90622585801
-0.685076131945	-0.90614429938
-0.684946327	-0.906109081909
-0.684856261425	-0.906094931101
-0.684754461059	-0.905926975163
-0.684640948527	-0.905691664877
-0.684540930371	-0.905535507059
-0.684438054853	-0.905381081187
-0.684331617935	-0.905348716267
-0.684230958049	-0.905333033266
-0.684148938255	-0.90499389196
-0.684046108459	-0.904984654809
-0.683939736473	-0.904896558626
-0.683834382497	-0.904683031629
-0.683724173376	-0.904623824204
-0.683613918926	-0.904463560159
-0.683538182852	-0.904337175144
-0.683431344725	-0.904212226871
-0.683347212492	-0.904106668401
-0.683239003066	-0.903953980008
-0.683145850109	-0.90365533302
-0.683040178821	-0.90337608251
-0.68294342059	-0.903329979262
-0.682831565972	-0.903190683056
-0.682755885363	-0.903024920306
-0.682660200656	-0.902991205956
-0.682558767593	-0.902984016969
-0.682464892134	-0.902806364923
-0.682352686816	-0.902715118624
-0.682249783491	-0.902431084821
-0.682154074985	-0.902380943548
-0.682055116339	-0.902329539298
-0.681949290081	-0.90225141873
-0.681827283538	-0.902122960968
-0.681733086631	-0.901738429094

-0.681631750413	-0.901707527192
-0.681509948666	-0.901528048233
-0.681420760308	-0.901522385968
-0.681315152937	-0.901491826785
-0.68122819799	-0.901464970295
-0.681132695755	-0.901435858437
-0.681023143622	-0.90135920816
-0.680926114221	-0.901092229541
-0.680810914969	-0.900918967154
-0.6807147771	-0.90091491956
-0.680632162944	-0.900796308522
-0.680543996542	-0.900598362777
-0.68045781985	-0.90055461304
-0.680349296607	-0.900548146555
-0.680253644452	-0.900371137859
-0.680173323196	-0.900294398974
-0.680084568511	-0.900219316175
-0.679978407534	-0.900109338182
-0.67985898934	-0.899954864325
-0.679773182016	-0.899923415768
-0.679669694895	-0.89984517272
-0.679569930837	-0.899782935323
-0.679476605763	-0.899681933656
-0.679367194613	-0.899634460806
-0.679266763759	-0.899544351928
-0.679137497387	-0.899453721911
-0.679041148974	-0.899244143611
-0.678941141848	-0.899232065586
-0.678838437383	-0.899113881146
-0.678740453801	-0.899069312612
-0.678672881797	-0.898966755489
-0.678583485663	-0.898936698624
-0.678496063311	-0.898908420078
-0.678420884488	-0.898814729789
-0.678333379926	-0.898783801258
-0.678246016722	-0.898768803839
-0.678157213362	-0.898763224928
-0.678075024137	-0.898758603767
-0.677980147935	-0.89834855727
-0.677878412125	-0.898339019666
-0.677776284223	-0.898229398455
-0.677689318717	-0.898113639888
-0.677597549748	-0.897997281564
-0.677496082197	-0.897690877989
-0.677385303501	-0.897658596029
-0.67729631213	-0.897562031853
-0.677192012463	-0.897502554628
-0.677101479205	-0.897249016044
-0.676987546278	-0.896869933397
-0.676880291956	-0.896691920849
-0.676778327522	-0.89661294129
-0.676676282093	-0.896593781496
-0.676562708191	-0.896214998765
-0.676458996931	-0.896213960106

-0.676370571925	-0.89605558032
-0.676269844947	-0.895951242238
-0.676165459116	-0.89595003227
-0.67607362174	-0.895937400562
-0.675981044698	-0.895919866184
-0.675883816636	-0.895914573159
-0.675770576804	-0.895823432887
-0.675680579869	-0.895821816363
-0.675574260929	-0.895712722041
-0.675474068622	-0.895703178042
-0.675358467694	-0.895648471576
-0.675259481303	-0.895528655132
-0.675165891367	-0.895506519955
-0.675075814245	-0.895491384535
-0.67498667277	-0.895440765755
-0.67489691544	-0.895422591947
-0.674798830983	-0.895269321561
-0.67469940412	-0.895224117944
-0.674590746279	-0.89519407207
-0.674494839267	-0.895045485267
-0.674407590083	-0.895017227811
-0.674322365535	-0.894866219301
-0.67421107712	-0.894693060054
-0.674106995497	-0.894615482314
-0.673996202413	-0.894492105855
-0.673906114098	-0.894385417071
-0.673815978672	-0.894352491001
-0.673708015139	-0.894268828139
-0.673622236891	-0.894208882287
-0.673551766294	-0.894036814704
-0.673482742612	-0.893653143112
-0.673398264165	-0.893583701332
-0.67329000662	-0.893490868582
-0.673193916154	-0.893340917819
-0.673107258213	-0.893205584032
-0.673002275965	-0.893117680357
-0.672900765171	-0.89309334241
-0.672796536746	-0.892959388272
-0.672701179217	-0.892950321706
-0.672600584522	-0.892904619123
-0.6725138406	-0.89286066631
-0.67243356943	-0.892831263421
-0.672331713653	-0.892509305861
-0.672231225215	-0.8924979284
-0.672131954015	-0.892486589525
-0.672047541366	-0.892445811421
-0.671956590265	-0.89161848416
-0.671846179893	-0.891535472428
-0.671761053363	-0.891499974683
-0.671665221817	-0.891470553535
-0.671566916252	-0.891348388042
-0.671453961932	-0.891308300936
-0.671353880217	-0.891234098519
-0.671263875104	-0.891182021904

-0.671156231878	-0.891115159377
-0.671041916978	-0.890898007923
-0.670943468594	-0.890782490595
-0.670843341572	-0.890601615425
-0.670742813832	-0.89054333414
-0.670648278379	-0.890493364118
-0.670537826787	-0.890468543483
-0.670433189129	-0.890400586231
-0.670330369773	-0.890362351989
-0.670214573579	-0.890224888126
-0.670113039218	-0.890167855012
-0.670001343392	-0.890095715974
-0.669899856073	-0.889973255008
-0.669802735588	-0.889790300713
-0.669697983765	-0.889499375799
-0.669588477541	-0.889421827603
-0.669483482376	-0.889391268859
-0.66936733154	-0.888556672512
-0.669274809156	-0.888524458862
-0.669187650888	-0.888376413244
-0.669083700978	-0.888295293448
-0.668969957606	-0.888232261082
-0.668886235074	-0.888162188179
-0.668798425744	-0.888090478199
-0.668711934081	-0.888084667996
-0.668617511534	-0.888019899819
-0.668524290233	-0.887861725939
-0.668408331182	-0.887833466166
-0.668323522951	-0.887397451182
-0.668214016109	-0.887343627075
-0.668113936636	-0.887289395267
-0.668018074565	-0.886978403136
-0.667933010803	-0.886915323266
-0.667831019058	-0.886902901053
-0.667726933858	-0.886855410118
-0.667612293669	-0.886657043665
-0.667498420604	-0.88637228856
-0.667396903332	-0.886364812322
-0.66729780645	-0.88618678762
-0.667199251517	-0.886111786377
-0.667106235981	-0.88605620968
-0.666991615579	-0.885909076655
-0.66690135604	-0.885890618857
-0.666814171314	-0.885831720038
-0.666720953188	-0.885796914543
-0.666617821324	-0.885569456715
-0.666524441986	-0.885507258892
-0.666396481864	-0.885459587923
-0.666303009619	-0.88542076501
-0.66620412851	-0.885405182874
-0.666121065416	-0.885395864115
-0.666036546259	-0.885194333809
-0.665931270468	-0.885103297002
-0.66583669466	-0.885092991447

-0.665731655728	-0.884759652083
-0.665622145196	-0.88465903638
-0.6655284342	-0.884615628068
-0.665430916849	-0.884488992304
-0.6653394387	-0.884382702245
-0.665231227499	-0.884334235941
-0.665139965425	-0.884214549643
-0.665049057602	-0.884082101381
-0.664949144313	-0.883947906374
-0.66484151023	-0.883744198003
-0.664753939629	-0.883692834605
-0.664678404164	-0.883634413489
-0.664587349011	-0.883048064282
-0.664468924337	-0.882973078896
-0.664380091082	-0.882962690517
-0.664283180325	-0.882898717369
-0.664178279636	-0.882679993793
-0.664081482766	-0.882624055983
-0.663975197797	-0.882597489003
-0.663890026485	-0.882424177904
-0.66378737051	-0.882353458517
-0.663694316013	-0.882257010477
-0.663588347346	-0.882142881599
-0.663493881898	-0.881820597145
-0.663405102194	-0.881552279627
-0.663291875311	-0.881288036827
-0.663175463383	-0.881285608736
-0.663089097783	-0.881158145119
-0.662981758126	-0.881131765064
-0.66288685834	-0.880865326726
-0.662775759357	-0.880862121888
-0.66268506171	-0.880721754922
-0.66257870807	-0.880637560816
-0.662468676094	-0.880497141503
-0.662396584706	-0.880174395397
-0.662290753651	-0.87965283218
-0.662201029976	-0.879427968637
-0.662118983678	-0.879396046898
-0.662024690052	-0.879073612175
-0.661932689393	-0.878961966356
-0.661842985231	-0.878429899907
-0.661759852633	-0.878419786617
-0.661673856146	-0.87829414718
-0.661557942479	-0.878217609263
-0.661453241653	-0.87819128076
-0.661351866593	-0.878106748029
-0.661256165819	-0.877906473652
-0.661150078271	-0.877896969547
-0.661039832367	-0.877784756433
-0.660943243509	-0.877567543679
-0.660839543148	-0.877185998222
-0.660756301083	-0.877015991699
-0.660662162877	-0.876752023664
-0.660573088916	-0.876526141067

-0.660471065779	-0.87634791274
-0.660392525891	-0.876285483427
-0.660286393161	-0.876192456412
-0.660194092693	-0.876156530344
-0.660100789705	-0.875786861599
-0.660005634792	-0.875605915237
-0.659900550027	-0.875583894622
-0.659810941559	-0.875577311657
-0.659694575647	-0.875465006309
-0.659611211697	-0.875414163213
-0.659516157248	-0.875390523334
-0.65940975537	-0.875170306625
-0.659288566864	-0.87516282325
-0.659201807941	-0.875061328061
-0.659099048489	-0.874975892554
-0.659015144295	-0.874772628438
-0.658942749837	-0.874717862971
-0.658852015652	-0.874658436055
-0.658769381781	-0.874435165951
-0.65867090117	-0.874355607626
-0.658579300356	-0.874131977257
-0.658497942684	-0.874114989617
-0.658396543455	-0.87408623761
-0.658284723135	-0.874047624611
-0.658193838701	-0.873649075593
-0.65807566079	-0.873407463754
-0.657969916131	-0.873388342629
-0.657887107359	-0.873354545581
-0.657781634039	-0.8733298851
-0.657696979303	-0.872859042898
-0.657598805997	-0.872687361089
-0.657504829677	-0.872669457678
-0.657407033248	-0.872587881739
-0.657314820949	-0.872517150854
-0.657221308728	-0.872412414269
-0.65712082166	-0.872299969156
-0.657024954247	-0.87228862895
-0.656922127988	-0.872227280972
-0.656812206857	-0.871884932865
-0.65670832743	-0.871878005571
-0.656597011456	-0.871772290613
-0.656495644609	-0.871629550071
-0.656389393788	-0.871547325494
-0.656286458654	-0.871430520075
-0.656196846754	-0.871428426144
-0.656091399253	-0.871368762714
-0.655973979375	-0.871315493911
-0.655870932718	-0.871167659988
-0.655775327253	-0.870927240295
-0.655673373413	-0.870800051178
-0.655579712893	-0.870524985393
-0.655496334907	-0.870389643881
-0.655406776535	-0.870355525023
-0.655307182251	-0.87006974696

-0.655206679605	-0.870042655022
-0.655109035587	-0.869887838429
-0.655000755508	-0.869854793969
-0.654889235586	-0.869776659039
-0.654786150678	-0.869713185427
-0.654689490055	-0.869684281944
-0.654613330962	-0.869496752571
-0.654503950582	-0.869484652279
-0.654397452118	-0.869320316837
-0.654294139298	-0.869204913101
-0.654209924651	-0.869181039339
-0.654118127006	-0.86912285901
-0.653990830127	-0.868994852752
-0.653912128933	-0.868990674197
-0.653811290261	-0.868866726617
-0.653707167836	-0.868767001349
-0.653627300204	-0.86867540674
-0.653537812699	-0.868613507657
-0.653433248024	-0.868592485688
-0.65333354984	-0.868355826608
-0.653234829943	-0.868344181608
-0.653138975877	-0.867927939632
-0.653045554773	-0.867870517244
-0.652933096024	-0.867690472286
-0.652847974744	-0.867687401751
-0.652760868484	-0.867685627492
-0.65267998955	-0.867543640502
-0.652574168629	-0.867366030353
-0.652489034955	-0.867228711674
-0.652364296641	-0.867227325349
-0.652241390062	-0.867211307649
-0.652153406761	-0.867050114879
-0.65205811539	-0.866845353281
-0.651957719826	-0.866801368986
-0.651855227064	-0.866195355266
-0.651763248349	-0.866113804594
-0.651673347641	-0.866033897662
-0.65156345829	-0.865982248246
-0.651483735091	-0.865795829221
-0.651370167018	-0.865730155907
-0.651248907273	-0.865711183091
-0.651150856508	-0.865702160199
-0.651041090723	-0.865659178121
-0.650951685711	-0.86563008147
-0.650851565881	-0.865601946251
-0.650749782568	-0.865583249969
-0.650667335194	-0.865481486868
-0.650563780214	-0.865288986169
-0.650460405068	-0.865211554911
-0.650350778626	-0.865085866648
-0.650255627878	-0.865045307249
-0.650157266118	-0.864941712157
-0.650059874202	-0.864814295115
-0.649960753832	-0.864692423941

-0.649852113096	-0.864673901899
-0.649754597801	-0.864498265176
-0.649671583712	-0.864319026537
-0.649576463722	-0.864314245725
-0.649490501883	-0.864250248197
-0.649385845168	-0.864092066906
-0.649277412239	-0.864056342573
-0.649171667717	-0.863941422585
-0.649060405777	-0.863916523264
-0.648959505249	-0.863884553111
-0.648856023277	-0.863830485323
-0.648769788827	-0.863754630942
-0.648658661297	-0.863654995706
-0.648540738515	-0.863595225187
-0.648423550688	-0.863510983943
-0.648311182821	-0.862889339314
-0.648222899699	-0.862870626215
-0.648146428429	-0.862686963477
-0.648053281862	-0.86265158885
-0.647950363078	-0.862522217595
-0.647857107545	-0.862412824071
-0.647748983173	-0.862333162111
-0.647649127078	-0.862299608955
-0.64756735756	-0.862296872266
-0.647460966586	-0.86225399277
-0.647382927239	-0.862014619619
-0.647298338086	-0.861995992914
-0.647201926778	-0.861920486026
-0.647115105325	-0.861901395631
-0.647005194789	-0.861861963035
-0.646905352328	-0.861747880189
-0.64681312446	-0.86173438908
-0.64671848712	-0.861377328845
-0.646604707282	-0.861301433246
-0.646505067069	-0.861267481683
-0.646393099355	-0.861226604681
-0.646270712776	-0.861115712335
-0.646186892288	-0.861039725961
-0.646067987026	-0.860963803209
-0.645971584896	-0.860485256372
-0.645871753081	-0.860356945541
-0.645786883228	-0.860282089734
-0.645666290255	-0.860261435063
-0.645566211086	-0.860225242288
-0.645471066722	-0.860207533074
-0.645381885746	-0.860168190913
-0.645288246928	-0.860113767722
-0.645196495522	-0.860024482922
-0.64509416504	-0.859964723661
-0.644984473456	-0.859911904464
-0.644879265979	-0.85935837374
-0.64477814803	-0.859348045235
-0.644681572711	-0.859330027489
-0.64458483314	-0.859222196254

-0.644473336705	-0.859074921205
-0.644379272185	-0.858943020786
-0.644289879469	-0.85885805588
-0.644178343435	-0.858786672567
-0.644074602608	-0.85866630123
-0.64399488303	-0.858354866971
-0.643881348036	-0.858064670775
-0.643775681065	-0.857948986997
-0.643679041698	-0.857922411878
-0.643573416414	-0.857819542866
-0.643488342319	-0.857727745958
-0.643386127238	-0.857643351465
-0.643303354361	-0.857591154505
-0.643211802881	-0.857579814868
-0.643125619845	-0.85750437774
-0.643035746911	-0.857344937429
-0.642950711409	-0.857291189717
-0.642851493333	-0.857261355774
-0.642747145753	-0.857227258322
-0.642644732838	-0.857102219499
-0.642548834961	-0.856887410478
-0.642461018357	-0.856815665678
-0.642373209139	-0.856682851984
-0.642263773113	-0.856669896223
-0.642166673646	-0.856535592357
-0.642056619952	-0.856500757487
-0.641940500392	-0.856283984391
-0.641855343856	-0.856148638111
-0.641753588568	-0.855914713765
-0.641647985483	-0.855503151675
-0.641548557103	-0.855431696478
-0.641433105019	-0.855415185223
-0.641320383615	-0.855375747054
-0.641237128586	-0.855203369105
-0.64113114044	-0.855139386658
-0.641043360663	-0.855012252765
-0.640952392134	-0.85500219755
-0.640857877849	-0.854952554767
-0.640772497614	-0.854811438801
-0.640669258301	-0.854402871453
-0.640564484334	-0.854371055381
-0.640468986444	-0.854235643937
-0.640381430793	-0.854164491769
-0.640290771196	-0.85402666818
-0.640197196782	-0.854006973875
-0.640109824953	-0.853937030424
-0.640028078837	-0.853933203045
-0.639944041627	-0.853925276128
-0.639838812938	-0.853871762858
-0.639735247677	-0.853804584366
-0.639632563313	-0.853677703914
-0.639534054154	-0.853656338677
-0.639436106375	-0.853618762739
-0.63934437885	-0.853307381898

-0.639234392059	-0.853117750778
-0.639136568209	-0.853102493622
-0.63904137796	-0.853019320581
-0.638940656082	-0.853019002084
-0.638848903051	-0.852994563805
-0.638753593766	-0.852717469328
-0.638659770641	-0.852245135017
-0.638537928428	-0.852030637581
-0.638433687272	-0.85197079363
-0.638349120994	-0.851962748411
-0.638255874551	-0.851950411094
-0.638177312177	-0.851923935157
-0.638087055704	-0.851625950785
-0.637991046132	-0.851550101779
-0.637896359118	-0.851514938734
-0.637804966398	-0.851217659687
-0.637711185424	-0.850945220904
-0.63761649638	-0.850719763668
-0.637521130227	-0.850705807899
-0.637418369317	-0.850564437249
-0.6372980844	-0.850297619396
-0.637202076842	-0.850224902163
-0.637101979543	-0.849671660358
-0.637009979809	-0.849490413567
-0.636922330754	-0.849414128838
-0.636829698557	-0.849342603606
-0.636714628723	-0.849282769514
-0.636606703422	-0.849233483995
-0.636518050739	-0.849198475651
-0.636413190202	-0.849169050446
-0.636326954301	-0.849087365046
-0.636225038843	-0.848909544311
-0.636136332009	-0.84886660462
-0.636036527758	-0.848771630759
-0.635948511649	-0.84860535347
-0.635840536307	-0.848573843217
-0.635738975134	-0.848513795522
-0.635648914928	-0.848380587496
-0.635555407889	-0.84824841934
-0.635455194474	-0.84816161649
-0.635357125743	-0.847899083704
-0.635253866141	-0.847800818023
-0.635159140714	-0.847637966207
-0.635059178385	-0.847606916203
-0.634946730656	-0.847555919938
-0.634856233373	-0.847552450309
-0.634765775275	-0.847399753449
-0.634678657785	-0.847284996809
-0.634593849231	-0.847178074221
-0.634497040543	-0.847145316264
-0.634392574802	-0.846891335435
-0.634296341262	-0.84685092816
-0.634178446042	-0.846558508402
-0.634066963666	-0.846114175147

-0.633965664291	-0.846107056495
-0.633851611329	-0.846072968536
-0.633750989337	-0.845907683787
-0.633648698654	-0.845663582335
-0.633550940649	-0.845393088361
-0.633461196038	-0.845259882469
-0.633368323642	-0.844957898326
-0.633267163925	-0.844936119759
-0.633175646934	-0.844850911981
-0.633089272306	-0.84482879683
-0.632974097613	-0.844589639804
-0.632875174161	-0.844576861479
-0.632788520167	-0.844536428779
-0.632707300695	-0.844533501468
-0.632608885871	-0.844434428108
-0.632497944959	-0.844385239381
-0.632419802664	-0.844338081111
-0.63232489181	-0.844219620077
-0.632214306208	-0.844210752564
-0.632139538365	-0.844063243473
-0.632037919188	-0.843984523336
-0.6319477142	-0.843834306454
-0.631848616177	-0.843696746103
-0.631753632005	-0.843671111958
-0.631640018694	-0.843521577995
-0.63154504028	-0.84327093555
-0.631448211188	-0.843196166651
-0.631340049188	-0.84312840221
-0.631258607008	-0.843122470837
-0.631169812308	-0.843028847969
-0.63107580981	-0.842711407034
-0.630973641462	-0.842694338308
-0.630884632951	-0.842657415568
-0.630782355297	-0.84265592187
-0.630676890501	-0.842498980096
-0.630579494988	-0.842376115426
-0.630496850867	-0.842198131575
-0.630403443317	-0.842179125001
-0.630303119414	-0.842091909562
-0.630216148027	-0.842051675616
-0.630121023934	-0.841999803373
-0.630023597331	-0.841811762532
-0.629915300893	-0.841702935141
-0.629809237906	-0.841399133081
-0.629725271137	-0.84133032784
-0.629621835039	-0.841310400107
-0.629530964187	-0.841298399636
-0.629435585243	-0.841244391654
-0.629340138332	-0.841036161736
-0.629239142238	-0.841030929925
-0.629143408661	-0.840890337968
-0.629031216804	-0.840859364964
-0.628924934662	-0.84078007956
-0.628824113752	-0.84071389863

-0.628734409782	-0.840698577216
-0.628636346748	-0.840690337027
-0.628541465045	-0.840525121866
-0.628443414588	-0.840265968723
-0.628346598675	-0.840218232157
-0.628262337188	-0.840185410961
-0.628172779448	-0.840120206352
-0.628093720914	-0.839850640633
-0.62797512491	-0.839641078809
-0.627874910376	-0.839390555072
-0.627759090758	-0.839387444436
-0.627663913305	-0.839377643832
-0.627561105213	-0.839303053828
-0.627467759211	-0.83926246273
-0.627361961348	-0.839039965699
-0.6272753385	-0.838885115532
-0.627189080357	-0.838701555496
-0.627112741599	-0.838632714759
-0.62701838321	-0.838489955788
-0.626928270479	-0.838215686288
-0.626852082162	-0.837999708026
-0.626751503425	-0.837812810678
-0.626653031189	-0.837722921613
-0.626524313424	-0.837160691919
-0.626436763594	-0.836809740864
-0.626333453806	-0.836787191065
-0.62624680684	-0.836745719482
-0.626138475088	-0.836721437617
-0.626056105455	-0.836615894307
-0.625968466314	-0.83638820415
-0.625868058317	-0.836184713922
-0.625787408599	-0.836093879344
-0.625659912677	-0.836011867844
-0.625579597839	-0.835841403203
-0.625492305913	-0.835751331103
-0.625412794751	-0.835571136552
-0.625297642613	-0.83552552941
-0.625200463539	-0.835494585271
-0.62510951847	-0.835271189431
-0.625043888612	-0.834925720113
-0.624948142577	-0.834867784042
-0.62485682704	-0.83486226958
-0.624755519506	-0.834833670081
-0.624655313162	-0.834802884458
-0.624566072981	-0.834684376554
-0.624469049955	-0.834669797204
-0.624369390251	-0.834603499814
-0.624289512425	-0.834330359926
-0.624210756835	-0.834083642788
-0.624119539507	-0.833985727725
-0.624019134137	-0.833970328915
-0.623914529247	-0.833738354311
-0.623820016083	-0.833619485656
-0.623703378311	-0.833612464974

-0.623609755056	-0.833600545897
-0.623506506107	-0.833520201312
-0.623422810608	-0.833497961984
-0.623321161165	-0.833482113501
-0.62323393062	-0.833402381261
-0.623155623561	-0.833318460387
-0.62308050059	-0.83305713627
-0.622979455752	-0.832913967895
-0.622864020433	-0.832845303481
-0.62275320699	-0.832332724496
-0.622654749831	-0.832312137627
-0.622573147398	-0.832156850745
-0.622460310485	-0.83213388583
-0.622372005233	-0.832095350327
-0.622286852149	-0.832073127716
-0.62219778776	-0.83191969579
-0.622102999032	-0.831894980019
-0.622011805822	-0.831851030324
-0.621930933857	-0.831820941456
-0.621847728515	-0.831802549511
-0.621754815329	-0.831705821042
-0.621663193212	-0.831334241665
-0.621570508095	-0.831287784935
-0.621461732367	-0.831284903304
-0.621380886414	-0.831167702981
-0.621284596613	-0.831094696212
-0.621197348471	-0.831041452387
-0.621097989792	-0.831012134906
-0.620993388796	-0.830949939334
-0.620908539011	-0.8309469666
-0.620804232804	-0.830936920054
-0.620685032293	-0.830795704668
-0.620606723726	-0.83067069009
-0.620518736645	-0.830532406771
-0.620429880088	-0.830106326494
-0.620341007699	-0.829874915437
-0.620236678838	-0.829770025945
-0.620142263321	-0.829736497046
-0.620029078212	-0.82967605533
-0.619937398021	-0.82961818273
-0.619825824717	-0.829610578274
-0.61971691809	-0.829473326379
-0.619594995133	-0.829414290459
-0.619501474228	-0.829357674629
-0.619423803201	-0.829308188497
-0.619315856486	-0.829122133673
-0.619207396983	-0.828999833867
-0.619105670081	-0.828872570367
-0.619022293741	-0.828865943455
-0.618929820386	-0.828851140582
-0.61883218719	-0.828822908523
-0.618746179433	-0.828809576567
-0.618616769768	-0.828805732716
-0.618523657149	-0.828712562814

-0.618435802768	-0.828490417294
-0.61835842764	-0.828476959129
-0.618258790827	-0.828448411195
-0.618186586627	-0.82839179869
-0.618080075757	-0.828379629168
-0.617984448446	-0.827975437806
-0.617898904855	-0.827933528146
-0.617816255216	-0.827896635979
-0.61771858441	-0.827771827181
-0.617606527018	-0.827639409127
-0.6175043018	-0.827631007093
-0.617404018175	-0.827087912558
-0.617316698983	-0.827060978556
-0.61721675353	-0.826927598767
-0.61713584589	-0.826834270831
-0.617052814707	-0.826661663866
-0.616949966145	-0.826445675486
-0.616864809036	-0.826166906377
-0.61678019988	-0.82604627715
-0.616696034334	-0.825967604773
-0.616615421416	-0.825948232614
-0.616534433793	-0.825896062049
-0.61643676804	-0.825699322601
-0.61633937277	-0.825611662286
-0.616244462107	-0.825456490658
-0.616160511802	-0.825378996539
-0.616076250414	-0.825202465861
-0.615993118068	-0.825168036584
-0.615886728071	-0.825053953013
-0.615804344212	-0.825022390199
-0.615713358797	-0.82498517873
-0.615626531615	-0.824918284871
-0.615545807477	-0.824836550296
-0.615460894226	-0.824604967117
-0.615376315122	-0.824470288572
-0.615273103433	-0.824070507315
-0.615162272169	-0.823892547709
-0.615064786069	-0.823848891461
-0.614964902741	-0.823711201089
-0.614836321022	-0.823699276695
-0.614727131372	-0.823644827813
-0.614639132676	-0.823554270945
-0.614542766986	-0.823513888951
-0.614440716152	-0.823481866408
-0.614352119057	-0.823403276831
-0.614243813051	-0.823311423544
-0.614154068294	-0.823159565331
-0.614058618771	-0.823115511421
-0.613959081361	-0.823031202736
-0.6138703778	-0.822975065593
-0.613780351294	-0.822942602557
-0.613690865991	-0.822940729934
-0.613586311357	-0.822740065173
-0.613475110452	-0.82269634191

-0.613382310389	-0.822690820344
-0.613273885606	-0.822568087197
-0.613179302766	-0.822528298808
-0.613052720822	-0.822454937326
-0.612973187931	-0.822428953698
-0.612877840402	-0.822382773278
-0.612782269186	-0.822184264942
-0.612668324716	-0.821939579193
-0.612575418109	-0.821577322087
-0.612477177034	-0.821514287701
-0.612385253598	-0.821354677628
-0.61229904126	-0.821344763503
-0.612215501379	-0.821342205529
-0.612122288141	-0.821331252771
-0.612025791264	-0.821292060647
-0.611937970426	-0.82124977483
-0.611834290672	-0.821171096323
-0.611746814861	-0.821154248117
-0.611675983363	-0.820746895597
-0.611598192877	-0.820718672774
-0.611497963192	-0.820668567704
-0.611398338025	-0.820589282189
-0.611311841653	-0.820546276281
-0.611215120081	-0.820432876028
-0.611124105493	-0.820392094042
-0.611017279318	-0.820390248491
-0.610921533741	-0.820270258229
-0.610826801473	-0.820081367901
-0.610726803612	-0.819822796995
-0.610629214411	-0.819666083575
-0.610526660768	-0.819633605612
-0.610424354547	-0.819575274695
-0.61032716688	-0.819260638576
-0.610226747493	-0.81925717092
-0.610133037513	-0.819098992657
-0.610003995259	-0.818865174193
-0.609894006137	-0.818822904444
-0.609783777712	-0.818639113739
-0.609681927474	-0.81862194582
-0.6095821018	-0.818487591796
-0.609484898099	-0.818447584664
-0.609407216539	-0.818444404694
-0.609321450147	-0.818000342324
-0.609221063446	-0.817947487587
-0.609124757159	-0.817930968057
-0.609037436167	-0.817369100834
-0.608942667899	-0.817257963931
-0.608866311692	-0.817110310403
-0.608764921544	-0.817045905359
-0.60867417504	-0.81691387533
-0.60858663717	-0.816868272021
-0.6084942625	-0.816532429743
-0.608401945492	-0.816496050475
-0.608292632986	-0.816434549935

-0.608200786803	-0.816429472078
-0.608118427504	-0.816369242075
-0.608002183668	-0.81634929075
-0.607896561206	-0.816156570172
-0.6078037383	-0.8161510679
-0.607695711273	-0.816103882131
-0.607605451656	-0.816070899242
-0.607524817305	-0.815979196104
-0.607418521263	-0.815961268335
-0.607323953818	-0.815917147925
-0.607205599291	-0.81590091907
-0.607112034577	-0.815765257915
-0.607007970343	-0.815465064207
-0.606907850744	-0.815302821309
-0.606828958444	-0.815299680572
-0.606711117865	-0.815019329384
-0.606618552555	-0.814838956342
-0.606516965638	-0.814782425156
-0.606430284148	-0.814489632388
-0.606345046816	-0.814485649248
-0.606238532074	-0.814462560708
-0.60613917321	-0.81437761669
-0.606044881759	-0.814202132613
-0.605967403449	-0.813979553981
-0.605873878365	-0.813823606788
-0.605780557351	-0.813811577997
-0.605691698792	-0.813709137709
-0.605584101143	-0.813588814729
-0.605493381991	-0.813386854208
-0.605405758959	-0.8132826894
-0.605321143257	-0.813270391483
-0.605222231818	-0.81280352078
-0.605123791524	-0.812526857446
-0.60501854284	-0.812463069942
-0.604923487086	-0.812401904044
-0.604832525188	-0.812269516964
-0.604724803106	-0.811913333787
-0.604624539023	-0.811343363075
-0.604512556215	-0.811342531762
-0.604409952948	-0.811325071284
-0.604317690507	-0.811293209474
-0.604222042199	-0.810698662116
-0.604112048825	-0.810596349203
-0.604021778036	-0.810491966792
-0.603938043187	-0.810379686568
-0.603850693346	-0.810164271916
-0.603736656803	-0.810140566137
-0.603646039755	-0.810106153104
-0.603553906393	-0.810078027056
-0.603444811426	-0.810050656708
-0.603356147833	-0.809791204824
-0.603270451058	-0.809685381592
-0.603184653883	-0.809345205126
-0.603103686089	-0.80932729017

-0.603029199233	-0.80921244015
-0.60292961658	-0.80918564416
-0.602837052289	-0.808899122998
-0.602721969596	-0.80885074625
-0.602618327333	-0.808841596977
-0.602514582509	-0.808659614395
-0.602417095629	-0.808627574678
-0.602314586156	-0.80830891196
-0.602186461422	-0.808033104482
-0.602075025335	-0.807930152252
-0.601978079682	-0.807923754736
-0.601884749488	-0.807921711604
-0.60178145735	-0.807911744143
-0.601678265774	-0.807277686011
-0.601585868085	-0.80711362479
-0.60147889131	-0.807095489591
-0.601383356246	-0.8070708732
-0.601289059562	-0.807033815262
-0.601196805531	-0.806993268236
-0.601096793022	-0.806863715202
-0.600985170259	-0.806765225044
-0.600869735205	-0.806735560633
-0.600778990638	-0.806512801136
-0.600684984144	-0.806505699618
-0.600590892549	-0.806424980707
-0.600507151655	-0.806354298926
-0.600406260722	-0.806285240785
-0.600320769242	-0.806209655589
-0.600228114651	-0.80607282699
-0.600146424943	-0.805934308605
-0.600045952776	-0.805910222122
-0.599965997715	-0.80588731879
-0.599878725078	-0.80582259456
-0.599773526861	-0.805730806141
-0.599667573896	-0.805659934872
-0.599578951724	-0.805618177177
-0.599472364023	-0.805595881721
-0.599372203935	-0.805493813577
-0.599270694116	-0.805293978804
-0.599158083889	-0.805293470113
-0.599059579339	-0.805187564903
-0.598967693297	-0.805156134701
-0.598871908873	-0.804985982731
-0.598783101698	-0.80489705791
-0.598703331887	-0.804843012489
-0.598590007106	-0.804806898505
-0.598496339708	-0.804436976846
-0.598401014253	-0.804340758704
-0.598302841888	-0.804296962883
-0.598202695935	-0.804056061169
-0.598106994306	-0.803956831153
-0.598008049206	-0.803773559959
-0.597903702744	-0.803564634759
-0.597807781864	-0.803322540623

-0.597715764961	-0.803189838606
-0.59761636598	-0.80281437917
-0.597539839865	-0.802778539698
-0.597452075982	-0.802719261533
-0.597375258419	-0.802568526696
-0.597266094259	-0.802512871434
-0.597173153434	-0.802399623526
-0.597087377542	-0.802354734304
-0.597006402095	-0.802270930114
-0.596908082313	-0.802049720675
-0.59681872503	-0.802015270279
-0.59673890731	-0.801984327914
-0.596634426055	-0.80196569443
-0.596555480868	-0.801877905532
-0.596442227108	-0.801565904434
-0.596335428515	-0.80147073789
-0.596229337828	-0.801366328841
-0.596165807921	-0.801326704075
-0.596066339549	-0.801197677431
-0.59597459835	-0.801119181717
-0.595879507391	-0.800939422777
-0.595783345098	-0.800694709418
-0.595701047917	-0.800628243224
-0.59560281994	-0.80061104328
-0.59551316792	-0.800313971662
-0.595426864146	-0.80027785024
-0.595308896537	-0.800199384229
-0.595207746005	-0.800156902248
-0.595125471813	-0.800144783975
-0.595035437759	-0.800005577073
-0.594935803277	-0.799954117874
-0.594844262336	-0.799824616
-0.59473791616	-0.79971551578
-0.594657476241	-0.799681147966
-0.594563420198	-0.79960823435
-0.594462558789	-0.799580423785
-0.594372058156	-0.799541806152
-0.594271588372	-0.799515436215
-0.594191659987	-0.799425863528
-0.594096149194	-0.799414612062
-0.594000433648	-0.799378902908
-0.593894310804	-0.799368664589
-0.593800873161	-0.799310856133
-0.593693810217	-0.799302822207
-0.593563533203	-0.799298167535
-0.593477635754	-0.799250109582
-0.593391425952	-0.798570473232
-0.593289607269	-0.798467087906
-0.593196768077	-0.798172399221
-0.593110712504	-0.798163306005
-0.593018340552	-0.798145918282
-0.592936265029	-0.797979944311
-0.592829651301	-0.79789167006
-0.592745754012	-0.797778919552

-0.592656783726	-0.797717997231
-0.592559624219	-0.797483876083
-0.59245432611	-0.797480284104
-0.592364548081	-0.797450403113
-0.592267846975	-0.797327426483
-0.592158544261	-0.797151980309
-0.592075352531	-0.797144936522
-0.591997282678	-0.797128957447
-0.591905793428	-0.796794508126
-0.59181425444	-0.796760732981
-0.591711461091	-0.796737654803
-0.591624934763	-0.796695780481
-0.591539342488	-0.796607538723
-0.591457308577	-0.796457928058
-0.591375829467	-0.796406630835
-0.591295624217	-0.796169746637
-0.591192756865	-0.796029081558
-0.59110196651	-0.796011421749
-0.590989258454	-0.795871203739
-0.590903430754	-0.795740537903
-0.590797108976	-0.795718166094
-0.590710129121	-0.795462958567
-0.590604729226	-0.79537401703
-0.590498317924	-0.795311865143
-0.590411812189	-0.795250564398
-0.590322915622	-0.795185562176
-0.590241409417	-0.79481227698
-0.590176782267	-0.794779129464
-0.590079606576	-0.794747598523
-0.589988242217	-0.794462245147
-0.58990485772	-0.794458501413
-0.589813452608	-0.794287999378
-0.589729471171	-0.794279104529
-0.589628481433	-0.794246838644
-0.589537552485	-0.794232363255
-0.589441357053	-0.794207843022
-0.589361035287	-0.794206965751
-0.589275906154	-0.79405728487
-0.589184975002	-0.793737618881
-0.589083704393	-0.793636398912
-0.588972674238	-0.79346510855
-0.588876268165	-0.79341351346
-0.588781527339	-0.79320416708
-0.588683311936	-0.793077812203
-0.588590740059	-0.792953420657
-0.588477258921	-0.792861430377
-0.588387402311	-0.792793312565
-0.588284444103	-0.792207344466
-0.588187239433	-0.792196193054
-0.588098982241	-0.792119952511
-0.587997928791	-0.792055314766
-0.587890562458	-0.79199051023
-0.587793990295	-0.791901667653
-0.587687440718	-0.791562893948

-0.587572618075	-0.791485063542
-0.587483622153	-0.791364024944
-0.587380041446	-0.791323208754
-0.587285596597	-0.791156941288
-0.587196696178	-0.791118150965
-0.587099077234	-0.790924171869
-0.587012637551	-0.790918637817
-0.586923512452	-0.790825732743
-0.586824171175	-0.790531559218
-0.586735065448	-0.790486156072
-0.586651985198	-0.790471381694
-0.586568580815	-0.790385859659
-0.586477908266	-0.790236077861
-0.586366766217	-0.790121132721
-0.586274544913	-0.79011745066
-0.586179838379	-0.790096071019
-0.586083436666	-0.790007409154
-0.586003950191	-0.789883171319
-0.585926339347	-0.789755356848
-0.58582900323	-0.789678383693
-0.585747805907	-0.789613863368
-0.5856709146	-0.789440773361
-0.585564713017	-0.789416678966
-0.585441104756	-0.789408973186
-0.585340133284	-0.789279030073
-0.585241401426	-0.78926168362
-0.58513000331	-0.788958908041
-0.585016581227	-0.788923259138
-0.584918426878	-0.788875027588
-0.584826474568	-0.788842331137
-0.584726155426	-0.788840316459
-0.584646930878	-0.78882991932
-0.584600367754	-0.788501282031
-0.584505308821	-0.788430152328
-0.584397874208	-0.788371340548
-0.584288075126	-0.788214754512
-0.584193114832	-0.788055110607
-0.584093018867	-0.787959568948
-0.584000487379	-0.787878334655
-0.583904764404	-0.787850329474
-0.583811786735	-0.787824833128
-0.583717075364	-0.787810537392
-0.583601270172	-0.787789769417
-0.583509883371	-0.787767261767
-0.583404382268	-0.787645572711
-0.58328871141	-0.787496392334
-0.583192365094	-0.787186936328
-0.583087774256	-0.787041479543
-0.582991244709	-0.78702119781
-0.582888207725	-0.786883511678
-0.582799985618	-0.786670587372
-0.582702301573	-0.786333285409
-0.582593175095	-0.786245436342
-0.582512864613	-0.786141427665

-0.582428388023	-0.786072805626
-0.582336545933	-0.786003464695
-0.582243597579	-0.785954006118
-0.582144817129	-0.785733216739
-0.58204185838	-0.785674285024
-0.581938582296	-0.785580363539
-0.581848180107	-0.785511085461
-0.581747502498	-0.785473621284
-0.581642521343	-0.785459480495
-0.581544273099	-0.785319974576
-0.581440473171	-0.785204823529
-0.581333194927	-0.785048261386
-0.581263476113	-0.78494348728
-0.581170979075	-0.784866259645
-0.581082935533	-0.784861099895
-0.580992214831	-0.784837932291
-0.580894463846	-0.78478683567
-0.58080993323	-0.784665396359
-0.580708663532	-0.784267497423
-0.580616833069	-0.784243510525
-0.580527801314	-0.7842041504
-0.580440495907	-0.783940091169
-0.580349915092	-0.783922807816
-0.580267600993	-0.783922149189
-0.580185241699	-0.783878502053
-0.580087574394	-0.783859134069
-0.579984653208	-0.783775709278
-0.579891136601	-0.783636674361
-0.579798314807	-0.783535602551
-0.579689392148	-0.783301518016
-0.5796069227	-0.783285490399
-0.579522719646	-0.783247495802
-0.579438437721	-0.783047764086
-0.579356447079	-0.782935138209
-0.579260699236	-0.782639668496
-0.579147771254	-0.782619088032
-0.5790724767	-0.782291729925
-0.578977723191	-0.782232250843
-0.578898576178	-0.782214416987
-0.578792712343	-0.782078804513
-0.578680199461	-0.782015002163
-0.578603863328	-0.782001196703
-0.578498072716	-0.781984527341
-0.578385545145	-0.781903421801
-0.578291650327	-0.781474094328
-0.57820128692	-0.781397029081
-0.578103211223	-0.781284262837
-0.578006298188	-0.781248980381
-0.577928534076	-0.781022743433
-0.577839346098	-0.780981940026
-0.577751807246	-0.780676417089
-0.57764579726	-0.78060112746
-0.577519303118	-0.780529555298
-0.577421103796	-0.7804255488

-0.577346980422	-0.78037760673
-0.57725931275	-0.780342269986
-0.577154972993	-0.780096397772
-0.577056426215	-0.780096233472
-0.576959859671	-0.780080903773
-0.576870976052	-0.780049331146
-0.576771668294	-0.780045020876
-0.576675796227	-0.779519733618
-0.576570805254	-0.779218574565
-0.576475949834	-0.779126985173
-0.576393922385	-0.779090174015
-0.576284646095	-0.779033657787
-0.576194723458	-0.778962770795
-0.576090663339	-0.778920629218
-0.576011524422	-0.778729143804
-0.575914318006	-0.778702501799
-0.575793956364	-0.778570614798
-0.575688190563	-0.778191703841
-0.575588070226	-0.778180339864
-0.575492919167	-0.778167197673
-0.57541891175	-0.777989045459
-0.575324921777	-0.777782439558
-0.57523636394	-0.777730339244
-0.575170614824	-0.77771353716
-0.575084095377	-0.777550140795
-0.574984781517	-0.77730274019
-0.574902281774	-0.777296817841
-0.574786795494	-0.777077532637
-0.574679396649	-0.777062997769
-0.574562908991	-0.777049458821
-0.574480187843	-0.777043202933
-0.574374302888	-0.776941800196
-0.574263409709	-0.776916256208
-0.574184752545	-0.776846646131
-0.574073619137	-0.776813647206
-0.573964707257	-0.776780657431
-0.573874402794	-0.776779035482
-0.57378456151	-0.776722794867
-0.573666721389	-0.776587159655
-0.573579936569	-0.776534214603
-0.573483852496	-0.77604237419
-0.573389519861	-0.776007920598
-0.57329898492	-0.775967160617
-0.573221425598	-0.775811062607
-0.573131130756	-0.775567390596
-0.573032912491	-0.775512510994
-0.572936710268	-0.775445819984
-0.572840344792	-0.775400359208
-0.57275085049	-0.775049976727
-0.572660887299	-0.775012002417
-0.572546610053	-0.775007915178
-0.572450714164	-0.774970098146
-0.572333561741	-0.77484417711
-0.57223403545	-0.774828399778

-0.572141733276	-0.774796218056
-0.572051783093	-0.774781264993
-0.571967314761	-0.774751011175
-0.571862033087	-0.77455900051
-0.571751896459	-0.774470571449
-0.571658732364	-0.774455674763
-0.571565380416	-0.774392558668
-0.571479599776	-0.774219270176
-0.571391416939	-0.774119434507
-0.571300283262	-0.774100521343
-0.571218573704	-0.774087966744
-0.571127830142	-0.773910292994
-0.571047055832	-0.773847774406
-0.570958091707	-0.773802624678
-0.570865874224	-0.773630402853
-0.570780296771	-0.773490442672
-0.57067818243	-0.773474220843
-0.570556998982	-0.773471029433
-0.570462227626	-0.773184308472
-0.570380226779	-0.773030619456
-0.570282470987	-0.772765690644
-0.570195784826	-0.77273787087
-0.570103926581	-0.772715532742
-0.570005220879	-0.772679911267
-0.569904311216	-0.77259964696
-0.569798579427	-0.772539333878
-0.569712401394	-0.772519797377
-0.569622925878	-0.772462405643
-0.569535154086	-0.772321001531
-0.569453148709	-0.772318558247
-0.569364641514	-0.772212666305
-0.569275216304	-0.772177669114
-0.5691794172	-0.772113311135
-0.569104509019	-0.772049688341
-0.569018708	-0.772005080858
-0.568929491811	-0.771981379065
-0.5688315111	-0.77173851549
-0.568732556829	-0.771616810163
-0.568630630579	-0.771593899635
-0.568542779958	-0.771499577019
-0.568459152392	-0.771419658416
-0.568372454534	-0.770789382245
-0.568271413285	-0.770597836583
-0.568171541777	-0.770545779193
-0.568058751735	-0.770276153438
-0.567984185537	-0.770224588542
-0.567887880139	-0.770145961791
-0.567805233766	-0.77009056925
-0.567699840533	-0.770021043351
-0.567595616728	-0.769993921091
-0.56748662397	-0.769987834385
-0.567391476545	-0.769954001703
-0.5673009525	-0.769895274425
-0.567206889259	-0.769811885796

-0.56710915491	-0.76976640037
-0.567005777375	-0.769756753162
-0.566901492696	-0.769712731566
-0.566790588584	-0.76965457155
-0.56670352773	-0.769553969456
-0.566617698965	-0.769460490807
-0.566534533301	-0.769306799801
-0.566436434186	-0.769129994172
-0.56635274391	-0.769077401092
-0.566279558422	-0.768998495838
-0.566201448254	-0.768814180434
-0.566096136845	-0.768676573534
-0.565972915446	-0.768373068603
-0.565862825972	-0.7682945517
-0.5657748125	-0.768259117973
-0.565683943009	-0.768093604037
-0.565585318691	-0.768091734498
-0.565494151758	-0.768071031558
-0.565381963855	-0.768056697617
-0.565281766788	-0.76791002382
-0.565191177769	-0.767610311365
-0.565094226452	-0.767548195854
-0.565011537614	-0.767533532622
-0.564917729565	-0.767505583639
-0.564829402673	-0.767419550163
-0.564735916125	-0.767351026913
-0.564644382303	-0.76728176605
-0.564532167925	-0.767236293884
-0.564443357067	-0.767200628494
-0.564342721562	-0.767174873932
-0.564259172495	-0.767158589047
-0.56416890237	-0.76692406499
-0.564079762736	-0.766832526759
-0.563986858369	-0.766816271727
-0.563878968893	-0.766701619224
-0.563786119203	-0.76664658857
-0.563696377513	-0.766560402697
-0.563598255045	-0.76653633733
-0.563504561652	-0.766518197577
-0.56341170453	-0.766348175062
-0.563319049612	-0.766346793027
-0.563228071702	-0.76626386894
-0.563137194858	-0.766137126311
-0.563051819282	-0.766072632898
-0.56297581395	-0.76601910231
-0.562883443712	-0.765834956963
-0.5627931605	-0.765730750704
-0.562694190162	-0.765724801028
-0.562604292945	-0.765646507585
-0.562507809361	-0.765463945683
-0.562414173431	-0.765462152915
-0.562314760386	-0.765399009331
-0.56219295104	-0.765322505908
-0.562108758274	-0.765246339239

-0.562018740952	-0.765057833756
-0.561931167289	-0.765041523441
-0.56183813433	-0.765036575089
-0.561741502263	-0.76495373091
-0.561641321136	-0.76488098525
-0.561549562739	-0.764752428453
-0.561449582328	-0.764414302812
-0.56134958636	-0.764283800074
-0.561261928348	-0.76421070899
-0.561167508603	-0.764087388351
-0.561068506105	-0.764064611777
-0.560975266435	-0.764028242241
-0.560880817305	-0.76401382089
-0.560776220973	-0.763831593307
-0.560696553564	-0.763779061459
-0.560594323873	-0.763726490717
-0.560485003297	-0.763665608959
-0.560395308309	-0.763616425962
-0.560298782266	-0.763551862022
-0.560210269578	-0.763378756938
-0.560097320327	-0.763185762075
-0.560010170947	-0.762945446362
-0.55992428453	-0.762826688551
-0.559822793156	-0.762812436092
-0.559725939961	-0.762655541206
-0.55962256158	-0.762477027789
-0.559524062652	-0.762477014596
-0.559417912126	-0.762466380733
-0.5593090714	-0.762235879192
-0.559228550292	-0.762054355447
-0.559144653801	-0.761969397672
-0.5590548727	-0.761959873774
-0.55896174169	-0.761929884368
-0.558863066768	-0.761546577233
-0.558770465439	-0.76126543749
-0.55864639947	-0.761092168502
-0.558540512932	-0.761020806172
-0.558447828131	-0.760868920976
-0.558357252872	-0.760779827794
-0.558257238348	-0.760701142011
-0.558144299459	-0.760691636162
-0.558055966193	-0.760475492727
-0.55795418688	-0.760362171938
-0.557843237064	-0.760219027852
-0.557732145299	-0.760138834671
-0.557649005633	-0.760057603811
-0.557560279254	-0.760040093829
-0.557469681629	-0.760028430121
-0.557379971078	-0.759914290291
-0.55726975202	-0.759599737017
-0.557177721811	-0.759522577775
-0.55707957906	-0.75938862532
-0.556984852349	-0.759296686963
-0.556888191128	-0.75907280061

-0.556781248133	-0.758791880953
-0.556708562286	-0.758651932898
-0.556623516317	-0.758457319026
-0.556533123426	-0.758265411765
-0.556442688002	-0.758157280653
-0.556364685459	-0.758142013229
-0.556282442876	-0.758136302634
-0.556170454637	-0.758060340451
-0.556064280582	-0.758040532619
-0.555970173946	-0.758013688406
-0.555882552483	-0.757892534032
-0.555797570095	-0.757758280637
-0.555713241504	-0.757755330577
-0.555628640292	-0.757658501228
-0.555517254634	-0.75758926667
-0.555422848587	-0.757475343582
-0.555334434195	-0.757315744015
-0.555231548953	-0.757179375889
-0.555147814065	-0.757150938531
-0.555070751456	-0.756949719443
-0.554972513463	-0.756888465424
-0.55487756503	-0.756742909128
-0.554777836066	-0.75665774785
-0.554680218212	-0.756484541036
-0.554592855722	-0.756460623288
-0.554506591554	-0.75616056982
-0.554411649076	-0.756159841241
-0.554310227501	-0.75602706947
-0.554207748018	-0.755943388699
-0.554107937325	-0.7559278806
-0.554014246244	-0.755655446725
-0.553920612086	-0.7555525848
-0.553837316684	-0.755436249824
-0.553744957849	-0.755319579669
-0.553644498285	-0.755280126665
-0.553549609076	-0.755155570016
-0.553468634219	-0.755147003905
-0.553370854444	-0.755097318475
-0.553271025076	-0.75504314222
-0.553179358327	-0.754764206793
-0.553062973708	-0.754732144541
-0.55298315586	-0.754681241397
-0.552896893266	-0.754607846661
-0.552783681716	-0.754277538903
-0.552684680266	-0.754157151748
-0.552582694515	-0.753800194736
-0.55247773203	-0.75372720186
-0.552374915774	-0.753724570751
-0.55227449868	-0.753575886753
-0.552180644085	-0.753483752496
-0.552097643251	-0.753387970659
-0.55200292428	-0.753356404451
-0.55190572473	-0.753100051836
-0.551818750416	-0.752893022775

-0.551732933945	-0.752888367378
-0.551632735434	-0.752812870008
-0.551553277371	-0.752400451654
-0.551455789728	-0.751775541883
-0.551361885536	-0.751764562923
-0.55127221374	-0.751742140935
-0.551193120156	-0.7514668936
-0.551104095917	-0.751163116716
-0.551006660069	-0.751120610345
-0.550906568159	-0.751120067855
-0.550816877652	-0.750959430116
-0.550738725656	-0.750932243296
-0.550644808013	-0.750748269378
-0.55053431692	-0.750666164355
-0.550430129847	-0.750624828453
-0.55033449577	-0.750575351629
-0.550242334464	-0.750567779213
-0.550148137157	-0.750551476393
-0.550056437521	-0.750302478326
-0.549960219057	-0.750122165701
-0.549847902058	-0.750037157697
-0.549765501213	-0.749878171086
-0.549678148385	-0.749621918026
-0.549581195795	-0.749527404191
-0.549480835649	-0.749511672981
-0.549390564939	-0.749205063985
-0.549304133	-0.749013581983
-0.549217215189	-0.748930918664
-0.549114829709	-0.748878290447
-0.549012455547	-0.748795270552
-0.548908677651	-0.748607658842
-0.548798977525	-0.74826958527
-0.54871496916	-0.748178689864
-0.54863003138	-0.748160914078
-0.548538870244	-0.748058266841
-0.548433746641	-0.747926833745
-0.548340875519	-0.747793885968
-0.548243901596	-0.747716551157
-0.548146505926	-0.747666312279
-0.548052497426	-0.747664023683
-0.547955723837	-0.74765069154
-0.547861612535	-0.747633556682
-0.547744453063	-0.747629527355
-0.547655768191	-0.747538094048
-0.547549839278	-0.747455302683
-0.547453592241	-0.747378278562
-0.547352772128	-0.747283414412
-0.547251228452	-0.747251038073
-0.547146050279	-0.747230046562
-0.54706708063	-0.747177717148
-0.546972392521	-0.747070440797
-0.546888371491	-0.747043127833
-0.546795039541	-0.747004935591
-0.546699827716	-0.746974585567

-0.546605761551	-0.746580971552
-0.546527378968	-0.746540190107
-0.546430053944	-0.746494654698
-0.546344106548	-0.746189510195
-0.54626068471	-0.746106086384
-0.546142250219	-0.746003451975
-0.546029812373	-0.745939591962
-0.545923569136	-0.745886082644
-0.545824703071	-0.745657200049
-0.545723189516	-0.745581396973
-0.545632193073	-0.745572472531
-0.545537086919	-0.745530429469
-0.545463594093	-0.745522879305
-0.545377007023	-0.74549589021
-0.545292599601	-0.745107059801
-0.545200672686	-0.745073530902
-0.545100962993	-0.745063932382
-0.545016026028	-0.744961137673
-0.544939179949	-0.74487316769
-0.544848427229	-0.744813543888
-0.544753785547	-0.744546957019
-0.544669905641	-0.744338206674
-0.544569770036	-0.744031536492
-0.54449416458	-0.744025347333
-0.544406059473	-0.744025021126
-0.544302462431	-0.74397748641
-0.544204636275	-0.743941038757
-0.544115768172	-0.743449980028
-0.544024543507	-0.74340559637
-0.543911408947	-0.74335857026
-0.543826660166	-0.743335102096
-0.543733968412	-0.743058495457
-0.543624307209	-0.7429034502
-0.543507791415	-0.742798700844
-0.543420976613	-0.742772705684
-0.543326892261	-0.742745274902
-0.543233030906	-0.742280510932
-0.543129487137	-0.742257356343
-0.543031878077	-0.742232864657
-0.542951841657	-0.742222005157
-0.542862732089	-0.742206808557
-0.542776353861	-0.742139870074
-0.542681085088	-0.742082101879
-0.542592476238	-0.742005608598
-0.542507908248	-0.741970280483
-0.542395332884	-0.741656558626
-0.542311121899	-0.741649362828
-0.542230582666	-0.741629846194
-0.542146807449	-0.741566120963
-0.542037625062	-0.741362422728
-0.541934302515	-0.741261164918
-0.541830971254	-0.741154000868
-0.541725424688	-0.74093335316
-0.541624625401	-0.740865307089

-0.541538319951	-0.740846560709
-0.541446096561	-0.740844491904
-0.541353307454	-0.740745541952
-0.541258683002	-0.740617411739
-0.541157802924	-0.740590291677
-0.541069275532	-0.740547512347
-0.540984348417	-0.74044718354
-0.540891590298	-0.740342118492
-0.540806249046	-0.740088742792
-0.540727238632	-0.740053149645
-0.540625219824	-0.739688119915
-0.540535205758	-0.73959956262
-0.540440516523	-0.739594905828
-0.540360087032	-0.739521885018
-0.540264851566	-0.739496741013
-0.540169112714	-0.739494565189
-0.540079288975	-0.739438114213
-0.539991647103	-0.73940425988
-0.539891428849	-0.73932576211
-0.53981253488	-0.739230171935
-0.539715648819	-0.739152605272
-0.539612605431	-0.739052552433
-0.539518988974	-0.738970464637
-0.539388726419	-0.73891346472
-0.539290747423	-0.73891053466
-0.539214419858	-0.738760512652
-0.539132146232	-0.738737940687
-0.53905378931	-0.738630603919
-0.538966074577	-0.73851606956
-0.538864961134	-0.738505648074
-0.538775578361	-0.738476232178
-0.538696477937	-0.73829453053
-0.538600493637	-0.738151625024
-0.53849046238	-0.73814803621
-0.538380142982	-0.738069366665
-0.538281815344	-0.737980532955
-0.538202303571	-0.737897561852
-0.538107829737	-0.737855783013
-0.538020927949	-0.737789645683
-0.537926341751	-0.737745036103
-0.53781099795	-0.737646171369
-0.537721120914	-0.736835303285
-0.537630775982	-0.736560612622
-0.537553321662	-0.736495085375
-0.537462641651	-0.736356306785
-0.537382675695	-0.736347474204
-0.537287511631	-0.736158980621
-0.537200178318	-0.735982742659
-0.537102115882	-0.735957656653
-0.536985882369	-0.735871746497
-0.536898623286	-0.735642458541
-0.536820140506	-0.735377162333
-0.536732872221	-0.735331705309
-0.536635609231	-0.735217880269

-0.536553277032	-0.73513047385
-0.536442747573	-0.735075448768
-0.53634960932	-0.735018084461
-0.536268583966	-0.73500798997
-0.536186118177	-0.734756516605
-0.536119036373	-0.734421976018
-0.536031262656	-0.734167596338
-0.535937540674	-0.734084532335
-0.535846467911	-0.733992402927
-0.535755734703	-0.733828593211
-0.535660371001	-0.733803181942
-0.535564238215	-0.733797329215
-0.535470159841	-0.733678868665
-0.535388210351	-0.733635232314
-0.535303835118	-0.733286346962
-0.535210016094	-0.733181614141
-0.535106631249	-0.733047079002
-0.535018538017	-0.732961635124
-0.534938351215	-0.732857917274
-0.534850811554	-0.732828516938
-0.534749900803	-0.732802896266
-0.534658125665	-0.732787374935
-0.534562336601	-0.732704165412
-0.534471512156	-0.732507060351
-0.534362584576	-0.732450220544
-0.534261034966	-0.732446265858
-0.534167473128	-0.732416598496
-0.534063143479	-0.732386547677
-0.533990258276	-0.732297663569
-0.533904684955	-0.732253204958
-0.533814244384	-0.732171713174
-0.53370772368	-0.732110197643
-0.533615055653	-0.732106707663
-0.533531689228	-0.732041593853
-0.533434396145	-0.731992351973
-0.533348397137	-0.731936286692
-0.533262651621	-0.731781338918
-0.533178743306	-0.731669171038
-0.53307658776	-0.731651261606
-0.533001572096	-0.731594793093
-0.532901516125	-0.73145744266
-0.532813542716	-0.731353021014
-0.532733446387	-0.731350885996
-0.532628865313	-0.731298109234
-0.532527591558	-0.731277813117
-0.53243611525	-0.7311932558
-0.532333641817	-0.73116174735
-0.532218930372	-0.731157405313
-0.532128834885	-0.731074735512
-0.532036795554	-0.730743670931
-0.531934811648	-0.730557135502
-0.531848836912	-0.730506774253
-0.531760946575	-0.730199795086
-0.531680758976	-0.730017056699

-0.531589305573	-0.72997278324
-0.531460814542	-0.729962752495
-0.531368157147	-0.729832260074
-0.531279740536	-0.729810287638
-0.531196472228	-0.729788790496
-0.531106200273	-0.72978303372
-0.531005282846	-0.729653080574
-0.530918537798	-0.7296419256
-0.530838724459	-0.729633060853
-0.530758071199	-0.729631414673
-0.53067288118	-0.729629755598
-0.530584511029	-0.729455178977
-0.530496942737	-0.729317966428
-0.530403663254	-0.729233687134
-0.53031857498	-0.729192027339
-0.530225619512	-0.729150082614
-0.530125673341	-0.729124013877
-0.530024603113	-0.728983284461
-0.529939038628	-0.728904888796
-0.52985019649	-0.72883919426
-0.529765331119	-0.728807228967
-0.52967631346	-0.72878439003
-0.529582508024	-0.728770961789
-0.529480043744	-0.728760188825
-0.529376379738	-0.728679145727
-0.529264977354	-0.728659363875
-0.529182219153	-0.728610509182
-0.529085283712	-0.728463240777
-0.528989045847	-0.728344202544
-0.528907022781	-0.728241195841
-0.528818002714	-0.728226970729
-0.528728941442	-0.72822272834
-0.528638861821	-0.728194830931
-0.528557992547	-0.728180991717
-0.528448477985	-0.728146431803
-0.528352211959	-0.728106761198
-0.528268271657	-0.728082906348
-0.528171124251	-0.728063712213
-0.528072509675	-0.727847160952
-0.527983579439	-0.72782382687
-0.527901546075	-0.727748492256
-0.527816679958	-0.727702668459
-0.527719562017	-0.727549548974
-0.527630759803	-0.727544681461
-0.527553992931	-0.72743977384
-0.52746873576	-0.727387020374
-0.52736747658	-0.7273650846
-0.527293558362	-0.727330742469
-0.527189523748	-0.727315563197
-0.527088970119	-0.726598207311
-0.526997007194	-0.726572289567
-0.526879985722	-0.726491442128
-0.526787679481	-0.726417056495
-0.526668786816	-0.726337655061

-0.526588491197	-0.726288875999
-0.52648225875	-0.726245532076
-0.526396239742	-0.726205121496
-0.526301495633	-0.726202475086
-0.526214920147	-0.726153530027
-0.526138500757	-0.726011119312
-0.526044249103	-0.725929954448
-0.525956663835	-0.725724880513
-0.525855629226	-0.725527689923
-0.525759009547	-0.725462722188
-0.525680795627	-0.725389104874
-0.525573271048	-0.725099099689
-0.525458022758	-0.725098676353
-0.525368645909	-0.724928535693
-0.525267039854	-0.72477250067
-0.525158777752	-0.724640182143
-0.525066326065	-0.724371427974
-0.524963280649	-0.724289461058
-0.524900321765	-0.724185903899
-0.52480619625	-0.724047036365
-0.524728510703	-0.723885095789
-0.524649071047	-0.723685436343
-0.524551657216	-0.723671156376
-0.524452179692	-0.723668543356
-0.524370588881	-0.723476655418
-0.524268247545	-0.723334809631
-0.52416553072	-0.723151530001
-0.524080332247	-0.723114402821
-0.523983792633	-0.723046304594
-0.523888447309	-0.722893934872
-0.523798466895	-0.722891464032
-0.523719935487	-0.722815036459
-0.523589149471	-0.722691378916
-0.523501727043	-0.722653749451
-0.523409483456	-0.722490142455
-0.523319782282	-0.722348823228
-0.523227062164	-0.722325571656
-0.523131217284	-0.722304888426
-0.523033388378	-0.72229979577
-0.522929677987	-0.722295710965
-0.52285236043	-0.722215442974
-0.52276412536	-0.722177960853
-0.52267073565	-0.722177230695
-0.522580259573	-0.722157899792
-0.522481390099	-0.721933153258
-0.522404044476	-0.72190753893
-0.522317434819	-0.721898833095
-0.522242046872	-0.721766026622
-0.522138198458	-0.721439414827
-0.522047959693	-0.721377656529
-0.521925373349	-0.721071186606
-0.521836141392	-0.721047074267
-0.521724385605	-0.721037237486
-0.521645219061	-0.720994182275

-0.521575481658	-0.720933038224
-0.521489985904	-0.720831373863
-0.521392449937	-0.720630088279
-0.52130109457	-0.720628131783
-0.521201114652	-0.720555645752
-0.521105750517	-0.720547485544
-0.521002416657	-0.720407940103
-0.520915041556	-0.720385914806
-0.520821585758	-0.720254438203
-0.520737141178	-0.720076129976
-0.520646722976	-0.720066074303
-0.520539901029	-0.72005109561
-0.520437492772	-0.720043596697
-0.520331578081	-0.719983290221
-0.520223362934	-0.719834579319
-0.520142659084	-0.719787137765
-0.520037585102	-0.719778177872
-0.519942191889	-0.719716513273
-0.519843488399	-0.719475495222
-0.519732407462	-0.719373205558
-0.519638873184	-0.71928788471
-0.519547100042	-0.719235679689
-0.51945623583	-0.719171817851
-0.519366336393	-0.718966587155
-0.519276157166	-0.718924295244
-0.519179672704	-0.718898814968
-0.51909768514	-0.718835357675
-0.519014399841	-0.718583653117
-0.518917942912	-0.718450921612
-0.518834728482	-0.718354119056
-0.518756756601	-0.718289937227
-0.518665219464	-0.718187348724
-0.518585367661	-0.718140302652
-0.518482874717	-0.718124129158
-0.518396766157	-0.718105154812
-0.51830311229	-0.718026255569
-0.518206301598	-0.717414470064
-0.518113331213	-0.717335965312
-0.518013318828	-0.717235260732
-0.517925430133	-0.717186683705
-0.517839231277	-0.717011451923
-0.517757822364	-0.716851059609
-0.517647329199	-0.716839484094
-0.517572282886	-0.716717771849
-0.517481281203	-0.716630467828
-0.517387279976	-0.716556390572
-0.517271375201	-0.716536011587
-0.517165377286	-0.716505892815
-0.51708235169	-0.716457364294
-0.516998045028	-0.716215086296
-0.516911045008	-0.716138062658
-0.516824260323	-0.716125740222
-0.516722857506	-0.716024987306
-0.516637451655	-0.715971478259

-0.516550745279	-0.715776685341
-0.516450328113	-0.715716249928
-0.516373196129	-0.71535747884
-0.516274794873	-0.715218545707
-0.51618977547	-0.715173610837
-0.516112637372	-0.715156626553
-0.516009731299	-0.715110522064
-0.515930451575	-0.715099747723
-0.51584169279	-0.715080989296
-0.515757429149	-0.714738463396
-0.515661527384	-0.714683910426
-0.515573327336	-0.714638344564
-0.51549313177	-0.714478287653
-0.515407006701	-0.714463143928
-0.515316879906	-0.714319399496
-0.515224545377	-0.714170181137
-0.515146864961	-0.714164352294
-0.515066824565	-0.714163710124
-0.514974088953	-0.713925430962
-0.514890300713	-0.713906114024
-0.514812031435	-0.713885746436
-0.514706634128	-0.713713865465
-0.514613403872	-0.713423426908
-0.514540585585	-0.713422888032
-0.514452607921	-0.713223069323
-0.514361742527	-0.713018807745
-0.514268627812	-0.712928480492
-0.51418070929	-0.712913465737
-0.514083468771	-0.712567345717
-0.51399562723	-0.712551546691
-0.513916021499	-0.712417404304
-0.513822228034	-0.712324379209
-0.513740735187	-0.712003146086
-0.513642998211	-0.711983359699
-0.513548431954	-0.711922362209
-0.513452171944	-0.71191041754
-0.513360290827	-0.711634721224
-0.513277657039	-0.711491955094
-0.513174635105	-0.711345214163
-0.513096988433	-0.711091927996
-0.513007511804	-0.711001713619
-0.512912311335	-0.710997428752
-0.512823579627	-0.710882102145
-0.512745283571	-0.710630286677
-0.51265595808	-0.710520998441
-0.512566835076	-0.710249929747
-0.512465694537	-0.71010352165
-0.512362270465	-0.710052672108
-0.512253885421	-0.710018328644
-0.512176138641	-0.709954631958
-0.512080690929	-0.709929865299
-0.511979496389	-0.709796756946
-0.511866546359	-0.709795112437
-0.511771302012	-0.709689857485

-0.511667056776	-0.709618060062
-0.511565117967	-0.709548471387
-0.51147490108	-0.709191928119
-0.51137396036	-0.709055507682
-0.51127245273	-0.7089488174
-0.511169739301	-0.70890823737
-0.511080765885	-0.708702281532
-0.510995511247	-0.70851181999
-0.510879079311	-0.708321463198
-0.510785495058	-0.708220457163
-0.510702077417	-0.707946174317
-0.510619525075	-0.707832614125
-0.510531997213	-0.707822347159
-0.510424648854	-0.70779423397
-0.510337213038	-0.70755842242
-0.510253359675	-0.707533550665
-0.510171386789	-0.707477280701
-0.510087385571	-0.707436991647
-0.510006627716	-0.707418447257
-0.50990213503	-0.707413351989
-0.509802476252	-0.707244817028
-0.50972124663	-0.70713862622
-0.509638693809	-0.707116803483
-0.509552594507	-0.707086244533
-0.509461162559	-0.706941052281
-0.509330122565	-0.706932699159
-0.50924752168	-0.706932157127
-0.509147639105	-0.706915908878
-0.509066622521	-0.706620726585
-0.50896981892	-0.706538188972
-0.508861565839	-0.70635573082
-0.508770046788	-0.706320330618
-0.508663458658	-0.706031861699
-0.508575954006	-0.70602760809
-0.508486217872	-0.705966088291
-0.508392524078	-0.705965221242
-0.508286254812	-0.705948671581
-0.508196629951	-0.705939285317
-0.508094820765	-0.705887478807
-0.507993815686	-0.705746863795
-0.507904359143	-0.705725347001
-0.507836554853	-0.705694825491
-0.507731815544	-0.705606067731
-0.507639040422	-0.705497024283
-0.507555813144	-0.705458126641
-0.50746813012	-0.705420474393
-0.507370205639	-0.705383465797
-0.507290131198	-0.704837430841
-0.50719458604	-0.70481804272
-0.507119954598	-0.704801688875
-0.507026523213	-0.704728228185
-0.506936317933	-0.704671610913
-0.506857748862	-0.70464846019
-0.506764924336	-0.704561059755

-0.506672365244	-0.704527769868
-0.506590167526	-0.704346204116
-0.506496729426	-0.704330771546
-0.506413959852	-0.704275729121
-0.50632992154	-0.704203769368
-0.50623001959	-0.704013984503
-0.506146625418	-0.703921966251
-0.506046807906	-0.703900646826
-0.505943378896	-0.703847448711
-0.505847317335	-0.703825784605
-0.505747071329	-0.703799103109
-0.505659515163	-0.703666680837
-0.50557719091	-0.70356807643
-0.505485294868	-0.703562484628
-0.505387102678	-0.703315274028
-0.505289372247	-0.703234312633
-0.505171884609	-0.702858229679
-0.505083641252	-0.702838953404
-0.505008039406	-0.702750583546
-0.504917031392	-0.702688079406
-0.504808558627	-0.702525610979
-0.504740144003	-0.70248777678
-0.504628031831	-0.702457007548
-0.50452515596	-0.702435261275
-0.5044317831	-0.702174563851
-0.504344176375	-0.70202402044
-0.504249044997	-0.702007660694
-0.504176369739	-0.701976770035
-0.504094141922	-0.701878366683
-0.504017365031	-0.701754496244
-0.503925511057	-0.701696920737
-0.503835874743	-0.701627689856
-0.503740371439	-0.701599373423
-0.503630875316	-0.70128585922
-0.503528697979	-0.701195236096
-0.503429747382	-0.701174997494
-0.503342654632	-0.701151118593
-0.503242742264	-0.701124764678
-0.503146223149	-0.701119015532
-0.503047918513	-0.700979566028
-0.502930795303	-0.700896741036
-0.502830456673	-0.700810124768
-0.502743604764	-0.700669302807
-0.502668919966	-0.70045063821
-0.50257755724	-0.70040378314
-0.50248032486	-0.700266636361
-0.502373071626	-0.700232432649
-0.502289431583	-0.700188263326
-0.50218286468	-0.70007958375
-0.502087326012	-0.70000247134
-0.501979668288	-0.699938990385
-0.501890142925	-0.699915217925
-0.501796155016	-0.699708297763
-0.501674005227	-0.69963334766

-0.50158482508	-0.699568717288
-0.501500449801	-0.699365598639
-0.501401173405	-0.699297982208
-0.501315690064	-0.699061553561
-0.501229658923	-0.699028454548
-0.501139704433	-0.698958347767
-0.501031644113	-0.698841849426
-0.50092885752	-0.698693054407
-0.500832573972	-0.698662870249
-0.500754618401	-0.69863257965
-0.500670767426	-0.698494166143
-0.500583948196	-0.698437157835
-0.500482744857	-0.698014070613
-0.500400224079	-0.697834169249
-0.500305291393	-0.697757002973
-0.50021010485	-0.697722625238
-0.500102667324	-0.697534599845
-0.500002783222	-0.697414881922
-0.499887854639	-0.697405982606
-0.499790908637	-0.697279670616
-0.499696074074	-0.697180021516
-0.499608880994	-0.697000678743
-0.49952519773	-0.696562886418
-0.499447114413	-0.696364447986
-0.499341736069	-0.696248822305
-0.49927295444	-0.696238178335
-0.499193566812	-0.696198258011
-0.499105059127	-0.696051136132
-0.498998799803	-0.695858113651
-0.498914011887	-0.695749497094
-0.498826371758	-0.69570527744
-0.498720978124	-0.695643159202
-0.498606008067	-0.69562062465
-0.498514860003	-0.695565264681
-0.498420142869	-0.69556050526
-0.498332921753	-0.695555570549
-0.498242330921	-0.69525177152
-0.498155157611	-0.695211404829
-0.498050658814	-0.695172916896
-0.497967471579	-0.695109443432
-0.49787801005	-0.695097263211
-0.497795231621	-0.695077057553
-0.497711435998	-0.694925399156
-0.497614740652	-0.694797145228
-0.497518840337	-0.694685707273
-0.497446269554	-0.694552026398
-0.497338486734	-0.694415482766
-0.497245123646	-0.694213756132
-0.497152015866	-0.694062111156
-0.497064918009	-0.693926040355
-0.496963084452	-0.693692072798
-0.496874970933	-0.693672655023
-0.496766961886	-0.693653211016
-0.496683036569	-0.693649691064

-0.49659651664	-0.693634684955
-0.49650776664	-0.693575452109
-0.496417670942	-0.693346561439
-0.496334464109	-0.693272816081
-0.496247109608	-0.69319164885
-0.496162432995	-0.693014278398
-0.496045666182	-0.692784681808
-0.495961266737	-0.692620956482
-0.495847238248	-0.692502973525
-0.495752860966	-0.692278064084
-0.495659399048	-0.69217802636
-0.495556820536	-0.692097181796
-0.495459695999	-0.692059830667
-0.495378129789	-0.691973565416
-0.49527846621	-0.69196639964
-0.495188226186	-0.691964177073
-0.495110179728	-0.691960464496
-0.495037787196	-0.691925278709
-0.494919505665	-0.691782604505
-0.494837549089	-0.691744904384
-0.49474600985	-0.691650166279
-0.49464895965	-0.691531645604
-0.494562562876	-0.691396200269
-0.494470024255	-0.691159047264
-0.494385966341	-0.691081568839
-0.494297320564	-0.691023917449
-0.494199126133	-0.69095163271
-0.494106283799	-0.690746452267
-0.494025958729	-0.690636954904
-0.493940460812	-0.690612309814
-0.493860149105	-0.690567927746
-0.493767357574	-0.690515807701
-0.493662434097	-0.690327224755
-0.493583541059	-0.690241245328
-0.493511234283	-0.690192942916
-0.493420927541	-0.690190423123
-0.49331124536	-0.689970593433
-0.493217126689	-0.689949975704
-0.49313888703	-0.68972324469
-0.4930311959	-0.689625933106
-0.492956629233	-0.689155801866
-0.492870016635	-0.689056136532
-0.492766333076	-0.688982322194
-0.492664918584	-0.688981051308
-0.492584637668	-0.688916282901
-0.492499857598	-0.688846777695
-0.492408215327	-0.688819129174
-0.492326183264	-0.688063605839
-0.492242973315	-0.68800709783
-0.492145333658	-0.687669106817
-0.49205816482	-0.687611530243
-0.491971965147	-0.687459800979
-0.491881396143	-0.68744082016
-0.491790274834	-0.687408171855

-0.491706400486	-0.687339777986
-0.491621377786	-0.687093643232
-0.49153267879	-0.686676941409
-0.491411710797	-0.686659607541
-0.491339795716	-0.686601711526
-0.491235321431	-0.686455883792
-0.49113640333	-0.686274799836
-0.491041274005	-0.686075955382
-0.490945894252	-0.686048194323
-0.490856398827	-0.685749470449
-0.490751296068	-0.685703426995
-0.490663687911	-0.685603125718
-0.490565858835	-0.685366330558
-0.490464652664	-0.685261660526
-0.490371051182	-0.685213898766
-0.49028949239	-0.685104597331
-0.490182966935	-0.684955302607
-0.490085833996	-0.684905363972
-0.489995154214	-0.684750617659
-0.489894347077	-0.684646438876
-0.489808294417	-0.68455270738
-0.489716659222	-0.684506332622
-0.48961357705	-0.684477007106
-0.489511646422	-0.684260645165
-0.489385580044	-0.684254307714
-0.489280238613	-0.684122133039
-0.48918083533	-0.684059034189
-0.489075133391	-0.683891318104
-0.488990932921	-0.683704843365
-0.488900009832	-0.683559543894
-0.488804148668	-0.683367429342
-0.488717257545	-0.683288825023
-0.488639840062	-0.6832878601
-0.488544844113	-0.68321417122
-0.488458025607	-0.683169397173
-0.488365735846	-0.683021457863
-0.488272602201	-0.682966594932
-0.488196219523	-0.68290890112
-0.488108224345	-0.682702630484
-0.488013285628	-0.682665565417
-0.487917405214	-0.682587976502
-0.487821348809	-0.682566116539
-0.487730824294	-0.682404997007
-0.487645980201	-0.682344201457
-0.487560688439	-0.682158489855
-0.487471645809	-0.682035816261
-0.487384284971	-0.681883766586
-0.487301599701	-0.681830711202
-0.487207964381	-0.681764438869
-0.487100151404	-0.681718965622
-0.487007032382	-0.681659650111
-0.486918966234	-0.681575552971
-0.486817855627	-0.681407328982
-0.486727915868	-0.681159539215

-0.48664204225	-0.681136159893
-0.486539688417	-0.681024188415
-0.486456345915	-0.680865414815
-0.486360301006	-0.680791123439
-0.486263933432	-0.680751651616
-0.486164123299	-0.680728081744
-0.486061583407	-0.680671319669
-0.485972993076	-0.680594955925
-0.485874122695	-0.680587909295
-0.485795206637	-0.680274509648
-0.485709561603	-0.680143027411
-0.485620217968	-0.679944362217
-0.48551805109	-0.679906720653
-0.485440192332	-0.679882016893
-0.485343082938	-0.679837519169
-0.485262043421	-0.679805040088
-0.485160683683	-0.679656875127
-0.485065731047	-0.679655376362
-0.48496421601	-0.679648937929
-0.484861725719	-0.679509597446
-0.484773790775	-0.679297079412
-0.484684262199	-0.679056884046
-0.484590868104	-0.678896359808
-0.484490188467	-0.678886918365
-0.484403604969	-0.678674159637
-0.48431488063	-0.67858677733
-0.484239580202	-0.678559087573
-0.484149363726	-0.678134386017
-0.484051711132	-0.677945935146
-0.483968153959	-0.677799223826
-0.483857601578	-0.677710529082
-0.483784367923	-0.677680522592
-0.483692968149	-0.677633990393
-0.483583818437	-0.67760984222
-0.483495797153	-0.677445514413
-0.483402224017	-0.677434305029
-0.483295040105	-0.677220157974
-0.48319612092	-0.677025830831
-0.483127874711	-0.676994153198
-0.48304374897	-0.676893947834
-0.482954697545	-0.676778672162
-0.482849242856	-0.676573825975
-0.482755101486	-0.676553154535
-0.48267916701	-0.676508900118
-0.482582968078	-0.676362864185
-0.482487586909	-0.676153373654
-0.482399527449	-0.67612509033
-0.482299712449	-0.675967488376
-0.482206837398	-0.675966155285
-0.482116538667	-0.675917569763
-0.48202725886	-0.675916806223
-0.481936749805	-0.675877779951
-0.481859254699	-0.675858118127
-0.481769598969	-0.675822700608

-0.481674799322	-0.675597751268
-0.481578915837	-0.675579465124
-0.481472169507	-0.675490274871
-0.481379626213	-0.674459354956
-0.481278210448	-0.674217009583
-0.481192309903	-0.674053242599
-0.481100812753	-0.674035365703
-0.480998320436	-0.673987262007
-0.480902750379	-0.673409737541
-0.480791816938	-0.673225783044
-0.4806982699	-0.673117324845
-0.480610687376	-0.673034462181
-0.480532115184	-0.673014629604
-0.48043214898	-0.672833436648
-0.4803329306	-0.672807916695
-0.480220903506	-0.672800240124
-0.480130056469	-0.672781959799
-0.480054245505	-0.672753036435
-0.479958944296	-0.67270486817
-0.479856423677	-0.67266216081
-0.47976899663	-0.672463127028
-0.47968727893	-0.672279448211
-0.479594621181	-0.672194832126
-0.479513082381	-0.671672903069
-0.47942085771	-0.671541755421
-0.479333080995	-0.671535877643
-0.479249007906	-0.671527281544
-0.479142856088	-0.671300572287
-0.479035572767	-0.671096342193
-0.478943776256	-0.670790004813
-0.478838135425	-0.670416982239
-0.478758759379	-0.670138376536
-0.478661309572	-0.670106906048
-0.478575251647	-0.670022978306
-0.47847504892	-0.669985104358
-0.478389783185	-0.669908158742
-0.478314767477	-0.669678533592
-0.478223710181	-0.669610025981
-0.478107285869	-0.669422776881
-0.478004179791	-0.669405770507
-0.477922880282	-0.669217483669
-0.477844787338	-0.669180375436
-0.477737367803	-0.669086009099
-0.477660521635	-0.668426254931
-0.477570089674	-0.668282575496
-0.477470270207	-0.668144850164
-0.477390412233	-0.668127967681
-0.477280642748	-0.668053559001
-0.477186797286	-0.668014396155
-0.477094110355	-0.668007589917
-0.477001190208	-0.667961004972
-0.476924887962	-0.667904366031
-0.476828303278	-0.667870586265
-0.476735332383	-0.667864165359

-0.476632676316	-0.667812508309
-0.476555300404	-0.667731075834
-0.476474818988	-0.667726538069
-0.476385934188	-0.667586220729
-0.476277639166	-0.667362090796
-0.476184523769	-0.667356832905
-0.47609577679	-0.667316523793
-0.4759923218	-0.667194751613
-0.475924296136	-0.667178392231
-0.475826209208	-0.667041383738
-0.475732680082	-0.666710091922
-0.475652364848	-0.666708027356
-0.475562533972	-0.666604195032
-0.475476995379	-0.666573434864
-0.475382554396	-0.666435104966
-0.475277079484	-0.666308008404
-0.47519979081	-0.666067660677
-0.475087822623	-0.665910974339
-0.474992775507	-0.665883692909
-0.474897004115	-0.665712650407
-0.474801291221	-0.665533504811
-0.474700056343	-0.665391157122
-0.474617943302	-0.665337121107
-0.474514074176	-0.664999509769
-0.474415930193	-0.664985929809
-0.474304482611	-0.664695635088
-0.474223034678	-0.664622076919
-0.474128369748	-0.664569119551
-0.474031839287	-0.664500982736
-0.473950801332	-0.664498783529
-0.473847157117	-0.664483655153
-0.473751262114	-0.66442610986
-0.4736740752	-0.66413156626
-0.473578562645	-0.664006379837
-0.473497592929	-0.663925411058
-0.473386926841	-0.663766566384
-0.473297989278	-0.66355180727
-0.473205864483	-0.663236172967
-0.473105719349	-0.663202015312
-0.473020052136	-0.663089907881
-0.472922397494	-0.662985247045
-0.472819104287	-0.662939219051
-0.472734772296	-0.662920813616
-0.472642978513	-0.662766014071
-0.472557654241	-0.662690971601
-0.472467051009	-0.66254583159
-0.472366568284	-0.662530466182
-0.472289723438	-0.662432059349
-0.472208825041	-0.662429939254
-0.472128021042	-0.662426501166
-0.472040603856	-0.66235329724
-0.471950091084	-0.66233878473
-0.471846143525	-0.662334100124
-0.471769988618	-0.662317835489

-0.471680498751	-0.662215707139
-0.471570161405	-0.662155939397
-0.47148224062	-0.662003676442
-0.471383473135	-0.661995524135
-0.471297698943	-0.661952497289
-0.47120688633	-0.661951580477
-0.471121990129	-0.66188652744
-0.471025158295	-0.661793895344
-0.470935789815	-0.661787016962
-0.470834938724	-0.661762480674
-0.470743427893	-0.661663007127
-0.470636025974	-0.661561446039
-0.470540384429	-0.661425535002
-0.47044776355	-0.661344725477
-0.470354607491	-0.661313121567
-0.470276016505	-0.661097064192
-0.470200366211	-0.660821272491
-0.470095556552	-0.660790880145
-0.470010758234	-0.660757769092
-0.469928148363	-0.660647636942
-0.469846652796	-0.660427376449
-0.469753818538	-0.660372458822
-0.469652326427	-0.660122086611
-0.469562676562	-0.659980733941
-0.469454152243	-0.659832181695
-0.469350180843	-0.659808018802
-0.469258357077	-0.659652235942
-0.469161188408	-0.659609660878
-0.469077741946	-0.659458076115
-0.468995873334	-0.65923427092
-0.468921101814	-0.659232526933
-0.468838249172	-0.659043944931
-0.468751307807	-0.658803971096
-0.468658985659	-0.658794157589
-0.468572935394	-0.658731292683
-0.468486619078	-0.658728751087
-0.46840302635	-0.658695441695
-0.468284132169	-0.658618612356
-0.468191409168	-0.658614366823
-0.468108409033	-0.658506657646
-0.468033347877	-0.65845220959
-0.467939602279	-0.658405117423
-0.467835867743	-0.658264230976
-0.467744187454	-0.658249972131
-0.467643360971	-0.658085193055
-0.467559948023	-0.658032056961
-0.467471119649	-0.657956810562
-0.467384113604	-0.657901415516
-0.467284232718	-0.657757130705
-0.467197722283	-0.657701650665
-0.46710421521	-0.657668051459
-0.467028134428	-0.657417217027
-0.466917106167	-0.65738725338
-0.466814545724	-0.657318543109

-0.466731923158	-0.657318351763
-0.466645677443	-0.657314294351
-0.466551455874	-0.657091606011
-0.466468344212	-0.656910918487
-0.46637788414	-0.656682152836
-0.466281148922	-0.656451105793
-0.466194930642	-0.656172779429
-0.46607911405	-0.655940556543
-0.465977431227	-0.655655958294
-0.465880650367	-0.655466089002
-0.465794067933	-0.655456970017
-0.465729330961	-0.655274475408
-0.465643411167	-0.654965806887
-0.465542183471	-0.654740876388
-0.465441875441	-0.654519260702
-0.46533926713	-0.654456993098
-0.465250071522	-0.654223391651
-0.46516933466	-0.65411289446
-0.465086397802	-0.654013281289
-0.464979825561	-0.653936472553
-0.464906164296	-0.65391612405
-0.464808151911	-0.653753376893
-0.464734411887	-0.653656161644
-0.464651109072	-0.653575385551
-0.464553901512	-0.653547649255
-0.464460664279	-0.653498042384
-0.464381801671	-0.653453800676
-0.464279721649	-0.653404997473
-0.46420228967	-0.65336698205
-0.464120522395	-0.653320014171
-0.464040539118	-0.653303681148
-0.463964695335	-0.653075037333
-0.463857793205	-0.653025040386
-0.463779843268	-0.652977484519
-0.463690663353	-0.652939468655
-0.463609831446	-0.652923044487
-0.463523172844	-0.652861313501
-0.46343722527	-0.65281321888
-0.463336328308	-0.652752846456
-0.463234845904	-0.652426506893
-0.463140590133	-0.652088418784
-0.463061618948	-0.652048048305
-0.462965004549	-0.651987814724
-0.462877864033	-0.651768793398
-0.462772601097	-0.651761463555
-0.462677210708	-0.651750791194
-0.462588348685	-0.651477579373
-0.462497542903	-0.651461408776
-0.46239651432	-0.651415501683
-0.462284965683	-0.651337450039
-0.462204232223	-0.6509522335
-0.462105999353	-0.650934637847
-0.46201108078	-0.650863278234
-0.461915261966	-0.650489811979

-0.461813500823	-0.65048412308
-0.46170752854	-0.650459846563
-0.461607639286	-0.650431560967
-0.461514400114	-0.650234006194
-0.46143274475	-0.649859718633
-0.46135459882	-0.649806016743
-0.461262198204	-0.649724225435
-0.461185224244	-0.649155192538
-0.461103091166	-0.648968716754
-0.461008642779	-0.648790179653
-0.46090267737	-0.648753783875
-0.460820396548	-0.64857573476
-0.460749663496	-0.648436722767
-0.460661923479	-0.648407410247
-0.460573913949	-0.648140287632
-0.460483710314	-0.648008555361
-0.460370194119	-0.647893298231
-0.460266154771	-0.647773949622
-0.460183473624	-0.647355061325
-0.460086587645	-0.647320100366
-0.460001998484	-0.647285325671
-0.459908208296	-0.647209923452
-0.45982470965	-0.647009999834
-0.459731553699	-0.646903921353
-0.459638420786	-0.646878383921
-0.459530221518	-0.646854944024
-0.459443655105	-0.646690555843
-0.45936929327	-0.646605194404
-0.459298601944	-0.646537098101
-0.459204516853	-0.646477625002
-0.459094610886	-0.646475208038
-0.459002281293	-0.646466040265
-0.458923531436	-0.646425114976
-0.458839803552	-0.646408912093
-0.458744006983	-0.646359284606
-0.458664667364	-0.646073144453
-0.458587458722	-0.646017732455
-0.458498660862	-0.645941350548
-0.458400432402	-0.645667127576
-0.458292640378	-0.645666893338
-0.458202555202	-0.645480614077
-0.458119869633	-0.645412143787
-0.458041572076	-0.64507877727
-0.457963626455	-0.644873927789
-0.457863634314	-0.644863584626
-0.45777405875	-0.644831627094
-0.45769539711	-0.644807436825
-0.457608943309	-0.644716162164
-0.457508194321	-0.644626753947
-0.457425017979	-0.644518988014
-0.457326041822	-0.644460011799
-0.457229166029	-0.644450773467
-0.457156165669	-0.644359353476
-0.457053126145	-0.64424451982

-0.456964942904	-0.644225789861
-0.456878300168	-0.644160348789
-0.456794714876	-0.644087292805
-0.456706129608	-0.644085123675
-0.456636200915	-0.643968996742
-0.456543936105	-0.643838341648
-0.456465964952	-0.643485061551
-0.456374471707	-0.64336804196
-0.456301858131	-0.643293979077
-0.456216443471	-0.643141100419
-0.456109093848	-0.642976859263
-0.456007477888	-0.642968302226
-0.455920152476	-0.642617417789
-0.455826927333	-0.642392609723
-0.455745609	-0.642376959252
-0.455670694668	-0.642344111352
-0.455594225252	-0.642311891989
-0.455517011877	-0.64222065226
-0.45542284495	-0.641907134861
-0.455322157089	-0.641791524426
-0.455220321032	-0.641747194708
-0.45512119941	-0.641739864942
-0.455028908861	-0.641707518399
-0.454925907887	-0.641666377996
-0.454830944435	-0.641633789582
-0.454734095682	-0.641567749428
-0.454638827787	-0.641511280269
-0.454537039569	-0.641261760528
-0.454446752314	-0.641156036817
-0.454347373368	-0.641061413773
-0.454224141644	-0.641002777914
-0.454136958166	-0.64095127073
-0.454040925192	-0.640768949464
-0.453960396936	-0.640711909951
-0.453865127049	-0.64062506445
-0.453778039379	-0.640602929915
-0.453686043675	-0.6405738994
-0.453602153987	-0.640571424624
-0.453502189133	-0.64055393036
-0.453415330011	-0.640416539365
-0.453325051011	-0.640416149787
-0.453230542999	-0.640314597332
-0.453135114332	-0.640266678636
-0.453049183349	-0.640129616628
-0.452951200212	-0.640016558728
-0.452868334701	-0.639938461352
-0.452791068625	-0.639928104445
-0.452712380795	-0.639836056086
-0.452622116625	-0.639607612883
-0.452552310752	-0.639589509771
-0.452448023932	-0.639537208454
-0.452365459389	-0.639491170608
-0.452267552321	-0.639329088268
-0.452177233391	-0.639271775152

-0.452084616993	-0.639240713852
-0.452007448536	-0.639232862105
-0.451909206639	-0.639025846167
-0.45182415107	-0.638970893519
-0.451727742188	-0.638947718424
-0.451634200668	-0.638806412498
-0.451545340529	-0.638741665918
-0.451462600597	-0.638725712005
-0.451373750096	-0.638632318074
-0.451290676147	-0.638499811409
-0.45119647461	-0.638414228859
-0.451106167448	-0.638207575824
-0.451021361715	-0.63812207948
-0.450955127567	-0.637869096777
-0.45087623409	-0.637728582993
-0.450768419518	-0.637703184418
-0.450677565392	-0.637662437312
-0.450592794266	-0.637628651747
-0.450500532275	-0.637610410635
-0.450404153412	-0.637469086944
-0.450300557885	-0.637412788777
-0.450176197093	-0.637186478949
-0.450094122425	-0.637174188899
-0.450004398202	-0.637127248369
-0.44992115187	-0.636997800797
-0.449835058175	-0.636945299407
-0.449736365261	-0.636711304796
-0.449636040471	-0.636633986837
-0.449546582734	-0.636605017979
-0.449455299735	-0.636602620494
-0.449363149	-0.63651077136
-0.449276276653	-0.636333864894
-0.449179550434	-0.636194630803
-0.449093583215	-0.635951218961
-0.44900848944	-0.635900466732
-0.44890574607	-0.635732067897
-0.448833123856	-0.635582004381
-0.448753282985	-0.635535830728
-0.448668866369	-0.635513891226
-0.448581431119	-0.63547869238
-0.448474848926	-0.635466873508
-0.448380695108	-0.635256195172
-0.448261920615	-0.635071495709
-0.448177365546	-0.635034353042
-0.448083709098	-0.634991355411
-0.447997360079	-0.634949865303
-0.447912973316	-0.634631690276
-0.447823321678	-0.634560628362
-0.447738586789	-0.634484879806
-0.44761854641	-0.634420891087
-0.447520506947	-0.634343708975
-0.447429785088	-0.634322034223
-0.447335391361	-0.634248511403
-0.447240467629	-0.634238841966

-0.447159655884	-0.634225993975
-0.447052691559	-0.633872575733
-0.446966779937	-0.633709654162
-0.446872997352	-0.633629059392
-0.446784769261	-0.633626820797
-0.446691260771	-0.633600090145
-0.446584058535	-0.633537748803
-0.446504389669	-0.633248386955
-0.446420361172	-0.632939585692
-0.446316261306	-0.632901406325
-0.446212731703	-0.632797580928
-0.446130863754	-0.632493762635
-0.446046578958	-0.632461073318
-0.445958569658	-0.632416827125
-0.445875070674	-0.632387999356
-0.445776804984	-0.632360260376
-0.445671958695	-0.632212730325
-0.445576435756	-0.632080155531
-0.445494762598	-0.631880218132
-0.445414380782	-0.631680519546
-0.445347342377	-0.631503071332
-0.445245141729	-0.631356751364
-0.445160134432	-0.631258995432
-0.445067519361	-0.631214933798
-0.444968537411	-0.631023348169
-0.444889359578	-0.630958776483
-0.444803145459	-0.630837644256
-0.444709268591	-0.630757817092
-0.444614736041	-0.630734095135
-0.44453456525	-0.630644625971
-0.444439770635	-0.630560656013
-0.444350067854	-0.630335937512
-0.444252543438	-0.630075607862
-0.444169142607	-0.630001392991
-0.444068571059	-0.629998465064
-0.443981429745	-0.629844097997
-0.443885584909	-0.629644268447
-0.443796485496	-0.629612657715
-0.443676232515	-0.629521778984
-0.443570164019	-0.629234772624
-0.443484931211	-0.629146966171
-0.443399020179	-0.629124586261
-0.443313123447	-0.629063967365
-0.443243431276	-0.629062810917
-0.443156453993	-0.628804408813
-0.443055811991	-0.6287510682
-0.442959496324	-0.628704681818
-0.442870237463	-0.628589185412
-0.442766633437	-0.628571605483
-0.442675270655	-0.628526490239
-0.442570273184	-0.628361678022
-0.442492373161	-0.628040600527
-0.442396740764	-0.627736826235
-0.442317391002	-0.62773319618

-0.442243552648	-0.62772349499
-0.442142317942	-0.627723155338
-0.442067970791	-0.627651392551
-0.441977218243	-0.627451870639
-0.441885987005	-0.627406764472
-0.441795754526	-0.627218429123
-0.441690541492	-0.627010177145
-0.441598822954	-0.626852806143
-0.44151750697	-0.626801380769
-0.441426778388	-0.626739833912
-0.441337386452	-0.626709854843
-0.441241326236	-0.626331229138
-0.441170871502	-0.626319444255
-0.441086127985	-0.626301531305
-0.441006535534	-0.626291546849
-0.440931089313	-0.62615041476
-0.440849384527	-0.626148656522
-0.440745097198	-0.626098717981
-0.440661311394	-0.625995443096
-0.440574444919	-0.625847669411
-0.440466434366	-0.625463075819
-0.440366967628	-0.625114296118
-0.440279340191	-0.625095327692
-0.440200303569	-0.625068657938
-0.440091688776	-0.624961019339
-0.440003937351	-0.624949573267
-0.439925735302	-0.624934639941
-0.439807999495	-0.624860180892
-0.439731419482	-0.624827079594
-0.43964250525	-0.624810551149
-0.439557222329	-0.624693824259
-0.439457543475	-0.624543125692
-0.439368376556	-0.624372082854
-0.439279170225	-0.624320855862
-0.439190858914	-0.624124263774
-0.439100138763	-0.624060372228
-0.439002831511	-0.623996328234
-0.438910941298	-0.62397324815
-0.43882550015	-0.623857900641
-0.438743026576	-0.62382509432
-0.438634555469	-0.623683495625
-0.438537585512	-0.623537075472
-0.438463426684	-0.623507533975
-0.438387630129	-0.623329226629
-0.438300550681	-0.623267853085
-0.438210955324	-0.623214756484
-0.438108667062	-0.623157553802
-0.438033075318	-0.623106163955
-0.437948084848	-0.623055680787
-0.437868662232	-0.623009169914
-0.437788687897	-0.622894574258
-0.437693948034	-0.622798951998
-0.437602653418	-0.622710802924
-0.437526031197	-0.622666412713

-0.437451472696	-0.622624326201
-0.437361311597	-0.622547421409
-0.437273743325	-0.622515490888
-0.437176763579	-0.622439487796
-0.4371043574	-0.622079158576
-0.437027369782	-0.622001940264
-0.436918171319	-0.621993015105
-0.436819205218	-0.62167238047
-0.436717415693	-0.621611542645
-0.436600557371	-0.621536740127
-0.436522221531	-0.621286706679
-0.436429733852	-0.621043980071
-0.43634671692	-0.62092093252
-0.43626183924	-0.620781329995
-0.436156212681	-0.620776409849
-0.436061184073	-0.620282079156
-0.435976202012	-0.620279590211
-0.435896698587	-0.619984436476
-0.435799568026	-0.619945771591
-0.435713048525	-0.619927661205
-0.435631457342	-0.619790087539
-0.435546619013	-0.619789329809
-0.435456852219	-0.619625355448
-0.435392782843	-0.619581782231
-0.43531177794	-0.619493775238
-0.435251228145	-0.619443030774
-0.435165521143	-0.619406327905
-0.435076903066	-0.61929865778
-0.434974099901	-0.619064427467
-0.434901497593	-0.619032002161
-0.434811583263	-0.618977452592
-0.43472846075	-0.618926619849
-0.434631183394	-0.618817327867
-0.434547523	-0.618654584753
-0.434453684862	-0.618586068033
-0.434362214776	-0.618482623918
-0.434254891674	-0.618426507443
-0.43417583075	-0.61842464059
-0.434080735656	-0.6183993323
-0.433989706014	-0.61809182757
-0.433907063832	-0.617843263048
-0.433805618595	-0.617814069924
-0.433708653567	-0.617754702809
-0.433637401181	-0.617699326025
-0.433543408713	-0.617602876163
-0.433458295228	-0.617580657562
-0.433372768589	-0.617559514398
-0.43328493979	-0.617463299529
-0.433184883446	-0.617460698947
-0.433103979316	-0.617446258843
-0.432999238252	-0.617319749523
-0.432895714394	-0.617206723148
-0.432791025488	-0.617170560203
-0.432688001871	-0.617135133303

-0.432597328328	-0.617050662225
-0.432501355607	-0.616809920472
-0.432413476909	-0.616690175296
-0.432344004958	-0.616589031346
-0.432250808185	-0.616581849536
-0.432167643182	-0.616361631487
-0.432063424741	-0.616357251951
-0.431961369938	-0.616254274818
-0.431873848262	-0.616249971062
-0.431788159004	-0.616139280491
-0.431715425034	-0.616101004445
-0.431625198262	-0.616040829327
-0.431534635861	-0.6159749501
-0.431458604313	-0.615723660875
-0.431379059604	-0.61567263528
-0.431285301233	-0.615669292795
-0.431197681166	-0.615664220248
-0.43110287564	-0.615591705759
-0.431009133691	-0.615428710595
-0.430913344584	-0.615421296622
-0.430809709996	-0.615416910602
-0.43071514948	-0.615341291489
-0.430612118896	-0.615324459797
-0.430526101306	-0.615252884915
-0.430443642029	-0.615250672959
-0.430353878262	-0.615220285884
-0.430243663522	-0.615217277415
-0.430151426155	-0.615121929271
-0.430067810644	-0.61510735785
-0.429996990595	-0.615036738055
-0.429925834216	-0.614993984922
-0.429837896088	-0.614962823333
-0.429743131019	-0.614952387205
-0.429660886932	-0.614950738411
-0.429576999319	-0.614436053365
-0.429487278996	-0.614351923019
-0.429403359531	-0.61428477247
-0.429306255737	-0.614253159108
-0.429234777446	-0.614247118157
-0.429157601533	-0.61421825324
-0.429069721624	-0.614124875561
-0.428974201551	-0.61393595756
-0.428873681347	-0.613877371554
-0.428786412458	-0.613778192154
-0.428696618155	-0.613696717475
-0.42860810166	-0.613610453858
-0.428524232065	-0.613529606747
-0.428429969069	-0.613403750302
-0.428343548947	-0.613364460879
-0.428277306515	-0.613289719174
-0.4281956217	-0.613268361687
-0.428122965004	-0.613181046158
-0.428045893968	-0.612881169251
-0.427941606011	-0.612869112436

-0.427861558787	-0.612807892367
-0.427770100662	-0.612796166321
-0.427685529501	-0.612735639709
-0.427586369563	-0.612681004799
-0.427499577447	-0.612590676462
-0.427431055158	-0.612233491757
-0.427339823404	-0.612184642601
-0.427247235052	-0.612088420505
-0.42715448007	-0.612073189745
-0.427068266708	-0.611995803176
-0.426984164007	-0.611789414659
-0.426880394032	-0.611730496092
-0.426771417053	-0.611620233639
-0.426692241272	-0.611567432458
-0.426620453232	-0.611472311006
-0.426530815863	-0.611353278262
-0.426451675707	-0.611311159298
-0.426348435748	-0.611049469599
-0.426241898474	-0.611011749739
-0.426153028365	-0.610880124079
-0.426077934968	-0.610726504913
-0.425997723197	-0.61069144344
-0.425919264459	-0.610575366124
-0.42582996129	-0.610479665501
-0.425745574382	-0.610451250477
-0.425654165993	-0.61034056398
-0.425576049625	-0.610238464115
-0.425490261451	-0.610178501396
-0.425400833321	-0.610149071572
-0.425309507637	-0.609986227953
-0.425225925544	-0.609924768629
-0.425136044202	-0.609847828699
-0.425029749418	-0.609844794807
-0.424913625295	-0.609807946571
-0.424837235427	-0.609711344681
-0.424758438855	-0.609598462002
-0.424656400341	-0.609466254739
-0.424556210251	-0.60935573381
-0.424474220751	-0.609297552235
-0.424383693728	-0.609284555836
-0.424291065014	-0.609221379523
-0.424186826943	-0.609125176175
-0.424100459632	-0.609108504598
-0.4240064047	-0.608393595467
-0.423926551508	-0.608349534327
-0.423849027851	-0.608326065127
-0.423771209949	-0.607952373666
-0.42367253185	-0.607883906668
-0.423570479522	-0.607758794146
-0.423482271206	-0.607593466497
-0.423391774109	-0.607094862385
-0.423293311362	-0.607075978233
-0.423176436549	-0.606914585767
-0.42309379013	-0.606675898899

-0.423018057439	-0.606504969084
-0.422938292135	-0.606456967045
-0.422864204006	-0.606351838687
-0.422769468585	-0.606267216855
-0.422679496102	-0.606240168044
-0.422595541731	-0.606154954922
-0.422486934459	-0.606153011853
-0.422412898624	-0.605980832904
-0.422318641877	-0.605908095484
-0.422231291553	-0.605845461283
-0.422136158746	-0.605838430049
-0.422050634793	-0.605705380809
-0.421955015922	-0.605674473913
-0.421874258833	-0.605575383809
-0.421785074784	-0.605531638591
-0.421705696957	-0.605488052801
-0.421618770065	-0.605468671085
-0.421545899011	-0.605392871054
-0.421448781903	-0.605361509825
-0.421375762034	-0.60535741021
-0.421285332906	-0.605314757452
-0.421196029218	-0.60526924539
-0.421094117946	-0.60526811563
-0.421027394023	-0.605263348442
-0.420933639762	-0.605142860066
-0.420851109584	-0.604820656036
-0.42074748609	-0.604361079567
-0.420649068403	-0.604259919922
-0.420570952767	-0.604240755777
-0.420466061113	-0.604133587407
-0.420379596978	-0.604093620535
-0.420306305588	-0.604022489159
-0.420225740865	-0.603983505531
-0.420128489223	-0.603879154654
-0.420022888073	-0.603743304067
-0.419923359086	-0.603731890775
-0.419836713075	-0.603720119041
-0.419752621762	-0.603687256205
-0.41966404209	-0.603533547469
-0.419565550107	-0.603446051376
-0.419463460363	-0.603235654935
-0.419387308652	-0.603182639531
-0.419324252778	-0.603133106722
-0.419249530295	-0.60303820938
-0.419165785734	-0.603037195104
-0.419073593858	-0.603026858051
-0.418989807596	-0.602664812711
-0.418909845099	-0.602655613587
-0.418834315609	-0.602249553087
-0.41873691632	-0.60218731281
-0.418653127683	-0.602163306311
-0.418578359335	-0.602154330269
-0.418498547962	-0.602143737044
-0.418416755659	-0.602060305991

-0.41833555271	-0.601999356442
-0.418236734185	-0.601930456432
-0.418146239774	-0.601801206438
-0.418061725921	-0.601772204358
-0.4179752726	-0.60165888446
-0.417889813826	-0.601650120798
-0.41779453124	-0.601579571457
-0.417714221618	-0.601567841233
-0.417630305954	-0.601460297626
-0.417560912891	-0.601369160713
-0.417447705722	-0.601148090311
-0.417362419292	-0.60101991294
-0.417268717845	-0.600970876476
-0.41719626365	-0.600871311449
-0.417117522432	-0.600787452182
-0.417037421899	-0.600601930206
-0.416961249901	-0.600475704647
-0.416868897982	-0.600364930243
-0.416772951047	-0.600316877646
-0.416690107546	-0.600272022704
-0.416587057983	-0.600227324443
-0.416485372829	-0.600131594657
-0.416392419277	-0.600031980277
-0.416307137339	-0.600009311851
-0.416216710895	-0.599951400787
-0.416124841659	-0.599842372289
-0.416038681431	-0.599725991571
-0.415952565599	-0.599621721169
-0.415868226027	-0.599608195993
-0.415785841569	-0.599506058701
-0.415700766252	-0.599494974143
-0.415610405708	-0.599066093666
-0.415524883426	-0.598872159876
-0.41542152734	-0.598834409046
-0.415334058235	-0.598765616315
-0.415253128841	-0.598383084106
-0.415172267814	-0.59801825095
-0.415078329829	-0.597860202429
-0.414977018869	-0.597630624546
-0.414902647064	-0.597550931282
-0.414817179848	-0.597522484752
-0.414739447465	-0.597407704322
-0.414662237704	-0.597350258526
-0.414557044369	-0.597270471897
-0.414471981338	-0.597186195051
-0.414375862834	-0.597033372364
-0.414288362428	-0.596721405317
-0.414197812337	-0.59670555064
-0.414102803016	-0.596674121674
-0.414019381283	-0.596609487161
-0.413933737869	-0.596403256598
-0.413849815599	-0.596336029246
-0.413765437177	-0.596316098093
-0.413679849008	-0.596310402592

-0.413598307397	-0.596177881861
-0.413515811184	-0.595837653899
-0.413436177067	-0.595763318584
-0.413353039117	-0.595582725248
-0.413261852002	-0.595506921159
-0.413172019458	-0.595475473319
-0.41308929103	-0.595117909598
-0.412982407771	-0.595096254288
-0.412896287174	-0.594724848145
-0.412803848547	-0.59470071451
-0.41272493815	-0.594672449925
-0.412629356346	-0.594250735386
-0.41253886961	-0.59417922425
-0.412456059511	-0.594118605146
-0.412371333707	-0.594082646672
-0.412303534695	-0.593926172453
-0.41222859852	-0.593881122543
-0.412136687326	-0.593677860204
-0.412044290961	-0.59361762536
-0.411963306864	-0.5934720817
-0.41185746263	-0.593231298331
-0.411769652411	-0.593101291314
-0.411676744057	-0.593082167496
-0.411585817357	-0.593002110237
-0.411503302702	-0.592986905075
-0.411416544816	-0.592985598348
-0.411327245048	-0.592866943547
-0.41122628562	-0.592854765659
-0.411131487817	-0.592798966643
-0.411049688163	-0.592785700765
-0.410921250236	-0.592761952018
-0.41083771395	-0.592687497572
-0.410755451965	-0.592671068392
-0.410677506099	-0.592606934555
-0.410584971467	-0.59232251279
-0.410489760256	-0.592318083178
-0.410379906444	-0.592243665049
-0.410297376414	-0.592211432471
-0.410194807024	-0.591774184338
-0.410105106187	-0.59175453103
-0.410023919021	-0.591696196401
-0.409943499607	-0.59164678199
-0.409855561147	-0.591564280175
-0.409782788761	-0.591466762038
-0.409699164499	-0.591406362063
-0.409618565466	-0.591393209834
-0.409514868656	-0.591368732852
-0.409436516861	-0.591323909617
-0.409358673501	-0.591280896815
-0.409267320414	-0.59126848174
-0.409193088368	-0.591103086304
-0.409105101396	-0.591018311693
-0.409013702845	-0.591015000915
-0.408923499772	-0.590952621306

-0.408829685784	-0.590717471361
-0.408747102504	-0.590708211458
-0.408649584668	-0.590554637829
-0.408553804147	-0.590509287108
-0.408463322143	-0.590504688735
-0.408389735999	-0.590111423537
-0.408295328425	-0.589848545167
-0.408211758883	-0.58983460166
-0.408124023297	-0.589621197833
-0.408043792379	-0.589599609328
-0.407960939862	-0.589558341724
-0.407875688629	-0.589423043219
-0.407774624431	-0.58936811675
-0.407656485587	-0.589146229759
-0.407577433283	-0.589076952083
-0.407480727519	-0.589003283685
-0.407390654972	-0.58897958251
-0.407307672525	-0.588856877079
-0.40720159321	-0.588812022373
-0.407105277034	-0.588570974985
-0.407013296547	-0.58856042656
-0.406925770993	-0.588542882733
-0.406831338145	-0.588542199648
-0.406733512844	-0.588428076173
-0.406648446064	-0.588412779986
-0.406571552675	-0.588406744321
-0.406476377501	-0.58779038346
-0.406393079009	-0.587732496212
-0.406314672577	-0.587409758597
-0.406228467813	-0.587160730899
-0.406139920545	-0.587112446304
-0.406062404209	-0.587104341637
-0.405977349255	-0.587008970836
-0.405877497268	-0.586874892915
-0.405794994143	-0.586735199359
-0.405710820717	-0.586700777605
-0.405625831441	-0.586629881906
-0.405539113576	-0.586465949211
-0.40545228243	-0.586301137231
-0.405356763687	-0.586141658241
-0.405280451973	-0.586101872962
-0.405176763174	-0.586093667757
-0.405085709697	-0.585941635297
-0.405004383883	-0.585908266707
-0.404925974426	-0.585883137393
-0.404840174865	-0.585854731412
-0.404748485744	-0.585791238187
-0.404661742901	-0.585661523997
-0.404573261913	-0.585589818182
-0.404490368449	-0.585510283385
-0.404412375493	-0.585428010201
-0.404316238654	-0.585397891971
-0.404234024704	-0.585158623699
-0.404140181765	-0.584898598994

-0.404062560301	-0.584846570624
-0.40395969008	-0.584747453586
-0.403867652996	-0.584556899062
-0.403773271114	-0.58440589291
-0.403681408357	-0.5839879351
-0.403575390411	-0.583971873679
-0.403491783552	-0.583927572112
-0.403419845261	-0.583878068258
-0.403328209886	-0.583876280533
-0.403222325798	-0.583824051489
-0.403126776923	-0.583549985007
-0.403020259417	-0.583529049091
-0.402924153067	-0.583279773575
-0.402833470817	-0.58321741336
-0.402724398262	-0.583028721674
-0.402633037872	-0.583026276373
-0.402527702005	-0.582944770548
-0.402439445347	-0.582944276344
-0.402348964091	-0.582878704068
-0.402256366721	-0.58285790307
-0.402161199132	-0.582835550095
-0.40207871654	-0.582659496114
-0.401984846725	-0.582582609688
-0.401890624788	-0.582416322421
-0.401800768714	-0.582328285514
-0.401722641313	-0.581909553246
-0.401642793588	-0.581897878983
-0.401553105769	-0.581831950744
-0.401467009283	-0.581587802066
-0.401376467265	-0.581330181124
-0.401275137144	-0.580953862772
-0.40119638018	-0.580347496011
-0.401114723698	-0.580113388917
-0.401029779467	-0.580111243209
-0.400947611037	-0.580050081723
-0.40085661034	-0.58001544336
-0.400771219078	-0.579742258947
-0.400699700398	-0.579351707774
-0.400618746743	-0.579138967477
-0.400539254984	-0.579025224405
-0.400454019093	-0.578801034982
-0.400351008302	-0.578710335963
-0.400272980311	-0.578710219893
-0.400191390439	-0.578675121959
-0.40009998198	-0.578664585022
-0.40001184356	-0.578518324062
-0.399919647416	-0.578502586511
-0.399818277938	-0.578489147387
-0.399721444723	-0.578310843508
-0.399636056611	-0.578277268683
-0.399545779915	-0.578221453685
-0.399439457292	-0.578205752491
-0.399338843464	-0.578075208858
-0.399247385944	-0.57802924485

-0.399147267134	-0.577961093922
-0.399051610319	-0.577866667508
-0.398970790068	-0.577857446817
-0.398869937929	-0.577692082771
-0.39876001382	-0.577677598887
-0.398665649448	-0.577645703282
-0.398575767225	-0.577621353159
-0.398495374589	-0.577354439242
-0.398403558698	-0.577280192542
-0.398324448502	-0.577077629577
-0.398236090772	-0.576940862186
-0.398134140587	-0.576852517618
-0.398051237707	-0.576284486372
-0.397964716717	-0.576264774405
-0.397861152425	-0.576156751598
-0.397785762272	-0.576040584765
-0.397691743079	-0.575781170359
-0.397613121034	-0.575780956142
-0.397517756252	-0.575532562078
-0.39742718914	-0.575431688071
-0.397342282763	-0.575143165751
-0.397248854574	-0.574992522143
-0.39716750751	-0.574898852548
-0.397088210151	-0.574884260616
-0.396995812448	-0.574834852751
-0.396915764677	-0.574725654738
-0.396839814974	-0.574713848014
-0.396752853693	-0.574678454696
-0.39667091796	-0.574600757194
-0.396578898844	-0.574559070952
-0.396487220529	-0.574532508055
-0.396395684436	-0.57452079789
-0.396303304002	-0.574343384126
-0.396203474475	-0.574276162864
-0.396105481526	-0.573745195259
-0.396033761549	-0.573740111429
-0.395954456905	-0.573578312623
-0.395852856522	-0.573534056344
-0.395766750694	-0.57331281419
-0.395684044865	-0.573282309707
-0.395595996645	-0.573177336493
-0.395500641976	-0.573136958408
-0.395408882648	-0.573080306423
-0.395305659887	-0.572986240524
-0.395197930603	-0.572970995415
-0.395102002183	-0.572910891608
-0.39502330317	-0.572658888206
-0.394918449399	-0.572575658803
-0.394812989474	-0.572572539869
-0.394741963141	-0.572432866284
-0.39466308722	-0.572205194385
-0.394575019544	-0.572073545873
-0.394473638629	-0.571943069345
-0.394393661636	-0.571881862973

-0.394311136015	-0.571803182176
-0.394203376862	-0.571742574523
-0.394112202722	-0.571693337252
-0.394010454741	-0.571414440814
-0.393924188507	-0.571316704366
-0.393835821415	-0.571250702804
-0.393737260589	-0.571213904802
-0.393636182707	-0.57101511591
-0.393544357793	-0.570992737949
-0.393450095615	-0.570832301318
-0.393348243908	-0.570832192054
-0.393254737695	-0.570808927383
-0.393153884202	-0.570736300091
-0.393066245572	-0.57057165479
-0.392983261262	-0.570520553149
-0.392899630651	-0.570505083397
-0.392791694264	-0.570384018424
-0.392690170115	-0.570248150891
-0.39260735577	-0.570234565173
-0.392511164731	-0.570120525768
-0.392430032894	-0.569874780632
-0.392361147278	-0.569863534991
-0.392260880371	-0.569668639454
-0.392171775277	-0.569526294257
-0.392075749536	-0.56950922422
-0.391984536287	-0.569429090083
-0.39189893409	-0.569380734302
-0.391794035842	-0.56935221954
-0.391707481956	-0.569313913736
-0.391615992972	-0.569121673399
-0.391526593569	-0.568978358255
-0.391441934133	-0.568964682051
-0.391365177	-0.568955739782
-0.391282931555	-0.568924207041
-0.391206109391	-0.568684807254
-0.391122451703	-0.568665396364
-0.391039546766	-0.568649649386
-0.390952654721	-0.568486360669
-0.390838459472	-0.568292535968
-0.390741201057	-0.568206941287
-0.390652534495	-0.567786316137
-0.390577284664	-0.567768788933
-0.39047701532	-0.567646910748
-0.390380853303	-0.567482071482
-0.390286944491	-0.567409571403
-0.390172962976	-0.567186026747
-0.39009278883	-0.566982585567
-0.389997294313	-0.566871124841
-0.389911571089	-0.566814374453
-0.389822576555	-0.566731176761
-0.389737505935	-0.56672922816
-0.389660570426	-0.566707066832
-0.389573443422	-0.566485150095
-0.389472821128	-0.566474661277

-0.389402616955	-0.566313533616
-0.389305151385	-0.566038312516
-0.38920749067	-0.566037440462
-0.389117524921	-0.566015809232
-0.389022111587	-0.565854189389
-0.388948331432	-0.56581274705
-0.388858310631	-0.565707027579
-0.388770183599	-0.5654552373
-0.388686569463	-0.56537026488
-0.388596733764	-0.565237883815
-0.388512132739	-0.565224617647
-0.388419607016	-0.565192995495
-0.388329591181	-0.565160800694
-0.38822123737	-0.564638532382
-0.388136855573	-0.564625318683
-0.388055355754	-0.564424882377
-0.387972773296	-0.564364076441
-0.387877134677	-0.564286167525
-0.387785072867	-0.56410385823
-0.387709984777	-0.564079507717
-0.387612778741	-0.56382439244
-0.387540661523	-0.563789587862
-0.387442651492	-0.563730768861
-0.387353030553	-0.563726847023
-0.387267640354	-0.563670611562
-0.387169265211	-0.563445672108
-0.387090158901	-0.563416254482
-0.387010251774	-0.563146192604
-0.386939871507	-0.562977674316
-0.386855550156	-0.562940765317
-0.386755459373	-0.562536285905
-0.386661193732	-0.562527977488
-0.386571789222	-0.562524034877
-0.386500856225	-0.562394344293
-0.386418092737	-0.562278054105
-0.386329069905	-0.562249150959
-0.386221609401	-0.562184375751
-0.38612688196	-0.562057542329
-0.386039179157	-0.562002844051
-0.385945809847	-0.56193123467
-0.385835926716	-0.561554569331
-0.385747151862	-0.561460000335
-0.385664167853	-0.56139807698
-0.385578180281	-0.561048389363
-0.385501110545	-0.561003964722
-0.385407148392	-0.560994055059
-0.385317482751	-0.560928735687
-0.385195927021	-0.56090556461
-0.385093218238	-0.560809692914
-0.385014005532	-0.56080501476
-0.384941143176	-0.560803215196
-0.384852389399	-0.560470870096
-0.384747083762	-0.560386369719
-0.384669154926	-0.560301173865

-0.384579797497	-0.560286041623
-0.384494188446	-0.560106875321
-0.38440746085	-0.560066134134
-0.384326931416	-0.56006293526
-0.384229070447	-0.559941968217
-0.384128441332	-0.559919237443
-0.38404562006	-0.559883370266
-0.38395865096	-0.559768591789
-0.383866108379	-0.559752101529
-0.383773854457	-0.559714958773
-0.383701154787	-0.559477639255
-0.383606722782	-0.559463646515
-0.383491456611	-0.559424276956
-0.383395556213	-0.559399483586
-0.383317117917	-0.559326733839
-0.383242883206	-0.559118044211
-0.383155277212	-0.559115201762
-0.383062429284	-0.558993676836
-0.382975007328	-0.558936286825
-0.382882034287	-0.558914935008
-0.382805683236	-0.558735649177
-0.382719021813	-0.55869363458
-0.382612269534	-0.558520707273
-0.382533899202	-0.558364416892
-0.382462372216	-0.55828877278
-0.3823856168	-0.558267496041
-0.382300476053	-0.558186095239
-0.382194569891	-0.558155804347
-0.382095335106	-0.558021081148
-0.382005206322	-0.557846307158
-0.381914808864	-0.557835729891
-0.381830862735	-0.557804141288
-0.381748692086	-0.557694183493
-0.381660525422	-0.557668338292
-0.381560444973	-0.557439723571
-0.381444984805	-0.557343474099
-0.38137041574	-0.557311970062
-0.381302266214	-0.557260356568
-0.381210812948	-0.557131999411
-0.381119519195	-0.55697917025
-0.381042171986	-0.556977905823
-0.380923102778	-0.556579986206
-0.380852003444	-0.556476138256
-0.380755934882	-0.556407952982
-0.380665065706	-0.556344980029
-0.380565891361	-0.556270078005
-0.380485477058	-0.556171498392
-0.380402541626	-0.556101387541
-0.380322845595	-0.556092738141
-0.380242181027	-0.556033148628
-0.380134754205	-0.55599690569
-0.380050779257	-0.555982303615
-0.379970315105	-0.555913431789
-0.379878873642	-0.555909367265

-0.379768632363	-0.55584880933
-0.379695178797	-0.555765863887
-0.379607384841	-0.555726018121
-0.379532494722	-0.555700155442
-0.379437310523	-0.555689644897
-0.379366235188	-0.55558577735
-0.379279238195	-0.555066716668
-0.379196467787	-0.555044988723
-0.379120154423	-0.555034899902
-0.379030995738	-0.554766744062
-0.378956915842	-0.554638520569
-0.378862769723	-0.554618471898
-0.378769483508	-0.554460554287
-0.378700406793	-0.554289111385
-0.378599876554	-0.554004932869
-0.378500954139	-0.553899754454
-0.378407403566	-0.553792993783
-0.378312459232	-0.553413026191
-0.378245553053	-0.553176655917
-0.378151653379	-0.553144688809
-0.378075109551	-0.553129573357
-0.377981578812	-0.553019385538
-0.377885894421	-0.552742714092
-0.377790782119	-0.552712441489
-0.377687936758	-0.552660423246
-0.377612958408	-0.552612978283
-0.377516479981	-0.552536646957
-0.377435789002	-0.552511326134
-0.377347965619	-0.552407223601
-0.377260542579	-0.552397347438
-0.37716104676	-0.552335201288
-0.377065854848	-0.552166961502
-0.376981798625	-0.5519608249
-0.37690114343	-0.551935775217
-0.376830048263	-0.55189124126
-0.376729837935	-0.551881770109
-0.376617584636	-0.551601875807
-0.376532061792	-0.551556673751
-0.376436011524	-0.551550190375
-0.376342687	-0.551253224327
-0.376249192336	-0.551216878834
-0.376151940107	-0.551153775671
-0.376075386435	-0.550946070486
-0.375993691177	-0.550924821999
-0.375908204823	-0.55086395054
-0.375805335869	-0.550755248575
-0.375713795481	-0.550710967784
-0.375624414773	-0.550595199169
-0.375522883652	-0.550500716506
-0.375427236461	-0.550474099529
-0.375341904569	-0.550449267828
-0.375258002634	-0.550401703994
-0.375184143038	-0.550329174219
-0.375093807377	-0.550296722557

-0.374986263354	-0.550295098974
-0.374891511533	-0.550230738979
-0.374799551209	-0.550072949294
-0.374700903988	-0.549971154295
-0.374622075935	-0.549714706567
-0.374539965365	-0.549698517164
-0.374461643666	-0.549335063742
-0.37439548662	-0.549197668021
-0.37431015597	-0.549153983652
-0.374227755282	-0.549135209736
-0.374139696004	-0.549099091763
-0.374034722286	-0.549078565601
-0.373943766066	-0.54901840736
-0.373867716256	-0.548807215535
-0.373795902229	-0.548652419355
-0.373720645861	-0.548531537092
-0.37361919932	-0.548471197396
-0.373525683755	-0.547928708512
-0.373433103025	-0.547849216503
-0.373362431569	-0.54779987428
-0.373287412695	-0.547791776499
-0.373190175709	-0.547623704164
-0.373110306734	-0.547595478246
-0.373015144313	-0.547584319509
-0.372917665902	-0.547583673865
-0.372819094196	-0.547520141224
-0.372726641848	-0.547171939152
-0.372633386735	-0.54715020186
-0.372551833397	-0.547012567796
-0.372475150043	-0.546891848165
-0.37238715283	-0.546839246776
-0.372295386987	-0.54680976033
-0.372217909621	-0.546792620173
-0.37213291467	-0.546393274005
-0.372051144049	-0.546324874703
-0.371967333348	-0.546203234349
-0.371880179735	-0.546183031291
-0.371801770177	-0.546050433734
-0.371711506569	-0.546017516602
-0.371632063775	-0.545995854735
-0.371528280358	-0.545881015155
-0.37144776798	-0.545738615527
-0.371357302196	-0.545635937139
-0.371279817636	-0.545534747071
-0.371177426891	-0.545534642391
-0.371092235024	-0.545465685373
-0.371022332811	-0.545015714349
-0.370920777394	-0.544908735307
-0.370842117013	-0.544864717642
-0.370760569771	-0.544766601423
-0.370668848147	-0.544746626679
-0.370590109539	-0.544678839659
-0.370508762142	-0.544481725592
-0.370431446425	-0.544423742172

-0.370336565932	-0.544422022247
-0.370261124032	-0.544367806986
-0.370163656868	-0.544348551487
-0.370069845899	-0.544260338115
-0.369992750191	-0.544147301486
-0.369908823298	-0.544077043063
-0.369825475315	-0.544058278605
-0.369736368613	-0.543952211438
-0.369635208173	-0.543906695714
-0.369550326774	-0.543665495418
-0.369475769854	-0.543653139787
-0.369381187524	-0.5436384038
-0.369284692969	-0.54356554309
-0.369189024118	-0.543351399733
-0.369105780279	-0.543344142937
-0.369004125263	-0.543183944818
-0.368907859642	-0.543175951614
-0.36880609183	-0.543168396313
-0.36870317936	-0.543145250611
-0.368601159861	-0.543138751223
-0.368511340533	-0.543063730146
-0.368425794275	-0.543048155613
-0.368337380374	-0.543007561996
-0.368251398635	-0.542896535067
-0.36816118302	-0.542794035642
-0.368081739905	-0.542647493604
-0.367994696941	-0.542592969439
-0.367895031204	-0.542483984185
-0.367822947763	-0.542474210171
-0.367752571055	-0.542178298152
-0.367668990523	-0.542006220448
-0.367583105225	-0.541937092035
-0.367489304156	-0.541914898853
-0.367400621564	-0.541862841988
-0.367309393737	-0.541735613801
-0.367211834536	-0.541607902645
-0.36713905187	-0.54146871173
-0.367056029728	-0.541345609456
-0.366965932024	-0.541106976749
-0.366871592262	-0.541024923117
-0.366789315153	-0.540829949103
-0.366695149244	-0.540803404069
-0.366608276541	-0.540740785091
-0.366524700046	-0.540733047695
-0.366449422253	-0.540410619866
-0.366366066444	-0.540254899583
-0.366281604695	-0.540219000243
-0.366173080708	-0.540180457353
-0.366065476405	-0.540059741317
-0.365980658351	-0.540016275978
-0.36588547651	-0.539925352509
-0.365782142757	-0.539905670404
-0.365680257122	-0.539780976635
-0.365586741323	-0.539769640356

-0.365497180861	-0.539508103021
-0.365408334934	-0.539450867436
-0.365328882369	-0.539310770891
-0.365238293128	-0.539241879379
-0.365147785168	-0.5390268184
-0.36507268636	-0.53899068887
-0.364976807124	-0.538701579263
-0.36487659028	-0.538443762646
-0.364800841696	-0.538309597792
-0.364711029234	-0.538211738269
-0.364616780086	-0.53795003546
-0.364526362524	-0.537827857256
-0.364466960931	-0.537783664232
-0.364360266481	-0.537747345139
-0.364285804777	-0.53758557968
-0.364201201834	-0.537490437519
-0.364118990294	-0.537364040125
-0.364008460302	-0.537346730843
-0.363913571093	-0.53730984554
-0.363819650263	-0.537157663637
-0.36372866618	-0.536649152122
-0.363648818632	-0.536569599392
-0.363575029995	-0.536562321188
-0.363470507159	-0.536546709313
-0.363376190939	-0.536284367771
-0.363287492097	-0.536233616895
-0.363215608362	-0.536223572962
-0.363125916099	-0.536170008178
-0.363036095632	-0.536141719352
-0.362952720885	-0.536137033168
-0.362868120582	-0.536057149165
-0.362780103114	-0.535887672471
-0.362693653908	-0.535794058338
-0.362612111953	-0.535611591175
-0.362537024981	-0.535455877482
-0.36244829303	-0.53530605874
-0.362359415781	-0.535279373892
-0.362272844258	-0.535237886627
-0.362175868931	-0.535219824966
-0.362082236608	-0.535124549212
-0.361982131783	-0.53510103393
-0.361905744181	-0.535001212049
-0.361827902862	-0.534947010914
-0.361737526688	-0.534886215956
-0.36164073662	-0.534851519353
-0.361549450141	-0.534816287509
-0.361455244045	-0.534725077354
-0.361373834206	-0.534567206831
-0.361271844257	-0.534487975547
-0.361185347944	-0.534486780827
-0.36110989734	-0.534483013553
-0.361025036326	-0.534422614526
-0.360950002508	-0.534329465409
-0.360861913215	-0.53430745968

-0.360777815303	-0.534270403163
-0.360684187548	-0.534262994791
-0.360596407081	-0.534251182643
-0.360502078121	-0.53424026375
-0.360411542811	-0.534177064222
-0.360337619674	-0.534160431102
-0.36024860005	-0.53387696572
-0.360152991327	-0.533844125165
-0.360069046559	-0.533795759015
-0.359984650213	-0.533717366037
-0.359898507528	-0.533689763086
-0.359781637345	-0.533430496828
-0.359696130109	-0.533391946885
-0.359607949135	-0.533101880537
-0.359516123262	-0.532986114866
-0.359420472306	-0.532951783167
-0.359323305572	-0.53287960437
-0.359238959593	-0.532606816587
-0.359141685195	-0.532555331863
-0.359059943229	-0.532377819177
-0.358998834956	-0.532274850984
-0.358904028807	-0.532124315282
-0.358807642077	-0.532102574875
-0.358717842086	-0.532011371055
-0.358631344682	-0.531950557068
-0.358543432769	-0.53181377239
-0.358468004444	-0.531746050666
-0.358384606063	-0.53173239958
-0.358310667851	-0.531730977395
-0.358230616517	-0.531637210143
-0.35814322065	-0.531567220045
-0.358045859647	-0.531557675437
-0.357963848718	-0.531482113204
-0.357889410267	-0.53132137066
-0.357822595839	-0.531292666595
-0.35773386261	-0.531262022921
-0.357650279983	-0.53122812831
-0.357568076525	-0.531065730661
-0.357480116467	-0.530964030244
-0.357396865066	-0.53093679955
-0.357306022966	-0.530918840619
-0.357221242878	-0.530886331996
-0.357150595889	-0.530847489661
-0.357064488488	-0.530825660858
-0.356966535189	-0.530678584827
-0.356862208497	-0.530580402083
-0.35677314364	-0.530423801363
-0.356679222825	-0.53031446751
-0.356581819031	-0.530221745862
-0.356513572108	-0.530166049508
-0.356426186202	-0.530082709206
-0.356325291265	-0.5299874282
-0.35621704952	-0.529983986345
-0.356113627498	-0.529868387295

-0.356028787276	-0.529817430953
-0.355941675051	-0.529810175899
-0.355850410447	-0.529746070341
-0.355776857303	-0.529631668864
-0.355690628258	-0.529601692704
-0.355606031635	-0.529588913221
-0.355524647943	-0.529563639845
-0.355444966993	-0.529544927159
-0.355361390012	-0.529534184625
-0.355276049678	-0.52949403447
-0.355187895071	-0.529384232875
-0.355094465438	-0.529259279415
-0.355022617326	-0.529212558526
-0.354924117964	-0.529179445953
-0.354833707439	-0.529164761853
-0.354744447625	-0.529124142011
-0.354658933591	-0.529031440032
-0.354570696625	-0.528947129139
-0.354486800955	-0.528943296991
-0.354405666939	-0.528860301573
-0.354314803666	-0.528781874385
-0.354232935051	-0.528711683929
-0.354152772576	-0.528682454457
-0.354075129512	-0.528622925931
-0.353981448719	-0.528560111397
-0.353886951054	-0.528313309498
-0.353791303473	-0.528281251293
-0.353698569256	-0.528067746946
-0.353621475901	-0.527886317544
-0.353536550619	-0.527875355023
-0.353462265248	-0.527795997262
-0.353377934003	-0.527683129939
-0.353289680879	-0.527606471354
-0.353207065717	-0.527582907613
-0.35313241177	-0.527552597976
-0.35303726829	-0.52740935738
-0.352938032785	-0.527259802205
-0.352844061207	-0.527202467313
-0.352748111815	-0.527181690834
-0.352658762812	-0.52688296672
-0.352578042079	-0.526774633881
-0.352489965914	-0.526716083798
-0.352397831996	-0.526506876274
-0.352302589826	-0.526454319144
-0.352208472293	-0.526400334983
-0.352122826136	-0.525951356709
-0.352049441273	-0.525626626821
-0.351958064801	-0.52559572232
-0.351862240619	-0.525566786824
-0.351781369144	-0.52555893258
-0.35168188206	-0.525526231222
-0.351581981611	-0.525427498411
-0.351495900057	-0.525397681024
-0.351423382585	-0.525367120986

-0.351336231441	-0.525257043326
-0.351237153902	-0.525149575742
-0.351149632557	-0.525083660266
-0.351062543037	-0.524821178575
-0.350989543355	-0.524809083829
-0.350901145901	-0.524719211044
-0.350814546756	-0.524653540914
-0.350722641142	-0.524606582163
-0.350637521594	-0.524506564658
-0.35056708001	-0.52446933653
-0.350490654388	-0.524290935743
-0.350415930951	-0.524248579418
-0.350329909232	-0.524202097946
-0.350240323305	-0.524159429451
-0.350153986657	-0.523837431943
-0.35007304363	-0.52374527463
-0.349988441646	-0.523638432158
-0.349904517297	-0.523600982404
-0.34981437669	-0.523485427727
-0.349739745816	-0.523478148616
-0.349649894367	-0.523466805538
-0.349573730396	-0.523099677521
-0.349480453878	-0.523034785139
-0.349404373154	-0.522932399497
-0.349322508324	-0.522729210776
-0.349237588618	-0.522579493353
-0.349130599799	-0.522487384545
-0.349041624866	-0.522458795097
-0.348952054601	-0.522397438321
-0.348864055434	-0.522317982693
-0.348787714391	-0.522270001055
-0.348685629427	-0.522269974564
-0.348593613335	-0.522266119736
-0.348514361418	-0.522185466977
-0.348422714082	-0.522125828768
-0.348343723658	-0.521905950433
-0.348255901589	-0.521840401257
-0.348168238227	-0.52173274646
-0.348084333199	-0.521653545741
-0.348012619771	-0.521541890853
-0.34791274217	-0.521419921342
-0.347809886592	-0.521152724481
-0.347732458622	-0.521133742682
-0.34764012439	-0.521126948034
-0.347572157118	-0.521091176994
-0.347499197113	-0.520938054272
-0.347408688677	-0.520897323061
-0.347318270101	-0.520847129771
-0.347215560966	-0.520780820854
-0.347126026071	-0.520704817541
-0.347024551122	-0.520673156435
-0.34692955682	-0.520363722976
-0.346848439372	-0.52032946254
-0.346763078482	-0.520208536214

-0.346678438046	-0.519546884366
-0.346601847062	-0.519500029693
-0.346492941405	-0.5194921033
-0.346390739583	-0.519452797807
-0.346288093554	-0.519432188844
-0.346198871941	-0.519418004731
-0.346103357408	-0.519210356919
-0.34602682082	-0.519062571291
-0.345941435166	-0.518908974828
-0.345849625881	-0.518874306755
-0.345756924928	-0.51880217383
-0.345657886844	-0.518768436371
-0.345563203708	-0.518728905627
-0.34546273998	-0.518504277129
-0.345382213257	-0.518394271663
-0.345280151552	-0.518292992109
-0.345173741936	-0.518279251343
-0.345089506049	-0.518259702102
-0.344997050398	-0.518214872485
-0.344916451839	-0.518195789009
-0.344850420315	-0.518193003336
-0.344761494031	-0.51810525069
-0.344658432144	-0.517946391562
-0.344590821244	-0.517922058362
-0.344494943147	-0.517903998026
-0.344410605572	-0.517783916046
-0.344328871638	-0.517768460392
-0.344221409676	-0.517704179863
-0.344127968103	-0.517703673851
-0.344024908798	-0.517650651783
-0.3439350249	-0.517607219565
-0.3438441286	-0.517533626794
-0.343761462833	-0.517492593179
-0.343656081169	-0.51747207883
-0.343585223179	-0.517370800951
-0.343493488033	-0.517295715459
-0.343394309309	-0.51719758951
-0.343284878354	-0.517119928482
-0.343209523343	-0.517025063712
-0.343133673725	-0.516999258977
-0.343054252151	-0.516873226663
-0.342964662023	-0.516856837894
-0.342874933983	-0.516582882275
-0.342766941917	-0.516569688093
-0.342673194759	-0.51649870867
-0.342579706445	-0.516435366027
-0.342499302109	-0.51642138137
-0.342407642629	-0.516365155187
-0.342331346219	-0.516332376625
-0.342244549537	-0.516329823708
-0.342152746778	-0.516257860172
-0.342053733618	-0.516148453835
-0.341980320448	-0.515876735588
-0.341879803758	-0.515778278982

-0.341798821101	-0.515465356366
-0.341717398619	-0.515386288417
-0.341643148903	-0.515183066923
-0.341567938115	-0.515108958181
-0.34148947728	-0.515091493494
-0.341403783677	-0.515082267695
-0.341317390944	-0.515050606564
-0.341231151565	-0.514819466377
-0.341134207117	-0.514577027859
-0.341048637911	-0.514304994781
-0.340947206443	-0.514292724467
-0.34086624542	-0.514190616408
-0.340776382798	-0.51403468377
-0.340688911554	-0.513984387389
-0.340598763878	-0.513838122575
-0.340497692481	-0.513804021384
-0.340403654671	-0.513795983474
-0.34030083299	-0.513690414605
-0.340208880587	-0.513538178396
-0.340120198106	-0.513450976352
-0.340035121124	-0.513379585191
-0.339955287444	-0.513360817874
-0.339865115062	-0.513048055792
-0.339778434525	-0.512937261576
-0.33970138697	-0.512922168603
-0.33960633993	-0.512798138846
-0.339526981145	-0.512742865659
-0.339438868028	-0.512742647603
-0.339363986221	-0.512740352699
-0.339249984924	-0.512673093462
-0.339157797386	-0.512586545041
-0.339070075203	-0.512543511242
-0.338970893339	-0.512480251535
-0.33888241844	-0.512441265666
-0.33880897562	-0.512432586161
-0.338732499152	-0.51220641457
-0.338649269464	-0.511881814612
-0.338551621469	-0.511719825339
-0.338468121882	-0.511493583874
-0.338377892941	-0.511474662518
-0.338287157512	-0.511446898691
-0.338207259794	-0.511434460508
-0.338109314122	-0.511366605772
-0.33802779938	-0.511188239258
-0.337944284994	-0.510884771304
-0.337847181968	-0.510861937884
-0.337767284072	-0.510713622841
-0.337672000118	-0.510682365005
-0.337573621037	-0.510495047029
-0.337477597041	-0.510457196058
-0.337388330583	-0.510200710099
-0.337293377171	-0.510171324582
-0.337204582073	-0.510162053962
-0.337116732033	-0.510114500486

-0.337040946355	-0.509970270214
-0.336952352672	-0.509967299173
-0.336868878565	-0.509720502125
-0.336772135736	-0.509693036606
-0.336686238594	-0.509671724121
-0.336604794805	-0.509558947293
-0.336519061766	-0.509449174052
-0.336430876733	-0.509446135702
-0.336337150313	-0.509276999726
-0.336260864364	-0.509228690859
-0.336167027091	-0.509130059672
-0.336085401852	-0.509013448678
-0.336001339193	-0.509002560688
-0.335917827493	-0.50883827615
-0.335836858075	-0.508743662065
-0.335744205574	-0.508714854807
-0.335668262416	-0.508553537945
-0.335581485492	-0.508425309373
-0.335483321058	-0.508316215038
-0.335404377973	-0.508280028034
-0.335309472686	-0.508088968283
-0.335221082458	-0.508087663826
-0.335123862656	-0.508035893154
-0.335055732326	-0.507911686253
-0.334971145056	-0.507842356724
-0.334876122181	-0.507801701203
-0.334793304294	-0.507787213777
-0.334704694699	-0.507778167538
-0.334625506782	-0.507759505928
-0.334525067724	-0.507697686339
-0.334441455466	-0.507676737876
-0.334367568406	-0.507146382661
-0.334298025793	-0.506976523168
-0.334216889137	-0.506917219774
-0.334139810712	-0.506732755196
-0.334057297687	-0.506611899857
-0.333963917572	-0.50655454158
-0.33387029169	-0.506543982081
-0.333770879487	-0.506435012796
-0.333670166511	-0.506406011968
-0.333587612179	-0.506398154509
-0.33350794498	-0.506395620379
-0.333424045945	-0.506282745027
-0.333317447399	-0.506247785006
-0.333229287486	-0.506206745735
-0.333123953135	-0.506047018708
-0.333023519763	-0.506003369648
-0.332935555597	-0.505941218205
-0.332842601803	-0.505921895876
-0.332751798032	-0.505768514481
-0.332677861753	-0.505737612623
-0.332584104848	-0.505730595102
-0.332502341583	-0.505510129204
-0.332412829018	-0.505490009325

-0.332333734427	-0.50542412791
-0.332249561104	-0.505402745253
-0.332179428696	-0.505212543277
-0.332099203455	-0.505176030329
-0.332010983546	-0.50503927768
-0.331928076554	-0.505022068334
-0.331822836723	-0.504892153644
-0.331729554802	-0.504798061902
-0.331643551594	-0.504730934565
-0.331569689562	-0.504677117432
-0.331491646914	-0.504581576316
-0.331389980861	-0.504570107425
-0.331304642013	-0.504321874477
-0.33122273936	-0.504260126061
-0.331122739857	-0.50425098641
-0.331025216497	-0.504111815438
-0.330938455082	-0.50408794916
-0.330861101569	-0.504082901589
-0.330783313305	-0.50407201771
-0.330684192985	-0.503798741599
-0.330605315754	-0.503786624498
-0.330514569865	-0.503722275294
-0.330439060884	-0.503611043511
-0.330347817825	-0.503588690633
-0.330246642258	-0.503402684961
-0.330157284585	-0.503266300581
-0.330068079391	-0.503127223916
-0.329988031278	-0.503119119985
-0.329889392358	-0.503064205236
-0.329789185585	-0.503048375618
-0.329699323825	-0.502986408581
-0.32960754025	-0.50285625047
-0.329513326517	-0.502700280744
-0.329411758283	-0.502614828007
-0.329316414331	-0.502485516876
-0.329244082571	-0.502384903578
-0.329151215702	-0.502314310725
-0.329062187706	-0.502211524021
-0.328980561623	-0.502167803162
-0.328892134187	-0.502028995246
-0.328813121511	-0.501877410178
-0.328721147699	-0.501767709215
-0.328638074215	-0.501714755131
-0.328540552832	-0.501653082816
-0.328443571327	-0.50158404214
-0.328371165001	-0.501252128841
-0.328287574693	-0.501233435258
-0.328205142173	-0.501140808219
-0.328117654356	-0.500976053454
-0.328019415867	-0.500863977797
-0.327931375613	-0.500739969557
-0.327855761806	-0.500471032254
-0.327749710664	-0.500416206676
-0.327670630255	-0.500276447643

-0.327580550188	-0.499908365667
-0.327490527692	-0.499893397537
-0.327387767446	-0.499776144047
-0.327298730546	-0.499741285706
-0.327224750121	-0.499672122845
-0.327121406377	-0.499588007616
-0.327042822613	-0.499577763868
-0.326939189371	-0.499510272784
-0.326868287458	-0.499475885786
-0.326759121469	-0.499375479812
-0.32665977252	-0.499275627788
-0.326573770574	-0.499222193274
-0.326500506032	-0.498948103684
-0.326419632666	-0.498835232099
-0.326328378217	-0.498598649841
-0.326250250649	-0.498549659754
-0.326148286472	-0.498298003675
-0.326057156903	-0.497859792474
-0.325966485326	-0.497600439565
-0.325885544555	-0.497542254775
-0.32579968737	-0.497384608167
-0.325706959027	-0.497360162229
-0.32561731563	-0.49725864829
-0.325533667904	-0.497216018601
-0.325439386833	-0.497215247422
-0.32536682079	-0.497137904483
-0.325291480545	-0.497031437462
-0.325220717505	-0.496899947428
-0.32514495898	-0.496875697726
-0.325051437226	-0.496741189673
-0.324975417435	-0.496680903763
-0.32489097024	-0.496591329734
-0.32480201968	-0.496579052294
-0.324722781548	-0.496412238031
-0.324649832965	-0.496355571512
-0.324575233935	-0.49627816791
-0.32449001384	-0.4961685136
-0.324413510257	-0.496144295221
-0.324317240008	-0.496087403653
-0.324238196875	-0.496043124369
-0.324151650164	-0.496006026118
-0.324064297184	-0.49598518983
-0.323981763806	-0.495915132163
-0.323881140647	-0.495833910307
-0.323806100831	-0.495829491872
-0.32372030986	-0.495715993437
-0.323629174908	-0.495619373019
-0.323554660125	-0.495563059894
-0.323471713178	-0.495540432041
-0.323400146949	-0.495475119219
-0.323303307002	-0.495122895491
-0.323194942615	-0.495022096083
-0.323122076788	-0.494916676201
-0.32304719685	-0.494136033865

-0.322965045164	-0.494048651842
-0.322870436601	-0.493953139442
-0.322789142721	-0.493896657383
-0.322706580619	-0.493802288564
-0.322608827987	-0.493707343983
-0.322519141988	-0.493614625306
-0.322424536716	-0.493558294225
-0.322338821321	-0.493527584418
-0.322254133008	-0.493505370533
-0.322158815165	-0.493381922716
-0.322055958929	-0.493337775231
-0.321986187629	-0.493293083612
-0.321890158706	-0.493201643228
-0.321807920736	-0.493126994175
-0.321716218551	-0.493086014189
-0.321623150888	-0.492904200659
-0.321539832996	-0.492863752617
-0.321459441262	-0.492822830785
-0.321375240873	-0.492757695342
-0.321289031287	-0.492720616074
-0.321195036041	-0.492639481035
-0.321123929201	-0.492504057484
-0.321049486516	-0.492443497278
-0.320957986763	-0.492364898334
-0.320863962879	-0.492329559396
-0.320785339387	-0.492303377755
-0.320701131275	-0.49228728343
-0.320631826494	-0.492256808394
-0.320566923136	-0.492157789737
-0.320488421016	-0.492086806249
-0.320408895921	-0.492056443188
-0.3203335232	-0.491928684545
-0.320238616464	-0.491897474415
-0.320144782846	-0.491821207274
-0.32005585679	-0.491683570271
-0.319967284115	-0.491678967219
-0.319885986664	-0.491549649709
-0.319805100969	-0.491518233098
-0.319720372732	-0.491183926264
-0.319657035683	-0.490994720553
-0.319562742634	-0.490827791359
-0.319474795451	-0.490633490359
-0.319384003263	-0.490430505482
-0.319299903433	-0.490399004095
-0.319225097421	-0.490343180437
-0.319139158712	-0.490337152622
-0.319059701863	-0.490320232429
-0.318974998294	-0.490307644579
-0.318887744172	-0.490305974245
-0.318781781422	-0.490269096169
-0.31870310354	-0.490089400985
-0.318614347026	-0.490015224498
-0.318512694024	-0.489875971276
-0.318415288295	-0.489807468287

-0.318325597281	-0.489669837374
-0.318244766747	-0.489657157263
-0.318138596639	-0.489609001964
-0.318050381623	-0.48949281397
-0.317955579797	-0.489430844139
-0.317880980244	-0.489351770141
-0.317784807173	-0.489255588602
-0.317688520161	-0.489226334112
-0.317592402856	-0.489200580853
-0.317508588442	-0.489160423612
-0.317436223622	-0.489137471641
-0.317346687804	-0.489134286664
-0.317246847199	-0.489114343392
-0.317150737265	-0.489080271977
-0.317062472288	-0.488953438259
-0.316974924426	-0.488907551913
-0.316892520088	-0.488745054055
-0.316815639463	-0.488602122955
-0.316729142148	-0.488555946247
-0.316650518908	-0.488533571973
-0.316562018305	-0.488451276081
-0.316477525174	-0.488346753461
-0.316390249982	-0.48832391496
-0.316317196062	-0.48830989753
-0.316211039031	-0.487907456095
-0.316129630796	-0.487715604673
-0.316049285003	-0.487641307636
-0.315953043429	-0.487364246178
-0.315864428277	-0.487307672629
-0.31577305972	-0.487262702946
-0.315691805441	-0.486986887327
-0.315608828935	-0.486928384775
-0.315515111138	-0.486889695578
-0.315430357271	-0.486797087564
-0.315326103615	-0.486744418094
-0.315236400594	-0.486551681539
-0.315151914246	-0.486396534193
-0.315058066788	-0.486248568968
-0.314952180244	-0.486189737497
-0.314864198989	-0.48615882488
-0.314793843005	-0.486023812588
-0.314699062356	-0.48599688089
-0.314613526141	-0.485498790067
-0.314528375627	-0.48510916407
-0.314431820019	-0.485088629837
-0.314335829503	-0.485068561716
-0.314241291093	-0.48489445011
-0.314161392217	-0.484851408675
-0.314068617208	-0.484467893599
-0.31399483418	-0.484365805293
-0.313912004117	-0.484338693843
-0.313822960363	-0.484239097209
-0.313737083385	-0.484199334403
-0.313661962164	-0.484167967645

-0.313581981515	-0.484062127774
-0.313479289378	-0.484056555042
-0.313390897196	-0.483912483129
-0.313297921957	-0.483869282523
-0.313208478754	-0.483850746578
-0.313118201205	-0.483813975293
-0.313013657727	-0.483755646771
-0.312934037512	-0.48313667601
-0.312841555863	-0.483095100331
-0.312753581457	-0.482999089295
-0.312655577043	-0.482903780551
-0.312565830558	-0.482808185434
-0.312464099577	-0.482376501628
-0.312374474709	-0.482252434546
-0.312292209649	-0.482082135089
-0.312219236281	-0.482044827803
-0.312138370902	-0.48188265307
-0.312069594555	-0.48172959375
-0.311983662091	-0.481716318807
-0.31189999776	-0.481684343332
-0.311813778943	-0.481621583999
-0.311736745848	-0.481606393217
-0.311642028605	-0.481546923825
-0.311554302419	-0.481530073728
-0.311473975391	-0.481189780292
-0.311378234424	-0.481117817334
-0.311291781331	-0.480672834539
-0.311219708306	-0.480651411999
-0.311125333304	-0.48045711017
-0.311017585924	-0.480247449486
-0.310913631772	-0.48018406377
-0.310826239645	-0.480153616251
-0.310723678513	-0.479783895578
-0.310613583617	-0.479573003786
-0.310534906007	-0.4795005173
-0.310447884573	-0.479402471311
-0.310361316202	-0.479376629377
-0.310281277931	-0.479375217869
-0.310187630243	-0.479360813934
-0.310097602002	-0.479335156695
-0.310020289001	-0.478981037338
-0.309931445155	-0.478940634476
-0.309846998807	-0.478921726061
-0.309776515412	-0.478872874639
-0.309676297782	-0.478835883939
-0.309585863036	-0.478707493612
-0.309505484677	-0.47819108241
-0.309423872403	-0.478177083348
-0.309349462668	-0.478153135888
-0.309266263003	-0.478056714186
-0.309161833621	-0.477718377022
-0.309071369712	-0.477362316439
-0.308995549288	-0.477361320444
-0.308908245867	-0.477343219318

-0.308820928017	-0.477308557758
-0.308729878643	-0.477150851743
-0.308652968431	-0.477066123116
-0.30856461858	-0.47703915998
-0.308484130084	-0.477001699396
-0.308388712457	-0.476951936837
-0.308295990614	-0.476880797189
-0.308208269279	-0.476770019084
-0.30811518185	-0.476636240676
-0.308034289637	-0.476215800813
-0.307942841385	-0.476193581039
-0.307830276793	-0.476187701234
-0.307733997586	-0.476148144681
-0.307656717856	-0.476097159627
-0.307560101238	-0.476087908896
-0.307480914888	-0.475937171074
-0.307383437132	-0.475820056169
-0.307282815205	-0.475817499496
-0.307191889618	-0.475666026597
-0.30710338556	-0.475415744279
-0.306998414616	-0.475331537255
-0.306912499911	-0.475268884116
-0.306831826469	-0.475244951865
-0.306765614244	-0.475189952724
-0.306672093222	-0.475147332231
-0.306586408565	-0.474953724613
-0.306498711998	-0.474790581483
-0.306411271738	-0.474772674511
-0.306315704536	-0.474596120644
-0.306226147315	-0.474428247907
-0.306143251275	-0.474422450041
-0.30606224854	-0.474234422422
-0.305983894213	-0.474206847961
-0.305888325949	-0.474135926763
-0.305808464145	-0.474113769154
-0.30572646117	-0.474090361465
-0.305629952344	-0.473966987694
-0.305531042242	-0.473954232002
-0.305446626354	-0.473805433047
-0.305361336212	-0.473800991293
-0.305278789656	-0.473582668311
-0.30520503992	-0.473254615014
-0.305094562363	-0.472809863575
-0.305008324032	-0.472576041018
-0.304917557435	-0.472548823393
-0.304844497782	-0.472112849186
-0.304753990703	-0.47188988531
-0.304674896356	-0.471864029917
-0.304598406193	-0.471375834904
-0.304521745447	-0.471309781017
-0.304421453954	-0.471006621348
-0.304327706846	-0.470940407712
-0.304247427906	-0.470684874194
-0.304164484261	-0.470533608158

-0.304081576724	-0.470516996998
-0.304002179234	-0.470470334162
-0.303910255455	-0.470266617048
-0.303838862051	-0.470224159857
-0.303758829974	-0.470156942333
-0.303674923566	-0.470129674514
-0.303585624613	-0.4700064586
-0.303506158671	-0.470005525382
-0.303401240236	-0.469747316843
-0.303328040367	-0.469730185561
-0.303252090409	-0.469667849822
-0.303168678419	-0.469522446421
-0.303085969824	-0.469407421984
-0.302982075537	-0.46928108384
-0.302894803577	-0.469254617871
-0.302818935921	-0.468919365923
-0.30273747942	-0.468881064021
-0.302651913582	-0.468859251067
-0.302554073203	-0.468822182965
-0.302463611273	-0.46880873708
-0.302381336508	-0.468734645066
-0.302302249063	-0.468536733893
-0.30222755967	-0.468498500113
-0.302141022975	-0.467904925295
-0.30205368315	-0.46788642709
-0.301963759006	-0.467832858303
-0.301870129149	-0.467606249579
-0.301785858345	-0.467463203142
-0.301714161404	-0.467297558469
-0.301637432981	-0.467229450751
-0.301557001499	-0.467184775181
-0.301472748836	-0.467053673552
-0.301390373631	-0.466840535681
-0.30129696369	-0.466823107782
-0.301199639066	-0.466634641881
-0.301116205486	-0.466550489542
-0.301026299562	-0.466380106627
-0.300936064925	-0.466350684994
-0.300858612668	-0.466343251871
-0.300757539704	-0.466305069821
-0.300682803562	-0.466231444786
-0.300597551201	-0.466193835234
-0.300511156593	-0.466143544169
-0.300436599187	-0.466070435794
-0.300354858615	-0.466036842899
-0.300280819147	-0.466023206354
-0.300201574589	-0.465968569425
-0.300109785129	-0.465678539192
-0.30001174591	-0.465651993767
-0.299935527891	-0.465593857781
-0.299859456027	-0.465043235514
-0.299779255754	-0.464895910155
-0.299703499165	-0.464774959139
-0.299629612363	-0.464772379158

-0.299538357438	-0.464649992648
-0.299450539124	-0.464524229106
-0.299358218981	-0.464510742302
-0.299270399629	-0.464306730514
-0.299182988057	-0.464215504448
-0.299100826325	-0.464172190695
-0.299023212598	-0.464168250511
-0.298926151456	-0.464157309936
-0.298826789569	-0.464142234511
-0.298721755327	-0.464107029278
-0.298632952776	-0.463999898255
-0.298526787451	-0.463954980475
-0.298441392549	-0.463899996176
-0.29835545505	-0.463693195935
-0.2982874361	-0.463619713951
-0.298200968187	-0.463603143871
-0.29812721444	-0.463583181016
-0.298031317455	-0.463276012545
-0.297950923829	-0.463025516582
-0.297863749266	-0.463000907903
-0.297762806789	-0.463000418302
-0.297669235861	-0.462997642811
-0.29758003729	-0.462952847404
-0.297486965806	-0.462860566398
-0.297395974544	-0.462787320127
-0.297327920108	-0.462754110012
-0.297230410137	-0.462661418218
-0.297137712997	-0.46259512139
-0.297049692976	-0.462590831616
-0.296956633102	-0.462534857432
-0.296858558606	-0.462501217006
-0.296768440839	-0.462434100387
-0.296678125341	-0.462422814313
-0.296597865933	-0.462392475088
-0.296514111191	-0.462363337405
-0.296434899065	-0.462275632293
-0.296351401207	-0.462268594118
-0.296260151225	-0.462198434048
-0.296168041725	-0.462195793705
-0.296090173985	-0.462165026018
-0.296009806491	-0.462035023098
-0.295925868617	-0.462030459449
-0.295836728356	-0.461843303266
-0.295759460781	-0.461752301315
-0.295671411543	-0.461154046401
-0.295584662695	-0.461142067706
-0.295498772397	-0.461051692595
-0.295408954914	-0.460903788189
-0.295325260155	-0.460895997578
-0.295238745544	-0.460851367794
-0.29514380265	-0.460785348625
-0.295060893334	-0.460687632947
-0.294992342563	-0.460624388672
-0.294904705703	-0.460428251715

-0.294802903182	-0.460350726195
-0.294709622308	-0.46031668126
-0.294635424158	-0.460200361242
-0.294529363334	-0.459865041689
-0.294456021664	-0.459787049156
-0.294373544479	-0.459702420893
-0.294292880117	-0.459604570192
-0.294204396128	-0.459561848646
-0.294116476869	-0.459553422646
-0.294011318645	-0.459082363973
-0.293929288588	-0.459065373676
-0.293835960721	-0.458970960288
-0.293752934213	-0.45892511471
-0.293669587356	-0.458735979147
-0.293582257077	-0.458648201462
-0.293482239544	-0.458501717907
-0.293397173996	-0.458490870655
-0.293308048249	-0.458429339714
-0.293221236139	-0.458428082385
-0.293130609632	-0.45821687607
-0.293051497793	-0.45812611876
-0.292972352849	-0.458110308157
-0.292884754323	-0.457975071384
-0.292794462834	-0.457957918175
-0.292701999318	-0.45793847504
-0.292618893556	-0.457908647546
-0.292551341815	-0.457881200151
-0.292465140742	-0.457825125778
-0.292371699073	-0.457720194219
-0.292289575521	-0.457415125061
-0.292204760257	-0.457340097685
-0.292120532705	-0.457326231786
-0.292032890865	-0.457263580945
-0.291961544698	-0.457199791191
-0.291885264295	-0.457141116421
-0.291798953419	-0.457082764955
-0.291711834418	-0.457069700975
-0.29163557671	-0.456987195112
-0.291552445396	-0.456767929718
-0.291468587938	-0.456731273606
-0.291381776777	-0.456715463771
-0.291304641677	-0.456687171252
-0.29121673995	-0.456405842084
-0.291144890715	-0.456376076047
-0.291064251534	-0.456360981616
-0.290979622626	-0.456231203555
-0.290882669901	-0.456087281092
-0.290806513923	-0.455919059491
-0.290715621857	-0.45564014188
-0.290636530893	-0.455620757893
-0.290565483358	-0.455609383564
-0.290483911315	-0.455603037382
-0.290400976875	-0.455466500121
-0.290314569602	-0.455170004823

-0.290215319656	-0.455135325349
-0.290121755078	-0.455073129252
-0.290034979739	-0.45507114067
-0.289955106427	-0.454764591591
-0.289873923285	-0.454708404536
-0.289785726964	-0.454561302644
-0.28968879657	-0.454497269035
-0.289585484467	-0.454365010644
-0.289505682571	-0.454331660069
-0.289418381844	-0.454212294081
-0.289326456855	-0.454006098855
-0.289234128252	-0.453685029929
-0.289142525381	-0.45368316808
-0.289057524726	-0.453608814609
-0.288973620094	-0.453596075712
-0.288897856977	-0.453530158243
-0.288813872038	-0.453527517803
-0.288716226738	-0.453498446872
-0.288622068218	-0.453463591299
-0.28852763729	-0.453416268421
-0.288438736281	-0.453355195366
-0.288351058975	-0.45323462845
-0.288260898425	-0.453065561708
-0.288172621795	-0.453041172618
-0.288089700699	-0.452902605074
-0.288002229525	-0.452873684066
-0.287917430077	-0.452758012969
-0.287834044841	-0.452713404269
-0.287740677287	-0.452639463798
-0.287667492204	-0.452463112632
-0.287583003534	-0.452403851751
-0.287494576064	-0.451887187961
-0.287426818196	-0.451776105515
-0.287345112452	-0.45141763889
-0.287248442749	-0.451349832634
-0.287151018628	-0.451310593982
-0.287082491975	-0.450935402864
-0.287005289947	-0.45093013354
-0.286934310328	-0.450871633991
-0.286845287624	-0.450827881153
-0.286759015005	-0.450629463555
-0.286671854752	-0.450550894486
-0.286570143599	-0.450132145588
-0.286483593949	-0.45000491905
-0.286409803522	-0.449871385582
-0.286326667555	-0.449842573221
-0.286249785542	-0.449683258393
-0.286142650415	-0.449535937207
-0.286069639162	-0.449516420947
-0.285992636649	-0.449476183039
-0.285908907444	-0.449384662042
-0.285800302302	-0.449345593778
-0.285697690356	-0.449243930171
-0.285623181173	-0.449222362979

-0.285517939701	-0.448918346149
-0.285430644541	-0.448848752805
-0.285352009603	-0.448789734017
-0.285274926878	-0.448700621259
-0.285193153059	-0.448602887813
-0.285104767806	-0.448598398394
-0.285035192802	-0.448388957569
-0.284954188228	-0.448298491552
-0.284865583336	-0.448243571962
-0.284781624183	-0.448232630949
-0.284703680952	-0.448146648429
-0.284623248392	-0.448133271647
-0.284509138077	-0.448124702906
-0.284421158982	-0.448073274465
-0.284340789825	-0.447983649972
-0.28424751535	-0.447916210062
-0.284162735803	-0.447862677138
-0.284079018089	-0.447787571031
-0.283967260991	-0.447766840939
-0.283878162424	-0.44761349518
-0.283799134235	-0.447592556485
-0.283715679797	-0.447559319266
-0.283630506243	-0.447462498818
-0.283560883042	-0.44745109448
-0.283487576955	-0.447444612634
-0.28339741598	-0.447388244528
-0.283307320141	-0.447341126416
-0.283210898188	-0.447256872384
-0.283135016427	-0.447239666344
-0.283055153755	-0.447216235178
-0.282966275735	-0.447175137141
-0.282884299146	-0.447101610753
-0.28280727437	-0.447057736699
-0.282721547436	-0.447052255668
-0.282653404554	-0.447039843228
-0.2825494601	-0.446923438436
-0.282452558926	-0.446899331766
-0.282378642907	-0.446889518325
-0.282292213805	-0.446756705884
-0.28221231152	-0.446183720789
-0.282135185906	-0.446151906515
-0.282055559961	-0.445823961362
-0.281964847685	-0.445794244765
-0.281867260601	-0.445746339006
-0.281777979891	-0.445619813308
-0.281684140465	-0.445577538532
-0.281608438959	-0.445575132502
-0.28151431305	-0.445463218528
-0.28143953033	-0.445351846096
-0.281356876014	-0.445261903954
-0.281279955904	-0.445066905402
-0.281187901685	-0.445058007241
-0.281102158643	-0.444896824963
-0.281015766859	-0.444888738677

-0.280938808629	-0.444841902054
-0.280861647118	-0.444786916668
-0.280789237676	-0.44466416383
-0.280708734291	-0.44444913019
-0.280619251952	-0.444294706682
-0.2805385815	-0.444171680822
-0.280447219854	-0.444030902855
-0.280352004961	-0.443853609042
-0.28025440619	-0.44385010168
-0.280173404618	-0.443706624089
-0.28007949741	-0.443675290578
-0.279972533314	-0.443626451862
-0.279890904216	-0.443523082312
-0.279809883114	-0.443440124404
-0.279726351145	-0.443406552113
-0.279648295971	-0.44335691205
-0.279550043626	-0.443289821438
-0.279465862376	-0.443242467111
-0.279380623047	-0.443139019809
-0.279283919741	-0.443111503358
-0.279194793984	-0.443086987378
-0.279115346829	-0.443042680397
-0.279030478581	-0.44296698866
-0.278930268733	-0.44293965266
-0.278842039102	-0.442647057554
-0.278746033886	-0.442464199588
-0.278644042407	-0.442456819947
-0.278552729677	-0.441984030828
-0.278473194044	-0.441936131298
-0.278388155811	-0.441927956444
-0.278290246593	-0.441763321628
-0.278215243714	-0.441729480815
-0.278109054047	-0.441662693068
-0.278032921319	-0.441556456777
-0.277941952776	-0.441498432625
-0.277846291424	-0.441372997376
-0.277760711874	-0.441299028606
-0.277662951549	-0.441132519017
-0.277588543185	-0.440989918845
-0.27750460579	-0.440698533738
-0.277419078012	-0.44064768269
-0.277316247544	-0.440628927007
-0.277223872587	-0.44058575507
-0.277150316873	-0.440222252309
-0.277082715608	-0.440014412558
-0.276985386239	-0.439964339465
-0.276896835509	-0.43996335712
-0.276796909398	-0.439774114744
-0.276700220232	-0.439737307803
-0.276617634042	-0.439720841432
-0.276540903753	-0.439706706685
-0.276453762339	-0.439621410263
-0.276357047191	-0.439584174711
-0.276262221362	-0.439524437025

-0.276186444066	-0.43950571712
-0.276099212597	-0.439499554057
-0.276014588998	-0.439486606868
-0.275935653517	-0.43925784276
-0.275857569046	-0.439210242163
-0.27576173428	-0.439149393418
-0.275681552979	-0.439098114166
-0.275577926162	-0.438814071847
-0.275503115523	-0.438750994046
-0.275432412187	-0.438466234372
-0.275327280453	-0.438447095699
-0.275247733195	-0.438342062265
-0.275156404426	-0.438290818774
-0.275051187034	-0.43819029769
-0.274961293744	-0.438108699196
-0.274875746552	-0.438012036393
-0.274785100731	-0.437947319133
-0.274715218233	-0.437943226496
-0.274622097727	-0.437822988559
-0.274552365762	-0.437452042536
-0.274464938806	-0.437318124835
-0.274373261346	-0.437116674656
-0.274282790402	-0.437071955365
-0.274189557269	-0.437054905153
-0.27411946768	-0.437039622762
-0.274038995742	-0.437017311056
-0.27395183036	-0.436983508535
-0.273858338485	-0.43674713873
-0.273781937777	-0.436618595591
-0.273702972787	-0.436607803722
-0.273626686216	-0.436356314697
-0.273537840073	-0.436279744973
-0.273429148417	-0.436265590123
-0.273339308412	-0.436198235966
-0.27325645876	-0.436103752442
-0.273163894597	-0.436046276277
-0.27308945067	-0.435758231141
-0.273005360952	-0.43573191322
-0.272900492706	-0.435702643741
-0.27280496428	-0.435632095324
-0.27271933374	-0.435601726373
-0.272627873182	-0.435522878816
-0.272545949627	-0.435511440208
-0.272457542221	-0.435414727581
-0.272380135204	-0.435377621924
-0.272299628441	-0.435148907224
-0.272222760089	-0.435057065778
-0.27212661773	-0.434964392139
-0.272052373037	-0.434902169518
-0.271953142424	-0.434739874178
-0.271857294733	-0.434444893457
-0.271761435785	-0.434372306283
-0.271662578591	-0.434347470589
-0.271563550008	-0.43424385185

-0.271479742474	-0.434239051399
-0.271412736866	-0.434157189328
-0.271317003496	-0.433988958482
-0.271247602283	-0.433822786726
-0.271172393797	-0.433708980338
-0.271086711146	-0.433644712372
-0.271008263124	-0.433347462696
-0.270926004591	-0.433256114902
-0.270824751074	-0.433193512512
-0.270751112872	-0.433158617747
-0.270672785846	-0.43300224056
-0.270578990007	-0.432996262784
-0.270494508565	-0.432953536058
-0.27042630394	-0.432946796666
-0.270339645701	-0.432915855163
-0.270247064456	-0.432903504157
-0.270153672081	-0.432870689825
-0.270056736163	-0.432805195706
-0.269970722761	-0.432647329942
-0.269885049364	-0.432641147744
-0.26980138445	-0.432602005658
-0.269709034065	-0.432341091939
-0.269606769651	-0.432248716192
-0.269542538193	-0.432226602259
-0.269445030061	-0.432216077606
-0.269371970177	-0.43220443884
-0.269278928713	-0.432008113875
-0.26918563818	-0.431988741969
-0.269100806898	-0.431984151625
-0.269020199572	-0.431909277602
-0.268949847634	-0.431902888166
-0.268860737839	-0.431672133069
-0.268781526407	-0.431462898804
-0.26870202051	-0.431448548538
-0.268612678069	-0.431316643107
-0.268530266447	-0.431313080073
-0.268456548673	-0.431285814493
-0.268368590822	-0.431028279041
-0.268293467643	-0.431024499179
-0.268212001436	-0.430927227939
-0.268141756806	-0.430784975809
-0.268050014365	-0.430717819301
-0.267975161569	-0.430704512658
-0.267886573717	-0.430679193334
-0.267809076867	-0.430454076826
-0.267722851558	-0.430324473717
-0.267645956226	-0.43030891721
-0.267562969632	-0.430032169971
-0.267480613521	-0.429973007894
-0.267385682925	-0.429805376432
-0.267318632264	-0.429759840701
-0.267222433059	-0.429607309319
-0.267147768368	-0.429466143798
-0.267061228826	-0.429440139739

-0.266978922923	-0.429423867137
-0.266904275102	-0.429179147608
-0.26682049657	-0.429120019507
-0.266740048422	-0.429066128327
-0.266645299377	-0.428899462488
-0.266555629169	-0.428838913861
-0.266473520118	-0.428821632219
-0.266378832375	-0.42856378389
-0.266302600866	-0.428366645864
-0.266197475284	-0.428270408801
-0.266089074714	-0.428224891042
-0.265994257754	-0.42811162964
-0.265879846174	-0.427828592367
-0.265803141273	-0.427732049359
-0.2657252076	-0.427689012625
-0.265648313768	-0.427668320157
-0.265573694702	-0.427665005802
-0.26548724223	-0.427540902739
-0.265382013254	-0.427448775361
-0.265294103028	-0.427407127621
-0.265210136749	-0.427334246197
-0.265133737218	-0.427281307188
-0.265056182985	-0.42694610553
-0.26497304039	-0.426887242915
-0.26488403263	-0.426744947054
-0.264814269881	-0.426609451418
-0.264739731833	-0.426540578426
-0.264653250302	-0.426461614969
-0.264570008003	-0.426448509479
-0.264481244381	-0.42643571508
-0.264391055296	-0.426329396739
-0.264317241931	-0.426179131367
-0.264226243925	-0.426121896912
-0.264126568327	-0.42606981676
-0.264028793042	-0.425771956226
-0.263946092392	-0.42566531979
-0.263848508887	-0.42550539617
-0.263771161994	-0.425347204395
-0.263699825358	-0.425199655633
-0.263624574528	-0.425118566572
-0.263539319554	-0.425101336274
-0.263478360357	-0.425002767967
-0.263405627477	-0.424956347827
-0.263329201604	-0.424899225688
-0.263246163676	-0.424888358914
-0.263161147209	-0.42480715341
-0.263068239157	-0.424405420868
-0.262989604048	-0.424398541427
-0.262910234851	-0.424396660619
-0.262822800433	-0.424374369465
-0.262718485176	-0.424357157889
-0.262642789334	-0.424349241549
-0.262553044795	-0.424331197935
-0.262480275109	-0.424293448478

-0.262386227983	-0.424256234273
-0.26230208747	-0.424130701043
-0.262221837381	-0.42406666492
-0.262125614049	-0.424055585155
-0.262016239433	-0.423733712023
-0.261925220451	-0.423716772576
-0.261837019556	-0.423548598443
-0.261764744519	-0.423547165561
-0.261691173198	-0.423341792504
-0.261609124624	-0.423204715783
-0.261526147364	-0.423151543004
-0.2614323245	-0.423092580375
-0.261356102355	-0.423079515876
-0.261272123015	-0.42296555044
-0.261196606662	-0.422850567667
-0.261111513421	-0.422797932621
-0.261020870937	-0.422640900846
-0.260927665146	-0.422537080322
-0.260837862988	-0.422434760199
-0.260752213227	-0.42241562736
-0.260667740624	-0.422337808626
-0.26057987367	-0.422299670168
-0.260506460146	-0.422240489294
-0.260420118207	-0.42204963622
-0.260325415065	-0.421683447476
-0.260229267028	-0.421628982773
-0.260139789032	-0.42155848888
-0.260053626909	-0.421519309981
-0.259978507964	-0.421514687922
-0.25989880071	-0.421271426427
-0.259818444384	-0.421182301013
-0.259740332491	-0.421119499322
-0.259640023855	-0.421070325744
-0.259549064968	-0.420874038159
-0.259466014866	-0.420751133956
-0.259404267731	-0.420650814464
-0.259303123918	-0.420580325031
-0.25922691437	-0.420536331932
-0.259147063534	-0.420504319078
-0.259045160683	-0.420483310853
-0.258952431912	-0.420167402265
-0.258863148179	-0.420082528864
-0.258770818428	-0.420059930344
-0.258689201189	-0.419980878812
-0.258607758806	-0.419897774517
-0.258525130649	-0.419847856789
-0.258447244685	-0.419736755138
-0.258349178494	-0.419732972202
-0.258272016626	-0.419491910016
-0.258185500845	-0.419333677627
-0.258093617226	-0.419316154168
-0.257982498886	-0.419312867772
-0.257889421894	-0.419271521952
-0.257793988205	-0.419185898052

-0.257699629394	-0.419104457051
-0.257622155527	-0.418979557732
-0.257527537818	-0.41883759974
-0.257427679815	-0.418807564666
-0.257339602961	-0.418741407578
-0.257243450168	-0.418486986682
-0.257146514804	-0.418442516718
-0.257045860462	-0.418419847104
-0.256959583867	-0.418401313969
-0.256846092328	-0.418388115591
-0.256754980256	-0.418359719353
-0.256679074819	-0.418298941987
-0.256584928819	-0.418269075233
-0.256498830865	-0.418259353479
-0.256412452883	-0.418252058726
-0.256327476985	-0.418224540708
-0.256253579961	-0.418002402253
-0.256168460429	-0.417975746008
-0.25608729375	-0.417889840852
-0.256000081105	-0.417867522116
-0.255908963777	-0.417815901124
-0.255819574133	-0.417583684685
-0.255730400392	-0.417532032409
-0.255659938636	-0.417424056855
-0.255545299865	-0.417403744918
-0.255465677391	-0.417397991519
-0.255401252355	-0.417347779903
-0.25531805323	-0.417014701441
-0.255233946459	-0.416996038974
-0.255159736409	-0.416979218346
-0.255056000076	-0.416902325169
-0.254968155061	-0.416864948125
-0.254895868296	-0.416486495347
-0.254813790791	-0.416446584395
-0.254738087796	-0.41643300888
-0.254659181364	-0.416425713864
-0.254573192724	-0.416347217203
-0.254483034626	-0.416324580749
-0.254378150093	-0.416268060558
-0.25430106663	-0.416232361883
-0.254194198127	-0.41618646599
-0.254111706191	-0.416105658264
-0.254025780437	-0.416047901562
-0.253948779232	-0.416047735232
-0.253859613859	-0.416021727006
-0.253777327303	-0.416004822777
-0.253709553295	-0.415960821087
-0.253625627329	-0.41544869074
-0.253537073208	-0.415409740431
-0.253446424504	-0.415326265904
-0.253366487626	-0.415326070165
-0.253270168252	-0.415258494412
-0.253184883793	-0.414869322068
-0.253092880596	-0.414844557758

-0.25302309483	-0.414678371636
-0.25294251627	-0.414662956001
-0.252855902508	-0.414588737654
-0.25277602166	-0.414574015368
-0.25268782544	-0.414537825411
-0.252613487277	-0.414503170249
-0.252532980776	-0.414446933137
-0.2524544116	-0.4143735236
-0.252366947225	-0.414343504071
-0.252283031974	-0.414335284917
-0.252194556746	-0.414273024647
-0.252112637839	-0.414206206681
-0.252023573717	-0.413934196547
-0.251942783587	-0.413921011386
-0.251881971965	-0.413896973069
-0.251799719474	-0.413801769135
-0.251713375184	-0.413780963589
-0.25160133894	-0.413572093484
-0.251516037781	-0.413526715233
-0.251419228294	-0.413526033555
-0.251344899822	-0.413454551376
-0.251278684939	-0.413374866609
-0.251197015094	-0.413234870546
-0.25111711029	-0.413220743326
-0.251029232849	-0.413185741458
-0.250924416316	-0.41317078011
-0.250829123047	-0.413151282244
-0.250738646233	-0.413078272861
-0.250656296637	-0.412896478502
-0.250572069144	-0.412851451762
-0.250495426119	-0.412769493356
-0.250408423615	-0.41274305422
-0.250320879157	-0.412683945604
-0.25022445412	-0.41251829632
-0.250143621131	-0.412486334013
-0.250063961333	-0.412434904802
-0.249989859981	-0.412360614909
-0.249895550589	-0.412288232131
-0.249799668181	-0.412026666024
-0.249720056736	-0.411956438385
-0.2496418223	-0.411903800322
-0.249552754063	-0.411452767558
-0.249470130669	-0.411392228933
-0.249395471894	-0.411370676949
-0.2493014228	-0.411119067569
-0.249216434696	-0.41106805962
-0.249118123439	-0.410926428556
-0.249025271915	-0.410785947042
-0.24895622512	-0.410596345478
-0.248858956136	-0.410571459486
-0.248781889338	-0.410393876047
-0.248694732884	-0.410334009292
-0.248613501035	-0.410320147521
-0.248524230589	-0.4103021546

-0.248425099766	-0.410222396275
-0.248339055056	-0.410194883121
-0.24825450869	-0.410163886513
-0.248164287009	-0.41012682469
-0.248048463954	-0.410046464919
-0.247953813775	-0.410018568346
-0.247863198618	-0.409912657912
-0.247761150719	-0.409840505574
-0.247682701316	-0.409817518864
-0.247583420291	-0.409729937104
-0.247494937725	-0.409545758129
-0.247421942026	-0.409377448671
-0.247339318257	-0.409332050131
-0.247257720578	-0.409089862429
-0.247181589463	-0.408926437468
-0.247105951942	-0.408688177646
-0.247038946042	-0.408630548715
-0.2469526802	-0.408507005733
-0.246869153557	-0.408489191002
-0.246776728708	-0.408428921978
-0.246696021006	-0.408276780555
-0.246616976895	-0.408149718871
-0.246517314679	-0.40811748906
-0.246425286921	-0.408058928239
-0.246356533928	-0.407973789335
-0.24625760107	-0.407961271448
-0.24617241889	-0.407941931541
-0.246089081559	-0.407890168173
-0.245999052938	-0.407822865982
-0.245904336491	-0.407757119519
-0.245821929328	-0.407751508787
-0.245742278847	-0.407707660721
-0.245657409039	-0.407700884188
-0.245578583319	-0.407203279325
-0.245510404226	-0.406789732623
-0.24543556409	-0.406580443455
-0.245347236257	-0.406565360731
-0.245264090554	-0.40640957427
-0.245188036575	-0.406292421535
-0.245113622117	-0.406250730829
-0.245030684615	-0.40624150598
-0.244957046298	-0.406124802072
-0.244867902076	-0.40604893994
-0.244796099014	-0.405854223684
-0.244720825004	-0.405764887571
-0.244644310062	-0.405755962302
-0.244562789831	-0.405695483325
-0.244485631361	-0.405640440829
-0.244405570902	-0.40555584459
-0.244324746047	-0.405490751147
-0.24424775595	-0.405369484219
-0.24414288963	-0.405041917688
-0.244065259383	-0.405031385427
-0.243991791241	-0.405011444589

-0.243896539846	-0.404785441066
-0.243808241318	-0.404568514707
-0.24371982715	-0.404555386254
-0.243628874013	-0.404412627607
-0.243552430855	-0.404357880687
-0.24347469869	-0.404280679576
-0.243389964014	-0.404268412936
-0.243304178526	-0.404257867041
-0.243201923751	-0.404111965288
-0.243112675604	-0.403981245872
-0.243036707251	-0.403926619204
-0.242954083351	-0.403907908382
-0.242854800881	-0.403712527887
-0.242755341561	-0.403649353762
-0.242671220652	-0.403637495913
-0.242581316759	-0.403373193334
-0.242498215686	-0.403365043721
-0.242415620053	-0.403328228709
-0.24232770832	-0.403191691277
-0.242247390905	-0.403134307907
-0.242146488409	-0.403120353949
-0.242045753072	-0.403104125396
-0.241965774302	-0.403062447517
-0.241873974295	-0.402993045336
-0.241778521683	-0.402877952468
-0.241690482444	-0.402617548576
-0.241610606469	-0.402281518085
-0.2415283904	-0.402227911924
-0.241457234078	-0.402212711473
-0.241359500341	-0.402136880547
-0.241255270816	-0.401947291672
-0.241167855273	-0.401888322781
-0.241093828153	-0.401637712216
-0.241004874818	-0.40141506425
-0.240909541144	-0.401374280123
-0.240812671221	-0.401336869073
-0.240730142292	-0.401271885547
-0.240635634988	-0.401153648384
-0.240539914202	-0.401058320731
-0.24046395566	-0.400862264496
-0.240386336835	-0.400835525287
-0.240304413896	-0.400698657927
-0.240211619008	-0.400503426651
-0.240110764368	-0.400421225723
-0.240027303755	-0.400370567105
-0.23995108851	-0.400226169116
-0.239871143016	-0.400162222974
-0.239795245612	-0.400085306092
-0.239712327535	-0.400070759396
-0.239617074345	-0.399939724009
-0.239529677284	-0.399890497328
-0.23942960573	-0.399788750387
-0.239340452409	-0.399573496666
-0.239257948685	-0.399499212149

-0.239164929066	-0.399491430014
-0.239083734537	-0.399403548292
-0.23900622324	-0.399328190436
-0.2389233157	-0.399038797824
-0.23883474247	-0.398981675561
-0.238771394893	-0.398910601326
-0.238687150949	-0.398752011756
-0.238587259233	-0.398497036317
-0.238492307166	-0.398325907091
-0.238404989589	-0.398061424943
-0.238314022507	-0.397971464978
-0.238233247278	-0.397938193029
-0.238161342446	-0.397924914112
-0.238060326316	-0.397853603021
-0.237971056718	-0.397818620066
-0.237880720451	-0.397702555629
-0.237796813577	-0.397664625192
-0.23771779706	-0.397560708222
-0.237629056431	-0.397549427936
-0.237534315128	-0.397545616076
-0.237440700774	-0.397513629935
-0.237356872247	-0.397417115515
-0.237268264464	-0.397287094443
-0.237181343938	-0.397085954061
-0.237093748506	-0.396876753384
-0.237012782657	-0.396648666205
-0.23692117914	-0.396516443916
-0.236833737225	-0.39640847075
-0.236761019624	-0.396126928724
-0.236670754641	-0.395746522184
-0.236595365404	-0.395604128599
-0.236503614146	-0.395287759924
-0.236418902824	-0.395273082394
-0.236344938989	-0.395051831077
-0.236259920364	-0.394975017273
-0.236189709848	-0.394941519187
-0.236109852977	-0.394719794448
-0.23602933195	-0.394666321923
-0.235940633283	-0.394660056932
-0.235850733573	-0.394610561852
-0.235767142131	-0.394550575844
-0.235699669668	-0.394374863874
-0.235618686685	-0.394370040844
-0.235525166854	-0.394229367379
-0.235412895337	-0.394188908094
-0.235326714018	-0.394130974999
-0.235224493736	-0.394086142204
-0.235127169181	-0.394058587846
-0.235022356556	-0.394027547731
-0.234926053332	-0.393883629003
-0.234842668388	-0.393862819003
-0.234750113327	-0.393841138019
-0.234650875663	-0.3938128198
-0.234570361796	-0.393636748064

-0.23447276117	-0.393550046637
-0.234387032464	-0.393502287573
-0.234318329939	-0.393320069449
-0.234238384966	-0.393189247918
-0.234155042415	-0.393094213514
-0.234077545138	-0.393075817958
-0.233994590965	-0.393049847262
-0.233901731601	-0.392838844549
-0.233817912489	-0.392834874531
-0.233729847945	-0.392707784166
-0.233642242666	-0.39263171837
-0.233538397089	-0.392538682363
-0.233454258204	-0.392523351763
-0.233375002756	-0.39245572921
-0.233287437928	-0.392193302073
-0.233217055942	-0.392085813027
-0.233131119585	-0.392047040796
-0.233054577415	-0.3920409713
-0.2329630457	-0.392040099115
-0.232871856953	-0.391848642269
-0.232785023744	-0.391543522385
-0.232695399087	-0.391236977625
-0.232614376716	-0.391089167203
-0.232509966435	-0.391037368001
-0.232420718726	-0.391005090335
-0.232344806292	-0.390880431795
-0.232258693691	-0.390779889734
-0.232179779938	-0.390753078213
-0.23209611456	-0.390603687969
-0.232012604198	-0.390453509678
-0.231930498744	-0.390368819371
-0.231820151686	-0.390311985915
-0.231722330835	-0.390185546342
-0.231637846618	-0.3899652519
-0.231546550361	-0.389769700723
-0.231464629672	-0.389732428178
-0.231390218389	-0.389646501635
-0.231307757871	-0.389591909529
-0.231215162892	-0.389392541397
-0.231134280501	-0.38939253664
-0.231035863853	-0.389386302783
-0.230961541897	-0.389121858265
-0.230872822521	-0.389092031251
-0.2307945104	-0.388901627015
-0.230714419992	-0.388890588932
-0.230617228411	-0.388854609298
-0.23052381634	-0.388786010903
-0.230444799044	-0.388641429985
-0.230381830731	-0.388432972457
-0.230293084221	-0.388333337437
-0.23021359213	-0.388300152255
-0.230123917207	-0.388273917973
-0.230041865322	-0.388143490275
-0.229959335554	-0.387880813078

-0.229873642851	-0.387749441847
-0.229777015116	-0.387712689673
-0.229682584097	-0.387450140555
-0.229607720412	-0.387340662499
-0.229527249501	-0.387230436952
-0.229448839625	-0.386938930513
-0.229363783321	-0.386823159404
-0.229273981583	-0.386797736421
-0.229207978345	-0.386779261057
-0.22914368412	-0.386725742046
-0.229063414131	-0.386715049877
-0.228976307358	-0.386611661908
-0.228894325909	-0.386594228368
-0.228802063624	-0.386594120921
-0.22870542556	-0.38649754719
-0.228625433676	-0.386487561044
-0.228527223488	-0.386482180902
-0.228433943723	-0.386449966378
-0.228346887595	-0.386423643399
-0.228269823494	-0.385970949822
-0.22817291673	-0.385828692861
-0.228086422597	-0.385207988007
-0.227990083155	-0.385060105601
-0.227901397155	-0.384899287687
-0.227802869715	-0.384884404101
-0.227701935345	-0.384878877774
-0.227621732215	-0.384836055416
-0.227539668058	-0.384768401934
-0.227445783856	-0.384460440718
-0.227365183431	-0.384421919682
-0.227283073866	-0.384296510124
-0.22717740302	-0.384259410098
-0.227080855844	-0.384188267386
-0.227002465857	-0.383877291115
-0.226899621402	-0.383639695565
-0.226812615048	-0.383465270498
-0.226735021435	-0.383450528046
-0.226639017094	-0.383332174743
-0.226557112383	-0.382821816274
-0.226480060317	-0.382464108125
-0.226393733826	-0.382205972969
-0.226319333059	-0.38217709574
-0.226231816458	-0.382169375237
-0.226130851264	-0.38216886158
-0.226030608321	-0.382150277725
-0.225945480471	-0.381886306033
-0.225848599812	-0.381877974969
-0.225771881509	-0.381768017592
-0.225697058794	-0.381664009166
-0.225612227526	-0.381629408299
-0.225518725913	-0.381628843042
-0.225435122321	-0.381455156897
-0.225351809486	-0.381401392209
-0.225267555862	-0.381376746712

-0.225192755952	-0.381341107401
-0.225103205914	-0.381313035787
-0.225002941624	-0.381258226418
-0.224906388805	-0.381197690172
-0.224818245471	-0.381182375644
-0.224739741534	-0.381117557399
-0.224660749082	-0.381090388311
-0.224588853415	-0.381063964514
-0.224500289861	-0.381058861518
-0.224429388184	-0.380927361867
-0.224341088928	-0.380509401112
-0.22426139303	-0.380363258159
-0.224170827973	-0.380290012832
-0.224092988705	-0.380168298952
-0.223994799337	-0.380096759138
-0.223909396787	-0.3795200639
-0.223832065447	-0.379294100013
-0.223769545098	-0.37919295449
-0.223690563504	-0.379143653053
-0.223615120813	-0.378821161588
-0.223538454971	-0.378787134157
-0.223451708521	-0.378785105612
-0.223364724242	-0.378743475576
-0.223270931131	-0.378454058122
-0.223190798276	-0.378383702145
-0.223112932978	-0.378265879164
-0.223035078273	-0.378247697157
-0.222945159853	-0.378241363873
-0.22285125995	-0.378135591376
-0.222763464749	-0.37809408369
-0.222667637756	-0.37770604884
-0.22257771544	-0.377279267652
-0.22249180275	-0.377052662325
-0.222399771795	-0.3768435656
-0.222325287995	-0.376686622559
-0.222240055149	-0.376682780587
-0.22214999478	-0.376674902083
-0.222064849917	-0.376579601662
-0.22197817	-0.376461053939
-0.221892227275	-0.376344833615
-0.22179832287	-0.376271303762
-0.221710088914	-0.376230469679
-0.221602860777	-0.376043658182
-0.221517023914	-0.375972439177
-0.221426977702	-0.375963727073
-0.221342309274	-0.37590619512
-0.221261218411	-0.375675558795
-0.221179743039	-0.375584389805
-0.221105779725	-0.375575636256
-0.22099988686	-0.375551935591
-0.220924160435	-0.375506987707
-0.220827756019	-0.375428515131
-0.220750883732	-0.375372605167
-0.220654046652	-0.375354529041

-0.220583379127	-0.3753235473
-0.220489768894	-0.375177340269
-0.220388277251	-0.37496283252
-0.220313129958	-0.37496102316
-0.22022276267	-0.374942604774
-0.220135466533	-0.374908217743
-0.220072912483	-0.374813883989
-0.219996187654	-0.374632491168
-0.219924335234	-0.37458189187
-0.219835845312	-0.374529857652
-0.219768822458	-0.374377355058
-0.219692211106	-0.374202260596
-0.219607827914	-0.374040191972
-0.219529067632	-0.374034697301
-0.219437233027	-0.37381739261
-0.219356379241	-0.373667352737
-0.219269961356	-0.373632080538
-0.219169426348	-0.37359503423
-0.219081443463	-0.373591157333
-0.219004132089	-0.373449394705
-0.218940216551	-0.373398336366
-0.218852677718	-0.373371821742
-0.218773474759	-0.373369332949
-0.218685085465	-0.373266043792
-0.218613058158	-0.373131190814
-0.21853659995	-0.372885542234
-0.218460934748	-0.372713395462
-0.218372531397	-0.372558926788
-0.218295517135	-0.372480253294
-0.218203786875	-0.372419415966
-0.218119863122	-0.372408194867
-0.218036740146	-0.372396982192
-0.217944011794	-0.372365452438
-0.217860060151	-0.372349663655
-0.217774669122	-0.372182097357
-0.217687746857	-0.372125174543
-0.217594359846	-0.372022551685
-0.217511123843	-0.371991019746
-0.217427881891	-0.371819753007
-0.217356845414	-0.371803217393
-0.217260713733	-0.371728674637
-0.217165491893	-0.371531201803
-0.217076724407	-0.371445254502
-0.216980041785	-0.37109217426
-0.216902642044	-0.370958004252
-0.216814917863	-0.370543539072
-0.216724409988	-0.370473230212
-0.216638513275	-0.370422619022
-0.216554489331	-0.370302415888
-0.216472249593	-0.370288750703
-0.216384213029	-0.370241322071
-0.216300371872	-0.370117516104
-0.216205025588	-0.370111639128
-0.216128200217	-0.370044685763

-0.216043918504	-0.370015718585
-0.215958872775	-0.369960837604
-0.215869884113	-0.369902561418
-0.215774249639	-0.36975945936
-0.21568827638	-0.369746469032
-0.215609946354	-0.369363396069
-0.215539263257	-0.369318855717
-0.215459182209	-0.369295579597
-0.215375570923	-0.369232070955
-0.215298584165	-0.369125709943
-0.215221819191	-0.369050447685
-0.21513921536	-0.369031379131
-0.215043978331	-0.368983851123
-0.214960925719	-0.36893011632
-0.214881307147	-0.368776177411
-0.21479126553	-0.368345748861
-0.214723513812	-0.368329498865
-0.214629501048	-0.368315134699
-0.214549530793	-0.368193488239
-0.214462317903	-0.368127969777
-0.214383699052	-0.36785852061
-0.214293115907	-0.367705719971
-0.21421053855	-0.367455072378
-0.214128242861	-0.367424237639
-0.214058254546	-0.367378188876
-0.213985627426	-0.367340852309
-0.213911751835	-0.367195099154
-0.213835425736	-0.36717821901
-0.213738114943	-0.367098018318
-0.213639644695	-0.366998163023
-0.213551304622	-0.366979147265
-0.213475388242	-0.36678854308
-0.213403530746	-0.366776177757
-0.213319203644	-0.366761141747
-0.213250310636	-0.366747635675
-0.213157948962	-0.366682196612
-0.213071033484	-0.366641015671
-0.212987251134	-0.366583245462
-0.212905614925	-0.366578076503
-0.212844899333	-0.36617507839
-0.212747107881	-0.366133059446
-0.212659808581	-0.365980160197
-0.212557369163	-0.365857562392
-0.212454963999	-0.365751706797
-0.212381422724	-0.365735577739
-0.212290467204	-0.365670602911
-0.21216679148	-0.365444466371
-0.212086997157	-0.365109988462
-0.211999710876	-0.365109579079
-0.211926796264	-0.365079162271
-0.211841831845	-0.364983470846
-0.211749670104	-0.364945901713
-0.211672465519	-0.364655546521
-0.211588414446	-0.364619305027

-0.211493174611	-0.364530281689
-0.211405913939	-0.364512422888
-0.211324036111	-0.364466750703
-0.211223445388	-0.364453067693
-0.211146312583	-0.364145803597
-0.211053960899	-0.364069664958
-0.210970164096	-0.364049767787
-0.21085750092	-0.363860630299
-0.210768963614	-0.363496978889
-0.21069760415	-0.363489738605
-0.210613086848	-0.363416802984
-0.210530520164	-0.363190551403
-0.210429851714	-0.363081316641
-0.210342946098	-0.36304256655
-0.210259032882	-0.362915798438
-0.210183028266	-0.362897951664
-0.210104465549	-0.362823573565
-0.210019959721	-0.362701205188
-0.209929310548	-0.36269824785
-0.209857372716	-0.362643320172
-0.209760898242	-0.362600476228
-0.209677495794	-0.362531128134
-0.209588594283	-0.362042994637
-0.20949291733	-0.361865668427
-0.209409115307	-0.36171213002
-0.209320557564	-0.361670440168
-0.20922435597	-0.36166108416
-0.20914411732	-0.361602009747
-0.209051057367	-0.361597936204
-0.208975995049	-0.361564510078
-0.208900362706	-0.361474744972
-0.208833410731	-0.361328735187
-0.208763831318	-0.361295652548
-0.208671272622	-0.361232423263
-0.208589146218	-0.36104730492
-0.208509497246	-0.360981036528
-0.208430546707	-0.360870006574
-0.208330973875	-0.360778393299
-0.20823837621	-0.36037542123
-0.208158924696	-0.360292707553
-0.208065517885	-0.360275193743
-0.207959400052	-0.360198469289
-0.207875166543	-0.360197811629
-0.207794343566	-0.360183996259
-0.207696606459	-0.359940674154
-0.207608944743	-0.359724549891
-0.207511043973	-0.359538697616
-0.207432403787	-0.359483507007
-0.207346943358	-0.359379567738
-0.207245426557	-0.359311740525
-0.207162201915	-0.359302253859
-0.20707374254	-0.359294579181
-0.206986615645	-0.359164357488
-0.206904419918	-0.359107439585

-0.20682111911	-0.359074028747
-0.206737614952	-0.358964728853
-0.20664958213	-0.35884800889
-0.206570483043	-0.358770897467
-0.206485840208	-0.358720974551
-0.206408392127	-0.358601000135
-0.20632275821	-0.35828244176
-0.206250267239	-0.358010672427
-0.206174706631	-0.357990809146
-0.206081504697	-0.357847088461
-0.205995913322	-0.357689574451
-0.205911583656	-0.357651717237
-0.205841561181	-0.357585669986
-0.205762088691	-0.357395251939
-0.205666820032	-0.357380260535
-0.205567646409	-0.357334664093
-0.205483520517	-0.357305875671
-0.205396877005	-0.357248572672
-0.205311397567	-0.357234881644
-0.20522036749	-0.357223128824
-0.205121505622	-0.357164546762
-0.205038306632	-0.357145468486
-0.204942896487	-0.357137513811
-0.204849561148	-0.356920260885
-0.204754881871	-0.356690718942
-0.204662439405	-0.356655908815
-0.204571572006	-0.3563996951
-0.204465985936	-0.356390488238
-0.20439073897	-0.356286585455
-0.204315782524	-0.356274947986
-0.204237059134	-0.35614878904
-0.204150188465	-0.356060025657
-0.204063194949	-0.355844363231
-0.203969669417	-0.355699922642
-0.203890767524	-0.355630264516
-0.203806863221	-0.355517246775
-0.203724456356	-0.355513931606
-0.203643006318	-0.355482833586
-0.203553553418	-0.355453121984
-0.203453146916	-0.355331520439
-0.203360710601	-0.355203279103
-0.203282685811	-0.355121554145
-0.203194271898	-0.354992524785
-0.203116770647	-0.354976581534
-0.203020245267	-0.354819356897
-0.202936754496	-0.354609733359
-0.202856352532	-0.354573098374
-0.202772680582	-0.354535928749
-0.202686710711	-0.354526708565
-0.202571774582	-0.354511908794
-0.202462469504	-0.354511522747
-0.202374325222	-0.354322335072
-0.202292518379	-0.354258510095
-0.202216281733	-0.354060190887

-0.202138973828	-0.353665553509
-0.202062098946	-0.353661729143
-0.201978055855	-0.353572334436
-0.201886470838	-0.353554406479
-0.20180899583	-0.353503072165
-0.201738847158	-0.353473792131
-0.201639322179	-0.353441454779
-0.201545054231	-0.353438588211
-0.20146180492	-0.353413370109
-0.20136739593	-0.353398030918
-0.201273578088	-0.353175183609
-0.201194943596	-0.35303562963
-0.20110295616	-0.352955212378
-0.201022413599	-0.352702978398
-0.200933875332	-0.352651691078
-0.200866722317	-0.352509612142
-0.200780055603	-0.352411610903
-0.200697770015	-0.352371759105
-0.200606624779	-0.352260271659
-0.200515973344	-0.351747785644
-0.200437745749	-0.351711301204
-0.200364807139	-0.351452351124
-0.200282183533	-0.351450133333
-0.200202296603	-0.351317483402
-0.200127207433	-0.351189273632
-0.200023921346	-0.351060335419
-0.199922707437	-0.35102557926
-0.199838474697	-0.350973802135
-0.199748715739	-0.350901904547
-0.199670151623	-0.35088960036
-0.199601086363	-0.35088478292
-0.199512846842	-0.350862996946
-0.199426192055	-0.350813489845
-0.199350690855	-0.350781641116
-0.199271218633	-0.35077275956
-0.199194513256	-0.350568668778
-0.199122833003	-0.35044847871
-0.199036069721	-0.350328561861
-0.198940021696	-0.350185457258
-0.198840677509	-0.350105676914
-0.198764597311	-0.350090119577
-0.19867150365	-0.349968877517
-0.198599610159	-0.34979308774
-0.198511651009	-0.349733954087
-0.198421512347	-0.349703408735
-0.198328343698	-0.34970018726
-0.198232919766	-0.349621518785
-0.19814748112	-0.349618142581
-0.19805715439	-0.349612429801
-0.197969761846	-0.349593726664
-0.197883917449	-0.349500423006
-0.197789519981	-0.349328610975
-0.197704647692	-0.349186403235
-0.197616673208	-0.349113821341

-0.197512816085	-0.348992699727
-0.197407108387	-0.348654607577
-0.197318099212	-0.348383948255
-0.197228178602	-0.348096643501
-0.197155274838	-0.348072537799
-0.197078560641	-0.348063084544
-0.19699389069	-0.347968880624
-0.19690784541	-0.3477764879
-0.196829141103	-0.347544945757
-0.196733252491	-0.347494470514
-0.196640710351	-0.347465281513
-0.196545831181	-0.347437972815
-0.196459021614	-0.347436784476
-0.196397514546	-0.347426366157
-0.196316905061	-0.347391390813
-0.196221567846	-0.347382454755
-0.196141179912	-0.347312226113
-0.196067648173	-0.347156657179
-0.19598021836	-0.347149482264
-0.19588008064	-0.34695194547
-0.195800790553	-0.346775796047
-0.195711870343	-0.34654900685
-0.195625500135	-0.34654090478
-0.195551396524	-0.346514842108
-0.195464572208	-0.346466135255
-0.195359307766	-0.346392346574
-0.195258549484	-0.346174372081
-0.195169698317	-0.346162099739
-0.195087526188	-0.346147726816
-0.194994220041	-0.346077940105
-0.194910857706	-0.346029391427
-0.194827336493	-0.34598119861
-0.194741477642	-0.345944708043
-0.194655606609	-0.345882976849
-0.194557711552	-0.345866809536
-0.194475869694	-0.34571122456
-0.194410962065	-0.345675665159
-0.19433002932	-0.345645124558
-0.194254501764	-0.345570195369
-0.19417135996	-0.345466291072
-0.194104527196	-0.345437830809
-0.194022622508	-0.345124622992
-0.193940505408	-0.345014700517
-0.193847726892	-0.344966737147
-0.193755369074	-0.344834941239
-0.193667921702	-0.344739317145
-0.19359996459	-0.344660049171
-0.193502331388	-0.344650113255
-0.193417037253	-0.344429442387
-0.193343337022	-0.344323998934
-0.193248547938	-0.344099157249
-0.193147471085	-0.344064259773
-0.19305726628	-0.343961727084
-0.192973073966	-0.343931674478

-0.192885027825	-0.343855977859
-0.192808130425	-0.343854340391
-0.192720456457	-0.343822165761
-0.192631060863	-0.343755360758
-0.192538789418	-0.343686419562
-0.192426020692	-0.343593642593
-0.19233586641	-0.343443103151
-0.192242415787	-0.34343151974
-0.192160727608	-0.343305739022
-0.192062134602	-0.343255339613
-0.191950795242	-0.343152895392
-0.191841609697	-0.342940362209
-0.191754073238	-0.342754705492
-0.191650505076	-0.342717572674
-0.191574214787	-0.342470683011
-0.191499217917	-0.34226924131
-0.191398983028	-0.342218541145
-0.191313252446	-0.342089086838
-0.191230855492	-0.34205388979
-0.191140959493	-0.342053456129
-0.191045859671	-0.341899734886
-0.190957813385	-0.341798978523
-0.190873354222	-0.341697626392
-0.190792436015	-0.341640979023
-0.190713048502	-0.34161923991
-0.190631966939	-0.341417922184
-0.190559576131	-0.341414950314
-0.190467992478	-0.341348824106
-0.190395485834	-0.341325910254
-0.190295742102	-0.341267002943
-0.19020376682	-0.341256092815
-0.190113583347	-0.341186908311
-0.190027183151	-0.340851251425
-0.189938633717	-0.340776151881
-0.189841776911	-0.340484544704
-0.189757868406	-0.340422451591
-0.189668398548	-0.340300083194
-0.189590327642	-0.340269286638
-0.189509375245	-0.340180163976
-0.189419457301	-0.340031930086
-0.189335592802	-0.340012747679
-0.18925260507	-0.339957942779
-0.18917301479	-0.339926034199
-0.189080467676	-0.339888918723
-0.188994142602	-0.339870978991
-0.18890993237	-0.339869183911
-0.188827671591	-0.339852592058
-0.188737454849	-0.33984824696
-0.188660313831	-0.339826164071
-0.18858033854	-0.339478929318
-0.188499664749	-0.339456886699
-0.188420778879	-0.339392517648
-0.188340425918	-0.33938690963
-0.188267350125	-0.33936859991

-0.188179692676	-0.339201792716
-0.188091171394	-0.339159094954
-0.188006299639	-0.339085524557
-0.18793965366	-0.338994067372
-0.187849905963	-0.338839296709
-0.187769503116	-0.338735405934
-0.187670199158	-0.338629040681
-0.187581848807	-0.338571054272
-0.18748600981	-0.338476177495
-0.187391418495	-0.338431340787
-0.187319760709	-0.338379444228
-0.187228750606	-0.338335951826
-0.187146959535	-0.338298553429
-0.187052605826	-0.33816474401
-0.186973426476	-0.338098243032
-0.186896317649	-0.338060087818
-0.186797562127	-0.33793977146
-0.186714897615	-0.337911620175
-0.186625655052	-0.33790535906
-0.186555914749	-0.337776799098
-0.186470950464	-0.337736739256
-0.186389895838	-0.337661621799
-0.186301696742	-0.337591024724
-0.186225746474	-0.337578739045
-0.186142684979	-0.33740628429
-0.18605531754	-0.337374656172
-0.185958891453	-0.337355619171
-0.18586644817	-0.337305048146
-0.185796511438	-0.337256483232
-0.185718154783	-0.337159029679
-0.185634148258	-0.337036604319
-0.185560679417	-0.336451302314
-0.185456583467	-0.336384345342
-0.185385085463	-0.336344878528
-0.18530088641	-0.336171475734
-0.185220451501	-0.336110896297
-0.185151115264	-0.336058318489
-0.185072708294	-0.335893144532
-0.184991780122	-0.335846037824
-0.184905159264	-0.335637385753
-0.184836661179	-0.335622913673
-0.184733138628	-0.335599176307
-0.18464479768	-0.335416735953
-0.184544385207	-0.335385307144
-0.184459610541	-0.335366295292
-0.184387941142	-0.335365887398
-0.184313370158	-0.335228129192
-0.184232916715	-0.335012190041
-0.184147640691	-0.334872997215
-0.184075837146	-0.334814577172
-0.183993805246	-0.334666291176
-0.183913102322	-0.334529711585
-0.183835621981	-0.334491751993
-0.183759855255	-0.334430916786

-0.183664328812	-0.334398014364
-0.183561740825	-0.334324868971
-0.183482984741	-0.334317674203
-0.183382357248	-0.334163475824
-0.183304283618	-0.334162772375
-0.18321834421	-0.334125731085
-0.183134739553	-0.334021695707
-0.183038323688	-0.33395729497
-0.182961543972	-0.333855540622
-0.182872812998	-0.333661741149
-0.182791435597	-0.333531481587
-0.18270020339	-0.33347790011
-0.182624354547	-0.333402698065
-0.182540328131	-0.333382354209
-0.18246021424	-0.333361856981
-0.18238184522	-0.333104618133
-0.182306000456	-0.332893506676
-0.182232690338	-0.332890509958
-0.182151027077	-0.332875610077
-0.182058194235	-0.332789492168
-0.18198420627	-0.332684964655
-0.181912997711	-0.332396106743
-0.181839707233	-0.331995840202
-0.181758716296	-0.331988042986
-0.18166052095	-0.331948792076
-0.181570099761	-0.331893137635
-0.181486895856	-0.331864307422
-0.181413655651	-0.33176308613
-0.181328141056	-0.331708015634
-0.181235103899	-0.331418234657
-0.181131774477	-0.331411933672
-0.181050067492	-0.331373071286
-0.180962188637	-0.331339333871
-0.180870166951	-0.331242311494
-0.18077812361	-0.331170439029
-0.180680883243	-0.331056849153
-0.18059214244	-0.331043964371
-0.180513598507	-0.331006917215
-0.180426920104	-0.330989061969
-0.180344489132	-0.330531024687
-0.180250185949	-0.330504205455
-0.180167863104	-0.330334281667
-0.180089125432	-0.330301357349
-0.180000947595	-0.330260396373
-0.179929615222	-0.33000894355
-0.179849347187	-0.329944277383
-0.179773891885	-0.32986620225
-0.179693049686	-0.32974150922
-0.179607416281	-0.329581370972
-0.179507700325	-0.329574822695
-0.179426167191	-0.329566812975
-0.1793162686	-0.329484463587
-0.179242010908	-0.329435875897
-0.179160018526	-0.329325340799

-0.179076007894	-0.3292854362
-0.178993528454	-0.328949831388
-0.178919270334	-0.328789834943
-0.178835368652	-0.328557433125
-0.178731750889	-0.328544664048
-0.178637913874	-0.328260253055
-0.17854287288	-0.328189376338
-0.178474859556	-0.328188550695
-0.178387554485	-0.327787823769
-0.178313977229	-0.327770295784
-0.178230181994	-0.327663763059
-0.178148891112	-0.327638592288
-0.178044657142	-0.327631239655
-0.1779586288	-0.327521100988
-0.177886349374	-0.327402801265
-0.17778623657	-0.327282211293
-0.177686805927	-0.327196789768
-0.177598213216	-0.327183181879
-0.177523669799	-0.326998359155
-0.177424716901	-0.326948663785
-0.177338871457	-0.326930435468
-0.177259528996	-0.326918681534
-0.177168711818	-0.32689979747
-0.177079677876	-0.326810416888
-0.176983727917	-0.326778187167
-0.176900486841	-0.326724723767
-0.176824421708	-0.326645965432
-0.176750667778	-0.326574237235
-0.176665775998	-0.326483306214
-0.176585521366	-0.326470094664
-0.176484180398	-0.326450840909
-0.176397965669	-0.326292554057
-0.176319039918	-0.326183793826
-0.176241580389	-0.326162432139
-0.176174814476	-0.326053271553
-0.176104719673	-0.325775923081
-0.176001519856	-0.325753831919
-0.175907692489	-0.325640332613
-0.175822769684	-0.325630685637
-0.175745807437	-0.325565163348
-0.175649842375	-0.325430710159
-0.17556129906	-0.325333039437
-0.175483195636	-0.325203963353
-0.175409132826	-0.325062866145
-0.1753247119	-0.325018795505
-0.175238696333	-0.324929490733
-0.175154393014	-0.324918830769
-0.175069033539	-0.324870407729
-0.174983117281	-0.3248333754
-0.174891304688	-0.324832349233
-0.174810035323	-0.324784581644
-0.17472518093	-0.324406833651
-0.174618479858	-0.324256536831
-0.174536364543	-0.323714942604

-0.174445335985	-0.32369817467
-0.174361873168	-0.323648871847
-0.174281707342	-0.323605884802
-0.174192575481	-0.323565440571
-0.174115887095	-0.323542345193
-0.174026867065	-0.323525030338
-0.173938284745	-0.323507734714
-0.17384697166	-0.323501004528
-0.173749578811	-0.323400355253
-0.173684423327	-0.323316867503
-0.17358134119	-0.323238196381
-0.17349409862	-0.323142901072
-0.173415988375	-0.323120745837
-0.173333195909	-0.323019377456
-0.173244760931	-0.322988094393
-0.173167085002	-0.322915788339
-0.173102269415	-0.322742240999
-0.173003802029	-0.322516414418
-0.172916215578	-0.322436127352
-0.172832298656	-0.322068212607
-0.172743376292	-0.321886501155
-0.172659435062	-0.321793915841
-0.172582781346	-0.321585692235
-0.172505140006	-0.321142317489
-0.172426441204	-0.320998842515
-0.172347122887	-0.320966677903
-0.172268077601	-0.320923937037
-0.172175245981	-0.320893070843
-0.172096105221	-0.320856759782
-0.172017907078	-0.320825646508
-0.171929128338	-0.320798583295
-0.171845810055	-0.32067876567
-0.171772508665	-0.320552057249
-0.171679723269	-0.320446356527
-0.171604520801	-0.320436626443
-0.171505031855	-0.320375949131
-0.171432041937	-0.320316334017
-0.171345934705	-0.320299193579
-0.171282986281	-0.320243596143
-0.171200787877	-0.319986036796
-0.171117388549	-0.319938194724
-0.171023779629	-0.319870917246
-0.170955691702	-0.319853566328
-0.17085886817	-0.319850626067
-0.170763522911	-0.319845747242
-0.170684865329	-0.319840656139
-0.170604979403	-0.319814405595
-0.170525155754	-0.319624838927
-0.170442079549	-0.319453231563
-0.170359204481	-0.319442916189
-0.170275717906	-0.319414144583
-0.170193551233	-0.319264275182
-0.170108458316	-0.319250817064
-0.170038205939	-0.319035261269

-0.169957455836	-0.319008455123
-0.169872856085	-0.318853835664
-0.169779337732	-0.318844464074
-0.169712316662	-0.318811629842
-0.169649726785	-0.318614925561
-0.169553701851	-0.318550731935
-0.16947489433	-0.318548890374
-0.169393008733	-0.31842584224
-0.169297491218	-0.318220379767
-0.169212527316	-0.3182186232
-0.169127952884	-0.317813351576
-0.169034096995	-0.317758560408
-0.16895253818	-0.317597448416
-0.168872898341	-0.317577525955
-0.168763029591	-0.317494073162
-0.168672849313	-0.317450990426
-0.168593457731	-0.317450809427
-0.16851670959	-0.317294089621
-0.16840956643	-0.317256865377
-0.168334055275	-0.317205757605
-0.168249068395	-0.317062851531
-0.168160826717	-0.317060117912
-0.168080120044	-0.317012877125
-0.168001303185	-0.31692300814
-0.167903676749	-0.316766914629
-0.167811730844	-0.316590143982
-0.167721297314	-0.316415018101
-0.167635436024	-0.316326276242
-0.167560494454	-0.316170611948
-0.167466265741	-0.316112406609
-0.167375783428	-0.316100340607
-0.167290113404	-0.315799066901
-0.167199479035	-0.315632589244
-0.167111869423	-0.315402888787
-0.167032816161	-0.315300521637
-0.166934395928	-0.315296549426
-0.166843539743	-0.315133869784
-0.166769626842	-0.314852754642
-0.16669451536	-0.314844240969
-0.166606749013	-0.314726658294
-0.166542432572	-0.314715362528
-0.166470524709	-0.314556324289
-0.166362093972	-0.314365708646
-0.166285369469	-0.314314706879
-0.166209168966	-0.314299369681
-0.166119228998	-0.313849059031
-0.166037002854	-0.313819620917
-0.165944927017	-0.313196088014
-0.165853122471	-0.3131085898
-0.165755991133	-0.313078679966
-0.165679365732	-0.312976605553
-0.165608831232	-0.312611615139
-0.165517251913	-0.312588915856
-0.165427898792	-0.312560118501

-0.165329664876	-0.312535397428
-0.165223550578	-0.312498457874
-0.165129274591	-0.312365345092
-0.16503459564	-0.312360906536
-0.164946959365	-0.312355861029
-0.164866487016	-0.312334692881
-0.164782571858	-0.312084365876
-0.164699859498	-0.312067718264
-0.164623553377	-0.311774265676
-0.164536066289	-0.311660582259
-0.164449067097	-0.311603644306
-0.164353690931	-0.311377826514
-0.164269584626	-0.311298529989
-0.164187793949	-0.311257460165
-0.164093095642	-0.311224403476
-0.164001178976	-0.311162779424
-0.16393648268	-0.311139976594
-0.16385122174	-0.311133874225
-0.163774540745	-0.311088262542
-0.163689690435	-0.311010204655
-0.16360874008	-0.310836005646
-0.16352723371	-0.310186609763
-0.163435041218	-0.310138591645
-0.163355911524	-0.310016301387
-0.163274238262	-0.309805988422
-0.163188695593	-0.309762693183
-0.163116747919	-0.309746550538
-0.163029054734	-0.309518199444
-0.162945261644	-0.309138734765
-0.162865118239	-0.309112331111
-0.162787158455	-0.308848625528
-0.162680616162	-0.308614520356
-0.162592260013	-0.307933088215
-0.16250851998	-0.307820671832
-0.162427003429	-0.307744750871
-0.162357105373	-0.307731552308
-0.162273662383	-0.307631235774
-0.162182511247	-0.30727439311
-0.162097805167	-0.307225772203
-0.162021804826	-0.307134538334
-0.161941515501	-0.307092788371
-0.161858901165	-0.307092503257
-0.161773428376	-0.307073439289
-0.161695351869	-0.3068780536
-0.161603401704	-0.306777245452
-0.16151770908	-0.306761994045
-0.16143227433	-0.306629275609
-0.161352707785	-0.306441569608
-0.161266365743	-0.306401516846
-0.161181780532	-0.30632419425
-0.161110501054	-0.306246485887
-0.161019335001	-0.306238276457
-0.160925394703	-0.306224114153
-0.160836964405	-0.306117887505

-0.160753434395	-0.30609929609
-0.160675283718	-0.305880219983
-0.160595616756	-0.305818109737
-0.160489270071	-0.305698691724
-0.160399962049	-0.305639439643
-0.160315189882	-0.305598556787
-0.160202130982	-0.305557870639
-0.160120794608	-0.305517787512
-0.16003662527	-0.30535472494
-0.159956347355	-0.305348487025
-0.159860437406	-0.305287863025
-0.159774023302	-0.305277363555
-0.159687251724	-0.305159528919
-0.159585594438	-0.305113892636
-0.159509523113	-0.305090589632
-0.159430821267	-0.304883791321
-0.159331892631	-0.304529136984
-0.159251373354	-0.304506803858
-0.159178353537	-0.304370734209
-0.159099514412	-0.304295436076
-0.159010974217	-0.304163327554
-0.158922619936	-0.304157329896
-0.158836123918	-0.303675183263
-0.158749355016	-0.303569968356
-0.158661037133	-0.303398316828
-0.158572727143	-0.30330255174
-0.158488631553	-0.303279231554
-0.15839251895	-0.303185397903
-0.15830645515	-0.303176873592
-0.158212759023	-0.303173776195
-0.158122433564	-0.302987658057
-0.158035205921	-0.302958044468
-0.157954818726	-0.302697049225
-0.15788808204	-0.302636036584
-0.157807973817	-0.302631359735
-0.157716749534	-0.302379654603
-0.157607510803	-0.302294813247
-0.157525726222	-0.302204776529
-0.157440157219	-0.302184195686
-0.157359343402	-0.302109759176
-0.157261241275	-0.301591234909
-0.157184525281	-0.301566483536
-0.157112445048	-0.301461545033
-0.157024020935	-0.301387685731
-0.156941598912	-0.301366615702
-0.156852061707	-0.301300302847
-0.156779892353	-0.301269497833
-0.15670499594	-0.301248758234
-0.156609737604	-0.301011857101
-0.156521753749	-0.300918600856
-0.156447690937	-0.300859479311
-0.156363333347	-0.300821515741
-0.156284184997	-0.300564230337
-0.156179141677	-0.300515438561

-0.156079620574	-0.300448846469
-0.156002880288	-0.300173654291
-0.155918772462	-0.300077616746
-0.155820369699	-0.300073511933
-0.155735802285	-0.299874867757
-0.155656115322	-0.299827676207
-0.155561051732	-0.29978027114
-0.155486350386	-0.299748497692
-0.155398175022	-0.299609801509
-0.155338056598	-0.299564048486
-0.155245069032	-0.299512719293
-0.155163533486	-0.29930781911
-0.15509029921	-0.299261222892
-0.155013859121	-0.299261137714
-0.154921608117	-0.298810375569
-0.154828526805	-0.29877402773
-0.154739021678	-0.298516291809
-0.15465610757	-0.298473399049
-0.154564301711	-0.298305626218
-0.15448421782	-0.298107563013
-0.154391607688	-0.297797487073
-0.154299965975	-0.29778474469
-0.154210116933	-0.29778089887
-0.15411970968	-0.297760795616
-0.154032509996	-0.297747063955
-0.153937977425	-0.297678574595
-0.153847837591	-0.29761488623
-0.153768559409	-0.297575317988
-0.153681724093	-0.297509016895
-0.153590655947	-0.297344989879
-0.153500090211	-0.297182354366
-0.153408243493	-0.297128617103
-0.153327101022	-0.296685003964
-0.153237428266	-0.296375885072
-0.153153682687	-0.296240268501
-0.153061166362	-0.296075353821
-0.152983868867	-0.29585830138
-0.152893102249	-0.295846664445
-0.152802973839	-0.295797345837
-0.15271261426	-0.295789903636
-0.152621441924	-0.295729974601
-0.152551634621	-0.295542522509
-0.152450260031	-0.295467241134
-0.152375680441	-0.295338181128
-0.152291238147	-0.295264203619
-0.152220029944	-0.295146790401
-0.152135659714	-0.295133635333
-0.152052824041	-0.295087027112
-0.151962104398	-0.294855161009
-0.15186989634	-0.294820692534
-0.151786331283	-0.29480101758
-0.151686090827	-0.294747454303
-0.151587032695	-0.294588843029
-0.151500882891	-0.294421458198

-0.151421812116	-0.294377644179
-0.15132956995	-0.294198458831
-0.151243375173	-0.294163500073
-0.151145230815	-0.294063460207
-0.151073288829	-0.293960759515
-0.150986201684	-0.293948408614
-0.150896100711	-0.293864572285
-0.150809641945	-0.293827712308
-0.150731124638	-0.293476406784
-0.150648386684	-0.293444643634
-0.150558042518	-0.293413282002
-0.150453870932	-0.293365976885
-0.150368549702	-0.293178786275
-0.150293809592	-0.293003016079
-0.150189785343	-0.292841301581
-0.150097532696	-0.292789618878
-0.150024500288	-0.292659717706
-0.149924957514	-0.292294611209
-0.149824010834	-0.292271108603
-0.149732427706	-0.292221270805
-0.149626490051	-0.292205652493
-0.149545135063	-0.29218015903
-0.149461588656	-0.29217076225
-0.149373984363	-0.292165270204
-0.149290662071	-0.292122947435
-0.149226252687	-0.292058517266
-0.149134518397	-0.291952544501
-0.149049335317	-0.29175252562
-0.148966714989	-0.291700450941
-0.148885535513	-0.291665260234
-0.148811104975	-0.291594486113
-0.14872495153	-0.2912008296
-0.148645767589	-0.291174055822
-0.148584672107	-0.291170304345
-0.14850402589	-0.291036924731
-0.148410050234	-0.290964237912
-0.148346990134	-0.290748341703
-0.148278096104	-0.290610127702
-0.148196277425	-0.290593318459
-0.148106768584	-0.290572063298
-0.148017163569	-0.290323501457
-0.14792832862	-0.290298847763
-0.147829765068	-0.290281200348
-0.147735285705	-0.290239778136
-0.147646079369	-0.290231441392
-0.14755592065	-0.290203132313
-0.147469846475	-0.289980703888
-0.147387276192	-0.289819937639
-0.147310718778	-0.289814029698
-0.147200348169	-0.289745886647
-0.147105883498	-0.289654725466
-0.147023691198	-0.289597207486
-0.146928787372	-0.289505231
-0.146830027675	-0.289465003122

-0.146747697077	-0.289379197471
-0.146652460274	-0.289379027713
-0.146570310042	-0.289235809781
-0.14649211485	-0.289160984438
-0.146398992024	-0.289116128905
-0.146302183332	-0.288943665698
-0.146224096934	-0.28889923278
-0.146151063136	-0.288887548537
-0.146057924782	-0.28882033767
-0.145985202636	-0.288773481977
-0.145917142859	-0.288420448667
-0.145826345041	-0.288207758214
-0.145736419679	-0.288073700039
-0.145657193941	-0.2880487117
-0.145561642827	-0.287851165214
-0.145465795818	-0.287822001484
-0.145385180681	-0.287611214491
-0.14530555662	-0.287523250454
-0.145217783712	-0.287492448551
-0.145116295924	-0.287443243083
-0.14501366553	-0.286895409655
-0.1449452172	-0.28687445904
-0.14485940614	-0.286820377273
-0.144780165015	-0.286765318506
-0.144693744005	-0.286750102085
-0.144610810534	-0.286739687081
-0.144510880118	-0.286718551019
-0.144416125684	-0.286660489274
-0.144318470947	-0.286614616989
-0.14424921286	-0.28658723341
-0.144160172793	-0.286575621639
-0.144067606113	-0.286446471589
-0.143987268097	-0.286421480719
-0.143893141785	-0.286078509901
-0.143808179913	-0.285917107782
-0.143720938861	-0.28584630501
-0.143642755049	-0.285599588034
-0.143557524786	-0.285593232334
-0.143467666024	-0.285484561281
-0.143395454237	-0.28541654043
-0.143313873628	-0.285343406069
-0.143230505117	-0.285310257859
-0.143130925126	-0.285236663538
-0.143049592754	-0.28521917105
-0.142952381114	-0.285096358751
-0.142868300107	-0.285081348478
-0.142778883232	-0.285019971111
-0.142695686729	-0.284965780242
-0.142612153525	-0.284772016533
-0.142530434633	-0.284714777565
-0.142460489163	-0.284630985371
-0.14236704645	-0.284381993151
-0.142287032833	-0.284143281601
-0.142192141635	-0.284069794378

-0.142117111515	-0.283923995168
-0.142013614911	-0.283874752581
-0.141919360363	-0.283860446791
-0.14183399771	-0.283793020213
-0.141754051356	-0.283709778713
-0.141676120349	-0.283554167769
-0.141585357929	-0.283543092918
-0.14150503153	-0.283423563099
-0.141432253804	-0.283086275274
-0.141349750833	-0.282846336489
-0.141277058727	-0.282836790129
-0.141192660691	-0.282723430739
-0.141111241585	-0.282710250731
-0.141026525126	-0.28271011155
-0.140936993503	-0.282570134181
-0.140850490155	-0.282464264764
-0.140759725068	-0.282409196183
-0.14066867884	-0.282404004075
-0.140592693751	-0.282399180698
-0.140509256507	-0.282306597709
-0.140426310159	-0.282278983199
-0.140340867189	-0.282233690626
-0.140262575329	-0.282199985442
-0.140167046077	-0.28218438431
-0.140084617685	-0.28217610475
-0.140015352007	-0.282066564879
-0.139942108461	-0.281895150732
-0.139850326372	-0.281814444066
-0.139765289929	-0.28173618676
-0.139683892239	-0.281623592497
-0.139575308962	-0.28157133817
-0.139490304801	-0.281466877816
-0.139400785883	-0.281151692534
-0.139313845828	-0.281097880338
-0.139226831623	-0.281088946825
-0.139156855935	-0.281002636866
-0.139070425982	-0.280912834699
-0.138977634275	-0.280907437217
-0.13891023029	-0.28039876452
-0.138820599643	-0.280334400833
-0.138744332253	-0.280310808167
-0.138667515546	-0.280220290683
-0.138586889532	-0.28018461811
-0.138514282131	-0.280156526634
-0.138428873229	-0.280093133511
-0.138360579195	-0.280063600678
-0.1382802379	-0.27977759901
-0.138212825014	-0.279641458322
-0.138125294179	-0.279618828521
-0.138046386743	-0.279544478199
-0.137960081153	-0.279520971494
-0.13787066143	-0.279318994251
-0.137773040598	-0.279271995267
-0.137695419389	-0.27920523577

-0.137597670064	-0.278969631175
-0.137507009477	-0.278946050699
-0.137414208119	-0.278872264266
-0.137314630471	-0.278801560671
-0.137232755621	-0.278665392278
-0.137151824273	-0.278616267117
-0.137073410235	-0.278557623622
-0.136996341286	-0.278379235147
-0.136909262355	-0.2783249833
-0.136808198189	-0.278316384921
-0.136725647264	-0.27813520901
-0.136638814618	-0.278104416925
-0.136528291632	-0.278076308801
-0.136438071622	-0.277981489801
-0.136341606461	-0.277979520186
-0.136248743773	-0.277872028046
-0.136177175705	-0.277856992575
-0.136087638967	-0.277664129365
-0.135999595678	-0.277509809062
-0.135907275361	-0.277457219878
-0.135821888794	-0.277413067439
-0.135739214432	-0.277240375776
-0.135647723593	-0.277233261952
-0.135558954301	-0.277231389578
-0.135463612413	-0.277157832176
-0.135390351366	-0.276582691008
-0.13532557863	-0.276518578784
-0.135235811575	-0.27641753707
-0.135151509714	-0.276144361563
-0.135052858053	-0.275880380894
-0.134973420019	-0.275828935291
-0.134878522714	-0.275798294313
-0.134789431449	-0.275769117749
-0.134703551386	-0.275690915117
-0.134607017695	-0.27568236767
-0.134528230291	-0.275605997208
-0.134464293632	-0.275582593463
-0.134372384613	-0.275542082958
-0.134298673114	-0.275531993278
-0.13420259432	-0.275485864109
-0.134101319974	-0.275321409075
-0.134001808319	-0.275254606487
-0.13390070935	-0.275163615945
-0.133814796289	-0.275146435877
-0.133733542836	-0.275141669594
-0.133660740979	-0.274917498049
-0.133563599279	-0.274881066803
-0.133474134065	-0.274841794057
-0.133396427192	-0.274840523205
-0.133324816551	-0.274789882548
-0.133233773546	-0.274767512555
-0.13313002408	-0.274743009895
-0.133058394658	-0.274724206549
-0.132978290446	-0.274473197342

-0.132886458292	-0.274347834084
-0.132800115359	-0.274326625386
-0.132720875451	-0.274307838155
-0.132635467417	-0.27428801323
-0.13255366911	-0.274256678331
-0.132474597302	-0.274130085093
-0.132393972291	-0.274061084994
-0.132313397424	-0.274054430042
-0.132229217472	-0.273997170275
-0.132139833686	-0.273993175538
-0.132054324938	-0.273825691307
-0.131976297325	-0.273780367573
-0.131874855124	-0.273698665685
-0.131800045086	-0.273540366228
-0.13172167572	-0.273437625557
-0.131631934956	-0.273357190372
-0.131558210483	-0.273346314035
-0.131483151058	-0.273339905779
-0.131384607297	-0.273309844416
-0.131292375337	-0.273152682016
-0.131204325464	-0.273092646976
-0.131113212507	-0.273041764189
-0.131026886018	-0.272982014687
-0.13095480722	-0.272937531916
-0.130874607562	-0.272721735057
-0.130798628669	-0.272621250702
-0.130703624027	-0.272405222775
-0.130621850308	-0.272336262449
-0.130544877151	-0.272320520355
-0.130470949649	-0.272207665365
-0.130377658441	-0.272070211116
-0.130288226786	-0.272056110594
-0.130214693847	-0.271808590727
-0.130115647126	-0.271566764113
-0.13003784053	-0.271550972667
-0.129947518104	-0.271538311513
-0.129865078434	-0.271525983334
-0.129779672159	-0.271501989447
-0.129699701487	-0.271404273924
-0.129626446841	-0.271370041298
-0.129541914259	-0.271348152823
-0.12946791174	-0.271348024463
-0.129375545632	-0.271338231136
-0.129297738767	-0.271313738832
-0.129205074649	-0.271207369041
-0.129118312185	-0.271192758265
-0.129049099627	-0.271149054399
-0.128984437769	-0.271048195855
-0.128900937702	-0.270859654604
-0.128811378093	-0.27084641626
-0.128730553076	-0.270634293022
-0.128651141198	-0.270608786814
-0.128555848207	-0.27027334567
-0.128473841517	-0.270244340037

-0.128367345095	-0.27006179822
-0.128292645292	-0.269848080762
-0.128227973319	-0.269768052072
-0.128139894958	-0.269761580951
-0.128046048304	-0.269630620377
-0.127966416392	-0.269549942304
-0.127871333574	-0.269527511164
-0.127782059978	-0.269495915069
-0.127691354616	-0.269314098284
-0.127603098616	-0.269235744579
-0.127513574138	-0.269006444939
-0.12742306556	-0.268672575843
-0.127356112261	-0.268537722796
-0.127270437781	-0.26848208714
-0.127202634225	-0.268217085097
-0.127106386968	-0.26817468821
-0.127032079538	-0.268101584322
-0.126921587459	-0.268093002838
-0.126828177132	-0.267990360089
-0.126733326155	-0.26792411063
-0.126641738565	-0.267869000616
-0.126540296152	-0.267649469654
-0.126453990143	-0.267602718958
-0.126348965495	-0.267363279726
-0.126253840723	-0.267282413582
-0.126163986629	-0.26719715358
-0.126078346173	-0.267168617429
-0.125987738683	-0.267062308214
-0.1259088054	-0.266968548354
-0.12581495222	-0.266936191856
-0.125727739901	-0.266912248491
-0.125657441418	-0.266817603367
-0.125569982728	-0.266658837015
-0.125489818011	-0.266545322698
-0.125397141346	-0.266444073987
-0.12530147936	-0.266294315435
-0.125206566516	-0.266281267622
-0.125144487656	-0.266280928132
-0.125059787828	-0.266191316965
-0.124979528463	-0.266094956995
-0.124878095685	-0.265960095034
-0.124786931779	-0.265899996513
-0.124700075521	-0.265495343356
-0.124610520403	-0.265392092456
-0.12452390178	-0.265219331518
-0.124431058684	-0.265150045671
-0.124348672433	-0.265148258713
-0.124254446789	-0.265002848359
-0.12418797066	-0.264742249765
-0.124099191914	-0.264676134274
-0.124007151853	-0.264656094175
-0.123927540235	-0.264490495842
-0.123845066507	-0.264442866597
-0.123759399609	-0.264422246979

-0.12367559094	-0.264370810281
-0.123593885585	-0.264165658434
-0.123519436215	-0.264165020513
-0.123439359255	-0.264125685843
-0.123353133906	-0.264065126166
-0.123266932449	-0.264056965625
-0.123192940754	-0.264029050295
-0.123117898086	-0.263999380326
-0.123040999248	-0.263989063877
-0.12293983709	-0.263935633615
-0.122857579217	-0.263926944212
-0.122785346049	-0.26387677583
-0.122694281944	-0.263632859734
-0.122601780017	-0.263628906889
-0.122519306847	-0.263614804277
-0.122447067145	-0.263553375878
-0.122366180408	-0.263363388363
-0.122268883647	-0.263354437811
-0.122198961229	-0.263325249607
-0.122111446561	-0.263206992059
-0.122024852389	-0.262986113367
-0.121936967809	-0.262933259068
-0.121857570447	-0.262668180598
-0.121777318168	-0.262666541984
-0.121676278763	-0.2623655637
-0.121594225807	-0.262220868611
-0.121504379174	-0.262025947276
-0.121406511852	-0.261920436072
-0.121326749662	-0.261846837288
-0.121240771909	-0.261757876338
-0.121175795397	-0.2617538902
-0.121080757596	-0.26168336466
-0.120994192764	-0.261602451134
-0.120913735472	-0.261566112455
-0.120848996135	-0.261521247753
-0.1207451287	-0.261446607478
-0.120667536507	-0.261289537591
-0.120581113596	-0.261066699036
-0.120502560873	-0.260981747177
-0.12043358454	-0.260784156963
-0.120362450287	-0.260608388971
-0.120278372676	-0.260375984103
-0.120182058003	-0.260240154409
-0.120107923144	-0.260032450717
-0.1200456319	-0.259802845662
-0.119952994231	-0.259420964278
-0.119878800953	-0.259359045588
-0.119801300361	-0.25929925604
-0.119707344483	-0.259179247693
-0.119635255967	-0.259153708505
-0.119548047496	-0.259021944304
-0.119462187543	-0.259020553409
-0.119380486417	-0.258793693276
-0.11929996612	-0.258786157915

-0.119220931586	-0.258770426972
-0.119131106341	-0.258635458036
-0.119042751223	-0.258625981702
-0.118969409125	-0.258409245132
-0.118895872856	-0.258274926823
-0.118810062868	-0.258215836354
-0.118716636038	-0.258138449957
-0.118613600123	-0.258099556171
-0.118535724451	-0.258029068454
-0.118456096344	-0.257925680756
-0.118365448912	-0.25784187013
-0.118271899063	-0.257813844019
-0.118188557297	-0.257707634443
-0.118116560821	-0.257641944712
-0.118035320059	-0.257594395108
-0.117947731413	-0.257592947334
-0.117855402843	-0.257363645612
-0.117780190485	-0.257313329855
-0.117696839177	-0.257258896301
-0.117602700198	-0.25716931936
-0.117510243333	-0.257108968203
-0.117418930561	-0.256776223531
-0.117345662305	-0.256619798864
-0.117259237276	-0.256556350636
-0.117181360262	-0.256555858266
-0.117100535923	-0.256442643478
-0.117008038721	-0.256423938897
-0.116921628785	-0.256410168712
-0.116835938494	-0.256386084845
-0.116740766889	-0.255852906796
-0.116657518477	-0.255814312962
-0.116582653169	-0.255807021239
-0.116489653119	-0.255792119802
-0.116400398298	-0.255756788289
-0.116310733664	-0.25572491288
-0.116221416803	-0.255619428808
-0.11614209463	-0.255383383008
-0.116071028576	-0.255069094619
-0.116002839828	-0.255001836256
-0.115920675764	-0.254951679741
-0.115834598233	-0.254820090885
-0.115746798766	-0.254759722412
-0.115652413748	-0.254752018463
-0.115570123082	-0.254178654757
-0.115486637454	-0.254175627203
-0.115398511237	-0.254157195911
-0.11532324431	-0.254024079632
-0.115237751852	-0.253807294558
-0.115135709061	-0.253636376555
-0.115064955633	-0.25363049748
-0.114992937913	-0.253572036497
-0.114905093703	-0.253554754054
-0.114824167565	-0.253547976574
-0.114758821223	-0.253444532705

-0.114655938549	-0.253406766798
-0.114575300549	-0.253226189686
-0.114489387425	-0.253188219386
-0.114390134428	-0.25290125203
-0.114302553802	-0.252809829633
-0.114227256511	-0.252758650202
-0.114146955607	-0.252586912753
-0.114071259617	-0.252580027735
-0.113999309021	-0.252462556557
-0.113909692533	-0.252406101323
-0.113827540016	-0.252326877489
-0.113737620586	-0.252267298515
-0.113653044867	-0.251815610779
-0.11356577826	-0.251549433583
-0.113474203263	-0.251495625971
-0.113385895398	-0.25148995922
-0.113311397945	-0.251473405547
-0.113225958545	-0.251274716589
-0.11311546834	-0.251070085191
-0.113023298761	-0.251041155017
-0.112934242573	-0.250910627453
-0.112856274582	-0.250829777465
-0.112773698345	-0.250817349133
-0.11268425851	-0.250701474044
-0.112590617146	-0.250544236376
-0.112491862908	-0.250500661035
-0.112416259257	-0.250488317512
-0.112325001889	-0.250326079628
-0.112232215795	-0.250052789442
-0.11213358825	-0.250034972734
-0.112051367062	-0.249964126791
-0.111959840511	-0.249831633397
-0.111880397907	-0.249166257734
-0.111780697303	-0.249059843485
-0.111689465806	-0.249014773536
-0.111583222331	-0.248948715112
-0.111510684081	-0.248844907659
-0.111422131735	-0.248714418182
-0.111328146944	-0.24864533484
-0.111256695608	-0.248494013159
-0.111174927994	-0.248492355087
-0.111109128293	-0.248107908514
-0.111014615458	-0.248040923172
-0.110931329298	-0.248020812099
-0.110845080419	-0.247778296189
-0.110758543322	-0.24771792897
-0.110677591227	-0.24740054501
-0.11059711493	-0.247384748006
-0.110519579504	-0.24735978516
-0.110424878371	-0.247076560438
-0.110333374719	-0.247005120351
-0.110251912864	-0.246773414507
-0.110160195941	-0.246638806998
-0.110084673961	-0.246372622551

-0.110002810044	-0.246207026489
-0.109910397034	-0.246024137626
-0.109829124236	-0.245940300422
-0.109742940941	-0.24569210987
-0.109665512981	-0.245596713216
-0.109584550619	-0.245583755415
-0.109500688998	-0.245462580728
-0.109418594921	-0.245458678941
-0.109346583954	-0.245435631787
-0.109265988977	-0.245048681402
-0.109182318362	-0.245001474224
-0.109089810402	-0.244969536788
-0.109012301383	-0.244881546253
-0.108931868281	-0.244826541662
-0.108851603186	-0.244775247606
-0.108750017154	-0.244616676514
-0.10866043893	-0.244588752904
-0.108584092707	-0.244534135069
-0.108486108608	-0.244401900614
-0.108391817279	-0.244373704584
-0.108314451181	-0.24417388912
-0.108229661662	-0.244114598538
-0.108124850596	-0.244091244951
-0.108045754131	-0.244059964896
-0.107963994648	-0.24404759635
-0.107881720821	-0.243892971245
-0.107799341078	-0.24384441646
-0.107705946033	-0.243826454336
-0.107619897461	-0.243626350229
-0.107531502321	-0.243474341927
-0.107455054503	-0.243456552308
-0.107375082504	-0.243370926363
-0.107292517794	-0.243235810272
-0.107204547119	-0.243117038649
-0.10711589986	-0.24303836808
-0.107016645834	-0.242993700213
-0.106925803286	-0.242950079614
-0.106849383571	-0.242855948174
-0.106767462006	-0.242290944581
-0.106683239919	-0.242165396248
-0.106610120406	-0.241846419653
-0.106520468458	-0.24180292502
-0.106443372171	-0.241540547779
-0.106366604432	-0.241367924533
-0.106293332117	-0.241332245008
-0.106210386267	-0.241162471048
-0.106121540415	-0.241020530207
-0.106025593609	-0.240889983905
-0.105945086684	-0.240865991098
-0.105845931441	-0.240648460111
-0.105764075004	-0.240585935908
-0.105667217275	-0.240498576659
-0.10556952158	-0.23992202583
-0.105481916371	-0.239919020263

-0.10541172594	-0.23953380458
-0.105337846903	-0.239410189328
-0.105237670074	-0.239383229447
-0.105143764696	-0.239381810113
-0.105066700273	-0.239298729407
-0.104973474402	-0.239209323553
-0.104876842669	-0.239181822753
-0.10480058708	-0.239099305394
-0.104711537097	-0.238869941282
-0.104610033873	-0.238796037023
-0.104516886497	-0.238738564611
-0.104430814094	-0.238685237719
-0.104356262175	-0.238572980588
-0.104270340642	-0.238512248608
-0.104182377849	-0.23850323436
-0.104087151331	-0.238484490966
-0.103985528078	-0.238271564791
-0.103898870547	-0.238242502713
-0.103812323083	-0.23821833213
-0.103740943999	-0.238023592695
-0.103665342593	-0.238005763641
-0.103583785735	-0.237991761148
-0.103491474763	-0.237990483401
-0.103394951406	-0.237939428276
-0.103316225161	-0.23759347349
-0.103229010227	-0.237487413719
-0.103134501721	-0.237358501331
-0.103052071028	-0.237204850028
-0.102976558663	-0.237063533026
-0.10290257555	-0.237057272901
-0.102833401012	-0.236993869333
-0.102741844484	-0.236914476921
-0.102648106272	-0.236902703221
-0.102550287195	-0.236692989921
-0.102456586514	-0.236650675667
-0.102357283577	-0.236532519129
-0.102255912017	-0.236404979811
-0.102172537446	-0.236366536746
-0.102092858478	-0.236090860694
-0.102012646133	-0.236062054939
-0.101921139446	-0.236039847876
-0.101851754469	-0.23552641977
-0.10175409465	-0.235376966504
-0.101669096686	-0.235182518096
-0.101579960053	-0.235182166938
-0.101502063819	-0.235047204473
-0.101424315581	-0.235007197304
-0.101348126336	-0.235005886077
-0.101249735583	-0.234860439652
-0.101162668317	-0.234853584726
-0.101087469661	-0.234766154364
-0.100996126871	-0.234298186683
-0.100897930648	-0.234163702065
-0.100822198527	-0.234152205554

-0.100742473182	-0.234148266127
-0.100655017548	-0.234132655427
-0.100561460645	-0.234124640671
-0.100488841145	-0.234080969585
-0.100404557578	-0.233977035352
-0.100307876134	-0.233894124912
-0.100213955355	-0.233495739474
-0.100136814885	-0.233368445447
-0.100052374803	-0.233299304466
-0.099959228444	-0.233219980743
-0.099875238935	-0.233179469135
-0.099788557456	-0.233149098298
-0.099698951956	-0.233024527816
-0.099612036501	-0.232967877467
-0.099528508985	-0.23272661879
-0.099440714972	-0.232667724913
-0.099343869567	-0.232595396086
-0.099258473393	-0.232594503192
-0.099160795584	-0.232534154596
-0.099079247023	-0.23224570281
-0.09900120477	-0.232241571381
-0.098922900459	-0.232215808435
-0.098834729481	-0.232135745736
-0.098754532942	-0.232115803696
-0.098665332319	-0.231941538461
-0.098601445755	-0.231883562896
-0.09853052217	-0.231793051247
-0.098460194506	-0.231687022802
-0.098378430588	-0.231629625637
-0.098300102694	-0.231612036802
-0.098235272239	-0.231591022852
-0.098149161609	-0.23136417983
-0.098068473421	-0.23132347744
-0.097993070395	-0.231251654608
-0.097894684925	-0.23113593743
-0.097824295904	-0.230883648141
-0.097717835404	-0.230697028405
-0.097625246521	-0.230676695382
-0.097543075515	-0.230670826381
-0.097465928153	-0.230503966693
-0.097397584072	-0.230425853529
-0.097306171611	-0.230217009297
-0.09723193175	-0.230172365941
-0.097161393914	-0.230161926561
-0.097074927061	-0.230017435381
-0.096992028108	-0.229935205774
-0.096914656573	-0.229909643664
-0.096824884469	-0.229856117196
-0.096744625144	-0.22984285381
-0.096668553502	-0.229561674767
-0.096568963308	-0.229471531085
-0.096469473007	-0.229437055317
-0.096381714775	-0.229409764621
-0.096310571702	-0.22939704212

-0.096234620954	-0.229364587642
-0.096125898925	-0.229236811258
-0.09604438633	-0.229216384329
-0.095964428892	-0.229141973329
-0.095882583117	-0.229128791862
-0.095787601969	-0.228771470457
-0.095723935628	-0.228753181388
-0.095640422133	-0.228657014585
-0.095549892286	-0.228439047398
-0.095469586648	-0.228301411645
-0.095374632379	-0.228194255126
-0.095296513166	-0.228192378262
-0.095201969495	-0.22814259571
-0.095113857978	-0.22810828852
-0.095019734	-0.228016646132
-0.094937780366	-0.228013241019
-0.09484827535	-0.22798314052
-0.094749076671	-0.227941415161
-0.094664399254	-0.227867115413
-0.094577166574	-0.227773396392
-0.09449839308	-0.227648866142
-0.094418217633	-0.227535566952
-0.094330746201	-0.227139065209
-0.09424206414	-0.226945962549
-0.094172152716	-0.2268148049
-0.094088759031	-0.22662815313
-0.094006201414	-0.226568389745
-0.093890801593	-0.226550509257
-0.093809158851	-0.226521897743
-0.093728170977	-0.226191594503
-0.093641886526	-0.226175136166
-0.093563904562	-0.226084573272
-0.093491393273	-0.225986416784
-0.093416266232	-0.225984009761
-0.093335254467	-0.225954150671
-0.093268813627	-0.225852569888
-0.093184663318	-0.225609000516
-0.093111612872	-0.225517068572
-0.09301373339	-0.225512611206
-0.092942525178	-0.225501670394
-0.092855561721	-0.225418554333
-0.092765080396	-0.225230199777
-0.092681293839	-0.225225516111
-0.092600001855	-0.225028315838
-0.09251284996	-0.224998855003
-0.092435918943	-0.224989028287
-0.092340440894	-0.224971850506
-0.092236576105	-0.22478098031
-0.092158006607	-0.224092487495
-0.092077056795	-0.224054023344
-0.09196388856	-0.224033556731
-0.0918715972	-0.223978029491
-0.091766560635	-0.223960633271
-0.091686953755	-0.223896256355

-0.091606104712	-0.223482486489
-0.091507521093	-0.223403640925
-0.091414624499	-0.223273119862
-0.091334333736	-0.22319522347
-0.091249845906	-0.22307563047
-0.091155322612	-0.222781630581
-0.091081693491	-0.222717447559
-0.091008899353	-0.222667883808
-0.090936819536	-0.222382931667
-0.090842791036	-0.222330418527
-0.090763991164	-0.222319570388
-0.090696173698	-0.222290510287
-0.090624172684	-0.22219533414
-0.090549230589	-0.222042042933
-0.090447521167	-0.222035082803
-0.090347655932	-0.221996094789
-0.090259749275	-0.221947677071
-0.090188435109	-0.221871080816
-0.09009601286	-0.221793346248
-0.090021951192	-0.221766795186
-0.089943877521	-0.221410573055
-0.089863514066	-0.221223906633
-0.089788025975	-0.221176133735
-0.089711998932	-0.221126511781
-0.089634680689	-0.220798365924
-0.089539746985	-0.22057129927
-0.089458494207	-0.220439503016
-0.089372639766	-0.220285898795
-0.089290040184	-0.21998341983
-0.08920510393	-0.219934228528
-0.089124984734	-0.219721481522
-0.089046921323	-0.219676721856
-0.088947279027	-0.219647487241
-0.088867761219	-0.219622436736
-0.08878025107	-0.2195216075
-0.088695138414	-0.219324998099
-0.088617715176	-0.219313706721
-0.088542150892	-0.219180230691
-0.088459323954	-0.219155926794
-0.088374951098	-0.219035404048
-0.088297594385	-0.218996321635
-0.088212640853	-0.218959446954
-0.088131821676	-0.218747947977
-0.088068336103	-0.218678292463
-0.087976453607	-0.218615799352
-0.087907097083	-0.218360372358
-0.087809301963	-0.21827577287
-0.087738932647	-0.218061987921
-0.087653102085	-0.21788189141
-0.08757498784	-0.217811324553
-0.08749101804	-0.217724291161
-0.08741364601	-0.217646346335
-0.087335683268	-0.217393217133
-0.087264403484	-0.217391047092

-0.08718032162	-0.217289928159
-0.087101355804	-0.217010979909
-0.087016805112	-0.216914573967
-0.086947355243	-0.216870411378
-0.086872909294	-0.216841426914
-0.086784841641	-0.216766279134
-0.086699141708	-0.216734222135
-0.08662028074	-0.216560158484
-0.086531652474	-0.216522259145
-0.086453444529	-0.216348765194
-0.086362656983	-0.21629171547
-0.086268509591	-0.216218526318
-0.086185510096	-0.216198045514
-0.086108258413	-0.216068508052
-0.086011852699	-0.21587856354
-0.085933875974	-0.215772283269
-0.085844987146	-0.215440923802
-0.085760580933	-0.215262892433
-0.085693767342	-0.215189308607
-0.085596817064	-0.214972692262
-0.085513431162	-0.214613447893
-0.085412502189	-0.214556053144
-0.085337871636	-0.21455397648
-0.085235807343	-0.214341404769
-0.085127772492	-0.213968296245
-0.085048562752	-0.213888052876
-0.084960667684	-0.213867700389
-0.084883228129	-0.213771934879
-0.084795405961	-0.213757004589
-0.08470930295	-0.213672608481
-0.084633205923	-0.213631514937
-0.084553675486	-0.213504614037
-0.084473267895	-0.213462688868
-0.084379392136	-0.213299658623
-0.084293069061	-0.21282109984
-0.08420106418	-0.212550157542
-0.084111914449	-0.212476976879
-0.084029595147	-0.212425289212
-0.083961973557	-0.212290358779
-0.083892022871	-0.212203381149
-0.083815524661	-0.212163437543
-0.083720003167	-0.212154803303
-0.083633916346	-0.212126768776
-0.083533623512	-0.212016433552
-0.083446544506	-0.211922778776
-0.083372209722	-0.211766559396
-0.083291244069	-0.211538258735
-0.083216142208	-0.211459810665
-0.083135270961	-0.211434934149
-0.083060413821	-0.211427750802
-0.082965813933	-0.211372547088
-0.082864210155	-0.21132515943
-0.0827750852	-0.211115660127
-0.082693994353	-0.211023858215

-0.082599387189	-0.210945635296
-0.082515595692	-0.21093256929
-0.082426625946	-0.210895071786
-0.082331885782	-0.210870624775
-0.082220615163	-0.210833572762
-0.08212696068	-0.210823284377
-0.082057337686	-0.210339150357
-0.081964794581	-0.210060090348
-0.081890800044	-0.210043676345
-0.081818525778	-0.210038887904
-0.081747136045	-0.209805751798
-0.081663210066	-0.209768829085
-0.081568583424	-0.209726270506
-0.081487136228	-0.209661540017
-0.081394056109	-0.209473414627
-0.08130009635	-0.209440754939
-0.081223973474	-0.209430952456
-0.081131667432	-0.209422665011
-0.081037093832	-0.209165053017
-0.080945047094	-0.209012862066
-0.080874078458	-0.208986758643
-0.080795152403	-0.208664595578
-0.080722098085	-0.208556346987
-0.080636556671	-0.208414778523
-0.080545750715	-0.208298315054
-0.080473195957	-0.208227507383
-0.080363336173	-0.208115009779
-0.080280350247	-0.208098955105
-0.080202137807	-0.208098875617
-0.080110841201	-0.20809572515
-0.080012117369	-0.208016357261
-0.079923078382	-0.20800691358
-0.079839677433	-0.20799344986
-0.079745882827	-0.207888388428
-0.079646256965	-0.207868555907
-0.079555772577	-0.207798150399
-0.079476820199	-0.207753367512
-0.07938751505	-0.20768172601
-0.079300547085	-0.207627825772
-0.079222262498	-0.20756459774
-0.079145289754	-0.207547064186
-0.07906100127	-0.207347403613
-0.078987936778	-0.20728601249
-0.078892916425	-0.20726306303
-0.078808198737	-0.207220207923
-0.078728491065	-0.207219684126
-0.07861340479	-0.206988440792
-0.078519601093	-0.206963775755
-0.078420143227	-0.206956497609
-0.078330665286	-0.206812060237
-0.07825425749	-0.206774929821
-0.078179391305	-0.206580989713
-0.078086364223	-0.206175533361
-0.078011220612	-0.20610547872

-0.077931614076	-0.205625117064
-0.077841903664	-0.205541028884
-0.077761299475	-0.205443787237
-0.077672796325	-0.205383688123
-0.077577496691	-0.205367385053
-0.077494744697	-0.20536401305
-0.077421600922	-0.20534271623
-0.077342267298	-0.20525231341
-0.077261176629	-0.205126118114
-0.077172632058	-0.204915483687
-0.07708190849	-0.204901833247
-0.077007545383	-0.204562826817
-0.076917880756	-0.204494565205
-0.076814720364	-0.204403211134
-0.076732640931	-0.20438323892
-0.076651455905	-0.204239815988
-0.076570841973	-0.204221945608
-0.076476333775	-0.204212516146
-0.076391033927	-0.204158373583
-0.076320855352	-0.204039988745
-0.076235773057	-0.203947511913
-0.076155657143	-0.203904732732
-0.076062670267	-0.203853001387
-0.07597834908	-0.203781119119
-0.075898744318	-0.203717348074
-0.075816352755	-0.203631533155
-0.07573794024	-0.203452113158
-0.075652004536	-0.20337913951
-0.075574974858	-0.203331864985
-0.075490331197	-0.203324067646
-0.075406976822	-0.203314756929
-0.075327516835	-0.203218633231
-0.075241007347	-0.203204672394
-0.075149239006	-0.203005870685
-0.075060861231	-0.202930604074
-0.074970578336	-0.202899049147
-0.074876271649	-0.20286701412
-0.074798484	-0.202610181197
-0.074710340092	-0.202602619554
-0.07463776023	-0.202581215564
-0.074554944556	-0.202520727619
-0.074470301611	-0.202457908147
-0.074387441024	-0.202414698822
-0.074301767764	-0.202315102955
-0.074211703991	-0.202218154621
-0.074120354067	-0.20218348721
-0.074026951845	-0.202170020585
-0.073936866541	-0.202081427579
-0.073853757223	-0.202061098454
-0.073769773661	-0.202035707142
-0.073673347556	-0.201966691441
-0.073594078806	-0.201957798042
-0.073515482688	-0.201897012676
-0.073444075973	-0.201756594485

-0.073361229813	-0.201700464914
-0.07327339957	-0.201378505273
-0.073203937897	-0.20130895344
-0.07312448555	-0.200642874713
-0.073022851353	-0.200590026927
-0.072933852467	-0.200414053793
-0.072843731801	-0.200343555889
-0.072765635186	-0.200337584897
-0.072670769983	-0.200263427833
-0.072599654942	-0.200215194877
-0.072523694868	-0.200206432094
-0.072442312095	-0.200186690848
-0.072358973789	-0.200156909094
-0.072254711053	-0.200015314816
-0.072173148737	-0.199949377778
-0.072053179446	-0.199915218791
-0.071964883644	-0.199870297095
-0.071879155993	-0.19985763398
-0.071794689732	-0.199791958445
-0.071715560326	-0.199732444634
-0.071622013169	-0.199496597744
-0.071539728196	-0.199430965048
-0.071456136974	-0.199268644989
-0.071371396106	-0.199229360789
-0.07130230087	-0.199217857965
-0.071203362024	-0.199161907894
-0.071126091812	-0.199011536421
-0.071047877415	-0.198821428134
-0.070967481008	-0.198611964688
-0.070894529151	-0.198482604198
-0.070798195789	-0.198446864564
-0.070705884659	-0.198421661545
-0.070624089608	-0.198417137669
-0.0705387421	-0.198400225551
-0.070471843303	-0.19820275096
-0.070405215717	-0.1981342773
-0.070313313022	-0.198074504634
-0.070229339253	-0.19789380794
-0.070143902744	-0.197820721549
-0.070052639904	-0.197772484045
-0.069968859643	-0.197686764148
-0.069879872439	-0.197673296745
-0.069783457857	-0.197604091006
-0.069690475768	-0.197436466653
-0.069615198491	-0.197401054301
-0.069520976445	-0.197250796522
-0.069444902173	-0.197155972616
-0.069359853959	-0.197051230657
-0.069281113338	-0.19684072945
-0.069195697806	-0.196768718484
-0.069108910444	-0.196754325505
-0.069034910122	-0.196639694942
-0.068939527958	-0.196528559552
-0.06885589167	-0.196383080142

-0.068764789037	-0.196302273626
-0.068677871346	-0.196232089087
-0.068579431877	-0.196175742126
-0.068490941415	-0.19613074459
-0.068411472235	-0.196090072938
-0.068340172569	-0.196014303658
-0.068249552078	-0.195776979445
-0.068157633745	-0.195682908355
-0.0680727501	-0.195277908264
-0.067981254166	-0.195067660395
-0.067890310216	-0.19495355518
-0.067810817642	-0.194830370842
-0.067736240513	-0.194655367011
-0.067664708383	-0.194257949965
-0.067575315023	-0.194220867249
-0.067493041539	-0.194049475017
-0.067405898481	-0.193970506776
-0.067325576944	-0.193897217663
-0.067242317581	-0.193698074469
-0.067158645167	-0.193686352691
-0.067087969118	-0.193665177545
-0.066995436543	-0.193536662937
-0.066907921931	-0.193328247452
-0.066832415878	-0.193293551111
-0.066742630692	-0.192821761342
-0.066652667051	-0.192765546122
-0.066563256161	-0.19265741733
-0.066490315835	-0.192653813927
-0.066389779088	-0.192635062228
-0.066305074633	-0.192233887163
-0.06622131688	-0.192225904005
-0.066154034167	-0.192203303817
-0.066078980264	-0.192185976052
-0.066004405325	-0.192034898488
-0.065935818765	-0.191997474847
-0.065856631722	-0.191922536927
-0.06579086774	-0.191813716475
-0.06572291312	-0.191767394901
-0.065655986508	-0.191572585064
-0.065586282441	-0.190935796558
-0.06550201989	-0.190934709489
-0.065412683313	-0.190695272039
-0.065326862736	-0.190673596376
-0.065244382528	-0.19054510219
-0.065153255479	-0.190414824523
-0.065072056203	-0.190390231334
-0.064980897639	-0.190242149408
-0.064893446195	-0.189848603391
-0.064816900867	-0.189836723567
-0.064728570048	-0.189561090201
-0.064638838639	-0.189366137907
-0.064551606306	-0.189260788143
-0.064450714019	-0.189235245137
-0.064370405023	-0.189217381461

-0.064296093263	-0.189137661286
-0.064213415243	-0.189132368896
-0.064120264883	-0.189099278265
-0.064044701453	-0.188991446956
-0.063970484493	-0.188866789603
-0.063895549303	-0.188512825132
-0.063806715542	-0.188503510782
-0.063716645128	-0.188402628917
-0.063640517151	-0.18835408964
-0.06356405264	-0.188280532311
-0.063484359968	-0.188160651805
-0.063422100661	-0.188108968965
-0.063342360005	-0.188108731864
-0.063251350896	-0.188066405317
-0.063156865133	-0.188056313016
-0.063071363695	-0.18801713856
-0.062975133812	-0.18790596052
-0.06288388447	-0.187871898811
-0.062791925594	-0.187801536694
-0.062711306144	-0.187675280455
-0.062611707347	-0.187674369772
-0.062525015233	-0.187014382185
-0.062442279943	-0.186954391442
-0.062352494658	-0.186780464696
-0.062267832861	-0.186523082568
-0.062185123831	-0.186472773812
-0.062086366796	-0.186311848537
-0.062012229854	-0.186088567116
-0.061927239115	-0.186072151944
-0.0618325488	-0.185952428066
-0.061732728596	-0.185914497511
-0.061636804308	-0.185340459638
-0.061555133204	-0.185304786659
-0.061470696487	-0.185257086275
-0.061396193964	-0.185112903127
-0.061317257416	-0.184325046807
-0.061235090518	-0.18427996633
-0.061149215958	-0.184135955643
-0.061073469419	-0.184047502966
-0.060978750612	-0.183832845904
-0.06087827115	-0.183802813773
-0.060799145742	-0.183559714141
-0.060718065716	-0.183543453971
-0.060625586883	-0.183378110903
-0.060537924302	-0.183002293745
-0.060476410367	-0.18275338317
-0.060390627715	-0.182664633348
-0.060301201387	-0.18262233153
-0.060226497076	-0.182490379887
-0.060143678011	-0.182325468634
-0.060064324378	-0.182187451319
-0.059964921607	-0.182172092023
-0.059893678109	-0.182060998939
-0.059797170201	-0.182014630224

-0.059704836365	-0.181643417994
-0.059622038474	-0.181548701276
-0.059532162258	-0.181542455219
-0.05945256775	-0.181406217564
-0.059358635271	-0.181369615542
-0.059280418446	-0.181354212734
-0.059189814832	-0.181262172605
-0.059112022421	-0.181250637985
-0.05902904342	-0.181186155205
-0.05894031792	-0.18118510537
-0.058850745481	-0.181057518699
-0.0587621829	-0.181037689634
-0.058687796857	-0.181013866561
-0.058602092591	-0.180837280765
-0.058510577032	-0.180798644272
-0.058427481909	-0.180675977791
-0.058338925166	-0.180666101184
-0.05825293968	-0.180540982799
-0.058169819717	-0.180475263562
-0.058084244707	-0.180230980961
-0.058004839075	-0.180162874904
-0.057929962669	-0.180080572323
-0.057851455729	-0.18003277284
-0.057766776293	-0.180024536777
-0.057689444321	-0.180013691108
-0.057607846216	-0.179839876984
-0.05752607843	-0.179726250591
-0.057435814143	-0.179506189831
-0.057353080333	-0.179451736681
-0.057289288206	-0.179306949872
-0.057206169131	-0.179287538391
-0.057125781552	-0.179240341722
-0.057045185929	-0.179143171552
-0.056959386935	-0.179026497829
-0.056881502935	-0.179008123876
-0.056810685084	-0.178858058033
-0.056735133738	-0.178834379482
-0.056666114886	-0.178738661127
-0.056594238427	-0.178569117838
-0.056517693162	-0.178552020391
-0.056426280918	-0.178511518694
-0.056334009818	-0.178502165663
-0.056257244397	-0.178381740963
-0.056172206511	-0.178259531982
-0.056094743597	-0.178235827304
-0.056019205573	-0.17816958176
-0.055924970208	-0.178155562261
-0.055839446714	-0.178079995292
-0.055772246996	-0.178013144658
-0.055669540133	-0.177960881143
-0.055594724333	-0.177687141209
-0.055509757098	-0.177675075772
-0.055437155026	-0.17762210604
-0.055373234443	-0.177314655278

-0.055288646717	-0.177150834291
-0.05521885958	-0.177098945488
-0.055141936121	-0.177054752673
-0.055060297538	-0.177035424916
-0.054971083122	-0.176794893737
-0.054887633744	-0.176780525339
-0.054806878786	-0.176701325281
-0.054737372716	-0.176678333169
-0.054662897745	-0.17666105885
-0.054564124088	-0.176545126106
-0.054482843122	-0.176539154735
-0.054405821119	-0.17646161226
-0.05432093423	-0.17640735937
-0.054239225371	-0.176143910208
-0.054154033617	-0.176099821921
-0.054059824021	-0.176099794009
-0.053947913624	-0.176010504927
-0.053877158204	-0.175868925749
-0.053794601575	-0.175798971619
-0.053707871153	-0.175697219463
-0.053627891389	-0.175603801437
-0.053551185874	-0.17536967247
-0.053472176155	-0.175350769819
-0.053394530924	-0.175171730567
-0.053304244909	-0.175141008916
-0.053225161875	-0.174979612113
-0.053139462991	-0.174921503498
-0.053057668629	-0.174895377356
-0.052973879175	-0.174777417393
-0.052893932576	-0.174716808895
-0.052799617574	-0.174635483197
-0.052712926305	-0.174497787306
-0.052624043033	-0.174332919991
-0.05253356504	-0.174196092558
-0.052457095336	-0.174142997707
-0.052390119412	-0.174083694534
-0.052304223736	-0.1739997728
-0.052224814274	-0.17398369129
-0.052153234768	-0.173855900582
-0.052065802285	-0.173771436165
-0.051973549413	-0.173696490214
-0.051887247521	-0.173549622482
-0.051819679007	-0.173525782279
-0.051724218201	-0.173428136709
-0.051645427143	-0.173408192347
-0.051562445486	-0.173350899909
-0.051481075264	-0.17325073933
-0.051409938595	-0.173237729177
-0.051315965105	-0.17317007914
-0.05123793048	-0.173131848882
-0.051162545322	-0.173120463702
-0.051093033459	-0.172963253363
-0.05101209596	-0.172905465289
-0.050938692124	-0.172856395249

-0.050870388692	-0.172788051697
-0.050764984326	-0.172739930221
-0.050672614647	-0.172625239402
-0.050568261488	-0.172273911802
-0.050484527017	-0.172221226221
-0.050417178697	-0.171996185617
-0.050304952697	-0.171931911289
-0.050227777133	-0.171842330649
-0.050137958695	-0.171725185395
-0.05006192363	-0.171599564939
-0.049981708677	-0.171546529642
-0.049899901728	-0.171500446192
-0.049799754517	-0.17099835309
-0.049731044558	-0.170854291253
-0.049651286475	-0.170765407862
-0.049561925683	-0.170559760632
-0.049484807222	-0.170559699767
-0.049376303595	-0.170497584326
-0.049298391196	-0.170482612227
-0.049215308945	-0.170220093306
-0.049125895823	-0.170087856418
-0.049048459049	-0.170027749686
-0.048972276934	-0.170019397705
-0.048900699345	-0.169943175465
-0.048814725919	-0.169789569795
-0.04873690345	-0.169782986455
-0.048664499251	-0.169684703417
-0.048579053803	-0.169578210006
-0.048496345672	-0.169505294353
-0.048423147208	-0.169443863838
-0.048338684843	-0.169205512656
-0.048253115459	-0.169201187897
-0.04816341831	-0.168915369678
-0.04807764512	-0.168799897405
-0.047981547832	-0.168691829497
-0.047882417899	-0.168588226482
-0.04780711173	-0.168565995833
-0.047727854989	-0.168430380451
-0.047641063711	-0.168364030743
-0.047554417386	-0.168350487839
-0.04747912349	-0.168318066923
-0.047389533737	-0.168293785982
-0.047304356017	-0.16821013649
-0.04721851966	-0.167872826413
-0.047115799259	-0.167839235457
-0.047033652815	-0.167710273665
-0.046957926272	-0.167650364825
-0.046879794195	-0.167643800539
-0.046792438477	-0.167522434763
-0.046703409656	-0.167501307144
-0.046620296142	-0.167427366568
-0.046544820332	-0.167314857306
-0.046456699052	-0.167299778601
-0.046379610213	-0.167275271351

-0.046276225893	-0.167250395016
-0.046193180799	-0.166938073349
-0.046108926979	-0.166793881103
-0.046036861505	-0.166516026499
-0.045942392853	-0.166439406572
-0.045847911941	-0.16635864257
-0.045752107227	-0.166325683987
-0.045666918768	-0.166252881737
-0.04558472817	-0.16596564935
-0.045486704877	-0.16596302047
-0.045403341541	-0.165858595271
-0.045336185543	-0.165746604298
-0.045257041956	-0.165570083097
-0.04518298815	-0.165566825324
-0.045101693039	-0.165515193233
-0.045027549241	-0.165458276461
-0.044938686977	-0.165419885703
-0.044859440485	-0.165358534298
-0.044780154871	-0.165356193608
-0.04470928338	-0.165318039188
-0.044623330117	-0.165291321768
-0.044538565886	-0.165249620739
-0.044449825907	-0.164853023422
-0.044336833598	-0.164683765444
-0.044246094572	-0.164672637819
-0.044154221937	-0.164535228371
-0.044069055607	-0.164473056762
-0.043990953694	-0.164191344967
-0.043911529257	-0.164125843402
-0.043833740918	-0.164104422574
-0.043749491119	-0.16404290228
-0.043659507613	-0.163986316973
-0.043575812695	-0.163706665672
-0.043481061667	-0.163651307933
-0.043402382837	-0.163641446869
-0.043315824638	-0.16331955466
-0.0432341352	-0.162831276277
-0.043150430536	-0.162776397034
-0.043060729913	-0.162760291374
-0.042965027934	-0.16249433264
-0.042886755941	-0.162487274777
-0.04283420671	-0.162363704298
-0.042765796064	-0.162228653107
-0.042683650037	-0.162160805153
-0.042589810702	-0.161899646003
-0.042508516486	-0.161688215391
-0.042423473456	-0.161664467066
-0.042348036115	-0.161599100173
-0.042241779648	-0.161485263066
-0.042168378571	-0.161483268688
-0.042090546243	-0.161450515929
-0.042008323802	-0.161384680291
-0.041910079962	-0.161336914678
-0.041827886823	-0.161268358866

-0.041746324926	-0.161178282746
-0.041671830785	-0.161117108945
-0.041584739077	-0.161004474461
-0.041504928826	-0.160935795355
-0.041423131127	-0.160910022607
-0.041338897646	-0.160779365627
-0.041266434581	-0.160682926578
-0.041176391945	-0.160567264138
-0.041064616328	-0.1605597659
-0.040973007496	-0.160122522819
-0.040879922884	-0.160061108616
-0.040804690629	-0.160034668309
-0.040720543464	-0.159877845663
-0.040641949768	-0.159773687258
-0.040554957754	-0.159565555572
-0.040467595104	-0.159492796889
-0.040376378763	-0.159474734168
-0.040299189797	-0.159459857602
-0.040204231049	-0.159424376189
-0.040129669816	-0.159333850891
-0.040049918977	-0.159296628801
-0.039964605787	-0.158923412248
-0.039882036981	-0.158781194935
-0.03978358158	-0.158776770811
-0.039691222288	-0.15877123956
-0.03960301168	-0.158757719564
-0.039514739358	-0.158655729734
-0.039433208634	-0.158573539939
-0.039333807817	-0.158555281482
-0.039254443953	-0.158529349657
-0.039169714629	-0.158518681489
-0.039066638882	-0.158489571351
-0.038992762879	-0.158317593657
-0.038920152517	-0.158114841574
-0.03883060435	-0.158107178549
-0.038730672106	-0.158029413924
-0.038648243811	-0.157874502711
-0.038576115184	-0.157816261053
-0.038497092177	-0.157774584073
-0.038413874828	-0.157725341166
-0.038326343121	-0.15749806414
-0.038225144484	-0.157427045828
-0.038147984945	-0.15742626513
-0.038070979254	-0.15723384012
-0.037975137419	-0.157220228783
-0.037899606596	-0.157148427281
-0.037826042009	-0.157116571535
-0.037735199947	-0.157058280702
-0.037648503593	-0.156990563583
-0.037555562031	-0.156931774008
-0.037461459373	-0.156901717993
-0.037383055984	-0.15678007115
-0.037298495739	-0.156732981923
-0.037200294478	-0.156699514738

-0.037115454317	-0.156671044057
-0.037045374881	-0.15666887093
-0.036961862749	-0.15666264409
-0.036873785652	-0.15658028719
-0.036782340719	-0.156311663769
-0.036698065931	-0.155948842362
-0.036625770129	-0.155654462718
-0.036544598308	-0.155613802901
-0.03646283329	-0.155532874648
-0.036376457461	-0.15549321697
-0.036273485623	-0.155466922469
-0.036190514003	-0.155398425357
-0.036109031215	-0.155395281818
-0.036029593571	-0.155054644188
-0.035941371986	-0.154989051357
-0.035847666331	-0.154800830417
-0.035767708304	-0.154794187669
-0.03569348015	-0.154727108542
-0.035604269622	-0.154722980422
-0.035518436036	-0.1546398126
-0.03544686551	-0.15441486852
-0.035342310235	-0.154344232172
-0.035247007037	-0.154328714787
-0.035163535837	-0.154327578736
-0.03507766739	-0.154178081083
-0.034985298519	-0.154046005715
-0.034891910041	-0.153954971182
-0.034806738956	-0.153805514319
-0.034705499996	-0.153776930143
-0.034616021312	-0.15367445456
-0.034528347072	-0.153578538298
-0.034429305894	-0.153523851957
-0.03434540578	-0.153399820728
-0.034248473166	-0.153322384332
-0.034155696571	-0.15315484748
-0.03406177789	-0.153049369869
-0.033968602339	-0.15278119957
-0.033877596174	-0.152518935737
-0.033800863228	-0.152461407313
-0.033708721698	-0.152458100144
-0.033633281408	-0.152433774767
-0.033548063646	-0.152402075109
-0.033481228374	-0.152328708967
-0.033390048953	-0.152248657063
-0.033309106825	-0.152158528806
-0.033224907658	-0.15213688279
-0.033145107992	-0.152089808081
-0.0330627174	-0.152010708261
-0.032961096918	-0.151998340145
-0.032869331728	-0.151959893932
-0.032791823911	-0.151886363888
-0.032703900567	-0.151656799074
-0.032612140066	-0.151535993819
-0.032532019831	-0.151524290822

-0.032456785828	-0.151519507185
-0.032362787376	-0.151450824367
-0.032278484901	-0.151364449214
-0.032195353495	-0.151316744906
-0.032110212801	-0.151270320229
-0.032028129939	-0.150858821644
-0.031946534452	-0.150773514454
-0.031861541355	-0.150689664707
-0.031769195688	-0.150446890295
-0.031699955228	-0.15037703533
-0.031604551664	-0.150375460014
-0.031530418453	-0.15031265239
-0.031440855883	-0.150133288554
-0.03137086645	-0.150099429678
-0.031277348034	-0.150061635231
-0.031197663302	-0.149978624001
-0.031110780095	-0.149897980863
-0.031035409558	-0.149849053585
-0.030946841693	-0.149604945421
-0.030865438742	-0.149489403104
-0.030782052007	-0.149301738982
-0.030714072263	-0.14928739911
-0.030637953047	-0.149282695753
-0.030546913081	-0.149246413268
-0.030474347947	-0.149234120097
-0.030397070079	-0.149230298919
-0.030326248522	-0.149227586597
-0.030251601088	-0.149179090906
-0.030160238505	-0.148992308852
-0.030067889998	-0.148946434307
-0.029974870926	-0.148944341884
-0.029884098477	-0.148890183158
-0.029800347353	-0.148880597707
-0.029709706237	-0.148776318488
-0.029614236069	-0.148769026889
-0.029516535716	-0.148763788231
-0.029446851243	-0.148682487686
-0.029365647237	-0.148647001834
-0.029278640181	-0.148226210679
-0.029193297003	-0.148177013684
-0.029106592367	-0.148086519036
-0.029035139156	-0.147802982361
-0.028949504105	-0.147781200825
-0.028859710634	-0.147728991136
-0.028789438904	-0.147541964053
-0.02870410522	-0.147389453008
-0.028625496915	-0.147339154045
-0.028551121294	-0.1472612443
-0.028476847456	-0.147258757841
-0.028398697348	-0.147150406872
-0.028328161395	-0.147149193464
-0.028253900453	-0.147131871947
-0.02817880184	-0.147037971282
-0.028075740291	-0.146866991938

-0.027986503299	-0.146818145499
-0.02790766738	-0.146659567319
-0.027816744657	-0.146486040881
-0.027721044158	-0.146470892355
-0.027636791018	-0.146383153086
-0.027563441955	-0.146349570949
-0.027483794564	-0.146236117089
-0.027385817653	-0.146205856394
-0.027304257749	-0.14607119581
-0.027215037032	-0.14576659968
-0.027140944849	-0.14567883606
-0.027051382411	-0.145621129305
-0.026962200944	-0.145461097714
-0.026882743341	-0.145458889005
-0.026788685133	-0.145422097188
-0.02671612059	-0.145330876431
-0.026651523661	-0.145120210473
-0.026582832396	-0.144992281008
-0.026515852784	-0.144925305987
-0.026430629601	-0.144890526422
-0.026347023496	-0.144844480456
-0.026264709781	-0.144674817422
-0.026179758178	-0.144611464252
-0.026095595679	-0.144489139227
-0.026020565981	-0.144470386498
-0.025931580424	-0.144349236653
-0.025852571002	-0.144134542372
-0.025777702093	-0.144100248014
-0.025688565657	-0.143887498208
-0.025608092432	-0.143832255223
-0.025525314402	-0.143782398448
-0.025430939603	-0.143531957265
-0.025355223235	-0.143275119562
-0.025270308571	-0.143079133671
-0.025190121232	-0.142897099965
-0.025120701839	-0.142894739935
-0.025047739616	-0.142869235202
-0.024944924188	-0.142819184848
-0.024867519311	-0.142800475945
-0.024791836743	-0.142502026611
-0.024711537808	-0.142422300828
-0.024643040619	-0.14231582722
-0.024560366106	-0.142307709482
-0.024475985076	-0.142188394041
-0.024388907403	-0.142008961637
-0.024297560056	-0.141803401397
-0.024209821847	-0.141710795288
-0.024135768316	-0.141635294399
-0.024045352373	-0.141532980966
-0.023943879003	-0.141459768943
-0.023856105899	-0.141429592411
-0.023763852357	-0.141299528212
-0.023685559421	-0.141199037737
-0.023606534241	-0.141149225398

-0.023514753996	-0.141087693264
-0.023445407415	-0.141002272359
-0.023368717027	-0.140961116044
-0.023293539545	-0.14076029282
-0.023195010242	-0.14066548545
-0.023122113306	-0.140585872882
-0.023050532997	-0.140468491279
-0.022948397026	-0.140467740023
-0.022863310699	-0.140297412244
-0.022799094703	-0.140028152622
-0.022698049309	-0.139929102924
-0.022621414853	-0.139722798425
-0.022558306566	-0.139631515098
-0.022460814813	-0.139563601998
-0.022377303298	-0.139555179791
-0.022282923748	-0.139464914516
-0.02220397798	-0.139388473081
-0.022107728127	-0.13929123814
-0.022020505296	-0.139198614428
-0.021926000411	-0.139080325009
-0.021840385264	-0.138976961814
-0.021759599719	-0.138884159073
-0.021676503473	-0.138868425451
-0.021600417991	-0.138788483309
-0.021511243579	-0.138728517181
-0.021413772125	-0.138666854052
-0.0213282187	-0.138606545873
-0.021252428916	-0.138552484719
-0.021163867307	-0.138517247165
-0.021084337962	-0.138385733373
-0.020998662223	-0.138300790029
-0.020923962073	-0.138116170251
-0.020847754922	-0.138066852301
-0.020759127586	-0.138025409539
-0.020669897654	-0.138006339818
-0.020592112242	-0.13794966732
-0.020500148732	-0.137684083969
-0.020417168756	-0.137607205266
-0.020339004798	-0.137539021575
-0.020259245039	-0.137392003433
-0.020158403349	-0.137211165252
-0.020078793023	-0.136909419215
-0.019998587209	-0.136816803756
-0.019925985818	-0.136754123908
-0.019849309517	-0.13672000398
-0.019764166182	-0.13670583331
-0.019689316247	-0.136696974231
-0.019592816044	-0.136629499155
-0.019510173292	-0.136623393956
-0.0194443129	-0.136556333858
-0.019360635062	-0.136274550052
-0.019277915709	-0.136183112163
-0.019196727275	-0.135967051299
-0.019120966159	-0.13596200521

-0.019026489086	-0.135835785731
-0.018932910258	-0.135636821564
-0.018853675014	-0.135518292949
-0.018768101062	-0.135509239295
-0.018681773702	-0.13533292697
-0.018587798465	-0.135329189806
-0.018510746622	-0.135319560888
-0.018423581845	-0.135245719692
-0.018334584537	-0.135107286936
-0.018262752552	-0.135079382139
-0.018176648428	-0.134974797136
-0.018095005957	-0.134931557247
-0.018004620187	-0.134842193638
-0.017938809298	-0.134837230887
-0.017870266396	-0.134794462077
-0.017800097917	-0.134768749862
-0.017706465569	-0.13475940193
-0.017617897132	-0.134744462139
-0.017543020038	-0.134729666183
-0.017452772525	-0.134624120376
-0.0173677756	-0.134609267202
-0.017290115516	-0.134586912779
-0.017209798003	-0.134471525078
-0.017125064351	-0.134438401042
-0.017034591066	-0.134364301945
-0.016939667599	-0.134343861554
-0.016858918095	-0.134264813972
-0.016774935151	-0.134182944582
-0.016681411169	-0.133954587369
-0.016589895996	-0.133818845582
-0.016509502241	-0.133331555677
-0.016430220749	-0.133166425342
-0.016348929974	-0.133144204337
-0.016266923966	-0.132855758632
-0.016180593578	-0.132788783308
-0.016093958686	-0.132745492875
-0.016006609223	-0.132719927579
-0.015929188419	-0.132651393544
-0.01584974619	-0.132620478073
-0.015758929737	-0.132591413557
-0.015672479063	-0.132518030749
-0.015594157591	-0.132498663278
-0.015525295302	-0.132362285111
-0.015443645936	-0.132086044133
-0.015346389549	-0.13183112955
-0.015258432832	-0.131671529032
-0.015172384372	-0.131539623295
-0.015089131525	-0.131486314632
-0.015002448404	-0.13142988309
-0.014934821865	-0.131371666468
-0.014850737488	-0.131307860455
-0.014759325294	-0.131264939486
-0.014677775065	-0.131137927816
-0.014592045672	-0.131047741488

-0.014492690999	-0.130776116597
-0.014414632602	-0.130728204848
-0.014331020335	-0.130630532542
-0.014253265347	-0.130398434852
-0.014175661321	-0.130339261667
-0.014078170653	-0.130299750578
-0.013980173978	-0.1302195201
-0.013895420645	-0.130127294045
-0.013800577415	-0.130089209209
-0.013707090989	-0.129829080224
-0.01361738233	-0.129747133983
-0.013551723557	-0.129626593547
-0.013461205118	-0.129532628868
-0.013378498651	-0.129509571442
-0.01329814121	-0.12938486547
-0.013226565568	-0.128959937215
-0.013157372569	-0.128907531285
-0.013070998644	-0.128595564037
-0.012977953095	-0.128593799408
-0.012898753681	-0.128555357228
-0.012821852719	-0.128478851832
-0.012736052853	-0.128452073192
-0.012643429541	-0.128202265908
-0.012571059042	-0.128051879346
-0.01248753922	-0.127933710585
-0.012402529069	-0.127930693816
-0.012300737382	-0.127890264117
-0.012204399547	-0.127887067407
-0.012117925026	-0.12777827362
-0.012043592042	-0.127624282193
-0.011967426878	-0.127491359685
-0.011860138391	-0.127377474143
-0.011766792185	-0.127336268967
-0.011673253867	-0.12729258495
-0.01160251704	-0.127101033563
-0.011536371895	-0.126965949304
-0.011439915999	-0.12679085801
-0.011377249073	-0.126751385384
-0.01130056041	-0.126554719423
-0.011230069408	-0.126499390632
-0.011149353807	-0.126281533411
-0.01106130942	-0.126179226427
-0.01097847382	-0.12604109404
-0.010895657699	-0.125879335191
-0.010790418456	-0.125821405665
-0.010711384263	-0.125797844975
-0.010641349004	-0.125494416593
-0.010546503063	-0.125459068976
-0.010454406801	-0.125326675833
-0.010378212826	-0.125313802758
-0.010293125034	-0.125186393775
-0.010197332499	-0.125173256434
-0.0101218569	-0.125037288481
-0.010030462347	-0.124672900684

-0.009944783268	-0.124656582009
-0.009855252166	-0.124337786808
-0.009770630462	-0.124218168924
-0.009672423027	-0.124140338404
-0.00957537009	-0.124052045296
-0.009498677879	-0.123933658992
-0.009416597818	-0.123742261031
-0.009319263091	-0.123669544082
-0.009227429339	-0.123451668071
-0.009125553241	-0.123433773707
-0.009039839966	-0.123367404684
-0.008959863543	-0.123358948057
-0.008864550797	-0.123319656582
-0.008785086211	-0.123132434145
-0.008701702728	-0.123095005036
-0.008607243461	-0.12297254614
-0.008512582129	-0.122877950539
-0.008434244228	-0.122744782597
-0.008344682845	-0.122599419705
-0.008243765867	-0.122451030013
-0.008170966131	-0.122289265034
-0.008089558286	-0.122013272493
-0.008014087557	-0.121986703293
-0.007920107286	-0.121822997063
-0.007843441508	-0.121672149094
-0.007752135433	-0.121144262404
-0.007662126211	-0.12111533983
-0.007592547329	-0.121039730885
-0.00750312514	-0.121033184628
-0.00743307721	-0.120772112865
-0.007349140533	-0.12067826985
-0.007271215199	-0.120623021826
-0.007170548858	-0.12051947415
-0.007098409123	-0.120490048173
-0.007012157433	-0.120266802558
-0.006929710892	-0.120256905791
-0.006838216764	-0.1202491079
-0.006744762968	-0.120151823712
-0.006643592135	-0.120127394779
-0.006573975324	-0.119954585626
-0.006493380038	-0.119786097663
-0.006403423095	-0.119531964311
-0.006309690542	-0.119507844888
-0.006232030495	-0.119439877421
-0.006140615144	-0.119139167591
-0.006055716233	-0.118936435426
-0.005985711557	-0.118888125129
-0.005905131229	-0.118827823961
-0.005824109132	-0.118752809288
-0.005736133914	-0.118744602598
-0.005651498784	-0.118638384692
-0.005583279974	-0.118476854594
-0.005509814806	-0.118423783656
-0.005417041776	-0.118361470863

-0.005334228704	-0.118035044366
-0.005245992196	-0.118008626142
-0.005166900479	-0.117806505904
-0.005072817524	-0.117497451624
-0.004982160798	-0.117470713811
-0.004899595123	-0.117469206475
-0.004799947197	-0.11735659011
-0.004708570962	-0.117001941716
-0.004621561592	-0.116603466698
-0.004539883519	-0.116494418542
-0.004444537592	-0.116474573412
-0.004368610169	-0.116473744803
-0.004296218294	-0.116448115681
-0.004207162381	-0.116378272011
-0.004131637827	-0.116367398521
-0.00405465654	-0.116327881455
-0.003959528616	-0.116273738432
-0.003891978642	-0.116268020868
-0.003820505332	-0.116260754875
-0.003751059792	-0.115732127323
-0.003674978179	-0.115505454721
-0.003569742667	-0.115485553479
-0.003493514306	-0.115471743979
-0.003395375646	-0.11541937333
-0.00329783132	-0.115342981037
-0.003205931544	-0.115325399595
-0.003103256786	-0.11526232645
-0.003027533932	-0.115209854276
-0.002955145133	-0.11502603022
-0.002877209177	-0.114864162263
-0.002791470241	-0.114804584496
-0.002714092623	-0.114721303072
-0.00264380393	-0.11471750284
-0.002556483214	-0.114633857115
-0.002466599253	-0.114611329174
-0.00238940613	-0.114421866811
-0.002297075216	-0.114357300143
-0.002216243932	-0.113997739151
-0.002117648443	-0.113669957581
-0.002038844668	-0.113620890411
-0.001961609423	-0.1134752552
-0.001862899413	-0.11342156405
-0.001777731423	-0.112640968619
-0.001696980318	-0.112630431911
-0.001617695977	-0.112627333797
-0.001540616881	-0.112614589056
-0.001452549729	-0.112528892149
-0.001372243004	-0.112449015769
-0.001289968745	-0.112437388522
-0.001208135654	-0.112393203449
-0.001127338619	-0.112265367251
-0.001025305404	-0.112258643936
-0.000946548478	-0.112220968146
-0.000857482153	-0.112200156965

-0.000775667468	-0.112184610041
-0.000698089715	-0.11206345179
-0.000619564533	-0.111931014698
-0.000543219551	-0.111924695502
-0.000458510049	-0.111804165739
-0.000371588986	-0.111745678747
-0.000297223557	-0.111741892686
-0.00022391128	-0.111720266785
-0.000146016488	-0.111656857573
-6.99722192E-05	-0.111623179158
2.932887027E-05	-0.111498393244
9.754951313E-05	-0.111447240008
0.0001759416797	-0.111372739988
0.0002583076705	-0.111356298163
0.0003454040305	-0.111328086597
0.000422042247	-0.111307195976
0.0005248910682	-0.111225870346
0.0006077383552	-0.111089015802
0.0006820361633	-0.110944476381
0.0007586060533	-0.110889599578
0.0008413886485	-0.110874323801
0.0009352726684	-0.110817816606
0.0010166583459	-0.110807695808
0.0010977470112	-0.110784285621
0.0011763011735	-0.110659559969
0.0012613469533	-0.110509040835
0.0013438845362	-0.110425611751
0.0014189101962	-0.110290787433
0.0015045029303	-0.110277335306
0.0015938941417	-0.110013630488
0.0016834115345	-0.110013517056
0.0017684749062	-0.109951948842
0.0018482809823	-0.10994586043
0.0019330141049	-0.10986783167
0.0020074910631	-0.109617637474
0.0020807583165	-0.109465057902
0.0021701847619	-0.109272005408
0.0022487701507	-0.109230069939
0.002336845205	-0.109188552901
0.0024040969216	-0.10918646313
0.0024853300841	-0.109031353174
0.0025549550529	-0.108926443289
0.002639033087	-0.108786867749
0.0027109665686	-0.108784103513
0.0027961653025	-0.108644383036
0.0028699445919	-0.108560004791
0.002957484381	-0.10822120041
0.0030521521226	-0.107910960187
0.0031317052082	-0.107884985683
0.003227578778	-0.107880785514
0.0033009844292	-0.10782621391
0.0033915732474	-0.107671307018
0.0035021600703	-0.10760783317
0.0035858207349	-0.106841497551

0.0036692974558	-0.106819310887
0.003763334459	-0.10662083
0.0038426923258	-0.106439210168
0.0039283617756	-0.106386886725
0.0040146108354	-0.106338383764
0.0040980286668	-0.106322478305
0.0041827008378	-0.106303131768
0.0042705442772	-0.106187742271
0.0043561931922	-0.106079597501
0.0044368323273	-0.106057835272
0.0045225591046	-0.105862459449
0.0046082700659	-0.105836931427
0.0047060862689	-0.105785461874
0.0047917032868	-0.105705959056
0.004863812842	-0.105650781332
0.004951263192	-0.105642043811
0.0050407230939	-0.105535676816
0.0051216304055	-0.105416615708
0.0052136382388	-0.105358548651
0.0053003260693	-0.105073290413
0.0053877564688	-0.105042304357
0.0054626965306	-0.105025287777
0.0055473682172	-0.105012530661
0.0056439536718	-0.104659436816
0.005741653625	-0.104575599856
0.0058213046759	-0.10454788365
0.005909152296	-0.104354899276
0.005988371292	-0.104059238169
0.006055177348	-0.10401833873
0.0061425888512	-0.103892394659
0.0062172227484	-0.103743108064
0.0062927406106	-0.103651132306
0.0063743566	-0.103650696307
0.00647505625	-0.103524108234
0.006573724522	-0.103295696381
0.0066552777645	-0.103225545919
0.0067350567131	-0.103222961153
0.006814428215	-0.103027493796
0.0068975908592	-0.102972135335
0.0069658743545	-0.10288913507
0.0070467849883	-0.102832739577
0.0071274185711	-0.102820790899
0.0072107772254	-0.102734884576
0.0073063140147	-0.102469249299
0.0074009795566	-0.102428831908
0.0074742070592	-0.10235149295
0.0075574535438	-0.102185878848
0.0076542067735	-0.102005607259
0.0077475433584	-0.101997991607
0.0078375343006	-0.101741705213
0.0079189283476	-0.101465857615
0.0079889928792	-0.101412110946
0.0080796898817	-0.101141506678
0.0081773911267	-0.101102264324

0.0082471990398	-0.100992150126
0.0083329379061	-0.100970658316
0.0084295971476	-0.100901651284
0.0085124176315	-0.100839099015
0.0085782507198	-0.100678760127
0.0086620631143	-0.100608103368
0.0087467992609	-0.100224666622
0.0088239116345	-0.100178323996
0.0089014294622	-0.100135943463
0.0089788436137	-0.099800531277
0.0090739326824	-0.099794550451
0.0091463770196	-0.09971656348
0.0092430946639	-0.09963153059
0.0093151040361	-0.099497880857
0.0093964983103	-0.099399800675
0.0094737183769	-0.099375997079
0.009552813474	-0.099351720797
0.0096362856055	-0.099349788182
0.0097158501369	-0.098939098115
0.0097933077195	-0.09891450886
0.0098764039693	-0.098674994848
0.0099568622446	-0.098561392733
0.0100531293026	-0.098424901111
0.0101405784705	-0.098420006607
0.0102061940987	-0.09811494335
0.0102752257465	-0.09788085689
0.0103699785068	-0.097859717845
0.0104649892763	-0.097830861296
0.0105387920838	-0.097689425858
0.0106169248132	-0.097659335138
0.0106979896834	-0.097554227487
0.0107788947099	-0.097452402194
0.0108475456821	-0.097411494285
0.0109187450046	-0.097235036777
0.0110147382365	-0.097225409426
0.0110885679104	-0.09722124701
0.0111824451329	-0.097169072599
0.0112619686855	-0.097022830836
0.0113470326081	-0.09656714314
0.0114331718739	-0.096047770392
0.0115227336678	-0.095976175334
0.011607544908	-0.095913574461
0.011685956856	-0.095704493295
0.0117702114336	-0.095684446006
0.0118466850956	-0.095664993691
0.0119219047398	-0.095488822073
0.0120062269917	-0.095070854834
0.0121015394287	-0.095049518716
0.0121811889312	-0.095029056181
0.0122699825461	-0.094880839744
0.0123560972114	-0.094829905172
0.0124426699569	-0.094761438426
0.0125163428956	-0.094738777765
0.0126036517652	-0.094631611689

0.0126861996014	-0.094350346748
0.0127742722106	-0.094069563777
0.012853263888	-0.094000053519
0.0129274952672	-0.093922354553
0.0130058386711	-0.093906015603
0.0130829821254	-0.093738711545
0.013166935084	-0.093638157674
0.0132391825247	-0.093533351977
0.0133255619457	-0.0934437899
0.0134074427932	-0.093440310062
0.0134934485949	-0.093362401843
0.0135804275368	-0.093226000652
0.0136717547245	-0.093203568817
0.0137498826251	-0.092932771023
0.0138181524148	-0.092815514776
0.0139048544316	-0.092760012393
0.013993116613	-0.092404466738
0.0140848352324	-0.092290521151
0.0141615169407	-0.092206565996
0.0142372243958	-0.092088417071
0.0143270746942	-0.092022217358
0.0143923857679	-0.091931935473
0.0144821456139	-0.091832105725
0.0145786396642	-0.091826269025
0.01465861174	-0.09154602972
0.0147372257373	-0.091302702687
0.0148128848085	-0.090778072064
0.0149191978153	-0.090745808216
0.0149880804961	-0.090646025246
0.0150698087223	-0.090567387662
0.0151710212199	-0.090379767779
0.0152551026443	-0.090362638902
0.015335173163	-0.090362351078
0.0154220147417	-0.090330902594
0.0155334720503	-0.090269328406
0.0156225321214	-0.089572816252
0.0157048640556	-0.08956929601
0.0157906224499	-0.089553154132
0.0158863903384	-0.089473240977
0.0159691880903	-0.08946128838
0.0160540521089	-0.08933279036
0.0161384045311	-0.089292130138
0.0162145734297	-0.089200980525
0.0162929376676	-0.08912878149
0.0163744249786	-0.088943239574
0.0164629991858	-0.088796843588
0.0165360119725	-0.088752747377
0.0166142407661	-0.088606049954
0.0167180964936	-0.08855592398
0.0167946164703	-0.088206214766
0.0168806345977	-0.088180849189
0.0169621590795	-0.088157131821
0.0170564002159	-0.088141800584
0.0171394302618	-0.088125816153

0.017230264904	-0.087983201877
0.0173132795786	-0.087830790692
0.0174045458588	-0.087758553522
0.0174931113366	-0.087659370457
0.0175744890048	-0.087519920669
0.0176608330631	-0.087481103706
0.0177521876536	-0.087477344368
0.0178229054091	-0.087390763242
0.0179047509709	-0.087300501742
0.0179862265898	-0.087296497444
0.0180803126866	-0.087294648781
0.0181444395074	-0.087228572017
0.0182415891951	-0.087120245074
0.0183402180105	-0.087096777396
0.0184225592759	-0.087050974253
0.0184959504223	-0.086986529518
0.0186174131	-0.086799107309
0.0187036852508	-0.086580743799
0.0187814862208	-0.086415400948
0.0188569051716	-0.086377856002
0.0189396476779	-0.086302808555
0.019012258963	-0.086261556045
0.0190913661555	-0.086252577658
0.0191751598555	-0.086220078055
0.0192605985788	-0.086053834378
0.019348392896	-0.085965446224
0.0194320272163	-0.085650693638
0.0195084043838	-0.085595315154
0.0195796133325	-0.085592940062
0.0196621591064	-0.085396235689
0.0197588970936	-0.085374619583
0.0198478715788	-0.085204235886
0.0199314816108	-0.085192246637
0.0200187009321	-0.085187935176
0.0200993543463	-0.08496230412
0.020198000157	-0.084698195346
0.0202783589156	-0.084649531454
0.0203650844898	-0.084462737183
0.0204427080231	-0.084449643183
0.02052248839	-0.084424558576
0.0206309164324	-0.084424224706
0.0207269794717	-0.084360680678
0.0208036178562	-0.084326298504
0.0208857816866	-0.084165842691
0.0209653614951	-0.084120225468
0.0210452190481	-0.084083775628
0.0211343742642	-0.084053885759
0.0212135821256	-0.083900642511
0.0212948756991	-0.083896219291
0.0213870622271	-0.083863271039
0.0214707632443	-0.083820668627
0.0215527160565	-0.083802328523
0.0216286248501	-0.08376306115
0.0217119190173	-0.083681955499

0.0218067749352	-0.083662820639
0.0218791924198	-0.083494603062
0.021948099801	-0.08345456424
0.022031673459	-0.083292019125
0.0220882388155	-0.083214900119
0.0221816289814	-0.083214361674
0.0222738181392	-0.08315518379
0.0223619153053	-0.083142578901
0.022442976647	-0.083127511465
0.0225283413426	-0.082959166206
0.0226037172269	-0.082927809902
0.0226794432084	-0.082793622233
0.022764862119	-0.082735971723
0.0228520959453	-0.082696685628
0.0229286725479	-0.082505255569
0.0229941883536	-0.082308771674
0.0230824915161	-0.08225924887
0.0231621048494	-0.082031536403
0.0232407489271	-0.081973484311
0.0233251161031	-0.081944418614
0.0233978935009	-0.081859240633
0.0234774791815	-0.081609347897
0.0235570948338	-0.081564136207
0.0236380055406	-0.081453700578
0.0237336842009	-0.081298002267
0.0238155042388	-0.081128774828
0.0238992197444	-0.081099047823
0.0239713892582	-0.081077294009
0.0240583068466	-0.08103932408
0.0241463276907	-0.080438358778
0.0242239294604	-0.080313734237
0.0243070182625	-0.080221585294
0.0243858896232	-0.080043823615
0.0244811794364	-0.07993274645
0.0245784285051	-0.079929575126
0.0246639162608	-0.07992364492
0.0247456814236	-0.079805161596
0.0248177782426	-0.079646902262
0.0249097122871	-0.079476587881
0.0249879444343	-0.079404057228
0.0250769124437	-0.079372361229
0.0251708787649	-0.079355047218
0.025253892012	-0.07929321083
0.0253535961309	-0.078981210248
0.0254391327718	-0.078747620917
0.0255149208629	-0.078741169883
0.0256094715348	-0.078669467336
0.0256984481294	-0.07833176975
0.0257802037264	-0.078329990243
0.0258592873002	-0.078266151098
0.0259582655775	-0.078200948317
0.0260513496902	-0.078191818813
0.0261280741229	-0.078107502599
0.0262017197366	-0.078106288579

0.0262865228527	-0.078029887155
0.0263793489011	-0.07799658651
0.026456175155	-0.077931241547
0.0265334352063	-0.077901904399
0.0266055574687	-0.0778588323
0.0266738810913	-0.077782882354
0.0267639570765	-0.077660741085
0.0268511907172	-0.077604244738
0.0269456835876	-0.077396297498
0.0270351279725	-0.077211836527
0.0271196316998	-0.077181258978
0.02719181188	-0.077154851588
0.0272942603551	-0.077143884171
0.0273896047663	-0.077083315582
0.0274893643265	-0.077046564
0.0275615767801	-0.077033716625
0.0276369863933	-0.077027303201
0.0277176665554	-0.0769251906
0.0277955424561	-0.076887047548
0.0278734006594	-0.076774325391
0.0279606422313	-0.076716072944
0.0280438490001	-0.076708127492
0.0281227031444	-0.076685912045
0.028210032098	-0.076675380531
0.0282880231812	-0.076447653734
0.0283663762468	-0.076439735084
0.0284585174155	-0.076373482237
0.0285618597956	-0.076359400728
0.0286510941219	-0.076278753657
0.0287383485846	-0.076177591048
0.0288250097325	-0.076175806129
0.0289067613295	-0.076006462252
0.028990836463	-0.075904257065
0.0290778882614	-0.075883357159
0.0291667826482	-0.075879690338
0.0292421506818	-0.075843738207
0.0293174473753	-0.075805461962
0.0294020713452	-0.075599775436
0.0295185403428	-0.075582166367
0.0296146925561	-0.075165267729
0.0296945267288	-0.075046331024
0.029797146474	-0.075013232835
0.0298841722474	-0.074778049202
0.0299684825553	-0.07469463332
0.0300571555972	-0.074658757629
0.030145199093	-0.074583911784
0.0302378251355	-0.074477079401
0.0303219764017	-0.074469324629
0.0304131629608	-0.074419465616
0.0304960756364	-0.07435359525
0.0305771118049	-0.074343411649
0.0306524162779	-0.074282401586
0.0307469647967	-0.074232226409
0.0308388299408	-0.07407901696

0.0309026517476	-0.073900091716
0.0309872588428	-0.073872440184
0.0310786889294	-0.073563092466
0.0311668698764	-0.073498506865
0.0312432348883	-0.073295727761
0.0313231311213	-0.073256636767
0.0314019404086	-0.073085242255
0.0314852764914	-0.072909335057
0.0315646111461	-0.07278202803
0.0316583968717	-0.072695600472
0.0317474063836	-0.072510534774
0.0318276715317	-0.072440170547
0.0319170394705	-0.072308387609
0.0319980829346	-0.072278476425
0.0320931950065	-0.072235197371
0.0321721892688	-0.072233737077
0.0322463392073	-0.072199854948
0.0323212339272	-0.072191444393
0.0324030563722	-0.072101136051
0.0324703809146	-0.072068083403
0.0325518878408	-0.072050507794
0.0326515585769	-0.07198969622
0.0327295754951	-0.071865872822
0.0328171056646	-0.071181627608
0.0329027392552	-0.071162615369
0.0329814745711	-0.070932827723
0.0330595993244	-0.070854151085
0.0331363178541	-0.070850896311
0.0332065764881	-0.070677031348
0.0332771983276	-0.070613717513
0.0333506615649	-0.070553148186
0.0334234938563	-0.070328232726
0.033509453028	-0.070276830342
0.0336041452996	-0.070035053398
0.0336828376989	-0.069906950323
0.0337623228183	-0.069474659812
0.033842071695	-0.069474313766
0.0339326284517	-0.069343324242
0.0340188291466	-0.069322145013
0.0341242872039	-0.069273653732
0.0341958222012	-0.0690986022
0.0342723104471	-0.069067055471
0.034346340302	-0.069034056449
0.0344479918242	-0.068950588096
0.0345444098109	-0.068729038368
0.034616973731	-0.06855816655
0.0346977200548	-0.068036080707
0.0347836413391	-0.067908788447
0.0348815790818	-0.067882923159
0.0349632487408	-0.067756829899
0.0350568786827	-0.067720334324
0.035166759317	-0.067651086958
0.0352534898319	-0.067501643851
0.0353302857261	-0.067086298386

0.0354044596092	-0.066969731909
0.0354797095987	-0.066685695692
0.0355624743547	-0.066435049344
0.0356534391686	-0.066354932239
0.0357352177184	-0.066290785062
0.035818142778	-0.0661138029
0.0359097802231	-0.066050847663
0.0359997321146	-0.066027312752
0.0360830129396	-0.065828261452
0.0361671061169	-0.065817461356
0.0362660895165	-0.065747129245
0.0363672206504	-0.06570848716
0.0364454876339	-0.06568323919
0.0365474853767	-0.065661006172
0.0366320848253	-0.065597683387
0.0367171959682	-0.06545700948
0.0367844108991	-0.065346607428
0.036866579676	-0.065050660495
0.036934344737	-0.065019833558
0.0370207134158	-0.064948575027
0.0371092262766	-0.064861858676
0.0371968585266	-0.064720433317
0.0372713472789	-0.064667051212
0.0373354948119	-0.064609916981
0.0374157482227	-0.064488260446
0.0374930480238	-0.064329655348
0.0375702300919	-0.064264556609
0.0376543627519	-0.064129487833
0.0377476438672	-0.064128106234
0.0378211509735	-0.064043739837
0.0379107061747	-0.06388358034
0.0379984797868	-0.06372187131
0.0380740826505	-0.063613687298
0.0381329893477	-0.06360312062
0.0382014050417	-0.063500595343
0.0382947578002	-0.063458771632
0.0383948700554	-0.063358890666
0.0384669670152	-0.063169610109
0.0385473551957	-0.063095478507
0.0386205360681	-0.063029602916
0.0387033176355	-0.063012821432
0.0388047981818	-0.063007706092
0.0388937579318	-0.062786751473
0.0389920752492	-0.062721659058
0.0390756273451	-0.062717297534
0.0391598424422	-0.062625553312
0.0392621904876	-0.062413604612
0.0393540546261	-0.062231774211
0.0394406972325	-0.062135461357
0.0395249829299	-0.062122535303
0.0396174005466	-0.06211445763
0.0397042994853	-0.062098412231
0.0397752233185	-0.062033185815
0.0398523169863	-0.061936561883

0.0399420361366	-0.061897102559
0.0400103386081	-0.061763071039
0.0400991116014	-0.06172101584
0.0401998973754	-0.061719001165
0.0402857796789	-0.061647938086
0.0403699151924	-0.061627205001
0.0404498031401	-0.061622709772
0.0405267080342	-0.06159559368
0.0406212159907	-0.061584956766
0.0406958989354	-0.061512426723
0.0407943152134	-0.061509068407
0.0408944176644	-0.061426873052
0.0409916801214	-0.061284232868
0.0410928800598	-0.061267735136
0.0411737865194	-0.061175055375
0.0412525190043	-0.061141574446
0.0413385978481	-0.061137130016
0.0414079303771	-0.060999552066
0.0414903923788	-0.060875362435
0.0415669735518	-0.060856467728
0.0416500411892	-0.060807841708
0.0417244423622	-0.060765943852
0.0418171747026	-0.06065937671
0.0418928248198	-0.060558231704
0.0419595163236	-0.060456853098
0.0420230548181	-0.059927664351
0.0421101701027	-0.059456597601
0.0422105270415	-0.059392984123
0.0422792879648	-0.059351147525
0.0423641386218	-0.059212943571
0.0424373051396	-0.059156304111
0.0425376253263	-0.058938524018
0.0426106381143	-0.058906500187
0.0426981165926	-0.058673690338
0.0427843504778	-0.058556541828
0.0428730699454	-0.058494270987
0.0429401045611	-0.058092382634
0.0430196437996	-0.058054420038
0.0431191677673	-0.05797469478
0.0432002626219	-0.057926896554
0.043284393909	-0.057911438359
0.043364655073	-0.057867638619
0.0434558511145	-0.057592197605
0.0435415773201	-0.057249872166
0.0436311375127	-0.057034710672
0.0437125947793	-0.056932869647
0.0437843325655	-0.056815929626
0.0438626915619	-0.056730598938
0.0439413272031	-0.056714693387
0.0440211878808	-0.056570638077
0.0440886386908	-0.056494114887
0.0441751773115	-0.056462693577
0.0442669432372	-0.056341457302
0.0443396119667	-0.056340928887

0.044432727879	-0.056277324841
0.0445276582325	-0.056221089696
0.0446203993771	-0.056161339165
0.0447269605783	-0.056158855475
0.0448033870894	-0.056045539682
0.0448978684963	-0.056016066164
0.0449774628441	-0.055978226833
0.0450538366162	-0.055912509091
0.0451462075132	-0.055795043364
0.0452357504659	-0.055711306185
0.0453170999193	-0.055701118891
0.0453956517328	-0.055569213873
0.0454800014573	-0.055537924366
0.045562510231	-0.0553799733
0.0456592556751	-0.055337780848
0.0457338453626	-0.055231254838
0.0458119193947	-0.055185737211
0.0459173971226	-0.055139491485
0.04599918924	-0.055079509273
0.0460798827923	-0.054931765537
0.0461584741649	-0.054713340525
0.0462245474883	-0.054280617454
0.0463214450217	-0.05422035412
0.0464113702573	-0.054179385705
0.0465014313657	-0.054070301615
0.0465884173082	-0.053823739979
0.0466788213562	-0.053748307093
0.0467730915332	-0.053308744349
0.046860950182	-0.053210691437
0.0469622771978	-0.053141637154
0.0470383035759	-0.053132454264
0.0471334616203	-0.052911739591
0.047206963821	-0.052714019583
0.0472946731169	-0.052594379763
0.0473879151923	-0.052549109458
0.0474584793709	-0.052488185336
0.0475364649828	-0.052437054312
0.0476175716274	-0.052070933297
0.0476982900473	-0.052006291195
0.0477784396438	-0.052005986845
0.0478501247755	-0.05191056072
0.0479454505883	-0.051704430033
0.0480326743402	-0.051702227071
0.0481235833942	-0.051659381299
0.0482132713586	-0.051545736358
0.0482987014628	-0.051478728193
0.0483786915283	-0.05142846077
0.0484574419468	-0.051116982022
0.0485424130883	-0.051091282983
0.0486180000011	-0.050801068915
0.048716095994	-0.050789650748
0.0487929120043	-0.050758888396
0.0488779201552	-0.050745502071
0.048973522651	-0.05071174238

0.0490546443253	-0.05066643774
0.049122212281	-0.050559018078
0.0492095846244	-0.050496734581
0.0492811062597	-0.050496704036
0.0493749145881	-0.050439482658
0.0494678439185	-0.050384706796
0.0495487611946	-0.050381603174
0.0496232214061	-0.050374124254
0.0497033631885	-0.050359290341
0.0497779482263	-0.050354699508
0.0498523849033	-0.050345175813
0.0499235560712	-0.050263679528
0.0499979262729	-0.050263452789
0.0500912406779	-0.050066652436
0.0501738652349	-0.049941583833
0.0502654027274	-0.049933342297
0.0503478441122	-0.04983598686
0.0504425376537	-0.049822444625
0.0505192028484	-0.049692433945
0.0506013564512	-0.049619073788
0.0506803076877	-0.049408753277
0.0507505635912	-0.049288421162
0.0508305154722	-0.049134461081
0.0509035170833	-0.049129871859
0.0509875870438	-0.049088989002
0.0510889540636	-0.048941795943
0.0511818606386	-0.048676472833
0.0512660255353	-0.048580920651
0.0513463356612	-0.048541184976
0.0514221165415	-0.048491572768
0.0515024509543	-0.048472010353
0.0515903548873	-0.048366579892
0.0516773593404	-0.048252075641
0.0517529762804	-0.048231153826
0.0518381297054	-0.048129314869
0.0519346438402	-0.048046337595
0.0520164174566	-0.048019801417
0.0521072776531	-0.047803420567
0.0521724114286	-0.047693915417
0.0522537351653	-0.047642327805
0.0523371550797	-0.04739145252
0.0524075104188	-0.047341108912
0.0524784812755	-0.047067436576
0.0525667353273	-0.047010239223
0.0526523453308	-0.046965324492
0.0527348878436	-0.046697203094
0.0528102430414	-0.04667736071
0.052904433054	-0.046637661847
0.0529945373001	-0.046588820765
0.0530711320345	-0.0463009506
0.053156287359	-0.046240033665
0.0532460940612	-0.04622529604
0.0533269823959	-0.0462245876
0.0534075763701	-0.046101666752

0.0534892076721	-0.046018542932
0.0535667789318	-0.046016428894
0.0536598452251	-0.045931037081
0.053753123806	-0.045872970761
0.0538274994242	-0.045769415083
0.0539000895166	-0.045702465201
0.0539901515664	-0.045662938634
0.0540657640955	-0.045629074072
0.0541550697239	-0.045372937858
0.0542394789294	-0.045195830441
0.0543260458397	-0.044985919294
0.054414735772	-0.044862577316
0.0545020020117	-0.044845892675
0.0546011039311	-0.044742565051
0.0546911156154	-0.044738380077
0.0547898843558	-0.044590760106
0.0548803671254	-0.044440119754
0.0549725632517	-0.04442577095
0.0550603358654	-0.044245689087
0.0551505945765	-0.043954739179
0.0552372062958	-0.043843373745
0.0553323350973	-0.04358288247
0.0554078557814	-0.043281859798
0.0554767404565	-0.043274759883
0.0555498769412	-0.043154480332
0.055633939039	-0.042979941366
0.0557320250377	-0.042945479824
0.0558180760858	-0.042933336474
0.0558998472529	-0.042825439463
0.0559779644429	-0.042353624428
0.0560588574399	-0.042318040883
0.0561334931617	-0.042274079151
0.0562016835959	-0.042260240461
0.0562751057523	-0.042198772997
0.0563465665528	-0.042126412354
0.0564803713374	-0.042108639121
0.0565699049146	-0.041946978926
0.0566603205598	-0.041824070077
0.0567511076491	-0.041770090135
0.0568246604881	-0.041725092695
0.0569138149305	-0.041648059891
0.056973151122	-0.041523962119
0.0570617629078	-0.041487106961
0.0571716384716	-0.041369337984
0.0572620792603	-0.041348597658
0.0573528750954	-0.04130937368
0.0574187371378	-0.041232561972
0.0575010981837	-0.041151147207
0.0575918763328	-0.040999233145
0.057673545516	-0.04096872968
0.0577727476153	-0.04085095808
0.0578614676167	-0.040842985998
0.0579334777432	-0.040624913787
0.0580038700056	-0.04058153538

0.0581007049937	-0.040469288164
0.05817651776	-0.04034875436
0.0582680459512	-0.040255059883
0.0583505384679	-0.040165672735
0.0584469047384	-0.040123988772
0.0585202164137	-0.040113293837
0.0586033377139	-0.039910879073
0.0586971791796	-0.039358847891
0.0587715608484	-0.0393213411
0.0588383579537	-0.039259845341
0.0589180191308	-0.039249420308
0.0590034468424	-0.038922514686
0.059070436634	-0.038752702707
0.0591583615295	-0.038660591343
0.0592305363322	-0.038243474542
0.0593042442002	-0.038060029018
0.059371578064	-0.037915993467
0.0594371523792	-0.037819162589
0.0595200789435	-0.037811610024
0.0595810672433	-0.037580612367
0.0596656909124	-0.037541212423
0.0597500174943	-0.037325846347
0.0598447831959	-0.037266926329
0.0599321711199	-0.037210366334
0.0600129225117	-0.037036380336
0.060095726434	-0.03682078404
0.0601714452211	-0.036777688329
0.0602627781554	-0.036701766982
0.0603377290541	-0.036485277731
0.0604167646446	-0.036367580895
0.0605054598857	-0.036229568736
0.0605874718032	-0.036204719744
0.0606755488378	-0.036092632938
0.060759312711	-0.03588369091
0.060845881837	-0.035741967816
0.0609229048938	-0.03557632386
0.0610044815672	-0.035481087994
0.0610884316756	-0.035439635026
0.0611531704621	-0.035416303515
0.0612259446197	-0.035248869823
0.061307480264	-0.034902134233
0.0613805595751	-0.034864980713
0.061460812728	-0.034858900988
0.0615359707986	-0.034796227245
0.0616170006797	-0.034551116487
0.0617042939227	-0.034416325095
0.0618037797746	-0.034331622873
0.0618854049806	-0.034243511075
0.0619681626036	-0.034221832321
0.062059757794	-0.034014404006
0.062159549059	-0.033995938168
0.0622412786152	-0.033902382465
0.0623145788658	-0.033872907011
0.0623975713537	-0.033863431746

0.0624742402988	-0.03362545729
0.0625585394859	-0.033518842365
0.062642571703	-0.03348514078
0.062725686435	-0.033361192079
0.0628259470437	-0.033315863718
0.0629011350013	-0.033315153363
0.0629827908251	-0.033212474929
0.0630680486652	-0.033064197106
0.0631374659971	-0.03302919335
0.0632336644438	-0.032936672055
0.0633169778951	-0.032926298696
0.0634183758202	-0.032894403197
0.0634827273818	-0.032876022484
0.0635707006495	-0.032806925454
0.0636601780451	-0.032740926764
0.0637426690389	-0.032616686861
0.0638199556307	-0.032480859303
0.0639181033255	-0.032413941508
0.0640040552554	-0.032115475935
0.0640922363105	-0.031827723996
0.0641742382444	-0.031682710809
0.0642524630065	-0.03163204683
0.0643355670378	-0.031616005697
0.064401188509	-0.031524170784
0.0644873454281	-0.031436725453
0.0645718691887	-0.031280229417
0.0646475709644	-0.031265157847
0.0647194412484	-0.031261362466
0.0648006902524	-0.031208177639
0.0648875090956	-0.031141591184
0.0649760492067	-0.031093436863
0.0650740995793	-0.030989516532
0.0651590790615	-0.03097515701
0.0652643604012	-0.030634275038
0.065341858498	-0.030609823492
0.065428065351	-0.030484814687
0.0655077343708	-0.030478068797
0.065602117228	-0.030430307815
0.0656782666233	-0.030316002869
0.0657522278628	-0.030300925056
0.065835031692	-0.03027260525
0.0659122367476	-0.030181654671
0.0659988365403	-0.029942474362
0.0660815456371	-0.029937072421
0.0661814740901	-0.029864907723
0.066266397236	-0.029766727115
0.0663464255529	-0.029631392093
0.0664360404719	-0.029314086078
0.0665174174737	-0.029306924253
0.0666298747936	-0.029242486677
0.0667087086167	-0.029218403138
0.066804509337	-0.029192643402
0.0668721879271	-0.029140016665
0.0669646355762	-0.029104283783

0.0670368676568	-0.029043145183
0.0671234718241	-0.029041518907
0.0672079790245	-0.028674240968
0.0673007182889	-0.02826294238
0.0673898013836	-0.02817010242
0.0674951640624	-0.028156827739
0.0675813550533	-0.028093983701
0.0676700236268	-0.028001086309
0.0677477823166	-0.027730454137
0.0678422273876	-0.027682259656
0.0679163112254	-0.027515687201
0.0679849081611	-0.027112580874
0.0680630603584	-0.026975565946
0.0681547541096	-0.026787177789
0.0682297915599	-0.026674599405
0.0683035990627	-0.026640538503
0.068398382434	-0.026583801966
0.0684821859484	-0.026571451716
0.0685629863387	-0.026489620079
0.0686536141145	-0.026464971515
0.0687457534768	-0.026285476087
0.0688359154469	-0.026149126744
0.0689342686782	-0.026042096519
0.0689961485649	-0.025901796158
0.0690836033718	-0.025705297737
0.0691700738746	-0.025648973194
0.0692571878132	-0.025496543076
0.069339348065	-0.025478968982
0.0694274084451	-0.025416704734
0.0694959966578	-0.025389589671
0.069566530408	-0.025368238819
0.0696562099574	-0.025353966633
0.0697546318001	-0.025268736347
0.0698367175283	-0.025111058491
0.0699267353753	-0.025007781445
0.0700028354605	-0.024891757211
0.0700828279492	-0.02487623178
0.0701667774888	-0.024857080744
0.0702506235044	-0.024836254594
0.0703353043103	-0.024799617569
0.0704190032033	-0.024649640059
0.0705063693933	-0.024621575798
0.0705999145495	-0.024592590346
0.0706809422576	-0.024526651976
0.0707772798421	-0.024406512301
0.0708440520284	-0.024293679836
0.0709354838138	-0.024172461234
0.0710130479632	-0.024166268557
0.0711000324196	-0.024073043551
0.0711731395782	-0.023911256006
0.0712615418792	-0.023709758929
0.0713632360702	-0.023686965933
0.0714334037789	-0.023676247912
0.0715098459862	-0.023639625221

0.071580013184	-0.023189349347
0.0716510851139	-0.02316900151
0.0717417295612	-0.023153788265
0.071817328379	-0.023028525348
0.0718983274985	-0.022922973421
0.0719724294455	-0.022892407938
0.0720605090182	-0.022874287733
0.0721415874528	-0.022855718947
0.072233559348	-0.022840138097
0.0723162647158	-0.022548632307
0.0723942119116	-0.022531647271
0.0724774483244	-0.022421456585
0.0725660749771	-0.022286748937
0.0726627145583	-0.02228674173
0.0727414129483	-0.022285350285
0.0728209342661	-0.021907313653
0.0729056751098	-0.021880183128
0.0729821471101	-0.021633052696
0.0730647387502	-0.021265597622
0.073163375794	-0.021118626049
0.0732494422232	-0.021089706356
0.0733248915014	-0.021089047264
0.0734174424193	-0.020993530058
0.0734976641127	-0.020871618548
0.0735695957391	-0.020828764394
0.0736435049915	-0.020782967011
0.0737304268737	-0.020771748477
0.0738245879722	-0.020470190256
0.073908283709	-0.020464632796
0.0740091360119	-0.020313429756
0.0741002383416	-0.020242979996
0.0741966712826	-0.020079267995
0.0742826250234	-0.019965840528
0.0743592997015	-0.019683996761
0.0744344487181	-0.019607302776
0.0745154726605	-0.019595210141
0.0745921499522	-0.019172058718
0.0746681535213	-0.019149577168
0.0747512122075	-0.019135633003
0.0748313009796	-0.018864975479
0.0749228288193	-0.018855117319
0.0750249667271	-0.018787010016
0.0751176683095	-0.018774826948
0.0751947561941	-0.018698343722
0.0752961683441	-0.018508674256
0.0753804194911	-0.018498056083
0.0754727050291	-0.018424037483
0.0755579744499	-0.018401436196
0.0756626059141	-0.018384779772
0.075743357594	-0.018068647936
0.0758184540901	-0.017956581678
0.0759012590765	-0.017943147195
0.0759759129439	-0.017899342546
0.0760657774446	-0.017709348562

0.0761624353756	-0.017704679313
0.0762431542071	-0.017628725044
0.0763142081023	-0.01762631653
0.0764017772222	-0.017617038446
0.07648813465	-0.017569788574
0.0765700230799	-0.017361907917
0.0766534647641	-0.017333278541
0.0767494038607	-0.017312142185
0.0768527418634	-0.017284066666
0.0769481098971	-0.017063644293
0.0770283936902	-0.017020383927
0.077122916001	-0.016839737042
0.0771997650534	-0.016829084284
0.0772949692586	-0.016655344204
0.077380064959	-0.016590495775
0.0774557193046	-0.01656184037
0.0775505565921	-0.016285004402
0.0776391945001	-0.016039037329
0.0777413466154	-0.016024949423
0.0778271644887	-0.016020155314
0.0779185547443	-0.01581332854
0.0779966677713	-0.015650834257
0.0780823647707	-0.015476297074
0.0781600594637	-0.015297375297
0.0782466685612	-0.015097369451
0.0783433346074	-0.014978202757
0.0784227518944	-0.014824759212
0.0784988988979	-0.014616877826
0.0785857046864	-0.014378584638
0.0786663321569	-0.014087784367
0.0787438293063	-0.013977575686
0.0788389694284	-0.013836614583
0.0789294243508	-0.013828955625
0.0790197228195	-0.013758456994
0.0790973850704	-0.013672954785
0.0791914897858	-0.013347180262
0.0792737071926	-0.01326841087
0.0793701721091	-0.013128419131
0.0794485986172	-0.013033289743
0.0795321379013	-0.01262691228
0.0796036052317	-0.012607361907
0.0796808821245	-0.012352882548
0.0797666909435	-0.012272726139
0.079842973657	-0.012270712358
0.0799249559117	-0.012244663879
0.0800159153861	-0.012216043793
0.0801034954638	-0.011981057918
0.0801848245098	-0.01193794317
0.0802674981341	-0.011931050119
0.0803420319596	-0.011775001909
0.0804112952871	-0.011697831245
0.0805048711495	-0.011656377279
0.0805789562738	-0.01154349518
0.0806735060325	-0.011353073049

0.0807405386248	-0.011345597461
0.0808443008117	-0.011248584965
0.0809167772338	-0.011146349318
0.0810082490107	-0.011074593479
0.0810987821526	-0.010938539886
0.0811724457028	-0.01084201163
0.0812546933401	-0.010780796855
0.0813413551549	-0.010773630295
0.0814306323554	-0.010637278862
0.0815222684326	-0.010562169811
0.0816242737575	-0.010517812786
0.0816983647536	-0.010492009709
0.0817862680378	-0.010432058564
0.0818594996054	-0.010412393786
0.0819444290505	-0.010341813302
0.082016733001	-0.010337369537
0.0820934965144	-0.010312752229
0.0821762884248	-0.010227470114
0.0822410892562	-0.010150681863
0.0823197256268	-0.010058574945
0.082402237114	-0.010024738718
0.0824786694354	-0.009773012195
0.0825599828633	-0.00964772845
0.0826576130228	-0.009545797614
0.0827564094596	-0.009460339019
0.082851546421	-0.009448233737
0.0829338525652	-0.009448200928
0.0830142535005	-0.009356895383
0.0831052772848	-0.009023107636
0.083197617028	-0.008930615659
0.0832786636734	-0.008929428779
0.0833455210553	-0.008863159505
0.0834223776346	-0.008717690938
0.0835121370526	-0.008557254074
0.0835819739871	-0.008534224262
0.0836515713761	-0.008312457002
0.0837308558866	-0.008220466686
0.0838257401246	-0.007819856196
0.0839000843525	-0.007805198411
0.0839841906734	-0.007373023023
0.0840580063387	-0.007265563132
0.0841462079254	-0.007005251454
0.0842373967936	-0.006734313501
0.084308742998	-0.006682449794
0.0843936769658	-0.006589412642
0.0844926151205	-0.006483277095
0.0845830874027	-0.006406796055
0.0846633070797	-0.006364177671
0.0847571561917	-0.00593282202
0.0848398814219	-0.005921430485
0.084939256299	-0.00587265762
0.0850232331668	-0.005793311069
0.0851001485455	-0.0057407495
0.0851761487729	-0.005707435376

0.0852535785475	-0.005531766084
0.0853311341774	-0.005485200864
0.0854066901338	-0.005464612809
0.0854858474685	-0.005392976013
0.0855693083433	-0.005376744695
0.0856325944115	-0.005202999313
0.0857171415016	-0.005164854564
0.0858028516916	-0.005060504692
0.0858904159252	-0.004860609899
0.0859652245694	-0.004843530798
0.086055149111	-0.004785066142
0.0861387204064	-0.004551573138
0.0862181527509	-0.004489326139
0.086299058938	-0.004208522818
0.0863732968341	-0.004199950082
0.0864749442945	-0.004174327073
0.0865668521179	-0.004070700373
0.0866509674433	-0.00387317221
0.0867549798241	-0.003597921036
0.0868403871679	-0.003185458882
0.0869189615191	-0.003150642947
0.0870137394693	-0.002878082299
0.087094385314	-0.002709016183
0.0871704213539	-0.002606079018
0.0872656520331	-0.002590926213
0.0873541755544	-0.002349063627
0.087442894145	-0.002341977522
0.0875239888876	-0.00227021772
0.0876230702048	-0.002254103106
0.0877159758298	-0.002141884404
0.0877908986664	-0.002088148323
0.0878778203047	-0.002001432978
0.0879659954085	-0.001925482178
0.0880445371057	-0.001646980094
0.0881254469254	-0.001631044314
0.0882280872672	-0.00151588091
0.0883149539266	-0.001261107465
0.0884051671921	-0.001244680756
0.0884958826335	-0.001189877796
0.088579238862	-0.001189177308
0.0886609635162	-0.00108919163
0.0887540401924	-0.001089188176
0.0888346120648	-0.001046871679
0.0889156322722	-0.000611311206
0.0890114476386	-0.000443213665
0.0890989544618	-0.000389515109
0.0891920807692	-0.00031573475
0.0893009640045	-0.000293620839
0.0893781235545	-0.000235238854
0.0894642636047	-4.95966822E-05
0.0895699444144	-1.03643493E-15
0.0896658661967	0
0.089755053079	0
0.0898324901867	0

0.0899133436715	0
0.0900014057364	0.0002522939821
0.090083663489	0.0004548675341
0.0901644321402	0.0006535298056
0.0902451029352	0.0006563046219
0.0903238899999	0.0006578818172
0.0904128480008	0.0007117892551
0.0904718016823	0.000887694143
0.0905688818852	0.0011027646471
0.09066240521	0.0011142388629
0.090748796499	0.0011148134663
0.090836156806	0.0011674683548
0.0909143937446	0.0014762491453
0.0909864084804	0.0014964837128
0.091062395325	0.0015881439133
0.0911509684513	0.0018125446398
0.0912401877411	0.0020527531887
0.0913167261271	0.0021112289728
0.0913908943897	0.0021535539068
0.0914830940585	0.0021884309188
0.0915702041134	0.0022633875095
0.0916634853188	0.002358076743
0.091751033376	0.0024079585155
0.0918329467121	0.0024240647148
0.0919345107683	0.002425697492
0.0920074208243	0.002755063514
0.0921009526783	0.0029048638432
0.0921748160832	0.0029238072174
0.0922560208965	0.0030115695219
0.0923464686055	0.0030217638948
0.092425248887	0.0030810784433
0.0925057667603	0.0031444524077
0.0925840411772	0.0032827682056
0.0926631656469	0.0033749377777
0.092749231777	0.003532137804
0.0928490198965	0.00356883626
0.0929306814462	0.0036485162168
0.093015435035	0.0036971359916
0.0931051496042	0.0037930997096
0.0931839704844	0.0037964698713
0.0932850339681	0.0039134754476
0.0933631444935	0.0041617009472
0.0934461972022	0.0042381035439
0.0935297151206	0.0042779914251
0.0936073199947	0.0043146633451
0.0936695624498	0.0043710466917
0.0937464205562	0.0047408756895
0.0938303120646	0.0047437088693
0.0939060357682	0.0050975690698
0.0939785351427	0.0051002416193
0.0940615303337	0.0052997388196
0.0941399995457	0.0053711157886
0.0942354776173	0.0054017363079
0.0943265893985	0.0054730989372

0.094414829156	0.0054799783931
0.0945104131157	0.0054824970646
0.0945991227895	0.0056518835249
0.0946878368618	0.0057520648693
0.0947788192648	0.0060293918833
0.0948615716538	0.0061380836215
0.0949505126744	0.0061536867596
0.0950294646035	0.0062899289996
0.0951060917771	0.0062936866338
0.0951880338135	0.0063420209476
0.0952800724743	0.0063479421184
0.0953488248958	0.0063525147133
0.0954390640608	0.0065124091993
0.0955286452947	0.0065215112462
0.0956129046891	0.0065431157517
0.0956935226839	0.0065468402989
0.0957705901845	0.0065848172348
0.0958379154892	0.0066628083167
0.0959235557378	0.0068112057181
0.095999209657	0.0068181651379
0.0960919195705	0.0068355344939
0.0961748731837	0.0068553283684
0.0962674846389	0.0068647767793
0.0963408908252	0.0069356884166
0.096423888989	0.0075329124832
0.0965188788999	0.0075459884083
0.0966167216944	0.0075788789828
0.0966918752716	0.0077082516796
0.0967714511607	0.0077884450029
0.0968568182419	0.0077960344894
0.0969433944673	0.0078404709532
0.0970214391486	0.0081085569574
0.0971118800529	0.0081368975388
0.0971879146624	0.008249680537
0.09727307744	0.008440301307
0.0973501814607	0.0084803352946
0.0974247390281	0.0092938147322
0.0975092094435	0.009295050256
0.0975868957612	0.0093540365491
0.0976594524435	0.009488901656
0.0977337258464	0.0095977962904
0.0978278013878	0.0097264877045
0.0979331520579	0.0097351404657
0.0980246657645	0.0099266595608
0.0980987775013	0.0103210536512
0.0981780368926	0.0105096986344
0.098248858884	0.0105178410057
0.0983411259249	0.0107335265616
0.0984500033349	0.0107899248618
0.0985395577389	0.0107958741629
0.0986191342723	0.0107980923101
0.0987091350646	0.0108276579665
0.0987796322892	0.0109819541336
0.0988679380925	0.0110770950609

0.0989535030805	0.0111898421788
0.0990241229104	0.0112592971394
0.0991164631489	0.011328374514
0.099217613161	0.0113684155191
0.0993065287877	0.0114547848125
0.0994002547427	0.0115469052762
0.09947065905	0.0116070873813
0.0995567257655	0.0117751689125
0.0996401848222	0.0123413381704
0.0997348937122	0.0127228828214
0.0998248085748	0.0128539964616
0.0999129926542	0.0134098648529
0.0999885814556	0.0135377279256
0.1000796654954	0.013538530732
0.1001777801317	0.0136661116182
0.100257023043	0.0137569616496
0.1003465518942	0.0138546777336
0.1004253848723	0.0138815170463
0.1005087375728	0.0140659909943
0.1006005111331	0.0146799774399
0.1006784523946	0.0146888458661
0.1007561592138	0.0148163731521
0.1008271264353	0.0148163972121
0.1009205018079	0.0149583043566
0.1010133195851	0.0149974532554
0.1011095845039	0.0150042967206
0.1011890519571	0.0150379438072
0.1012898128558	0.0153165061756
0.1013764160224	0.0156964614253
0.1014626431844	0.0157767864498
0.1015452116585	0.0157984136076
0.1016262361545	0.0160132978685
0.1017081923021	0.0163497302781
0.1018070895493	0.0166027304936
0.1018908988973	0.0166333012967
0.101977913007	0.0168786469837
0.1020807429379	0.0169685637644
0.102160440431	0.0170061741568
0.1022470337431	0.0170111358721
0.1023471144532	0.0171309603104
0.1024225346608	0.0171364871474
0.1025062281232	0.0174073375994
0.1025870114982	0.017509575651
0.1026676277584	0.0175146604933
0.1027626255632	0.0175672895869
0.1028386957098	0.0178620204153
0.1029079319453	0.0179565759355
0.1029921243975	0.0181193678411
0.1030569319376	0.0182161410693
0.1031317318416	0.0182246006419
0.1032030518538	0.018272862936
0.1032728815469	0.0182902919977
0.1033751462182	0.0183165198189
0.103468012176	0.0185565303

0.1035495604978	0.0185729479465
0.1036402562088	0.0186321136202
0.1037245784126	0.0186700161776
0.1038118998155	0.0187676037814
0.103887240037	0.0188033836402
0.1039749947463	0.0188231771856
0.1040608864269	0.0189031773839
0.1041446473714	0.0189287368564
0.1042308132124	0.0189771652865
0.1043189494023	0.0189772526616
0.1044038749902	0.0191284186956
0.104484021094	0.019193522928
0.1045579286511	0.0191947394409
0.1046427230026	0.0192294453242
0.1047244353271	0.0193381024151
0.1048147758684	0.0194155859614
0.1048957547463	0.0195560523639
0.1049901733559	0.0196664198753
0.1050830435071	0.0196754817273
0.1051778057902	0.0199336778585
0.1052658172043	0.0201755258504
0.1053542901201	0.0202479029854
0.1054303494588	0.0203451864669
0.1055208537147	0.020385733125
0.1056144391695	0.0205931713363
0.1056963540002	0.0207003950528
0.1057633715286	0.0207278549456
0.1058466650019	0.0207562917536
0.1059274689619	0.0210503316113
0.1060211760193	0.0211500569979
0.1061006272943	0.021178389344
0.1061762202324	0.0212906717901
0.1062676794307	0.0213381049817
0.1063390215849	0.0214372427235
0.1064204175949	0.0215053392841
0.1064962778455	0.0215741043649
0.1065832544661	0.0215802902086
0.106657990149	0.0216046859462
0.1067379550084	0.0217210620066
0.1068282608399	0.0218243443051
0.1069184130312	0.0218478083858
0.1069871591559	0.0219105306069
0.1070711938215	0.0219144650441
0.1071549803081	0.0220337261349
0.1072434521323	0.0223895348265
0.1073484438937	0.0224176331021
0.1074170564059	0.0225705435564
0.1075070151364	0.0226366594458
0.1075670685509	0.0226552986677
0.1076484999751	0.0226925379446
0.1077569800648	0.0230146672009
0.1078652083294	0.0232228774705
0.1079509116589	0.0233819940466
0.1080181508133	0.0233853974459

0.1080998724037	0.0234392606858
0.1081822147074	0.0235127639073
0.1082836283287	0.0236243900823
0.1083674990104	0.0236940202163
0.1084490380633	0.0237256608235
0.1085516916735	0.0237531604751
0.1086454022126	0.0238623797994
0.1087290640721	0.0240659776979
0.1088248104734	0.0241074134405
0.1089284372451	0.0241624735463
0.1090061440016	0.0241813239877
0.1090931385085	0.0242473031329
0.109185292421	0.0243197047318
0.1092701414482	0.0245876481288
0.1093545079124	0.0246301785056
0.1094450159384	0.0246364155352
0.1095431754337	0.0247332417254
0.1096224845889	0.0248009626597
0.1097086029549	0.0248105136858
0.109789031339	0.024874467766
0.1098669826024	0.0249223743379
0.1099397716812	0.0251123265275
0.110012353323	0.0251274397244
0.1100836857087	0.0251594393022
0.1101505779545	0.0252637874832
0.1102300818058	0.0253366165138
0.1103151968549	0.0255286518832
0.1104016261684	0.0258787313198
0.1104766580694	0.0260650748444
0.1105602932412	0.0261012701467
0.1106340989646	0.0261227338878
0.1107135416928	0.0261244663411
0.1107894847892	0.0261980795441
0.1108822765411	0.0263257415317
0.1109667389904	0.0263596600411
0.1110505601718	0.0263601613949
0.1111411373247	0.0265530427365
0.1112188964685	0.0266955481005
0.1112972700069	0.0267067325167
0.1113774539866	0.026723202669
0.111462764874	0.0267510259638
0.1115470076106	0.0268432030073
0.1116294966955	0.0270171761708
0.111711882379	0.0270429481674
0.1117886575516	0.0270827716878
0.1118718901005	0.0271269623356
0.1119553042028	0.0272282518634
0.1120528393349	0.0275058324288
0.1121439883516	0.0275222854845
0.1122363333906	0.0275248697833
0.1123049495964	0.0275728225852
0.1123945609865	0.0278490850732
0.1124811338368	0.0278620267607
0.1125510239629	0.0278985745584

0.1126386522987	0.0279250008922
0.1127159866769	0.0280035091981
0.1128032874031	0.0281389426198
0.112882633115	0.028159903006
0.1129747817373	0.0284505554291
0.1130585316051	0.0285703639318
0.1131427046962	0.0286879498091
0.1132220578518	0.0287135239787
0.1133075711644	0.0288687919511
0.1133859317858	0.0289377382548
0.1134613714595	0.0293285559709
0.1135403470914	0.0293747829568
0.1136085777439	0.0294032925256
0.1136938684211	0.0294426679166
0.1137895609593	0.0295390424349
0.1138696006855	0.029726356426
0.1139489694775	0.0298725988832
0.1140267863441	0.0298736301113
0.1141022075275	0.0301179604691
0.1141911122791	0.0301710612757
0.1142756078319	0.0301908765298
0.1143436219317	0.0302053315497
0.1144233091853	0.0302435250903
0.1144972078759	0.0303803753398
0.1145767647029	0.0304235218181
0.1146569479475	0.0306858198628
0.1147376694393	0.0306984630945
0.114810252234	0.030784529088
0.1148928359635	0.0307896136746
0.1149838753179	0.0309401555922
0.1150684966478	0.0309584595951
0.1151529344353	0.0309809197482
0.115241511439	0.0309958184938
0.1153243819538	0.0311417581984
0.1154087795311	0.031174084187
0.1155013253492	0.0312000052057
0.1155848764026	0.0312618219261
0.1156779615936	0.0313569927875
0.1157541534765	0.0314030653646
0.1158442880297	0.0314124878961
0.1159190917975	0.0314805448374
0.1160068466793	0.0315807314545
0.1160938533378	0.0316326009026
0.1161982736186	0.0317349844212
0.1162653249245	0.0319753308159
0.1163591683087	0.0320515740004
0.1164467379278	0.0322449364095
0.1165336890989	0.0323945634296
0.1166204281611	0.0324675486239
0.116706007421	0.0326359973305
0.1167894755171	0.0326990650033
0.116879960194	0.0327583018663
0.1169631865799	0.0327777366662
0.1170547547732	0.032824164363

0.1171209505271	0.0330654885345
0.1172018473739	0.0330700547719
0.1172885767912	0.033097870007
0.117383124524	0.033163042811
0.11748159294	0.033230736172
0.1175599247901	0.0333821067901
0.1176277576859	0.0334401595934
0.1177037241481	0.0335221460258
0.1177846745763	0.0335221469173
0.1178553688162	0.0335526128155
0.1179304422574	0.0336486394026
0.1180255336751	0.0337314747185
0.1180959678982	0.0337700838677
0.1181675352185	0.0338068150456
0.1182553782561	0.0338225296224
0.1183705776461	0.0339742422648
0.1184694225481	0.0339774248299
0.1185727513665	0.0341618798034
0.118646138439	0.0341676867937
0.1187259041672	0.0341819347938
0.1188021686734	0.0341845018882
0.1188830424972	0.0342083965354
0.1189686609781	0.0342522790326
0.1190543667338	0.0343443716316
0.1191382290374	0.0344598117105
0.1192322184443	0.0344780021527
0.1193086558156	0.034685424973
0.119392190872	0.0348541582991
0.1194843820066	0.0349747794409
0.119565866294	0.0350005000502
0.1196514085092	0.0350142469052
0.119757357583	0.0351026781652
0.1198326976678	0.0351479463184
0.1199146876151	0.035376219272
0.12001071604	0.0354346993394
0.1200924995536	0.0357481570104
0.1201798171977	0.0359523810467
0.1202595890895	0.0362343828964
0.1203637594558	0.0362390353177
0.120457604457	0.0363651697385
0.1205463303916	0.0364847350449
0.1206441363463	0.0366161080901
0.1207220175174	0.0367576743978
0.120810219906	0.0369320066349
0.1208928522857	0.0371455961525
0.1209797084641	0.0372007231264
0.1210503193983	0.0372413937406
0.1211341330012	0.0372524760206
0.1212028367987	0.0374887720294
0.1212761591525	0.037682228916
0.1213784041536	0.0376938098659
0.1214681290605	0.0378497537827
0.1215373839751	0.0379174198995
0.121635400439	0.0380527002723

0.1217469206241	0.0381648777021
0.1218582127395	0.0386579116868
0.1219568991	0.0387688201275
0.1220385060165	0.0388638810916
0.1221296488453	0.0389527306344
0.1222166282258	0.0389783322873
0.1222886284451	0.0390233451125
0.1223706323382	0.0390867537882
0.1224404137409	0.0391161483965
0.1225184861059	0.039205988295
0.1225942669133	0.0393331922275
0.1226855500914	0.0395805922206
0.1227728374197	0.0396659246962
0.1228705133803	0.0400465801067
0.1229798089578	0.0400638078365
0.1230717584216	0.0401072332855
0.1231703217907	0.0404034424765
0.1232653547382	0.0405122744932
0.1233370626435	0.0405406975587
0.1234246301743	0.0405466545738
0.123526130066	0.0405613216213
0.1236202566404	0.0405663146739
0.1236984424792	0.0405961595393
0.1237811818862	0.0406702668086
0.1238550192106	0.0407183517177
0.1239554615829	0.040796118726
0.1240444135142	0.0408088780863
0.1241045942101	0.040827779337
0.1241794029216	0.0409182693515
0.1242507108975	0.0410256938485
0.1243469475949	0.0410322621459
0.1244210573095	0.0410340927845
0.1245053348506	0.0410770911579
0.1245695974908	0.0410801270842
0.1246361659036	0.0411043033497
0.1247190733667	0.0412145469734
0.1247864215936	0.0413828577644
0.1248599907337	0.0415179359857
0.1249382315798	0.041604186783
0.1250220067723	0.0416583869615
0.1251083428033	0.0417075624893
0.1251849164147	0.0418708409777
0.1252640838129	0.0418818123096
0.1253691479967	0.0419718957536
0.1254453829324	0.0420467883206
0.1255293670614	0.0421209414828
0.1256109658335	0.0424427961416
0.1256942365935	0.042660364099
0.1257796142735	0.042766316093
0.125858789962	0.042845196602
0.1259323313643	0.0430136547001
0.1260219645861	0.0431302676129
0.1261001677923	0.0431848519909
0.1261931248365	0.0432090898107

0.1262867204245	0.0432730447873
0.1263689249351	0.0434795857541
0.1264653687156	0.043703986569
0.1265449157942	0.0437231321039
0.1266251495715	0.0438596969991
0.1267271435867	0.0439157350912
0.1268148877921	0.0440237914014
0.1269037878237	0.0440655010907
0.1269907253998	0.0440806772285
0.1270502010337	0.0441339388504
0.1271304280572	0.0444942495254
0.1272179722037	0.0445793947784
0.1272963919711	0.0446296183316
0.1273736757427	0.044850422008
0.1274559195926	0.0451418926715
0.1275323979295	0.0452789981654
0.1276250935489	0.0453399747247
0.1277079048454	0.045472243307
0.1278019450559	0.0454723036194
0.1278848869124	0.0455096290335
0.1279716165575	0.0455225047198
0.1280494023136	0.04556010525
0.1281281018665	0.0458405947319
0.1282152597258	0.0459049916241
0.128310075772	0.0460789268466
0.1283883373999	0.0461746512517
0.1284646591824	0.0462335086375
0.1285483216711	0.0462720710849
0.1286443229722	0.0466201397743
0.1287212675307	0.0468119912088
0.1288104082031	0.0468561653036
0.1288998497594	0.0468653989868
0.1289753128198	0.046915208708
0.1290717204526	0.0469187027273
0.1291532554116	0.0469573086551
0.1292456907038	0.0470715384093
0.1293118184608	0.0470886819322
0.1293978974817	0.0472130625468
0.1294759117461	0.0472204540871
0.1295556709136	0.0472422036099
0.1296390147575	0.0474189886135
0.1296977943155	0.047496333184
0.1297985211552	0.0475549615551
0.1298903230835	0.0478486985589
0.1299870071668	0.0479886416805
0.1300705364853	0.0480639116629
0.1301575960842	0.0482726758061
0.1302517267242	0.0484983267926
0.1303484750325	0.0485794164807
0.1304454707404	0.0486100950931
0.1305418498835	0.048633435522
0.1306229325554	0.048682201477
0.1307001352769	0.0486904149882
0.1307958318511	0.0488796514925

0.1308759011218	0.0490087200781
0.1309736763122	0.049096967141
0.1310708363728	0.0491891050213
0.1311700060341	0.0493075139463
0.1312578529309	0.0493532245327
0.1313368302104	0.0493725656419
0.1314254222729	0.0495402700905
0.1315046256191	0.0495443923966
0.13159135896	0.0496233049113
0.1316869902804	0.0498184616354
0.13179251293	0.0498475479262
0.1318744079299	0.0498510291166
0.1319526339131	0.0499802501574
0.1320234246691	0.0500573375389
0.1320912249105	0.0502536668901
0.1321640510292	0.0503577682018
0.1322357558916	0.0503703683765
0.1323208381239	0.0503815580852
0.132406250717	0.0504224907269
0.1325026091477	0.0504722283703
0.132588699374	0.0505633966038
0.1326673452234	0.0505935010628
0.1327439251904	0.0507290952667
0.1328383406072	0.0508227787251
0.132926337444	0.0508816878626
0.1330036511455	0.0511666915502
0.1330857746643	0.0512327529711
0.1331768676021	0.0512846720771
0.1332638276044	0.0514318439894
0.1333403590353	0.0514657810383
0.1334290286616	0.0514814613193
0.133495237861	0.0516274034379
0.1335697386853	0.0518137300615
0.1336424393987	0.0518235219082
0.1337265184752	0.0520566179976
0.1338128252424	0.0521219848223
0.1338917008199	0.0521659130733
0.1339677615379	0.0521802189379
0.1340516248958	0.0522250860153
0.1341324015683	0.0525012099949
0.134211627656	0.0527002777913
0.1343026197566	0.0529742012906
0.1343802527908	0.0530763904809
0.1344640503247	0.0530803657835
0.1345642490269	0.0530823264439
0.1346632753432	0.0534301515441
0.1347551224577	0.0535385889893
0.1348470616161	0.053628385245
0.1349293891773	0.0538133429976
0.1350004827954	0.0538467936887
0.1351012633347	0.0541462558319
0.1352001367149	0.0542246844703
0.1352796646632	0.0544160327733
0.1353543577668	0.054500119392

0.135453274061	0.0546178587298
0.135541555469	0.0548662630925
0.1356292515521	0.0549182392094
0.1357010614792	0.0549918068759
0.1357935412531	0.0552233798784
0.1358786656157	0.0552880464287
0.1359723829443	0.0552938111111
0.1360606290417	0.0553057547692
0.1361563960167	0.0553263168318
0.1362567350986	0.0553645810681
0.1363411475361	0.0554047363279
0.1364092585554	0.0554134242245
0.1364858324259	0.0555896201816
0.1365465431698	0.0557124183011
0.1366251804422	0.0557398050383
0.136698432602	0.0557762778884
0.1367772634165	0.0558332212637
0.1368672691525	0.0558943943056
0.1369521036791	0.0560996708496
0.137031744083	0.0561492130245
0.1371104152736	0.0565490413133
0.137191358181	0.0566464346699
0.1372710790068	0.0571551115212
0.1373555977677	0.0571563805304
0.1374482777049	0.0572321309383
0.137528095153	0.0572378844425
0.1376166808865	0.057641378983
0.1377267933178	0.057667169811
0.1378076408195	0.0578026082527
0.1378986663709	0.0578026808103
0.1379925836303	0.0578039153016
0.1380803083544	0.0580490924392
0.1381707805987	0.0580627188914
0.1382673528503	0.0581936497969
0.1383559783458	0.0582027328071
0.1384335363738	0.0582593287537
0.1385048382307	0.0584151709669
0.1385863882772	0.0584680092973
0.1386707555286	0.0584844980408
0.1387702772822	0.0585780667477
0.1388630531507	0.0586079489114
0.1389279993653	0.0586236879496
0.1390256412193	0.0586997504972
0.1391093260292	0.0587046102561
0.1391947760657	0.0588416615605
0.1392743897426	0.0589201855232
0.1393715092015	0.058954586729
0.1394566580646	0.0589836172512
0.1395450340587	0.0590440265592
0.1396206397083	0.0590705556297
0.1397168472962	0.0593060998224
0.1398074714001	0.0594577938949
0.1399053484654	0.059797641532
0.1400048805421	0.0599675033914

0.14011114695	0.0602351526568
0.1401827945922	0.0603865956698
0.1402748164582	0.060422868654
0.1403636281762	0.0604232750697
0.1404376862686	0.060556087493
0.1405207630759	0.0606700310044
0.1406022221599	0.0608057888341
0.1406738318047	0.0608356297477
0.140749504659	0.0608493126594
0.1408284367264	0.0608699669486
0.1408953461283	0.0610413757992
0.1409769503708	0.0610477431288
0.1410615419492	0.0613719364849
0.1411479568578	0.0614193669647
0.1412180124278	0.0615566559628
0.141299921553	0.0618436966825
0.1413820706862	0.0619742962427
0.1414658754575	0.0620073945942
0.1415429268512	0.0620159144881
0.1416288229381	0.0620600395175
0.1417279168591	0.0620718816561
0.1418242607517	0.0621044666327
0.1419171452463	0.0624331666374
0.1419930969649	0.0626241543737
0.1420792663391	0.0627360468249
0.1421672359209	0.0628256569768
0.1422545347419	0.0630494410497
0.1423476488627	0.0630789923565
0.1424381762983	0.0631166913647
0.1425405074282	0.0634122874776
0.1426281789446	0.0634317946396
0.1427222939027	0.0635248596933
0.1428201320222	0.0635298071064
0.1429026637765	0.0636748620676
0.1429892912595	0.0637478227752
0.1430785738963	0.0637652128027
0.1431564643821	0.0637948384675
0.1432445399002	0.063973733748
0.1433296430219	0.0639743250191
0.1434058707872	0.0640043694302
0.1434835213198	0.0641873175952
0.1435525990628	0.06427657489
0.1436445559045	0.0642867011545
0.1437412565724	0.0643283286052
0.1438250821549	0.0645240756466
0.143909850537	0.0645298311712
0.1439839716076	0.0645675602333
0.1440735846584	0.064615920096
0.1441706489364	0.0648914097813
0.1442444898547	0.0649201853634
0.1443297770877	0.0650615409497
0.1444171179422	0.0651962121814
0.1445005149315	0.0652330694671
0.1445710483034	0.0653446361279

0.1446601515988	0.0653680817607
0.1447338949594	0.0656398973646
0.1448232922323	0.0657071669387
0.1449029922312	0.0658309215787
0.1449867271222	0.0658447925922
0.1450634326474	0.0658718531709
0.1451423289609	0.066087689744
0.1452110706151	0.0661146179224
0.1452839350839	0.0661345695756
0.1453672013317	0.0662316647986
0.1454624658703	0.0662787320891
0.1455436108516	0.0662947214342
0.1456316561583	0.0663734587782
0.1457261219447	0.0668514670662
0.1457937650637	0.0669578579924
0.145875967377	0.0669700605094
0.145943537451	0.0670879831363
0.1460444794467	0.0671753312114
0.1461319694572	0.0671863430631
0.1462315098664	0.0672616115155
0.1463301307396	0.0673060252058
0.1464023677909	0.0673278357492
0.1464864084948	0.0673847716264
0.1465899947366	0.0674071349668
0.1466860076727	0.0674361678707
0.1467786259598	0.0678403884015
0.146864925981	0.0679010726194
0.1469356362355	0.0679068053704
0.1470250947309	0.0679094897109
0.1470961014769	0.0682204577509
0.1471754542596	0.0682612200799
0.1472512531573	0.0685553487912
0.1473487697067	0.0686162132592
0.1474144478619	0.0686820825581
0.1475190537199	0.068783530593
0.1476142944326	0.0688852033087
0.1477030040033	0.0689621428653
0.1477823223446	0.0690944829354
0.1478663641413	0.0692163451492
0.1479580424425	0.0695045026162
0.1480392925936	0.0695352394659
0.1481225947526	0.069557931285
0.1481940457834	0.0696166891284
0.1482799014676	0.0696629231093
0.1483573607908	0.0697058046692
0.1484432759935	0.0697786139441
0.1485112195637	0.0699487228556
0.1485986031904	0.0701198308981
0.1486666402151	0.070395955568
0.1487438850966	0.0705861605879
0.148831178943	0.0705869570819
0.1489235247626	0.0706123554847
0.1489992583119	0.0706557164748
0.1490778923595	0.0708276282616

0.1491688573226	0.07085182019
0.1492394093205	0.070875151344
0.1493243748996	0.070899831871
0.1494183700309	0.0710686552179
0.1495187445835	0.0712247324736
0.1496132654272	0.0712886312535
0.1497098738365	0.0713548031707
0.1497988711798	0.0713966340735
0.1498732771783	0.0715349201528
0.1499552285843	0.0716152311891
0.1500598548655	0.0716164704292
0.150141054688	0.0719188792681
0.15022325441	0.0721951881469
0.1503038506019	0.0722083743021
0.1503696357232	0.0723841559748
0.1504621119874	0.0725878380783
0.1505357473088	0.0727076316285
0.1506061913584	0.0732905995294
0.1506875292684	0.0734070764684
0.1507783202172	0.0734467981153
0.1508778134366	0.0734559500577
0.150974686854	0.0736670808699
0.1510519639745	0.0738505406639
0.1511293920429	0.0738888335979
0.1512173542486	0.0742565124311
0.1513080870883	0.0744602231952
0.1514080874415	0.0745010160454
0.1514826556944	0.0745319137775
0.1515765892357	0.0746119058357
0.1516478412453	0.0748334442146
0.1517280132756	0.0751760308904
0.1518114548467	0.0753236267501
0.1518836578726	0.075544194319
0.1519776249554	0.0757283571171
0.152071951722	0.0759430303557
0.1521560163163	0.0761400745861
0.1522265165203	0.0762159582757
0.1523076450008	0.0763902820279
0.1523960088749	0.076427321467
0.1524817482058	0.0764564731592
0.1525516681166	0.0764917060937
0.1526380173632	0.0767270137532
0.152717250775	0.0767620080546
0.1527973118414	0.076781213964
0.1528812202305	0.0769995633523
0.1529677442746	0.0770560120161
0.1530521926735	0.0771045085251
0.1531354753564	0.0771848560349
0.1532102342779	0.0773051514819
0.1532846256074	0.0773357450117
0.1533817268527	0.0773381293543
0.153472090881	0.0773736639051
0.153552508833	0.0774430000877
0.1536316203689	0.0775399454051

0.1537160948279	0.0776692385953
0.1538091134576	0.0778319349246
0.1538971370747	0.0778792163663
0.1539686572654	0.0780890571785
0.1540738753883	0.0782978740614
0.1541527335929	0.078313636533
0.1542591196815	0.0784121144361
0.1543563762381	0.0786035167323
0.1544442299658	0.0787142891169
0.1545295314991	0.0787188829833
0.1546045845304	0.0787675794505
0.1547002357058	0.0788210405753
0.1547691992249	0.0788572666152
0.1548573589356	0.0789107338411
0.1549576268913	0.0789423711492
0.1550326455111	0.0789726305954
0.1551219480672	0.079080359176
0.1552097943923	0.0791084066217
0.1552848171423	0.0791287460065
0.155372311592	0.0793661139666
0.1554609434485	0.0794551502489
0.1555380381413	0.0796798219097
0.15562341832	0.0799094107071
0.1557093717309	0.0800684257347
0.1557747571908	0.0802314330364
0.1558548159535	0.0803257795655
0.1559631066261	0.0804162661342
0.156048648722	0.080816879627
0.1561233049882	0.0808493753909
0.1562028001041	0.0809050065833
0.1562979182182	0.0810137173698
0.1564069482573	0.0811264080218
0.1565065736934	0.0812505398087
0.1565882236484	0.0813030665797
0.1566656303682	0.0815265866911
0.1567519858462	0.0815312768179
0.1568311042436	0.0819521634059
0.1569230525741	0.0819733049642
0.1569862185833	0.0819967329703
0.1570725594994	0.082041734632
0.1571572947173	0.0820922518632
0.1572502221815	0.0821144485511
0.1573417379758	0.0823581690189
0.1574237170231	0.0824932904305
0.1574892799105	0.0827379167949
0.1575773164568	0.082945063785
0.1576671175586	0.0829546813501
0.1577588513381	0.0830458695915
0.1578365107064	0.0831120488795
0.1579167675757	0.083310551978
0.1579973459105	0.0833639282007
0.1580639972518	0.0834472630892
0.1581634196979	0.0835343970301
0.1582549730961	0.0835390220428

0.158353675798	0.0838677552488
0.1584331214587	0.0843804243538
0.1585176365209	0.0843909078979
0.1586094877382	0.0844118093507
0.1586857570827	0.0845672044442
0.1587714681657	0.0846065105091
0.1588660044089	0.0846380423668
0.158938995082	0.0847333370978
0.1590149633196	0.08490829372
0.1590958145559	0.0849712828086
0.1591834465782	0.0849771222811
0.15926657092	0.0851030725415
0.15934630685	0.085139861811
0.1594204095104	0.0851892171268
0.1595074604024	0.0854978018034
0.1595993667201	0.0856093687128
0.159692392452	0.0856115054331
0.1597841373717	0.0856609668887
0.1598544936298	0.0856640594948
0.1599356202046	0.0856758191013
0.160026601023	0.08568829706
0.1601148525964	0.0857869909803
0.1601973429932	0.0858159623987
0.1602854886776	0.0858185415608
0.1603637367637	0.0858363718557
0.1604487622238	0.0858634040302
0.1605407699081	0.0861007325657
0.1606287767019	0.086138892283
0.160711638808	0.0862685072192
0.1607986423829	0.0863009589494
0.1608928456622	0.0863945832409
0.1609763102682	0.0865655199564
0.1610584158242	0.0865795802636
0.1611378890509	0.0867982042757
0.1612161241107	0.0868360272378
0.1612875032562	0.0869551989944
0.161374492938	0.0870561639156
0.1614657509497	0.0870676249608
0.1615590880471	0.0871825963512
0.161648612475	0.0872906821639
0.1617468477372	0.0873763124554
0.1618332419292	0.0875048205329
0.1619185056056	0.0876485698537
0.1619961629147	0.0876596488711
0.1620904131286	0.0877343735063
0.1621635820871	0.087797346914
0.1622443227543	0.0884137606689
0.1623194651649	0.0884743243171
0.1623996364374	0.0885883762992
0.1624800437446	0.0886352165886
0.1625681452291	0.088647500161
0.162652967903	0.0886640018294
0.1627394560327	0.0887267897822
0.1628193170015	0.0887846353334

0.1629136513952	0.0889007175194
0.1629999357181	0.0889449225989
0.1630835302729	0.0889464547189
0.1631584348238	0.0890241472024
0.1632445642881	0.0891178345612
0.1633351260309	0.0892135416204
0.1634200253409	0.0894044123048
0.1634975026085	0.089742795222
0.1635809983666	0.0897439396833
0.163673527358	0.0897467466469
0.1637640577914	0.0898609046207
0.1638476035545	0.0899326941333
0.1639177801088	0.0900179423516
0.1640198857313	0.0900483663677
0.1641073458124	0.0901614104797
0.1641981072571	0.0906148730254
0.1642790530904	0.0908141396172
0.1643548391673	0.0910568816978
0.16444751305	0.0912947486842
0.1645274301366	0.091517812823
0.1646082318268	0.0915615916179
0.1646949745093	0.0916577217318
0.1647770456974	0.0917032656949
0.1648560944385	0.0917768181216
0.1649559289617	0.0917820197952
0.1650410331346	0.0918337932855
0.1651318326503	0.091849474358
0.1652052098481	0.0918812555833
0.1652799653153	0.0919139252033
0.1653687193571	0.0919965300586
0.1654658082549	0.0921386604291
0.1655451576437	0.092244305665
0.1656238014194	0.0922877291883
0.1656983048935	0.092369382082
0.1657770976777	0.0924193196402
0.1658683475743	0.0924860853607
0.1659549160052	0.0926587582073
0.166029680451	0.092685510278
0.1661074761913	0.0927222339291
0.1661859726241	0.0929456875684
0.1662778182821	0.0930570277494
0.1663705741222	0.0931229446942
0.1664393070374	0.0932577709862
0.1665208370105	0.0933656898331
0.1666134098792	0.093521797189
0.1666815578323	0.0935741942427
0.1667565006842	0.0937108562074
0.1668416515564	0.0938021750345
0.1669387543017	0.0940614438246
0.1670108531609	0.0941255574796
0.1670884937924	0.0943876079359
0.1671708162213	0.0944769303278
0.1672476695135	0.0944926136837
0.1673332710895	0.0946686366666

0.1673990999213	0.0947337504578
0.1674629027593	0.0947511594339
0.1675628273306	0.0947833940493
0.1676352782159	0.0949516634723
0.1677146141272	0.0951419564867
0.1677885110319	0.0951465291756
0.167877938444	0.0952159447672
0.167960646098	0.0952452777077
0.1680467447477	0.0955081589439
0.1681341448011	0.0956486605187
0.1682220481898	0.0957772027281
0.1683022054876	0.0958161179976
0.1683767695667	0.0958726838567
0.1684688344134	0.0959970430248
0.1685508609379	0.0960218868447
0.1686430282406	0.096046386509
0.1687161297872	0.0960725460681
0.1688046987836	0.0964634233667
0.1688955753531	0.0966196124951
0.1689823497587	0.0967578870465
0.1690740420304	0.0967690769186
0.1691538727853	0.0968157701691
0.1692397989951	0.096873683665
0.1693311568463	0.0969661240258
0.1694070000374	0.0969702862841
0.1694972063506	0.0970063477757
0.1695728552816	0.0970924610128
0.1696498754156	0.0973390709756
0.1697433457584	0.0973938272501
0.1698221194404	0.0976740334299
0.1699116846173	0.0976909796297
0.1699855142986	0.0977772080714
0.1700634502133	0.0978582744848
0.1701471031228	0.0979743734419
0.1702368213803	0.0980159082353
0.1703380598077	0.0984092789266
0.1704172990892	0.0985265785779
0.1704928502	0.0986066352532
0.1705859550288	0.0987945221191
0.170654952379	0.099301445308
0.1707451408051	0.099446277436
0.1708202484657	0.0995118505348
0.1709061541101	0.0997471911112
0.1709927009715	0.0997758064348
0.1710775456896	0.1000218604594
0.1711761705659	0.1000766726495
0.1712690349771	0.1000823184466
0.171356105983	0.1002878281731
0.1714354601355	0.1003239099731
0.1715148916204	0.1003604072333
0.1715958983303	0.100464846526
0.1716950020403	0.1005464030764
0.1717785110478	0.1009756064051
0.1718582217689	0.10100652725

0.1719586215024	0.1010763048744
0.1720405292265	0.1010784521554
0.1721274901602	0.1010949683008
0.1722263715724	0.1011893137322
0.1723029479931	0.1012429102687
0.1723826481263	0.1012799882696
0.1724589616216	0.1013693648846
0.1725463402663	0.1014293537003
0.1726375468377	0.1014825777251
0.1727246429037	0.1015402625784
0.1728175324938	0.1016780249072
0.1728982279212	0.1017387482217
0.1729854011178	0.1017806394114
0.173065755273	0.1019831891801
0.173154270433	0.1020422205164
0.173236748327	0.1021181685277
0.1733305597389	0.1025485999729
0.1734238620936	0.1025512664945
0.1735059425164	0.1026109600119
0.1735875312537	0.1028917586955
0.1736678396616	0.1029453054401
0.1737481235841	0.1029690560341
0.1738274835076	0.1029864767723
0.1739188554441	0.1030245794451
0.1740146685081	0.1031869567984
0.1741102498412	0.1033377852671
0.1741987626308	0.1034212923939
0.1742776469555	0.1034636389635
0.174360202962	0.1034778377961
0.1744432997666	0.103549133829
0.1745217831294	0.1036282854024
0.1746074555898	0.1036607014631
0.1746960325035	0.1037178862819
0.1747738911452	0.1037966818324
0.1748468232303	0.1037979209002
0.1749337355611	0.1038670523127
0.1750107726138	0.1040351949806
0.1751015167154	0.1043363009951
0.1751923463613	0.1043420861595
0.1752824901939	0.1044075502702
0.1753673368165	0.1044669361738
0.175447501061	0.1045467773724
0.1755395038993	0.1046449734843
0.175635702775	0.1048891276911
0.1757385263194	0.1049242447351
0.1758233260066	0.1050365429627
0.1759143201299	0.1051444617794
0.1759870852962	0.1051694255568
0.1760735975184	0.105364804271
0.1761577074443	0.1054025598361
0.1762471903716	0.1055809609075
0.1763406901799	0.1056447483348
0.1764153057456	0.1056501340664
0.1764930298526	0.1057715551979

0.1765696312123	0.1059296175683
0.1766454230308	0.1061553257201
0.1767456534331	0.106190555908
0.1768345904663	0.1063813962931
0.1769407521503	0.1065356779183
0.1770241197152	0.1065644633487
0.177115991979	0.1065809162303
0.1772158914795	0.1067316608565
0.1773124064	0.106755243284
0.1774040783459	0.1071369975077
0.1774855840428	0.1071643395076
0.1775649794508	0.1071681527559
0.1776478546984	0.1072166706152
0.1777350796994	0.1072967475681
0.1778144779826	0.1073674294139
0.1778967841966	0.1074002256368
0.1779698984906	0.1075237484621
0.1780637960132	0.1076251941871
0.1781473011414	0.1077350818204
0.1782264312893	0.1078631299394
0.1783130275636	0.1082074411383
0.1783943629584	0.1082518149845
0.178471837522	0.1083899301527
0.1785632932956	0.1083901221471
0.1786465539445	0.1084453579042
0.1787250671237	0.1084608678843
0.1788356662303	0.1085858468635
0.1789236592818	0.1086762680665
0.1790041749553	0.1086853158543
0.1790792680343	0.108739823627
0.1791659778525	0.1089076409864
0.179258333814	0.1091258092423
0.1793335771629	0.109154981578
0.1794122907662	0.109171545063
0.1794976281531	0.1092262466441
0.1795917468894	0.1093058575274
0.1796805392743	0.1094232574207
0.179752663119	0.1095689436653
0.1798321646517	0.1097245360594
0.1798992028837	0.1100156684349
0.1799888586174	0.1101433621997
0.180072332879	0.1101446403226
0.1801544369425	0.1103603687057
0.1802296508048	0.1111612189584
0.1803173964833	0.1114628000277
0.1803958299236	0.1115087861713
0.1804630208685	0.1117160894688
0.1805558969637	0.1117721750513
0.180649053416	0.1118420906447
0.1807296751287	0.1119089419208
0.1808180560973	0.1119901963996
0.1809248579022	0.1120087181081
0.1810100071634	0.1120468661933
0.1811026431192	0.1121786886402

0.1811867757412	0.1122517839686
0.181253108922	0.1122794664367
0.1813355756164	0.112531189051
0.1814233457217	0.1126202811373
0.1814982944432	0.1126437881786
0.1815940360974	0.1128577229994
0.1816744220631	0.1129182725212
0.1817533213052	0.1129232372553
0.1818254534369	0.113026712608
0.1819017572743	0.1131368831723
0.1819755640411	0.1131437038587
0.1820631593964	0.1132165007133
0.182155355251	0.1132195292387
0.1822313285353	0.113364498604
0.182328976823	0.1135309178294
0.1824141337946	0.1135360549929
0.1825003538274	0.1137624176094
0.1825741591096	0.1139116857956
0.1826870350667	0.1140309516616
0.1827655881394	0.1141044804447
0.1828629393328	0.114112304693
0.1829337666881	0.1141280170425
0.1830266133677	0.1142387336659
0.1831091914084	0.1142582453591
0.1832044884786	0.1145725027284
0.1832920616482	0.1145880097103
0.1833717048021	0.1146358156498
0.1834558562655	0.1146638400654
0.183548764593	0.1147657482003
0.1836313352977	0.1148766068285
0.1837028105529	0.1151247600577
0.1837894194099	0.1152452417138
0.183892268286	0.1153196155381
0.1839642556801	0.1154709024058
0.1840592942291	0.1156780397804
0.1841434657813	0.1157628116656
0.1842176218877	0.115995899915
0.184298773303	0.1160226999305
0.1843884833721	0.1160988803014
0.1844711009477	0.1162245896678
0.1845604041941	0.1163305826299
0.1846598657339	0.1163993468813
0.1847330278744	0.1164540686756
0.1848116084909	0.1164690851954
0.1848907312211	0.1164906503078
0.1849873666495	0.1167200879594
0.1850770757186	0.1167562312471
0.1851654789683	0.1168823722699
0.1852415623332	0.1169315267486
0.1853168071791	0.1169608512634
0.1854017776363	0.1169682939715
0.185479094436	0.1170221504856
0.1855677211842	0.1170347080915
0.1856608600386	0.1170987892045

0.1857465391033	0.1172310335999
0.1858191943948	0.1172532249045
0.1858931605183	0.1173654477451
0.1859799021782	0.117441224687
0.1860671970938	0.1175110274041
0.1861359328335	0.1176664998591
0.1862303832233	0.1177209143003
0.1863393569806	0.1178062060797
0.1864172364347	0.1180199079796
0.1864964404767	0.118111681695
0.1865853190284	0.1182208492063
0.186665533135	0.1183556477057
0.186748242914	0.1183586210521
0.18683740884	0.1184714651427
0.1869199363991	0.1187404825181
0.1870080539816	0.1188868326312
0.1870914373086	0.1188940736711
0.1871774887721	0.1189351744573
0.1872578115807	0.119242375093
0.1873451449957	0.1192816793187
0.1874228721123	0.1192947517375
0.1874961817433	0.1193648147497
0.1875819558669	0.1193781860407
0.1876886087487	0.1194179784321
0.1877955214109	0.1194785852885
0.1878821497477	0.1194953470908
0.1879716094826	0.1196552759489
0.1880627007362	0.1197549421111
0.1881423439595	0.1200098759842
0.1882260342158	0.1200381708661
0.1883140056591	0.1200518792194
0.188401210989	0.1200558461001
0.1884783715347	0.1200880808432
0.1885700721157	0.120261926745
0.1886430337234	0.1203779593146
0.1887421666878	0.1206294399457
0.1888203566463	0.1208359657413
0.1888943233722	0.1209855657549
0.1889726813774	0.1212661883605
0.1890721776889	0.121299055605
0.1891735775574	0.1214959267854
0.1892719702806	0.121615223601
0.1893586759053	0.1217903349325
0.1894434308502	0.121810805387
0.1895234690193	0.1219318883681
0.1896290539115	0.1219335193881
0.1897057496711	0.1219607588828
0.1897818857494	0.1221535623298
0.1898683370918	0.1221867659593
0.1899750058567	0.1223189560296
0.1900724210451	0.1223367078715
0.1901504143949	0.1225841068603
0.190237803898	0.1229287166748
0.1903263426648	0.1229568501981

0.1904117024461	0.1229846003562
0.1905090281415	0.1231204576912
0.1905944929646	0.1233626870424
0.1906757233171	0.1233907078682
0.1907561055437	0.1235824003301
0.1908476374142	0.1236462370105
0.1909370015967	0.1236954890731
0.1910073425573	0.1237699636463
0.1910798658736	0.1238002918756
0.1911659007431	0.1238053997133
0.1912308292583	0.1239776075257
0.1913181154091	0.1242167572038
0.191394830275	0.1242611519215
0.1914845895275	0.1243434075607
0.1915662549284	0.1243648366059
0.1916454537816	0.1246865302384
0.1917351643407	0.1246991807142
0.1918249788331	0.1248919151298
0.1919084933928	0.1249196189337
0.1920062112	0.1249459585851
0.1921034435742	0.1249616595668
0.1921814755706	0.1251582024094
0.1922628248003	0.1252169003834
0.192353946185	0.1254951325505
0.1924339079866	0.1255225876128
0.1925272888375	0.1255720678042
0.1926322976496	0.126069555165
0.1927208088213	0.1261808447112
0.1928183777891	0.1265274228396
0.1929296647678	0.1265996869451
0.193031805921	0.1266700090563
0.193127154044	0.1267482068148
0.1931941608517	0.1267714744014
0.1932807689882	0.126782109527
0.1933649642692	0.1268103478924
0.1934471020677	0.126862675872
0.1935493950882	0.1268776812682
0.1936288133437	0.1269277081463
0.1937094503039	0.1270104496462
0.1937955883537	0.1272232015236
0.1938752369588	0.1273436436681
0.1939651136647	0.1276002199445
0.1940447670684	0.1276908371742
0.1941219707775	0.1277716899551
0.1942119856367	0.1278176690671
0.1942938506358	0.1278407287214
0.1943889147324	0.1279235266779
0.1944798526077	0.127970632466
0.1945691883998	0.1280082551943
0.1946475337434	0.1280215588268
0.1947331717977	0.1280717323169
0.1948165809352	0.1281902578239
0.1948896676482	0.1282011788859
0.1949695518292	0.1282751918764

0.1950554695283	0.1283312773761
0.1951386945059	0.1284062632938
0.1952463895803	0.1284593572537
0.1953361549242	0.1284790143317
0.1954245639088	0.1289043882845
0.1955024376355	0.1289518069993
0.1956020350006	0.1290279360533
0.1956644029445	0.1291440433758
0.1957538934738	0.1292202165842
0.1958393980485	0.129331150509
0.1959332845775	0.1295026292823
0.1960171175264	0.1296103687667
0.1961017532529	0.1298728928129
0.1961934580466	0.1299543106286
0.1962850372119	0.1299605025384
0.196349651681	0.1299720273253
0.1964458673493	0.1299754180462
0.196523434006	0.1299803311574
0.1966203665968	0.1300093891876
0.1966957185264	0.1300668318714
0.1967635578496	0.1301394567826
0.1968434903984	0.1301782679992
0.1969282898182	0.1302584165119
0.1970074573611	0.1303854476093
0.1971176782228	0.13038786287
0.1971996468422	0.1305113694964
0.1972855104672	0.1305943256195
0.197372972871	0.1306391433271
0.1974501256533	0.1306695938861
0.1975215131864	0.1306806445237
0.1976049408244	0.130762577461
0.1977111557354	0.1309081383406
0.1978093419962	0.1310327187294
0.1978847993721	0.1313677486306
0.1979737379297	0.1314480500541
0.1980682204036	0.1314562658352
0.1981464666231	0.1316106780864
0.1982336864388	0.1316992286404
0.1983110054617	0.1317508183977
0.1984009474799	0.1321020749666
0.1984908895723	0.1323520489936
0.198574102375	0.1324448707349
0.1986800145049	0.1326270012837
0.1987655642347	0.1327347622823
0.198853588608	0.132885298148
0.1989358823868	0.132968183971
0.1990133044846	0.1331664175794
0.1990989650737	0.1331834592226
0.1991851773185	0.1333313902509
0.1992537671848	0.13376949649
0.1993323592574	0.1337887071457
0.1994258424158	0.133838761574
0.1995206966251	0.1338784302976
0.1996063021974	0.1340657983037

0.1997174308772	0.1340757903437
0.1997794980909	0.1344110338153
0.199853736737	0.1344782047698
0.1999380827927	0.1344853566103
0.2000158781564	0.1346353714095
0.2000956626739	0.1346361723539
0.200174771044	0.1347511797029
0.2002549031623	0.1348434562661
0.2003380686012	0.1349668362542
0.2004187076031	0.135059078837
0.2005028811933	0.135086918525
0.2005903650154	0.1352298275204
0.2006706546816	0.1353223818664
0.2007548342244	0.1354268833508
0.2008470300406	0.1356541658919
0.20092962394	0.1356570604032
0.2010254657173	0.1358615063815
0.2010959593713	0.1359609575067
0.2011677097889	0.1359882481721
0.2012371372485	0.1362633551925
0.2013414339847	0.1363105083185
0.2014301959796	0.1363764444443
0.2015327457815	0.1364655490924
0.2016142301397	0.1364749697362
0.2016955458781	0.1364886416465
0.2017783302011	0.1366063633129
0.2018586187658	0.1368709252918
0.2019363330771	0.1368990942811
0.2020048835123	0.1369274766718
0.2020870658052	0.1369990222517
0.202168225874	0.1370416067135
0.2022556623236	0.1370439371548
0.2023491015902	0.1370617122936
0.2024468627428	0.1372428481216
0.2025187984969	0.1373484065221
0.2026058267226	0.1373568081012
0.2026704498406	0.137404939924
0.202768010244	0.1374130331991
0.2028434735907	0.1374805058547
0.2029242626801	0.137558456079
0.2029829616776	0.1377349435181
0.2030802419008	0.1378308884189
0.2031506546849	0.138041871875
0.2032312095698	0.138206941097
0.2033120297181	0.1382296531282
0.2033963118259	0.1382578973606
0.2034750181705	0.1384304073747
0.2035620773492	0.1385119589552
0.2036493731562	0.1385445579191
0.2037262358534	0.1385586598969
0.2038058873695	0.1386494449899
0.2038916288511	0.1388668830141
0.2039925369843	0.1388812487686
0.204077154947	0.1390081956404

0.204177066679	0.1391935399722
0.2042744875001	0.1392061467322
0.2043630382181	0.1392296079337
0.2044546769961	0.139245906885
0.2045420651122	0.1392525443071
0.204621588739	0.1393557780265
0.2047065743383	0.1393934343212
0.2047899306553	0.139429562064
0.2048784948467	0.1394450553096
0.2049704778723	0.1395754426426
0.205062629271	0.1400073708563
0.2051433991517	0.1401124384656
0.2052153987562	0.1401288872244
0.2052998067499	0.1403750409208
0.2053836067784	0.1404556047847
0.2054694383541	0.1404609548037
0.2055505322136	0.1404747096808
0.2056452081962	0.1407678867032
0.2057278308039	0.1408685740481
0.2058172116412	0.1409403543923
0.2059058496111	0.140959294132
0.2059918987565	0.1409596521045
0.2060765912336	0.1410420896013
0.206172059591	0.1411081163862
0.2062607740128	0.1413918777912
0.2063447446921	0.141563083505
0.2064132155613	0.1415801423524
0.2065010411102	0.1415945828395
0.2065961308035	0.1418250579819
0.2066984436464	0.1419326887497
0.2067847907465	0.1419331966269
0.2068650351781	0.1419445972773
0.2069527193173	0.1419472459655
0.2070392599844	0.1420378145699
0.2071244681995	0.1420409995971
0.2072037276057	0.1421324438889
0.2072808822958	0.1421887013349
0.2073666149465	0.1423908258423
0.2074462862685	0.1423916807979
0.2075330125557	0.1427479693323
0.2076075885924	0.1427490873461
0.2076897314411	0.1429679469573
0.2077783955003	0.1430780014099
0.2078572634101	0.1431966923966
0.2079471423404	0.1432693744921
0.208042738666	0.1433490805877
0.2081414916381	0.1433931571957
0.2082194138942	0.1435733451503
0.2082989631593	0.1435819164897
0.2083961493646	0.1437311043877
0.208489036176	0.1438100837978
0.2085861466613	0.14391241806
0.2086823969818	0.1439832636268
0.2087761128331	0.1439882637148

0.2088591844754	0.1440429260015
0.2089468227773	0.1440630783534
0.2090425456832	0.1440956012977
0.2091071059242	0.1441344412993
0.2091790276238	0.14417397261
0.2092544179653	0.1445473176724
0.2093389685453	0.1446291578492
0.2094191494101	0.1446470596691
0.2095024708616	0.1447428143921
0.2095895307007	0.1448935413728
0.2096615811522	0.1449494796472
0.2097457726931	0.1449716275874
0.2098117748051	0.1449999628582
0.2098925747885	0.1450455106039
0.2099593804231	0.1452382506451
0.2100446963679	0.1452596042972
0.2101341701437	0.1453504914967
0.210222272281	0.145378666855
0.2103119113348	0.1455156619309
0.2103920826011	0.1458728101368
0.210484272838	0.1458752978251
0.2105620667737	0.1460287850309
0.2106399029436	0.1460775878235
0.2107120870815	0.1461552586403
0.2108005932851	0.1462766545215
0.210879868424	0.1463131574341
0.2109430944599	0.1464860371297
0.2110282765021	0.1466191250945
0.211122081811	0.1467062224273
0.2112134490107	0.146756460112
0.2112939207425	0.1468091741272
0.2113729942056	0.1468714819444
0.2114618876077	0.1469095066214
0.2115572381533	0.1469850518567
0.2116643011873	0.1475217153541
0.2117407282633	0.1480219841981
0.2118354326652	0.1480818932768
0.21191594764	0.1481351846114
0.2119976924477	0.1481371891909
0.2120751985074	0.148184398251
0.2121727352782	0.1484140211852
0.212268974631	0.1484183344171
0.2123363849372	0.1484500421243
0.2124183505317	0.1484528077817
0.2124928853185	0.1485271635109
0.2125707776498	0.1488761161099
0.2126515358661	0.1488887267195
0.2127355741818	0.1489778927145
0.2128118902921	0.1490466752757
0.2129120042702	0.1491780280481
0.2129915661779	0.1492481424536
0.2130626977608	0.149455227502
0.2131368711828	0.149554638961
0.2132134281081	0.1496560032792

0.2133013401427	0.1496846684775
0.213382981382	0.1498454256224
0.2134757226525	0.1499213938515
0.2135531828513	0.1502321091258
0.213656914822	0.1503710240053
0.2137433331437	0.150439117074
0.2138264163588	0.1504842325177
0.2139076517679	0.1505177560088
0.2139895183286	0.1505927335923
0.2140748423482	0.1506408997022
0.2141601386151	0.1509638504953
0.2142399433953	0.1510114988856
0.2143113991448	0.1510732089256
0.2143951814425	0.1512506975434
0.2144796255877	0.1513535052766
0.2145738833752	0.1514391674062
0.21467182092	0.1515094114621
0.2147570758962	0.1516372257366
0.2148570998477	0.1516807399799
0.2149466019393	0.1517635040391
0.2150314390477	0.15177127703
0.2151239274836	0.1521850672804
0.2152095677138	0.152283314066
0.2153058784019	0.1523455099182
0.2153896421546	0.1523601681589
0.2154702661681	0.1524356195405
0.2155562181243	0.1524943534815
0.2156545199838	0.1525068913804
0.215741009186	0.1525691919038
0.215820800538	0.1526854992025
0.2159092885114	0.1527278612972
0.2159917834167	0.1530214640278
0.2160849584932	0.1533764285844
0.2161829676057	0.1535860704225
0.216283656766	0.1537502066433
0.2163738402167	0.1537667113873
0.2164709465325	0.1538011998701
0.2165668801934	0.153852861826
0.2166421646194	0.1538877831476
0.2167466215073	0.1539036925055
0.2168245138041	0.1540301375287
0.2169052480653	0.154097774077
0.2169977447957	0.1541053001334
0.2170803942628	0.1542817652179
0.2171599365295	0.1543370037121
0.2172322371827	0.154435049787
0.2173207031076	0.1545419872565
0.2174189344413	0.1546393248843
0.2174974027293	0.1546840459519
0.2175945320056	0.1546862338008
0.2176801010263	0.1547657952932
0.2177635895237	0.1551096901084
0.217866887467	0.1551336387485
0.217963239109	0.1552892659199

0.2180470619224	0.1554463179609
0.2181276489423	0.1559934143945
0.2182027137821	0.156022406706
0.2182722459483	0.1560895346666
0.2183832288941	0.1562237855699
0.2184680469514	0.1563199025909
0.2185556680791	0.1563698667667
0.2186540585185	0.1563815094073
0.2187323438536	0.1564359159022
0.2188166433446	0.1564571693559
0.2189055775126	0.1565276596011
0.2189895538055	0.1569044918263
0.2190650088425	0.1569573862479
0.2191485791249	0.1570190322879
0.2192359055486	0.1571099652896
0.2193151113987	0.157168705907
0.2193891060625	0.1571944611334
0.2195031123941	0.1572316168161
0.2196042523843	0.1572962475904
0.219674461472	0.1573689956452
0.2197660175442	0.1575917241774
0.2198464793689	0.1579163255367
0.2199439294192	0.1580837060292
0.2200265568584	0.1583632298493
0.2201152591234	0.1583888879001
0.2202167837508	0.1583897494176
0.2203096923649	0.1586135375315
0.2203929223214	0.15866148007
0.2204882902097	0.1587398766782
0.220556152946	0.1589731102116
0.2206367787969	0.1592158512016
0.2207337791848	0.1592757716697
0.2208219342521	0.1593305530267
0.2209136466009	0.1593446180677
0.2209922355912	0.1594301744998
0.2210783689324	0.1594407230303
0.221146886067	0.1598345503759
0.2212211353341	0.1600101899562
0.2213196712239	0.1600399083624
0.2214010106453	0.1602699885741
0.2214982741251	0.1605426479225
0.2215752193622	0.1605950034126
0.2216528124584	0.1607528370007
0.2217439725985	0.1609488629062
0.221836924993	0.1610335412542
0.2219189476728	0.1610362299161
0.2220182136305	0.1611894957151
0.222114047645	0.161226266963
0.2221976436194	0.1612409249096
0.2222877599967	0.1612887915567
0.2223806675307	0.1613564745608
0.2224712633044	0.1613571994995
0.2225529902291	0.1615582602317
0.2226517969987	0.1615774021193

0.2227533133435	0.1617087478065
0.22283535544	0.1618031969581
0.2229378815973	0.1620073523287
0.2230331142711	0.1620236775444
0.2231253783723	0.1625137609824
0.2231992812236	0.1627073338493
0.2232722899657	0.1627117583626
0.2233643725642	0.1629263139206
0.2234531019247	0.1629937420972
0.223545709362	0.1630452814806
0.2236196916732	0.16312642524
0.2237083843441	0.1632506133267
0.2237962798961	0.163279009812
0.2238774916028	0.1634550015789
0.2239559750764	0.1637450246824
0.2240593188567	0.1639183509183
0.2241417343937	0.1639308637839
0.2242258469643	0.1642113099749
0.2243056955138	0.1642851784681
0.2243988517548	0.1643142830385
0.2244865673811	0.1646070538288
0.2245605014002	0.1646526896759
0.2246426598396	0.164682964097
0.224739564526	0.1649869193697
0.2248157163963	0.1650597579387
0.2249016148767	0.1651259576816
0.2249701337154	0.1652458669096
0.2250357103609	0.1653652932738
0.2251311616869	0.1654656081412
0.2252160101839	0.1655111081321
0.2253030826842	0.1655563170393
0.2253786799554	0.1656585726455
0.2254709355682	0.1657073635423
0.2255636050203	0.1657987804353
0.2256677601263	0.1659203901694
0.2257349747981	0.1659260528231
0.2258210475148	0.1659599943751
0.225914549141	0.1660588352513
0.2260055202188	0.1662144737744
0.2260816938563	0.1663732650691
0.226168055964	0.1665031064756
0.2262539453339	0.1666369960203
0.2263336513322	0.1666680257535
0.2264249903668	0.166690492323
0.2265016247336	0.1667284219746
0.2265954139554	0.1667625041794
0.2266718860221	0.1668393068435
0.2267485704986	0.1671671506072
0.2268336779377	0.1673588981939
0.2269201095798	0.1674694505274
0.2270050456888	0.167530237774
0.2270938108687	0.1675769673326
0.2271837067484	0.1677043965164
0.2272759180234	0.1677448267613

0.2273621334296	0.1677539196839
0.2274471883843	0.1678426650246
0.2275281368596	0.1678478849762
0.2276030315284	0.1678718894014
0.2276923530079	0.1679068648164
0.2277763259351	0.1680552804393
0.2278627741803	0.1682529968086
0.2279534191993	0.168549534538
0.2280443721325	0.1686888396515
0.2281323214195	0.1689079693474
0.2282174037376	0.1692603580729
0.2283031878042	0.1693560283496
0.2283754984094	0.1693743516659
0.2284668297028	0.1693923278978
0.228555756542	0.1694087806268
0.228627599717	0.1695138445505
0.2287088193207	0.1697855252279
0.2287797624436	0.170154784985
0.2288580359643	0.1702480352063
0.2289516089856	0.1703234918327
0.2290399739416	0.1703774365537
0.2291310861961	0.1705095051883
0.229233378651	0.1705136814483
0.2293260278976	0.1705564615017
0.2294207071081	0.1706012205275
0.2295349274672	0.171072246554
0.2296237501366	0.1711145092996
0.2297054127498	0.17121438561
0.2298090077215	0.1713840596167
0.2299001957732	0.1714553233295
0.2299942192696	0.1714971124137
0.2300978714722	0.1717199403864
0.2301864190185	0.1718253089284
0.2302661663186	0.1718888266914
0.2303541794377	0.1719253615129
0.2304479092881	0.1720523923735
0.2305407577326	0.1721993740612
0.2306366133958	0.1722917744867
0.2307146797367	0.1725032659122
0.230798225743	0.1729103919669
0.2308992678964	0.1732166321588
0.2309728605306	0.1733050954781
0.2310751223096	0.1733276560683
0.2311535351242	0.1733927322045
0.2312220106195	0.1738273102654
0.2313051177582	0.1738381749404
0.2313812994926	0.1738384923913
0.2314554418387	0.1738729222506
0.2315485995318	0.1739364552106
0.2316423387643	0.1739471036683
0.2317458930404	0.1739564340958
0.2318371541337	0.1740699826572
0.2319309843952	0.1741042730531
0.2320255082587	0.1741164751094

0.2321202454677	0.1742945979553
0.2322028986838	0.1743132031277
0.2323026973745	0.1743727034898
0.2323900983824	0.1744810465506
0.2324972968985	0.1745677201183
0.2325869497374	0.1748477611043
0.232688129719	0.1751637457839
0.2327766989368	0.1751842835873
0.2328453722342	0.1752050658698
0.2329273918339	0.1754899977687
0.2330192396981	0.175708322928
0.2330985690547	0.1759144426655
0.2331912829896	0.1760186418676
0.2332998098291	0.1760898290024
0.233391533084	0.1762245610971
0.2334609769651	0.1764185898985
0.2335508850271	0.1765598541654
0.2336154875401	0.1765953941402
0.2336977020159	0.1766756689952
0.233782505214	0.1767031903525
0.2338643812188	0.1767429281787
0.2339457066438	0.1767736008255
0.2340322188761	0.1767939490057
0.2341187954253	0.1768005220103
0.2342059696172	0.1768484308511
0.2342878460801	0.1769060328276
0.2343570042086	0.1769362398207
0.2344465140173	0.1769775905952
0.2345306254957	0.1770600650217
0.2346175717017	0.1770870557444
0.2346970379369	0.1772184507114
0.2347760553522	0.1775701257781
0.2348709243957	0.1776539550539
0.2349547932308	0.1777676719658
0.2350413919223	0.1779877219971
0.235125424305	0.1780263187979
0.2352121776031	0.1780463291126
0.2353059904733	0.1781356005498
0.2353947855297	0.1781436110694
0.2354814509259	0.1783622246425
0.2355768152119	0.1783870983856
0.2356622369525	0.1784705125964
0.2357394224398	0.178548147818
0.2358232785102	0.1785505232521
0.2359276873848	0.1788806775835
0.2360169566935	0.1793296676927
0.2360793113166	0.1793693766201
0.2361719810361	0.1793744752655
0.236243153227	0.1795906608798
0.2363445075169	0.1796811302551
0.2364355138792	0.1797393936536
0.2365196647572	0.1798443741679
0.236607494631	0.1802903486613
0.2367014810579	0.180414784009

0.23677974868	0.1804261994015
0.236864547943	0.1804739352511
0.2369520144392	0.1805835781512
0.2370284513786	0.1806047138113
0.2371048389536	0.1809139661959
0.2371891089404	0.1810109140798
0.2372672537596	0.1810625119043
0.2373619943015	0.1811061220556
0.2374412726825	0.18123451591
0.2375288626652	0.1813659794511
0.2376133307662	0.1814500471802
0.2376939708039	0.1815753961867
0.2377739938972	0.1815796439385
0.2378467797782	0.1816818122233
0.2379464507342	0.1817828482907
0.2380352487316	0.1821264325055
0.2381224793928	0.1821447351534
0.2382112694628	0.1821586367383
0.2383020047092	0.1822443601607
0.2384017751366	0.182273164367
0.2384927301172	0.1824715700785
0.2385794237243	0.1825581955334
0.2386620046848	0.1825670463138
0.2387617708265	0.1827288888055
0.2388506957889	0.1828106925448
0.2389397692322	0.1828490538599
0.2390262927722	0.1828692812482
0.2391006251192	0.1829881166317
0.2391839294549	0.1833007368142
0.239263596404	0.1836533425047
0.2393252339271	0.1838057092827
0.2394101402269	0.1838364812435
0.2394914578409	0.1838552730238
0.2395672783279	0.1839138915128
0.239656888624	0.1840393851286
0.2397518769677	0.1845023004472
0.2398353776313	0.1846226615527
0.2399078988466	0.1846306951479
0.2399963606913	0.1846636912779
0.2400709488618	0.184809556274
0.2401592083587	0.1849051388515
0.2402504629497	0.1850198075379
0.2403258786258	0.1850805417622
0.2403969579294	0.1851190807039
0.2404796122491	0.1852308672117
0.2405828507886	0.1854850485595
0.2406637160735	0.1856870476156
0.2407647580503	0.1857812196379
0.2408497705869	0.1859214761831
0.2409395877286	0.186011061742
0.2410384492689	0.186065362103
0.2411442861013	0.1864400845955
0.241228922766	0.1867832072349
0.2413103982198	0.18697674703

0.2413899199877	0.1869783794972
0.241473467802	0.1870442569084
0.2415547279902	0.1871204980929
0.2416442828299	0.1871662708228
0.2417265249035	0.1873779568606
0.2418089752819	0.1875753132998
0.2418807795817	0.1876536341518
0.2419803975273	0.1877014716482
0.242063424116	0.1877818418093
0.24214985957	0.1879045528024
0.242247239639	0.1880416923423
0.2423428998584	0.1880698680838
0.2424034360351	0.1883277078841
0.2424891866223	0.188426699871
0.242584303877	0.1884327522007
0.2426688060015	0.1884659720561
0.2427522246717	0.1886448106428
0.2428489061774	0.1889060513089
0.2429298146607	0.1891285798965
0.2430002844097	0.1892843390653
0.2430785129692	0.1895765821828
0.2431549515553	0.1895767567317
0.2432358926254	0.1895883789587
0.2433183712087	0.1899944942289
0.2433904278455	0.1900912395873
0.2434740694389	0.1901046377191
0.243562596144	0.1901553167201
0.2436456631928	0.1904600146922
0.24372818324	0.1905391947445
0.2438063682029	0.1905647803745
0.2438957351596	0.1906174642588
0.2439986172798	0.1908342631991
0.2440763625836	0.1908621156443
0.244181978369	0.1908845600653
0.2442554450313	0.1909357277291
0.2443503716558	0.1910643004839
0.2444437621486	0.1910949253123
0.2445381808666	0.1911505452065
0.244621506586	0.1913306034827
0.244728164184	0.1914156173994
0.244824464697	0.1917077958679
0.2449150382897	0.1917329028863
0.2450137779851	0.1919579102805
0.2450992474586	0.1921807829274
0.24518658621	0.1922062120575
0.2452643921995	0.192235831122
0.245354490954	0.1922370344576
0.2454384978286	0.1922912159235
0.2455214016284	0.1924173364768
0.2456202905481	0.1926245978915
0.2457038768447	0.1927182686628
0.2457904909615	0.1927293564236
0.2458871537979	0.1929490281192
0.2459616181711	0.1929692780814

0.2460380073949	0.1930302254492
0.246117563643	0.1931251571023
0.2462033587941	0.193194480316
0.2462996213792	0.1932042081017
0.2463655089633	0.1932145304808
0.2464443610081	0.1932267100836
0.2465213920448	0.1933250671605
0.2465973775755	0.1933860059246
0.246673966525	0.194049041221
0.2467695239935	0.1946555064241
0.2468622677309	0.194702128755
0.2469396195241	0.19479797108
0.2470236942565	0.1949723831545
0.2471006261814	0.1950033086577
0.2471964136959	0.1950644831301
0.2472778660786	0.1951552135785
0.2473823918782	0.1952220304316
0.2474617004617	0.1953933286923
0.2475349006499	0.1956075752189
0.2476270955032	0.1956350863781
0.2476932592541	0.1956365263339
0.2477829266017	0.1956582281696
0.2478747316433	0.1959039111389
0.247993656075	0.1959763702215
0.2480556368869	0.1960365954041
0.2481385610114	0.1960882140101
0.2482282557437	0.1961240983107
0.2483141413793	0.1961351432658
0.2484046107914	0.1962233422638
0.2484748694566	0.1962734603792
0.2485702906832	0.1964075692291
0.2486584003256	0.1964152922196
0.2487409623822	0.1964397584947
0.2488228975069	0.196450074304
0.2489000326951	0.1965115905263
0.2489930681214	0.1965117923758
0.2490969803373	0.196585707033
0.2491783748589	0.197212995794
0.249262621483	0.1973350664913
0.2493332075398	0.1973909463688
0.249401087657	0.1977093660644
0.2494727273668	0.1978034649763
0.2495442607242	0.1978650997557
0.2496310269409	0.1979147941117
0.2497181968436	0.1980599372907
0.2498158972689	0.1988324809113
0.2499010611167	0.1988938890971
0.2499762165368	0.1991210108387
0.2500548632029	0.1991688838332
0.2501533785973	0.1994249726383
0.2502332899409	0.1995206547276
0.2503134659585	0.199971146109
0.2503947653024	0.2002630941229
0.2504817461194	0.2003246981004

0.2505645626092	0.2003485834926
0.2506567621267	0.200661758198
0.2507615847061	0.2007020147484
0.2508390486758	0.200731722643
0.2509248981831	0.2007475299819
0.2510214278281	0.2007844018807
0.2511103266409	0.200921699771
0.2511897081359	0.200946221565
0.2512713934593	0.2010483230061
0.2513610278236	0.2013357952288
0.2514660337056	0.2014047166033
0.2515605515566	0.201417142685
0.2516530550669	0.2014446631598
0.251735241887	0.2016262610495
0.2518369934608	0.2016503026571
0.2519278234186	0.201691432559
0.2520148599379	0.2017576472947
0.2521039850611	0.2018475472974
0.2521868908307	0.2018782337822
0.2522549013133	0.2018913098606
0.2523466021529	0.2019633670479
0.2524244809473	0.2022952313605
0.252525582841	0.2023084742166
0.2526094008096	0.2024136194528
0.2526798103184	0.2024885575445
0.2527721812027	0.2025312914726
0.2528585700437	0.2025630468838
0.2529387818654	0.2027416556669
0.2530083188549	0.2028243374166
0.2530824855729	0.2028315967906
0.2531710130655	0.2028576340369
0.2532570061403	0.2028807912157
0.253352787925	0.2029074015902
0.2534242682819	0.2030447163903
0.2535085429233	0.2031679157008
0.2536204158681	0.2032534539015
0.2536934233135	0.2033615221237
0.2537665480214	0.203439941491
0.2538626957741	0.2034724610782
0.2539453412537	0.2036107534272
0.2540389359522	0.2036696597269
0.2541199186422	0.2038427890211
0.2542048993326	0.2040808695097
0.254283333128	0.2040999935064
0.2543688929297	0.2042941857224
0.2544441078358	0.2043909937617
0.2545244052683	0.2047433918734
0.2546247505614	0.2050142860812
0.254714906236	0.2051503968285
0.2548232483573	0.205279028342
0.2549107943634	0.2052935723563
0.2549926378396	0.2053712108644
0.2550889579242	0.2054560198785
0.255177256828	0.2054881225858

0.2552592933948	0.2055686667596
0.2553422125494	0.2058981389751
0.2554327736869	0.2059045430528
0.2555079249253	0.2059258630637
0.2556108040518	0.205927406342
0.2557065756276	0.2059901964717
0.2557795014093	0.2060789468588
0.2558528730412	0.206141020794
0.2559316337772	0.2067086441263
0.2560118790796	0.2068888840946
0.2560994004551	0.2069564276995
0.2561889276414	0.2070008673262
0.2562693911036	0.2071415919816
0.25634287098	0.2073618612364
0.2564309917697	0.2073807365114
0.2565172051296	0.2074806045484
0.2566061188299	0.2076056753357
0.2566953703889	0.2077248847253
0.2567777977301	0.2077506249071
0.2568610089247	0.2077558916486
0.256932157766	0.2077795928525
0.2570172600076	0.2078203415909
0.2571171826855	0.2078751575211
0.2571989264656	0.2080091122257
0.2572880963544	0.2081021290543
0.2573790773264	0.2081226555244
0.2574580863887	0.2081460715417
0.2575453370697	0.2082352225614
0.2576188094144	0.2082668894374
0.2576960505126	0.20862792895
0.2577782266596	0.2086650571585
0.2578610906352	0.2086730208607
0.2579401693371	0.2089253416294
0.2580233024747	0.2091670900529
0.2581073581201	0.2092122148762
0.258191970462	0.2092998019255
0.2582695536121	0.2093072974175
0.2583473923001	0.2093202875242
0.25844240372	0.2093706301667
0.258521370668	0.2093938263305
0.2585891043253	0.2094351230211
0.2586877625069	0.2095390750131
0.2587802146091	0.2095702245736
0.2588719085825	0.2097151443734
0.2589694767547	0.2097393871469
0.2590513696473	0.2097909020616
0.2591409799787	0.210128019053
0.2592139961594	0.2104923052119
0.2592875310227	0.2105285836924
0.2593755450326	0.2105714571085
0.259468705536	0.2106210866972
0.2595428104935	0.2106242737483
0.2596138999029	0.2106481563724
0.2596935368752	0.2106543101548

0.2597726354972	0.2107501878959
0.259849295257	0.2109287839721
0.2599288728656	0.2110158648981
0.2600135892663	0.2110517631006
0.2600900824723	0.2111249844338
0.2601895008337	0.2112913349406
0.2602699325639	0.211595795142
0.2603557707949	0.2116545269361
0.2604610558433	0.2118239822412
0.2605443894718	0.2120546819431
0.2606530251458	0.2122407125972
0.260747713008	0.2122545171985
0.2608450385144	0.2123740690039
0.260959083664	0.2125746268356
0.2610460639902	0.2125770826867
0.2611311346481	0.2126125722086
0.2612283175918	0.212735159513
0.2612987221967	0.2127608099234
0.2613895337827	0.2128827979836
0.2614649732029	0.2131643512806
0.2615548399815	0.2133005478504
0.2616414794647	0.2133508444518
0.2617103795488	0.213504300343
0.2617948065556	0.2135248302639
0.2618691156988	0.2135625116323
0.2619422163478	0.2140187365922
0.2620260838116	0.2140977852018
0.2620962961927	0.2141273122368
0.2621733427131	0.2147014638117
0.2622717424162	0.2147750185064
0.2623441741226	0.2151034458075
0.2624220318711	0.2151173650542
0.262504491332	0.2151432578866
0.2625911971566	0.2152522044096
0.2626695430945	0.2152574448647
0.2627572927742	0.2153171109282
0.2628327882617	0.2153406635453
0.2629081620436	0.2153564281676
0.2629889087236	0.2153705104595
0.2630583116349	0.2157902330388
0.263140851652	0.2160443897548
0.2632370300751	0.2160540839609
0.2633236869735	0.2161701032767
0.2634200723067	0.216240651551
0.2634922500868	0.2164756420131
0.2635718499857	0.216478596156
0.2636464393024	0.2165843195175
0.2637353010621	0.2166975173851
0.2638346202806	0.2167076714047
0.2639139574349	0.2168153893468
0.2640159496615	0.2170641867223
0.264111255432	0.2170870122405
0.26419995766	0.2171659900661
0.2642977783216	0.2172923877477

0.2643773479108	0.2173000824407
0.2644675457846	0.2173261190071
0.2645484947712	0.2173365155106
0.2646400010204	0.2174637375859
0.2647307989572	0.217563509226
0.264838750256	0.2177192905358
0.2649245252349	0.2178848369809
0.265014762022	0.2179858937162
0.2650926964169	0.2179890691431
0.2651874650634	0.218337700921
0.2652637770199	0.2185072531447
0.2653439021865	0.2185795288193
0.2654298970488	0.2186841998112
0.2655072489197	0.2187545541675
0.2656013362776	0.2188893381068
0.2657009348908	0.2190161238036
0.2657643884728	0.2190618945798
0.265842230007	0.2190878200176
0.2659270150477	0.2192262668659
0.2659877011751	0.2192456102606
0.2660809095283	0.2192654649023
0.2661828385092	0.2200967947514
0.2662727167571	0.2200990888412
0.266355890171	0.220119813102
0.2664311931387	0.2203041875565
0.2665211678218	0.2203508741969
0.2665906644547	0.2203927089065
0.2666973113149	0.2204425837529
0.2667725151475	0.2204447411813
0.2668570962164	0.2206235721492
0.2669352403644	0.2206940133985
0.2670162901022	0.2207516711508
0.2670937570223	0.2207546388119
0.2671857667824	0.2207826035629
0.2672666954993	0.2208422697196
0.2673519232824	0.220850665131
0.2674435879311	0.220889879795
0.2675164827538	0.2209238512694
0.2675919020108	0.2211786732494
0.2676807338815	0.2211865262826
0.2677697733406	0.221243509246
0.267847844423	0.2212607312539
0.2679655883963	0.2214699895397
0.2680433495128	0.2214849819545
0.268116210677	0.2214904188532
0.2682112756226	0.2215011561724
0.2682838428022	0.2215083685237
0.2683593460595	0.2218262082939
0.2684551136824	0.2220291768643
0.2685384433474	0.2226019888679
0.2686163833442	0.2227425003174
0.2687095036548	0.2228253296561
0.2687771992138	0.2229972147279
0.2688638441752	0.2230393845733

0.2689573849738	0.2231167797083
0.2690296669037	0.2231382002082
0.2691208102129	0.2232008720214
0.2691786273545	0.2232756095207
0.2692555228936	0.2233257727911
0.2693344512361	0.2233861427973
0.2694383568531	0.2236562400966
0.269538781915	0.2238645700242
0.2696444355095	0.2241687883642
0.269744013559	0.2242838569202
0.2698193847966	0.2243871298757
0.2698971606693	0.2244840717351
0.2699778635693	0.2244905078552
0.270085712135	0.22456826688
0.2701740748902	0.2245833578015
0.2702511078053	0.2246000431513
0.2703434532812	0.2246117849808
0.2704114515611	0.2246515430308
0.2704905055481	0.2247244640668
0.270578588559	0.2247645242763
0.2706484223918	0.2249573029951
0.2707319961441	0.225106495638
0.2708150353422	0.2251192835863
0.2709127166582	0.2253075630004
0.2709940864001	0.2253698119813
0.2710763778052	0.2254097446669
0.2711626361241	0.2254773201282
0.2712399484934	0.2255473567972
0.2713208100303	0.225559161374
0.2714106523601	0.2255628386115
0.2714900002511	0.2256155001524
0.2715763859435	0.2256729175368
0.2716525540253	0.2257632710852
0.2717537765169	0.2258330426159
0.2718482939349	0.2258527628512
0.2719439678137	0.2258807544845
0.2720240253156	0.2259264102884
0.2721159768103	0.2260311914235
0.2722065572426	0.2261990920356
0.2722997863058	0.2262886568981
0.2723708281047	0.2262972867268
0.2724404685325	0.2263860429884
0.2725275074556	0.2265314253202
0.2726208897802	0.2265735430226
0.2727322150131	0.2266024352934
0.2728083373686	0.226729361567
0.2728982805171	0.226800442387
0.2729771277836	0.226931760566
0.2730631318463	0.2269719883042
0.2731531439494	0.2269859650801
0.2732290710361	0.2270055012608
0.2733278434928	0.2274018645756
0.2734049745308	0.2274638339893
0.2734974961642	0.2275120830121

0.27359155401	0.2275883820656
0.2736773103621	0.227662725331
0.2737605549397	0.2278817980995
0.2738514487516	0.2281800173645
0.2739339465068	0.2284279834954
0.2739966611768	0.2284646700157
0.274100960855	0.2286128335664
0.2741923499752	0.2286323035172
0.2742633570668	0.2287658000551
0.2743600596417	0.2288275823758
0.2744378885982	0.2289522169602
0.2745260104988	0.228973186347
0.2746133039976	0.2290813406168
0.2747078041935	0.2290962497637
0.2747956251755	0.2291457272482
0.2748784477099	0.2291792885622
0.2749803508032	0.2295235742991
0.2750595949732	0.2295409272285
0.2751285541032	0.2300914135497
0.275211405573	0.2301586651158
0.2752873272465	0.2301769459829
0.2753808102577	0.2302882407231
0.2754616665733	0.2304697810111
0.2755496802114	0.2304980284035
0.2756387925804	0.2305584954161
0.2757220230855	0.2306248023691
0.2758125748792	0.2306349538194
0.2759005834065	0.2307536246763
0.2759810327809	0.2308491937897
0.2760746064549	0.2311277123095
0.2761598113148	0.2311417624526
0.2762361227545	0.231308295002
0.2763175219586	0.2313588644741
0.2764018695563	0.2313957352456
0.2764797573676	0.2315673216257
0.2765709678657	0.2316959102143
0.2766505395399	0.231771833866
0.2767335303391	0.2317919330473
0.2768274850705	0.2318556829254
0.2769051746571	0.2319755374248
0.2769782915496	0.2320470592658
0.2770685171958	0.2323329977928
0.2771490775211	0.2323602777754
0.2772357916137	0.2326134146369
0.2773120685996	0.2330079768602
0.2773969289279	0.2332763582869
0.2774769510595	0.233304576723
0.2775711008052	0.2333598525798
0.2776834467503	0.233447179927
0.2777693155828	0.2336960564295
0.277868255371	0.2337361015884
0.2779362423141	0.2337417016498
0.2780084923924	0.233961422595
0.2780808403839	0.2340419790395

0.2781762180793	0.2343679443484
0.2782671474742	0.2345334709411
0.2783662312272	0.2346941592982
0.2784475795333	0.2348080596371
0.2785483656543	0.2349490516573
0.2786649791641	0.2351036787817
0.2787509098348	0.2353022685709
0.278853338233	0.2353119271022
0.2789332236589	0.2354022208882
0.2790103975061	0.2354105561395
0.2790922147255	0.2355863237814
0.279190881561	0.2356155312701
0.2792735880243	0.2356612173803
0.2793543628443	0.2357564478554
0.2794301279004	0.2358571700868
0.2795174250548	0.2358689416469
0.279604586464	0.2359059726574
0.2796914577621	0.2361311193903
0.279774525693	0.2361336669375
0.2798786094231	0.2361566975779
0.2799785706483	0.23621381882
0.2800664144972	0.2363070371789
0.2801453188974	0.2363081717413
0.2802354275129	0.2363546386842
0.2803113085232	0.2364969869198
0.2804041233396	0.2365457846538
0.2804879271751	0.2365809983999
0.2805927394704	0.2365909932789
0.2806730580729	0.2366547021019
0.2807461358304	0.2367281653596
0.2808089804351	0.2367899959896
0.2808833129752	0.2369859154116
0.2809686935201	0.2370309064093
0.2810469084297	0.2371154614555
0.2811350968105	0.2371534870994
0.281212715666	0.2372054805614
0.2812831102527	0.2372267438595
0.2813705197264	0.2373405153989
0.2814469050344	0.2374435284222
0.2815498406658	0.2374989637842
0.2816523054604	0.2375101446674
0.2817404622101	0.2376306860082
0.2818150918591	0.2376713315873
0.2818914865189	0.2377103527678
0.2819724971874	0.2377578910352
0.2820517961235	0.2378718944284
0.2821221953728	0.2379203843671
0.2821974266452	0.2380246654586
0.2822927492821	0.2380842479142
0.2823663251249	0.2381039992389
0.2824363230686	0.2382670315817
0.2825137749071	0.2385429621103
0.2826040908787	0.2385780990209
0.2826760277711	0.2386139084437

0.2827679026637	0.238827569209
0.2828722766596	0.2388683858777
0.2829598819761	0.239070423382
0.2830475379416	0.2391438771806
0.2831248392619	0.239167628577
0.283212213275	0.2391976496402
0.2832856485592	0.2392112076498
0.2833707199939	0.2392537989263
0.2834576216678	0.2392583141392
0.2835381649052	0.2393048744992
0.2836405596752	0.2393190078227
0.2837287249619	0.2393371427014
0.2838208529262	0.2393575740892
0.283909317967	0.2395289285285
0.2839866094274	0.2396238273764
0.2840702347738	0.2396708597152
0.2841655809077	0.2398285085028
0.2842545480347	0.2398307972851
0.2843424953266	0.2398388847332
0.2844168042908	0.239965452821
0.2845058022071	0.2399861213945
0.2845856799922	0.240082435054
0.2846751236856	0.2401935347366
0.2847518728384	0.2402496392503
0.2848481380008	0.2402928211017
0.2849387919859	0.2403056048949
0.2850118503245	0.240460727205
0.2851011975554	0.2404641558609
0.28518315325	0.2405069637694
0.2852793138155	0.2405808487347
0.2853722436959	0.2407827487919
0.2854440039942	0.2409183597154
0.2855294629209	0.2411325306481
0.2855976919427	0.2414570697073
0.2856717847071	0.2419097455841
0.2857536883724	0.2419468023416
0.2858499149816	0.2421279185569
0.2859426805558	0.2422233609729
0.2860367080955	0.2422601429238
0.2861212810298	0.2423204695288
0.2862067355449	0.2423813955055
0.2862866635463	0.242487366915
0.286368388778	0.2425092655133
0.2864687812171	0.2427148283733
0.2865546811857	0.2427910808594
0.2866356529586	0.2428249047048
0.286726065699	0.2429095510564
0.2868022093738	0.2430548016882
0.2868868997156	0.2430581848356
0.2869632784849	0.2430894264415
0.2870417581581	0.2431687076064
0.2871245589344	0.2432842864358
0.2872020433564	0.2433473512576
0.2872796820927	0.2434870466401

0.2873701831925	0.2437361019783
0.2874662610731	0.243832899249
0.2875598273513	0.2439640633065
0.2876447043604	0.244065336066
0.28772128384	0.2442421404237
0.2878060634388	0.2442570672517
0.2878916493287	0.2447713047872
0.2879655522181	0.2448508973389
0.2880439692659	0.2448884326277
0.2881293402894	0.245355389459
0.288232448737	0.2455263111554
0.2883273658876	0.2456720425261
0.2884141528177	0.2457336630812
0.2884954613008	0.2458462729503
0.2885898988822	0.2458506648217
0.2886781338828	0.2458846342839
0.2887717590173	0.2459225865009
0.2888604275112	0.2459431624882
0.2889363637091	0.245966050113
0.289020168192	0.2463441331122
0.2891144012091	0.2464213810379
0.2891912833123	0.2465457017539
0.2892706945644	0.2466106955717
0.2893954475052	0.2466820445326
0.2894944871837	0.2467036995406
0.2895756277433	0.2467588619686
0.2896643588797	0.2469158832161
0.2897403770298	0.2469467123042
0.2898199208035	0.2469657679194
0.289900484644	0.2469820349179
0.2899883220769	0.2469967992325
0.290075787306	0.2470600762593
0.2901796355425	0.2472653486838
0.2902618550709	0.2475078692117
0.2903380916542	0.2475857717748
0.2904292770636	0.2476147272415
0.290518123423	0.2476333593378
0.2905931925839	0.247661536205
0.2906742501731	0.2476901340351
0.2907549553495	0.2477432558818
0.2908399641217	0.24793672234
0.2909217289502	0.2479654135061
0.2910006590912	0.2479946117817
0.2910940916858	0.2480160132286
0.2911763371125	0.2484083131861
0.2912695349179	0.2484289128083
0.2913587453231	0.2484905021708
0.2914443218159	0.2487191733407
0.2915117546833	0.2487795637136
0.2915974601411	0.248839216068
0.2916844516681	0.2491811098968
0.2917716203045	0.2491979752705
0.2918606527303	0.2495343490304
0.2919601801256	0.2495562524355

0.2920573425603	0.2495902359865
0.2921488991015	0.2497106419369
0.2922312401014	0.2497117454208
0.2923213035575	0.2497274873838
0.292411830047	0.2500982587622
0.2924986539209	0.2501013994374
0.2925869891074	0.250241841329
0.2926900454507	0.2503071480415
0.2927652526507	0.2503579461819
0.2928503694582	0.250754194987
0.2929274975327	0.250833520391
0.2930314296697	0.2510650338912
0.2931036136168	0.2511477364681
0.2931904116845	0.2512468069922
0.2932754653251	0.251274366096
0.2933495416854	0.2512992262694
0.2934435466413	0.2515483370993
0.2935393850193	0.2515964922481
0.2936273875211	0.2517449568703
0.2937234883629	0.2518015867675
0.2938008803599	0.2520933777377
0.2938884042329	0.2521612628793
0.2939715000258	0.2522711073393
0.294065355601	0.2525871550711
0.2941445846861	0.2526529278296
0.2942365079831	0.2526794279403
0.2943052064735	0.2527060505701
0.2943871539475	0.2528755846443
0.2944640362312	0.2531106640278
0.294542016952	0.2531506146229
0.2946168298213	0.2534562598179
0.294688055202	0.2535362120627
0.2947790884389	0.253539067307
0.2948649222027	0.2537210636801
0.2949343158267	0.2539191931854
0.2950157906598	0.2540563033761
0.2950954008766	0.2540989914997
0.2951811252253	0.2541959885427
0.2952644117217	0.2541970189134
0.2953296847279	0.2545048447497
0.2954159241874	0.2546339466516
0.2955192563896	0.2547497715279
0.2956004320044	0.2547696785411
0.2956844646435	0.2548489205835
0.295778463208	0.2549409086092
0.295869134566	0.2550135823309
0.2959523416159	0.2551167350006
0.2960255247954	0.2552022173492
0.2961027072543	0.2554281580512
0.2961960644038	0.2555116468732
0.2962865400305	0.2555269576809
0.2963576276735	0.2555272087282
0.2964347035353	0.2556961420397
0.2965179467203	0.2557024272521

0.296586676995	0.2557552914932
0.2966858413446	0.2557637229786
0.2967866416426	0.255910467012
0.2968914949414	0.2562785761231
0.2969780638896	0.2564915792972
0.2970511043459	0.2565958169897
0.2971511347247	0.2566357419635
0.2972248804091	0.2567373323884
0.2973165760253	0.2567492888096
0.2974073643681	0.2567646959251
0.2975037762773	0.2568986820099
0.2975895563188	0.2570126610345
0.2976675611026	0.257028356957
0.2977498593902	0.257046092738
0.297845706154	0.2571638340164
0.2979274435112	0.2572267580936
0.2980035765192	0.2572666631848
0.2980845968608	0.2572942227548
0.2981768156306	0.2573314412896
0.2982715788671	0.2576131720456
0.2983733054207	0.2580857551714
0.298462323551	0.2585058244713
0.298541456343	0.2585177173097
0.2986478018534	0.258672199207
0.29872670693	0.2587820577505
0.2988119159375	0.2587923233288
0.2988911959186	0.2589174498022
0.2989755649531	0.2591052363404
0.2990711011577	0.2591540127434
0.2991613357696	0.2592635747448
0.2992600696213	0.2594656143236
0.2993461055463	0.2595296794788
0.299452997084	0.2595520026828
0.2995451671403	0.2595576935474
0.2996356662849	0.2598020272781
0.299731489623	0.2599703650863
0.2998270134932	0.2599933324997
0.2999202744756	0.2601168414383
0.3000003987587	0.260378639251
0.3000916741932	0.2604248145908
0.3001735299042	0.2606240354088
0.3002589928744	0.2606751194406
0.3003364456697	0.2607973490705
0.3004258924248	0.2608314531993
0.3005069399788	0.2608760657082
0.3005818676456	0.2610822215967
0.3006618008986	0.2611379020752
0.3007566197719	0.2612049491814
0.3008440989759	0.2612882350229
0.3009326522489	0.2612953281643
0.3010376713544	0.2614127113038
0.3011170837135	0.2614295667102
0.301219225623	0.2614868631707
0.3012891514986	0.26156738218

0.3013851232177	0.2616918557529
0.3014713589103	0.2617074247227
0.3015601311685	0.2617127013317
0.3016499756656	0.2618178391686
0.3017506835325	0.2620269460752
0.3018313488033	0.2620639050232
0.3019224309839	0.2621794862196
0.3020050427433	0.2623662644507
0.3020816876675	0.2625502629116
0.3021652369523	0.2625643623419
0.3022458206585	0.2626260220385
0.3023356434427	0.2626654681791
0.3024300921803	0.2627300294799
0.3025253026935	0.2628494249014
0.3026009585399	0.2628737217393
0.3026875949753	0.2629042196727
0.3027751009232	0.2629880519969
0.3028625717	0.2631201163201
0.3029540550272	0.2632151281059
0.3030422758672	0.2637132794609
0.3031269003682	0.2637981580391
0.3032177784367	0.2638490329128
0.3033137531941	0.2638564329865
0.3034053644741	0.2638786602303
0.3035032998946	0.2639008052949
0.3035885959975	0.2639755218667
0.3036675464673	0.2643650603498
0.303750552058	0.2644389656924
0.3038331899814	0.2644978571665
0.303920125292	0.2646777312815
0.3040095917641	0.2648260756303
0.3040938611864	0.2648388739153
0.304189257671	0.2648714085755
0.3042821223691	0.2648793681495
0.3043641210949	0.2649257396288
0.3044605608682	0.2649525142348
0.3045625575382	0.26527973699
0.3046472311303	0.2654225382765
0.30472289139	0.2655705264765
0.3048060186589	0.265599480329
0.3048921894414	0.2656046283209
0.3050014214074	0.2656199639246
0.3050968757423	0.2656458462788
0.3051749591335	0.266228191657
0.305266039956	0.2662745820193
0.3053743591777	0.2662974098948
0.3054518331084	0.2663956985311
0.3055304209332	0.2664507171689
0.305616729956	0.2664720910627
0.3056998093469	0.2665028089373
0.3057862502399	0.2665567016258
0.3058733651982	0.2665894242874
0.3059556816331	0.2667979044087
0.3060445822014	0.2668230784185

0.3061460704737	0.2668344366746
0.3062227039589	0.2668497729161
0.3063129243567	0.2668534174501
0.3063930810723	0.266965828907
0.3064878187762	0.2671223030691
0.3065687797923	0.2672182141768
0.3066519149711	0.2672198485085
0.3067453932137	0.2676799406721
0.3068371530496	0.2677263034261
0.3069224221855	0.2678533365904
0.3070059597321	0.2679837206961
0.3071079040967	0.2680362363176
0.3072185997267	0.2682060348863
0.3073053733265	0.26821388587
0.3073849604862	0.2683597654392
0.3074846816188	0.2684634376379
0.3075750394523	0.2685351334876
0.3076717470767	0.2685382299609
0.3077563007993	0.2686112721726
0.3078541316449	0.2686544910327
0.3079443763472	0.2686794408828
0.3080206127681	0.2689500937945
0.3080921392062	0.2689624815602
0.3081844829159	0.2694396427144
0.3082753430503	0.2694636400771
0.3083599089033	0.2694943509307
0.3084569477509	0.2698644305245
0.3085442558989	0.2700070312562
0.3086517627237	0.2700558216712
0.3087507649715	0.2701524623979
0.3088458756414	0.2701585377825
0.3089329047351	0.2703070651631
0.3090216416242	0.2703404149415
0.3091020257699	0.2706386063856
0.3091750489563	0.2707826860736
0.3092522443841	0.2708694962621
0.3093435825259	0.2713480804863
0.3094308899887	0.2714395907
0.3095277237063	0.2715852678913
0.3096156260623	0.2717816097541
0.3096879669811	0.2717914437899
0.3097708362304	0.2718749151682
0.3098597195261	0.2723148487877
0.3099426774833	0.2723855771602
0.3100322715821	0.2724082129355
0.3101125357267	0.2727345091531
0.3101969587551	0.2727870967643
0.3102831073449	0.2728637306201
0.3103567497713	0.2728667057313
0.3104286764641	0.2728864066388
0.3105073410337	0.2729111749977
0.3105822220205	0.2730097887212
0.3106614445832	0.2733123825002
0.3107602171743	0.273597601708

0.3108460097703	0.2736547806385
0.3109275073657	0.2737265668462
0.3110250216581	0.2737360711075
0.3110971750947	0.2741036454749
0.3111833770296	0.2741239429602
0.3112703165764	0.2742276529616
0.3113437999589	0.2742516806927
0.311436087696	0.2744148624146
0.311532576133	0.2744205215565
0.3116200707522	0.274908240638
0.3116921298865	0.2750601632728
0.311786868023	0.2751845887913
0.3118657178023	0.2753901227376
0.3119623234936	0.2753945465308
0.312045925792	0.2754231677465
0.3121426791501	0.2754435745211
0.3122423155905	0.275480887374
0.3123210505587	0.2755733540039
0.3124189501936	0.2757096729996
0.3124918674065	0.2759205298895
0.312582981648	0.2759356524267
0.3126667907761	0.2761216041833
0.3127660914494	0.2762242489837
0.3128552924804	0.2762272267084
0.312941148423	0.2763544862714
0.3130401121868	0.2765230327164
0.3131407769516	0.2766887069864
0.3132262946606	0.2768456941961
0.3133157344093	0.2770242516939
0.313405909244	0.2771652762454
0.3135048143069	0.2772409193673
0.3135753044274	0.277303699575
0.3136556952809	0.2776751760463
0.3137569574542	0.277785664703
0.3138547289243	0.2778195291335
0.3139533826299	0.277828761754
0.3140332750227	0.2778963487136
0.3141251347147	0.2780530113627
0.3142212503183	0.2780831589119
0.3143087944516	0.278121600205
0.3144037999284	0.2782074775995
0.3145021811248	0.2785718491837
0.3145895334263	0.2786949160168
0.3146751527759	0.2787264568554
0.3147558725989	0.278783791476
0.3148489880529	0.2788401939246
0.3149311117992	0.2792257691847
0.315003753319	0.2792574728567
0.3150957034316	0.2792706558201
0.3151745580682	0.2793144833545
0.3152723751318	0.2794534975434
0.3153545375332	0.2796984968011
0.3154226506832	0.2797520900476
0.3154910474982	0.2800062072465

0.3155611667944	0.2800425527541
0.3156545153498	0.2800439774462
0.3157425033729	0.2801987677869
0.3158297202667	0.2803063464979
0.3159316844808	0.2803198249297
0.3160070220544	0.2803349220964
0.316106265053	0.2803505510042
0.3161930914009	0.2805663369486
0.3162793017052	0.280708657216
0.3163624714971	0.2807154146511
0.3164683981962	0.2810085450386
0.3165502630263	0.2810596877036
0.3166491947997	0.281176943251
0.3167132782317	0.2812785077466
0.3167919665512	0.2813309692743
0.3168810880802	0.2813446254467
0.316978271263	0.2815498753235
0.3170690857066	0.2816037470211
0.3171502234285	0.2817363918559
0.3172328806794	0.2817537160982
0.3173155193579	0.2817625390738
0.3174039847873	0.2819277553883
0.3174901944764	0.2819322431195
0.31758905834	0.2821265799855
0.3176689521004	0.2822395031733
0.3177455938122	0.2822496722228
0.3178432858731	0.2823526967925
0.3179257206908	0.2824293030283
0.3180119823617	0.2825836208003
0.3180920829459	0.282606843964
0.3181804553672	0.2826478901945
0.3182755382667	0.2827320703682
0.3183653972361	0.2828762807258
0.3184521838039	0.282976798831
0.3185448435548	0.2831127675244
0.3186163820125	0.2831658005578
0.318710470373	0.283446117618
0.318786303497	0.2836449884375
0.318876061298	0.2836492243192
0.3189688005787	0.283690541064
0.3190504249627	0.283692635053
0.3191266992514	0.2842110170471
0.3192126909742	0.2842176756188
0.3193003398443	0.2844132473273
0.3193855612797	0.2848107723685
0.3194781303401	0.2850895238068
0.3195547844886	0.2855966313139
0.3196347506141	0.285633378518
0.3197308499285	0.2856978245012
0.3198268933158	0.2857443012415
0.3199168514351	0.2859663404659
0.3200078218707	0.286151073316
0.320079003168	0.2866690678957
0.320170412977	0.2869784513756

0.3202640373213	0.2870212788762
0.3203607283958	0.2871094736549
0.3204541746352	0.2872216062527
0.3205695055612	0.2872490393609
0.3206724053616	0.2872958115823
0.3207606618538	0.2873263520769
0.3208512068054	0.2873496863778
0.3209372283331	0.2874052330609
0.3210156825623	0.2874521451346
0.3211110914714	0.2876735877922
0.3212049900815	0.2878603315011
0.3212901637238	0.2878953796621
0.3213878714549	0.2879374831898
0.3214816708548	0.2879663043498
0.3215757308211	0.2880173911754
0.321674217131	0.2882687763718
0.3217509421815	0.2883760420387
0.3218320139423	0.288409644756
0.3219486408747	0.2884448398505
0.3220435834269	0.2885031535408
0.3221256010055	0.2887506653423
0.3222107984146	0.2888425782974
0.3223177850675	0.2891555173588
0.32239118676	0.2892542426555
0.3224944098434	0.2893299948191
0.3225768584245	0.2893994559962
0.3226535657466	0.2893996619233
0.3227212251578	0.2895197207682
0.3228102517416	0.2895589809556
0.3229085403225	0.2896884275618
0.3230000892508	0.2901211521382
0.3230852105227	0.2904438441554
0.3231773944321	0.2904955702142
0.323272014088	0.29054415864
0.3233508892527	0.290671364717
0.3234521265939	0.2911991972768
0.3235447710121	0.2912152676
0.3236269443796	0.291529821888
0.3237215472754	0.2915305509141
0.323793466333	0.2916738966996
0.3238864082594	0.2916746671339
0.3239725086879	0.291742545584
0.3240407639323	0.2919191404587
0.3241270386819	0.2920748407335
0.3242120907746	0.2921132473612
0.3243092979481	0.2922314812378
0.3244002250294	0.2922513784771
0.3244878076155	0.2924725287935
0.3245787428931	0.2925977658326
0.3246669270434	0.2928957729392
0.3247654560568	0.2929359112887
0.324849935378	0.2930422362267
0.3249282008505	0.2930479909491
0.3250242878178	0.293062730051

0.3251142022915	0.2931590116019
0.3252029984476	0.2932520927963
0.3252973117834	0.2935791951427
0.3253787265215	0.2936712126447
0.3254760906343	0.293707569288
0.3255503939663	0.2938070618925
0.3256328429565	0.2938316198224
0.3257070907465	0.2938598078388
0.3257972392743	0.2940134042573
0.3258977985192	0.2946208161532
0.326001239271	0.2946585303227
0.326084545484	0.2946653558911
0.3261589425588	0.2949393418573
0.3262523335107	0.29494168895
0.3263434079596	0.2949531891478
0.3264380381833	0.2951628112878
0.3265242013	0.2952078683678
0.326620284309	0.2953025501096
0.326695281582	0.2953166781858
0.3267765981463	0.2955333341422
0.3268617114978	0.2955851122768
0.3269468242896	0.2958214972608
0.3270198043292	0.2958255440412
0.3270934790297	0.2960917540258
0.3271739669462	0.2960997080813
0.3272538232844	0.2963554354128
0.3273273979273	0.2964474083777
0.3273973918707	0.2964971712158
0.3275062009705	0.2965725412088
0.3275960747866	0.2965768392089
0.3276651930069	0.2966641554108
0.3277535839874	0.2967043831641
0.3278316458996	0.2967604628253
0.327916118806	0.2967680880454
0.3280009148061	0.2967874285112
0.3280949187912	0.296807005173
0.3281821017128	0.2968161499214
0.3282649914144	0.2968867337056
0.3283717349213	0.2970445949251
0.3284635812021	0.2970583048604
0.3285481786488	0.2971484661721
0.3286136383149	0.2974102983776
0.3287007552207	0.2974207229994
0.3287984731582	0.2974275799183
0.3288780712829	0.297563676275
0.3289692710116	0.2976507351002
0.3290565720453	0.298400019572
0.3291613178905	0.2984456740576
0.3292369459276	0.2988387597185
0.3293097299158	0.2988535221186
0.3294112076619	0.2988617344889
0.3294799192277	0.2988688919456
0.3295730457834	0.2989887163793
0.3296484738297	0.2989914373645

0.3297245802906	0.2991860623664
0.3298019516781	0.2991947264328
0.32989267742	0.299400749781
0.3299737526352	0.299470406212
0.330061764253	0.2998137002832
0.3301722748387	0.2999160942786
0.3302585665397	0.2999592015413
0.3303458152779	0.2999782937073
0.3304353870452	0.3000802875302
0.3305285226556	0.3000890239332
0.3306030005106	0.3003162826232
0.3306857444748	0.3005320634379
0.3307737401613	0.3005373098831
0.3308538399979	0.3006096910472
0.3309387887337	0.3006548030728
0.3310200145567	0.3007996065791
0.33110865877	0.301179405466
0.3311891479434	0.3012694716901
0.3312729204663	0.3013266289045
0.3313906256007	0.301425786394
0.3314873429422	0.3016112998411
0.331574072636	0.3016619527774
0.3316707006613	0.3016988862152
0.3317604277708	0.3017444007185
0.3318353431008	0.3020107973024
0.3319122861987	0.3020852065483
0.3320138020891	0.3023017423547
0.3321160727236	0.3023646041316
0.332191770519	0.3023752391602
0.332300598606	0.3025542915373
0.3323987820996	0.3027909455354
0.3324894861023	0.3027964331382
0.3325758224263	0.3030273283321
0.3326584339412	0.3034086727698
0.3327413288591	0.3034672269741
0.3328243279421	0.3035417761991
0.3329072437245	0.3038336017984
0.3329950748973	0.3040201815754
0.3330831218993	0.3041477718898
0.3331911810664	0.304520290741
0.333279449478	0.3045471252204
0.3333692785177	0.3045725283558
0.3334654658598	0.3045837500523
0.3335602671674	0.304619271857
0.3336499749896	0.3047269770979
0.3337448601926	0.3049860577706
0.3338472952036	0.3054896380108
0.3339316644298	0.3056275558301
0.3340212821674	0.3057001674579
0.3341016172888	0.3057759771788
0.3341995432766	0.3058156850285
0.3342668789913	0.3060869479442
0.334364318217	0.3062749146756
0.3344399758413	0.307188175546

0.3345300878427	0.3072770863886
0.3346140195518	0.307346241319
0.3346883259451	0.3078772907834
0.3347641258439	0.3079610725727
0.3348458127595	0.3081198634961
0.3349324004493	0.3082260254829
0.3350302805877	0.308320052925
0.3351033746733	0.3084817009106
0.335193925743	0.3088371597265
0.3352731612109	0.3088396529409
0.335356127372	0.3089326847987
0.3354390035368	0.3091215719323
0.3355268872937	0.3093794227891
0.3356064284068	0.3095843174126
0.3356809119908	0.3096618055234
0.3357705012024	0.3099002687109
0.3358432716265	0.3099493241252
0.335922598845	0.3099497815596
0.3359946396395	0.310024283488
0.3360826209632	0.3102124399439
0.3361578153929	0.3103521829755
0.3362376478818	0.3103657236528
0.3363193518664	0.3104929849749
0.3364213224857	0.3106096473429
0.336529583378	0.3106765857035
0.3366221675437	0.3107257392094
0.3367056297874	0.3107755345964
0.3368020957026	0.3109115501865
0.3368886804097	0.3109566678266
0.3369795687393	0.3109946960571
0.3370736475996	0.3110192896003
0.3371656892331	0.3110723721741
0.3372572229345	0.3111165269714
0.3373474795438	0.3112988828886
0.337438376254	0.311448152197
0.3375168577397	0.3120312620843
0.337598519479	0.3120563266497
0.3376799864425	0.3120729036633
0.3377736699263	0.3122028954416
0.3378774360756	0.3122080832603
0.3379564555829	0.3122699699999
0.3380613220941	0.3122711601437
0.3381384088235	0.3123357398633
0.3382223695646	0.312363528778
0.3383188780014	0.312537403133
0.3384194877223	0.3125659072729
0.3385021821008	0.3125761257838
0.3386044864599	0.3131790910688
0.3386998999357	0.3131995457081
0.3388019119864	0.3132229845503
0.338889185476	0.3132938466537
0.3389670936956	0.3134680498585
0.3390600448623	0.313514698551
0.3391576517424	0.3135618880873

0.3392353735128	0.3136529654639
0.3393044214329	0.3136615011491
0.3393851265878	0.313691519227
0.3394693693208	0.3139028766864
0.3395604668944	0.314106652928
0.3396476521132	0.3142144746321
0.3397254909853	0.3142480537192
0.3398040093103	0.3144232186873
0.3399075278908	0.3144667618832
0.3399980985345	0.3145883834463
0.3400757590535	0.3145970199179
0.3401615219098	0.3146140130692
0.3402506952095	0.3147086973986
0.340328551649	0.3147230892737
0.340435073577	0.3148912471289
0.3405228921562	0.3150180123005
0.3406197850099	0.315225089879
0.3407172373474	0.3152787846188
0.3407986412374	0.3152926051373
0.3408775856828	0.3154088132333
0.3409811253466	0.3154770082058
0.3410737495831	0.3156077849321
0.3411572034651	0.3158058353311
0.3412471362979	0.3160736944747
0.341334356166	0.3164050084519
0.3414287765422	0.3164463228933
0.3415148174343	0.3164701805884
0.3416020195453	0.3166137321125
0.34171621321	0.3167292121968
0.3418012799571	0.3168302645388
0.3418736810116	0.3168313721205
0.341970525244	0.3171437579249
0.3420397525761	0.3171859587713
0.342145051087	0.3171969194926
0.3422521297679	0.3172217007468
0.342337893597	0.3173820762457
0.3424266758443	0.3174549812616
0.3424972243266	0.3174592599947
0.3425688262007	0.3176115959296
0.3426628433996	0.3178077128246
0.3427590689498	0.3180742693483
0.3428418125867	0.3180909569269
0.342952770508	0.3182316657725
0.3430603600143	0.318788661739
0.3431556136384	0.3189670217196
0.343238159356	0.3190478007752
0.3433272624939	0.3190740425916
0.3434154396437	0.319187345203
0.3435019918573	0.3192109258402
0.3435733176928	0.3192448415384
0.3436635481902	0.3193769351276
0.3437640838085	0.3196529584854
0.3438611539719	0.3197340756594
0.3439518395045	0.3197859981822

0.3440498681941	0.3200464886154
0.3441343764734	0.3201259978231
0.3442097847673	0.3201660345973
0.3442762915197	0.3201718716874
0.3443582213067	0.3204156771093
0.3444429537346	0.3204646548343
0.3445299628968	0.3204727481965
0.3446201658844	0.3206329228204
0.3447245690173	0.3206386308113
0.3448066540325	0.3206601124471
0.3448827667542	0.3208744113702
0.3449762198959	0.320947167117
0.3450623456919	0.3210198753979
0.3451489290934	0.3210358684567
0.345231993024	0.321176820843
0.3453345162384	0.321228162556
0.3454250296996	0.3213517720211
0.345514917593	0.32142520224
0.3456041156436	0.321460505667
0.3457040551896	0.3214793406587
0.3457890372622	0.3214965691238
0.3458647585994	0.3219345267683
0.3459526999183	0.3219418839029
0.3460380691057	0.3221583391177
0.3461261733351	0.3222930313879
0.3462028162763	0.3223794727306
0.3462934635535	0.3224898261737
0.3463770375896	0.3227428048642
0.3464567142625	0.3227479491391
0.346557045647	0.3228046880121
0.3466173425668	0.3228203874455
0.346708158708	0.3228819652234
0.34676952751	0.3228869664054
0.3468871668825	0.323118982342
0.3469698274606	0.3231491179481
0.347056360802	0.323205749723
0.347130525318	0.3233918031492
0.3472091513741	0.3235079532517
0.3472994708738	0.323549372093
0.347397963718	0.3235691181333
0.3474741645571	0.3238841868918
0.347552483242	0.3238891887281
0.3476617503999	0.3240850019448
0.3477561719052	0.3240982574463
0.3478325969004	0.3242107901864
0.3479151030024	0.3242519033076
0.3480002396026	0.3243644547046
0.348089008477	0.3244319569998
0.3481706656737	0.3244857457893
0.3482695438275	0.3246640877099
0.3483545744529	0.3246815321072
0.3484395315476	0.3247740187841
0.3485013791002	0.324902712399
0.3485844808663	0.3249363893103

0.3486789192206	0.3249878715457
0.3487659650644	0.3252665349945
0.3488459968384	0.3252837342733
0.3489484715459	0.3252946347252
0.3490465244443	0.3253349310253
0.3491285567923	0.3254516987827
0.349223951282	0.3255224945614
0.3493259365031	0.3255700902901
0.3493985397436	0.3255817248959
0.3494881393264	0.325634941238
0.3495688171253	0.3257310302507
0.3496511242843	0.3257389697764
0.349751418521	0.325745124723
0.3498249161413	0.325853475714
0.349903825963	0.3259365746111
0.3500127257862	0.326206341568
0.3500923204726	0.3265714571036
0.3501885774897	0.3266026792666
0.350267841298	0.3266667022963
0.350348344415	0.3270217178814
0.3504322674772	0.327027850183
0.3505264134192	0.3270417710669
0.3506225389915	0.3270669188203
0.3507282778668	0.3271449274399
0.350804779135	0.3271617288689
0.3508913612662	0.3272373815976
0.3509735898697	0.327262235979
0.3510673415821	0.3276808990902
0.3511515110178	0.3277263049016
0.3512522627132	0.3278991544643
0.3513525536974	0.3282120564843
0.3514438245969	0.3282446603068
0.3515319301266	0.3284686322712
0.3516221920456	0.3285271677613
0.3517008315819	0.3288083291756
0.3517977848276	0.32902551347
0.3518745598232	0.3292397841639
0.3519552522808	0.3292768816004
0.3520409957956	0.3293300690776
0.3521173536221	0.3295083304597
0.3521981953773	0.329538447237
0.3522772314608	0.32962207721
0.3523596808737	0.3298543150885
0.3524544314788	0.3299034443978
0.3525558685274	0.3299976425305
0.3526432441984	0.3300208222819
0.3527289513748	0.330049511234
0.3528180761744	0.3300521553567
0.3529056523606	0.3304084688716
0.3530118559228	0.3306707832787
0.3530978081348	0.3306980877581
0.353181587364	0.3307739512216
0.3532586977094	0.3307912045864
0.3533501000283	0.3308807362863

0.3534171433025	0.3309061587325
0.3535062847769	0.3309090803449
0.3535992751221	0.3311057527768
0.3537058891762	0.3315985596314
0.3538112169587	0.3316794670713
0.3539001997363	0.331729942472
0.3539838710194	0.3319035144688
0.3540604615176	0.3321375821684
0.3541507945609	0.3323937765716
0.3542759382675	0.332577466791
0.3543585208508	0.3325928314082
0.3544533214606	0.3327384059666
0.3545503711066	0.3331233044622
0.3546352478907	0.3332218531605
0.3547037779225	0.3333146551082
0.3547862609002	0.3333702104717
0.3548927127731	0.3333932935011
0.3549846389593	0.3336082617265
0.3550798105886	0.3336225563266
0.3551657152444	0.3340041116097
0.3552504957622	0.3343333057698
0.3553145815534	0.3346314953227
0.3554094822963	0.3349682609846
0.3554924629834	0.3351363660306
0.3555626999393	0.335143737377
0.3556567829359	0.3351575681789
0.355728059043	0.3352786694048
0.3558179965236	0.3355082323857
0.3559076871278	0.3359264643206
0.3560038086909	0.3359678428438
0.3560874209315	0.3359776943763
0.3561803056447	0.3359783027175
0.3562656010114	0.3363129646074
0.3563493078656	0.3363716445237
0.3564379277771	0.3363829915923
0.3565357769785	0.3365525340293
0.3566214725403	0.3366113035666
0.3567058330232	0.3366660301205
0.3568060898927	0.3369645191213
0.3569021632061	0.3369969976048
0.3569740435477	0.3373458235777
0.3570609785961	0.33742019491
0.3571382914149	0.3375195252454
0.3572182257655	0.3381802499553
0.3573172780768	0.3382987260266
0.3574092401139	0.338348454049
0.3575173386218	0.3384392199795
0.3576068242471	0.3384787893308
0.3576734427209	0.3385239435084
0.3577727314406	0.3385662360266
0.3578563702125	0.3388550860778
0.357953115975	0.3388752462859
0.3580535311727	0.3389018850939
0.3581314243559	0.3389935889853

0.3582183657243	0.3390516665767
0.3583059588885	0.3391114276151
0.358399638054	0.339579321741
0.3585142942312	0.3397858155776
0.3585881727541	0.3399048883778
0.3586868049209	0.3401072677554
0.3587756899713	0.3401082522981
0.358847931327	0.3401188387526
0.3589244084334	0.3402918447149
0.3590087736856	0.3403093001827
0.3591040709223	0.3403431840584
0.3592017582001	0.3403726972394
0.3592665682292	0.3404846143739
0.3593513484647	0.3407348218754
0.3594386623794	0.3407727825418
0.3595220636233	0.3410726334
0.3596239574851	0.3411181733145
0.3597138412754	0.3414719882586
0.359802717103	0.3414776637983
0.3598841379375	0.3415009706019
0.3599864949492	0.3415680709234
0.3600847267607	0.3416417644932
0.360174075101	0.3417625874655
0.3602679860692	0.3418317552832
0.3603567162801	0.3418757463435
0.3604397260169	0.3419320804687
0.3605373572214	0.3421299275519
0.3606314776583	0.342226073144
0.3607214569014	0.3422789984651
0.3608106748558	0.3424439106568
0.3608909796534	0.3424968534862
0.3609829214983	0.3425074936378
0.3610692430991	0.342509829749
0.3611441676713	0.3425446496752
0.3612264262632	0.3425689043184
0.3613007285857	0.3425759275806
0.3613736083886	0.342724346329
0.3614515545634	0.3427697834948
0.3615306219988	0.342806080096
0.3616249956495	0.3429013608373
0.3617005923451	0.343030678372
0.3617856075828	0.3431734636711
0.3618655353116	0.343176342942
0.3619655054616	0.3431867598387
0.3620553049729	0.3432137311038
0.362134657121	0.3432280994363
0.3622225851406	0.343272998927
0.362325023671	0.3432873954146
0.3623935334839	0.3433097822416
0.3624760884445	0.3434580622592
0.3625632598102	0.3437355054142
0.3626576351246	0.3437425906683
0.3627405477487	0.3438491122735
0.3628423718552	0.3439445495655

0.3629252845498	0.3439745416369
0.3630088228614	0.3441136358685
0.3630964217067	0.3441309274767
0.3631823183464	0.3443308530519
0.3632614879121	0.3444147921797
0.3633705143847	0.3445478756748
0.3634594797774	0.3446793918066
0.3635298727433	0.3447047205103
0.3636050986124	0.3447109672938
0.3637102549242	0.3447479311936
0.3638317535676	0.3450377312857
0.3639167989158	0.3451210817633
0.3639947642873	0.3453032604845
0.3640868851909	0.3453038353791
0.3641740913899	0.3453673517514
0.3642639509899	0.345594112987
0.3643507088705	0.3458721398107
0.3644339592159	0.3459776402804
0.3645322736311	0.3460142728284
0.3646062137056	0.3460738952063
0.3647009266457	0.3461305461167
0.3647875270769	0.3461336309919
0.3648858305767	0.3462240442166
0.3649691856531	0.3462760372365
0.3650593715173	0.3463052684899
0.365155268024	0.3463609331913
0.3652496226494	0.3465173258029
0.3653512217488	0.3466020151462
0.3654391570815	0.3469372608188
0.365523579397	0.3472291531819
0.3656161090811	0.3472537172826
0.3656966585303	0.3475586202761
0.3657744004365	0.3476252236788
0.3658701725883	0.3476963799052
0.3659675939071	0.3477393549601
0.3660693549962	0.3477731032469
0.3661419202302	0.3481713889445
0.3662234637448	0.3482481857552
0.3663153379761	0.3483770493297
0.3663897901326	0.3484346936843
0.3664816046616	0.3484514173468
0.3665664216565	0.3486077943674
0.3666560245732	0.3488532628051
0.3667370315417	0.3488880387654
0.3668348797425	0.3489224366414
0.3669383137897	0.3489235039842
0.367023078598	0.3489698286402
0.3671035260926	0.3490391923632
0.367195012077	0.3491105661129
0.3672931157396	0.3491209950451
0.3673850764641	0.3492147834318
0.3674711570733	0.3493505215216
0.3675664716835	0.3493893852418
0.3676565119144	0.3494418754759

0.3677403896467	0.3494878646334
0.3678222484882	0.3496281323436
0.3679035025493	0.3498957208225
0.367995320594	0.3500595084037
0.3680791625367	0.3501601584991
0.3681638089681	0.3504059453219
0.3682368610593	0.3506196239316
0.3683188865055	0.3508434716618
0.3684006619725	0.3509440263701
0.3684954136997	0.3509730611267
0.3685811828404	0.3511753468036
0.3686672088718	0.3512546406918
0.3687478952019	0.3512840753443
0.3688417084482	0.3514534239186
0.3689232532321	0.3515776893488
0.3690137053876	0.3515876197106
0.3690877402215	0.3516676238933
0.3691766328851	0.3516857067106
0.3692682817868	0.351791042583
0.369367938862	0.3520361475525
0.3694524019381	0.3522117976831
0.3695602894719	0.3522326938829
0.3696468443584	0.3523683136583
0.3697433924888	0.3525215177506
0.3698274596228	0.3526339529455
0.3699096512864	0.3527241829688
0.3699978369236	0.3529517171355
0.3700753080917	0.3530007209114
0.370162981251	0.3533086156509
0.3702689899509	0.3536212550416
0.3703541962845	0.3536722735467
0.3704318979703	0.3538300709995
0.3705077287654	0.3539697533742
0.3705963987409	0.354149817416
0.3706887302931	0.3542071099305
0.3707759673211	0.3543608321826
0.3708795496685	0.3544291991588
0.3709619273506	0.3545730993513
0.3710484035521	0.3546547086301
0.3711428266582	0.3546759723352
0.3712447001559	0.3547233536105
0.3713444374872	0.3548641912321
0.3714492320214	0.3549651850473
0.3715329341364	0.3549817392819
0.3716294073895	0.3550263753887
0.3717106866881	0.3552103995611
0.3718058569457	0.3552838262533
0.3718823045519	0.3553060991724
0.3719833985611	0.3554074263165
0.3720826815187	0.3557202893908
0.3721751016921	0.3557286133233
0.3722564618531	0.3558874635529
0.3723406628106	0.3559487690015
0.3724266735907	0.3559542461169

0.3724974209541	0.3561468486453
0.3725961318911	0.3562055988116
0.3726855793112	0.3562546966275
0.3727654670915	0.356384523871
0.3728574619865	0.3564680821531
0.3729458182628	0.3565341955829
0.373039287991	0.3566533232618
0.3731260072658	0.3566850949594
0.3732143726503	0.3566895981735
0.3732936734208	0.3569059212995
0.3733735495378	0.3569819974065
0.3734620301192	0.3570289295812
0.3735660574432	0.3570319040926
0.373637636859	0.35718079074
0.3737281777065	0.3576417013175
0.3738235929977	0.3577219200702
0.3739261392876	0.3578601015309
0.3739998373715	0.3580993641446
0.3740842286486	0.358168572816
0.374166082699	0.3582384860471
0.3742688990187	0.3583326723216
0.3743692098245	0.3585814304976
0.3744442660643	0.3586147408207
0.3745352336842	0.35884795712
0.3746381110788	0.3588981967902
0.3747320595888	0.3589605853843
0.3748145046071	0.3591284452512
0.374884800329	0.3591731665897
0.3749628780142	0.3592881399255
0.37505873497	0.3593115315158
0.3751581632328	0.3593557094613
0.3752529620306	0.3594670360833
0.3753373542075	0.3595258573835
0.3754336270107	0.3595955819186
0.3755256612317	0.3596501865022
0.3756137713302	0.3596591024759
0.3756979301445	0.3598459840718
0.3757826889355	0.3599691949734
0.3758561769681	0.359991937659
0.3759570532813	0.3603206233655
0.3760425957559	0.3604828737442
0.376123292744	0.3604989454822
0.3762038648387	0.3605389409813
0.3762875392488	0.3606074938389
0.3763649393716	0.3606813811814
0.3764517887521	0.3607079735424
0.3765486701779	0.3607480809974
0.3766372786314	0.3610738095936
0.3767237731314	0.3611620816603
0.3767876591794	0.361266793997
0.3768763793103	0.3614194863568
0.3769813585414	0.3615406985707
0.3770643090818	0.3615836416921
0.3771428876723	0.3616061165421

0.377240157685	0.3616074897578
0.3773317702078	0.3617102003274
0.3774185271361	0.3617371463264
0.3775206430484	0.3617953735998
0.3776155048897	0.361853321599
0.3776942660798	0.3619200349752
0.3777675217553	0.3619455187042
0.3778492459653	0.3619548212684
0.3779323250516	0.3621070381551
0.3780294392828	0.36214185909
0.378107581164	0.3621932300282
0.3782074398609	0.3622693325223
0.3782831552735	0.3622961214049
0.3783819747698	0.3623014780449
0.3784645335128	0.362411297469
0.3785565885815	0.3624398893072
0.3786547827747	0.3625335230209
0.3787371064652	0.3626415737456
0.3788173013227	0.3627687617195
0.3789176128542	0.3632129930945
0.3790059575968	0.3634417039341
0.3791038927921	0.3634476781975
0.3791893195875	0.3634511639033
0.3792908783025	0.3634818110478
0.3793600360615	0.3635487550449
0.3794390831766	0.3635552274565
0.3795285169115	0.3635819465938
0.3796262502603	0.3637305083047
0.3797138354309	0.3638671963484
0.3797993108371	0.3638821474203
0.3798786153222	0.3639109914732
0.3799630440584	0.3641687997659
0.3800529756521	0.3643327090485
0.3801416904439	0.3644663378706
0.3802331318033	0.3645968118012
0.3803223356818	0.3646197781728
0.3804200149786	0.3650712166706
0.3805121079684	0.3651586807441
0.3806166558164	0.3651640997367
0.3807015431307	0.3654180181689
0.380784762519	0.3654389087678
0.380862596963	0.3655089958561
0.3809599534325	0.3656452668404
0.3810527296856	0.3657136178953
0.3811424744714	0.3657318966372
0.3812247098263	0.3657615961328
0.3813111107157	0.3658277642835
0.3813956705366	0.3658490621851
0.3814811342942	0.3659602572943
0.3815684635784	0.3662058022587
0.3816485951762	0.3663183991788
0.3817444627376	0.3663231376333
0.3818410973446	0.3663589935817
0.3819243630779	0.3663727345565

0.3820124472972	0.3665996014897
0.3820889822982	0.3668098503793
0.3821750633996	0.3669230605054
0.3822623707223	0.3669859752918
0.3823255656643	0.3671070238324
0.3824048977833	0.3672057397811
0.3824983748426	0.3673964025225
0.3825970655953	0.3676347088705
0.3826791098771	0.3677784039356
0.3827667261883	0.3678299346112
0.3828610440331	0.3684629113192
0.3829393816946	0.3685147678642
0.38302279229	0.3685329701965
0.3831242411204	0.3685785537407
0.3832145784383	0.3687049646063
0.3833023703455	0.3687197827211
0.383386892759	0.3687552442577
0.3834680809843	0.3687638579678
0.38355368919	0.3688436959661
0.3836514929007	0.36884868674
0.3837321008091	0.3688637567519
0.3838258328376	0.3689533711571
0.383908007218	0.3689569671313
0.3839844038172	0.3690562118099
0.3840750276851	0.369754430599
0.3841733042888	0.3698007116276
0.3842671360701	0.3701596117387
0.3843660976982	0.3701843360234
0.3844378528892	0.3703490539831
0.3845413540413	0.3704140045771
0.3846433069023	0.3704403170785
0.3847122602897	0.3706841844671
0.384787117905	0.3708742126101
0.3848744403617	0.3709592865886
0.384967506181	0.3711367013404
0.3850568602923	0.3713478797685
0.3851676015537	0.371516214237
0.3852675825388	0.3719616891017
0.3853388988211	0.3720401669727
0.3854301308036	0.3720443235929
0.3855279983561	0.3721203412482
0.3856228415958	0.3724033053282
0.3857249238575	0.3724271131334
0.3858129315635	0.3724757411084
0.3859072510135	0.372646477282
0.3860045171222	0.3728012101986
0.3860960633465	0.3728281852174
0.3862015125147	0.3729147875694
0.3862948446619	0.3731391964788
0.3864007958702	0.3732234101668
0.38648603631	0.3732479619513
0.3865779736998	0.3732565769786
0.3866822697782	0.3734280183541
0.3867648936362	0.3737850797206

0.3868577894655	0.3738067570742
0.3869449578698	0.374141017239
0.3870359751868	0.3743526034009
0.387131490229	0.3745918156105
0.3872306233466	0.3746152467796
0.3873170283628	0.374685675396
0.3873992558233	0.3747424880769
0.3874756868251	0.3747688398742
0.3875744519065	0.3748045854532
0.3876562046601	0.3748783320165
0.3877648479524	0.3750388337845
0.3878666898567	0.3751012407643
0.3879633754909	0.3751930427842
0.3880491278028	0.3752632239863
0.3881322560425	0.3752842985386
0.3882225291041	0.3754154658108
0.3883299206503	0.3754937608358
0.388414860494	0.3755820450709
0.388500540363	0.3759621912289
0.3885878840853	0.3759910937132
0.3886691721606	0.3760601361234
0.388749338791	0.3761119117365
0.3888518284031	0.3761979661764
0.3889417555486	0.376561912067
0.3890177995218	0.3766294701095
0.3891039920613	0.3767227874281
0.389176936378	0.3767954997894
0.3892805465484	0.3769355831597
0.3893731801673	0.3772621489628
0.3894821922633	0.3774544313953
0.3895662021307	0.3774762327279
0.3896657547414	0.3775558928943
0.3897626435218	0.3778908202428
0.3898643778912	0.3779055497718
0.3899434683442	0.3781755995111
0.3900271574115	0.3783591728613
0.3901174562597	0.3786122366673
0.3902056032464	0.3789741447116
0.3902904064652	0.3790547058002
0.3903673475475	0.3790669765957
0.3904539335747	0.3790710588247
0.3905363755602	0.3791910091753
0.390641958749	0.3796902907112
0.3907183921889	0.3797161570969
0.3907949156528	0.3797178663034
0.3908915154038	0.379770079529
0.3909727939645	0.3799577103647
0.3910567813438	0.3799685969038
0.3911486669633	0.3800085625321
0.3912391029231	0.3800425255023
0.3913438880828	0.3802943230432
0.3914182563559	0.3805044802607
0.3915145216273	0.3807247645015
0.3916132278634	0.3808371464433

0.3917047185777	0.380853052479
0.3917846178765	0.3810004477671
0.3918865598401	0.3812071359238
0.3919612834146	0.381277646617
0.392046345871	0.3814176718943
0.3921334841059	0.3814982523585
0.3922150845966	0.3815265952422
0.3922955895835	0.3818606378496
0.3923909685216	0.3818789735299
0.3924928330427	0.3819440290514
0.3925878987781	0.3821238227703
0.3926768544002	0.3821900046854
0.3927636059373	0.3822259965538
0.3928507206526	0.382330144336
0.3929303033217	0.3828880472651
0.3930092255958	0.3829756347283
0.3931053258892	0.3830751355078
0.3931984055778	0.3830772340914
0.3933126474929	0.3831128598802
0.3934031628583	0.3831521445225
0.3934872201397	0.3832095385424
0.3935726631624	0.3835421584024
0.3936522819292	0.3837377552979
0.3937340265701	0.3838012958252
0.3938344552812	0.3838750609985
0.3939156561347	0.3839001682285
0.3940067489681	0.3839387850956
0.3940922398478	0.3843734314638
0.3941693633097	0.384430563809
0.3942675798932	0.3844478932311
0.3943633737377	0.3845028756447
0.3944488518833	0.3845555186028
0.3945364968961	0.3846432761249
0.3946311619231	0.3847198608269
0.3947075599504	0.3847229851672
0.3947873046363	0.3847829824702
0.3948520056736	0.3848053239415
0.3949400780267	0.3848915635127
0.395015976044	0.3850800053957
0.3951047938554	0.3851966551696
0.3951855566337	0.3851998243057
0.3952767089188	0.3852142567985
0.3953496029442	0.3855108372152
0.3954367580205	0.3855597507761
0.3955311401778	0.3856732254576
0.3956293060328	0.385716840073
0.3957177071095	0.3857576735628
0.3958163232273	0.386203865259
0.3959148605298	0.3862418499889
0.3959947636069	0.3862810409672
0.3960692176323	0.3863340258436
0.3961602352003	0.3863588297507
0.3962389392358	0.3864155643906
0.3963220869401	0.3864618765402

0.396411427441	0.3864811125744
0.3964940095468	0.3867605984544
0.3965925179619	0.3868340276603
0.3966735114159	0.3869036467231
0.3967679236121	0.386927053907
0.3968516899422	0.3869599233182
0.3969458167106	0.3872380684361
0.3970341617876	0.3874340349405
0.3971155685788	0.387597548617
0.3972031492467	0.3877434387463
0.3972935934386	0.3879579165597
0.3973735354522	0.3880863277282
0.3974623778976	0.388256053481
0.3975425445992	0.3884059855654
0.3976336626607	0.3884149135151
0.3977269530773	0.388438009667
0.3978082690359	0.3885892866318
0.3978881699491	0.3886832038308
0.397979615152	0.3887331286007
0.3980753357955	0.3888269692673
0.3981671877348	0.3890314723313
0.3982526103601	0.389059401906
0.3983275932372	0.3891487457344
0.3984271517369	0.3899525451936
0.3985179656126	0.3900159859134
0.3986047071569	0.3901084701997
0.3986981930422	0.3901127636152
0.3987828906578	0.390297195782
0.3988716291454	0.3906664727417
0.3989430891845	0.390690928964
0.3990356915393	0.3907387207067
0.3991036472555	0.3909579612946
0.3991839661761	0.3913488297075
0.3992785280397	0.3914102003886
0.3993547536004	0.3917021487889
0.3994466372873	0.3918635704847
0.3995300683515	0.3918932462652
0.3996274917318	0.3919427579253
0.3997186335983	0.3919981523954
0.3998114053801	0.3920396389535
0.3999050372355	0.3920663927223
0.3999800533867	0.3922066036991
0.4000737922469	0.3922464575452
0.4001643929974	0.3923600631435
0.40026135923	0.3926489205721
0.4003419663412	0.3926729109654
0.4004332020164	0.392772647902
0.4005248111836	0.3932337951632
0.4006162135465	0.3933308784171
0.4007018288757	0.3934566311956
0.400798231767	0.393856752157
0.4008864072969	0.393974378968
0.4009834143894	0.3939967946881
0.4010691516791	0.3940053583327

0.4011537245578	0.3940463947089
0.4012431994224	0.3940822471354
0.4013476370564	0.3943592205979
0.401432767398	0.3946927373615
0.401522720333	0.3949564999471
0.4016166257933	0.3950367676752
0.4017005391401	0.3952108768369
0.4018167078802	0.395275913903
0.401899333275	0.3952850397381
0.4019843346235	0.395454314852
0.4020690963254	0.3955624989886
0.40214253071	0.3955869211496
0.4022364353719	0.3958602808934
0.4023125453452	0.3961137037003
0.4024070482914	0.3961547873386
0.4024892231022	0.3963240102471
0.4025825912367	0.3963463963831
0.4026741757477	0.3963495495067
0.4027757017405	0.3964723222932
0.4028565416369	0.3966245167437
0.4029564382735	0.3967398631851
0.4030639419938	0.3967985939058
0.40316132596	0.3968482351774
0.4032633966618	0.3968698493074
0.403342603372	0.3969844268863
0.4034131518408	0.3970945259197
0.4034987597387	0.3971241874258
0.4035682908138	0.3976118295403
0.4036587973514	0.3977187624097
0.4037594247827	0.3978214993353
0.4038329106908	0.3979971862266
0.4039075223693	0.3980096924013
0.404002992098	0.3981543486164
0.4040896150017	0.3981829612191
0.4041780991045	0.398186685908
0.4042582201646	0.398237353984
0.4043566170664	0.3982749032867
0.4044385823883	0.3983737747168
0.4045219845771	0.3985384881595
0.4045869344645	0.3985941790092
0.4046809414967	0.3987827394679
0.4047712136508	0.398913290187
0.404875120186	0.398974742856
0.4049565126312	0.3990300643585
0.4050628526373	0.3995071532347
0.4051449989304	0.3995203175814
0.4052295541318	0.3995599548595
0.4053305804583	0.3997198514111
0.4054000433893	0.39991470446
0.4054871718538	0.399953672799
0.4055784876124	0.3999941885004
0.4056532670791	0.4000777920224
0.4057413066957	0.4003007704389
0.4058152059564	0.400343746044

0.4058949078385	0.4003590101049
0.4059640546213	0.4004777513259
0.4060418149986	0.4005273060877
0.4061233328243	0.4006171417349
0.4061951699961	0.4006284652106
0.4062782304015	0.4009623537708
0.4063623173945	0.4012298164025
0.40643708838	0.4013649561569
0.406522342112	0.4014293452814
0.4066111834831	0.4014801273032
0.4067007880403	0.4015154342784
0.4068006193619	0.4015227003037
0.4068978214885	0.4015322871366
0.4070042126379	0.4015450063989
0.4071029949065	0.4015754569236
0.4071990596957	0.4016499627007
0.4072973435901	0.4016762586849
0.407404302815	0.4016780553493
0.4074823734915	0.4017205338165
0.4075889881506	0.4017212419714
0.407663291926	0.401732603445
0.4077436189483	0.4017401417591
0.407846116718	0.4018101407245
0.407952969968	0.4018467231011
0.4080390398105	0.4019449910292
0.4081293161063	0.4022794688679
0.4082165905594	0.4025390393953
0.4083075856829	0.4027242204964
0.4083837416717	0.4028218276822
0.4084836458447	0.4028363309542
0.4085569179964	0.4029298049481
0.4086540427457	0.4029669870336
0.4087399184693	0.4030150361135
0.4088190781484	0.4030328313401
0.4088931633075	0.4031340442114
0.4089855755143	0.4031659704116
0.4090753139023	0.4031930572497
0.4091644296588	0.4032047744705
0.4092492935027	0.403368537643
0.4093275676427	0.4034108394775
0.4094198414322	0.4034870524927
0.4095160530734	0.4035848091777
0.409593915893	0.4036708805767
0.4096820051664	0.4037880158148
0.409772235583	0.4038971407109
0.4098702187314	0.404072403364
0.4099414116731	0.4043333441022
0.4100345980675	0.4043459217971
0.4101417321411	0.404391520479
0.4102401658493	0.4043929019627
0.410333457892	0.4047333949893
0.4104240858357	0.4047547449197
0.4105080061219	0.404942870562
0.4105982313314	0.4049853632886

0.4106828079293	0.4051638209692
0.410766565883	0.4052068943151
0.4108578450185	0.405644977357
0.4109368773518	0.4057100952579
0.4110239724039	0.4057509802625
0.4111059443831	0.4057534674034
0.4111925313543	0.4058352561934
0.4112757944847	0.4058419437413
0.4113514122296	0.4059189035269
0.4114308072506	0.405949688269
0.4115169120974	0.4061599623173
0.4115913208599	0.4061659473713
0.4116890862703	0.4062143296504
0.4117761160664	0.4063979931229
0.4118630359067	0.4064169938921
0.4119488255754	0.4064179760483
0.4120365150825	0.4067184500424
0.4121354055126	0.4067986875054
0.4122285140384	0.4068241087939
0.4123265468154	0.4068640046781
0.4124102680782	0.4068938597079
0.4124973736021	0.4070013766901
0.4125824928514	0.4071339332798
0.4126631802649	0.4071795500738
0.4127584448325	0.4071844481534
0.4128426416102	0.4072571256044
0.4129481647826	0.4073726662514
0.4130253221225	0.4076714581227
0.4131126612116	0.408036447239
0.4132062037171	0.4081354946428
0.4132922166539	0.4081392182832
0.4133698848631	0.4081402895545
0.4134555940308	0.4083646415774
0.4135677737297	0.4085465441365
0.41366182548	0.4085934192048
0.4137515277138	0.4086671355255
0.4138344184413	0.4087186773384
0.4139271329916	0.4088332551382
0.4140201612041	0.4088450305904
0.4141233077579	0.4088668875133
0.4142025892237	0.4088797797481
0.4143109818006	0.4090423740553
0.4143880971251	0.4092626019062
0.414462996006	0.4093047449607
0.4145558064963	0.4094876637145
0.4146472029953	0.4095632912963
0.4147391249289	0.4096417959871
0.4148257306478	0.4096594377572
0.4149199681994	0.4097013870125
0.4150098365173	0.4098565138411
0.4150990325434	0.4101514516801
0.4151918626225	0.4102591530656
0.4152907267695	0.4102660879595
0.4153732836803	0.4102672774494

0.4154460899463	0.4103293528218
0.415534641041	0.41049336419
0.4156419590256	0.4106192209905
0.4157238863101	0.4107789265295
0.4157964687324	0.4108992883651
0.4158873523425	0.4109472675654
0.4159910746476	0.4110443038506
0.4160796627751	0.4110447574137
0.4161734039685	0.4111301875494
0.4162760059996	0.4111531333264
0.4163574225709	0.4112159444916
0.4164367456267	0.4113637818326
0.4165461935032	0.4113866786139
0.416627434419	0.4113999945462
0.4167030626087	0.4114713622464
0.4167828334792	0.4117201645845
0.4168676107509	0.4119838448242
0.4169586716137	0.412039787978
0.4170470761909	0.4120527243437
0.4171352648402	0.412182545867
0.4172322855642	0.4121991064562
0.4173096909028	0.4129400416093
0.4174211484526	0.4130721996318
0.4175111906721	0.4133254194918
0.4176091022426	0.4133943852677
0.4176959960434	0.4135903853315
0.4177784167053	0.4135974766934
0.4178804306354	0.4138502491873
0.4179765224681	0.413897423129
0.4180497956001	0.4139114327277
0.4181228801585	0.4139232939562
0.41821977837	0.4140823734525
0.4183119437733	0.4141663309586
0.4184143236638	0.4141895099686
0.4185200934228	0.4142391410383
0.4186024547785	0.4142471470635
0.4186896166692	0.4142690856527
0.4187740377384	0.4143068995044
0.4188683556804	0.414453141562
0.4189646560495	0.4144727796482
0.4190547332825	0.4145023557279
0.4191709536172	0.4145277197931
0.4192687883969	0.4145360939651
0.4193530713884	0.4145761141969
0.4194453374947	0.4145958694628
0.4195424998612	0.4146585977676
0.4196210551006	0.4148199328954
0.4196910236167	0.4149378496259
0.4197687086541	0.4149684169618
0.4198580402066	0.4150898043909
0.419943492852	0.4151428691041
0.4200202488202	0.4152971994786
0.4201033201033	0.4153620560856
0.4201792382433	0.4154386183052

0.4202587580725	0.4159618676713
0.4203441972113	0.4160030317801
0.4204402476714	0.4160840299943
0.4205357427935	0.4161645846321
0.4206251531685	0.416239270423
0.4207086127522	0.4162706439797
0.4207813376737	0.4163436260034
0.420867286875	0.4164097118437
0.4209644971303	0.4165971587592
0.4210645727562	0.4168518647192
0.4211578999539	0.4169243320403
0.4212665062154	0.4169270660183
0.4213521623519	0.416987940022
0.421447732892	0.4170134700578
0.4215428150661	0.4170402660474
0.4216196439007	0.4171265394244
0.4217053472308	0.417206491862
0.4217850357188	0.4172319094859
0.4218798569682	0.4172473940453
0.4219791541824	0.4173553543227
0.4220678574703	0.4174453583109
0.4221507225584	0.4174968150467
0.4222282688695	0.4175482987995
0.4223252992876	0.4177437392466
0.4224250269238	0.4177492497874
0.422517207838	0.4180422710704
0.4225909367896	0.4180593011074
0.422666601861	0.4181589127908
0.4227552330493	0.4182186511327
0.422840483049	0.4183078107727
0.4229296334283	0.4183242469518
0.4230167011833	0.4183378303177
0.4231122003494	0.418416064738
0.4232094015767	0.4185610955391
0.4232929374194	0.4185802554204
0.4233720252835	0.4188967842494
0.4234568281393	0.4189930764554
0.4235474124023	0.4191990306241
0.4236222634034	0.4192761659387
0.4237242203343	0.419602112823
0.4238110597591	0.4197453396492
0.4239178669785	0.4197886243667
0.4239977119103	0.4198117478126
0.4241007390744	0.4198632420938
0.4241870433816	0.4200909090245
0.424279548775	0.4201965774666
0.4243690430996	0.4203089291057
0.4244736541771	0.4209378169176
0.4245708758435	0.4209454001756
0.4246681261885	0.4213588426714
0.4247495838148	0.4214587910994
0.4248289007936	0.4214691939391
0.4249347832678	0.4214898746457
0.425021676005	0.4215258325647

0.4251105123619	0.4216034454756
0.425182498498	0.4216064640327
0.4252694272268	0.4218345428077
0.4253659331284	0.4218384171213
0.4254561733387	0.4218492104145
0.4255497920384	0.4218876512776
0.4256315001431	0.4219731474055
0.4257262798507	0.4220071135357
0.4258205286566	0.422090566438
0.4259158646416	0.4221150266288
0.4260092691689	0.4221296765642
0.4260938232042	0.4222083423423
0.4261881013202	0.4222340867642
0.4262787330196	0.4222518025594
0.4263708988642	0.4225605487127
0.426461313344	0.4227520859216
0.4265595837569	0.4227952471362
0.4266410034469	0.4229309012535
0.4267449406519	0.4229540916388
0.4268482685657	0.4230034865098
0.4269362782994	0.4231108106934
0.4270282826412	0.4232185430252
0.427126021032	0.4232357112729
0.4272146440462	0.4234222638726
0.4273202806036	0.4234379278851
0.4274122577682	0.4234876482362
0.4275084760865	0.423688547967
0.4276048233849	0.4240325391075
0.4276859110606	0.4242990792256
0.427778027719	0.4243953122837
0.4278623398938	0.424423687562
0.4279618372593	0.4244767957439
0.4280803814223	0.4245820992592
0.4281727247866	0.4247103237438
0.4282408583535	0.4248257721061
0.4283408507471	0.4248987579717
0.428432846391	0.4249939704025
0.428519562447	0.4250423094222
0.4286239931715	0.4250973557105
0.4287346216457	0.4251518778669
0.4288301480694	0.425167714631
0.4289181384853	0.4253156355249
0.4290121762618	0.4253671799628
0.4290908695876	0.4255204398112
0.429184982803	0.4257651567877
0.4292656827001	0.4258217273454
0.4293727048493	0.4259298127696
0.4294535952734	0.4259302867241
0.4295249957992	0.4262430470948
0.4295869886142	0.4262854944072
0.4296781446398	0.4263564749214
0.429748111534	0.4264324717759
0.4298402383362	0.426639787852
0.4299203654157	0.4267752043305

0.4300140680086	0.4269297593949
0.4300985144421	0.4270385868085
0.4301834735334	0.4273267832027
0.430266603913	0.4274406307857
0.430342222662	0.4274952927993
0.4304470171932	0.4276099772852
0.4305403431921	0.4276264154955
0.4306389276292	0.4276338066571
0.4307329435782	0.4276375736846
0.4308124547506	0.4276742115424
0.4308918981314	0.4278425617416
0.4310086549944	0.4280528690363
0.4311180679168	0.4281328747808
0.431214786721	0.4282755771414
0.4312957908002	0.428400652872
0.4313694523846	0.4286571471441
0.4314545975272	0.428697023711
0.431554327536	0.4287656800207
0.4316436205581	0.4288589731172
0.4317313745511	0.4289287063834
0.4318075147354	0.4290580505067
0.431908821436	0.4292018641508
0.4319808907627	0.4292530230391
0.4320770405871	0.4293064177062
0.432149170006	0.4293573524433
0.4322347976412	0.4294179749985
0.4323203139487	0.4294873397886
0.4324074012039	0.4294928003762
0.4324987039682	0.4295096640417
0.4325711164397	0.4295591908606
0.4326506579533	0.4295998870622
0.4327336591784	0.4297467408394
0.4328131687022	0.4298058408642
0.4329095204277	0.4299475257877
0.4330117427988	0.4299679456323
0.43309864248	0.4300498890808
0.4331738451469	0.4302082903471
0.4332802067987	0.4303629976071
0.433380158183	0.4303737715959
0.4334749741791	0.4304106806873
0.4335641355041	0.4305891939803
0.4336621592173	0.4306816921349
0.4337300970374	0.4308130968057
0.433837855527	0.430849107276
0.4339227014083	0.4311363171174
0.4340073674597	0.4311902019366
0.4340902630109	0.4312220669612
0.4341903797013	0.4313355329466
0.4342740410462	0.4313977251089
0.434353492881	0.4315357151248
0.4344528309391	0.4316821159553
0.4345529013265	0.4316957018762
0.4346532779773	0.4321221636248
0.4347291060215	0.4321588392589

0.4348223147124	0.4322237912156
0.434900783602	0.4322730685049
0.4349898812829	0.432349558573
0.4351176027423	0.4324278808579
0.4352159768475	0.4325347519051
0.4352954599606	0.4325766686839
0.435378430092	0.4326757364993
0.4354495481011	0.4327815911821
0.435534552012	0.4329878051623
0.4356182053595	0.4330261708737
0.4357018416179	0.4331390410663
0.4357967783869	0.4333659851403
0.4358847742316	0.4334326175429
0.4359695807017	0.4335609176443
0.4360698547262	0.4337625317829
0.4361407289144	0.4338145437961
0.4362353425591	0.4338645882562
0.4363379926936	0.4340633220458
0.4364140024146	0.4340973582063
0.4365030767492	0.4341706437121
0.4365835907839	0.4342551599979
0.4366674947841	0.4344021199991
0.4367450896341	0.4344730156439
0.4368267254101	0.4348559490032
0.436937265419	0.4349729103326
0.4370439001966	0.4350048139577
0.4371355946486	0.4350171701645
0.437220237114	0.4350235507932
0.4373026838272	0.4350305325389
0.4373980155685	0.4350577465984
0.4374844609288	0.4350625614771
0.4375739777356	0.4352023993719
0.4376494181653	0.4352240154315
0.4377347770427	0.4353487098844
0.4378423166769	0.4354189234854
0.4379430103458	0.4355712172322
0.4380373670907	0.4356894891402
0.4381101071736	0.435720828731
0.4381864764521	0.4358931616618
0.4382669055231	0.4360160667802
0.4383560324942	0.4360536296838
0.4384355831861	0.4360982957211
0.4385491930948	0.4361062204181
0.4386490375912	0.4361955962396
0.4387536217984	0.4364786772356
0.4388346398359	0.4365320948114
0.4389167813239	0.4367865293333
0.438995021168	0.4368924711712
0.4390971802236	0.4370220794636
0.4391890815163	0.4371393402337
0.4392725318952	0.4373419342801
0.4393496315589	0.437442806124
0.4394374201971	0.4374478922602
0.4395392814553	0.4375895565948

0.4396200830735	0.4376702718658
0.4397150554755	0.4377712749456
0.439790830523	0.4378918263911
0.4398960385108	0.4379230100003
0.4399749676778	0.4380322340679
0.4400613330762	0.4380884230047
0.4401523081223	0.4381336499409
0.4402374972753	0.438252846421
0.4403079124723	0.4384104296104
0.4403980226105	0.4384190513313
0.4404960675628	0.4386023666021
0.4405809033482	0.4386150024924
0.4406535064424	0.4387046345676
0.4407516739017	0.4387443845907
0.4408624950111	0.4387772584619
0.4409471228622	0.4388790965756
0.4410259084744	0.4389035495102
0.4411127437646	0.4389157296078
0.44120839862	0.4390228461244
0.4412821232357	0.4390506066111
0.4413706600795	0.4391571694627
0.441460268411	0.4392436891736
0.4415503480635	0.4393327654749
0.4416266882157	0.4393536986324
0.4417139584884	0.4393735299584
0.4418107515702	0.4395983425677
0.4418955996022	0.440021112658
0.4419787239716	0.4401121979172
0.4420692205758	0.4401863241227
0.4421503002858	0.4402961413432
0.4422343828508	0.4403283136754
0.4423379610702	0.4403312397685
0.4424330348135	0.440865594787
0.4425272806282	0.4410069789642
0.4426343720934	0.4410830292747
0.4427405366356	0.4412056795026
0.4428510132225	0.4413611824777
0.4429379892235	0.4414626123741
0.4430225876299	0.4415181690898
0.4431086825848	0.4416568122158
0.4432042210888	0.4416595651157
0.4432947665977	0.4417552537319
0.4433833221045	0.4418213654212
0.4434743574218	0.4419578095713
0.4435738570294	0.4422077970424
0.443662247608	0.4424193808001
0.4437433874887	0.4427113921604
0.4438408697566	0.4428630696388
0.4439310377983	0.4430946702491
0.4440360076502	0.4432153606273
0.4441349133915	0.443227773574
0.4442341152281	0.4433390214371
0.4443159143027	0.4434398777924
0.444414227702	0.4434401898105

0.4445019313755	0.4435950119828
0.4446090072114	0.443595143676
0.4446934944084	0.4440145358803
0.4447793371004	0.4440484613501
0.444861540945	0.4440824281652
0.4449551638949	0.4441161751247
0.4450315358544	0.4445512808415
0.4451116581413	0.44459760883
0.4451922617831	0.4450376831026
0.4452673878937	0.4450412679693
0.4453663068878	0.4451559260652
0.4454604556685	0.4451853663118
0.4455506122632	0.4452356719878
0.4456414805645	0.445572458293
0.4457215905161	0.4456142442554
0.4458147527789	0.4456421892942
0.4459038861143	0.4457022341166
0.4459790268207	0.4457064525399
0.4460797172728	0.4457803129175
0.4461656368858	0.4460337375421
0.4462434302062	0.4460631244056
0.4463349296895	0.4462100198561
0.4464263152153	0.4462785229889
0.4465082784359	0.4463412531056
0.4466122012013	0.4463836038933
0.4466974711097	0.4463869211234
0.446790154929	0.4464297984462
0.4468867833202	0.4464898840344
0.4469696262846	0.4466151595271
0.4470657360623	0.4466527163399
0.4471666496191	0.4469166715544
0.4472467055563	0.4469307591739
0.4473266236936	0.4469773693178
0.4474109953795	0.4470628388194
0.4475132772445	0.4473056426414
0.4476107832969	0.4473576570061
0.4477114657637	0.4480517232754
0.4477999807095	0.4481829580463
0.4478806967747	0.44845531316
0.4479714458804	0.4485027066587
0.4480678422792	0.4490005645548
0.4481473732447	0.4490450186636
0.4482242675162	0.4491331287283
0.4483015721487	0.4491409514377
0.448394668724	0.4493167893523
0.4484729692042	0.44934145066
0.4485604585982	0.4494680185017
0.4486415403236	0.4498345235276
0.4487387691213	0.4498544797399
0.4488325211094	0.4500320250029
0.4489124154181	0.4500510386683
0.4490021738642	0.4502817700937
0.4491115736057	0.4503289333154
0.4492061831352	0.450335784173

0.4492975974516	0.4503386785666
0.4493828815266	0.4503666221555
0.449472731209	0.450388357059
0.44957225241	0.4504358621755
0.4496697320279	0.4504600630161
0.4497598532609	0.4506048379669
0.4498473105685	0.450726298978
0.4499380619299	0.4507572118585
0.4500232189587	0.4508957768743
0.4501036543843	0.4509908064918
0.4501893849418	0.4511600864891
0.4502915525219	0.4511652448566
0.4503659179948	0.4512601988916
0.4504596934264	0.451522319277
0.4505519779784	0.4515373917983
0.4506451851108	0.4515738391692
0.4507282437965	0.4516852461407
0.4508181201137	0.4518304701586
0.4509061251169	0.4518752829282
0.4509861188062	0.4519188366384
0.4510559172755	0.4520522142204
0.4511440076169	0.4521785262006
0.4512263826192	0.4527183946996
0.4513156952984	0.4528055531669
0.4514041697021	0.4528574227572
0.4515014034078	0.4529528685991
0.4515982678289	0.4530240508099
0.4516986968276	0.4530617765354
0.4517699644634	0.4530964559412
0.4518626539353	0.4532351866245
0.4519525137103	0.4533892888143
0.4520346191617	0.4534933027603
0.4521240071147	0.4535203635821
0.4522211932036	0.4535837656046
0.4523235318807	0.4536408821757
0.4524150757853	0.4536504390177
0.452504644679	0.4539775932739
0.4526060954156	0.4541776568064
0.4526981176161	0.4542289516576
0.4527742192783	0.4544645277678
0.4528672959521	0.4545446683523
0.4529290821339	0.4546689173912
0.453015582546	0.4547507557658
0.453094602549	0.4549443694072
0.4531940366381	0.4550239148539
0.4532798914999	0.455033167894
0.4533669146978	0.4551109879539
0.4534581101543	0.4552705272026
0.4535449601605	0.4556978110503
0.4536536026687	0.4558022887578
0.4537397307572	0.4558367328321
0.4538198668376	0.4559591520494
0.4539162989438	0.4559907944293
0.4540002445386	0.4560223337765

0.4540901087339	0.4560909185138
0.4541697984721	0.4563704919216
0.454248022883	0.4566479638066
0.454324970777	0.4566771657145
0.4544195867963	0.456711906754
0.4545076469965	0.4568580435815
0.4545811169055	0.4569088903294
0.454663317496	0.4570213735969
0.4547383390774	0.4575249678572
0.4548247906892	0.4575619933774
0.4549132574918	0.4575646641664
0.4549999963303	0.457681602461
0.4550960782921	0.4577108936387
0.4551776272167	0.4577183616103
0.455269542306	0.457890969079
0.4553592640612	0.457962614852
0.4554605471909	0.4582193770201
0.4555441021858	0.4583752492614
0.4556318486752	0.458617531594
0.455724779767	0.4586203293452
0.4558092718225	0.4586478874177
0.4558907489797	0.4588637349628
0.4559780762804	0.45891373847
0.4560921053236	0.4591260726957
0.4561732153282	0.4591857998353
0.4562777516729	0.4595324117567
0.4563701672093	0.4595896500454
0.4564573627072	0.4596782305641
0.4565499900415	0.4597343437692
0.4566411062522	0.4600366155388
0.4567261709015	0.4601850155408
0.456836043113	0.4604243333747
0.4569219830702	0.4604519217162
0.4570001124794	0.4606127375374
0.4570925929181	0.4606450174637
0.4571816858542	0.4613762039317
0.4572745057918	0.4616104164981
0.4573738249898	0.4616729504422
0.4574605867966	0.4617148157016
0.4575525493622	0.4618335190103
0.4576411422301	0.4618681272468
0.457730119545	0.46208176861
0.4578423792922	0.4621542140633
0.4579401871557	0.4621742695106
0.4580235781178	0.4622255447134
0.4581081098685	0.4622307840876
0.4581908992373	0.4624002099679
0.4582882755115	0.4624397618004
0.4583736356887	0.4626138696245
0.4584648851241	0.4628602997078
0.4585598789483	0.4631388815668
0.4586502003265	0.4631767618864
0.4587304590458	0.4632463739561
0.4588288693425	0.4633256330656

0.4589004635007	0.4635551993052
0.4590005169992	0.4637665023544
0.4590771329002	0.464044178404
0.4591598636276	0.4645975388228
0.4592389381997	0.4646312322716
0.4593168719624	0.4647163616163
0.4594112339298	0.4648273192079
0.4594894703112	0.4648868131213
0.4595741216171	0.4652385538521
0.4596522314952	0.4653938042768
0.4597493700191	0.4654416395539
0.4598556523414	0.4655793752817
0.4599395615947	0.4657178902911
0.4600209412381	0.465841991529
0.4601199445032	0.4659914642322
0.4602174790047	0.4660393238418
0.4603151256428	0.4665559661429
0.4603984160422	0.4666377887855
0.4604850891093	0.4670572756477
0.4605725054906	0.467146945088
0.4606535488241	0.4672472982431
0.4607408560557	0.4674745536937
0.4608287985298	0.4675128057048
0.460909006515	0.4675369041541
0.4610086064153	0.4675849456669
0.4611013925624	0.4676299131863
0.4611939678381	0.4677009207694
0.4612711971808	0.4678434769175
0.4613554696165	0.4680773017412
0.4614462829234	0.4680791116902
0.4615463937699	0.4681177707371
0.4616342854221	0.4681548136766
0.4617384538253	0.4682496399334
0.4618353638407	0.4682994182842
0.4619169804718	0.468402353121
0.4619952559719	0.46848509683
0.4621036638359	0.4686017060303
0.4622000279164	0.468639934334
0.4622823056943	0.4686753225652
0.4623731701953	0.4686875319815
0.4624510143799	0.468747117604
0.4625472472751	0.4687928131599
0.462640154989	0.4689985960554
0.4627221231216	0.4692270850747
0.4628164302239	0.4693299159193
0.4629065077926	0.4694113851305
0.4629909587208	0.4695182381713
0.463074528605	0.469714181557
0.4631718843006	0.4697785776216
0.4632632373345	0.4698383041789
0.4633626608382	0.4699664612129
0.4634418512573	0.4701040020001
0.4635583114758	0.4702084579793
0.4636582899619	0.4702193416341

0.4637709596019	0.4703382104121
0.4638491463764	0.4703730740436
0.4639433487145	0.4704947027733
0.4640300978015	0.4705418099553
0.4641119121337	0.4707166235876
0.4641916032299	0.4708617046604
0.4642807593344	0.4709183085424
0.4643675089326	0.4713843130504
0.464454251257	0.4715163946603
0.4645495195674	0.4716089548556
0.4646340557292	0.4717612615146
0.4647432183851	0.4717857601811
0.4648311324844	0.4717937286012
0.4649412250387	0.4719086220332
0.4650227972757	0.4719573806309
0.4651086018877	0.4720750896785
0.465197829633	0.4724549448444
0.4653248413208	0.472477115234
0.4653997521974	0.4726136393437
0.4654923936484	0.4726217927178
0.465592987725	0.472694427725
0.4656778643393	0.4728524049406
0.4657660010158	0.4728884581958
0.4658516050746	0.4732119184081
0.4659312981231	0.473319435403
0.4660159770283	0.4738197090897
0.4661024460301	0.4738517559359
0.4661822192158	0.474196229873
0.4662620461277	0.474246097325
0.466368268491	0.4743009031265
0.4664495638736	0.4743664505195
0.4665555987424	0.4744212482263
0.4666479366762	0.4747966055657
0.4667290967336	0.4749214557333
0.4668334821193	0.4750411377866
0.4669309902746	0.4752663856782
0.4670306803402	0.4753182670158
0.4671348601639	0.4754035379314
0.4672275806519	0.4754627467853
0.4673112694626	0.4755329162588
0.4673988786432	0.4756260285213
0.4675128545463	0.4756957521628
0.4675933614983	0.4757180801722
0.4676755040143	0.475962940087
0.4677787824244	0.4760277519745
0.4678587320044	0.4760612862963
0.4679404166917	0.4761240992225
0.4680531865669	0.4761295796527
0.4681335905953	0.4761349343429
0.4682247508195	0.4762902746822
0.4683235074237	0.4763634485401
0.4684323535675	0.4765784446884
0.4685257113769	0.4766403342609
0.4686319154499	0.4767154657023

0.4687140333427	0.4767494359047
0.4688171952637	0.4770090250747
0.4688960123191	0.4771856184723
0.4689873323606	0.4773795455237
0.4690801796382	0.4776821284827
0.4691716398909	0.4780065303339
0.4692734830037	0.4780435005177
0.4693374773299	0.4781701454497
0.4694285487772	0.4786418674511
0.469518253439	0.4786935845463
0.4696174991891	0.4787018422378
0.4697247386983	0.478966505937
0.4698214474627	0.47901726143
0.4699028453004	0.4790314817348
0.4700008010498	0.4797685029523
0.4701075825557	0.4798493583212
0.4702085551371	0.4798964999053
0.4702891225309	0.4799091894668
0.4703769719912	0.4801497898666
0.470462325211	0.4803531896669
0.4705626858427	0.4803632928153
0.4706485187822	0.480476288898
0.4707321521483	0.4805008554859
0.4708038659408	0.480674288078
0.4709028389233	0.4807552495538
0.4709889540341	0.4808280487588
0.4710739017566	0.481058966119
0.4711700530778	0.4812755456124
0.4712651985157	0.4813839016029
0.4713667306775	0.4814688044191
0.4714645057891	0.4816600918757
0.4715566608359	0.4818522288448
0.4716558091419	0.481973075893
0.4717484502245	0.4819811405142
0.4718568930238	0.4820158933248
0.4719687895497	0.4820283491834
0.4720620684272	0.4821559388398
0.4721400363772	0.482207137167
0.4722253739735	0.4822180280122
0.4723210017992	0.4822896962568
0.4724229637012	0.482423894359
0.4725053945592	0.4824314776058
0.472595795313	0.4824713234476
0.4726888913959	0.4825156852084
0.4727954359277	0.482604699625
0.4728924787252	0.4828862419825
0.4729839395155	0.4830291619537
0.4730772191742	0.4830690994229
0.4731631573531	0.483192604557
0.4732609965612	0.4833792725755
0.473336932222	0.483389548037
0.4734175284816	0.4834535110952
0.4735181904278	0.4835418613661
0.4736036455473	0.4835533712589

0.4737056045229	0.4839345347063
0.4738200035944	0.4839577658314
0.473909572014	0.4844161549691
0.4739828338625	0.4845103981556
0.4740638949053	0.4845385465016
0.4741718249626	0.4846269192532
0.4742655879649	0.4847328824629
0.474345890849	0.4848037002575
0.4744265446742	0.4848278545988
0.4745298128124	0.4848438775807
0.4746222151472	0.4852166563513
0.4747208307196	0.4852816470969
0.4748063494262	0.4854534059623
0.474895558473	0.485844534497
0.4749886956688	0.4860498907859
0.475089980397	0.4861022210274
0.4751742855781	0.4861850418482
0.4752635325493	0.4862606761362
0.4753574183655	0.4862955512409
0.475448709492	0.4865901659162
0.4755373399895	0.4866292907625
0.4756349424661	0.4866520340779
0.4757108223313	0.4869332908382
0.4758014990918	0.486935190916
0.4759009975584	0.487623097821
0.47599240049	0.4876461165579
0.4760726031152	0.4878371029599
0.4761794580838	0.4878570202224
0.4762712183213	0.4878956338671
0.4763632593616	0.4879280794573
0.4764443957682	0.4879767775295
0.4765317757131	0.4881957221506
0.4766107854503	0.488429822657
0.4767171024352	0.4887323060775
0.4768041568351	0.4889114681312
0.4768869355642	0.4890839320721
0.4769789580657	0.4890874712722
0.4770661589752	0.4892566627478
0.4771610552815	0.4895982558812
0.4772506848623	0.4896774575975
0.4773407734944	0.4898366519064
0.4774451247787	0.4899754730079
0.4775293069523	0.4900440444781
0.4776046938133	0.490149358763
0.4776859530529	0.4904106656831
0.4777783238329	0.4908294363533
0.4778794786214	0.4908511834227
0.4779652638557	0.4910733244982
0.4780411226586	0.491597524031
0.4781414935165	0.4916108180931
0.4782305034342	0.491649906743
0.4783263619865	0.4916582952438
0.4784204247563	0.4916628666063
0.4785027289475	0.4918258183386

0.4785957868784	0.4920188614607
0.4786818480788	0.4921633360707
0.4787841693728	0.4923861606236
0.4788707782951	0.4924661514968
0.4789497955365	0.492521039469
0.4790489648377	0.4926146268519
0.479140431445	0.4931943652101
0.4792238308404	0.4932468946578
0.4793244343339	0.4933598513821
0.4794085500259	0.4934294397047
0.4795161723507	0.4935109066326
0.4796070625317	0.4935648111195
0.4796991852879	0.4936517295017
0.4797902728498	0.4937394161826
0.4798839918998	0.4939589462249
0.479979172736	0.4939919263253
0.4800789363107	0.4942282713387
0.480169364454	0.4943325249014
0.4802525146575	0.4943789838885
0.4803771267556	0.4945462757944
0.4804546752479	0.4945963157039
0.4805617793071	0.4948411877483
0.4806468418432	0.494873000653
0.4807338815552	0.4949844353568
0.4808332572265	0.4951091171836
0.4809334660541	0.4951514612654
0.4810304786762	0.495211762048
0.4811213645052	0.495372112804
0.4812324679788	0.4954305067585
0.481326757005	0.4956929715359
0.4814218659392	0.495695264476
0.4815235248977	0.4957460307778
0.4816130693869	0.4958557180324
0.4817064521407	0.4960512834127
0.4817971197512	0.4960641171528
0.4818815791228	0.4964601672257
0.4819670524271	0.4965014701365
0.4820506329302	0.4966158538312
0.4821386058561	0.4966260402835
0.4822164458092	0.4966577615398
0.4822872710615	0.4966853667492
0.4823734643953	0.4967950644802
0.4824569985931	0.4968825075889
0.4825330564486	0.4970684262656
0.4826151029806	0.4973502433458
0.482696154283	0.4973534054509
0.4827757432282	0.4976005648
0.4828893148222	0.4976341654078
0.4829729286188	0.4978251665879
0.4830833460379	0.4978987794353
0.4831947272857	0.4979433258658
0.4832944492255	0.4981737032874
0.4834055617509	0.4983401686093
0.4834918024489	0.4984298145772

0.4835855200324	0.4985046597711
0.4836643257236	0.4985786406543
0.4837565798064	0.4986470807231
0.4838426873205	0.4988029992001
0.4839427926675	0.4988473511799
0.4840464837953	0.4989396388688
0.4841265295452	0.4989695477219
0.4842070328829	0.4990030938112
0.4843072393742	0.4990790932793
0.484402774125	0.4991481270605
0.4844949559135	0.4991613408408
0.4845739433466	0.4998501465198
0.4846734587722	0.4998878668616
0.4847522350963	0.5000232873479
0.4848584073691	0.5001190210054
0.4849435749028	0.5001426989904
0.4850216307759	0.5001449071127
0.4850960834237	0.5002827340635
0.4851725714246	0.5003850013974
0.4852589139133	0.5005785276735
0.4853397246049	0.5005963472098
0.4854361973152	0.5009732819067
0.4855386833373	0.5011361862162
0.485625953715	0.5011906996201
0.4857242373042	0.501319908186
0.4858286102698	0.5015640221812
0.4859149503772	0.5015741085157
0.4860118805976	0.5016393684733
0.4861058159905	0.5016463997303
0.4862102941421	0.5017064762459
0.4862878465228	0.5017384272265
0.486395321641	0.5019234384576
0.4865027908896	0.5019381252359
0.4865644076453	0.5019455787921
0.4866382045837	0.5019704625868
0.4867307569259	0.5021638715743
0.4868097056122	0.502205800377
0.4869137037136	0.5025737835527
0.4870293981407	0.5028154150769
0.487138130749	0.503149638278
0.4872229653164	0.5031879978629
0.4873144816516	0.5032291080525
0.4873890026681	0.5033142263119
0.4874787854485	0.5033456370949
0.4875637736584	0.5035317029065
0.4876722595315	0.5035507790715
0.4877774237257	0.5035695922799
0.487864908965	0.5036181461397
0.4879549334576	0.5036294188544
0.4880450492675	0.5037619926287
0.4881465068776	0.5038028543105
0.4882154556289	0.503821034169
0.4883099361325	0.5039236992724
0.4884034801615	0.5039316838429

0.4884840865238	0.5039320395016
0.4885742016684	0.5041292803164
0.4886661941667	0.5041499024835
0.4887765359168	0.5041685210121
0.4888607661617	0.5042689002308
0.4889597314614	0.5043729628022
0.4890453444063	0.5046447510529
0.4891374969622	0.5046574399347
0.4892595524877	0.5047085748288
0.4893501732691	0.504784756208
0.4894334153558	0.5050648840074
0.4895405128908	0.5052351301463
0.4896444399238	0.5053249410217
0.4897442171831	0.5054379493195
0.4898541976651	0.5057112426095
0.4899204168615	0.5058595771747
0.4900114204395	0.5059191789472
0.4901007327525	0.5059293584691
0.4901929084085	0.5059394661167
0.4902867525395	0.5060043923676
0.4903793817648	0.5060183020569
0.49046176671	0.5060402552857
0.4905384244203	0.5060999824074
0.4906398087517	0.5061238833724
0.4907325404373	0.5062071812178
0.4908326573836	0.5062677267199
0.4909258773762	0.5062826219787
0.4910126380137	0.5062978599206
0.4910983232241	0.5063596752774
0.4911842983446	0.5063607978836
0.4912652625868	0.5064889379237
0.4913501426858	0.5065315096631
0.4914518444031	0.5066904408699
0.4915448057463	0.5066927241442
0.4916402440323	0.5070060454033
0.4917350609522	0.5074222895958
0.4918350088957	0.5076380919239
0.491929575613	0.5076523451912
0.4920415573912	0.5076761963848
0.4921290180844	0.507739612267
0.492221160591	0.5077749183426
0.4923191017091	0.507850927163
0.4924260817477	0.508027382006
0.4925274829879	0.5081144301604
0.4926135936592	0.5081566641831
0.4926938651162	0.508217446504
0.49278129957	0.5082465866074
0.4928754953473	0.5083155838474
0.4929681076333	0.5083361308936
0.4930560337275	0.5085043174661
0.4931600620387	0.508783183436
0.4932596252821	0.5088113553552
0.4933474072393	0.508945770194
0.4934358062284	0.5090021613856

0.4935305733358	0.5091374544744
0.4936204025769	0.5095674170235
0.4937243534877	0.509860167732
0.4938130621551	0.5100603011886
0.4939039019955	0.5103451193573
0.4939987904478	0.5103575100599
0.4940933402126	0.5105636723122
0.4941830047625	0.5106437439117
0.494274607707	0.5110849025163
0.494363586495	0.5114650341976
0.4944407888601	0.5117530052691
0.4945071208251	0.5118046316242
0.4945860814079	0.5118387029818
0.4946804512556	0.5118545185563
0.4947847751679	0.5119206359628
0.4948624955579	0.5121264844071
0.4949506882268	0.5122038781979
0.4950380939511	0.5122873127948
0.4951217755088	0.5126137387071
0.4952073017028	0.5126331670678
0.4953194639469	0.5126786608633
0.4954215461149	0.5126947224022
0.4955295314729	0.5129594443854
0.4956176588112	0.5130095448086
0.4957193890712	0.5130121041347
0.4958180445023	0.5130590238708
0.4958954432317	0.5130764667302
0.4960000943681	0.5138329656986
0.4961146780606	0.5143144832206
0.4962058563585	0.5144658600354
0.4963034792865	0.514612617004
0.4963818691066	0.5150043799729
0.4964747746067	0.5150877367385
0.496557801517	0.5152174724008
0.496661749856	0.5153283637516
0.4967538333416	0.5153938803851
0.4968452419963	0.5154379397837
0.4969232180521	0.5154755864709
0.4970385433434	0.5155423979923
0.4971352222581	0.5156026972664
0.4972248238161	0.5156810168779
0.4973048039877	0.5157870677711
0.4973880469888	0.5159444581602
0.4974753069314	0.5159674754733
0.4975605017782	0.5160691453018
0.4976424673082	0.5161280425457
0.4977294970746	0.5161682876288
0.4978203914945	0.5162563022517
0.4979000319881	0.5163710956687
0.4979776031571	0.5164110169237
0.4980761230475	0.5167033866475
0.4981654222964	0.5167720780727
0.4982646186183	0.5168149799264
0.4983347939336	0.5170154667288

0.4984279662175	0.5170569103108
0.4985163243936	0.5171303000464
0.4986059149701	0.5171624813031
0.4986971535787	0.5171767912428
0.4988067573968	0.5173230912169
0.4988935057199	0.5175650740304
0.4989978485159	0.5175693881567
0.4990850171411	0.517736259644
0.4991567542708	0.5178118225319
0.4992357831588	0.517961191157
0.4993156926374	0.5180191444393
0.4994079399476	0.5181318264239
0.4995051659817	0.518239664286
0.4996025672844	0.5183789197982
0.4996880557655	0.5184567531405
0.4997778262464	0.5190589176068
0.4998782672821	0.5191660064879
0.4999831516269	0.5192685139332
0.5000815387701	0.5193668132119
0.50015648991	0.5193672947798
0.5002464425567	0.5196478581245
0.5003391784244	0.5197339887407
0.5004378263143	0.519788381387
0.500533488189	0.5198087976086
0.5006281159536	0.5198175375838
0.5007080632773	0.5198717495225
0.5007915506215	0.5200508929376
0.5008840332909	0.5201910384193
0.5009872527731	0.5202109293641
0.5010850453433	0.5203598592307
0.5011785276133	0.5205580619492
0.5012651271589	0.5207422327557
0.5013645776918	0.5207555574625
0.5014548776913	0.5209262681025
0.5015616143638	0.5209400036894
0.5016512004317	0.5211779942627
0.5017333350402	0.5211969367577
0.501835455133	0.5215514892054
0.5019390353513	0.521569049287
0.5020338605956	0.5222308798765
0.5021401842609	0.5222567663671
0.5022456720113	0.5222578539448
0.5023421962176	0.5222846865011
0.502413412542	0.5223542199103
0.5025291513496	0.5226432534232
0.502610973002	0.5226770755764
0.5027035948181	0.5226990854935
0.5027899042334	0.5230155535029
0.5028790947485	0.5230173658324
0.5029709887334	0.5230743780351
0.5030923154703	0.5231313580627
0.5031773366349	0.5231393828505
0.5032830918363	0.5231656325481
0.5033759132127	0.5232048066039

0.5034496183017	0.523404462514
0.5035585385419	0.5235070028156
0.5036497230759	0.5235369132732
0.5037419073613	0.5236478545542
0.5038476837647	0.5237068043398
0.5039421909246	0.5238255948293
0.5040241596736	0.523854840392
0.504138180582	0.5238979809038
0.5042275762035	0.5240642459428
0.5043304105376	0.5241332898373
0.5044110273896	0.5242337584468
0.5044814368969	0.5242538656501
0.5045755906147	0.5243295337219
0.5046724206305	0.5243472201867
0.504762699799	0.5244418249111
0.5048635781255	0.524550592501
0.5049479655691	0.5246877714507
0.5050464659319	0.5247808434987
0.5051255039694	0.5248059335706
0.5052132062647	0.5250197421719
0.5053097310846	0.5250545850447
0.5054035400567	0.5250674670702
0.5055165183579	0.5254400045625
0.505601658134	0.5254681864519
0.5056989519807	0.5255700967987
0.5057945709738	0.525670362979
0.5058946963982	0.5256754933849
0.5059938487943	0.5257028837853
0.5060762329055	0.5258943510963
0.5061778465639	0.5259274848414
0.5062840715584	0.5260485947246
0.5063698273073	0.526073357009
0.5064671852788	0.5261274036342
0.5065637804074	0.5261309519362
0.5066686304304	0.5261349481485
0.5067537489825	0.5263570179263
0.5068634246775	0.5263648852067
0.5069775664159	0.5264475944853
0.5070667045741	0.5265897215636
0.5071757430552	0.5266969319793
0.5072710058268	0.5267000557731
0.5073852543533	0.5267109037554
0.5074546070251	0.5267264262013
0.5075449234869	0.5267299431392
0.5076399456853	0.5268945232888
0.5077380620802	0.5269061666737
0.5078248753377	0.5271756798725
0.5079165715624	0.5273868655225
0.5080242272682	0.527801613868
0.5081150323731	0.5278555897035
0.5082233467579	0.5282258496499
0.5083175226752	0.5283607368076
0.5083952907071	0.5284054779628
0.508488448343	0.528466632939

0.5085805644232	0.5284675999538
0.5086771225962	0.5285388296507
0.5087681937259	0.5286271393429
0.5088700198394	0.5286449674667
0.5089613077226	0.5287462137999
0.509052964772	0.5288263648702
0.5091535527368	0.5288562202201
0.5092503526373	0.5289090603878
0.5093464287616	0.52891307385
0.5094309168261	0.5290568468469
0.5095357905885	0.5292713795962
0.5096230587056	0.5294300584564
0.5097011335846	0.5294973236276
0.5097818641732	0.5295725974783
0.5098636653798	0.5295872797405
0.5099424665832	0.5296251111553
0.5100153147412	0.5299528960898
0.5100929518522	0.5307405605502
0.5101931833216	0.5307571682271
0.510300831518	0.5308801469342
0.5103954959769	0.5309093628514
0.510497206091	0.5309686750388
0.5105906514299	0.531127386257
0.5106951252823	0.5312642976804
0.5107934713691	0.5312917373511
0.5108787540949	0.5313099549018
0.5109589065177	0.5314664388351
0.5110553688923	0.5320601667335
0.5111409711637	0.5321667681505
0.5112282679559	0.5321776673952
0.5113268319237	0.5324606108151
0.5114178084421	0.5324945561825
0.5115169284751	0.5327450733331
0.5116028534516	0.5329569942425
0.5116894422173	0.5331705036641
0.511783533697	0.5332740329457
0.5118805342526	0.5336014719004
0.5119708028144	0.5336798485677
0.5120593474881	0.5337174008819
0.5121430915505	0.534246612846
0.5122351293983	0.5344421988288
0.5123338655963	0.5344583047761
0.5124216749437	0.5345952908069
0.5124997084227	0.5346265094156
0.5125849239801	0.5347887141818
0.5126714080049	0.534868731891
0.5127758501581	0.5349496147175
0.5128755548984	0.5349647423282
0.5129638048291	0.5350351791994
0.5130541781513	0.5351557550928
0.513129125066	0.5351674071142
0.5132150163138	0.5352312337531
0.5133346899529	0.5353559478913
0.5134258909956	0.5356603122865

0.5135221634841	0.5357626816367
0.5136080167719	0.535925133208
0.5136890669096	0.5359683335319
0.5137900885562	0.5360777640001
0.5138945197627	0.53612599946
0.5139890375779	0.5361399898525
0.5140824750616	0.5362212760342
0.5141763130056	0.536369994196
0.5142645617449	0.536578208186
0.5143714960709	0.5366058663221
0.5144739286162	0.5366210638399
0.5145590514309	0.5367519148197
0.5146474097974	0.5367655941236
0.5147315201296	0.5368140391928
0.5148142052989	0.5368517908783
0.51489840525	0.5368971064152
0.5149933294204	0.5373425301639
0.5150792094824	0.5377056054172
0.5151563076007	0.5377666949309
0.5152508206694	0.5379316674308
0.5153357930594	0.5380027224999
0.5154205802868	0.538219355747
0.5155286261336	0.5383197006513
0.5156235976388	0.5383564361117
0.5157345891807	0.5383985951421
0.5158232936095	0.5384175364331
0.5159211196558	0.5384403161192
0.5160190177016	0.5387085819509
0.5161062070354	0.5387494941787
0.5161907833041	0.5387665654948
0.5162815431375	0.5388858606727
0.5163665641298	0.5389346941378
0.5164637005281	0.539106240149
0.5165720303125	0.5392135855956
0.5166504482466	0.5394942520491
0.5167500768098	0.5398140781322
0.5168526525503	0.5398500224035
0.5169464464274	0.5399402154596
0.517034533332	0.5400370240061
0.5171266446903	0.5401611574849
0.5172030483395	0.540255016214
0.5172818408905	0.5403275099073
0.5173763360231	0.5406295608577
0.5174582393627	0.5406327426028
0.5175733763035	0.5406492291989
0.5176662149235	0.5407468441494
0.5177497876585	0.5407810538468
0.5178391616895	0.5411293936402
0.5179364688238	0.5412077033844
0.5180353394969	0.5413663839389
0.5181318780109	0.5413868029493
0.5182233530304	0.5414500924672
0.5183188568139	0.5416750522493
0.5184088217777	0.5418358009053

0.5184942670632	0.5419559748656
0.5185972695856	0.5421701383185
0.518682732034	0.5426436123371
0.5187897126414	0.5427138317476
0.5188832859188	0.5427546776239
0.518964792251	0.5427772544236
0.5190707290844	0.5427815799352
0.5191523097965	0.543091610527
0.5192428489918	0.5434238766388
0.5193522166518	0.5436951581639
0.5194279401233	0.5440323646259
0.5195199545711	0.5440549957234
0.5196256025504	0.544193464271
0.5197062993685	0.5443120390625
0.5197880502576	0.5443183108919
0.5198690442195	0.5444456917931
0.5199516062709	0.5444507339056
0.5200240659918	0.5445215603941
0.520121704914	0.5445741648311
0.52020175573	0.5448039342826
0.5202952514951	0.5449263001896
0.5204040204831	0.5451947616574
0.5205082800957	0.5452468066362
0.5206054268798	0.545440535239
0.5206915399418	0.5454924205768
0.5207866092677	0.5455713824906
0.5208724128437	0.5457927260486
0.5209706190239	0.545904601213
0.5210800780788	0.5459594062426
0.5211861997377	0.5461004015391
0.5212757880311	0.5462769411713
0.5213673257706	0.5465088179659
0.5214648807059	0.5466652127335
0.5215524700384	0.5466939041998
0.521642317212	0.5468347549786
0.5217336416014	0.5468666901898
0.5218360513411	0.5468841926414
0.5219362520678	0.5472326232187
0.5220211145327	0.5472562983659
0.5221095704456	0.5472722511125
0.5222085192744	0.5473024547499
0.5222844203161	0.5474307271956
0.5223648237732	0.5475807228018
0.5224778850992	0.547594579245
0.5225702285757	0.5475986119767
0.5226706033362	0.5476973682037
0.5227846650271	0.5477474367638
0.5228772573746	0.5477514105781
0.5229611138311	0.5478189543195
0.5230478862505	0.5480310683975
0.5231320265518	0.5481538656855
0.5232220007266	0.5482011529851
0.523297339486	0.5482871268364
0.5233998802294	0.5485247986213

0.5234963588063	0.5486418983718
0.52358226909	0.5486513781155
0.5236853660224	0.548845087419
0.5237761523011	0.5488919071661
0.5238672673223	0.548948364578
0.5239610145377	0.5489663821949
0.5240590258063	0.5492527823318
0.5241526676102	0.5493375647047
0.5242533558494	0.549373569915
0.5243447660773	0.5493977060047
0.5244258067677	0.5495109571379
0.5245213628975	0.5495163419297
0.5246129451482	0.5496325814553
0.5247011183356	0.5496788166129
0.5248214720728	0.5497109482542
0.5249174643791	0.5502910652584
0.5250148397386	0.5503643324111
0.5251030906832	0.5505103948139
0.5252142227461	0.5506221570188
0.5253091998223	0.5507347448981
0.5254183563617	0.550993919462
0.5255043070716	0.5510500299569
0.5256043619292	0.5510669551679
0.525702663583	0.5510684375078
0.525772846661	0.5510973294476
0.5258677144408	0.5513831072373
0.5259457347712	0.5514077819173
0.5260576791143	0.5515330818609
0.5261471507078	0.5516855533805
0.5262300643321	0.5518209090142
0.5263308769602	0.5518538374622
0.5264412359558	0.5523114465168
0.5265132313078	0.5525331016134
0.5265915358473	0.5526808698822
0.5266867486547	0.5528366706695
0.526777645721	0.5528373778206
0.5268845987791	0.5528547307558
0.5269929675364	0.5530507619892
0.5270781179233	0.553166930727
0.5271674409763	0.5531858064237
0.5272752946426	0.5533461604627
0.5273575138383	0.5534971580274
0.5274577554286	0.5535951662214
0.5275504945212	0.5536018421914
0.5276467647176	0.5537860114065
0.5277212639515	0.5537919517436
0.5277860059574	0.5541068846902
0.5279039496134	0.5543405260842
0.528010610605	0.5543630171104
0.52810839885	0.5545324542251
0.5282027281784	0.5545568234706
0.5282975310694	0.5545636820137
0.5283846553088	0.554634784423
0.5284720690528	0.5546785955695

0.5285521521227	0.5547199243256
0.5286359779569	0.5549339110457
0.5287345674686	0.5551007264434
0.5288387128665	0.5552012560618
0.5289325623303	0.5552459213083
0.5290162919298	0.5552624560008
0.5290876891839	0.5555597831324
0.5291659357688	0.5555967069906
0.5292651173824	0.5556346428337
0.5293504056234	0.5556633574055
0.5294564608477	0.5557289805468
0.5295484209103	0.5557645351662
0.529653177686	0.5558706966414
0.5297381923447	0.5560500519292
0.5298259956872	0.5562268021484
0.5299185933751	0.557000771913
0.5300046650163	0.5570178221187
0.5300919231722	0.5571405063626
0.5301753829811	0.5571763826048
0.5302726796856	0.5573484332752
0.5303729345872	0.5574202813627
0.5304634651727	0.5574990375224
0.5305473519884	0.5575006375998
0.530632026126	0.5575613228819
0.530712174871	0.5576563271471
0.5308103494906	0.5578452856759
0.5309222031312	0.5579622221491
0.5310289875313	0.5581265940441
0.5311171344145	0.558213469985
0.5312274321362	0.5582278209651
0.531314750991	0.5585391331785
0.5313912694438	0.5585573037062
0.5314854764776	0.5586125091699
0.5315793541795	0.5586315258289
0.5316726361606	0.5588738516344
0.5317679853323	0.5589601100973
0.5318567942254	0.5589989146241
0.531925790407	0.5590927421479
0.5320085670929	0.5593413794394
0.5321034949331	0.5594404323352
0.5322027786036	0.5601150831679
0.5322917977551	0.5602241925429
0.532386796886	0.5604215403932
0.5324631470486	0.5604694061477
0.5325425923132	0.5607651478439
0.5326388565782	0.560977602599
0.5327474760026	0.5611758861244
0.5328486608334	0.5612432955718
0.5329284548921	0.5612445286732
0.5329998060226	0.5612648957113
0.5330971530592	0.5614304751096
0.5332097130941	0.5614992943746
0.5333104753356	0.5615347270976
0.5334042362469	0.561692818593

0.5334870286741	0.5621698936858
0.533572097666	0.5622561736554
0.5336635751313	0.562330808876
0.5337652534651	0.5625524808322
0.5338583742952	0.5626556085589
0.5339461322651	0.5626557761489
0.5340390582976	0.562852627438
0.5341310461568	0.5628780250572
0.5342250097239	0.5629198987824
0.5343130353757	0.562943108874
0.5344177279649	0.5629587963822
0.5345123290577	0.5629624974718
0.5346101004334	0.5630080185978
0.534694570253	0.5631387476094
0.5348102508616	0.5631801141372
0.534910255232	0.5634102856717
0.5350040097573	0.5634215104568
0.5351202616045	0.563669477817
0.5352269754773	0.5637290318591
0.5353174095206	0.5641589608213
0.5353932558325	0.564357597561
0.535489906862	0.5643611610195
0.5355828048492	0.5645727671863
0.5356686963232	0.5646298898589
0.535777498535	0.5646433338809
0.5358807719576	0.5646596763068
0.535982740924	0.5647238088471
0.5360614009496	0.5648075173481
0.5361607915567	0.5648121994881
0.5362558293876	0.5648535929317
0.5363473322288	0.5651396855683
0.5364279119359	0.5652393730231
0.5365209040659	0.5652593530148
0.5366164798496	0.5652635958274
0.5367017543903	0.565284525639
0.5367962543371	0.5652976468627
0.5368763566397	0.5654151123232
0.536968126738	0.5654410646004
0.5370547006924	0.5654756420161
0.5371319935742	0.5655179291442
0.5372194884735	0.5656812048208
0.5373217612828	0.565742805949
0.5374227315411	0.5658376034688
0.5375086380243	0.5658725342661
0.5375878391291	0.566113621389
0.5376751357985	0.566337757935
0.5377651631619	0.5663560125592
0.5378563032089	0.5663591078265
0.5379579711559	0.5664678177342
0.5380613576084	0.5665053066421
0.5381480677178	0.5666455608883
0.5382173783616	0.5666611121978
0.5383081437737	0.5668150817795
0.5383876223628	0.5669003759896

0.538476730778	0.5670743352986
0.5385611907289	0.5671337601101
0.5386439668936	0.5675693623673
0.538736364812	0.5681296611688
0.5388326074061	0.5682649933592
0.5389307558462	0.5684222526856
0.539004143465	0.5686983264899
0.5390846351166	0.5688874682223
0.5391753907018	0.5692077388532
0.5392834596239	0.5693172102109
0.5393676799901	0.5694815181363
0.5394683358811	0.5695275453618
0.5395651135467	0.5696450139902
0.5396540146816	0.5697326707423
0.5397659464401	0.5697991009379
0.539848790983	0.5698663059888
0.5399246491615	0.5699966654073
0.5400325884014	0.5700582935492
0.5401302544193	0.5701062470405
0.540227156137	0.5704480631271
0.5403327475159	0.5707971888579
0.5404391216135	0.570979212114
0.5405253941119	0.5710450538293
0.5406024968239	0.5710616545565
0.5407060487757	0.5711141349979
0.5408037517206	0.5711588904203
0.5408836434355	0.5712628506148
0.5409792095317	0.5714617622719
0.5410939855759	0.5714885464965
0.5411945687234	0.5716508781923
0.541305917077	0.5716914481216
0.541390817757	0.5718983391225
0.5414722914976	0.5722771327173
0.5415639459211	0.5722826702851
0.5416670228208	0.5723671882742
0.5417765745949	0.5724431813714
0.5418701698377	0.5728213699736
0.5419611342232	0.5729352369877
0.5420453591796	0.5734020121212
0.542149949382	0.5734287704721
0.5422539385884	0.5736014843723
0.5423316681426	0.5736286260118
0.5424471521886	0.5740243082828
0.5425217228205	0.5740705448399
0.5425937671688	0.5740909351678
0.5426929545326	0.574310888028
0.5427981868073	0.5744801735185
0.5428868022207	0.5746164051565
0.5429749631341	0.5747175730015
0.5430578094397	0.574792376155
0.5431578810751	0.575362323678
0.5432457299428	0.5755229152594
0.5433433712809	0.575652365166
0.5434424141081	0.5756768880481

0.5435361612093	0.5757350017752
0.5436224965417	0.5757686649699
0.5437269363015	0.575768805761
0.543832274147	0.5758580429139
0.5439571698006	0.5759666338979
0.5440450940307	0.5759887293549
0.5441387641737	0.5760526496298
0.5442476493448	0.5760640692437
0.5443405903158	0.5761637597184
0.5444248442571	0.5761927755559
0.5445065726135	0.5763611956424
0.5445945059474	0.576400348097
0.5446950001992	0.5765053000699
0.5447839677212	0.5770408250954
0.5448629959119	0.5770833073324
0.5449611769032	0.5771512059691
0.5450517168845	0.5772722121772
0.5451326105304	0.5773577193755
0.5452182014017	0.5773625316973
0.5453144227229	0.5773665063914
0.5454090609564	0.5774011570838
0.5454918930722	0.5774092475135
0.5455889966916	0.5775208365389
0.5456840960284	0.5780968060872
0.5457769350328	0.5781568486191
0.545867675727	0.5783080049245
0.5459591571274	0.5783118875425
0.5460625600132	0.5783719216737
0.5461613887551	0.5784150132532
0.5462713148907	0.5785107040779
0.546364291522	0.5786434244593
0.5464611017927	0.5786610339069
0.5465530335376	0.5790567473832
0.5466400002005	0.5793798019817
0.5467330339606	0.5794797012255
0.5468508208531	0.5796071920031
0.5469334828822	0.5798129867276
0.5470374403183	0.5798157582392
0.547115268447	0.5798343837129
0.5472006816851	0.5800512604435
0.5473027941022	0.5800703257014
0.5474073232283	0.5804093305681
0.5475018526589	0.5804292256517
0.5475868414716	0.5806699099689
0.5476792382932	0.5812733578918
0.5477607526388	0.5817927504469
0.5478537052744	0.5818373788529
0.5479430439154	0.5819165616661
0.5480515521868	0.5826232873547
0.5481450372325	0.5826728158786
0.5482409116999	0.5827691703161
0.5483436608252	0.5828226198115
0.5484283593078	0.58293490733
0.5485275595796	0.5830567187764

0.5486192276111	0.5831446090197
0.5487139944869	0.5832995546838
0.5488071532116	0.5835314448496
0.548902855766	0.5835751518881
0.5489893941883	0.5837726658864
0.5490744821247	0.5839677183277
0.5491582560876	0.5841809041567
0.5492514164976	0.5846994650777
0.5493564421034	0.5847362838235
0.5494410102405	0.585013681329
0.54953801227	0.5850268942568
0.5496343252136	0.5851327008817
0.5497184913809	0.5852052587114
0.5498099548242	0.5852562475337
0.5499027823422	0.5855134916305
0.5499812299608	0.5856415645408
0.5500824018067	0.5857195397984
0.5501648166378	0.58588961079
0.5502638253135	0.5859485321728
0.5503440937763	0.5859508064692
0.5504340668474	0.5860492421762
0.5505392307321	0.5861022512651
0.5506289491625	0.5861332752378
0.550727580225	0.5861552192761
0.5508245378225	0.5861958168263
0.5509370110749	0.5862146816983
0.5510295910939	0.5863759370097
0.5511105575893	0.5864927090536
0.5511975222384	0.5868343024314
0.5512756399232	0.5868393551107
0.5513634328706	0.5870002767067
0.5514691143418	0.5872534708795
0.5515457761585	0.5874166644307
0.5516456997252	0.5874552156602
0.551725432082	0.5874658882937
0.5518307869146	0.5877428403429
0.5519103770248	0.5878031893525
0.5519866501069	0.5878071561048
0.552102370308	0.587850907305
0.5521976020316	0.587890494987
0.552296567262	0.5879656421754
0.5523879999595	0.5880171503217
0.5524767681855	0.5880783008982
0.5525848603793	0.5881528029535
0.5526764074767	0.5882069650044
0.5527433905151	0.5884201290587
0.5528248222879	0.5886133124305
0.5529365633328	0.5886887793832
0.5530279761761	0.5887706811219
0.5531304511384	0.5888960515532
0.5532405216055	0.5892848091974
0.5533331274431	0.5892867628482
0.5534460370651	0.5895454664909
0.5535412410632	0.5896301513365

0.5536619384383	0.5898942008381
0.553745037861	0.5899010824285
0.5538332933632	0.5900452812259
0.5539350795332	0.5910001393802
0.5540312480771	0.5910564642948
0.5541075304046	0.5910760769194
0.5541892159379	0.5911390535975
0.5542740162117	0.591198240009
0.5543665152767	0.5914307806297
0.554448312182	0.5916334616913
0.5545525398948	0.5919349049313
0.5546631377245	0.5919910242035
0.5547594526031	0.5923239356502
0.5548710318149	0.5925904630985
0.5549592224639	0.5926847272125
0.5550510420071	0.5927184302987
0.5551581511362	0.5928398132081
0.5552662045963	0.5929852007366
0.5553789923016	0.5929893405758
0.5554834982777	0.5931242290372
0.5555852373131	0.5931663265888
0.55566807883	0.5932554589423
0.5557868883516	0.5933329560236
0.5558763417285	0.5937406870364
0.5559779344729	0.5937969342989
0.5560780425194	0.5940469645111
0.5561743739885	0.5940750462078
0.5562601689478	0.5941622568351
0.5563698390899	0.5942871406873
0.5564768619209	0.5943839185529
0.5565621249425	0.5944925817678
0.5566603205356	0.5946641617151
0.5567486933185	0.5946768725727
0.5568362189151	0.5947414133588
0.5569344785439	0.5950187001345
0.5570484771994	0.5951413036802
0.5571492651909	0.5952708210833
0.5572313009756	0.5952795994937
0.5573216988467	0.5952915241458
0.5574125308148	0.5956705716406
0.5575222083237	0.5957531812011
0.5576402956426	0.5957610442879
0.5577392094264	0.5958068937619
0.5578162476755	0.5958408912561
0.5578966351806	0.595965690986
0.5580066846364	0.596095173916
0.5580984262127	0.596488475555
0.5582128308574	0.5964918296965
0.558309029376	0.5967126469465
0.5584076400183	0.5970200150419
0.5584886412102	0.5972419149666
0.5585861904299	0.597309321052
0.5586917982505	0.5973339092854
0.5587909000131	0.5973735370241

0.5589119534864	0.5974240326871
0.5590054974079	0.5975127755485
0.5591016713391	0.5976005665795
0.5591869801348	0.5976702284894
0.5592734759468	0.5978184035844
0.5593712530235	0.5978227599462
0.5594633703388	0.5978994066476
0.559563675083	0.5980260154248
0.5596977558417	0.5980361880397
0.5598083750453	0.5988496739657
0.5598978358534	0.5989529832977
0.55999135263	0.5990465668647
0.5600857615351	0.5993028561161
0.5601736973887	0.5994914275971
0.5602642276905	0.5998484680385
0.5603788072544	0.6001580311834
0.5604631384112	0.6002766729075
0.5605706553759	0.6004269240911
0.5606641195542	0.6004347047816
0.5607596655687	0.6004577047243
0.560846660213	0.6005497450016
0.5609330752059	0.6007149918821
0.5610241379479	0.6008578514739
0.56111115728	0.6010516620405
0.5611901285555	0.6013822112356
0.5612978471013	0.6017465285323
0.5613926756233	0.6017747776756
0.5614601200773	0.6018738296454
0.5615633271277	0.6019421114411
0.5616332018314	0.6019548464755
0.561741777171	0.6021166764229
0.5618379821672	0.6022613521154
0.5619206590443	0.6023459050491
0.5620164328062	0.6024317386407
0.5621013576781	0.602603239138
0.562203697628	0.6031304146743
0.562316466475	0.6031662042401
0.5624045697715	0.6032159110866
0.5624882552997	0.6036700913237
0.562584932104	0.6037485106174
0.5626707081176	0.6041104897648
0.5627581711538	0.6047277543496
0.5628349219109	0.6048325171753
0.5629142897385	0.6049290851526
0.5630111051335	0.6050248063924
0.563108371345	0.60506913573
0.5632076642032	0.6052140874246
0.5633003437355	0.6052497048573
0.5633829414726	0.6052869758129
0.5635022273152	0.6058317873716
0.5636068080165	0.6060440903765
0.5637036989355	0.6061137798832
0.563796583865	0.6063096184043
0.56390308592	0.6063198381761

0.563982305669	0.6063456471598
0.564071243543	0.6063513025008
0.5641608263347	0.6064822778735
0.5642617230114	0.6067485656068
0.564354766253	0.6070467995887
0.5644456269385	0.607163610124
0.564526646369	0.6072344249194
0.5646093842445	0.6074252843457
0.5647019239084	0.6074870156893
0.5647838143177	0.6075020896554
0.5648782387113	0.6075480093592
0.5649537292674	0.6081858377023
0.5650345238021	0.6082977759256
0.5651273189187	0.6084367425907
0.5652316727119	0.6088254406259
0.5653236782123	0.6088676458039
0.5654054607445	0.6089554165958
0.5655100747757	0.6091350767132
0.5655991639328	0.6091396877816
0.5656933447075	0.6091724718943
0.5657930884862	0.6093118234428
0.5659078116817	0.6093756485109
0.5660019809844	0.6094525124723
0.5660883075765	0.6094887347847
0.5661871279488	0.6095477673831
0.5662651346952	0.6097524708939
0.5663515699315	0.6099813347705
0.5664343209753	0.6100496372678
0.5665253668588	0.6100553761681
0.566602522787	0.610060444691
0.5667046689427	0.6106045325803
0.5667915276947	0.6110184760544
0.5668821200484	0.6110319901757
0.5669875817685	0.611110230756
0.5670793280636	0.6112751602961
0.5671617617758	0.6115392134613
0.5672566785449	0.6116981051623
0.5673522110556	0.6117775450205
0.5674545137878	0.6118401319006
0.5675698505016	0.6121394834544
0.5676663024241	0.6123156918411
0.5677343586813	0.612364237023
0.5678280968329	0.6123865841302
0.5679335144153	0.6123900224551
0.5680387430502	0.6124558448995
0.5681290576726	0.6126343908504
0.5682213706739	0.6126656606207
0.568317884879	0.6127602083678
0.5684112227878	0.6127968619796
0.5685175537816	0.6128148036234
0.5685971917914	0.6130191416512
0.568679805327	0.6131147922466
0.5687657475885	0.613231221006
0.5688570640533	0.6134511457575

0.568936913927	0.6136865508504
0.5690257913125	0.6136898334234
0.5691342502034	0.6137410235126
0.5692223477114	0.6138175438149
0.5693299826327	0.6138250508849
0.5694128860927	0.6143287473024
0.5695109565486	0.6144107820388
0.5696120803814	0.6144294566913
0.5697068122846	0.6145004911435
0.5697958574423	0.6145185727586
0.5698988562543	0.614675091337
0.5699978014525	0.6146844137936
0.5700775095077	0.614689469047
0.570185879797	0.6149105270103
0.5702841240927	0.6150032044287
0.5703729965691	0.6150774896466
0.5704647274517	0.6150978912864
0.570559422231	0.6151476269349
0.5706655071825	0.6152442238174
0.5707594534107	0.6154206176472
0.5708379167758	0.6155131069243
0.5709181936409	0.6157383920341
0.5710211459893	0.6160682711601
0.5711190017073	0.6162496562174
0.5712077037193	0.6163082997469
0.5713024503943	0.6163394563989
0.5713965137259	0.6165886542004
0.5714758225955	0.6166739577522
0.5715570598235	0.6166962319005
0.5716517948042	0.6167127115384
0.5717395995459	0.6167501401256
0.5718414568522	0.6168884849198
0.5719518130833	0.6173348868113
0.5720340850883	0.6173422639625
0.5721203434719	0.6175598623341
0.572219107554	0.6175930242182
0.5722989202616	0.6176162742112
0.5723779055378	0.6179112369681
0.5724627499978	0.6180722337202
0.572562502992	0.6180774899105
0.5726595187472	0.6182257980873
0.5727463757918	0.6184693030187
0.5728434045938	0.6185174523581
0.5729571922112	0.6185297588502
0.573047815647	0.6185521080378
0.5731475637165	0.6191024830985
0.5732257941917	0.6191469056822
0.5733238784702	0.6192025218952
0.5734195084754	0.6192118080107
0.5735087146212	0.619332798264
0.5735940082915	0.6198377037767
0.5736844754372	0.6199082968994
0.5737874456851	0.6199179819521
0.5738798094879	0.6199227025754

0.5739750181931	0.620255641046
0.5740722852546	0.6205032264437
0.5741605285701	0.6205229166589
0.5742404453308	0.6205252747824
0.5743391144885	0.6205353199851
0.5744297838444	0.6209257053874
0.5745282412832	0.6211884374441
0.5746101610862	0.6212597690492
0.5746854767659	0.6213454609707
0.5747828925194	0.6215362919944
0.5748689273332	0.6224858272207
0.5749624328448	0.6225102918154
0.5750529438265	0.6225349810269
0.5751549314695	0.6226750895028
0.5752358612095	0.6227678311099
0.5753263852187	0.6229998774536
0.575417011999	0.6231548693665
0.5755030799841	0.6231683870858
0.5755865245886	0.623323895016
0.5756783255416	0.6233443647579
0.5757544900145	0.6236451922544
0.5758501099968	0.6237510243265
0.5759630198676	0.6237940393533
0.5760644176985	0.6238712297953
0.5761571013964	0.6239611426568
0.5762771175129	0.6241314411098
0.5763759683778	0.6241584027589
0.5764723419013	0.6242551865446
0.5765541355504	0.624510253384
0.5766403636589	0.6246708848307
0.5767571414216	0.6247009070058
0.5768506597144	0.6249610689338
0.5769613267076	0.6249832746414
0.577061230369	0.6249893199498
0.5771390883551	0.6250463373681
0.5772296342316	0.6252688457593
0.5773243042429	0.6253277197373
0.5774116216828	0.6254154208353
0.5774936443405	0.6254516772818
0.5775842471095	0.6256045341362
0.5776916906684	0.6256135006392
0.5777910275836	0.6257263444627
0.5778583315596	0.6258015963127
0.5779315464836	0.6259425543059
0.5780429391983	0.6260203762506
0.5781413278495	0.6260510455139
0.5782451301429	0.6261082264298
0.5783408811281	0.62613574569
0.5784505046142	0.6263211866282
0.5785560254723	0.6263722527059
0.5786432123706	0.6266824029184
0.578747172877	0.6268038640045
0.5788315753831	0.626871567431
0.5789138929614	0.6269593114889

0.5789926851759	0.6269689693456
0.5790812773359	0.6270999232254
0.5791709040101	0.6271935281914
0.5792558933883	0.6273885099403
0.5793451715632	0.6274967032381
0.5794441627321	0.6275645200826
0.579538719126	0.6276233660574
0.5796424431008	0.6277386899747
0.5797231226293	0.6278662679489
0.5798185240211	0.6279827715328
0.5799065463038	0.628109559153
0.579995052647	0.6282962438558
0.5800931622022	0.6286535572331
0.5801726709377	0.6287867722327
0.5802776071621	0.6288655919927
0.5803673686598	0.6292069307444
0.5804560515506	0.6292749320061
0.5805594414877	0.6293060599884
0.5806495880865	0.6293965386919
0.5807458875913	0.6296295380473
0.5808498543372	0.6299148048316
0.5809282252257	0.6300997383748
0.5810033903003	0.6301074243085
0.5811029903708	0.630348679703
0.5811902590325	0.6308094125033
0.5812604220778	0.6308697062324
0.5813532155012	0.6309224358742
0.5814679294099	0.6309451342772
0.5815667232955	0.6309534715323
0.5816657503669	0.6309600863331
0.5817773820713	0.6310710057063
0.581872251715	0.6312136371386
0.5819566871509	0.6313134977359
0.5820413591333	0.6314160995644
0.5821315550791	0.6314986607956
0.5822344327295	0.6315833850036
0.5823360111225	0.6316348963043
0.5824155397363	0.6316496636571
0.5825100689349	0.6317570268103
0.5826113387183	0.632142105804
0.5827004294567	0.6325105492411
0.5827950654348	0.6326398457402
0.5828825831513	0.6327683554229
0.5829801878676	0.6329757285111
0.5830736924905	0.6330498389177
0.5831634605384	0.6330581990735
0.5832654479052	0.6331478817558
0.5833516136254	0.6331495590472
0.5834432234941	0.633376181519
0.5835327712681	0.6333839432195
0.5836025681308	0.633526571276
0.5836932417799	0.6336479035214
0.5838010320923	0.6339382001217
0.5838976799513	0.6340263282858

0.5839957643087	0.6344812902116
0.5840905810496	0.6345167023868
0.5841716417758	0.6345234355004
0.584256567067	0.6345433794134
0.5843522195481	0.6346522484698
0.5844356713531	0.6346892786099
0.5845254937401	0.6350044314179
0.5846009110719	0.6350478334083
0.5846981527568	0.6350950759364
0.5847986121603	0.6351681695757
0.5848909221058	0.6352525878588
0.5849908453145	0.6355029178775
0.5851013276929	0.6356286934081
0.5852090528385	0.635662760769
0.5853096513059	0.6357447936819
0.585399584689	0.6357599506546
0.585508523111	0.6358304387367
0.5856027969784	0.6359414125456
0.5857079171556	0.6359824916762
0.5857953503586	0.6360488331065
0.5858789686247	0.636264625627
0.585979196612	0.6363637654824
0.5860777143368	0.6363922794068
0.5861769051531	0.6368174515933
0.5862766055233	0.6369038649583
0.5863697499821	0.6370012580294
0.5864618937396	0.6370030012622
0.586566235137	0.6370879299174
0.5866576709683	0.6374381648254
0.5867492344739	0.6378528809265
0.5868519116442	0.6380729985584
0.5869533138937	0.6382070456626
0.5870461407317	0.6382675907145
0.5871360525753	0.6384231548445
0.587228635782	0.6384517206316
0.5873215964223	0.6385582691947
0.5874153778178	0.6387071615792
0.5875026522842	0.6387103989553
0.5875984419659	0.6387576865518
0.5876804851212	0.6387649258411
0.5877682906241	0.6387931320933
0.5878460211441	0.6390444683878
0.5879522979974	0.6390619242495
0.5880409889683	0.6391004140103
0.5881274460617	0.6393377200154
0.5882270668527	0.6393440959489
0.5883032921597	0.6393694552231
0.5883992408929	0.6394406959683
0.588502011127	0.6398588966501
0.5885922523644	0.6399203331204
0.5886911632631	0.6399498466723
0.5887980658064	0.6400414710748
0.5888849162706	0.6403559544825
0.5889793475537	0.6404767696213

0.589061757971	0.6408082906729
0.5891602576372	0.6409538115864
0.58925867863	0.6416136707676
0.5893408017035	0.6418894168378
0.589421633747	0.6419048817269
0.589516843342	0.6419611271262
0.5896366953989	0.6422048823252
0.5897180621076	0.6422300514905
0.5898040216527	0.6422407505548
0.5898832018855	0.642272215817
0.5899705833546	0.6424460582351
0.5900591318609	0.6428192433455
0.5901427027918	0.6432518086165
0.5902207623515	0.6434763698644
0.5903234341565	0.6436574252361
0.590395412605	0.6436772264837
0.5904861800298	0.6438665120035
0.5905987582528	0.6438714069129
0.5906869151001	0.6438881586916
0.5907663551193	0.6438953806791
0.5908584630899	0.6440605988267
0.5909416346768	0.6442652418996
0.5910489241565	0.6444743482809
0.5911454302656	0.6447048621354
0.5912526228784	0.6447662769289
0.5913411394564	0.6447947973113
0.591438477943	0.6448040399744
0.5915343848332	0.6451724057591
0.5916331114361	0.6452218039995
0.591735981877	0.6453463094067
0.5918219458722	0.6457153230426
0.591910476317	0.6459335403833
0.592005189996	0.6461019469022
0.5920863093121	0.6463205607359
0.5921803634533	0.6465240926168
0.5922801971569	0.6466144357165
0.5923834741028	0.6466363123136
0.5924737876037	0.6467370444919
0.5925596571013	0.6467383916003
0.5926609211482	0.647066951264
0.5927683773321	0.6471144802042
0.5928791379574	0.6471959281125
0.5929731476435	0.647234407168
0.5930641322612	0.6474311121289
0.5931870807959	0.6475011609266
0.5932797919203	0.6475440339203
0.5933902486335	0.647747108555
0.5934722258561	0.6477481828832
0.5935639146377	0.6480204257197
0.5936470072962	0.6482334289582
0.593740787686	0.6482401539707
0.59384399144	0.6485618558764
0.5939342078244	0.6487496764925
0.5940392437533	0.6488517783133

0.5941290545011	0.6488708747921
0.594208824341	0.6489814118513
0.5943280258	0.6490160338232
0.5944041034316	0.6496037214228
0.5944867215545	0.6496054717058
0.5945883964138	0.6496083695102
0.5946932181918	0.6496576675004
0.5947778443845	0.6497038056512
0.5948705117325	0.649818124903
0.594958328149	0.6498902122401
0.5950610852121	0.6499705637187
0.595167164588	0.6503776701609
0.5952710304937	0.6505715357442
0.5953506269069	0.6509554529254
0.5954461992665	0.6511787625036
0.5955596351246	0.6518053853249
0.595673102245	0.6518815980732
0.5957784716733	0.651932012855
0.5958978664868	0.6520184831069
0.5959721798469	0.6520599472848
0.5960478113826	0.652202653662
0.596128676045	0.6522427237065
0.5962210387635	0.6524054048837
0.5963148666603	0.6526495186902
0.5964165500192	0.6527503601047
0.5965192450642	0.6528291402256
0.5966064021987	0.6528988872559
0.5966856011753	0.6529560930157
0.5967766476446	0.6530636023955
0.5968707421336	0.6532958892279
0.596967711542	0.6533472861627
0.5970638164992	0.6534766969021
0.597144047565	0.6535081791161
0.5972477860616	0.653658352577
0.597338238685	0.6538711864165
0.5974631709167	0.6542341794661
0.597589439096	0.6545306956758
0.5976751396616	0.6550138938324
0.5977642972919	0.6550528812244
0.5978567374445	0.6551349779493
0.5979567179396	0.6552513181253
0.598060225021	0.6553157041032
0.598148994699	0.6555985212792
0.5982559759365	0.6556861052271
0.5983527989926	0.6558407837236
0.598453715809	0.6558428882289
0.5985436814107	0.6559230798157
0.5986394327302	0.6559259014194
0.5987386960285	0.6560971030534
0.5988183046908	0.6562030906956
0.5989048098287	0.6563862427404
0.5989894888439	0.6564768924294
0.5990750530281	0.6564859640962
0.5991811265585	0.6565072149215

0.5992615153312	0.656576710724
0.5993434360578	0.6565909213345
0.5994351695411	0.6567395343524
0.5995280041444	0.6569037094344
0.5996150485704	0.6569704001477
0.599696857872	0.6571012109174
0.5997724942734	0.65720509118
0.5998904409248	0.6572091345553
0.5999722399738	0.6573834641962
0.6000730124978	0.6575859403729
0.6001697354552	0.6581942624717
0.6002653974257	0.6585958281489
0.6003635611061	0.6586325706078
0.6004565618616	0.6587718815975
0.6005550086742	0.6588609939257
0.6006604696527	0.6589366120185
0.6007571830055	0.6591002653643
0.6008525673501	0.6591229595923
0.6009579033716	0.6591610917846
0.6010507904864	0.659281086369
0.6011625700967	0.659300013229
0.6012651378337	0.6593770598885
0.6013539608424	0.6596064961749
0.6014447212306	0.6598526523627
0.6015247955414	0.6599766218353
0.6016143882245	0.6599844470061
0.6016965469765	0.6600744029368
0.6017997805656	0.660115473269
0.6018860212679	0.660181778028
0.601963053542	0.6601988539677
0.6020651818718	0.6602359457068
0.6021807202261	0.660265006903
0.6022782246145	0.6607370352157
0.6023571541604	0.6608711843371
0.6024508703816	0.6609498234424
0.6025627178471	0.660950865828
0.6026529027983	0.6609752720044
0.6027455460038	0.6611130076357
0.6028530798611	0.6611693198702
0.6029619619079	0.661289534939
0.6030509036232	0.6614238728678
0.6031354101215	0.6614839239099
0.6032374672534	0.6615592711599
0.6033302665603	0.6615759746402
0.6034285844152	0.6616138612976
0.6035084322102	0.6616582813136
0.6035874531559	0.6617373476774
0.6036796176294	0.6617497541962
0.6037612916528	0.6619515766943
0.6038605182624	0.6621900209648
0.6039604211443	0.6623528547287
0.604069007178	0.6623688826157
0.6041719952402	0.6624128265347
0.6042542596462	0.6624240647793

0.6043538641677	0.6624624898435
0.6044489492856	0.6624887623183
0.6045275717068	0.662702755362
0.6046165378836	0.6628038644221
0.6047301662816	0.662842900801
0.6048180404877	0.662886745668
0.6049026833776	0.662965303285
0.6049826145685	0.6629998311819
0.6050854606871	0.6631878833149
0.6051855915213	0.6632033399872
0.6052757599686	0.6636285473527
0.6053597033208	0.663638158089
0.6054573551589	0.6637290719207
0.6055521666799	0.6637957613841
0.60565594454	0.6638067046397
0.6057433366382	0.6638083673513
0.6058284723974	0.6639850796722
0.6059332187418	0.6642212877152
0.6060282800386	0.664629501374
0.6061167965303	0.6646582649646
0.6062108330266	0.6647424005125
0.6063143384499	0.6649591063479
0.6064254114252	0.6649914666475
0.6065186386183	0.6651015696533
0.6066088656481	0.6652985751929
0.6067058928644	0.6653341921531
0.6068032628618	0.6653455185244
0.6069104945502	0.6654539741532
0.6069925930723	0.6654730593244
0.6071064617576	0.6655045795656
0.6071929917726	0.6655052409258
0.6072978597745	0.6655747230117
0.6073992121696	0.6656157438599
0.607479108451	0.6656289554133
0.6075925438009	0.6656393397103
0.6076836280021	0.6656737681006
0.6077799281631	0.6659941744382
0.6078871438288	0.6660153623551
0.6079916905812	0.6660901492817
0.6080948106706	0.6662533897802
0.6081937236523	0.666672330024
0.6082986090475	0.6666948794125
0.6083807942093	0.6668376805615
0.6084494164797	0.6670536789873
0.6085365902588	0.66707340788
0.6086196356649	0.6671155303344
0.6087081266267	0.6671155421191
0.6087970431489	0.6672842118433
0.6088822245338	0.6673691262883
0.6089611797545	0.6675061547274
0.6090389317997	0.6676298324844
0.6091422887568	0.6678620635474
0.60923465814	0.6679126654678
0.6093241881308	0.6679632361633

0.6093992857103	0.6679720404603
0.609504792628	0.6680369652
0.6095986543172	0.6681284905637
0.6096802018215	0.6682660029551
0.6097938158075	0.6685416452505
0.6098934906683	0.6685488515075
0.6099875199491	0.6686079480018
0.6100673343549	0.6686273181683
0.6101522503231	0.6687103138155
0.6102284571209	0.6687948886791
0.6103383104058	0.6688334731496
0.6104260040551	0.6688974577859
0.6105245984778	0.6689902546277
0.6106002891086	0.6694314833705
0.6107044966085	0.6697242852554
0.6108050158936	0.6698106926932
0.6108889076581	0.6698644967417
0.6109783956986	0.6698795853075
0.6110910672627	0.6698806182721
0.6111674048673	0.6700108864814
0.6112683730151	0.6700206958
0.6113613088528	0.6700791676307
0.6114568021392	0.6700993283376
0.6115398445795	0.6701175771903
0.6116423260616	0.6702511873381
0.6117512811288	0.6703653171469
0.611849529454	0.6704337066504
0.6119316032433	0.6705351506138
0.6120540702067	0.6707456680289
0.6121618582392	0.6708079863457
0.6122540365952	0.670834358258
0.6123753263373	0.671263145001
0.6124757376952	0.6713316504142
0.6125846140126	0.6714216666939
0.6126805372238	0.6716275997769
0.612788493313	0.6718569214203
0.6128926060641	0.6718631974769
0.6129890897683	0.6719052881635
0.6130997295899	0.67191290954
0.6131991074587	0.6723101131533
0.6133025443013	0.6723235354778
0.6134020964152	0.6724591150307
0.6134925700512	0.672546123815
0.6135935908789	0.6725671222955
0.6136814499118	0.6726615801865
0.613775594054	0.6726846091888
0.6138737388712	0.6731103667884
0.6139594473914	0.6733117733689
0.6140428854086	0.6735253032053
0.6141334793803	0.6735391185691
0.6142261476015	0.6735807788192
0.6143253095065	0.6735885591811
0.614409510585	0.6735920547774
0.6145025335765	0.673597323205

0.6146044292827	0.6736719307143
0.6146839767477	0.6738872416615
0.6147818429264	0.6739386088765
0.6148734771828	0.6740026600103
0.6149763995145	0.6740202239425
0.6150626737977	0.6740834998194
0.6151503878049	0.6741311890816
0.6152582080501	0.6742644176541
0.6153476040519	0.6742671349912
0.6154407499765	0.6742751698456
0.6155442892813	0.6745119949587
0.6156462072971	0.6746810958508
0.615761336017	0.674730323454
0.6158395170998	0.6750245730985
0.6159335738102	0.6752596122443
0.6160293821476	0.6752655744816
0.6161167140884	0.6753633621113
0.6162303678085	0.6753913695565
0.6163177251405	0.6757403884702
0.6164282097513	0.6757999911958
0.6165191978259	0.675843189996
0.6166201472126	0.6758705735699
0.6167064226343	0.6759776481859
0.6167950877176	0.675978189384
0.6169004397238	0.6761258187943
0.6169930346258	0.6761333652387
0.617088915829	0.6762336528829
0.6171855180166	0.676240487808
0.6172662626675	0.6764310812222
0.617367682603	0.6765879163172
0.6174602986108	0.6766376002212
0.6175685208468	0.6767813100185
0.6176557550908	0.6769419236117
0.6177535302773	0.676956451889
0.6178448039689	0.6770465694787
0.6179345188454	0.6772159604896
0.6180263063758	0.6774372439754
0.6181231180096	0.6774664431679
0.6182383245332	0.67747770939
0.6183389481426	0.6774907337247
0.6184444926566	0.6775802653225
0.6185331537076	0.67763489957
0.6186573784532	0.6777682459009
0.6187603492406	0.6777838546713
0.61885903142	0.6780367817749
0.6189384640383	0.6780728682311
0.6190250930912	0.6782626221918
0.6191250936658	0.6783148301861
0.6192125575862	0.678355113376
0.619302306698	0.6784105359019
0.6193879564507	0.6784949536926
0.6194762453222	0.679021369658
0.6195647050374	0.6790515576959
0.6196498064069	0.6791406900818

0.6197442050047	0.6792863555404
0.6198378739211	0.679453914219
0.6199267970441	0.6801027924433
0.6200166216268	0.6804060478845
0.6201247386304	0.6805748679046
0.6202125853692	0.6809919584216
0.6203118888764	0.6811777839006
0.6204380121112	0.6814535041855
0.6205262147393	0.6816103765871
0.6206213944209	0.6816587078334
0.620727860556	0.6816713753547
0.6208219498809	0.681779523886
0.6209157212	0.6820294362453
0.6209872247443	0.6821158740836
0.62109690423	0.6822415507766
0.6211939446417	0.6824341339774
0.6212875457487	0.6827566893659
0.6214014500371	0.682994394046
0.621513833799	0.6832461060446
0.6216161392882	0.6832464574753
0.6217099290322	0.6832816789214
0.6218006660908	0.683298685073
0.6218876158549	0.6833582763598
0.6219784516086	0.6835951574392
0.6220863481335	0.6836730879638
0.6221894587724	0.6836740324221
0.6222930227918	0.6837861803491
0.6223855674439	0.684237845456
0.6224807642383	0.6843893900207
0.6225642038709	0.6846120843232
0.6226515642116	0.6849419581535
0.622744302249	0.6850405080735
0.6228560726425	0.6851529866874
0.6229586072646	0.6851540389939
0.6230855205636	0.6852216300716
0.6231897012235	0.6853165164198
0.623277860287	0.6853325193426
0.6233755118509	0.6857387450653
0.6234731209712	0.6858106799768
0.6235547403647	0.6861346181602
0.6236559148519	0.6861914187657
0.623748798936	0.6862854694988
0.623851345584	0.6863959146472
0.6239675805628	0.6866696668116
0.6240832867926	0.6867592171127
0.6241659169797	0.6869430859767
0.6242636032169	0.6870112669946
0.6243774986282	0.6870562060688
0.6244606194738	0.6871419645537
0.6245343789103	0.6874673871543
0.6246319671345	0.6878257763959
0.6247423136528	0.6878408248621
0.624840126766	0.6879535907096
0.6249391932674	0.6880549657957

0.6250331994986	0.6880588218088
0.6251203780754	0.6880667032872
0.6252159425389	0.6882629533245
0.625321022267	0.6886084835524
0.6254035466756	0.6888612346843
0.6255167240629	0.6889119000634
0.625620863817	0.6890089631011
0.6257107693829	0.6890375398729
0.6258037380713	0.6892302292841
0.6259098262325	0.6892467886767
0.6260077518562	0.6894878177225
0.6261165355311	0.6895177222998
0.6262288476407	0.6896045264088
0.6263404897725	0.6896308738082
0.626441565061	0.6897201492873
0.6265375001204	0.6900072707126
0.6266328220349	0.690030253978
0.6267509708267	0.690034506836
0.6268651234519	0.6900667845437
0.6269672712898	0.6900864806594
0.6270747886987	0.6904662894836
0.627173598594	0.6909550258762
0.6272683456579	0.6910075033459
0.6273700861783	0.6911163172406
0.6274689100546	0.6911922360432
0.6275637776849	0.6912253399511
0.6276524307472	0.6912296454641
0.6277538790386	0.6913092166662
0.6278552659197	0.6913275616309
0.6279443612345	0.6916975203905
0.6280483830381	0.6919406309395
0.6281647271978	0.692073006794
0.6282687229541	0.6921506427604
0.628351450696	0.6921711506441
0.6284319542871	0.6922704587181
0.628516350574	0.6922767535773
0.6286149966989	0.6924368385594
0.6287231322351	0.6924590226795
0.6288201109652	0.6927247741358
0.6289189361521	0.6927500344802
0.6290207567602	0.6927975804606
0.629141913937	0.6928561757069
0.6292353699587	0.6928637894813
0.6293409242178	0.6929101822833
0.6294602396514	0.6929332399105
0.6295451086163	0.693071588029
0.629635785002	0.6932399787529
0.6297330676622	0.6936510845467
0.6298266547881	0.6939898399624
0.6299043655698	0.6940011523055
0.6299882415331	0.6941625688569
0.630077821513	0.6944005310563
0.6301623307659	0.6944699742839
0.6302774482718	0.6945075902787

0.6303943878725	0.6946154441607
0.6304933875072	0.694624321628
0.630588736328	0.6946247543743
0.6306860775087	0.6947482055256
0.6307826448668	0.6948426745261
0.6308861550196	0.6949285649881
0.6309675828455	0.6950729432171
0.6310582861868	0.6950791431955
0.6311523072061	0.6951402991646
0.6312455965649	0.6952968405622
0.6313448944755	0.6955242996163
0.631430359298	0.6955964859106
0.6315203958014	0.6956399143427
0.6316265178502	0.6957803313842
0.6317234127097	0.6959011852908
0.6318181664762	0.695968132182
0.6319057936015	0.696012457701
0.6320236750777	0.6960553422846
0.6321144304967	0.6961243433807
0.6322242528896	0.6962169593873
0.6323511154035	0.6962507316864
0.6324657830536	0.6964382465715
0.6325517111462	0.6965190683498
0.632656842953	0.6966408103252
0.6327554205813	0.6967391666554
0.6328369544693	0.6968154983141
0.6329531772257	0.6968867448398
0.6330375638582	0.697602755267
0.633137203449	0.6976948355775
0.6332383658296	0.6978975688474
0.6333370173234	0.6979135188284
0.6334294474882	0.6979173230054
0.6335200895024	0.6981859615399
0.6336033213785	0.6984326932543
0.6336884974998	0.6985337437569
0.6337885149537	0.6985588501537
0.6338738877849	0.6986228444884
0.6339749297821	0.6986519857182
0.6340557582771	0.6989461983934
0.6341623661856	0.6990119622785
0.6342506338273	0.6990953293934
0.634342248004	0.6994565370282
0.6344437969686	0.6994855979819
0.6345483335369	0.6998197554213
0.6346522669963	0.6999188654653
0.6347533920947	0.699938187031
0.6348646139853	0.7000019464289
0.6349500867477	0.7001029879158
0.6350540411759	0.700132744809
0.635154373059	0.7005266704938
0.6352535709844	0.7005713702456
0.6353516701463	0.7008298465013
0.6354521126769	0.7008957967164
0.6355313557544	0.7009020436097

0.6356103952986	0.70096253047
0.635698693564	0.7010761321098
0.6357912204872	0.7011920924154
0.635876463902	0.7012649440929
0.6359589087082	0.7013364993688
0.636049403076	0.7013767340013
0.636144311409	0.7013771207704
0.6362376521209	0.7015224942909
0.636323486809	0.701532300235
0.6364169702837	0.7018308236062
0.6365132390747	0.7019071627407
0.6365992384018	0.7023120726487
0.6366943014102	0.7025987847201
0.6368025210451	0.7026178564988
0.6369019144631	0.7027634504731
0.6370071995424	0.7028766681101
0.6371064823688	0.7031831331519
0.6372102858398	0.7033368047931
0.6372891027387	0.7033606275976
0.6373866853212	0.7042248464415
0.63748197895	0.7045417367817
0.6375664789787	0.7048172743897
0.637658786375	0.7050253350723
0.6377351934084	0.7051498094139
0.6378367613424	0.7051550907213
0.6379232221713	0.7052847299715
0.6380337349127	0.705504963104
0.6381272918592	0.7055749510855
0.638213362751	0.7056946986021
0.6383032006119	0.7057155067114
0.6384060760664	0.7059200560693
0.6384978840076	0.7061580036063
0.6385774268522	0.7062769069533
0.6386591352983	0.706412774671
0.638779683541	0.7064176571336
0.6388622430153	0.7065019187533
0.638965767319	0.7065292210079
0.6390481821857	0.7066672970326
0.6391845383833	0.7066697299344
0.6392721423773	0.7067350714638
0.6393642974617	0.7067640831111
0.6394634809991	0.7068631007217
0.639551133061	0.7069019191343
0.6396497525971	0.7069450818833
0.6397443648944	0.7070385817846
0.6398367375911	0.7070494429208
0.6399142318684	0.7073663637777
0.6399933349105	0.7073680139386
0.6400744767762	0.7077629843415
0.640167780891	0.7077963631154
0.6402741796639	0.7078728835271
0.6403575483967	0.7080634993546
0.6404615064636	0.7084288633032
0.6405587452054	0.7086345477008

0.6406471315093	0.7086700522059
0.6407463343906	0.708898546525
0.6408365563675	0.7089248135725
0.6409178237039	0.7090630594098
0.6410068576644	0.7092029917382
0.6411070345803	0.7092452577159
0.6412109072985	0.7094112490818
0.6413151970699	0.7094757181667
0.6414110561097	0.7096256233326
0.6414946912437	0.7096685813417
0.6415919132023	0.71018546857
0.6416927373216	0.7102172330467
0.6418055919981	0.7104411563374
0.6419119380702	0.7107164072988
0.642031611685	0.7111779821716
0.6421349425696	0.7114935595856
0.6422391819862	0.7114939321427
0.6423491175459	0.7115165438845
0.6424545976838	0.7122726417873
0.6425423016815	0.712274603204
0.6426510857555	0.7122908710812
0.6427569402538	0.7123403087484
0.6428621930108	0.7125762308199
0.6429450562395	0.7129904214982
0.6430379682706	0.7131438615823
0.6431404058321	0.7134393450744
0.643249985982	0.7135780433179
0.6433361553581	0.7135916210078
0.6434423241109	0.7135988280064
0.6435647342901	0.7136035834558
0.6436589887155	0.7137077008266
0.6437585739545	0.713793902206
0.6438708113652	0.7140528295846
0.6439549206002	0.7140703087256
0.6440499106564	0.7143116201825
0.644157560937	0.7143220061932
0.6442381612321	0.71453506187
0.6443451590824	0.71470050952
0.6444409324322	0.7147111181978
0.6445365634737	0.7147200666021
0.6446249591843	0.7152996932775
0.644716292561	0.7155331601949
0.6448022359111	0.7155731113931
0.644882359929	0.7155771416596
0.6449748977415	0.7156946565875
0.6450685317842	0.7157237809697
0.6451636688072	0.715740043922
0.6452671033893	0.7159655986745
0.6453654918521	0.7159806802547
0.6454607734306	0.7161096730056
0.6455404822486	0.7161903150948
0.6456501094076	0.7163285964326
0.645736926554	0.7165437598274
0.6458133373792	0.7167085288398

0.6459190419914	0.7167285679462
0.6460334946042	0.7171406826671
0.6461473503632	0.7172235966143
0.6462290202861	0.7173736221363
0.6463233351801	0.7175497134065
0.6464174982269	0.7175651200183
0.6465160796306	0.7176768255716
0.6466017419289	0.7178276724418
0.6466957882558	0.718527704302
0.6467983721773	0.7186392742921
0.6468923215459	0.7189157573825
0.6469980124894	0.7190762995756
0.647104493872	0.7191241800795
0.6472010117879	0.7191783161068
0.6472903648838	0.7191898437129
0.6473735768691	0.7192164810472
0.6474686102519	0.7192462096637
0.6475689574677	0.7192801049053
0.6476632370039	0.7194568748292
0.6477661545263	0.7194645275446
0.647879455102	0.7194718168165
0.6479637458038	0.719494125069
0.6480570286117	0.7197391074357
0.6481468052071	0.7197487302936
0.6482753220543	0.7199054924404
0.6483741612473	0.7199533138642
0.6484754686421	0.7200341573794
0.6485535548053	0.720073654118
0.6486606826349	0.720206967159
0.6487442776945	0.7202400267289
0.6488367626295	0.7203040703359
0.6489262910774	0.7203889822534
0.6490213589166	0.7205625507978
0.6491058825975	0.7208475399496
0.6491957464389	0.7208717102099
0.6492860276518	0.7210725860257
0.6493772371163	0.7212011741213
0.6494770456878	0.7213157396169
0.6495810813565	0.7214770428319
0.6496878967966	0.7215676439475
0.6497712746467	0.7216267955395
0.6498674150871	0.7217447166986
0.6499714648345	0.7218355787149
0.650064588814	0.7218730071687
0.6501657351686	0.7219840707554
0.650271324161	0.7223485650635
0.6503739470709	0.7224233562572
0.650478041727	0.7227199537933
0.6505610126331	0.7229773557202
0.6506595377185	0.7230179768576
0.6507515591966	0.723040925393
0.6508561887214	0.7233442183964
0.6509506652463	0.7236327133274
0.6510319500835	0.7238892489516

0.651116640296	0.723930293071
0.6512030497753	0.7239985401544
0.6512865851853	0.7241018093036
0.6513838505057	0.7246919438347
0.6514691783216	0.7250226681202
0.6515585489531	0.7253660841249
0.6516695588898	0.7254175595976
0.6517656935769	0.7255022897187
0.651878319192	0.7256977541971
0.6519585116878	0.7259065064801
0.652056294963	0.7262587130093
0.6521462423373	0.726467750728
0.6522579940435	0.7270247350913
0.6523574217947	0.7270965239905
0.6524467463033	0.7271938806577
0.6525423729386	0.7272819510366
0.6526346208316	0.7273245016521
0.6527204777154	0.7273523235505
0.6528330668903	0.7274759149418
0.6529342027685	0.7274855809596
0.6530335023117	0.7275304348811
0.6531332974387	0.7276972459001
0.6532369065473	0.727701786959
0.6533608488664	0.7278069936604
0.6534569890745	0.7287511780749
0.6535626343866	0.7289211805809
0.6536611410447	0.7296782226079
0.6537588784361	0.7296956048643
0.6538426842169	0.7298374696434
0.6539354562262	0.7299617111651
0.6540294354594	0.7301208112704
0.6541448985573	0.7304392707978
0.6542469457937	0.7304746543142
0.6543518349836	0.7306519462686
0.6544336269328	0.7306534801899
0.6545224273745	0.7308245272595
0.6546246679501	0.7309100054167
0.6546958637307	0.730971664803
0.6547935438475	0.7311217800411
0.6548944287514	0.7311605297212
0.6549656135782	0.7314507153167
0.6550657258926	0.7314565201363
0.6551474386644	0.7316809158003
0.65522840317	0.7317310801756
0.6553445569089	0.7318904374463
0.655448289417	0.7319038514995
0.6555475510582	0.7319500417651
0.6556390667954	0.7321455855639
0.6557529265383	0.732183724791
0.6558627265085	0.732486624411
0.6559471228694	0.732910452622
0.6560389924967	0.7332496910627
0.6561384470511	0.7333975756458
0.6562269629889	0.7334351890572

0.6563260972022	0.7336123920685
0.6564206970567	0.7338021868911
0.6565151752097	0.733829152308
0.6566201488569	0.7340810454996
0.6567140599488	0.7346725062308
0.656819075222	0.7351812854716
0.6569248139132	0.7353489499115
0.6570320613445	0.7354681610683
0.6571198003853	0.7355388918021
0.6572146004626	0.7355654013099
0.6573088528421	0.7356009524167
0.6573969985236	0.7356816592921
0.6574739007215	0.7361206910608
0.6575709056367	0.7362454812742
0.6576964835335	0.736384628331
0.6578112445135	0.7364423300074
0.6579196044259	0.7365092975917
0.6580252020324	0.7367126893908
0.6581166131494	0.7369317866873
0.6582290882009	0.7373082020885
0.658349367986	0.737313702294
0.6584489084349	0.7373270418693
0.6585526947889	0.7374629183601
0.6586483590028	0.737554303717
0.6587384665118	0.7376993431859
0.6588296198504	0.7378530746062
0.6589031572935	0.737861621692
0.6590307907339	0.7379805498961
0.6591230640312	0.73803269699
0.6592027491386	0.7381627649764
0.6592886207018	0.73857884846
0.6594023381628	0.7385840138103
0.6594965707383	0.7390686394419
0.6596048442572	0.7391030028664
0.659706854577	0.7394452188459
0.6598300336032	0.7394658680531
0.6599498530557	0.7396406677055
0.6600620701519	0.7396899274191
0.6601614030451	0.7396916085156
0.6602766385145	0.7398767965171
0.6603720210526	0.7399798593808
0.6604588986554	0.7400692244411
0.6605605322866	0.7403597275002
0.6606546984859	0.7405236385488
0.6607618575403	0.7407698681924
0.6608611181254	0.7411432805372
0.6609453429569	0.7411987937117
0.6610406711208	0.7413780910773
0.661139196144	0.7413831148839
0.6612396351722	0.7415461260033
0.6613264994444	0.74158477128
0.6614102015068	0.7416769925064
0.6615185624633	0.7416774986371
0.6616090450195	0.7416932276432

0.6617327349814	0.7418268892533
0.6618232786171	0.742107534064
0.6619123874911	0.7421612166164
0.6620174392969	0.7426211743568
0.6621229798505	0.742634711948
0.6622233481026	0.7427643245846
0.6623252772629	0.7428903322678
0.6624156545309	0.7429799154606
0.6625383928483	0.7429992858066
0.6626505211716	0.7431103023244
0.662741046398	0.7431181535308
0.6628355588517	0.7431539402041
0.6629314002553	0.7434243003285
0.6630227338163	0.7440820620079
0.6631115426991	0.7441883458317
0.6632147371412	0.7444072435972
0.6633037797655	0.7444381485879
0.6634283188124	0.7447629312182
0.6635258210226	0.7451465694505
0.6636343470327	0.7453479946955
0.6637305653462	0.7453969610331
0.6638306052969	0.7453983561941
0.6639169261993	0.7455542947484
0.664019757129	0.745608467692
0.6641050822221	0.7457800694066
0.6642110138821	0.7458062799314
0.6642922975104	0.7458890274292
0.6643809496025	0.7460764993874
0.6644792186514	0.746090814403
0.6645700169555	0.7460937221445
0.664663859074	0.7464491835076
0.6647477172841	0.7464652904491
0.6648498594401	0.7467208286563
0.66492884788	0.7473895367315
0.6650091784115	0.7475495706956
0.6651051091725	0.7477266776259
0.6652123118207	0.7477921114014
0.6653226406	0.7480277207063
0.6654201275808	0.7486537388663
0.6655216225505	0.7488890067901
0.6656137604568	0.7489632784223
0.665712081811	0.7491809266769
0.6658196207643	0.7494774565687
0.6659030742337	0.7496324272376
0.6659799936207	0.7498736528583
0.6660693089481	0.7499262809951
0.6661522521512	0.7503269439412
0.6662663923815	0.7504193831491
0.6663523240328	0.7504840665306
0.6664538687244	0.7505072744219
0.6665650502784	0.7506163412441
0.6666569932136	0.7509781111361
0.6667368878411	0.7510479055831
0.6668324806423	0.751065249701

0.6669494883872	0.751209727483
0.6670572607453	0.751381891696
0.6671666612787	0.7514589903973
0.6672722308625	0.7514692958894
0.6673553043073	0.7517187133308
0.6674669042829	0.7520086499245
0.6675639424906	0.752229770027
0.6676525784159	0.7522982351309
0.6677430591998	0.7523100136212
0.6678379680335	0.7523522117802
0.6679484749687	0.7524832131756
0.668052103447	0.7527795416866
0.6681394478961	0.7529421096555
0.6682362044319	0.7529452754796
0.6683284506322	0.7529731058883
0.6684269138799	0.7529757983443
0.6685142108744	0.7529813018458
0.6686304273309	0.7531351431311
0.6687322426659	0.7532170258506
0.6688298418705	0.7533297796288
0.6689242213517	0.7535468038283
0.6690186602872	0.754017171501
0.6691373060207	0.7541060876154
0.6692314343148	0.7541137469141
0.6693302226252	0.7543119002272
0.6694167410821	0.754367750787
0.6695051382221	0.7543847541369
0.6696114166841	0.7544023919375
0.6697091157184	0.7545032395442
0.6698050271453	0.7545592479144
0.6699124020966	0.7545951201768
0.670023287133	0.7546128053282
0.6701226273037	0.7546501648514
0.6702196282676	0.7547818335563
0.6703127703087	0.7548676063229
0.6704164572823	0.7548788971185
0.6705075788246	0.7550465362198
0.6705996179272	0.7552735557068
0.6706750699237	0.7555610753516
0.6707550704934	0.7556524607452
0.6708581569432	0.7557869933063
0.6709314584142	0.7558071715307
0.6710340901056	0.7559259717562
0.6711118902636	0.7560727394945
0.6712055254973	0.7561990400494
0.6713093807103	0.7565933029578
0.6714122690697	0.757112384472
0.6714963743909	0.7574718284575
0.671599014443	0.7576583815233
0.6717211772977	0.7577566217605
0.6718310662451	0.7577926105419
0.6719432365871	0.7578389457953
0.6720353167432	0.7578878807313
0.6721301562766	0.757915776177

0.6722273660345	0.7579519638833
0.6723170085428	0.7586115724667
0.6724040692542	0.7586356341805
0.6724934355504	0.7589400417203
0.6726006993376	0.7590014558172
0.6727227310428	0.7590202291155
0.6728221740785	0.7590329465556
0.6729256894839	0.7593868168413
0.6730034154757	0.7593896401358
0.6730825987922	0.7594352073682
0.6731817688352	0.7596609435172
0.6732751495274	0.7596798607292
0.6733742052515	0.759760044283
0.6734750664396	0.7598386467623
0.6735799707513	0.7602255637294
0.6736770240517	0.7602588977573
0.6737632568521	0.7615875421783
0.6738638401588	0.7615886089776
0.6739609763292	0.7616048934553
0.6740721622114	0.7618933287444
0.6741553298273	0.7619075755762
0.6742510050482	0.7621487437374
0.6743596051233	0.7622997807435
0.674470560779	0.7623397067823
0.6745601027112	0.7624267338178
0.6746423707021	0.7624823830537
0.67474289171	0.7624892866706
0.6748436164524	0.7627213345523
0.6749372173715	0.7627978150107
0.6750197493076	0.7630961857609
0.6751123786856	0.7633522939938
0.6751892858903	0.7636528803093
0.6752681071199	0.7638016672732
0.6753585213916	0.7639166033982
0.6754510626062	0.7639506704817
0.6755517106015	0.76410894057
0.6756475652519	0.7642520797324
0.6757492571228	0.7643962180229
0.6758619206434	0.7645505704328
0.6759605686008	0.7645752867228
0.6760520643935	0.7646683102211
0.6761676986895	0.7651986637055
0.6762653704305	0.7652181130204
0.6763559920981	0.7654506033571
0.6764555225231	0.7655561670093
0.6765315761414	0.7655709851045
0.6766240808626	0.7657479615369
0.6767270862618	0.7663726657455
0.676810964305	0.7664617797387
0.676926257441	0.7667234844295
0.6770458225585	0.7671056176731
0.6771795854354	0.7671457491036
0.677284698147	0.767193682236
0.6773910949033	0.7673550535558

0.6774884813835	0.7674087445759
0.677581461396	0.767493564925
0.6776716611107	0.7675238548869
0.6777597410154	0.7675501952503
0.6778526139522	0.7676779338001
0.6779547631241	0.7678662424151
0.6780442198014	0.7679056954659
0.6781637240355	0.7680435232685
0.6782662033066	0.7680650116656
0.6783574242077	0.7680686356281
0.6784577882422	0.7680969473252
0.6785574355422	0.7681701795983
0.6786496108263	0.768290460275
0.6787440966088	0.7683398846371
0.6788201266548	0.7695069711818
0.6789186970799	0.7695214233708
0.6790174491713	0.7695714969426
0.6791183909187	0.7696890162397
0.6792103812814	0.7697049234557
0.6793100010512	0.7697512475943
0.6794231927767	0.769993914195
0.6795111550433	0.7703849424236
0.6796106146519	0.7704089846518
0.6796896267913	0.7704744212104
0.6797822420012	0.7704944381995
0.6798954209701	0.7707291915403
0.6800186280268	0.7707439499853
0.6801169880526	0.77102835885
0.6802164687659	0.7711146750486
0.6802974455012	0.7712099289283
0.680407820617	0.7712550766228
0.6805089148935	0.7713356415758
0.6806105112508	0.7713819941071
0.6807259973374	0.7714343469841
0.6808100296495	0.7717444653781
0.6809138548891	0.7718863081865
0.6810351789412	0.771969063128
0.6811358278682	0.7720867470454
0.6812271281032	0.7725136469399
0.6813351488104	0.7726236020052
0.681462138583	0.7727383431737
0.6815576693759	0.7727654577658
0.6816495934011	0.7728556283556
0.6817494465885	0.7728674874504
0.6818603775012	0.7732370468344
0.6819536970742	0.773449569742
0.6820393582953	0.7740589543592
0.682142331333	0.7741811549064
0.6822657569254	0.7742627976123
0.6823408320231	0.7742793192467
0.6824288804714	0.7747679955749
0.6825337795194	0.7748063993142
0.6826529208809	0.7748343421244
0.6827584922163	0.774938362217

0.6828722232707	0.7752386011835
0.6829822125971	0.7754190375149
0.6830625571557	0.7754266237967
0.6831499244631	0.7754779187946
0.6832463757067	0.7756540364512
0.6833426865195	0.7758319720997
0.6834396764198	0.7759005740964
0.6835251892283	0.7759341593907
0.6836274937189	0.7762060753421
0.6837213309188	0.7762861332091
0.6838305653555	0.776299836081
0.6839109365522	0.7763233894948
0.6840244218445	0.7764336748001
0.6841239748237	0.7766549332098
0.6842149385338	0.7768539307205
0.6843157810298	0.7769807432082
0.6844261847509	0.7771385782093
0.6845324038757	0.7772187576696
0.6846240190769	0.7773321961325
0.6847135369859	0.7773681894201
0.6848028186321	0.7774037300404
0.6849023303776	0.7774721885622
0.6849988495437	0.7776239911035
0.6851063981522	0.7777891299676
0.6851906377253	0.7778941813728
0.6852927675387	0.7779950831484
0.6853857926795	0.7781891900119
0.6854775773284	0.7782622665812
0.6855782265212	0.7783408956101
0.6856856358337	0.778355708954
0.6857928340357	0.7783897776441
0.6858985354889	0.7784624523931
0.6860136620462	0.7786498266146
0.6861144896945	0.7786791909097
0.686222494807	0.7787178099706
0.6863027582201	0.7789257124944
0.6863904420247	0.7790092258329
0.6864755403929	0.7791479629796
0.6865876838891	0.7793366342438
0.6866896082773	0.7794976650544
0.6867926938049	0.7797698314593
0.6868766823861	0.7798189176928
0.6869952026559	0.7805483659418
0.6871082809673	0.7806287531418
0.6871893696294	0.7807981167625
0.6872909170331	0.7808319560164
0.687388221589	0.7808997746781
0.6875105584805	0.7809723015938
0.6876088700999	0.7811902281413
0.6876955823924	0.7812269280002
0.6877769933874	0.7813987874478
0.6878769679751	0.7818552876107
0.6879897233589	0.7819581128566
0.6880827020634	0.7822646006638

0.6881802536559	0.7823248663139
0.6882703640904	0.7827223215368
0.6883708697694	0.7828434467783
0.6884742141094	0.7832087837311
0.6885726260418	0.7832834943503
0.6886664143423	0.7832874411349
0.6887989037547	0.7834545293118
0.6889086714178	0.783625505288
0.6890069199471	0.7839622476757
0.6891257107658	0.7839872771274
0.6892308117686	0.7840278561275
0.6893106900088	0.7842760585614
0.6894010859652	0.7844181050359
0.6894946636028	0.7845082061615
0.6896016071497	0.7846375318994
0.6897216836542	0.7847288098943
0.6898302053149	0.7848976722915
0.6899411541635	0.7849233254095
0.6900210504509	0.7849303758377
0.6901106263906	0.7850603688468
0.6902052018759	0.7851071139919
0.6903071515623	0.7851399388288
0.6904018080143	0.7852779638352
0.6905101187527	0.7860540845734
0.6906065028887	0.7861406570681
0.6907038836888	0.7862393422996
0.6908089268968	0.7864016627726
0.690915361298	0.7864045324496
0.6910105518358	0.7864911471036
0.6911018286941	0.786557772326
0.6911944394966	0.7866327737652
0.6912823426104	0.7866569461455
0.6913955195619	0.7866935689852
0.6914939775297	0.7867426519776
0.6915776085737	0.7867489868072
0.6916596904626	0.7868208004249
0.6917648173667	0.7869557639013
0.6918811631864	0.7870528216053
0.6919931737069	0.7873525730936
0.6920866766097	0.7874456500003
0.6922174535695	0.7874966910247
0.6923137881508	0.787711475874
0.692413810689	0.7877134162489
0.6925359009529	0.78794772416
0.6926568315345	0.7879611847606
0.6927572289255	0.7880419311193
0.6928500538034	0.7881410518233
0.6929698459654	0.7881909273868
0.69309881291	0.7883039482917
0.6931889783733	0.7883351230804
0.6932994426334	0.7885992540456
0.693415422323	0.7887101892181
0.6935151605191	0.78873105293
0.6936262511705	0.7890041332216

0.6937102878639	0.7890172294379
0.6938100430146	0.7890470662112
0.6939079627227	0.7893199234133
0.6940205233969	0.7893317842865
0.6941117995049	0.7897264614901
0.6942224060484	0.7899407844931
0.6943130181088	0.7900322854644
0.6944065451311	0.7900528585574
0.6944954548608	0.7902660351225
0.6945811143167	0.7905112876666
0.6946589262589	0.7905909465723
0.694755888129	0.7907050468639
0.694856881604	0.7907684586177
0.6949447579776	0.790904279555
0.6950548189125	0.7910173138329
0.6951599364414	0.7910212359029
0.6952501486256	0.7910795789811
0.6953596276657	0.791081799463
0.6954435351781	0.791177218631
0.6955403586407	0.7912389351989
0.6956402062314	0.7912582628169
0.6957434412427	0.7913026032182
0.6958303878442	0.7914660266257
0.6959211169594	0.7915251315872
0.6960290929449	0.7915786939898
0.6961273998228	0.7916415237812
0.6962223327121	0.7919824041663
0.6963329255208	0.7920273488206
0.6964131125567	0.7920299427623
0.6965008840538	0.7922003979404
0.6966008474857	0.7922518443339
0.6967055280624	0.7923275702736
0.696800314892	0.7925178760448
0.6968968996019	0.7927452502397
0.696987742509	0.7929759402216
0.6970674891339	0.7932053401147
0.6971820894667	0.7933068571289
0.6973071361351	0.7935521642372
0.6974172607156	0.7935732469392
0.6975143050301	0.7938855883679
0.6975962526361	0.793934851107
0.6976828853053	0.7946556309705
0.6977930484479	0.7951328642937
0.6978916712188	0.7951434226144
0.6979888774574	0.7951448717192
0.6980908342306	0.7951510588005
0.6981932840275	0.7952514310136
0.6982875845248	0.7953225938347
0.6983891818228	0.7953476326494
0.6984853767202	0.7953684207248
0.6985787506147	0.795445506387
0.698681133696	0.7954698354351
0.6987846026902	0.795700652693
0.6988634034973	0.7958151722261

0.6989794295392	0.7958952310422
0.6990781575219	0.7959040888565
0.6991872055913	0.7959431952885
0.6992913001123	0.7961018960442
0.6993822172899	0.7961623665769
0.6994883596355	0.7962653625997
0.6996140781743	0.7963200703272
0.6996992767132	0.7964822776005
0.6998005559225	0.7965320461882
0.6998968183479	0.7966398830706
0.6999820902414	0.7967265960253
0.7000559739931	0.796806156689
0.7001677041209	0.7969951489087
0.700263736738	0.7970500653678
0.7003786340657	0.7972434865944
0.700458958927	0.7972556606928
0.7005654022953	0.7973918745446
0.7006646413224	0.7974695155291
0.7007747024517	0.7975409596722
0.7008471108411	0.7977579215508
0.7009631354883	0.7978209321905
0.7010630516662	0.797848816026
0.701170320629	0.7980401551929
0.7012734160469	0.7982102714598
0.7013761256202	0.7983954710055
0.7014832757938	0.7984945212546
0.701580960598	0.7985757398209
0.7016929181056	0.7987289478736
0.7018068029555	0.7988286297846
0.7019080780637	0.7993427975178
0.7020093688425	0.7995767009389
0.7021221381879	0.7997241432272
0.7022105003245	0.8000998050233
0.7023140740344	0.8001433532347
0.70242713449	0.8003057036255
0.7025353612174	0.8003438557789
0.7026539333004	0.8003519674717
0.7027451354952	0.8004212432265
0.702835536448	0.8005100891536
0.7029426092223	0.8005612252082
0.7030373804343	0.8008028403776
0.7031392455173	0.8011230869543
0.7032328699061	0.8014574885321
0.7033275216037	0.8015162948938
0.7034239793117	0.8015779319671
0.7035149520706	0.8021520414906
0.7036235450366	0.8022062824867
0.7037258312063	0.8022290301028
0.703822213094	0.8023848223663
0.7039195580362	0.8023945123398
0.704033097509	0.8024114763222
0.704151749686	0.8026832547226
0.7042433179581	0.8030258991088
0.7043805996663	0.8030375513309

0.7044754510841	0.8031453191517
0.7045743878253	0.8031976783734
0.7046955168517	0.8032998259339
0.7047686099183	0.8033465346986
0.704899983297	0.8038847316117
0.7050131030928	0.8039340543313
0.7051323633637	0.8041550560433
0.7052402342814	0.8043398899393
0.7053309582751	0.8044084668641
0.7054229711089	0.8046697716291
0.7055110553937	0.8049302470551
0.7056096623763	0.8049400092511
0.7057024535321	0.8049432459454
0.7057916182342	0.8049554665554
0.7058982144521	0.8049960736375
0.7059933082877	0.805291668828
0.7060885573308	0.8053067576134
0.7061884944942	0.8054735972772
0.7062784335801	0.8056959221845
0.7063808327362	0.8056959376734
0.7064667957314	0.8057277756853
0.7065633427866	0.8059985877448
0.706672635711	0.8060868720287
0.7067708764269	0.8061621547773
0.7068759991502	0.8064908582674
0.7069885504965	0.8066176594313
0.7070883907402	0.8066213962617
0.7071850990056	0.806728973459
0.7072912123375	0.806869669185
0.7073923952214	0.8069306582359
0.7075185840117	0.8069327055617
0.7076204936252	0.8070056511434
0.7077141092927	0.8071167384987
0.7078231906589	0.8071168803695
0.70793656334	0.8071770677647
0.7080477174178	0.8072639123614
0.7081306447773	0.8073395394032
0.7082360242326	0.8073522371938
0.7083504512774	0.807836632139
0.7084470107423	0.8079643906191
0.7085245335539	0.8079662161325
0.7086467640719	0.8081301320297
0.7087435174924	0.8081831762767
0.7088349331648	0.8081937031897
0.7089240989292	0.8084372790503
0.7090445749947	0.8085339573361
0.7091419709911	0.8085937429374
0.7092307340031	0.8087809868478
0.7093233143553	0.8090634098464
0.7094121262842	0.8093934145458
0.7095415675629	0.8094451174462
0.7096249442875	0.8099388899837
0.7097173962583	0.8100273699244
0.7098141815251	0.8101534719898

0.7099116927035	0.8104082348286
0.7100207587095	0.8105187457614
0.7101305053553	0.8108438260518
0.7102208206633	0.810889482826
0.7103265765162	0.8110845409538
0.7104322448263	0.8111642248346
0.7105308887489	0.8111663093336
0.7106339148782	0.811370846748
0.7107313584779	0.8113993528104
0.7108304256152	0.8115079548843
0.7109367385575	0.8115547955009
0.7110250777908	0.8117922094351
0.7111372329151	0.8117925373217
0.7112341109048	0.8120274702097
0.7113486183721	0.8120727170757
0.7114405756872	0.8120729510713
0.7115279950352	0.8121860556509
0.7116212422441	0.8122509535286
0.7117094086405	0.8122546455476
0.71180067066	0.8130601856673
0.7118844674994	0.813364490504
0.7119961802072	0.8134371673568
0.7120912411538	0.8137091802034
0.7121975266551	0.8139329888211
0.7123047902928	0.8140392568334
0.7124031011761	0.8140778743572
0.7125049696731	0.8140848999195
0.7125908151445	0.8140856828941
0.7126761168093	0.814346164921
0.7127898697015	0.8148684882784
0.7128783038538	0.8149721495278
0.7129882772856	0.8150559581353
0.7130833078519	0.8156073150385
0.7131854631679	0.8156886924528
0.7132890270377	0.8156918936704
0.7133840014623	0.8157865661206
0.7134940379609	0.8161167289063
0.7135968610832	0.8161638466289
0.7136989322745	0.8162848411584
0.7138151957586	0.8162870786111
0.7139167896089	0.8163949423471
0.7140138012206	0.8166238256119
0.7141052533965	0.8167543819736
0.7141978314528	0.8169617979256
0.7142822413475	0.8171570477947
0.7143880891942	0.8172306582834
0.7144731884926	0.8173986862822
0.7145776050092	0.8176214163849
0.7146668856448	0.8179626477679
0.7147374868832	0.8183122276404
0.7148325525893	0.8184715040112
0.7149391408415	0.8185187903794
0.7150251213598	0.8185277714012
0.7151287092354	0.8185413099337

0.7152334659241	0.8191271632331
0.7153379871	0.8193476195019
0.7154422988842	0.8193484754148
0.715537619049	0.819388492514
0.7156488923645	0.8194608915366
0.7157484367025	0.8194971941141
0.7158347056522	0.8195490755996
0.7159207488399	0.819824181363
0.716040438258	0.8200812327751
0.7161400553149	0.8201563260902
0.7162321918713	0.8205213027242
0.7163249048949	0.8205465726378
0.7164369628514	0.8205492320602
0.7165549479886	0.8206964114301
0.7166546916844	0.8208614932748
0.7167589740723	0.8208748461673
0.7168604140292	0.8209995594446
0.716954787911	0.8212138028637
0.717067120493	0.8212261827079
0.7171638617712	0.8214005637567
0.7172774556022	0.8216091195973
0.7173716373383	0.8216401633935
0.7174819572	0.8217006238981
0.7175948549777	0.8217031991279
0.7177028829952	0.8217891428856
0.7177878173374	0.8218702921068
0.7178983356239	0.8219893158642
0.7179986336183	0.8222307632695
0.7180866869443	0.822343313555
0.7181934463621	0.8224863250461
0.7182769503407	0.8227082761865
0.7183856505914	0.8227447641123
0.718498732576	0.8228925674602
0.7186034787722	0.8229038897595
0.7187217202882	0.8229079240662
0.7187971718275	0.8229293301285
0.718903037205	0.822989764365
0.7189878285878	0.8230558890223
0.7190725190781	0.8235910520175
0.7191805068671	0.8236823721328
0.7192800281158	0.8240120610395
0.7194014635461	0.8243192084411
0.7195101065217	0.8243874032886
0.7196001747448	0.8244434445224
0.7197049076284	0.8245346454399
0.7198096911051	0.8247715206062
0.7199090720464	0.8249324204723
0.7200087465253	0.8251137356321
0.7200961667369	0.8251349134082
0.720187428874	0.8252871504021
0.7202878542962	0.8253601681478
0.720378997628	0.8257448238525
0.7204901608997	0.8257566662298
0.7205962222277	0.8259685496694

0.7207132487566	0.8260021752797
0.7208147387909	0.8261306481892
0.7209146689889	0.8262989693665
0.7210243191235	0.8264998189103
0.7211223207903	0.8271998211167
0.7212233251491	0.8274285801496
0.7213216347543	0.8274850781566
0.7214163685039	0.8275360926443
0.7215054269436	0.8277208259603
0.7216232273866	0.8279868798974
0.72173598335	0.8281930359089
0.7218429515934	0.8282380205223
0.7219519792247	0.828276285523
0.7220462361325	0.8282880964321
0.7221382162118	0.8283027611188
0.7222437544798	0.8284328141283
0.7223489721311	0.8284899462996
0.7224479323213	0.8286671536626
0.722539566765	0.8288984762868
0.7226269994047	0.8289923824949
0.7227390517872	0.8290801899436
0.7228427957012	0.8292818065035
0.7229329330774	0.8293166630071
0.7230271899936	0.8296958571871
0.7231438186546	0.8299959807797
0.7232546238039	0.8303523646016
0.7233796167707	0.830437424485
0.7234983850691	0.830710731357
0.7236006417898	0.830898502463
0.7237003400756	0.830942706086
0.7238120141116	0.8310124852461
0.7238992979562	0.8310890589121
0.7240184521777	0.8311385712294
0.7241375130049	0.8312953277677
0.7242136447601	0.8315313102982
0.7243426166722	0.8315495023259
0.7244487932288	0.8316793376513
0.7245584248417	0.8320345064073
0.7246626940696	0.8321816284001
0.7247456232451	0.8323318504053
0.7248349070699	0.8323486398113
0.7249324323792	0.8325459685597
0.7250149693322	0.8325627635011
0.7251258936772	0.8325827445567
0.7252149693096	0.8326239385873
0.7253136486869	0.8327000328113
0.7254102869184	0.8327989090749
0.7255047071532	0.8329877297232
0.7256080034164	0.8330261915838
0.7257130017976	0.8337158834904
0.7257960322421	0.8337282136346
0.725921134403	0.8337486885956
0.726033675311	0.8340632575629
0.7261412151829	0.8343153086972

0.726223653197	0.834364441336
0.7263126736544	0.8345088850591
0.7264064497584	0.834526850536
0.7264889309274	0.8345658535231
0.7265925178677	0.834737400285
0.726702097865	0.8351159413448
0.7267916230443	0.8351807950764
0.7268982360554	0.8352221688048
0.7269955050816	0.8352523300629
0.727100606932	0.8352753865353
0.7272015060318	0.8353809752863
0.7273036605756	0.8356535038505
0.7274353342521	0.8359958413861
0.727532454938	0.8361643367651
0.7276486117183	0.8362148240817
0.7277413112656	0.8363173398385
0.7278200080518	0.8363180456204
0.7279035758684	0.8363552052543
0.7280073893482	0.8366043951653
0.7281149372521	0.8368118528516
0.7282107580299	0.8369217267185
0.7282869632713	0.8376202843181
0.7283798500535	0.8376489595645
0.7284884731243	0.8377473824819
0.7285853615152	0.8378466883856
0.7286933729166	0.8378618014289
0.7287908760643	0.8379455045325
0.7288987544456	0.8382040140322
0.729010851432	0.8382216701442
0.7291011064094	0.8383137399227
0.7292067525603	0.8384224796948
0.7292962370801	0.83874059504
0.7293812737421	0.8388596073975
0.7294916021449	0.8389128264858
0.7296082963275	0.8390412337135
0.7296867189837	0.8392704030846
0.7297832478876	0.8393674760111
0.7298851189865	0.8394157830183
0.7299791963694	0.8394360786016
0.7300679480458	0.8394835644159
0.7301641965	0.8394837335612
0.7302511530833	0.8394942646109
0.7303541882302	0.8395146176852
0.730474859636	0.8396361034361
0.7305813131699	0.8396516886926
0.7306965232818	0.8396595554182
0.7307833474702	0.8396610627044
0.7308906927968	0.8397140428786
0.7309890936133	0.8397459618502
0.7310870191907	0.8398984776792
0.7311775179912	0.8399136303884
0.7312740723505	0.8400201902967
0.7313720632923	0.8403482480207
0.7314829038209	0.8405061432649

0.7315938508828	0.8405784741726
0.7316950706983	0.8406504859915
0.731814760899	0.840988458929
0.7319083272602	0.8417951736155
0.7319981117276	0.8419942416446
0.7320921685944	0.8420096241436
0.7321942129471	0.8422026477456
0.7322800825703	0.8422107575301
0.7323840637222	0.8433028004083
0.7324695228239	0.8435274906796
0.7325720685875	0.8436178113986
0.7326812634015	0.8438158282781
0.7327916112213	0.8439888084215
0.7328927402186	0.8447476232539
0.7330059139248	0.8448029675944
0.7331131062273	0.8448237310552
0.7332176754111	0.844847058523
0.733316081589	0.8448572924043
0.7333931105983	0.8448869765172
0.7334889310189	0.8451093414958
0.7335997258082	0.8451306773086
0.7336940787589	0.8455026143365
0.7337918353275	0.8456344107634
0.7339238489553	0.845789988222
0.7340225835293	0.8461552457299
0.734138043696	0.8464153399294
0.7342396789195	0.8464721466319
0.7343283809688	0.8465855620435
0.7344186956036	0.8467436039333
0.7345179303926	0.8471706251087
0.7346086105119	0.8471928917457
0.7347244809742	0.8472005554468
0.7348294148996	0.8472179229955
0.7349444885497	0.8477116531637
0.7350314071219	0.8478485841496
0.7351289351122	0.847869815117
0.7352244551523	0.8480667530037
0.7353228642117	0.8481934710009
0.7354303997092	0.8482262078836
0.7355327994132	0.8484146355779
0.7356406602692	0.8485917559248
0.7357491211995	0.848849338104
0.7358323505488	0.8491856624668
0.7359325723466	0.8492215886479
0.7360424685598	0.8492295709853
0.7361516460051	0.8494594669534
0.73626404589	0.8495236562894
0.7363592905881	0.8498747646675
0.7364958922435	0.8501527696195
0.7365968831531	0.8501849737487
0.736701389963	0.8502292747923
0.7368237444347	0.8503567526913
0.7369240210136	0.8504261187951
0.7370265194535	0.8505545318172

0.7371229387107	0.8507764867559
0.7372115355075	0.8507844460198
0.7373389130078	0.8508568514255
0.7374366730243	0.8510396825403
0.7375376575317	0.8511109064469
0.7376214650318	0.8513288946338
0.7377339424327	0.8513684822051
0.7378312237757	0.8514128952968
0.7379323562739	0.8514388111933
0.7380457134145	0.8517141878104
0.7381519867288	0.8517366620174
0.7382635152122	0.8520565127811
0.7383638573906	0.8520953042287
0.7384595961502	0.8523003763453
0.7385569360038	0.8525186907116
0.7386713768537	0.8528556511695
0.7387730992027	0.8528683070307
0.7388516284066	0.8530008314363
0.7389434891229	0.8531757922077
0.7390749787957	0.8534864824357
0.7391863999423	0.8536121114941
0.7392706830915	0.8536252019967
0.7393669359368	0.8536969652572
0.7394829579889	0.8537180502622
0.7395797699783	0.8540516940637
0.7396855553195	0.8541686265897
0.7397882360675	0.8542314077087
0.739890639807	0.8542620747188
0.7400075591045	0.8542666864381
0.7401059081496	0.854499532294
0.7402157693746	0.8548862021003
0.7403124366969	0.854900324649
0.7404278593855	0.8550616716419
0.7405077085817	0.8555616682392
0.7406059017746	0.8561121256138
0.7407019113229	0.8561127865308
0.7407917581474	0.856288001146
0.7408908230306	0.8565353956315
0.7409959686006	0.8566562165986
0.7410864161279	0.8567163565397
0.7411889109554	0.8570112295855
0.7412787394719	0.8572309155664
0.7413903435353	0.8574493539946
0.7414887286407	0.8576084197031
0.7415897566209	0.8576908783726
0.7416957158056	0.8578413867923
0.7418085874868	0.8578997646597
0.7419130585821	0.8581324472409
0.7420254983606	0.858476210219
0.742111070801	0.8585479856511
0.7422114503621	0.858564084387
0.7423138445957	0.858634424084
0.7424244460458	0.8586710891855
0.7425412109208	0.8588550117161

0.7426431437062	0.8592579586375
0.742761323295	0.8593892432601
0.7428632706694	0.8594082019618
0.7429664685662	0.8595003500698
0.7430571682366	0.859509967543
0.7431407565119	0.8597487488878
0.7432531174871	0.8598627569204
0.743345504039	0.8599166880259
0.7434286926014	0.8599997586898
0.7435232131319	0.8603626152733
0.7436105738464	0.8605247500549
0.7437180276388	0.8605355742312
0.7438137063101	0.8605415143175
0.7439064333536	0.8605767566463
0.7440121129658	0.8606056769047
0.7441411573821	0.8606461853751
0.7442499383251	0.8607447200699
0.7443478701217	0.8608286452041
0.7444370043111	0.8611704395178
0.7445257253106	0.8611729687803
0.7446087561584	0.861279895912
0.7446948238407	0.8613601237363
0.7447982711262	0.8614819510069
0.744915450064	0.8617411949415
0.7450217138107	0.8618056785428
0.7451163200078	0.8619077762592
0.7452295895513	0.862069500966
0.7453364961468	0.8621629839544
0.7454441762593	0.8623658935678
0.7455458421087	0.8625387979248
0.745637579367	0.8627605386368
0.7457567251846	0.8629416542555
0.7458486054782	0.863015064508
0.7459547861919	0.8634201816026
0.7460537148921	0.8634573411544
0.7461580628207	0.8636825177255
0.7462473814683	0.8637813430885
0.7463567545052	0.8638316780018
0.7464613324711	0.8639622969607
0.7465762359356	0.8641860994739
0.7466685460827	0.8644589529043
0.7467915244758	0.8645307208907
0.7468995520785	0.8647660657175
0.7469990043015	0.8650379077534
0.7470772614378	0.8652119591366
0.7471795616902	0.8652308040949
0.747287093945	0.8652491315254
0.747389158384	0.8654145903094
0.7474918041549	0.8656529525881
0.7475997115733	0.86573127638
0.7477146683103	0.8661547371965
0.7478249407501	0.8665933185532
0.7479034619529	0.8667018638657
0.7480139760841	0.8669298643221

0.7480996533263	0.8669625961972
0.748224128267	0.8672155378074
0.7483127818447	0.8672685569895
0.7484131280962	0.8673105730811
0.7485035823289	0.8673348823738
0.7485961806727	0.8674318801667
0.748704524659	0.8674759093243
0.7488194841453	0.867660813636
0.7489216035923	0.8676645220345
0.7490074814363	0.8677000876793
0.7491016056276	0.8678074292999
0.7492055909408	0.8679369682223
0.7493049373903	0.8679805024222
0.7494013181633	0.868095469181
0.7494919145043	0.8681473019682
0.7496018249896	0.8685163489546
0.7497065660912	0.8686366940095
0.7498201108712	0.8687366410448
0.7499375638917	0.8688056886848
0.7500492425345	0.8688796599729
0.7501240662594	0.8690546978232
0.7502444277254	0.8690996439608
0.7503695287395	0.869296959195
0.7504685033621	0.8696107536248
0.750552386011	0.8696678295901
0.750640844709	0.8697600294612
0.7507533137632	0.8697762296938
0.7508524356365	0.8701090684233
0.7509705122479	0.8702048850374
0.7510491425227	0.8702515739288
0.7511434765622	0.8704129857258
0.7512524423622	0.8705425744322
0.7513535144664	0.8708296886411
0.7514538748053	0.8712013438293
0.751564814604	0.8712966371259
0.7516653094152	0.8714695060752
0.751761662649	0.8715965838377
0.7518539140788	0.871826815373
0.7519480842511	0.8719084035856
0.7520524526777	0.8719366247336
0.7521795108911	0.8720020322762
0.7522919058044	0.8723436526838
0.7523872784353	0.8723648688324
0.752469105364	0.8725990486495
0.7525637824596	0.8726537712204
0.7526664280887	0.8727964031294
0.752788242893	0.8728271692398
0.7529020712627	0.8728275386063
0.7529983973548	0.8730692721652
0.7530900464505	0.8730785470845
0.7531716145805	0.8731035013946
0.7532641083664	0.8731191528594
0.753349742488	0.8733129743329
0.7534585523085	0.8733743516224

0.7535694349035	0.8735768099511
0.7536788297416	0.8737177988922
0.7537925855093	0.8742524776614
0.7538898786846	0.8743976605373
0.7539919360385	0.8744167346783
0.7540945540599	0.8748100806545
0.7541925398769	0.8749650716963
0.7542994252064	0.8749864714721
0.7544014452711	0.8750960477981
0.7544996952684	0.8753800725851
0.7546078105139	0.8756210084629
0.7547026651636	0.8756950048524
0.7547897024269	0.8757838109573
0.7549070375623	0.8761276715805
0.7549905953085	0.876273858213
0.7550859753976	0.8763040037783
0.7551895773399	0.8763042965592
0.7553127210292	0.8765968208388
0.7554219288271	0.8766535765306
0.7555441190635	0.8766605829047
0.7556584827358	0.8766825448612
0.755753884163	0.876793150402
0.7558514712059	0.8768309029221
0.7559503502553	0.8768309315727
0.7560559551782	0.8769886588925
0.7561792205129	0.8770514853941
0.7562759165696	0.877322287925
0.7563862867611	0.8773232081112
0.7564944926026	0.8774073032873
0.756567751136	0.8774755295699
0.7566917962492	0.8775151176766
0.7568010915002	0.8775654914105
0.7569108646276	0.8778655077414
0.7570312834569	0.8779173864409
0.7571394508534	0.8779598554224
0.7572509474651	0.8779664747712
0.7573551733588	0.8781356729523
0.7574363608477	0.8784416176402
0.7575549835604	0.8789349446898
0.7576539799227	0.8789777794494
0.7577467741504	0.8791902767033
0.7578370548654	0.8794034354002
0.7579387886225	0.8797250044426
0.7580407996343	0.8797251261695
0.7581524326823	0.8798647593767
0.7582418727412	0.880022190195
0.758326089174	0.880024527941
0.758435469628	0.8800462695801
0.7585424731679	0.8803504396742
0.7586535527438	0.8804382103886
0.7587555084238	0.8805721792896
0.7588557693586	0.8806737245498
0.7589571556444	0.8806874501984
0.7590534500887	0.8807935602936

0.7591664322902	0.8808221720981
0.7592663524321	0.881133655412
0.7593659072436	0.8811633032827
0.75947316032	0.881225306507
0.759592612766	0.8813083228601
0.759694874114	0.8814775680824
0.7598192031551	0.8816097740979
0.7599189798898	0.8817848426072
0.7600108707824	0.8818749345007
0.7601064602109	0.8825945417352
0.7602016834119	0.8828842556226
0.7603092931852	0.8829445742981
0.760410326729	0.8832599866483
0.7605199297622	0.8833067065693
0.7606185729541	0.8835237610565
0.7607076089397	0.8835436404528
0.7608105441756	0.8836555892353
0.7609169422849	0.8836617142084
0.7610221796505	0.8837662912862
0.7611403644134	0.8839055300878
0.7612447329143	0.8840036007557
0.7613619509944	0.8840471115045
0.7614674177433	0.8844504004015
0.7615741282814	0.8846723543981
0.761674020278	0.8847596160835
0.7617786602736	0.8854702008319
0.7618817739334	0.8856310024016
0.7620155689869	0.8858997392327
0.7621347124127	0.8860234320789
0.7622439221298	0.8861377857383
0.7623389001888	0.8862072934007
0.7624310948345	0.8862660788809
0.7625298900667	0.8863067222237
0.762633403178	0.8865512684221
0.7627379046519	0.8874873563622
0.7628447665215	0.8877066035342
0.76294068755	0.8877200544699
0.763032143059	0.8879654065839
0.7631368944535	0.8881514744716
0.7632357317484	0.8881669901329
0.7633545500923	0.8881828050312
0.7634569164727	0.8887825170325
0.7635561540441	0.8888256830075
0.7636545243734	0.8889855994558
0.7637562389106	0.8890354524572
0.7638653762174	0.88906964286
0.7639668478501	0.8891074482555
0.7640944721802	0.8891845993209
0.7641819273987	0.8892647242054
0.7643028757369	0.8893613943269
0.7644041542777	0.8895895235441
0.764514056592	0.8896653641065
0.7646221364999	0.8897434238796
0.7647403044036	0.8897599512731

0.7648556367622	0.8898086235394
0.7649381107817	0.8899331407972
0.7650546396192	0.890111675042
0.7651652886063	0.8902178385919
0.7652676282541	0.8902215255836
0.7653764498301	0.8903680499903
0.7654751403681	0.8905349086935
0.7655897686878	0.8906779501404
0.7656831987866	0.8909549770283
0.7658026244226	0.891115067712
0.7658799611155	0.8913188911512
0.7659738928154	0.8913321288011
0.7660720076216	0.891468682009
0.766174165058	0.8914847493424
0.7662784085149	0.8918072510846
0.7663723877364	0.8918335731556
0.7664772107183	0.8918468741438
0.7665741049762	0.8919281325908
0.7666980947849	0.891984999998
0.7668289569867	0.8919931278793
0.7669330493891	0.8920034982929
0.767046908323	0.892200825548
0.7671517267988	0.8924089101335
0.7672428187338	0.8924863545554
0.7673601829545	0.8925023610042
0.7674595819841	0.8925985884912
0.767545988729	0.8927337245426
0.7676490543663	0.8927530952859
0.7677472866716	0.8927820919313
0.7678326952771	0.8930408214706
0.7679432995567	0.8930414329125
0.7680484048597	0.8931016240006
0.7681479828277	0.8932466649342
0.7682653113234	0.8933701579346
0.7683631500335	0.8933804326741
0.7684749900905	0.8934361128132
0.7685872966955	0.8935231959979
0.7686932268896	0.8936416248574
0.7688043807331	0.8939778445613
0.768908208098	0.8940223073175
0.7690110104619	0.8940383514379
0.7691094304117	0.8943213160019
0.7692264878553	0.8944060152637
0.7693281772982	0.8944115677036
0.7694441592396	0.8944726414565
0.7695443057884	0.8946130433513
0.7696432754863	0.8947139837468
0.7697335826485	0.894765822654
0.769858033789	0.894834370163
0.7699588014307	0.8949275264278
0.7700629381142	0.894991720123
0.7701631168033	0.8953819382758
0.7702902134054	0.8956977013382
0.7703898263886	0.89582093146

0.7705091303776	0.8958549547293
0.7706139038284	0.8959752771555
0.7707058340883	0.896054368908
0.7708020277107	0.8960679279015
0.7708948862528	0.896109433726
0.7710187789626	0.8961649284125
0.7711217697994	0.8962131502207
0.7712052537723	0.8962850461502
0.7713075258132	0.8966715251965
0.7713879898118	0.8967782904238
0.7714989453737	0.8968061636217
0.771594396874	0.8969191084167
0.7716965346018	0.8970558415794
0.7718155540529	0.8971416304161
0.771905989376	0.8972295192051
0.7720030011273	0.8972637019608
0.7720899612901	0.8973950058573
0.7721971478208	0.8974030154701
0.7722797555314	0.8976796414447
0.7723861395402	0.8977609195064
0.772490004082	0.8978969782126
0.7725944637514	0.8979811822933
0.7726904318165	0.8982128391085
0.7727772333759	0.8983049514542
0.7728744287832	0.8983257905132
0.7729788193792	0.8983567109234
0.7730887247345	0.8987932033923
0.7731869994829	0.8988386233648
0.7732802455354	0.8988931402562
0.7734073980672	0.899295522563
0.7735084492192	0.899479223875
0.7736137430985	0.8996522069465
0.7737102643346	0.8997637551441
0.7737993859571	0.8997643478504
0.7739084918948	0.8998374704633
0.7740076762545	0.8998403366339
0.7741111407228	0.8998622453627
0.774201106496	0.8999639429257
0.7742951427156	0.9000368979161
0.7744267537779	0.9008509949726
0.7745349308866	0.9012495142392
0.7746374165455	0.9013414544257
0.7747375493753	0.9014144121704
0.774821359016	0.9014538094974
0.7749300927161	0.9015136688191
0.7750308134728	0.9016290612679
0.7751352342754	0.9017389621759
0.7752352055447	0.9018249865054
0.7753377442916	0.9019292902602
0.775431404655	0.9020119285924
0.7755284459879	0.9022345245773
0.7756075001137	0.9022414271302
0.7757295822677	0.9022737715021
0.7758350424634	0.9026116128117

0.7759590981458	0.9027206249778
0.7760511796246	0.902758725006
0.7761658222809	0.9027919052818
0.7762593382711	0.90286359061
0.7763735892178	0.9030737528457
0.7764903714496	0.9030886737787
0.7766113959851	0.9031558395271
0.7767104937421	0.9032214223859
0.7768131057883	0.9035401530725
0.7769218178004	0.9037606440343
0.777018472856	0.9038913027654
0.7771391327968	0.903942889061
0.7772469897501	0.9041486250713
0.7773776444199	0.90429909302
0.7774769591865	0.9044845831977
0.7775743694449	0.9045565463525
0.7776925193162	0.9047391705365
0.777789764506	0.9049205339365
0.7779013602544	0.9049301092017
0.778008711755	0.9050000495418
0.7781025318781	0.905064466672
0.7782252946929	0.9052003108152
0.7783184383612	0.9052375147532
0.7784259532808	0.9054327757303
0.7785462547416	0.9055136727761
0.7786536838539	0.9056444006109
0.7787643035413	0.9060077868968
0.7788673147814	0.9060480161539
0.7789726488209	0.9061283611901
0.7790842994575	0.9064026824898
0.7791692144993	0.9065177936023
0.7792657283903	0.9067955108229
0.7793747957482	0.9068345950595
0.7794619148844	0.9069144927356
0.7795659258696	0.9069649510476
0.7796688278815	0.9072474885559
0.7797760688163	0.9075925522091
0.7798729629423	0.9076929481084
0.779983313973	0.907696601059
0.7801067202048	0.9079754889521
0.7802066969005	0.9080356893043
0.7803075623137	0.9080632634317
0.7804069195588	0.9081262692325
0.7805064840322	0.9083364974146
0.7805994903816	0.9084687810106
0.7807061223689	0.9084804301724
0.7808340846777	0.9085003246554
0.7809223231042	0.9085175656676
0.7810339933087	0.9085727177613
0.7811315054173	0.9086757678012
0.7812140301864	0.9087575515765
0.7813232255886	0.9088032146191
0.7814338258791	0.9089679635809
0.7815364713406	0.9091046423657

0.7816367505685	0.9092641969613
0.7817373757356	0.909277413429
0.7818172725984	0.909298467691
0.7819092147565	0.9093203817853
0.7820110457266	0.9094437991568
0.7820952484565	0.909577656913
0.7821943323272	0.9099334322686
0.7822871161476	0.9103986828264
0.7823831132775	0.9106790493058
0.7824666709105	0.9107343502952
0.7825792152269	0.9109806483772
0.7826993972846	0.9110613477577
0.7828304810748	0.9113861652969
0.7829370020552	0.9116375192949
0.7830188292649	0.9116414745841
0.7831180308389	0.9119643662141
0.7832152064252	0.9120310565372
0.7833166029704	0.9120580723654
0.7834166043122	0.912085222408
0.7835197110611	0.9121693857054
0.7836142606618	0.912412173405
0.7837331072181	0.9124203938144
0.7838229526547	0.9125968579846
0.7839347357699	0.9126777253683
0.7840490489699	0.9127702900193
0.7841650439697	0.9129567299157
0.7842642039646	0.9131514300073
0.7843578670886	0.9132044245045
0.7844518731162	0.9133698376644
0.7845475422885	0.9136608965855
0.7846417871872	0.9136632052687
0.7847535107363	0.9137062198533
0.7848694918644	0.9137555077243
0.7849782938446	0.9139601670417
0.7850836354466	0.9139637394994
0.7852163018776	0.9144350528395
0.7853141909485	0.9147495175128
0.7854050838156	0.9148661693943
0.7855045724804	0.9153476183453
0.7856126168717	0.9153666900669
0.7857117050531	0.9153984147608
0.7858434616696	0.9155074189051
0.7859420353296	0.9156944817615
0.7860591829028	0.9157378339722
0.7861398386058	0.9159693711683
0.7862428595321	0.9160237599263
0.7863334920408	0.9160773310167
0.7864354394023	0.9162342878212
0.7865419516	0.9162678000798
0.7866486028126	0.9163983208583
0.7867746924984	0.9164347723496
0.7868875982631	0.9166386715735
0.7869861957914	0.9167628938819
0.787075939183	0.9170192166229

0.7871699616128	0.9172044459069
0.7872979888248	0.9173973612052
0.7873911361698	0.9174859774767
0.7874810577759	0.9177975144547
0.7875801694523	0.9178105553656
0.7876797482143	0.9182124538498
0.7877829014747	0.9183135657872
0.7879023521544	0.9186071591553
0.788009107994	0.918916510637
0.7881106344636	0.9189236854355
0.7882133364908	0.9189385907121
0.788325404409	0.9190766509508
0.7884462092172	0.919141229751
0.7885446767847	0.9193309879281
0.7886595436636	0.9193377486524
0.7887464156332	0.9193776803233
0.7888618488204	0.9193879717307
0.788977690776	0.9196553377831
0.7890704442754	0.9199126556345
0.7891872565114	0.9201086266283
0.7892857717657	0.9201867055807
0.7894169629199	0.9205820143703
0.7895355040139	0.9206069830013
0.7896465663396	0.9208312730175
0.7897568516767	0.9209191300217
0.7898606407634	0.9212255056135
0.7899892608609	0.9213569309234
0.7900694240941	0.9216633425886
0.7901579362593	0.9217090422558
0.7902642200865	0.9218044553415
0.7903558155697	0.9219055494821
0.7904576164459	0.922021406567
0.7905651389232	0.9220783406907
0.7906809328719	0.9222231090344
0.7907844519936	0.9222313020835
0.7908816879915	0.9223477624461
0.7909735040177	0.9224131339659
0.7910827928851	0.9224345440946
0.7912085672039	0.9225559699798
0.7913393519548	0.9225632300402
0.7914580300645	0.9227424664641
0.7915639604306	0.9229488436942
0.7916747910037	0.9233692133744
0.7917722624787	0.923395868562
0.7918769932124	0.9233994449705
0.7919888049285	0.9234744854315
0.7920685765828	0.9235036319108
0.7921775175481	0.9236701253542
0.79228715994	0.9237738965784
0.7924034616093	0.9241135194028
0.7925175834921	0.9244413833553
0.7926408104252	0.9245943475754
0.7927302718843	0.9247314360161
0.7928178681449	0.9248029207064

0.7929162322743	0.9248654958072
0.7930375110832	0.9251345285467
0.793155792187	0.9254765671271
0.7932482783134	0.9256054722259
0.7933641497143	0.9256186907983
0.793478013361	0.9256755975567
0.7935964777738	0.9257582201183
0.7937126930986	0.9264118003445
0.7938115520599	0.926543098223
0.7939288690445	0.9265994653483
0.7940180343359	0.9268315564785
0.7941231113372	0.9275522751827
0.7942168116758	0.9276259009958
0.7942995266917	0.9277485782783
0.7944094315178	0.9278159090195
0.7945229547373	0.9278680748886
0.7946387196014	0.9279338992853
0.7947411886475	0.927994362649
0.7948739653144	0.9280440705696
0.7949697538605	0.9281936830793
0.7950641221788	0.9285206978861
0.7951391897414	0.928551838653
0.7952626760716	0.9286325024707
0.7953688310862	0.9287221805392
0.7954785536516	0.9287964912472
0.7955844542401	0.9288976627133
0.7956853639134	0.9290953846293
0.7957785765347	0.929215730311
0.79589992424	0.9292965826934
0.7960111246155	0.9294964567886
0.7960981201183	0.9301566774808
0.7962102849111	0.9302667993571
0.7963197655124	0.9303199080419
0.7964213904497	0.9305649459764
0.7965327892902	0.9305908445538
0.7966459023709	0.9307881713999
0.7967546334073	0.9308977321107
0.7968994897552	0.9309472367729
0.7970064337212	0.9309981211505
0.7971096357241	0.9312804241049
0.7972326366658	0.9312952855956
0.7973537782531	0.9313379988839
0.7974680795033	0.9317951905132
0.7975849293737	0.9318027886737
0.7976768904381	0.931918824922
0.7977711655378	0.9319425688781
0.7978700005365	0.9319970118486
0.7979688932038	0.9320233334058
0.7980784534971	0.9321963280166
0.7981939140768	0.9322284912479
0.7982932039362	0.932298086679
0.7983947630484	0.9328722573072
0.7984968561466	0.9328761352991
0.7985977148132	0.9330339186244

0.7986948506108	0.9330498563019
0.7988317520474	0.9331671634818
0.7989284231941	0.9334068544794
0.7990468964382	0.9334121135603
0.7991419353429	0.9334379073723
0.7992356815675	0.9336204089129
0.7993600611998	0.9337982474479
0.7994370603436	0.9340157247936
0.7995727386738	0.9340434996615
0.7996879498974	0.9341518734328
0.7998067512894	0.9345087313099
0.7999410095898	0.9346955060648
0.8000413842093	0.9349338244975
0.8001472466601	0.9349815522498
0.8002629381329	0.9349936747498
0.8003637647474	0.9350795563397
0.8004770410034	0.9351544743767
0.8005906001808	0.935290545101
0.8006754709984	0.9353408646414
0.8008065809198	0.9353579356173
0.8008934977471	0.9354599036877
0.8009925591543	0.9354702320704
0.8010934300916	0.9357368946971
0.8011954453934	0.9359314825526
0.8012809538737	0.9364342876956
0.8013640983596	0.9364597408426
0.8014756039937	0.9364856608165
0.8015843586335	0.936891423637
0.8016748492396	0.9370049185523
0.801771857549	0.9371986656881
0.8018816782273	0.9374229050947
0.8019861329987	0.9379252225237
0.8021015964689	0.9380931675915
0.802220897237	0.9380964461931
0.8023483065499	0.938129792642
0.8024469943692	0.9382713993809
0.8025384218577	0.9385108346584
0.8026563386622	0.9385481887778
0.8027795739429	0.938570649485
0.8028956541319	0.9385720510283
0.8030229025432	0.9386354172561
0.8031294003394	0.938644896238
0.8032321808914	0.9386774278086
0.8033246948762	0.9388421985776
0.8034255475825	0.9389412100636
0.803514091805	0.9391194680694
0.8036177885737	0.9391242896488
0.8037169199066	0.9391707929636
0.803812448465	0.9395574383056
0.803919724295	0.9396291848892
0.8040464830598	0.9398065331019
0.8041471310298	0.9398964116893
0.8042484653214	0.9400090485595
0.8043467161808	0.9401254932978

0.8044155084737	0.9403019569542
0.8045153174736	0.9408320577515
0.8046321612769	0.9410525219701
0.8047459838436	0.9410593171722
0.8048523374972	0.9410610587462
0.8049343603585	0.9410711667988
0.8050437021475	0.941170207032
0.8051445079451	0.941695533674
0.8052434939755	0.941704711957
0.8053470318337	0.9419329441241
0.8054310924093	0.9425491204152
0.8055435352089	0.9427033511161
0.805632055622	0.9429445786203
0.8057449300268	0.9431680855925
0.8058411296086	0.9431970724809
0.8059542481433	0.9432115820252
0.8060689437605	0.9432398292697
0.806187615572	0.9432761433991
0.8063073812002	0.9433403531691
0.8064031235461	0.9433766392762
0.8065180345331	0.943514850999
0.8066227831851	0.9435929809895
0.8067443521302	0.9438134187348
0.806862778929	0.9442630467943
0.8069601666189	0.9443215299921
0.8070692990794	0.9443859386799
0.8071928041801	0.9445366564501
0.8072994018182	0.9445592310595
0.8073868766367	0.9447908065041
0.8074713715735	0.9448089557595
0.8075759670531	0.9451086791502
0.8076674121876	0.9452681366941
0.8077806824121	0.9452828431351
0.8078833783057	0.9453205104868
0.8080118238421	0.9455698113569
0.8081086451708	0.9455705098021
0.8081884432741	0.9460039794173
0.8082896479418	0.9460484800481
0.8084063142138	0.9462826971204
0.8085093243586	0.9463730030741
0.8086273063417	0.9464810279256
0.8087283975906	0.946548441422
0.8088471661953	0.9465761049001
0.8089510169965	0.9465876044625
0.8090550700045	0.9471258679839
0.8091816895535	0.9471697512742
0.8092874919087	0.9472470179979
0.8093706775096	0.9472554060311
0.8094904922946	0.9473166437225
0.8095930720348	0.9474521628473
0.8096941841859	0.9474977496379
0.8097816330375	0.9477229153929
0.8098918035184	0.9480242263635
0.810023789449	0.9481136617989

0.8101336112525	0.9482788899354
0.8102519829219	0.9482835471851
0.8103461868535	0.9483668392161
0.81044515031	0.9484138020062
0.8105597169295	0.9485148116385
0.8106791625503	0.9487780865252
0.8107710354275	0.9489827769659
0.8108764586652	0.9491189875997
0.8110158909976	0.9491609693464
0.8111430250252	0.9493012713583
0.8112519313486	0.9495013587682
0.8113664146709	0.9498704402326
0.8114647006412	0.9498727520466
0.8115633195546	0.9499861586307
0.8116515260005	0.9501119734541
0.8117712895661	0.9501706201161
0.8118705500324	0.9502323952496
0.8119704412894	0.9502536858165
0.8120591700584	0.9503617507556
0.8121679331093	0.950400364167
0.8122917228604	0.9504433135679
0.8124210993834	0.9505768116898
0.8125297788859	0.9509491060107
0.8126261611219	0.9512135372131
0.8127350642032	0.9513096800836
0.8128428926266	0.9514745293218
0.812940259046	0.9517241693282
0.8130399057731	0.9518633490276
0.8131503479892	0.9520610886267
0.8132485553103	0.9520890507334
0.8133397729582	0.9526141227823
0.8134336493824	0.9530743836914
0.8135326145571	0.9531979396999
0.8136376321191	0.953214451791
0.8137484494211	0.9532270071768
0.8138410917122	0.9533864471724
0.8139405803845	0.9534492601911
0.8140450751754	0.9535023395264
0.8141334662571	0.9535057072058
0.8142483931658	0.9535730732901
0.8143839860356	0.9536885694163
0.8144939225322	0.9537234127716
0.8145795196283	0.9537947253354
0.8146914461424	0.9540226367325
0.8147831093875	0.9541722270566
0.8148989973624	0.9542059930211
0.8150185595433	0.9542870521932
0.8151282064809	0.9542928359026
0.8152597826063	0.9543291622197
0.8153674311729	0.9547491623864
0.8154786324485	0.9551591983864
0.8155761635261	0.9551667609679
0.8156769217891	0.9553153950463
0.815792259338	0.9554858906447

0.8158939824268	0.9556471745763
0.8159960937345	0.9557285659314
0.8161104517925	0.955735919221
0.8162252479631	0.9562158690891
0.8163371581283	0.9563956263117
0.8164568343697	0.956493274694
0.8165623617217	0.956501493557
0.8166764927274	0.9568619268345
0.8167889429904	0.9569787844099
0.8168839203227	0.9569930160739
0.8170163430777	0.957078770608
0.817115913608	0.9572672616366
0.8172330706789	0.9572961912278
0.8173377707431	0.9573952321413
0.817466332191	0.9574344841005
0.8175714101071	0.9574920509255
0.8176659288758	0.9578523000493
0.8177629052444	0.9578594640754
0.8178597880036	0.9580053270606
0.8179783948038	0.9581516133806
0.8180976471684	0.9581680315625
0.8182003413267	0.9583233434047
0.8183259127409	0.9584805423151
0.8184249937585	0.9587063464394
0.8185318922219	0.958773939447
0.8186194892769	0.9588807986827
0.8187408079888	0.9589164004674
0.8188448233635	0.9593235661628
0.8189372552897	0.9593375586336
0.8190351764243	0.9593383164425
0.8191457569327	0.9594358257291
0.8192491140011	0.9595526592761
0.819329506853	0.9597266352482
0.8194442362533	0.9598215095014
0.8195204706563	0.959945353245
0.8196197738028	0.9600184186414
0.8197231827477	0.9600774734821
0.8198197009531	0.9600956044174
0.8199386253392	0.9601694959785
0.8200299916883	0.9601715217699
0.8201525852916	0.960267306438
0.8202500102724	0.9604386880809
0.8203412698554	0.9605779531057
0.8204303901123	0.9606687491217
0.8205454502021	0.9606812267547
0.8206521100194	0.9611097335993
0.8207622626379	0.9612858176354
0.82087696836	0.9615212455686
0.8210048033591	0.9616051252462
0.8211081296407	0.961621718023
0.8212300110909	0.9616476465254
0.8213319086462	0.9617526706572
0.8214401373639	0.9618109500951
0.8215629600886	0.9618145050681

0.8216687573101	0.9621182158373
0.8217787072574	0.9621493927854
0.8218801994617	0.9626227233792
0.8219883951619	0.9631811357871
0.8220905820649	0.9632619536215
0.822196149205	0.9635347072222
0.8223022535973	0.9635712908175
0.8224143144662	0.9635779297273
0.8225035580553	0.9638216796004
0.8226024854011	0.9639732803654
0.8226990075752	0.9640754947605
0.8228303676138	0.9642320989389
0.8229529280432	0.9642475622046
0.8230368342386	0.9642919836151
0.8231330274996	0.9643021134034
0.8232288845112	0.9643144461874
0.8233308881717	0.9644268041692
0.823424918734	0.9644383829924
0.8235112503073	0.9647459558355
0.8236025171207	0.9647918330684
0.8237121550827	0.9648559739419
0.823861836315	0.9649058779468
0.8239547524192	0.9649104148386
0.8240671583261	0.9650891029626
0.8241644257477	0.9651895650407
0.8242680055787	0.9652136076833
0.8243681964763	0.9652313386739
0.8244644798554	0.9652627753162
0.8245554888534	0.9654999759372
0.8246603944946	0.9655362033649
0.8247765196609	0.9658276755701
0.8248757562794	0.9659167984403
0.8249770676321	0.9660069621916
0.825080210994	0.9663826891438
0.8251922485548	0.9664472295634
0.8252957810821	0.9664692330481
0.8254038345719	0.9668482117656
0.8255012648198	0.9673178556194
0.8255981754501	0.9679104972816
0.8257050819719	0.9681972770366
0.8258072698106	0.968295450072
0.8259145765029	0.9685027070751
0.8260283365628	0.9685952663927
0.8261258599005	0.9686832499823
0.8262577182239	0.9688067438318
0.8263734143336	0.9688346279227
0.826487352163	0.968893535838
0.8265845640895	0.9690650037122
0.8267313670238	0.9690856975857
0.8268407444391	0.9691566712166
0.8269260580352	0.9691905971171
0.8270051470241	0.9694600519686
0.8271154943113	0.969491095523
0.8272226835452	0.969550115591

0.8273335894868	0.9698084957316
0.8274348280377	0.969878102285
0.8275420071998	0.9699391225577
0.8276597083311	0.9699441162419
0.8277632466093	0.9699491271886
0.8278730047426	0.9700530419377
0.8279646169056	0.9700591541073
0.8280575444323	0.9703282494983
0.8281588442655	0.9705164408492
0.8282777216199	0.9705249085918
0.8283918053037	0.9705297718314
0.828493964926	0.9705600180931
0.8286088948939	0.9705731390893
0.8287160572362	0.9707215077432
0.828819823415	0.970804327986
0.8289504051371	0.9708116077819
0.8290558997873	0.9710895809686
0.8291567724557	0.9710923202385
0.8292443272664	0.9713315329874
0.8293443863059	0.9716031440527
0.8294565593084	0.9717504590754
0.8295804681519	0.9717717437917
0.8296875169717	0.9717785982326
0.8297758953729	0.9721891157622
0.8298589243151	0.972277171658
0.8299575051174	0.9723686920748
0.8300718232573	0.9723724203581
0.8301837456336	0.9726057598533
0.8302845910165	0.9729745992618
0.8303871667015	0.9730284805662
0.8304801463388	0.9732402787203
0.8305891390437	0.973298223297
0.830687931778	0.9733931259765
0.830789405385	0.973477046169
0.8309059346448	0.9735163828161
0.8309974518122	0.9735962190103
0.831115347556	0.974605090607
0.8312222174873	0.9747270635425
0.8313237686285	0.9747617737666
0.8314306854669	0.9749687586283
0.8315247030403	0.9750633969671
0.8316311404719	0.9751573939809
0.8317216239168	0.9751873140255
0.8318210056827	0.9752078538111
0.8319287494147	0.9752425239991
0.8320508140083	0.9752913890026
0.8321320926901	0.9754301593296
0.8322267802628	0.9755184524049
0.8323395430322	0.9755511901395
0.8324347888699	0.9759164464958
0.8325453051606	0.9761154066001
0.8326589870529	0.9762539756725
0.8327596813569	0.9763168798256
0.8328429334579	0.976346159176

0.8329592300352	0.9767045048152
0.8330510636396	0.9767305231451
0.8331608117871	0.9770618846081
0.8332587637267	0.977154175136
0.833383109464	0.9771762989446
0.8334814918062	0.9773896604796
0.8336001621078	0.9774778293566
0.8337198788435	0.9776172949415
0.8338390687482	0.9777479450973
0.8339438143392	0.9778552526307
0.8340562344199	0.9780353137807
0.8341618250779	0.978114685908
0.8342758959249	0.9783606443302
0.8343832921881	0.978516084608
0.8344794520689	0.9785497383124
0.8345602342948	0.9786459946804
0.8346586457336	0.9786478347816
0.8347720696215	0.9786608827454
0.8348805189524	0.9787397407066
0.8350027823898	0.9789166862158
0.8351251819996	0.9791581636596
0.8352399955033	0.9792234963045
0.8353419036922	0.9792433660435
0.8354550933529	0.9793847850317
0.8355788482332	0.9794350253363
0.8356868073272	0.9795457889271
0.8357899984202	0.9797789839821
0.8359097892837	0.9798445463756
0.8360091636125	0.9798589003413
0.8361378154163	0.9798895694871
0.8362396557838	0.9799772966062
0.8363585843585	0.980020251363
0.8364503911429	0.9800642674743
0.8365708632689	0.980180427134
0.836672756525	0.9801929350277
0.8368049093527	0.9807544415431
0.8369292352046	0.9810708027732
0.8370337125586	0.9811608671401
0.8371520057562	0.9814061963474
0.83724058036	0.9815736999219
0.8373521981885	0.9816318822974
0.8374662093848	0.9817237781594
0.8375605011159	0.9818893510506
0.8376665876049	0.9823981039116
0.8377872522244	0.9825679784723
0.8379070625136	0.9833855704246
0.8380263152338	0.983394980424
0.8381189776473	0.9834488328413
0.8382319397588	0.9834847604184
0.8383541674106	0.9837011263858
0.8384362664032	0.9838153363755
0.8385363194043	0.9843079680645
0.8386365006944	0.9845331191363
0.8387449945692	0.9848255181824

0.8388356836706	0.9848419112971
0.8389466580812	0.9849579256081
0.8390590579506	0.9851457931574
0.8391633985663	0.9852141723663
0.839264172334	0.985345443427
0.8393726382592	0.9855376300162
0.8394901822859	0.9855729882061
0.8395751766492	0.9860945172752
0.8397016544516	0.9867275412419
0.8398031989292	0.9868424652152
0.8399050899238	0.9869444089133
0.8400105914571	0.9870032171514
0.8401332243123	0.9870398549487
0.8402329593593	0.9870448812917
0.8403341973674	0.9870526008406
0.8404391148623	0.9870809033014
0.8405498780572	0.9871787168521
0.8406570773911	0.9874122732209
0.8407542732366	0.9874482385235
0.8408794528263	0.9874505152967
0.8410200707683	0.9875275992196
0.8411249786683	0.9877218409713
0.8412432397656	0.9878784385427
0.8413479820033	0.9879540071459
0.8414743385169	0.9879607255285
0.8415789844097	0.9880536731253
0.841680139665	0.9886406473186
0.8417875718028	0.9886850295543
0.8419164408583	0.9889057243584
0.8420183515696	0.9890964118933
0.8421366335761	0.9891864618423
0.8422576267728	0.9891950989869
0.842372740752	0.9892598107169
0.8424772718584	0.9894503373679
0.8425796602905	0.9894618335583
0.8426853941631	0.9895602932564
0.842798974836	0.9897950431571
0.8429042933688	0.9898965215274
0.8430092375488	0.989982960028
0.8431271839629	0.9900268958938
0.8432481366399	0.9901906532295
0.8433694314515	0.9902065810996
0.8434991323616	0.9902245802669
0.8435840253082	0.9903537687204
0.8437029471256	0.990463019216
0.8438099032462	0.9907237507707
0.8439308104041	0.9908730796563
0.8440272126058	0.9909027685353
0.844139730029	0.9910069620083
0.8442557341767	0.9910679166168
0.8443537823758	0.9911826771494
0.8444612123179	0.9917143602341
0.8445871817269	0.9918169786525
0.8446921612874	0.9918277905826

0.8448150064313	0.991832631576
0.8449102695809	0.9919689625775
0.8450250921807	0.9919935000975
0.8451397283252	0.9922171653893
0.8452352562829	0.992337809829
0.8453538005086	0.992379601662
0.8454563123371	0.9924237352316
0.8455636923251	0.9927917343953
0.845649628281	0.992914024722
0.8457655279988	0.9930415793172
0.8458677437058	0.9930756844168
0.8459786862691	0.9933640292462
0.8460766712679	0.993384593559
0.8461723153381	0.9934705518301
0.8462862511922	0.9935560571198
0.846407642029	0.9937445041229
0.8465189932732	0.994357844964
0.8466110086063	0.9944290570567
0.8467294496014	0.9946789653722
0.8468241013324	0.9946870508741
0.8469247705279	0.9948608879521
0.8470599377708	0.9948820832713
0.8471496580403	0.994955499045
0.8472639225156	0.9950254226711
0.8473780168579	0.995115188569
0.8474876244162	0.9952590771832
0.8475805054062	0.9957867686157
0.8476832130848	0.995892527012
0.8477903392524	0.9959103231538
0.8478755980914	0.9962865685143
0.8479727748897	0.9963471756964
0.8480828486133	0.9965347383409
0.8481801088134	0.996544946664
0.8482967775195	0.9968437935275
0.8484141402195	0.9973102139205
0.8485301337074	0.9973150335199
0.8486455104318	0.9973171061288
0.8487654117666	0.9974696285915
0.8488577847843	0.9976274388443
0.8489513461494	0.9977130384081
0.8490768671576	0.9981335858007
0.8491776889801	0.9982444650936
0.8493075500097	0.9982941099474
0.8494219661263	0.9984782823162
0.8495463725599	0.9985146629951
0.8496561545754	0.9985672982949
0.8497570893663	0.9986708514055
0.849864161512	0.9988766028277
0.8499916758928	0.9989895777516
0.8501011279074	0.9991035041698
0.8501907416939	0.9993411639921
0.8503074410982	0.9993540649131
0.8504129256925	0.9994597563853
0.8505144062023	0.9996901931536

0.8506133033111	0.9997651065368
0.8507034685643	0.999768186887
0.850821957748	0.9997982758344
0.8509511584892	1.000139335288
0.8510312781372	1.0001918114869
0.8511263510514	1.0002887613012
0.8512430447862	1.0005791250173
0.8513370327992	1.0005853037272
0.8514416865356	1.0006683096232
0.8515505479433	1.000730201738
0.8516761746667	1.0009123504133
0.8517804104345	1.0012569870471
0.8518913810494	1.0014280488077
0.8520204126597	1.0014308477857
0.8521347648901	1.0014870997675
0.8522297305841	1.0017801030968
0.8523602619682	1.0018910163182
0.8524835759918	1.0020694261098
0.852562387347	1.0021171826361
0.8526702618859	1.0023192595978
0.8527638671298	1.0026200004981
0.8528581860522	1.002628469943
0.8529621479123	1.0027069549011
0.853070814104	1.0027386770203
0.8531879108869	1.0027785277717
0.853298265378	1.0029015010327
0.8533915447838	1.0029627284073
0.8535103901352	1.0031214023426
0.8536170133942	1.0031438409968
0.853730448354	1.0032200086526
0.85383686409	1.0033296589675
0.8539512786915	1.0035327788232
0.8540599415217	1.0035961581292
0.8541856297379	1.0036926540651
0.8542838013831	1.0038361875523
0.8543841157544	1.0040100124399
0.8544715733598	1.0040148015967
0.8545788019575	1.0042096801836
0.8546884762894	1.004239149952
0.8548064976382	1.0043158304619
0.8549137032528	1.0044623174077
0.8550190354676	1.0044717936793
0.855130479035	1.0045427984656
0.8552490837133	1.0047923886615
0.8553715611975	1.0049336684415
0.855477939716	1.0052061622039
0.8556096119413	1.0054134794843
0.8557120791746	1.0054397923447
0.8558061582938	1.0054448836409
0.8559187121752	1.005623932714
0.8560275039798	1.0060489664521
0.8561496760886	1.0063668122935
0.8562405085379	1.006382595402
0.8563401389377	1.0064064872893

0.8564568900247	1.0064561458421
0.8565600947492	1.006463026559
0.8566913213611	1.0067273107871
0.8568008205241	1.0068672008424
0.8569177552409	1.0070047908606
0.8570432856483	1.0072307996828
0.857142423564	1.0072596898805
0.857267766966	1.0075147872353
0.8573859517858	1.0076053166282
0.8575175509492	1.0076972994905
0.8576407031343	1.0080110830188
0.857772642451	1.0080302751121
0.8578740384738	1.0081666081914
0.8579861044408	1.0082862240865
0.8580654764618	1.0084142430709
0.8581613432137	1.0084741983477
0.8582751922311	1.0085041040892
0.8583681367968	1.0086490892849
0.8584901860555	1.0086578170856
0.858591327129	1.0087069106816
0.8587071965836	1.0090890452703
0.8588037062609	1.0090912551991
0.8589056853449	1.0091757657447
0.8590052534965	1.0093668604664
0.8591319067269	1.0094614506755
0.8592547734394	1.0094643967165
0.8593672470055	1.0096440506072
0.8594582827453	1.009755199459
0.8595806867831	1.0097850259678
0.8596913219733	1.0105713765382
0.8598056721012	1.0109335075056
0.8598973311273	1.0111016063163
0.8600017096915	1.0111152941257
0.8601141950965	1.0112135357132
0.860226085385	1.0112931733597
0.8603547447141	1.0113247714671
0.8604908967179	1.0115834800136
0.8605890133777	1.011645346569
0.8606911661703	1.0124559058818
0.8608008808793	1.0124894643592
0.8609109292284	1.012608679945
0.8610177401284	1.0127807773684
0.8611238655793	1.0128442833784
0.8612279007138	1.012961808152
0.8613347316566	1.0131043920874
0.861439017992	1.0132687945769
0.8615495779457	1.0132861589959
0.8616497408381	1.0134721272719
0.8617556696551	1.0135618441687
0.8618892624837	1.014046452058
0.8620238561805	1.0141776152943
0.8621170554314	1.0143453623933
0.8622387130336	1.0144757761787
0.8623517934622	1.0145356201236

0.8624709356276	1.0148265148983
0.862590254505	1.0148661589954
0.8626992761081	1.0150214941355
0.8627989541616	1.0152104259254
0.8629068741242	1.0153663924203
0.8630150823767	1.0156823208846
0.8631113738399	1.0158253810242
0.8632184570252	1.016281105651
0.8633231233158	1.0165329723732
0.8634234194999	1.0165875208638
0.8635376678666	1.01675989752
0.8636376757015	1.0168913978154
0.8637565065791	1.0169853967719
0.8638678433584	1.0172482158309
0.8639823363426	1.0173895717613
0.8640848977535	1.0174619718326
0.8641786530288	1.0177123327489
0.8642753918965	1.017716322417
0.8643919112401	1.0177168748883
0.864482559158	1.0177800372399
0.8645966703144	1.0189344024509
0.8646799367322	1.0192098300461
0.8647936749235	1.0196373221615
0.8648991187219	1.0196487788283
0.8650283084927	1.0199184956409
0.86513766677	1.0200082206931
0.8652470332039	1.0201787395649
0.865350738301	1.0202072311275
0.8654654927557	1.0203738556753
0.8655567433947	1.0204681658656
0.8656659399248	1.0204866528922
0.8658099522753	1.0205905534554
0.8659288706364	1.020773766374
0.8660249707286	1.0208938422036
0.8661413597408	1.0213599781713
0.8662756756987	1.0213724648525
0.8663665432708	1.0216713987404
0.8664933163716	1.02182075567
0.8666084014569	1.021847883621
0.8667246624957	1.0223384258635
0.8668251660184	1.0223893381351
0.8669590935112	1.022515701937
0.8670687420505	1.0227098351578
0.8671709971095	1.0227927566213
0.8672762006726	1.0229278744429
0.8673740904277	1.0230229142805
0.8674751159417	1.0231172322426
0.8675998761316	1.0232000375987
0.8676985625832	1.023223356228
0.8678235135203	1.023391588244
0.8679295218448	1.0235601823037
0.8680266319176	1.0237507122481
0.8681267636072	1.0240356804746
0.8682313595583	1.0240552131939

0.8683244859955	1.0240999563539
0.8684170316646	1.0241900711531
0.8685029943539	1.024279749791
0.8686122658842	1.0245296622495
0.8687163408966	1.024553423091
0.8688311909925	1.0245945666073
0.8689478406884	1.0247401339415
0.8690639076966	1.0250294803017
0.8691828598731	1.0252263247477
0.8692849886962	1.0254364054762
0.8693792894372	1.0254476763061
0.869473283662	1.025506040077
0.8695827244975	1.0256491418078
0.8696963992451	1.025793733718
0.8698353231681	1.0258009648497
0.8699746408919	1.0261466400103
0.8700982904706	1.0261650850659
0.8702337443076	1.0263025291804
0.870347560612	1.0263728051458
0.8704529060463	1.026410412258
0.8705478167322	1.0264745085664
0.8706694585834	1.026612711536
0.8707684875274	1.0266550339763
0.8708921302132	1.0266641652866
0.8709885512318	1.0266982386169
0.8710657762434	1.0270532145298
0.8711620487058	1.0270858934255
0.8712702283588	1.027145432865
0.8713959865889	1.0272768679995
0.8715072282702	1.0273075353992
0.8716103793399	1.0273502498453
0.8717223339805	1.0275394447467
0.8718356378982	1.0277203790205
0.8719636447592	1.0277348867427
0.8720712078589	1.0278115106399
0.8721769992467	1.0280072299268
0.8722944105226	1.0282118185003
0.8724036266295	1.0283691995414
0.8725014457616	1.0284442155964
0.8725988135741	1.0284453644643
0.872727771933	1.0286529161932
0.8728280079976	1.0286810522464
0.8729206615693	1.0287399850732
0.873011981563	1.0288756601494
0.8731301200818	1.0289224078506
0.8732405660633	1.0289941596314
0.8733600588485	1.0290493985632
0.8734505672052	1.029514526375
0.8735522181317	1.029735660454
0.8736712592444	1.0299172015411
0.8737892595876	1.0301089472087
0.8738940121101	1.0301795665289
0.8739925596047	1.0303481118587
0.8740935850418	1.030374869439

0.8742104273867	1.0306157000749
0.874324952078	1.030772043426
0.8744403293608	1.0312058172454
0.8745418046228	1.0314306570919
0.8746379001021	1.0316562148057
0.8747370866703	1.0317942234902
0.8748433384009	1.0318438409148
0.874958674772	1.031925156869
0.8750761557776	1.0320510972461
0.8751911322084	1.032251186216
0.8752997216231	1.0324759344919
0.8754238651275	1.0325719439568
0.8755453626414	1.0327316234242
0.8756654136744	1.0328087454385
0.875787320616	1.0329883075789
0.8759039111925	1.033119303498
0.8760191759492	1.0331388006905
0.8761466323503	1.0331681938961
0.8762746047678	1.0333001219932
0.8763964348131	1.0334165611175
0.8765242070096	1.0334243142281
0.8766237735095	1.0334330658075
0.876718815658	1.0340267785189
0.8768356681921	1.0343762894091
0.8769477332668	1.0345418842987
0.8770338459743	1.0350049885527
0.8771411726966	1.0350358273625
0.8772545405305	1.0350575835802
0.8773773855896	1.0350863443509
0.8775013503189	1.0353844431476
0.877607729829	1.0354176612666
0.8777220262973	1.0354560936607
0.8778467344059	1.0356174513688
0.8779568758263	1.0356638490991
0.8780683999271	1.0357441071613
0.87817770135	1.0357923984563
0.8782841344483	1.0359362038652
0.878394971066	1.0360494263427
0.8785014810613	1.0360691368947
0.8786059678035	1.0363922670153
0.8787147380862	1.0365572420215
0.8788489370611	1.0365896268102
0.8789569065534	1.0366128808897
0.8790544279915	1.0367109372018
0.8791599361268	1.0367221285497
0.8792725396511	1.0367851052126
0.8793946213823	1.0368192704748
0.8794973250283	1.0370216864935
0.879610142309	1.0371113107902
0.8797208901799	1.0371255407137
0.8798545383798	1.0371520372106
0.8799612922253	1.0371597513169
0.8800707440482	1.0371843570473
0.8801810736909	1.0373833757902

0.8802937913952	1.037447585058
0.8804145115167	1.0375140528552
0.8805484332234	1.0375565475995
0.8806720997216	1.0377426660604
0.8807539283621	1.0384481936216
0.8808638197097	1.0387134811587
0.8809766200282	1.038757491227
0.8811055630642	1.0388278785627
0.8812153328825	1.0390595171068
0.8813061766997	1.0390858798765
0.8814202874242	1.0393441508865
0.8815279972386	1.0393940729253
0.8816427590278	1.0395754668357
0.8817361773991	1.0399366225958
0.8818392013059	1.0401724436965
0.8819595226228	1.0402393616154
0.8820671331064	1.0403676730246
0.8821687240529	1.0405837488302
0.8822803596606	1.0407113798354
0.882384301624	1.0407731159945
0.8825084135062	1.0410146591488
0.8826094284191	1.0412779202512
0.8827249693959	1.0413286284923
0.8828280163226	1.0414234006525
0.8829309434204	1.0423315400551
0.8830336515741	1.0424131956134
0.8831476393801	1.0426068140432
0.8832616900007	1.0428816177689
0.8833659154801	1.042986378278
0.8834620013029	1.0433633921837
0.8835702810523	1.0439427347494
0.8836772603802	1.0439571131467
0.8837995369599	1.0441532548729
0.8839196974782	1.0442756338493
0.8840395344946	1.044519649513
0.8841516814156	1.0446834466904
0.884285571486	1.0454370917895
0.8843866135722	1.0455746685616
0.8845084703423	1.0456485058276
0.8846189395794	1.0457452770631
0.8847236740914	1.0458012893483
0.8848256937885	1.0458221599005
0.8849537917917	1.045862916488
0.885062887808	1.0459835434955
0.8851808359749	1.0462375052495
0.885276362749	1.0464350392228
0.8854032419709	1.0465204231971
0.8855137010158	1.0466436149065
0.8856421092178	1.0466972379677
0.8857614528048	1.0467551308335
0.8858742447391	1.0467562962552
0.8859906390761	1.0472885852363
0.886083172513	1.0473139313746
0.8862017706439	1.0476127093147

0.8863164340518	1.0479478783849
0.886433710893	1.0479525020321
0.8865327268375	1.0479696141023
0.886643698398	1.0480400194684
0.8867649348299	1.0481579960802
0.8868586158364	1.0486576391276
0.8869859954467	1.0489706719851
0.8871024342286	1.0491559778948
0.8872137129711	1.0495915923862
0.8873057732091	1.0497491939034
0.8874170864036	1.0498392708875
0.8875459864384	1.0500132649185
0.887667328849	1.0500912059648
0.8877653514186	1.0501987997386
0.8878715047289	1.0502469148729
0.887985396102	1.050391154528
0.8881047646637	1.0505002076673
0.8882072712711	1.050597755364
0.8883056739675	1.0506847808516
0.888411638589	1.0508348924121
0.8885090592833	1.0508906202595
0.8886122147378	1.0509213558902
0.8887315039674	1.0509281611264
0.8888658313452	1.0510312630064
0.8889869338834	1.0512433897661
0.8890894069264	1.0513312368656
0.8891867045759	1.05150518944
0.8892818286956	1.0515991218899
0.8893881100888	1.0516744651383
0.8894962009567	1.0518906472163
0.8895879140713	1.0519772328755
0.8896843147319	1.0519825471389
0.8897941230582	1.0519994059649
0.8899117872282	1.0520243056889
0.8900177314228	1.0524190528319
0.8901352241727	1.0524664064655
0.890257962439	1.0527396815664
0.890372360722	1.0529118461254
0.8904860287049	1.0531809198024
0.8905948421697	1.0534405777101
0.8907048587128	1.0535128238792
0.8908281678552	1.0535584022005
0.890928131174	1.053590162567
0.8910380689828	1.0536706163048
0.8911327392598	1.053847589375
0.8912430999085	1.0543126718654
0.8913449963441	1.0544495688594
0.891450848951	1.0546964460061
0.8915520823365	1.0547039294363
0.8916491732363	1.054863463452
0.8917537441366	1.0548885554874
0.8919001434576	1.0551099920987
0.8920134923496	1.0552630477818
0.8921205472417	1.055344441007

0.8922493860165	1.0553752189188
0.8923437986614	1.0555248390875
0.8924730973081	1.0557088675569
0.8925591348167	1.0560443013041
0.8926892779895	1.0561567209833
0.8928033049183	1.0565493176433
0.8929079606929	1.0567575827904
0.8930208938102	1.0568408226816
0.8931229016311	1.0570162475375
0.8932264599221	1.0570562523601
0.8933206746698	1.0574480367639
0.8934373709432	1.0574802342392
0.8935379912738	1.0575432756273
0.8936662847674	1.0577218949798
0.8937729915486	1.0578875073066
0.893882892858	1.0579791575283
0.8939926275994	1.0580684715749
0.8940978049005	1.0586269257345
0.8942035510154	1.0587786203559
0.8943259312241	1.0588722600034
0.8944250671311	1.0590050776568
0.8945345456811	1.0591202839961
0.8946405592443	1.0592748454822
0.8947694181154	1.0592838749484
0.8948844941285	1.0595083906667
0.8949834281817	1.0597111640749
0.8950980233557	1.0601697811692
0.8951977166828	1.0601748671136
0.8953124758223	1.0601793783191
0.8954680424943	1.0604763629196
0.8955697935798	1.0609454488708
0.8956706533677	1.0610806402689
0.8957659639962	1.0611681861764
0.8958657837992	1.0613064362355
0.8959846351827	1.0614875151558
0.89610345159	1.0618075172687
0.8962160247743	1.0618519995543
0.8963412605462	1.0620369218631
0.8964536440472	1.0627142541458
0.8965428617395	1.062745638415
0.8966829689894	1.0627625445308
0.8968042920399	1.0630269602178
0.8969149377597	1.0630923784658
0.8970338264267	1.0630944013583
0.8971429001431	1.0635856095986
0.8972522305546	1.0637072326076
0.897351031478	1.0637137728308
0.8974610298996	1.0638448759621
0.8975936806381	1.0639333286896
0.8977014859055	1.064103717803
0.8978091988737	1.0642594996568
0.8979341746099	1.0644111876712
0.8980581372009	1.0644831898688
0.8981800005328	1.0644960391767

0.8982839481418	1.0645417306691
0.8984058685717	1.0645775772904
0.8985128148022	1.0646104107982
0.8986235510964	1.0647082041959
0.8987407920979	1.0651205699437
0.8988698660383	1.0651286742837
0.8989855919711	1.0652214470227
0.8990907286322	1.0653830872302
0.8991950210023	1.0654150455535
0.8993137230802	1.0655023789884
0.8994314386832	1.0655442139553
0.8995274926373	1.0657367013326
0.8996383626244	1.0658759128379
0.8997418139047	1.0659836130188
0.899847211831	1.0660911925419
0.8999701163172	1.066123885384
0.9000989136817	1.0664056157624
0.9002053750488	1.0664872661197
0.9003171099881	1.0666654422932
0.9004413304395	1.0667591460376
0.9005407216295	1.0667923990478
0.9006708084309	1.067195488268
0.9007764391638	1.067451465354
0.9008830611225	1.0677707333773
0.9009835854137	1.0677800583414
0.9011024859731	1.0678007751977
0.9012226488574	1.0680238859896
0.9013331483083	1.0680551168298
0.9014583174316	1.0682694931577
0.9015772772508	1.0683072385611
0.9016768512438	1.0683381621321
0.901784487287	1.0684198666364
0.9018886501226	1.0685421658665
0.901990524213	1.0689345545954
0.9021193328911	1.0689442266756
0.9022231148732	1.0691043122925
0.9023225255206	1.0691282606399
0.9024341351361	1.0691741657481
0.9025584452182	1.0696398032556
0.9026576160425	1.0697584761401
0.9027663222513	1.0698255203386
0.9028848000277	1.0698263776136
0.903002954264	1.0700484676512
0.9031108342753	1.0701390526117
0.9032243316564	1.0703031762913
0.9033145664386	1.0704693321721
0.9034164622526	1.070611927921
0.903524027811	1.0706146378013
0.9036263960483	1.0706297036645
0.9037620152032	1.070639741185
0.9038827739052	1.0707296623013
0.9039978323395	1.0709172980431
0.9041247518675	1.0713956920563
0.9042497557303	1.0716420218378

0.9043698666932	1.0717471240692
0.9044759391049	1.0717902886308
0.9045872252504	1.0719859330146
0.9046783060743	1.0720039420844
0.9047986673315	1.0722516531064
0.9049354256871	1.0725273219871
0.9050322655744	1.0727111200692
0.9051354184763	1.0727478963108
0.9052649195672	1.0732429411151
0.9053800586188	1.0733196007675
0.9055015403882	1.0733804591593
0.9056171554671	1.0736607716662
0.9057477256795	1.0738402246262
0.9058524588317	1.0739008975671
0.9059809353071	1.0743375747694
0.9060988791881	1.0745563924232
0.9062112267128	1.0748681814217
0.9063322764958	1.0749289177575
0.9064493848055	1.0756791497706
0.9065646030999	1.0758687393745
0.9066625867968	1.0761999649556
0.9067667158088	1.0762620116858
0.9068616544155	1.0765016293562
0.9069756074936	1.0765479589999
0.9071143096851	1.076629946853
0.9072233349219	1.0767622956158
0.9073225738825	1.0768954988369
0.9074687380522	1.0769370381357
0.9075813284039	1.0769587879915
0.9077248190425	1.0770928510182
0.9078292468406	1.0771060290915
0.907937411356	1.0775545436708
0.9080338615703	1.0776889729279
0.908157621624	1.0778215169902
0.9082640338968	1.0779916882615
0.9083744388417	1.0782005179277
0.9084950174011	1.0782574907106
0.9086009704257	1.0786989100812
0.9086906962362	1.0788818885663
0.9087934876614	1.0791747148061
0.9089008619034	1.0794560179223
0.9090004420903	1.0795238245557
0.9091139761987	1.0797167619115
0.9092269267794	1.0797212918097
0.9093338630977	1.0801559196295
0.9094594503479	1.0801724636965
0.9095490130723	1.0802438343089
0.909664923321	1.0804380600694
0.909759122095	1.0805129010333
0.9098747403928	1.0805962950366
0.9100065793945	1.0806224412974
0.910147366697	1.0806944304579
0.9102592902726	1.0807011686811
0.9103769118725	1.0807936473898

0.9104836457864	1.0808091650778
0.9105765016864	1.0808195414573
0.9106659927708	1.080847787016
0.9107679531235	1.0809658405519
0.9108746465968	1.0813696417052
0.9109770851498	1.0814609375828
0.9110784160164	1.0815723773192
0.9111770280509	1.0816665759917
0.9112883457102	1.0819390289237
0.9113849357735	1.0821296415621
0.9115095448797	1.082387044653
0.9116296639729	1.0829253095165
0.9117254793913	1.0829906328322
0.9118209062515	1.0834416858023
0.9119188406487	1.0834614953034
0.9120377862864	1.0834694789935
0.912146211142	1.0835322263956
0.9122652406186	1.0835608389024
0.9123674137382	1.0835973114533
0.9124723294619	1.0838992918983
0.9125730046575	1.0843586124351
0.9126726673009	1.0846540621703
0.9127776020046	1.0847677071742
0.912889645633	1.0848058362125
0.9130138046784	1.0849347782955
0.9131278174087	1.0849796057055
0.9132408045676	1.0851051628819
0.9133453846611	1.0851921464474
0.9134575426208	1.0852914998076
0.9135613255015	1.0854978465972
0.9136665978058	1.0855281861028
0.9137708515185	1.085562258894
0.9138731388089	1.0855844881464
0.913997273696	1.0858580257551
0.9141181964666	1.0859849033815
0.9142358911359	1.0861975631127
0.9143307332813	1.0862908077582
0.914444869615	1.0863625433848
0.9145709170011	1.086560038831
0.9146827759721	1.0867686823773
0.9147854413699	1.0869387625351
0.9148910126965	1.0870268657502
0.9149948891644	1.0870721182468
0.9151163201101	1.0872052441364
0.9152646074412	1.0880221172724
0.9153638597858	1.0880984429416
0.9154816082749	1.0881181792591
0.9156151545545	1.0882784654998
0.9157154265507	1.0883729340542
0.9158365413261	1.088411798588
0.9159794654346	1.0884944552074
0.9160927560799	1.0886622849884
0.9162021633314	1.0900008023936
0.9163254450055	1.0900553459024

0.9164228077287	1.0900612168172
0.9165422985	1.0900652942844
0.9166561268466	1.090095000081
0.916773783053	1.0901041357597
0.9168674231832	1.0903921491098
0.9169807774017	1.0903938173942
0.9171080014567	1.0904500074451
0.9172181830739	1.0906158902622
0.9173200780709	1.0907269871268
0.9174395093835	1.0907309861267
0.9175515827256	1.090828196232
0.9176551660923	1.0915003496811
0.9177607576068	1.0915596408017
0.9178470473964	1.0916031731547
0.9179969270608	1.0917992443964
0.9181114602627	1.0918856808112
0.9182494067315	1.0924890962925
0.9183393787291	1.0925837209423
0.9184348462587	1.092689202276
0.9185458035618	1.0927373439471
0.9186810130268	1.0934314474243
0.9187928217208	1.093623801975
0.9189099679647	1.0937158646051
0.9190159085101	1.09372535434
0.9191212613859	1.0938802927223
0.9192126895114	1.0938844030203
0.9193155520456	1.0939785360873
0.91941434727	1.0945327018483
0.9195267050774	1.0947409014633
0.9196479549977	1.0947475141018
0.9197607531028	1.0948179906627
0.919860256323	1.0955226004145
0.9199751881606	1.0956121504936
0.920092579926	1.0956566402037
0.9202082165277	1.0957822224167
0.9203177812544	1.0958483842568
0.9204309752079	1.0960985945553
0.92054925211	1.0962225959454
0.9206538212071	1.0962425885562
0.9207565797535	1.0964977417575
0.920886523299	1.0968381190856
0.92099818572	1.0974061206186
0.9211175685393	1.0974889421281
0.9212028051757	1.0975714660606
0.9213206656479	1.0976092548025
0.9214376993165	1.0976252649249
0.9215731678479	1.097648177735
0.9216855247179	1.0978069951815
0.9217995086619	1.0981090349447
0.9219112839687	1.0981336634656
0.9220282012309	1.0983276743645
0.9221446211769	1.098602221968
0.9222441108682	1.0986733395203
0.9223561763237	1.0990125708563

0.922477410515	1.0990265373511
0.9225853770077	1.099180564168
0.9227065825554	1.0993262142486
0.9228078890569	1.0995718974827
0.922908439014	1.0997109827287
0.9230277404768	1.0997556380218
0.9231337672348	1.0997819014971
0.9232679681286	1.0999440228448
0.9233679626883	1.0999619900097
0.9234877644716	1.0999756446436
0.9235849812259	1.1001611876127
0.9236958442054	1.1007082851738
0.9238060282861	1.1010093796938
0.9239059242656	1.1014559702093
0.9239824081092	1.1014844778588
0.924076165536	1.1020870600386
0.9241988246292	1.1021385803603
0.9243028686051	1.102250626786
0.9244023839342	1.102256786636
0.9245166013729	1.1024575317998
0.9246076543042	1.1031023787262
0.9247319220161	1.1034239832826
0.9248717643905	1.1035743510071
0.9249843537977	1.1037013417538
0.925088547338	1.1040623853871
0.9251968530894	1.1042786193485
0.9252989503598	1.1042870228692
0.9254096488697	1.1043375024199
0.9255203981517	1.1046329392086
0.9256176019144	1.1048243091921
0.9257289153894	1.1048882786221
0.9258312045283	1.1049852304272
0.9259509895506	1.1049963900836
0.9260791029303	1.1050394644639
0.9261894305261	1.1051176236218
0.9263280548571	1.1051903014921
0.9264440483051	1.1052148252456
0.9265423674786	1.1052727691574
0.9266474347136	1.1052807695869
0.9267700905001	1.1052932558432
0.9269003393378	1.1055540033442
0.9270479251956	1.1055965993953
0.9271516827315	1.1058278315619
0.9272627664234	1.1058943204409
0.9273521511369	1.1059194953091
0.9274538627379	1.1061681414616
0.9275494245557	1.1062886849783
0.9276725698328	1.1064263762321
0.9277940855489	1.1064878665994
0.9279098937689	1.106491178263
0.92802138822	1.106630068954
0.9281351246984	1.1069820305327
0.9282301546235	1.1070207584654
0.928366007383	1.1072248877019

0.928484236306	1.1073895160544
0.9286092324308	1.1076728091782
0.9287187211404	1.1077075089418
0.9288334731423	1.1079206912782
0.9289412205779	1.1079800807595
0.9290476746572	1.108000440858
0.9291833058256	1.1083400790524
0.9292905779313	1.108479483913
0.9294127641948	1.109047045946
0.9295248021179	1.1093070454156
0.929649380528	1.1096472360406
0.9297561314681	1.1097250419588
0.9298817692811	1.1097910953912
0.9300165549653	1.1098657763209
0.9301297880941	1.110063574185
0.9302680430698	1.1101614381633
0.930381817844	1.1101771260994
0.9304749057073	1.1102561863524
0.9306177545568	1.1104080376066
0.9307344658972	1.1106134980308
0.9308370795075	1.1111024659838
0.9309306410767	1.1111119821299
0.93102568418	1.1111188035863
0.9311429879007	1.1113828380796
0.9312519919776	1.1115448044419
0.9313531446873	1.1116681022979
0.9314622605502	1.111781515741
0.9315859739358	1.1119945250773
0.9317209524295	1.1122923580443
0.9318214450269	1.1126252384995
0.9319271609407	1.1126673126537
0.9320471733272	1.112782092618
0.9321808337716	1.1131968001619
0.9322930407731	1.1132651986163
0.9324061919128	1.1135104904973
0.9325315298951	1.1135863252016
0.9326279362757	1.1138517745174
0.9327333660137	1.114255894317
0.9328518248041	1.1144490900025
0.9329632231362	1.1145882391439
0.933084681728	1.1146808837564
0.933205281465	1.1147981615753
0.9333365229349	1.1149628595694
0.9334627823315	1.1150704355532
0.933594555693	1.1151711030013
0.9337022705962	1.1152132485857
0.9338375431153	1.1152856706887
0.933965641006	1.1158189988393
0.9341031267336	1.1159687417435
0.9342030254238	1.1162247624864
0.9342986526217	1.1164208649673
0.9344086393311	1.116590097866
0.9345273861737	1.1166304610609
0.9346139045125	1.1167524011566

0.9347453485308	1.1169297502496
0.9348609471734	1.1171173985378
0.9349712962174	1.117142840595
0.9350610018949	1.1175556388004
0.9351753929832	1.118201306801
0.9352898672921	1.1183184140268
0.9353907685753	1.1188098284665
0.9355289621726	1.1188619156713
0.9356465025983	1.1188926172273
0.9357889346895	1.1190109078117
0.935902360701	1.1191475724215
0.9360125465793	1.1193021042966
0.9361439414792	1.1193205579198
0.9362729838868	1.1194868491113
0.9363921524564	1.1194872659271
0.9365159856442	1.1196944138769
0.9366520929238	1.1203742448515
0.9367715373274	1.1203957099029
0.936904591419	1.1205588666448
0.9370078169711	1.1206046366408
0.9371199147361	1.1206497681271
0.9372227553881	1.1206565358463
0.9373629088614	1.1208118701886
0.9374850358731	1.1209985557386
0.9376082051723	1.121071235005
0.9377187387156	1.1210756014656
0.9378333712357	1.1212101224303
0.9379583375509	1.1213858947021
0.9380835846021	1.1215641144042
0.9381761766714	1.1216630904832
0.9382859917128	1.1223472437999
0.9384030710552	1.1223633520117
0.9385301226822	1.1223639360488
0.9386303821915	1.1223656542515
0.938744962197	1.1223787496927
0.9388526697954	1.1224372876136
0.9389351533703	1.122450163782
0.9390370286357	1.1225816033846
0.9391701858907	1.1227681261392
0.9392880721753	1.1229048516681
0.9394061296683	1.1233982478204
0.939522676139	1.1234419443605
0.9396093920185	1.1235912027459
0.9397033789065	1.1240375119746
0.9398369022697	1.1240488123597
0.9399554814704	1.1244988041913
0.9400730601967	1.1245219013425
0.9401711281499	1.1246149453151
0.9402802743484	1.1248963994182
0.9403853561575	1.124916045564
0.9404947291478	1.1250559481566
0.9406331788511	1.1251196721099
0.9407534936551	1.1254588238685
0.9408640660822	1.1254898450375

0.9409733919986	1.1255117595268
0.9410840952109	1.1255400050839
0.9411857942752	1.1258407358336
0.9412816009507	1.125995378254
0.9414062625319	1.1260953582906
0.9415211649988	1.1263251709406
0.941639152536	1.1263722882553
0.9417395487979	1.1269489467498
0.9418402157842	1.1270414307538
0.9419550457671	1.1272872789498
0.9420765645836	1.1274385721466
0.9421700520735	1.1275779881108
0.9422926667845	1.127611148146
0.9423996778743	1.1276337037388
0.9425074199416	1.1276944680389
0.9426172335885	1.1279363352716
0.9427507872862	1.1280418192456
0.9428864037975	1.1281686577725
0.9429821514189	1.128487254695
0.9431188607813	1.1285247356441
0.9432340980753	1.1285481300339
0.9433535689641	1.1287254505716
0.9434799214993	1.1288030773557
0.9435759450397	1.1291589288523
0.9436967170912	1.1292512568293
0.9438195886457	1.129428147189
0.9439473899973	1.1295422216892
0.9440673806162	1.1297189327798
0.9441828238474	1.130394557848
0.9442799346942	1.1304070238685
0.9443931485074	1.1304352083936
0.944532682554	1.1304783466219
0.9446593525113	1.130857255851
0.944765245658	1.1313714675554
0.9449019712904	1.1314049257643
0.9450289571945	1.1316192880548
0.9451169851875	1.13176110379
0.9452304825247	1.1318253150161
0.9453654318799	1.1321334360713
0.9454944562075	1.1322333819056
0.9456077656617	1.1325346598154
0.9457369685014	1.1326931890134
0.9458542139426	1.132760493827
0.9459875258781	1.1327697015098
0.9461057465049	1.1332683970581
0.9462034346908	1.1333922294897
0.9463297501889	1.1334333834011
0.9464565267003	1.1335173539849
0.94656153889	1.1335242301848
0.9466715766313	1.1337892431177
0.946794160084	1.1338245785435
0.9469309702647	1.1341223570191
0.9470549415181	1.1342804719912
0.9471410812751	1.1344407035767

0.9472419892508	1.1348178329994
0.9473710876519	1.1349001582755
0.947484665456	1.1350192723971
0.9475962001379	1.1350998992256
0.9477126795461	1.1351807178626
0.9478342618538	1.1352407385557
0.9479549747656	1.1352606685704
0.9480757421783	1.1353587747533
0.9482040637829	1.1356313047314
0.948339626218	1.1356966877487
0.9484391541307	1.1358493622183
0.9485595403423	1.1359091404672
0.9486526824144	1.1359098766432
0.948765750068	1.1359199798185
0.9489430205471	1.1359381799601
0.9490657618998	1.1361054390556
0.9491863209722	1.1362678480317
0.949288627072	1.1364938356252
0.9494184079849	1.1368652785535
0.9495244745233	1.1371078191847
0.9496173498552	1.1373151170834
0.9497431435207	1.1379761250447
0.9498704426788	1.1379809159412
0.9499991739552	1.1383398938684
0.9501009324027	1.1386271725976
0.9502319835823	1.1386815912834
0.9503535263668	1.1387009383907
0.9504848447444	1.1387593095928
0.9505825915403	1.1388938240115
0.9507038190359	1.1390494280901
0.9508065864407	1.1392731766922
0.9509267731606	1.139559062905
0.9510334493766	1.1395702591526
0.9511687091606	1.1397147928549
0.9512749918629	1.1398085786337
0.9513944926038	1.1398623357175
0.9515283626474	1.1399989182796
0.9516479064832	1.1402562181889
0.9517491398171	1.1410603237004
0.951869089214	1.1413045318865
0.9519724346271	1.1416003381386
0.952064624453	1.1416592426963
0.9521853365587	1.1420253145783
0.9523033037922	1.1424151440716
0.9524129780688	1.1424680962603
0.9525232879592	1.1425500529054
0.952638505939	1.144001566282
0.9527371719249	1.1445102891496
0.9528452967706	1.1450366820377
0.9529663066138	1.145533649153
0.9530993727722	1.1456336814782
0.9532039602308	1.1456775399222
0.9533131014762	1.1459744122882
0.9534134100019	1.145987855539

0.9535116881478	1.1460491443391
0.9536276951222	1.1462840261435
0.9537536893641	1.1463884498977
0.9538597119144	1.1464066389263
0.9539878900687	1.146443910925
0.9541012066418	1.146997383897
0.9542302770188	1.147261275074
0.9543551656971	1.147498671773
0.9544528937729	1.1476804112767
0.9545627457649	1.1477770052399
0.9546935553516	1.1478273693165
0.9548273089834	1.1481641510948
0.9549376300778	1.1485336865527
0.9550609491468	1.1486413296617
0.9551646604419	1.1486564092395
0.955254693314	1.1486823194196
0.9553717641	1.1488015334501
0.9554973832342	1.1490610752044
0.95563029863	1.1492796102958
0.9557426980573	1.1494769365626
0.9558525121899	1.1494838404669
0.9559842275018	1.1495157636577
0.956117087763	1.1496155310658
0.9562295920241	1.1498619419249
0.9563375241137	1.1500095383432
0.9564654515389	1.1505436361833
0.9565710508084	1.1505869881061
0.9567029997856	1.1506576331623
0.9568174773743	1.1508840148263
0.956950458126	1.1509055521224
0.9570940617204	1.151450452943
0.9571931960429	1.1515106101088
0.9572987229284	1.1515595966962
0.957409724561	1.1521363851565
0.9575418595186	1.1523096096716
0.9576634483748	1.1523428163123
0.9577893456831	1.152360877939
0.9579131235833	1.1523835290852
0.9580250092553	1.1524470823746
0.9581324070576	1.1525832830619
0.9582376232579	1.152814261438
0.9583556979045	1.1529241536834
0.9584901271936	1.1532841587834
0.9586003425391	1.1533625325471
0.9587341853886	1.1536537038561
0.9588276300528	1.1536873678881
0.9589482034238	1.1538405138052
0.9590789155006	1.1540654757383
0.9592070524332	1.1541054312577
0.9593237459223	1.1545035880048
0.9594285857919	1.1545906176111
0.9595587240613	1.1546336293551
0.959669007638	1.1550209598834
0.959767016052	1.1550784609656

0.9598818789955	1.1550863045283
0.9600079634266	1.1553114981222
0.9601248581476	1.1553787693934
0.960285321626	1.1555096656377
0.960393964848	1.155640963036
0.960503880495	1.1560973016804
0.9606185385575	1.1561729671695
0.9607240143744	1.1563940267681
0.9608283702782	1.1565407325921
0.9609345503565	1.156574081587
0.9610530864647	1.156657136296
0.9611986575614	1.1569531482601
0.9613096877221	1.1576028087833
0.9614152330202	1.1576028390358
0.9615408281392	1.1577586517798
0.9616458254946	1.1581207285051
0.9617857300597	1.1581872780442
0.9619039956167	1.1583407028315
0.9620201157334	1.1586089693983
0.9621261093792	1.158775489051
0.9622226131556	1.1591129937096
0.9623549716556	1.1593356179679
0.9624692127277	1.1595087119049
0.9626062475231	1.1596100930089
0.9627194241112	1.1598340113194
0.9628429309797	1.1602922585913
0.9629564386939	1.1605364400233
0.963082512891	1.1605854547314
0.9632020569098	1.1609004634113
0.9633245701361	1.1610199438039
0.9634288242297	1.1614848098667
0.9635232146639	1.1615437923226
0.9636222543783	1.1615854349181
0.9637592687147	1.1616182517107
0.963856308012	1.1617893817562
0.9639753536286	1.1619283099578
0.9640778522925	1.1623463274404
0.9641861338947	1.1623627404908
0.9643228003164	1.1623693968514
0.9644460192923	1.162419580411
0.9645727451391	1.1625172885075
0.9646792115524	1.162551314146
0.9647974354866	1.1627105081708
0.9648993803929	1.1629210775042
0.9650144612153	1.1632562905
0.9651311141817	1.1633169146035
0.9652308689496	1.1634732774502
0.9653657337536	1.1640292128896
0.9654821299749	1.1643753354344
0.9656208823805	1.1645751729884
0.9657423436079	1.1646525004024
0.9658685965658	1.1650429608478
0.9659899505149	1.1650483196413
0.9661178965393	1.1651390475621

0.9662195128661	1.1664331975258
0.9663269084142	1.1664660517974
0.9664674627578	1.1665593891782
0.9666064431633	1.1665656512979
0.9667211617896	1.1667543503578
0.9668662133639	1.1671634970262
0.9669771317063	1.1674797061126
0.9670999359484	1.167506613001
0.9672032585473	1.1675871884589
0.9673290798725	1.1677484294886
0.9674474720401	1.1677831318003
0.9675642035571	1.1678058482324
0.9676767974466	1.1678483577498
0.9677819412045	1.1678634495303
0.9679130076656	1.1682470612998
0.968028047981	1.1689063826091
0.968154307834	1.168981138178
0.9682740085537	1.1690842498162
0.9683962170602	1.1697283868482
0.9685077509969	1.1703842891937
0.9686147235805	1.1704836638874
0.9687369919396	1.1705547753927
0.9688564408085	1.1707356595012
0.9689687209085	1.1708022096318
0.9690995005214	1.1711953749763
0.9692159453356	1.1714596945102
0.9693199052906	1.1715317431955
0.9694584318931	1.1719000051809
0.9695535121882	1.1720583402918
0.9696999638495	1.172471383803
0.9698301744204	1.1726617976025
0.9699500856212	1.1729297128889
0.9700784099843	1.1733464696194
0.9702189035204	1.1739516088531
0.9703245020157	1.1740562836917
0.9704369060562	1.1741573957101
0.970562353953	1.1741609317958
0.9707033281853	1.1742274778265
0.9708457116164	1.1742797499714
0.9709780159514	1.1742887875372
0.971114630222	1.1745411649116
0.9712260461841	1.1745510230969
0.9713371505816	1.1747610177363
0.971446980746	1.174797989428
0.9715627556515	1.1749121093718
0.9716841157624	1.1749314952819
0.9717863686501	1.1749683021792
0.9718871985999	1.1751262460404
0.9719931856463	1.17516536064
0.9721248741262	1.175255966766
0.9722411634947	1.1753188066591
0.9723551425657	1.1756724305167
0.972460840145	1.1763455568808
0.9725930961582	1.1768590263081

0.9726973093809	1.1768866950527
0.972838063041	1.177015916213
0.9729603454749	1.1774226769775
0.9730864017838	1.1775053607038
0.9731894997763	1.1776138706906
0.9733242613519	1.1781210396587
0.9734484537894	1.1781723627591
0.9735863356871	1.1783583501025
0.9737108364015	1.1785573146176
0.9738326850456	1.1785734105975
0.9739469233474	1.1786149885873
0.9740849955448	1.1786331612029
0.9742343526206	1.1786712603023
0.9743389069926	1.1788259523835
0.9744500152188	1.1788512363551
0.9745755226248	1.1789204413875
0.9746850183315	1.1789309247639
0.9747964588744	1.1790023217089
0.9749202120285	1.1791236781379
0.9750363960844	1.1794671389417
0.9751453493987	1.1796450033691
0.9752613629998	1.1800245084417
0.9753941903055	1.1804027567976
0.9755228617802	1.1804777105202
0.9756399776796	1.1804806780657
0.9757659165672	1.1807889888369
0.9758782076191	1.1811036685066
0.9760051804469	1.1811701632458
0.9761494022673	1.1813875846357
0.9762578757468	1.1819783077274
0.9763903368818	1.1819856635811
0.976487271504	1.182282069182
0.9766092214062	1.1823315759018
0.9767099737878	1.1827141171728
0.9768298756855	1.1828468238508
0.9769405112457	1.1830667957634
0.9770562011326	1.1832464139656
0.9771689589314	1.1834340923002
0.9772725643438	1.1835729222811
0.9773756630734	1.1836279712987
0.977504435315	1.1839451865377
0.9776154054693	1.1839966145415
0.9777313875896	1.1841067253415
0.9778291191986	1.1841480676474
0.9779486468336	1.1849376524025
0.9780808006566	1.1850600033389
0.9782216000515	1.1852289166203
0.9783345918763	1.185423899418
0.9784703435093	1.1854716690564
0.9785789519929	1.1856351068095
0.9786836037296	1.1857109903041
0.9788064721708	1.1858609992603
0.978927676967	1.1863519044152
0.9790282770756	1.1867367432355

0.9791437404178	1.1867905219713
0.9792476076076	1.1868364677595
0.9793658936804	1.1869052476395
0.9794945342585	1.1870645589821
0.9795988415177	1.1871015300008
0.9797153291733	1.1872147552033
0.9798448177839	1.187541814189
0.9799480970433	1.1875603423601
0.9800716088223	1.1877118875751
0.98019728116	1.1879016879306
0.980302774264	1.1885160865145
0.9804236412925	1.1885738219348
0.9805952652518	1.1885912609382
0.9807190171887	1.1886555501287
0.9808557196871	1.1886598333284
0.9809651754195	1.1888607368593
0.9810971477098	1.188922088519
0.9812153388994	1.1890358521662
0.9813280888583	1.1892757593833
0.9814430278744	1.1893483703298
0.9815597864838	1.1895088304186
0.981675718783	1.189601390162
0.9817864342295	1.1899743754385
0.9819022147509	1.1900522039438
0.9820261486026	1.1900585592718
0.9821279010737	1.1902850836282
0.982236042194	1.1903078327448
0.9823433030717	1.1909198176156
0.9824538785813	1.1912545188493
0.9825569449737	1.1912571614788
0.9826844185617	1.1912606708351
0.9828046718176	1.1921219404126
0.9829269859696	1.1922251945324
0.9830455313834	1.1923777270984
0.9831907062917	1.192591662799
0.9833233876601	1.1926864160512
0.9834318483355	1.1927071703635
0.9835526698316	1.1927406283196
0.9836610060191	1.1928772327233
0.9837930414817	1.1930511689541
0.9838826867032	1.1931587304434
0.984046005572	1.193499590518
0.9841561468551	1.193674504607
0.9842683907768	1.1938357424246
0.9843856209306	1.1939013553984
0.9844865966702	1.1940021739977
0.9845927814408	1.1940380539474
0.9847176335711	1.194043613819
0.9848358187083	1.1943188142276
0.9849532626115	1.194550913884
0.9850671728993	1.1946097882026
0.985186284566	1.1955888712082
0.9852945380459	1.1957489455923
0.9854109690559	1.1958342278335

0.9855547752817	1.1964945552902
0.9856633183226	1.1967121216351
0.9857756156334	1.19683558401
0.9858930114587	1.1970261780691
0.9860040823129	1.1970310382768
0.9861295553624	1.1971984047717
0.9862587838393	1.1974448947923
0.9864108778769	1.197542579313
0.9865356850137	1.1980838874946
0.9866474923146	1.1981995751289
0.9867789880095	1.1982169143633
0.9869125364786	1.1982918993087
0.9870227255622	1.1984511485712
0.9871355295481	1.1985504424096
0.9872669871092	1.198607480385
0.9874032139452	1.1987783674371
0.9875207613749	1.1988368455862
0.9876527695478	1.1989476297416
0.9877672955394	1.1992610458718
0.9878892749184	1.1993499842247
0.9880058958222	1.1995189659802
0.9881309018047	1.1996001881144
0.9882476066245	1.1997285086592
0.9883730828953	1.1999567470366
0.9884830003938	1.2001464255135
0.9886018563093	1.2003695789615
0.9887318817823	1.2005536159804
0.9888690345801	1.200661472319
0.988995968423	1.200699504537
0.9891246626521	1.2007631025756
0.9892303050062	1.2010213739448
0.9893182741648	1.201332267141
0.9894415958923	1.2018394599844
0.9895427810899	1.2019665422992
0.9896784378102	1.2022343319314
0.9898250406952	1.2026808161514
0.9899613620673	1.2030976799865
0.9901008122454	1.2031452533929
0.9902194117229	1.2033927229245
0.9903246313124	1.203538065468
0.9904480487898	1.2035564940053
0.9905809698488	1.2037093338004
0.9906765775172	1.2038853287278
0.9907890492703	1.2039605027704
0.9909079738672	1.2040664558266
0.9910546528562	1.2044424545059
0.9911765372273	1.204537330901
0.991293253886	1.204629190297
0.9913915772871	1.2048383690401
0.9915115721938	1.2048543088073
0.991621106477	1.204897292725
0.9917367518557	1.2049148949368
0.9918487320425	1.2050677837457
0.9919677503286	1.2053674298744

0.992091284193	1.205668109872
0.9922135414858	1.2060042171527
0.9923385153611	1.2060596617861
0.9924587529813	1.2061676755767
0.9925941797788	1.206570099455
0.9927159296947	1.206614622967
0.9928266838839	1.2066768207735
0.9929546583381	1.2070817676467
0.9930682289539	1.2070987045241
0.9931847732427	1.2072242761555
0.9933037559969	1.207530752039
0.9934117976776	1.2081907126719
0.9935408593714	1.2085179030347
0.9936545932417	1.2086231486271
0.9938177223233	1.2087996846951
0.9939710050597	1.208931467812
0.9941132509242	1.208933931122
0.9942470951106	1.2089905262688
0.9943566563929	1.2090086400747
0.9944674089456	1.2090794411442
0.9945810836205	1.2095737183686
0.9947143417668	1.2098369512359
0.9948190265261	1.2102559524316
0.9949407772162	1.2106516910782
0.9950480561207	1.2111073586583
0.9951606482142	1.2112147180878
0.9952793312679	1.2112632047814
0.99539653369	1.211291311809
0.9955325258688	1.2116929936965
0.9956611734097	1.2117549893216
0.9957928527272	1.2119476242096
0.9959165424879	1.2128884092377
0.9960337897439	1.2128908165994
0.9961383323418	1.2134007385207
0.9962620861892	1.2135259656185
0.9963650810629	1.2135564470932
0.9964841107007	1.2136484439642
0.996605267494	1.2137094884819
0.9967403899853	1.2137940302579
0.9968833540436	1.2141035059702
0.9970340529024	1.2143202733154
0.9971686539252	1.2143788568585
0.9972952319382	1.2145557970394
0.9974014956078	1.2145663783142
0.9975392762106	1.2146376116367
0.9976500014391	1.2147381039111
0.9977737792059	1.2151796211705
0.9978792446569	1.2153489308452
0.9980051863451	1.2159166422728
0.9981245831602	1.2162079318321
0.9982426934116	1.2162248444765
0.9983705199802	1.216253943955
0.9984894390208	1.2164595696982
0.9986429966656	1.2166278784361

0.9987786642044	1.2167832831289
0.9988875054634	1.2173107991284
0.9989867360197	1.2174803416098
0.999091833226	1.2175092000085
0.999194697023	1.2176314684149
0.9993000364719	1.2176566774575
0.999417651307	1.2179150029748
0.9995413787929	1.2181129031735
0.9996487399187	1.2183969888972
0.9998005671604	1.2186614555411
0.9999249932335	1.2186745305655
1.0000536164188	1.2193286856639
1.0001840142508	1.2197234526492
1.0002935298468	1.2197875122093
1.0004083260556	1.2200014274469
1.0005555882083	1.2200223385042
1.0006853008438	1.2202383234342
1.0007969615563	1.2203869574692
1.0009270292946	1.2206298951916
1.0010456808414	1.2208758679918
1.0011643926833	1.2209636857641
1.0012759084853	1.2213052262687
1.0013996211331	1.2215068688009
1.001497714532	1.2217284004681
1.0016288987135	1.2221489630632
1.0017738328316	1.2222045626624
1.0018951779406	1.2224617332552
1.0020014499786	1.2227395882567
1.0021335759005	1.2227636976279
1.0022381806538	1.2228608350282
1.0023569492379	1.2231581806699
1.0024771688165	1.2232588595851
1.0026019154248	1.223354020076
1.0027241356039	1.2235713278692
1.0028542466145	1.2237403825149
1.0029817396075	1.2239247326242
1.0031098711698	1.2243701891692
1.0032153075947	1.2244569118361
1.0033167634651	1.2244773014778
1.0034439763715	1.2246568249381
1.0035717360637	1.2249087464836
1.0037123365897	1.2250439797997
1.0038170946133	1.2250735392325
1.0039162363097	1.2250738391385
1.0040236264157	1.2252330869863
1.0041261884017	1.2255950831662
1.0042559951667	1.2256840236388
1.0043798505841	1.2259062883793
1.0044891294446	1.2259632182621
1.0046202094127	1.2261952878849
1.0047530333644	1.2262048258165
1.0048621192928	1.2263224534618
1.0049779115535	1.2264488908513
1.0050930215821	1.2267484383993

1.0051922189757	1.2269092251733
1.0053125715451	1.2269909418213
1.0054466179206	1.227000804735
1.0055732519475	1.2273431112543
1.0056806618426	1.2274065005051
1.005815939508	1.2275438169101
1.0059287163727	1.2283290207399
1.0060269520902	1.2283318998615
1.0061382593599	1.2287931837427
1.0062562643118	1.229005054237
1.0063664736429	1.2293757945431
1.006493034729	1.2295036233105
1.0065943101771	1.2297147051347
1.006709156749	1.2297306171053
1.0068360803717	1.2300577327159
1.0069786618464	1.2301319750722
1.0071146805898	1.2301912994492
1.0072370475549	1.2302227643732
1.0073540229804	1.2302843940113
1.0074778373625	1.2305118698833
1.0076043906897	1.2305873801879
1.0077071146276	1.2306222776572
1.0078179214761	1.2306535720215
1.0079317037601	1.2308810470638
1.0080477995518	1.2312521678942
1.0081789023527	1.2313755394847
1.0082889193169	1.2314577627463
1.0083871451285	1.2320940283249
1.0084969114626	1.2324916802747
1.0086254410714	1.23253674094
1.008760816333	1.2326371267651
1.0088865692194	1.2326769029691
1.0090129752102	1.2331630291408
1.0091341932067	1.2332179570105
1.0092571504371	1.2332478654015
1.0093938279168	1.2333888543722
1.0095019053209	1.2336236328074
1.0096156775757	1.2342126389298
1.0097546549074	1.2347184141466
1.0098841257672	1.2348754815743
1.0100064668272	1.2350304585043
1.0101265943939	1.2351425421919
1.010235185619	1.2354545837571
1.0103478191798	1.2354605294458
1.0104698667112	1.2356766628893
1.0105624193376	1.2356919156478
1.0106955233839	1.2358862097777
1.0108037107372	1.2359510083621
1.0109000487189	1.2362059858525
1.0110283997795	1.2366791224259
1.011151327694	1.2367046302381
1.0112868842343	1.2367083186916
1.0113965542874	1.2372554353667
1.0115393341055	1.2374938140714

1.0116699463495	1.2378775810789
1.0117819393052	1.2379139353373
1.011914346321	1.2380424725655
1.0120184241208	1.2381769259529
1.0121451790892	1.2383963560991
1.0122602545937	1.2384565790634
1.0123758346094	1.238693385846
1.012468680528	1.2387761864074
1.0125932235501	1.2388668833415
1.0127288348563	1.2388805434198
1.0128511808275	1.2389641747727
1.0130002540177	1.2390278428399
1.0131342521204	1.2393122355427
1.013252025286	1.2395780906771
1.0133758962935	1.2399183226252
1.0135054708038	1.2405170277867
1.0136270963929	1.2405509648101
1.0137371744105	1.2405847579441
1.013867131868	1.2409076605032
1.0140065219485	1.2410399249315
1.0140993403433	1.2412774869656
1.0142162554922	1.2414572461641
1.0143497485105	1.2419132295786
1.0144602507659	1.2422661204348
1.0145825864233	1.2425097665092
1.0147313418324	1.2425594091989
1.0148586873123	1.2425654246592
1.0149965912907	1.2426154319339
1.0151027771601	1.2430354740843
1.0152177767924	1.2431109527508
1.0153432892335	1.2433704911477
1.0154676990086	1.2435689576271
1.0155658057017	1.2441080375985
1.0157314831712	1.2445480019363
1.015866454375	1.2448367175417
1.0159988815262	1.2449903144943
1.0161243295989	1.2452177208587
1.0162505376868	1.2452747512868
1.0163853363942	1.2452961640218
1.0165191870281	1.2456133137343
1.0166317521214	1.2458257393724
1.0167507061292	1.2461368850315
1.0168727624249	1.2461961614016
1.016974783558	1.2463545825453
1.0171203647601	1.2469869277604
1.017261986495	1.2471249952825
1.0173806274413	1.2471535842285
1.0175102962102	1.2471752384425
1.0176307048998	1.2471770143174
1.0177457213222	1.2471835181732
1.0178697641636	1.2477104959144
1.0179785243036	1.248079508899
1.018107143894	1.2483397950547
1.0182408880561	1.2485173002177

1.0183385146452	1.2485937618591
1.0184573781472	1.2487525899259
1.0185747651705	1.2489370113281
1.0186956254263	1.2491095391714
1.0188261633245	1.2491533217829
1.0189473542218	1.2492052736776
1.0190751488981	1.2492625563057
1.0192079193056	1.249284312073
1.0193471291123	1.2493837402317
1.0194643984856	1.2494284630692
1.0195774834764	1.2494434315221
1.0197085372048	1.2498167842396
1.0198335032256	1.2498439779799
1.0199440993848	1.2502096535254
1.0200498482527	1.2502331908165
1.0201787194423	1.250500260788
1.0202932637105	1.2505846758906
1.0204106675735	1.2507598326374
1.0205471176839	1.2509128507169
1.0206733389703	1.2509822741394
1.0208044351161	1.2512549880898
1.0209286040292	1.2517608715433
1.021050980793	1.2519557447437
1.0211689475174	1.2519565237689
1.0212910892359	1.2520287759512
1.0214064194826	1.2524069787124
1.0215415363546	1.2524327790266
1.0216540714696	1.2525566175834
1.0217846656635	1.2526269130173
1.0219045185475	1.2532823782238
1.0220216569527	1.2534029184766
1.02215102475	1.2534114618156
1.0222725644812	1.2541697366156
1.0223901131657	1.2542096744812
1.0225236786642	1.2542769529652
1.0226602881914	1.2543604800219
1.0227921444197	1.2544457898442
1.0229146221207	1.2545322936553
1.0230380923875	1.2549691675838
1.0231514178771	1.2550955602636
1.0232669004012	1.2554009978094
1.0233884135701	1.2554023226096
1.0235347673424	1.2557318986148
1.0236912059053	1.2558961541831
1.0238267042685	1.2561145930106
1.0239558577813	1.256196562334
1.0240986213172	1.2564125232188
1.0242213936291	1.256431148206
1.0243320368278	1.2570632285267
1.0244459690188	1.2573713857474
1.024594235615	1.2577619291989
1.0247227414984	1.2578540171372
1.0248246359929	1.2579893297225
1.0249586060029	1.2588895781447

1.0250800372683	1.2589317639452
1.0252090237516	1.2589884940082
1.0253262209073	1.2591040739895
1.0254566452213	1.2593506476246
1.0255899748218	1.259556791448
1.0257136135958	1.2597224969965
1.0258505165201	1.2598746698213
1.0259711338028	1.2599621180346
1.0260918330999	1.2600371864699
1.0262144666915	1.260275502553
1.0263268272093	1.2604084608564
1.0264499808306	1.2604125943011
1.0265595318112	1.2604849286236
1.026671628487	1.2607174640723
1.0267888435921	1.2608925421789
1.0269098934643	1.2611442151276
1.0270379987142	1.2612265956121
1.0271598891595	1.2612523793418
1.027283537713	1.2615471950498
1.027412321037	1.2616310802208
1.0275355808294	1.2618522111605
1.027664310512	1.2618765308832
1.0278038442051	1.2618854866698
1.0279216933014	1.2619416619845
1.0280391407363	1.2620291377476
1.0281389180103	1.2620753899781
1.0282473048766	1.2622311632117
1.0283642743233	1.2622866336476
1.02850394755	1.2631691006887
1.0286470056212	1.2631769840377
1.0287706021345	1.2633602953867
1.0289007536906	1.2634832279626
1.0290355335515	1.2635029053302
1.02916452117	1.2635065845219
1.0292840550092	1.2635645873614
1.0294146860663	1.2636987380296
1.0295411180749	1.2637627698199
1.0296745674763	1.2637969239493
1.0297982170856	1.2638885060652
1.02992306532	1.2641078391255
1.0300371316733	1.2641254787344
1.0301820006607	1.2644292484655
1.0303118146119	1.2649964186315
1.0304530078137	1.2650095838652
1.0305835407752	1.2654273158354
1.030707370344	1.265822541157
1.0308299879324	1.266000825739
1.0309642147256	1.2661057753371
1.0310775651849	1.2661319152023
1.0312069378161	1.2662134829424
1.0313416330018	1.2663502539644
1.0314454988191	1.2666199992201
1.0315748727109	1.2667088633408
1.0316881854418	1.2670216705086

1.0318297920784	1.2673961153012
1.0319462255073	1.2675588255372
1.0320803119581	1.2678135421025
1.0322056034505	1.2679638907912
1.0323412363472	1.2679791279784
1.0324505722352	1.2680218476756
1.0325608909017	1.2680220403823
1.0326834021263	1.2680292236774
1.0328144008544	1.2681158747945
1.032926939723	1.2682895655502
1.0330569988776	1.2687635276943
1.0331671807232	1.2693143116839
1.033280656305	1.2695185612506
1.0334472699162	1.2697830014063
1.0335642339147	1.2701802997211
1.0336893972726	1.2703662630411
1.0338153920859	1.2704625414922
1.0339424465536	1.270768451864
1.0340755479413	1.270947519878
1.0341884035329	1.2716470130015
1.0342947248406	1.2721642968754
1.0344210933051	1.2725733235701
1.034555587777	1.2726861565146
1.0346768521005	1.2728266047921
1.0348217647711	1.2731203711291
1.0349420996985	1.2732694376305
1.0350754951533	1.273359618974
1.0351992717444	1.2733759048369
1.0353215593546	1.2735218953367
1.0354674769181	1.2744888455476
1.0356170735851	1.2749058334076
1.0357606980962	1.2753371859123
1.0358766661	1.2754380530286
1.0359886124499	1.2754424072469
1.0361156473275	1.275658448508
1.0362410140962	1.2757191177575
1.0363446425314	1.276213964299
1.0365082060108	1.2762203258375
1.0366434603645	1.2762349129702
1.0367604030383	1.2762975214672
1.0368897347153	1.2765517359229
1.037008135411	1.2772200112624
1.0371395500487	1.2773311656934
1.0372737412239	1.2774911274243
1.0373902513538	1.2775992956653
1.0375073838159	1.2779359740003
1.0376230681763	1.2780772784164
1.0377357369825	1.2780860582353
1.037892029599	1.2781389079939
1.0379947946093	1.2781532042397
1.0381170462328	1.2783188588258
1.0382419220595	1.2783557331407
1.0383494650063	1.2789047215936
1.0384834021863	1.279157399846

1.0386070359873	1.2791588840354
1.038746722509	1.27926016447
1.0388399079114	1.2799788986062
1.0389406761695	1.2801931130473
1.0390605178254	1.2802176015086
1.0391830350207	1.2802617293978
1.0393046432542	1.2815369495154
1.0394374058886	1.2816356911228
1.0395647709968	1.282061596491
1.0396935653338	1.2821773541693
1.0398409238385	1.282242122705
1.0399649004008	1.2824356801301
1.0400934280658	1.2831241777394
1.0402310497768	1.2832610217009
1.0403614507562	1.2834573856829
1.0404853700464	1.2836755327768
1.0406273015342	1.2843587490399
1.0407526241504	1.2845662054492
1.0408705874323	1.2851658781604
1.041016427216	1.2851860450986
1.0411430678308	1.2856556070164
1.0412822314068	1.2857686515977
1.0414056951874	1.2858554908948
1.0415307274244	1.2859282359296
1.0416396775116	1.2860324669263
1.0417585072871	1.2861698053955
1.0418858930806	1.2862568849683
1.0420089003391	1.2868216890084
1.0421205762151	1.2871636536034
1.0422698650653	1.287287225846
1.0424147198349	1.2880481008798
1.0425404757559	1.2880511735484
1.0426415347261	1.2887542533949
1.0427595968016	1.289166269833
1.0428803473282	1.2891797158074
1.0429961095498	1.2896718955727
1.0431069687395	1.2897570104342
1.0432205474724	1.2899161574118
1.0433679329826	1.2901440387948
1.0434890162408	1.2907494315316
1.0435954493481	1.290926666262
1.0437325753829	1.2913764296434
1.0438661802021	1.2914048462776
1.0439848863558	1.2916381939318
1.044126004547	1.2919591936087
1.0442673504344	1.2928107055426
1.0443934875013	1.2930232086
1.0445013070406	1.2933131334376
1.0446492286038	1.2934483151285
1.0447752967039	1.2938110135596
1.044887948347	1.2939447871808
1.0449947239661	1.294558347715
1.0451401249938	1.2945648894795
1.0452510348034	1.2945974849283

1.0453745077959	1.2946316250737
1.0454886139788	1.2947706947297
1.0456272788357	1.2947735897389
1.045756005889	1.2947833110801
1.0458880573335	1.294860483135
1.0460148280339	1.2950701828512
1.0461286296428	1.2951775572186
1.0462727185578	1.2958198594659
1.0463962721654	1.2960168886128
1.0465059686383	1.2960439660588
1.0466368466148	1.2960576399672
1.0467638825538	1.2960826941083
1.0468978048133	1.2961881528044
1.0470550437217	1.2963112431137
1.0471966294455	1.2963738412649
1.0473150091144	1.2965166270414
1.0474193478312	1.2965475167673
1.0475569665724	1.2967219280276
1.0477039143607	1.2967550551911
1.0478424178447	1.2978637700876
1.0479893497247	1.2984442925798
1.0481422088182	1.2985097423231
1.0482661440934	1.2985254199483
1.0484235341702	1.2985416297722
1.0485793746402	1.2986534381264
1.0486818707794	1.2987092155735
1.0487854064877	1.2987915595612
1.0489249848002	1.2988011367921
1.0490420701228	1.2988783013733
1.0491539614006	1.2989576563833
1.0492922596293	1.2991296695572
1.0494293591867	1.2993388933291
1.0495496593759	1.2995561516977
1.0496590551201	1.2999803744197
1.0497900774458	1.3002055338861
1.0499287758825	1.300605765955
1.0500403185947	1.3007506995763
1.050163068305	1.3010399410717
1.0503168857343	1.3011294907417
1.0504378449469	1.3011714453319
1.0505710388533	1.3012457781762
1.0506989277872	1.3019804020664
1.0508381889775	1.3022261916399
1.0509849710565	1.302236181333
1.0511147090441	1.302423185032
1.0512286244901	1.3024859761727
1.0513656080718	1.3025411455857
1.0515051906942	1.3031304648119
1.0516111494212	1.3031640898619
1.0517578107659	1.3034259420572
1.0518876557412	1.3034292905433
1.0520002877275	1.3036195007447
1.0521167100094	1.3036716138674
1.0522587283284	1.3036786094344

1.0523801059765	1.3037842559193
1.0525208560076	1.3038095091971
1.0526481603228	1.3038145230735
1.0527814772175	1.3042479026171
1.0529130680139	1.3043763492961
1.0530254684482	1.3047483813259
1.0531411937178	1.3052761661278
1.0532466665223	1.3054848889722
1.0533690639148	1.3057924745531
1.0534870617466	1.3059141711037
1.0535899867696	1.3059248038855
1.0537119997756	1.3060652100356
1.0538560411712	1.3061914680021
1.0539955887468	1.3062064529049
1.0541164156773	1.3066129904218
1.0542405288418	1.3067543136333
1.0543676946634	1.3067545042138
1.0545147995061	1.3067585424562
1.0546724283893	1.306771577352
1.0548013606187	1.3068601556247
1.054924246754	1.3068654267988
1.0550465782366	1.3068795842703
1.0551628745228	1.3069852116672
1.0553279247851	1.3070108639063
1.0554616923893	1.3071425852135
1.0555858599333	1.3074680799857
1.0557634773606	1.3075271009897
1.0558811782651	1.3076088550987
1.0560291930752	1.3077377749868
1.0561409684294	1.3080107202593
1.056273415679	1.3081784110456
1.0563869661718	1.3085473528661
1.0564982516229	1.3086189455123
1.0566101964384	1.3091401062349
1.0567372566513	1.3091403543286
1.0568524004503	1.3091458563423
1.0569931417098	1.3092476839955
1.057109411789	1.3092602468588
1.0572423550747	1.3093418128528
1.0573726144556	1.3094947088975
1.0574865676776	1.3095503381132
1.0576013791785	1.3096948573648
1.0577490172448	1.3101594079399
1.0578647325664	1.3102595705973
1.0580131557587	1.3104065369515
1.0581394569248	1.3104474955726
1.0582745961235	1.3105091016121
1.058389524318	1.3105519744936
1.0585188254255	1.3110765543395
1.0586211273562	1.3111228316959
1.0587377586002	1.3111576556894
1.0588646267715	1.3113620064356
1.0589946682941	1.3115118184232
1.059122473246	1.3115613834129

1.0592566040879	1.3115745770103
1.059393495729	1.3122448852842
1.0595167900496	1.3123189862667
1.0596435318738	1.3124867490354
1.0597781144763	1.3124967129791
1.0598987335897	1.3127118220111
1.0600566988747	1.3129137494811
1.0601553158643	1.3131087381236
1.0603030921488	1.3132935773169
1.0604293371112	1.3134345327884
1.0605471917343	1.3134581790027
1.0606465049891	1.3138822304507
1.0607783807982	1.313916931712
1.0608888470807	1.3141506548128
1.061013750785	1.3148249723048
1.0611423341593	1.3149071103091
1.0612869861278	1.3152674164409
1.0614425302491	1.3153430560513
1.0615688629092	1.3153686078663
1.0617030300526	1.3154926282325
1.0618355724495	1.3155965096159
1.0619429133202	1.315767195455
1.0620736886532	1.3158123476037
1.0622093086023	1.3163333023485
1.0623510107998	1.3164450657482
1.0624903410165	1.3166913488419
1.0626146507424	1.3168000944594
1.0627665250135	1.3173533891468
1.0628840856637	1.3177415448866
1.0629983919322	1.3181183029721
1.063118677194	1.3181502125467
1.0632451419506	1.3182264524889
1.0633881989399	1.3183413298504
1.0635344526728	1.3185166425181
1.0636984412803	1.3190602094068
1.0638260006071	1.319161894659
1.0639764915329	1.3196214409339
1.0641251754512	1.319883085954
1.0642562219588	1.3201680796872
1.0643837815029	1.3203416338944
1.0645093297705	1.3207897133502
1.0646477110118	1.320932398761
1.0647729406987	1.3210394673188
1.0648954190573	1.3210737319442
1.0650283193516	1.3210977397425
1.0651414163056	1.3211845044266
1.0652749091045	1.321297289047
1.0653813966043	1.3214673453075
1.0655175635539	1.3215772571638
1.065636611811	1.3218226688072
1.0657654395095	1.3218795761935
1.0658927933425	1.3223293677923
1.0660316363401	1.3224763671072
1.066200376572	1.3229839772046

1.0663223394311	1.3238242292739
1.0664482522078	1.3239812573696
1.0665657572911	1.3241695769022
1.0666806055401	1.3242362642328
1.0667962720756	1.3243515325593
1.0669307509666	1.3244092674866
1.0670607189502	1.3245010655122
1.0671868854073	1.3245916961666
1.0673070385155	1.324622991148
1.0674330353032	1.3247754296144
1.0675556735635	1.3250001976632
1.0676848943605	1.3252028708135
1.0678321245885	1.3254851062935
1.0679731472833	1.3255823238801
1.0681048584719	1.325662684022
1.0682328529551	1.3258660549312
1.0683850021483	1.3259606344281
1.0685228304822	1.3259734337716
1.0686541137796	1.3262534789613
1.0688060479851	1.3266581134845
1.0689277763316	1.3270440342915
1.0690556526461	1.3274029711439
1.0691712263636	1.3275131378962
1.0692965991669	1.3275757164217
1.0694479951551	1.3276204849185
1.0695726098322	1.3277714709555
1.069683273103	1.3278080589052
1.0698088023193	1.3278759656975
1.0699256235887	1.327984119041
1.0700225291889	1.3282230059005
1.0701889290899	1.3282903847735
1.070332934899	1.3287846220086
1.0704672225137	1.329098009062
1.0705761512308	1.3292415677156
1.0706968561163	1.3292641764097
1.0708016776589	1.3293412839901
1.070918306573	1.3293781676587
1.0710523685062	1.3293899012346
1.0711945004315	1.3296192965361
1.0713292083632	1.3306348664047
1.0714667130785	1.3308288109616
1.0715743439778	1.3309463696186
1.0717181809806	1.3310406438658
1.0718418886501	1.3310569357778
1.0719652874675	1.3312681934378
1.0720959809258	1.3314425075345
1.0722415078134	1.3316204155778
1.0723503688071	1.3317229903132
1.0724796565793	1.3318926357581
1.0726099701837	1.3322586231775
1.0727200108994	1.332532147888
1.0728286073993	1.3327163305239
1.0729354762452	1.3327392926405
1.0730772858366	1.3329692227614

1.0732271409694	1.3329776066862
1.0733514526873	1.3334300908554
1.0734831594525	1.3335071326377
1.0736359113489	1.3335314057311
1.0737760999484	1.3335338261316
1.0739086601976	1.3335597369286
1.0740396042849	1.3336018697923
1.0741658465831	1.3336674099837
1.0742921519279	1.3338627676625
1.0744115561265	1.3339403592834
1.0745449739936	1.3342763127555
1.0746750595217	1.3343299802937
1.0748067416406	1.334346806897
1.0749250659476	1.3345718501894
1.0750543642857	1.3349636328024
1.0751884961298	1.3350955938529
1.0753116718741	1.3351428230048
1.0754393010125	1.3356933364655
1.0755442968499	1.336005003672
1.0756935178329	1.3361704429476
1.0758160613775	1.3361769948052
1.0759416498505	1.3366119632885
1.0760356948405	1.3367429439842
1.0761294696029	1.3368776568978
1.0762670384905	1.3372046438779
1.076419422622	1.3373082543821
1.0765503780541	1.3375137107621
1.0766991685839	1.3376428571099
1.0768462455909	1.3381248957668
1.0770024898615	1.3383900636776
1.0771493408262	1.3383955532279
1.0772760941685	1.3387314841886
1.0773980947523	1.3388821271159
1.0775555551228	1.3389829905255
1.077691308737	1.3389971391542
1.0778177134547	1.3390036638527
1.0779639980386	1.3390626376341
1.0780997288674	1.3392268196551
1.0782433785016	1.3393739993323
1.0783698373086	1.3394699985602
1.0785142794955	1.3396535930718
1.0786412790462	1.3398650164881
1.0787949078941	1.3399248989453
1.0789295241973	1.3402237168117
1.0790603116209	1.34048958846
1.0791924783364	1.3405051466764
1.0793152312963	1.3406059164035
1.0794525352351	1.3407532391122
1.0796045952358	1.3408095407759
1.079739850051	1.3410264087085
1.079901162835	1.3410281104376
1.0800190805744	1.3410553347617
1.0801877404704	1.3414910482741
1.0803065210918	1.3415139276863

1.0804247721303	1.3417128583038
1.0805636372443	1.3421479577929
1.0807314635654	1.3421823864064
1.0808747307411	1.3423623172887
1.0810072865292	1.3433807614227
1.081155626038	1.3441134335205
1.0812928925261	1.3441603130798
1.0814289172038	1.3445151236615
1.0815454571017	1.3449998281075
1.0816915600143	1.3450663679753
1.0818222164547	1.3452506218607
1.081937343422	1.3453985213504
1.0820357089549	1.3455330107317
1.082165886233	1.3458417916145
1.0823182005901	1.3459287716803
1.0824414334505	1.3460009723202
1.0825491816323	1.3464677257934
1.0826759540266	1.3467568980548
1.0828161429871	1.3468141014999
1.0829366782245	1.3473125300529
1.0830787930436	1.3473665782146
1.0832142057745	1.347376110053
1.0833363517875	1.3478846616879
1.0835011139984	1.3479924744534
1.0836540517892	1.3481605306745
1.083827558819	1.3483444501869
1.0839303646642	1.3484885311317
1.0840529945595	1.3485088750605
1.0841808501502	1.3485246392134
1.0843004669409	1.3486754784591
1.0844339943072	1.348722925666
1.0845689977081	1.348785237987
1.0846832050043	1.3488235042713
1.0848239299265	1.349245595473
1.0849788804045	1.3494913938951
1.0851260389363	1.3495568838615
1.0852597550803	1.3496408085089
1.0853971264262	1.3497027710975
1.0855278061466	1.3500294788943
1.0856498415432	1.3500990445397
1.0857730599036	1.3501456260669
1.0859184370123	1.3502253573384
1.0860415815548	1.3505642559151
1.0861804288703	1.3507717738375
1.0862970915021	1.3509789879603
1.0864383474206	1.3510849167714
1.0865630288401	1.351299910512
1.0867005606088	1.351596063491
1.0868513682176	1.3517993414418
1.0869783604458	1.3520400886156
1.0871173720381	1.3520622748108
1.0872462235103	1.3523672820538
1.0873720122724	1.3527363055708
1.087490104531	1.3528097787713

1.0876162045294	1.352970475008
1.0877404966166	1.3530786942268
1.0878682481286	1.3540514071874
1.0879881631256	1.3542032033558
1.0881251126032	1.3543635839834
1.0882440561004	1.3545434926157
1.0883991387357	1.3547784689734
1.0885102239796	1.3547883095324
1.0886645558673	1.35483221591
1.0888035196397	1.3549200685684
1.0889659978007	1.3549653137991
1.0891061666807	1.3549968598855
1.0892224150702	1.3550534294426
1.0893587804344	1.3553561035207
1.0895056401988	1.3556075498512
1.0896339573173	1.3556286197273
1.0897639755219	1.3556645756672
1.0899092454862	1.3558476454881
1.0900596681723	1.3558853364226
1.0901869169233	1.3569255586538
1.0903335506271	1.3570218724051
1.0904782618524	1.3572374462675
1.0906230827802	1.3572866751644
1.0907687973019	1.3573765297593
1.0908914496035	1.3576422868199
1.0910132970259	1.3577308135162
1.0911551346762	1.3581468852296
1.0913137113243	1.3583887064161
1.0914511099913	1.3587854581469
1.0916126472389	1.3587944747375
1.0917325934567	1.3589269272236
1.0918629189238	1.3591360214043
1.091995522774	1.359296247882
1.0921384604413	1.3593941096225
1.092268192027	1.3594357251855
1.0923861951754	1.3594952984271
1.0925177026381	1.3601126736177
1.092640527971	1.3603975400577
1.0927719513855	1.3605672456777
1.0929302243309	1.360650579687
1.0930709085977	1.3606767019832
1.0932193971633	1.360875064379
1.0933836270968	1.3612597498732
1.0935036660847	1.3612682288051
1.093638642512	1.3615658589696
1.0937885072855	1.3618096760437
1.0939383439989	1.3618388883922
1.0940752142766	1.3619346679248
1.0941875617436	1.3620654971012
1.0943058318716	1.3623292202734
1.09442547019	1.3625704971724
1.0945574417387	1.3626101348945
1.09471083445	1.3628390379491
1.0948320974623	1.3631546314448

1.0949783414262	1.3632187245958
1.0951184641074	1.3632356236447
1.0952742356358	1.3633645413332
1.0953990128332	1.3639442203623
1.0955017226335	1.3639981462307
1.0956586323756	1.3642542296483
1.0957997701395	1.3643264014097
1.0959229529937	1.3643866967177
1.0960744512233	1.3646047147409
1.0961979563345	1.3646692208672
1.0963218380188	1.3647101682312
1.0964646825999	1.3647186185215
1.0966239441172	1.3651577706731
1.0967627366926	1.365485292539
1.0969039529286	1.3655534155648
1.0970482281493	1.365572823993
1.0971485522424	1.3656749845964
1.0972654953593	1.3658023637133
1.0973975343004	1.365874303153
1.097525081461	1.3659615954584
1.0976344563606	1.3663223035896
1.0977646959634	1.3665565808616
1.0978806765014	1.3671299042188
1.0980055644102	1.3674652327955
1.0981553626029	1.3677348424508
1.0982680330113	1.3677624648848
1.0983967559741	1.367773992589
1.0985492270822	1.36821238248
1.0986741708959	1.3683583847461
1.0988219964824	1.3683816577966
1.0989695387269	1.3687450270584
1.0991070753009	1.3690116867727
1.0992287653759	1.3690637535877
1.0993902121298	1.3694427378609
1.0995170367975	1.3697225687781
1.0996380097743	1.3698033291023
1.0997887220278	1.3699274250479
1.0999118930537	1.3700556894974
1.1000564500902	1.3701704050484
1.1001973310213	1.3701914150756
1.1003013422948	1.370297521814
1.100411369016	1.3702977042667
1.1005506988612	1.37130875622
1.1006950923435	1.3717724580834
1.100846898241	1.3718750075558
1.1009897212463	1.3721665132619
1.1011311052943	1.3722264448175
1.1012712403039	1.3723455516075
1.1014189341634	1.3723931552995
1.1015502458793	1.372698654756
1.1016774247404	1.3727047404347
1.1018195541469	1.3730547588366
1.1019535034291	1.3731078218169
1.1020913357247	1.3737421825558

1.1022080690976	1.3739020322045
1.1023461913013	1.3741060266879
1.1024697439143	1.3742926423066
1.1026453119007	1.3743492982976
1.1027781456256	1.3747758604554
1.1029075010226	1.3749079998268
1.1030597840508	1.3749511487906
1.1032048688577	1.3750580054728
1.1033407122038	1.375288893926
1.1034722819489	1.3756634098826
1.1036038653579	1.3756983575101
1.1037204651121	1.3757725976926
1.1038600466428	1.3759249693066
1.1040014167839	1.3761496643896
1.1041261961937	1.3761529289073
1.1042763330852	1.3764043661195
1.1044086928207	1.3768298830978
1.1045385735467	1.376945118692
1.1046709469913	1.3771504854732
1.1048205950765	1.3782061133239
1.1049654923296	1.3783527444022
1.1050916913666	1.3785394246731
1.1052374489334	1.3786373080771
1.1053704658873	1.3789088863655
1.1055062668588	1.3789158423779
1.1056404483643	1.3789168543649
1.1057718577374	1.3790689855055
1.1059142961168	1.3792567406496
1.1060667743829	1.3792570496036
1.1062205482281	1.379548732056
1.1063487646465	1.3795592919144
1.1064739948433	1.3797238892328
1.1066348501684	1.3798178492234
1.1067780414658	1.380852785981
1.1068928172815	1.3809058800358
1.1070458212362	1.3811071213774
1.1071843222505	1.3812665541684
1.1072978399098	1.3813318034604
1.107423717801	1.3813403598279
1.1075405464805	1.3813487560914
1.1076474901844	1.3813963903882
1.1077802899294	1.3819156235083
1.1079111514408	1.3819879820933
1.108047232972	1.3822931936903
1.1081666229263	1.3823152264272
1.1083086276609	1.3823515695069
1.108450083305	1.3824631881658
1.1085707613374	1.3828819491986
1.1086821381337	1.3833160367053
1.1088002257574	1.3833202765471
1.1089348788194	1.3837144872986
1.1090652628555	1.3837892844771
1.1091734810461	1.3841795989527
1.1092844106757	1.3845644870756

1.1094239152469	1.3845834605786
1.1095678152993	1.3847082100603
1.1096976465068	1.3853344496793
1.1098306047939	1.3854998725331
1.1099740257969	1.3855926926157
1.1101071919135	1.3856324681682
1.1102482025678	1.3856659235817
1.1103912777214	1.3862876590642
1.1105515677523	1.3863039917147
1.1106731086616	1.3863565408173
1.1108257633612	1.3863782014116
1.1109502546489	1.3870832407485
1.1110868829626	1.3872499400216
1.1111951460652	1.3874072714246
1.111336185379	1.387718131308
1.1114476856964	1.3879370812239
1.1115925013264	1.3883503879248
1.1117052538066	1.3886260599521
1.1118549542284	1.3888406374162
1.1119694856411	1.3888450122231
1.1120967896364	1.3894379357827
1.1122102163355	1.38944369078
1.1123734516503	1.3895860819718
1.1125478443319	1.3897137213528
1.1126897472391	1.3898561046577
1.112830049242	1.3900264800408
1.1129674612614	1.3901682150573
1.1131315665972	1.3906030591669
1.1132815389965	1.3906888829141
1.1134478071895	1.3908337275815
1.113578276384	1.3909054930354
1.1137175115212	1.391136447426
1.113837553739	1.3911682490338
1.1139751683659	1.3914892610449
1.1141218274391	1.3915220319352
1.1142735587623	1.3919572291017
1.1144082610688	1.3919954288018
1.1145538691854	1.392347900996
1.1146903612764	1.3928428779063
1.1148255188839	1.3937890264491
1.1149510212011	1.393930862399
1.1150783669518	1.394033854004
1.1152031810659	1.3941450759073
1.1153445568856	1.3942489880516
1.1154808585813	1.3943483769872
1.1156119261173	1.3945446143472
1.1157256947965	1.3945896560899
1.1158544957271	1.3947673351386
1.1159988702013	1.3949687090736
1.1161542527203	1.3954172930806
1.1162874495426	1.3958848159356
1.1164324699202	1.3959702106844
1.1165786210896	1.3959855382352
1.1167023855357	1.3962267534526

1.1168144593152	1.3964573387958
1.1169444542159	1.3965573446873
1.1171094664595	1.3965994250894
1.1172476394609	1.3966181046214
1.117367056943	1.3967605170848
1.1174982973981	1.3970581455464
1.1176560426299	1.3973365654896
1.1177801912852	1.3975490569225
1.1179315168697	1.3978362459573
1.118062411955	1.3980290411744
1.1181885740788	1.3984326621367
1.1183366779611	1.3990389904528
1.1184660489383	1.3991201683782
1.1185921472596	1.3993281949641
1.1187429604271	1.3995588808347
1.1188823668726	1.4002387807283
1.1190276065782	1.4005284967023
1.1191606643422	1.4006964259885
1.1193045019624	1.4010003469299
1.1194268531571	1.4017702574798
1.1195528249541	1.4018090021388
1.1196926475907	1.4022647146115
1.1198074225925	1.4026427564195
1.1199493609786	1.4027333426444
1.1200844313893	1.4031129694472
1.1202373515394	1.4031841667243
1.1203757409914	1.4032064821093
1.1205289147323	1.4032467378617
1.1206544761836	1.4032909897231
1.1208103763024	1.4032928113479
1.1209369541538	1.4036064978405
1.1210926066181	1.4038231984963
1.1212255404063	1.4038614479162
1.1213775725293	1.4038661377962
1.1215153899107	1.4042386050112
1.121642372039	1.4043578483744
1.1217764024648	1.4043921370639
1.1218920404938	1.4044779072244
1.1220019462465	1.4045062931924
1.1221521157327	1.4049177575329
1.122286955391	1.4049598771338
1.1223890983693	1.4050251377674
1.1224988213104	1.4052432404823
1.1226334079007	1.4053561793712
1.1227867865294	1.4055722373587
1.122954876664	1.4059737786465
1.1231028799487	1.4059987340705
1.1232726001727	1.4060327471031
1.1234086210737	1.406630132583
1.1235424235271	1.4070974452215
1.1236941783177	1.4071179460407
1.1238398073846	1.4072657505693
1.1239930250952	1.4073272280277
1.124149842425	1.4078017718289

1.1242650734866	1.4078813316739
1.1243818887198	1.4081483008973
1.1245332251761	1.4083613594352
1.1246789729627	1.4084052641155
1.1248242410558	1.4085671077634
1.1249606868476	1.4087126066422
1.1251046079561	1.4087384094806
1.125242574144	1.4088951483735
1.1253854465818	1.4091321702586
1.1255452036469	1.4092115536606
1.1256743500532	1.409257029212
1.1258003437521	1.4097825021222
1.1259360929998	1.4103091564768
1.1260858772368	1.4107257750482
1.126213962386	1.4107808072025
1.1263681556187	1.4107958372738
1.1265097402058	1.4108984495472
1.1266443557973	1.4109627774619
1.1267876498272	1.4111525501972
1.1269213872151	1.4114503546098
1.127046487065	1.4115387842959
1.1271979776493	1.411646264044
1.1273462972614	1.4116819605104
1.1274884318962	1.4117699155169
1.1276582916105	1.4119083810646
1.1278003938256	1.4123731875019
1.1279180522272	1.4124167519119
1.1280617283796	1.4128985463686
1.1282304980462	1.4133996020489
1.1283641234943	1.4138135113473
1.1284917862467	1.4138637307093
1.1286432144734	1.4140949117101
1.128775064797	1.4141980289344
1.1289163648238	1.4144765540181
1.1290454258914	1.4146959156559
1.1291718406218	1.415421884127
1.129314582713	1.4156112806131
1.1294522671403	1.4157875866366
1.1295886548114	1.4160488391545
1.1297287061764	1.416060137796
1.129866124434	1.4161472281892
1.1300059226071	1.4165841471
1.1301430027666	1.4175082830609
1.1302967217488	1.4176057152385
1.1304246196692	1.4178771039588
1.130543405515	1.4179994714939
1.1306894122121	1.4184391439509
1.1308040864217	1.4190330545442
1.1309637086004	1.4194078728762
1.1310907185056	1.4194645534233
1.1312333897424	1.419677651765
1.1313788488766	1.4197186664898
1.1315028742193	1.4198803401874
1.1316492254973	1.4200041258293

1.1317818096107	1.4201350255355
1.1319367174013	1.420218849648
1.1320844354301	1.4202850090473
1.1322397446871	1.4203185789435
1.1323566702847	1.4204529009419
1.1324782735874	1.4207614739003
1.1325896455233	1.4220956808713
1.1327579811667	1.422180142158
1.1328965298363	1.4227913121602
1.1330562695909	1.4230098650389
1.1332216810312	1.4230200316555
1.1333478674091	1.4230203509128
1.1334789941909	1.4230931964583
1.1336188597702	1.4239280446408
1.1337614230203	1.4240675634031
1.1338696014922	1.4241716893735
1.1340303923448	1.4242383236279
1.1341562891868	1.4245352518767
1.1343030286229	1.4245810419028
1.1344461917994	1.4248874484226
1.1345626095497	1.4249795762476
1.1347005467478	1.4252529685467
1.1348379602833	1.425358669881
1.1349494310814	1.425592059085
1.1351073294926	1.4255945667839
1.1352332780871	1.4256005946815
1.1353609273012	1.4257755723418
1.1355043021759	1.4259563360188
1.1356360813206	1.4267218445658
1.135756118571	1.4267368149061
1.1358893079958	1.4267477813582
1.1360390374227	1.4267547830466
1.13618289695	1.4272403178336
1.1363065268399	1.4272510929108
1.1364792063806	1.427339110338
1.1366278440518	1.4273947617754
1.1368083725348	1.4274248124616
1.1369383211489	1.4275017960985
1.1370990170247	1.4276218938617
1.1372381555727	1.4276372768977
1.1373976696054	1.4285589168095
1.1375456972621	1.4285996568092
1.1376845051961	1.4291099620525
1.1378383741181	1.4293449599474
1.1379874720712	1.429364597216
1.1381447957507	1.4295504126003
1.1382977994318	1.429626740822
1.1384405224148	1.4302702128207
1.1385875979308	1.4303384901445
1.1387258392331	1.430371441745
1.1388723924355	1.4307096773394
1.139009188182	1.4308360977594
1.139141563934	1.4309737604546
1.139280793171	1.4309914546513

1.1394201664599	1.4310517387926
1.1395375495385	1.431183314812
1.1396453934587	1.4312817740301
1.1397977908702	1.4315448815421
1.1399663769447	1.4322498794341
1.1400947765365	1.4328132776579
1.1402302635796	1.4328722443714
1.1403699309182	1.4330241169959
1.1405442208968	1.4331702280931
1.1406789785571	1.4335091282619
1.1408012486784	1.4339152098674
1.1409713745628	1.4340137813791
1.1411182095117	1.4340170385102
1.1412571580483	1.4340290855663
1.1413871194459	1.4344403865203
1.1415367173974	1.434486312914
1.141697632742	1.434543202109
1.1418294698203	1.4348202625428
1.1419619621829	1.4348390521068
1.1420845201052	1.4352268623331
1.1422326941581	1.4354437592999
1.1423868489009	1.4356199830393
1.1425543664987	1.4356304092946
1.1426819077859	1.4357670857883
1.1428292435195	1.4359386245446
1.1429428994124	1.4362572377875
1.1430751123006	1.4373715563084
1.1432104608921	1.4373882575422
1.1433935253192	1.4376382537529
1.1435286751288	1.4377164475362
1.1436844681171	1.4377249462819
1.143839919378	1.4379738160207
1.14396612961	1.4380577355235
1.1441067633532	1.4381129446338
1.14428797278	1.4381591968434
1.1444417049723	1.4381738663988
1.1445886190192	1.4381991259833
1.1447126534831	1.4382355193113
1.1448378066057	1.4383730676995
1.144962844536	1.4388709560498
1.1451242791189	1.4391194198001
1.1452681730884	1.439877161807
1.1454023219937	1.4399290829295
1.1455292349121	1.440196930331
1.1456809362934	1.440433815371
1.1458247987202	1.4406175162501
1.1459910057671	1.4407013578256
1.1461298172304	1.4408617967654
1.146270031669	1.4410295886136
1.1464142305988	1.4414807872066
1.1465710378494	1.4422832985893
1.1467181881339	1.4424850541431
1.1468530772215	1.4427350703953
1.1469920121681	1.4430031136299

1.1471496306017	1.4432881670164
1.1472938051597	1.4436778464948
1.1474199328561	1.4442823394939
1.147565193823	1.4445380898957
1.1477080469892	1.4449852674326
1.1478337254759	1.4451726102589
1.1479759471964	1.4453577186661
1.1481291020781	1.4461452949786
1.1482793448647	1.4463020311032
1.148416273386	1.4464069099951
1.1485532273318	1.4464386884828
1.1486952129512	1.4466171905244
1.1488295685672	1.4469064718376
1.1489655984071	1.4471770913008
1.1491138236908	1.4476085631715
1.1492520174255	1.4478660118981
1.1493968566601	1.4481810449777
1.1495487809035	1.4483555928875
1.1496811609526	1.4484695510656
1.1498195811412	1.4485243025629
1.1499730618752	1.4492101918748
1.1501373377767	1.4494094054767
1.1502611845816	1.4495753897043
1.1504009928173	1.4495871200376
1.1505293915357	1.4502111774783
1.1506760014705	1.4506291059254
1.150830117306	1.4509597255299
1.1509586748668	1.4514288695499
1.1510959168549	1.451439691429
1.1512290671445	1.451605958058
1.1513561342398	1.4524999593446
1.1514881995639	1.4526079693924
1.1516291395981	1.453011292357
1.151748756865	1.4530324598843
1.1518880743721	1.4537855879117
1.1520063518044	1.4539932600892
1.1521311944825	1.454456137351
1.152291309833	1.4547582307025
1.1524528933175	1.4551018831518
1.1526254575353	1.4554552364438
1.1527844923781	1.4554876772019
1.1529095698064	1.4563903773058
1.1530717418315	1.4568452758594
1.1532039196781	1.4568650660731
1.1533368465934	1.4570580361869
1.1534915774437	1.4573623333979
1.1536245686747	1.4574277242893
1.1537579956583	1.4575297667123
1.1539282892856	1.4575691471852
1.1540931045522	1.457601028458
1.1542260568263	1.4578043798897
1.1543536831498	1.4579250170253
1.1545132237662	1.4585336513708
1.1546509987426	1.4587105366299

1.1547848229837	1.4588720454204
1.1549077847207	1.4590352784205
1.1550706507076	1.459039074481
1.155204026557	1.4592277713373
1.1553468948289	1.4593734830319
1.1554774619001	1.4594860063831
1.1556096963907	1.4596575027861
1.1557481547593	1.460013967593
1.155884006511	1.4600207546768
1.1560610828299	1.460332918238
1.1562085865682	1.4607056044701
1.1563218534484	1.4607245927532
1.1564707216045	1.460760065933
1.1565995867283	1.4607755692046
1.1567415827445	1.4609513040516
1.1568921883522	1.4612944988388
1.1570550049639	1.4613336700259
1.157195784662	1.46136648058
1.1573490035935	1.4613716744977
1.1574833950487	1.462045793624
1.1576150244548	1.4620753071899
1.1577792372131	1.4623346118271
1.157916431396	1.4625195863741
1.158071106273	1.462652786295
1.1582238976612	1.4630931578568
1.1583590177228	1.4631465430229
1.1584960125022	1.4634090388337
1.1586551290864	1.4634259818797
1.1588185317625	1.4635136580946
1.1589416354136	1.4637253882798
1.1590666926617	1.4638919714317
1.1591688235462	1.4640098095139
1.1593210727209	1.4644835841669
1.159437180732	1.4644939124302
1.1595708661332	1.4646142689256
1.1597001410456	1.4648235387063
1.1598784468269	1.4655059050075
1.1600301341638	1.4657245168073
1.1601796764304	1.4658907894171
1.1603228277049	1.4664086936828
1.1604639491676	1.4664886578535
1.1606046087757	1.466925872226
1.1607327010797	1.467517775385
1.1608811053689	1.4681629229317
1.161036801139	1.4683380245525
1.1611676803469	1.4694012781761
1.1613210370197	1.4694269484474
1.1614547929207	1.4694334378236
1.1615948373859	1.4694558497045
1.1617322630345	1.4694693682667
1.1618679425761	1.4695577644113
1.1620178724807	1.469559149311
1.1621532313766	1.4697997038896
1.1622965944515	1.4698386923573

1.1624569604732	1.4698793973243
1.1626239167194	1.4700077933095
1.1627748145574	1.4701492715189
1.1629232339928	1.4702043473216
1.1630679660327	1.4702256407848
1.1631924449428	1.4702502348981
1.1633352205513	1.4705362147836
1.1634699401508	1.4706944096968
1.1636039635854	1.4707164398362
1.1637663939987	1.4707617843958
1.1639195747	1.471101516053
1.1640565024902	1.4711792296063
1.1642159807765	1.4712608857004
1.1643772016556	1.4715059906245
1.1645333326534	1.4716229782236
1.1646703163712	1.472366441078
1.1648132434556	1.4725627987882
1.1649905695486	1.4727625789388
1.1651260752953	1.4727699062226
1.1652643537878	1.4728921685504
1.1653798541803	1.4730711477026
1.1655385441373	1.4731954236674
1.165688521105	1.4733721357544
1.1658372216178	1.4737479784663
1.1659866595455	1.4744059392544
1.1661135396969	1.4746358295841
1.1662421319367	1.4751364877528
1.1663835093813	1.4752062035425
1.1665277937098	1.4752159546896
1.1666659352657	1.4756754917924
1.1668123656924	1.4757753422423
1.1669575776091	1.475841483694
1.1671160194953	1.4760441602686
1.1672785068553	1.4762371311634
1.16742998434	1.4762888903995
1.1675697504347	1.477474876983
1.167692733032	1.478179338183
1.1678433524475	1.4784078344258
1.1679675594867	1.4784550368296
1.1681580547755	1.4785225765821
1.1682636905712	1.4787117104563
1.168403287848	1.4787125899414
1.1685580004279	1.478790170136
1.1686797003596	1.4788636385463
1.1688125000118	1.4791425982599
1.1689482425769	1.4795020564971
1.1690995312689	1.4796169835578
1.1692537542725	1.4797448160794
1.1694060721018	1.4798576724604
1.1695715760313	1.4801076563837
1.1697416180164	1.4801300298703
1.1698926569541	1.4802249675015
1.1700751770877	1.4805267651862
1.1702279469694	1.4808523593856

1.1704016670717	1.4810745335133
1.1705511717421	1.4814137938326
1.1706856381567	1.4820538236127
1.1708199480559	1.4835539325887
1.170958934861	1.4843557810078
1.1711219053416	1.4844246913908
1.1712634395779	1.4845517703083
1.1714095788244	1.4845730471384
1.1715463165713	1.48477564896
1.1717030939838	1.4847935808391
1.1718393797491	1.4848243605281
1.1719661662071	1.4848599053659
1.1721115002353	1.4850193359424
1.172239473507	1.4851386186342
1.1723920198185	1.4851578678647
1.1725230811464	1.4853080171679
1.1726470707296	1.485349654865
1.1727881509344	1.4854443697431
1.1729288749297	1.4855381232766
1.1730812748238	1.4856462424259
1.1731986653356	1.4857546433516
1.1733654618269	1.4865178845501
1.1734923489557	1.4866497653532
1.1736458974309	1.4868205786204
1.1737889538753	1.4871573577101
1.1739208741812	1.4872558828004
1.1740684874463	1.4874007194785
1.1742068193735	1.4876150556545
1.1743372046814	1.4885660603335
1.174510406392	1.4887235970372
1.1746721238172	1.4893755755696
1.1748344557761	1.4907692139353
1.1749894487655	1.4909983254307
1.1751693150145	1.4913319281256
1.1752979218215	1.4913717264794
1.1754585900459	1.4914163891936
1.1756290724816	1.4914656778589
1.1757778300083	1.4916739728871
1.1759433814021	1.4916970754839
1.1760788358732	1.4920333229307
1.1762336366802	1.492108874805
1.1763877576878	1.492122177063
1.1765560084258	1.4921419930984
1.1767054027737	1.4922712413228
1.1768386708259	1.4924005977832
1.1769747227011	1.4927950405497
1.1771007313092	1.492874069382
1.1772427857415	1.4932401167521
1.1774333023857	1.4933497629552
1.1775976769619	1.493439033183
1.1777907332196	1.4935828736143
1.1779632891564	1.4938018133374
1.1781178140958	1.494049532897
1.1782809207376	1.4948518923158

1.178432635154	1.4950534860904
1.1786030805538	1.4951104330978
1.1787517716476	1.4957832647594
1.1789204374152	1.4958080403833
1.1790659636947	1.4959209787407
1.1792104342129	1.495926052676
1.1793452740649	1.4960229993191
1.1794783026928	1.4965256358851
1.1796275399034	1.4969346369432
1.1797957089616	1.4969928585538
1.1799171290548	1.4973055747214
1.1800693294205	1.4977989652046
1.1802168630018	1.4979458302793
1.1803689038836	1.4984930217951
1.1805429007377	1.4990936229204
1.1807061004676	1.4992917006535
1.180853369351	1.4994038169825
1.181020723116	1.4995407270455
1.1811694435348	1.4995894766711
1.1813316584556	1.5009115885316
1.1815052261203	1.5009188163713
1.1816498914984	1.500938509152
1.1817877463743	1.5011742313707
1.1819262294865	1.5012780912452
1.1820743339585	1.5013028414358
1.1822238016972	1.5015842830472
1.1823337055533	1.5017725257651
1.1824775826237	1.5022171490977
1.1826173587953	1.5024252734961
1.1827523919348	1.5025122273311
1.1828994573838	1.5031240630689
1.1830293880824	1.5040215232798
1.1831871974884	1.5040579576644
1.1833550839789	1.5052675473394
1.1835000886054	1.5056770593088
1.1836328107243	1.5057092322656
1.1837773847042	1.5058955663825
1.1839269221461	1.5067462831634
1.1840746540861	1.5068395926582
1.1842455699778	1.5068653205803
1.1843927247969	1.5072267838121
1.1845466675173	1.5076345918498
1.1846953281571	1.5078878213584
1.1848455571152	1.5085951332287
1.1850017448217	1.5093029748119
1.1851511005921	1.5096308738105
1.185300036831	1.5098837070351
1.185452709877	1.5098935525558
1.1856390073751	1.5099491015879
1.1858114484703	1.5100505136348
1.1859422031726	1.5100679803127
1.1860876549721	1.510167862241
1.1862445525114	1.5102567302289
1.1863856575254	1.5102730390099

1.1865460160959	1.510738000392
1.1867030046527	1.5111665579576
1.1868466959551	1.5113000792758
1.1870212318209	1.5113723519496
1.1871677013923	1.51158795749
1.1873241822345	1.5117142085383
1.1874675496312	1.5120327398353
1.1876437828584	1.5122316411388
1.1877865593963	1.5123144037145
1.1879524777234	1.5127431635641
1.1880758441958	1.5132235886467
1.1882305854948	1.5132619267436
1.1884052601398	1.5142651532853
1.1885788994512	1.5142988206714
1.1887303950391	1.5154151547772
1.1888948028834	1.515437151082
1.1890479397481	1.515461538647
1.189176449386	1.515548389662
1.1893241849684	1.5158497560574
1.1894803063017	1.5161027586926
1.1896234860804	1.5162965665301
1.1898026393493	1.5166440868149
1.18998245206	1.516795380225
1.1901509430934	1.5169934585188
1.1903054971557	1.5170935939005
1.1904547640455	1.5172498781612
1.1906064829329	1.5173133337551
1.1907349484294	1.5175870020208
1.1908845264535	1.5176689497323
1.1910232253077	1.5180810856696
1.1911813403872	1.5180937794109
1.1913490787075	1.5182989236025
1.1915063634034	1.5185896623965
1.1916458279189	1.5187260416557
1.1917957051513	1.5190747060415
1.1919310907924	1.5191933057966
1.192101812915	1.5193526689605
1.1922642492155	1.5194788724012
1.1924073413254	1.5194866106033
1.192549576197	1.5199181192828
1.1927338765886	1.5200510397411
1.1928815089323	1.5200651168177
1.1930399442381	1.5202691922444
1.1931915450435	1.5208007098008
1.1933904335317	1.5208041752263
1.1935191907445	1.5209998673079
1.1936473069684	1.5215450733296
1.1937670116366	1.521747441817
1.1939119692313	1.5217718534776
1.1940321539675	1.522241726035
1.1941785028527	1.5223706271604
1.194337643049	1.5224135834567
1.1944881098849	1.5226268564131
1.1946426809187	1.5226785265724

1.1948243561558	1.522731864179
1.1949840829606	1.5231419055966
1.195143604903	1.5235506612759
1.1953021608028	1.5246911428156
1.1954552831286	1.5248770623532
1.195609391308	1.5249425755861
1.1957829716124	1.5250254198241
1.1959567594301	1.5257001915889
1.1961385521076	1.526418915936
1.1962853850146	1.5265233972316
1.1964421848098	1.5266758290495
1.1965795235666	1.5267742407201
1.196739316793	1.5274590200223
1.1968732040338	1.5275322238803
1.1970318103201	1.5278164687555
1.1971978112421	1.5278517699456
1.1973812231129	1.5279585298463
1.1975588520505	1.5279717018125
1.1977060205484	1.5280646641467
1.1978556304947	1.5281846691397
1.198039131492	1.5287354062635
1.1981747257584	1.5292339090808
1.1983263994266	1.5293661309645
1.1984826505905	1.5297447640534
1.1986417869423	1.5300501585889
1.1987956593102	1.5301940267373
1.1989721251394	1.5304465370149
1.1991098490799	1.5308500289332
1.1992599729239	1.5309327208308
1.1994286618612	1.5309902362847
1.1995747923616	1.5312713000902
1.1997186887298	1.531325238994
1.1998873288412	1.5318254774393
1.2000426071711	1.5318530682633
1.2002073029532	1.5320570302867
1.2003728323809	1.5322633088006
1.2005155378482	1.5322729573887
1.20068203004	1.5323019695579
1.2008421028821	1.5329573988533
1.2010062729261	1.5330434926
1.2011838149385	1.5334684808031
1.2013511616818	1.5334696479086
1.2014756526412	1.5336541379317
1.2016201521909	1.5338584754584
1.2017560991381	1.5338611827837
1.2019176296645	1.5342834644369
1.2020636844869	1.5343524217595
1.2022146359883	1.5343536390846
1.2023730162173	1.5343546609578
1.2025097648229	1.5346385518049
1.2026620459711	1.5348637150383
1.2028202800971	1.534908157693
1.2029952044313	1.5349666260791
1.2031437686953	1.5351815100904

1.2033173947951	1.5353507433222
1.2034603991792	1.5353946690671
1.2035977265208	1.5355170938466
1.2037354862661	1.5359851591201
1.2038984310193	1.5362739705453
1.2040192228919	1.5362751190451
1.2041675046521	1.536282496191
1.2042943146996	1.536534122333
1.2044332073411	1.5374265619673
1.2045867623402	1.5378299771377
1.2047418445435	1.5379710433511
1.2048823691935	1.5387164431624
1.2050562738236	1.5387658672185
1.2052097692472	1.5388251664439
1.2053656111398	1.5389334829976
1.2055324059849	1.5389788544637
1.2057058019972	1.5394724728263
1.2058653795764	1.539484787309
1.205993755368	1.5398575657897
1.2061457075707	1.5398643209001
1.2063094637485	1.5404323867538
1.2064580553723	1.5404383047989
1.206646683963	1.5404635692591
1.2068202985009	1.5406622502588
1.2069665165376	1.5416747869834
1.2071167422957	1.5417553463519
1.2072622439318	1.541996812651
1.2074021862632	1.542176766709
1.2075591529043	1.542286507141
1.2077202967919	1.5425191768888
1.207882407937	1.5425576977766
1.2080310618062	1.5426049611455
1.2081521600611	1.5431264361231
1.208279424963	1.5431769450636
1.2084266950368	1.5432609803652
1.2085568765268	1.5433608124125
1.2087208631504	1.5438813331191
1.2088707244485	1.5441211142997
1.20901426411	1.5443297585007
1.2092025069531	1.5444440405934
1.2093780928289	1.544464704439
1.2095265360609	1.544613029211
1.2097141326374	1.5450225662802
1.2099174088476	1.5450329637925
1.2100726217755	1.5451995440068
1.2102195898745	1.5453791981865
1.2103709177556	1.5455599109177
1.2105272483213	1.5455713985205
1.2106761628472	1.5457085052654
1.2108407106949	1.5457373968885
1.2109927874003	1.5466085402654
1.211156861057	1.5468074922441
1.2113076093084	1.5468830927114
1.2114488893753	1.5473266567399

1.2116008560791	1.5473394283174
1.2117368015591	1.5474108067841
1.211892771821	1.5476139842605
1.2120480855116	1.5482019721928
1.2121849323566	1.5488089428668
1.2123230279059	1.5488894027805
1.2124946672753	1.5490348446092
1.2126157013258	1.5498416287266
1.2127645702325	1.5503334265887
1.2128846686926	1.5504629156366
1.2130598357743	1.5510818168031
1.2132067080837	1.5512190900749
1.213343201627	1.551256915295
1.2134995498123	1.5513222653437
1.2136339826835	1.5514057475361
1.2137974640722	1.5518310242453
1.2139404757328	1.5519653545559
1.2140961893751	1.5532229051582
1.2142624106821	1.55330158656
1.2144006451524	1.5534838106817
1.2145641597854	1.5538615127659
1.2147277059307	1.5540847624784
1.2148695145213	1.5543677914737
1.2150323354778	1.5545663209476
1.2151780905631	1.554968829146
1.215323049951	1.5550855605364
1.2154944770772	1.5553830257603
1.215647294301	1.5554474798117
1.2157734835425	1.5563378018792
1.2159082967854	1.5564491307913
1.2160708957873	1.5565060132492
1.21621491745	1.5566201596472
1.2163664326225	1.5571085341161
1.2165164042477	1.5573967978354
1.2166532628331	1.5577337136989
1.2168231666249	1.557770175244
1.2169908770132	1.5580534287194
1.2171048996502	1.5583069154871
1.2172500528327	1.5593202394674
1.2173859258548	1.5596062770658
1.2175185737809	1.559762473351
1.2176952954152	1.5598007663027
1.2178718017883	1.5602044361628
1.2180069082411	1.5602200883385
1.2181711468339	1.5603392327478
1.2183382176569	1.560361789527
1.2184549747486	1.5604353257521
1.2186147319728	1.5610106181364
1.2187671750115	1.561240652193
1.2189419101091	1.5613044897717
1.2191086229094	1.5615467311852
1.2192647344949	1.5620858157287
1.2194420904785	1.5622621494849
1.2195758353476	1.5627179635268

1.2197702827508	1.5627615591006
1.2199071857293	1.5628922554141
1.2200749401573	1.5631598684234
1.2202293080912	1.5633835490862
1.2204052485316	1.563448911711
1.2205807635328	1.5634970750516
1.2207521518974	1.5636162106747
1.2209085615876	1.5636574548393
1.2210258435405	1.5638473426698
1.2212236822758	1.5638634176974
1.2213828494229	1.5640363721552
1.221527585606	1.5648335694386
1.2216668047403	1.5648704909191
1.2217966830177	1.5653944206947
1.2219249354493	1.5654867361021
1.2220819759681	1.5657068927118
1.2222405490863	1.5657094836931
1.2223973024502	1.5657728429737
1.2225808976066	1.5658619128358
1.2227518395756	1.5659423857172
1.2229077736086	1.5659806283443
1.2230686790988	1.5662094278989
1.2232515722369	1.5662671457787
1.2233847035241	1.5664541110086
1.2235539237432	1.5664552771783
1.2237036306228	1.5666195928335
1.223845656002	1.5674715818837
1.2240019837653	1.5677548469506
1.2241716867058	1.5679037033237
1.2243056087808	1.5679296423578
1.224477413698	1.568039487457
1.2246484265305	1.5680663693548
1.2248037680616	1.5686060296852
1.2249637235579	1.568642319501
1.225115900784	1.5696247730624
1.2252789797958	1.569674820516
1.225444414583	1.5698094993996
1.2255746371744	1.5698233312894
1.2257311132378	1.5698349201396
1.2259233669679	1.570278114008
1.2260957888073	1.5702884831505
1.2262712756512	1.5704477097225
1.2264171223322	1.5706758791231
1.2265568797868	1.5708101938787
1.2267565172261	1.5708780167273
1.2269167782111	1.5709874823888
1.2270503049481	1.5711206103678
1.2271964696041	1.5715449239626
1.2273642913419	1.5722944985133
1.2275249307155	1.5725702793786
1.227668972705	1.5728019185712
1.2278431300465	1.5729117865371
1.2280068597523	1.5735155416702
1.2282087860113	1.5736605797052

1.2283916973261	1.5736834635767
1.2285748796499	1.5738405256139
1.2287326380233	1.5739016550183
1.2289159015609	1.5742687376454
1.2290715216328	1.5743854983688
1.2292104020716	1.574592942342
1.2293644196595	1.5746774211831
1.2295150613404	1.5749831241086
1.2296761589484	1.5750998709252
1.229843548006	1.575358127402
1.229989749546	1.5754069993642
1.2301605870533	1.5756740296768
1.2303217195365	1.5757013303682
1.230509944693	1.5759598243417
1.2306754405085	1.5759862381776
1.230855275122	1.576196972145
1.2309856605797	1.5763678021761
1.2311403858047	1.576371136933
1.2313415080335	1.5765302684617
1.2314883930175	1.5768058975032
1.2316237325677	1.5769611372677
1.2317501173395	1.5769935392826
1.231911806167	1.5770347840067
1.2320390399254	1.5776591227825
1.232167993566	1.5776918413322
1.2323632854498	1.5780244690495
1.2325169754407	1.5782605016547
1.232682473541	1.5782953169533
1.232853593361	1.5784178439309
1.2330518018286	1.5784211633707
1.2332165506586	1.5788435112726
1.2333762871352	1.5789832180573
1.2335152716308	1.5790849312455
1.2336739273379	1.5791083291362
1.233826120616	1.5791577319551
1.233965912444	1.5793250180917
1.2341315779365	1.5794240121028
1.2343187079409	1.5795032432231
1.2344922490586	1.5796988703753
1.2346817521093	1.5799596384659
1.2348386183511	1.5799760709499
1.2350211025218	1.5802261851363
1.2351728291189	1.5804112813978
1.2353106199333	1.5806214810965
1.235486859369	1.5808390514151
1.2356582614553	1.5810035247138
1.2358181092651	1.5810533347323
1.2359991375728	1.5810546609451
1.236156437764	1.5811865413204
1.2362944714917	1.5812296962169
1.2364827465626	1.5812869577156
1.2366178854178	1.5812976841575
1.236754429151	1.5813728401656
1.2369197005121	1.5814185573751

1.2370847416356	1.5820274773148
1.2372615786387	1.5823383519783
1.2374121831136	1.5823774043507
1.2375510567433	1.5828438449576
1.2376898470856	1.5830523634193
1.2378307763058	1.5831711378841
1.2379736175463	1.5832763319001
1.238146953286	1.5834532392873
1.2383140869127	1.5835303210888
1.2384620833835	1.5835393613921
1.2386378409759	1.5837417325515
1.2387827355837	1.5837523369567
1.2389449653227	1.5837666357102
1.2391184954905	1.5837674300675
1.2392566580805	1.5837845280014
1.2394617938677	1.5838339312133
1.2396356263408	1.5839116311532
1.2398075903413	1.5839130498782
1.2399677926181	1.5841878015808
1.2401274933735	1.5846006494602
1.2403067764409	1.5851258013395
1.2404407832411	1.5854139579298
1.2406025217145	1.5854739361822
1.2407705883103	1.5857439135431
1.2409609335849	1.5864749553424
1.2411033204429	1.5871835459197
1.2412721632875	1.587223079814
1.241450153041	1.5872794939639
1.2416141441422	1.58737944478
1.2417631067811	1.5874724341403
1.2419284968905	1.5876637792318
1.2420867845913	1.5879882793664
1.2422480590654	1.5883179077971
1.2424337916624	1.588322768162
1.242621753847	1.5889294064077
1.2427547920149	1.5890842655831
1.2429022864522	1.5895075463535
1.2430666992772	1.5895560251187
1.2432426189507	1.5898645477554
1.2434148119078	1.5898702534653
1.2435872084023	1.5899557526791
1.2437304339037	1.5902093973942
1.2438816748434	1.5906167364405
1.2440546500097	1.5906888532113
1.2442103722276	1.590908430552
1.244376230138	1.591882946919
1.2445514929052	1.5920179524935
1.2447515356741	1.5924280834603
1.2449117828554	1.5927068503628
1.2450568878907	1.5931782536376
1.2452190542485	1.5933230554094
1.2453914454913	1.593889120348
1.2455676380457	1.5940418066832
1.2457486251611	1.5940732080929

1.2459082286035	1.5940922988874
1.246118573145	1.5942530909069
1.2462937001402	1.594995314037
1.2464654474646	1.5949955409098
1.2466039845595	1.5952474615416
1.2467759869866	1.5952572330242
1.2469076200415	1.5952633938965
1.2470633761801	1.5955637963597
1.247204006069	1.5960781583148
1.2473956002041	1.5961980482538
1.2475859058966	1.5962841560338
1.2477649351101	1.5971624047575
1.2479138501569	1.5971868702369
1.2480704789505	1.5973339018643
1.2482423403691	1.5978576654572
1.2484439875051	1.5981537271031
1.2485880187856	1.5983027125092
1.2487448924577	1.5984243043806
1.2489123486015	1.598542018336
1.2490653053503	1.5990554996496
1.2492358617054	1.5991432654231
1.2493684918996	1.5993589901542
1.2495388892107	1.5995808245277
1.2496856801455	1.5999420126284
1.249793590651	1.6000687473787
1.2499650870448	1.600518884789
1.2500833810185	1.6006185635366
1.2502713348487	1.6006446675555
1.2504461546708	1.600975492698
1.2506255428788	1.6018401759294
1.2507729699252	1.6018758261083
1.250932265266	1.6020208947269
1.2511018792778	1.6023821273096
1.2512743020892	1.6025244542929
1.2514027968962	1.6029470767457
1.2515640675332	1.6029539568753
1.2517145640995	1.6034665024161
1.2518528901014	1.603556605469
1.2520094061223	1.6038927474808
1.2521317300107	1.604349739273
1.2523232459066	1.6045294909127
1.2524781660606	1.6049530916997
1.2526617621368	1.6053436363129
1.2528383989383	1.6056047421681
1.2530265056852	1.6057165676919
1.2531875727381	1.6059241653033
1.2533291526775	1.6061010505885
1.2534980155439	1.6067130221483
1.2536591407972	1.6070110596313
1.2538142331436	1.6070770953406
1.253994039033	1.6072963498575
1.2541495613405	1.6073318484907
1.254333869572	1.6075047510415
1.2544758916447	1.6075293450111

1.2546394927583	1.6076271685471
1.254788457345	1.6076606872919
1.254964342509	1.607769918883
1.255096752143	1.6078086102979
1.2552699734553	1.6080880979836
1.2554380940574	1.608252602079
1.2556044381466	1.608341225151
1.2557669408534	1.60866698699
1.2559436744613	1.6087551762277
1.256122337407	1.6088414804034
1.2562816921358	1.6088591493214
1.2564128093868	1.6089881222112
1.256550545893	1.6091168055219
1.2567123018453	1.6091600247816
1.2569008063274	1.6094173079299
1.2570405685062	1.609581517937
1.2572096245277	1.6098283660743
1.2573517835027	1.6098471403588
1.2575125694045	1.6105250996854
1.257681101061	1.6106785878839
1.2578130622258	1.6107810409183
1.257991510601	1.6111274917357
1.2581588606397	1.611624606081
1.2583336425751	1.6118703072037
1.2585161769653	1.6125520155284
1.2586742980976	1.6126218114781
1.2588565288054	1.6129511908188
1.2590178639292	1.6130672904405
1.2592062917486	1.6131992410797
1.2594002846616	1.6133611843808
1.2595651249378	1.6134097915348
1.2597432675303	1.6136895316672
1.2599184767735	1.6143225955242
1.2600987491911	1.6143761917628
1.2602548106686	1.6155590457394
1.2604142942839	1.6155976620639
1.2605742313684	1.6156228246999
1.2607488319339	1.6157651720235
1.2609293347701	1.6158093733124
1.26108655301	1.6158716676723
1.261257815085	1.6163820826371
1.2614242329068	1.616515144919
1.2615852152174	1.6166882682312
1.2617435866723	1.6167864258273
1.2619498941871	1.6168731858256
1.2621246698019	1.6173098603553
1.262274022744	1.6174415145638
1.2624646595678	1.6176186960689
1.2626200608204	1.6178461153319
1.2628058500442	1.6182527898132
1.2629754374851	1.6192959828849
1.2631299673887	1.6196188865953
1.2632582191922	1.6198894415159
1.2634219927871	1.6212473887148

1.26357258582	1.6215984052709
1.2637436933958	1.6216470830123
1.2639221756542	1.6217018347959
1.2640767796055	1.6219083067683
1.2642258857586	1.6227846467599
1.2643871092884	1.622787751157
1.264558380299	1.6228309728452
1.2647094188699	1.6229339069911
1.2648961733752	1.6229347548352
1.2650768623536	1.622999427263
1.2652493209141	1.6233326371829
1.2654153360333	1.6234520773735
1.2655933630146	1.6237067872972
1.2657075259939	1.6243059866029
1.2658714635274	1.6244864343161
1.2660521773281	1.6245410705197
1.2662402182489	1.6247932182584
1.2663788786465	1.6248242018152
1.2665597252495	1.6250251918519
1.2666886269666	1.6250674514406
1.2668775408508	1.6250958352762
1.2670428723578	1.6257183257229
1.2672064937738	1.6258088028559
1.2673567413738	1.6261742341707
1.2675409444354	1.626237405678
1.2677381780149	1.6263866577681
1.2678569781384	1.6265929656972
1.2680514322692	1.6266584413282
1.2682521376152	1.6270604133429
1.2684304671407	1.6270827252872
1.268569081652	1.6273117070832
1.2687315033182	1.6278653237887
1.2688857483414	1.6279681716816
1.2690388033173	1.6281667390945
1.2692062170997	1.6282631884324
1.269429407255	1.6282716853333
1.2696251655841	1.6283505963217
1.2697856419234	1.628484938383
1.2699532082269	1.6295069179236
1.2701243779999	1.6303280427264
1.2702714827932	1.630598449773
1.2704321916315	1.6308469784824
1.2705808773644	1.6309372554612
1.2707520848565	1.6310474748588
1.2708806270749	1.6311367919371
1.2710529144189	1.6312656144866
1.2712027765445	1.6313134636438
1.2713748839829	1.6314096302933
1.2715683153459	1.6319618784727
1.2717412482324	1.6319921261114
1.2718991652165	1.6325455524482
1.2720955837379	1.6325920984465
1.2722699549005	1.6326301166201
1.2724170965737	1.6333456383997

1.2725960310026	1.6334397628651
1.2727368526669	1.6334627768587
1.2728928431586	1.6339014356397
1.2730653870218	1.6339214852764
1.2732571073115	1.634043950307
1.2734358215795	1.6347676605016
1.2735532835557	1.6348463484337
1.2737518962344	1.6351860192782
1.2739167352154	1.6355568405511
1.2741078882042	1.6357437339202
1.2742807476581	1.6358368342171
1.2744671499721	1.636035750319
1.2746660412251	1.6361705468919
1.2748278824413	1.6361868166194
1.2749766343728	1.6366567026208
1.2751470436061	1.6366948968641
1.2753235448993	1.6371351461413
1.2754775342658	1.6377242482804
1.2756236199088	1.6385976223247
1.2757900525924	1.6395564935679
1.2759801856968	1.6400950932705
1.276141415083	1.6402576767084
1.2763115551662	1.6403780204948
1.2764697370583	1.641014384561
1.2766764767433	1.6410790743966
1.2768503541565	1.641174258633
1.2769788740298	1.6412796152853
1.2771506240525	1.6413632379973
1.2773446632751	1.6418800452124
1.2775034032563	1.6424580336137
1.2776650886722	1.643066549754
1.2778544261937	1.6431690004608
1.2780439061681	1.6440979252738
1.2782343820583	1.6443734578314
1.2784188837142	1.6445122380441
1.2785962142087	1.6448085250373
1.278780773148	1.645023827477
1.2789494701542	1.6453519529567
1.2791435938201	1.6457005877574
1.2793279961032	1.6460854094987
1.2795220700635	1.6466867375002
1.2797268035723	1.6466934959403
1.2798906607087	1.6466975095479
1.2800561910745	1.6469270182167
1.2801977319639	1.6471157628632
1.280349172141	1.6471411557061
1.2805179295678	1.6474617274633
1.2806947641359	1.6478221005244
1.2808419031978	1.6481122871163
1.2810363135144	1.6482213054937
1.281221887921	1.6488935839447
1.2813726985621	1.6493019133451
1.281546223866	1.6495053645896
1.2817092431337	1.6499588071379

1.2818671106426	1.6500078816039
1.2820091027164	1.6503735222634
1.2821871126082	1.6507568038725
1.2823802934371	1.6508197408173
1.282519774368	1.6510567951075
1.2826690695057	1.6511226575372
1.282837367374	1.6513798508921
1.2829790499992	1.6524416685148
1.283159064552	1.652545291089
1.283322555761	1.6526680488062
1.283481622631	1.6528971242458
1.2836686217907	1.6528983327253
1.2838314199472	1.6533146151402
1.2839980523185	1.6534012104048
1.2842014794201	1.6537263276217
1.2843595641345	1.6539881375946
1.2845218964255	1.6540854367398
1.2846765418666	1.6543356921121
1.2848671887519	1.6543553836712
1.2850526681451	1.6547760457072
1.2852178772579	1.6547772981472
1.2854278808341	1.6547800063796
1.285600347164	1.6552223608673
1.2857789105003	1.6553439786771
1.2859657656779	1.6559836541564
1.2861797650139	1.6560540440126
1.2863190164484	1.6561189079539
1.2864774952659	1.6563553597866
1.2866726468315	1.6564703569222
1.2868384541481	1.6566886231464
1.2870052765625	1.6567309430705
1.2872124145171	1.6567462510378
1.2873902027613	1.656865094985
1.287566800752	1.6569914458774
1.2877482758161	1.6569967980495
1.2879154625097	1.657187234641
1.2880709855289	1.6573284225457
1.2882278686207	1.6573386536584
1.2884038334551	1.6574024967158
1.2885585635133	1.6576457370429
1.2887322500631	1.6577585955933
1.2889224998703	1.657888050569
1.2890713945269	1.6583942126063
1.2892568083755	1.6586857699777
1.2894554006847	1.6588163075299
1.2896268898899	1.6593966739566
1.289818655549	1.6595840218152
1.2900136052143	1.659674867954
1.2901796572637	1.6596858107931
1.2903734251919	1.659750582483
1.2905207571337	1.6597988903601
1.2906938668917	1.6599173426578
1.2908526509646	1.6599516989135
1.2910328767569	1.6600456922413

1.2912316041248	1.6604489756555
1.2914021132471	1.6608584370701
1.291569067618	1.6613833235957
1.2917605691138	1.6616450740981
1.2919575363841	1.6617475997225
1.2921650901189	1.6619087991324
1.2923209535212	1.6619549828894
1.2924909103017	1.6621244221656
1.2926412322175	1.6621327814563
1.2928437814369	1.6621743858617
1.293016613573	1.6626751667668
1.2931966747551	1.6633608110322
1.2933559330984	1.6641358437503
1.2935057595951	1.6643109431553
1.2936781312189	1.6646799148205
1.2938596610966	1.6652065395894
1.2940072624725	1.666206733053
1.2941602539889	1.6662132699185
1.2943352315224	1.6663597477919
1.2945400714212	1.6663606744926
1.294686107334	1.6663670743722
1.294855115092	1.6664591966642
1.2950222375487	1.6665332823729
1.2951683163443	1.6667585024221
1.2953367862706	1.6671385783433
1.2955156603035	1.6675602846236
1.2956659140965	1.6678432466312
1.2958320147268	1.6683489824388
1.2960117853988	1.6683992113846
1.2961605546599	1.6686401749091
1.296350662931	1.6694573793556
1.2965094039743	1.6696606411483
1.29667108273	1.670281877841
1.2968776695969	1.6705135395278
1.2970771474457	1.6705632178104
1.2972421408143	1.6708230809877
1.2974051635064	1.6712602577326
1.2975763211075	1.6720411066068
1.2977412637253	1.6721622029066
1.2979507010877	1.6722183100867
1.2981168870817	1.6722458325187
1.2982631652876	1.6724010944099
1.2984147159665	1.6724705943258
1.2985963487398	1.6725142482851
1.2987923094733	1.6729011049148
1.2989591475941	1.6744453716531
1.2991303233148	1.6744780138705
1.2993051142296	1.6746799396577
1.2994595057553	1.6749383149297
1.2996797398141	1.6750169459892
1.2998353892396	1.675080090928
1.3000130676987	1.67537433801
1.300235997024	1.6754087734872
1.3004302365247	1.6756731024783

1.3006187714302	1.6767657975129
1.3007934169274	1.6772492623887
1.3009384084882	1.677512856576
1.3011022206236	1.6775309539511
1.3012957058898	1.6776993263696
1.3014749313786	1.6787498384131
1.3016659650927	1.678841943751
1.3018327067954	1.6789426470135
1.3019989362552	1.6791453743198
1.3021482499193	1.6792901822245
1.3023450117184	1.6795442713298
1.3024896704782	1.6801665874528
1.3026472231878	1.6803382983766
1.302817895075	1.6804445343393
1.3029654938451	1.6805518232229
1.3031287503151	1.6807317745152
1.3033071297523	1.681237299286
1.3034811814088	1.6814585451865
1.3036428299323	1.6826364913873
1.3038160356413	1.6832253554231
1.3040245504868	1.6832261905424
1.304198208242	1.6832985558115
1.3043765829342	1.6834756545701
1.3045506504949	1.6835364106433
1.3047512304141	1.6836884323181
1.3048832127881	1.6841651331817
1.3050663573564	1.6850956998504
1.3052011620465	1.6851544793826
1.3053620182868	1.6852209593675
1.3055321695188	1.6854480561718
1.3057348110271	1.6862107866825
1.3059206081126	1.6862808116568
1.3060947510908	1.6865808300771
1.3062545404008	1.6867058612908
1.3064027575319	1.6871845344593
1.3065735082413	1.6872742637983
1.3067658541967	1.6875464439942
1.3069276767331	1.6876560433333
1.3071114106786	1.6880758934483
1.3073131710196	1.6880853273044
1.3075022841814	1.6883010543107
1.3077007860011	1.6883640865264
1.3078763078151	1.6883805008399
1.3080492038203	1.6891816603662
1.3082331001442	1.6896215569354
1.3083941422146	1.6906095526057
1.3086135287345	1.6908111597286
1.3088194943649	1.690960947853
1.3089714472455	1.6912076556926
1.3091307968155	1.6912509675393
1.3092850572876	1.6912680627075
1.3094834406066	1.6919880850628
1.309668055609	1.6922151295107
1.309838077877	1.6922646838793

1.3100098491098	1.6928408504938
1.3101627188295	1.6937953599904
1.310384904363	1.6943027546377
1.3105903105696	1.6943193666409
1.3107585915952	1.694483763074
1.3109489860797	1.6949945642365
1.3111548950233	1.6950854797599
1.3113081604647	1.6954565670049
1.3114910030648	1.6955380772245
1.3116822456928	1.6956261965579
1.3118628059576	1.6958668777971
1.3120680325826	1.6959220533991
1.3122363949204	1.6960542910381
1.3123738492235	1.6960602370102
1.3125349377348	1.6962355758484
1.3126841233713	1.6962708191053
1.3128360205765	1.6967285750517
1.3130291156401	1.6969968239151
1.3132063396297	1.69721815252
1.3134150003135	1.697227157775
1.3135802449214	1.6972380677394
1.3137432456456	1.697405316353
1.3139414884751	1.6974133051032
1.3141074015054	1.6976415912811
1.3142822195395	1.6980354290788
1.314469138888	1.6981048517156
1.3146558780732	1.6981995957058
1.3148264985819	1.6982686654353
1.3149917595352	1.6984397378023
1.3151498666516	1.6985782480521
1.315358120042	1.698700618907
1.3155368388019	1.6992868964645
1.3157020987427	1.6996553369269
1.315873275326	1.7002371976801
1.3160585204692	1.7003385881698
1.3162037485128	1.7005349869576
1.3163596399606	1.700763080448
1.3165781099126	1.7011473599763
1.316753243265	1.7015886804598
1.316942782434	1.7017298307424
1.3171380053404	1.7018025075134
1.3173503131204	1.7019575299578
1.31756547422	1.70204228642
1.3177356219544	1.7021213450445
1.3179093234911	1.7029310496686
1.3181201216764	1.7033015810943
1.3183024222716	1.7038966488868
1.3184659277321	1.7040195887967
1.3186207675078	1.7046843640389
1.3188147113624	1.7048135915875
1.318975282503	1.7051227427916
1.3191493847012	1.7051523988886
1.3193386419921	1.7053860659714
1.3195283419198	1.7055097751221

1.3196940825364	1.7063694252489
1.3198820034788	1.706408038603
1.3200874292052	1.7071840177214
1.3202415229848	1.7072891255178
1.3204038125613	1.7073419738669
1.3206026582219	1.7074139010111
1.3207814278802	1.7075736579416
1.320930256002	1.7076227682153
1.3211575030573	1.7078661881128
1.3213593703399	1.7083539746147
1.3215648192192	1.7083622466393
1.3217572573641	1.7086794063342
1.321963149092	1.7087248266599
1.3221543705309	1.7087789067403
1.3223153754919	1.709403516694
1.3225097676721	1.7096127524667
1.3227074171107	1.7097519961867
1.3229021585645	1.7097939497412
1.3230625224593	1.7102178139235
1.3232740936916	1.7112887810938
1.3234506268977	1.7114030841097
1.3236371409231	1.7114665848932
1.3238251549067	1.7116227695425
1.3240215140284	1.7119982740446
1.324251657285	1.7125818285991
1.3244122923211	1.7128753902084
1.3245557273334	1.7131968009956
1.3247557965696	1.7132908235827
1.3249516850643	1.7135149189209
1.3251281562723	1.7137181178516
1.3253620098785	1.7137678786862
1.3255631304255	1.7138305828628
1.3257452218555	1.7139342413632
1.3259066053107	1.7139942294786
1.3260591241911	1.7141685376117
1.3262241310163	1.7142232862844
1.3264046027286	1.7144561563742
1.3265770747134	1.7145634625899
1.3267628516406	1.7145783170847
1.3269335402396	1.7146820259542
1.3271045440773	1.7147201391522
1.3272711380907	1.714857275115
1.3274261373066	1.715034427973
1.327617419223	1.7152041343667
1.3277669330556	1.7154745190114
1.3279411670099	1.7161671230098
1.3281308906284	1.7164399542384
1.3283330297141	1.7166459780936
1.3285426735431	1.7168997454488
1.328744513706	1.717330914577
1.3289553255143	1.7174185844364
1.3291431498034	1.7183999238051
1.3293440964316	1.7184466946453
1.329549989684	1.7184997231394

1.3297214985818	1.7189248578289
1.3299100824833	1.7189688953233
1.3300856827498	1.7190181243825
1.3302633789503	1.7194335834607
1.3304418991455	1.7198720209878
1.3306251312832	1.7201886878151
1.3308097927083	1.7205416427215
1.3310333419115	1.7205580229792
1.3311750749591	1.7216884755861
1.331383281507	1.7218098144196
1.3315498056844	1.7219329654395
1.3317478693928	1.7222755840241
1.3319779051076	1.7225902695171
1.3321844796054	1.722812771966
1.3323823691346	1.723036363741
1.3325613844716	1.7230581680428
1.3327432198801	1.7236384394871
1.3329342284	1.7237075288456
1.3331188982885	1.7237902048058
1.3333004579684	1.723902643336
1.3334777953057	1.7249078658337
1.3336752372065	1.7249546655933
1.333857843307	1.7254501334921
1.3340367219333	1.725920664018
1.3341876745348	1.7260967509418
1.334379623544	1.7261252266133
1.3345645222212	1.7266594047342
1.3347666045114	1.726702565312
1.3349544878846	1.7268716760965
1.3351237561894	1.7269509045148
1.3352823159816	1.7272053627406
1.3354449281099	1.7272091982181
1.3356152429711	1.72737129809
1.3357635663462	1.7273750423905
1.3359310601708	1.7275439726789
1.3361421477577	1.7275880491718
1.3363187520569	1.7279152176008
1.3364843734246	1.7289604670551
1.3366671228476	1.7302992402911
1.3368697006032	1.7305499871187
1.3370534859365	1.730809558866
1.3372559223881	1.7308492468996
1.337437243695	1.7313212735133
1.3376203023103	1.731698284288
1.3378380190599	1.7317514715471
1.3380558985392	1.7319242976772
1.3382454448375	1.7321861067666
1.3384456348463	1.7324057721612
1.338620100172	1.732776999306
1.3387836436797	1.7333121660961
1.338993869205	1.7340681736933
1.3391638356222	1.7343103087633
1.3393347250185	1.7345159151327
1.3395230123418	1.7345306792954

1.3397148310528	1.7346159249957
1.3398770013714	1.7351433891354
1.3400545167134	1.7357793679694
1.3402385601223	1.7372903995957
1.3404580915569	1.7373660791496
1.3406454541968	1.7377547646297
1.3408305651703	1.7384203474612
1.3410185539167	1.7385731345606
1.341198704354	1.7390300190398
1.3414474714719	1.7390810676421
1.3416497498799	1.739171997797
1.3418357628444	1.7394557740299
1.3419810373892	1.739499100049
1.3421889966433	1.7395345912262
1.3423739439353	1.739935943126
1.3425596750289	1.7403196871833
1.3427579415838	1.7406334080301
1.3429710592831	1.7407764038344
1.3431492212396	1.7410363650072
1.3433190806311	1.7413734344646
1.343503026091	1.7414539691688
1.3437046794444	1.742032632652
1.3439002040087	1.7426066759829
1.3441038967228	1.7426979686318
1.3443203194673	1.7429619846669
1.3445189090045	1.7436502637217
1.3446897299865	1.7439381736771
1.3449203841463	1.7440081929725
1.3450895961586	1.7440330696983
1.3452738309491	1.7444623884926
1.3454649440916	1.7446541302633
1.3455951014343	1.7449879826186
1.3457614406282	1.7460558695664
1.3459589563179	1.7462482162847
1.3461641037808	1.7464734616811
1.346380857895	1.7472251811829
1.3466098346814	1.7475574225388
1.3468053281873	1.74961723312
1.3469829751244	1.7498228791548
1.3471432594542	1.7507083314335
1.3473238292616	1.7514431556529
1.3475059276275	1.7516137763145
1.3477322125037	1.7516768097815
1.3479214601309	1.7526916973475
1.3480730950739	1.7529936048725
1.348244610883	1.7531569761066
1.3484145079336	1.7534755561375
1.3485840839108	1.7539309510552
1.3487498378542	1.7540746146262
1.348997643555	1.7541430419824
1.3491735324651	1.7546156905478
1.3493555045503	1.7549927979166
1.3495468770832	1.7550895079191
1.3497061037813	1.7551524043148

1.3498740678377	1.7553173882024
1.3500281999181	1.7557391470238
1.3502134522342	1.7567128087274
1.3504034637333	1.7568328691132
1.3506120704567	1.7587125383737
1.3507864787213	1.7589598648082
1.3510126166596	1.7589769702347
1.3512073990427	1.7591934044919
1.3513838060476	1.7594277884346
1.351558853665	1.7596620488253
1.3517333254149	1.7596750236014
1.351922030888	1.7597027349369
1.3520988804174	1.7600415201703
1.3522472502024	1.7601215369153
1.3524639658719	1.7603429383714
1.3526584215925	1.7605081814278
1.3528727792049	1.760857758884
1.3530342943115	1.7614985484049
1.3532331716816	1.7615493551031
1.3534196571093	1.7622253715661
1.3536212674458	1.7622607202814
1.3537883265658	1.7627147427956
1.3539632592829	1.7627501768145
1.35412168317	1.7627990103578
1.3542874614281	1.765152703226
1.3544902370818	1.7663331599996
1.3547057805637	1.766552492471
1.3548717278091	1.7667243911774
1.3550916851063	1.7672590472899
1.3552260912308	1.7675581035466
1.3554261544085	1.7679810562529
1.3555879447988	1.7680950046325
1.3557827314191	1.7681805837029
1.3559516240468	1.7682382639578
1.3561445930911	1.7695718987803
1.3563463020917	1.7697685450407
1.3565432392556	1.7700118898167
1.3567227450372	1.7702607788511
1.3569336792164	1.7704934597281
1.3571294226852	1.7706848884587
1.3573154763069	1.7707001634416
1.3574671395345	1.7707850597078
1.3576221783528	1.7708830424973
1.3577944502884	1.7710280326741
1.3579876549677	1.7723877982133
1.3581705293532	1.7725966653831
1.3583800205411	1.772761122572
1.3585808179143	1.7733170566538
1.3587651864409	1.7735734800097
1.3590255244294	1.7739522011292
1.3591793102069	1.7744690336207
1.3593661397361	1.7747259136046
1.3595960696318	1.7749184958223
1.3597760624316	1.7751471286387

1.3599642959846	1.7757981786752
1.3601792173243	1.7759407500367
1.3603856021159	1.7761901606729
1.3605811978985	1.7767541277612
1.360775648126	1.7767822565414
1.3609407738458	1.7773065956038
1.3611088858459	1.7776766677448
1.3613123097263	1.7777608209625
1.361522247411	1.7780980830412
1.3617225112155	1.7791560600225
1.3619266974453	1.780000219173
1.3620981262203	1.7802320829895
1.3622491712095	1.7811817570769
1.3624267510459	1.7813109367624
1.3626329035965	1.7815538386019
1.3628086082389	1.7818535334097
1.362972357936	1.7820233281876
1.3631417255679	1.7820817124901
1.3633182164599	1.7821779017712
1.3635298945654	1.7825976034037
1.3637228626188	1.7826705908116
1.3639215276201	1.783182446768
1.3641301140631	1.7837031304734
1.3643632975043	1.7838290075943
1.3645683357641	1.7848047344966
1.3647916804041	1.7849466364017
1.364975963453	1.784997307913
1.3651382671979	1.7851025682242
1.3653516718522	1.7851279459317
1.3655104605681	1.7852672760727
1.3656849075969	1.7855481338944
1.3658787537016	1.785796696244
1.3660414094459	1.7857972916003
1.3662345071079	1.7860088699393
1.3664113232163	1.786340528995
1.3666154271462	1.7872413006227
1.366804882258	1.7872592764018
1.3670096045708	1.7876792042029
1.3671801808832	1.7878440870165
1.3674251126668	1.7878970429033
1.3676041303705	1.7879410438779
1.3677896276601	1.7881618260352
1.3680103220607	1.7882382501742
1.3682319966242	1.7886639696335
1.3684725877996	1.7892676420107
1.3686794096934	1.7894911667159
1.3688442885382	1.7900449828049
1.3690495534511	1.7904880313086
1.3692077829142	1.7911527794636
1.3693932235009	1.7912013521294
1.3696019263645	1.7912924824951
1.3697716691931	1.7915321472199
1.3699539048812	1.7915784953609
1.3701277671908	1.7917662095243

1.370320540111	1.7917844956991
1.3705310412378	1.7919026583151
1.3707131215754	1.7919417594989
1.3709519015075	1.7924063296815
1.3711331661125	1.7928262957388
1.3713733473619	1.792952251035
1.3715238497197	1.7930204707988
1.3717137842544	1.7935176861299
1.3719105543097	1.7935301187557
1.3721348293037	1.7936808831727
1.3723301049124	1.7943349621
1.372534806639	1.7953599741853
1.3727271503328	1.7961483488423
1.3729169028953	1.7965433145473
1.3730957710881	1.7966991165488
1.3732400808485	1.7967269991372
1.3734121296616	1.7971284728487
1.3735927984786	1.7972439744316
1.373758606208	1.7973351820203
1.373922313353	1.797535884005
1.37413001966	1.7977107246142
1.374287246261	1.7977780415852
1.3744798220155	1.797891232465
1.3746673055015	1.7983550778326
1.3748221711417	1.79842559701
1.3750082406382	1.7985373654125
1.3752435880055	1.7987744301566
1.3755135724461	1.7998151285712
1.3756888673661	1.7998336614684
1.3759088776861	1.7999165352802
1.3761032152276	1.8000055021114
1.3762967170241	1.800488164173
1.3765102759728	1.8008304726936
1.376686084418	1.8016366121753
1.3768934554102	1.8016653865856
1.3771043794818	1.8018403178903
1.3772761458103	1.8023386573674
1.377461052036	1.8029132445647
1.3776606697236	1.8032208510766
1.3778552601383	1.8035656775182
1.3780676290823	1.8041804526776
1.3782491524927	1.8042408543228
1.3784616479801	1.8042601290575
1.3786300481046	1.8048004491185
1.3788438558396	1.8059529678512
1.3790148132833	1.806544267949
1.379214736997	1.8067617143514
1.3794453766464	1.8070378062268
1.3796296833796	1.8077352238559
1.3798233038084	1.8084231716055
1.3800118004967	1.8086768892527
1.3802125134313	1.8093190508136
1.3803881643453	1.8094605425921
1.380592675586	1.8095215058096

1.3808402488231	1.8096875072761
1.3810212448632	1.8103653465976
1.3812100324536	1.8108439872771
1.3813941013023	1.8109027265367
1.3815933505959	1.8113632055532
1.3817957344062	1.8114016946566
1.3819705776752	1.8114542450067
1.3821536082936	1.8119156154288
1.3823070934853	1.8119725874535
1.3824800029681	1.8121451718684
1.3826896137361	1.8122320427916
1.382849192708	1.8126942317914
1.3830384746327	1.8127793971081
1.3832391628731	1.8137518965194
1.3833786170057	1.8138757358004
1.3835738552092	1.8144783018372
1.3837660028388	1.814569137003
1.3839560801011	1.8146776270236
1.3842115046906	1.814943588018
1.3844062378627	1.8153718634269
1.3845946819966	1.8174830445185
1.3847900589421	1.8174963176809
1.3850302796209	1.8179806363797
1.3852039386227	1.8179866512103
1.3854000659771	1.8180013747278
1.3855799767249	1.818169695849
1.3857789726698	1.818462685971
1.3859697847286	1.8195296912254
1.3861878166553	1.8203850411593
1.386344635327	1.820396582022
1.3865510060536	1.820517862396
1.3867687991758	1.8205613956486
1.3869714863053	1.8209644229208
1.3872360736931	1.8210837077676
1.3874439165463	1.8213944596673
1.3876481640457	1.8216746001288
1.3878132562262	1.8220110867178
1.3879984711265	1.8225966218766
1.388192726428	1.8226984939009
1.3883985216519	1.823649770962
1.3885810654087	1.8237476191697
1.3887760750221	1.8248483800842
1.3889844782689	1.8252217410848
1.3891663448575	1.8255800100897
1.3893841010824	1.8257764040882
1.3895784572567	1.8265615942334
1.3897715860599	1.8266393953048
1.3899284455021	1.8267692830429
1.3901150152414	1.82697821994
1.390318474162	1.8280811153992
1.3905278214381	1.8281723095496
1.390731778512	1.8281837372665
1.3909051731607	1.8281849052356
1.3911474194185	1.8285165194777

1.3913306431254	1.8291231843833
1.3915270868417	1.8292276123135
1.3917364364801	1.8298952531815
1.3919335826693	1.830177228157
1.3921404080979	1.8301841360645
1.3923398420105	1.830552150369
1.3925369975198	1.8309889016548
1.3927653593789	1.8311760800837
1.3929490242399	1.831348143358
1.3931449315737	1.8314096574362
1.3933246040534	1.8317538503904
1.3935012656036	1.831977745791
1.3936469671336	1.8320010111711
1.3938392731179	1.8322314509812
1.39406862367	1.8330109559769
1.3942908262455	1.8330682144385
1.3944643129778	1.8334860654277
1.394633368226	1.8334888163197
1.3948496100369	1.8335911618183
1.3950247104141	1.8336024989395
1.3952503246091	1.8337546583845
1.3954625102714	1.8338976197722
1.3956465460544	1.8339550793898
1.3958479836831	1.8340663095966
1.3960428185996	1.8342295606311
1.3962487045908	1.834324763767
1.3964380958731	1.8343548365066
1.3966719624603	1.8346793888957
1.3968903485864	1.8349888948416
1.3971251211378	1.8356084899345
1.3973145953043	1.8360837850122
1.3975458337207	1.8361028608779
1.3977693873797	1.8365081744816
1.3979649100489	1.8369533778604
1.3981490810235	1.8370295903118
1.3983005144029	1.8371608946988
1.3984920390861	1.8373003218661
1.3986934182534	1.8392950652881
1.3989061745159	1.8403242615312
1.3991362578874	1.8404341348796
1.3993327500529	1.8404406364837
1.3995418554455	1.8405395750309
1.3997466079119	1.8408376650973
1.3999397010897	1.8408562142792
1.4001176989829	1.8411157226711
1.4003210816695	1.8416318180715
1.4005318091532	1.8417920859379
1.4007640627303	1.8418402266061
1.4009758900373	1.8419293035201
1.401157436897	1.8420073137707
1.4013978733538	1.8421952760995
1.4015989633379	1.8422924819544
1.4018076376331	1.8424519167819
1.4020220954313	1.84247692353

1.402236737485	1.8426572887548
1.4024459132717	1.842766861148
1.4026435979376	1.843132015422
1.4029213898561	1.8432381552118
1.403115643974	1.8433900392752
1.4033123186812	1.8436804319414
1.4034966934613	1.8436824166231
1.4036923227329	1.843872201281
1.4038953542656	1.8440625306673
1.4041103321135	1.8443088500136
1.4043303737799	1.8448009493748
1.4044987777527	1.8448173575403
1.4047129490889	1.845064079178
1.4048965249624	1.8452379761857
1.4051131574788	1.8454138084759
1.4052986493753	1.8454902578298
1.4054853377761	1.8463429081904
1.4056697084546	1.8465197528503
1.4058976590499	1.8473745321293
1.4061286247069	1.8481672952668
1.4063496884277	1.8482829179703
1.4065494147139	1.848340017583
1.4067482602363	1.8488877465936
1.4069754707547	1.8489675822958
1.4071545839225	1.8493101437121
1.4073679008005	1.8495324747854
1.4075310837627	1.8496923180676
1.4077391099466	1.8501609134611
1.4079316878056	1.8507611459525
1.4081235864762	1.8508242405542
1.4083587124459	1.8509377706225
1.4085521281976	1.8511034015178
1.4087520444822	1.8512785718268
1.4089418915153	1.8513036308184
1.4091438136181	1.8514340548333
1.4093386104812	1.8518295964238
1.4095777818635	1.8519142103
1.409821180099	1.852540060054
1.4100031194897	1.8531920365125
1.4102189726191	1.8532525693249
1.4103656683251	1.8541243529815
1.4106094503953	1.8543250716933
1.4108317048259	1.8544516598814
1.4110306781772	1.8544844953486
1.4112546294507	1.8545046663917
1.4114653829616	1.854718961252
1.4116831158964	1.8547770461829
1.4119025352597	1.8553124764578
1.4120602869311	1.8553253924087
1.4122324604781	1.8559228511355
1.4124349698831	1.8559664277116
1.4126558311352	1.8565057952119
1.4128553898562	1.8565665104587
1.4130863161236	1.856741876448

1.4133202415885	1.8569520286097
1.4135266926982	1.8569711401735
1.4136850729022	1.8574710486376
1.4139278422249	1.857494673643
1.4141081921069	1.8577286435671
1.4142997572101	1.8578612141309
1.4145034972922	1.8587599844311
1.4146807920607	1.8591721024687
1.4148850575235	1.8592714930122
1.4150510354827	1.8593135751329
1.415231427033	1.8603253668366
1.415431017098	1.860337005374
1.4156043563558	1.8616814746087
1.4157990037324	1.8618285328622
1.4159821189801	1.8622109953636
1.4161938680534	1.8622354546284
1.4163790746318	1.8623592784761
1.4165983877799	1.8624732297589
1.4168237705371	1.8625509201777
1.4169964283723	1.8625720701525
1.4171912356459	1.8626284793215
1.4173622871406	1.8634184053694
1.4175264623351	1.8635157888102
1.4177627438496	1.8636307807692
1.4179690114073	1.8636474010895
1.4181643922856	1.8638875840901
1.4183232658674	1.8644817624929
1.4185069143222	1.864765028698
1.4186746675335	1.8653282489514
1.4188612605771	1.8657915394525
1.4191185381722	1.8661976023438
1.4193250262597	1.8665488305594
1.4195204311156	1.8667478797679
1.4197422653152	1.8668204326737
1.4199490631784	1.8670833250638
1.4201885399916	1.8671363839986
1.4203800052574	1.8678500612437
1.4205737272968	1.8678640667314
1.4207736947518	1.86795085194
1.4209531996923	1.8681062915357
1.4211633112385	1.8684393194507
1.4213766410094	1.8687210447287
1.4215619685984	1.8698472547254
1.4217832182973	1.8701885533002
1.4219650849602	1.8702189374325
1.4221733122542	1.8706399189412
1.4223705293956	1.870674624737
1.4226049970941	1.8708849196893
1.4228201862659	1.871283275129
1.423058695516	1.8714116379066
1.423243166949	1.8718666927712
1.4234195987	1.8721366395722
1.4236772700073	1.8723548586944
1.4238900358269	1.872417701725

1.4240607872935	1.8734965209534
1.4242574874493	1.8737653812851
1.4245244604289	1.8746756768835
1.4247110265574	1.8753112413335
1.4249230205691	1.8759169740767
1.4251855048717	1.8763388636879
1.4254105596655	1.8763708354527
1.4256095370797	1.8764588699268
1.4258272856776	1.8765504919037
1.4260173903399	1.8773835296031
1.4262076444335	1.8775575027452
1.4264127690993	1.8780877640923
1.4266376607917	1.8781244236712
1.4268251340038	1.8782033206322
1.4270461054762	1.8785931557465
1.4272788433907	1.8797648852612
1.4274699396366	1.8799629738448
1.4276563251383	1.8800017279612
1.427877207943	1.8802754552489
1.4280959255576	1.8808134884909
1.4283349858158	1.8810188493343
1.4285668561655	1.8812501411662
1.4288019631511	1.881259468957
1.4289971741774	1.881311256527
1.4291926642008	1.8821818728931
1.4294380592766	1.8823014935473
1.4295712526414	1.8824940088092
1.4297676989924	1.883070842778
1.4299914599563	1.8834587339976
1.4301552511657	1.8843926779406
1.430400749118	1.8859738059657
1.4305946143485	1.8860812703199
1.4307978689412	1.8864672713701
1.4309994764353	1.8865747527318
1.4311877079733	1.8867780184713
1.4314192082416	1.8869870319945
1.4315737240643	1.8885286002731
1.4317514891398	1.8886824388953
1.4319745791391	1.8888592986924
1.4321796781828	1.8890622772425
1.432394188808	1.889072740736
1.4325899705157	1.8891142053395
1.4327612288812	1.8892767095526
1.4329557878896	1.88933276309
1.4331663058211	1.8899249817917
1.4333682268521	1.8899925937482
1.4336296826519	1.8904749742249
1.4338382930537	1.891045932428
1.4340135682007	1.891167801132
1.4342354595191	1.8915297227638
1.4344118114735	1.8917042717931
1.4346584587402	1.8918079730794
1.4348814948471	1.8921257891656
1.435065885946	1.8922396833604

1.4352722204559	1.8923219832988
1.4354927626568	1.8925699594292
1.4356813713585	1.8926096821424
1.4358807279212	1.8927440584595
1.4361360557811	1.8930444372146
1.4363408858932	1.8935115936988
1.4365876655651	1.8936084006659
1.4368246672737	1.89362778753
1.4370303063517	1.8942360989155
1.4372384833731	1.8946464977111
1.4374427099062	1.8959568050961
1.4376938076324	1.8960375419323
1.4379480073409	1.8968774912324
1.4381338815829	1.8968811606263
1.4383720904168	1.8973146811643
1.4385542127679	1.8974455047513
1.4387440575265	1.8977034557136
1.4389571490661	1.8978111601957
1.4392125042333	1.8978771312488
1.4394264048525	1.8984330747385
1.4396461727423	1.8984780490642
1.4398569967542	1.8986930396856
1.4400710852084	1.8987337891977
1.440323119986	1.8994574490734
1.4405217828297	1.9000606999862
1.4407125990738	1.9003703969613
1.4409334031283	1.9004816677599
1.4411443500463	1.9008548623063
1.4413811388463	1.900884183087
1.4415870500459	1.9012243400654
1.441763055258	1.9015267736299
1.4419585715237	1.9015623375115
1.4421815335339	1.9016194398734
1.4424225787601	1.9016513150749
1.4426279679721	1.901767413685
1.4428401227208	1.9029364785786
1.4430790796046	1.9029800311102
1.4433221185167	1.9030586985163
1.4435470211195	1.9030863575044
1.4437643647805	1.9031486726675
1.4439849724841	1.9049115714686
1.4441911675447	1.9051946252939
1.4443724833029	1.9053055144391
1.444598308782	1.9058587970075
1.4448254596441	1.9061480811656
1.4450260491379	1.906785569995
1.4452041447592	1.9068480642712
1.4454191731475	1.9079578065213
1.4456441522305	1.9080027506238
1.4458636367174	1.9083675971214
1.4460713672529	1.9084634888708
1.4462623444772	1.9084892932983
1.4464130464418	1.9085329936436
1.4466001665995	1.9089666172889

1.4468363435642	1.9091493075119
1.4470695812642	1.9097648210355
1.4472553236273	1.9100518274609
1.4474916953628	1.910532848394
1.4477849073268	1.9105643758451
1.4479907016052	1.9108768914913
1.4482192036031	1.9109015112689
1.448443606204	1.9114934465796
1.4486473315497	1.9121618462122
1.4488768678781	1.9124857465766
1.4490967537418	1.9130495327656
1.4493334719372	1.9135089147752
1.4495015229245	1.9136159089531
1.4497009182136	1.9138090433444
1.4499369585901	1.9139807967758
1.4501479564733	1.9142797196526
1.4503420041585	1.9142906692288
1.4505524594704	1.9151262514783
1.4507446158359	1.9154638570128
1.4509609938388	1.9158454637154
1.4511674395547	1.9164255408852
1.4513601787095	1.9183198913581
1.4515778462644	1.9190009436058
1.4518524376804	1.9190028266356
1.4520231439186	1.9190112688856
1.4522432247756	1.919209787098
1.4524722732635	1.9192434178575
1.4526910838161	1.9197829165679
1.4529358577606	1.9198929472546
1.4531735547001	1.9205029735039
1.4534129905998	1.9212860402333
1.4536151920452	1.9214798294055
1.4538274450101	1.9216951109528
1.4540184766437	1.9217606882025
1.4542265932228	1.9224394332702
1.4544576066603	1.9226197957583
1.4547368963102	1.9228354752412
1.4549588908152	1.9229886259435
1.4552562789344	1.9234527452287
1.4554672325431	1.9239126185297
1.4557181018056	1.924267488896
1.4559653452592	1.9243795398957
1.4562230437738	1.9245982710481
1.4564189814475	1.9247868419124
1.45662151119	1.9252220378489
1.4568150748465	1.9253144266488
1.4570419709874	1.9255970315775
1.4572724303696	1.9255994255779
1.4575113909584	1.9257579716657
1.4576977958124	1.927046016686
1.4579449257183	1.9276333702162
1.4581240805992	1.9279374369042
1.4583584977459	1.9290225623713
1.4585497730759	1.9290348854486

1.4587582358615	1.9302891479624
1.458981702104	1.9310833265544
1.4591931534197	1.9328428300748
1.4594245473153	1.9337994604709
1.4596104276771	1.9344039594206
1.4598596392022	1.934605870652
1.4600869129253	1.9348910736038
1.4603215908566	1.9351819866156
1.4605373043455	1.9353331288224
1.4607737571836	1.9364344562885
1.4609939642609	1.9367545699647
1.4612052105693	1.9372671676181
1.4614505682358	1.9372712436281
1.4616349239403	1.9374948665019
1.4618551494847	1.9375708487543
1.4620547081254	1.9380196519915
1.4622591991558	1.9381494144456
1.4624771352538	1.9381629195028
1.4627098178375	1.9388918871186
1.462955528536	1.9404090865392
1.4631805031252	1.9411716769352
1.4634072678259	1.9414515559887
1.4636496431778	1.9420924217563
1.4638688029203	1.9422259781287
1.4641126151218	1.9430480130746
1.4643323264269	1.9436656405389
1.4645473261223	1.9439211908805
1.4647704274345	1.9441591414237
1.4649977556535	1.9442700077935
1.4652020505443	1.9448667312479
1.4654197886731	1.9456909532937
1.4656710337628	1.9458675960491
1.465945180324	1.9470906321824
1.4661567168807	1.9471578390266
1.4663890771454	1.9476511550768
1.4665993818016	1.948845782766
1.4668037574224	1.9489776032786
1.4670472323906	1.9493844528798
1.4672248285533	1.9499773397466
1.4674424462157	1.950024805977
1.4676542434882	1.9500552570053
1.467845785537	1.9505353704963
1.4681100461022	1.9511621646322
1.4683075030947	1.9512537043245
1.4685276935486	1.9525773078448
1.4687408174028	1.952707961236
1.4690129835112	1.9529652555623
1.4692936491088	1.9532028830626
1.4695255766235	1.9532905708041
1.4697172783481	1.9540585822301
1.469887848829	1.9546908640637
1.4700658994244	1.9551420145741
1.4703020124686	1.9557773242398
1.4705229043653	1.9557836266242

1.4707485632677	1.955946384743
1.4709627719949	1.9559843254335
1.471181369902	1.956506860643
1.4714049410916	1.9567815898584
1.4716572211532	1.956921295583
1.4718717815597	1.9572858664727
1.472087020421	1.9577583135246
1.4723124955902	1.9579480295345
1.4725360647632	1.9580309392729
1.4727672108265	1.9587335142369
1.4729934214301	1.9590927164361
1.4732487087523	1.959245621717
1.4734905128212	1.9592973198722
1.4737156686825	1.9596324094126
1.473919591426	1.9598336448541
1.4741324446483	1.9601579262088
1.4744209441764	1.9606074926313
1.4746641614471	1.9606703766857
1.474913736199	1.960919764039
1.4751215708552	1.9611623535264
1.475360856027	1.9612002296349
1.4755844554035	1.9615233255574
1.4757853418084	1.9620576440421
1.4759922849002	1.9621314307004
1.4761943928411	1.9621773333104
1.476430713422	1.9623762622125
1.4766702329409	1.9631059583885
1.4768868664349	1.9636665155254
1.4771450817654	1.9639405905655
1.4773625107309	1.9647304294995
1.4775786059437	1.9649830225733
1.4778143655843	1.9655400191516
1.478042504466	1.9656833500988
1.4782356107386	1.9661398114529
1.4785029015154	1.9662041938003
1.478763742734	1.9667087351503
1.478936853847	1.966830227988
1.4791826147195	1.9668395606879
1.4793716080841	1.9671073618364
1.4796233772784	1.967202766502
1.4799039031287	1.9685015318109
1.4801388083985	1.9687692120826
1.4803601177778	1.9690267488597
1.4805468249739	1.9690959872451
1.480767813728	1.969831329163
1.481007118954	1.9702343665135
1.4812283076538	1.970266604178
1.4814894853152	1.9703727676158
1.4816981678898	1.9706274617695
1.4819415695175	1.9708446332514
1.4821344681843	1.9712276399478
1.4823573415676	1.9712805342123
1.4825301461006	1.971290696761
1.4827820661387	1.9717358302265

1.4830006375884	1.9723012183381
1.483280764328	1.9729406424911
1.4835426438067	1.9731268171696
1.4837837270915	1.9739668025536
1.484030746641	1.9741472656997
1.4842396033737	1.9744220748687
1.4844581436084	1.9751877548776
1.4847379570887	1.9751922504744
1.4849146707329	1.9752977070489
1.4851930836803	1.9757107550192
1.485426142054	1.975829597657
1.485677295577	1.9758381606239
1.4858955638383	1.9758699860725
1.4861242311472	1.9765541745087
1.4863579317384	1.9769782679933
1.486591384377	1.9770105450216
1.4868015665192	1.9775960836182
1.4870380250705	1.9779913684716
1.487292139293	1.9783919342717
1.4875355054659	1.9790776741062
1.487764811868	1.979853340729
1.4879887835983	1.9801720426099
1.4882249128525	1.9804228411109
1.4884556441714	1.9807451733753
1.4887013911891	1.9810205455906
1.4888935422237	1.9817792565847
1.489091336039	1.9821140705606
1.4893154266825	1.9826011551514
1.4895689026882	1.9829510780611
1.4898352019096	1.9830434079463
1.4900698973756	1.9830805957646
1.4902835082406	1.9833884091259
1.4905302490691	1.98359462578
1.4908283237926	1.9838860699437
1.4910734213561	1.9844084415812
1.4912812715315	1.9846826331471
1.4914944650356	1.9849068682104
1.4917348113457	1.986079782855
1.4919471821524	1.9862659115042
1.4921959301541	1.9869494449862
1.4924242782547	1.9875891386868
1.4926562782662	1.9876915004731
1.4929059875788	1.9877839940674
1.4931582402027	1.988090961061
1.4933726459982	1.9881303086138
1.4935802597435	1.9885290788658
1.4938100044216	1.9885558884728
1.4940531660859	1.9887554571335
1.4943270291044	1.9888326494941
1.4945575250915	1.9892737389993
1.4947822799427	1.9907245702313
1.4950223107559	1.9908398640343
1.4952531058634	1.9911583186784
1.4954996561154	1.99138805164

1.4957334850456	1.9915506838555
1.4959712213364	1.9916027464435
1.4961763076416	1.9917309805793
1.496431701709	1.9922095892088
1.4966653777327	1.9941233881093
1.4969120081993	1.9942224865936
1.497166366996	1.9942957335255
1.4974366863217	1.9958133458299
1.4976938915867	1.996994005965
1.4979341179807	1.9971287611701
1.4981720936641	1.9972490941988
1.4984071293876	1.9977813901927
1.4986211818233	1.9978122695733
1.4988581450018	1.9983348132872
1.4991191707155	1.9984714301687
1.4993308726809	1.9985973293573
1.4995308491365	1.9989887631001
1.4997395171615	1.9994519237026
1.4999939324354	2.0003321108831
1.5002355496574	2.0009786110363
1.5004787494403	2.001051681935
1.5007424481296	2.0014725406842
1.500968191982	2.0019620709658
1.5012052775501	2.002570350153
1.5013955522044	2.0033581482553
1.5015874917123	2.0037898030557
1.5018177680564	2.0042039488485
1.5020268525311	2.0042143881463
1.5022777784878	2.0045499146307
1.502527815738	2.0048652291254
1.5027729780225	2.0052905846023
1.5029878374466	2.0054005989863
1.5032147672465	2.0066999617917
1.5034860933819	2.0067967424776
1.5037300341714	2.0068733631728
1.5039524288606	2.0082707924619
1.5041877195033	2.0093778221159
1.5044207402432	2.0097806902295
1.5046350399435	2.0098664130203
1.5048772930723	2.0101040035239
1.5050725406023	2.0103517037371
1.5053293730424	2.0112882914394
1.5055440527099	2.0123949610133
1.50577800099	2.0124325342511
1.5060174960667	2.0125770081616
1.5062575614092	2.012931158884
1.50645435753	2.0131196778155
1.5066897711519	2.0147567640564
1.5068828822047	2.0148306054338
1.5071191444856	2.0154482176172
1.5074011188981	2.0155590208018
1.5075988026013	2.0155929403493
1.5078357952996	2.0157368679792
1.5080461292916	2.0161715178786

1.5082856075954	2.0162402692462
1.50851359679	2.0163357412257
1.5087356974585	2.0165009613987
1.508987325702	2.0166146271645
1.5092690817658	2.0170422582166
1.5095538661074	2.0181792508804
1.50974670237	2.0181822105603
1.5099857237151	2.0190729924763
1.5101718923085	2.0194936944872
1.510504281353	2.0197417703923
1.5107424259078	2.0200604799272
1.5109725345682	2.0201964637749
1.511223500476	2.0205039363344
1.5114648695817	2.0214210385461
1.5116959953931	2.0215701420239
1.5119475052957	2.0216682296412
1.5121361729353	2.0217379421173
1.5124128483705	2.0227210333576
1.5126623834589	2.0236047193858
1.5128700660411	2.0236049752571
1.5130885032093	2.0236549061982
1.5133306819878	2.0240469427249
1.5136253009318	2.024591761357
1.513888016	2.0246763023308
1.5141129250046	2.0250711750183
1.5143486257108	2.0253932825957
1.5145848459655	2.025396431156
1.5148333308701	2.0256598502449
1.5150769003666	2.0261935632011
1.5152914746334	2.0261992439447
1.5155915542466	2.0264779505955
1.5158373830379	2.0270032771628
1.5160168753475	2.0275144669715
1.5162510730782	2.0279416480174
1.516496195093	2.0280696034017
1.5167141519007	2.028563191246
1.5169658658157	2.0300770061527
1.5171714248175	2.030228416322
1.5173920546832	2.0302605883475
1.5176403037856	2.0307727741557
1.5178741838084	2.0311325697037
1.5181193094672	2.0313680707427
1.5183356113616	2.031563425176
1.5185405602304	2.0319368284573
1.5188094537671	2.0322629673349
1.5190475107644	2.0330523163966
1.5192680977971	2.0330805234437
1.5195125408018	2.0330827191883
1.5197834586239	2.0345339348632
1.5200158062268	2.0354491688939
1.5202326718458	2.0359513830192
1.5205156879548	2.0365111938934
1.5207742570881	2.0369956899757
1.5209836071415	2.0370440601055

1.5212159578978	2.0371180148929
1.5215088679196	2.0372312591603
1.5217631724178	2.0376626273012
1.5220138618509	2.0377900997946
1.5222912767836	2.0378610101939
1.5225968818841	2.0379289819427
1.5228222000322	2.0381854933423
1.5230389909025	2.0399683948543
1.5232587997576	2.0402190791651
1.5235051979722	2.0404339911038
1.5237447001716	2.04107608543
1.5239811466224	2.0411399418673
1.5241996140983	2.0420655567229
1.5244636816975	2.0424466716638
1.5246939203329	2.0425146319682
1.5249263710784	2.0427310204802
1.5251628900684	2.0427520551311
1.5254543203157	2.0433181219902
1.5257460100387	2.0434619675621
1.5260046663632	2.0444684520451
1.5262626103819	2.0445000730593
1.5265068278788	2.0447319463
1.5267746737578	2.0452053800362
1.5270060707005	2.0459819811202
1.5272405828303	2.0472094912404
1.5275070685983	2.0472760259512
1.5277527656243	2.0474049656285
1.5279840515579	2.0477936177824
1.5282659297027	2.0499828091821
1.5285272024562	2.0502359841006
1.5287699427246	2.0512680058779
1.5289772615345	2.0513221006892
1.5292204855004	2.0517966454585
1.5294455676849	2.0527486902132
1.5296972844458	2.0528359345249
1.5299673035169	2.0532777479435
1.5302442477616	2.0533290164094
1.5305009440925	2.053755280766
1.5307488977866	2.0541849951549
1.5310185665416	2.0542211861693
1.5312495391908	2.0549434774021
1.5315078312461	2.0551246038716
1.5317276067215	2.0552495327668
1.5319904580287	2.0552888170462
1.5322022204084	2.0554248759493
1.5324575036443	2.0557924781736
1.5327379360186	2.0570974397701
1.5330150673984	2.057280209895
1.5332351108094	2.0577074588242
1.5334571937946	2.0577975750133
1.5336972106769	2.0578640822644
1.5339316578623	2.0578913271333
1.5341942218356	2.0583013717228
1.5344069079626	2.0584337255009

1.5346142469663	2.0587824515414
1.5348594701346	2.0588107171148
1.5351569079797	2.0593067639764
1.5354043206855	2.0594537034675
1.5356498679023	2.059697104755
1.5359255500273	2.0597775705826
1.5361728205484	2.0599535564781
1.5364018872623	2.0599605173051
1.5366954090215	2.0606095719954
1.5369666154283	2.0607344056067
1.5372086535122	2.0613685920114
1.5374400430927	2.0624932363403
1.5376754322587	2.0631224178382
1.5379267127538	2.0632456855835
1.5381326278248	2.0642745910713
1.5383758769089	2.0643482327978
1.5386417135202	2.0645151011817
1.5388993936092	2.0648730355531
1.5391472824382	2.0656919098964
1.5394133143812	2.0663287669519
1.539657629902	2.0677693768107
1.539927799763	2.0690130622875
1.5401419996401	2.0692079283485
1.5404424489606	2.0697110592651
1.5406723720683	2.0698487385381
1.540937760265	2.0700635781215
1.5412215527389	2.0702451630905
1.5414940886792	2.0704112633517
1.5417601228586	2.0711833327111
1.5419835240003	2.0714110927558
1.5422436198261	2.0722272977469
1.5425502042203	2.0735473258932
1.542789774944	2.0739508266245
1.5430562751615	2.0743608203683
1.5433208306545	2.0760155843249
1.5435840077097	2.0760684190753
1.5438544270866	2.0761645341653
1.5440998319516	2.0762171197974
1.5443726473364	2.0770980363295
1.5445745684656	2.0771280646545
1.5448736380525	2.0778175589071
1.5450997408348	2.0778661459865
1.5453410010383	2.0782056304497
1.5456374526412	2.0798053461445
1.5458532198902	2.0817510036353
1.5460691386694	2.08235304275
1.5463325267827	2.0829134317168
1.54654246317	2.0839421693321
1.5467908575803	2.0841505057794
1.5470445631586	2.0843602624669
1.5473115950547	2.0853391792689
1.5475509205329	2.0854639080501
1.5478455256946	2.086360627927
1.5480840175136	2.0863821675642

1.548344302992	2.0865536731455
1.5485541681261	2.0869629673369
1.5488072709822	2.087074542734
1.5491216438621	2.0892698968553
1.5493386461668	2.0926279945966
1.5495735250775	2.0926404923818
1.5498448805399	2.0927682149235
1.5501216317107	2.0930828661124
1.5504251741162	2.0931424298013
1.5506916910412	2.093566664753
1.5509469829861	2.0935700020088
1.5512349275943	2.0937879542923
1.5515636905795	2.0950589388498
1.5518281444072	2.0953586206847
1.5520926846164	2.0962730229328
1.5523459945444	2.097206731966
1.5525480216685	2.0980875367012
1.5528708861594	2.0984131945656
1.5531415436682	2.0989108715425
1.5533585198826	2.098938088909
1.5536258335654	2.0991409092706
1.553904010076	2.0992426624394
1.5541585058873	2.0996184046779
1.5543577583682	2.100663252902
1.5545923116047	2.1022200691592
1.5548829004026	2.1026554134322
1.5551266634162	2.1027439542549
1.5553859104558	2.1028155752759
1.5556662097472	2.1028836076277
1.5559959282109	2.1030148129945
1.5561929916444	2.1031881550032
1.5565087516558	2.1037692418999
1.5567714647352	2.1043474277877
1.5570258777067	2.1045087677418
1.5573175429883	2.1045128976349
1.5575684595583	2.1046842033552
1.5578456613976	2.1049718605906
1.5581150753804	2.1050117259907
1.5583485789581	2.1072174578535
1.5585910279518	2.1073520926436
1.5588587658746	2.1075555736838
1.5591393852712	2.1084441037853
1.5593739090601	2.1085066868346
1.559647730409	2.1096734988111
1.559901357802	2.1098330304549
1.5601312002897	2.1102663062952
1.5604154021306	2.1102803448121
1.5606649439473	2.1111269925813
1.5608706705265	2.1124520452202
1.561085499213	2.1125107555336
1.5613157019796	2.1127865335778
1.5615868988921	2.1127930149175
1.5619136800235	2.1128872969792
1.5621416017806	2.113631912931

1.5624347492041	2.1136666528206
1.5627036118101	2.1141609349283
1.5629889617277	2.1151681811661
1.5632502736779	2.115550428354
1.5635028839273	2.1159023383534
1.5637374861486	2.1159143884098
1.5639625054523	2.1163165858679
1.5641886952213	2.1168570103597
1.5644674349959	2.1171523802987
1.5647442866751	2.1177837283281
1.5649832569224	2.1185964324449
1.5652737408671	2.1193650201722
1.5655092798418	2.1195308861031
1.565749296987	2.1196819267767
1.5660357772375	2.1197858382906
1.566328520768	2.1198891530139
1.5665876652592	2.1200169750782
1.5668531937298	2.1207580666981
1.5670892834489	2.1216978472655
1.567364564909	2.121858205924
1.5676183367498	2.1239443574241
1.5679114093374	2.1240213464495
1.5682143423857	2.1241252171036
1.5684373154302	2.124306593521
1.568738347519	2.1247756760472
1.5689766778338	2.1247840106659
1.5692681614131	2.1247873557255
1.5695481291058	2.1250137255828
1.5698723846332	2.125187985567
1.5701185770483	2.1269172115603
1.5703802773814	2.1279805636664
1.5706778563333	2.1288821559157
1.5709326974363	2.1290080803121
1.5711588147118	2.1299997215662
1.5713925898171	2.1310934477538
1.5716774465243	2.1313429575539
1.5719177065299	2.1317020129191
1.5721935653618	2.1318474190658
1.5724377122862	2.1319928413383
1.5727013754771	2.1329196932124
1.5730025839091	2.1329840935834
1.5732276016386	2.1338225644092
1.5735053575799	2.1338978277661
1.573828258793	2.134286146378
1.57411014614	2.1343937667858
1.5744231853398	2.1353807402648
1.5746866546501	2.1356851134376
1.5748945928609	2.1358257625453
1.5751689896323	2.1367022127706
1.5754338446432	2.136920722828
1.5756279529776	2.1370290206679
1.5759234232669	2.1373641162249
1.5762169543809	2.138982436698
1.5764796690139	2.1392287965788

1.576846653596	2.1392613214865
1.5771202308459	2.1398758759557
1.5773681235081	2.1405510986386
1.5776413469435	2.1407104223029
1.5778954583239	2.1407633168162
1.578142637168	2.1410177054702
1.5784108701189	2.1419348327192
1.5786923893826	2.1421514985202
1.5789468804079	2.1425248011274
1.5791680689729	2.143811935161
1.579434088236	2.1440390051782
1.5797351113614	2.1452579011454
1.5799454171987	2.1476975704534
1.5801775840202	2.1478295646984
1.5804340686598	2.1480707776232
1.5807414386007	2.1485652666549
1.5809955149878	2.1489334167182
1.5812567323015	2.1492062479874
1.5815479551971	2.1492321758443
1.5818252351915	2.1496005290107
1.5820748361472	2.1504944095188
1.5822987446006	2.1517047854567
1.5825831906563	2.1524271348202
1.5828446013765	2.1524342826868
1.5831427961271	2.152574185559
1.5834310405353	2.1528293353245
1.5837025111669	2.1528492501324
1.5839578689592	2.1531708268261
1.584208710439	2.1533881785482
1.5845421006448	2.1535404345163
1.5847440518655	2.1540021716748
1.5850228168945	2.1540039862038
1.5852597367779	2.1545226716607
1.5855166634336	2.1551698641296
1.5857632708992	2.1558386215685
1.5860049359404	2.1563652801991
1.5862666954061	2.1566277306645
1.5865298914073	2.1570054248256
1.5868254918181	2.1575786522165
1.5870660886981	2.1578999467152
1.5873181405123	2.1596693284642
1.587556103957	2.1599276431044
1.5878917162155	2.1602807187375
1.5881653039923	2.1605768627304
1.5884560070404	2.1616954976452
1.5887148810408	2.1619672088827
1.5889507935228	2.1620173254679
1.589208493248	2.1626221758734
1.5895164431127	2.1627782138288
1.5898198817297	2.1629512885097
1.5900827912424	2.1634700305804
1.5904074647321	2.1645148572822
1.5906856789266	2.1667254273476
1.5909684272032	2.1669739162439

1.5912544632114	2.1674430041891
1.5915214840335	2.1676859800666
1.5917608751858	2.1684468204806
1.5919920611695	2.1690420346047
1.5922805646223	2.1690578282031
1.5925594390527	2.1694603892948
1.5928377976374	2.1701590552113
1.5931113297914	2.1712859459839
1.5933398061548	2.1713988731587
1.5935786899928	2.1720924000054
1.5938220218173	2.1727011430543
1.5940459701145	2.173011152412
1.5942798737322	2.1732088280155
1.59451950447	2.1741660728673
1.5947681718025	2.1742488582326
1.595041890952	2.1749702371679
1.595295817262	2.1750191791487
1.5955746252707	2.1752118273325
1.5958769387467	2.1762599182564
1.5961575402422	2.1777377533939
1.5964543359706	2.177814243702
1.5967791327121	2.1778460251365
1.5970158669434	2.1787869952148
1.5972718089437	2.1789158532422
1.5974693345528	2.1803971257726
1.5977790451017	2.1806652929571
1.5980669969523	2.1806709256386
1.598325410269	2.1812297973953
1.5986039672903	2.1814338439006
1.5988778890337	2.1820240052246
1.5991350953266	2.1830460390374
1.5994023691161	2.1838273754544
1.599650535566	2.1841798145416
1.59994053701	2.1844478737506
1.6002272484071	2.1852047495747
1.600575639783	2.1856190435088
1.6008439892237	2.1866884657463
1.6011227433644	2.1879780238088
1.6014218753975	2.1898552296116
1.601697569381	2.190002025079
1.6019757122443	2.1912776843605
1.6022409521142	2.1913818065433
1.6024986272876	2.191411632464
1.6027590166291	2.1919949898763
1.6030390145461	2.1943480793595
1.6033509441374	2.1947004628951
1.6036136381353	2.1952430904459
1.6038618825584	2.1953810655896
1.60412653561	2.1954710665013
1.6043594693583	2.1957097770102
1.6047353646082	2.1961190660495
1.6049871558994	2.1961391201616
1.6052456197737	2.1964755229827
1.6054821558634	2.1965668436116

1.6057248925152	2.1966957726106
1.6060130489142	2.1968680094254
1.6062720605785	2.1973807487374
1.6065131418456	2.1978552079639
1.6067532115082	2.1989192901264
1.6070103521051	2.1993655389792
1.6072960959739	2.1996182496679
1.6075549757742	2.2000690424845
1.6078603044602	2.2010928693892
1.6081022535577	2.2014426223196
1.6083884238885	2.2016609694513
1.6086614595747	2.2016694301945
1.6090088518803	2.202184521738
1.6093012639607	2.2024152795869
1.6096293980671	2.2025120014772
1.6099064500245	2.2026222921857
1.6101907440391	2.2028298288989
1.6104443738073	2.2028341405568
1.6107434119835	2.2031287614047
1.6110406816864	2.2040254538192
1.6113313019407	2.2041363444855
1.6115562950676	2.2048456283097
1.6118467219625	2.2049999914763
1.6120963853116	2.2058219389551
1.6123406195592	2.2064391407032
1.6126565237133	2.2075672523414
1.6129256661827	2.2078819712858
1.6132716713262	2.2084988577415
1.6135855173797	2.208746661197
1.6138462660386	2.2087864441505
1.6141290725323	2.2107455319164
1.6144427368817	2.2107955645005
1.6147754496146	2.212846687033
1.6150219289022	2.2130175985728
1.6153020279266	2.2135413532934
1.6156332565167	2.2136199030689
1.6159098605807	2.2138046693628
1.6162165766158	2.2141812606113
1.6165197723869	2.2150677131067
1.6167774400029	2.2156973434874
1.6170735926001	2.2158157115279
1.6173154724567	2.2164480343887
1.6175363318664	2.2178186445493
1.6178298006593	2.2178371857024
1.6181081286964	2.2194913714815
1.6183604748233	2.2197274795098
1.618625027479	2.2209617638274
1.6189046318772	2.221139870372
1.6192045541419	2.2219394440338
1.6194678241484	2.2239915009808
1.6197486867784	2.2242752100949
1.6200358121083	2.2244837005626
1.6203128195343	2.2246959254403
1.6206061806033	2.2250858143748

1.6209046085487	2.2251706659001
1.621233420385	2.2253024213297
1.6215322674908	2.2261587620968
1.621838383161	2.2261615215895
1.6221132127598	2.2271042585738
1.6224409365193	2.2274801727312
1.6227757561025	2.2278056686601
1.6230651843403	2.2281454425991
1.6233766098642	2.2281587401695
1.6236525680672	2.228321405463
1.6239743430612	2.2300561565872
1.6242677268327	2.2302765627813
1.6245625468845	2.2304584125663
1.6249038017003	2.2304584360309
1.6251685594718	2.2308966005443
1.6254177252884	2.2310331990985
1.6257096149071	2.2314213795047
1.6259501544618	2.2317177851485
1.6262439292765	2.2330126245811
1.6265546780552	2.2336755542006
1.6269082143045	2.2339588409091
1.6271808817438	2.2341427533934
1.6274851925619	2.2344186005383
1.6278172956945	2.2364003486608
1.6281437489534	2.2375221366045
1.6284337345344	2.2395029977629
1.6287224637281	2.2403367630329
1.6290500179239	2.2409049347282
1.6293576913829	2.2414748403223
1.6296952011028	2.2439967703465
1.629995754509	2.2442885334782
1.6303287410476	2.244851928467
1.6306208258425	2.2449676942527
1.6309381924339	2.2450092622606
1.6312627428435	2.2453301439772
1.631490952894	2.2463241000275
1.6318618180153	2.2463244391927
1.632181736243	2.2464973473471
1.6324990340612	2.2467997089339
1.6327847173983	2.2468956515329
1.6330785974645	2.2476102025647
1.6333934288487	2.2480726879338
1.6336884138542	2.2480936039707
1.634014616503	2.2481799112526
1.6342840525174	2.2482181457513
1.6345930787634	2.2486313513646
1.6348551170865	2.2488237142332
1.6351391300022	2.2491447632054
1.6354437088522	2.2495475637714
1.6357412752212	2.2507859096035
1.6360408220431	2.2530813556507
1.636323501401	2.2531602765159
1.6365979337681	2.2540507049962
1.6368769777959	2.2550145409771

1.6371670136989	2.2550299405725
1.6374538544971	2.2557524444913
1.6377595610037	2.2561341084963
1.6380360362845	2.256646347262
1.6383191935399	2.2576983810363
1.6386192326742	2.2579065095718
1.6389409184988	2.2580664128635
1.6392918199722	2.258229504555
1.6395823049739	2.2586889661825
1.6399240948972	2.2591496840623
1.6402016428191	2.2591974030701
1.6404670286436	2.2612271459579
1.6408052669949	2.2612882389028
1.641091787825	2.2613102075362
1.6413722185085	2.262683374198
1.6416311284013	2.2627827067536
1.6419407040566	2.262825205075
1.6423000740693	2.2630543047038
1.6426447624405	2.263856228915
1.6429103872553	2.2642654080313
1.64319169817	2.2645279595454
1.6435002483621	2.2658645518573
1.6437734157115	2.2659963987642
1.6440439072558	2.2660946900665
1.6443515362918	2.2662651234812
1.6446316835742	2.2671993405412
1.6449483707656	2.2686719418145
1.6452989681543	2.2688767498257
1.6456210710103	2.2693448976409
1.6459346649204	2.2695206654719
1.6462589169505	2.2700108926485
1.6465269282975	2.270309807853
1.6468136362816	2.2706665669487
1.6471181466354	2.2707711279851
1.6474169648124	2.2708037656753
1.6477065266983	2.2715881054377
1.6480327426261	2.2723884140496
1.6482946424419	2.2727737662918
1.6485815935346	2.2728149723762
1.6488794646711	2.2732514871625
1.6492345759294	2.274163216776
1.6495444715949	2.2742267451056
1.6498217214609	2.2755670887049
1.6501167467779	2.2774231386991
1.6504152850264	2.2776465151413
1.6507425987578	2.2785155387038
1.6510424449358	2.2785511501202
1.6513234416057	2.2792295977653
1.6516702672008	2.2793275735409
1.6519341946145	2.2802529181995
1.6522503096791	2.2807591708383
1.6525354662036	2.2815201456606
1.6528230076194	2.281525491259
1.6531199620477	2.2827906612464

1.6534121810006	2.2839998401499
1.6537217044961	2.2840961535747
1.6541152151839	2.2847162068001
1.6544082516365	2.2848901104831
1.6547408836726	2.2856028062068
1.6550850122979	2.2856903322682
1.6554012065826	2.285981671884
1.65574535886	2.2862745708103
1.6560712060653	2.2876771970388
1.6563716277489	2.2885203958543
1.6567190142913	2.288681725689
1.6570101855255	2.2892632155551
1.6573235301337	2.2906939542777
1.6576082761724	2.291073423741
1.6579389373992	2.2915582163151
1.6581945642681	2.2917564929177
1.6585202320504	2.2917952102207
1.6587969363292	2.2918797551659
1.659108673498	2.2921486781927
1.6594102799652	2.2926780499591
1.6597006526836	2.2940087285715
1.6600337343745	2.2943746128623
1.6603166405936	2.2948201023245
1.6605908587403	2.2951300515028
1.6609135588476	2.2953243328099
1.6612383449218	2.2956139995227
1.6615213417636	2.2961814317586
1.6617874519374	2.2965359063851
1.6620630157526	2.2967431888218
1.6623363636599	2.2972689097115
1.6626149810702	2.2977789296181
1.6629209598648	2.2984944862349
1.6632103046359	2.2986983928782
1.6635322617147	2.3002445310103
1.663828213322	2.3002868043984
1.6641232033098	2.3004644038313
1.6643859500402	2.3013012724315
1.6647155676438	2.3013521505033
1.6650189884723	2.3020893798011
1.6653089940409	2.3024131699378
1.6656180499788	2.3025740997934
1.6659088656137	2.3034784643542
1.6662093141121	2.3035655955633
1.6665422614947	2.3046479435077
1.6668964742187	2.3049017764645
1.6672019168413	2.3053390995532
1.6674525902516	2.3053789485756
1.6677323711744	2.3056425040474
1.668050300048	2.3057279920088
1.6683308636203	2.3064752204044
1.6686311958724	2.3075730070037
1.6689682435276	2.3076394699912
1.6692822214206	2.3082844396923
1.6695388903888	2.3093824807399

1.6698597188096	2.3096297272057
1.6701883258833	2.3101415658032
1.6705082593059	2.3104519837894
1.6708314911123	2.3107701641235
1.6711294098544	2.3117625664732
1.671438401114	2.3131488885412
1.6717437819982	2.3132733127739
1.6720421243419	2.3133771670832
1.6723263344873	2.3134337821911
1.672624136858	2.3134910133319
1.6729197659382	2.3138836054174
1.6732698812685	2.3149310749843
1.6736087207424	2.3151722376335
1.6739156255892	2.3157815867757
1.6742352409831	2.3177035269957
1.6746076826598	2.3183898013323
1.6748990588857	2.3187165857826
1.6752222275251	2.3195955911972
1.6755323179177	2.3208272141846
1.6757947031382	2.3225675498737
1.6761694012938	2.3232826903691
1.6765082558374	2.3236799854913
1.6768703851765	2.3244400313282
1.6772215603387	2.3253833485517
1.6775424796607	2.3254090541428
1.6778259215603	2.3254581162112
1.6781364859102	2.3259854589513
1.6784671185102	2.3260104473974
1.6788109163172	2.3260835858759
1.6790676687396	2.3261486250672
1.6794195237967	2.326886384107
1.6796992282743	2.3274822163383
1.6800337929179	2.3278035815128
1.6803901572824	2.3280926711556
1.680715619586	2.3283575150507
1.6810409409725	2.3284202069326
1.6814084031258	2.3286603226052
1.6816875959329	2.3286672320495
1.6820458969216	2.3289239754534
1.6823802716328	2.3305351307313
1.6827521089454	2.3317463016188
1.6830734377964	2.332527021612
1.6833471697194	2.3326114647506
1.6836184769273	2.3338052657329
1.6839429265128	2.3341580631539
1.684202433877	2.334297770168
1.6845139552856	2.3350960457865
1.6848297502939	2.335275928548
1.685124142669	2.3355573771113
1.6854808137005	2.3362007953301
1.6857816060475	2.3364586628027
1.6861117815988	2.337829775457
1.6864288495525	2.3383146132506
1.6867306267241	2.3385043621325

1.6870152691635	2.3391152722581
1.687344738376	2.3400482821264
1.6876754090812	2.3406696620429
1.6880195636256	2.3410360429217
1.6883654451571	2.3415074527564
1.6887240282412	2.34211717577
1.6890654510684	2.3426231488795
1.6893852362588	2.3426776508631
1.6896895630019	2.3449376429028
1.6900833897744	2.3449516058795
1.6904182471119	2.3450825482785
1.6907112414467	2.3468064441624
1.6909779306639	2.3468547676565
1.6912838941771	2.3469208768959
1.6915675587156	2.3473720485209
1.691875825719	2.3492478359976
1.6922105976793	2.3501819906482
1.6925855170627	2.3502398913904
1.6929010349746	2.3502939241533
1.6931831507664	2.3505970043944
1.693547746475	2.351300527803
1.693929905326	2.3513619392117
1.6942111687057	2.3528353868692
1.6944880812005	2.3529672135382
1.6947962037168	2.3535601194178
1.6951378411347	2.353670334229
1.695406798121	2.3553285257088
1.6957220598308	2.3576176018539
1.6960203435717	2.3581676132186
1.696323071208	2.3587694712562
1.696600568862	2.3588814160484
1.6969543729379	2.3589768472974
1.6972899814861	2.3597556618992
1.6976237273377	2.3599420461445
1.6979164158544	2.3622537251433
1.698279515928	2.3630545688563
1.6986017308973	2.3641039362515
1.6989333544944	2.3650361248678
1.699267951236	2.3664559209423
1.6996007686083	2.3678413573819
1.6999492773997	2.3680474594951
1.700284369773	2.3691431074788
1.7005987136006	2.3697276280295
1.7009037325483	2.3705928925761
1.7012982385818	2.3713831659975
1.7016199347366	2.3723158599413
1.7019450975625	2.3744972089648
1.7023173894832	2.3745343620887
1.702643368302	2.3746927083642
1.7029353260187	2.3752926822974
1.7032417960385	2.375890926443
1.7035747974728	2.3761456691232
1.7038862334767	2.3809953293175
1.704268003014	2.381111803733

1.7046314810364	2.3816342177539
1.7049402064031	2.3823190896503
1.705280329158	2.3823773903181
1.7056375023237	2.3837268983533
1.7059559792964	2.3847522144082
1.7062411125549	2.3849043273409
1.7065600320448	2.3854065259721
1.7068590450908	2.3857907634391
1.7072102408991	2.3866315728106
1.7075101677043	2.3872431817967
1.7079005402712	2.3873804511693
1.708254038361	2.3874568426202
1.7085549610882	2.3882830978641
1.708886121371	2.3890937731894
1.7091901805166	2.3918451606846
1.7095577968132	2.3923018620597
1.7099060336703	2.3924400779775
1.7102662453107	2.3927459522371
1.7106279738406	2.3931963056996
1.7109003730509	2.3952115367571
1.7112663080311	2.3956844568864
1.7115878466956	2.3960386010663
1.7119408681787	2.3963311510905
1.7122592875015	2.3986727598042
1.7125931414471	2.3989909426583
1.71295892461	2.399266154977
1.7132644744229	2.4012295812727
1.7135829816779	2.4022018190305
1.7139087101731	2.4034727527948
1.7142901555522	2.4041223471774
1.7146291671188	2.4043701316509
1.7149744073502	2.4044532320011
1.7153016712392	2.4048926670078
1.7156441652443	2.4049649712629
1.716010403892	2.4053985270693
1.7163431879041	2.4056949165609
1.7167249232725	2.4059732534404
1.7170755267332	2.4062699693231
1.7174618957127	2.4072700069479
1.7178311956626	2.4080973356658
1.7181697907404	2.4089635486699
1.7185196375347	2.4096348569468
1.7188797313173	2.4099865546717
1.7192048750962	2.4103967082486
1.7195709161224	2.4111589547319
1.719957444679	2.4113079450269
1.720285531879	2.4117806677159
1.7206714646473	2.4119540072104
1.7210104934744	2.4124821685181
1.7213017443303	2.4144408121192
1.721633742526	2.4151761526148
1.721902065231	2.4152681707564
1.7222839760791	2.4154653259994
1.7226566035866	2.4154860532234

1.7230397323414	2.4165447034908
1.7233457431035	2.4171628346967
1.7236283917678	2.417374129771
1.7239557567737	2.4195179872608
1.7243465518987	2.4196829085189
1.7247606182662	2.4199258465517
1.7250926801974	2.4199300794363
1.7254319597538	2.4207204439065
1.7257634297158	2.4219296355579
1.7260900716158	2.4231166771478
1.726396996008	2.423659344978
1.7266301165821	2.4245143741773
1.7269631218005	2.4251621541716
1.7273070997049	2.425215376266
1.7276820956958	2.4253032716166
1.7279846578053	2.4274992631137
1.7283572114876	2.4276354012474
1.7286947310336	2.4276904285209
1.729159352122	2.4281963372724
1.7295084036583	2.4287713793017
1.7298419907614	2.4292273296627
1.730217552378	2.4294668828552
1.7305529187084	2.4298198617885
1.7308711302307	2.4300872437715
1.731197880122	2.4305437009761
1.7315328819893	2.4308976339209
1.7318538193773	2.4311513590322
1.7321434773333	2.4323615559307
1.7324792453845	2.4328914782686
1.732825726172	2.4332209660152
1.7331763109411	2.4346503250331
1.7335192606909	2.4352679269528
1.7338326178195	2.4355880149184
1.7341498636759	2.4367058101467
1.7344950331063	2.4371623895945
1.7348854045298	2.4373846223552
1.7351241259933	2.4384886318553
1.735420687987	2.440152932472
1.7357339051643	2.4414457125374
1.7360901497651	2.4419742752189
1.7363853644742	2.4421636313414
1.736741343607	2.4424280582388
1.7371541883333	2.4424929771442
1.7375950808365	2.4426817744891
1.737998008897	2.4432075085423
1.738291611699	2.4435265461187
1.7386398121554	2.4435999011995
1.7390280077489	2.4449700022781
1.7393901789732	2.4455198164297
1.7397494358059	2.4465654693197
1.740122489951	2.4468833867478
1.7404592034873	2.4478702090946
1.7407955429962	2.4481335330581
1.7411210476081	2.4489015168105

1.741411370584	2.4489700825181
1.7418261943552	2.4489920496089
1.7421660763522	2.4492088859996
1.742548369793	2.4498746293442
1.7429852452366	2.4503278363485
1.7433794514747	2.4523188320169
1.7436574081501	2.4542455219397
1.7440077759041	2.4547841655307
1.7442803646895	2.4550982807822
1.7447015107601	2.4552906956343
1.7450604750809	2.4558433081019
1.7454537298771	2.4559618662889
1.7457163963157	2.4561329470821
1.7460300935765	2.4562145425697
1.7464307951688	2.4572213758207
1.7467910591681	2.4576356231388
1.747100778766	2.4577749122785
1.7474587107123	2.4589344264886
1.747793877588	2.4590558984609
1.7481559219095	2.4598673019401
1.7485232040932	2.4605570230948
1.7488789683715	2.4609474686887
1.7492088613158	2.4612683379327
1.7495049703235	2.4613020112265
1.7498190701831	2.4614021094018
1.7502210822138	2.4615503576821
1.7505168749891	2.4617558435872
1.7508037260072	2.4622594524347
1.7511187135298	2.4633560067757
1.7515038608166	2.4644561474593
1.7518195334942	2.4647032287843
1.7522282907433	2.4659842573776
1.7525659913963	2.4663784731364
1.752886911238	2.4665847265791
1.753279091364	2.4665923793488
1.753616116226	2.46675740141
1.7539850552176	2.4668039635972
1.754348618941	2.4671178810855
1.7547469683084	2.4676885857115
1.7550533486517	2.468171526287
1.755434243447	2.4689284534684
1.7558493767113	2.4692886810775
1.7562292938436	2.4698979375463
1.7565089840252	2.470069162532
1.7568213034457	2.4704552990438
1.7571441167747	2.4706916839913
1.7574700531906	2.4711622476935
1.7578462269091	2.4723694040885
1.7581784277866	2.4731234932069
1.7586404364841	2.4733036279258
1.7590024769955	2.4733610398589
1.7593238119515	2.4738478240063
1.7597634785212	2.4745441191651
1.7600683295754	2.4745742609706

1.7604074149001	2.475253591419
1.7607697625156	2.4756036278577
1.7611483622862	2.4761199947517
1.761524723611	2.4763371199033
1.7619605255413	2.4777615658492
1.7623509257902	2.4777883045069
1.762666702841	2.4787404526983
1.7630715725833	2.4801642507204
1.7634644064746	2.4824819978905
1.7638463161724	2.4839546379129
1.7641885502299	2.4845441944245
1.7646325424886	2.4847247418386
1.7649969617055	2.486549499922
1.7653539787504	2.4869762835658
1.7657187436873	2.4875866570312
1.7660699191317	2.4886501780979
1.7664428146096	2.4908411470247
1.7668199012024	2.4924199171435
1.7671971355526	2.4927580195218
1.7675791050068	2.4927809085389
1.767953839704	2.4950529600424
1.7682926565172	2.4957840854926
1.7686323769999	2.4958365229936
1.7689533038245	2.4966502531083
1.76933318196	2.496835021456
1.7696355523727	2.496900800089
1.7699496458336	2.4975103444631
1.7703193330146	2.5005282792069
1.770617639689	2.5008761850355
1.7710459932233	2.5013347497639
1.7714439304289	2.5028661654829
1.7718295836511	2.5032079057208
1.7722245793111	2.5039216281432
1.7725411777335	2.5047344498931
1.7729545718367	2.5067561131051
1.773294804648	2.5069144702425
1.7736732679048	2.5070431063041
1.7740530754092	2.5073587870927
1.7744272351677	2.5080074434584
1.7748973801	2.5084205750498
1.7752557451312	2.50908194419
1.7756895428219	2.509571665889
1.7761259423356	2.5097022755473
1.7764743078923	2.5104146259363
1.7768847474078	2.5106668164305
1.7772500416325	2.5107178696661
1.777641710456	2.5119650722707
1.7780568466075	2.5121669911979
1.7783929154959	2.512213347056
1.7788128679633	2.5122336332369
1.7793303529114	2.5128741520591
1.7796925596999	2.5131351360448
1.7800491634847	2.5133126468662
1.7804444329415	2.5141501533501

1.780770748833	2.514182395353
1.7811342515338	2.5160935619421
1.7814017808406	2.5163223426482
1.7818109762289	2.5166058227256
1.7821448763335	2.5168548306532
1.7824999097067	2.5168822186057
1.7828485789732	2.5173782854006
1.7833258176921	2.5180003743201
1.7837617034554	2.5187898633545
1.7841696466064	2.519423057637
1.7845284040552	2.5195085422656
1.7849553524587	2.5200873968211
1.7853609051647	2.5201910189049
1.7857499395252	2.5224707456192
1.78609250315	2.5233689140906
1.7865611710615	2.5244057432361
1.7869391833976	2.5252238846762
1.7872925374984	2.5254392706229
1.7877099981072	2.5268956771986
1.7880697569634	2.5270924125915
1.7884658554085	2.5276637202115
1.7888274734168	2.5277136339713
1.7892068488069	2.5299590455586
1.7896109865081	2.531505669261
1.7900074248491	2.5317543631417
1.790400689934	2.5318707529951
1.7907461258772	2.5328675918045
1.7911564390151	2.5344356063841
1.7915061726412	2.5348113422727
1.7918888356763	2.5363855739649
1.7923311374823	2.5369736320113
1.7927323992504	2.5370056451463
1.7930732856292	2.5373603628124
1.7934532379736	2.5375828058776
1.7938186628958	2.5375977122659
1.7942002723232	2.5385583514912
1.7945600234862	2.5392704602646
1.7949325336817	2.5427324316473
1.7953602245489	2.5427350753061
1.7956964363135	2.5434575531788
1.7960761377324	2.5439218116111
1.7964311598195	2.5451790821012
1.7968139287964	2.545961437991
1.7971899148509	2.5462575529724
1.7976054254392	2.5475365929048
1.7980898235365	2.5476403450984
1.7984439583704	2.5481544330315
1.7987831501779	2.5491123523413
1.7992112903855	2.5492751656725
1.7995216798633	2.5496826940467
1.7998868882383	2.549952236483
1.8002343309378	2.5501945935249
1.8005834211262	2.5502000394451
1.8009527430669	2.5510548158137

1.801341362737	2.5518217493325
1.8018529491589	2.5524369416709
1.8022372300564	2.5527505502993
1.8025429902884	2.5529943365399
1.8029580155523	2.5530369216287
1.8033606637135	2.5538620767528
1.8037331954022	2.5539582818357
1.8040259666435	2.5555775783816
1.8043558136886	2.5564222976905
1.8047932138763	2.5565931623525
1.8051909861728	2.5575725187972
1.8055373065304	2.5584254026464
1.805876721169	2.5598064801001
1.8062694825602	2.5598911124528
1.806616372593	2.561362484927
1.8069765845852	2.5627195400821
1.8074176286651	2.5638214499472
1.8077770242292	2.5657798228328
1.8081353252046	2.566669664596
1.8085417793683	2.5671965962133
1.8089718058886	2.5673696329607
1.8093936942911	2.567902447054
1.8098552511604	2.5682389621821
1.8102665910119	2.5703624847788
1.8106919515933	2.5707139312322
1.8111513077066	2.5709085618597
1.8115859658727	2.5752370019943
1.8120235763945	2.5753979781084
1.8124781723948	2.5765386013733
1.8129230143598	2.5794975810151
1.8133703593877	2.5808580876008
1.8137699306737	2.5824725573306
1.8142059599599	2.5828418227779
1.8146190603768	2.5837936043995
1.8150021537352	2.5838098332329
1.8154440295584	2.5848525273199
1.8158606978658	2.5856937590242
1.8162360505706	2.5858994277059
1.816627817146	2.5864247641257
1.8171077707571	2.5868557976918
1.817477896433	2.5870067206677
1.817837768204	2.5871114377147
1.818288983561	2.5876649740917
1.8186567771624	2.5876853271934
1.8190517391323	2.5889802880205
1.8194369106104	2.5894590348753
1.8198327602188	2.5898615450518
1.8202727419985	2.5909261207126
1.8207325135359	2.5929307986374
1.8211272070356	2.5935288242909
1.8215231623925	2.5948596010776
1.8219017948861	2.5948959216112
1.8222317759164	2.5954913769543
1.8226708414775	2.5956012499592

1.823070489812	2.595686278778
1.8234535394759	2.5966629342473
1.8239313608466	2.5972493095058
1.8243237128561	2.5977142191956
1.8247265786471	2.5986006932195
1.8251433314078	2.5990462184037
1.825572226895	2.6000796619345
1.8260021479999	2.6002681869555
1.8263440268296	2.6011112675623
1.8267791024518	2.6012943770292
1.8271837644477	2.6064631508924
1.8275983656772	2.6068069664473
1.828019283875	2.6068104138868
1.8285255622277	2.6078483277862
1.8288781624483	2.6079767944648
1.8292121183528	2.6080533987051
1.8295983334417	2.6100736096913
1.8299746947865	2.6107349921861
1.8303549875102	2.6107543552011
1.8307478427281	2.6108155711734
1.8311696201882	2.6109811035523
1.8316064909981	2.6114468435091
1.83197488539	2.6120810320753
1.8323150585861	2.6146343889162
1.8327944605809	2.6161537537957
1.8331778795592	2.6175958055026
1.8335715043994	2.6178904154791
1.8339839290262	2.617952462258
1.8343692163449	2.6184196108886
1.8347440919698	2.6191348443199
1.8351516872575	2.6197538511813
1.8356238134765	2.6199236850906
1.8360475160316	2.6201997033983
1.8364802131497	2.6202079176642
1.8369798334227	2.621533157575
1.8374141819478	2.6228562495672
1.8378340516026	2.6238017427879
1.838325471099	2.6242543728315
1.8388050526557	2.6267853328281
1.8391815234889	2.6271455405293
1.8396409548145	2.6297191444182
1.8400784123171	2.6300150998764
1.8404998423562	2.6302686308234
1.8409344352148	2.6308995100736
1.8413046432569	2.6312062764156
1.8416858679496	2.6317258568953
1.842065090965	2.632049571557
1.8424699349379	2.6321409431237
1.8428502535962	2.6343808428185
1.843211676215	2.6347992203629
1.8435656066832	2.6351680338165
1.8440668048194	2.6355535086632
1.8445298254756	2.6362462960438
1.8449560255743	2.6370017993056

1.8453852093669	2.6375130367771
1.8457749377793	2.6386532310693
1.8461424016701	2.6387023251542
1.846512452488	2.6387424008427
1.8468819285525	2.6397697580799
1.8472720348544	2.6397734950488
1.8477177778875	2.6429249072215
1.8481632963624	2.6431035700518
1.8486084047474	2.6444408279234
1.8489792316239	2.6451107583118
1.8493730685429	2.647188549478
1.8497036260951	2.6481079592024
1.8500966811124	2.6482733236473
1.8505184671653	2.6485972776737
1.8509498659757	2.649228714826
1.8513852505853	2.650593455007
1.8517799848552	2.6519948672793
1.8522144850692	2.6520352472228
1.8526117606893	2.6521668647318
1.8530248794678	2.6525254506945
1.8534398772559	2.6532251131645
1.8539178673635	2.6547635247404
1.8544082668152	2.6548756426691
1.8548835192293	2.6553476641933
1.8552913816446	2.6554451609438
1.8557023108065	2.6557152249465
1.8561574277843	2.656074172057
1.8565757704438	2.6565606999318
1.8569350727041	2.6571518360348
1.8573573462447	2.6580802836003
1.8577452792489	2.6590644329601
1.858037664146	2.659785876519
1.8584498313837	2.6599198839639
1.8588085342909	2.6630437074949
1.8592422610541	2.6630955496305
1.859695460577	2.6657783767416
1.8601452121217	2.6666330518833
1.8605646747538	2.6674184463451
1.861015846203	2.6690244155115
1.8614997028696	2.6714860758318
1.8619164788528	2.6731306045021
1.8623221400058	2.6731575925518
1.862725066105	2.6733546459829
1.8632028079559	2.6737075924334
1.8636321513689	2.6739288855258
1.8641291700742	2.6741374187672
1.8645206530504	2.6747845124767
1.86486255101	2.6752656291457
1.8653465174842	2.6757203559352
1.8657377852179	2.6766435303198
1.8662006989526	2.6773235153851
1.8667088111051	2.6776788063421
1.8671172380026	2.6788785210424
1.8675560238767	2.6789525395756

1.868046353067	2.6814867312325
1.8684665563628	2.6828747153825
1.8689120516756	2.6836643192898
1.8693388328877	2.6846304944278
1.8697420518134	2.6862447780347
1.8701323147926	2.687638919019
1.8706032516581	2.6878582466915
1.870989511504	2.6900587573113
1.8713482061139	2.6901791476415
1.8718462004224	2.6904169251505
1.8723023985264	2.6910048467883
1.8728199347129	2.6910951087388
1.8732864118737	2.691710495238
1.8737217640195	2.6926306893227
1.8741893083951	2.6929244319436
1.8745777548527	2.693099954382
1.8749884671065	2.6980500789143
1.8753988116019	2.6981504170285
1.875814354976	2.6985368445358
1.8763085177253	2.6985797750388
1.8767848877776	2.6986117292561
1.8772690415638	2.6996028958246
1.8777335770347	2.7006464330503
1.8781880113488	2.7009638382579
1.8785823506631	2.7009960562074
1.8790007904214	2.7021149878482
1.8794789800825	2.7021899585646
1.879970476382	2.7034936241419
1.8804958445634	2.7040643957044
1.8809202240156	2.7043098288568
1.8813469222058	2.7046623595345
1.8817434336123	2.7053514582749
1.8821567862369	2.7070707329117
1.8825652255832	2.7071435634809
1.8829592856429	2.7073752388107
1.8833725322968	2.707456011674
1.8837527253408	2.7075445203052
1.8842097197119	2.7080324653101
1.8846118412792	2.7081785509077
1.8850480611605	2.7095338571387
1.885537885044	2.7100193598313
1.8859761229584	2.7105307253529
1.8863940674778	2.7119112146514
1.8869112446118	2.7125216796307
1.8873686767635	2.7135623202356
1.8878434750019	2.7158529911008
1.8883328553119	2.7162412500149
1.8888148885575	2.7183754519654
1.8892636148315	2.7184691725923
1.889662201906	2.7189246096473
1.8901233903859	2.7213514688791
1.8905199315169	2.7223455179966
1.8908902603305	2.7224221996122
1.8913372219949	2.7230533481938

1.8917437244281	2.7237931637737
1.892150708332	2.7241727643818
1.8926003401613	2.7246271938171
1.8929636098123	2.7249714630961
1.893425562766	2.7259312276434
1.8938410248432	2.7270230312696
1.8942929104583	2.7279955991469
1.8947119554373	2.7282308714888
1.8952062385869	2.7299898627179
1.8956359650398	2.7307272614569
1.8961063342828	2.734073035731
1.8965252020362	2.7348431037309
1.8969579719755	2.7360423874215
1.8973846567009	2.7385177515024
1.8978925552917	2.739402306794
1.8983541456512	2.7397895751744
1.8987505348885	2.7404047821086
1.8991256790311	2.7415394851187
1.8995377525819	2.7415944886791
1.8999861245938	2.7426067064522
1.9004018570166	2.7450717004015
1.900828635854	2.7466673543444
1.901284090628	2.7472707493997
1.9017794515202	2.7475412081687
1.9022538317815	2.7483025489357
1.9027196685099	2.7488101471716
1.9031410092709	2.7488462637682
1.9036029913216	2.750116420127
1.9041247864438	2.7513326615935
1.9045606329467	2.7514906245959
1.9050831744365	2.7514947576877
1.9056313048211	2.7521612136873
1.90613055146	2.7537028685922
1.9065507119188	2.7540940869274
1.9070277964129	2.7541362488262
1.9073960892318	2.7542906074855
1.9078467949593	2.7550310348069
1.9083402104672	2.7563966350496
1.9087609365364	2.7567085836679
1.9091771913799	2.7598579984959
1.9096961886041	2.7605713157015
1.9101165392139	2.76279341147
1.9105478364294	2.7651039402552
1.9109739703672	2.7652505277304
1.9114580175471	2.766467270309
1.9119381241176	2.7665797009636
1.9123348294801	2.7671306233594
1.9127484588314	2.7675806852141
1.9132602338652	2.7677797024152
1.9136828028182	2.7695909727916
1.9141496121815	2.7699541943356
1.9147414554679	2.7711076433337
1.9151562257086	2.7728333338671
1.9155812451706	2.7735851718832

1.9161158033426	2.7740378646859
1.9166294126254	2.7742182450247
1.9171007339963	2.7759442546266
1.9175616485825	2.7771687893345
1.9180180510341	2.7783964119637
1.9185611815191	2.7788125298339
1.9190369100689	2.7809469007168
1.9196765993165	2.7825206780486
1.920229359855	2.7839604325035
1.9207515389631	2.7862196921022
1.9212571050603	2.7865500268858
1.9217937728534	2.7870746456441
1.9222507892333	2.7874358212643
1.9227878743867	2.7875045690481
1.9233496561272	2.7875943245025
1.9238037691237	2.7890684702165
1.9243434742245	2.7896956353877
1.9248152201434	2.7900936394709
1.9253518525853	2.790502559679
1.9258536807466	2.7924606612042
1.926313954986	2.7927634799312
1.9267846838424	2.7929615926049
1.9272900774037	2.7929922321535
1.9277439563993	2.7937482683182
1.9282245188736	2.7950845375332
1.9286770578842	2.7962866170708
1.9291522517168	2.7975876698773
1.9296743541132	2.7992481950895
1.930203690143	2.8000532283521
1.9307200765752	2.8010248182019
1.931209008711	2.8018143734435
1.9316021791687	2.8019092359362
1.9320467048767	2.80302031963
1.9326153930942	2.8042643707074
1.9331226106873	2.8047576154329
1.9336070544359	2.8068217529497
1.9339627438506	2.80776196666
1.9345268545534	2.8104788637508
1.9349764241112	2.8104937113953
1.9354784639034	2.8106415058091
1.9360013069463	2.8119377924194
1.9364917668743	2.8128203273841
1.9368806554379	2.8129814895681
1.9374296955441	2.8132840348276
1.9378622850503	2.8135442419325
1.9383087194261	2.8142206073948
1.9387461663502	2.8189947518782
1.9391771801824	2.8194269786457
1.9397504291489	2.8206600577639
1.9402887320054	2.8209755967287
1.9408731883882	2.8213773133545
1.9413823024389	2.8220894898724
1.9418811257177	2.825183930366
1.9423000461261	2.8257825826377

1.9428605385766	2.8313103817497
1.9433755936596	2.8314222551532
1.9438283205933	2.8341075379204
1.9443517463031	2.8348448538161
1.9448661854331	2.8348927356839
1.9453670023686	2.8356354513909
1.9458439549855	2.8360925066344
1.9463388830584	2.8362883524103
1.9467403380868	2.8371622217078
1.9471605772751	2.8374293170367
1.9476445474467	2.8376907156194
1.9481014619823	2.8392651310713
1.948695091835	2.8400791134738
1.9491802896994	2.8405703270537
1.9498355694291	2.8406642155472
1.9503553618652	2.8411977444456
1.9509558483292	2.8412093040742
1.9513578338139	2.8419564104105
1.9518907612502	2.8424609400663
1.9524842710923	2.8433859204587
1.953066491256	2.8436465223041
1.9535937047446	2.8441244207715
1.9540688189359	2.8475768690582
1.9544890099343	2.8479126963459
1.9549788656361	2.8481057968808
1.9555778371623	2.8500562562966
1.9561453762311	2.8532529956648
1.9566258180022	2.8539428786511
1.9570874846802	2.8572784859482
1.9575764063016	2.857630807667
1.9581661619551	2.8582118384047
1.9586801632948	2.8582297980154
1.9593217558103	2.8585150946177
1.9597751255592	2.8600334218829
1.9602433057192	2.8604460035182
1.960837860263	2.8607051507713
1.9613415966273	2.8610294307229
1.9618838299283	2.861219644347
1.9623646193043	2.861544840202
1.9628080673649	2.8624939106309
1.9633795246249	2.8699550479886
1.9639057513578	2.8708748540165
1.9644806640172	2.8717881364518
1.9649293727171	2.8722493594148
1.9655101393538	2.8739046324135
1.9660513866044	2.8739274139701
1.9665961320432	2.8739921281627
1.9670835483635	2.874949080858
1.967605352022	2.8750181461574
1.9681134461104	2.8751389916895
1.9686154986264	2.8755226687507
1.9690290220458	2.8755239188689
1.9694582828191	2.8763981569972
1.9699674508078	2.8769555129732

1.9704420981914	2.877057561875
1.9710077732199	2.8776615752121
1.9715404675401	2.8791173057955
1.9720954115928	2.8808970481242
1.9725622302585	2.8827636206719
1.973106487996	2.8841575322051
1.9736223762115	2.8844523525231
1.9741284117853	2.8858733279896
1.9746063244145	2.8877548195379
1.9751618931078	2.8891010366374
1.9757743846885	2.8961496865771
1.9763253429477	2.8979198521049
1.9769422143564	2.8982972280098
1.9776444809227	2.8986146106729
1.9781742676689	2.8990612096671
1.9786944366603	2.8991102146462
1.9791402936133	2.8998205515675
1.9797776089333	2.9001319171671
1.9802485990523	2.9028206467432
1.9807757507151	2.9029710725089
1.9813420499088	2.9061542270009
1.9819313876907	2.9063151642982
1.982485303329	2.9092460101258
1.9830533527765	2.9104374550206
1.9836079790593	2.911070172057
1.9841445699901	2.9144586719552
1.9846184323745	2.9157866364215
1.9851507409105	2.917081545144
1.985640775149	2.9173673022224
1.9861171015891	2.9177726711016
1.986604308079	2.9181112895589
1.9871643939364	2.9186455009272
1.9877303609841	2.9191499792358
1.9881871425163	2.919274889587
1.9887304464152	2.9196985152612
1.9893795230175	2.9210305516871
1.9899036571132	2.9211406393622
1.9904619563443	2.9216222063511
1.990993436207	2.9219370272728
1.9913977255969	2.9224358377003
1.9919683123555	2.9261710096254
1.9925099866713	2.9272163374706
1.9931503468033	2.929544642943
1.9936830457866	2.9297555348899
1.9941760771739	2.9297994992436
1.9946476789872	2.9299270649828
1.995216376452	2.9300978250331
1.9958318865987	2.9307305999651
1.9962934888096	2.9314437162035
1.9968304897801	2.9351670805686
1.9973944912119	2.9359913702378
1.9979059645234	2.936227429449
1.9984906547422	2.9370769599689
1.9989738340348	2.9397321760357

1.9995730568367	2.9410344370795
1.9999797401034	2.9434374089679
2.0005367929567	2.9436525765435
2.0010122820047	2.9449795392852
2.0016609120545	2.9452636828174
2.0021256815726	2.9456911092827
2.0026367252357	2.9458594641072
2.0031907214639	2.9463941275527
2.003740720733	2.94650960253
2.0043767217498	2.9472629101627
2.0049147933802	2.9483608709566
2.0055396404524	2.9492357668195
2.0061294647247	2.9493617763554
2.006656506552	2.9509705824688
2.0070976782671	2.9511470647842
2.0076195413881	2.9523176535622
2.0081633050134	2.9523392929642
2.0086489626479	2.9539568337329
2.0091623291244	2.9553689008217
2.0097604671301	2.9562053158338
2.0102366390077	2.9566758529985
2.0108139895738	2.9570633414672
2.0113974979518	2.9592019721604
2.0119895169101	2.9596064941246
2.0124633075668	2.9597390827964
2.0128676070824	2.9600865179123
2.0134744560606	2.9628306438584
2.0140026642669	2.9637090557381
2.0145828901172	2.9674876160199
2.015132796073	2.9676195399546
2.0157019643708	2.9676613215496
2.0162825848576	2.9690149902153
2.0168587119851	2.9709877957201
2.017361786069	2.9724549547633
2.0179636526759	2.9747918602184
2.0186033099124	2.9750120685873
2.0191534158146	2.9764029682921
2.0196879114434	2.9764219111327
2.0202684238018	2.9765009315923
2.0208468406916	2.9766586827901
2.021405287954	2.9768825462757
2.0221385708831	2.9772844384104
2.0227575628745	2.9792126357407
2.0232842897144	2.9801201379562
2.0238372326451	2.9806759909811
2.0243272820004	2.9825758151138
2.0249261849809	2.9829017818998
2.0255572707758	2.9841087411395
2.0260602199633	2.9862130832566
2.0267297283261	2.986377911105
2.027301088374	2.9864327015047
2.0279619987854	2.9864950229226
2.0284850875165	2.9883272563043
2.0291892770471	2.988453912723

2.0298302619573	2.9907506519217
2.0303728389075	2.9908683560378
2.0308752422562	2.9920098651304
2.0314933335128	2.9924878129209
2.0320856806885	2.9932293450219
2.0326914136382	2.9932860726269
2.0332336088653	2.9941549324187
2.0338681826358	2.9949987486996
2.0344596811766	2.9953662201535
2.035191842302	2.9960147896532
2.035703250672	2.9987900437314
2.0363357039789	2.9991723715153
2.0369298197261	3.0003077272661
2.0374825628155	3.0007732580945
2.0380710863551	3.0021378014188
2.0387184420451	3.0028915866859
2.0392835713366	3.0046227228797
2.0398339113487	3.0046517221297
2.0403897277109	3.0060086084964
2.0409324130742	3.0078602167713
2.0415616717966	3.0079109585936
2.0421403437815	3.0083903363802
2.0426624850581	3.0093255072647
2.0432733932334	3.0097661580392
2.0437854008984	3.0120149796464
2.0445044480628	3.0128190667258
2.0450540599806	3.0145489250249
2.045588127035	3.0147735005625
2.046089569745	3.0195501955539
2.0465580347705	3.0199047051126
2.0471759669761	3.0202934406462
2.0477937075656	3.0223018000663
2.0483910309194	3.0235892893511
2.0489171905422	3.0240397494539
2.0494990832868	3.0241576303372
2.0500383836228	3.0243439816112
2.050576210283	3.0262600194187
2.0511720153704	3.0274478806848
2.0518602441788	3.0275755211153
2.0524557262507	3.027668250142
2.0532028203618	3.0294724492649
2.0539055989355	3.0297812750149
2.0545388200534	3.0313446436439
2.0551363369009	3.0316605222906
2.0557314486213	3.0321628966506
2.056336368653	3.0322515006869
2.0569384918761	3.0337770899161
2.0575195551003	3.0345148012905
2.0580462363998	3.0347362841792
2.0586476504403	3.0386269785271
2.05926914378	3.0407630336638
2.0598701069064	3.0407793204074
2.0606045833348	3.0420320832589
2.0610827606335	3.0420793179216

2.0616904833226	3.0427304200922
2.062352414457	3.0466018933925
2.0629194495638	3.0467388217835
2.063481769341	3.046878815475
2.0640898297457	3.0481142511986
2.0646900698675	3.0492597315965
2.0651849042228	3.0509963100424
2.0659381897594	3.0522221578905
2.0665814919888	3.0529691331619
2.0671581034811	3.0536250743216
2.0676903411172	3.0549745600602
2.0682163887003	3.0557067562156
2.0688914579156	3.0559723642182
2.0694415463629	3.0572046105058
2.0700213411852	3.057885772227
2.0707100673073	3.0618826219629
2.0713808618595	3.0660788138108
2.0720152954489	3.066758366566
2.0726448283689	3.0675723717191
2.0732567230742	3.0708832787867
2.0738194441937	3.0711627535911
2.0744149787231	3.0721883992251
2.0750864939878	3.0722167120378
2.0756830171826	3.0722556308949
2.0762858737515	3.0729019806479
2.0769454329929	3.0737808891689
2.0776096461182	3.0739390576362
2.0781410572067	3.0741054194713
2.078814489506	3.0753128777825
2.079397635154	3.0756838486628
2.0799722997445	3.0811482749245
2.0805737195289	3.0816381089098
2.0813435405577	3.0830727106265
2.0819461433719	3.0857670418874
2.0824794636658	3.0863853672728
2.0831593440594	3.0875424456691
2.0837932135919	3.087605191492
2.0844432521671	3.0878567332509
2.0852113565865	3.0900705445427
2.0857710280725	3.0927421147408
2.0863865160649	3.0938360096983
2.0870024396017	3.0947845159469
2.0877322828902	3.0948532037302
2.0883157517798	3.0951724236895
2.088982987246	3.0962482114433
2.0896583449181	3.0962674515242
2.0902830232553	3.0983295748074
2.0909672934732	3.0984461175196
2.0915580058336	3.0984534117064
2.0923751232798	3.0998481839405
2.0930014459513	3.1003605855638
2.0936533933229	3.100564695282
2.0943565769491	3.1020224868089
2.0950198695038	3.1022619320896

2.0955703638742	3.1041111262876
2.09616398966	3.1056886319672
2.0967616743278	3.1071321783318
2.0975362492144	3.1073026761576
2.0982388904642	3.1097465723749
2.0989279799462	3.1100228826889
2.099480699753	3.1106511121753
2.1003179289224	3.1126338467675
2.1008928116538	3.1162337908035
2.1015320049676	3.1167804535691
2.1021706353018	3.1170325345742
2.1026745463564	3.1186280094223
2.1032793869142	3.1201729194284
2.1039727194465	3.122123251679
2.1046324954207	3.1228875736705
2.1050562951244	3.123105230575
2.1057580419917	3.1245952154913
2.1062987910993	3.1247212941115
2.1069172375862	3.1255650971425
2.1075725369629	3.1264454509565
2.1081679087572	3.1285617353377
2.1087885088034	3.1287327908062
2.1094344933111	3.1292163838193
2.110048037311	3.1293497442734
2.1105984951028	3.1298112568301
2.1112568169342	3.1304545671803
2.1119240494709	3.1308714381353
2.1126182726255	3.1323784460921
2.1133482332317	3.1341970298792
2.1139575552149	3.1359769587138
2.114710550746	3.1367388878997
2.1153254541888	3.1406743340807
2.1158752081329	3.1417559722477
2.1165454578794	3.1418775413088
2.1172035736884	3.142717883932
2.1178231925098	3.1437805705657
2.118470946512	3.1453451495455
2.1192023878667	3.1462551098157
2.1198436076324	3.1463512283519
2.1204827899215	3.1508099834303
2.1211754823164	3.1522689488371
2.1218696777047	3.1545260959774
2.12255998496	3.1561378720184
2.1233372681878	3.1608035971251
2.1240043044868	3.1624447535361
2.1247131769619	3.1640720157501
2.1254127257064	3.1705546670958
2.1260165813744	3.1727937815021
2.1267732844099	3.1738903258502
2.1275751658452	3.1743358060605
2.1282626428596	3.1753508630024
2.1288558715561	3.1789536663516
2.1296484030131	3.179224578425
2.1304010316677	3.1803834856495

2.1311174194204	3.1833056716386
2.1318508419055	3.1843372944065
2.132607288695	3.1851978232151
2.1333613359489	3.1865945434064
2.1340509964833	3.1867939150543
2.1348155203586	3.1875899663115
2.1355352550559	3.1887662811632
2.136094025508	3.1913866130762
2.13693223263	3.191972039335
2.1376568675164	3.1957409000287
2.1385286151681	3.1966192327572
2.1391300938115	3.1973658843505
2.1397809523162	3.197390130799
2.1404653790516	3.1974898152797
2.1411934217015	3.197591719312
2.1418531421353	3.198435722948
2.1425278422631	3.1988969638216
2.1430887359832	3.1995275852148
2.1437298100746	3.2005595959509
2.144489175791	3.2023443240222
2.1453168509312	3.2051014650024
2.1459233090527	3.2056246179269
2.1466769841695	3.205816992564
2.1473904124677	3.2077550877578
2.1481913538235	3.2146569469635
2.1489343729439	3.2147365990894
2.1495721772201	3.2150017919902
2.1502529043532	3.215946561826
2.1508986044039	3.2173870558466
2.1516769831891	3.2174708520828
2.1522527606561	3.2180241150635
2.1530266624813	3.2215595717056
2.1536919495128	3.224328011104
2.1544461654065	3.224549205956
2.1550422397427	3.226895125843
2.155692776833	3.2276711360461
2.1564021114503	3.2303794548883
2.157305004238	3.2327160383907
2.1580046586015	3.2343161283924
2.1587207102654	3.2413303362646
2.1593744034491	3.2414790945366
2.1601317396355	3.2425059339825
2.1610028864642	3.2425963224947
2.1616625587151	3.2435339375203
2.1623381195474	3.2474125166662
2.1631750993442	3.2474248740199
2.1638463074126	3.2489084913936
2.1645952613522	3.2499388397161
2.1653046456441	3.2508351698802
2.166004072833	3.252383727182
2.1668530856169	3.253050555362
2.1676115676014	3.2552427740874
2.1684520408818	3.256576114109
2.1691187308676	3.2577572886322

2.1699151803573	3.2578758234361
2.1706448923871	3.2581376610315
2.1713244929873	3.2582013404902
2.171995359703	3.2586710565918
2.1728006672924	3.2597302185665
2.1735041818729	3.2598800833732
2.1742620881807	3.2626606988458
2.1749652554923	3.2655179655296
2.1757420971827	3.267282247284
2.176452068533	3.268419595513
2.177385629874	3.2694648446079
2.178238253981	3.2699356941689
2.1790489213505	3.2710741577976
2.1797717278799	3.2711065069754
2.1806882144299	3.2744346270237
2.181396114843	3.2767886497126
2.1822101254207	3.2773331909134
2.1831307723724	3.2786687353628
2.1841141200247	3.2789252983466
2.184809747245	3.2797455106089
2.1855537225137	3.2801872557094
2.1863881435468	3.281021862198
2.1872504197019	3.281484816157
2.1880078280849	3.2818531850324
2.1886944555426	3.282902320912
2.189356980816	3.2844271650681
2.1902356713353	3.2846826104492
2.1909497377683	3.2861687529242
2.1917006361132	3.2876558302089
2.1924706449549	3.2888535920559
2.1933443368249	3.2901874748744
2.1942207268384	3.2911222133826
2.1951262535842	3.2923408352851
2.1959142110084	3.297993733388
2.1966996988746	3.2982095720036
2.1974517604637	3.3027849462555
2.1981837977068	3.3041741070776
2.1988502180569	3.3044096043155
2.1996645909204	3.3045413768832
2.2003815340944	3.3053244723126
2.2011139529419	3.3056479541183
2.201996334293	3.3078994471918
2.2030947893894	3.3085890912762
2.2039869181206	3.3085891381334
2.2047672451659	3.3130893854299
2.205683561408	3.3148553046059
2.2064339641069	3.3149210538408
2.2070824479925	3.3191856017537
2.2077470118316	3.3191859845603
2.2088046991836	3.3204761384271
2.2095223896078	3.3211219465571
2.2104233616793	3.323203070815
2.2111121016735	3.3239219444559
2.2118048811373	3.3247813865322

2.2125034820184	3.3248018563626
2.2132959810256	3.3256674514425
2.2140802360962	3.3267646568641
2.2148076187834	3.3279712207734
2.215604539823	3.3283827742812
2.2163949034551	3.3349883735297
2.2171705889817	3.3352945871896
2.2179790316705	3.3389533849557
2.218913590838	3.3395818217048
2.2197361245418	3.3398098618314
2.2205470450794	3.3421729398881
2.2214021315819	3.3426258065246
2.2223068804046	3.3450474985576
2.2230445103282	3.3456639791402
2.2239095839213	3.3457004281013
2.2246182982012	3.3523155210228
2.2254714374308	3.3591365715814
2.2263969849078	3.3609981010521
2.2270628677216	3.3639252373774
2.2279039244181	3.3648310699197
2.2289094754267	3.366244109884
2.2297712026177	3.3680391208973
2.230592717006	3.3686230554012
2.2314663877608	3.3687147575333
2.2323474733089	3.3688835050094
2.2331809425863	3.3690259737615
2.2340084737945	3.3694834945148
2.2349367451419	3.3709414253862
2.2356011086458	3.3724092845967
2.2364728044757	3.3752900095981
2.2375672922305	3.3758320812573
2.2383666726592	3.3775800585295
2.239200297757	3.3783725832038
2.240171301792	3.3783779292386
2.2410765652388	3.3837141699777
2.2419030271647	3.3860267853282
2.2429299165706	3.3892882991432
2.2437598019063	3.3907172475933
2.2446595786086	3.3911069549944
2.2454339062787	3.3913842847189
2.246385525353	3.3958313091919
2.2474164594775	3.395924290877
2.2482266864447	3.3966226836399
2.2488492389098	3.3991241571851
2.249610297042	3.4015338350618
2.2504170965244	3.4015881212408
2.2512700130624	3.4034449135855
2.2520589126911	3.4035884748786
2.2531095167203	3.4045449665176
2.2539073854926	3.4068188154452
2.254724635851	3.4075655864432
2.255402919146	3.4083586512126
2.2562512762008	3.409711827389
2.257059106514	3.413459820661

2.257816465329	3.4147048198876
2.2586525826485	3.4215998868372
2.2597098156428	3.4226547198121
2.2605232824601	3.4234318527201
2.2614816719066	3.4250528480376
2.2623217470573	3.4259274775528
2.2631002647412	3.42794901457
2.2637268002504	3.4299941043116
2.2645806203594	3.4341615298149
2.2654201542621	3.4365223834078
2.2663050844157	3.4383860030918
2.2672709996629	3.4389508117352
2.2682957237821	3.4422388069146
2.2691959335778	3.4434427686044
2.2700419748139	3.4517371204306
2.2710574911364	3.4522904377033
2.2721442424242	3.4536875532956
2.2730820773621	3.4553416153914
2.2739902006519	3.455366977312
2.2750662476879	3.4567774154049
2.2758936787249	3.4577809831293
2.2768776828217	3.4583645527863
2.2777751735848	3.4605122077643
2.2787289311881	3.4606072196573
2.2796477733624	3.4611215993217
2.2805397528798	3.4650135973741
2.2812750347741	3.4651592203853
2.2821371107059	3.4655665665495
2.2832248145783	3.4699059029719
2.2840218229666	3.4706364802542
2.284885487898	3.4706537637134
2.2857500259865	3.4714079664896
2.2866653325465	3.4746679841731
2.2876420559965	3.4763236456867
2.2886699466691	3.4764454146275
2.289724710538	3.4774426397462
2.2907208581022	3.4779055963655
2.2917839068991	3.4782267422595
2.2926661471399	3.4813803815325
2.293605813397	3.4834271894601
2.2944570655079	3.4857066675559
2.2952631853877	3.4901018187967
2.2963687587137	3.4927484020818
2.2973650227234	3.4975798259003
2.2983135700745	3.5001025570773
2.2993214086874	3.5001630982479
2.3002081229121	3.5014315088263
2.3011697532673	3.5031896021182
2.3022790378907	3.5032050981847
2.303113229846	3.507112340068
2.3039495466298	3.5076403739685
2.3048536461564	3.5083145530403
2.305876211987	3.5104781772317
2.3067309297696	3.5133290440063

2.3076395400275	3.5149217768839
2.3086301686814	3.5162529385453
2.3096775096143	3.5190908289422
2.3106538211913	3.5195880706814
2.311671127462	3.5201769097031
2.3126936796396	3.5218795525191
2.3136157307565	3.523042145833
2.3145443795416	3.5233301416317
2.3154538666494	3.5255812133328
2.3163899081209	3.5265641408048
2.317237725788	3.527191248949
2.3181126475047	3.5286250062015
2.3193605250853	3.5300106227323
2.3203550854398	3.5317139776567
2.3214227910767	3.5336406963285
2.3224159725966	3.5346540778665
2.3236141007197	3.5376519938369
2.3245161047427	3.5397122326769
2.325352555033	3.5401840527562
2.32641343912	3.5417165445486
2.3273459850219	3.5433402577114
2.328218909806	3.5459613527653
2.3291121792609	3.5530127021012
2.3302133527501	3.5530460215043
2.3312285414103	3.5535864235668
2.3323153173656	3.5539322900489
2.3331953722633	3.5542962407526
2.3342659326672	3.5554088982191
2.3350986351949	3.5557142340019
2.3362062603866	3.5603116830391
2.3376246603275	3.5618872356596
2.338639100317	3.561907429351
2.3395345503509	3.5658319212127
2.3405543403172	3.5699523562705
2.3415877015016	3.57135069508
2.3426561143217	3.5720408513153
2.343613132256	3.5728087063912
2.3445221335331	3.5748594305306
2.3456260326746	3.5829951527073
2.3465943931574	3.5842157589988
2.3476403946925	3.5854523724213
2.3487182338736	3.5876421947443
2.3497008830175	3.5880906363795
2.3507452385806	3.5925064129744
2.3517721002653	3.5941256102643
2.3527795807236	3.5963076453397
2.3538303956237	3.5975074897604
2.3549389744421	3.5988925371502
2.3560364805225	3.600305760091
2.356964877468	3.6021618159228
2.3578739147835	3.6031556301412
2.3591785630937	3.6032012653923
2.3600648402471	3.6036379212771
2.361145257438	3.6095209394992

2.3622948960702	3.6101608483927
2.3632854046392	3.6104415754059
2.3644198825486	3.611439124424
2.3655979826641	3.6120619080371
2.3666758700519	3.6132278388336
2.367767138528	3.6138283486225
2.3689252682626	3.6192401120898
2.3699229664073	3.620578567999
2.3709473407321	3.6220251669954
2.3719829802006	3.6220428905287
2.3730205603457	3.6222726939491
2.3742438777627	3.6234613719557
2.3753114653136	3.6240353194331
2.3765984937643	3.6270316312568
2.3777013598523	3.633661144059
2.3789292625125	3.6357450122287
2.379926488531	3.6360781274349
2.3809447208127	3.6378154091352
2.3820443062011	3.638906094382
2.3832133629311	3.6405108856163
2.3843237893828	3.641632348252
2.385681939082	3.643419386163
2.3867080068818	3.6482511679888
2.3879436171432	3.653100466757
2.3890411083929	3.6572522573647
2.3901029847244	3.6577570100418
2.390951638621	3.6577782795463
2.3920916085612	3.6585440019675
2.3931541868046	3.6629349375231
2.3942649972915	3.6737171944582
2.3952584753749	3.6752010088086
2.3964046394567	3.6755930164733
2.3976059514669	3.6774253286982
2.3986707476417	3.6822363255377
2.3996770048452	3.6843802499201
2.4009792387439	3.6861617204744
2.4021746760218	3.6862330066779
2.4033729053173	3.6881743392414
2.4047978574721	3.6915663158263
2.40590684236	3.6916769947186
2.4072099525118	3.6969142880084
2.4083898637883	3.7018570069086
2.4094465919957	3.7041373309101
2.4108887111323	3.7046691670965
2.412105023121	3.7067160522317
2.4131649565167	3.7165009180015
2.4144117720497	3.7174607797909
2.41551665978	3.7194702961997
2.4168300317407	3.720627630103
2.4181642074679	3.7232558658781
2.4193934124588	3.7237512497815
2.4204287741263	3.7247105918521
2.4217035873076	3.7258764688724
2.4228620795501	3.7290322840138

2.423937552189	3.7293679521971
2.42481182429	3.7312611486559
2.4262488771903	3.7326097881911
2.4276941465847	3.7327310190731
2.4289033815091	3.736086189645
2.4297862787383	3.7367253189842
2.4310693706671	3.743584703155
2.4323747202371	3.7493013565504
2.4335748801051	3.7567279611444
2.4347354911581	3.7614380737356
2.4361141857184	3.769130211726
2.437380191943	3.7693398529109
2.4384775811272	3.7712850329443
2.4396940513573	3.7728216055341
2.4409067866267	3.7734983720709
2.4420875422298	3.7772495840939
2.443553536784	3.7780528529316
2.444917347075	3.7789671545813
2.4462845463138	3.7792691653862
2.447765838023	3.7800826029273
2.4490147671169	3.7822843220996
2.4503508271316	3.7891129964656
2.4515156133913	3.7908627123362
2.4528746864828	3.7928587656034
2.4543465382843	3.7939224810499
2.4555747688703	3.7992905962434
2.4568373079242	3.8080318467858
2.4580074507571	3.8122920701626
2.4592668998128	3.8179360368049
2.4603977463096	3.8181923214245
2.4614784716213	3.8185017401842
2.4626469432716	3.8220752115336
2.4639206481879	3.8229480475349
2.4654737941123	3.8238239772628
2.4669229156832	3.8306887648339
2.4682990563938	3.8373600096895
2.4694230117547	3.8401521550373
2.4704011685214	3.8436045889199
2.4718342009068	3.8458181077203
2.4730114082956	3.8460397598909
2.4741960169345	3.8531432708554
2.4758678661836	3.865928468289
2.4771715630027	3.8665017108775
2.4783356701276	3.8693678044839
2.4796517940776	3.8693948397068
2.4809212961901	3.8703005871467
2.4822471781912	3.8703188636464
2.4833892144934	3.8705333790645
2.4848217686787	3.8737108389525
2.4860360401255	3.8832144169197
2.48732714409	3.8834144271754
2.4885324112142	3.8885575173932
2.4899569349532	3.8938142215149
2.4911620397648	3.8940701819308

2.4923146332712	3.8950495492077
2.4936416404589	3.8974630322726
2.4950986777354	3.902288557717
2.4963119426008	3.9081845279793
2.497703657177	3.9087556817246
2.4993560820068	3.9140153374374
2.5009076651254	3.9163428326772
2.5021811315122	3.9169892792436
2.5034066995397	3.9206303493555
2.5050413954566	3.9216479959343
2.5067360163006	3.9224378323619
2.5080534083872	3.9267932243761
2.5094771405847	3.9290546598198
2.5108984902727	3.9321942763786
2.5123714867588	3.934440793112
2.5137203044113	3.9410331124626
2.5152070853695	3.9414424615745
2.5169220603379	3.9457962051567
2.518514031022	3.9460113988407
2.5198115351719	3.9466503842263
2.5214250923528	3.9485122895532
2.5227984139606	3.9503232103137
2.5244064939333	3.9515447153882
2.5259078053917	3.9551247141422
2.527385778683	3.9577393716471
2.5290381458507	3.958649582088
2.5304530525386	3.9611372300321
2.5321111870733	3.9619380484244
2.5338455448236	3.9630281255051
2.5352539235616	3.9653457118403
2.5368032995763	3.9665767512526
2.5383965457557	3.9673399566132
2.5402169589512	3.9687897860093
2.5416421560209	3.9756771814759
2.5430603312715	3.9797537357487
2.5443668548899	3.9833005930872
2.545725415764	3.9875890275579
2.5475139530496	3.9890751217456
2.5490344730377	3.9996163761161
2.5505017043115	4.0029441086487
2.5524781979127	4.0054834820245
2.5539233512378	4.0067489670222
2.5556561512116	4.0079392763448
2.5571552865947	4.0100598896678
2.558776417628	4.0124457598147
2.5602975293681	4.0138277187093
2.5617684098279	4.0157518838705
2.5632187119593	4.0285970573302
2.5648062134923	4.0316563037976
2.5663058849902	4.0367198466318
2.5679015032437	4.0367889859514
2.5697757629974	4.0377460696996
2.5713596221496	4.0391894142698
2.5733191075342	4.0414313159626

2.5748699232462	4.0421662404655
2.5762710202152	4.0432430544923
2.5776231114792	4.0436904607778
2.579513044281	4.0440604048525
2.5811031677636	4.0444257648196
2.5827262258391	4.0474371749811
2.584820705001	4.0569174380978
2.5862947436597	4.0573268780465
2.5878146978611	4.0588807122722
2.5894184442511	4.0632701572876
2.5911167481137	4.0649725934714
2.5928793847861	4.0658842483771
2.5946173249685	4.0663110246451
2.5960052077056	4.0664287849004
2.5975663888335	4.0679106877762
2.5991472786188	4.0683185706072
2.6009747075067	4.0850348397377
2.6030392702707	4.0850821759966
2.6048470451148	4.0895460489698
2.6065139924228	4.0999600426091
2.6082693022522	4.1031630131144
2.6100869109887	4.103221189321
2.6115312370013	4.1035251618725
2.6132620481072	4.1035374140333
2.6152198062485	4.1053306315734
2.6172036948929	4.1062180086774
2.6186858539422	4.1110796312104
2.6204936016045	4.1144721838443
2.6219473800453	4.1146517307383
2.6236556021364	4.1155957460518
2.6254130775964	4.1164811065232
2.6271566237013	4.116789986359
2.6288528402313	4.1249835513792
2.6307360531008	4.1274950792164
2.6323710743579	4.1353068420117
2.6345661714606	4.1364461149045
2.635895133229	4.1385512620447
2.6375137983192	4.13935122673
2.6391861296347	4.1424940107604
2.64085386578	4.1439861334423
2.6425102663265	4.1455051594683
2.6445717898342	4.1461939602783
2.6464394130057	4.1480971365818
2.648545640781	4.1600524388364
2.6502880464512	4.1624024632529
2.6522123929901	4.1633612849904
2.6539233260195	4.1637676669632
2.6556975280403	4.1640576818012
2.6574086003739	4.1650879600627
2.6593996152494	4.1735341191364
2.6610159402362	4.1741966000672
2.6629573218246	4.178039935462
2.6649422009515	4.1815829079734
2.6668635138434	4.1828711821627

2.6687699727324	4.1852581480285
2.670893640626	4.1872260327211
2.6726931215473	4.1911984037673
2.6748412134839	4.1939645651839
2.6767419517522	4.1944521198184
2.6785843207869	4.2044218086405
2.6807817317	4.2136677831196
2.6827770904335	4.2203293928291
2.6847536895409	4.2222934457818
2.6866000445902	4.2231560742833
2.6880991168474	4.2240375205185
2.6900039165959	4.2253463900287
2.6921717825109	4.2357128705874
2.693835884458	4.2387250273652
2.6960986238181	4.2394584605483
2.6984489035928	4.239925018737
2.7003772766859	4.2499875581175
2.7026938468818	4.2500054121075
2.7047818461507	4.2534229195632
2.7066718951121	4.254221289397
2.7089679642439	4.2578664523809
2.7108117206498	4.2588278460534
2.7127608896988	4.2654183675263
2.7149091784445	4.273169649673
2.716886878849	4.2795728907559
2.719076494381	4.28241632921
2.7212005491383	4.2834893820379
2.7232929054576	4.2854409522091
2.7252790576326	4.2928982215782
2.7275602233228	4.2952569325866
2.7296853231466	4.2981707782597
2.7319175374159	4.3029403455221
2.7341037406306	4.3039277760499
2.7364171294726	4.3125719683876
2.7382430020335	4.3258629973605
2.7404548291587	4.3279753926083
2.742188853033	4.3363320211143
2.7445254382666	4.3373132969313
2.7471584890484	4.3553680180862
2.7492618433941	4.3713366127597
2.7513662360875	4.3733630169294
2.7538667799495	4.3863469134986
2.756163813917	4.3965967969109
2.7585911767797	4.3986151643069
2.7608602919393	4.4007409270742
2.7632713896961	4.4034921157245
2.7653705740691	4.409753995451
2.7679339713961	4.4140897163317
2.7702402050261	4.4148591820531
2.772593403552	4.4186264566061
2.7748931073398	4.4214433972441
2.7776024992378	4.4235014845664
2.7799511011649	4.4251570466289
2.7822666868105	4.4264414845799

2.7845424724219	4.4267212360237
2.7872498156552	4.4368858952175
2.7893816827185	4.4376295160326
2.7920632588117	4.4406765643536
2.7940771922941	4.4439607857517
2.7967314922824	4.4671097342133
2.7993286576597	4.4906742749978
2.8015007412486	4.5029801406059
2.8041886903957	4.5105550478423
2.8068224904936	4.5118932911521
2.8095728217788	4.5170293849491
2.811593252382	4.523497949727
2.81383025559	4.5249559069626
2.8163742812959	4.5305738251848
2.8186046469888	4.5404225505899
2.8211888191437	4.5483973838096
2.8244474217359	4.5519753252238
2.8271864145553	4.5636009264731
2.8298124376879	4.5711194685982
2.8324090351365	4.5734202033068
2.8344846351709	4.5736562008689
2.8371962504271	4.576060446477
2.8398856469331	4.5764575676907
2.8426447530465	4.5810940027961
2.8454115809423	4.5891769590171
2.848116686809	4.5962522750077
2.8505445887668	4.59738130771
2.8529721608358	4.5997024015345
2.8554969777516	4.6003055526145
2.8585141107641	4.6018632993086
2.8608512042312	4.621970201058
2.8639782264595	4.622997084804
2.8667571837981	4.6241673127478
2.8697209245722	4.6276466085635
2.8723056558409	4.6291852103915
2.8752622881991	4.6324942863316
2.8778719807626	4.6356478677343
2.8804936843654	4.6360781816525
2.8837830227539	4.6509632186893
2.8867393186567	4.6529098944751
2.8894965143727	4.6564286357568
2.8923380343484	4.6896884737773
2.8958534658818	4.6940142816458
2.8990181708532	4.7060520246015
2.9014739773086	4.7180818628554
2.9047522927662	4.7181759320619
2.9081711245623	4.7252229764792
2.9104872364706	4.7285116664723
2.9135322900814	4.7354105416048
2.9162655809397	4.7364228845265
2.919162267086	4.7382738991601
2.922107426891	4.7411243066387
2.9252771072384	4.7433883140084
2.9287675641966	4.7511798151072

2.9314642117984	4.7674914177159
2.9344885743395	4.7846965707575
2.937800309102	4.7857525869981
2.9411272878868	4.8033198198397
2.9439625354222	4.8034446125148
2.9469381658954	4.8110438447022
2.9508970417102	4.8199266477466
2.9537885219081	4.8254066921238
2.9567313942083	4.8286387118852
2.9599829172631	4.8286905315234
2.9631253356981	4.8340191464841
2.966672315327	4.8479184870143
2.969999757122	4.8538162864708
2.9729195323351	4.8562011343676
2.9764996109417	4.8596713183225
2.979678812548	4.8614109645788
2.9831482295428	4.8764039657129
2.9870125781098	4.8830416833137
2.9906781470334	4.8917195225458
2.9947092842361	4.895910042823
2.9978684915582	4.9026966656394
3.0019008777744	4.9052817849948
3.0052937951106	4.907906098047
3.0096193392033	4.9192201250722
3.012763113306	4.9228046892585
3.0161602103231	4.9283533445784
3.0198016597612	4.9311017548082
3.0233291190128	4.9389085653614
3.0270787142173	4.9474930026366
3.0306519360389	4.9502717001137
3.0351060072022	4.9503461379731
3.0397240279072	4.9574584254203
3.0441794065988	4.9575597596099
3.048260025164	4.959518526809
3.0520537082684	4.9679177646598
3.0556322626445	4.9837215741807
3.0592396390928	4.9874066608267
3.0626148737962	4.9876680693271
3.0670906107067	4.9896187887498
3.0713102919852	4.9926149627703
3.0760196191825	4.9968216985626
3.0806786967326	4.9983239168181
3.0844648065833	5.0003427042033
3.0888224535503	5.0186486865432
3.0936510893403	5.0202870926682
3.0981749590548	5.0213845805627
3.1022821949863	5.0221433227008
3.1071502556257	5.0254876718583
3.1112362021107	5.026128549188
3.1143911431586	5.0332421930327
3.1186519685419	5.035451458398
3.1226237546136	5.057509140318
3.1277561850717	5.0597411756113
3.1319398255531	5.0654285302613

3.1369353529337	5.0712621395132
3.1412866260099	5.0845938625131
3.1461815452562	5.0877947582344
3.1500606838956	5.091846617239
3.1542760381221	5.0944986736045
3.1598767609558	5.094705559652
3.1646659086507	5.116823866531
3.1697384483928	5.1168662041462
3.1753402280787	5.1176419316108
3.180833937044	5.1213187040743
3.186508401463	5.1272452842161
3.1922194733094	5.1461598702946
3.1966659172918	5.1658484055346
3.2004958073237	5.1742814932855
3.2059369313446	5.1778078278217
3.2104192189793	5.1809904344344
3.2141814757599	5.1908929442842
3.2182436788305	5.2091208168552
3.2234418922213	5.2181460225705
3.228343049033	5.2555993252558
3.2327199878998	5.2721219907379
3.2401315684884	5.2809205163993
3.2460469918906	5.3219309883729
3.2515854206504	5.3674055926084
3.2572039557657	5.3862287414505
3.2634570806375	5.391185121722
3.2692177944791	5.4088906021961
3.2748427154906	5.4228144944846
3.2820161842183	5.4261151018015
3.2872403733261	5.4632475107463
3.2931033163825	5.4633764459544
3.2993156234214	5.4808577366517
3.3055500917949	5.4988825818575
3.3115742119751	5.5076068645754
3.3177805281016	5.5201030217569
3.324191143703	5.5241111147989
3.3317746794842	5.5455891785024
