# Supplementary material for: Comprehensive Expression Profiling of Tumor Cell Lines Identifies Molecular Signatures of Melanoma Progression
Source: PLoS One. 2007 Jul 4;2(7):e594. doi: 10.1371/journal.pone.0000594 (PMC1895889; doi:10.1371/journal.pone.0000594)
Supplement: Supplementary file 2 [file pone.0000594.s004.doc]

Table S2. Results of SAM analysis between HPMs and Group 2 melanoma cells. The result[illegible]

Current settings

Input parameters

Data type?
Arrays centered?
Delta
Minimum fold change
Test statistic
Are data are log scale?
Number of permutations
Input percentile for exchangeability factor s0
Number of neighbors for KNN
Seed for Random number generator

Computed values

Estimate of pi0 (proportion of null genes)
Exchangibility factor s0
s0 percentile
False Discovery Rate (%)

List of Significant Genes for Delta = [illegible]

Negative genes (187)

Row	Gene ID	Gene Name	Score(d)	Numerator(r)
22675	LOC57105	220813_at	-101.1576267	-2367.3166667
18644	D4S234E	209569_x_at	-39.44172217	-8669.75
25076		226311_at	-39.271454619	-631.93333333
25041	MGST1	224918_x_at	-38.649291371	-1941.15
24708	FLJ11585	213924_at	-35.985472964	-2570.8333333
24493	GNAL	206356_s_at	-32.222318668	-798.18333333
22310	MLSN1	206479_at	-32.019186974	-1213.5
14179	CITED1	207144_s_at	-30.361867894	-7682.9
24570		209570_s_at	-29.447208509	-4032.1
24673		213122_at	-29.335098016	-1295.6666667
24691	D4S234E	213533_at	-28.736669687	-2592.9166667
24480	RAB33A	206039_at	-28.057873775	-2287.05
24083		1556698_a_at	-27.853643475	-593.66666667
25117	LOC56849	227705_at	-25.793152598	-939.35
24430	SCYA3	205114_s_at	-25.409502845	-583.25
8261	DKFZP564D037	221036_s_at	-24.531532273	-2319.6166667
6463	CIS4	214462_at	-23.814777853	-1586.3
20240	SERPINF1	202283_at	-23.664743146	-20841.033333
23694		226778_at	-23.261659796	-464.5
4779	RGSZ1	210138_at	-22.594264188	-2991.4
24822	SLC7A8	217248_s_at	-21.944422541	-1616.7666667
18097		225408_at	-20.753619409	-1912.4166667
17060	SLC7A8	216092_s_at	-20.619049196	-5719.9
24403	COX7A1	204570_at	-19.532104709	-336.16666667
24375	DPP4	203716_s_at	-18.567596224	-807.46666667
25519		237472_at	-18.560519582	-500.48333333
24541	MCF2	208017_s_at	-17.669995972	-399.41666667
25095		226970_at	-16.990666428	-588.85

25641		240173_at	-16.977932395	-493.71666667
25260		230903_s_at	-16.91283864	-378.85
22001		1556427_s_at	-16.711313888	-2432.2333333
14115	MC1R	205458_at	-16.676399555	-1657.7333333
22167	UVO	201130_s_at	-16.669948531	-1684.5
24441	CD79B	205297_s_at	-16.412893508	-438.41666667
22777		225407_at	-16.251397622	-4870.9666667
20321	KIT	205051_s_at	-16.205376339	-13244.1
24259		1565162_s_at	-16.083457698	-1033.2333333
27948		211748_x_at	-15.789508435	-507.01666667
25116		227704_at	-15.504239672	-1065.2166667
21510	GNAL	206355_at	-15.249695692	-981.7
18707	KIAA0727	212338_at	-15.122806178	-4149.45
12364		235479_at	-14.850496791	-1625.5333333
25228		229925_at	-14.804849583	-1343.7333333
11275		227542_at	-14.766027231	-1992.8333333
24333	ALDH2	201425_at	-14.749073632	-1054.5666667
22943	MGST1	231736_x_at	-14.628398846	-1903.5
22168	CDH1	201131_s_at	-14.620399622	-9809.75
9943		225144_at	-14.393501388	-2259.75
25114	FLJ10287	227532_at	-14.354129269	-916.53333333
11987		231666_at	-14.270502453	-3721.4166667
11138		227185_at	-14.179551106	-2737.7
24791	PAX-3	216059_at	-14.054991974	-663.26666667
15280		227521_at	-14.019360965	-1238.3333333
24364	CDH3	203256_at	-13.958059999	-6953.8166667
24639	CD26	211478_s_at	-13.814990064	-819.03333333
25012	DKFZp434H241	223693_s_at	-13.59717931	-764.26666667
9490		224566_at	-13.568773708	-14813.2
25354		232687_at	-13.521853083	-722.61666667
3599	HSPC060	206020_at	-13.392849263	-1922.6666667
8767	FLJ12701	222555_s_at	-13.226757322	-3509.0333333
2210	MIR16	202593_s_at	-13.139586355	-2341.95
22183	VAMP8	202546_at	-13.115530313	-4715.1333333
20580	ATP10C	214255_at	-13.110012941	-1764.3666667
15403		229251_s_at	-13.05069501	-2673.8833333
22205	DPP4	203717_at	-13.011709922	-1627.7166667
18495	HNMT	204112_s_at	-12.95500424	-2918.4
28767		229095_s_at	-12.92893245	-198.71666667
17135	FLJ20898	219315_s_at	-12.874778992	-2781.5
3255	MYO5A	204527_at	-12.828919499	-7146.2
29001		232443_at	-12.784822926	-226.48333333
18580	MLANA	206426_at	-12.692190801	-39822.866667
8056	FLJ12584	219637_at	-12.639202213	-2972.7833333
8055	FLJ12584	219636_s_at	-12.546328895	-1713.3166667
19306		236377_at	-12.494904199	-922.33333333
4568	HSNFRK	209481_at	-11.997944677	-2256.7833333
27736	EMR2	207610_s_at	-11.934706344	-360.68333333
2802	STAM	203544_s_at	-11.891176723	-3165.0166667
19616	MAL	204777_s_at	-11.866061852	-2030.6666667
24414	MGMT	204880_at	-11.840153612	-868.55
24348	SLC7A8	202752_x_at	-11.815768197	-4608.55
22448	KIAA0442	212599_at	-11.596244841	-1388.1833333
20289	GMPR	204187_at	-11.581570854	-7484.9833333
28758		228938_at	-11.568373747	-139.35
20058		1555505_a_at	-11.566637065	-28261.366667
8985		223028_s_at	-11.564396312	-2730
17817		51158_at	-11.48748035	-1658.95

3411	AUH	205052_at	-11.423591237	-1333.8166667
15814		241966_at	-11.394773372	-1980
3397	CDK5R1	204995_at	-11.359534441	-1113.1833333
16994	MET	213816_s_at	-11.263455162	-923.88333333
5877	ABR	212895_s_at	-11.257579212	-3251.5333333
2597	KIAA1073	203211_s_at	-11.252511076	-1519.7666667
18582	OCA2	206498_at	-11.219917477	-21281.1
9384		224078_at	-11.099306219	-3518.2666667
25127		228057_at	-11.088704278	-1130.9666667
17578		235279_at	-11.087353594	-629.51666667
11156	KIAA1211	227231_at	-11.043146091	-659.95
25251		230700_at	-11.008804093	-369.25
25495		236901_at	-10.932855723	-540.93333333
22393	PRKCB1	209685_s_at	-10.905938026	-504.7
4055	SAP18	208741_at	-10.886197309	-716.3
23339	FLJ32447	1553485_at	-10.686448588	-623.66666667
5567	KIAA0263	212439_at	-10.60388582	-870.76666667
16990	MGP	213710_s_at	-10.583241259	-1154.6833333
11668		228914_at	-10.572658857	-343.51666667
10474		225924_at	-10.532900384	-2162.95
24694		213556_at	-10.468237718	-368.1
14053	CHC1L	204759_at	-10.442506842	-772.86666667
21117		235758_at	-10.320367561	-1153.9333333
20452		209550_at	-10.32005201	-2198.4
28316	LOC51705	219436_s_at	-10.318037978	-137.21666667
15003	VRL-1	222855_s_at	-10.303382164	-2033
22839		228256_s_at	-10.266503295	-581.53333333
27843	PARC	209924_at	-10.232499777	-990.71666667
11729		229250_at	-10.203856662	-5150.5333333
12337		235372_at	-10.194016109	-3753.7833333
6396	ARF6	214182_at	-10.191085832	-3382.4833333
16180	DKFZP566O084	220690_s_at	-10.12039128	-836.88333333
25058		225792_at	-10.102042458	-296.18333333
2413	UNC13	202893_at	-10.007294832	-1092.1
20634	DCT	216513_at	-9.9987613467	-1284.6666667
12346		235400_at	-9.9166621659	-3461.9333333
22321	HRK	206864_s_at	-9.9013236262	-289.75
20366	ASPA	206030_at	-9.893254521	-1013.1333333
11419	CTSB	227961_at	-9.8911385919	-2955.45
17085		217354_s_at	-9.8789430603	-1058.3166667
11683		228977_at	-9.8340389973	-3094.25
21742		232662_x_at	-9.7994322749	-1275.3166667
2128	IDH3G	202471_s_at	-9.7282688447	-1972.8166667
23357	DBL	1555313_a_at	-9.718188257	-288.25
13492		229713_at	-9.7179059799	-3644.0666667
22433	beta3GalNAcT-1	211812_s_at	-9.712187955	-691.88333333
11159	HSPC182	227244_s_at	-9.7097602462	-648.81666667
22732	CTRP5	223499_at	-9.6836702054	-935.66666667
24636		211427_s_at	-9.6833281291	-373.51666667
13486	RAB5C	229017_s_at	-9.659801132	-2046.9666667
12022		231873_at	-9.5925351661	-765.4
14124	PIP5K2A	205570_at	-9.57687329	-528
28488	DKFZP434N014	221887_s_at	-9.5719332222	-470.75
15202		226397_s_at	-9.5643306104	-2756.0833333
4647	CDC16	209658_at	-9.5415092894	-3994.4166667
11271		227536_at	-9.5179124623	-3274.6333333
23322		32128_at	-9.5042014456	-801.86666667
23205		241600_at	-9.4609191971	-911.63333333

20420	NR4A3	207978_s_at	-9.4174525397	-1732.8
17326	DREV1	226220_at	-9.4140889375	-468.45
7416	FLJ13397	218297_at	-9.3908376043	-1704.6666667
16454		91920_at	-9.3747898987	-767.33333333
14553	MET	213807_x_at	-9.3532478505	-1956.8666667
1531	GYS1	201673_s_at	-9.3104121154	-1894.6833333
27811	SYK	209269_s_at	-9.2423360951	-149.68333333
20388	TYR	206630_at	-9.2056898521	-52330.3
7343	FLJ12701	218202_x_at	-9.1947683936	-1901.7666667
3224	DKFZp564A012	204458_at	-9.185916911	-1294.0333333
10088		225332_at	-9.1845893437	-734.28333333
17131	PELI2	219132_at	-9.151152154	-1150.8333333
1672	LRRFIP1	201861_s_at	-9.1440509831	-1325.8166667
7071	DREV1	217868_s_at	-9.1398163348	-8509.8
8486	RAB9	221808_at	-9.1005680436	-3036.3333333
24393	PPP1R5	204284_at	-9.0786282458	-3014.8333333
25848		244740_at	-9.0610677333	-1012.6833333
1238		201301_s_at	-9.0421288574	-2442.5666667
7867	LOC51167	219079_at	-9.0376463369	-569.85
17373	CYB5-M	227382_at	-9.0328024759	-271.6
8819		222642_s_at	-9.0065273951	-1023.2333333
17556	LOC54472	233881_s_at	-8.9213754941	-572.76666667
15629		235678_at	-8.8907370112	-583.56666667
8691	FLJ10702	222442_s_at	-8.8659418776	-1073.35
23746		230472_at	-8.8475680714	-146.33333333
7682	LOC51309	218694_at	-8.8342013894	-2311.0833333
23686		224494_x_at	-8.8280082943	-525.05
12347		235401_s_at	-8.8160173238	-2535.9666667
14407	tpr/met fusion	211599_x_at	-8.8154739399	-2242.5
24915	FLJ10044	220765_s_at	-8.8138604464	-683.36666667
14990	MEF-2	222771_s_at	-8.7835643171	-1097.9833333
18702	LPIN1	212272_at	-8.7630810777	-453.18333333
13933	ICAP-1A	203337_x_at	-8.7399046024	-2116.4166667
8732	PRO1635	222494_at	-8.7390365978	-1500.9833333
20562	KIAA0455	213496_at	-8.7220295211	-383.21666667
9469		224481_s_at	-8.7027250345	-2040.7333333
26182	MCF2	214884_at	-8.6854627683	-148.46666667
1149	hLAT1	201195_s_at	-8.6791818373	-13092.75
3929	XPNPEPL	208453_s_at	-8.6399986435	-1153.8
17394		227801_at	-8.6160323609	-477.93333333
12045		231972_at	-8.5857610293	-5809.8666667
2573	LOC57020	203173_s_at	-8.5641790252	-710.1
5704		212631_at	-8.5493164783	-7694.4833333

Estimated Miss rates for Delta=4.64707118018563

Quantiles	Cutpoints	Miss Rate(%)
0 -> 0.05	-8.447 -> -3.276	81.79
0.05 -> 0.1	-3.276 -> -2.252	62.73
0.1 -> 0.15	-2.252 -> -1.679	38.27
0.15 -> 0.2	-1.679 -> -1.279	24.09
0.2 -> 0.25	-1.279 -> -0.975	15.33
0.25 -> 0.75	-0.975 -> 0.807	1.54
0.75 -> 0.8	0.807 -> 0.968	19.31
0.8 -> 0.85	0.968 -> 1.169	20.9
0.85 -> 0.9	1.169 -> 1.436	28.16
0.9 -> 0.95	1.436 -> 1.895	36.85
0.95 -> 1	1.895 -> 10.24	56.15

s include the SAM Output showing the list of Affymetrix probesets and associated gene symb

Two class unpaired
0
4.64707118019
0
standard
0
100
Automatic choice
10
1234567

0.7960487323
5.88807646388
0
0.8513890185

4.647

Denominator(s-	Fold Change	q-value(%)
23.4022559046	0.03980341655	0
219.811649265	0.03070636376	0
16.0914165125	0.02373963644	0
50.2247242097	0.0368652162	0
71.4408654827	0.02892145753	0
24.7711327529	0.05192619868	0
37.8991509365	0.03812618897	0
253.044378786	0.05669944872	0
136.926391471	0.02878408325	0
44.1677974273	0.06168905626	0
90.2302422288	0.04093924151	0
81.5118785664	0.03037690253	0
21.313788525	0.02979789726	0
36.4185803357	0.03393839667	0
22.954010693	0.0430680886	0
94.5565340481	0.20448011157	0
66.6099012059	0.198595534	0
880.67861986	0.0288744853	0
19.9684804985	0.07525383237	0
132.396433676	0.24079995939	0
73.6755165766	0.03537086205	0
92.1485852171	0.09151009873	0
277.408523822	0.09129326163	0
17.2109801616	0.04970553592	0
43.4879484101	0.01875481022	0
26.9649419628	0.05800238409	0
22.6042307709	0.04147668187	0
34.6572632982	0.32066220581	0

29.079905325	0.09922155324	0
22.400142759	0.09840552118	0
145.544111594	0.05701805399	0
99.4059495823	0.19968458574	0
101.050102037	0.04134536038	0
26.7117231008	0.09595490944	0
299.726016177	0.04539515802	0
817.265808747	0.00956105878	0
64.2419902937	0.01811904083	0
32.1109848824	0.13048076373	0
68.7048632625	0.12302583735	0
64.3750550717	0.05691916038	0
274.383599911	0.05298292861	0
109.459862269	0.21896296296	0
90.7630520543	0.16994574338	0
134.960697424	0.22946551702	0
71.5005357595	0.08166790032	0
130.123605467	0.06074212968	0
670.963192105	0.02408511866	0
156.997935323	0.29961722636	0
63.8515451636	0.07224078011	0
260.776849229	0.21721129002	0
193.0738131	0.21633342398	0
47.1908250048	0.14971262526	0
88.3302267764	0.34206448299	0
498.193636314	0.00701609084	0
59.2858430977	0.02923630042	0
56.2077361224	0.08596942335	0
1091.71250979	0.13364993216	0
53.4406536032	0.09355661482	0
143.559195574	0.17955720554	0
265.29808084	0.27279195637	0
178.236204456	0.43665207351	0
359.507638727	0.03329882147	0
134.581611367	0.11894001814	0
204.884362954	0.15064932315	0
125.09629222	0.05814334761	0
225.272021992	0.11261117446	0
15.3699207133	0.0263759595	0
216.042543464	0.06912534931	0
557.03833829	0.14616675926	0
17.7150152682	0.11667966719	0
3137.58808788	0.04123530979	0
235.203399965	0.23176945374	0
136.55920238	0.25562989674	0
73.8167591092	0.12162912877	0
188.097494544	0.35813898369	0
30.221383161	0.12983514274	0
266.165135754	0.30974709034	0
171.132317693	0.0680312696	0
73.3563117882	0.08123975247	0
390.033887178	0.07038830055	0
119.709729517	0.07408148519	0
646.28394781	0.06272513639	0
12.045772642	0.12137452711	0
2443.35207445	0.06179638823	0
236.06939147	0.36635409897	0
144.413739951	0.11057795411	0

116.75983839	0.2809226014	0
173.763877116	0.14568636334	0
97.9955066946	0.19567678227	0
82.0248600498	0.15803943012	0
288.830597783	0.3692833912	0
135.060224018	0.52358411703	0
1896.72518032	0.07504645174	0
316.980773141	0.29199954387	0
101.992679969	0.11529184756	0
56.7779011763	0.31395306597	0
59.7610494858	0.28922994076	0
33.5413362677	0.2032581724	0
49.4777711345	0.02084653211	0
46.2775415369	0.16668042599	0
65.7989176271	0.29289239882	0
58.3605172022	0.15732108274	0
82.1176954765	0.48489061098	0
109.104886213	0.37873489006	0.35537889835
32.4910385657	0.39098188695	0.35537889835
205.351794969	0.19473194341	0.35537889835
35.1635117486	0.09479896717	0.35537889835
74.0116026117	0.21830012474	0.35537889835
111.811263168	0.11923571092	0.35537889835
213.022182245	0.10322462216	0.35537889835
13.298716962	0.0756708207	0.35537889835
197.31384972	0.27859195912	0.35537889835
56.643758503	0.12194876441	0.35537889835
96.8205901047	0.09062676886	0.35537889835
504.76339526	0.15768012604	0.35537889835
368.234000553	0.27848628423	0.35537889835
331.906078409	0.35714398842	0.35537889835
82.6927843153	0.41221847638	0.35537889835
29.319153485	0.08344937851	0.35537889835
109.130391217	0.30921281508	0.35537889835
128.482581204	0.10603899192	0.35537889835
349.102679451	0.21797796803	0.58532995022
29.2637642137	0.19747957347	0.58532995022
102.406476168	0.07094604921	0.58532995022
298.797754427	0.36585130351	0.58532995022
107.12853189	0.29038710831	0.58532995022
314.646911695	0.19177473912	0.58532995022
130.141893009	0.22663553763	0.58532995022
202.792161499	0.57825070726	0.58532995022
29.6608783835	0.09611163374	0.58532995022
374.984762582	0.19984483188	0.58532995022
71.2386680053	0.06609525095	0.58532995022
66.8210800492	0.28445914898	0.58532995022
96.6231446161	0.15370236372	0.58532995022
38.5731704728	0.07304462919	0.58532995022
211.905673699	0.18933618476	0.58532995022
79.7912112643	0.42414324945	0.58532995022
55.1328167358	0.31223134037	0.58532995022
49.1802428068	0.166150031	0.58532995022
288.162700099	0.22122539324	0.58532995022
418.635725806	0.30791266355	0.58532995022
344.049532532	0.2436463025	0.58532995022
84.3697044154	0.06905826126	0.58532995022
96.3577972016	0.11177148796	0.58532995022

183.998803572	0.10366232154	0.58532995022
49.7605241579	0.35452979676	0.58532995022
181.524453781	0.28118630965	0.58532995022
81.8507232299	0.42307933286	0.58532995022
209.21787789	0.21587327029	0.58532995022
203.501553943	0.47950404974	0.58532995022
16.1954003613	0.09447469248	0.58532995022
5684.56040134	0.07859132511	0.58532995022
206.831383376	0.30681003584	0.58532995022
140.87143895	0.2862081012	0.58532995022
79.9473232669	0.34345195517	0.58532995022
125.758299498	0.214743043	0.58532995022
144.992265367	0.3969311712	0.58532995022
931.069037742	0.32572143955	0.58532995022
333.642176927	0.36137022509	0.58532995022
332.08027157	0.01830535702	0.58532995022
111.762031048	0.10350271483	0.58532995022
270.131813556	0.43751415292	0.58532995022
63.0529209441	0.41863905325	0.58532995022
30.0681876666	0.50388163303	0.58532995022
113.610194967	0.51831976023	0.58532995022
64.2016096114	0.40491774892	0.58532995022
65.6376030388	0.27240612597	0.58532995022
121.064407462	0.63921614763	0.58532995022
16.5393848516	0.26428691135	0.58532995022
261.606367283	0.34773200871	0.58532995022
59.4754765169	0.25381937043	0.58532995022
287.654455921	0.27024641977	0.58532995022
254.38223915	0.22182701483	0.58532995022
77.5331843323	0.269946406	0.58532995022
125.004302774	0.18332155652	0.8513890185
51.7150679443	0.3438306909	0.8513890185
242.155579833	0.19431384865	0.8513890185
171.756156018	0.36609864082	0.8513890185
43.9366394873	0.17043691597	0.8513890185
234.493601169	0.55170393802	0.8513890185
17.0936967468	0.09443936159	0.8513890185
1508.52352737	0.31850299947	0.8513890185
133.541687633	0.57754059645	0.8513890185
55.4702342464	0.11665588516	0.8513890185
676.686277062	0.16424036673	0.8513890185
82.9151279891	0.46651140077	0.8513890185
900.011521728	0.15992211881	0.8513890185

ools that are downregulated in Group 2 melanoma cell lines.

Significant: 187
Median number of false positives: 1.592
False Discovery Rate (%): 0.851

Tail strength (%): 29.7
se (%): 18.4

SAM Plotsheet

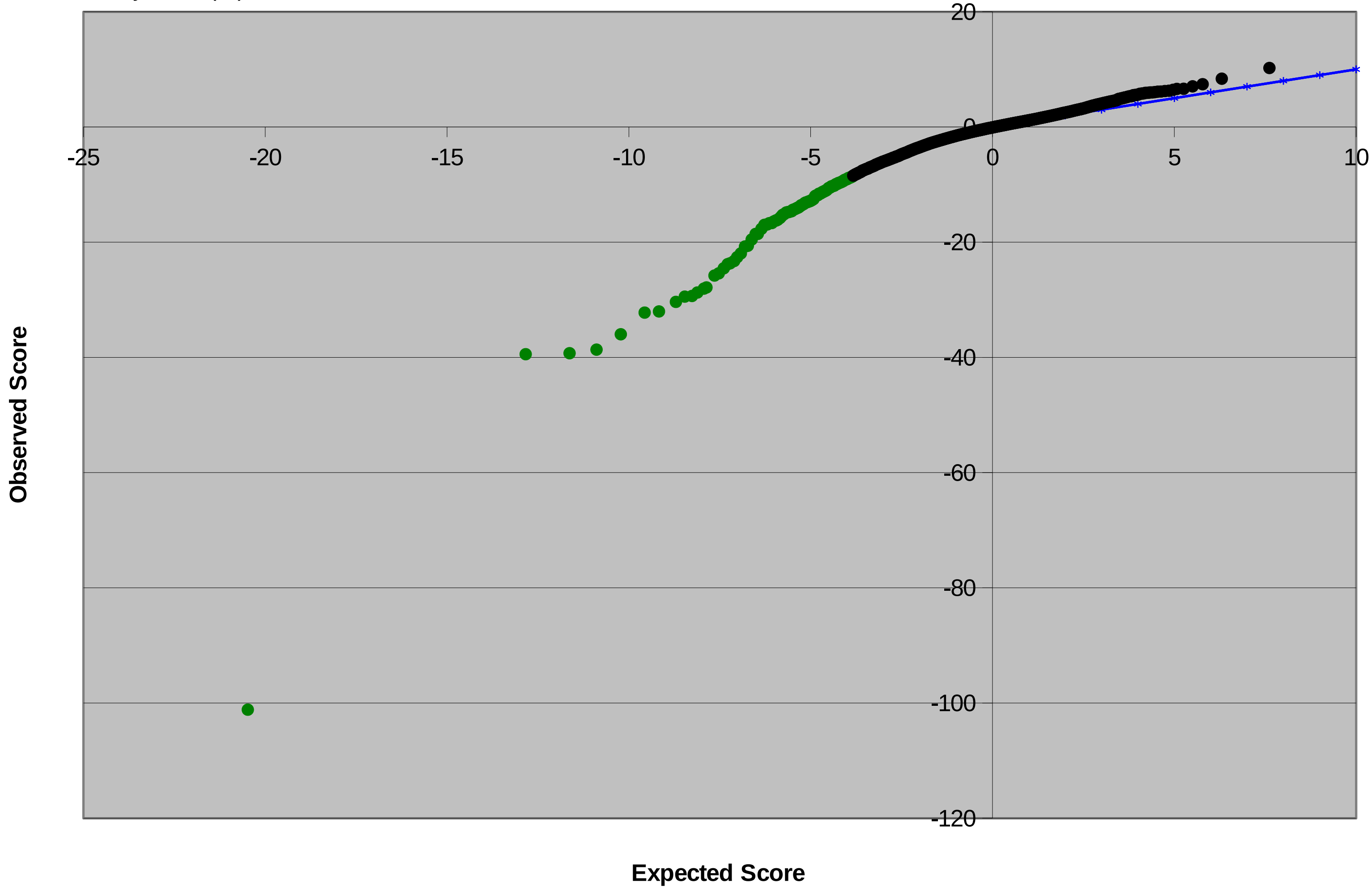

Expected Score	Observed Score	Negative X	Negative Y
-3.829354078575	-8.447361677246	-20.479308	-101.15763
-3.822105128206	-8.402574028153	-12.837857	-39.441722
-3.816901602376	-8.390339416111	-11.631398	-39.271455
-3.811158815522	-8.376686266295	-10.889789	-38.649291
-3.806471472995	-8.374324121382	-10.22282	-35.985473
-3.800137346366	-8.357353597597	-9.5668088	-32.222319
-3.795105214934	-8.327799351141	-9.1725488	-32.019187
-3.789379479024	-8.319169668631	-8.707021	-30.361868
-3.784311391636	-8.287750182387	-8.4620955	-29.447209
-3.77908312456	-8.262057053234	-8.2625064	-29.335098
-3.774798151366	-8.257390789864	-8.1148184	-28.73667
-3.769983520864	-8.256730330962	-7.9355276	-28.057874
-3.765383257877	-8.248582575285	-7.8645571	-27.853643
-3.759161594355	-8.224290952465	-7.6450516	-25.793153
-3.753932540088	-8.182328192328	-7.5273225	-25.409503
-3.74944966512	-8.17158347355	-7.3893196	-24.531532
-3.74314544305	-8.164287518458	-7.2818942	-23.814778
-3.738066139199	-8.16242792624	-7.2140615	-23.664743
-3.732895521843	-8.151880636456	-7.1025596	-23.26166
-3.728403364031	-8.146280956468	-7.0201922	-22.594264
-3.723219063542	-8.126975771917	-6.9192985	-21.944423
-3.716513614806	-8.100585852493	-6.8059023	-20.753619
-3.710530892393	-8.077062514346	-6.7275584	-20.619049
-3.703353296012	-8.042318527649	-6.6216663	-19.532105
-3.696728564454	-8.012165147049	-6.5132218	-18.567596
-3.691365433757	-7.983415611733	-6.4531256	-18.56052
-3.687725754848	-7.982712228979	-6.3526713	-17.669996
-3.683830341906	-7.971161670355	-6.2714726	-16.990666
-3.678462475985	-7.9453279725	-6.2352424	-16.977932
-3.673327819159	-7.942520488899	-6.1911372	-16.912839
-3.670307652057	-7.934156609566	-6.139788	-16.711314
-3.666733597463	-7.929619026798	-6.1088111	-16.6764
-3.663219594812	-7.919454465512	-6.0660734	-16.669949
-3.659286018797	-7.911910977195	-6.017405	-16.412894
-3.655190760134	-7.906130300892	-5.9630438	-16.251398
-3.650781670922	-7.87838599992	-5.9370252	-16.205376
-3.646287770194	-7.871812398881	-5.9019918	-16.083458
-3.64066862676	-7.864535935715	-5.842667	-15.789508
-3.634670055676	-7.851098996152	-5.8024911	-15.50424
-3.631486512175	-7.831373454464	-5.7675973	-15.249696
-3.627755648001	-7.828327076987	-5.7267655	-15.122806
-3.62270931676	-7.807496814466	-5.6723851	-14.850497
-3.618178346041	-7.77689807634	-5.6461392	-14.80485
-3.614912180463	-7.772452863729	-5.6140742	-14.766027
-3.610216445034	-7.759085734161	-5.5897036	-14.749074
-3.60363012434	-7.699684789364	-5.5447777	-14.628399
-3.598370361838	-7.672124352421	-5.5220375	-14.6204
-3.594261573445	-7.666047081673	-5.4928323	-14.393501
-3.588956451607	-7.627248453134	-5.4702917	-14.354129
-3.58550404713	-7.61580586377	-5.4409429	-14.270502
-3.582209638781	-7.605784482954	-5.4060314	-14.179551
-3.577974259235	-7.597879183654	-5.3722235	-14.054992
-3.573932882617	-7.589382075104	-5.3506979	-14.019361

-3.570083742567	-7.55772177302	-5.3323973	-13.95806
-3.56638168241	-7.5492415407	-5.3005565	-13.81499
-3.56249501766	-7.544891516794	-5.2645186	-13.597179
-3.55840464868	-7.542730646459	-5.2453922	-13.568774
-3.554151283586	-7.525757910126	-5.2278607	-13.521853
-3.55118878323	-7.525298486661	-5.1955511	-13.392849
-3.546466747831	-7.494709808979	-5.1664654	-13.226757
-3.544238534193	-7.494297028073	-5.1431196	-13.139586
-3.541208889485	-7.490169845999	-5.1285569	-13.11553
-3.538652540785	-7.490115277855	-5.1165097	-13.110013
-3.534557272959	-7.489321263084	-5.0983828	-13.050695
-3.530489155495	-7.471256137438	-5.0819479	-13.01171
-3.527134263245	-7.463489499741	-5.0633478	-12.955004
-3.523160194818	-7.446657197053	-5.0463859	-12.928932
-3.518565048911	-7.433463845119	-5.021583	-12.874779
-3.514170021452	-7.418842007895	-5.0104877	-12.828919
-3.509263648755	-7.40761656579	-4.9906497	-12.784823
-3.506127785439	-7.403606358183	-4.9692898	-12.692191
-3.501058217125	-7.401040611165	-4.9521255	-12.639202
-3.495405131024	-7.362748353724	-4.9346723	-12.546329
-3.491634925311	-7.358057055328	-4.9182943	-12.494904
-3.48856822224	-7.356607879256	-4.8774357	-11.997945
-3.485551974024	-7.35352002535	-4.861329	-11.934706
-3.481143913403	-7.351879443783	-4.8439785	-11.891177
-3.477456346938	-7.339361089132	-4.8290673	-11.866062
-3.472303302934	-7.309495930827	-4.8128457	-11.840154
-3.468791215121	-7.304263126418	-4.7995473	-11.815768
-3.462579956392	-7.273379372722	-4.7743757	-11.596245
-3.460192837559	-7.272464055806	-4.7649858	-11.581571
-3.457559714845	-7.268840214344	-4.7504507	-11.568374
-3.45335534179	-7.257313522581	-4.7352622	-11.566637
-3.450050095242	-7.250009954736	-4.7277197	-11.564396
-3.446036967039	-7.246170312735	-4.7172582	-11.48748
-3.443439929748	-7.245568603903	-4.7060091	-11.423591
-3.440762442542	-7.241866702796	-4.6956351	-11.394773
-3.437707501833	-7.238050602201	-4.6818349	-11.359534
-3.434315223889	-7.234788523169	-4.6669023	-11.263455
-3.429429983063	-7.221898618868	-4.6564042	-11.257579
-3.426809054842	-7.221151326036	-4.6455424	-11.252511
-3.423011672592	-7.210286862724	-4.6352747	-11.219917
-3.420306896499	-7.204956808792	-4.6132276	-11.099306
-3.415099084448	-7.179542038491	-4.6035179	-11.088704
-3.412128652959	-7.176559017371	-4.5951776	-11.087354
-3.407950923372	-7.157331508425	-4.5846957	-11.043146
-3.404497904602	-7.143303669643	-4.5727396	-11.008804
-3.401808968134	-7.131687799554	-4.5606956	-10.932856
-3.398697521729	-7.118278400459	-4.5499377	-10.905938
-3.394210267003	-7.091616688472	-4.5401041	-10.886197
-3.390373180269	-7.083343273481	-4.519767	-10.686449
-3.387290676602	-7.069095692028	-4.5043334	-10.603886
-3.383534944187	-7.042330837103	-4.4941229	-10.583241
-3.380748647802	-7.040441290828	-4.4846971	-10.572659
-3.378540635486	-7.029880961783	-4.4725829	-10.5329
-3.375850272829	-7.028952580753	-4.4548329	-10.468238

-3.372464743468	-7.012818264782	-4.4439623	-10.442507
-3.369088846158	-7.009286801201	-4.4324072	-10.320368
-3.366571212688	-7.005714070968	-4.424323	-10.320052
-3.363650238342	-7.004013749722	-4.4162133	-10.318038
-3.360239630363	-6.98815692079	-4.4065883	-10.303382
-3.356337741336	-6.979023957474	-4.3948024	-10.266503
-3.353612921051	-6.968240314645	-4.3858325	-10.2325
-3.350136447597	-6.96591496947	-4.3759272	-10.203857
-3.347067489036	-6.957727916913	-4.3666922	-10.194016
-3.343939262526	-6.951763027217	-4.3523797	-10.191086
-3.341529191344	-6.950704361576	-4.3393105	-10.120391
-3.338960944735	-6.94926090211	-4.3316866	-10.102042
-3.33537164365	-6.937128611023	-4.321022	-10.007295
-3.33230708228	-6.930405124359	-4.3130779	-9.9987613
-3.329348667697	-6.923561224692	-4.300714	-9.9166622
-3.326247605488	-6.919147892467	-4.289663	-9.9013236
-3.323127931342	-6.910638234216	-4.283497	-9.8932545
-3.319716414552	-6.899044614087	-4.2780006	-9.8911386
-3.316981759391	-6.88636099153	-4.2692427	-9.8789431
-3.313361149924	-6.879850288371	-4.2587225	-9.834039
-3.311470060579	-6.879769633955	-4.2504705	-9.7994323
-3.308860332839	-6.872928638132	-4.2385792	-9.7282688
-3.307046826543	-6.867870824702	-4.2310115	-9.7181883
-3.303373671878	-6.853941141405	-4.2254849	-9.717906
-3.300133049711	-6.847190573945	-4.2191055	-9.712188
-3.297423901889	-6.831636726738	-4.2094297	-9.7097602
-3.295448559373	-6.830205537192	-4.2031034	-9.6836702
-3.293290122232	-6.829427236382	-4.1940173	-9.6833281
-3.289618454957	-6.807644348288	-4.1865034	-9.6598011
-3.286406251003	-6.799772051346	-4.1761694	-9.5925352
-3.28328624105	-6.799589235568	-4.1702473	-9.5768733
-3.279830023559	-6.782772761779	-4.1616599	-9.5719332
-3.277453284814	-6.776506010742	-4.1550224	-9.5643306
-3.274579457932	-6.769466080327	-4.1475589	-9.5415093
-3.27194480832	-6.76431076094	-4.1414179	-9.5179125
-3.268278322697	-6.760976253631	-4.1366684	-9.5042014
-3.265222824349	-6.753112597812	-4.1292051	-9.4609192
-3.261330647638	-6.751523030513	-4.1212288	-9.4174525
-3.258296225797	-6.738692515882	-4.1146193	-9.4140889
-3.255592311365	-6.735217928752	-4.1066054	-9.3908376
-3.252349736473	-6.720323297853	-4.0992318	-9.3747899
-3.249267749222	-6.703408440755	-4.0944074	-9.3532479
-3.247319899789	-6.699305575695	-4.0872822	-9.3104121
-3.242730835913	-6.698508760338	-4.0782154	-9.2423361
-3.239395151874	-6.685916541059	-4.0697232	-9.2056899
-3.236063992293	-6.666532883397	-4.0650735	-9.1947684
-3.233712261991	-6.655125985333	-4.0596311	-9.1859169
-3.23106798076	-6.644603244423	-4.0522623	-9.1845893
-3.228129603453	-6.633984905777	-4.0457986	-9.1511522
-3.225626634254	-6.623408014928	-4.0381528	-9.144051
-3.221013335704	-6.591038421213	-4.0323502	-9.1398163
-3.218617720388	-6.582367730832	-4.0241216	-9.100568
-3.216405250684	-6.580800233413	-4.0151551	-9.0786282
-3.213932718227	-6.578468893348	-4.0090582	-9.0610677

-3.21212653416	-6.577861828519	-4.0015355	-9.0421289
-3.209721364712	-6.569552934728	-3.9966457	-9.0376463
-3.206557268965	-6.55335612453	-3.990568	-9.0328025
-3.203273451479	-6.541703766416	-3.9839437	-9.0065274
-3.200558552087	-6.528323114475	-3.9748888	-8.9213755
-3.198197411936	-6.524187224566	-3.9662763	-8.890737
-3.195226058544	-6.514142509048	-3.9597794	-8.8659419
-3.193023457311	-6.507957259264	-3.9523557	-8.8475681
-3.190740956391	-6.507887752328	-3.9467517	-8.8342014
-3.187889862807	-6.499774880713	-3.940479	-8.8280083
-3.184975803963	-6.482885498008	-3.9360457	-8.8160173
-3.182562378377	-6.48094762124	-3.9310333	-8.8154739
-3.18019492659	-6.478000571405	-3.9254683	-8.8138604
-3.178183297022	-6.473771393644	-3.9162809	-8.7835643
-3.175802138934	-6.469062324233	-3.9099021	-8.7630811
-3.172839722917	-6.458859988859	-3.9043711	-8.7399046
-3.170703530958	-6.457874272984	-3.9002753	-8.7390366
-3.168402056937	-6.450240750118	-3.8941089	-8.7220295
-3.166482689012	-6.446695825491	-3.8879528	-8.702725
-3.163992976844	-6.441827643598	-3.8814202	-8.6854628
-3.161781860588	-6.440639187282	-3.8750607	-8.6791818
-3.159262802661	-6.439365088045	-3.8681242	-8.6399986
-3.156528199474	-6.432059349292	-3.8608734	-8.6160324
-3.151997972471	-6.413163192036	-3.8538799	-8.585761
-3.149925744054	-6.40185338183	-3.8481346	-8.564179
-3.147770042287	-6.391583768871	-3.8423845	-8.5493165
-3.144738973149	-6.382571073565		
-3.143282022133	-6.382033140415		
-3.139976481479	-6.375768160017		
-3.137615418841	-6.360646987642		
-3.135538395736	-6.353376669314		
-3.13289208138	-6.349933590656		
-3.13031050592	-6.345827044422		
-3.127734354474	-6.326630984489		
-3.125630326428	-6.325719237893		
-3.123899171973	-6.320432409693		
-3.121261668322	-6.30692923451		
-3.119441645708	-6.305391516806		
-3.117724375116	-6.304633073663		
-3.115862608059	-6.302919955207		
-3.113360193654	-6.299955919844		
-3.111418911683	-6.298886531468		
-3.109801650713	-6.297811866428		
-3.10829632036	-6.296904995171		
-3.105928361465	-6.288345454659		
-3.103172728756	-6.286930895499		
-3.10048162147	-6.282387265983		
-3.098930255211	-6.280326254913		
-3.097653978527	-6.279328745381		
-3.09542725676	-6.273399834257		
-3.093023005444	-6.267872348505		
-3.090090157847	-6.241899605639		
-3.087787682833	-6.236726200928		
-3.085398539395	-6.228346803688		

-3.083212540901	-6.223810151069
-3.080434923561	-6.22124937725
-3.077850649428	-6.21083254747
-3.074716506462	-6.20187129819
-3.072315105708	-6.19020025049
-3.070252195974	-6.1901687908
-3.067648092704	-6.174247866118
-3.065454036693	-6.173806374259
-3.063026904779	-6.170722580868
-3.061160433283	-6.169488875183
-3.058971791707	-6.164137859581
-3.05675872033	-6.159000432331
-3.05482440616	-6.148123370461
-3.052541471653	-6.135154156687
-3.051272274577	-6.130959941473
-3.049387269628	-6.126504967352
-3.047271541896	-6.117587753111
-3.044677979629	-6.106442827422
-3.042950719239	-6.105973431159
-3.040017003889	-6.084975475304
-3.03819143602	-6.08396044767
-3.036191714344	-6.08176936386
-3.034326105305	-6.08108445313
-3.032426379132	-6.074460678248
-3.030320037324	-6.070213147483
-3.02878782648	-6.065616342542
-3.026758668928	-6.064190569402
-3.024959768841	-6.061070189056
-3.023065990699	-6.05491485901
-3.02166819022	-6.053774131893
-3.020154851254	-6.053658551247
-3.01835708394	-6.050786472788
-3.016579852299	-6.040823724421
-3.014527021464	-6.034338427233
-3.012169755803	-6.033317956526
-3.010179501905	-6.024684603657
-3.008483134214	-6.022848405415
-3.006623930339	-6.022342355337
-3.004310899592	-6.006327204975
-3.001740915097	-6.002607280128
-2.999250448403	-5.997312826421
-2.996931358977	-5.996203429944
-2.995046550591	-5.990721282572
-2.993081492693	-5.980838002468
-2.989951442585	-5.980367221747
-2.988189389449	-5.97955393967
-2.986840728862	-5.978962623671
-2.98446211658	-5.973761546745
-2.982695749612	-5.969098459086
-2.981345069034	-5.967947415942
-2.979846008126	-5.96592857261
-2.978704804096	-5.964387467297
-2.976947163456	-5.961814416188
-2.97465441425	-5.952051369774

-2.971897573251	-5.93911237671
-2.969158995592	-5.93147652319
-2.967785121958	-5.929222137734
-2.965772173267	-5.92350222358
-2.964516132896	-5.922101190108
-2.962463939536	-5.921481672417
-2.959864356993	-5.915354299472
-2.957501792555	-5.905097337765
-2.95615660966	-5.904433032072
-2.9544067617	-5.901600321803
-2.95306216533	-5.892917989497
-2.951243169414	-5.889649953518
-2.948353975618	-5.877734441873
-2.947014528603	-5.87574935511
-2.945124458381	-5.87533851383
-2.943618800841	-5.874818013097
-2.94189403267	-5.863819845482
-2.940468704135	-5.863502766711
-2.938858780351	-5.862500115288
-2.937012184113	-5.861851074402
-2.93516826159	-5.859678630876
-2.9333997418	-5.853454327514
-2.931556959211	-5.851230603088
-2.92991482886	-5.848468464082
-2.92792892999	-5.842523191466
-2.926702771468	-5.83968761078
-2.92440980554	-5.833871708107
-2.922213694724	-5.829983794877
-2.920150240967	-5.825439444906
-2.918280092576	-5.820490596433
-2.91730539816	-5.819885720243
-2.915675400868	-5.817432261758
-2.914190790939	-5.811905329979
-2.912328825571	-5.804719913046
-2.910152585358	-5.800389596035
-2.90839146557	-5.798236774305
-2.906201231034	-5.784419029498
-2.904594802717	-5.780293960167
-2.902930223571	-5.776507635676
-2.901584482193	-5.774666401188
-2.899991025505	-5.773328694154
-2.897926499407	-5.766515670111
-2.896954825563	-5.766024415891
-2.895639696528	-5.764172181835
-2.893851803087	-5.763745273988
-2.892306019032	-5.760657382799
-2.890967325959	-5.75824474069
-2.88998298959	-5.754792262505
-2.887569016176	-5.750300937861
-2.886151903761	-5.748130219577
-2.885148304223	-5.747830777833
-2.883790385264	-5.744345700573
-2.881801338414	-5.734411855095
-2.880632684482	-5.732178549191

-2.878685710655	-5.728383359349
-2.8766274421	-5.727853515236
-2.87474637636	-5.72382891455
-2.872661329896	-5.713590562919
-2.871081683691	-5.709093443898
-2.869029705584	-5.701098872793
-2.867466988192	-5.700111115928
-2.865788024107	-5.695862677684
-2.864272708358	-5.694218690877
-2.862590449616	-5.69196747678
-2.861209776333	-5.69054554745
-2.859338465567	-5.68022222277
-2.857729464268	-5.679499332974
-2.85632168143	-5.675878262676
-2.855044956312	-5.672118810531
-2.853984045015	-5.671999440832
-2.851847895015	-5.663030915957
-2.848904344737	-5.641257136289
-2.847638436754	-5.639564698288
-2.846583654025	-5.639096658715
-2.845382618662	-5.63875409859
-2.843923460985	-5.63830653648
-2.842197800913	-5.631794587286
-2.84043746865	-5.628214045577
-2.839213686597	-5.628201851807
-2.838128463921	-5.626261297911
-2.836036333451	-5.619048577982
-2.834853369227	-5.618677326423
-2.833233178509	-5.61286395788
-2.832114552293	-5.611391331748
-2.829919677465	-5.595752332629
-2.827969687651	-5.591944687763
-2.826809249844	-5.591922286534
-2.824803180417	-5.578324016853
-2.82314431551	-5.575966507136
-2.82225020485	-5.575326918666
-2.820611098455	-5.57439241971
-2.819210099768	-5.574290537819
-2.817985369684	-5.57300581403
-2.816835322244	-5.571741175171
-2.815102061233	-5.571500626177
-2.813475515669	-5.570806945116
-2.810839649842	-5.550115580549
-2.809336554259	-5.549682157079
-2.808387650517	-5.549616971238
-2.806774710458	-5.546800824246
-2.805058526575	-5.541779371336
-2.803754822288	-5.541574748541
-2.802471910858	-5.539719057951
-2.800519584579	-5.533060635426
-2.79935433708	-5.532071823058
-2.797922280611	-5.529044471984
-2.796620375229	-5.525691876091
-2.795368747325	-5.521951837516

-2.794323512529	-5.521726449077
-2.792780934284	-5.519757629513
-2.791675364625	-5.51787086311
-2.790448101915	-5.517091233402
-2.7890106137	-5.511502634453
-2.78774364318	-5.508475973632
-2.78627444314	-5.507924005116
-2.784349157299	-5.503831550693
-2.783057092327	-5.499224235848
-2.781557356936	-5.49782136599
-2.779929510056	-5.494983302468
-2.778403207255	-5.487257749362
-2.775804137387	-5.471011066992
-2.774521772253	-5.470432252072
-2.77269578988	-5.468543008152
-2.771283929297	-5.465487000201
-2.770258959618	-5.462654789842
-2.768993934714	-5.458290174025
-2.767330229607	-5.453303761721
-2.766159992696	-5.45293043043
-2.764564988332	-5.445218426453
-2.76326781741	-5.442697037615
-2.761290141016	-5.427793888596
-2.759967918838	-5.426991089126
-2.75870950589	-5.421354708893
-2.757310356094	-5.415526669915
-2.756538681557	-5.413241522602
-2.755200416462	-5.408257668917
-2.753299676956	-5.4065043021
-2.751370387675	-5.398757911978
-2.749529671227	-5.389278627211
-2.747969042026	-5.385271819704
-2.746717277545	-5.383935669233
-2.744997942417	-5.381261096999
-2.743589828856	-5.373821982929
-2.742205109268	-5.37361780917
-2.740502570667	-5.369020923593
-2.739218342413	-5.365550690363
-2.737512481467	-5.362009330715
-2.736490419456	-5.360786364764
-2.73488411874	-5.356358840996
-2.733440066073	-5.35456133054
-2.731387940196	-5.35361658462
-2.729868239899	-5.349339686717
-2.728359684383	-5.347742639168
-2.72674957953	-5.34382943491
-2.724848368788	-5.332056505176
-2.723177840986	-5.331403435529
-2.72162940457	-5.32837831949
-2.720511639877	-5.32185606801
-2.719439970324	-5.320721951101
-2.717614677208	-5.316467093329
-2.716755076238	-5.316075065927
-2.715410431757	-5.314598465765

-2.714005574877	-5.310263399171
-2.71317294192	-5.310073088089
-2.711295643447	-5.307040421976
-2.709763085893	-5.305680365671
-2.708555244507	-5.304543553222
-2.707424565029	-5.300044735466
-2.705500037955	-5.294766303834
-2.704533406928	-5.29407105535
-2.703193377327	-5.290783825986
-2.702230667519	-5.286779360961
-2.701185789311	-5.285125591082
-2.699737305004	-5.283657020904
-2.698742357954	-5.282385251325
-2.697857453451	-5.282069831496
-2.696585439118	-5.278745155336
-2.69553245591	-5.278496799673
-2.694483308858	-5.278141275174
-2.693497036216	-5.278041617153
-2.692539762816	-5.276923384137
-2.691494083385	-5.276214692095
-2.690565718695	-5.276172679998
-2.689339873939	-5.272915723735
-2.687931991799	-5.267216760021
-2.686532732657	-5.264505622626
-2.68529195194	-5.261874498186
-2.683488873659	-5.249920446285
-2.681937400175	-5.248656520857
-2.680715308064	-5.245938172688
-2.679694239574	-5.244212690179
-2.677671000206	-5.232039249905
-2.676506702011	-5.228059885885
-2.675273269793	-5.22802069527
-2.674140874899	-5.22764869703
-2.673017016101	-5.220947746692
-2.671281337368	-5.213557853193
-2.67026596279	-5.209571896345
-2.669125567064	-5.204835745955
-2.66786320924	-5.20365029919
-2.666225748487	-5.199044723803
-2.664992617598	-5.197444735637
-2.663898705367	-5.193567814327
-2.662515050931	-5.189284736422
-2.661356468626	-5.182033267882
-2.659668180508	-5.171722465555
-2.658114308278	-5.167612120518
-2.657368296563	-5.167359211221
-2.656202753431	-5.163123954278
-2.655311495083	-5.162996148265
-2.654474481775	-5.162595898881
-2.653506764238	-5.161330376172
-2.652023953637	-5.15609274937
-2.651060801291	-5.155447359912
-2.649951294221	-5.152560614287
-2.649234029958	-5.152455343859

-2.648207207612	-5.151585608515
-2.647243043077	-5.150448257243
-2.645758208471	-5.149815688373
-2.644894664857	-5.149760858533
-2.643664000615	-5.14655812029
-2.642096121855	-5.143843991632
-2.641339493857	-5.143625478995
-2.640291826962	-5.141060876777
-2.638573444867	-5.131596464953
-2.637208917456	-5.125693038517
-2.636116585796	-5.124095062347
-2.635094319102	-5.123003350282
-2.634043517816	-5.122264756375
-2.633051470361	-5.122040010866
-2.631616568676	-5.11878867675
-2.630708092634	-5.117917231814
-2.629587400322	-5.112381864905
-2.628662002585	-5.111710796947
-2.627547838128	-5.11038381021
-2.626507231835	-5.106835539433
-2.625525987505	-5.103814589162
-2.624719326623	-5.103430363142
-2.623954472722	-5.102671637635
-2.622264929817	-5.093739946149
-2.621079756451	-5.089719231117
-2.619770003055	-5.088207644257
-2.618590880531	-5.086910322855
-2.617832137652	-5.086813675938
-2.616976193458	-5.086190293456
-2.615975910561	-5.085751332695
-2.614531580582	-5.082567257117
-2.613433961459	-5.081188071223
-2.612345032367	-5.073769342676
-2.611241967668	-5.072708809002
-2.609668526491	-5.072019364872
-2.608510512284	-5.071333228475
-2.606475736519	-5.06970171821
-2.605346551173	-5.064905413255
-2.604537596034	-5.064417885586
-2.603498279921	-5.063725601137
-2.6024244119	-5.062153045152
-2.601805139754	-5.060078670289
-2.600890291574	-5.05957362474
-2.599700289549	-5.058589002455
-2.598192179431	-5.054209628907
-2.596425975863	-5.043872754105
-2.595286370386	-5.04031480244
-2.594422574247	-5.037598202193
-2.593368326661	-5.037015000641
-2.592581823643	-5.036445498617
-2.591653939342	-5.034307295335
-2.590057411148	-5.025952554946
-2.588647602617	-5.022445063698
-2.587457487762	-5.017521165921

-2.58634979536	-5.016903130934
-2.584862898582	-5.007648394719
-2.583812807315	-5.006776441062
-2.582466843325	-5.002292041191
-2.581662263147	-5.002142892568
-2.580568352159	-5.002086628626
-2.579436942924	-4.996415574167
-2.578537416899	-4.995923227116
-2.576871437871	-4.989520696045
-2.575506925542	-4.987680877143
-2.573558479034	-4.976648272582
-2.572471927596	-4.975845999349
-2.571145861738	-4.970879082299
-2.569798457457	-4.962797390876
-2.568467414524	-4.958873244466
-2.567498233739	-4.95377693989
-2.56670948363	-4.953451817097
-2.565025991499	-4.947009301863
-2.563768704956	-4.942556549671
-2.562891092773	-4.941667632652
-2.561939163771	-4.940307439392
-2.561184324304	-4.939495182521
-2.559994986056	-4.931934882328
-2.558754279659	-4.929949321042
-2.557466384016	-4.928039695452
-2.555374325984	-4.917880198751
-2.553883558257	-4.911759120579
-2.552649493756	-4.907739173998
-2.551732384759	-4.905014336242
-2.550699130133	-4.90223512349
-2.549868015645	-4.901600638934
-2.548332831633	-4.892145168824
-2.547553210314	-4.890773078611
-2.546045470085	-4.887853541238
-2.545121369087	-4.883644539507
-2.543999987178	-4.879237336821
-2.54258020465	-4.868924713129
-2.541236465844	-4.86501984496
-2.539690717621	-4.857113743348
-2.538978755363	-4.856485700507
-2.53802265638	-4.855460330642
-2.537194358894	-4.854334842881
-2.536025092896	-4.851539402216
-2.534829616105	-4.847734783301
-2.534127687746	-4.847525387952
-2.533334424692	-4.846741072791
-2.532571909254	-4.846282569357
-2.531394660109	-4.844140790883
-2.529716072888	-4.830130011783
-2.528728582791	-4.829758381866
-2.52745256099	-4.825737245192
-2.52638755863	-4.820343306901
-2.525192026192	-4.819261623554
-2.523515173446	-4.811735447145

-2.522585436382	-4.810622590713
-2.521392012556	-4.805241182215
-2.520638085579	-4.80454639086
-2.519612207706	-4.801004137764
-2.518801573215	-4.797973917012
-2.516919659814	-4.78972708043
-2.516139332798	-4.788897351646
-2.515140030113	-4.786376026227
-2.513945382382	-4.785586840216
-2.512952071204	-4.784900376261
-2.511849735005	-4.779620906426
-2.510592900518	-4.774260474618
-2.509495121186	-4.771863394961
-2.508695704866	-4.770710118162
-2.507544780912	-4.763784502327
-2.506680448066	-4.763148612869
-2.505722733621	-4.760739893835
-2.504906164699	-4.757796070459
-2.503924305235	-4.757129985536
-2.503188542331	-4.75576884674
-2.502389387523	-4.754626221995
-2.501671005996	-4.754117938937
-2.500945705926	-4.75073607159
-2.500075491624	-4.745915783444
-2.499074801226	-4.745363230781
-2.497923500436	-4.74166186558
-2.497033746187	-4.741128684294
-2.495691254283	-4.735176421177
-2.494891479986	-4.735170847034
-2.494047452942	-4.732002364088
-2.493432544183	-4.731312678758
-2.492548019143	-4.731087480077
-2.491590737286	-4.729727083991
-2.490130023012	-4.724596701373
-2.489307882019	-4.723192582542
-2.488228882414	-4.722437949617
-2.487252957805	-4.720191648117
-2.485417321856	-4.70561371585
-2.484173646536	-4.703332974649
-2.483091456275	-4.703045675405
-2.482066981948	-4.699672559281
-2.481209378938	-4.699125478172
-2.47992188909	-4.697018483643
-2.479221415094	-4.696237868425
-2.478229303671	-4.690387000322
-2.477308444848	-4.687374497388
-2.475968013167	-4.681223759944
-2.474771291566	-4.675714089769
-2.473822093727	-4.675315593739
-2.47310855192	-4.674793801982
-2.472088851809	-4.673699762599
-2.471403263734	-4.67168382319
-2.470361455967	-4.667937732366
-2.469324092677	-4.667812950879

-2.468076538205	-4.665430812859
-2.46726933425	-4.66508416879
-2.466587382058	-4.664990552348
-2.465886796075	-4.663930502489
-2.464781422745	-4.661610511439
-2.463657605591	-4.656352229122
-2.462538053972	-4.655212969576
-2.461795302295	-4.654134097711
-2.460899786757	-4.653661385556
-2.459993826318	-4.653409975706
-2.458959848257	-4.649316274186
-2.457706395135	-4.646295320088
-2.456754102176	-4.645233372897
-2.455615371663	-4.643283298604
-2.454950737755	-4.641119431809
-2.454196671477	-4.641042124054
-2.45316670113	-4.637978360254
-2.452056544162	-4.630337191098
-2.451444972834	-4.62951003923
-2.450417165464	-4.623998032713
-2.449593359422	-4.622100013837
-2.448918175488	-4.621851436548
-2.447956958374	-4.620191156237
-2.446780157807	-4.616701260546
-2.44528738905	-4.60884955055
-2.444498766856	-4.608668614297
-2.443523010706	-4.603406741497
-2.442558996278	-4.601413302331
-2.441533575304	-4.598705115576
-2.440517228909	-4.596193521175
-2.439758778779	-4.596013812614
-2.438904686999	-4.595874312614
-2.437655552204	-4.593778112841
-2.436930689497	-4.592078479259
-2.436342255527	-4.59045508542
-2.435500585111	-4.5888368503
-2.434489573017	-4.587926767491
-2.433712282185	-4.586398296757
-2.432998261543	-4.585880111204
-2.432215723801	-4.585240530814
-2.431583982305	-4.584646209033
-2.430861909746	-4.584040909258
-2.429987225699	-4.583627014088
-2.428495150985	-4.576678740036
-2.427679133134	-4.576538636541
-2.42693062616	-4.575019326794
-2.425754575229	-4.57073439052
-2.424954207701	-4.568882083687
-2.42386006406	-4.564631731975
-2.423148794148	-4.564119931635
-2.422557328094	-4.562980458664
-2.421692120638	-4.561116677347
-2.420927080591	-4.55870704794
-2.420111294695	-4.555112505151

-2.419021324128	-4.551264638337
-2.418378126456	-4.550137247209
-2.417560650187	-4.550077821012
-2.416344406308	-4.542087596015
-2.415606595861	-4.541777966788
-2.4148473894	-4.537237424249
-2.413897838122	-4.532048646279
-2.412945937877	-4.531201490423
-2.411404446027	-4.523291630782
-2.410422670615	-4.522798315683
-2.409538048292	-4.518381811258
-2.408706737756	-4.516758956436
-2.407891671612	-4.513483616352
-2.407066862015	-4.513254873682
-2.406384254085	-4.510762564403
-2.405515979135	-4.508966329261
-2.404747673573	-4.507674208791
-2.403880825692	-4.506856815835
-2.402900586384	-4.506632437815
-2.40229903103	-4.504726866937
-2.401309605787	-4.50316183673
-2.400639953914	-4.501998735666
-2.399585949939	-4.50004743849
-2.398897293919	-4.499036815768
-2.397653971666	-4.498896181076
-2.396429453706	-4.49743085474
-2.395680950158	-4.496510239386
-2.395031042164	-4.495918019106
-2.393847602344	-4.494749218606
-2.392891162713	-4.490149999509
-2.391897769253	-4.488855690023
-2.390992076018	-4.487952109167
-2.390165381499	-4.487250242741
-2.389388792975	-4.486280644592
-2.388315882814	-4.483264128779
-2.387601925641	-4.481846877248
-2.386923997083	-4.48127603953
-2.385829224622	-4.480102120306
-2.384680307687	-4.477173019393
-2.384064765533	-4.475437547402
-2.383351680291	-4.475416422443
-2.382671291198	-4.474529220404
-2.381664187042	-4.471557813103
-2.380672836991	-4.469931792728
-2.379729785695	-4.469529071989
-2.378729141278	-4.463332462038
-2.377714280042	-4.460079379274
-2.376707948435	-4.458529507248
-2.375824541215	-4.457139979568
-2.374962831202	-4.456920708946
-2.374412817531	-4.455773016478
-2.373569217134	-4.452206888221
-2.372977585352	-4.450386823324
-2.372286887889	-4.44944810254

-2.37133730202	-4.447072223802
-2.369844823923	-4.433140334489
-2.369061921151	-4.431011385854
-2.368100455068	-4.430264891472
-2.367226896066	-4.427274404214
-2.36662235504	-4.425992504689
-2.365803592518	-4.42541532961
-2.36525106115	-4.424978540612
-2.364414219757	-4.421240847462
-2.363631564037	-4.419309075394
-2.362929329129	-4.419144471186
-2.362021907611	-4.419002031573
-2.361369598114	-4.41762161674
-2.360511085295	-4.415304582586
-2.359547150047	-4.415193576405
-2.358897278464	-4.414762756019
-2.358257414609	-4.414369001668
-2.357674348924	-4.414245003058
-2.356755893066	-4.410433786175
-2.355926937454	-4.407221516065
-2.355111221884	-4.405390332762
-2.354369138255	-4.404556486805
-2.353822406006	-4.404241131239
-2.352991485781	-4.403242384399
-2.352248871406	-4.402425444145
-2.35156557648	-4.40218142225
-2.350362273503	-4.396691107721
-2.349889275959	-4.395768471135
-2.349031068458	-4.393486631694
-2.347999697474	-4.388550514307
-2.347266141258	-4.386182969024
-2.346393510144	-4.383515493462
-2.345617672879	-4.38097033519
-2.344797738308	-4.379713894511
-2.343930141716	-4.37634989288
-2.34283547617	-4.369899075269
-2.342274250938	-4.369418016704
-2.341507516678	-4.369375801823
-2.340759773666	-4.367709689225
-2.339093025996	-4.351080069599
-2.338171514586	-4.348185222185
-2.337391130043	-4.346622236272
-2.336101973246	-4.343926781931
-2.335225595451	-4.343722633438
-2.334637042057	-4.343021490331
-2.33402309398	-4.342756519565
-2.33333620614	-4.341502152006
-2.332768658068	-4.340165263385
-2.332156099026	-4.339443720891
-2.331578897699	-4.33703061862
-2.330840064911	-4.336145828972
-2.3302355575	-4.335258487234
-2.32940847095	-4.331865441743
-2.328568993744	-4.329593202955

-2.327727811882	-4.326787366234
-2.327122955573	-4.325463085859
-2.326411990858	-4.324403757239
-2.325630157246	-4.323602650117
-2.325029175978	-4.321967999576
-2.324113705283	-4.320366152143
-2.322997303776	-4.312269084059
-2.322327302009	-4.311852790653
-2.321387264956	-4.309564352215
-2.320655982828	-4.309342379168
-2.319932881965	-4.304012528996
-2.318998776542	-4.301517551943
-2.318110491463	-4.295670059953
-2.317340712609	-4.291805317339
-2.3166405094	-4.290652438333
-2.315702194554	-4.288471142744
-2.314774122788	-4.28662830911
-2.314095549367	-4.28394746788
-2.313309876708	-4.283917098321
-2.312821345127	-4.281616548462
-2.312304468981	-4.279198851081
-2.311268177019	-4.274701334695
-2.310879798044	-4.274259989461
-2.310283003803	-4.271689811857
-2.309672279927	-4.271201051787
-2.308788997402	-4.268177680964
-2.308092271246	-4.266397261274
-2.307165515853	-4.262739715174
-2.306315719563	-4.26126252595
-2.304940393768	-4.247750277098
-2.303935806758	-4.246184419265
-2.303326225337	-4.245924699481
-2.302447353391	-4.244815144682
-2.301556139906	-4.243498529656
-2.300587264893	-4.239336565249
-2.300048139156	-4.238971788929
-2.299426589603	-4.238736411712
-2.298765851997	-4.23868546933
-2.298024612027	-4.234734797996
-2.297302809648	-4.233746714388
-2.296497357639	-4.231876623718
-2.295867193229	-4.231256249554
-2.295005623874	-4.228346101614
-2.294079170993	-4.22535141383
-2.293118275874	-4.221024407268
-2.291626152722	-4.211555312624
-2.29090309873	-4.21006042336
-2.290024804416	-4.208241453747
-2.289212779151	-4.205643254009
-2.288466051276	-4.204651516735
-2.287394641128	-4.199266317543
-2.286624313533	-4.196602560897
-2.285362216384	-4.191138555477
-2.284696306202	-4.190041606636

-2.283921857018	-4.186653635086
-2.282810234184	-4.181699350805
-2.282016648026	-4.178848390682
-2.280835107506	-4.173398473154
-2.280153069752	-4.173115715934
-2.279593355998	-4.171716406923
-2.278996854932	-4.170929625363
-2.278105424885	-4.167887755631
-2.277575753916	-4.167850894587
-2.276697354345	-4.164749075582
-2.276213798152	-4.163926970864
-2.275622875321	-4.16300703585
-2.27462321756	-4.156590304995
-2.274075794993	-4.155317024927
-2.273496572566	-4.153290333146
-2.272897838719	-4.153173532778
-2.272288411602	-4.152259124691
-2.271520606205	-4.150498747649
-2.27089398335	-4.150165196093
-2.26972752517	-4.145094542059
-2.268969853113	-4.14279858594
-2.268410799802	-4.141492659266
-2.26777269538	-4.138573508598
-2.266849443753	-4.136997551713
-2.266210197159	-4.135102363876
-2.26547836646	-4.134289829684
-2.264543337317	-4.133694842709
-2.263789497706	-4.132134652507
-2.263039855022	-4.132006563167
-2.262436461419	-4.13157146763
-2.261629868465	-4.130940883296
-2.260914132527	-4.127769165855
-2.260068911989	-4.126742359705
-2.259409912686	-4.126612517651
-2.258875566461	-4.126211924096
-2.258160373463	-4.125748889578
-2.257522968716	-4.125203158569
-2.256708602664	-4.123795134953
-2.255864838673	-4.12228219386
-2.254980888075	-4.121449829457
-2.2542652396	-4.120987846209
-2.253862024787	-4.12034555691
-2.253179674812	-4.120056595363
-2.252163584344	-4.113997548271
-2.251213735657	-4.112145072437
-2.250759276333	-4.111947692214
-2.249863375528	-4.107918473679
-2.249210853564	-4.106876462102
-2.248730077856	-4.106175767695
-2.24821580204	-4.106138861212
-2.247477432081	-4.10492435246
-2.246647677811	-4.1023784028
-2.24609890582	-4.101845442859
-2.24528094945	-4.100696057227

-2.244306512976	-4.096639198637
-2.243284388744	-4.095272488012
-2.242224999054	-4.090938530348
-2.241382486599	-4.089479791149
-2.240536295639	-4.084869795948
-2.239904927115	-4.083070952992
-2.23942003002	-4.0825264724
-2.238916262597	-4.082365474569
-2.238366592279	-4.081325445082
-2.237290938397	-4.080138705932
-2.236524928914	-4.079278196756
-2.235770728223	-4.074648779425
-2.235210703146	-4.074415460832
-2.234361546606	-4.073655384531
-2.233880008345	-4.073174623745
-2.233324057493	-4.072705161217
-2.232641093132	-4.071795507325
-2.231598958567	-4.06807108919
-2.231070999442	-4.067445312597
-2.230509180281	-4.066972708426
-2.22984517883	-4.066458907423
-2.228909098142	-4.061974838507
-2.228152141457	-4.060472554388
-2.226975232655	-4.055535358736
-2.226376368589	-4.053537200067
-2.22598428812	-4.052947593714
-2.225452835184	-4.052468414942
-2.224337065549	-4.050500353868
-2.223635679328	-4.04657159224
-2.22274862452	-4.045933062474
-2.221904836579	-4.041459547574
-2.22078414624	-4.031910163751
-2.2200884243	-4.028608133426
-2.219473636538	-4.026077339202
-2.218982477722	-4.025820796894
-2.218486947309	-4.024781024907
-2.217934191553	-4.024582459294
-2.217063288031	-4.024196696969
-2.216319041582	-4.024007081756
-2.21563111631	-4.021178353999
-2.214958008087	-4.01963483398
-2.214165980958	-4.014773327852
-2.213449496154	-4.013264915904
-2.21287656173	-4.012956380371
-2.21229815293	-4.012710975447
-2.211691150973	-4.011027681937
-2.211208678309	-4.010755384331
-2.210437645848	-4.009491609923
-2.209629934586	-4.004945961893
-2.208975694086	-4.003178241443
-2.208061067878	-4.002474839907
-2.207358284068	-4.001256301802
-2.206431326022	-3.999310415638
-2.205909763742	-3.998579732596

-2.205278445586	-3.997330766041
-2.204623209236	-3.996957916227
-2.203841532113	-3.99461837083
-2.203266527448	-3.993408072412
-2.20250053881	-3.988851823834
-2.201991873043	-3.988179347216
-2.201176649512	-3.9853326915
-2.200671191269	-3.985141472378
-2.200111113536	-3.984814954054
-2.199541174464	-3.983183056083
-2.198639030666	-3.982677283698
-2.197989418648	-3.980190840355
-2.197183180545	-3.975694446451
-2.196447201006	-3.971534941307
-2.195878332101	-3.969218114751
-2.195103657039	-3.969031259243
-2.194589439659	-3.968926714382
-2.193955075878	-3.967618372921
-2.193348745416	-3.967118768175
-2.192634347053	-3.965507098635
-2.191468116815	-3.95445415655
-2.190748036376	-3.951817460311
-2.190251819793	-3.951243409977
-2.189561204993	-3.94986551888
-2.188790743978	-3.947965281882
-2.187830031828	-3.943736375316
-2.187140699646	-3.942340462373
-2.186399527478	-3.940043052402
-2.185539895157	-3.938133383578
-2.184791247418	-3.934041749686
-2.184196078989	-3.933755801048
-2.183649159515	-3.931997929039
-2.182938361703	-3.931915148592
-2.182124702788	-3.931653362517
-2.181645044536	-3.930319981826
-2.180876588885	-3.926793600076
-2.180367754124	-3.9251240039
-2.179560339688	-3.920856010245
-2.179012579299	-3.920320386322
-2.178260109765	-3.920081936946
-2.177537152804	-3.918145548301
-2.176905754912	-3.917320005325
-2.176280658573	-3.916486546459
-2.175561003989	-3.912002384336
-2.175154090824	-3.911601434944
-2.174377624661	-3.910049377456
-2.173712504172	-3.90900481541
-2.173279959832	-3.908583279501
-2.172647296039	-3.908293130718
-2.171528786044	-3.904121459866
-2.170955001354	-3.903347649274
-2.170442302719	-3.903022786815
-2.169706875988	-3.900757586349
-2.169230940955	-3.899746913528

-2.168489766977	-3.898974160148
-2.167944041515	-3.897147892433
-2.167388484749	-3.896414579971
-2.166517750644	-3.89272210731
-2.165963906069	-3.891848124649
-2.165433124763	-3.891815757936
-2.164747290275	-3.890943970926
-2.163929689142	-3.88875807783
-2.162955063346	-3.885143199238
-2.162393890228	-3.884831652461
-2.161658558641	-3.882658044956
-2.160845257212	-3.882315784958
-2.160425774343	-3.881720964711
-2.159571803538	-3.875028884124
-2.158927467885	-3.874735789124
-2.158250478343	-3.873438914746
-2.157738170135	-3.872508320744
-2.157098799561	-3.872389626689
-2.156183591504	-3.866689933472
-2.155562829177	-3.866551269358
-2.154999923047	-3.86643093166
-2.154183663347	-3.864439899747
-2.153360600821	-3.863910429779
-2.152782428046	-3.863826444422
-2.152199819349	-3.863419513113
-2.151774808978	-3.863347265773
-2.151029650609	-3.863015802374
-2.150306168957	-3.857252826935
-2.149652340439	-3.855510920455
-2.149094969574	-3.85465325725
-2.148412906312	-3.85439669676
-2.147759078177	-3.851381698348
-2.147182039317	-3.850657544639
-2.146391653297	-3.846164790867
-2.145602802902	-3.842192160857
-2.145041320844	-3.840245351453
-2.144398128582	-3.836633076473
-2.143759329165	-3.835743773347
-2.143102105248	-3.834902608016
-2.142609275174	-3.833955991638
-2.141898925894	-3.831540305933
-2.141455138712	-3.830532592742
-2.140759346322	-3.82903218399
-2.13999731495	-3.826765268889
-2.139482173004	-3.826524373736
-2.138745167967	-3.823043429588
-2.138016540869	-3.82111737096
-2.137447987462	-3.820439156876
-2.136883789497	-3.818995220648
-2.136441472965	-3.818922508896
-2.135886188386	-3.81812750843
-2.135441634257	-3.818102531841
-2.13479210394	-3.817841658395
-2.133974201162	-3.81371516951

-2.133457841486	-3.813024529629
-2.133014314213	-3.812169102897
-2.132395202694	-3.807452823684
-2.131702702962	-3.807041991579
-2.13121384019	-3.806617681607
-2.130642330869	-3.804466005206
-2.130068982042	-3.803768312162
-2.129418440073	-3.802058314779
-2.128613909783	-3.79840322822
-2.127685938416	-3.793799448102
-2.126970703502	-3.791217090539
-2.126258116735	-3.789798954384
-2.125667777789	-3.788529417063
-2.124884952577	-3.786716901282
-2.124308323893	-3.785976727781
-2.123665159713	-3.784593316154
-2.123081181748	-3.783990553841
-2.122310329296	-3.783807089919
-2.121810721601	-3.783432141231
-2.121254886849	-3.781231541378
-2.12040089862	-3.775204990226
-2.119833472559	-3.774435546745
-2.119325656636	-3.773482573552
-2.118780400352	-3.771133466072
-2.118264487349	-3.770912606023
-2.117414345498	-3.767102695011
-2.116670671023	-3.765842188547
-2.11612021608	-3.764332900176
-2.115293115994	-3.763542682188
-2.114811770892	-3.763097961143
-2.113762505131	-3.757971171597
-2.113051296363	-3.756373221011
-2.112380169272	-3.754773067535
-2.11189912487	-3.753672887416
-2.111456112597	-3.7532022798
-2.11063443597	-3.75113718591
-2.110195013096	-3.750763153784
-2.109481026314	-3.750097577594
-2.108859694505	-3.748899746868
-2.108072530612	-3.744809330936
-2.107316062061	-3.744104884827
-2.10671430034	-3.741355167673
-2.106205117423	-3.741352799964
-2.105587663252	-3.740663445442
-2.104949928185	-3.739716057386
-2.104411426835	-3.739210062722
-2.103993115055	-3.738578995325
-2.103292615699	-3.7376905096
-2.102826876639	-3.736826014354
-2.102033549876	-3.735898981488
-2.101602635891	-3.735897120065
-2.100967318561	-3.734370258379
-2.100333435766	-3.732959245024
-2.099746236587	-3.731666184635

-2.098779547242	-3.721307192229
-2.098238197459	-3.72091016884
-2.097755759766	-3.719900260086
-2.09739748273	-3.719378140211
-2.096936552225	-3.718966921158
-2.096449977297	-3.718146530805
-2.095937294417	-3.715765468801
-2.095422816321	-3.714330750267
-2.09476055918	-3.713620562626
-2.094131736855	-3.712140401092
-2.093535442115	-3.710955575591
-2.093072650907	-3.710840049404
-2.092376260078	-3.708434266351
-2.091608538022	-3.704570086941
-2.091174884662	-3.704071624253
-2.090677837478	-3.702903927512
-2.090130450076	-3.701974476018
-2.089746794036	-3.70078959983
-2.089104219409	-3.699340607141
-2.088577960135	-3.697793678511
-2.088019901399	-3.69773678851
-2.08743515573	-3.697039640354
-2.086890041568	-3.695515937987
-2.08629476087	-3.694711344033
-2.085678993526	-3.694632505721
-2.08515564802	-3.694144681376
-2.084751328583	-3.692375503222
-2.084133612403	-3.691153340078
-2.083197531358	-3.690369979277
-2.082792177058	-3.689368752882
-2.082214421462	-3.687409086565
-2.081780682874	-3.686570680332
-2.081051556215	-3.684754315492
-2.080401893871	-3.683321495804
-2.079885609579	-3.683258726423
-2.079418935369	-3.682940844792
-2.079040699817	-3.682559526011
-2.078587932654	-3.682043659194
-2.077984481728	-3.680669348154
-2.077470795746	-3.679769991497
-2.076952087844	-3.679701899806
-2.076396634412	-3.679596008736
-2.07593357857	-3.678605580795
-2.075457214606	-3.677223389922
-2.075018411581	-3.677056014151
-2.074318620745	-3.674270842459
-2.073763355177	-3.671420845576
-2.073117441304	-3.670577877983
-2.072582810888	-3.66895375455
-2.072082144188	-3.66796478777
-2.071623649597	-3.667215600254
-2.070840402957	-3.663775720821
-2.070197071203	-3.663128407479
-2.069596464151	-3.66098348705

-2.06899070435	-3.659324554241
-2.068449698152	-3.657252958534
-2.067919290115	-3.655403279499
-2.067556618923	-3.654230045922
-2.06706587699	-3.65400283979
-2.066567162505	-3.65378540739
-2.066056875756	-3.653775179746
-2.065532291559	-3.653459402613
-2.064885167769	-3.652369356863
-2.064407697011	-3.650892615323
-2.063876826524	-3.650890177066
-2.063398475348	-3.649997362255
-2.062890713697	-3.647999383718
-2.062248196141	-3.647292608731
-2.061810231609	-3.646991069962
-2.061120930139	-3.646087486174
-2.060668935784	-3.645888831892
-2.059642526037	-3.639799596714
-2.05902889461	-3.637524735512
-2.058572860134	-3.636941701125
-2.057832252023	-3.63366365535
-2.057248139495	-3.632629005528
-2.056880709604	-3.632603240437
-2.05632336235	-3.630660990255
-2.055450313479	-3.629695114656
-2.054926509308	-3.629636640843
-2.054339445515	-3.62623197427
-2.053669862865	-3.625252196171
-2.053155570868	-3.62377381649
-2.052594516889	-3.623293782995
-2.051939569796	-3.622301078439
-2.051346803152	-3.621926042627
-2.051070978638	-3.621886858495
-2.050325873283	-3.619697953337
-2.049762645931	-3.61907308874
-2.049155339046	-3.618725065425
-2.048465064972	-3.618209663225
-2.04789086526	-3.615698355798
-2.047527051696	-3.614267158377
-2.046855019217	-3.613479638761
-2.04651243797	-3.613458583191
-2.046105297121	-3.612815532115
-2.045513604662	-3.612670212613
-2.044955250738	-3.609686167078
-2.044069972256	-3.607938718839
-2.043430953374	-3.60751213863
-2.042797014622	-3.606577287125
-2.042414994194	-3.606331696772
-2.041790411561	-3.605412324644
-2.041231922174	-3.60509701828
-2.04071213912	-3.604762586302
-2.039972944339	-3.603515739547
-2.039531554359	-3.603146470142
-2.038897371096	-3.603138548387

-2.038326264553	-3.602270486451
-2.037907784804	-3.602171898483
-2.037386862916	-3.601151860223
-2.036919872236	-3.600596189963
-2.036374911496	-3.598964314139
-2.035393154423	-3.594143289091
-2.034381184876	-3.587565146375
-2.033754107687	-3.583598805146
-2.033044175766	-3.581950320398
-2.032480634073	-3.581660591063
-2.031970822123	-3.581336171603
-2.031397491997	-3.577199371432
-2.030927983818	-3.576412470166
-2.030413905273	-3.57563273818
-2.029962519387	-3.573879578069
-2.02932886954	-3.570983836548
-2.028783160069	-3.570207437159
-2.028321225671	-3.569055128746
-2.027644962189	-3.566742339922
-2.027298659923	-3.565198905624
-2.026737331565	-3.56498825642
-2.026117318591	-3.564969319638
-2.025702332247	-3.564079318777
-2.02510662259	-3.563502898565
-2.024361743957	-3.56133231287
-2.02377663753	-3.560067212118
-2.023223027744	-3.559548785812
-2.022767261213	-3.559478105715
-2.022271232956	-3.558517030869
-2.021930223417	-3.558068877331
-2.021477431884	-3.557836548243
-2.020974147371	-3.557022037021
-2.02036972888	-3.554863813041
-2.019748259441	-3.553264123335
-2.019211741725	-3.552959659902
-2.018862963289	-3.552861090734
-2.018288765899	-3.551756994122
-2.017808998116	-3.551310455176
-2.017107802517	-3.546247090739
-2.016570409108	-3.546202143131
-2.016032944369	-3.542816147793
-2.015583685505	-3.542749722294
-2.014906644917	-3.54095542795
-2.014519733886	-3.539985730724
-2.013856692689	-3.536479832081
-2.013425115835	-3.536473527218
-2.012938857457	-3.535720298909
-2.012534137591	-3.535687353422
-2.012009839503	-3.535054776267
-2.011440239893	-3.533988967165
-2.010902378806	-3.533553024502
-2.010440998459	-3.533505677643
-2.009872694062	-3.53221347619
-2.00917790159	-3.527278704454

-2.008673567214	-3.526899850835
-2.007664143595	-3.522772739638
-2.007289247371	-3.521473272528
-2.006791400779	-3.52089300099
-2.006437706897	-3.520757762492
-2.005971491762	-3.520467118012
-2.00539611576	-3.520442398231
-2.004835650479	-3.518484660596
-2.004501149079	-3.51832585977
-2.004053704116	-3.517414859417
-2.003674247508	-3.515707899543
-2.002984507455	-3.511576086366
-2.00245355671	-3.511087159682
-2.001928252142	-3.510816757826
-2.001575164879	-3.510422190091
-2.001160885206	-3.510046479391
-2.000517633169	-3.507994953122
-1.999995134755	-3.50701456726
-1.999557581645	-3.506619040356
-1.998894309248	-3.505532151486
-1.998464350337	-3.504656925652
-1.998058670724	-3.504254837271
-1.997772463996	-3.50387977216
-1.997433803995	-3.503820555579
-1.996927243493	-3.503629741552
-1.996378823624	-3.501810638299
-1.995768905277	-3.50125444847
-1.994972737745	-3.497681677385
-1.994413874687	-3.495628370238
-1.993345991206	-3.486193846401
-1.992836524532	-3.486044831465
-1.992514456002	-3.48566088591
-1.991930085473	-3.482826776909
-1.991149569409	-3.477491493905
-1.99076198487	-3.477199094374
-1.990327729948	-3.476994003695
-1.989838585308	-3.476216451212
-1.98946047949	-3.475177540806
-1.988984616122	-3.47510285443
-1.988310494349	-3.47442340357
-1.988013099872	-3.474236505513
-1.987315896668	-3.473026617572
-1.986789247905	-3.472881702292
-1.986237959302	-3.471523685732
-1.985772652039	-3.470553926186
-1.985229348152	-3.468343283663
-1.984676487746	-3.465902669986
-1.984045406801	-3.463038921142
-1.983356373789	-3.462935335005
-1.982627814917	-3.460298559054
-1.98218191917	-3.459846751514
-1.981762937191	-3.459386529007
-1.981102988163	-3.457501439313
-1.980614964556	-3.457361977076

-1.979998155408	-3.456650497963
-1.979019815217	-3.449448988878
-1.978374921846	-3.448656525876
-1.977865746684	-3.447970311697
-1.977446490378	-3.447801609297
-1.976805704334	-3.444056493164
-1.976240851381	-3.440661871208
-1.975770692886	-3.440555722429
-1.975184755599	-3.439949444181
-1.974584439148	-3.43958638053
-1.974056658861	-3.43890799119
-1.973712294585	-3.438739886754
-1.973339647467	-3.438457041071
-1.972912933207	-3.437137251886
-1.972583832525	-3.436640586042
-1.972139794026	-3.435321965269
-1.971728100503	-3.434518969762
-1.971297175849	-3.433721054282
-1.970884976502	-3.432346939522
-1.970515458531	-3.431758077239
-1.969988527718	-3.43071127534
-1.969493785318	-3.429666882175
-1.969048266806	-3.428779293357
-1.968678923886	-3.427028381368
-1.967956010883	-3.425838530648
-1.96743727723	-3.424588398217
-1.967034390994	-3.424106286459
-1.966605475411	-3.423739867714
-1.966259448913	-3.423639946053
-1.965722224987	-3.421565822415
-1.965183621856	-3.41903307831
-1.964623784624	-3.418086219955
-1.964174277916	-3.417767143449
-1.963744575737	-3.417573833633
-1.963237252378	-3.417400175658
-1.962880546231	-3.416402189233
-1.96219466947	-3.411508366147
-1.961765639068	-3.411272598972
-1.961098260541	-3.4103231022
-1.960623359465	-3.409819885135
-1.960174187199	-3.409684702882
-1.959668112556	-3.407626101641
-1.959225295176	-3.406846017705
-1.958804313553	-3.40608948153
-1.95831552926	-3.405849357732
-1.957864886747	-3.405597832245
-1.957333729316	-3.405171256317
-1.957011939799	-3.404947257355
-1.956674560867	-3.404701403903
-1.956170554226	-3.403900984832
-1.955362947648	-3.398120983215
-1.955008127812	-3.397381366294
-1.954393751952	-3.393178603854
-1.953785329282	-3.391046136983

-1.953434449831	-3.390924346519
-1.952792213655	-3.38846245007
-1.952445808667	-3.388253227188
-1.95177477991	-3.387979023595
-1.951442714756	-3.387450689494
-1.950930073079	-3.385808303959
-1.950313874704	-3.38339418746
-1.949759625344	-3.382323285443
-1.949501453817	-3.381770034591
-1.948936843102	-3.380692665062
-1.948486920739	-3.379586892812
-1.94792835404	-3.376895138767
-1.947463919031	-3.375582630167
-1.947149930878	-3.375479641385
-1.946509608917	-3.372653698634
-1.945946048434	-3.371074464358
-1.945463784236	-3.368064571381
-1.945066249594	-3.367375299838
-1.944636393177	-3.366508246877
-1.944171839138	-3.366010005579
-1.943709602614	-3.365169724848
-1.942995070182	-3.362876299986
-1.94261545879	-3.362288662534
-1.9421571876	-3.360862118971
-1.941688806653	-3.360775313777
-1.94119605695	-3.358256397626
-1.940835849866	-3.357255398556
-1.940417267283	-3.356861459396
-1.93993565724	-3.356507900318
-1.939650935475	-3.356279484366
-1.939094494194	-3.356178916054
-1.93872214108	-3.355890173528
-1.938275943978	-3.355261244614
-1.937749199576	-3.355010862842
-1.937098800333	-3.351192434916
-1.936667941985	-3.34987817763
-1.936236956154	-3.347550880711
-1.935631252336	-3.346154563447
-1.935081665697	-3.345619184829
-1.93469738901	-3.344877405885
-1.934151245947	-3.343348929985
-1.933685141555	-3.343255196278
-1.933046586608	-3.341413348681
-1.932669603609	-3.341410973018
-1.932218474555	-3.34009278768
-1.931745337925	-3.339476891965
-1.931417498356	-3.337485232632
-1.931039006739	-3.337226627898
-1.930541097023	-3.336641269073
-1.930034324964	-3.335331634754
-1.929341860648	-3.331489997125
-1.928944835444	-3.330408370289
-1.92838140558	-3.330063359905
-1.927830715041	-3.329201036849

-1.927363108457	-3.328952999946
-1.926891302568	-3.328782630219
-1.926371994955	-3.326830484447
-1.925875479791	-3.326256192173
-1.925371851885	-3.326055035233
-1.924744983136	-3.325016029856
-1.924271261444	-3.32476809499
-1.923683608576	-3.322976302588
-1.923243594874	-3.322694429227
-1.922624318397	-3.320723605172
-1.922212656278	-3.319916795282
-1.921816630445	-3.319228285344
-1.921391154984	-3.318925043022
-1.920885783879	-3.316231487213
-1.920415921861	-3.315724512104
-1.920133835125	-3.314185788895
-1.919767479041	-3.312765841485
-1.919270061156	-3.312073197564
-1.91884820105	-3.311395755117
-1.918482413043	-3.310604135054
-1.918065813983	-3.30903587792
-1.917709246594	-3.30858038675
-1.917184262365	-3.308400254183
-1.916751223651	-3.307067072249
-1.916176779375	-3.305428639683
-1.91576970253	-3.305289173455
-1.915245445638	-3.30440247578
-1.914749153105	-3.304192913317
-1.914220529825	-3.30253344365
-1.913835682859	-3.30228447097
-1.913314017096	-3.301516967973
-1.91288117396	-3.300715023357
-1.912350803467	-3.300098370185
-1.911658255351	-3.297352119327
-1.911355162827	-3.296785731201
-1.910832094673	-3.295661578676
-1.910331182904	-3.293650231566
-1.909914399703	-3.29353719619
-1.90946109776	-3.293028295681
-1.908914032023	-3.292418065519
-1.908542612999	-3.291748821584
-1.908122115498	-3.291037913458
-1.907769016682	-3.291018954583
-1.907275244892	-3.28945670255
-1.906753592866	-3.287739951744
-1.906255044433	-3.284753464414
-1.905880694575	-3.283752580717
-1.905424158709	-3.283128492831
-1.904809848199	-3.28149507707
-1.904506771917	-3.281479057966
-1.903913258614	-3.277900387839
-1.903589584777	-3.27699386934
-1.903234631055	-3.276591249739
-1.902748282997	-3.274599837808

-1.902255397803	-3.274126705978
-1.901789566457	-3.273179972704
-1.901292456437	-3.271917614933
-1.9009379361	-3.271682759812
-1.900130343	-3.269531823749
-1.89986735975	-3.269263243855
-1.899334589875	-3.267567163825
-1.898846506513	-3.266313723526
-1.898477511339	-3.266276369965
-1.898130295784	-3.265631709529
-1.897769774693	-3.265537024679
-1.897350947984	-3.264723880159
-1.896870718986	-3.26239312844
-1.896560630523	-3.261688391168
-1.896201571113	-3.261191139716
-1.895681784064	-3.257669466579
-1.895356116783	-3.257362214289
-1.894969559317	-3.257121277982
-1.894514364558	-3.256212297456
-1.894013289005	-3.255732980492
-1.893685077902	-3.254849862378
-1.893380473749	-3.254565316094
-1.892768482202	-3.252502629762
-1.892362317704	-3.252423272624
-1.891872196269	-3.251568495884
-1.891411032119	-3.251184440613
-1.890982007647	-3.250054463809
-1.890508098649	-3.249150505139
-1.890025602796	-3.246690877587
-1.889667349733	-3.246162795929
-1.889264231833	-3.245496918405
-1.88883842862	-3.244334206229
-1.888372401844	-3.242581625824
-1.887990991691	-3.241269935576
-1.887553803654	-3.241146802637
-1.88711919677	-3.238989744087
-1.886821016578	-3.238933670743
-1.886419691441	-3.238700399855
-1.885694833835	-3.235074118846
-1.88529889586	-3.234692000171
-1.884803851539	-3.231191331016
-1.884384578818	-3.230104616267
-1.883934905809	-3.230082011915
-1.883343870213	-3.228610398571
-1.882834845794	-3.227941541079
-1.882294580655	-3.225834775076
-1.881835383944	-3.225376147382
-1.881262169959	-3.224637622515
-1.88084474045	-3.223980136608
-1.880441003262	-3.223366634266
-1.879917594668	-3.222849588997
-1.8793990823	-3.222843679951
-1.878919002988	-3.221102158193
-1.878582961537	-3.220404313001

-1.878018824777	-3.21871367605
-1.877546224963	-3.217578464643
-1.876959060054	-3.215088767919
-1.876450981234	-3.214047352147
-1.876017570254	-3.213412384882
-1.875686321165	-3.213337412386
-1.875260058595	-3.210759868075
-1.874893331364	-3.210673362783
-1.874397632133	-3.209467538412
-1.874089609156	-3.208642735628
-1.873585337586	-3.208482400548
-1.873138277004	-3.207940771418
-1.87270707665	-3.207561324525
-1.872253445977	-3.20624274897
-1.871950091022	-3.206199378894
-1.871397295144	-3.203149944116
-1.870838991941	-3.202057493398
-1.870391013861	-3.201819589888
-1.86998753458	-3.201646438315
-1.869514291757	-3.201397649324
-1.869198461175	-3.201304863826
-1.868764316949	-3.201263718795
-1.868214388472	-3.19725054745
-1.867663426318	-3.194168259365
-1.867150835684	-3.193979841158
-1.86680455541	-3.192470502114
-1.86648841867	-3.192261868812
-1.866183559635	-3.19196699248
-1.865676826133	-3.190162170341
-1.864917668877	-3.187126258349
-1.864631098465	-3.185707689181
-1.864211516315	-3.185370399633
-1.863760211478	-3.18465171084
-1.863369288761	-3.183919708862
-1.862945249914	-3.183793546285
-1.862402920633	-3.182823816392
-1.862031773687	-3.182076010941
-1.861703020753	-3.181715307945
-1.86130568459	-3.181163206415
-1.86080490338	-3.179809521313
-1.860324112489	-3.179564851917
-1.860058635996	-3.179217054448
-1.859510892192	-3.175590620266
-1.859145372257	-3.174555347587
-1.858728814785	-3.174090385998
-1.857991986476	-3.171444109997
-1.857643548026	-3.17117295351
-1.857372482159	-3.171007707012
-1.856779906611	-3.169338004628
-1.856412406734	-3.169161403812
-1.855749853137	-3.167057396551
-1.855289606469	-3.165546572567
-1.854774344995	-3.164048888552
-1.854218257881	-3.161737029503

-1.853768740126	-3.159970339608
-1.853347705891	-3.158592218216
-1.85292741835	-3.157571457302
-1.852325377786	-3.156129919248
-1.851825958938	-3.155827417458
-1.85142129979	-3.155359916029
-1.851081154525	-3.155112883869
-1.850481521429	-3.152749266453
-1.849967794236	-3.151241536315
-1.849506084334	-3.150379936235
-1.849074044378	-3.149604327202
-1.848630052363	-3.14878718057
-1.848308671806	-3.148165601178
-1.84778808278	-3.145151993212
-1.847374791773	-3.144646728202
-1.847002884961	-3.144528811477
-1.846659940985	-3.14442172422
-1.846126107172	-3.141250113954
-1.845500857512	-3.139119616091
-1.845061545242	-3.137653547609
-1.844668031816	-3.136166663628
-1.844322569482	-3.136102550451
-1.843806251818	-3.134241213845
-1.843120842953	-3.130497785127
-1.842736331626	-3.130152575187
-1.842321714164	-3.12967173557
-1.841793929238	-3.128353350063
-1.841280726049	-3.128255335812
-1.840939219544	-3.126481200133
-1.840695552844	-3.126312320611
-1.840289473939	-3.125926325057
-1.839789498663	-3.123947306823
-1.839292838325	-3.121373092155
-1.838962309739	-3.1213529425
-1.838503577025	-3.120673849754
-1.838074931337	-3.120060321239
-1.837631939265	-3.11997427584
-1.83719027204	-3.118578224592
-1.836741910611	-3.11773487455
-1.836346787473	-3.116922403059
-1.835925296891	-3.116089210058
-1.835595349049	-3.115642658266
-1.83499861963	-3.112024922686
-1.834613151439	-3.112012580416
-1.834276123993	-3.111856851897
-1.834026306299	-3.111269233074
-1.833675052584	-3.111251662243
-1.83328144999	-3.11120962918
-1.832948494726	-3.110103560005
-1.832498937567	-3.108541361028
-1.832038497786	-3.106907299788
-1.8316901027	-3.106156663793
-1.831319907782	-3.104933557579
-1.830934114457	-3.104083382679

-1.830361511535	-3.10291625195
-1.829803271965	-3.100998036653
-1.829309079386	-3.099270360775
-1.828816671232	-3.098599998142
-1.828459588095	-3.097978214116
-1.828092546869	-3.097774844468
-1.827676940834	-3.096519415388
-1.827364484412	-3.096371376411
-1.826911585495	-3.094837370983
-1.826405809587	-3.094694234091
-1.825986650983	-3.094174681097
-1.825657638082	-3.093373322708
-1.825283684126	-3.092479352843
-1.824786724755	-3.091203753179
-1.824454748366	-3.091034670653
-1.824166785061	-3.09086055142
-1.823809098028	-3.090166200892
-1.823486754765	-3.089691975602
-1.823109432437	-3.088966896257
-1.822720578032	-3.087775712562
-1.822243239331	-3.087691511517
-1.821858922111	-3.087609411976
-1.821470015324	-3.08713394375
-1.821028560941	-3.086857150724
-1.820619092084	-3.086690806069
-1.820275655146	-3.085748898206
-1.81985597403	-3.084320577368
-1.81945657464	-3.083877698877
-1.819006471471	-3.083534788718
-1.818615622047	-3.083356160524
-1.818364197626	-3.082722284692
-1.817844623873	-3.080902519923
-1.817436469091	-3.080806959755
-1.816992631749	-3.080025082472
-1.816666893065	-3.079431216736
-1.816118582509	-3.077854721764
-1.815529214922	-3.073993371585
-1.815293347857	-3.073548661756
-1.814895676595	-3.073480072129
-1.814474710663	-3.07224831039
-1.813897884515	-3.06978981826
-1.813577981414	-3.069629861548
-1.813170999871	-3.068964943534
-1.812760158522	-3.068466458393
-1.812438259511	-3.068368739885
-1.812132915519	-3.067730059471
-1.811679705458	-3.066608511038
-1.811225397957	-3.065246673132
-1.810837545089	-3.064740423053
-1.810243632179	-3.064153354005
-1.809909280327	-3.063947593713
-1.809587094613	-3.063680698311
-1.809280306906	-3.063656986165
-1.808980651018	-3.063302904305

-1.808609902288	-3.063155592677
-1.808309616968	-3.062346228896
-1.807975171805	-3.061563440718
-1.807468388968	-3.060250344536
-1.806979264812	-3.060110463153
-1.806605283273	-3.059973033909
-1.806196753314	-3.057447626876
-1.805960501691	-3.05724116343
-1.805565682456	-3.057115003866
-1.805186453896	-3.056132477894
-1.804806282697	-3.055062033458
-1.804503035526	-3.054162501049
-1.804152472657	-3.05310241144
-1.803748568016	-3.052321988651
-1.803192363936	-3.049744863485
-1.802779574683	-3.049149486395
-1.802320554449	-3.048020592506
-1.801847544314	-3.045885511641
-1.801538174632	-3.045280781671
-1.801173298564	-3.04472214148
-1.800848951429	-3.044402457471
-1.800353657366	-3.04333243551
-1.80006507754	-3.04329530541
-1.799788876385	-3.043265603132
-1.799455771921	-3.042372982014
-1.799028493911	-3.042246892243
-1.798514710525	-3.041592840992
-1.797809580548	-3.035145100216
-1.797616208991	-3.035039630119
-1.797208315522	-3.034213584892
-1.796751917686	-3.032032787142
-1.796487859703	-3.031895430725
-1.795941742048	-3.03132510354
-1.79560665275	-3.031310474225
-1.795071780298	-3.027069627869
-1.794608210807	-3.026140161194
-1.794202455629	-3.024899933875
-1.793793449675	-3.024108655487
-1.793471604971	-3.023794801647
-1.793165509858	-3.023128447807
-1.792827067927	-3.022770855554
-1.792294851412	-3.022558876788
-1.791942390727	-3.022068041349
-1.791495537154	-3.020976867686
-1.791060655679	-3.020767960288
-1.79073637352	-3.020491525552
-1.790324193582	-3.019129839557
-1.78996489636	-3.018554427342
-1.789594532195	-3.018179102102
-1.789195136867	-3.016308183592
-1.788689726146	-3.01591422156
-1.788302115509	-3.014808509132
-1.787866818331	-3.013608234691
-1.787438717049	-3.013537326391

-1.787154539663	-3.01334140334
-1.786821011117	-3.012307405178
-1.786541535386	-3.011727051221
-1.786213065753	-3.010912388904
-1.785903619495	-3.010802642888
-1.785356040256	-3.006780146913
-1.785033796096	-3.006542668742
-1.784563532118	-3.005455236015
-1.784139747758	-3.005198644362
-1.783650644221	-3.003420033583
-1.783145158443	-3.003073723667
-1.782777452248	-3.002544202248
-1.782487026953	-3.001631195691
-1.782077666865	-3.001060024048
-1.781733930473	-3.000876041647
-1.781316924092	-2.999559871345
-1.780876579883	-2.999502739742
-1.78056724971	-2.999151096157
-1.78022248269	-2.998314787884
-1.779883101772	-2.998281931627
-1.779404965779	-2.996497985526
-1.778994508981	-2.996471340082
-1.778533549687	-2.996205930288
-1.778173101029	-2.995450904465
-1.777843071065	-2.993966203406
-1.777486251248	-2.99348979065
-1.777129394829	-2.993470114413
-1.776799125855	-2.993465699206
-1.776552052334	-2.993184998345
-1.776221865007	-2.991742969567
-1.775776625581	-2.99074145022
-1.775429321598	-2.990649863105
-1.775059864285	-2.990200650083
-1.774732969082	-2.988593124505
-1.774364553354	-2.98817320702
-1.774030540534	-2.987819014166
-1.773695191929	-2.987298781516
-1.773399300745	-2.98672223704
-1.773104013807	-2.985399369848
-1.772742328446	-2.985252121992
-1.772474122883	-2.984971412585
-1.772182052443	-2.984829302567
-1.771930141126	-2.984458391713
-1.771501021355	-2.983434830997
-1.77106768921	-2.982731338202
-1.770656876309	-2.981885823739
-1.770050003295	-2.977102258776
-1.769656983123	-2.975871285979
-1.769222356124	-2.975415031524
-1.768895942973	-2.973995795298
-1.768474153985	-2.97340551731
-1.768071936346	-2.973120968557
-1.76777414523	-2.972540061976
-1.767473461065	-2.971890725235

-1.767059452625	-2.970980876094
-1.766827136629	-2.970707154346
-1.76649795305	-2.97035589732
-1.766037337309	-2.968567922843
-1.765619321265	-2.967500176018
-1.765259895723	-2.966941007783
-1.764713640977	-2.96679843641
-1.764361993356	-2.966096895561
-1.764036066762	-2.965984596643
-1.763643195042	-2.96439452835
-1.763283614041	-2.963814825794
-1.762897969884	-2.962627684319
-1.762432011018	-2.961268455174
-1.762034817176	-2.960237401984
-1.761631317962	-2.959037793956
-1.761245939788	-2.957123430608
-1.760977104321	-2.956403564774
-1.760449791642	-2.954489018548
-1.760130359533	-2.953999258401
-1.759700911555	-2.950782268087
-1.759407361334	-2.950698068576
-1.758991626762	-2.949478074075
-1.758472208853	-2.947141692128
-1.758167593748	-2.946620610865
-1.757851387102	-2.944667210329
-1.757383328849	-2.941519169775
-1.75697078499	-2.940444061974
-1.756649264397	-2.93983754591
-1.756112151858	-2.93655954928
-1.755662073115	-2.936117693132
-1.755343673251	-2.935935006604
-1.754960440237	-2.93462930943
-1.754635365203	-2.934412004031
-1.754294563354	-2.933263295249
-1.753783916186	-2.9318627697
-1.75346622815	-2.931262009214
-1.753155696786	-2.93113689724
-1.752829614859	-2.930234673019
-1.752393276211	-2.929156614756
-1.752005276759	-2.927544481341
-1.751509027824	-2.926494277495
-1.751092840621	-2.924560994341
-1.750617667125	-2.92369594399
-1.750333857579	-2.92288006853
-1.749965568729	-2.922599304458
-1.749678680895	-2.922035378544
-1.749229266993	-2.920210164447
-1.748959737853	-2.920126450733
-1.748532455085	-2.918363430407
-1.748039236367	-2.91673094851
-1.747636733578	-2.916679585483
-1.747300742233	-2.915171443256
-1.746896047604	-2.914830779388
-1.746618711982	-2.914695450067

-1.746085729682	-2.911999920605
-1.745830836888	-2.911307662067
-1.7455261146	-2.910145342048
-1.745129957129	-2.908275638929
-1.744864220928	-2.907833323334
-1.744540655863	-2.907105718805
-1.74406978184	-2.905249364389
-1.74366770975	-2.904330436234
-1.743320300062	-2.903556690486
-1.742996009399	-2.903191997392
-1.742635747924	-2.902508611188
-1.742326641463	-2.90156762281
-1.741936024559	-2.900055390099
-1.741503765011	-2.899344285854
-1.741265667483	-2.899183488887
-1.740963422517	-2.89910310938
-1.740512131892	-2.896440174516
-1.740204529859	-2.896000881761
-1.739880429803	-2.895819298347
-1.739574831728	-2.895481165077
-1.739140862802	-2.893459068514
-1.738820814172	-2.893408698544
-1.738473964314	-2.89281214388
-1.738141230118	-2.892126203092
-1.737757034661	-2.890370436692
-1.73734534608	-2.889312673487
-1.736943226856	-2.88904948281
-1.736662843191	-2.888289625672
-1.736318853796	-2.887825082196
-1.735961357748	-2.887492345251
-1.735577048893	-2.886155968292
-1.735305825095	-2.885717928714
-1.734990507098	-2.885137730672
-1.734560970176	-2.883180615733
-1.734182083595	-2.881313343421
-1.733891506392	-2.880921454037
-1.733491664193	-2.879498712999
-1.733192978527	-2.878986172786
-1.732832685512	-2.878042966533
-1.732415628585	-2.876865284489
-1.732011449379	-2.87640031354
-1.731644122874	-2.876083967784
-1.731297848218	-2.87581218226
-1.730831681065	-2.875631373648
-1.730524050261	-2.875484766243
-1.730272290331	-2.875356984243
-1.729946476188	-2.87495463626
-1.729661660147	-2.87417149744
-1.729218572151	-2.873861653725
-1.7289279307	-2.873740187622
-1.728552652551	-2.873543084917
-1.72827099767	-2.873285889414
-1.727945733456	-2.873248886217
-1.727623151429	-2.872523084819

-1.727134321166	-2.871537815283
-1.726727127953	-2.87119015712
-1.726383137838	-2.87110218306
-1.726063632243	-2.870738235009
-1.725506056232	-2.869768504073
-1.725230646479	-2.868272667418
-1.724722188789	-2.866575532373
-1.724366525322	-2.866095810093
-1.724054041738	-2.864382863998
-1.723708141495	-2.864366319064
-1.72335879124	-2.863967032891
-1.722967142321	-2.863758455431
-1.722538708119	-2.862907208996
-1.72199727637	-2.86121933304
-1.721673502284	-2.860827375648
-1.721184332811	-2.858680174828
-1.720820432189	-2.857800624436
-1.720413948304	-2.857323023973
-1.720103528648	-2.856920794375
-1.719616988731	-2.855096854589
-1.719191087152	-2.852611451975
-1.718947251106	-2.851903860662
-1.718682617147	-2.851666765244
-1.718305875147	-2.851235693092
-1.717965212145	-2.849884005948
-1.717651663007	-2.849470964616
-1.717264130001	-2.84795743445
-1.716924451226	-2.847320651921
-1.716617179037	-2.847230490247
-1.71622849548	-2.846368205553
-1.715890766393	-2.846190062424
-1.715667890251	-2.846133253999
-1.715374252813	-2.845749011387
-1.715070592845	-2.845155321612
-1.714725080561	-2.844590882756
-1.714500945984	-2.844434652388
-1.713881501633	-2.843226083587
-1.713399751491	-2.843014943559
-1.712844937191	-2.839367080045
-1.712551813378	-2.838968050535
-1.712235524474	-2.838440292617
-1.711690137944	-2.83806993245
-1.711357728678	-2.836915151615
-1.711009767429	-2.836662133006
-1.710809872546	-2.836364107278
-1.710337198028	-2.836345671225
-1.710059395728	-2.836061494882
-1.709675061099	-2.836056442365
-1.70922442291	-2.834477756172
-1.708855061021	-2.834477579825
-1.708331904099	-2.833366378547
-1.707795787431	-2.83178851319
-1.707514440507	-2.831470472608
-1.707208788132	-2.830745827993

-1.706683977959	-2.828513436968
-1.706499450853	-2.828163164436
-1.706168872033	-2.827971251595
-1.705798675574	-2.826817966785
-1.705471581132	-2.826564899115
-1.705152779564	-2.826524310501
-1.704650671765	-2.825064353699
-1.704387408268	-2.825027028708
-1.703915537454	-2.82457639265
-1.703571021031	-2.824532374046
-1.703276040961	-2.824015922312
-1.70283315384	-2.82219442286
-1.702445757863	-2.82032715037
-1.701932097	-2.818471416376
-1.701568037177	-2.818260906331
-1.701225544088	-2.818023023313
-1.700893471711	-2.817136171686
-1.700537799675	-2.817064978596
-1.700116039198	-2.814103552267
-1.699752119221	-2.812651676136
-1.699497689844	-2.812546850911
-1.699107268031	-2.810375040101
-1.698778058535	-2.808695694596
-1.698460872546	-2.808648364209
-1.698167213119	-2.808410558645
-1.697909747362	-2.808316687615
-1.69748382488	-2.808288516478
-1.697233354037	-2.808181785418
-1.696752764888	-2.807775106787
-1.696435049497	-2.807750574674
-1.696215548893	-2.807456407983
-1.695813673289	-2.806917583177
-1.695352228193	-2.806092004201
-1.694850591775	-2.803413816938
-1.694553493873	-2.802906622483
-1.694198247847	-2.80159057739
-1.693828969083	-2.801070338265
-1.69352651755	-2.80106515295
-1.693125372459	-2.800646491609
-1.692802249537	-2.799811649305
-1.692473859909	-2.799498991855
-1.692104587506	-2.799476064054
-1.691732826275	-2.798641363024
-1.691435465344	-2.798633813258
-1.691025740847	-2.796261498127
-1.690550868968	-2.795442848485
-1.690164410181	-2.793167833324
-1.689841371534	-2.793080796403
-1.68951333152	-2.792963235912
-1.689058868865	-2.792210000589
-1.688613001516	-2.791326794456
-1.68837013872	-2.790793113028
-1.687987494235	-2.789749137987
-1.687747146768	-2.789572770085

-1.687361062443	-2.789207371655
-1.687075248197	-2.789187645502
-1.686831786284	-2.789095303339
-1.68647411656	-2.788802561704
-1.686245367616	-2.78830533017
-1.686012398383	-2.78770483639
-1.68574861655	-2.78755633383
-1.685427367827	-2.785969717905
-1.685128825461	-2.785722126734
-1.684798667874	-2.785695676216
-1.684441344541	-2.785200158198
-1.684218351743	-2.78510024551
-1.683831082361	-2.784835581358
-1.683436113033	-2.783243019486
-1.683103295966	-2.78306767162
-1.682792952333	-2.782962381641
-1.682446939803	-2.781443667858
-1.681900370548	-2.781211022486
-1.681531008123	-2.780815242336
-1.68133713736	-2.780392124135
-1.680986709959	-2.780011354786
-1.680745094406	-2.780008101009
-1.680457355243	-2.779262170601
-1.680156636274	-2.778572345862
-1.679791022984	-2.778366780123
-1.679418499507	-2.776939679351
-1.679152603898	-2.776916705659
-1.678855284102	-2.776297783168
-1.678554559753	-2.775623399861
-1.678255576368	-2.774911047834
-1.677798669338	-2.774455804072
-1.677431529614	-2.773157406219
-1.677084091122	-2.772559495459
-1.67683184808	-2.77213500996
-1.676460199108	-2.771984938694
-1.676103796222	-2.771448239921
-1.675575869957	-2.76933861828
-1.675221625326	-2.769312891575
-1.674898089232	-2.769293395236
-1.674504125836	-2.76760138837
-1.674155987688	-2.76666561508
-1.673887189304	-2.765785910236
-1.67322172196	-2.762159020499
-1.672958521305	-2.761885546025
-1.67247389621	-2.761499333956
-1.672121238561	-2.761176728159
-1.671691835885	-2.760991337175
-1.671350662153	-2.760541746291
-1.670967318881	-2.760327605227
-1.67071609823	-2.760313799436
-1.67034708655	-2.760179975162
-1.670000271367	-2.759669082832
-1.669687863814	-2.759505407718
-1.669324532439	-2.758860057347

-1.668996610029	-2.75752441284
-1.668653564937	-2.755983502472
-1.668364977913	-2.755439692026
-1.667894636482	-2.752530271327
-1.667451779768	-2.752370508688
-1.667159754674	-2.752251439579
-1.666874709096	-2.7518347982
-1.666572654027	-2.750933166347
-1.666250024679	-2.749011071662
-1.666038353231	-2.748765965675
-1.665679620216	-2.74853690616
-1.665320160975	-2.747870018398
-1.665012863811	-2.747555025342
-1.664722984438	-2.746497769623
-1.664438539632	-2.746457936512
-1.664177126444	-2.745579543695
-1.663901787226	-2.743695591113
-1.663560657921	-2.742972761757
-1.663337225109	-2.74242010466
-1.66302766772	-2.742073816082
-1.662784722758	-2.741623484778
-1.662460104392	-2.741205915881
-1.662077184007	-2.740707556321
-1.661726350063	-2.739596207061
-1.661365081858	-2.738930958923
-1.660947070391	-2.737817968443
-1.660783074552	-2.737517672034
-1.660436185365	-2.73596009038
-1.660114270912	-2.733848569392
-1.659632707467	-2.73273575626
-1.65926617391	-2.730958425988
-1.658937820987	-2.730835042109
-1.658618339083	-2.730474463451
-1.658271373951	-2.729320213895
-1.657908795007	-2.728509867931
-1.657676747993	-2.727946636701
-1.657463684315	-2.727560217361
-1.657227771708	-2.727003432053
-1.656968471954	-2.726906386376
-1.656672802434	-2.726303996414
-1.656342496171	-2.725892240717
-1.655761104692	-2.720434671219
-1.655415644532	-2.720244034169
-1.655124981945	-2.719958819486
-1.654978776253	-2.719917268277
-1.654581506755	-2.71923742168
-1.654188319487	-2.719106330454
-1.653927019419	-2.718417513432
-1.653560209164	-2.718097723646
-1.653225604946	-2.717114618331
-1.652889228605	-2.717004337182
-1.65255523093	-2.716104859218
-1.652229739839	-2.715928472946
-1.651907685304	-2.715001965842

-1.651614941747	-2.714095888051
-1.651296291997	-2.713110282859
-1.651037600293	-2.71256395894
-1.650621026471	-2.711331234362
-1.650399451054	-2.711167611151
-1.649958881353	-2.710561428428
-1.64959261592	-2.70964154881
-1.649298782838	-2.708812816548
-1.649027354214	-2.708323989144
-1.648696406725	-2.707995674857
-1.648423208196	-2.707858568571
-1.648194665822	-2.707042932627
-1.64784362188	-2.705612336663
-1.647554332577	-2.705126537659
-1.647212462529	-2.704922618793
-1.646926086547	-2.704897367629
-1.64649374453	-2.703528229989
-1.646164557871	-2.703016949226
-1.645923073336	-2.703003875686
-1.645703809093	-2.702865110458
-1.645408755251	-2.702235459069
-1.645134328753	-2.701853588095
-1.6446869774	-2.700600451142
-1.644323204742	-2.699635586347
-1.643949004857	-2.699357061173
-1.643594374203	-2.698636903048
-1.643346930225	-2.698342570652
-1.642908111494	-2.698107920407
-1.642578334332	-2.696932202675
-1.642323764272	-2.69675408553
-1.641981368756	-2.695395852392
-1.641618145309	-2.695287589914
-1.641364268218	-2.695160862893
-1.641076324875	-2.694417047218
-1.640756582502	-2.69438344467
-1.640476208196	-2.693727675198
-1.640041691285	-2.692865994763
-1.639656686169	-2.691609584079
-1.639300438256	-2.690748435985
-1.639007996383	-2.689965356701
-1.63870181345	-2.68985960158
-1.63825315634	-2.689568171384
-1.637890330937	-2.688642266643
-1.637604418047	-2.688218851252
-1.637254397168	-2.688050863306
-1.636747431652	-2.687870153056
-1.636481632605	-2.687797149337
-1.636024911067	-2.686865397376
-1.63563180536	-2.686010036815
-1.635418466649	-2.685866242691
-1.635002202021	-2.684482578294
-1.634745525449	-2.684458683247
-1.634429148369	-2.683926189094
-1.634112078437	-2.683450108503

-1.633868973115	-2.683443882245
-1.633635945894	-2.683411101079
-1.633314191353	-2.682721567217
-1.633058847153	-2.682635297353
-1.632745493755	-2.681980424013
-1.632455809144	-2.681518261537
-1.63219581556	-2.681099404582
-1.631849171562	-2.68010184525
-1.631422580888	-2.678296021979
-1.631203012548	-2.678121682287
-1.630887985215	-2.677967233104
-1.630577119146	-2.677921826436
-1.630183873027	-2.677121887733
-1.629779732943	-2.675449222889
-1.629428681829	-2.67543797434
-1.629118741697	-2.674938592063
-1.628774114928	-2.673739294957
-1.628460172676	-2.673300892333
-1.628164492686	-2.672731726798
-1.627911033628	-2.672399152135
-1.627660591554	-2.671537118587
-1.62736483885	-2.671377262066
-1.626942835924	-2.670800044768
-1.626641648737	-2.67062421652
-1.62632917364	-2.670476856852
-1.626045748909	-2.669949285285
-1.625726747478	-2.66837864504
-1.625492968966	-2.667987405414
-1.625180734624	-2.667700723088
-1.624837008342	-2.667530753294
-1.624514976475	-2.667330735914
-1.624147620091	-2.667060003121
-1.623749084341	-2.666760571544
-1.62337286063	-2.666479736906
-1.623012499678	-2.666233678031
-1.622671184976	-2.665880377106
-1.622349886131	-2.66515247235
-1.622090858373	-2.66353792346
-1.621663788152	-2.661743610367
-1.621411342542	-2.661416455643
-1.621092728008	-2.66001334238
-1.620803870484	-2.659898931685
-1.620466955722	-2.658278550054
-1.620168667562	-2.657983332329
-1.619761581693	-2.657686007313
-1.61940699325	-2.656810206523
-1.619069382837	-2.65635094747
-1.618668342833	-2.655560762064
-1.618200015644	-2.653504422258
-1.61789149256	-2.65346424959
-1.61750827407	-2.653051626241
-1.617198318836	-2.652624761527
-1.616867835026	-2.652598699129
-1.616565533753	-2.652240600547

-1.616074026859	-2.64890892649
-1.615706787312	-2.64777866156
-1.615477945843	-2.647046603972
-1.615039769372	-2.646541675764
-1.614661242029	-2.645968556218
-1.614276639121	-2.645053801718
-1.613856525826	-2.644067292754
-1.613582915804	-2.644056191495
-1.613212252633	-2.643667851394
-1.61288229537	-2.643421130155
-1.612619356777	-2.642796706937
-1.612284176275	-2.642357076832
-1.611928536025	-2.642174629895
-1.611739644009	-2.641923657059
-1.611467249273	-2.641634724928
-1.611108693986	-2.641089140173
-1.610812152047	-2.640949302277
-1.610410406966	-2.639462534494
-1.610056735735	-2.638608019095
-1.609776645866	-2.637894743154
-1.609495268163	-2.637806337112
-1.609208079028	-2.637270176586
-1.608823250973	-2.636650459095
-1.608557719082	-2.636472536075
-1.608253419631	-2.636144719567
-1.607838338343	-2.635431948162
-1.607524760549	-2.63435022392
-1.607297732863	-2.634236628603
-1.607016631311	-2.63417715771
-1.606676022825	-2.633608389475
-1.606457748379	-2.63360445474
-1.606225237689	-2.633272210917
-1.605882183333	-2.633147547396
-1.605632340866	-2.633141075968
-1.605378992143	-2.631426919144
-1.605031590909	-2.630889797966
-1.604776298551	-2.630764087081
-1.604472162518	-2.630737413436
-1.604137113869	-2.629334626982
-1.603890759243	-2.628796634862
-1.603678897063	-2.628622774097
-1.60325557325	-2.627744077408
-1.602840849494	-2.627359695048
-1.602621674913	-2.62722354632
-1.602369184511	-2.627064337803
-1.602155667075	-2.626839903179
-1.601840426353	-2.625858640509
-1.601563576018	-2.625089485689
-1.601314343053	-2.625063059891
-1.600983298948	-2.62447650831
-1.600508019555	-2.622522542061
-1.600263811595	-2.621811542654
-1.600001784346	-2.621770422143
-1.599619848902	-2.621261864304

-1.599112823557	-2.61904415631
-1.59883667665	-2.618439211324
-1.598495291882	-2.617452831695
-1.598211636263	-2.616974164385
-1.597938141906	-2.616072552151
-1.597629729956	-2.615759204443
-1.597403286452	-2.615494314557
-1.596958302442	-2.613854270576
-1.596696958275	-2.613751951408
-1.596296273442	-2.613416408832
-1.595987889416	-2.613159414346
-1.595638313539	-2.611821404922
-1.595287063398	-2.611738567992
-1.59507172077	-2.611424490778
-1.594856638787	-2.61111887641
-1.594498420786	-2.609851428253
-1.594182079985	-2.609380452449
-1.593896253955	-2.608982991896
-1.593527423699	-2.608053178666
-1.593240165812	-2.607276824951
-1.592939391826	-2.607194032933
-1.592708303594	-2.606844719294
-1.592478185879	-2.606731270275
-1.592116614789	-2.606692344359
-1.591730775867	-2.604835668073
-1.59134714389	-2.602585901191
-1.591038284043	-2.60229946551
-1.590656974878	-2.601895889901
-1.590368840882	-2.60163731845
-1.590058396745	-2.601632993387
-1.589674616577	-2.601211957362
-1.58944845199	-2.600793565747
-1.589219689886	-2.600700213841
-1.588855544699	-2.600074316526
-1.588525127786	-2.598370657803
-1.588175482041	-2.597396874702
-1.587875244146	-2.596518934596
-1.587691903377	-2.596266222272
-1.587260396216	-2.594993630261
-1.587025480584	-2.594091603641
-1.586785913252	-2.593612375609
-1.586550441626	-2.593571415027
-1.586221367238	-2.592981706656
-1.585826498898	-2.59240419817
-1.585512217272	-2.590836415939
-1.585158561587	-2.589597526281
-1.584882656189	-2.589556522705
-1.5846017997	-2.589421889999
-1.584301129695	-2.588545882746
-1.58406664634	-2.58849398017
-1.58377023979	-2.588260480771
-1.583426564544	-2.587893500652
-1.58317525372	-2.58748016326
-1.582842174629	-2.586623121593

-1.582509433435	-2.585533843218
-1.58228285595	-2.58520589828
-1.58194622978	-2.584042071367
-1.581626742172	-2.58219163948
-1.581384479998	-2.582075081016
-1.581013291734	-2.581097468531
-1.580725215008	-2.581021552473
-1.580360206052	-2.580755644824
-1.58012367249	-2.580375733262
-1.579869110123	-2.579714532922
-1.579568967349	-2.579347985886
-1.579192203818	-2.578090770597
-1.578925956312	-2.577399757757
-1.578606958661	-2.576874718524
-1.578376366931	-2.576451669141
-1.577920912009	-2.575633276989
-1.577674482206	-2.575463645864
-1.577345858994	-2.574283919994
-1.577062274157	-2.573410903926
-1.576829153754	-2.573111438001
-1.576533347703	-2.572859531645
-1.576228489365	-2.572372618836
-1.575950009558	-2.572271248417
-1.575738828715	-2.572237876055
-1.575396082013	-2.571828341229
-1.57510887115	-2.571761054466
-1.574842917256	-2.57079576592
-1.574481303573	-2.570153454742
-1.574250552256	-2.569405518127
-1.573898802716	-2.568975064473
-1.573503355192	-2.567859981218
-1.573220223851	-2.56781404098
-1.572947788036	-2.567304590881
-1.572605969189	-2.565104841156
-1.572225309274	-2.564633878134
-1.57194042585	-2.564491261282
-1.571446711598	-2.561954599823
-1.571121031275	-2.561264815613
-1.570916172013	-2.560882412808
-1.570549079752	-2.560794915462
-1.570163438017	-2.56020979682
-1.569850473105	-2.560177742885
-1.56964078951	-2.559184554258
-1.569256827759	-2.558792693528
-1.568897707097	-2.557822235021
-1.568561384854	-2.557795533868
-1.568184189061	-2.557310861579
-1.567857047951	-2.557069539238
-1.567656088932	-2.556826256753
-1.56730221925	-2.556646020549
-1.567078716164	-2.556566645448
-1.566785095198	-2.555086869784
-1.566553444934	-2.554536902957
-1.566214127861	-2.553767554737

-1.565940767872	-2.553491959354
-1.565639901889	-2.552885610338
-1.565344381366	-2.551771422508
-1.565092374479	-2.551462953271
-1.564796523096	-2.551356552576
-1.564475730544	-2.551047758949
-1.56424522634	-2.550306542242
-1.56400460427	-2.549720436725
-1.563789133753	-2.54964670985
-1.563538677137	-2.547909831414
-1.563175974982	-2.54780582143
-1.562828390979	-2.545429068759
-1.562498204346	-2.544710010047
-1.562198880072	-2.544206364444
-1.561962195012	-2.544003510954
-1.561668142506	-2.543448580339
-1.561464236251	-2.543218618671
-1.561189751274	-2.542594361499
-1.560910843117	-2.542176434494
-1.560697797794	-2.541845307346
-1.560450004037	-2.541769229768
-1.560174283238	-2.541379333742
-1.559863903229	-2.54131738075
-1.559558996716	-2.540665638792
-1.559271891418	-2.540564984236
-1.559074260322	-2.540333121666
-1.558790755325	-2.539928653249
-1.558429712465	-2.539493675832
-1.5579959882	-2.538868646085
-1.557679401562	-2.538162063767
-1.557425048438	-2.53761870441
-1.557031244676	-2.536322350139
-1.556726641617	-2.53587024025
-1.556452534282	-2.535174665131
-1.556157839979	-2.534874730413
-1.555853222823	-2.533758039194
-1.555375563931	-2.532012613237
-1.555033699973	-2.530736901446
-1.554724346851	-2.529671190878
-1.554489375002	-2.52958985336
-1.55415924865	-2.528332998983
-1.553791883627	-2.528314760833
-1.553384596853	-2.528268133413
-1.553132071783	-2.527665064489
-1.55289792139	-2.527597723164
-1.552674090704	-2.527369554399
-1.552418805015	-2.527289673
-1.552006999473	-2.527073369803
-1.551704678062	-2.526923293068
-1.551329461513	-2.526303307629
-1.551010664268	-2.525712028469
-1.550779150249	-2.525542195943
-1.550371804603	-2.524693805719
-1.550141670727	-2.524641490031

-1.549842815185	-2.524601964373
-1.54959663998	-2.524418928397
-1.549328016772	-2.523755474326
-1.549071588004	-2.523325991865
-1.548858143639	-2.522895620566
-1.548613173452	-2.522420062772
-1.548364671156	-2.521696731881
-1.548061302214	-2.521574224832
-1.547810417695	-2.520223120984
-1.547448003432	-2.519960579464
-1.547120888017	-2.519929018254
-1.546772203832	-2.51924525302
-1.546542452198	-2.518786566187
-1.546214811737	-2.517771983602
-1.545979858436	-2.51704320475
-1.54562947569	-2.516275726574
-1.545272107476	-2.515180022368
-1.545054882922	-2.514828696385
-1.544745972983	-2.514508294315
-1.544328567542	-2.514356083818
-1.544051542718	-2.513959913964
-1.543688924122	-2.512615862306
-1.54334557863	-2.512145794429
-1.543059695985	-2.511315406694
-1.542787128462	-2.511133230251
-1.542479213816	-2.510066616808
-1.542063406033	-2.508515046317
-1.541743165537	-2.50750724815
-1.541455163297	-2.507359970241
-1.541069654602	-2.507143009137
-1.540881379214	-2.507131900174
-1.540643894587	-2.506605764514
-1.540261262065	-2.506331145591
-1.539984230827	-2.506262696887
-1.539651289231	-2.504115948231
-1.539471948118	-2.50379461593
-1.539157493857	-2.502425631324
-1.538883302896	-2.502238209346
-1.538613839451	-2.501696272586
-1.538262086799	-2.501625139579
-1.538063726019	-2.50160816462
-1.537722824644	-2.499625521997
-1.537412192957	-2.497378534908
-1.537225569927	-2.496892104867
-1.537034718635	-2.495969003275
-1.53668139742	-2.493794636511
-1.536448641205	-2.493367364303
-1.536121140885	-2.492344657159
-1.535906451675	-2.492324970648
-1.535618052395	-2.491904878793
-1.535321605574	-2.491896953882
-1.534919718667	-2.491513723413
-1.534678418893	-2.491401291337
-1.534453100927	-2.491136655443

-1.534177067671	-2.490891966954
-1.533823828832	-2.488879456272
-1.5335147514	-2.488302632881
-1.533282813926	-2.487861389126
-1.533020697459	-2.487496171591
-1.532881532069	-2.48748579677
-1.532554429243	-2.486633315547
-1.532263963175	-2.486110528753
-1.531947707618	-2.486048662101
-1.531710696766	-2.484416086797
-1.531479836688	-2.483781271927
-1.53124540833	-2.483587125906
-1.530965949811	-2.48320917475
-1.530736426868	-2.483086993605
-1.530423327329	-2.482781031676
-1.530034332182	-2.48250388234
-1.529805314372	-2.481604049094
-1.529473419526	-2.481546675047
-1.5291524042	-2.481357749335
-1.528894579193	-2.480906510459
-1.528730612544	-2.480808136053
-1.528467096396	-2.480669364267
-1.528195435674	-2.480163829417
-1.527904539541	-2.479717855461
-1.527628368677	-2.479438238332
-1.527394871937	-2.479340309567
-1.527116274947	-2.479162252897
-1.526809328116	-2.477744371678
-1.526364463699	-2.477658065617
-1.526001354371	-2.476809749613
-1.525775825701	-2.476373683018
-1.525545796978	-2.476233279485
-1.525299008888	-2.47576857285
-1.524984238432	-2.4757265143
-1.524705008354	-2.475069055961
-1.524483958895	-2.474968448562
-1.524255692017	-2.474929050726
-1.523881807694	-2.473676103766
-1.523707902146	-2.473562001487
-1.523393904851	-2.472520572555
-1.52313233843	-2.472215401474
-1.522729352311	-2.470386379877
-1.522507857617	-2.46989259513
-1.522270433772	-2.469634528597
-1.52200508972	-2.46952080283
-1.521750785759	-2.468939493515
-1.521530663431	-2.468485499182
-1.521179534562	-2.468056780436
-1.520946869344	-2.468004985011
-1.520691722247	-2.467316007581
-1.520330584821	-2.467055116501
-1.519967077163	-2.46641607859
-1.519652825383	-2.46612630071
-1.519365744082	-2.464913760764

-1.519015688082	-2.464235059407
-1.51875581024	-2.463750587977
-1.518525755132	-2.463380298245
-1.518260365392	-2.463013356296
-1.518038820805	-2.462069718653
-1.517787907293	-2.461673478435
-1.517576682126	-2.461664280939
-1.517300991222	-2.460867484485
-1.516936343536	-2.4605228064
-1.516719723617	-2.460462640325
-1.516473367845	-2.460307767107
-1.516178884292	-2.45988127982
-1.515898594923	-2.459711511451
-1.515647752826	-2.459429605837
-1.515370262742	-2.459279440676
-1.515092910312	-2.458811557172
-1.514778997656	-2.458244156059
-1.514496339008	-2.457956772673
-1.514240593531	-2.457816772072
-1.514071287118	-2.457787628495
-1.513805388845	-2.456538563742
-1.513539035036	-2.456078059256
-1.51320217856	-2.455669040769
-1.5129644712	-2.455589777323
-1.512733414164	-2.45532831432
-1.512474514513	-2.455260778502
-1.512271224032	-2.454835458355
-1.511997717565	-2.454658557412
-1.51165440296	-2.45421742282
-1.511402602452	-2.453675438388
-1.511104445464	-2.452814676209
-1.510910556725	-2.452710862957
-1.510632891198	-2.452649514439
-1.510419438179	-2.452567162229
-1.510056811903	-2.451723117217
-1.509793430753	-2.45160827058
-1.509361193946	-2.450088681272
-1.509064165262	-2.449079517939
-1.508743995081	-2.447504596827
-1.508399330626	-2.446509111058
-1.508177707289	-2.446501306333
-1.507940904475	-2.446273865122
-1.507570714114	-2.444752528389
-1.507363976335	-2.444046337801
-1.50707828242	-2.443698621382
-1.50690125674	-2.443494677597
-1.506640336938	-2.44345820801
-1.506456822449	-2.443319185752
-1.506021268164	-2.440209136797
-1.505713461937	-2.440013475903
-1.505519448164	-2.439888743294
-1.505311578089	-2.439563422075
-1.504993962783	-2.43939471192
-1.504807308512	-2.43909967629

-1.504581783823	-2.438830467516
-1.504268896446	-2.438783030741
-1.50395701381	-2.438685405988
-1.503680852073	-2.437351828343
-1.503450228168	-2.436694089102
-1.503057809595	-2.436327758249
-1.502765521143	-2.436302146791
-1.502362479026	-2.434933756902
-1.50207857748	-2.43488134157
-1.50182186292	-2.434712608888
-1.501645627263	-2.434660475999
-1.501408765131	-2.434128258007
-1.501116632532	-2.433609084656
-1.500934035515	-2.433201133993
-1.500642904734	-2.432064477619
-1.500362040275	-2.431617966573
-1.500076009996	-2.431601613533
-1.499857585704	-2.431265719548
-1.499710031751	-2.431025996889
-1.499503374239	-2.430856615296
-1.499243518077	-2.430592204411
-1.499088639176	-2.43039925893
-1.498727253582	-2.430059902148
-1.498316858042	-2.427134579559
-1.498051552688	-2.426838917701
-1.497701942764	-2.425500030335
-1.497478523181	-2.425172163002
-1.497257176911	-2.424936471752
-1.496955592159	-2.424654184316
-1.49662803873	-2.423679201605
-1.496281549659	-2.421818487464
-1.4960592717	-2.421818046595
-1.495811336525	-2.421168031947
-1.495517993649	-2.420438793315
-1.495295928934	-2.42011008474
-1.495076595001	-2.419877126315
-1.494696171261	-2.418472354232
-1.49448951519	-2.418237292267
-1.494319266053	-2.418160122867
-1.494056044522	-2.418093994312
-1.493774987957	-2.41806716701
-1.493516203101	-2.418022845039
-1.493280798668	-2.417212929995
-1.492917182307	-2.415179219257
-1.492703099184	-2.414441854621
-1.492462185436	-2.414358850714
-1.492159148259	-2.413006455355
-1.491890643309	-2.412132411052
-1.491635096505	-2.411971203973
-1.491391705466	-2.411246471753
-1.491115914205	-2.411227664499
-1.490775552487	-2.41070459909
-1.490396651745	-2.408595732898
-1.49013294394	-2.40788886295

-1.489859345338	-2.407593840887
-1.489546357412	-2.40676547191
-1.489352576916	-2.406751048045
-1.489083306426	-2.405939422175
-1.488821397713	-2.405759102754
-1.488552994079	-2.405638009349
-1.488303030048	-2.405476176047
-1.488025454424	-2.405475810837
-1.487702579379	-2.404283822015
-1.487375751907	-2.403082007916
-1.487107011608	-2.402668975357
-1.486811597088	-2.402360922593
-1.48660916594	-2.402302719311
-1.486376895425	-2.401941455789
-1.486138587614	-2.401311361127
-1.485943489958	-2.401208349289
-1.485689665495	-2.400444719584
-1.485443606312	-2.400195506218
-1.485116256142	-2.399493481223
-1.484855134512	-2.398695196498
-1.484632656165	-2.398526791355
-1.484329988798	-2.398406238962
-1.483927498671	-2.397698103951
-1.4837028925	-2.397536759691
-1.483412868707	-2.397016148262
-1.483118261501	-2.396770875066
-1.482853729275	-2.39664265291
-1.482613164042	-2.396343319305
-1.482276399383	-2.395300428811
-1.481978937735	-2.395087903684
-1.481756097574	-2.394893416482
-1.481402203698	-2.394085835698
-1.481029242335	-2.392707598993
-1.480822327529	-2.392680027284
-1.480476666837	-2.39160049584
-1.480200591334	-2.391235708469
-1.479932024761	-2.389517326527
-1.479556125179	-2.387693535735
-1.479311203194	-2.38727266626
-1.479107621425	-2.386769892613
-1.478803638182	-2.386765761316
-1.47856053679	-2.386552344189
-1.478268170135	-2.385843673878
-1.478036164254	-2.385585253229
-1.477762484454	-2.385299018159
-1.477469486615	-2.385295592082
-1.477257109407	-2.385248285434
-1.477038432874	-2.385207872946
-1.476792855274	-2.385015883478
-1.476554529155	-2.384783878329
-1.476324852833	-2.384121425188
-1.476027652838	-2.383573910694
-1.475570441054	-2.382174376643
-1.475296929612	-2.381918256891

-1.474950319668	-2.381738966063
-1.474667304442	-2.381527001197
-1.474362654244	-2.381351336738
-1.474075332155	-2.379620969696
-1.473788228569	-2.379371178702
-1.473453419553	-2.379260539073
-1.473197386834	-2.378685118604
-1.472985275598	-2.378228352481
-1.472691427856	-2.3781916121
-1.472450041846	-2.378155534202
-1.47213494627	-2.376746064231
-1.471891223516	-2.376260807165
-1.471625280533	-2.375862264363
-1.471338046524	-2.375828039973
-1.471085605627	-2.375612718652
-1.47086181641	-2.375082074385
-1.470549509831	-2.373896352563
-1.470273088871	-2.373450951119
-1.469949789254	-2.372994976604
-1.469693637228	-2.37216043227
-1.469235100299	-2.371960354065
-1.468972545701	-2.371182286277
-1.468718697775	-2.371008008483
-1.468506516935	-2.370977893517
-1.468205014773	-2.369903683391
-1.467912071631	-2.36990090186
-1.467594976521	-2.369454383876
-1.467275782585	-2.369298091429
-1.466957144499	-2.36863192795
-1.466784188863	-2.368371608506
-1.466505697437	-2.367762228467
-1.466202340762	-2.367390968836
-1.465884854697	-2.366053184311
-1.465703105235	-2.365859818586
-1.46541616163	-2.3651752507
-1.465206729253	-2.364721125789
-1.465012993708	-2.364289844856
-1.464810313239	-2.364051226943
-1.464535133694	-2.363935001193
-1.464223720056	-2.363543557223
-1.463980036705	-2.363314088478
-1.463628554661	-2.363195780546
-1.463415000471	-2.363164109912
-1.463010809723	-2.362631166241
-1.462762139523	-2.361616973238
-1.462533455164	-2.361582545143
-1.462312596583	-2.361543127031
-1.461994112552	-2.361355361877
-1.461751077695	-2.361136520887
-1.461511569508	-2.360818602062
-1.461098540735	-2.359812297282
-1.460913983312	-2.35937067887
-1.460681287688	-2.358846583744
-1.460448986381	-2.357451510922

-1.460208412636	-2.356955990809
-1.460001951356	-2.356810383683
-1.459804112399	-2.356713773362
-1.459615785404	-2.356566912052
-1.459299945465	-2.355643001758
-1.459051152958	-2.355550990496
-1.458863842659	-2.355545380517
-1.458588746023	-2.355306015538
-1.458245302302	-2.355117315869
-1.457992575164	-2.354847066618
-1.457583057471	-2.354042807271
-1.457303889577	-2.353137348648
-1.457009639004	-2.35229882532
-1.456770240648	-2.352262068443
-1.456478966851	-2.351542771238
-1.456317506744	-2.35082984976
-1.456110369774	-2.350614772365
-1.455849558096	-2.350347490265
-1.455624043553	-2.350096109428
-1.455433229583	-2.349966622427
-1.455227125841	-2.349614595363
-1.454976904379	-2.349344156631
-1.454711078809	-2.349092528596
-1.454499738339	-2.348690178984
-1.454341388943	-2.348391864758
-1.454016032977	-2.347036290218
-1.453787405668	-2.347034471862
-1.453560464086	-2.346849948701
-1.453213961114	-2.346083318538
-1.452813219182	-2.344368809378
-1.45263152706	-2.344153369072
-1.45238324456	-2.343725965743
-1.452023752985	-2.343182473428
-1.451798858157	-2.343046454926
-1.451515618891	-2.342381888069
-1.451224193684	-2.342283801197
-1.450912402243	-2.341939759448
-1.45064554092	-2.341938832783
-1.45039683311	-2.341500817053
-1.449985401575	-2.338859418196
-1.44975980975	-2.338706722043
-1.449554101895	-2.338572484893
-1.44933810252	-2.338265391348
-1.449178041452	-2.338209058136
-1.448934547724	-2.337717844818
-1.448689798466	-2.337464244965
-1.448371042105	-2.336787648424
-1.448105182365	-2.336571331609
-1.447851155565	-2.335160226784
-1.447606079188	-2.33489783443
-1.44733719102	-2.334855497901
-1.447129618643	-2.334416461244
-1.446948249709	-2.334254272528
-1.446481799191	-2.332448124044

-1.446227131667	-2.331952525389
-1.445901339705	-2.331289728812
-1.445661036543	-2.331253724812
-1.44542077	-2.33031892532
-1.445104502268	-2.330274955493
-1.444814044129	-2.329025599209
-1.444538651607	-2.328683768009
-1.444358675151	-2.328489139816
-1.444180065188	-2.327570914566
-1.443980097581	-2.327564589582
-1.443736292859	-2.327285519273
-1.443463567537	-2.327242310978
-1.443181883142	-2.326853602123
-1.442967026598	-2.32649886018
-1.442670481811	-2.326334020537
-1.442495282852	-2.326246329316
-1.442105247573	-2.325315570701
-1.44178632371	-2.325301615111
-1.441589392789	-2.325092730309
-1.441325412252	-2.324699075847
-1.441095916515	-2.323265559371
-1.440773418672	-2.323111511541
-1.440541885222	-2.323011571498
-1.440267148117	-2.32211192794
-1.439994173699	-2.3207546508
-1.439771246306	-2.320632609589
-1.439588193895	-2.320423185104
-1.439310091886	-2.320122049914
-1.439001514024	-2.319742992346
-1.438766585542	-2.319664425034
-1.43837610809	-2.318635717424
-1.438032404988	-2.31695021808
-1.437753389411	-2.316663918726
-1.437510141055	-2.315447874818
-1.437218577872	-2.315204143337
-1.436904139742	-2.313939630672
-1.436594752285	-2.3129574305
-1.436388124769	-2.312078502769
-1.43612749111	-2.311718642611
-1.435862731001	-2.311544737824
-1.435585652315	-2.311468558472
-1.435163825583	-2.310578945287
-1.434926538451	-2.310296947622
-1.434734448331	-2.309833724853
-1.434486952521	-2.30917948086
-1.43426099903	-2.308726674644
-1.434002635963	-2.308667931928
-1.433731412567	-2.306999014849
-1.433371988982	-2.306894583532
-1.433150424769	-2.306811032391
-1.432933328395	-2.306102221778
-1.432644691965	-2.305617512898
-1.432385535459	-2.30529230029
-1.432101060747	-2.30485151191

-1.431827992268	-2.30366701255
-1.431601046795	-2.303294418491
-1.431404091552	-2.303212924144
-1.43117542141	-2.302739748117
-1.430917101182	-2.301069056084
-1.4306842384	-2.300226660264
-1.430426321319	-2.299490126941
-1.43009728467	-2.298545028396
-1.429873262676	-2.297645671466
-1.429547768315	-2.296459336451
-1.429328250678	-2.296218774067
-1.429102455881	-2.296206694141
-1.428911981951	-2.296136802101
-1.428593611701	-2.295520068835
-1.428365704392	-2.295501633278
-1.42810452753	-2.293913520467
-1.427875036878	-2.293829039421
-1.427645000419	-2.293081125783
-1.427394672321	-2.292805160663
-1.427080370498	-2.291815260096
-1.42676849653	-2.291501539122
-1.426483865324	-2.290133208102
-1.426154943574	-2.289834630409
-1.425905589204	-2.289432759471
-1.425645702334	-2.289235136069
-1.425428315314	-2.288813893762
-1.425102667455	-2.288189362056
-1.424771487995	-2.285736800272
-1.424524968292	-2.285264041893
-1.424304746508	-2.285211816725
-1.424091697123	-2.284896846412
-1.423718674393	-2.284068566483
-1.423327472773	-2.282366309547
-1.422957445982	-2.281886039102
-1.42283267287	-2.281625158119
-1.422520570333	-2.28048955811
-1.422350701844	-2.280052967943
-1.422086582754	-2.27982376675
-1.421918732054	-2.279585333035
-1.421624619236	-2.279556302697
-1.421262726125	-2.278178213107
-1.420992772216	-2.277635009983
-1.420768855029	-2.277094023469
-1.420476113058	-2.276361590932
-1.420246682492	-2.275774780068
-1.419970261341	-2.27576636298
-1.419667305993	-2.275119910251
-1.419442263818	-2.274813792492
-1.419172062485	-2.274525116346
-1.418939605027	-2.274120797679
-1.418733196557	-2.273673617192
-1.418503759488	-2.273636348426
-1.41829261421	-2.273631436009
-1.418073032494	-2.273481798758

-1.417832053985	-2.273354151973
-1.417588128354	-2.272335670942
-1.417265856449	-2.271651848946
-1.417017866676	-2.27094785731
-1.416771240615	-2.270468414019
-1.416501170447	-2.269959707222
-1.41621667777	-2.269387312947
-1.415865059922	-2.268237985721
-1.415628298695	-2.267881947996
-1.415245669343	-2.264356149051
-1.414980753789	-2.263975654596
-1.414718331183	-2.263615664344
-1.414456737667	-2.263512862016
-1.414236166492	-2.262834401706
-1.414002410727	-2.262567270602
-1.413745525763	-2.262369324511
-1.413548432449	-2.262353078879
-1.413331208986	-2.262276561003
-1.413094439264	-2.262260053084
-1.412890357565	-2.262101975264
-1.412584377566	-2.261800523674
-1.412347591469	-2.261465792201
-1.412178074215	-2.261443959611
-1.41199590516	-2.261332448678
-1.411727729776	-2.261080861137
-1.411483654797	-2.26055939208
-1.411179738605	-2.259316424365
-1.410896386182	-2.259238331122
-1.41071900973	-2.25903024209
-1.410468124924	-2.258286037283
-1.410247171473	-2.257633574016
-1.410086153958	-2.257442441935
-1.40990041696	-2.257403454321
-1.409721334767	-2.257204011842
-1.409508003495	-2.257146689722
-1.409347389294	-2.25699075326
-1.409021980488	-2.256947411627
-1.408809108363	-2.256574806829
-1.408444785323	-2.255688668349
-1.408135966616	-2.254835670351
-1.407888653428	-2.254757987009
-1.407616068498	-2.254145971352
-1.407394492982	-2.254005115161
-1.407249742966	-2.253964039648
-1.407021668282	-2.253914142103
-1.406717227216	-2.253681419219
-1.406480193118	-2.25262076012
-1.40628082062	-2.252382780321
-1.406088550892	-2.252262724414
-1.405746553985	-2.251604612095
-1.405588509826	-2.251598867329
-1.405348479418	-2.250948655001
-1.405165324649	-2.25075108398
-1.404994918059	-2.250682312212

-1.404747637634	-2.24958846165
-1.404457822893	-2.248582893592
-1.404171174739	-2.248485524775
-1.403886200254	-2.247975407402
-1.403641633782	-2.247706400535
-1.403371381816	-2.2476634135
-1.403063556248	-2.246428725471
-1.402741115923	-2.245915603149
-1.402397360581	-2.244886937404
-1.402149873287	-2.244790895028
-1.401881459013	-2.244602942582
-1.401686814811	-2.244313900496
-1.401473037739	-2.244230981227
-1.401163748144	-2.243336420387
-1.400925767085	-2.24288500414
-1.400643026162	-2.242250792803
-1.400414128338	-2.242165471029
-1.40023069132	-2.241943154492
-1.400035275162	-2.24184330683
-1.399719708875	-2.241303790616
-1.399451282809	-2.240967295115
-1.399220449471	-2.240773517982
-1.398891365063	-2.240679016808
-1.398714665028	-2.240524804002
-1.398407913934	-2.240078650095
-1.398100229845	-2.239228480456
-1.397882595092	-2.239080982174
-1.397632397584	-2.238894424112
-1.397402173197	-2.238858516884
-1.397205852363	-2.238854729998
-1.397040190665	-2.238597043958
-1.396820976293	-2.238053754389
-1.396638744205	-2.237702866937
-1.39648283596	-2.237157546976
-1.396340641188	-2.237063584701
-1.396057906097	-2.236221204348
-1.395797546721	-2.236097397467
-1.395589615558	-2.236013032897
-1.395391350196	-2.235559314141
-1.395198133055	-2.23518486037
-1.394957275301	-2.234643718273
-1.394684568751	-2.234582557901
-1.394460586077	-2.234440662311
-1.394147344843	-2.233878629322
-1.393923742602	-2.233366489173
-1.393650971722	-2.233322602909
-1.393476711543	-2.233314118768
-1.393277911452	-2.232970472301
-1.393060746963	-2.231989110539
-1.392793850893	-2.231737832046
-1.392487135576	-2.23094705315
-1.392175469747	-2.230757689171
-1.391901976785	-2.230756317815
-1.391647902497	-2.230637159856

-1.391385830463	-2.230233084861
-1.391010391841	-2.228987884869
-1.390775668108	-2.228729009931
-1.390498322545	-2.228150582134
-1.390252537568	-2.227805569855
-1.390039747584	-2.227342094101
-1.389750924823	-2.225968045133
-1.389512990098	-2.225582872645
-1.389290734081	-2.225431377337
-1.389006531339	-2.225315229515
-1.388768797728	-2.225033138248
-1.388507254432	-2.224898609835
-1.388243034141	-2.22458070627
-1.387990569889	-2.224143890881
-1.387659549223	-2.222649953263
-1.3874241429	-2.222519762868
-1.387078864903	-2.221981369622
-1.386784891133	-2.220309177295
-1.386524160006	-2.21994262965
-1.386348190795	-2.219710614406
-1.386093682901	-2.21964850604
-1.385983321092	-2.219573192167
-1.385698814258	-2.219322468101
-1.385516018324	-2.219315782375
-1.385224208839	-2.218668839487
-1.384995395008	-2.21864262432
-1.384776748111	-2.218426742355
-1.384555280488	-2.218369178329
-1.384284592682	-2.218213336754
-1.383928133281	-2.218143185295
-1.38360289544	-2.21786783022
-1.383328209446	-2.217505502161
-1.38303142742	-2.216564151573
-1.38284555382	-2.21574905094
-1.382561006465	-2.215359309435
-1.382302264599	-2.214537239262
-1.38210127081	-2.214042176397
-1.38181203605	-2.213192297041
-1.381654255067	-2.212895209124
-1.38129439555	-2.212393845011
-1.381043433935	-2.211866302389
-1.380841882465	-2.211491412479
-1.380571552789	-2.211363968451
-1.380342578386	-2.210918023844
-1.380028657551	-2.210384453826
-1.37981570967	-2.210366388893
-1.379569215319	-2.209945935835
-1.379420792106	-2.209681718246
-1.379089831754	-2.207752267255
-1.378864038367	-2.206700327832
-1.378546591428	-2.205025115998
-1.378292718164	-2.204696213169
-1.378084964932	-2.204661180005
-1.377809837904	-2.204559713634

-1.377617981163	-2.204544849815
-1.377360944095	-2.204514874006
-1.377173132279	-2.204088512677
-1.377016934215	-2.203965256044
-1.376804055189	-2.20302053698
-1.376538691519	-2.202474126372
-1.376312930798	-2.202458537379
-1.376074730236	-2.202082358849
-1.3758090851	-2.201810606612
-1.375615667903	-2.201748956652
-1.375367355437	-2.201580086269
-1.375217615474	-2.201489736486
-1.374889697295	-2.201327234725
-1.374749898278	-2.2012615237
-1.374565856156	-2.201261243791
-1.374328466135	-2.201159841835
-1.374063015291	-2.200159150295
-1.373901171482	-2.199757167516
-1.373630307505	-2.198427920262
-1.373435195708	-2.198162530393
-1.373178576957	-2.197102582509
-1.372960010612	-2.196914974885
-1.372659772737	-2.195821543383
-1.372388240332	-2.194696526007
-1.372140088528	-2.194152131599
-1.371908241866	-2.193944561434
-1.37170273722	-2.193594290954
-1.371483565951	-2.193574808406
-1.371256771631	-2.193364009631
-1.371054604581	-2.193260018133
-1.370845579354	-2.192864610783
-1.370526008604	-2.192650007546
-1.370265751095	-2.191590335276
-1.370114323481	-2.191040183948
-1.369871804283	-2.190936452298
-1.369600021149	-2.190633528385
-1.369386095811	-2.19010225188
-1.369173962256	-2.189920367092
-1.368930910411	-2.189118464777
-1.368687353299	-2.188591531586
-1.368444226634	-2.188437715017
-1.368279593617	-2.18822256251
-1.368022829102	-2.186051778318
-1.367689849355	-2.185353827604
-1.367449829398	-2.18527070373
-1.367264297709	-2.185132405353
-1.366951235459	-2.184756688026
-1.366625230048	-2.182157876598
-1.366415227186	-2.181785244743
-1.366246473687	-2.18142635547
-1.366006539665	-2.180676187796
-1.365610479629	-2.180486875629
-1.36538818561	-2.180202276488
-1.365220991026	-2.180181928796

-1.364984923613	-2.179880892139
-1.364711930841	-2.179794200031
-1.364488521185	-2.179737852603
-1.36426645429	-2.179735180685
-1.363958661118	-2.17896133892
-1.363774922758	-2.178677890397
-1.363503407303	-2.178155846714
-1.3632264072	-2.177959416763
-1.363043414748	-2.177803440031
-1.362775424388	-2.176954436619
-1.36245231205	-2.176693577051
-1.362131146558	-2.176476097127
-1.361895510542	-2.175208390705
-1.3616801018	-2.175042068394
-1.361448619654	-2.174823560839
-1.361223011662	-2.174795236315
-1.360985947168	-2.174379001943
-1.360771915434	-2.173932970534
-1.360546659895	-2.173898883931
-1.360294134794	-2.173106629688
-1.360080690685	-2.173064602332
-1.359799917107	-2.172741194901
-1.359620720255	-2.172411206356
-1.359427415653	-2.172350750771
-1.359240392947	-2.172044290759
-1.358906382181	-2.171987186251
-1.358604508575	-2.17115617487
-1.358339800484	-2.17097699439
-1.358062958673	-2.170280693079
-1.357777657662	-2.17017665832
-1.357562976514	-2.169661137591
-1.357355097538	-2.169442896082
-1.357143344785	-2.168898247707
-1.356888951264	-2.168554200222
-1.356637658316	-2.168149955676
-1.356422637073	-2.167950915177
-1.356200059279	-2.167320808871
-1.355931574706	-2.16665240493
-1.3556583089	-2.166120874526
-1.355397291776	-2.165276627353
-1.355209132569	-2.164867328572
-1.355033445321	-2.164591746555
-1.354879654173	-2.164562201567
-1.354642689412	-2.164019322466
-1.354478063679	-2.163710103984
-1.354337623219	-2.163590779423
-1.354088011281	-2.162620036971
-1.353797584423	-2.16170978331
-1.353556386443	-2.161097632897
-1.353306316641	-2.160322308539
-1.352993357985	-2.159641883298
-1.352757609176	-2.158679173124
-1.352568498433	-2.158559688253
-1.352329393847	-2.1581758098

-1.352137645898	-2.158026370852
-1.351923793246	-2.157725222665
-1.351699018833	-2.157396181116
-1.351499204485	-2.157118017864
-1.351225637144	-2.156313236665
-1.351083759882	-2.155953987463
-1.350818347083	-2.155885343996
-1.350508127981	-2.1545367871
-1.350276324379	-2.154050987461
-1.350008591816	-2.154032941291
-1.349700374412	-2.154029799633
-1.349505428917	-2.154005660096
-1.34927356746	-2.153875137426
-1.349040837497	-2.153721701777
-1.348734447397	-2.152357816698
-1.348501877526	-2.151428188236
-1.348282337295	-2.15130165394
-1.348015202244	-2.149936663218
-1.347760000872	-2.149355089599
-1.347510376739	-2.149159678166
-1.347255821563	-2.148288268442
-1.346973206634	-2.1474854388
-1.346758388787	-2.147047986667
-1.346495202978	-2.146402715769
-1.346319689314	-2.145957273704
-1.346005073069	-2.145129327948
-1.34578787319	-2.144987249764
-1.345589469475	-2.144156541928
-1.345405092005	-2.143513545186
-1.345144297998	-2.143396804633
-1.344989810878	-2.143250959316
-1.344801748586	-2.142761398825
-1.344586916444	-2.142580317554
-1.344415855005	-2.142526881649
-1.344269916272	-2.142425854817
-1.343977671189	-2.140752008787
-1.343782987877	-2.140750188694
-1.343530831034	-2.140511827282
-1.343258843934	-2.139722478221
-1.343038762882	-2.138869000704
-1.342819695736	-2.138401923926
-1.342625658587	-2.138188615033
-1.342485037659	-2.138134610601
-1.342254408465	-2.137496787764
-1.342012934489	-2.136971009846
-1.341812935366	-2.136955491401
-1.341549389034	-2.136655463726
-1.34132296408	-2.136558819357
-1.341163032299	-2.136557960022
-1.34092787456	-2.136195436469
-1.340689786803	-2.135243884073
-1.340503464857	-2.134674799524
-1.340309254752	-2.134537583202
-1.340079222836	-2.134377796212

-1.339881750146	-2.134310971021
-1.339596710276	-2.133701242382
-1.339346737638	-2.132739445593
-1.339058518027	-2.131597019544
-1.338858033473	-2.131505311483
-1.338646900644	-2.130487957694
-1.3383394277	-2.130075914954
-1.338103944861	-2.129825700802
-1.337794885662	-2.129820321083
-1.337486319938	-2.129337064371
-1.33728724129	-2.128852960333
-1.336959723221	-2.126930590283
-1.336660085256	-2.125500265079
-1.336442088973	-2.125360008237
-1.33621781334	-2.125202103519
-1.3359503197	-2.123889541258
-1.335617220072	-2.1221251355
-1.335473190327	-2.121858999651
-1.335246913657	-2.121530815255
-1.334994289193	-2.121351068381
-1.334803931542	-2.120741707649
-1.334463905307	-2.120438862589
-1.334213442509	-2.11926602574
-1.334045668799	-2.119213208978
-1.333838959406	-2.119170766185
-1.3336305829	-2.118708561475
-1.333350969504	-2.117587742236
-1.333078204795	-2.117535348788
-1.332766998349	-2.116180058486
-1.332586516252	-2.116049218191
-1.332415359762	-2.115236238273
-1.332080031057	-2.114595427677
-1.33179663828	-2.114411818855
-1.331624740164	-2.113790378647
-1.331360221105	-2.112967537245
-1.331098171594	-2.112864325734
-1.330826355058	-2.111593420744
-1.330546812351	-2.111523849463
-1.330319162037	-2.110819817476
-1.330142512814	-2.110547167697
-1.329944920181	-2.110377456307
-1.329725083195	-2.109642740887
-1.329421714104	-2.109516213952
-1.329236667562	-2.108843096028
-1.329024615355	-2.108707947067
-1.328760781884	-2.107819828508
-1.328501050757	-2.106905555421
-1.328288816573	-2.106715254772
-1.328063445928	-2.106461372569
-1.327849989911	-2.106457389704
-1.327617487527	-2.106164807841
-1.327327391014	-2.104215090672
-1.327078992813	-2.103745272377
-1.326752365689	-2.103697646308

-1.326467441245	-2.103531127074
-1.326309070529	-2.103446448256
-1.326062108108	-2.102780360752
-1.325780212088	-2.102053434043
-1.325531711192	-2.101960393306
-1.325278817232	-2.101463700893
-1.324997116319	-2.10095596438
-1.32482128136	-2.100848632385
-1.324527201508	-2.100595912602
-1.324257683431	-2.09956425745
-1.324026676607	-2.099512979311
-1.323756613258	-2.098891938964
-1.323515423171	-2.098105128165
-1.323305473771	-2.098081735777
-1.32312344572	-2.097620929436
-1.322942969174	-2.09752431272
-1.322799314139	-2.097503976616
-1.32258057487	-2.097426758356
-1.322345817991	-2.097059234655
-1.322067757112	-2.096126224636
-1.321901815386	-2.096031276274
-1.321727149229	-2.095735035185
-1.321423734425	-2.095153853779
-1.321291547569	-2.095092700454
-1.321055430852	-2.094772469968
-1.320831341838	-2.094292996446
-1.320561628116	-2.093010398998
-1.32027690409	-2.091898630716
-1.320160124365	-2.091830916169
-1.319951047088	-2.091817038867
-1.319761305323	-2.091692052113
-1.319527166057	-2.091285399707
-1.319318620411	-2.09125519566
-1.319140997584	-2.090976596187
-1.318942927238	-2.090965548512
-1.318714437824	-2.090676072313
-1.318518324224	-2.090453109149
-1.318372563464	-2.090260186913
-1.31814606351	-2.090132290041
-1.317953662994	-2.089846092726
-1.317720797593	-2.089439081778
-1.317433421526	-2.089361057762
-1.317229946135	-2.088788669259
-1.317004871426	-2.088640321246
-1.316761920649	-2.088582294302
-1.316567953845	-2.088289483099
-1.316293103218	-2.087790384707
-1.316011156455	-2.087761211327
-1.315825489546	-2.087512159258
-1.315621447087	-2.087306720351
-1.31546987797	-2.087255289516
-1.315258078029	-2.08674043647
-1.315081456223	-2.086153002085
-1.31472237457	-2.085701827456

-1.314489839967	-2.085480720912
-1.3142615681	-2.085454479426
-1.314045086214	-2.084864071899
-1.313824713797	-2.084352178203
-1.313572639232	-2.084049499974
-1.313389110179	-2.083955618786
-1.313169506447	-2.083881254946
-1.312954023384	-2.08365558272
-1.312729126776	-2.082975182366
-1.312418587793	-2.082857900386
-1.312238323757	-2.082743258362
-1.312016862167	-2.082115906407
-1.311728558654	-2.081436349987
-1.311495968605	-2.081359558098
-1.311263874982	-2.081016254584
-1.311067766901	-2.080850552068
-1.310842805997	-2.080628861008
-1.310700309697	-2.080471850143
-1.310492039734	-2.080197344702
-1.310238886608	-2.080152785705
-1.310044589385	-2.079883540221
-1.309841725795	-2.079690894188
-1.309604825884	-2.079064409028
-1.309390041333	-2.078905464801
-1.309196051718	-2.078836436228
-1.30891052454	-2.077844348884
-1.30859987628	-2.076971590843
-1.30844644241	-2.07693953405
-1.308299518146	-2.076553695538
-1.308044502971	-2.075012916621
-1.307743601114	-2.074999741933
-1.307531699198	-2.074089830367
-1.307239392182	-2.074025127283
-1.306966602896	-2.073594640572
-1.306722695019	-2.073101804716
-1.306586064086	-2.072835009705
-1.306409636774	-2.072687649298
-1.306257020128	-2.072288387979
-1.306031915961	-2.072191507176
-1.305748241972	-2.07082548977
-1.305460514971	-2.070056067105
-1.305202479893	-2.07004569037
-1.304946039782	-2.069541525567
-1.304650854565	-2.069203172676
-1.304419364285	-2.06892186576
-1.304184288135	-2.068573206795
-1.303848610448	-2.067363875649
-1.303355432786	-2.063319466909
-1.303162060761	-2.06327555328
-1.302888765205	-2.062882701081
-1.302703257824	-2.062879049179
-1.302527145738	-2.062237779225
-1.302268851158	-2.062107089314
-1.302068849435	-2.061927481516

-1.301815349892	-2.060883228399
-1.301504859658	-2.059240699691
-1.301237903061	-2.058719520043
-1.301029435922	-2.058668050027
-1.300702858355	-2.057990892665
-1.300396247849	-2.057360605955
-1.300138930111	-2.057240265856
-1.299857147771	-2.056618042234
-1.299631189727	-2.056433129658
-1.299445166923	-2.05631612444
-1.299260426886	-2.056226001439
-1.299012913375	-2.056211331686
-1.298841504817	-2.055934936735
-1.298569968324	-2.054953738456
-1.298314479276	-2.05369242546
-1.298049262456	-2.053596174837
-1.297799851395	-2.053544969574
-1.297591350402	-2.053462365019
-1.297416956114	-2.053249641073
-1.297221169057	-2.05323805807
-1.296954462413	-2.053085844781
-1.296750317802	-2.052907923445
-1.296471259323	-2.051458501235
-1.296232154572	-2.050690984876
-1.295938051419	-2.049870425583
-1.295752570754	-2.049565667273
-1.295500070531	-2.048799830259
-1.295213103414	-2.047801897107
-1.29502821141	-2.047514772873
-1.294836322078	-2.047425048584
-1.294572834092	-2.047074771117
-1.294440004749	-2.047064139295
-1.294258155766	-2.047016351982
-1.294112738116	-2.046958195204
-1.293966508697	-2.046750040733
-1.293742095636	-2.046681691457
-1.293517095536	-2.045176752422
-1.29325051624	-2.044179640462
-1.292990619881	-2.042991113063
-1.292800757273	-2.04263106746
-1.292552983015	-2.041531103544
-1.292327019821	-2.041280124517
-1.29208624428	-2.041091601493
-1.291864219357	-2.040486368884
-1.29171243334	-2.04035027392
-1.291526990903	-2.039914018894
-1.291336491335	-2.039522265941
-1.29115596993	-2.039479760327
-1.290874195984	-2.038484853705
-1.290570628714	-2.037347850808
-1.290386519746	-2.036799032335
-1.290158094004	-2.036696951402
-1.289962630116	-2.036053444746
-1.289748676077	-2.035267186026

-1.289522285708	-2.034626379435
-1.289292838868	-2.033534283064
-1.289087868074	-2.033200125655
-1.288841665345	-2.032623745172
-1.288682097445	-2.032468785857
-1.288478596991	-2.032325182198
-1.288335247264	-2.031784606004
-1.28810387584	-2.03128055582
-1.287882617667	-2.030835914853
-1.287688056056	-2.030740406356
-1.287546001114	-2.030393493441
-1.287302881358	-2.030256454108
-1.287123409213	-2.030247106745
-1.286910338669	-2.030051588811
-1.286671995497	-2.029903746487
-1.286467974294	-2.029481979536
-1.286267869972	-2.029445508733
-1.286072940212	-2.028954418057
-1.285833257025	-2.028642950428
-1.285628540128	-2.028595209973
-1.285427031801	-2.028566167278
-1.285198266037	-2.027928784861
-1.284979396872	-2.027919842562
-1.284630461839	-2.027570670004
-1.284443567081	-2.027314781356
-1.284263290803	-2.027256229693
-1.28409975399	-2.027106225413
-1.283848291389	-2.027038924185
-1.283591721022	-2.02687044695
-1.283373563274	-2.026719516024
-1.283141611606	-2.025365912319
-1.282785879143	-2.024251108389
-1.282626769027	-2.023863189451
-1.282379994855	-2.02290295602
-1.282079774896	-2.021120693708
-1.281885921198	-2.021064861964
-1.281639243162	-2.020885180339
-1.281429470752	-2.020095934131
-1.281256314731	-2.019157986939
-1.281016804063	-2.01862326658
-1.280683352685	-2.018586493845
-1.280419234223	-2.018237327663
-1.280229913421	-2.017922850204
-1.280004820721	-2.01773942767
-1.279744395221	-2.017723529219
-1.279494379159	-2.017511806656
-1.279272734007	-2.017368411802
-1.278970303407	-2.016880491784
-1.278758591413	-2.016730296328
-1.27848622438	-2.015462049606
-1.278272360292	-2.015138338011
-1.278103133037	-2.014957288125
-1.27793759903	-2.014913836645
-1.2777246255	-2.014591340253

-1.277528746297	-2.014557272455
-1.277335393602	-2.014354975451
-1.277087749976	-2.013671009453
-1.276909433725	-2.013611222773
-1.276658564336	-2.013225140596
-1.276341934857	-2.012916418345
-1.276158899715	-2.01270677431
-1.275890932937	-2.012612255035
-1.275652432749	-2.011661558819
-1.275422672035	-2.010934505416
-1.275217542141	-2.010692386638
-1.274947348275	-2.010553951082
-1.274783880043	-2.01020976393
-1.274651972836	-2.010125829845
-1.274455989154	-2.010063714043
-1.274237422887	-2.009904905262
-1.274099889177	-2.009842019763
-1.27394172442	-2.009656581003
-1.273760531812	-2.008523009236
-1.273570518963	-2.008373799937
-1.273366494361	-2.008021875337
-1.273200878438	-2.00799496454
-1.273026161895	-2.007868994113
-1.272783514613	-2.007690965435
-1.272586342108	-2.007308090535
-1.272417668806	-2.007040702204
-1.27223710183	-2.006735656645
-1.272025411273	-2.006663257615
-1.271748207966	-2.005066440194
-1.271572887752	-2.004659521015
-1.271273556219	-2.003545440336
-1.2710340855	-2.002726166298
-1.270798414593	-2.002626325125
-1.270569277544	-2.002209590919
-1.270333159206	-2.002062066556
-1.270079897277	-2.001843464615
-1.269691732722	-2.001541531264
-1.269461282126	-2.001390253973
-1.269222690078	-1.999815873859
-1.268983531503	-1.999104732002
-1.268760877573	-1.99879410494
-1.268576284451	-1.998416072306
-1.268385990713	-1.998414463335
-1.268126345525	-1.998090662313
-1.267894610115	-1.997673412998
-1.267702014153	-1.99759903264
-1.267396085017	-1.997357304093
-1.26722718961	-1.997250317698
-1.2670295705	-1.997005888709
-1.266861368795	-1.996786362317
-1.266521920188	-1.995215380951
-1.266329067334	-1.994807430006
-1.266090911778	-1.994753277581
-1.265818524885	-1.993675761403

-1.265612140736	-1.993624193138
-1.265396698324	-1.993238694698
-1.265031404148	-1.993058085625
-1.264760249905	-1.991770410404
-1.264620800296	-1.991764942638
-1.264362639283	-1.991222086017
-1.264100931449	-1.990995093686
-1.263876661917	-1.990819829711
-1.263694567633	-1.990581485668
-1.263512721878	-1.990047254251
-1.263340492186	-1.989865642429
-1.263104738973	-1.989702761592
-1.262943794609	-1.989541379188
-1.262543738353	-1.989240029803
-1.262368380022	-1.989181240259
-1.262148826826	-1.989095353804
-1.261894343388	-1.988436687535
-1.261674785517	-1.988253237963
-1.261405417397	-1.98707588148
-1.261225783782	-1.986830958287
-1.261017097982	-1.986363875117
-1.260792789682	-1.985689103846
-1.260576585489	-1.985412985114
-1.260426305145	-1.985300464808
-1.260253213272	-1.984737894487
-1.260088952545	-1.984563147747
-1.259854189656	-1.984454759889
-1.259597708287	-1.983767774986
-1.259429138913	-1.983588154631
-1.259204963502	-1.982793036209
-1.258946034445	-1.982426981703
-1.258575225416	-1.981427476223
-1.258435658162	-1.981420718596
-1.258190476328	-1.98133937371
-1.25802004569	-1.981079859028
-1.257773686901	-1.980732089667
-1.257507737405	-1.980706440076
-1.257337887347	-1.980692980638
-1.257211848614	-1.980678340606
-1.256961002101	-1.980305892438
-1.256726247014	-1.980167845267
-1.256600056918	-1.98011451334
-1.256470202903	-1.979947682525
-1.256311256214	-1.979714601444
-1.256085831872	-1.979429558586
-1.255897412939	-1.979308740386
-1.255668326257	-1.979286364233
-1.255377134485	-1.977934547501
-1.255224431371	-1.977848907255
-1.254981859064	-1.977426754502
-1.254825921385	-1.976762434698
-1.254592007539	-1.976759666429
-1.254380784555	-1.976518302275
-1.254110817046	-1.976164153058

-1.253859918041	-1.975494271506
-1.253650975757	-1.974315386597
-1.253441356201	-1.974287679741
-1.253182873007	-1.974143172884
-1.252952897168	-1.973924928989
-1.252744513322	-1.973566066738
-1.252481393418	-1.973311054965
-1.252229540532	-1.973141401399
-1.252013041604	-1.972681459238
-1.251760050665	-1.972641069433
-1.251558484184	-1.972617915614
-1.251262461804	-1.972440126973
-1.25110190043	-1.972404265356
-1.250922676638	-1.972301449637
-1.250642815917	-1.971748721143
-1.250506994689	-1.971605266876
-1.250323721904	-1.971397237879
-1.250104205062	-1.971319909262
-1.249854416162	-1.970684954762
-1.249682873877	-1.970554221415
-1.249516395632	-1.970222069602
-1.249252635097	-1.969945720651
-1.249062645511	-1.969341506727
-1.248842744373	-1.968883797765
-1.24864904339	-1.968855592337
-1.248412135763	-1.968708939702
-1.248225066447	-1.968596988857
-1.248058839543	-1.967906364294
-1.247906811512	-1.967805687575
-1.247714445775	-1.967780227914
-1.247564246472	-1.96768907411
-1.247394325268	-1.967665124191
-1.247191359356	-1.967646895684
-1.247057625131	-1.967391943138
-1.246800762032	-1.966868618873
-1.246601222001	-1.966520810478
-1.24640074674	-1.966184919402
-1.24615666381	-1.965823204929
-1.245936214827	-1.965072015608
-1.245733472895	-1.96501458422
-1.245520379961	-1.964829091575
-1.245256818352	-1.963573210005
-1.244972917538	-1.963435341692
-1.244751835129	-1.963290480958
-1.244566030371	-1.963096470742
-1.244337221066	-1.962949574978
-1.244086782231	-1.962617483925
-1.243812789551	-1.962319745787
-1.243658489674	-1.962100510675
-1.24342465048	-1.961696672097
-1.243154888645	-1.96104128045
-1.242922419934	-1.960984183411
-1.242705627901	-1.960893750517
-1.242544984921	-1.960718684758

-1.242319506176	-1.959923313965
-1.242083654559	-1.958868465974
-1.241829020333	-1.958061904672
-1.241587423386	-1.956992744015
-1.241400956785	-1.956930555726
-1.241232181489	-1.956811320479
-1.240961286516	-1.956408345146
-1.240710015677	-1.955483118448
-1.240522762022	-1.954643664039
-1.240328354684	-1.954047342743
-1.23997965195	-1.953947815359
-1.239821521377	-1.953734737725
-1.239658614383	-1.953500693297
-1.239484444599	-1.953476200916
-1.239314637491	-1.953224654304
-1.239121465759	-1.953159872092
-1.2389081815	-1.953147071434
-1.238665770498	-1.952991713122
-1.238438235101	-1.952326497825
-1.238222431639	-1.951707037375
-1.238058260514	-1.951516401073
-1.237872120584	-1.951275696692
-1.237635232104	-1.951206503739
-1.237454073478	-1.950738457375
-1.237130904059	-1.950169959065
-1.236947912501	-1.949787013143
-1.236746199088	-1.949547399761
-1.236545069198	-1.949015655734
-1.236345504895	-1.948292622837
-1.236068622729	-1.947497314812
-1.235872173144	-1.947309691584
-1.235697526296	-1.947068985968
-1.235454659194	-1.945758384433
-1.235267742722	-1.945361603843
-1.235084738501	-1.945085687802
-1.234903308437	-1.944896549431
-1.23472218705	-1.944260454758
-1.234529580356	-1.944015472537
-1.234323803594	-1.943722742053
-1.234084023502	-1.941958797436
-1.233790781978	-1.941559169087
-1.233609085339	-1.940837875903
-1.233416174712	-1.940817812542
-1.233205659898	-1.940788562638
-1.232948326345	-1.940423905916
-1.232684549457	-1.940237349129
-1.232468790861	-1.94014860867
-1.232311565649	-1.940067818297
-1.232134631316	-1.939967170749
-1.231966021555	-1.939757706827
-1.231722100982	-1.939445190938
-1.231474078304	-1.938673731538
-1.231295943805	-1.938393183147
-1.231106220509	-1.938307666669

-1.230895823018	-1.93826980928
-1.230645811944	-1.937704159092
-1.230337450785	-1.936066449407
-1.230050974803	-1.935998156886
-1.229758718325	-1.935831534438
-1.229543157538	-1.935670315008
-1.229349937035	-1.935478003855
-1.229168183595	-1.935381358914
-1.228971015635	-1.935056604633
-1.228774668797	-1.934151170262
-1.228573404466	-1.93413149228
-1.22832273434	-1.933975796564
-1.228034844981	-1.932614857287
-1.227835904451	-1.931784868497
-1.227580863433	-1.931600260007
-1.227343859461	-1.93154263493
-1.227130114329	-1.931206433584
-1.226975993425	-1.931015184716
-1.226743886163	-1.930597808052
-1.226518110097	-1.930043002217
-1.226332915585	-1.929950342632
-1.226104681104	-1.928823256198
-1.22586570484	-1.927836197771
-1.225722160939	-1.927830856688
-1.2254622092	-1.927585092622
-1.22525451637	-1.926903684016
-1.225107111333	-1.926782294302
-1.22485025168	-1.926227424374
-1.224729577869	-1.926170786394
-1.224552552821	-1.926027859778
-1.224276401239	-1.924238620804
-1.224124827435	-1.92384573047
-1.223983754994	-1.923636839392
-1.223748731211	-1.923373398679
-1.223496285722	-1.923148076932
-1.223288909189	-1.923073628463
-1.223144672448	-1.923070079888
-1.222939078216	-1.922563313627
-1.222727869622	-1.92249849815
-1.222487999264	-1.921876285711
-1.222190027583	-1.92120782974
-1.222025369118	-1.921044252054
-1.221829471671	-1.920712118617
-1.221570510942	-1.920382417335
-1.221408240674	-1.919676285665
-1.221198715022	-1.919247704405
-1.221029779406	-1.919117612389
-1.220745682329	-1.918594148018
-1.220580747994	-1.918553799385
-1.220348807264	-1.917638511785
-1.220091249828	-1.916910788229
-1.219870084568	-1.916669200266
-1.219676421431	-1.915712886768
-1.219448297457	-1.915616450571

-1.219240368767	-1.914990961296
-1.218975660683	-1.914920797388
-1.218769491912	-1.914861407136
-1.218519421569	-1.914658578693
-1.218321075356	-1.913966403531
-1.218086076475	-1.913793505811
-1.217876640602	-1.91290788316
-1.21771891559	-1.912799620543
-1.217339035697	-1.911263703974
-1.217185208911	-1.911028216805
-1.216955681193	-1.910371854511
-1.2167215973	-1.909819320271
-1.216511641717	-1.909490587537
-1.21634855533	-1.909431851123
-1.216124497747	-1.909086408321
-1.215866325083	-1.908522306885
-1.215615675853	-1.908315537159
-1.215319527783	-1.907130526766
-1.215143753618	-1.906198673799
-1.214949574116	-1.906037364555
-1.214753488994	-1.905716895371
-1.214522017159	-1.905667471232
-1.214339876125	-1.90524953945
-1.214170010339	-1.905129363118
-1.214036495307	-1.905038059851
-1.213898976557	-1.90497638545
-1.213738809393	-1.904918739859
-1.213574363068	-1.904743687022
-1.213404151383	-1.904690982427
-1.213172724447	-1.904366184571
-1.212978530003	-1.904242414688
-1.21281589421	-1.903681887908
-1.212598489517	-1.902727681587
-1.212412975997	-1.902567565337
-1.212220658217	-1.90211843644
-1.212046694216	-1.90199594304
-1.211820087176	-1.901968294501
-1.211625878288	-1.901794463186
-1.211439736492	-1.901075488897
-1.211285779478	-1.900968795374
-1.210966159363	-1.9007214158
-1.210728368843	-1.900184058414
-1.210582084408	-1.900113080566
-1.210440970473	-1.899984004546
-1.210241492608	-1.899869217742
-1.210069550963	-1.899695779296
-1.209950202593	-1.899594155713
-1.209780397522	-1.899468533747
-1.209605195302	-1.899409576066
-1.20937929126	-1.899171796296
-1.20920530555	-1.89869090984
-1.20891953391	-1.897197484305
-1.208731388775	-1.896896213337
-1.208504637111	-1.896033387678

-1.208243295455	-1.89564476119
-1.208031260977	-1.895523437472
-1.207863291714	-1.895245373862
-1.207652859934	-1.893929589341
-1.207419906039	-1.893621635358
-1.207162210391	-1.892027686563
-1.20697627885	-1.891766937702
-1.206749917061	-1.891609733745
-1.206562528702	-1.891608099844
-1.206292825527	-1.890306122682
-1.206082929665	-1.890069513005
-1.205796568372	-1.888988053327
-1.205610238396	-1.888710815166
-1.205439334421	-1.888681890817
-1.205197985309	-1.88757583975
-1.204928921017	-1.887239765015
-1.20477106346	-1.887159573245
-1.204529753318	-1.886318266803
-1.204247794006	-1.885600175827
-1.204089625696	-1.885093702635
-1.203840750615	-1.885075867819
-1.203628747574	-1.884441692456
-1.203463980496	-1.884159884204
-1.203280454839	-1.883903327092
-1.203106453666	-1.883141224658
-1.202879109818	-1.882708155356
-1.202699498572	-1.8825403622
-1.202549577052	-1.882403557539
-1.202271976656	-1.882247326309
-1.202018859448	-1.88222774738
-1.201836123795	-1.8820732603
-1.20159995071	-1.882012637716
-1.201326554986	-1.880653791796
-1.201192887569	-1.880550861705
-1.200970493841	-1.880473489374
-1.200752155381	-1.880169272906
-1.200580992923	-1.880037764022
-1.20034609581	-1.879904559341
-1.200145439363	-1.879693339292
-1.199919736542	-1.879097698644
-1.199723205729	-1.878729679599
-1.199523641363	-1.878524453578
-1.199306916398	-1.878319934966
-1.199154995942	-1.878317902332
-1.198965420946	-1.878173176431
-1.198696162813	-1.877833937645
-1.198535509922	-1.877804910948
-1.198328823639	-1.877113486832
-1.198112030037	-1.877039157646
-1.197871536664	-1.876964220554
-1.197640190257	-1.875808949377
-1.197414848582	-1.87569545116
-1.197255230588	-1.875502898089
-1.197036033295	-1.875224432486

-1.196802201488	-1.874968220821
-1.196610213054	-1.874245795015
-1.196400517881	-1.873603142887
-1.196237139908	-1.873075382188
-1.196040078934	-1.872671510478
-1.195886281109	-1.872337356261
-1.19573652227	-1.872325444738
-1.195572035794	-1.872011733228
-1.195391550492	-1.871597676367
-1.19522316447	-1.871365249998
-1.195005075896	-1.871266330908
-1.194765784042	-1.870854049123
-1.194499652243	-1.870176090183
-1.19430093296	-1.869695341976
-1.194115368472	-1.869634111359
-1.193968222635	-1.869325789367
-1.19379666841	-1.868873575532
-1.193569575289	-1.867705764259
-1.193397685748	-1.867659235731
-1.193161951716	-1.867149603299
-1.192918638516	-1.866629362329
-1.192724366384	-1.866531060384
-1.192485070527	-1.866054200131
-1.192308105669	-1.865977793509
-1.192072048548	-1.865414287428
-1.191904757468	-1.865117061597
-1.191675932669	-1.864489222981
-1.191477959349	-1.864187981775
-1.191319612401	-1.863966805897
-1.191121741046	-1.863558039443
-1.190922139845	-1.863164125636
-1.190723317131	-1.86208961787
-1.190504296143	-1.861329714865
-1.190333776728	-1.861032704541
-1.190174296027	-1.860757496751
-1.190015384071	-1.860742542107
-1.189872416942	-1.860486209764
-1.189654955653	-1.860254221806
-1.189488214862	-1.860078182509
-1.189341994187	-1.859588334793
-1.189152573359	-1.859439812257
-1.188951643473	-1.858968627898
-1.188752897803	-1.858677540691
-1.188546177191	-1.858510928916
-1.188426119431	-1.858330402059
-1.188250175332	-1.857893997948
-1.188089584225	-1.857771861537
-1.187859557787	-1.857217628339
-1.187673591625	-1.857079614247
-1.187492259936	-1.856835265059
-1.187310875113	-1.856420693691
-1.187055906867	-1.856160171251
-1.18688534077	-1.856101593975
-1.186693312836	-1.855836321439

-1.186521653434	-1.855720737474
-1.186301934074	-1.855469485496
-1.186064271553	-1.854516665074
-1.185835642153	-1.854400787614
-1.185539619809	-1.85380928855
-1.185354853926	-1.853665656309
-1.185146252734	-1.853215075831
-1.185023496311	-1.853080842916
-1.184804492474	-1.852985978425
-1.184622260711	-1.852752159485
-1.184511396873	-1.852730537894
-1.184373460564	-1.852635154769
-1.184024998164	-1.850012882663
-1.183839908907	-1.849478944398
-1.183676302326	-1.848835636851
-1.1834937707	-1.84833549444
-1.183247450185	-1.848070050252
-1.183070496213	-1.847939104529
-1.182918990337	-1.847692866527
-1.182747007528	-1.847282926906
-1.182561145116	-1.847268805534
-1.182358539006	-1.846414367013
-1.182126657909	-1.84640595635
-1.18195199923	-1.846263817784
-1.181759206927	-1.846218220205
-1.181528078025	-1.845911350993
-1.181379700628	-1.84574349245
-1.181218430874	-1.845404485898
-1.181020083757	-1.845179414192
-1.180847470161	-1.845105966292
-1.18066140626	-1.844822276351
-1.180445214856	-1.844641330144
-1.180302826704	-1.844599551903
-1.180137712146	-1.844488103196
-1.179984013083	-1.844195736265
-1.179763769345	-1.843750394446
-1.179516412279	-1.843562130461
-1.179358005409	-1.842812749266
-1.179207424208	-1.842547600232
-1.179036064996	-1.842186948291
-1.178873844262	-1.842052097228
-1.178711748589	-1.842019829646
-1.178542073427	-1.841187439034
-1.178269010035	-1.839569900868
-1.178097164354	-1.838919321544
-1.177931062174	-1.838103598841
-1.177773862938	-1.837990932111
-1.177574805483	-1.837297651215
-1.177357178192	-1.836800965557
-1.177092585985	-1.835524076122
-1.176899620235	-1.835488827242
-1.176746365133	-1.8354791219
-1.1765765415	-1.835301067668
-1.176379152302	-1.835179059067

-1.176213755512	-1.835064482968
-1.175956184424	-1.834925603596
-1.175791610495	-1.834750118957
-1.175536989127	-1.833810704289
-1.175301820758	-1.833505147229
-1.175143261036	-1.833140426811
-1.174902292547	-1.83218635842
-1.174728366211	-1.831524323749
-1.174518204544	-1.831137118464
-1.17432253156	-1.830769269233
-1.174122497553	-1.830183030488
-1.173868980962	-1.829573885898
-1.173668836656	-1.828963616518
-1.173513330865	-1.828920617952
-1.173349187779	-1.828893516834
-1.173132412389	-1.828543493056
-1.172894804642	-1.828471840522
-1.172745758353	-1.828365962864
-1.172548451617	-1.828221970706
-1.172377312376	-1.828148383697
-1.172158332562	-1.827993887772
-1.172017957822	-1.827964692861
-1.171782430643	-1.827580180973
-1.171567763544	-1.827560358709
-1.171268124522	-1.827444495459
-1.171009404428	-1.826563898808
-1.17083082627	-1.826529826068
-1.17070024704	-1.826429309533
-1.170498749625	-1.826376132403
-1.170357228892	-1.825761332209
-1.170149534123	-1.824989145476
-1.169886655778	-1.824887780425
-1.169732805097	-1.82486190973
-1.169578275245	-1.824781615079
-1.169383719349	-1.82434097952
-1.169132212914	-1.82391402677
-1.168898920686	-1.823843515029
-1.168672646671	-1.823574840712
-1.168466262463	-1.823019597914
-1.168234055525	-1.822998327153
-1.168050683885	-1.822810863121
-1.167872009538	-1.822576829193
-1.167720295892	-1.822234575044
-1.167599120499	-1.822042743404
-1.16737297697	-1.821135394703
-1.167200878379	-1.821080415904
-1.167025307038	-1.82065577019
-1.166862857126	-1.820584560198
-1.166700092472	-1.820505731847
-1.166465933129	-1.819434089172
-1.166247969947	-1.819005515134
-1.166069918546	-1.818382668332
-1.165906113161	-1.818342616316
-1.16573393878	-1.817940715388

-1.165517134611	-1.817276133028
-1.165269310869	-1.815983576302
-1.165078671244	-1.815265772803
-1.164907635231	-1.815230558869
-1.164737616223	-1.81516768754
-1.164552156377	-1.814581523278
-1.164385248581	-1.81431449284
-1.164241461769	-1.814281555833
-1.164054496841	-1.813896401636
-1.16379960163	-1.813531352117
-1.163593530693	-1.813485378858
-1.163404567383	-1.813079057664
-1.163197180452	-1.812838209458
-1.162984670351	-1.812674018556
-1.162864367307	-1.812670642745
-1.162704450072	-1.812647299552
-1.16253992256	-1.811967951858
-1.162346829971	-1.810950158338
-1.162177413641	-1.810464328567
-1.161981733204	-1.80985263051
-1.161783206683	-1.809427383174
-1.161568087797	-1.808383013487
-1.16140994465	-1.807981852179
-1.16120775448	-1.807620450914
-1.161033501169	-1.807323295112
-1.160842533266	-1.807178441649
-1.160646335489	-1.806894225877
-1.160391949939	-1.806556840317
-1.160164573117	-1.804988961297
-1.15999554611	-1.80480055525
-1.15980540366	-1.80479047754
-1.159607922586	-1.804583346959
-1.159382732277	-1.804356174337
-1.15919545205	-1.804259113738
-1.158981178817	-1.804175198701
-1.158793828876	-1.803766009695
-1.158558957996	-1.803643668077
-1.158418935839	-1.803439159567
-1.158194108546	-1.802800609289
-1.158041125539	-1.802755429213
-1.157898499127	-1.802751043606
-1.157742349531	-1.802669583062
-1.157624036221	-1.802551169628
-1.157381268456	-1.801234724734
-1.157179125243	-1.80116393611
-1.156960397148	-1.800482670472
-1.156782057598	-1.800171266836
-1.156588251164	-1.799960957889
-1.156406499066	-1.799149327751
-1.156137415419	-1.798321190659
-1.155935005247	-1.798029794237
-1.155783319245	-1.797510215033
-1.155613042547	-1.797198834403
-1.155410329942	-1.796492141593

-1.155108253804	-1.795781938401
-1.154905504919	-1.795663219332
-1.154608249413	-1.794892292198
-1.154395865049	-1.794774325557
-1.154221638132	-1.794743432095
-1.154065081532	-1.794688010567
-1.153858291337	-1.794546858427
-1.153572139638	-1.79400735833
-1.153362245137	-1.793871048076
-1.153126257414	-1.793570366105
-1.152945251086	-1.79279729494
-1.152717752146	-1.792794907012
-1.152537346443	-1.792693153621
-1.152369103121	-1.792455038745
-1.152206662412	-1.792388993785
-1.152038056141	-1.792325780733
-1.151852671895	-1.792243087726
-1.151701303772	-1.79221834712
-1.151423701232	-1.791816694828
-1.151213456732	-1.791724432438
-1.150997547186	-1.791464755231
-1.15078600601	-1.79141114575
-1.150620929874	-1.791313325774
-1.150404725267	-1.791258914098
-1.150236266398	-1.791005160858
-1.149990346592	-1.790277962279
-1.149807159666	-1.790199501483
-1.149561633728	-1.790198698053
-1.149391852067	-1.789473807116
-1.149253061186	-1.789432966538
-1.149025848603	-1.789418838075
-1.14882405465	-1.789045021168
-1.14869702447	-1.788970849536
-1.148521339155	-1.788747430404
-1.148331173176	-1.788400671046
-1.148193734682	-1.788291153211
-1.147892541007	-1.787047991588
-1.147724331289	-1.786682951176
-1.147434925939	-1.785421031088
-1.147215911215	-1.784521151192
-1.147031296756	-1.784282709783
-1.146841041123	-1.784006225076
-1.14653434908	-1.782433186141
-1.146332405068	-1.781870087643
-1.146198197367	-1.781785369596
-1.146034604752	-1.78167281657
-1.145800431993	-1.781247292696
-1.145621662949	-1.781111563211
-1.145451604164	-1.780831657433
-1.145293258868	-1.780711708047
-1.145168705925	-1.780432347196
-1.144967185231	-1.780336602755
-1.144735267947	-1.780236637328
-1.144536900398	-1.780075165755

-1.1443605078	-1.779864662718
-1.144217674329	-1.779666538989
-1.14404555519	-1.779545469041
-1.143841282747	-1.779489934511
-1.143650499317	-1.778881213214
-1.143499296404	-1.778356926138
-1.143305310618	-1.778254752483
-1.143140950038	-1.778224735811
-1.142959401436	-1.777585032949
-1.142739968316	-1.776868214138
-1.142534100987	-1.776688097492
-1.142384334062	-1.776493450125
-1.142197701312	-1.776159908532
-1.14198408335	-1.77549809581
-1.141790032625	-1.774442902781
-1.141658361085	-1.774360060164
-1.141462824854	-1.773316097719
-1.141315306947	-1.773001411878
-1.141172980425	-1.772818397218
-1.141059411899	-1.772788649575
-1.140929976072	-1.772756916652
-1.140742185339	-1.772688749423
-1.140559327143	-1.772343428743
-1.140370135997	-1.77226441219
-1.140142849256	-1.772239695734
-1.139963097861	-1.772062935734
-1.139821709239	-1.771829653474
-1.139601400427	-1.771790756257
-1.139403762872	-1.77105476882
-1.139165810934	-1.770406120344
-1.138953813976	-1.770299169579
-1.138792312634	-1.770200643642
-1.138627499725	-1.770153997086
-1.138461134578	-1.770015342974
-1.138325728853	-1.769869599479
-1.138150890672	-1.769387960433
-1.137969283804	-1.769313033978
-1.137757816598	-1.769178665072
-1.137648716226	-1.769135592313
-1.137431936682	-1.769010129685
-1.137268235337	-1.768845134932
-1.137154136758	-1.76878683676
-1.137017815483	-1.768705605442
-1.136847192394	-1.768548575103
-1.136569841558	-1.767634368272
-1.136385172001	-1.767497763846
-1.136230397831	-1.767376177368
-1.136033292265	-1.767005729548
-1.135872739417	-1.765999201949
-1.135661063185	-1.765731410485
-1.135456825577	-1.765609354981
-1.135261062888	-1.76530506475
-1.135104130727	-1.765240011126
-1.134935965035	-1.765075777696

-1.134841612091	-1.764978078549
-1.134629398349	-1.764779350395
-1.134414089033	-1.764191621041
-1.134214910511	-1.76398673785
-1.134057707747	-1.76377794111
-1.133860887326	-1.76340844386
-1.133684534215	-1.763067255233
-1.133455295011	-1.762973181261
-1.133316451678	-1.762709638409
-1.133135959966	-1.762444959113
-1.133001934629	-1.762170176936
-1.132839353973	-1.762018885091
-1.132612841997	-1.761186807985
-1.132438205847	-1.760870645964
-1.1322123467	-1.76082916994
-1.132023783552	-1.76063392652
-1.131903716369	-1.760600619009
-1.131694370883	-1.760465336991
-1.13145712722	-1.760288545786
-1.131273075803	-1.760098740972
-1.131082556158	-1.760063592573
-1.130899581064	-1.759917929965
-1.130679655649	-1.759576706463
-1.130411656349	-1.758473226366
-1.130221535747	-1.758309371321
-1.130011461278	-1.757771568702
-1.12980484238	-1.75735377746
-1.129656700535	-1.757315472768
-1.129482056408	-1.757036917214
-1.129266149934	-1.756830156271
-1.1290692934	-1.756124133233
-1.128895719792	-1.756081496946
-1.128699275684	-1.755968508467
-1.128522370746	-1.755912722562
-1.12832068687	-1.755635606632
-1.128135055656	-1.755157992462
-1.127973360317	-1.75482473859
-1.127757735338	-1.754077507681
-1.127610588414	-1.753952809394
-1.127264370059	-1.75156876466
-1.127102997014	-1.751406794731
-1.126915920975	-1.751146336666
-1.126813882358	-1.751055233225
-1.126606558968	-1.750386866686
-1.126305616781	-1.750020013021
-1.126137920576	-1.749711878885
-1.126011922356	-1.749641110477
-1.125844035315	-1.749639575318
-1.125639303987	-1.749304644439
-1.125439132911	-1.749089787558
-1.125285949329	-1.748785767489
-1.125143535505	-1.748677234044
-1.12492848503	-1.748343475155
-1.124778816835	-1.748223306682

-1.124557544268	-1.747892166404
-1.124391032729	-1.747822947944
-1.124223672696	-1.747435945978
-1.124060810613	-1.746981182628
-1.123912201538	-1.746780871363
-1.123696444266	-1.745920976052
-1.123465093017	-1.745842210077
-1.123287822437	-1.745800442196
-1.123117607107	-1.744949456786
-1.122939395956	-1.744576896804
-1.122755158329	-1.743925368504
-1.12258425556	-1.743873977235
-1.12240466259	-1.743740099213
-1.122219171981	-1.743694964927
-1.122052359916	-1.74366416139
-1.121784697443	-1.742551477169
-1.121640008613	-1.742313496717
-1.121496691826	-1.742224172084
-1.121248361269	-1.741732862286
-1.121075428197	-1.741400776365
-1.120841557219	-1.740666268498
-1.120698983958	-1.740386723546
-1.120563951152	-1.74013415449
-1.120342088716	-1.740080862089
-1.120117441433	-1.739812870834
-1.119930327506	-1.739491273587
-1.119654158943	-1.739455326233
-1.119444021713	-1.739326805249
-1.119210706811	-1.739185489714
-1.118998930387	-1.73901635638
-1.118779567106	-1.738832552386
-1.118558711558	-1.738123063051
-1.118359006366	-1.738056106608
-1.118199961179	-1.737964083132
-1.118024911923	-1.737453978711
-1.117814717529	-1.737192297315
-1.117668541463	-1.737137794595
-1.117539123898	-1.737116157026
-1.117378739372	-1.737061278655
-1.117246342665	-1.736585976853
-1.117025251282	-1.735930390985
-1.116833731673	-1.735821574718
-1.116660312816	-1.735229888585
-1.116478183131	-1.735072191453
-1.116332811847	-1.735007113442
-1.116207712428	-1.734984774473
-1.116019063121	-1.734406878849
-1.115840176475	-1.734277742854
-1.115666427931	-1.733282173817
-1.115414156299	-1.73302615849
-1.115239526063	-1.73300606382
-1.114953847202	-1.732538438684
-1.114777697434	-1.732130837748
-1.11465432817	-1.73196855775

-1.114451831507	-1.731671719626
-1.114265028387	-1.731612674353
-1.113970638604	-1.730079271321
-1.113791184228	-1.729837509384
-1.11362302805	-1.729714225141
-1.113448387644	-1.729217355013
-1.113278851636	-1.728899384544
-1.112984220399	-1.728043545319
-1.11275096762	-1.727434387522
-1.112497814734	-1.72718858991
-1.112371169531	-1.727002786701
-1.112209314049	-1.726577375707
-1.112043044042	-1.726085178474
-1.111779567167	-1.725114277358
-1.111666344319	-1.7250733301
-1.111527486037	-1.724551833533
-1.11135421428	-1.724542926327
-1.111157645147	-1.723901657126
-1.110945834661	-1.723242883041
-1.110741845605	-1.722897208692
-1.110561300724	-1.722632295484
-1.110393735958	-1.722414763519
-1.110154686971	-1.722339055312
-1.109987116495	-1.722191806622
-1.109761078098	-1.722070321914
-1.109561869268	-1.721890092702
-1.109410373188	-1.721577951458
-1.109239485976	-1.720719299608
-1.108965009503	-1.720400531836
-1.108746562118	-1.719524052272
-1.108593392156	-1.71934315891
-1.108437909757	-1.718878043046
-1.108311948233	-1.718719986534
-1.108030099504	-1.716856493221
-1.107870052985	-1.716688588988
-1.107639989754	-1.716185535704
-1.10749379459	-1.715754530605
-1.107330080407	-1.715571799924
-1.107196935362	-1.715339333803
-1.10697009751	-1.714834372004
-1.106795148029	-1.714639551939
-1.106645767488	-1.714490571104
-1.106512684506	-1.714482516563
-1.106328195798	-1.714476066786
-1.10613238138	-1.714399557216
-1.105994963624	-1.714313541226
-1.105788947485	-1.713958418503
-1.10563216275	-1.713670776481
-1.105424501514	-1.712857146307
-1.105220350999	-1.71282323383
-1.105029738789	-1.712475346161
-1.104870716125	-1.71210992068
-1.104717816557	-1.712049911369
-1.104576474179	-1.71191141043

-1.104381124465	-1.71099595635
-1.10422319935	-1.710963770133
-1.104057815	-1.710940349863
-1.103840509116	-1.710068622162
-1.103635204494	-1.709716981302
-1.103457407166	-1.70939959954
-1.103319068886	-1.709341270856
-1.103105212164	-1.709180262863
-1.102935418167	-1.708693461902
-1.102769980314	-1.708216636943
-1.102658430405	-1.708152537478
-1.102519782237	-1.707764398073
-1.102345550542	-1.707521333255
-1.102161171646	-1.707256552286
-1.101923309644	-1.706489826677
-1.101787079827	-1.706455514452
-1.101571240364	-1.706248350418
-1.101319163505	-1.705827392345
-1.101187275297	-1.705601462104
-1.100912071924	-1.705521277564
-1.100703448355	-1.705336816258
-1.100483546941	-1.705075012012
-1.100329170878	-1.704996022331
-1.100106819853	-1.704832349414
-1.099928717522	-1.704815929507
-1.099789511266	-1.704804882325
-1.099623452662	-1.704321693937
-1.099501743071	-1.704179548596
-1.099384655619	-1.704073464902
-1.099067654913	-1.701857429636
-1.098909242383	-1.701676296697
-1.098746696225	-1.701661913609
-1.098586599518	-1.700734287993
-1.09836725245	-1.70044764841
-1.098189884881	-1.700380604925
-1.09798395327	-1.699918323211
-1.097813172448	-1.699706016102
-1.097599318362	-1.699517828823
-1.097460109841	-1.699421737828
-1.09731302556	-1.698875196427
-1.097129522025	-1.698478463317
-1.096918007404	-1.698476201987
-1.096738951549	-1.698291221276
-1.09656196916	-1.697823990859
-1.096398115861	-1.6975175799
-1.096193763509	-1.697454110444
-1.096037355583	-1.697193081585
-1.095841118204	-1.697122013058
-1.095669894449	-1.696819913675
-1.095540751876	-1.696757684581
-1.095415121926	-1.696681012511
-1.09521331576	-1.696144915163
-1.095044992471	-1.695774594083
-1.094918575485	-1.695717407596

-1.094745964271	-1.695245512867
-1.094539980845	-1.694999665438
-1.094400254625	-1.694912638599
-1.094272695121	-1.694388135535
-1.094112995006	-1.694284300648
-1.093908128436	-1.693501801173
-1.093798005034	-1.693451236509
-1.093577007971	-1.692614225332
-1.093398135993	-1.69228992455
-1.093220575176	-1.691871158582
-1.09301583761	-1.691816988954
-1.09288480806	-1.691784695215
-1.092756378359	-1.691621118011
-1.092554067165	-1.691201294274
-1.092388342168	-1.691159723983
-1.092174291722	-1.69057366804
-1.092002451194	-1.690519747609
-1.091851675906	-1.690229740035
-1.091681980189	-1.690066245193
-1.091569859033	-1.689899374666
-1.091389088554	-1.689682072104
-1.091240553843	-1.689670284562
-1.091015837013	-1.68928503452
-1.090829435325	-1.689167736128
-1.090626745501	-1.689142543964
-1.090479005975	-1.688930310546
-1.090324397157	-1.688780992403
-1.090104412982	-1.687819309509
-1.089922990813	-1.687466289567
-1.08975348754	-1.687060754603
-1.08953794214	-1.686229787068
-1.089367273471	-1.686158974827
-1.089088614315	-1.684223312562
-1.088849961372	-1.683710747406
-1.088697102458	-1.683631674924
-1.088495660369	-1.683214939045
-1.088347308984	-1.683037986061
-1.088204251458	-1.683012389722
-1.087912317272	-1.682395923363
-1.087642836705	-1.682296565884
-1.087499462421	-1.682006875479
-1.087358949278	-1.681619834254
-1.08708203101	-1.680086106919
-1.086916785981	-1.67960498662
-1.086718755247	-1.67949790775
-1.086470681006	-1.678907550703
-1.0862849423	-1.678780139788
-1.086135916935	-1.678491891831
-1.085965883641	-1.67824723487
-1.085776393301	-1.6781848112
-1.085577735397	-1.677829792334
-1.08543442919	-1.677526104234
-1.085281585832	-1.677122466085
-1.085102531886	-1.676239361763

-1.084939351269	-1.676148393539
-1.08471380111	-1.675465513221
-1.084582547174	-1.675412345144
-1.084436652737	-1.675049957485
-1.084224753484	-1.674957473843
-1.084072015698	-1.674682788858
-1.08390603789	-1.674584610398
-1.083625341596	-1.674179851201
-1.083439002311	-1.673950098435
-1.083254774474	-1.673945183128
-1.083103856533	-1.67368439776
-1.082960158943	-1.673371536863
-1.082835562023	-1.673209754789
-1.082636940152	-1.672310154759
-1.082472248119	-1.67203442329
-1.082340583333	-1.671883847946
-1.082158240159	-1.671842008606
-1.081872796718	-1.671552012447
-1.081732796687	-1.671131804669
-1.081555060238	-1.670965494818
-1.081409818956	-1.670809663128
-1.081217466074	-1.670716247491
-1.081048219827	-1.670638950952
-1.080799919589	-1.670278969763
-1.080619564104	-1.669648773116
-1.080474554242	-1.669623075013
-1.080311026883	-1.669447432754
-1.080062745325	-1.66900583975
-1.079803513215	-1.668352990245
-1.079644214227	-1.668173035854
-1.079463121861	-1.667458960366
-1.079228545535	-1.666782598916
-1.079097827247	-1.666552561529
-1.078946488444	-1.666258670467
-1.078769577534	-1.666178082581
-1.078507926397	-1.666158148001
-1.078358678848	-1.665921651787
-1.078213652663	-1.665843772464
-1.078005724419	-1.665751470566
-1.077856244603	-1.665601981862
-1.0776523225	-1.665482553123
-1.077511184137	-1.665306490275
-1.077286371402	-1.665127774168
-1.077128684475	-1.665043395597
-1.076980837395	-1.665035372967
-1.07678634851	-1.664243233292
-1.076623664112	-1.663898313998
-1.07642496471	-1.663455945143
-1.076252352983	-1.663105029396
-1.076051065792	-1.662814259197
-1.075923046297	-1.662550434712
-1.075703714169	-1.662469376906
-1.075506246396	-1.662353642912
-1.075310070379	-1.661967010549

-1.075148396879	-1.661901211768
-1.074969822484	-1.661704908274
-1.074767152174	-1.661336990746
-1.074631938625	-1.661205268982
-1.074358514607	-1.660766469894
-1.074096754666	-1.660315878758
-1.073894959567	-1.660110589532
-1.073713699826	-1.659498215461
-1.073510692671	-1.659447659536
-1.073350579382	-1.65939843454
-1.073230244525	-1.659374404732
-1.073130178832	-1.659233801309
-1.072963733831	-1.658929342344
-1.072763478227	-1.658235255613
-1.072543510409	-1.658010198512
-1.072383055148	-1.657913690729
-1.072172660293	-1.657464770783
-1.071987195112	-1.656932883044
-1.07177357903	-1.655463281137
-1.071454781766	-1.653538648561
-1.071332928641	-1.653482002813
-1.07120875708	-1.653426021274
-1.070941054015	-1.652553987954
-1.070760715688	-1.652482542061
-1.070605032248	-1.652056321036
-1.070433116266	-1.651889140883
-1.070309159817	-1.651855099067
-1.070181174456	-1.651699855194
-1.070003313465	-1.651520494827
-1.069842961529	-1.651470108803
-1.069664241886	-1.650830782734
-1.069474013105	-1.650468452924
-1.069280773274	-1.649935920149
-1.069104980735	-1.649928545747
-1.068965390625	-1.649861433786
-1.068803989399	-1.649716308936
-1.068606828747	-1.649188278906
-1.068392687673	-1.648331174564
-1.068222970552	-1.648046617289
-1.068078369147	-1.64803712605
-1.067874820456	-1.647671792318
-1.067664954224	-1.646968687023
-1.067440600938	-1.646623282687
-1.067224496671	-1.646228672895
-1.067061846604	-1.646159972795
-1.066893838444	-1.6458462473
-1.066720132051	-1.645599733807
-1.066521881136	-1.645369482844
-1.066358467433	-1.644844041081
-1.066178400643	-1.644585895681
-1.066026969054	-1.644130109513
-1.065862927871	-1.644053846231
-1.065695480742	-1.643955868754
-1.065562590666	-1.643855490025

-1.065429447749	-1.643416808086
-1.065221358542	-1.642904871672
-1.065053672287	-1.642572067662
-1.064783527155	-1.641762969927
-1.064652912141	-1.641559111715
-1.064428742965	-1.641428931378
-1.064227335375	-1.641173848043
-1.064097473923	-1.640807740472
-1.06387933851	-1.639757791304
-1.063630953429	-1.639582590571
-1.063460840073	-1.638972160707
-1.063299830169	-1.638884889396
-1.063175599925	-1.638751081544
-1.063019804627	-1.638400992158
-1.06281121895	-1.637780874686
-1.062681569075	-1.63762683531
-1.062464729685	-1.637381577357
-1.062306641413	-1.637112863002
-1.062163018852	-1.636948155657
-1.06200569024	-1.636023325031
-1.061893979365	-1.636021507052
-1.061739327616	-1.635588058401
-1.061577260607	-1.635270661513
-1.061374194342	-1.635138785653
-1.061189925069	-1.634638713185
-1.060985717158	-1.634421288174
-1.060837297663	-1.634398422313
-1.060623330602	-1.633827434479
-1.060399167747	-1.633202563728
-1.060288448596	-1.633056871405
-1.06014833804	-1.633043282609
-1.059991458106	-1.632906140174
-1.059728809879	-1.631789919792
-1.05957101722	-1.631464119943
-1.059402111082	-1.631453529217
-1.059190611212	-1.631058281785
-1.059042397135	-1.630874999136
-1.058846168551	-1.630731841627
-1.058697887561	-1.630239044731
-1.058533128531	-1.630222910076
-1.058373318959	-1.629913295238
-1.058214486026	-1.629542301798
-1.058069070257	-1.629468640839
-1.057930285463	-1.62895267478
-1.057740487751	-1.628205587101
-1.057579418366	-1.628045470314
-1.057392073596	-1.627184248615
-1.057198771329	-1.626938423093
-1.057052405238	-1.626787268567
-1.056854563397	-1.626515773595
-1.056701265178	-1.626247735435
-1.056509740965	-1.625350206715
-1.056327560007	-1.625086229161
-1.056162699451	-1.624348090865

-1.05603814403	-1.624281765997
-1.055833626136	-1.623578471071
-1.055658653407	-1.623457042047
-1.055445451415	-1.6233791224
-1.055307317221	-1.623171371937
-1.055174829987	-1.622877923291
-1.055023026783	-1.622484737233
-1.054857066525	-1.622222418901
-1.054677474064	-1.62216493685
-1.05448730621	-1.621385739903
-1.054325198425	-1.621223805966
-1.054169200922	-1.621009214088
-1.053964689232	-1.620517446842
-1.053824547331	-1.620503986633
-1.053694376657	-1.620434425986
-1.053541057313	-1.620239890394
-1.053378849194	-1.620139668267
-1.053238111613	-1.619791615942
-1.05308776412	-1.619512190802
-1.052897495014	-1.619284626034
-1.052722122849	-1.619198497239
-1.052574910095	-1.619110415013
-1.052395403773	-1.618727791796
-1.052265061643	-1.618665447405
-1.052051935066	-1.618080387899
-1.051912350451	-1.618013977196
-1.051735925319	-1.617630223188
-1.051599891581	-1.616991999919
-1.051439881305	-1.616498702771
-1.051269462695	-1.616452640958
-1.051079673355	-1.616362485071
-1.050969238403	-1.616324409666
-1.050753745082	-1.616264875348
-1.050592894616	-1.616186141646
-1.050458473217	-1.615791097185
-1.050248015199	-1.615026727996
-1.050089418929	-1.614978554462
-1.049925266108	-1.614095732436
-1.049813314946	-1.614050634261
-1.049661754796	-1.613781734079
-1.049455871667	-1.613350623691
-1.049294180213	-1.613291980076
-1.049101378373	-1.612373092757
-1.048951038111	-1.61235202796
-1.048837212296	-1.612244710691
-1.048683327539	-1.612014787412
-1.048541467872	-1.611929513389
-1.048371106114	-1.611772502559
-1.048177207368	-1.611176631641
-1.04800028227	-1.610889195043
-1.047858254161	-1.610758481242
-1.047664428198	-1.610744902553
-1.047528663401	-1.61050689087
-1.047343421993	-1.609208165731

-1.047086363536	-1.608994893418
-1.04697455006	-1.608824767165
-1.046776207054	-1.607910902027
-1.046573927538	-1.607453892912
-1.046422345576	-1.607385654876
-1.046239635389	-1.607145187919
-1.046065590606	-1.607118935456
-1.045865848215	-1.607011236212
-1.045711530599	-1.606785247091
-1.045543638167	-1.606763959682
-1.045353104898	-1.606221132717
-1.045161382467	-1.606027344644
-1.04502445982	-1.606002459136
-1.04490008254	-1.605950472355
-1.044794704941	-1.605933680949
-1.044641752193	-1.605824934613
-1.04440111702	-1.605692083359
-1.04427787944	-1.605353220357
-1.044123998231	-1.605111659186
-1.043961197632	-1.604833204416
-1.043808055497	-1.604562313057
-1.043657269748	-1.604510725269
-1.043493069149	-1.604440379124
-1.043318651395	-1.604352618295
-1.043174969094	-1.604345595641
-1.043033376349	-1.604272078448
-1.042847644561	-1.603860123074
-1.04270184815	-1.603806080923
-1.042545089888	-1.603778088545
-1.04238750751	-1.603748371852
-1.042237747896	-1.603516042559
-1.042058191168	-1.603349729898
-1.041906423183	-1.60332626394
-1.041769590351	-1.603293184304
-1.041544989231	-1.602763182313
-1.041383727315	-1.602588534004
-1.041260591249	-1.602483345262
-1.041052738614	-1.602311744044
-1.04080199771	-1.600904002408
-1.040633080903	-1.600848546881
-1.040475569511	-1.600791923085
-1.040289550034	-1.600376160125
-1.040078540091	-1.59999442903
-1.039948867195	-1.599892546545
-1.03981096547	-1.599857535727
-1.039668128636	-1.59972581461
-1.039540887681	-1.599622704915
-1.039390119843	-1.599527501713
-1.039180261579	-1.599423248746
-1.039014209406	-1.599397271703
-1.038882252715	-1.599274961406
-1.03875017326	-1.599139914147
-1.038565374049	-1.598967483609
-1.038415311206	-1.598732221615

-1.038284589969	-1.598692400692
-1.038148808778	-1.598641343272
-1.038024985744	-1.598422903673
-1.037891393131	-1.59807006272
-1.037641201409	-1.59782946549
-1.037494740832	-1.597577887615
-1.037350496842	-1.597327208268
-1.037196185592	-1.597262491777
-1.037050064694	-1.597217856996
-1.036901079129	-1.596983612986
-1.036770219388	-1.596821813024
-1.036566964684	-1.59676948774
-1.036433062515	-1.596584522116
-1.03630043127	-1.596564741216
-1.03616955954	-1.596423978379
-1.035961789788	-1.596002344503
-1.035756789867	-1.595344948711
-1.035591913648	-1.594630124459
-1.035390601389	-1.594469960075
-1.03522533194	-1.59409860323
-1.035060481183	-1.593844439646
-1.034890473341	-1.593730344721
-1.03469543268	-1.593604217425
-1.034528714197	-1.593546059488
-1.034332819561	-1.593170031975
-1.034227498994	-1.593141788331
-1.034094718971	-1.593126505952
-1.033970202993	-1.593039996854
-1.033799090099	-1.593009113139
-1.033606682781	-1.592776480634
-1.033454579677	-1.592659003126
-1.033317572726	-1.592360092125
-1.033168199544	-1.59156229186
-1.033009280288	-1.590639701592
-1.032843559825	-1.59037954963
-1.032638719961	-1.58912446038
-1.032433965885	-1.588528828642
-1.032252836943	-1.588457180688
-1.032058448406	-1.588151497503
-1.031854303207	-1.587645047516
-1.031675941339	-1.587219243228
-1.031515680181	-1.587183702789
-1.031394156132	-1.587134630894
-1.031242253914	-1.587085852883
-1.031097356437	-1.586901178551
-1.030927168825	-1.586418807833
-1.03073291347	-1.585970779274
-1.030518723065	-1.585855293868
-1.030286185014	-1.585456297305
-1.030089911629	-1.585325521943
-1.029931020233	-1.585298463666
-1.029814830753	-1.5852449329
-1.029658851032	-1.585110491369
-1.029433395883	-1.584327529439

-1.029233232666	-1.583910410217
-1.029030881639	-1.58375382259
-1.028868443312	-1.583676510866
-1.028662683925	-1.583259600939
-1.028471668508	-1.582340947222
-1.028329368202	-1.58219315738
-1.028182155772	-1.581955230043
-1.028050290215	-1.581789359219
-1.027893048536	-1.581606668629
-1.027712524568	-1.581097206908
-1.027532066813	-1.580893251694
-1.027373392451	-1.580851529037
-1.027190103346	-1.580305310785
-1.0270629676	-1.580120014701
-1.026833224139	-1.57969496507
-1.026688163508	-1.57955690239
-1.026499934445	-1.579296642982
-1.026387229635	-1.579280358643
-1.026254373985	-1.579214822731
-1.026089055875	-1.579187139387
-1.025867549341	-1.577273260037
-1.025711227189	-1.576991321591
-1.025514819437	-1.576939480958
-1.025289248341	-1.575325718807
-1.025119616389	-1.575258676156
-1.025002634172	-1.575178326154
-1.024776945965	-1.574578284631
-1.024550692273	-1.573813564102
-1.024383290375	-1.573673457078
-1.024229933097	-1.573585832199
-1.02404220918	-1.573241432674
-1.023802829669	-1.572685633468
-1.023644651995	-1.572518714027
-1.023551808854	-1.572506581816
-1.02334391981	-1.572484403786
-1.023197367082	-1.572463748169
-1.023050795747	-1.572374371707
-1.022830575689	-1.57125910461
-1.022664288566	-1.571049786272
-1.022508007067	-1.57084173823
-1.022307290049	-1.570584906769
-1.022199395842	-1.570412648426
-1.021975367219	-1.570278724602
-1.021789722071	-1.570060969684
-1.021643592543	-1.569982659644
-1.021496838502	-1.56948820127
-1.021363480043	-1.569390218983
-1.021209363228	-1.569199623725
-1.021081482872	-1.568960881038
-1.020909571222	-1.568487907442
-1.020749175554	-1.568237894991
-1.020607687142	-1.568169538877
-1.020479318815	-1.567980320019
-1.020290318127	-1.567046713721

-1.020161169509	-1.566883219801
-1.020030767461	-1.566875311976
-1.019942840106	-1.566796342827
-1.019819646358	-1.566664630179
-1.019579362148	-1.566641172761
-1.019444274294	-1.56663566374
-1.019284186496	-1.56628261119
-1.019142116699	-1.566143990656
-1.0189490426	-1.56592087747
-1.018804602733	-1.565497866518
-1.01860553347	-1.564943584122
-1.018465664139	-1.564821129609
-1.018311069153	-1.564743714773
-1.0181698599	-1.564658197878
-1.017946985361	-1.564314818081
-1.0177766604	-1.564107638716
-1.017610316986	-1.564010345035
-1.01744058447	-1.563665142863
-1.017290202419	-1.563610958121
-1.017087946414	-1.563600358499
-1.016892870432	-1.563352262347
-1.01667439411	-1.562414209461
-1.016477779212	-1.561229041768
-1.016312323951	-1.560713415479
-1.016121255079	-1.560562900486
-1.015957620964	-1.560277091172
-1.015776748437	-1.560018828445
-1.015659094038	-1.559614416534
-1.015444763404	-1.559479478717
-1.015320780507	-1.55913655059
-1.015209266621	-1.558674544864
-1.015033452519	-1.558437119811
-1.014895400788	-1.558199930292
-1.014702528322	-1.557775106661
-1.014489972678	-1.55777426354
-1.014374155498	-1.55751079814
-1.014193975623	-1.557293787544
-1.014032553443	-1.557220900748
-1.013843234701	-1.557040369582
-1.0136741459	-1.556995602566
-1.013490805003	-1.556975596921
-1.01334390704	-1.556770005648
-1.01320362402	-1.55664084318
-1.013039550695	-1.556430399902
-1.012838233921	-1.555207579446
-1.012611624457	-1.554856477673
-1.012451020156	-1.554370299617
-1.012302111712	-1.553979965544
-1.012172796613	-1.553701714393
-1.012026563587	-1.553669136092
-1.011841442119	-1.553537451331
-1.011616496555	-1.552787436684
-1.011487405733	-1.552698325007
-1.011336446131	-1.552678729662

-1.011188171587	-1.552585908399
-1.011055900309	-1.552141171949
-1.010928678999	-1.552039169146
-1.010802278814	-1.552034668022
-1.010702612851	-1.551714150962
-1.010556360492	-1.551163266533
-1.010362457752	-1.551143907976
-1.01023664699	-1.550877784759
-1.010048078304	-1.550291299655
-1.009869886774	-1.549377401242
-1.00975126037	-1.549305962115
-1.009594390678	-1.549305039871
-1.009417426379	-1.549044154603
-1.009164762569	-1.547802537755
-1.009056211915	-1.547676251002
-1.008837014126	-1.54739255436
-1.008665015854	-1.547323693788
-1.008504312698	-1.546657701262
-1.008258698051	-1.545712949904
-1.008084965643	-1.545253095337
-1.007900459335	-1.544652103709
-1.007723659432	-1.544007536487
-1.00756762733	-1.54385962831
-1.007439040038	-1.543601089894
-1.007288027561	-1.543094112909
-1.007141251417	-1.542931758676
-1.006985667414	-1.542783855955
-1.006808178492	-1.542689603636
-1.006693744031	-1.542589783607
-1.006498168132	-1.542196317216
-1.006319136464	-1.542162772638
-1.006194434265	-1.541916164301
-1.006011257954	-1.541773740896
-1.005828701557	-1.541567977727
-1.005635815998	-1.540395898615
-1.005443264041	-1.540365325137
-1.005249243384	-1.540116054773
-1.005081783074	-1.539570020087
-1.004927015701	-1.53868507606
-1.004802189939	-1.53868224975
-1.004659546993	-1.538479150085
-1.004522501908	-1.538142255501
-1.004327451293	-1.537625493694
-1.004136809212	-1.537500203668
-1.003932539163	-1.537304092282
-1.003794847913	-1.536792369131
-1.003680123451	-1.536552217574
-1.003525045047	-1.536477658255
-1.003372533866	-1.536460952129
-1.003168896226	-1.536062548359
-1.00295865758	-1.535679157691
-1.002773922451	-1.535620017302
-1.002649539359	-1.535407315941
-1.002489111027	-1.535184507245

-1.002346469518	-1.535168887887
-1.002206907828	-1.535166718461
-1.002047336664	-1.535048450034
-1.001907173851	-1.534908296406
-1.001732191209	-1.534727229276
-1.001497140149	-1.534274333398
-1.001326564014	-1.534043961546
-1.001202828689	-1.53395583288
-1.001074701414	-1.533948674474
-1.000867530746	-1.53333784705
-1.000703459701	-1.533283331157
-1.000459350913	-1.532511006613
-1.000290350924	-1.532142669276
-1.000124160401	-1.531828352909
-0.999878437551	-1.531565711268
-0.999775068848	-1.531564076617
-0.999593739939	-1.53149361795
-0.999416341393	-1.531275484245
-0.999219088438	-1.530880315013
-0.999027097419	-1.530839521823
-0.998816574572	-1.529960128089
-0.998689389645	-1.529944589451
-0.998516869638	-1.529891834179
-0.998392413094	-1.529823496027
-0.998286199512	-1.529701413939
-0.998155486376	-1.529676666263
-0.998038132942	-1.529480676493
-0.997859743693	-1.529072693682
-0.99764636328	-1.528388843471
-0.997498872577	-1.52826956178
-0.997353394583	-1.528203777619
-0.997159036585	-1.527656637294
-0.996927471166	-1.526908809112
-0.996833088858	-1.52688690729
-0.996639566924	-1.526520531277
-0.996534837408	-1.526152081888
-0.996358656534	-1.525567834522
-0.996162589198	-1.525415325415
-0.995998173019	-1.525066643157
-0.995825659458	-1.523992117228
-0.995606446359	-1.523627481114
-0.995423991382	-1.523518225552
-0.995264706545	-1.52337380263
-0.995106864292	-1.523339432486
-0.994969035447	-1.522995205654
-0.994726384413	-1.522622161353
-0.994610615077	-1.522527142388
-0.994447820094	-1.522508066562
-0.994290543674	-1.522326382681
-0.994095486608	-1.522233848599
-0.99387301198	-1.521179182451
-0.993712108045	-1.52104043842
-0.993541154685	-1.520880860584
-0.993433226591	-1.520828518954

-0.993240117881	-1.519835499155
-0.993081878017	-1.519716764299
-0.992927227365	-1.519674211267
-0.992775602925	-1.519275427242
-0.99260999099	-1.519004221436
-0.992387630239	-1.518657390352
-0.99223089496	-1.518410880693
-0.992094800157	-1.518240358857
-0.99193028204	-1.518209158363
-0.991757515967	-1.51779506949
-0.991616117524	-1.517751212415
-0.991444840053	-1.516936861904
-0.991282968773	-1.516671574382
-0.991089676905	-1.516662339063
-0.990875662322	-1.516092171613
-0.990732952486	-1.516047351545
-0.990546853392	-1.516017423174
-0.990384568801	-1.515746147776
-0.990260212185	-1.515680557159
-0.990104418133	-1.515677071044
-0.989968607046	-1.515506519725
-0.98967406485	-1.514149519716
-0.989523195321	-1.51352075543
-0.989323210024	-1.512807746043
-0.989098923624	-1.512603949134
-0.988884583269	-1.511998944621
-0.988738106181	-1.511901257975
-0.988542734794	-1.511768537749
-0.988370361441	-1.511661472561
-0.988264710756	-1.511482499994
-0.988129001292	-1.51132714407
-0.987960803983	-1.511291184869
-0.98774129638	-1.510838911116
-0.987624952365	-1.51077859808
-0.987490482084	-1.510683948335
-0.987314705794	-1.510491330365
-0.98707266712	-1.509433188128
-0.986895257564	-1.508927429372
-0.98676906115	-1.508747056708
-0.986599414229	-1.508729862772
-0.98641991859	-1.507953424065
-0.986235816556	-1.507003542295
-0.986098337427	-1.506591519966
-0.985963091313	-1.506573010586
-0.985694384554	-1.505702875226
-0.985502749376	-1.505678043875
-0.985314627235	-1.505105297032
-0.985067583385	-1.504455222676
-0.984886047297	-1.504376987471
-0.984746398149	-1.504282150521
-0.984580736767	-1.50408051285
-0.984457551385	-1.504058654132
-0.984240377216	-1.503798692258
-0.984084390212	-1.503783748905

-0.983954527479	-1.503627353721
-0.983825454427	-1.503269953563
-0.983672180537	-1.502976324234
-0.98349624889	-1.502956757584
-0.983347096144	-1.502594904311
-0.983198970522	-1.502547262599
-0.983063856407	-1.502120161036
-0.98290666665	-1.502080827713
-0.982815684319	-1.501836005595
-0.982672759789	-1.501795463692
-0.982456157705	-1.501125623428
-0.982216328222	-1.500737592126
-0.982065466165	-1.500609415591
-0.981937756148	-1.500503484301
-0.981764959628	-1.499861855248
-0.981586649753	-1.499562476747
-0.981378281592	-1.499379240969
-0.981240390661	-1.499336641774
-0.981094551833	-1.499311468846
-0.980888562458	-1.498818222831
-0.980676757505	-1.498254117933
-0.980493424477	-1.498132427468
-0.980373714902	-1.497690521966
-0.980192105212	-1.497554526573
-0.980091772608	-1.497416211552
-0.979933359003	-1.497391642254
-0.979725777752	-1.496937856274
-0.979584885985	-1.496326135627
-0.979347881684	-1.495382320605
-0.979050750093	-1.493150505977
-0.978931746284	-1.492900345743
-0.978768778339	-1.492495351623
-0.978578831401	-1.492473245048
-0.978450267571	-1.492366619494
-0.978192975988	-1.491968741822
-0.978016935776	-1.491927469563
-0.977870697465	-1.49189212719
-0.977717835486	-1.491661517544
-0.977580088777	-1.491274871203
-0.977369446386	-1.49047126604
-0.977211089422	-1.490326166857
-0.977064919885	-1.49023553773
-0.976889274311	-1.490072847447
-0.976736941004	-1.48997874233
-0.97660918481	-1.489846024224
-0.976445090269	-1.489052419351
-0.976292310323	-1.488926655196
-0.976125041595	-1.488061510197
-0.975989434094	-1.487898743309
-0.975781806445	-1.487385038296
-0.975671481876	-1.48738079743
-0.975552867076	-1.486998392474
-0.975375820732	-1.486961954809
-0.975255858826	-1.48692930753

-0.975078586584	-1.486715031181
-0.974941381607	-1.486707077505
-0.974776157908	-1.486543662167
-0.974681028413	-1.486427697638
-0.974475363129	-1.48607747284
-0.974328237334	-1.486055315067
-0.974166079968	-1.485516025062
-0.97398431406	-1.485512980088
-0.973841929932	-1.485285626831
-0.973705619464	-1.485168264617
-0.973484970525	-1.48454691927
-0.973357535039	-1.48414406677
-0.973171482686	-1.484125784902
-0.972994138352	-1.484024403355
-0.972858851626	-1.483751539518
-0.972710599027	-1.483532417952
-0.972552769914	-1.483319802784
-0.972418323606	-1.483199868219
-0.972294860186	-1.48282250912
-0.972165340259	-1.482716174699
-0.972005506107	-1.482702451311
-0.971839002556	-1.482660708806
-0.971691065681	-1.482371344634
-0.971520614124	-1.482249519226
-0.971329940356	-1.48219774871
-0.971168288747	-1.481535673555
-0.970990269588	-1.481496098798
-0.970814633441	-1.481125068316
-0.970709157563	-1.481094846593
-0.97052096569	-1.480295282715
-0.970378764184	-1.480283256292
-0.970221825852	-1.480202574137
-0.970104179838	-1.480036363672
-0.969948359309	-1.479480500631
-0.969781159287	-1.479255151294
-0.969631970858	-1.479089554192
-0.969395541524	-1.478625046046
-0.969221634365	-1.478438577661
-0.969103110535	-1.47835869957
-0.96895800103	-1.477935593451
-0.968797256231	-1.477514229134
-0.968635223164	-1.47735279005
-0.968454880654	-1.477169953105
-0.968277823674	-1.476284811687
-0.968130058154	-1.476012543781
-0.967996807909	-1.475616312001
-0.967869783726	-1.475010205021
-0.967747906199	-1.474993000585
-0.967545064219	-1.474450368686
-0.96742578263	-1.474191429282
-0.967243122855	-1.474142077591
-0.967089274912	-1.474104656232
-0.966928756499	-1.474093303465
-0.966766395494	-1.473335572075

-0.966587379629	-1.472303525488
-0.966415711844	-1.472237932788
-0.966296781799	-1.471867343565
-0.966118064409	-1.471716473896
-0.965966301084	-1.471393194767
-0.965789973289	-1.470909788765
-0.965629125328	-1.470464611043
-0.965433082304	-1.470349423549
-0.96529204054	-1.470062590495
-0.965146180716	-1.469765067125
-0.964966090376	-1.469741112139
-0.964795771153	-1.469414517329
-0.964616614621	-1.46929447528
-0.964464670768	-1.469167871912
-0.964281695262	-1.46896320122
-0.964180576452	-1.468734854248
-0.963986167879	-1.468631107646
-0.963823747881	-1.468214826403
-0.963688295406	-1.468142855255
-0.963550456771	-1.46813176368
-0.963333752166	-1.467918541533
-0.963108241818	-1.466633102577
-0.962948864626	-1.466459223716
-0.962719209199	-1.466014271275
-0.962635214204	-1.465983973829
-0.96246453944	-1.465362631138
-0.962333529426	-1.465198182091
-0.96217437494	-1.464861245277
-0.961987143831	-1.464095488805
-0.961798409013	-1.463741279002
-0.961668320511	-1.463670414009
-0.961514759381	-1.463574446828
-0.96134465347	-1.462902831677
-0.961138592038	-1.462199897184
-0.961047130497	-1.46214983861
-0.960859232595	-1.462059192708
-0.960739999712	-1.462059152514
-0.960558775068	-1.461468329403
-0.960426885487	-1.461444377572
-0.960282767847	-1.460836161146
-0.960108491966	-1.460590357155
-0.959928984492	-1.459542075554
-0.959799964568	-1.45933718167
-0.959636436371	-1.459067284629
-0.959444951444	-1.458682943539
-0.959316785085	-1.458606947699
-0.959143065759	-1.458236448333
-0.958987710635	-1.458103493451
-0.95881832877	-1.45784263923
-0.958687835941	-1.457565671391
-0.958547167447	-1.457420764917
-0.958457002492	-1.457343322711
-0.958237982162	-1.456474210289
-0.958135530205	-1.456398112027

-0.958017777923	-1.456286158385
-0.95786098421	-1.455989584637
-0.957671226805	-1.45578496141
-0.957558930005	-1.45571051411
-0.957428163519	-1.455503382581
-0.957294693562	-1.455294194881
-0.957114181338	-1.455252272947
-0.956995204782	-1.455176724038
-0.956778094902	-1.454807549264
-0.956674339718	-1.454787071153
-0.956530367684	-1.454683384961
-0.956337720666	-1.454214137137
-0.956187676433	-1.454199062656
-0.956014593724	-1.453744411923
-0.955873645974	-1.45338656478
-0.955688693173	-1.453341237646
-0.955529708362	-1.452858401009
-0.9553552662	-1.452439534069
-0.955209289201	-1.452253730364
-0.955042523395	-1.452099212887
-0.954893973385	-1.452046397714
-0.95477388146	-1.452043060793
-0.954622611897	-1.45172559793
-0.95448510233	-1.451717287081
-0.95435475096	-1.451713337214
-0.954225672189	-1.451588306678
-0.954092534262	-1.451467467162
-0.953979533014	-1.451322880255
-0.953861308587	-1.451196374902
-0.953676109117	-1.451182539163
-0.953495980269	-1.45112176371
-0.953344721314	-1.450933268667
-0.953135388747	-1.450580525033
-0.952890126747	-1.449397626138
-0.952740114017	-1.449392751207
-0.952592127604	-1.449107895466
-0.952399462984	-1.449002528201
-0.952216139505	-1.448774419717
-0.952108705442	-1.448563690967
-0.95194863439	-1.448435052304
-0.951791902603	-1.448261858348
-0.951660779273	-1.44778781482
-0.951502092053	-1.44761964959
-0.951361632182	-1.447149104795
-0.951253816096	-1.447056584388
-0.951133714142	-1.446790085563
-0.950972714279	-1.446528442696
-0.950765652869	-1.446339222303
-0.950562343442	-1.446047964214
-0.950440671537	-1.445977742365
-0.950304655018	-1.445961828939
-0.950143675873	-1.445362426027
-0.949942784633	-1.445304292521
-0.949785509186	-1.445242782432

-0.949650593162	-1.445200320295
-0.949532421308	-1.444989989811
-0.949384670222	-1.444932144912
-0.949209432765	-1.444910907487
-0.949084629925	-1.444622596541
-0.948977144361	-1.44455026924
-0.948769411009	-1.443415829578
-0.948586713141	-1.442741901604
-0.948414544918	-1.442574336687
-0.948237260327	-1.442574188677
-0.948093374724	-1.442272234086
-0.947983445918	-1.44218316178
-0.947871156528	-1.442166359936
-0.947654955139	-1.442044719494
-0.947561000262	-1.441941251341
-0.947402742746	-1.441901085185
-0.947287516437	-1.441897161703
-0.947115480115	-1.441745180498
-0.946993286154	-1.441596800163
-0.946828318538	-1.441172188591
-0.946696921648	-1.441091548417
-0.94654953913	-1.440656541601
-0.946388660018	-1.440353012311
-0.94611962015	-1.439351485669
-0.94601248085	-1.439256518337
-0.945848490004	-1.439219269797
-0.945709878834	-1.439190082955
-0.945576996799	-1.439186129237
-0.945439168463	-1.439045588511
-0.945293572743	-1.438984351774
-0.945110019316	-1.438907999261
-0.944980360677	-1.438753282477
-0.944830420567	-1.438196170532
-0.944634497679	-1.437418324617
-0.944478794798	-1.437395601225
-0.944357733547	-1.437365057338
-0.944190047899	-1.436961355499
-0.944028399941	-1.436597136391
-0.943879002189	-1.436584999628
-0.943684834911	-1.435462093097
-0.943567069085	-1.435407801266
-0.943401807471	-1.435003689944
-0.943222946711	-1.434737877688
-0.943073978318	-1.434719835166
-0.942914165597	-1.434492978008
-0.942744959199	-1.434226233292
-0.942577191263	-1.434024136184
-0.94245279973	-1.433861272158
-0.942292387182	-1.43371075161
-0.942127877744	-1.433618523651
-0.941980561704	-1.432929986135
-0.941834308262	-1.432742840389
-0.94158290417	-1.432345981192
-0.941443913076	-1.432008868154

-0.941250897581	-1.431643900133
-0.941017040271	-1.430881045819
-0.940845669108	-1.4305878223
-0.940728962669	-1.43058551165
-0.940536033608	-1.430287322444
-0.940401104689	-1.430118690899
-0.940202403187	-1.430091044043
-0.940056298403	-1.42981467679
-0.939860628321	-1.429487402899
-0.939687203594	-1.428870749505
-0.93954605789	-1.428827034934
-0.939354886226	-1.428560099402
-0.939199270117	-1.427993217443
-0.938971397565	-1.427438980314
-0.93880406356	-1.427364368847
-0.938685571178	-1.426767462075
-0.938550856727	-1.426439668859
-0.938427846332	-1.426415704844
-0.938210906588	-1.42561847144
-0.937997959748	-1.425064627783
-0.937810909691	-1.424629695576
-0.937657959567	-1.424571992078
-0.937509728488	-1.424532073059
-0.9373273831	-1.424111528884
-0.937149043395	-1.424029109645
-0.936878612439	-1.423638530506
-0.936749180696	-1.423300123016
-0.936570638305	-1.423125789426
-0.936411516079	-1.423021257053
-0.936240731401	-1.422939812854
-0.936108632538	-1.422751162724
-0.935997054998	-1.422713589886
-0.935857494706	-1.422623332518
-0.935685466602	-1.422354160392
-0.935553838166	-1.421726300328
-0.93540147866	-1.421673693874
-0.935263276494	-1.421654434464
-0.935134192405	-1.421519290794
-0.935002015273	-1.421313190413
-0.934902357581	-1.421270847014
-0.93476395103	-1.421237269551
-0.934662553667	-1.420989259257
-0.934490261783	-1.420705563831
-0.934350892079	-1.420542092678
-0.934196889522	-1.420527433793
-0.93408747514	-1.420342948624
-0.933969587989	-1.420254152518
-0.933787048353	-1.419998797312
-0.933663776651	-1.419892621717
-0.933483895769	-1.419703962392
-0.933324443292	-1.419537253803
-0.933182384019	-1.419438155708
-0.933068843256	-1.41911756326
-0.93290346229	-1.418903609118

-0.932736054588	-1.418853111464
-0.932495967965	-1.41778748047
-0.932352378468	-1.417312907205
-0.932197736613	-1.417026971114
-0.932002431215	-1.416602163419
-0.931840211393	-1.416474099621
-0.931676812204	-1.416399340179
-0.931503664128	-1.416327456476
-0.931363001089	-1.416294046963
-0.931212345997	-1.416114283712
-0.931055380588	-1.415568979791
-0.930862015441	-1.415412417207
-0.930674407153	-1.415127097686
-0.930481191554	-1.414872717569
-0.930302525731	-1.414751964671
-0.930130640517	-1.414070732173
-0.930006458624	-1.414061744468
-0.929818689032	-1.414059036289
-0.929678757135	-1.413861293545
-0.929509456179	-1.413105649973
-0.929339738327	-1.41310418565
-0.929189149391	-1.412564746567
-0.929069348639	-1.412530443626
-0.928906034216	-1.412108283976
-0.928728536994	-1.411585643766
-0.928556540559	-1.411244448916
-0.928419426389	-1.41124266539
-0.928249888016	-1.410865429265
-0.92810859434	-1.410774413503
-0.92794653289	-1.410760216914
-0.927757836932	-1.410075147205
-0.927561705863	-1.409541963386
-0.927447140411	-1.409486022505
-0.927358366245	-1.409422977152
-0.927185851821	-1.409416234438
-0.927038769035	-1.409347197217
-0.926851460176	-1.408769869244
-0.926719011976	-1.40866278497
-0.926614778466	-1.408523018123
-0.926444155326	-1.408521780539
-0.926342269264	-1.408103464911
-0.9261646284	-1.40785031359
-0.926055799007	-1.407434830088
-0.925918841318	-1.40737701164
-0.925839966741	-1.407128337786
-0.925649260047	-1.406828033522
-0.925454557445	-1.406000498115
-0.925247011775	-1.405964387625
-0.92508459607	-1.405669147181
-0.92493985947	-1.405557546017
-0.924734091385	-1.405247498882
-0.924596079186	-1.405196406831
-0.924393190076	-1.40488107916
-0.924244431994	-1.404844843607

-0.924097301181	-1.404829691851
-0.923927962631	-1.404770082103
-0.923739600387	-1.404449020838
-0.923533999012	-1.404445895125
-0.923372119596	-1.404405235745
-0.923202334682	-1.404329042303
-0.923083392326	-1.404322104762
-0.922951592588	-1.404268526068
-0.922792751743	-1.403890657557
-0.922578485163	-1.403811358244
-0.922429351485	-1.403511018522
-0.922259654318	-1.403213414935
-0.922103288228	-1.40261415818
-0.921919892448	-1.402321705746
-0.921767011829	-1.402219518252
-0.921635938386	-1.401965594972
-0.92143350366	-1.400863221201
-0.921275312545	-1.400789500833
-0.921059047888	-1.399986977335
-0.92090078671	-1.399714511698
-0.920670679675	-1.398929928867
-0.920482993463	-1.398399280067
-0.920361442126	-1.398349453044
-0.920249553047	-1.398317131108
-0.920094007771	-1.398092539021
-0.919952035201	-1.397905769694
-0.9197755105	-1.397494032313
-0.919634999028	-1.397202448549
-0.919496663595	-1.396779151938
-0.919351991985	-1.396620009942
-0.919144732092	-1.395483877801
-0.919062830601	-1.395454170545
-0.918913105551	-1.395213741295
-0.918771478735	-1.395211061531
-0.918641505545	-1.394759178258
-0.918466734149	-1.394379716045
-0.918362325568	-1.394337329986
-0.918164257645	-1.394271498054
-0.918001269916	-1.393090409794
-0.91781485348	-1.39281468863
-0.917654651141	-1.392812280993
-0.917531872828	-1.392589639721
-0.91739068387	-1.392578728025
-0.917205766281	-1.392218483179
-0.917034702426	-1.39206893309
-0.916870726519	-1.391755778344
-0.91667927974	-1.391563086477
-0.916539471117	-1.391473925106
-0.91635189748	-1.391197187869
-0.916207782855	-1.391055759404
-0.916088764801	-1.390844972481
-0.915951653377	-1.390748777544
-0.915754478265	-1.38979091917
-0.915592095183	-1.389600415605

-0.915498136796	-1.389574949051
-0.915367944757	-1.389206858816
-0.915204335919	-1.388775166869
-0.915037092828	-1.388696727704
-0.91483587787	-1.388440592316
-0.914639004665	-1.388297270237
-0.914487385664	-1.388180784868
-0.914356409851	-1.387393377331
-0.914256751461	-1.387363597673
-0.914121382259	-1.386923266801
-0.913985231866	-1.386850137131
-0.913851553168	-1.386708291473
-0.913711139378	-1.386683939764
-0.913562463803	-1.386434176192
-0.913416944123	-1.386088310873
-0.913269206162	-1.386083989176
-0.913167056452	-1.386072732305
-0.913032159252	-1.386068599739
-0.912851673612	-1.385910778695
-0.912743111278	-1.385885190939
-0.912606348737	-1.38541534365
-0.912390491514	-1.385053601892
-0.912255620534	-1.384764704772
-0.912072118242	-1.384580851837
-0.911934715095	-1.384223172486
-0.911771017448	-1.384037565613
-0.911643167719	-1.38387726003
-0.911517087675	-1.383750460613
-0.911375984544	-1.383491838283
-0.911249214859	-1.383319614546
-0.911144267275	-1.383306199643
-0.910969631575	-1.383250141722
-0.910826542382	-1.3828706149
-0.910664242804	-1.382670681485
-0.910511762101	-1.382649049777
-0.910378868943	-1.382485253569
-0.91023324586	-1.382444797251
-0.910107053444	-1.382404230933
-0.909959421552	-1.38208896473
-0.909774073514	-1.382001757163
-0.909526255193	-1.380621637568
-0.909414320555	-1.380588122843
-0.909237932735	-1.380112760687
-0.90908853645	-1.379898363742
-0.908915164823	-1.379386193598
-0.908774359412	-1.379039514433
-0.9086667192	-1.378859399778
-0.908502604288	-1.3783586891
-0.908323111139	-1.377567440741
-0.908168072142	-1.377206289849
-0.908009419339	-1.376701686636
-0.907893042382	-1.376609948311
-0.907773875873	-1.376465048917
-0.9076449631	-1.376393943544

-0.907525943144	-1.376344153445
-0.907393665181	-1.376134637929
-0.907248675275	-1.376023254364
-0.907103902398	-1.37590103131
-0.907001983997	-1.375792160149
-0.90681633109	-1.374797587046
-0.906648645751	-1.374761991587
-0.906510660431	-1.374631633669
-0.906349999794	-1.374479402412
-0.906228887358	-1.374430115623
-0.905993808678	-1.374065905956
-0.90582978117	-1.373924863914
-0.90566463415	-1.373802372394
-0.905526697549	-1.373788038043
-0.905368891551	-1.373392947594
-0.905190973279	-1.373245491315
-0.905047907804	-1.372953427048
-0.904887377267	-1.372666745074
-0.904762646476	-1.372662824639
-0.904636774434	-1.372511583585
-0.904524992309	-1.372315332439
-0.904367093177	-1.372191569316
-0.904232231534	-1.372079933998
-0.904059977411	-1.371347155453
-0.903962273652	-1.370976564871
-0.903802659766	-1.370909563118
-0.903682566702	-1.370897317318
-0.903497487941	-1.370541134997
-0.903389860839	-1.370433143449
-0.903238410109	-1.370055901748
-0.903105915978	-1.369952659584
-0.902963590986	-1.369904305708
-0.902712787993	-1.369827242105
-0.90255605444	-1.368807654898
-0.902410289533	-1.368707132981
-0.902232370709	-1.368201097298
-0.902086516235	-1.367949674343
-0.90193973141	-1.367902678553
-0.901781524687	-1.367788071389
-0.901673308775	-1.367722132775
-0.901573325067	-1.367422078681
-0.901381543575	-1.367066640193
-0.901256833457	-1.366983740901
-0.901130576168	-1.36678124937
-0.900955151399	-1.366689377649
-0.900834635307	-1.366635035269
-0.900697148931	-1.365966955442
-0.900549297543	-1.365771547443
-0.900423666152	-1.365716221752
-0.900277798207	-1.365326527573
-0.900118117566	-1.365147211046
-0.899940516076	-1.36501604381
-0.899807201379	-1.365009282718
-0.899712173104	-1.364947788775

-0.899597582595	-1.364812751629
-0.899452027221	-1.364784769322
-0.899302404157	-1.364773251694
-0.899173906074	-1.364645991161
-0.898990108744	-1.364456850753
-0.898830923182	-1.364239858966
-0.898682765048	-1.3640821854
-0.898541120741	-1.364032119232
-0.898357848544	-1.363688055736
-0.898216398889	-1.363481708557
-0.898077574576	-1.363306087767
-0.897927444964	-1.363284135911
-0.89776060851	-1.363219042858
-0.897582937117	-1.362745129605
-0.897429369626	-1.362495609709
-0.89728401346	-1.362408798226
-0.897111767102	-1.361579974038
-0.896967239671	-1.361050048328
-0.896847636543	-1.361018520248
-0.896679357378	-1.360560258782
-0.896594477763	-1.360351819852
-0.896421358917	-1.360342803482
-0.896292175463	-1.360183188466
-0.896138038996	-1.360070028431
-0.896001504583	-1.359262872665
-0.895826441649	-1.359181806459
-0.895690984383	-1.359083596228
-0.895553540901	-1.358850603972
-0.895451925717	-1.358816476899
-0.895314019907	-1.358391543043
-0.895121686825	-1.358298435713
-0.894950206821	-1.357650937007
-0.894697506071	-1.356972387175
-0.894498910035	-1.356637493556
-0.894371183003	-1.355905671312
-0.894244263609	-1.355585334303
-0.894062991645	-1.355305712577
-0.893867389739	-1.355067220206
-0.893721318821	-1.354919113343
-0.893598327462	-1.354801329901
-0.893455572677	-1.35456604367
-0.893297053634	-1.354526767251
-0.893151664591	-1.354375091948
-0.893024852054	-1.354023473508
-0.892923868715	-1.353986393667
-0.892819695955	-1.353979703648
-0.892714017876	-1.353842580889
-0.892555014227	-1.353824923084
-0.892394075428	-1.353626421254
-0.892290382955	-1.353599719279
-0.892168165718	-1.353531276589
-0.892011840364	-1.353003628011
-0.891787832999	-1.35253443618
-0.891635917644	-1.352362787088

-0.891529769796	-1.352333894312
-0.891372643401	-1.3521292205
-0.891223521911	-1.35182199699
-0.891062830969	-1.35180242021
-0.890885950477	-1.35152652118
-0.890716231092	-1.351408330409
-0.890550727088	-1.351282066335
-0.890377770742	-1.350467954743
-0.890233584667	-1.35045992267
-0.890138556971	-1.3504575363
-0.890009782168	-1.350211058438
-0.889894012392	-1.350148478337
-0.889723293801	-1.350130289598
-0.889558961016	-1.350075345245
-0.889433039376	-1.349978367875
-0.889345494001	-1.34995430526
-0.889195445214	-1.349585420902
-0.88902857341	-1.349479962603
-0.888885516828	-1.349253074431
-0.888744132838	-1.349106422191
-0.888521422432	-1.348627973448
-0.888353916375	-1.348501023367
-0.888202030554	-1.348362011549
-0.888035226783	-1.348099135516
-0.887874840311	-1.346953830449
-0.887727794234	-1.346758576203
-0.887589635073	-1.346643015649
-0.887405462502	-1.346510610674
-0.887265788689	-1.34650544125
-0.887143236461	-1.346298961562
-0.886975175671	-1.346282235701
-0.886863281924	-1.346210395993
-0.886646876635	-1.344780107187
-0.886531511745	-1.344718610475
-0.886398513289	-1.344421485224
-0.886265268744	-1.344140611911
-0.886121630616	-1.343997813701
-0.886009462649	-1.343798705834
-0.885872286346	-1.343607388997
-0.885718133484	-1.343555955175
-0.885577927359	-1.343310507575
-0.885463319569	-1.343152390241
-0.885313257924	-1.343037643452
-0.885165938974	-1.342972392329
-0.885012235434	-1.342726965081
-0.884836320192	-1.34232599708
-0.884704009288	-1.342255710094
-0.884539536172	-1.34198515203
-0.884438939617	-1.341653818401
-0.884304240444	-1.341575481546
-0.884229145115	-1.341429137672
-0.884082751135	-1.34128034154
-0.883906844927	-1.340215625264
-0.883792989862	-1.340133282099

-0.883631115973	-1.340068776441
-0.883485215104	-1.339471270981
-0.883327855455	-1.339137857818
-0.883162785269	-1.33906508211
-0.882929860881	-1.338510263748
-0.882785314523	-1.338373069604
-0.882591437153	-1.338144047011
-0.882491410671	-1.338062925928
-0.882408707236	-1.338002942359
-0.882204425949	-1.337633938233
-0.881947998567	-1.337367894409
-0.881812196258	-1.337225841896
-0.881697612948	-1.337004117392
-0.881557507939	-1.336766850906
-0.881407598982	-1.336610621358
-0.881257296104	-1.336318399056
-0.88109997214	-1.336102894506
-0.880973275552	-1.33592700599
-0.880831504317	-1.33581768518
-0.880672503073	-1.335358982367
-0.880563505049	-1.335214868042
-0.880427965328	-1.335085842504
-0.880266831157	-1.334624404904
-0.88014325799	-1.334210360091
-0.879991742673	-1.334179751096
-0.879833313051	-1.334021332438
-0.87971963754	-1.333934597697
-0.879565044917	-1.332981324793
-0.87941324531	-1.332743913468
-0.879268071299	-1.332468326176
-0.879090276872	-1.331902823792
-0.878963396507	-1.331869994021
-0.878807413507	-1.331858363397
-0.878631131297	-1.331505347809
-0.878415635144	-1.330549175579
-0.878305457233	-1.330528795139
-0.878195290104	-1.330491376682
-0.878077766451	-1.330424292433
-0.87797122121	-1.330378398017
-0.877748403792	-1.329739818062
-0.877624627543	-1.329520618515
-0.877421892686	-1.328841543408
-0.877302966798	-1.328603937872
-0.877131606691	-1.328602916335
-0.877018402309	-1.32839066204
-0.876846902486	-1.328282947398
-0.876693541726	-1.328194069869
-0.876562117546	-1.328080113702
-0.876445354938	-1.328052134861
-0.876265326202	-1.327879333476
-0.87609751294	-1.327263912644
-0.875953889831	-1.327193050484
-0.875787780295	-1.327142089785
-0.875639741544	-1.32702409214

-0.875538885305	-1.327002836409
-0.875383461561	-1.326713031956
-0.87528073102	-1.326676330943
-0.875149485035	-1.326246448194
-0.875021595616	-1.325774558548
-0.87486069944	-1.32503116065
-0.874635795328	-1.324843214154
-0.874484830115	-1.324558902549
-0.87427256321	-1.323495828995
-0.874102952208	-1.323074498926
-0.873981793596	-1.323052886106
-0.873827193435	-1.322959418386
-0.873703060248	-1.322594106939
-0.873536272774	-1.322582327464
-0.873438340439	-1.322387279056
-0.87333041127	-1.322364839544
-0.873196362326	-1.322301659424
-0.873076843859	-1.322223709008
-0.872941423892	-1.32218323819
-0.872831884629	-1.321902190948
-0.87271210399	-1.321774249226
-0.872600778648	-1.321735262074
-0.872491490837	-1.32168206582
-0.872381232092	-1.321649930518
-0.87221872524	-1.32121981051
-0.872107669555	-1.321215755939
-0.871988732235	-1.320969165175
-0.871862760098	-1.320882875481
-0.871669599136	-1.320620817367
-0.871490744836	-1.320162127549
-0.871327318554	-1.319758934216
-0.871166856176	-1.319213628262
-0.87105493047	-1.319155539867
-0.870936757567	-1.319138666052
-0.870837997304	-1.319084993549
-0.870719380003	-1.318940423252
-0.870606981728	-1.318802056445
-0.870465724816	-1.318706434045
-0.870338330835	-1.318065536751
-0.870184545616	-1.317551119887
-0.87005966321	-1.317548244048
-0.869924394065	-1.317522299894
-0.869791105869	-1.317376241963
-0.86961090484	-1.317340069311
-0.869457901132	-1.317070201013
-0.869266562564	-1.316756715476
-0.869063508321	-1.31635476575
-0.868922310981	-1.315964237385
-0.868737756868	-1.315200463342
-0.868553746056	-1.314794198896
-0.868419567735	-1.31475573094
-0.868240878126	-1.314647378883
-0.868040112777	-1.314596275359
-0.867908936329	-1.314364427656

-0.867782302011	-1.31407241167
-0.867665573503	-1.313991815196
-0.867508369819	-1.313725456921
-0.867398052284	-1.313678714789
-0.867208145018	-1.313254969782
-0.86704941217	-1.312696291681
-0.866940599444	-1.312661869958
-0.866783418892	-1.312068468806
-0.866663899965	-1.311955139205
-0.866498275065	-1.311909172063
-0.866355540282	-1.311903303499
-0.866219006879	-1.311723274146
-0.86608786107	-1.311106512262
-0.865885458528	-1.310817233838
-0.865774612662	-1.310767912897
-0.865614947133	-1.310628817418
-0.865308044223	-1.309479643498
-0.865165917341	-1.308957117604
-0.865016878623	-1.308523702534
-0.864862233098	-1.308488106404
-0.864655198902	-1.308205803686
-0.864483218763	-1.308203970748
-0.864302877496	-1.307997861393
-0.864205559797	-1.307919083487
-0.864029113812	-1.307539681163
-0.863896550868	-1.307347639948
-0.863819586846	-1.307337750787
-0.863660590558	-1.30724539667
-0.863513268746	-1.307045752169
-0.863333960173	-1.306826611749
-0.86318549703	-1.306611741244
-0.86303626697	-1.306573688652
-0.862922767809	-1.306485450276
-0.862701786449	-1.306403298765
-0.862577209552	-1.306277426853
-0.86242485765	-1.305977824801
-0.862279149789	-1.305652054974
-0.86215148322	-1.305563417236
-0.862001207355	-1.305523611704
-0.861879664489	-1.305220426224
-0.861730844587	-1.305150593158
-0.861574633662	-1.304816710784
-0.861426276174	-1.304526571466
-0.861301221885	-1.304424904637
-0.861125584696	-1.303608029983
-0.860962938583	-1.303356383725
-0.860814874066	-1.303324068773
-0.860706119035	-1.303067454258
-0.86055040669	-1.302135916206
-0.860401508817	-1.302052881407
-0.860250359606	-1.301825225707
-0.860114839613	-1.301750193242
-0.859996234872	-1.301538578956
-0.859857396815	-1.301513756152

-0.859687189072	-1.301246811902
-0.859505891456	-1.301075179236
-0.859406930419	-1.301035963942
-0.85931796454	-1.300999194032
-0.859181800423	-1.300474888963
-0.859068273404	-1.300379541208
-0.858934872615	-1.300240634062
-0.858810831735	-1.299915856838
-0.858683928572	-1.299710353172
-0.858528037672	-1.298893845547
-0.85839883932	-1.298852327017
-0.85821282213	-1.298154510456
-0.858077973585	-1.297699409685
-0.857947099485	-1.297668892037
-0.857761930516	-1.297652598672
-0.857632698711	-1.297464117421
-0.857484621884	-1.296982372984
-0.857328469165	-1.296863418216
-0.857200858151	-1.296750537796
-0.85704650472	-1.296179517586
-0.856923331788	-1.296062530478
-0.856702334097	-1.295768743325
-0.856600654668	-1.295750660764
-0.856414703085	-1.295328716319
-0.85621452181	-1.295258760101
-0.856016049549	-1.294806429279
-0.855898402467	-1.294800594181
-0.855754903542	-1.294616197505
-0.855554670554	-1.294347064144
-0.855375411488	-1.294311336775
-0.855238456185	-1.293851938468
-0.855086307423	-1.293830518932
-0.854932537418	-1.293730673779
-0.854829953997	-1.293706809435
-0.854653538862	-1.29356022906
-0.854533852622	-1.293434499001
-0.854419697714	-1.293432467705
-0.854305110191	-1.293260500295
-0.854172392773	-1.29310625493
-0.854012994319	-1.29293147212
-0.853830769174	-1.292850673559
-0.853700702089	-1.292488122085
-0.853553791841	-1.292349198263
-0.853425194177	-1.29211584132
-0.85327281729	-1.291645413244
-0.853162098565	-1.2914220696
-0.85296438675	-1.290682768297
-0.852823159665	-1.290103412072
-0.852717905007	-1.290065331758
-0.852580716389	-1.289847561404
-0.85247588866	-1.289533114501
-0.852357590649	-1.289372060525
-0.85222953402	-1.289267388919
-0.852044136086	-1.288449980045

-0.851901732641	-1.288430613844
-0.851752665841	-1.287682214014
-0.851603476408	-1.287235694041
-0.851472612773	-1.286998198235
-0.85135672445	-1.286631683868
-0.851179355288	-1.286604938105
-0.851036655259	-1.286437820729
-0.850877251063	-1.285983651385
-0.850740731198	-1.285945295631
-0.850571202762	-1.285772975921
-0.850446497561	-1.285504669368
-0.85032703965	-1.285396505611
-0.850226314399	-1.285333806403
-0.850057343085	-1.285069258322
-0.849921985192	-1.284897949466
-0.849793258079	-1.284809093257
-0.849654088692	-1.28459483076
-0.849559021947	-1.284452901985
-0.849440828959	-1.284137179727
-0.849297139038	-1.284109483189
-0.849159666191	-1.284055971719
-0.849047625718	-1.283997835161
-0.848929123267	-1.283928163421
-0.848764515319	-1.283535194641
-0.848635828805	-1.283058933133
-0.848507253943	-1.282935666402
-0.848320857602	-1.282295821745
-0.848182636442	-1.282207682511
-0.848041772567	-1.281510873815
-0.847917967723	-1.281375246801
-0.847774371821	-1.281343592628
-0.847673132098	-1.281281767653
-0.847508762219	-1.281278530893
-0.847355558509	-1.281034492282
-0.847165424411	-1.280753130772
-0.847024750963	-1.280585012515
-0.846883547882	-1.2805444691
-0.846759318919	-1.280452165567
-0.84661167963	-1.28029669304
-0.846464253538	-1.280273088169
-0.846335273827	-1.280021154899
-0.846204889574	-1.279898855969
-0.846022426048	-1.279284877868
-0.845858511576	-1.27910899031
-0.845766062469	-1.279043426823
-0.845576806207	-1.278863041697
-0.845453041908	-1.278391549402
-0.845309498408	-1.278274043503
-0.84509843059	-1.276973223367
-0.844918180537	-1.276559946529
-0.844729287995	-1.275933905474
-0.844560337964	-1.275923565583
-0.844397001906	-1.275705735576
-0.844252924462	-1.275582435732

-0.84412981103	-1.275502755951
-0.844026828414	-1.275391942412
-0.843884645855	-1.275234160215
-0.843744955924	-1.275068698225
-0.843626822878	-1.275044584839
-0.843527119144	-1.274891281997
-0.843346207497	-1.274339844076
-0.843243597382	-1.274198907362
-0.843096031312	-1.274005032798
-0.842955600817	-1.273884918251
-0.842810897586	-1.273656670092
-0.842681056012	-1.273470074233
-0.842558831058	-1.273468136823
-0.842417812163	-1.273255614665
-0.842322093302	-1.27317515499
-0.842161599194	-1.273020545028
-0.842026274205	-1.272518819971
-0.841907211116	-1.272332186692
-0.841808263022	-1.272166468426
-0.841698511453	-1.272139641329
-0.841553049947	-1.271941500833
-0.84141257176	-1.271600029987
-0.841246660971	-1.271467907594
-0.841053694985	-1.271164135274
-0.840896202041	-1.271130253659
-0.840795315228	-1.271088017538
-0.840648688235	-1.270769095908
-0.840561167652	-1.270354317967
-0.840392503656	-1.269819356487
-0.840242501321	-1.269540852839
-0.840100092564	-1.269053787062
-0.839988169783	-1.269025435468
-0.839846281564	-1.268832651363
-0.839656105517	-1.268756368697
-0.839548934738	-1.26865739452
-0.839360874746	-1.268651107947
-0.839212992695	-1.268350705682
-0.839061970293	-1.268335769657
-0.838906734488	-1.268167162692
-0.838791072135	-1.268146238093
-0.838664800641	-1.268103345742
-0.838537620893	-1.268045684972
-0.838394275022	-1.267651996906
-0.838176949252	-1.267546269586
-0.838026000608	-1.267250543552
-0.837880908294	-1.267088341738
-0.837691366704	-1.267048906109
-0.837595871836	-1.266906961021
-0.83747054328	-1.266818272757
-0.837279022508	-1.266687392983
-0.837142819182	-1.266437006276
-0.837024005274	-1.266435948646
-0.836916357519	-1.266103459123
-0.836783726744	-1.266003597395

-0.836654496828	-1.265966146948
-0.836505354607	-1.265091111569
-0.836427316461	-1.265037459491
-0.836254830179	-1.264799105211
-0.836136356428	-1.264494735972
-0.836003859645	-1.264344243456
-0.83585314639	-1.26421158435
-0.835711091182	-1.264019889383
-0.835600791723	-1.263947506792
-0.835470798991	-1.263857580488
-0.835326093684	-1.263507882706
-0.835176620407	-1.263120353797
-0.83501116466	-1.262674322613
-0.834872565243	-1.262107014304
-0.834750448491	-1.261962444538
-0.834596883674	-1.261749963398
-0.834482384794	-1.261730273722
-0.834398721637	-1.261505468429
-0.834286458168	-1.261463830761
-0.834173577209	-1.261345809473
-0.834055871088	-1.261315757874
-0.833919946817	-1.261242279396
-0.833772970453	-1.261135901115
-0.833564478285	-1.260837834852
-0.833416221403	-1.260353744325
-0.8332664181	-1.260195511863
-0.833127834591	-1.259955799888
-0.833010656501	-1.259922506828
-0.832864071633	-1.259646810083
-0.832773478951	-1.259541802664
-0.832623969039	-1.25934935149
-0.832485253216	-1.259216035218
-0.832379091154	-1.259065294602
-0.83221970882	-1.25868529062
-0.832048883991	-1.258469533364
-0.831922575618	-1.25842165382
-0.831754150264	-1.258416705185
-0.831608250718	-1.257770747036
-0.831443991545	-1.257522988285
-0.831294693838	-1.257461467945
-0.831148698299	-1.257387153844
-0.830970145048	-1.257120538293
-0.830864739503	-1.256952524856
-0.830725496564	-1.256926072285
-0.830558082452	-1.256630175515
-0.830404635978	-1.256203542668
-0.830249786158	-1.255559630931
-0.830091387918	-1.254962652855
-0.829979878291	-1.254785736722
-0.8298041108	-1.254529169631
-0.829573498448	-1.253095153469
-0.829441988074	-1.252840063148
-0.829333274117	-1.252813181096
-0.829177068166	-1.252514238012

-0.829047648079	-1.252481915433
-0.82885689304	-1.251377572995
-0.82873228951	-1.251202128098
-0.828566769025	-1.250563190841
-0.828390039537	-1.250463458035
-0.828252609094	-1.25043910612
-0.828139698799	-1.250111368661
-0.827979570273	-1.249631280534
-0.827841951204	-1.248975356738
-0.827727436238	-1.24887218435
-0.82759259905	-1.248828028588
-0.827427697866	-1.248438924614
-0.82731155366	-1.248431291621
-0.827130821615	-1.248412381008
-0.826978810287	-1.248206267674
-0.826887023859	-1.248067931117
-0.826752530145	-1.247962917157
-0.826619294106	-1.247768887575
-0.82647339279	-1.247435809952
-0.826353060417	-1.247098950233
-0.826172831138	-1.246766295279
-0.826027286735	-1.245908227789
-0.825919076476	-1.2458234512
-0.825815191315	-1.245727161258
-0.825675815268	-1.245463333982
-0.825552283458	-1.245450514725
-0.825432887149	-1.245387673182
-0.825327489938	-1.245270907325
-0.825120648063	-1.244625631636
-0.824994302187	-1.244468994883
-0.824834944867	-1.243928838288
-0.824675122812	-1.243637544138
-0.824529651139	-1.243048571395
-0.824395150037	-1.24291381277
-0.824299421342	-1.242813269925
-0.82418250358	-1.242650965586
-0.824026334053	-1.242176598854
-0.823867896133	-1.242170840689
-0.823709798428	-1.242105381168
-0.823563753906	-1.241955870488
-0.823445950835	-1.241540753691
-0.823320594537	-1.241235011439
-0.823199049354	-1.241113198968
-0.822994051891	-1.240331772927
-0.822834499673	-1.239899337536
-0.822687122365	-1.239788018918
-0.822548032619	-1.239742330694
-0.822470549264	-1.239672246259
-0.822313468157	-1.239630099854
-0.822171793348	-1.239549098578
-0.822039634237	-1.239274762802
-0.821909169204	-1.239172488222
-0.821822108064	-1.239162042814
-0.821632097031	-1.239115444981

-0.821502866255	-1.239065796781
-0.821353229692	-1.238976467084
-0.821140521294	-1.238824619452
-0.821040848411	-1.238678427901
-0.820936464566	-1.238552423869
-0.820753623227	-1.238490355735
-0.820651872783	-1.238377263612
-0.820546966888	-1.238364467155
-0.820442059391	-1.238335074545
-0.820318022812	-1.238325282142
-0.820194484055	-1.238275587625
-0.820062929224	-1.238272268201
-0.819944566528	-1.238220251972
-0.819782763447	-1.237329928787
-0.819668949243	-1.237020827368
-0.819523741941	-1.236970450741
-0.819390639303	-1.236733466772
-0.819258678771	-1.236455087672
-0.819144050736	-1.236376311795
-0.819020865804	-1.236006083292
-0.818904878717	-1.235741186319
-0.818792672188	-1.235221154898
-0.818666769925	-1.23500268731
-0.818548694988	-1.234947790611
-0.818426145809	-1.234929452293
-0.818309471547	-1.234924467087
-0.818121407261	-1.234633884748
-0.818007758128	-1.234630450382
-0.81784967593	-1.234097589943
-0.817717430476	-1.233906280102
-0.817581168385	-1.233561635422
-0.817384751443	-1.233253510047
-0.817239559378	-1.233245534722
-0.81710714786	-1.23314889993
-0.816971344365	-1.232980111145
-0.816839472199	-1.232876198872
-0.81672488773	-1.232182492772
-0.816543177457	-1.231739196738
-0.81642638348	-1.2317217503
-0.816230869772	-1.23155703372
-0.8160692576	-1.23118190297
-0.81588720245	-1.23035957155
-0.815770843241	-1.230196714537
-0.815644705712	-1.229946666996
-0.815508479468	-1.229504688701
-0.815375440081	-1.229118164761
-0.815235563736	-1.228880006367
-0.815086243537	-1.22845270754
-0.814960886447	-1.22839139015
-0.814805659155	-1.22828727105
-0.81467223196	-1.228082196837
-0.814526862737	-1.228032613875
-0.814403516688	-1.227877956792
-0.814288112529	-1.227748965211

-0.814178003663	-1.227725770777
-0.814046666123	-1.2277001461
-0.813935452548	-1.227536745778
-0.813808255888	-1.227432950326
-0.813678215578	-1.22728806584
-0.813571170748	-1.227193910338
-0.813411928405	-1.226778843726
-0.813281328008	-1.226762819434
-0.813174161076	-1.22671466446
-0.813040083567	-1.226560353482
-0.812844698442	-1.225220612085
-0.812681465035	-1.224875421805
-0.812524949373	-1.224776853533
-0.812390538712	-1.224011294095
-0.812230369798	-1.223937953389
-0.811999566645	-1.223894437144
-0.811874022285	-1.223847578054
-0.811748026911	-1.223637060514
-0.811606037875	-1.223303119278
-0.811507887118	-1.223277684674
-0.811387029321	-1.223256317866
-0.811215883487	-1.222898216217
-0.811084358113	-1.222747644142
-0.810947237821	-1.222537284319
-0.810773870508	-1.22243906747
-0.810638930285	-1.222288380431
-0.810529595764	-1.222156194204
-0.81034499407	-1.221752532195
-0.810190091815	-1.221439828313
-0.810029265557	-1.221286842127
-0.809887467454	-1.221283845457
-0.809711537503	-1.221146929675
-0.809597256828	-1.221057762679
-0.809462943405	-1.221046404166
-0.809266442884	-1.220750789991
-0.809098113902	-1.220648290552
-0.808948956815	-1.220634528303
-0.808775390823	-1.220472364136
-0.808648410746	-1.220334606011
-0.808528177662	-1.220332475584
-0.808378205643	-1.219504503642
-0.808249050844	-1.218952119685
-0.808097115225	-1.218925047583
-0.807918204207	-1.21846381506
-0.807795187632	-1.218401790311
-0.807655754272	-1.218192759044
-0.807551050741	-1.2180949529
-0.807397644372	-1.218069790811
-0.807305395368	-1.218001646841
-0.807237531037	-1.217867031255
-0.807108830668	-1.217752338329
-0.806999083944	-1.21769163509
-0.806910995087	-1.217633874943
-0.806803506836	-1.217417581146

-0.806680491724	-1.217342790297
-0.806513422948	-1.217190482024
-0.806404754041	-1.216746719047
-0.806294899686	-1.216542014272
-0.806162341161	-1.216479891948
-0.805975693109	-1.215724863688
-0.805837661288	-1.215649181845
-0.805664824931	-1.215372509821
-0.805496440225	-1.214782294044
-0.805364536482	-1.214642928716
-0.805265969031	-1.214503430082
-0.805137489574	-1.214471626976
-0.805023463244	-1.21415140029
-0.804887541791	-1.213949341794
-0.804759500729	-1.213697427534
-0.804618295636	-1.213472305188
-0.804482533316	-1.212995610394
-0.804367001959	-1.212852576868
-0.804217762093	-1.212116535536
-0.80408237179	-1.212108793096
-0.803969646115	-1.212105967409
-0.803773491631	-1.211374723652
-0.803628311041	-1.211239551544
-0.80345708234	-1.211150913698
-0.803356545782	-1.210680698716
-0.803276109474	-1.210647692434
-0.803111701353	-1.210435515094
-0.802957465644	-1.210403757309
-0.802795612471	-1.209634009134
-0.802667680569	-1.209622766898
-0.802532791012	-1.20933893573
-0.802389955338	-1.209305345247
-0.802256453465	-1.209197602829
-0.802141096493	-1.209158216738
-0.801987648191	-1.209094368029
-0.801842720685	-1.208687008684
-0.801714233868	-1.208537605971
-0.801600403908	-1.208353559615
-0.801398363818	-1.207166454344
-0.801279985149	-1.207135888429
-0.801153229301	-1.207004127174
-0.800978410755	-1.206960952692
-0.800862449787	-1.206891310887
-0.800714729471	-1.206710553877
-0.800527833368	-1.206635335088
-0.800398104307	-1.206560238216
-0.800233105883	-1.205954278778
-0.800053705052	-1.205842364534
-0.799884411702	-1.205188567769
-0.799756447554	-1.204821340479
-0.799630128752	-1.204661336974
-0.799518194455	-1.204588666254
-0.799343945003	-1.204546321643
-0.799210488788	-1.204373920793

-0.799109528994	-1.204372303936
-0.798983849411	-1.204171417271
-0.798876128906	-1.20413742083
-0.798773771223	-1.20389484903
-0.798598264215	-1.203597526676
-0.798444274527	-1.203518178067
-0.798325957627	-1.203355134689
-0.798170096416	-1.203351743757
-0.798038226897	-1.203217241888
-0.797909131302	-1.203064662438
-0.797744713785	-1.202718519506
-0.79758281427	-1.202586247904
-0.797464149711	-1.202062162124
-0.797338242576	-1.20157056035
-0.797174849327	-1.201492584395
-0.797007635664	-1.20147105684
-0.796842506768	-1.201086386989
-0.79675052078	-1.200897037058
-0.796595033046	-1.200061630363
-0.7964632457	-1.199512778523
-0.796386163882	-1.199320179031
-0.796265605296	-1.199176798157
-0.796131813099	-1.19910826769
-0.795973235679	-1.199009467168
-0.795825588379	-1.198952126645
-0.795700355432	-1.1988405576
-0.795572506129	-1.198663926449
-0.795450202707	-1.198654125291
-0.795297557667	-1.198445028198
-0.795167324514	-1.197817214193
-0.795018704962	-1.197756705581
-0.794891409339	-1.197695604995
-0.794749323973	-1.197584654154
-0.794656598947	-1.197398459257
-0.794538028126	-1.197161546069
-0.794425450796	-1.197110311002
-0.794317715316	-1.19670446423
-0.794172794972	-1.196416311534
-0.794069819747	-1.1962899939
-0.79394458866	-1.196224284581
-0.793757296363	-1.195963117169
-0.7936258402	-1.19596049435
-0.793464456032	-1.195781336501
-0.793367863782	-1.195773009891
-0.79325096882	-1.195648334599
-0.793075886511	-1.195550943746
-0.792953445026	-1.195475800126
-0.792794035519	-1.195432579312
-0.792588852022	-1.195392327803
-0.792401893714	-1.195315097672
-0.792217002569	-1.195256921733
-0.792038222819	-1.194708974119
-0.791882822557	-1.194541907542
-0.791762717822	-1.19429942227

-0.791613084107	-1.194186021699
-0.791464644302	-1.19414183158
-0.791376667193	-1.194055439642
-0.791253989587	-1.193978660605
-0.791112989234	-1.193811958007
-0.790952787179	-1.193608263232
-0.79085630573	-1.193475869425
-0.790690342518	-1.193424723474
-0.79056718566	-1.1933113432
-0.790422716733	-1.193277429699
-0.790309470482	-1.193106491235
-0.790163731057	-1.19251170428
-0.790046916765	-1.192425297897
-0.789943979301	-1.192393191292
-0.789815575113	-1.192335123374
-0.789652513708	-1.192007182906
-0.789471123675	-1.191683021717
-0.789353815963	-1.191609036435
-0.789187794216	-1.191526736729
-0.789054637064	-1.191498130201
-0.788971880709	-1.191371900973
-0.788801882007	-1.191037026172
-0.78861529472	-1.190224180286
-0.788465571583	-1.190170845749
-0.788279806181	-1.189034151219
-0.788161310726	-1.188774521343
-0.788005858575	-1.188302014436
-0.787842214677	-1.188168037537
-0.787651892454	-1.188041367576
-0.787490514311	-1.187876086
-0.787368714783	-1.187673105468
-0.787214934573	-1.187537456292
-0.787079705783	-1.187411758236
-0.786929284115	-1.187164355548
-0.786823148231	-1.186938989131
-0.786678702984	-1.186816542794
-0.786552833451	-1.186435417682
-0.786415568241	-1.186353844773
-0.786264131795	-1.18619122824
-0.786136815573	-1.185907640994
-0.786037231696	-1.185865807773
-0.78589823863	-1.18583453414
-0.785764950733	-1.185802707518
-0.78562900804	-1.185710189352
-0.785510680218	-1.185698945317
-0.785421934628	-1.185612503436
-0.785281297131	-1.185535824905
-0.785109170002	-1.185140020626
-0.784987760871	-1.184726219647
-0.784851319615	-1.184594250511
-0.784710966062	-1.184208100745
-0.784582410959	-1.183949773011
-0.784493348641	-1.183728796251
-0.784354097314	-1.183248230742

-0.784244328353	-1.183217943686
-0.784110758207	-1.182750380481
-0.783985814205	-1.182736915904
-0.783856385986	-1.18247294009
-0.783712221416	-1.182371825292
-0.78356791218	-1.182290559716
-0.783421682119	-1.182217409734
-0.78329233723	-1.182166811956
-0.78319837684	-1.182103022692
-0.783071828943	-1.182064223966
-0.78296701121	-1.181734221697
-0.782880975018	-1.181728026228
-0.782765407999	-1.181613811922
-0.782647053098	-1.181405154102
-0.782497880972	-1.181381007334
-0.78236427772	-1.181157635958
-0.782207681229	-1.181057752374
-0.782096292193	-1.181013087086
-0.781991887513	-1.180840835955
-0.781893028106	-1.180838458175
-0.781756973124	-1.180785016541
-0.781587763994	-1.180614470081
-0.781471660894	-1.180571187038
-0.781382787582	-1.18039079631
-0.781204343591	-1.180345453259
-0.781084979086	-1.180181625631
-0.78096043156	-1.180035786998
-0.780788631702	-1.179924517368
-0.780638096122	-1.179776016397
-0.780491612457	-1.179692559648
-0.78038953829	-1.179632627562
-0.780283887995	-1.179610908244
-0.780089346376	-1.179225661998
-0.779885103959	-1.178989465819
-0.779758582356	-1.178967184847
-0.7796322959	-1.178654986309
-0.77953431492	-1.178604445975
-0.779389183393	-1.178548481651
-0.779187553067	-1.178441554313
-0.779078811021	-1.178377823384
-0.778946830823	-1.178184841133
-0.778812807026	-1.178136260591
-0.778664651357	-1.17728805896
-0.778574266666	-1.177241855855
-0.778423757133	-1.177111034186
-0.778301507363	-1.177045673406
-0.778170738763	-1.176976284929
-0.77802539819	-1.176841921317
-0.777908082445	-1.176755087465
-0.777775233539	-1.176581150405
-0.777619353572	-1.176546173074
-0.777485104483	-1.176271849648
-0.777369845035	-1.175698633722
-0.777208608991	-1.175349009018

-0.777102624855	-1.175312895577
-0.776957909885	-1.175013153548
-0.776808840069	-1.174806465263
-0.776670093182	-1.174650250202
-0.776529881886	-1.174574683868
-0.776401257309	-1.174510975823
-0.776270493997	-1.174255903793
-0.776083818288	-1.173823910331
-0.77593431876	-1.173734213224
-0.775816015494	-1.173543054673
-0.775644134363	-1.173380077015
-0.775522015943	-1.173003958596
-0.775425244673	-1.17287034019
-0.775316949612	-1.172794725929
-0.77517398552	-1.172750076133
-0.775018056781	-1.172696556742
-0.774915974697	-1.172368186898
-0.774828742282	-1.172098189506
-0.774699658586	-1.172069526278
-0.774591998839	-1.172014441529
-0.774493713705	-1.171597707921
-0.774368846821	-1.171436792674
-0.774228382926	-1.171178901044
-0.774107335158	-1.17112351091
-0.774007005809	-1.171004651476
-0.773888019157	-1.170840158544
-0.773773821885	-1.170657586826
-0.773653254156	-1.17053098744
-0.773560984133	-1.170442872425
-0.773457412934	-1.170259436428
-0.77331427816	-1.169943483295
-0.773204666168	-1.169838550881
-0.773084991689	-1.169399808182
-0.772954513224	-1.169398178765
-0.772854218688	-1.169033196122
-0.772729977527	-1.168931923694
-0.772581125913	-1.168640194855
-0.772448298286	-1.168278872421
-0.772277967677	-1.167752046754
-0.772138653205	-1.167548995146
-0.771997457732	-1.167408558315
-0.771838309916	-1.16670582914
-0.77167035883	-1.166352036065
-0.771558498168	-1.166317097499
-0.771422721267	-1.16592394785
-0.771306771469	-1.165783020466
-0.771212953905	-1.165650803463
-0.771080989257	-1.165537222898
-0.770976997955	-1.165453938233
-0.7708298019	-1.16538243541
-0.770691319999	-1.164584043796
-0.770584810266	-1.164381219598
-0.770472375065	-1.164167455
-0.77036764235	-1.164073990976

-0.770222149724	-1.163775129453
-0.770106305588	-1.163745916968
-0.769972513825	-1.163703125531
-0.769804498695	-1.163032724858
-0.769695273545	-1.162861595128
-0.769534418283	-1.162847542051
-0.769371787797	-1.16260423633
-0.769280661134	-1.162568308449
-0.769117124661	-1.162532915022
-0.768989001575	-1.162375810656
-0.768877353969	-1.162373320088
-0.768753404669	-1.161990372572
-0.768619015863	-1.161838501281
-0.768473962317	-1.161737499391
-0.768327821942	-1.161424007679
-0.768210614224	-1.160941406016
-0.768055066187	-1.16054128009
-0.767927739345	-1.160495283894
-0.767795072194	-1.160447647448
-0.767709434686	-1.160431431593
-0.767579615511	-1.160311434431
-0.767478130574	-1.160273944695
-0.767339821631	-1.160199274306
-0.767227750409	-1.159895923903
-0.767115740909	-1.159801125634
-0.766955523236	-1.159612997018
-0.76685126114	-1.159599790132
-0.766711254327	-1.159578458479
-0.766593149317	-1.15937416319
-0.766487666982	-1.159372958086
-0.766378360159	-1.159278459851
-0.766228779841	-1.158778050503
-0.766111632672	-1.158753220929
-0.765963974588	-1.158721303397
-0.765837160347	-1.158676192875
-0.765672913157	-1.158270551591
-0.765578781466	-1.158181185392
-0.76545874968	-1.158178428817
-0.76532052816	-1.157894132646
-0.765223714279	-1.157793577671
-0.765136400117	-1.157581469644
-0.764984181664	-1.157575392315
-0.764898390108	-1.157468612251
-0.764736440523	-1.157468332625
-0.764503308386	-1.157376367117
-0.764361549397	-1.157159200516
-0.764264861433	-1.157122449965
-0.764167875037	-1.157031096518
-0.7640717577	-1.156861989672
-0.763960781203	-1.156665164887
-0.76382532185	-1.156610413057
-0.763719550463	-1.156361107139
-0.763631332605	-1.156061728433
-0.763425071097	-1.155555946847

-0.763240306682	-1.155187813234
-0.763062629361	-1.154809948811
-0.762912612028	-1.154519715912
-0.762783988404	-1.154494050587
-0.762651883136	-1.154458572801
-0.76250352103	-1.153983184785
-0.762337035494	-1.15388601395
-0.762216337286	-1.153872853548
-0.762080376532	-1.153345509558
-0.761998369338	-1.153272742938
-0.761898769527	-1.153249027229
-0.761752907706	-1.152788083212
-0.761615528575	-1.152525287365
-0.761523546662	-1.152452485736
-0.761373540745	-1.152330413691
-0.761258638083	-1.152244681469
-0.761114945728	-1.152218320476
-0.761003232588	-1.15221516527
-0.760894452706	-1.151970735969
-0.760770798394	-1.151908906739
-0.760659197436	-1.151764490069
-0.760552580648	-1.151622942492
-0.76043162743	-1.151465216756
-0.760339461025	-1.151435792381
-0.760191772	-1.150624836579
-0.760045290238	-1.150438950028
-0.759908040755	-1.150064401284
-0.759765623939	-1.150046858161
-0.759615362738	-1.149262142508
-0.759503400389	-1.14913305619
-0.759410178177	-1.149068725647
-0.759239575087	-1.148930814305
-0.759092356801	-1.148779658168
-0.758931051308	-1.148743011939
-0.758768500258	-1.148347296613
-0.758653345525	-1.148118717126
-0.758550715244	-1.148038826769
-0.758403711364	-1.147474431484
-0.758275763377	-1.147466119575
-0.758165321905	-1.147249598749
-0.757993289124	-1.147161454472
-0.757850103955	-1.147161315695
-0.757732098865	-1.146885171035
-0.757617895791	-1.146759181083
-0.757449205468	-1.146078720078
-0.757278869709	-1.145422987834
-0.757170598698	-1.145244872712
-0.757056391444	-1.145193612533
-0.756956775152	-1.145046386134
-0.756817006348	-1.144900314078
-0.75666305625	-1.14471850864
-0.756553912064	-1.144588959519
-0.756415404959	-1.14453447984
-0.75626506445	-1.143804920947

-0.756156472163	-1.143753911028
-0.756065298327	-1.143678689229
-0.755932190397	-1.143165200584
-0.755835591595	-1.143009330455
-0.75568389872	-1.142704798301
-0.755592029091	-1.142474132437
-0.755436556355	-1.142219126483
-0.755295761473	-1.142129873745
-0.755120163957	-1.142111303627
-0.755026960058	-1.141891381112
-0.754839327706	-1.14130919701
-0.754723311876	-1.14116691922
-0.75456599246	-1.140903367414
-0.754486237523	-1.140754838247
-0.754365760103	-1.140327534211
-0.754204631715	-1.140283230177
-0.754102549489	-1.140201435408
-0.753951912578	-1.13987171366
-0.753828528954	-1.139848319537
-0.753658316998	-1.139828199474
-0.753487137824	-1.139434781761
-0.753313816786	-1.13920075802
-0.753198079022	-1.139185709865
-0.753055413307	-1.139003723556
-0.752961976612	-1.138963858591
-0.75281524927	-1.138426290897
-0.752690299338	-1.138063486007
-0.752593497096	-1.138026670181
-0.752453100589	-1.137738103702
-0.75232540063	-1.137399079227
-0.752202491979	-1.13735106115
-0.752095606608	-1.136841455392
-0.751988164721	-1.136723035133
-0.751871927142	-1.136694693527
-0.751739149781	-1.136346221198
-0.751615628869	-1.135988458101
-0.751474238924	-1.135714128723
-0.751322606219	-1.135635589989
-0.75125177016	-1.135554622743
-0.751135020102	-1.135372919339
-0.750996757304	-1.135067050512
-0.750857155306	-1.134875644497
-0.75071814458	-1.134792577283
-0.750625978327	-1.134736780035
-0.750515152262	-1.134542283472
-0.750350340706	-1.134115244522
-0.750267567934	-1.134046294521
-0.750090418889	-1.133964908019
-0.749980691037	-1.133903796106
-0.749890499957	-1.133872558349
-0.749774945585	-1.133646198304
-0.749651704488	-1.133499077778
-0.749498235759	-1.133456483119
-0.749386495348	-1.133449628356

-0.749273335925	-1.133416088852
-0.749130283075	-1.13335506498
-0.748950823583	-1.132599733406
-0.74879834634	-1.132440440041
-0.748658174623	-1.132264120986
-0.748542577738	-1.132178365642
-0.74844652802	-1.132122009633
-0.748325418718	-1.131842649975
-0.748176564945	-1.131613971805
-0.748080894732	-1.131359458496
-0.747977188725	-1.131106401127
-0.747844667097	-1.130993501116
-0.747743241781	-1.130867810233
-0.74760610309	-1.130723958941
-0.747495012624	-1.130723438867
-0.747308402668	-1.129784333373
-0.747205262595	-1.129782283707
-0.747114488941	-1.129709067054
-0.74693709169	-1.129102981395
-0.74676065323	-1.128538859986
-0.746621304647	-1.127666504336
-0.746503351458	-1.127595311783
-0.746356926458	-1.127566582843
-0.746246123025	-1.127524973012
-0.746128791235	-1.127286316802
-0.745976632859	-1.127023458898
-0.745863809266	-1.127004465997
-0.745729330538	-1.126675197399
-0.745610371284	-1.126265536022
-0.745497995135	-1.126254151624
-0.745368305026	-1.126247146201
-0.745207123273	-1.125572124061
-0.745066977917	-1.12516865923
-0.744933392869	-1.124994158657
-0.744785345903	-1.124833496291
-0.744707276242	-1.124777561071
-0.744604348979	-1.124666718823
-0.744472127283	-1.12452826243
-0.744322280295	-1.124415262091
-0.744151610528	-1.124102700314
-0.744066259888	-1.124029282479
-0.743955520343	-1.123546686846
-0.74385954234	-1.123358638339
-0.743761182491	-1.123207819918
-0.743689856984	-1.123188933397
-0.743569978933	-1.123150513008
-0.743427745366	-1.122715604935
-0.743286599998	-1.122625710607
-0.743160313406	-1.122294765815
-0.74301666408	-1.122118296299
-0.742841354637	-1.121416669862
-0.742649179278	-1.120415142397
-0.742523168056	-1.120337337675
-0.742385242676	-1.120157453709

-0.742264566826	-1.120119292634
-0.74211652794	-1.12011334778
-0.742037853761	-1.120087281824
-0.741893558253	-1.120031528235
-0.741791045773	-1.119957497633
-0.741664646834	-1.119850175264
-0.741559907996	-1.119724490952
-0.741433660445	-1.119335674388
-0.741270105629	-1.119164933049
-0.741135238904	-1.118674902111
-0.741009689793	-1.11854888506
-0.740880785981	-1.118497532124
-0.740724975634	-1.118482833893
-0.740570836499	-1.118429262194
-0.740433480101	-1.118365374337
-0.740260681173	-1.118170016807
-0.740146452476	-1.118020360901
-0.739973841181	-1.117995460367
-0.739810381408	-1.117977294433
-0.739622233927	-1.117663474178
-0.739469294782	-1.117421142304
-0.739345052068	-1.116881732175
-0.739193990414	-1.116832758403
-0.739027951674	-1.116618104413
-0.738890648258	-1.116480373599
-0.738693936226	-1.11594219237
-0.738543506517	-1.11590919471
-0.738395272822	-1.115840301841
-0.738252653617	-1.1157929487
-0.73810937272	-1.115606895993
-0.737926769583	-1.115260912632
-0.737793682877	-1.114885765887
-0.737689040771	-1.114688960775
-0.737526285099	-1.114564214652
-0.737374636831	-1.114184937421
-0.737200711393	-1.11414601029
-0.737092775419	-1.113846043742
-0.736891366021	-1.113427471631
-0.736765652659	-1.113011837198
-0.736652639494	-1.113004153371
-0.736564239099	-1.112842356508
-0.736400873452	-1.112838418508
-0.736223732191	-1.112702424103
-0.73606126686	-1.112424838772
-0.73592195602	-1.112355206844
-0.735809067059	-1.112315503014
-0.735698031054	-1.112148922958
-0.735594850872	-1.112065164108
-0.735422441413	-1.111324925527
-0.735290907315	-1.111032919632
-0.735141488119	-1.110774129004
-0.735026934196	-1.110566418896
-0.734921085357	-1.110367936184
-0.734798244143	-1.110361493319

-0.734668188261	-1.110194931849
-0.734492135533	-1.109223620434
-0.734409917455	-1.109024221561
-0.734274732824	-1.108721771028
-0.734186381769	-1.108533550442
-0.734079809377	-1.108313355498
-0.73393081869	-1.108309606021
-0.733846938454	-1.108290035237
-0.733727890418	-1.108183604518
-0.733641285698	-1.108148736987
-0.733504422067	-1.107936652019
-0.733410294746	-1.107935629567
-0.733302686101	-1.107638130197
-0.733147394496	-1.107237291866
-0.733037730055	-1.107229373351
-0.732890538303	-1.106918462004
-0.732785865808	-1.106870697321
-0.732658917611	-1.106584505772
-0.732570832144	-1.106572765195
-0.732424485295	-1.106211072773
-0.732276645708	-1.106155979578
-0.732122158965	-1.106138091053
-0.731998284163	-1.106102294215
-0.731882364556	-1.105971167872
-0.731749741312	-1.105845164433
-0.731635713065	-1.105832638953
-0.731443659651	-1.105380843357
-0.731284071112	-1.104691693278
-0.73117747028	-1.104686374816
-0.731092459291	-1.104618133438
-0.730954938148	-1.10459603528
-0.730817634959	-1.104408616222
-0.730688676705	-1.104231962838
-0.730547350656	-1.104131457097
-0.730450327861	-1.10410627974
-0.730344313556	-1.103962555397
-0.730228117289	-1.103950528363
-0.730083439701	-1.103945622712
-0.729956906823	-1.103894984618
-0.72981409301	-1.103859272528
-0.729670509638	-1.103732792148
-0.729513068836	-1.103329115131
-0.729333881452	-1.102908508273
-0.729130697334	-1.102864002883
-0.729027720521	-1.102578626665
-0.728931402432	-1.102401086799
-0.728859764619	-1.102400376576
-0.728732230809	-1.101904459124
-0.728608587006	-1.101891631281
-0.728513240745	-1.101531191457
-0.728361859122	-1.101421286792
-0.728230599409	-1.101230017153
-0.728079298821	-1.100962830307
-0.727992772001	-1.100872462013

-0.727894514343	-1.100828080555
-0.72776237684	-1.100205719421
-0.727632729919	-1.100087293592
-0.727527348864	-1.100056773429
-0.727292357149	-1.098727049916
-0.727127057779	-1.097826337402
-0.726976562632	-1.097819277551
-0.726820550735	-1.097667819272
-0.726758681879	-1.097595633752
-0.726593047505	-1.097481975593
-0.726498370092	-1.097456709458
-0.726383058805	-1.09729790441
-0.726285758909	-1.097181531241
-0.726111261212	-1.096429289022
-0.725928727392	-1.096296409788
-0.725832996105	-1.096100846182
-0.725739950884	-1.095837854219
-0.72562481082	-1.095812797139
-0.725512698801	-1.095608965405
-0.725387469039	-1.095517008739
-0.725304489979	-1.095498368316
-0.725187224123	-1.095361017822
-0.725071370489	-1.095152038871
-0.724947316208	-1.095051516682
-0.724839816541	-1.094746222715
-0.724683547987	-1.094687728125
-0.724573012922	-1.094598613752
-0.724429202271	-1.094350252144
-0.724307666303	-1.094069537512
-0.724172746748	-1.093686427785
-0.724094463738	-1.093675532371
-0.723993679306	-1.093269297656
-0.723864823238	-1.093090803078
-0.723721645398	-1.092958207906
-0.723566213707	-1.092590769686
-0.723416638091	-1.092479264817
-0.723182304601	-1.090793437344
-0.723060169478	-1.090577651922
-0.722969501261	-1.090517303654
-0.722833199878	-1.09050780531
-0.722693670754	-1.09017155343
-0.722544747784	-1.090111417852
-0.722399736227	-1.089593272063
-0.722315220434	-1.089528903183
-0.722190630216	-1.08951989503
-0.722113690885	-1.089504053788
-0.721984737078	-1.089461234127
-0.721880305445	-1.089459484782
-0.721771056133	-1.089419037657
-0.721628263331	-1.089277791567
-0.721493234441	-1.089102484452
-0.72130508056	-1.088698755666
-0.721196803293	-1.088603405532
-0.721088755828	-1.088580221692

-0.720936507333	-1.087812650043
-0.720812664204	-1.087616858925
-0.720684851502	-1.087492539649
-0.720560030721	-1.087175071161
-0.720455889158	-1.087160581105
-0.720340300677	-1.086853877402
-0.720205957961	-1.086769391183
-0.720100424274	-1.086487606153
-0.719977176292	-1.086450307724
-0.719886563121	-1.086448659435
-0.71974331456	-1.086326857426
-0.719607946575	-1.086161652885
-0.71952227096	-1.086089588616
-0.71938929865	-1.085946511945
-0.719265672638	-1.085701928983
-0.719169785182	-1.084999800367
-0.719038710556	-1.084976104556
-0.718859220731	-1.08480814381
-0.718700359109	-1.084722227968
-0.71860464811	-1.084644078129
-0.718491927069	-1.084526275994
-0.71835662398	-1.084328389363
-0.718216146367	-1.084165896122
-0.718073198385	-1.084144948453
-0.717944556011	-1.084135524003
-0.717859468631	-1.084113681079
-0.717740060848	-1.084043011729
-0.717581101673	-1.083992342354
-0.717449826205	-1.083582904519
-0.717275634542	-1.083035538856
-0.71711991619	-1.082565265141
-0.71698461682	-1.082563081421
-0.716867163084	-1.08223243313
-0.716728522262	-1.08184014252
-0.71664075193	-1.081732705602
-0.716530591503	-1.081732124201
-0.716439319067	-1.081726044283
-0.716302671147	-1.081664653047
-0.716171647615	-1.081335682156
-0.716029983245	-1.081243438084
-0.715924204623	-1.080902937193
-0.71581926205	-1.08083590763
-0.715687842702	-1.080778721995
-0.71555295643	-1.080621604319
-0.715402456923	-1.080058310076
-0.71522850013	-1.079742613676
-0.715110404477	-1.07971247631
-0.714929126853	-1.078199777657
-0.714775086698	-1.077656642701
-0.714594243762	-1.077418291113
-0.714399283324	-1.077042549124
-0.71424080357	-1.076716107399
-0.714157943404	-1.076610737296
-0.714021083423	-1.076403593466

-0.713905085501	-1.075974899233
-0.713787082767	-1.075908436411
-0.713635712302	-1.075902720938
-0.713523994997	-1.075874777828
-0.713344010457	-1.075054178279
-0.713181045333	-1.074980934268
-0.713055465128	-1.074943700324
-0.712955480753	-1.074843556347
-0.712805436863	-1.074534833498
-0.712670052032	-1.074448852012
-0.712509740934	-1.074236214996
-0.712361799978	-1.073969226431
-0.712218297269	-1.073936552386
-0.712119425225	-1.073626760276
-0.7119821005	-1.073177849882
-0.711879229231	-1.073128364098
-0.711742261212	-1.072893861209
-0.71165491796	-1.072693084809
-0.711473712275	-1.072567877618
-0.711325210321	-1.072253073092
-0.711182326794	-1.072214772597
-0.711021706116	-1.072178718338
-0.710906721086	-1.072126264434
-0.710802988969	-1.072021440204
-0.710708957766	-1.071981405166
-0.710551273972	-1.071908807766
-0.710382693137	-1.071503296234
-0.710289565831	-1.071139882383
-0.710138746937	-1.071045115938
-0.710015032317	-1.070860831503
-0.709877012538	-1.070214814155
-0.709765977438	-1.070203774066
-0.709639916333	-1.070118431836
-0.709466592662	-1.069889446397
-0.709326005544	-1.069803117931
-0.709197053697	-1.069401302649
-0.709039909758	-1.068768224905
-0.708941059107	-1.068745378843
-0.70884024672	-1.068696904873
-0.708700088075	-1.068315996375
-0.708585513566	-1.068217748654
-0.708494650835	-1.068200249085
-0.708362167053	-1.068182132076
-0.708246730259	-1.068027561821
-0.708139080462	-1.068006161104
-0.707957669966	-1.068004043066
-0.707816141527	-1.067968961743
-0.70770520878	-1.067938934841
-0.707531818916	-1.066717087126
-0.707424679274	-1.06644494265
-0.707265874005	-1.06585129255
-0.707132683117	-1.065659300458
-0.707025356064	-1.065401615463
-0.706939313752	-1.065342305864

-0.706846256671	-1.065089045577
-0.706739793099	-1.064817480872
-0.70663655573	-1.064642970349
-0.706523014956	-1.064583953982
-0.706386121826	-1.064240766707
-0.706274755143	-1.064160392417
-0.706148834346	-1.064123087035
-0.706056682165	-1.06396502672
-0.705963403137	-1.063918013968
-0.705829630101	-1.063757382457
-0.705673893062	-1.062884562489
-0.705576048941	-1.06271932599
-0.705367466146	-1.06245266766
-0.705215658292	-1.062122769374
-0.705083494508	-1.061637551889
-0.704960654536	-1.061596598507
-0.70481847182	-1.06094612345
-0.704633710096	-1.060447062583
-0.704545534081	-1.060181600628
-0.704412926618	-1.060158230151
-0.704292425838	-1.059925467657
-0.704153828711	-1.059668505761
-0.704012235179	-1.059323308159
-0.703876322633	-1.059209107498
-0.703728814706	-1.058972214257
-0.703619275473	-1.058905603165
-0.703499371219	-1.058687105465
-0.703344492496	-1.058250944335
-0.703211938337	-1.05809870576
-0.703109671299	-1.058028523398
-0.702936570835	-1.057855286317
-0.702797438649	-1.057696836356
-0.702621090943	-1.057499999065
-0.702508108939	-1.057260794735
-0.702398875851	-1.057239139234
-0.702282548875	-1.057144438608
-0.702151468034	-1.056722045505
-0.701983737879	-1.056008688038
-0.701832331967	-1.055621277257
-0.701726504899	-1.055580238385
-0.701586918382	-1.055123990276
-0.701431851854	-1.055028115142
-0.701296890691	-1.054980837075
-0.701213013903	-1.054964598549
-0.701115677067	-1.054827818665
-0.701007267062	-1.054792997303
-0.70086078299	-1.054457693983
-0.700741713824	-1.054263294017
-0.700581428441	-1.054205641882
-0.700343498209	-1.053160580155
-0.700247770122	-1.053026495367
-0.700105053594	-1.052718046356
-0.699972275041	-1.052175496278
-0.699842452398	-1.052143361434

-0.699658929367	-1.051229926396
-0.699535193356	-1.051203494845
-0.699404824147	-1.051158726032
-0.699296167135	-1.051140483872
-0.699200754904	-1.050978054784
-0.699131165267	-1.050972939604
-0.699020378073	-1.050895303632
-0.698924975093	-1.050821366478
-0.698819348642	-1.050705713878
-0.69866666011	-1.050391547314
-0.698563580529	-1.049974439038
-0.698458973003	-1.049893337638
-0.698344213221	-1.049483091819
-0.698226104179	-1.04893918919
-0.698132713538	-1.048917181607
-0.698044190459	-1.048784762307
-0.697934930944	-1.048690337263
-0.697809653481	-1.048689323482
-0.69770911506	-1.048689053888
-0.69757561678	-1.048554594666
-0.697485723407	-1.048468499443
-0.697328034394	-1.048101065428
-0.697201270334	-1.047915292222
-0.697032719289	-1.047823124419
-0.696895808154	-1.047807444321
-0.696788640924	-1.047791369176
-0.69664809431	-1.04763770795
-0.696502930247	-1.04740304331
-0.696387498972	-1.047324167676
-0.696241166231	-1.047086225496
-0.69610778741	-1.047045846687
-0.695966458193	-1.046803674146
-0.695829040734	-1.046565823997
-0.695728902619	-1.046456231888
-0.695571068583	-1.046120662731
-0.695488047823	-1.046052005259
-0.695382360191	-1.046043452107
-0.695229348565	-1.045924384732
-0.695126917705	-1.045917718933
-0.695039321439	-1.045814919574
-0.694938587531	-1.045698158385
-0.694810618756	-1.045439582605
-0.694681687778	-1.045318984433
-0.694602226897	-1.045254483659
-0.694487642761	-1.045090292014
-0.6943530069	-1.044614476881
-0.69420653104	-1.044413008233
-0.694067241368	-1.044102705573
-0.693941443426	-1.044093346562
-0.693834947694	-1.043886488653
-0.693708632999	-1.043858669677
-0.693589509164	-1.043655654139
-0.693444770415	-1.043361701915
-0.693306033859	-1.043238536749

-0.693217189463	-1.04321812849
-0.693100289911	-1.043122408644
-0.692961243719	-1.043116795588
-0.692839093818	-1.043062348163
-0.692684960469	-1.042550003436
-0.692588173662	-1.04246514831
-0.692470015076	-1.042407473073
-0.692335840357	-1.041596187738
-0.692213658533	-1.040920273213
-0.692064358717	-1.040656317203
-0.691940001283	-1.040560797536
-0.691790346734	-1.040433864506
-0.691666484228	-1.040107213233
-0.69155670498	-1.039946143761
-0.691464968821	-1.03990781965
-0.691306091387	-1.039703889441
-0.691156343285	-1.039518337092
-0.691048133561	-1.039408784992
-0.690941921333	-1.038878028368
-0.690804380886	-1.038730747458
-0.690664961686	-1.038682451989
-0.690555907103	-1.038401284299
-0.690437471529	-1.038184338688
-0.690338837535	-1.03811649154
-0.690222779013	-1.038078771925
-0.690132123777	-1.037902465972
-0.690058533985	-1.037812928618
-0.689924916483	-1.037715547535
-0.689791824604	-1.037706248049
-0.689648296649	-1.037446513535
-0.689479774904	-1.037328498605
-0.689359612301	-1.03729000732
-0.689202291755	-1.037106701795
-0.689106060648	-1.037079643876
-0.68899290575	-1.036987052531
-0.688871295249	-1.036930230233
-0.688757817084	-1.036883769827
-0.688615917513	-1.036816753366
-0.688469575174	-1.036690540237
-0.688344757542	-1.036537110707
-0.688243599364	-1.036465771106
-0.688100974137	-1.036378513619
-0.687933607381	-1.036054698646
-0.687781845697	-1.035698941696
-0.687641193988	-1.035371100964
-0.687509594439	-1.034879263177
-0.687426547221	-1.034862378074
-0.687317376717	-1.034509193917
-0.687180995922	-1.034343647022
-0.687087294932	-1.034155965707
-0.686957493053	-1.033980708688
-0.686813372765	-1.033819879668
-0.68667468003	-1.03376750379
-0.686580687005	-1.033684537593

-0.686483414682	-1.033673489889
-0.686412127539	-1.033598293793
-0.686280293694	-1.033553501779
-0.686137517007	-1.033431941504
-0.686030343242	-1.033309463353
-0.685924701934	-1.033227804586
-0.685794564421	-1.033218691201
-0.685670130339	-1.033135108299
-0.685600085676	-1.033127914275
-0.685461094922	-1.032867367865
-0.685347958186	-1.032611328042
-0.685216837128	-1.032524370784
-0.685098431371	-1.032365596537
-0.684970530124	-1.03231976553
-0.684850381768	-1.032141122554
-0.684702287514	-1.032125164021
-0.684571119765	-1.032004122327
-0.684426805085	-1.031983946357
-0.684318596471	-1.03177191615
-0.684190247194	-1.031535783648
-0.684074966568	-1.03151094544
-0.683988898055	-1.031347886171
-0.683836737093	-1.031251569086
-0.683702373599	-1.030801319914
-0.683611814659	-1.030690790288
-0.683520243294	-1.030573402522
-0.68334354223	-1.030232093269
-0.683246613466	-1.030143126896
-0.683108968834	-1.029996609146
-0.682994039576	-1.029951927687
-0.682876144021	-1.029942036611
-0.682765157341	-1.029912377142
-0.68260972284	-1.029548956094
-0.682494861587	-1.029446642426
-0.682342700015	-1.029395676789
-0.682228059843	-1.029349517809
-0.682074081004	-1.029120551971
-0.681936724453	-1.028938300388
-0.681810193115	-1.028759159554
-0.68168455021	-1.028625863623
-0.681554281888	-1.028246620628
-0.681457137315	-1.028139428719
-0.681317648176	-1.028053156928
-0.681175754043	-1.027870687699
-0.681020348202	-1.02766825948
-0.680836219632	-1.027151962597
-0.680655426667	-1.027003266253
-0.680515777684	-1.026952881908
-0.680427695727	-1.026838428783
-0.680231177454	-1.025842712209
-0.680129674706	-1.025743827049
-0.680031136187	-1.025666853027
-0.679906389432	-1.025483638346
-0.679807638658	-1.025399390967

-0.679714958	-1.025395301843
-0.679598457373	-1.025189463751
-0.679469116832	-1.025097077782
-0.679334862017	-1.025077882034
-0.679223214383	-1.025039234006
-0.679130511675	-1.025025732448
-0.679011771335	-1.024736788531
-0.678882112283	-1.0246775161
-0.678785734474	-1.024507100523
-0.678667688533	-1.024452331762
-0.678527940903	-1.024426431767
-0.678421885822	-1.024347939603
-0.678333890664	-1.024312671159
-0.678208435377	-1.024103461451
-0.678090406993	-1.02385449284
-0.677951419133	-1.023394921224
-0.677874278671	-1.023306199727
-0.677779093218	-1.023256646619
-0.677601381918	-1.022613955871
-0.677497066114	-1.022410043173
-0.677399845897	-1.022356429551
-0.677271855433	-1.022276449704
-0.677143805021	-1.022060151787
-0.677038651807	-1.022022006122
-0.676948688931	-1.021960825184
-0.676775120694	-1.021930979501
-0.67665117204	-1.021848667244
-0.676527831442	-1.021640113099
-0.67641107078	-1.021559794256
-0.67630587787	-1.021280658002
-0.676201129333	-1.020956904014
-0.676101008105	-1.020765659548
-0.6759394785	-1.020200241745
-0.675835052228	-1.019903340446
-0.675718342603	-1.019623414754
-0.675618667807	-1.019453058131
-0.675480296787	-1.01940800218
-0.675364960873	-1.019375964768
-0.675229808503	-1.019239782903
-0.67511537738	-1.019062035513
-0.674984750544	-1.018872281565
-0.674892678802	-1.018795322182
-0.674762287292	-1.018793696657
-0.674662995657	-1.018770083758
-0.674559119294	-1.018587132995
-0.674406330618	-1.017537674233
-0.674291906336	-1.017395804941
-0.674158689803	-1.01717189233
-0.674052365574	-1.017013635608
-0.673868314853	-1.016628064997
-0.673697659373	-1.015672208139
-0.673553021205	-1.015621712678
-0.673388234486	-1.015614227769
-0.673222403849	-1.015512594565

-0.673115412439	-1.015511120893
-0.672981172993	-1.015266445445
-0.67286673411	-1.015202330871
-0.672727214137	-1.014904959703
-0.672615045246	-1.01473048823
-0.672501071783	-1.014516560469
-0.672419709631	-1.014483266011
-0.672360361823	-1.014446163448
-0.672238971925	-1.01443897562
-0.672116533255	-1.014258630435
-0.672008754943	-1.014146793637
-0.671885461519	-1.014049666452
-0.67177996444	-1.014049319671
-0.671631584823	-1.014020954083
-0.671503989108	-1.013957038759
-0.671380099876	-1.013620331901
-0.671193581962	-1.013070643703
-0.671043470386	-1.012801962991
-0.670910938267	-1.012671171444
-0.670743993728	-1.012133489235
-0.670622980335	-1.011825470167
-0.670482952176	-1.011693739784
-0.670363820899	-1.011489294033
-0.670273647522	-1.011352003492
-0.670151934642	-1.011299300863
-0.670053517304	-1.011083135874
-0.669938482642	-1.011071223811
-0.66984329915	-1.011054620412
-0.669712212352	-1.010664721746
-0.669598933738	-1.010476861479
-0.669501625126	-1.010342538248
-0.669392596509	-1.009947152039
-0.669191654116	-1.009216119942
-0.669058886185	-1.009135072452
-0.668904051784	-1.009114395955
-0.668772510025	-1.00902005317
-0.66868494862	-1.008836510237
-0.668576820597	-1.008762486049
-0.668506424638	-1.008707423237
-0.668348203505	-1.00868111593
-0.668223959128	-1.008653789158
-0.66810433061	-1.008582882079
-0.667957210779	-1.008083677844
-0.66788156246	-1.008057648975
-0.667733388763	-1.007526326421
-0.667628727857	-1.007323015293
-0.667485470205	-1.007031700998
-0.667386706414	-1.006860477689
-0.667217875185	-1.006521735912
-0.667091401082	-1.006450416188
-0.666914626792	-1.00594648111
-0.66679967933	-1.005413255436
-0.666678267338	-1.005140592626
-0.666603881654	-1.004878749825

-0.666436449247	-1.004596134836
-0.666324295731	-1.004392897252
-0.666234292499	-1.004370917759
-0.666067427614	-1.004141710792
-0.665924140302	-1.003801355236
-0.665789203354	-1.003416918062
-0.665637982668	-1.002719164018
-0.665547532946	-1.002695148184
-0.665389466123	-1.002521438721
-0.665319302069	-1.002516247243
-0.665220916851	-1.002319785155
-0.66510338261	-1.002192546709
-0.664953921592	-1.001896027271
-0.664815955794	-1.001767636555
-0.6647107003	-1.001656772831
-0.664565629888	-1.001523025743
-0.66440232984	-1.000785045431
-0.664244921544	-1.000449513005
-0.664130049616	-1.00044460533
-0.664022547823	-1.000338523658
-0.663906750068	-0.999974726014
-0.66380713822	-0.999849594031
-0.663671949848	-0.999638361414
-0.663545358856	-0.999518877759
-0.663401435326	-0.999199629714
-0.663276904317	-0.998987351097
-0.663183841703	-0.998898394843
-0.663068443983	-0.998774330821
-0.66300549557	-0.998751952772
-0.662873899786	-0.998403864314
-0.662732022042	-0.998398491129
-0.662646347565	-0.998388493098
-0.662525917575	-0.99837608034
-0.662387660947	-0.998070261615
-0.662246784117	-0.997885346666
-0.662111418809	-0.997510139022
-0.661974799538	-0.99745347336
-0.661865655626	-0.997412873382
-0.661746820477	-0.997387449364
-0.661657768676	-0.997336636431
-0.661512583712	-0.996923368524
-0.661365892361	-0.996840090709
-0.661169369355	-0.996524693201
-0.661028398565	-0.996439565367
-0.660903248875	-0.996363387722
-0.660805650894	-0.996099145722
-0.660706797064	-0.996098783865
-0.66058380383	-0.995831815453
-0.660451064503	-0.995231810418
-0.660342862423	-0.995053108881
-0.660207023002	-0.994476822715
-0.660097294926	-0.994458733052
-0.659997184863	-0.994345263228
-0.65983692366	-0.994099708794

-0.659716062404	-0.993759873374
-0.659614208362	-0.993646545193
-0.659472102936	-0.992914006811
-0.659372605175	-0.992821360291
-0.659247138027	-0.992641020016
-0.659103417582	-0.991517451512
-0.65897633825	-0.991227918962
-0.658875504408	-0.991220021705
-0.658774717462	-0.990979350446
-0.658693649399	-0.990912647898
-0.658569250867	-0.990794397874
-0.658413279642	-0.990246247243
-0.658311865148	-0.989872561982
-0.658174288418	-0.989596630992
-0.657979849229	-0.988368686556
-0.657834065182	-0.988296235049
-0.657722868349	-0.988079678033
-0.657620622955	-0.987987805388
-0.657495219748	-0.987641932139
-0.657348504699	-0.987329782367
-0.657212168436	-0.987167876462
-0.657143233411	-0.987128030557
-0.657030677194	-0.987065865918
-0.656929002582	-0.986940853107
-0.656824218347	-0.986787326866
-0.656711953561	-0.986671871984
-0.656523469546	-0.985964670762
-0.656409139964	-0.985722070533
-0.656282612803	-0.985492054542
-0.656137804184	-0.985146948852
-0.656056021301	-0.985136861257
-0.65592295643	-0.985131491261
-0.655814932008	-0.984948633418
-0.655718407459	-0.98484586623
-0.655573788495	-0.984682874002
-0.655449528472	-0.984609970284
-0.655357039156	-0.984529869931
-0.655234544966	-0.984489901615
-0.65514852682	-0.98442842501
-0.654988972818	-0.984022183208
-0.654850488529	-0.984007308496
-0.654740217118	-0.983976024851
-0.654620543555	-0.983884028633
-0.654467904512	-0.983793032355
-0.654347830431	-0.983720427526
-0.654229114182	-0.983568157007
-0.65414853274	-0.983460695144
-0.654030504365	-0.983250623686
-0.653897730794	-0.98322773809
-0.653768527937	-0.982834036601
-0.653666623901	-0.982687681827
-0.653550939203	-0.982559513422
-0.653417278783	-0.982426749169
-0.653350060885	-0.982209747758

-0.653235784546	-0.982194526948
-0.653102469379	-0.98208492261
-0.652989630521	-0.981929742797
-0.652898629971	-0.981839701212
-0.652678513498	-0.981412944164
-0.65255555464	-0.981052297275
-0.652395887607	-0.980364539823
-0.652273652497	-0.98019973615
-0.652184723943	-0.980191569488
-0.65202772379	-0.979962733938
-0.65193041893	-0.979934990104
-0.651793330641	-0.979825436106
-0.651662095095	-0.979634561827
-0.651562989977	-0.97943754853
-0.651457074921	-0.979286146397
-0.651348845513	-0.979081373271
-0.651246119931	-0.978980151321
-0.651147228174	-0.97886118001
-0.651033521191	-0.978827808996
-0.650932736299	-0.97863917545
-0.650828185055	-0.978627807853
-0.650693421419	-0.978198318599
-0.650584083978	-0.978144249388
-0.650477528298	-0.978123861903
-0.650305312019	-0.9773155443
-0.650189541493	-0.977221385782
-0.65007654845	-0.977194087102
-0.649961216905	-0.977140459369
-0.649855491117	-0.977044883313
-0.649749352293	-0.976981751486
-0.649646556311	-0.976754863335
-0.649525545174	-0.976646482647
-0.649406209581	-0.976633886764
-0.649324208165	-0.976622369572
-0.649178490755	-0.976596937292
-0.64905939144	-0.976212991922
-0.648937701286	-0.975851494785
-0.64885554678	-0.97581857389
-0.648726949745	-0.975615173822
-0.648621353065	-0.975143022577
-0.648534741804	-0.97484463584
-0.648456090631	-0.974768511248
-0.648307717492	-0.974608207515
-0.648177117123	-0.974237638194
-0.648076561888	-0.974140557978
-0.647975162719	-0.973870645061
-0.647800486868	-0.973557724711
-0.647663762544	-0.973474785491
-0.647555845391	-0.973468518436
-0.647423558453	-0.973055414824
-0.647279787955	-0.972898547463
-0.647143142666	-0.972833154521
-0.647037844295	-0.972725225237
-0.646935114304	-0.972550274721

-0.64680356968	-0.972196461068
-0.646744570999	-0.972178235023
-0.646618315131	-0.972094224383
-0.64653551066	-0.97181521024
-0.646399403071	-0.971771172953
-0.64629533224	-0.97176878412
-0.646170765583	-0.971287382007
-0.64601979011	-0.970977931535
-0.645926043768	-0.970917458331
-0.645791087328	-0.97090652972
-0.645681356069	-0.970851868328
-0.645569931416	-0.970686201142
-0.645433123051	-0.970551287603
-0.645321929453	-0.970124795859
-0.645198212704	-0.969714105664
-0.645094232402	-0.969620743058
-0.644991092795	-0.969441599534
-0.644877338548	-0.969423333953
-0.64478712161	-0.96935052488
-0.644680388471	-0.969198040593
-0.644564106211	-0.968965676196
-0.644432184455	-0.96894199133
-0.644304903525	-0.968828008911
-0.644210914336	-0.968685494374
-0.644075063488	-0.968454098352
-0.643931183571	-0.967873912112
-0.643798999897	-0.967781466196
-0.643678606464	-0.967695142947
-0.643570322172	-0.967577512312
-0.64347235386	-0.967251785034
-0.643369566466	-0.967148522031
-0.643282103719	-0.967052676975
-0.643162628027	-0.966684596314
-0.64306235777	-0.966532751941
-0.64292530077	-0.96643571137
-0.642806195472	-0.965956095382
-0.642712679992	-0.96588342627
-0.642588560732	-0.96572164274
-0.642378802298	-0.964576714728
-0.642266062933	-0.964506078119
-0.642137010419	-0.964218135034
-0.642024189325	-0.96416005437
-0.641901017408	-0.964122875747
-0.641801221833	-0.964102444349
-0.641669600209	-0.964037005195
-0.641545175545	-0.96398214588
-0.641453413104	-0.963940665537
-0.641281025701	-0.963692535238
-0.641144574679	-0.963462613326
-0.64102249364	-0.963400410672
-0.640918874704	-0.963303674817
-0.640767425105	-0.962935027119
-0.640676653522	-0.962804957402
-0.640513449392	-0.962122543336

-0.640348715329	-0.961421899178
-0.64021211666	-0.961186006933
-0.640081640666	-0.961085088328
-0.6399388065	-0.960981797645
-0.639786646204	-0.960593945532
-0.63968322354	-0.960585742206
-0.639570771818	-0.960584677412
-0.639471387275	-0.960402780661
-0.639366381066	-0.960338691823
-0.639248909535	-0.960031254146
-0.639126535369	-0.959895424199
-0.639022048105	-0.959885823847
-0.638892351291	-0.959756913952
-0.638751034426	-0.959641621111
-0.638634609657	-0.959515925732
-0.638480416969	-0.958947399317
-0.638371976763	-0.958839762945
-0.638265308232	-0.958584701469
-0.63813566693	-0.958551371074
-0.638040191446	-0.958518063617
-0.63782412263	-0.957817526695
-0.637754784258	-0.95777663259
-0.637615371222	-0.957563048903
-0.637503956151	-0.957496183478
-0.63739891704	-0.957312382253
-0.637283250863	-0.957243780025
-0.637207461845	-0.957228684251
-0.637091527158	-0.957065709945
-0.63696764376	-0.957058458842
-0.636909383492	-0.957024630559
-0.63678335456	-0.956911764262
-0.636658084441	-0.956631705454
-0.636567682836	-0.956627693998
-0.636411234622	-0.956357045861
-0.636292946294	-0.956174883525
-0.636206787778	-0.95609764242
-0.636096874849	-0.956073992585
-0.63597353906	-0.956012303743
-0.635877450082	-0.955940389204
-0.635746749285	-0.955876341374
-0.635647391654	-0.955857328939
-0.63548703162	-0.955613616615
-0.635394237884	-0.955569527911
-0.63524285249	-0.954807099442
-0.635146265891	-0.954577887356
-0.635015009595	-0.954137832482
-0.634892376629	-0.953975266037
-0.634805694255	-0.953898553137
-0.634733609858	-0.953849236776
-0.634639494652	-0.953746099119
-0.634532652925	-0.953557320928
-0.634377047412	-0.9528503005
-0.634282129758	-0.952774446246
-0.634151741908	-0.952631796486

-0.634001931657	-0.952619454042
-0.633862825013	-0.952494739653
-0.633701049561	-0.952420895726
-0.633567790758	-0.952153019732
-0.63345038928	-0.952034532278
-0.633340343269	-0.95188810097
-0.63320690104	-0.951383526635
-0.63306621748	-0.951177400176
-0.63295420301	-0.950914186912
-0.632807359093	-0.950862216367
-0.632726466661	-0.950623349476
-0.632628847843	-0.950505414313
-0.632494345373	-0.95035327936
-0.632342321139	-0.950294052239
-0.632216153548	-0.950086583388
-0.632110635494	-0.94984985337
-0.632006497555	-0.949829952427
-0.631847086178	-0.949445674052
-0.631656065278	-0.948942926359
-0.631505354794	-0.948485484422
-0.631420894927	-0.948470146868
-0.63126779882	-0.948195573144
-0.631194523814	-0.948155506329
-0.631079199381	-0.947989701441
-0.630971938659	-0.947946325302
-0.630884902801	-0.947740462275
-0.630737405869	-0.947635548377
-0.630627552776	-0.947613552193
-0.630490042577	-0.947589051929
-0.630340346024	-0.947343148608
-0.630257140933	-0.947301802578
-0.63012066341	-0.947246157498
-0.629988676521	-0.947195713663
-0.629888208706	-0.947078119785
-0.629745706598	-0.946926475347
-0.62964907376	-0.946851479561
-0.629532242305	-0.946770944335
-0.629413436818	-0.946702832535
-0.629314590504	-0.946453981963
-0.629202732802	-0.945924865608
-0.629056559231	-0.945765425807
-0.628951011637	-0.945695129698
-0.628820331306	-0.945440648968
-0.628701443884	-0.945107352228
-0.628553171502	-0.944450568614
-0.62842867976	-0.944063892438
-0.628255592927	-0.944014734789
-0.628154846631	-0.94382485497
-0.628036448817	-0.943749599647
-0.62790709311	-0.94374225939
-0.627725579178	-0.942576597602
-0.627582903142	-0.94242496039
-0.627489565512	-0.942294325113
-0.627370949336	-0.942259816027

-0.62724345366	-0.942082665749
-0.627112805728	-0.94185363291
-0.626978427687	-0.941839044631
-0.626861477699	-0.941663906638
-0.626729962353	-0.941161421864
-0.626578160312	-0.940693528697
-0.626464791217	-0.94064901444
-0.626334191456	-0.940216071295
-0.626213097433	-0.939826180148
-0.626095471874	-0.939692900802
-0.625986992172	-0.939329554556
-0.625891127622	-0.939225897614
-0.625775483427	-0.93903886757
-0.625704024854	-0.938989824713
-0.625595275167	-0.938825480979
-0.625476826411	-0.938771236209
-0.625317518383	-0.938488143046
-0.625183568131	-0.938361163858
-0.625070088	-0.938226690755
-0.624923410595	-0.937728221107
-0.62482240412	-0.937639594778
-0.624680464992	-0.937431990383
-0.62455792805	-0.937359682618
-0.624292172328	-0.936902599681
-0.624201488965	-0.936822804433
-0.624090206056	-0.936626481036
-0.623977630669	-0.936608914001
-0.623835343247	-0.936474757914
-0.623716084036	-0.936442699215
-0.623589559759	-0.936333218902
-0.623446747569	-0.935809998567
-0.623336650513	-0.93577154377
-0.623201380421	-0.935409279292
-0.623095876968	-0.935329994868
-0.622940706797	-0.93527913482
-0.622812872147	-0.934655211119
-0.622721693139	-0.934651660642
-0.622556266553	-0.934155918666
-0.622437768113	-0.933861384607
-0.622302824227	-0.933740695536
-0.622195889263	-0.933669621218
-0.622079127704	-0.933582267078
-0.621962007398	-0.933381090177
-0.621821573713	-0.933341065364
-0.621703242693	-0.933145705945
-0.621563132025	-0.93304672147
-0.621469113282	-0.932975879945
-0.621350251863	-0.932807583308
-0.621191267566	-0.932660423568
-0.62105371425	-0.932479538958
-0.620940422436	-0.93224714651
-0.620817337945	-0.932233353902
-0.620671504947	-0.932048325396
-0.620523987608	-0.931708693543

-0.620407634662	-0.931657730092
-0.6202438604	-0.931494455119
-0.620131743064	-0.93142012728
-0.620016873084	-0.931405889783
-0.619864475952	-0.931128209897
-0.619767241575	-0.931058288769
-0.619627607784	-0.930847161807
-0.619536348722	-0.930743200892
-0.6194197482	-0.930535041607
-0.619290596761	-0.930351824535
-0.619149579037	-0.930025467576
-0.619007899141	-0.92953034065
-0.618816964078	-0.928880992659
-0.618686480009	-0.928809512826
-0.618550604163	-0.928786597841
-0.618421992836	-0.928585602535
-0.618329637391	-0.928583632214
-0.618226217998	-0.928236409703
-0.618073313683	-0.928219999357
-0.61794086186	-0.927909768953
-0.617874910398	-0.927881089439
-0.617800673548	-0.927797260732
-0.617701030892	-0.927706474658
-0.617552797333	-0.927374412749
-0.617388073616	-0.926806709466
-0.617231226782	-0.926776765167
-0.617077458102	-0.926551722955
-0.616979169041	-0.926465546473
-0.616879002279	-0.926293002304
-0.616774785985	-0.926068232276
-0.616667646516	-0.925812947687
-0.616572546573	-0.925796151212
-0.616472197139	-0.925688002231
-0.616346181535	-0.925656711642
-0.616246170044	-0.925529217143
-0.616108794614	-0.925182205441
-0.615967171396	-0.92496524241
-0.61580422821	-0.9239139983
-0.615683375363	-0.923714144986
-0.615566289874	-0.923447888626
-0.615479376294	-0.923225432652
-0.615332286085	-0.923058299116
-0.615205142727	-0.92289229157
-0.615090827115	-0.922797799048
-0.614974614876	-0.922787080786
-0.614836880794	-0.92275090111
-0.614729388065	-0.922678347247
-0.614616159979	-0.922419916238
-0.614491642219	-0.922408506638
-0.614354494707	-0.922333207984
-0.614220261491	-0.922091238752
-0.614096880433	-0.922080400917
-0.614005983947	-0.922019849682
-0.613884377585	-0.92186100744

-0.613734729071	-0.921856235366
-0.61357285541	-0.921360993661
-0.61341987043	-0.921223414293
-0.613300682671	-0.921048926633
-0.613203276557	-0.921002167137
-0.613088037954	-0.920809732158
-0.612985260737	-0.920653207825
-0.612890751113	-0.920619149057
-0.612742435886	-0.920041485239
-0.612631765082	-0.920040942377
-0.612540585671	-0.91999557777
-0.612397818857	-0.9199616447
-0.612303498105	-0.919925657749
-0.612149938086	-0.919145657218
-0.612046033326	-0.918726538304
-0.611916430825	-0.918614232077
-0.611789251323	-0.91858564109
-0.611682845142	-0.918477966278
-0.611563550322	-0.918471589422
-0.611465408917	-0.918467836334
-0.611323604334	-0.91814172701
-0.61121656051	-0.917969633896
-0.611092663746	-0.917797303739
-0.61099225593	-0.917768391159
-0.610876785383	-0.917687608685
-0.610721663969	-0.917358060879
-0.610557347523	-0.916785435722
-0.610425275423	-0.916739441073
-0.610314341211	-0.916724424428
-0.610162421283	-0.916653313265
-0.610064449941	-0.916232688327
-0.609981647493	-0.916095628794
-0.609820126625	-0.915454926903
-0.609717091048	-0.915404750074
-0.609642716057	-0.915301773007
-0.609522850203	-0.915254805068
-0.60936178615	-0.915048252199
-0.609266414307	-0.914967811434
-0.609178027286	-0.914922791949
-0.60908776202	-0.914810344915
-0.608953386005	-0.914464574479
-0.608828973446	-0.914449974767
-0.608703373376	-0.913981461652
-0.608570570661	-0.913782975056
-0.608457612309	-0.913782607486
-0.608309577521	-0.91371912064
-0.608239403868	-0.913713214216
-0.60811439168	-0.913520132287
-0.608006622978	-0.913445287988
-0.607914642738	-0.91325320877
-0.607768003017	-0.912898197291
-0.607683120247	-0.912834277339
-0.607577299907	-0.91269508975
-0.607472512545	-0.91264648003

-0.6073555024	-0.912620680245
-0.60727763075	-0.912563427034
-0.607162335962	-0.912223341399
-0.607037096983	-0.91217354094
-0.606932690167	-0.911946528272
-0.606799817375	-0.911864206695
-0.606685515966	-0.911772313478
-0.606523186041	-0.91151600362
-0.606419907493	-0.911470740815
-0.606228036703	-0.910712888616
-0.606119224	-0.91051579311
-0.606027332114	-0.910323170079
-0.605934211628	-0.910294992158
-0.605866403412	-0.910230185751
-0.605763046162	-0.910207659997
-0.605655263903	-0.909955342429
-0.605562972673	-0.909928765867
-0.605416826256	-0.909667838156
-0.605280413098	-0.909667466753
-0.60515103407	-0.909577538277
-0.605029283472	-0.909378693403
-0.604877628189	-0.909174571253
-0.604741924218	-0.90910107457
-0.604668643173	-0.908921111357
-0.60457928163	-0.908896809509
-0.604451030261	-0.908375705057
-0.604376467225	-0.908153845521
-0.604220428992	-0.908008210762
-0.60409761437	-0.907898220123
-0.603999736866	-0.907895303568
-0.603882207573	-0.90762972973
-0.603791724861	-0.907366932396
-0.60369075882	-0.906925458627
-0.603579175045	-0.906818508875
-0.603432319075	-0.906692883907
-0.603317808618	-0.906632533672
-0.603178711652	-0.906548114672
-0.603078535695	-0.906424807167
-0.602957649837	-0.906340832038
-0.60284667481	-0.90623054182
-0.602730728879	-0.906032308346
-0.602552385534	-0.905615919354
-0.602450943277	-0.905575436825
-0.602331857522	-0.905239002569
-0.602216380502	-0.905204882326
-0.60209323744	-0.90477351865
-0.601949441687	-0.904614437256
-0.601794967574	-0.90459308937
-0.601687304593	-0.904291768899
-0.601592554153	-0.904158836919
-0.601509012138	-0.904154146425
-0.601386817534	-0.904004783791
-0.601287936637	-0.903946330283
-0.601161946123	-0.903529406852

-0.601023717091	-0.902993590346
-0.600919752848	-0.902981264055
-0.600774422282	-0.902547178662
-0.600676130427	-0.902479583752
-0.600554090048	-0.902304449696
-0.600435440484	-0.902259603481
-0.600290224002	-0.902169710589
-0.600169434593	-0.902013422604
-0.600038930889	-0.901874458901
-0.599912608051	-0.901616026885
-0.599777726239	-0.901371392852
-0.599671095608	-0.901306261163
-0.59954324133	-0.901237702126
-0.599376142155	-0.900522174334
-0.599236231861	-0.900497635678
-0.59909008062	-0.900453952242
-0.598977328492	-0.900335085432
-0.598812942449	-0.900195139157
-0.598705415413	-0.89994298857
-0.598550002724	-0.899723627871
-0.598449898201	-0.899537901191
-0.598345283708	-0.899536314634
-0.59823500562	-0.899489082769
-0.598132324382	-0.89944621819
-0.598002853097	-0.899328032315
-0.597868993119	-0.899310926109
-0.597703197783	-0.89921692992
-0.597593350733	-0.899059977708
-0.597445423667	-0.898829350969
-0.597340133977	-0.898701013761
-0.59721488711	-0.898397810842
-0.59705871755	-0.898285823912
-0.596958061096	-0.898083445097
-0.596858057469	-0.897852434285
-0.596709707571	-0.89745928251
-0.59660258145	-0.897382963747
-0.59644423518	-0.896418570155
-0.596354484487	-0.896387078539
-0.596265422917	-0.89632681442
-0.596103196882	-0.896233981274
-0.595986783235	-0.895860439666
-0.595884238571	-0.895805115412
-0.595775513397	-0.895764730263
-0.595672373199	-0.895426844376
-0.595559958505	-0.895200072554
-0.595382666932	-0.894931361584
-0.595278768937	-0.894910028369
-0.595141676944	-0.894690811897
-0.595035297775	-0.894557863397
-0.594890772273	-0.89421351371
-0.594772012933	-0.894068887283
-0.594610414394	-0.893330107074
-0.594506016964	-0.893205509295
-0.594376091856	-0.893068806235

-0.59429380159	-0.893060242013
-0.594213789706	-0.892940650576
-0.594097699554	-0.892930729519
-0.593943053962	-0.892920096281
-0.593811965778	-0.892686502498
-0.593685840982	-0.892125262748
-0.593600052001	-0.891980759847
-0.593462251817	-0.89184473088
-0.593350074219	-0.891796036094
-0.593222009612	-0.891646895787
-0.593094150049	-0.891532592895
-0.592984317249	-0.891528869501
-0.592901268232	-0.891523893635
-0.592797243147	-0.891449478805
-0.592663582353	-0.891141071694
-0.592544190373	-0.89095293876
-0.592378610068	-0.89063499996
-0.592206509818	-0.890493819566
-0.592123565623	-0.890436693841
-0.592033687461	-0.890426482483
-0.591900199642	-0.890296288612
-0.59180005801	-0.890090186886
-0.591649297989	-0.889981964746
-0.591515099158	-0.889873724429
-0.591413407896	-0.889713088343
-0.591312377669	-0.889648754651
-0.591233411345	-0.889632665342
-0.591158938687	-0.889525856356
-0.590992740185	-0.889476645537
-0.590823082929	-0.889105904329
-0.590711916342	-0.888992646452
-0.590629071514	-0.88897674512
-0.590517605131	-0.888599600585
-0.590419945483	-0.888414215674
-0.59028338106	-0.888383458198
-0.590175415469	-0.888273956674
-0.58998238649	-0.887643153376
-0.58978703205	-0.887409129515
-0.589698678394	-0.887351474127
-0.589610485312	-0.887345136427
-0.589498675783	-0.887216425365
-0.589387583693	-0.887166978466
-0.589298963107	-0.887058427742
-0.589203253317	-0.887011148484
-0.589080534814	-0.886974507083
-0.588970235714	-0.886956972234
-0.588853549245	-0.886927677637
-0.588738759563	-0.886661029777
-0.588618275938	-0.886543929954
-0.588486801546	-0.886511786493
-0.588339206116	-0.886258357485
-0.588219940809	-0.886045657105
-0.588107520894	-0.885391176116
-0.588001150747	-0.885382819349

-0.587950961361	-0.885332182662
-0.587851674191	-0.885163091476
-0.587759842409	-0.885115906901
-0.587625438938	-0.884999784438
-0.58748723733	-0.884853055318
-0.587340418172	-0.884480661366
-0.58722682755	-0.884252583241
-0.587130739469	-0.884157307814
-0.587033970809	-0.884102607925
-0.586937112618	-0.883936093088
-0.586783048169	-0.883791487199
-0.586670888007	-0.883693975089
-0.586513368125	-0.883601578305
-0.58639527974	-0.883519449317
-0.586259045706	-0.883411839925
-0.586118606558	-0.883241188936
-0.586027230363	-0.883197026374
-0.58588529409	-0.883121645416
-0.585775963555	-0.883021314865
-0.585654218236	-0.882934470435
-0.585549723909	-0.882627625555
-0.585416481067	-0.882552916045
-0.58527792518	-0.882396357725
-0.585164942305	-0.882057769095
-0.585051309813	-0.881800067031
-0.584923304574	-0.88172767113
-0.584779778719	-0.881350243951
-0.584688180254	-0.881292667817
-0.584616324086	-0.881267248695
-0.584517743334	-0.881228820135
-0.584370202248	-0.880934074169
-0.584226143107	-0.880179268685
-0.584106413586	-0.880083035523
-0.583966630234	-0.879667646559
-0.583865097146	-0.879115253712
-0.583739116853	-0.879003101317
-0.58363163674	-0.878869512294
-0.583518949774	-0.878819080538
-0.583431274274	-0.878594326097
-0.583362171043	-0.878589360831
-0.583252611541	-0.878540888858
-0.583085372376	-0.877874925649
-0.582965654698	-0.877860257927
-0.582796997522	-0.877291571582
-0.582683256849	-0.876961668239
-0.582525387368	-0.876690808309
-0.582407967611	-0.876690465956
-0.582299272324	-0.87665865281
-0.582182872154	-0.876521802791
-0.58206739507	-0.875976760757
-0.581911702085	-0.875494439259
-0.581838835468	-0.875369724758
-0.581699664653	-0.87533445184
-0.581598037642	-0.875211048586

-0.581458003828	-0.874975900121
-0.581334918177	-0.874974321856
-0.581192871034	-0.874789867938
-0.581068301525	-0.874720503958
-0.580994981616	-0.874688573988
-0.58087888752	-0.874457077485
-0.580799987025	-0.874392922861
-0.580721966264	-0.874363539193
-0.580647640093	-0.874164949607
-0.5805461812	-0.874127668697
-0.580465478364	-0.874028796312
-0.580340330599	-0.87401222624
-0.580185033342	-0.87378789696
-0.580083755282	-0.873730540939
-0.579943993337	-0.873476366265
-0.579823035597	-0.873447094495
-0.579682841239	-0.872569944497
-0.579581390466	-0.872500904197
-0.579471279652	-0.872414809388
-0.579354614275	-0.872245244751
-0.579257540572	-0.87207045044
-0.57911121598	-0.871569423562
-0.578938627672	-0.870961646382
-0.578785511403	-0.870932465313
-0.57869880132	-0.870930877461
-0.57854367454	-0.870761071871
-0.578373039821	-0.870433103408
-0.578264784234	-0.870220651685
-0.578155499445	-0.870187290325
-0.578054987648	-0.870096727659
-0.577931233045	-0.869766722875
-0.577819487088	-0.869753287303
-0.577713503156	-0.869743224446
-0.57756471428	-0.869709558418
-0.577438453312	-0.86926910886
-0.57736902148	-0.869186113753
-0.577246395738	-0.868692137755
-0.577143645272	-0.868671421875
-0.576987008281	-0.867828434557
-0.576848754531	-0.867696707972
-0.576734848048	-0.867389616537
-0.57661714502	-0.867355317763
-0.576460792877	-0.866856305972
-0.576336141731	-0.866800747389
-0.576227015171	-0.866799991104
-0.576048015972	-0.86636260625
-0.575947401266	-0.866292711338
-0.575799507077	-0.865632786716
-0.575715752917	-0.865321015434
-0.575589594587	-0.865166526218
-0.575460823384	-0.865097443345
-0.575307766197	-0.864800783275
-0.575222036549	-0.864787748392
-0.57509071297	-0.864477580996

-0.574905596898	-0.864120838463
-0.574814645933	-0.86406939048
-0.57466329813	-0.86385234765
-0.574586771757	-0.863833759355
-0.574475695123	-0.863508718977
-0.574355235095	-0.863434286116
-0.574242958031	-0.863354939959
-0.574152095006	-0.863326836527
-0.574037519311	-0.863137100002
-0.573931321458	-0.863079797823
-0.573822625166	-0.86264810245
-0.573680327838	-0.862593314829
-0.573559935375	-0.862507797529
-0.573464895847	-0.862434676316
-0.573334389832	-0.862379366483
-0.573184919197	-0.862295806319
-0.573105384388	-0.8622513832
-0.572959079289	-0.862159363434
-0.572836443889	-0.862048077295
-0.572750803965	-0.861991574401
-0.572626101107	-0.861869429928
-0.572496234985	-0.861863878545
-0.572417155414	-0.861851993678
-0.572271257332	-0.861570919938
-0.572128004116	-0.861381724519
-0.571994879275	-0.861136075393
-0.571866777169	-0.861018966108
-0.57177787008	-0.860953732053
-0.571634873702	-0.860618638189
-0.571500562701	-0.860530620337
-0.571404987973	-0.860470626376
-0.571315688303	-0.860469813369
-0.571211171234	-0.860250734942
-0.571086091613	-0.860155596126
-0.570994380454	-0.860032318388
-0.570836576185	-0.859811211458
-0.570725760581	-0.85953116125
-0.570602813767	-0.859454154979
-0.570427405849	-0.858793208708
-0.570335813896	-0.85852929032
-0.570238925152	-0.858504574749
-0.570151568155	-0.858501498729
-0.570047426119	-0.8581177329
-0.569917049983	-0.85799525808
-0.56973896016	-0.857839236004
-0.569649554112	-0.857808292701
-0.569551195409	-0.857582445592
-0.569411213549	-0.857552027486
-0.569301964051	-0.857490311009
-0.56919512907	-0.857324259577
-0.569093644467	-0.857282401902
-0.568983363393	-0.857266852106
-0.568826310714	-0.856741691179
-0.568738453847	-0.856730972883

-0.568563949577	-0.856188360599
-0.568440039061	-0.855988831828
-0.568348539773	-0.855934022104
-0.568223277917	-0.855873292196
-0.568108447098	-0.855446650245
-0.567998294174	-0.855283567547
-0.567910918547	-0.85514848897
-0.567757447154	-0.854632546872
-0.567639652475	-0.854598008596
-0.567525567046	-0.854506778747
-0.567468523657	-0.854493681714
-0.567348499367	-0.854330603327
-0.567177718346	-0.854218777232
-0.567070376073	-0.854173848942
-0.566953061399	-0.854062752714
-0.566791900439	-0.853342810034
-0.566681752295	-0.853114029915
-0.566579931377	-0.85284659967
-0.566459525608	-0.852504880392
-0.56635292654	-0.852445103198
-0.566220219929	-0.852241303975
-0.56606475321	-0.851963810034
-0.565936157013	-0.851927284907
-0.565847774336	-0.851821732236
-0.565738877144	-0.851524249333
-0.565574218074	-0.850729167634
-0.565496035909	-0.850580321896
-0.565356896161	-0.850523229087
-0.565215850426	-0.850074933294
-0.565140790588	-0.849999073464
-0.565039550618	-0.849954184171
-0.564928850738	-0.849279143469
-0.564748297039	-0.84906792431
-0.564636017248	-0.848647352247
-0.56445526525	-0.848131212685
-0.564355544465	-0.848111957998
-0.564221266263	-0.847477370602
-0.564069992288	-0.847190789808
-0.563985986251	-0.847090322691
-0.563879712878	-0.847016025151
-0.563721657041	-0.84661167552
-0.563633220584	-0.84658893768
-0.563515871561	-0.846530846844
-0.563429251812	-0.846460559283
-0.563331698225	-0.846421767893
-0.563229676827	-0.846257177915
-0.563143260454	-0.846243405264
-0.56301784725	-0.846173444943
-0.562904370245	-0.846103211113
-0.562834563705	-0.84604947428
-0.562728405682	-0.845973958318
-0.562630143443	-0.845826571894
-0.562515637005	-0.845673960239
-0.56238536688	-0.845481813514

-0.562268944689	-0.845468255425
-0.562153495987	-0.845461305509
-0.562048397001	-0.845234311536
-0.561948324088	-0.845207251883
-0.56182752973	-0.845011698547
-0.561740088309	-0.844970408894
-0.561617378577	-0.844754023182
-0.561498575641	-0.844584643285
-0.56141262825	-0.844581871983
-0.561276577299	-0.844576924149
-0.561173045693	-0.844551347793
-0.561058166701	-0.844527395795
-0.560958693952	-0.844464514013
-0.560844334172	-0.844457599674
-0.560723278145	-0.844250062883
-0.560634872544	-0.844231953218
-0.56051208373	-0.843578539023
-0.560441744169	-0.843537233369
-0.560293085209	-0.842848139161
-0.56016842352	-0.842805886808
-0.560062589494	-0.842439686665
-0.559966584941	-0.842358577361
-0.559877385854	-0.84234630002
-0.559809830878	-0.842209212119
-0.559699566015	-0.842158567073
-0.55957260885	-0.842042921161
-0.559456227938	-0.841915218986
-0.559339556616	-0.841867820534
-0.559206699859	-0.841714997801
-0.559123045887	-0.841564477362
-0.558982070271	-0.841104996707
-0.558875746228	-0.841015129099
-0.558763489892	-0.840776474878
-0.558644583629	-0.840139225707
-0.558521016574	-0.839925127741
-0.558429296624	-0.839878256023
-0.55826519291	-0.839223043959
-0.558126711168	-0.838725253711
-0.558053651298	-0.838574301473
-0.557962250709	-0.838280876849
-0.557821338276	-0.837960718929
-0.557712998374	-0.83784712115
-0.557606482566	-0.837744509791
-0.557536304943	-0.837729997545
-0.557436930607	-0.837719716175
-0.557326671378	-0.837202631727
-0.557215883513	-0.837000554075
-0.557144716815	-0.836961907272
-0.557046228474	-0.836906226356
-0.556974584066	-0.836770164672
-0.556847987618	-0.836766245631
-0.556725902714	-0.836724077217
-0.556596001082	-0.8364894247
-0.556510825308	-0.83647713741

-0.556422803962	-0.836443560145
-0.556340877493	-0.836313199636
-0.556241624077	-0.836302908653
-0.556121912924	-0.836253116032
-0.556014777911	-0.83603029135
-0.555873343458	-0.835930691855
-0.555785206048	-0.835804684517
-0.555697797403	-0.835720135298
-0.555600021529	-0.835710194981
-0.555432916545	-0.835434219967
-0.555293956258	-0.835122843023
-0.555173711951	-0.834893559895
-0.555084901923	-0.834850206594
-0.55492220907	-0.834251016609
-0.55479987581	-0.834176232833
-0.554693653736	-0.834026742411
-0.554603618205	-0.833901061804
-0.554506720483	-0.833652346107
-0.554339511388	-0.833067051528
-0.554209233465	-0.832865969493
-0.554109884649	-0.832812492595
-0.554046493042	-0.832800091165
-0.55391786296	-0.832570885965
-0.553838291825	-0.832474313541
-0.553737937519	-0.832436655508
-0.553677647867	-0.832434801022
-0.553595517735	-0.832364930619
-0.553475293128	-0.83223430597
-0.553364156184	-0.832139567999
-0.553241758887	-0.831863123039
-0.553158524934	-0.83181705167
-0.553053886582	-0.831620479351
-0.552932990097	-0.831500436806
-0.552798909747	-0.831096560001
-0.552700835789	-0.831074633508
-0.552630782953	-0.831002135344
-0.552513563583	-0.830985925095
-0.552401027035	-0.830591751576
-0.552224249359	-0.830525684554
-0.552146076026	-0.830482237122
-0.552067612473	-0.830383149357
-0.551942702668	-0.83000741197
-0.551810658292	-0.829883781967
-0.551729591254	-0.829799427894
-0.551661666276	-0.829793273615
-0.551570177276	-0.829650492634
-0.551459853398	-0.829645434579
-0.551351408186	-0.829473631179
-0.551252625215	-0.829436283844
-0.551142966859	-0.829432938951
-0.551036422121	-0.82927009195
-0.550940808535	-0.829257443444
-0.550822126712	-0.829171916866
-0.550742186023	-0.828988021713

-0.550613085996	-0.828897400886
-0.550509790798	-0.828868536192
-0.550388362713	-0.8287294929
-0.550295277351	-0.828708012562
-0.550183344142	-0.828683031551
-0.550081811464	-0.828413571653
-0.549962502182	-0.828227916762
-0.549822295832	-0.828044773038
-0.549738119865	-0.827966976673
-0.549658195545	-0.827919600846
-0.549542239745	-0.827779678464
-0.549422275482	-0.827752954992
-0.54928487068	-0.827482061243
-0.54917666541	-0.82738640639
-0.549096134659	-0.827362309318
-0.548977358909	-0.827295261509
-0.548882065947	-0.827284744683
-0.548753269449	-0.826901568987
-0.54866672409	-0.826871389498
-0.548556679397	-0.826568048436
-0.548450383256	-0.826548347704
-0.548326831313	-0.826394652133
-0.548180693946	-0.826362081885
-0.548023985422	-0.826263947619
-0.5478903059	-0.826235443495
-0.54777776024	-0.826194589587
-0.547630106091	-0.826051919305
-0.547475699961	-0.825686584969
-0.547374833049	-0.825645397222
-0.547273975393	-0.825454296703
-0.547179922497	-0.825377373713
-0.547063519369	-0.825374995391
-0.546948421764	-0.82525625871
-0.54684318695	-0.825251866879
-0.546734149949	-0.825234750363
-0.546632170232	-0.82508919354
-0.546490403699	-0.824903936135
-0.546372445547	-0.824740643468
-0.546270615083	-0.824669502501
-0.546163664923	-0.824665548078
-0.546084496163	-0.824535843996
-0.545988363644	-0.824442038964
-0.545911259788	-0.824286422838
-0.5457555434	-0.8239601653
-0.545675622879	-0.823896057004
-0.545596610197	-0.82382727911
-0.545493376518	-0.823812028719
-0.545403802385	-0.823794836446
-0.54532226703	-0.823765590148
-0.545125001426	-0.823065131575
-0.544907695419	-0.822549770971
-0.544800995555	-0.822457382166
-0.544698144206	-0.822180709044
-0.544613962014	-0.822147343682

-0.544510213305	-0.822135173045
-0.544402885249	-0.82203254643
-0.54431799996	-0.822012992039
-0.544219937012	-0.821973162555
-0.544122468706	-0.82178624241
-0.543987241463	-0.821565941089
-0.543876174375	-0.821542517795
-0.543782859048	-0.82141540219
-0.543676894046	-0.821186169308
-0.543544850623	-0.821110955395
-0.543401992683	-0.820965739201
-0.543322273236	-0.820826748804
-0.543178333371	-0.820812276108
-0.543005455618	-0.820717605622
-0.542905490674	-0.820670857857
-0.542807197772	-0.820503927939
-0.54265395179	-0.820002239503
-0.542445786989	-0.819637812128
-0.542334330737	-0.819503497438
-0.542236438585	-0.819452531039
-0.54212838632	-0.819275356232
-0.541975659388	-0.819169708724
-0.541878595003	-0.819056840039
-0.541768603054	-0.818982835434
-0.541661126526	-0.818895336446
-0.541540755727	-0.818695290973
-0.541442751937	-0.818554647922
-0.541286606	-0.818365027829
-0.54117229684	-0.818208988553
-0.541090493614	-0.817972461345
-0.540985950467	-0.817863150848
-0.540886258599	-0.817799636295
-0.54075418396	-0.817291456837
-0.540671329171	-0.817264739892
-0.540563204612	-0.817076895939
-0.540424208207	-0.816620127049
-0.540266379233	-0.816291526832
-0.540181420483	-0.816291045902
-0.540068537819	-0.816198999255
-0.539955603293	-0.815810132555
-0.53985599926	-0.815571539607
-0.539761222958	-0.815566156194
-0.539650967498	-0.815489330322
-0.539537396588	-0.815168462544
-0.539428378761	-0.815120245184
-0.539285861922	-0.814849779905
-0.539208314071	-0.814764390182
-0.539072067504	-0.814262183784
-0.538955019567	-0.814192039264
-0.538892367378	-0.814065009862
-0.538785594199	-0.813897908191
-0.53871503651	-0.813882255841
-0.538627034848	-0.813752346175
-0.538522382839	-0.813588277205

-0.538417704317	-0.81350532748
-0.538263307401	-0.813031675893
-0.538124115236	-0.81283396666
-0.538013799776	-0.812761709822
-0.53791474473	-0.812601037882
-0.537827645184	-0.812479528116
-0.537718477923	-0.812434038406
-0.537630120868	-0.812145876715
-0.537523559206	-0.812047195366
-0.537446493438	-0.812012001056
-0.537352828831	-0.812001842718
-0.537262286709	-0.811820311277
-0.537142177051	-0.811706038082
-0.537007994664	-0.811229090422
-0.53686346074	-0.810626982699
-0.536791406489	-0.810569695578
-0.536698490809	-0.810289675314
-0.536565589562	-0.810165912562
-0.536434017666	-0.810093946104
-0.536297923845	-0.809398489691
-0.536188821366	-0.809394181854
-0.536072761443	-0.809364335114
-0.535995087949	-0.809358160403
-0.535866741508	-0.809327570588
-0.53574443814	-0.8092546246
-0.535644587236	-0.809216062837
-0.53552476082	-0.809099456681
-0.535416297837	-0.80890839496
-0.535293313866	-0.808826223986
-0.535110676921	-0.808138841698
-0.535034082398	-0.808117379856
-0.534943397522	-0.807987500731
-0.53483802801	-0.807925391783
-0.534739246786	-0.807921616598
-0.534656155406	-0.807790383741
-0.534553419285	-0.807782081553
-0.534457345953	-0.807521840304
-0.53431594875	-0.807500457543
-0.53420423911	-0.807253433537
-0.534101966396	-0.807192989291
-0.534019596279	-0.807188025701
-0.533944549332	-0.806850676512
-0.533839629176	-0.806556138441
-0.53374221505	-0.806539438322
-0.533614455304	-0.80641299742
-0.533514554521	-0.806214889062
-0.533391736295	-0.806085281059
-0.533275804237	-0.805964740853
-0.533181047422	-0.805923349977
-0.533067521164	-0.805865414219
-0.532966465169	-0.805846930946
-0.532842698778	-0.805771886753
-0.532720665079	-0.8056079965
-0.532604772416	-0.805142113757

-0.532500239568	-0.805121338847
-0.53235869269	-0.805073350844
-0.5322008649	-0.804917062117
-0.532083855157	-0.804862188577
-0.531980603581	-0.804629581374
-0.531874742099	-0.804310159952
-0.531763094533	-0.804032517852
-0.53165209102	-0.803977870523
-0.531581392911	-0.803966651581
-0.531472435958	-0.803870476618
-0.53137070367	-0.803742822121
-0.531249348306	-0.803584023452
-0.531139661687	-0.803526172362
-0.531026914296	-0.803374469159
-0.53091062188	-0.803268915072
-0.530820644546	-0.803218208158
-0.530664043093	-0.802976736386
-0.530569234303	-0.802910932685
-0.530459652728	-0.802428009761
-0.530326832968	-0.802314561688
-0.530227750942	-0.802156280144
-0.530138687303	-0.802024812483
-0.530027862903	-0.801679437801
-0.52991481899	-0.801608130576
-0.529784583433	-0.801483504654
-0.529656799002	-0.801366059974
-0.529558029459	-0.801321637265
-0.529419123137	-0.801319445971
-0.52929433476	-0.800898196288
-0.529200499381	-0.800764525029
-0.529077309748	-0.80068186092
-0.528962254557	-0.800523661066
-0.528855268038	-0.800328628534
-0.528726085608	-0.800274530315
-0.528625510674	-0.799786414645
-0.528517923871	-0.799318318217
-0.528377882612	-0.799059830667
-0.528275982576	-0.798942659539
-0.528162413535	-0.798686755582
-0.528033919938	-0.798261801226
-0.527945415433	-0.798189204336
-0.527855151348	-0.798103091609
-0.5277687458	-0.798095956734
-0.527639202954	-0.798006741311
-0.527477047394	-0.797342584252
-0.527334927991	-0.797327737659
-0.527203004349	-0.797254003851
-0.527112637467	-0.797100818008
-0.526983517069	-0.796876608123
-0.526838600922	-0.796652486621
-0.526745906281	-0.796576518314
-0.526571428449	-0.795502273823
-0.526455083811	-0.795449360432
-0.526363400459	-0.795217442518

-0.526232833849	-0.795149572134
-0.526100523563	-0.79505598721
-0.525978061293	-0.79493283008
-0.525852444561	-0.794277085628
-0.525778004563	-0.794264836196
-0.525661966086	-0.793890193431
-0.525577387598	-0.793849803532
-0.525438282606	-0.793734707943
-0.525303779912	-0.793546369315
-0.525207950253	-0.793519534506
-0.525093625573	-0.793515442706
-0.524979358809	-0.793486285049
-0.52487276931	-0.793452858788
-0.524753864076	-0.793260221829
-0.524636557019	-0.793175961871
-0.52451860964	-0.792980580744
-0.524441538138	-0.79295587394
-0.524320827369	-0.792589660717
-0.524197552253	-0.792222165061
-0.524074586809	-0.792066871304
-0.523937062234	-0.791543445396
-0.523847717667	-0.791456880998
-0.523713356882	-0.791190800814
-0.52359817403	-0.791089452823
-0.523468961295	-0.790958302112
-0.523330210341	-0.79074408084
-0.523199205253	-0.790624932802
-0.523100235792	-0.790606609622
-0.523015433232	-0.790576634087
-0.522897674924	-0.790455807376
-0.522776788033	-0.790123921663
-0.522648682112	-0.78995213815
-0.522539213319	-0.789611802624
-0.522444473455	-0.789597880517
-0.522349369316	-0.789498186596
-0.522219048251	-0.789156057061
-0.522123175721	-0.78910695602
-0.522021301284	-0.788816752275
-0.521930497975	-0.788718905783
-0.521813345798	-0.788540895225
-0.521703969039	-0.788535833155
-0.52155078282	-0.788295991745
-0.521427100671	-0.788164784923
-0.521361304205	-0.788112644557
-0.521282361498	-0.788008913876
-0.521168304216	-0.787983760999
-0.521046662522	-0.787646705962
-0.520944434277	-0.787483911688
-0.520820036235	-0.787480389728
-0.520711102581	-0.787468075924
-0.520611449916	-0.787452482397
-0.520520279678	-0.787443154309
-0.520434975352	-0.78740479303
-0.520341559161	-0.787369693647

-0.520235212439	-0.787325040771
-0.520100413692	-0.787292551089
-0.519984321561	-0.787142069468
-0.51989493516	-0.787137974964
-0.519804071589	-0.787046973893
-0.519684523159	-0.786690483802
-0.519597887119	-0.786667372056
-0.519481882628	-0.786308110689
-0.519363981603	-0.786179537196
-0.51926839014	-0.785895462943
-0.51916230884	-0.785854286753
-0.519072068036	-0.785769792397
-0.518973288463	-0.785724719019
-0.518870673139	-0.78569006435
-0.518747503174	-0.785207673457
-0.518673369193	-0.785122686769
-0.518579792657	-0.785094819817
-0.518456078352	-0.784979479439
-0.518353761109	-0.78458632365
-0.518252057376	-0.784550605051
-0.518144798098	-0.784323823099
-0.518054946794	-0.784286017487
-0.517913086096	-0.784237333959
-0.517803451111	-0.784189298723
-0.517715931064	-0.784176917075
-0.517618853139	-0.784129755655
-0.517517741611	-0.783963013265
-0.517395914582	-0.783807061083
-0.517293205779	-0.783792836209
-0.517151891237	-0.783549204584
-0.51707778501	-0.783508911965
-0.516950523461	-0.783424901769
-0.516840057389	-0.783322706431
-0.516781930003	-0.783218430646
-0.516656787045	-0.783183085232
-0.516504985333	-0.783042714995
-0.516411919249	-0.782775939179
-0.51631811931	-0.782612490832
-0.516228318462	-0.782590379101
-0.51611409877	-0.782208602702
-0.515997729952	-0.782145447642
-0.515897501811	-0.782120180443
-0.515781640721	-0.781969519227
-0.51565685232	-0.781885257181
-0.515569458798	-0.781877097882
-0.515480630111	-0.78185396424
-0.515381803064	-0.781541736671
-0.515269507291	-0.781470377675
-0.515173204426	-0.78146132549
-0.515077633073	-0.781414641847
-0.51498998037	-0.781237594374
-0.514863534614	-0.780997469821
-0.514755419898	-0.780969312279
-0.514684501425	-0.780964715991

-0.514554965548	-0.780624422967
-0.514430398977	-0.780324880791
-0.514272564455	-0.78025973243
-0.514165723678	-0.780082650633
-0.514099430951	-0.780059228269
-0.513983297024	-0.779810967054
-0.513878665518	-0.779539202745
-0.513776371465	-0.779521446449
-0.513692564502	-0.779489835654
-0.513547270043	-0.778935628989
-0.51342094216	-0.77854345279
-0.513317226173	-0.778422981048
-0.513215799806	-0.778272153828
-0.513082555824	-0.777549821915
-0.512950008957	-0.777545149673
-0.512821626394	-0.777374726693
-0.512726620336	-0.777219327361
-0.51262806292	-0.777143920567
-0.512558191011	-0.777072661377
-0.512409009053	-0.776968432205
-0.512326476769	-0.776918806533
-0.512244548477	-0.776913240591
-0.512117871739	-0.776830929947
-0.512011415233	-0.776804735501
-0.511881383302	-0.776681966382
-0.511743616234	-0.776444529385
-0.511632046984	-0.77641885742
-0.511549372003	-0.776416686153
-0.511418709357	-0.776206042506
-0.511293026555	-0.776057146853
-0.511135113483	-0.775620504096
-0.511015958477	-0.775392311536
-0.510928958931	-0.775312765784
-0.510841248101	-0.775059453382
-0.510724551	-0.774898314005
-0.510635784876	-0.774869653257
-0.510546218225	-0.774743420532
-0.510418835967	-0.774531630138
-0.510280881117	-0.773754835835
-0.510163061787	-0.773107341333
-0.510031883603	-0.773089504362
-0.509912791771	-0.772855280632
-0.509783773587	-0.77274073089
-0.50968822794	-0.772702182215
-0.509567717632	-0.772627476036
-0.509452427575	-0.772422585698
-0.509327252643	-0.772286195777
-0.509195790767	-0.771413655056
-0.509114329397	-0.771345301486
-0.509027290346	-0.771310257273
-0.508910048254	-0.771300048913
-0.508798145597	-0.771201684085
-0.508668783323	-0.770470357914
-0.508540746433	-0.769880201648

-0.508436392338	-0.769774524101
-0.508343601425	-0.769727548194
-0.508261604076	-0.769713324085
-0.508155048266	-0.769673840858
-0.508067808447	-0.769565264785
-0.507934029623	-0.769379675514
-0.507860229243	-0.769206396369
-0.507745591409	-0.768997834737
-0.507590374442	-0.768205410703
-0.507454206355	-0.767902078631
-0.507347835589	-0.767888875113
-0.507222616415	-0.767679754129
-0.507126070155	-0.767333035522
-0.506993539687	-0.767295683817
-0.506903042594	-0.767289484923
-0.506766607245	-0.767025877111
-0.50665875889	-0.766780325469
-0.506558612678	-0.766624092627
-0.506455685983	-0.766333691445
-0.506330934869	-0.766147603854
-0.506193994278	-0.765929336247
-0.506080798892	-0.765836416388
-0.505992191283	-0.765827968321
-0.505900010496	-0.7657214745
-0.505813022705	-0.76556547262
-0.505677484602	-0.765476594035
-0.50556348763	-0.765310861303
-0.505421050404	-0.764668567556
-0.505332063306	-0.764459228867
-0.505215779919	-0.764227004139
-0.505107473286	-0.764038770155
-0.505023328131	-0.763864640415
-0.504934684699	-0.763785553478
-0.504817269015	-0.763557482418
-0.504679655341	-0.763323656084
-0.504568271739	-0.763301066301
-0.504477650657	-0.762996923801
-0.504376459052	-0.762894698002
-0.504295865882	-0.762776905317
-0.504214617198	-0.762735065166
-0.504043625082	-0.762702741369
-0.503939296473	-0.762101662789
-0.503862300819	-0.761995776015
-0.503720810108	-0.761680026289
-0.50357482119	-0.761668432173
-0.503469182429	-0.761601526871
-0.503352026897	-0.761277145934
-0.50323316035	-0.761052892231
-0.503140398023	-0.760873636547
-0.503005336329	-0.760767118067
-0.502919553457	-0.760538519293
-0.502813578011	-0.760308507178
-0.502714185893	-0.759976864974
-0.502603623201	-0.759614826272

-0.502483539637	-0.759374837896
-0.502371184191	-0.759239471102
-0.502267173699	-0.759159240772
-0.502142764759	-0.75877921529
-0.502024657661	-0.758687355411
-0.501880433583	-0.757912328224
-0.501784860695	-0.757752445246
-0.501669595986	-0.757304866409
-0.501560783011	-0.756925493329
-0.501449875441	-0.756916050594
-0.501339885838	-0.756851379445
-0.501194334025	-0.756650299036
-0.501086245847	-0.756623677972
-0.500967420586	-0.756521180524
-0.500813246257	-0.756225760521
-0.50066979323	-0.756152788224
-0.500568173465	-0.755851349814
-0.500469406025	-0.755831059639
-0.500379609408	-0.755790182594
-0.50025308519	-0.755777810158
-0.500141063058	-0.755645487873
-0.500032622882	-0.755411883375
-0.499935310545	-0.755347745112
-0.499835102726	-0.755192193409
-0.499706827085	-0.755174230442
-0.499597077453	-0.755128666309
-0.499484588001	-0.754773134964
-0.499405300849	-0.754719180315
-0.499280218738	-0.754648372713
-0.499186822551	-0.754364392103
-0.499085545868	-0.754337740478
-0.498969793751	-0.754331237561
-0.498857439253	-0.754100951989
-0.498748729178	-0.753799825846
-0.498638427676	-0.753731629039
-0.498542852392	-0.753586345558
-0.498435248112	-0.753564153409
-0.498298450723	-0.753487547156
-0.498199602675	-0.753353419764
-0.498129975992	-0.752904609954
-0.498028580031	-0.752880070147
-0.497904882995	-0.752359458482
-0.497792522307	-0.752338874281
-0.497699129991	-0.752327866679
-0.497592659434	-0.75219504553
-0.497497441883	-0.751972363206
-0.497354008202	-0.75186598198
-0.497261997892	-0.751802638734
-0.497152030855	-0.751772670998
-0.497003553592	-0.75161091907
-0.496898652186	-0.751602695097
-0.496782890671	-0.751196805596
-0.496673980816	-0.751058282256
-0.496548276412	-0.750665450229

-0.496484927331	-0.750637863126
-0.496407168882	-0.750262176407
-0.496322455705	-0.750257644121
-0.496195931283	-0.749895836637
-0.496136888782	-0.749866618929
-0.495995175024	-0.749372961563
-0.495889849353	-0.749261996238
-0.495809732742	-0.749211702147
-0.49570825815	-0.749196865363
-0.495612901252	-0.749010377555
-0.49551412223	-0.748927048894
-0.495407544181	-0.748792605945
-0.495309265919	-0.748667149392
-0.495213282977	-0.748528429135
-0.495112554892	-0.748512914718
-0.495045978897	-0.748373013774
-0.494950507574	-0.74832770636
-0.494803780028	-0.748317276708
-0.494678909658	-0.747788938293
-0.49454968321	-0.747492427188
-0.494416999835	-0.747070008853
-0.494279613723	-0.746962413066
-0.494166421656	-0.746942413426
-0.494043956697	-0.746671528602
-0.493957133189	-0.746655450996
-0.493854492947	-0.746461102098
-0.493755567513	-0.746280757573
-0.4936453673	-0.746173993533
-0.493533906882	-0.746153565724
-0.49339864666	-0.745786023825
-0.493292811537	-0.745681055634
-0.493176887124	-0.745643829239
-0.493092501516	-0.745551726758
-0.492955623758	-0.74532753003
-0.492837176383	-0.745135205046
-0.492743304441	-0.745129256745
-0.492647293414	-0.745071234298
-0.492540681069	-0.744805146986
-0.492421195107	-0.74443279649
-0.492306366483	-0.744180574334
-0.492190324185	-0.744123742958
-0.492061782774	-0.743682788839
-0.491955092377	-0.743333041044
-0.491836563394	-0.74322124663
-0.491763519997	-0.743213906573
-0.491680878351	-0.743111718676
-0.491578655738	-0.743077575536
-0.491469276862	-0.74299586473
-0.491360147213	-0.742994475116
-0.491236285076	-0.74295341122
-0.491147619698	-0.742910976983
-0.491018892904	-0.742903734355
-0.490919922403	-0.742854163628
-0.490766113025	-0.742785291124

-0.490644018178	-0.742590066459
-0.490533559775	-0.742527810235
-0.490428874178	-0.74252280224
-0.490339039339	-0.742504432185
-0.490206078444	-0.742437297508
-0.490097703046	-0.742326261167
-0.490008676814	-0.742311751867
-0.489900972993	-0.74192606866
-0.489787484775	-0.741866777939
-0.48969135541	-0.741829209496
-0.489581984576	-0.741764821453
-0.48951625808	-0.741732511421
-0.489389980487	-0.741340402415
-0.489256250775	-0.741101644899
-0.489111922747	-0.740906878035
-0.488994729348	-0.740672154195
-0.488902499781	-0.740650629281
-0.488824979942	-0.740598589926
-0.4887089771	-0.740570004986
-0.488596293525	-0.740550966431
-0.488491576153	-0.740550746384
-0.488362180114	-0.740296846025
-0.488257325279	-0.740109702218
-0.488170309628	-0.739970235183
-0.488057540079	-0.739942850429
-0.487929867224	-0.739833636143
-0.487818697678	-0.73959655414
-0.487707046826	-0.739524769001
-0.487589342604	-0.739365637288
-0.487431503582	-0.739193243859
-0.487316005025	-0.738620353779
-0.487201261352	-0.738600498334
-0.487082406469	-0.738531245031
-0.486989814542	-0.738318486769
-0.486848338105	-0.737745669854
-0.486701616185	-0.737422790679
-0.486597164995	-0.73715385664
-0.486457148576	-0.73647790589
-0.486350292083	-0.736155892657
-0.486216759678	-0.7360591246
-0.486130767706	-0.735905783959
-0.486031856613	-0.735535484869
-0.485932536676	-0.735280277362
-0.485810559559	-0.735128330453
-0.485702166162	-0.734979261292
-0.485586754374	-0.734852657814
-0.485470278577	-0.73472061397
-0.4853455168	-0.734566861792
-0.485197181224	-0.734148152777
-0.485100038673	-0.733886353212
-0.484985437695	-0.733726200597
-0.484857408038	-0.733498712313
-0.484759055817	-0.733146360479
-0.484676914503	-0.733141546555

-0.484508402127	-0.732552086873
-0.484393299451	-0.732211251673
-0.484304529047	-0.732192512169
-0.484191524324	-0.732124021081
-0.484087364961	-0.732092793732
-0.484008161007	-0.732040977378
-0.48392217024	-0.732028741854
-0.483858455643	-0.731976178326
-0.483714470166	-0.731741553393
-0.483618292403	-0.731470455573
-0.483519449685	-0.731398182057
-0.483416072481	-0.731154048522
-0.483354123367	-0.731147105247
-0.483255485677	-0.731049498051
-0.48312123	-0.731037737257
-0.482988046014	-0.730932487634
-0.48288192372	-0.730928204409
-0.482763494752	-0.730545483768
-0.482648048226	-0.730406271162
-0.482577717716	-0.730382258351
-0.482445456297	-0.730102268558
-0.482360652415	-0.729995661218
-0.48225687923	-0.729931379431
-0.48211001277	-0.729241488757
-0.481989559042	-0.728883442738
-0.481885733941	-0.728694289363
-0.481785581985	-0.728665573354
-0.481696559975	-0.728525093901
-0.481614497772	-0.728236244393
-0.481518094309	-0.728206419124
-0.481422212801	-0.728152369761
-0.481304660166	-0.727905197538
-0.481183050252	-0.727819970043
-0.481086931486	-0.727786474389
-0.48102898235	-0.727634890671
-0.480939836174	-0.727569953304
-0.480868113636	-0.727387230785
-0.480744401125	-0.727189452633
-0.480601827463	-0.72677847563
-0.480510377977	-0.726689037715
-0.480384000698	-0.726561589647
-0.480281238275	-0.726557294058
-0.480197596681	-0.726501220509
-0.48004822962	-0.726398138286
-0.479900263009	-0.726063913807
-0.479776303794	-0.725881758134
-0.479663569102	-0.72573095883
-0.47958134228	-0.725726283508
-0.4794863165	-0.725689919982
-0.47935236386	-0.7254882819
-0.479197408593	-0.725471305591
-0.479113005863	-0.725403330878
-0.479037545998	-0.725373757838
-0.478936095288	-0.725349116732

-0.478810200681	-0.724835158431
-0.478739595459	-0.724747480816
-0.478664841144	-0.72464589683
-0.4785276625	-0.72439056168
-0.478398213955	-0.724117522096
-0.478286233896	-0.723955274025
-0.478140929358	-0.723840941758
-0.478062378113	-0.72371844525
-0.477978054478	-0.723676749151
-0.477896002414	-0.723524832429
-0.477769180097	-0.723500451654
-0.477653008353	-0.723390088734
-0.477587284839	-0.723386234374
-0.477460032253	-0.723375341262
-0.477351866317	-0.723188846248
-0.477285637955	-0.723185267976
-0.47718086242	-0.723002399592
-0.477062545267	-0.722784547574
-0.476951656979	-0.722713148413
-0.476781897362	-0.722243981107
-0.476673895043	-0.722054547916
-0.476542986355	-0.721983346992
-0.476430568193	-0.721953527847
-0.476357688627	-0.721764479719
-0.476296565735	-0.721675998369
-0.476160622792	-0.721577985851
-0.476038170542	-0.721377350904
-0.47595010769	-0.721351615648
-0.475876563251	-0.721199240113
-0.475757643566	-0.72105262526
-0.475648964884	-0.720961185923
-0.475544460271	-0.720843085878
-0.475476891262	-0.720782717398
-0.475343449727	-0.720627033124
-0.475257638522	-0.720513892194
-0.475162683477	-0.720241563591
-0.4750551177	-0.720147108356
-0.474936171866	-0.71990509153
-0.474840617777	-0.719544292212
-0.474671233326	-0.719488072082
-0.474580068662	-0.71938645218
-0.474480586402	-0.719382478654
-0.474405713359	-0.719246898104
-0.474318064364	-0.719229518256
-0.474213609361	-0.719103645442
-0.474116856481	-0.719100353539
-0.474017306929	-0.719096563205
-0.473903139954	-0.718898897406
-0.473760654115	-0.718223985119
-0.473630329897	-0.718222226571
-0.473512671389	-0.717956735101
-0.473437464117	-0.717949589382
-0.473337114684	-0.717779155784
-0.473233415104	-0.717682388066

-0.473133027279	-0.717192528629
-0.473037843241	-0.717178127848
-0.472890937234	-0.71710369909
-0.47279789204	-0.717058990466
-0.472700571445	-0.717030878952
-0.472599402133	-0.716723273223
-0.472500333779	-0.716652919945
-0.472362494731	-0.716213654047
-0.47224244986	-0.715927414727
-0.472144347039	-0.715792879454
-0.472012503472	-0.715781503495
-0.471897525274	-0.715601177409
-0.471799604542	-0.715582889066
-0.47171825229	-0.71551488191
-0.471625024185	-0.71547432312
-0.471552248313	-0.715432596633
-0.471419055491	-0.715163951873
-0.471315899231	-0.715141969478
-0.471192746959	-0.714908502521
-0.471076698966	-0.714901222838
-0.470924657753	-0.714796835444
-0.47082303283	-0.71452894842
-0.470721152089	-0.714198141755
-0.470641844386	-0.71417359548
-0.470573083203	-0.714147386618
-0.470471006925	-0.713956249895
-0.470322072955	-0.71377372499
-0.470165930606	-0.713770823139
-0.470074802111	-0.713743995727
-0.470000108352	-0.713573735641
-0.469910780558	-0.713506049873
-0.469818438459	-0.713360192846
-0.469729482027	-0.713283882914
-0.46964201552	-0.713280020557
-0.469525716267	-0.713078632492
-0.469428284218	-0.713054756215
-0.469305944156	-0.713041621406
-0.469199642457	-0.712402468184
-0.469078149808	-0.712166435761
-0.468995962126	-0.712132959873
-0.468891351319	-0.712079764007
-0.468791335042	-0.711926309046
-0.468674687652	-0.711909951977
-0.468521402135	-0.711619420346
-0.468417093711	-0.711370243024
-0.46829023116	-0.711312951851
-0.4681784712	-0.711125618251
-0.468076849253	-0.710907046379
-0.46795090129	-0.710697005192
-0.467861721833	-0.710515812406
-0.467745755393	-0.710084357016
-0.467642473433	-0.709934035037
-0.46754918434	-0.709912354809
-0.467455520782	-0.709775111754

-0.467373225663	-0.709726367884
-0.467257389901	-0.709651667603
-0.46715215261	-0.709113006558
-0.466981978746	-0.708860322375
-0.466873628789	-0.708729192644
-0.466714066281	-0.708500190844
-0.466596882944	-0.708406654722
-0.466513311375	-0.70839056734
-0.466379722036	-0.708316504498
-0.466259395633	-0.70803344708
-0.466171959263	-0.707931565326
-0.466066819529	-0.707781889095
-0.465972114767	-0.707769221066
-0.465884078632	-0.707723252047
-0.465768964632	-0.707360735248
-0.465649767028	-0.707328176188
-0.465555381071	-0.707209054524
-0.465465223546	-0.70714059602
-0.465340217823	-0.707023140069
-0.465250286479	-0.706845913517
-0.465142805288	-0.706688873422
-0.465056710718	-0.706509422847
-0.4649701095	-0.706449122261
-0.464825917523	-0.706230192167
-0.46470458986	-0.706190357783
-0.464611496207	-0.706066707036
-0.464505343674	-0.706037851479
-0.464414432625	-0.70590743566
-0.46430580771	-0.705825357861
-0.464232819033	-0.705774299564
-0.464138496046	-0.705695469948
-0.463978401641	-0.705473147497
-0.46382475307	-0.704823994349
-0.463738274357	-0.70480522075
-0.463651998749	-0.704783763486
-0.463514909	-0.704064209738
-0.463414522931	-0.704050185806
-0.463289929825	-0.703890827459
-0.463184364582	-0.703745842814
-0.463084239706	-0.703591305187
-0.462992800504	-0.703588607732
-0.462848305986	-0.703439759278
-0.462749699722	-0.703416525277
-0.46267617241	-0.703098618448
-0.462570014895	-0.70277443389
-0.462397221968	-0.702763598357
-0.462286490456	-0.702477855456
-0.462156005367	-0.702072101413
-0.462069367466	-0.702006362008
-0.461952228656	-0.70199193708
-0.461864487436	-0.70193176371
-0.461787640627	-0.701895927728
-0.46160513449	-0.701733572241
-0.461490042022	-0.701646526086

-0.461406714068	-0.701631118096
-0.461281723133	-0.701493939073
-0.4611657203	-0.701234964904
-0.461048421044	-0.701094694841
-0.460911990431	-0.701055665939
-0.460797306604	-0.701001219221
-0.46071113074	-0.70086165743
-0.46061808172	-0.700839330441
-0.460501098215	-0.700834965831
-0.460373484116	-0.700814063228
-0.460247874023	-0.70075653449
-0.460149215905	-0.700690634355
-0.460056953917	-0.700463778094
-0.459941812448	-0.700141151527
-0.459852542011	-0.700088126802
-0.459735338115	-0.700023428745
-0.459608723781	-0.699778189106
-0.459484269481	-0.699566530045
-0.459348965542	-0.699566116732
-0.459239924854	-0.6992745111
-0.459132035141	-0.69901223277
-0.459025047494	-0.699005865295
-0.458887676516	-0.698807125583
-0.458768656945	-0.697913508672
-0.458669303087	-0.697722810619
-0.458590921206	-0.697683934236
-0.458505364763	-0.697634334379
-0.458372779786	-0.697564081915
-0.458275100061	-0.6974427657
-0.458085899442	-0.696098472768
-0.457983719146	-0.695727617129
-0.457901728942	-0.695701022732
-0.457780995037	-0.695482857142
-0.457693348832	-0.695282470484
-0.457603083585	-0.695225064751
-0.457491834586	-0.695221885614
-0.45735768857	-0.695139374123
-0.457276789104	-0.694923558987
-0.45715556834	-0.694557458969
-0.45708055031	-0.694348317107
-0.456950607624	-0.694243713103
-0.456824717211	-0.693816465844
-0.456730978914	-0.693696611489
-0.456654357741	-0.693689395852
-0.456571643472	-0.693601900667
-0.45644929527	-0.69338938003
-0.456329859174	-0.693282423017
-0.456227748959	-0.693143369926
-0.456121494768	-0.693051240807
-0.456007170497	-0.693042400451
-0.455905721937	-0.692951577622
-0.455845076168	-0.692876056881
-0.455765665247	-0.692826012115
-0.455678134966	-0.692806205074

-0.455546172974	-0.692507518411
-0.455458137469	-0.692351598778
-0.455374476979	-0.692161574635
-0.455263886638	-0.692071843315
-0.455128886792	-0.692038161096
-0.454992639442	-0.691628248488
-0.454865989553	-0.691621838895
-0.454759962272	-0.691391957279
-0.454700087391	-0.691248190372
-0.454596546315	-0.690925176614
-0.454509486598	-0.690622815654
-0.454387898433	-0.690525807671
-0.45425375353	-0.690513721377
-0.454107604538	-0.690377343648
-0.454021955303	-0.6903377881
-0.453908268747	-0.690247488345
-0.453808262923	-0.690226670624
-0.453671414517	-0.690226009308
-0.453553835304	-0.689995171893
-0.453418849141	-0.689111227764
-0.453347726405	-0.688972065483
-0.453209263093	-0.688669235485
-0.453137720779	-0.688614024559
-0.453011337948	-0.688593684029
-0.452911579123	-0.68851042203
-0.452840495524	-0.688472536286
-0.452749764569	-0.688462933007
-0.452618027644	-0.688315071632
-0.452507680648	-0.688205137142
-0.452382275749	-0.688059985369
-0.452315353746	-0.687803594876
-0.452177360211	-0.68776541777
-0.452102068858	-0.687760365187
-0.452007907561	-0.687697443639
-0.451924193084	-0.687638928349
-0.451837787313	-0.687598702773
-0.451761713284	-0.687596136403
-0.451614574834	-0.687335088567
-0.451482281825	-0.686989190474
-0.451363204917	-0.686969613157
-0.451231713829	-0.686544632835
-0.451131583949	-0.686375877171
-0.450997427899	-0.686341221469
-0.450893565455	-0.686305051262
-0.450809333837	-0.686223961416
-0.450661364672	-0.685825030089
-0.450570928621	-0.685697161768
-0.45042214323	-0.68521348875
-0.450278280593	-0.684852151367
-0.450180636221	-0.684849880238
-0.450011536835	-0.684207405904
-0.449918611021	-0.684206979346
-0.449788753605	-0.683890411443
-0.449693471918	-0.683824150874

-0.449545301154	-0.68349767851
-0.449433663541	-0.683222531017
-0.449358180976	-0.683123980519
-0.449223185694	-0.683074010769
-0.449112186757	-0.682897327978
-0.4489840266	-0.682739030454
-0.448861653913	-0.682136565399
-0.448742501828	-0.68207829795
-0.448651069374	-0.682011023243
-0.448542685625	-0.681888388281
-0.448447034293	-0.681791265967
-0.448353172028	-0.68177938911
-0.44824185462	-0.681765518236
-0.448147391855	-0.681688759895
-0.448038007194	-0.681590164435
-0.447882280249	-0.681451639062
-0.447799043139	-0.681379099149
-0.447705339539	-0.681323021803
-0.447601801843	-0.681314711172
-0.447510507739	-0.681312560067
-0.44739483028	-0.681292323227
-0.447251584883	-0.681244335115
-0.447111927211	-0.681141531206
-0.446966134972	-0.68109514741
-0.446862865077	-0.680997261972
-0.446745022098	-0.680643121212
-0.446664357686	-0.680577217728
-0.446590319013	-0.68055285959
-0.446499586148	-0.680467572574
-0.446393976169	-0.68034823754
-0.446333106966	-0.680276404617
-0.446178613626	-0.679772033454
-0.446097132156	-0.679617407489
-0.445988689382	-0.679458273699
-0.445843968086	-0.679309993579
-0.445739557768	-0.679271288271
-0.445629689149	-0.679032451701
-0.445568645083	-0.679021132571
-0.44544874623	-0.678531464878
-0.445328635444	-0.678293821121
-0.445182131553	-0.678213584733
-0.445036700058	-0.678017289175
-0.444925016664	-0.67783722147
-0.444783166264	-0.677738695281
-0.444675925877	-0.677568903041
-0.444535581135	-0.677394080536
-0.444378102238	-0.676550055571
-0.444274008318	-0.676541838024
-0.444182091612	-0.676361988411
-0.44410181495	-0.676357158405
-0.443970563173	-0.676111467118
-0.443849235959	-0.67604624779
-0.44371023237	-0.675784821052
-0.443603366722	-0.67576098092

-0.443525421833	-0.675755051144
-0.443398163591	-0.675385531696
-0.443264647908	-0.675317394742
-0.443172022298	-0.675212402353
-0.44307177349	-0.675154069611
-0.442951919166	-0.674913378299
-0.44284707657	-0.674899694461
-0.442730527937	-0.674707711601
-0.442627836252	-0.674633206435
-0.44246818854	-0.674573591999
-0.442363984778	-0.67451891117
-0.44225363434	-0.674498335475
-0.442148207249	-0.674359944206
-0.442034594713	-0.674277751009
-0.441898878594	-0.674009415624
-0.441809507859	-0.673934456607
-0.441712612413	-0.673884546664
-0.441627533995	-0.673879230377
-0.441541596791	-0.673843867991
-0.441442629864	-0.673825696515
-0.441342175574	-0.673592882281
-0.441258504051	-0.673558035964
-0.441133537027	-0.673552477193
-0.441038807708	-0.673399572431
-0.440968825998	-0.673284477575
-0.440867960423	-0.673172018993
-0.440779601792	-0.672971952249
-0.440668656631	-0.672487104459
-0.440576574444	-0.672466520017
-0.440510957082	-0.672444474955
-0.440398564997	-0.672335148197
-0.440303808067	-0.672140019475
-0.440198684151	-0.672138801371
-0.440083335881	-0.6718567247
-0.439960648808	-0.671808650083
-0.439898216862	-0.671676462316
-0.439784927754	-0.671637936813
-0.439628594966	-0.671169367442
-0.439501553099	-0.671160950793
-0.439396320249	-0.671019013448
-0.439287036683	-0.670850560529
-0.439132764126	-0.670236502781
-0.439009356683	-0.670030717273
-0.438885655993	-0.66980024871
-0.438747395917	-0.669794342406
-0.438672086647	-0.669700950849
-0.438562599249	-0.669549355295
-0.438471171039	-0.669422295492
-0.438368354035	-0.669392340119
-0.438289634382	-0.66924556202
-0.438212856679	-0.669076813168
-0.438105339361	-0.668778176235
-0.437976023579	-0.668580164439
-0.437852842398	-0.66848285564

-0.437756791973	-0.668136690461
-0.437624179166	-0.667984047183
-0.437505743627	-0.667876431376
-0.437380569841	-0.667863926234
-0.437306795917	-0.667760259679
-0.437216521918	-0.667638255358
-0.43715508221	-0.667620517377
-0.437043712622	-0.667264085356
-0.436940986031	-0.66719742066
-0.436853343114	-0.667056441588
-0.436755160291	-0.6668579115
-0.436663606335	-0.666524318389
-0.43654845263	-0.66644237411
-0.436424433715	-0.666266469524
-0.436270724584	-0.665716021704
-0.436121579106	-0.665518897997
-0.436023108701	-0.665336724117
-0.435902023642	-0.665321055879
-0.43577311551	-0.665250087598
-0.435686037509	-0.665236603628
-0.43557943791	-0.665033531327
-0.435466687016	-0.664936695636
-0.435374079758	-0.664912466215
-0.435261074934	-0.664332989919
-0.435179304627	-0.66429998771
-0.435064030697	-0.664014350075
-0.434951727439	-0.663939377763
-0.434860035119	-0.663832823164
-0.434763381493	-0.663733520347
-0.434668471356	-0.663519775106
-0.434595135482	-0.663494265389
-0.434473261195	-0.663250014892
-0.43436133436	-0.663137552215
-0.434257381086	-0.663051468487
-0.434139254508	-0.663033460373
-0.434073903256	-0.662998776241
-0.433957347675	-0.662837094273
-0.433802129819	-0.662582866698
-0.433685040638	-0.662352347996
-0.433561537652	-0.662126009595
-0.433441845667	-0.661437860396
-0.433334227112	-0.661403313445
-0.433244453132	-0.661031199626
-0.433155928709	-0.661007889172
-0.433052823589	-0.660892449077
-0.432953108319	-0.660528154173
-0.432859129754	-0.66041768627
-0.432753631098	-0.660263290773
-0.432656359179	-0.660111429223
-0.432547685863	-0.6601059183
-0.432446520969	-0.66003486212
-0.432294013852	-0.659655385503
-0.432199638325	-0.65940994752
-0.432091118654	-0.659354885246

-0.43197369171	-0.659142361971
-0.431813893953	-0.659031601035
-0.431692305903	-0.658954807666
-0.431582031214	-0.658918669618
-0.431489387836	-0.658693431715
-0.431392924842	-0.658664843431
-0.431306635355	-0.65851162462
-0.431240429976	-0.658432895111
-0.431109599787	-0.658343371794
-0.431008161767	-0.658199915558
-0.430915407379	-0.658061183717
-0.430832654934	-0.65803157176
-0.430702592565	-0.657524580462
-0.430561976939	-0.657469258676
-0.430480861204	-0.657325123863
-0.430395577032	-0.657292584715
-0.430216590949	-0.657238445568
-0.430120464493	-0.657190114407
-0.430033901802	-0.657076379091
-0.429965188269	-0.656993420473
-0.429813285092	-0.656910193659
-0.429737645187	-0.656889304274
-0.429641259599	-0.656641809227
-0.42952230432	-0.656414842909
-0.429399238266	-0.656010123273
-0.42928862979	-0.655890197673
-0.429153623488	-0.655870328791
-0.429076271606	-0.655831196502
-0.428964582036	-0.655632615301
-0.428832222949	-0.655611139565
-0.428720426714	-0.655508006405
-0.428592615153	-0.655170774888
-0.428494281668	-0.655109326949
-0.428387959041	-0.655038590772
-0.4282685799	-0.654890279953
-0.428171249408	-0.654802253893
-0.428055636639	-0.654552968382
-0.427951987854	-0.654321736398
-0.42787125164	-0.65411408303
-0.427761945893	-0.653948262235
-0.427668411848	-0.653787571084
-0.427523672253	-0.653184180899
-0.427408454645	-0.653101731048
-0.427286003035	-0.652970454173
-0.427165430951	-0.652683807714
-0.427065687733	-0.652652753256
-0.42699984003	-0.652560123669
-0.426884597819	-0.651937994782
-0.426774849719	-0.651812523776
-0.426653152906	-0.65168133382
-0.426534729753	-0.651558590714
-0.426460790892	-0.651475353689
-0.4263751723	-0.651371669161
-0.426270125548	-0.651334717615

-0.426149250336	-0.651307690639
-0.426064231179	-0.651292124052
-0.425939451357	-0.651021480066
-0.425811028747	-0.650979223169
-0.42569010316	-0.650323526948
-0.425595018455	-0.650307362486
-0.425502131928	-0.65027613888
-0.425412940109	-0.65004871516
-0.42529723884	-0.65002320069
-0.425196930007	-0.649777887445
-0.425090424267	-0.649699333066
-0.425010372244	-0.649659349565
-0.42490805918	-0.649597455975
-0.424776424752	-0.64952723838
-0.424673017888	-0.649512619323
-0.424537435058	-0.64939399207
-0.424435552865	-0.649316497323
-0.424321599488	-0.649182464585
-0.424186944208	-0.649080531398
-0.424077729831	-0.648704109581
-0.423991155897	-0.648473898948
-0.423885976957	-0.648335662914
-0.423740829439	-0.648280399542
-0.423620057686	-0.648229772847
-0.423547550317	-0.6481108846
-0.423429161524	-0.647708957346
-0.423321299679	-0.647509457871
-0.423225676654	-0.647507569659
-0.423093471728	-0.647420769643
-0.423013461209	-0.647365298315
-0.42293102448	-0.647338204308
-0.422819161444	-0.646858947922
-0.422701949339	-0.646757036915
-0.422608576959	-0.6466257402
-0.422532753124	-0.646519811197
-0.422455573875	-0.646490758757
-0.422342173157	-0.64625764792
-0.422208569011	-0.646081605861
-0.422096372592	-0.645544467994
-0.421981513577	-0.645437847297
-0.421842215731	-0.645100399201
-0.421739581854	-0.645053758062
-0.421600216569	-0.644945837736
-0.421473412826	-0.644551493401
-0.42137718064	-0.644532169052
-0.421239691382	-0.644341835347
-0.421153615422	-0.64416641912
-0.421025672012	-0.64391258316
-0.420946004598	-0.64379741522
-0.42081067651	-0.643590461832
-0.420729216866	-0.643469913828
-0.420634627242	-0.643370714684
-0.420552135063	-0.643353163364
-0.420459409827	-0.643291192701

-0.420328205609	-0.6430953669
-0.420244833369	-0.642921880503
-0.420116418249	-0.642866383859
-0.420021244826	-0.642779978246
-0.419925830447	-0.642593844934
-0.419829358251	-0.64252479895
-0.419698276562	-0.642253650209
-0.419584450036	-0.642215487742
-0.419483527014	-0.642107873888
-0.419347382548	-0.642033678313
-0.419260988059	-0.641889613304
-0.41917681758	-0.641748802852
-0.419062841399	-0.641692558065
-0.418916434558	-0.641442243231
-0.418836119266	-0.641379314087
-0.418710569923	-0.641170868821
-0.418612255265	-0.640909307011
-0.418483764537	-0.640820448115
-0.418397141153	-0.64072142271
-0.418315047687	-0.640693194875
-0.418213020827	-0.640593467168
-0.418122200112	-0.640535513703
-0.418023241713	-0.640114523994
-0.417926034852	-0.640099099547
-0.417857209624	-0.640048465394
-0.417797456938	-0.63999604062
-0.417675474906	-0.639760694553
-0.417555402379	-0.639306083982
-0.417460406382	-0.639303506205
-0.417390489114	-0.639239183869
-0.417264410302	-0.639222221758
-0.417155111967	-0.639076175273
-0.417059963114	-0.639057037711
-0.416953023845	-0.639009622929
-0.416867227824	-0.638757449937
-0.416786051815	-0.638686908762
-0.416714579515	-0.638594923817
-0.416635111251	-0.638558372564
-0.416536694281	-0.638481658575
-0.416406147136	-0.638183911676
-0.416271886769	-0.638148025266
-0.41612101032	-0.637947548478
-0.416034995056	-0.637901379721
-0.41593733323	-0.637874250329
-0.41583010611	-0.637517380218
-0.41568871052	-0.636933810473
-0.415597705057	-0.636729937174
-0.415479598072	-0.636601410589
-0.41539178509	-0.636560537887
-0.415306540515	-0.636313723534
-0.415208661375	-0.636217655103
-0.415121433311	-0.636120124341
-0.415031702109	-0.635994672052
-0.414922010165	-0.63591877615

-0.414846714825	-0.635836127027
-0.414739304037	-0.635660035315
-0.414646941516	-0.63555420213
-0.414512088085	-0.635454069731
-0.414401097291	-0.635257018167
-0.414315109575	-0.635161941825
-0.414223547002	-0.635012676957
-0.414093771995	-0.634566082714
-0.413981668207	-0.634535126302
-0.413879233039	-0.634441724443
-0.41377534583	-0.63435359256
-0.413644178622	-0.634224576791
-0.413543468987	-0.634161807837
-0.413418151665	-0.634134276641
-0.413302897687	-0.633924374656
-0.413178726123	-0.633860194014
-0.413106223692	-0.633800149332
-0.413003257341	-0.633525795585
-0.412904777353	-0.633297608699
-0.41283199875	-0.633216159396
-0.412724006438	-0.633091769998
-0.412614336164	-0.632960048037
-0.412526493533	-0.63281422026
-0.412410034373	-0.632795696334
-0.412317147428	-0.632702558455
-0.41222977177	-0.632634807549
-0.412123308217	-0.63258832673
-0.41201734616	-0.632516288961
-0.411892402258	-0.63191408573
-0.411798999057	-0.631828127039
-0.4116821771	-0.631648033964
-0.411565903167	-0.63159891692
-0.41147278955	-0.631441565413
-0.411349174313	-0.631320922264
-0.41125085618	-0.631219241238
-0.411141975403	-0.631105462447
-0.411024950239	-0.630993652942
-0.410922977982	-0.6308270035
-0.410823525394	-0.63081424747
-0.410706585779	-0.630766984828
-0.410603340647	-0.630624772879
-0.410484212521	-0.630393645855
-0.41039353169	-0.630377696596
-0.410294331428	-0.630348574236
-0.410173523421	-0.630250112457
-0.410086842437	-0.630241282446
-0.410003506514	-0.630201497581
-0.409908617859	-0.630123320116
-0.409848268988	-0.630065281735
-0.409750809599	-0.629950992319
-0.409633418747	-0.629804590638
-0.409541213325	-0.629776882462
-0.409438335563	-0.629746182823
-0.409354666328	-0.62967761943

-0.409243307509	-0.629583694415
-0.40913540537	-0.62954271765
-0.409030147647	-0.629489955702
-0.408938769214	-0.629279756037
-0.408833919343	-0.629215652553
-0.408740980855	-0.62915069907
-0.408630864595	-0.629098725096
-0.408534114818	-0.629075980321
-0.408470215418	-0.628902099756
-0.408359294955	-0.628679648045
-0.408217510598	-0.628378093576
-0.408153354999	-0.628370642664
-0.408033264079	-0.62831500996
-0.407932650634	-0.628125533408
-0.407802343807	-0.628092070098
-0.407700323173	-0.62796243794
-0.407579582078	-0.627875788656
-0.407457420882	-0.627123266115
-0.407336578299	-0.627120595153
-0.407224874952	-0.626710994188
-0.407130024869	-0.626635099993
-0.407043785079	-0.626574831385
-0.406920074732	-0.626101437267
-0.406841100405	-0.626068237187
-0.406720941534	-0.626035962979
-0.40655684337	-0.626012424485
-0.406441599458	-0.625975208673
-0.406381145453	-0.625968557285
-0.406271830523	-0.625905359683
-0.406197152356	-0.625767514449
-0.406099958296	-0.625696245552
-0.405976660744	-0.625560065373
-0.405901278489	-0.62552639843
-0.405785865594	-0.625348160587
-0.405682254657	-0.625302803301
-0.405595980192	-0.625281758116
-0.405507904768	-0.625263495443
-0.405380974592	-0.625117915896
-0.405291335966	-0.625020255828
-0.405193925889	-0.624922643151
-0.405124456242	-0.624920744118
-0.405007496275	-0.624868327792
-0.404920030058	-0.624804215114
-0.404798980148	-0.624603443366
-0.404676034503	-0.624556200042
-0.404563968753	-0.624072631819
-0.404447349871	-0.624006663867
-0.404373897139	-0.623986610591
-0.404279671468	-0.623932869033
-0.404180788988	-0.623824475272
-0.404056532948	-0.623691885723
-0.403972300155	-0.623623315439
-0.403859811161	-0.623141243271
-0.403739619564	-0.623005213033

-0.40360481943	-0.622788672071
-0.403520837129	-0.622786843286
-0.403450605776	-0.622769413966
-0.403326363656	-0.622645526021
-0.403239310868	-0.622492844423
-0.403140519701	-0.622464454006
-0.403010377586	-0.622449843383
-0.402931137762	-0.622336492457
-0.402843445722	-0.622328003562
-0.402722578634	-0.622125620161
-0.402626072983	-0.622074447619
-0.402530415366	-0.621991346367
-0.40243072936	-0.621877748237
-0.402312261922	-0.621670968858
-0.402213142638	-0.62146202653
-0.402079456877	-0.621451686488
-0.401966378245	-0.621375301712
-0.401854002537	-0.620973407721
-0.401767660047	-0.620958598779
-0.401652426001	-0.620932509293
-0.4015552932	-0.620899968298
-0.401430710064	-0.620619295162
-0.401323811174	-0.620602685691
-0.401200906664	-0.620375278366
-0.401118370208	-0.620356378728
-0.40100000046	-0.620095142734
-0.400913863304	-0.620085351337
-0.400809918132	-0.619896871045
-0.40071588249	-0.619848238971
-0.400611517693	-0.619748217579
-0.400536970461	-0.619734607738
-0.400420966678	-0.619416680479
-0.400302872473	-0.619366031615
-0.400228315618	-0.619151188108
-0.400134177731	-0.619096755322
-0.400049509127	-0.618754302945
-0.39995260487	-0.618675867321
-0.399831789836	-0.618355245919
-0.399687402036	-0.617782190288
-0.399569457904	-0.617679974538
-0.399462106124	-0.617614014001
-0.399331627797	-0.61752167962
-0.399238265307	-0.617473741184
-0.39912998586	-0.617349087412
-0.399044268391	-0.617187928365
-0.39895410175	-0.617185012728
-0.39883571217	-0.617136509377
-0.398720473649	-0.616999050484
-0.398619087668	-0.6169018746
-0.398489425787	-0.616854712491
-0.398403254657	-0.616734620073
-0.398261023012	-0.615848747704
-0.398164877258	-0.615819868376
-0.398055431335	-0.615756679457

-0.397971073596	-0.615700763477
-0.39787042887	-0.615628344227
-0.397787586876	-0.615620980524
-0.397694878103	-0.615546068482
-0.397602622286	-0.615545269143
-0.397514065364	-0.615405873953
-0.39742008763	-0.615136839618
-0.397310592216	-0.615072923483
-0.397186930372	-0.614426087066
-0.397110997586	-0.614406890905
-0.396995915589	-0.614357019054
-0.396910612092	-0.613996636925
-0.396802374353	-0.613704645704
-0.396710522931	-0.613531056082
-0.396633614234	-0.613431769061
-0.396530564455	-0.613072378518
-0.396406992988	-0.613001801923
-0.396306358008	-0.612941962625
-0.396214353899	-0.612865095062
-0.396116807191	-0.612726304301
-0.395981946677	-0.612342315337
-0.395879292448	-0.611844823554
-0.39579161556	-0.611841099064
-0.395662016729	-0.611493222894
-0.395576159991	-0.61148828096
-0.395447011507	-0.61102612687
-0.39534137316	-0.610331276785
-0.395253024735	-0.610052867742
-0.395131210538	-0.609911524448
-0.395034852039	-0.60954184743
-0.394949056228	-0.609508914358
-0.394862598278	-0.609344617066
-0.394748180635	-0.609179216198
-0.394653415905	-0.608943998879
-0.394557130218	-0.608605188049
-0.394462192365	-0.608511469051
-0.394335113664	-0.608489487194
-0.394256955886	-0.608460425985
-0.394160802871	-0.608434991135
-0.394052917651	-0.608076939486
-0.393981740159	-0.607823192431
-0.393896704515	-0.607765801762
-0.39381001436	-0.607676998057
-0.393672054312	-0.607565449355
-0.393566387512	-0.607524284639
-0.393441886161	-0.607451334237
-0.393386962972	-0.607448053701
-0.393290488945	-0.607418498651
-0.393189012939	-0.607389377426
-0.393069138099	-0.606902405042
-0.392988798987	-0.606803481566
-0.392866191269	-0.606233519922
-0.392780372878	-0.606155247843
-0.392677456829	-0.606147931301

-0.392577822276	-0.605895030556
-0.392426862254	-0.605653925291
-0.392351730363	-0.605650056581
-0.392230228886	-0.605336465716
-0.392125098992	-0.604926675608
-0.391997041699	-0.604923119591
-0.391909121715	-0.604910672899
-0.391784446404	-0.604626101358
-0.391668720997	-0.604609546801
-0.391581025489	-0.604512397058
-0.391489365968	-0.604309299174
-0.391352066766	-0.604218055226
-0.391247799508	-0.60413895462
-0.391150041414	-0.604080379432
-0.391069340836	-0.603917767395
-0.390918810311	-0.603330541181
-0.390812618106	-0.603326365596
-0.390723237742	-0.603134407885
-0.390629502571	-0.602816134113
-0.390510061971	-0.602649324858
-0.390380730718	-0.60263254842
-0.39028520121	-0.602624735108
-0.390176880364	-0.602252060675
-0.390092592661	-0.602066989612
-0.389977233647	-0.60181921226
-0.389850354819	-0.601770218522
-0.389749307046	-0.601602692777
-0.389612894949	-0.601192973854
-0.389552510374	-0.601041918362
-0.389496421224	-0.600893251692
-0.389419286129	-0.60086023146
-0.389343638306	-0.600805563872
-0.389276857906	-0.600804007555
-0.389183551457	-0.600798121556
-0.389057772211	-0.600689516023
-0.388966275853	-0.6006518308
-0.388844821373	-0.600424833695
-0.388757836438	-0.600329193737
-0.388633617691	-0.600245791083
-0.388546261252	-0.600186546767
-0.388464799181	-0.600170160623
-0.38834319462	-0.600112224638
-0.388238642277	-0.600038004302
-0.38814018988	-0.599858136506
-0.388036353069	-0.5997344498
-0.387957960307	-0.59970878452
-0.387866036161	-0.599688294509
-0.387789773456	-0.599686611918
-0.38768138114	-0.599565436024
-0.387588059163	-0.599477100411
-0.387498409913	-0.599471500982
-0.387426267829	-0.599230716256
-0.387326300237	-0.599001707607
-0.387227203917	-0.598550670003

-0.387132072589	-0.598466427089
-0.387041216736	-0.59813971969
-0.386969632344	-0.59810715688
-0.386863205757	-0.598040880212
-0.386793634065	-0.597986849753
-0.386717453951	-0.597963518774
-0.386610328566	-0.597912598758
-0.386500980042	-0.597758789843
-0.386422825117	-0.59766600612
-0.386314024776	-0.597628634112
-0.386237097307	-0.59760142341
-0.386133674557	-0.597543068421
-0.386035011154	-0.597451877609
-0.385951449286	-0.597450269612
-0.385876348577	-0.597371061623
-0.385799045182	-0.597354152658
-0.385733233916	-0.597266451363
-0.385609056699	-0.597029841385
-0.385533025641	-0.596974050912
-0.385424095208	-0.596888843655
-0.385340214707	-0.596880210566
-0.38526591742	-0.596845831126
-0.385128149888	-0.596820852547
-0.385048898904	-0.596745988884
-0.384926500012	-0.596545558835
-0.384794134112	-0.596507142996
-0.384699940952	-0.596152777315
-0.384596328216	-0.596052629029
-0.384470073248	-0.595569684449
-0.384374060317	-0.595539833407
-0.384251293441	-0.595310521757
-0.384152340073	-0.595172924956
-0.384049693522	-0.595090497247
-0.383974122878	-0.59497487906
-0.383872104455	-0.594933816862
-0.383765837143	-0.594853804879
-0.383673557707	-0.594800471271
-0.383598063225	-0.594798504271
-0.383487642688	-0.594687473438
-0.383417967445	-0.594625325949
-0.383321867495	-0.594599027467
-0.383234237302	-0.594501289185
-0.383106592292	-0.594423603169
-0.3829930524	-0.594323503064
-0.382914092279	-0.594312832538
-0.382836565152	-0.594225751139
-0.382725049225	-0.594197294281
-0.382631054006	-0.594159792306
-0.382559859091	-0.594041049005
-0.382487405797	-0.593838638986
-0.382364422453	-0.593682318278
-0.382298773775	-0.593668671291
-0.382186097387	-0.593334886396
-0.382084533837	-0.593138288705

-0.381968125329	-0.59288570423
-0.38184377623	-0.592332129159
-0.381757310498	-0.592126452895
-0.381606103216	-0.591731724314
-0.381518478532	-0.591612754645
-0.381434449273	-0.591602247103
-0.381352953642	-0.591471699594
-0.381263406619	-0.59142088687
-0.381188984823	-0.591339080129
-0.381037498078	-0.59126856117
-0.380923648056	-0.59124415033
-0.380807803378	-0.591194532645
-0.380734114445	-0.591174197093
-0.380626084753	-0.591109295221
-0.380498421853	-0.591056929338
-0.380394455108	-0.59094667605
-0.380299758296	-0.590853930489
-0.380221185687	-0.590779755848
-0.380124267383	-0.59075096412
-0.380021268237	-0.590559541655
-0.379915323926	-0.590402854459
-0.379820456045	-0.590379018242
-0.379715519331	-0.590237546017
-0.379597724699	-0.590190668427
-0.37948247114	-0.589944366358
-0.379388778174	-0.589568465008
-0.379310378219	-0.589501253744
-0.379205863529	-0.589289452573
-0.379069050477	-0.589190597658
-0.378909835412	-0.589023376625
-0.378817239137	-0.588995907267
-0.378711407278	-0.588943894532
-0.378625423811	-0.588855971805
-0.378508582033	-0.588763675437
-0.378356602221	-0.587972892805
-0.378255183583	-0.587955712242
-0.378144768791	-0.587529228237
-0.378045154517	-0.587424039015
-0.377975227485	-0.587387221279
-0.377893605915	-0.587383744682
-0.377793694854	-0.587270633956
-0.377691842127	-0.587170550656
-0.377581162666	-0.587117253584
-0.377470004343	-0.586999468516
-0.377332800608	-0.586885791491
-0.377206894752	-0.586716323166
-0.3771316201	-0.586583808524
-0.377044948523	-0.586500669284
-0.376949956865	-0.586353722995
-0.376855545121	-0.586306997827
-0.376780371064	-0.586288778006
-0.376670992829	-0.586091302441
-0.376572129461	-0.586033163984
-0.376484118188	-0.585923694416

-0.376407293013	-0.585751331691
-0.376278507473	-0.585417550877
-0.376154308588	-0.585019492739
-0.376070527489	-0.584668706044
-0.375946544758	-0.584570589845
-0.375873488055	-0.584503538533
-0.375744233503	-0.584472285887
-0.375668185178	-0.584309248154
-0.375562013913	-0.584307108333
-0.375473466963	-0.584061377298
-0.375385663956	-0.583815134165
-0.375298277958	-0.58365707888
-0.37513763111	-0.583265739369
-0.375023011892	-0.583064890174
-0.37493670917	-0.583062318164
-0.374855538211	-0.582903605952
-0.374777002671	-0.582866112117
-0.3746568093	-0.582804020268
-0.374554175912	-0.582769448141
-0.37447095863	-0.582764845121
-0.374370810547	-0.582729491332
-0.374302896273	-0.582705985486
-0.374215373552	-0.582398694226
-0.374140974967	-0.582366809525
-0.374057885904	-0.582289870151
-0.373947084324	-0.582061861269
-0.373859854869	-0.582038362293
-0.37375425624	-0.581843777822
-0.373650233769	-0.581720186346
-0.373548409041	-0.581624131773
-0.373421099706	-0.581270551016
-0.3733284025	-0.581048395042
-0.373232458503	-0.580934761846
-0.373132298427	-0.580822314321
-0.373065694462	-0.580809095687
-0.372970986021	-0.580612147559
-0.372873444104	-0.580563696227
-0.372768733284	-0.580335682466
-0.372649529466	-0.580213688323
-0.37252768121	-0.580061674707
-0.372462747076	-0.580030565712
-0.372368717989	-0.580020334672
-0.372273853153	-0.5795916875
-0.37218739586	-0.579523946631
-0.372067966216	-0.579503938223
-0.371980952995	-0.579482573528
-0.371853674614	-0.579411726184
-0.371764767807	-0.579177851658
-0.37165962562	-0.579127449996
-0.371526831678	-0.579019067581
-0.371423538189	-0.578980864917
-0.371353685345	-0.578949564513
-0.371237474365	-0.578538863665
-0.371135971718	-0.578296880775

-0.371056284807	-0.578212061454
-0.370978351608	-0.578058957473
-0.370849267214	-0.578045566218
-0.370766994348	-0.577872529183
-0.370671185412	-0.577817630231
-0.370552902152	-0.577802046486
-0.370474844853	-0.57773632098
-0.37036004742	-0.577414866196
-0.370268768577	-0.577262126286
-0.370199920904	-0.577260448512
-0.370125282711	-0.577120005215
-0.370048812772	-0.577041419801
-0.369956186185	-0.577001330591
-0.369820881596	-0.576590299586
-0.369713139552	-0.576229358352
-0.369576163792	-0.576169598719
-0.369463595008	-0.576151906846
-0.369350450533	-0.576120633226
-0.369256263064	-0.576115978308
-0.369109535272	-0.575921260649
-0.36902558055	-0.575884947461
-0.368916366736	-0.575793139997
-0.368822731864	-0.575587075881
-0.368745980148	-0.575276645007
-0.368673947407	-0.575078097251
-0.368594349736	-0.574980015384
-0.368464392348	-0.574697411831
-0.368355505771	-0.57427294445
-0.368232437952	-0.574196858721
-0.368104779694	-0.573801146148
-0.367957232085	-0.57371880297
-0.367862370189	-0.573612378758
-0.367781497129	-0.573581112558
-0.367657963456	-0.573355785651
-0.36754445752	-0.573148304108
-0.367456340237	-0.572982054417
-0.367338393816	-0.572465287032
-0.367248203018	-0.572182776061
-0.367116439293	-0.571937091159
-0.366942084146	-0.571368697979
-0.366841575902	-0.571346554982
-0.366755536268	-0.571138326161
-0.366670466855	-0.571090630692
-0.3665898447	-0.57096340645
-0.366504190514	-0.570933641505
-0.366399566022	-0.570844638043
-0.36632018128	-0.570758157802
-0.366261991375	-0.570650924135
-0.366174694378	-0.570612936013
-0.366053276515	-0.570257221858
-0.365980083998	-0.570072846077
-0.365864951183	-0.569705098073
-0.365701470648	-0.569650315287
-0.36562634093	-0.56958021691

-0.365567409144	-0.569359556843
-0.365479147483	-0.569329308088
-0.365332787085	-0.56876509567
-0.365217187017	-0.56871204795
-0.365126531477	-0.568659083727
-0.365055493765	-0.568655006354
-0.364979233587	-0.568652174151
-0.364914689679	-0.568545886931
-0.364808194589	-0.568484903895
-0.364745019718	-0.568455436418
-0.364638142744	-0.568304391249
-0.364552153323	-0.568302841573
-0.364430832807	-0.568242623564
-0.364352429075	-0.568217723915
-0.36423404462	-0.568015481435
-0.364130730686	-0.567899748662
-0.364050577627	-0.567870009872
-0.363915205092	-0.567630419219
-0.363812038496	-0.567592194073
-0.363699250192	-0.567369143079
-0.363611815848	-0.567265821385
-0.363499062862	-0.567124596781
-0.363384693025	-0.567068624471
-0.363318253183	-0.56699429522
-0.363194263075	-0.566742550889
-0.363055348424	-0.56635446997
-0.362964302975	-0.566231548686
-0.362893192223	-0.566096456149
-0.362777052742	-0.565960821885
-0.362685227783	-0.565721491319
-0.362625243938	-0.565680851427
-0.362491676923	-0.565479188604
-0.36236185462	-0.565440671937
-0.362284672361	-0.565133061753
-0.362190346833	-0.565063730629
-0.36204702577	-0.565060263636
-0.361973205171	-0.565003050935
-0.361880919197	-0.564886370586
-0.361782876736	-0.564879203511
-0.361685320958	-0.564686730906
-0.361611710852	-0.564518105001
-0.36149601964	-0.564463517992
-0.361421626983	-0.564360675882
-0.361328810803	-0.564242448418
-0.361211763382	-0.56411035554
-0.361127298051	-0.563995724069
-0.360989525957	-0.563753490243
-0.360913707823	-0.563602756227
-0.360805588718	-0.563577433202
-0.360721144074	-0.563570000078
-0.360633309858	-0.563561992112
-0.360558253578	-0.563514686571
-0.360478268469	-0.563486093088
-0.360366124121	-0.563408318137

-0.360300462434	-0.563370630735
-0.360194717804	-0.563362766193
-0.360095125998	-0.563270911362
-0.36000947713	-0.563121799979
-0.359893510208	-0.562779270385
-0.359736345285	-0.562408773474
-0.359633930261	-0.562385509395
-0.35952547819	-0.562083161485
-0.359426442269	-0.562059196176
-0.359332477736	-0.562028998242
-0.359236186145	-0.561683045542
-0.359126642349	-0.561602615609
-0.359047269975	-0.561481864946
-0.358946587696	-0.561412289588
-0.358862041179	-0.561340274434
-0.358734562427	-0.56085020388
-0.358617362982	-0.560742648447
-0.358530655343	-0.560687196709
-0.358435156882	-0.560663374685
-0.358368032529	-0.560640739435
-0.358252694508	-0.560447445394
-0.358142097406	-0.560305475045
-0.358046901228	-0.560136516656
-0.357916674588	-0.559990187504
-0.35781920906	-0.559732500013
-0.357742859891	-0.559623868592
-0.357681372403	-0.559551903023
-0.357585877164	-0.559483536073
-0.357476095974	-0.559268006894
-0.357383880798	-0.559156442478
-0.357289258904	-0.559112302988
-0.357138525393	-0.558740074839
-0.357018133968	-0.558737429568
-0.356944428232	-0.558585476213
-0.356879881094	-0.558298117846
-0.356740671992	-0.558137781324
-0.356655855435	-0.558134358284
-0.356559016239	-0.557689059233
-0.356431193735	-0.556960038238
-0.356317866761	-0.556954559001
-0.356222956072	-0.556656702991
-0.35610505446	-0.556598662307
-0.35602300626	-0.556550635964
-0.35590452391	-0.5563177671
-0.355778546168	-0.5562085786
-0.355668751298	-0.556207077063
-0.355563417207	-0.556086078051
-0.355475129646	-0.555753699389
-0.355357575411	-0.555641811501
-0.355265261824	-0.555567773148
-0.35517141744	-0.55538024432
-0.355038888032	-0.55467976986
-0.354925632999	-0.554431804226
-0.35481169397	-0.554312817649

-0.354717261319	-0.554108620551
-0.354640040654	-0.554020440347
-0.354536670641	-0.553891817026
-0.354419538194	-0.553537341911
-0.354351734838	-0.553330452846
-0.354270405968	-0.553313361659
-0.354174539398	-0.553306444013
-0.354054824977	-0.553009091325
-0.353960838755	-0.552906136003
-0.353882457071	-0.552819496304
-0.353795828532	-0.552717658184
-0.353674490684	-0.552567757669
-0.353596425923	-0.552476131235
-0.353492639694	-0.552437038129
-0.353401378895	-0.552110618347
-0.353307209456	-0.552026245457
-0.353219884926	-0.552013717162
-0.353120712178	-0.551960442352
-0.353032082171	-0.551902345876
-0.35294689793	-0.551694528794
-0.352874085214	-0.551621199517
-0.352756289876	-0.551500291594
-0.352617505753	-0.551462585767
-0.352541192548	-0.551407173234
-0.35243722582	-0.551086407749
-0.352307983478	-0.550727615515
-0.352206322135	-0.550673080674
-0.352136069319	-0.550502590637
-0.352033056048	-0.550476644468
-0.351932075093	-0.550464503791
-0.351825400837	-0.550343539802
-0.35167953709	-0.550010552712
-0.351595030635	-0.549855659749
-0.351489128981	-0.549541433076
-0.351419638339	-0.549502694149
-0.351322019252	-0.549173510966
-0.35122226885	-0.548924525924
-0.351111075043	-0.548590264744
-0.350981758154	-0.548520167755
-0.350875746281	-0.548422565759
-0.350796409705	-0.548383918986
-0.350711576023	-0.548237292446
-0.350612469534	-0.548104813167
-0.350471784287	-0.547794320523
-0.35038044205	-0.547663772239
-0.350301667084	-0.547618945002
-0.350187526425	-0.547598287761
-0.350055550147	-0.547054153891
-0.349945878333	-0.546905096029
-0.349869498897	-0.546771415935
-0.349783360668	-0.5466018846
-0.349660399438	-0.546439831453
-0.349569397758	-0.546137940198
-0.349489156517	-0.546115877029

-0.349374968356	-0.545926573844
-0.349295446941	-0.545925734788
-0.349183877167	-0.545784846753
-0.349094701241	-0.54568592573
-0.348970442576	-0.545655543806
-0.348885145588	-0.545641388137
-0.348786974342	-0.545368724523
-0.348711109138	-0.545167079352
-0.348627241167	-0.545090862823
-0.34852480842	-0.545076668359
-0.348365336054	-0.545000691569
-0.348271405186	-0.544964456072
-0.348126067351	-0.544785921781
-0.348040061149	-0.544690841762
-0.347978614315	-0.544674865178
-0.347898498939	-0.54457792178
-0.347822966455	-0.544501053989
-0.347719824115	-0.544124062992
-0.347598823545	-0.543916243503
-0.347529360156	-0.543876683167
-0.347411544624	-0.543817890006
-0.347297733302	-0.543783883903
-0.347159115443	-0.543717493851
-0.347063073565	-0.543637434869
-0.346960695352	-0.543231483653
-0.346876930621	-0.543039679586
-0.346782843323	-0.542900629028
-0.346674784809	-0.542793320767
-0.346559115629	-0.542716501475
-0.346477375047	-0.542682903047
-0.34639136849	-0.542484788302
-0.346312369365	-0.542427330147
-0.346171310725	-0.542305698575
-0.346086028612	-0.542207004606
-0.345995049245	-0.541949333612
-0.345887877079	-0.541938077268
-0.345786845146	-0.541912189155
-0.345708247491	-0.541799342187
-0.345607129793	-0.541532134263
-0.34550700125	-0.541305479722
-0.345385884184	-0.540919182194
-0.345256605154	-0.54058639174
-0.345161986024	-0.540401834165
-0.345051848396	-0.540286139118
-0.344943076476	-0.540054655766
-0.34482383597	-0.539724173506
-0.34470146713	-0.539475653083
-0.344635276207	-0.539466759553
-0.34455409438	-0.539368844185
-0.344473147929	-0.539287454951
-0.344354749311	-0.539156847619
-0.34424944005	-0.539005103372
-0.344179056934	-0.538842025797
-0.344091663318	-0.538770016507

-0.343998957121	-0.538746287924
-0.343906260967	-0.53859282007
-0.343805941109	-0.538547311447
-0.343716793885	-0.538447343133
-0.343648092809	-0.53838631191
-0.343545385212	-0.538376610513
-0.34344844569	-0.538165531571
-0.343355150845	-0.538160420172
-0.343160192183	-0.537973034477
-0.343061908592	-0.537883904863
-0.34296584038	-0.537852287621
-0.342882786386	-0.537799141916
-0.34279075673	-0.537375648437
-0.342692117295	-0.537048490402
-0.342577115408	-0.536977520763
-0.342456151899	-0.536840890847
-0.342334584033	-0.536338706059
-0.342233541621	-0.536244908737
-0.342112525435	-0.536109898048
-0.342009901482	-0.535955751523
-0.341911311001	-0.5358072717
-0.34179169384	-0.535715109909
-0.341693614184	-0.535713404505
-0.341563996748	-0.535622272696
-0.341452316495	-0.535486558769
-0.341362515735	-0.535382338737
-0.341249929085	-0.535264288099
-0.341112291096	-0.535147393685
-0.341038303408	-0.535140424729
-0.340954323777	-0.535127565664
-0.340874153006	-0.53500173741
-0.34075215998	-0.534987324595
-0.340643890573	-0.534728885973
-0.340573889127	-0.534716247152
-0.340472631669	-0.534551858047
-0.340364381891	-0.534517820666
-0.340288146382	-0.534379462838
-0.34019035306	-0.534333150438
-0.340121104887	-0.534140398106
-0.340036430008	-0.53411516521
-0.339940601666	-0.53369704484
-0.339856080959	-0.533575842769
-0.339747941545	-0.533335565753
-0.339638795101	-0.533170461166
-0.339545587277	-0.53316123442
-0.339433024815	-0.532702835204
-0.33933930724	-0.532701518084
-0.339279139942	-0.532691280083
-0.339206677283	-0.532656017844
-0.339123928539	-0.532583065543
-0.339021877327	-0.532308332797
-0.338940895726	-0.532217001458
-0.33886186534	-0.532003344019
-0.338798306867	-0.531948823395

-0.338696655013	-0.531870512323
-0.338611004631	-0.531836517906
-0.338480247816	-0.531674738749
-0.338385928332	-0.531653044911
-0.338299279596	-0.531584455911
-0.338224358187	-0.531518523324
-0.338128937957	-0.531514007899
-0.338041595979	-0.531481069352
-0.337929941624	-0.531042565686
-0.337832402992	-0.530965889417
-0.337709050961	-0.530111388598
-0.337632007648	-0.530065370244
-0.33752600795	-0.530040680441
-0.337434633864	-0.530036763871
-0.337346332927	-0.529693453238
-0.337255732843	-0.529527121976
-0.337160304508	-0.529364149972
-0.337043983128	-0.529202472656
-0.336924812929	-0.529118532982
-0.336815286048	-0.529014469649
-0.336707486914	-0.528532684904
-0.336631133224	-0.528375603919
-0.33653972694	-0.528366744215
-0.336458377758	-0.528282402305
-0.336325008833	-0.528010636496
-0.336199341366	-0.527886119042
-0.336104690323	-0.52768035389
-0.336005752158	-0.527609369421
-0.335918378064	-0.527448500537
-0.335839815433	-0.527362471005
-0.335762945469	-0.527292119364
-0.335684680871	-0.527286799558
-0.335574089364	-0.527280502887
-0.335476671865	-0.527239615648
-0.335364622284	-0.527169189705
-0.335228407384	-0.527123291399
-0.335141717498	-0.527097709424
-0.335062170312	-0.526954753268
-0.334983532708	-0.52689254782
-0.33488526465	-0.526699157628
-0.334798662818	-0.526685743335
-0.334707052975	-0.52665297991
-0.334593580573	-0.526531969808
-0.334469845726	-0.526405986803
-0.334364576908	-0.52600994539
-0.334267061197	-0.525821110793
-0.334154629255	-0.525463430447
-0.334054881903	-0.525200413897
-0.333988512093	-0.525185285816
-0.333899764504	-0.525026555465
-0.333792428487	-0.524998632024
-0.333660625886	-0.524992562915
-0.333585966555	-0.524977986753
-0.333463177989	-0.52470038122

-0.333340775453	-0.524673260644
-0.333257217158	-0.524672086332
-0.333156105602	-0.524437573049
-0.3330633835	-0.524289324886
-0.332903179566	-0.524204794483
-0.332819164289	-0.523903779873
-0.332707070298	-0.523682762346
-0.332619045052	-0.523619062281
-0.332526409188	-0.523573143936
-0.33244797848	-0.523512917643
-0.332375334209	-0.523480807089
-0.332275607524	-0.523363064448
-0.332181749729	-0.523329153978
-0.33213807986	-0.523304197297
-0.332037677425	-0.523222855767
-0.331932154703	-0.522946838247
-0.331790549936	-0.522632007519
-0.331663002525	-0.522329386354
-0.331518116127	-0.522106079211
-0.33143607365	-0.522042020762
-0.331314390885	-0.521975171538
-0.331218601141	-0.521893248035
-0.331135908793	-0.521787296787
-0.33105455447	-0.521626833628
-0.33095462198	-0.521488302414
-0.330849069569	-0.521476352634
-0.330729596837	-0.521274673339
-0.330628803122	-0.52109691053
-0.330539314168	-0.520969002252
-0.330479540106	-0.520905157582
-0.33035104665	-0.52068618111
-0.330276173893	-0.520663479648
-0.330185196795	-0.520655857576
-0.330078927382	-0.520634221298
-0.330003906357	-0.520613649974
-0.329895700783	-0.52057570432
-0.329787873155	-0.52051660109
-0.329715632958	-0.520467134812
-0.329631299387	-0.520239108785
-0.329525625628	-0.520092973644
-0.329422953155	-0.520040475928
-0.329361974025	-0.52002820297
-0.329230505466	-0.519679674732
-0.329146571071	-0.519580145043
-0.329034569709	-0.519536646548
-0.328943372147	-0.51942291405
-0.328888284974	-0.519355166668
-0.328763206395	-0.519273300163
-0.328693245321	-0.519109786971
-0.328610953087	-0.518982112614
-0.32850412958	-0.518780323355
-0.328371468045	-0.518706725319
-0.328286178339	-0.518607716945
-0.328201663841	-0.518554132076

-0.328103259489	-0.518491993658
-0.328039008171	-0.518373690285
-0.327957095338	-0.518370502152
-0.327805216596	-0.518042091596
-0.327710038478	-0.518009029518
-0.327609529784	-0.517979073448
-0.327505573812	-0.517650675469
-0.327420399106	-0.517463784709
-0.327297030059	-0.517173072691
-0.32722892078	-0.517110365616
-0.327140936512	-0.517070694368
-0.327049598321	-0.516880847048
-0.326955520231	-0.516875677994
-0.326882938371	-0.516862530222
-0.326796152589	-0.516729379212
-0.326686935377	-0.516585599179
-0.326580475843	-0.516498637589
-0.326487071839	-0.516429579446
-0.326427482033	-0.516378188912
-0.326332437871	-0.51614558469
-0.326251878401	-0.516145218066
-0.32613325159	-0.515817380315
-0.326071928441	-0.515798103317
-0.325987911661	-0.515562137296
-0.325914264296	-0.515455951789
-0.325815938462	-0.515114192139
-0.32569547438	-0.514993552599
-0.325615619075	-0.514742012986
-0.32553405841	-0.514525494285
-0.325430377178	-0.514379987319
-0.325347448151	-0.514304011716
-0.325191636863	-0.51427707724
-0.325094272344	-0.514216552955
-0.325009539559	-0.514053599283
-0.324929046064	-0.51401701894
-0.3248314995	-0.513951152992
-0.324723421162	-0.513910985281
-0.324639218255	-0.513908077258
-0.324551613354	-0.513873348009
-0.32445083894	-0.51360863165
-0.324367992743	-0.513600706587
-0.324287664379	-0.513408240892
-0.324187657259	-0.513388443223
-0.324098196019	-0.513373840763
-0.324022403747	-0.513351601238
-0.323930593577	-0.513327890557
-0.323812944172	-0.513243200975
-0.32371649059	-0.51316141853
-0.323622136856	-0.513153922078
-0.323517841283	-0.512875870077
-0.323395992332	-0.512619731913
-0.323290781723	-0.512575988009
-0.323198843512	-0.512159895613
-0.323068288776	-0.512111149798

-0.322992773829	-0.5120676907
-0.322872165999	-0.511767523641
-0.322753823176	-0.511392209863
-0.322627576977	-0.511163567384
-0.322554857945	-0.511123782519
-0.322493857949	-0.511111932456
-0.322397237215	-0.5110872025
-0.322284902539	-0.510697352972
-0.322177227924	-0.510463832027
-0.322081656266	-0.509986331835
-0.322014044752	-0.509970076583
-0.321924776313	-0.509931999731
-0.321828285511	-0.50979933898
-0.321766832896	-0.509770789541
-0.32168262282	-0.509468063244
-0.321617339397	-0.509230458868
-0.32150410922	-0.509196590917
-0.321413332584	-0.508964851945
-0.321310031163	-0.508935375398
-0.321218545316	-0.508687009506
-0.321105183124	-0.508523867296
-0.320992913505	-0.508246937599
-0.320899585234	-0.508208110262
-0.32080001758	-0.508167401427
-0.320727749769	-0.508102120797
-0.32064011933	-0.507979694577
-0.320551240301	-0.50796868973
-0.32048246123	-0.507922972869
-0.320374158243	-0.507918497022
-0.320237213132	-0.507552965087
-0.320132544901	-0.507515529944
-0.320023198087	-0.507507229667
-0.319931787179	-0.507496237323
-0.319819189647	-0.507423415486
-0.319759058804	-0.507419304968
-0.319650625822	-0.506772387097
-0.319496110041	-0.506586959976
-0.319377044016	-0.505838689572
-0.319285402125	-0.505630100302
-0.319196273693	-0.505607885371
-0.319080562223	-0.505177486932
-0.318988710588	-0.50500783491
-0.318894718181	-0.50498970548
-0.31878433532	-0.504688825556
-0.318722877577	-0.504496833143
-0.318635168232	-0.504382734448
-0.318549411511	-0.504298420175
-0.318442772759	-0.504118905026
-0.318302489038	-0.503959662725
-0.318191292439	-0.503783418488
-0.318099531843	-0.503597517572
-0.318026243963	-0.503521279132
-0.31788971072	-0.503276880263
-0.317806171989	-0.503222580945

-0.317713241192	-0.503086387983
-0.317619174139	-0.503081349812
-0.317506908685	-0.503034416757
-0.317415759747	-0.502960270303
-0.317329463415	-0.502710265914
-0.317243694852	-0.502682649913
-0.317117364356	-0.501973645245
-0.31702577529	-0.50197001539
-0.316919234406	-0.501630950951
-0.316787854551	-0.501470769673
-0.31671698523	-0.501448621455
-0.316619211837	-0.501398435262
-0.316536436652	-0.501326133054
-0.316443149993	-0.50132047523
-0.316339904029	-0.501304752573
-0.316246513813	-0.501169072954
-0.316142473725	-0.500877077912
-0.316058434156	-0.500734597691
-0.315969020587	-0.500660715321
-0.315898580346	-0.500642872192
-0.315811018045	-0.500586457478
-0.315687001988	-0.500183660466
-0.315586953331	-0.500130141613
-0.315499302212	-0.499896540142
-0.31542137923	-0.499828209862
-0.315298812467	-0.49959131552
-0.315206666129	-0.499571323499
-0.315083078295	-0.499026583583
-0.314988100312	-0.498821445011
-0.314917492095	-0.498751065432
-0.314794051402	-0.498527298209
-0.31470926915	-0.498471599075
-0.314632441602	-0.498446108881
-0.314540109545	-0.498411302633
-0.314415865931	-0.498235698887
-0.314360338815	-0.498112561562
-0.314260071912	-0.497961816293
-0.314109305124	-0.497650672733
-0.314052922633	-0.497614930932
-0.313948674556	-0.49757982871
-0.313862225424	-0.49726985858
-0.313797000304	-0.497088926687
-0.313699405193	-0.497010038049
-0.313607189606	-0.496905873245
-0.31350356228	-0.496629582919
-0.313399875145	-0.496624370932
-0.313289353847	-0.49654537021
-0.313175583516	-0.496502457882
-0.313064271013	-0.496322748784
-0.312965983279	-0.496249814137
-0.312905377271	-0.496244489174
-0.312792759604	-0.496019882763
-0.312732328584	-0.496017612258
-0.312604252772	-0.496001585584

-0.31250987726	-0.495930764157
-0.31241018572	-0.495851991988
-0.312298948079	-0.495782180887
-0.312168454787	-0.495200298797
-0.31206111977	-0.495129286277
-0.311934861896	-0.495121782356
-0.311801870619	-0.495009470262
-0.311685629445	-0.494628984369
-0.311620747436	-0.494625811591
-0.311528143639	-0.494612486795
-0.311460430626	-0.494453145784
-0.3113952877	-0.494420882195
-0.31129384693	-0.494121805475
-0.311191947352	-0.494078379438
-0.311111504483	-0.494075874708
-0.311016415841	-0.49403427908
-0.310926078802	-0.493976638903
-0.310808538898	-0.493928136286
-0.310706963867	-0.493850286202
-0.310609605479	-0.493694255926
-0.31049561769	-0.49355900243
-0.310387714767	-0.493301087324
-0.31027867793	-0.492884775752
-0.310197965561	-0.492764561724
-0.31013353029	-0.492702905642
-0.31005814037	-0.492670716725
-0.309946629377	-0.492586529356
-0.309858614431	-0.492258036101
-0.309761551287	-0.492254390214
-0.309684190982	-0.49200262593
-0.309580107053	-0.491870552739
-0.309504900004	-0.491808560399
-0.30941494229	-0.491709373508
-0.309319896311	-0.491622363184
-0.309234161206	-0.491615720039
-0.309159326781	-0.491598008693
-0.309071523587	-0.49158529724
-0.308973003965	-0.491392230724
-0.308883381842	-0.491391563536
-0.308738502768	-0.491335937955
-0.308638183614	-0.491227565361
-0.308556801044	-0.49104520968
-0.308472383436	-0.490860168065
-0.308405937622	-0.490840609005
-0.308330887846	-0.490549164425
-0.308214172191	-0.489958738484
-0.308076067114	-0.489340181369
-0.308002824545	-0.489234386093
-0.307909890398	-0.489227071214
-0.307847479552	-0.489098189325
-0.307778428506	-0.489084940171
-0.307694688784	-0.489043315417
-0.307586063696	-0.488960678711
-0.307499190928	-0.488930160718

-0.307395977401	-0.488845454234
-0.307277985905	-0.488649849149
-0.307217248329	-0.488635824912
-0.307140828605	-0.488579476704
-0.307040740608	-0.488578543093
-0.306969866801	-0.488518567567
-0.306879588026	-0.488156632418
-0.306775416844	-0.487689614185
-0.306687068017	-0.48765039166
-0.306586467776	-0.487562026117
-0.306475812872	-0.487549933053
-0.306382030939	-0.487421894459
-0.306279992468	-0.487377254569
-0.306133776469	-0.487311127829
-0.306056349911	-0.487249397375
-0.305984749398	-0.487196545104
-0.305893656114	-0.4871621997
-0.305771452365	-0.487029389139
-0.305659217295	-0.486806518011
-0.30555733501	-0.486781434191
-0.305456692021	-0.486701955929
-0.305372921929	-0.486570643391
-0.30529656974	-0.4865301655
-0.305217914091	-0.486433712545
-0.305126495837	-0.486432035565
-0.305016029605	-0.486382686757
-0.304917070767	-0.485993233612
-0.304846393471	-0.485955005047
-0.30477233029	-0.485745400153
-0.304669661682	-0.485663710727
-0.304565704301	-0.485379650909
-0.304474054308	-0.485324607317
-0.304382181067	-0.485214367089
-0.30428362096	-0.485050684105
-0.304205237401	-0.485034500033
-0.304108915674	-0.4849665272
-0.304014654243	-0.484902413967
-0.303919376609	-0.48480508189
-0.303827555816	-0.484632424181
-0.303766899575	-0.484622108822
-0.303673123583	-0.484526731154
-0.30354494583	-0.484500761596
-0.303487363959	-0.48449245844
-0.303393357499	-0.484414849223
-0.303272244288	-0.48435517613
-0.303185478992	-0.484199261086
-0.30310518554	-0.484159458626
-0.303025273776	-0.483886072924
-0.3029102382	-0.483864500531
-0.302817850896	-0.483738498605
-0.302720560035	-0.483708256476
-0.302650786772	-0.483544978253
-0.302558083318	-0.48353769351
-0.302452280938	-0.4831959967

-0.302353718013	-0.483004254889
-0.302263902758	-0.482829325048
-0.302152755898	-0.482571098363
-0.302029574886	-0.482333549538
-0.301932911387	-0.482266938376
-0.301840519358	-0.48212358422
-0.301763755467	-0.481904019261
-0.301686535737	-0.48183490065
-0.301576501452	-0.481710445513
-0.301476777527	-0.481638666076
-0.301396791326	-0.481622119156
-0.301304527386	-0.481548890668
-0.301211191784	-0.481482479596
-0.301088389563	-0.481021771131
-0.301002651065	-0.481002109056
-0.300907132004	-0.480994702208
-0.300826821167	-0.48097602592
-0.300744580879	-0.480887142299
-0.300633031504	-0.480833505495
-0.300547204366	-0.480724581857
-0.300446400012	-0.480714171957
-0.300368349977	-0.480710040531
-0.300295403942	-0.480703332594
-0.300185084253	-0.48046074781
-0.300097287171	-0.480441742065
-0.300020958984	-0.480336536551
-0.299956290539	-0.480209606718
-0.299860448109	-0.480183818522
-0.299773635758	-0.480168916929
-0.299679606261	-0.479931990467
-0.299595361634	-0.479874643778
-0.299531047916	-0.479851023432
-0.299418459874	-0.479695332892
-0.299338064898	-0.479492152133
-0.299227490071	-0.479278005229
-0.29915977079	-0.479139672029
-0.299077635696	-0.47913076424
-0.298991452023	-0.479106266108
-0.298903533216	-0.478952734044
-0.298792775866	-0.478881577341
-0.298694018659	-0.478625142721
-0.298597053087	-0.478579924759
-0.298490865708	-0.478383130684
-0.298390927559	-0.477961822506
-0.298302520536	-0.47795399175
-0.298203296645	-0.477808788522
-0.29804585491	-0.477170248116
-0.297954277994	-0.477155795576
-0.297869819148	-0.47713547953
-0.29779085551	-0.477055412501
-0.297713856666	-0.476991205988
-0.297603530105	-0.476813990594
-0.297507832194	-0.476807208358
-0.297401008768	-0.476768325278

-0.297272686567	-0.476400267511
-0.297190094683	-0.476343378863
-0.29706783907	-0.476145590014
-0.296979773945	-0.476087811513
-0.296900051496	-0.476069597627
-0.296830695199	-0.475983656546
-0.296769077806	-0.475938441531
-0.296629849588	-0.475406043781
-0.296509185622	-0.47539405
-0.296411253415	-0.475290100063
-0.296341881714	-0.47528214636
-0.296233474067	-0.475271898649
-0.296143102254	-0.475266948703
-0.296035228268	-0.475208438181
-0.29590213852	-0.474989786037
-0.295785501565	-0.474600624754
-0.295697155734	-0.474506289136
-0.295617307368	-0.47448361639
-0.295508355524	-0.474313878459
-0.295421075071	-0.47425380795
-0.295356081623	-0.474086125227
-0.295274663834	-0.473985605594
-0.295195195065	-0.473983805222
-0.295088551985	-0.473891921354
-0.295006838435	-0.473769452767
-0.294885351256	-0.473736662021
-0.294742770274	-0.473655887232
-0.294654902936	-0.473573663954
-0.294557467833	-0.473480046951
-0.294429922861	-0.473093659187
-0.29429637057	-0.472573529738
-0.294231485397	-0.472472549867
-0.294151080144	-0.472425129748
-0.294054194927	-0.472301015792
-0.293968106196	-0.472256867079
-0.293876546207	-0.471910090294
-0.293777683536	-0.471840148725
-0.293696406844	-0.47181616775
-0.293591540262	-0.471465093576
-0.293496342103	-0.471289507811
-0.293414673618	-0.471259470926
-0.293298043599	-0.471186079414
-0.293157664965	-0.471031675721
-0.293039034297	-0.470720343754
-0.292887722447	-0.470497960671
-0.292773837224	-0.470427562694
-0.292696406434	-0.470386182297
-0.292624746056	-0.470347598321
-0.292499137595	-0.470044262175
-0.292410210268	-0.46984531384
-0.292311821642	-0.469757599121
-0.292229620349	-0.469696886938
-0.292150516253	-0.469691542375
-0.292038580113	-0.469261175562

-0.291961216448	-0.469130411969
-0.291864479709	-0.469080142626
-0.291780182089	-0.469013546366
-0.291704013243	-0.468992943709
-0.291602015851	-0.468750404375
-0.291485444224	-0.468222233915
-0.291377757036	-0.467962323717
-0.291299613148	-0.467886961884
-0.291217676299	-0.467875882722
-0.291116137042	-0.467667632669
-0.290993156314	-0.467544809548
-0.290897806618	-0.467499034734
-0.29081801831	-0.467485655313
-0.290714731617	-0.46738818765
-0.290599530805	-0.46730941787
-0.290532127644	-0.467289027883
-0.290460945673	-0.467183831396
-0.290397209648	-0.467164874375
-0.290323209315	-0.46713418872
-0.290244422963	-0.467092652203
-0.290126462845	-0.467034673552
-0.290024360131	-0.467004036125
-0.289947371019	-0.466876824072
-0.289848501038	-0.466846657779
-0.28971917215	-0.466821656717
-0.28965340877	-0.466702821834
-0.289564635214	-0.466611186082
-0.289447662005	-0.466575060847
-0.289332078652	-0.466380509007
-0.289215422697	-0.466201954589
-0.289125471816	-0.466088021601
-0.289034796196	-0.465637972603
-0.288943644114	-0.465440241002
-0.288864344677	-0.465290434875
-0.288762733781	-0.465164923302
-0.288670754507	-0.464996531789
-0.288588907298	-0.464981315447
-0.288474558872	-0.464865799821
-0.288331274566	-0.464617830223
-0.288268218253	-0.464480668192
-0.288159770729	-0.464272724268
-0.288061363405	-0.46423608065
-0.287915189377	-0.463961601913
-0.287802461462	-0.463803090985
-0.287713808674	-0.46362331299
-0.287653328929	-0.463614049347
-0.287563914168	-0.463608859534
-0.287464434266	-0.463541658979
-0.287369103443	-0.463510648853
-0.287277940971	-0.463439720099
-0.287187552804	-0.463429367534
-0.287106905902	-0.463423953586
-0.286982279943	-0.463067109919
-0.286865783111	-0.463002329408

-0.28676768344	-0.462888052551
-0.286655388734	-0.462810867884
-0.286560675276	-0.462715479301
-0.28642568567	-0.462667855991
-0.286347400478	-0.462660258421
-0.286265558158	-0.462437413902
-0.286190486309	-0.462429244405
-0.286066026092	-0.462002727071
-0.28595054426	-0.461963848415
-0.285857239469	-0.461686464328
-0.2857305835	-0.461182914312
-0.285667401881	-0.461102570728
-0.285569892317	-0.460967883711
-0.285492545991	-0.460866510856
-0.285381168126	-0.460781316236
-0.285278624018	-0.460478522689
-0.285215434236	-0.460328436689
-0.285144258446	-0.460214157663
-0.285044600382	-0.460164970748
-0.284960281563	-0.460156869994
-0.284873460188	-0.459920206904
-0.284814633027	-0.459902072903
-0.284711224625	-0.459828036341
-0.284590826661	-0.459113294978
-0.284462258522	-0.45890632091
-0.284399631608	-0.458902697303
-0.284291367243	-0.458850993776
-0.284176679555	-0.458262439248
-0.284060494435	-0.458049555211
-0.28395039786	-0.457854863077
-0.283840513375	-0.457708402328
-0.283753034077	-0.457583331766
-0.28366684258	-0.457513231516
-0.283564446109	-0.457497102549
-0.283455795849	-0.456973654454
-0.283372597507	-0.45692962841
-0.283304426746	-0.45690100084
-0.283231545589	-0.456768053224
-0.283129802473	-0.456627819243
-0.283029547728	-0.456522640516
-0.282947842627	-0.456487610102
-0.282842102828	-0.456081681072
-0.28271885013	-0.456018387186
-0.282607185918	-0.455974631856
-0.282519016629	-0.455902351333
-0.282387474009	-0.455745484066
-0.282273524795	-0.455410140724
-0.282161384382	-0.455367250525
-0.282097923482	-0.455345241931
-0.281988120323	-0.455240914875
-0.2818527057	-0.454700755801
-0.281735873285	-0.45431673887
-0.281637467184	-0.454184794372
-0.281536969697	-0.454145389424

-0.281440746457	-0.454047079533
-0.281313389016	-0.453772944355
-0.281239585919	-0.453638256811
-0.28114732639	-0.453434574864
-0.281087570034	-0.453429068243
-0.281028333963	-0.453230184441
-0.280919932312	-0.453123041624
-0.280797351209	-0.452824943084
-0.280721135571	-0.452784309533
-0.280641839569	-0.452668074767
-0.280541785796	-0.452633731116
-0.280431345501	-0.452486424172
-0.280339246791	-0.452484985434
-0.280200610729	-0.452439701402
-0.280146858793	-0.452366191402
-0.280016517047	-0.452341776175
-0.279913225627	-0.452248225015
-0.279827622826	-0.452221927795
-0.279751038435	-0.4521854353
-0.279649758928	-0.452140189329
-0.279526808115	-0.451959591365
-0.279417387471	-0.451730746001
-0.279343114588	-0.451654053338
-0.279206853666	-0.451589746869
-0.279142847861	-0.451583864795
-0.2790329554	-0.451270308714
-0.278948513668	-0.451217945543
-0.278853457717	-0.4511251939
-0.27875143313	-0.451036227541
-0.278681902952	-0.450963400037
-0.278589691165	-0.450611409733
-0.278487669725	-0.450577144594
-0.278407787363	-0.450561516618
-0.278325001322	-0.450334115223
-0.278234649103	-0.450282964202
-0.278132753068	-0.450260006825
-0.27805174183	-0.450148394899
-0.277955447655	-0.450117946031
-0.277860483125	-0.450091031248
-0.277767929596	-0.450050798472
-0.277672415093	-0.450045791083
-0.27754447372	-0.449533687632
-0.27747859066	-0.449401328736
-0.277409979336	-0.449291374781
-0.277301312654	-0.449242449226
-0.277186006032	-0.448746916291
-0.277107479295	-0.448713436763
-0.276978421969	-0.448539504838
-0.276856119206	-0.448485824314
-0.276770903901	-0.448475599785
-0.276711408197	-0.448429897195
-0.27662667454	-0.448382333116
-0.276529481151	-0.448346939294
-0.276405216144	-0.448320020591

-0.27633654776	-0.448216234144
-0.276195638449	-0.447749020694
-0.276110734118	-0.447693471135
-0.276023659045	-0.447623414946
-0.275928740998	-0.447361322277
-0.275838261264	-0.447260140383
-0.275755975938	-0.447249070329
-0.275691810464	-0.447218247814
-0.275605798346	-0.447211051207
-0.275524991585	-0.447205001411
-0.275399669559	-0.447186759863
-0.275339135642	-0.447158174709
-0.275263775924	-0.447033906415
-0.275150702243	-0.446868573415
-0.275031139331	-0.446662131286
-0.274969129814	-0.446623533012
-0.274868736938	-0.446522338803
-0.274763170007	-0.44647179496
-0.27465310977	-0.446045144814
-0.274511448713	-0.445527646647
-0.274409436699	-0.44528751581
-0.274282768614	-0.445022348473
-0.27419488771	-0.444998081808
-0.274079689519	-0.444764762334
-0.273988461419	-0.444693970632
-0.273916852876	-0.444647387061
-0.273852675676	-0.44457119207
-0.273713142136	-0.444064858312
-0.273622469981	-0.444051233201
-0.273529845817	-0.444010147846
-0.273436929121	-0.443890169037
-0.273322842424	-0.443660838964
-0.273212175482	-0.443637768339
-0.273132559189	-0.443473696386
-0.273024593239	-0.443268983433
-0.272944143595	-0.443224465462
-0.272834602028	-0.443048503068
-0.272760933306	-0.443038476256
-0.272671159363	-0.442916494597
-0.272574687555	-0.442906233293
-0.272474318055	-0.442651969823
-0.272398856726	-0.442529793638
-0.272305567099	-0.442307130593
-0.272222404915	-0.442210827301
-0.272131119268	-0.442048444012
-0.272049306954	-0.441949902985
-0.271933394783	-0.441774776419
-0.271832670962	-0.441664615096
-0.271722877392	-0.441536483519
-0.27163928246	-0.441512533335
-0.271516783981	-0.441398537956
-0.27142100272	-0.441207211033
-0.271325414578	-0.441149394287
-0.27124352433	-0.441062609287

-0.271137812219	-0.441062231563
-0.271062044725	-0.440968451519
-0.270972542851	-0.440936083054
-0.270865070912	-0.440861119585
-0.270747159176	-0.440725818775
-0.270657374285	-0.440559303262
-0.270580326201	-0.440526834548
-0.270527806487	-0.440470875677
-0.270441764294	-0.440283691633
-0.27030101193	-0.439911840315
-0.270191392078	-0.439894729065
-0.270120464648	-0.439831295375
-0.270006069552	-0.439825319531
-0.269919568538	-0.439738880499
-0.269827438915	-0.439487744242
-0.26973857345	-0.43930006148
-0.269649405987	-0.439288693227
-0.269564057645	-0.439278499202
-0.269464248579	-0.439234895815
-0.269376148737	-0.439151236448
-0.26929998183	-0.439146599725
-0.269194093556	-0.438923490442
-0.269042523789	-0.438632940653
-0.268974932803	-0.438536782082
-0.268875240898	-0.438533416593
-0.26878677767	-0.43838805377
-0.268683124593	-0.437919540633
-0.268580389886	-0.43790830206
-0.268483282082	-0.437897813998
-0.268372011377	-0.437614641619
-0.26830422784	-0.437448852493
-0.268239060711	-0.437306415639
-0.268190464125	-0.437209065559
-0.268093338181	-0.437024214928
-0.267968704898	-0.436784277243
-0.267869994759	-0.436633773281
-0.267780313297	-0.436612791953
-0.267668814311	-0.436334943929
-0.267573529505	-0.436330298649
-0.267458643361	-0.436272460556
-0.267351575423	-0.436186382615
-0.26724928708	-0.436180760128
-0.26716497051	-0.436064917981
-0.267038421719	-0.435721038978
-0.266939445608	-0.435562864688
-0.266861490206	-0.435543556128
-0.26674788088	-0.435494615089
-0.266664406954	-0.435461665334
-0.266564335081	-0.435433516634
-0.266473194589	-0.435280043072
-0.266368768772	-0.43525621986
-0.266283478079	-0.43504910754
-0.266191167939	-0.434971883301
-0.266049195058	-0.43493861593

-0.265977476207	-0.434908100051
-0.265856212134	-0.434646384934
-0.265742265914	-0.434569900112
-0.265641786663	-0.434569510212
-0.265576703403	-0.434545353262
-0.265511317077	-0.43446898884
-0.265405408323	-0.434183630739
-0.265321535151	-0.434158175229
-0.265226038984	-0.434148574973
-0.265140099793	-0.434090264818
-0.265033315836	-0.433864888953
-0.264917640847	-0.433834628607
-0.2648163654	-0.433767423238
-0.264689333752	-0.433291144178
-0.264605454679	-0.433172905205
-0.264505775289	-0.433098476542
-0.264450639822	-0.433097566826
-0.26434309718	-0.432448856283
-0.264237469795	-0.432445213742
-0.264137142552	-0.432290545792
-0.264041746047	-0.432162934853
-0.263955906958	-0.431994748063
-0.263829057997	-0.431799140532
-0.263735956214	-0.431604228312
-0.263617708688	-0.431300992733
-0.263517764411	-0.43110777328
-0.263400568716	-0.430757836918
-0.263268765432	-0.430524369812
-0.263186676027	-0.430519409365
-0.263070333075	-0.43012123063
-0.262955094819	-0.429897961179
-0.262796841003	-0.429877145157
-0.26267416502	-0.429539864545
-0.262550120217	-0.429121708706
-0.26244911594	-0.429050228238
-0.26234662112	-0.429038074888
-0.26224606204	-0.428989579373
-0.262130223629	-0.428964168435
-0.262007476933	-0.428547406598
-0.261881504736	-0.427804812117
-0.261787623943	-0.427753626363
-0.261679717918	-0.427572160703
-0.261573509132	-0.427553517248
-0.261451669711	-0.4274425397
-0.261382349761	-0.427430576082
-0.261288398164	-0.427360819439
-0.261179559095	-0.427157444955
-0.261092286243	-0.427043015221
-0.260998757148	-0.426998217209
-0.260888532728	-0.426980632015
-0.260787394796	-0.426747820497
-0.260701615605	-0.426552886236
-0.260581863036	-0.42641019007
-0.260518153844	-0.426246590435

-0.260425350119	-0.426135989632
-0.260374556631	-0.426087761911
-0.260252764398	-0.42540156324
-0.260126115844	-0.424803529687
-0.260053738664	-0.42476488963
-0.259925982828	-0.424693486706
-0.259824006938	-0.424625020079
-0.259735037065	-0.424525506725
-0.259660373489	-0.424501694619
-0.259563967864	-0.424413023447
-0.25945655867	-0.424275506874
-0.259381452054	-0.424202533714
-0.259298477345	-0.424156851053
-0.259191022495	-0.424125565871
-0.259112920871	-0.424120946637
-0.258992223261	-0.423958617065
-0.258917189327	-0.423815167818
-0.258834396751	-0.423748521578
-0.258746137672	-0.423576198546
-0.258647091957	-0.423355452011
-0.25856027843	-0.423128288197
-0.258476216007	-0.423088058379
-0.258398452344	-0.423014592784
-0.258319445256	-0.422963535642
-0.258186895615	-0.422837946408
-0.25808873246	-0.422663007983
-0.258043079782	-0.422575095629
-0.257953353298	-0.422563718
-0.257800796879	-0.421933198597
-0.257714322261	-0.421839798784
-0.257630530971	-0.42183406395
-0.257550666271	-0.421784601645
-0.257472782339	-0.421596008107
-0.25739547469	-0.421581448924
-0.257307325953	-0.421366850306
-0.25725668742	-0.421252986006
-0.257167470079	-0.421051968692
-0.257116091982	-0.420922854867
-0.257035162847	-0.420910407632
-0.256929553887	-0.420820766533
-0.256822227731	-0.420517412465
-0.256735769911	-0.42040001796
-0.256586446877	-0.420341492409
-0.256505397986	-0.420333143587
-0.256424436296	-0.420046489001
-0.25633942345	-0.41985134456
-0.256282469412	-0.419759030867
-0.256183512136	-0.419724907732
-0.256046444066	-0.419369584538
-0.255954714315	-0.419341007467
-0.255860271889	-0.419284325793
-0.255772830144	-0.419159975199
-0.255681864334	-0.419078483137
-0.255576410796	-0.419021346448

-0.255457888918	-0.418980047279
-0.255396310385	-0.418928729093
-0.255318960777	-0.418867201791
-0.25521356898	-0.418624406582
-0.255105343972	-0.418532999809
-0.255032441012	-0.418412129739
-0.254937800332	-0.418354547244
-0.254763758839	-0.417829092485
-0.254663933544	-0.417782915316
-0.254558425176	-0.417567352129
-0.254500017267	-0.417560614264
-0.254398330307	-0.417554792389
-0.254324324768	-0.417536886243
-0.254236567532	-0.417365648082
-0.254156901842	-0.417270784441
-0.254078639928	-0.417059678949
-0.253935920601	-0.416859065241
-0.253826214052	-0.416729641748
-0.253746237637	-0.416536849879
-0.253607584682	-0.415982606025
-0.253512876017	-0.415584364501
-0.253417990811	-0.415412238582
-0.253312442122	-0.41519792961
-0.253181777422	-0.415122004189
-0.253084882666	-0.414952410878
-0.252986632182	-0.414776264392
-0.252884159518	-0.414730539628
-0.252801060433	-0.414664399559
-0.25270378264	-0.414572419583
-0.252635176066	-0.41442756469
-0.252525818492	-0.414165748523
-0.252464891884	-0.414102559831
-0.252370415924	-0.414047234989
-0.252274018979	-0.414003629039
-0.252199511585	-0.413807603504
-0.252131795862	-0.413779743407
-0.25204536187	-0.413513328254
-0.25195050718	-0.413267078743
-0.251905547311	-0.413256496516
-0.251828679433	-0.41306659838
-0.251728692853	-0.413031791347
-0.251609997391	-0.412862898299
-0.251513884249	-0.41279048867
-0.251390372703	-0.412677365248
-0.251283214986	-0.412650378062
-0.251163949433	-0.412410610546
-0.251084629548	-0.412174591221
-0.250989016006	-0.411781065615
-0.250868591076	-0.411646954726
-0.250759267551	-0.411437564022
-0.250668791096	-0.411328360267
-0.250576899343	-0.411314808968
-0.250485953071	-0.411199272706
-0.250385934106	-0.411189340444

-0.250288964842	-0.411177592611
-0.250194104281	-0.411028327329
-0.250129713881	-0.410954624256
-0.250055751693	-0.410907118053
-0.249948469132	-0.410625448954
-0.249855342217	-0.410502440079
-0.249750967676	-0.410287829203
-0.249681992123	-0.410255489297
-0.2495845042	-0.410193496056
-0.249501359869	-0.410013037275
-0.249408037771	-0.409918825523
-0.249296538715	-0.409164474204
-0.249223203297	-0.408925175906
-0.249105944799	-0.408631637189
-0.249034480872	-0.40854834337
-0.248939844308	-0.408266126917
-0.248855498682	-0.408213414904
-0.248741084259	-0.408132939359
-0.248675765313	-0.407999480107
-0.248585842336	-0.407896026368
-0.248501547315	-0.407895806263
-0.248440803651	-0.407872364151
-0.248367068777	-0.40783979763
-0.248264945336	-0.407790518661
-0.248161267489	-0.407671312113
-0.248085387702	-0.407610160713
-0.247984769822	-0.407592345736
-0.247920122383	-0.40758480987
-0.247795536273	-0.407185979215
-0.247690907676	-0.406903494102
-0.24758236932	-0.406669644108
-0.247473349281	-0.406639197576
-0.247363419214	-0.406465950124
-0.247271056127	-0.40646359905
-0.247147909437	-0.406287933484
-0.247051457663	-0.406179216234
-0.246948253929	-0.406177442569
-0.246852267517	-0.406024233315
-0.246730908582	-0.405967991659
-0.246636685266	-0.405784634993
-0.246579975692	-0.405745373609
-0.246494566446	-0.405670991724
-0.246380961054	-0.405576738944
-0.246269275038	-0.405508492048
-0.246145654678	-0.40546585378
-0.246017898748	-0.40525417489
-0.245946284688	-0.405228985272
-0.245834508257	-0.404761816286
-0.245753072523	-0.404273221094
-0.245682926695	-0.404068244367
-0.245559749302	-0.403516485191
-0.245425002171	-0.402960049413
-0.245322275796	-0.402933794766
-0.245242056731	-0.40290648458

-0.245140590627	-0.402817881972
-0.245044621196	-0.40251430248
-0.244928508266	-0.402352374145
-0.244845132166	-0.402307207626
-0.244758718416	-0.402088242065
-0.244640983961	-0.401813090031
-0.244546834155	-0.401797236685
-0.24445531876	-0.401699192836
-0.244344574126	-0.401672242962
-0.244282216696	-0.401641614117
-0.244196800068	-0.401343214166
-0.244071501414	-0.400939918029
-0.243945760063	-0.40090324488
-0.243846727607	-0.400854630351
-0.24375490905	-0.400608150458
-0.243674266645	-0.400582174054
-0.243567658653	-0.400546061523
-0.243465644696	-0.400476741095
-0.24338386214	-0.40042605898
-0.24329013412	-0.400389045718
-0.243220196564	-0.400374951759
-0.243127006575	-0.40034338071
-0.243012510141	-0.400183824489
-0.242932151463	-0.400176694658
-0.242834815147	-0.39999806274
-0.242744463449	-0.399795795801
-0.242623288786	-0.399750375008
-0.242497274917	-0.399562174221
-0.242371301414	-0.399178648618
-0.242261027027	-0.399097001113
-0.242158073771	-0.398993569751
-0.242092392282	-0.398803538564
-0.24201659153	-0.398767442465
-0.241888844373	-0.398386998927
-0.241812786819	-0.398275892097
-0.241700736481	-0.398041375162
-0.241539626946	-0.397421696769
-0.241481831686	-0.397345345727
-0.241396808844	-0.397321489639
-0.24131767068	-0.39702491581
-0.241251692136	-0.397020941585
-0.241138678446	-0.396713814411
-0.240988838598	-0.396617554927
-0.240900870806	-0.396565358195
-0.240815255414	-0.396454666817
-0.240731968931	-0.396307915249
-0.240664773162	-0.396263060533
-0.240594253545	-0.396234671668
-0.240457738388	-0.396121013042
-0.24033519476	-0.395676704589
-0.240254101698	-0.395675144544
-0.240183239262	-0.395660756771
-0.240060915036	-0.395586456473
-0.239966112925	-0.395486805387

-0.239880923594	-0.395484635124
-0.239768915018	-0.395481074882
-0.239687375334	-0.395442728237
-0.239580819039	-0.395433576864
-0.239484530499	-0.395323348815
-0.23940476677	-0.39519987073
-0.239302433645	-0.395141212072
-0.239245417704	-0.395136080981
-0.23914348264	-0.395116070637
-0.23904495044	-0.394941231669
-0.238977547159	-0.394841934373
-0.238859766925	-0.394787901649
-0.23878963072	-0.394689464976
-0.238656301501	-0.394571653846
-0.238557754075	-0.394259863179
-0.238501100083	-0.394245469081
-0.238390425596	-0.394181989278
-0.238272205264	-0.394107257867
-0.238142739573	-0.394031111841
-0.238063573367	-0.394006696178
-0.23798416938	-0.393972087358
-0.237925167383	-0.393914435706
-0.2378122661	-0.393831035633
-0.237731107109	-0.393479887845
-0.237644258309	-0.393468736697
-0.237549581914	-0.393285236921
-0.237453818197	-0.393265453645
-0.237360295185	-0.393037299927
-0.237248208399	-0.392705226385
-0.237180254967	-0.392662024612
-0.237098839966	-0.392599373726
-0.236989268587	-0.392574550363
-0.236907704868	-0.392256289439
-0.23680856618	-0.39207644935
-0.236709419767	-0.391937731847
-0.236618791802	-0.391908498902
-0.236526038859	-0.391829164753
-0.236451875297	-0.39176110178
-0.236336698405	-0.391654812928
-0.236219408552	-0.391598334438
-0.236126091863	-0.391469072273
-0.236057950685	-0.391442659674
-0.235973604123	-0.39141947588
-0.235867284461	-0.391416953865
-0.235765424605	-0.391411369111
-0.235670897053	-0.391251017574
-0.235589377732	-0.391237468976
-0.235480447885	-0.391105480124
-0.235390942806	-0.391063795733
-0.235256802694	-0.390838011033
-0.23519760008	-0.390822853758
-0.23512417979	-0.390821845952
-0.235013728617	-0.390586453479
-0.234912230093	-0.390393757799

-0.234819757907	-0.390333214507
-0.234750848294	-0.390237501434
-0.234635608066	-0.390151452297
-0.234571218	-0.390060521869
-0.234490615143	-0.38998221182
-0.234402841277	-0.389883243662
-0.234339275207	-0.389723025236
-0.234242099615	-0.389585649857
-0.234151391145	-0.389578630019
-0.234057896443	-0.389523545885
-0.233982707421	-0.389407450202
-0.233904224906	-0.389326172227
-0.233827923849	-0.389319537432
-0.233740866111	-0.389094979215
-0.233661465755	-0.388870689753
-0.233546572098	-0.388172382709
-0.233457347792	-0.387939393864
-0.233353189666	-0.387892057898
-0.233272190488	-0.387670028795
-0.23316771915	-0.387339267411
-0.233093068893	-0.387304360587
-0.233004493048	-0.387166806618
-0.232906445127	-0.387146494858
-0.232791288696	-0.38688626638
-0.232711504712	-0.386861883235
-0.232624552951	-0.38677006159
-0.232506540553	-0.386307153753
-0.232411734903	-0.386238950911
-0.232309589171	-0.386210070668
-0.232221951367	-0.386096329045
-0.232148385267	-0.386052583401
-0.232064220012	-0.386024070226
-0.231951318473	-0.385800375956
-0.231879966501	-0.385635998761
-0.231808977252	-0.38544583292
-0.231726767291	-0.385180903228
-0.231665734414	-0.385035022243
-0.23157501692	-0.384888910769
-0.231508649034	-0.384841177961
-0.231432619594	-0.384699279867
-0.231316097712	-0.384503582015
-0.231269632135	-0.384434089838
-0.231161304227	-0.384217321391
-0.231045192852	-0.383679395835
-0.230932119876	-0.383517925169
-0.230842112072	-0.383509977629
-0.230741162812	-0.383502359828
-0.230656558995	-0.383461198896
-0.230580874479	-0.383397514136
-0.230464727569	-0.383308057757
-0.230339348127	-0.383219478352
-0.230261453393	-0.383008402516
-0.23018994145	-0.38288742217
-0.230093074017	-0.382748653777

-0.229964657998	-0.382410912099
-0.229872981679	-0.382327507055
-0.229744558299	-0.381853811087
-0.229672414809	-0.381770398076
-0.229575448261	-0.381666520168
-0.229478797378	-0.381523890522
-0.22937930403	-0.381462261516
-0.229278611151	-0.381279687596
-0.229171382197	-0.381088190111
-0.229069156865	-0.380683588405
-0.228951197216	-0.38053410229
-0.228850783051	-0.380431316596
-0.228747508614	-0.380397728286
-0.228642316726	-0.380219058236
-0.228542692481	-0.380031572241
-0.228411809601	-0.379845651254
-0.228310191296	-0.379681865391
-0.228188417694	-0.379454125417
-0.228108607522	-0.379412414951
-0.227989432569	-0.379350527928
-0.227890468696	-0.379260602438
-0.227792205982	-0.379158231366
-0.227715592072	-0.379136143654
-0.227644323157	-0.379098405259
-0.227515617353	-0.378541858286
-0.227421407381	-0.378287679577
-0.227340327199	-0.378246915997
-0.227232884154	-0.378063487677
-0.227156989793	-0.378057613834
-0.227051989435	-0.378012411286
-0.226975728247	-0.377984826824
-0.226884956193	-0.377957277381
-0.226801747298	-0.377897154765
-0.226696504807	-0.377763672517
-0.226626420051	-0.377625862999
-0.226550064758	-0.377594228119
-0.226476922136	-0.377485914431
-0.226398220598	-0.377290279093
-0.22631700181	-0.377192042119
-0.226211718052	-0.376936730284
-0.226130149176	-0.376720584958
-0.226054931649	-0.376648178624
-0.22593833269	-0.376516541034
-0.225836850858	-0.376471564409
-0.225713779834	-0.376355300044
-0.225580995786	-0.376168325319
-0.225501731351	-0.376133997189
-0.225422851212	-0.376079205556
-0.2253405376	-0.376033187538
-0.225255324969	-0.375745283191
-0.22516608469	-0.375683968038
-0.225029362169	-0.375509872576
-0.224973456509	-0.375506255962
-0.224843354891	-0.375286119411

-0.224763156104	-0.375283554498
-0.224630098424	-0.37489989475
-0.224528479478	-0.374862138767
-0.224422386506	-0.374827279401
-0.224329772862	-0.374817377632
-0.224225990141	-0.374485993659
-0.224159348934	-0.374414913936
-0.224056052158	-0.374360309203
-0.223988448764	-0.374336617487
-0.223906790836	-0.374294869058
-0.223805331337	-0.37419587692
-0.223733525657	-0.374131638196
-0.223611089297	-0.374063833591
-0.223490118681	-0.37383374613
-0.223409934953	-0.373702777362
-0.223334988712	-0.373506984616
-0.223205404887	-0.373034145722
-0.223107544477	-0.372873180883
-0.223014018833	-0.372848825125
-0.222944586438	-0.37269467057
-0.222860181271	-0.372247901299
-0.222739253624	-0.371960243782
-0.222643985134	-0.371786714883
-0.222576639686	-0.371770991866
-0.222494594157	-0.37167294916
-0.222430232478	-0.371659647568
-0.222357796786	-0.371622567091
-0.222246170604	-0.37114866673
-0.222154209352	-0.370936610042
-0.222075169469	-0.370822574901
-0.222008219527	-0.370730592631
-0.22188626622	-0.370345288412
-0.221792966782	-0.370147645782
-0.22169893134	-0.370111603877
-0.221592624009	-0.370040529088
-0.221500721853	-0.369805012168
-0.221394414306	-0.36954706108
-0.221282743821	-0.368667856652
-0.221205299908	-0.368579824179
-0.221135292809	-0.368433101408
-0.221045912747	-0.368431209746
-0.220921698719	-0.367944071883
-0.220819139505	-0.367930586848
-0.220706511435	-0.367639873268
-0.2206235779	-0.367250290481
-0.220528292417	-0.367242203855
-0.220413288237	-0.367116252477
-0.220310411047	-0.36702054502
-0.220250009616	-0.366918790575
-0.220163884776	-0.366793132929
-0.220072453812	-0.36668413058
-0.219997697955	-0.366679834687
-0.219916297479	-0.366543800622
-0.219813977379	-0.366225080202

-0.219734933303	-0.365988846487
-0.219641364518	-0.36596853954
-0.21954655052	-0.365955140738
-0.219467767861	-0.365644833858
-0.219376657531	-0.365544439256
-0.219276984382	-0.365417425251
-0.219178516978	-0.36538178003
-0.219087356099	-0.365166819314
-0.218996490959	-0.365050166453
-0.218904348494	-0.364962304471
-0.218818416291	-0.364746583255
-0.218746621858	-0.364688549951
-0.218645268424	-0.364472579144
-0.218564617343	-0.364391431123
-0.218496454109	-0.364216666917
-0.218408867965	-0.364028159405
-0.218331580902	-0.364010471134
-0.21821467688	-0.363523933689
-0.218145740601	-0.363511548864
-0.218021762901	-0.363259810419
-0.217910697979	-0.363090267801
-0.217802115561	-0.363019909642
-0.217695512519	-0.362708067327
-0.217622563013	-0.362610610874
-0.217524880137	-0.3625560678
-0.217443344671	-0.362395406236
-0.217324560507	-0.361811877888
-0.217233117865	-0.361679333952
-0.217154077201	-0.361263386362
-0.2170597492	-0.361238885552
-0.216984524625	-0.361203840756
-0.216908527733	-0.361100838496
-0.216807596748	-0.361084593005
-0.21673219335	-0.361074830789
-0.216613183837	-0.360877707713
-0.216516120239	-0.360739097957
-0.216433549433	-0.360735302291
-0.216308491781	-0.360721391904
-0.216231307772	-0.36048377332
-0.216132690177	-0.360284792197
-0.216030828241	-0.36026469167
-0.215944740594	-0.360148691007
-0.21587353623	-0.360102849483
-0.215796242423	-0.360092552975
-0.215714143824	-0.359876844087
-0.215599281155	-0.359730821855
-0.215517275437	-0.359635229825
-0.215420856281	-0.359465905985
-0.215332510908	-0.359355471472
-0.215239190824	-0.359326776588
-0.215145875459	-0.359325217699
-0.215032118079	-0.359318900424
-0.214947783753	-0.359283232764
-0.214844268742	-0.358970768394

-0.214763352744	-0.3588005786
-0.214653836219	-0.358743813424
-0.214574290185	-0.358708545265
-0.214478600501	-0.358675844601
-0.21441345895	-0.358665823327
-0.214340743164	-0.358552784287
-0.214259493774	-0.358258681954
-0.21417608779	-0.358136333983
-0.214086072868	-0.357954308565
-0.213991194137	-0.357840853718
-0.213888579676	-0.357720066403
-0.213827630901	-0.357664858283
-0.213735678521	-0.357622984226
-0.213630851124	-0.357575018022
-0.213529795158	-0.357431140163
-0.213473437455	-0.357380374777
-0.213404131862	-0.357380363654
-0.213334870414	-0.357300522303
-0.213257892176	-0.357170192819
-0.213171858034	-0.357002645889
-0.21308537505	-0.356974367539
-0.212973799373	-0.356712602898
-0.212858923216	-0.356311273389
-0.212746089332	-0.355767789206
-0.212659305091	-0.355619693886
-0.212575061606	-0.355613342227
-0.21249423346	-0.355579484389
-0.212379378329	-0.355299891636
-0.212256372349	-0.35499948777
-0.212176933767	-0.354860986786
-0.21207116574	-0.354852062005
-0.211980649822	-0.35474770821
-0.211907721104	-0.354554950376
-0.211808534922	-0.354464083549
-0.211676623105	-0.354375994094
-0.211577985198	-0.354322997212
-0.211489223501	-0.354258603892
-0.211425555817	-0.354243917896
-0.211349277409	-0.354200734454
-0.211269094667	-0.35416135154
-0.211167092618	-0.354100490211
-0.211061174511	-0.353929504916
-0.21098701294	-0.353858243593
-0.210872152371	-0.353541453357
-0.210754510044	-0.35328272388
-0.210685223374	-0.353125346446
-0.210608749706	-0.353119431705
-0.210523949546	-0.353054154815
-0.210418534732	-0.352723267295
-0.210319066666	-0.352561273979
-0.210223111045	-0.352376958774
-0.210141558245	-0.352189489018
-0.210056828236	-0.3521040275
-0.20997323986	-0.352045089717

-0.209869306346	-0.351944864187
-0.209728271026	-0.351811138036
-0.209627742136	-0.351435304454
-0.209514599659	-0.351283357405
-0.209449103219	-0.351205743495
-0.209346595152	-0.350880380113
-0.209239454629	-0.350562688371
-0.209130803735	-0.350337077037
-0.209036148224	-0.350306400302
-0.208957321576	-0.350289873002
-0.208836223078	-0.350280020379
-0.208751560354	-0.35022729854
-0.208651813376	-0.349831017463
-0.208568407524	-0.349828685083
-0.208465512086	-0.349678494227
-0.208378352329	-0.349596083823
-0.208264953508	-0.349353890349
-0.208161905229	-0.349325752289
-0.208098977956	-0.349174993082
-0.20799791639	-0.349114513324
-0.207932112614	-0.349010625327
-0.20785283094	-0.348782991913
-0.207748418178	-0.348634108484
-0.207656924231	-0.348327087428
-0.207559657764	-0.348260614007
-0.207421224709	-0.34782949024
-0.207321605573	-0.347756842487
-0.207203434447	-0.347579138386
-0.207104116073	-0.347500919419
-0.207029300655	-0.347355810671
-0.206955831654	-0.347340980584
-0.206894479693	-0.347125700863
-0.206798988863	-0.3469644304
-0.206702095043	-0.346495797051
-0.206590024158	-0.346230250329
-0.206515858616	-0.346108179451
-0.206437223857	-0.346020749604
-0.206353710344	-0.345964604581
-0.206274600872	-0.345895805979
-0.206215081918	-0.345877426575
-0.206071437043	-0.345636320139
-0.205976250133	-0.345124879852
-0.205903276881	-0.344932394439
-0.205813876862	-0.344780292068
-0.205747293781	-0.344648758966
-0.205665066029	-0.344564599036
-0.205574702011	-0.344508700785
-0.205481079604	-0.344230970396
-0.205392188019	-0.344123391691
-0.2052898685	-0.343894998148
-0.205244374572	-0.343838231269
-0.20518437467	-0.343828878758
-0.205083558761	-0.343445498236
-0.205000524247	-0.343212263732

-0.204900522052	-0.343153144631
-0.204844653703	-0.343121242751
-0.204755939538	-0.342982986051
-0.204688363141	-0.342892907141
-0.204609129007	-0.342878988282
-0.204533619953	-0.342854125735
-0.204420075974	-0.342794509782
-0.204298707759	-0.342662496728
-0.204225780283	-0.342528796187
-0.20414962791	-0.342447656305
-0.204063577761	-0.342445910436
-0.20398813186	-0.342283874309
-0.203900845355	-0.342147128295
-0.203831453864	-0.342147073284
-0.203741191834	-0.341996445865
-0.203642959797	-0.341985157725
-0.203564122724	-0.341930025787
-0.20347559937	-0.341870133975
-0.203372123445	-0.341719749643
-0.20325975525	-0.341445065972
-0.203168098298	-0.341360167819
-0.203036249989	-0.340885031938
-0.20295175253	-0.340801525881
-0.202826453157	-0.340700179942
-0.202754092905	-0.340666843795
-0.202668015245	-0.340571285569
-0.202583072157	-0.34052997479
-0.20246552006	-0.340482492712
-0.202376408812	-0.340096250561
-0.202280116739	-0.340086569148
-0.202147606181	-0.340075952976
-0.20206036277	-0.339955456276
-0.201970840551	-0.339922188691
-0.201846199192	-0.339306173404
-0.201766214435	-0.339265152118
-0.201708881806	-0.339196203761
-0.201606775643	-0.338963907549
-0.201524015707	-0.338660364919
-0.201412281476	-0.338489605875
-0.201309503073	-0.338415003879
-0.201218422415	-0.338368544398
-0.201063556098	-0.337758339356
-0.200987442995	-0.337592597865
-0.200913897492	-0.337552914125
-0.200844885109	-0.337296727109
-0.200727088225	-0.337207933864
-0.200592538621	-0.336958446417
-0.200512315178	-0.336813350843
-0.200426958431	-0.336622313422
-0.200329095055	-0.336422055812
-0.200246936698	-0.336343148902
-0.200135716338	-0.336317786694
-0.200073647794	-0.336279297577
-0.199991483704	-0.336265116324

-0.199918781686	-0.336260851238
-0.1997888754	-0.33603905185
-0.199686212139	-0.335759972267
-0.199616250532	-0.335698720771
-0.199522725607	-0.335596730722
-0.199420398484	-0.335426688219
-0.199287851124	-0.335311859156
-0.199190930141	-0.335152519721
-0.199113006479	-0.335065372789
-0.199006000517	-0.334832353877
-0.198915705659	-0.334786407687
-0.198843012771	-0.33469656144
-0.198743566433	-0.334375525901
-0.198635794454	-0.334056919125
-0.198537575936	-0.333937307594
-0.198437294113	-0.333765029177
-0.198324594188	-0.333713064229
-0.198223359896	-0.333326451592
-0.198129000504	-0.333275808233
-0.198052067619	-0.333193117214
-0.197964288989	-0.332945943061
-0.197886631003	-0.332881943118
-0.197779149739	-0.332724971823
-0.197695353082	-0.332701917038
-0.197609485247	-0.332544874747
-0.197518840814	-0.332530702443
-0.197391402595	-0.33231668516
-0.197285919374	-0.33220545756
-0.197191293548	-0.332116034009
-0.197063866639	-0.331781377047
-0.196963271109	-0.331707416801
-0.196853615681	-0.331553082943
-0.196764327677	-0.331523662727
-0.196647073319	-0.331514627453
-0.196582734429	-0.33149622271
-0.19646827223	-0.331136914213
-0.196382782662	-0.331102764772
-0.196301842808	-0.331082840821
-0.196205070985	-0.331069980047
-0.196122260523	-0.331045845182
-0.196038623447	-0.330832302265
-0.195909485877	-0.330520168267
-0.195822590291	-0.330174217497
-0.19573190898	-0.330067442903
-0.195647459865	-0.33006642945
-0.195565971781	-0.330059103021
-0.195468202578	-0.329917079898
-0.195363960903	-0.329747610791
-0.19523937284	-0.329532068644
-0.195147667472	-0.329482309606
-0.195084614471	-0.329421338854
-0.1949852472	-0.329407150933
-0.19490365366	-0.329291255969
-0.194778180133	-0.329205448862

-0.194666125416	-0.329091187165
-0.194552633656	-0.328701643112
-0.194463787365	-0.328563054722
-0.194382162502	-0.328541643829
-0.194255683082	-0.328288391331
-0.194166649603	-0.328128882286
-0.194066258438	-0.327996921171
-0.193973906031	-0.327963160176
-0.193899574077	-0.327947520123
-0.193812986093	-0.327670846206
-0.193715394332	-0.327634619837
-0.193614327055	-0.327392084896
-0.193530312477	-0.327320872619
-0.193412670436	-0.326964998643
-0.193310675488	-0.326824922789
-0.193219920472	-0.326268648578
-0.193125047416	-0.325986421053
-0.193033887161	-0.325762438368
-0.192941865945	-0.325655143222
-0.192842941433	-0.325618032455
-0.192761416213	-0.325397509577
-0.192650753706	-0.325141851754
-0.192562820591	-0.324891386193
-0.192468324124	-0.324776608447
-0.192394295259	-0.324714301072
-0.192327190634	-0.3246756008
-0.192244203712	-0.324607052124
-0.19211857918	-0.324398169918
-0.192043323065	-0.324362309468
-0.191986127471	-0.324295221363
-0.191914987119	-0.324284095195
-0.191789075412	-0.324260100789
-0.191698744681	-0.324251027021
-0.191613481705	-0.324190836351
-0.191538892738	-0.324136637253
-0.19142096522	-0.323810038317
-0.191307353035	-0.323384130389
-0.191224857421	-0.323336163709
-0.191152717456	-0.323232307129
-0.191051134748	-0.323217420263
-0.190946453735	-0.323090905413
-0.190865783234	-0.322952182972
-0.190796253376	-0.322873262523
-0.190702455621	-0.32286373037
-0.190646137823	-0.322753259819
-0.190554989777	-0.322587385445
-0.190467476885	-0.322486904033
-0.190377794209	-0.322471909162
-0.190289096594	-0.322448493434
-0.190182040723	-0.322384761669
-0.190085423776	-0.322256054514
-0.190014368491	-0.322250642635
-0.189936612506	-0.322003622952
-0.189858350705	-0.321944312063

-0.189784260834	-0.321938373218
-0.189675190955	-0.321661216504
-0.189540571394	-0.32116788673
-0.189463903871	-0.321143845418
-0.189355103316	-0.320711142834
-0.189268392526	-0.320680760076
-0.18920191254	-0.320608366358
-0.189099826683	-0.320373150782
-0.18900124831	-0.320253661611
-0.188894134191	-0.320195819092
-0.188806591792	-0.320125053161
-0.188709154184	-0.320030712385
-0.188591855907	-0.319796399931
-0.18848046637	-0.31977875367
-0.18838857894	-0.319612175942
-0.188267178137	-0.319596497272
-0.188151062215	-0.319217892944
-0.188080645599	-0.319176354995
-0.187981407069	-0.319107848152
-0.187870278054	-0.319056720529
-0.187764185435	-0.319023606382
-0.187689256225	-0.318975672028
-0.187581760525	-0.318680136077
-0.187465350532	-0.318488600656
-0.187363078623	-0.318333908089
-0.187277377903	-0.318082848706
-0.187171551725	-0.318004356508
-0.18707318398	-0.317941405967
-0.186982891616	-0.317831735524
-0.186861961903	-0.317332689036
-0.186778501092	-0.317157963784
-0.186696306201	-0.317114820723
-0.186610894495	-0.317089034617
-0.186501748443	-0.31702724719
-0.186416405473	-0.316783829393
-0.186321255123	-0.31664431496
-0.186190521422	-0.316625154699
-0.18610307363	-0.316346602649
-0.186024319806	-0.316292319765
-0.185917768365	-0.316036261043
-0.185808004795	-0.316013585628
-0.185704441698	-0.315864750952
-0.185596334683	-0.315580042061
-0.185520423828	-0.315449475048
-0.18543474506	-0.315447410314
-0.185344309889	-0.315356166281
-0.185231608719	-0.315163636433
-0.185125058874	-0.315122787795
-0.18504436314	-0.315118277214
-0.18494681409	-0.3147837852
-0.184855429326	-0.314766299536
-0.184765450718	-0.314626111972
-0.184675649833	-0.314524147855
-0.184547548179	-0.314339533699

-0.184420163264	-0.314316899675
-0.184344986021	-0.314266056416
-0.184264003196	-0.314200097218
-0.184176983355	-0.314086634735
-0.184101715407	-0.314029817818
-0.184018154078	-0.313978292784
-0.18396434119	-0.313942143258
-0.183876340042	-0.313934344568
-0.183803732847	-0.313906528997
-0.183723093023	-0.313882916626
-0.183618745085	-0.313764899931
-0.183529147615	-0.313735784013
-0.183419159232	-0.313579158119
-0.183356167525	-0.313556971981
-0.183295446937	-0.313470881177
-0.183179610105	-0.313393539189
-0.183070836757	-0.313320925554
-0.182985144939	-0.31327613423
-0.182884219887	-0.31312200352
-0.182803596581	-0.313083460851
-0.182724908912	-0.313063044832
-0.182601210196	-0.312954375101
-0.182525222794	-0.31291173976
-0.182435479106	-0.312725886333
-0.182342324085	-0.312520866583
-0.18225986532	-0.312462605585
-0.182143359212	-0.312395099295
-0.182048325112	-0.312258543263
-0.18194618693	-0.311874147244
-0.181841659364	-0.311705243442
-0.181739346204	-0.311373788527
-0.181643582749	-0.311266877489
-0.18152537141	-0.311220703779
-0.181440047226	-0.310876263496
-0.181336683807	-0.310358474996
-0.18128331164	-0.310247148636
-0.181207844363	-0.310047089773
-0.181120530831	-0.310033017957
-0.181028470406	-0.310026063017
-0.180895450382	-0.30984445797
-0.180832064204	-0.309634253129
-0.180757828287	-0.309620789804
-0.180661945829	-0.309578449449
-0.18057416452	-0.309550479905
-0.180486582383	-0.309411385429
-0.18033898375	-0.309152665566
-0.180262062248	-0.308928579365
-0.180175912563	-0.30884987237
-0.180056309333	-0.308436909889
-0.179945128298	-0.308300266435
-0.179870642524	-0.308285244437
-0.179761555883	-0.308076134834
-0.179687781089	-0.307968026318
-0.179598628693	-0.307887606028

-0.179542753496	-0.307814903332
-0.179419938664	-0.307757982638
-0.179331704065	-0.307592841783
-0.179253558639	-0.307517715502
-0.179179510833	-0.307495303354
-0.179084805753	-0.307336848112
-0.178965042676	-0.306755768908
-0.178865738458	-0.306700704935
-0.178793966071	-0.30663385738
-0.178695334855	-0.30645604448
-0.178608099616	-0.306392952259
-0.178509733926	-0.306144548746
-0.178421542389	-0.305989304262
-0.178315469719	-0.305972424479
-0.178226270969	-0.305894486272
-0.178104983872	-0.305782932957
-0.178001753788	-0.3057507726
-0.177944313865	-0.305702335524
-0.177859291688	-0.305638884524
-0.177750098372	-0.305550381279
-0.177675776873	-0.305261257022
-0.177579715696	-0.305078653858
-0.177481085951	-0.305022661389
-0.177423496074	-0.304949988411
-0.177320645839	-0.304817698758
-0.17725419875	-0.304764935522
-0.177175782488	-0.304731138194
-0.177096864694	-0.30471496121
-0.176987689971	-0.304624704307
-0.176925476146	-0.3045988713
-0.176834946919	-0.304535015481
-0.176732014638	-0.304459231778
-0.176641577301	-0.304283472031
-0.176522724929	-0.304166695593
-0.176418151548	-0.304057255204
-0.17634180226	-0.303885842505
-0.176251591196	-0.303762476447
-0.176158559862	-0.303743637583
-0.176066794922	-0.303622174325
-0.176000678064	-0.303592853554
-0.175926060484	-0.30333852006
-0.175828351571	-0.3032765156
-0.175727575802	-0.303270976319
-0.17563499575	-0.303207449225
-0.175523908148	-0.303052583293
-0.175440924066	-0.302886235808
-0.175376169082	-0.302850413041
-0.175270799411	-0.302574084749
-0.175160360441	-0.302536366898
-0.175096629106	-0.302522922982
-0.175023329321	-0.302418702109
-0.174926928915	-0.302182966035
-0.174855085261	-0.302131301251
-0.17474827161	-0.302115701183

-0.174669875643	-0.301886453602
-0.174558647365	-0.301637965687
-0.174476803184	-0.301574500152
-0.174365373384	-0.301393099338
-0.174275478546	-0.301326920756
-0.174173426763	-0.301098072338
-0.174093117822	-0.30108725264
-0.17397266768	-0.300931122905
-0.173860564163	-0.300331634223
-0.173752230005	-0.300085806576
-0.173624274171	-0.299597447006
-0.173514462943	-0.299275372628
-0.17342998114	-0.299100327937
-0.173361440227	-0.299072665711
-0.173299198927	-0.299036458097
-0.173200375898	-0.298859317012
-0.173116812195	-0.298689400152
-0.17299102858	-0.298637546779
-0.172907785397	-0.298568008109
-0.172734655849	-0.298122579497
-0.172606927693	-0.298073726545
-0.172497087384	-0.297923856738
-0.172417244075	-0.297823564595
-0.172335693362	-0.29774904126
-0.172256008643	-0.297627151673
-0.172154465719	-0.297431316378
-0.172083566309	-0.297399316396
-0.171995514033	-0.296948943652
-0.171895504134	-0.296673373283
-0.171798178399	-0.296451464924
-0.171697924314	-0.296434138404
-0.171625622316	-0.296286701859
-0.171525792472	-0.295880322397
-0.171453034489	-0.295870295737
-0.171354087356	-0.29554772888
-0.171258284879	-0.295495722844
-0.171181247929	-0.295400587294
-0.171095590664	-0.295337068427
-0.170996773302	-0.295281510943
-0.170943495852	-0.295269630922
-0.170809406279	-0.295240183133
-0.170700012141	-0.295083712168
-0.170568431563	-0.294797585194
-0.170496198083	-0.294795034647
-0.170423492794	-0.294642827835
-0.170344758961	-0.294509260935
-0.17025001367	-0.294460116081
-0.170179063463	-0.294366400836
-0.170061819068	-0.294266658056
-0.169988200437	-0.294190416431
-0.169909490461	-0.293865991728
-0.169837717192	-0.29370391166
-0.169776466273	-0.293624047643
-0.169711799216	-0.293563638801

-0.169615053972	-0.293265876822
-0.169518296815	-0.293251381275
-0.169405840025	-0.293241364518
-0.169302459994	-0.293038238438
-0.16919966668	-0.292892415747
-0.169129213099	-0.292877441219
-0.169037468297	-0.292687825241
-0.168946417382	-0.292595773133
-0.168867769033	-0.292578847893
-0.168813090916	-0.292544616173
-0.168725202477	-0.292457514547
-0.168650487444	-0.292298153002
-0.168578582532	-0.29222811037
-0.1685024835	-0.292105486818
-0.168382389217	-0.291704611498
-0.168295734725	-0.291686077756
-0.168208189029	-0.291420043927
-0.168109521052	-0.290894374011
-0.168036589226	-0.290882370643
-0.167967174342	-0.290879458579
-0.16788334469	-0.290845871711
-0.167775703791	-0.290797025524
-0.167691086285	-0.290768582187
-0.16762024297	-0.290743142669
-0.167528555002	-0.290614812256
-0.167444274607	-0.290571859385
-0.167333665405	-0.290378336289
-0.167240137662	-0.290345229125
-0.167142197703	-0.290282006827
-0.167048199792	-0.290142451226
-0.166934922127	-0.289883350754
-0.166853877963	-0.289854377938
-0.166781279755	-0.2898057109
-0.166681312587	-0.289802257447
-0.16658146401	-0.28961742628
-0.166511047178	-0.289597728946
-0.166384799631	-0.289431944836
-0.166312607491	-0.289354025053
-0.166210063047	-0.289257661598
-0.16612787365	-0.28902662061
-0.166038407296	-0.288890172667
-0.165917069272	-0.288677852346
-0.165834813586	-0.288626978061
-0.165759745674	-0.288596058137
-0.165645390366	-0.288155644776
-0.165530881994	-0.288142744397
-0.165448465547	-0.288139064403
-0.165342080502	-0.287797674383
-0.165262178248	-0.287669602993
-0.165208065923	-0.287658805644
-0.165120698956	-0.28759618453
-0.165033282418	-0.287539076963
-0.164942044078	-0.287537258684
-0.164852806656	-0.287178666846

-0.164735177437	-0.287076440466
-0.164650574413	-0.286982274421
-0.164578773745	-0.286945920377
-0.164518829577	-0.286912840632
-0.164430884035	-0.286736491102
-0.164287619845	-0.286297345778
-0.164171760049	-0.28626458918
-0.164069248647	-0.286232933606
-0.163968199792	-0.285985415044
-0.163897343938	-0.285955324967
-0.163812717545	-0.285827611534
-0.163715595037	-0.285673020742
-0.163612603798	-0.285476782526
-0.163532987982	-0.28540269792
-0.163405364409	-0.28527092433
-0.163304288393	-0.28520359714
-0.163238338257	-0.284986375545
-0.163143366643	-0.284695226167
-0.163056546268	-0.284652668512
-0.162971747791	-0.284537067984
-0.162904083139	-0.284530925315
-0.162799209662	-0.284433354406
-0.162728707393	-0.284422166364
-0.162640069903	-0.284406975554
-0.16254471639	-0.284242614284
-0.162432418296	-0.283973565633
-0.162367270439	-0.283928583958
-0.162274911664	-0.283862493479
-0.162156654501	-0.2837089014
-0.162055484854	-0.283318842249
-0.161988603937	-0.283125439723
-0.161906420738	-0.283125346054
-0.161808280083	-0.282878491456
-0.161718044432	-0.282800861917
-0.16163472848	-0.28271007822
-0.161520181199	-0.282503066574
-0.161453022918	-0.282469644236
-0.161355408543	-0.282398481872
-0.161258310885	-0.282003221045
-0.161179297016	-0.281958453993
-0.161083881151	-0.281749303954
-0.161002900499	-0.281576984243
-0.160936273773	-0.281412577169
-0.160858166537	-0.28138374811
-0.160767570107	-0.281361319252
-0.160718560837	-0.281276519095
-0.160645928811	-0.281210432889
-0.160568205787	-0.281203548822
-0.16047043809	-0.281096968497
-0.160369866445	-0.281089874515
-0.160255442705	-0.281051515669
-0.160129384413	-0.281035097329
-0.160025521745	-0.281025069229
-0.159939943374	-0.280971446918

-0.159855687974	-0.280838155412
-0.159746990203	-0.280732388943
-0.159637298292	-0.280704426177
-0.159488871738	-0.280361886212
-0.159415658434	-0.280359697982
-0.159325717851	-0.280087545829
-0.159245768099	-0.280084967521
-0.159144028274	-0.279631674804
-0.159063413335	-0.279398274523
-0.158957247889	-0.279322244868
-0.158898108532	-0.279296207936
-0.158800450383	-0.278851030066
-0.158701213156	-0.278631489577
-0.158634235968	-0.278584584209
-0.158542756361	-0.278532520726
-0.15845250372	-0.278452912983
-0.158388237198	-0.27841616623
-0.158273748284	-0.278307627585
-0.158155238265	-0.278274139314
-0.158041428233	-0.278026893178
-0.157966798048	-0.277949802565
-0.157895176849	-0.277926159767
-0.157792090722	-0.277864052586
-0.157709653741	-0.277698078921
-0.157619887535	-0.277539233253
-0.157517188791	-0.277521383454
-0.157433217148	-0.27733749152
-0.157346608454	-0.277330754213
-0.157247823153	-0.277247659174
-0.157153491543	-0.277238477892
-0.157065170719	-0.277090517505
-0.156979709521	-0.277038808974
-0.156893127526	-0.277035409987
-0.156813092352	-0.276880664063
-0.156708872305	-0.276493983101
-0.156628495951	-0.276461950167
-0.156560769072	-0.276239597494
-0.15646919304	-0.276216183797
-0.156368045684	-0.276191639083
-0.15629455942	-0.276183828579
-0.156219233692	-0.276128791223
-0.156129586634	-0.276113023111
-0.156036915633	-0.276080691241
-0.155962256642	-0.276074578052
-0.155884819423	-0.27605575671
-0.155789470495	-0.275893336247
-0.15573610066	-0.275858024602
-0.155639801198	-0.275696039781
-0.15556266128	-0.275606814699
-0.155463970308	-0.27532723774
-0.155382686744	-0.275324077704
-0.155307657426	-0.275242814987
-0.155204137639	-0.274994741108
-0.155115004157	-0.274714900686

-0.155000361626	-0.274554308867
-0.154909108171	-0.274499061808
-0.15478283226	-0.273934412215
-0.154693608712	-0.273883242032
-0.154618283459	-0.273869450477
-0.154513225766	-0.273841956485
-0.154431677536	-0.273743510609
-0.154365852402	-0.273724608972
-0.15424288657	-0.273471774852
-0.154132929431	-0.273455299443
-0.154054667967	-0.273432418476
-0.153988470374	-0.273426956313
-0.153888590381	-0.273126614622
-0.153776172593	-0.273095847932
-0.153713566187	-0.273064928276
-0.153642613452	-0.273028054152
-0.153555186048	-0.272978214826
-0.153482159208	-0.272966728378
-0.153354102657	-0.272962498506
-0.153263478718	-0.27285950336
-0.153183169556	-0.272787029481
-0.153107864752	-0.272631690811
-0.153033308753	-0.272566903331
-0.152944896504	-0.27246445954
-0.152858935009	-0.272169843329
-0.152764578247	-0.272161299286
-0.152678024983	-0.271989013913
-0.152586631054	-0.271716933852
-0.152485399842	-0.271688220956
-0.152361257752	-0.271648046244
-0.15229057521	-0.271639422746
-0.152204191145	-0.271615624815
-0.152138537426	-0.271479242125
-0.152062897932	-0.27111754079
-0.151945791584	-0.27110136603
-0.151842520279	-0.271000040697
-0.151730703557	-0.270613496693
-0.151647185374	-0.270397992892
-0.151565490567	-0.270353062254
-0.151486895993	-0.270336312714
-0.15138762688	-0.270291272951
-0.151274920472	-0.270124803002
-0.15119580188	-0.270070690214
-0.151114165566	-0.269998829826
-0.151037653696	-0.269985191219
-0.150884339639	-0.269618811971
-0.150815220946	-0.269611704232
-0.150732912662	-0.269580377456
-0.150638913856	-0.269119232826
-0.150586038741	-0.269072973994
-0.150515374556	-0.268725669397
-0.150429720379	-0.268343767822
-0.150340796469	-0.268302573732
-0.150242589239	-0.268223142188

-0.150157152692	-0.268175913627
-0.150077615381	-0.268165972641
-0.149984338196	-0.267989037945
-0.14991056181	-0.267871133699
-0.14983865601	-0.267854647431
-0.149762981867	-0.267632328034
-0.149680590259	-0.267604939993
-0.149578375052	-0.267414534953
-0.149514200453	-0.267403090897
-0.149435854315	-0.267357397446
-0.149344541237	-0.267228187736
-0.149234085875	-0.267118484411
-0.149093076253	-0.266931950398
-0.148992811556	-0.266640914346
-0.14890460436	-0.266634009448
-0.148828900408	-0.266579802975
-0.148735547963	-0.266460637358
-0.148669346467	-0.266447209499
-0.148589580691	-0.266325565357
-0.148504813555	-0.26627745167
-0.148415476143	-0.266248686306
-0.148343992401	-0.266218208462
-0.148265335376	-0.266068371023
-0.148157611364	-0.266057361869
-0.148064220146	-0.265841745548
-0.147969996349	-0.265550832713
-0.147867398305	-0.265481878274
-0.147778648026	-0.265417512062
-0.147703580892	-0.265155708097
-0.14762640902	-0.265101498449
-0.147516543503	-0.265057561608
-0.147430983733	-0.26496221034
-0.147352741756	-0.264869601401
-0.147259493177	-0.264708917855
-0.14718034535	-0.264653201056
-0.147081041806	-0.264457981934
-0.146983656645	-0.264262441602
-0.146897570637	-0.264123674428
-0.14680776837	-0.263986428053
-0.146715316571	-0.263961193502
-0.146607058854	-0.263811835454
-0.146510122988	-0.263449373073
-0.146418888307	-0.263372659044
-0.146323789752	-0.263346747833
-0.146209479768	-0.262869907461
-0.146117686811	-0.262620900306
-0.146019081352	-0.262423989647
-0.145922844399	-0.262412381393
-0.145814804834	-0.262311630425
-0.145703123509	-0.262014595151
-0.145628396929	-0.261949609341
-0.1455250126	-0.261825473059
-0.145437436413	-0.261746189104
-0.145336814073	-0.261635763516

-0.145238500628	-0.261623235196
-0.145149697199	-0.261419187724
-0.145021176882	-0.261216445352
-0.144920271749	-0.261063422985
-0.144844042791	-0.260986509513
-0.144777364916	-0.260882760427
-0.144697959982	-0.260856378063
-0.144628605075	-0.260634157543
-0.144572101703	-0.260615231158
-0.144489512762	-0.260473513367
-0.144423901868	-0.260389549845
-0.14432932522	-0.260190950823
-0.14422381405	-0.259691364351
-0.144143934945	-0.259669140049
-0.144062423517	-0.259655779481
-0.14396173801	-0.259391559341
-0.143882955697	-0.259353678197
-0.143791416198	-0.25930800235
-0.143696770999	-0.259208518048
-0.143596473569	-0.259146069001
-0.143506985002	-0.258889901838
-0.143400146894	-0.258871030756
-0.143306732	-0.25877829247
-0.143208794703	-0.258771392417
-0.143099851347	-0.258484533526
-0.143036774251	-0.258383569153
-0.142975945825	-0.258308381802
-0.142892779875	-0.258205248998
-0.142794301018	-0.258101081878
-0.142693656364	-0.258062349151
-0.142630521062	-0.258045880761
-0.142540802109	-0.257899937814
-0.142443991351	-0.257632480298
-0.142330016711	-0.25749376451
-0.142224702478	-0.257480736496
-0.142113285438	-0.257204474284
-0.142030636964	-0.257159869646
-0.141956624386	-0.257039757322
-0.141876204999	-0.257027400394
-0.141788927607	-0.25700274892
-0.141714106437	-0.256925662991
-0.141633010882	-0.256896224606
-0.141592752767	-0.256752783934
-0.141465382316	-0.256471193843
-0.141361355917	-0.256339197462
-0.141254809828	-0.256009638094
-0.141184927728	-0.255970870864
-0.141080123224	-0.255883522951
-0.140999420799	-0.255852082576
-0.140936303244	-0.255822023968
-0.140818282286	-0.255687804696
-0.140734858022	-0.255490470488
-0.140658159044	-0.255449812531
-0.140554391047	-0.255439532748

-0.140483172814	-0.25542492725
-0.140385386548	-0.255089009444
-0.140273244824	-0.255086299159
-0.140182151911	-0.255014328128
-0.140085383106	-0.254467844575
-0.139986284045	-0.254462525284
-0.139910262685	-0.254457697593
-0.139778867013	-0.254106266231
-0.139653964626	-0.253922150911
-0.139551991729	-0.253668170783
-0.139474772931	-0.253639274037
-0.139351250109	-0.253558983861
-0.139270531032	-0.253438424177
-0.139183264202	-0.25342995807
-0.139087117908	-0.253079055278
-0.138995197482	-0.253032000384
-0.138928390278	-0.253027871683
-0.138800782585	-0.252974823132
-0.138736885998	-0.252938715722
-0.138672895679	-0.252897669863
-0.138595038434	-0.252690157021
-0.138503674169	-0.25267159629
-0.138424920565	-0.25265299698
-0.138361508506	-0.252637978259
-0.138250348296	-0.252533856546
-0.138153938595	-0.252508040693
-0.138078455171	-0.25248658052
-0.137967740219	-0.252464012341
-0.137872961346	-0.252189679616
-0.137761220152	-0.25205635498
-0.137685119473	-0.251937782985
-0.137600692403	-0.251794198208
-0.137498252831	-0.251496673588
-0.13739868392	-0.251494718256
-0.137305235406	-0.251300847304
-0.137171587362	-0.251125303647
-0.137096075245	-0.251040453131
-0.137018323185	-0.250849616854
-0.136888337886	-0.250847695223
-0.136759294766	-0.250422815396
-0.136681693939	-0.250316957221
-0.136578532766	-0.250260773466
-0.136485866387	-0.249778986819
-0.136418268718	-0.249707637754
-0.136329035392	-0.249574851247
-0.13625852453	-0.249422516673
-0.136208951838	-0.249404237777
-0.136105826342	-0.249327775671
-0.136046539451	-0.249314602164
-0.135964675173	-0.249177758902
-0.135856497275	-0.249024734424
-0.135748624033	-0.248442165162
-0.135652713536	-0.248434168846
-0.135571716063	-0.248304913604

-0.135488847654	-0.248163260363
-0.135410601646	-0.247885434963
-0.135331630591	-0.247778162425
-0.135268072417	-0.247745153891
-0.13517112224	-0.247495692085
-0.135081806523	-0.247460645192
-0.135015631986	-0.24745940183
-0.134946758032	-0.247458127162
-0.134845099642	-0.24715282383
-0.134736924475	-0.247111810799
-0.134663633267	-0.247039780892
-0.134580761932	-0.246968599326
-0.134474186346	-0.246919325526
-0.13436457515	-0.246911049321
-0.134293913307	-0.246791721187
-0.134248236856	-0.246767536999
-0.134148622694	-0.246500692614
-0.13403854269	-0.246408490868
-0.133958986821	-0.246375686343
-0.133861445972	-0.246369838281
-0.133787951622	-0.246270022478
-0.133639175238	-0.245683141748
-0.133571194967	-0.245557291989
-0.133471943132	-0.245543019009
-0.133388312103	-0.245522905775
-0.133317098278	-0.245492215286
-0.133258609629	-0.245483322723
-0.133176600854	-0.245399070159
-0.133116322814	-0.245375474295
-0.133024680971	-0.245354599878
-0.132885011205	-0.245017665718
-0.132824494461	-0.244943613021
-0.1327363864	-0.244918848405
-0.132627731581	-0.24490858877
-0.13256198265	-0.244883287158
-0.132494189037	-0.244813403604
-0.132434313187	-0.24475217155
-0.132325229431	-0.244375806091
-0.132176539268	-0.244198299057
-0.132068890513	-0.244176426967
-0.131999221858	-0.244169120349
-0.13192536603	-0.244148553943
-0.13187141045	-0.244143398655
-0.131796509166	-0.244133796446
-0.131729863936	-0.244113400404
-0.131646779973	-0.244046733038
-0.131518517486	-0.243788870555
-0.131414933568	-0.243639689838
-0.131362629911	-0.24360021869
-0.131298074289	-0.243583812966
-0.131228592201	-0.243541778487
-0.131128012561	-0.243281539196
-0.13104543184	-0.243038642618
-0.130944870276	-0.2428429966

-0.130831108203	-0.24233213855
-0.130697597262	-0.24200698348
-0.130578636218	-0.24197754948
-0.130478211033	-0.241742578866
-0.130369976338	-0.241396927209
-0.130274299016	-0.241122748716
-0.13019400959	-0.241054023614
-0.130121533344	-0.241023711588
-0.130048044785	-0.241021314967
-0.129979078341	-0.24092940736
-0.129862847611	-0.240922085148
-0.129782969451	-0.240739235821
-0.129680389198	-0.2407129945
-0.129580761308	-0.240303373675
-0.129502100308	-0.24029271102
-0.129437500713	-0.240208518196
-0.129367986117	-0.240108299755
-0.12928657195	-0.239887802153
-0.129191382858	-0.239880725437
-0.129077281115	-0.239696212483
-0.12895839612	-0.23954345642
-0.128869715129	-0.239520671618
-0.128783197868	-0.239334615534
-0.128718681822	-0.239276727713
-0.12863823231	-0.23913419285
-0.128573812059	-0.23911418579
-0.128472774749	-0.238938128194
-0.128412871562	-0.238909976432
-0.128319239389	-0.238898620907
-0.12823039147	-0.238842987185
-0.128159606394	-0.238765951244
-0.12806949099	-0.238744488034
-0.127981595372	-0.238567663148
-0.127878543956	-0.238532636487
-0.127807817537	-0.238532242995
-0.127705080649	-0.238401682256
-0.127584587803	-0.238244229453
-0.12750051322	-0.238240401704
-0.127418239308	-0.237893853838
-0.12726461734	-0.237878995546
-0.127138552017	-0.23722430682
-0.127066334439	-0.237188255202
-0.126971342005	-0.237186006002
-0.126902147493	-0.237012121278
-0.126807699818	-0.236995924149
-0.126714553234	-0.236953613817
-0.126645343366	-0.236950745104
-0.126552071383	-0.236871382103
-0.126475292793	-0.236768469584
-0.126386857137	-0.236740369896
-0.126270559215	-0.236473701974
-0.12618048875	-0.236458139064
-0.126116877502	-0.23642906569
-0.126003520782	-0.236350596853

-0.125857327955	-0.236242489084
-0.125724816935	-0.23616828309
-0.125654295845	-0.236166644776
-0.125546613553	-0.236099689671
-0.125458985892	-0.235754714382
-0.125381167808	-0.235720893065
-0.125298759538	-0.235464892324
-0.125195836857	-0.235377638645
-0.125112910397	-0.235273956316
-0.125029977024	-0.235186662166
-0.124939159174	-0.235014555063
-0.124848965161	-0.23499305688
-0.124750813962	-0.234685649397
-0.124652728964	-0.234670776774
-0.124539614867	-0.234474039445
-0.124466428074	-0.234269297959
-0.124400959681	-0.234223870816
-0.124338395166	-0.234208537808
-0.124242805431	-0.234198969798
-0.124165955252	-0.234198046061
-0.124091521119	-0.234145857071
-0.123987483736	-0.234108837115
-0.123880072414	-0.234108133206
-0.123766170882	-0.233901398825
-0.123674390594	-0.233877879751
-0.123591476543	-0.233843819951
-0.123516878837	-0.233839495293
-0.123437562167	-0.233714498658
-0.123337187476	-0.233542738514
-0.12323801293	-0.233302838726
-0.123169426103	-0.233275994092
-0.12305895095	-0.232991136976
-0.122940392223	-0.232728500666
-0.122863991188	-0.232652135052
-0.122752138743	-0.232647158013
-0.122661763348	-0.232507542436
-0.122596644633	-0.232486933935
-0.122498030943	-0.232357160628
-0.122390014431	-0.232136286468
-0.122320805068	-0.232114313705
-0.122238081506	-0.232024068381
-0.122169143556	-0.231985193929
-0.122068531512	-0.231721393159
-0.121959728107	-0.231461058158
-0.121881787109	-0.231331605605
-0.121815625506	-0.231302348616
-0.121726325399	-0.231291369122
-0.121657301037	-0.231248126552
-0.121584537757	-0.231223448794
-0.121484898608	-0.231169234769
-0.121408543786	-0.231117658785
-0.121295049149	-0.230986609562
-0.121174315281	-0.230919207547
-0.121065130128	-0.230799163699

-0.121006009085	-0.230713189064
-0.120915768379	-0.230309419725
-0.120827898328	-0.230278088097
-0.120696430586	-0.229396474403
-0.12059140276	-0.229314366618
-0.120508648414	-0.229223726517
-0.120428331623	-0.229207443242
-0.120322841485	-0.229159906734
-0.120245084404	-0.22907270014
-0.120148152672	-0.228870835168
-0.120082898395	-0.228795951053
-0.11999245526	-0.228732296621
-0.119916150388	-0.228695950226
-0.119830878547	-0.228668857025
-0.119746866314	-0.228425137041
-0.119667847728	-0.228354847016
-0.119601506736	-0.22833661987
-0.119528482697	-0.228333030252
-0.11941694771	-0.228216561034
-0.119334868636	-0.228102920906
-0.119239834776	-0.228091620255
-0.119165430283	-0.22806468578
-0.119072145411	-0.227842646505
-0.118981426358	-0.227818480307
-0.118888305254	-0.227805999784
-0.118791444206	-0.227727675034
-0.118693651811	-0.227156131857
-0.118604584865	-0.227113659303
-0.118507178752	-0.226820502503
-0.118430646518	-0.226684629032
-0.118355071672	-0.226569253727
-0.118286643155	-0.22656384976
-0.118238701143	-0.22652535721
-0.118152769102	-0.226327407726
-0.118064309689	-0.22629576493
-0.118006380546	-0.226275197344
-0.117920899333	-0.226271330072
-0.117835109117	-0.226109131909
-0.117740865508	-0.225901356118
-0.117671199463	-0.225851067447
-0.117584910237	-0.22574552497
-0.117512608573	-0.225565596529
-0.117439715408	-0.225329511388
-0.117328510068	-0.225212983867
-0.117230511866	-0.225045708856
-0.117130666577	-0.224971477536
-0.116999281275	-0.224864308225
-0.116931152742	-0.224812396513
-0.116868862705	-0.224706293445
-0.116818672575	-0.224615018946
-0.116759288629	-0.224468939816
-0.11668094376	-0.224445775522
-0.116577595618	-0.224394462787
-0.116496649914	-0.224371310725

-0.116398164965	-0.224325881909
-0.116289046924	-0.22405396599
-0.11619562883	-0.223956422864
-0.116105552596	-0.223950583728
-0.116023851879	-0.223883215572
-0.115948698424	-0.22382415294
-0.115823820952	-0.223794877053
-0.115723424216	-0.22353053582
-0.115635131829	-0.223493141534
-0.115545313441	-0.223430321623
-0.115440203692	-0.223427859827
-0.115317436245	-0.223277131028
-0.115193542514	-0.223237434998
-0.115108380316	-0.223230220646
-0.114993066266	-0.223035756381
-0.114932413838	-0.222941117166
-0.114849083725	-0.22292805475
-0.114756493814	-0.222874828942
-0.114665262485	-0.222857362366
-0.11458165485	-0.222851715714
-0.114494310725	-0.222707619253
-0.114430675143	-0.222653238714
-0.114347987283	-0.222556250666
-0.114242561116	-0.222489680108
-0.114187072493	-0.222482949215
-0.114088545195	-0.22248075652
-0.113997056595	-0.222434003606
-0.113904600319	-0.222419117483
-0.113827230721	-0.222378050621
-0.113741172966	-0.222361204231
-0.113671811501	-0.222342168977
-0.113600902283	-0.222288732686
-0.11349908412	-0.222142376929
-0.113383698434	-0.221963997466
-0.113292375619	-0.221688183702
-0.113219673392	-0.22164654796
-0.113118244814	-0.221614239941
-0.113054625307	-0.221497441745
-0.112963469091	-0.221395897964
-0.112862997787	-0.220936906338
-0.112705810729	-0.220420848098
-0.112631745342	-0.220181435803
-0.112541206903	-0.220102439002
-0.112464808186	-0.220019537445
-0.112350566361	-0.21983796564
-0.112228309283	-0.219794211099
-0.112149671957	-0.219732105172
-0.11204662137	-0.219293111039
-0.111976117357	-0.219287811048
-0.111862228091	-0.21922357758
-0.111758737722	-0.21919782997
-0.111687214748	-0.21915537848
-0.111590029793	-0.218993284228
-0.11150037715	-0.218787863258

-0.111416485808	-0.218647558742
-0.1113318737	-0.218595257109
-0.11122054589	-0.218425009447
-0.111134977165	-0.218415254729
-0.111016800516	-0.218184214969
-0.110918482043	-0.218098144595
-0.110813806586	-0.218049467079
-0.110702876772	-0.217807975201
-0.11063132207	-0.217704367375
-0.110562562157	-0.217702684633
-0.110447393518	-0.217563070703
-0.110365880969	-0.217348659929
-0.110259627322	-0.217262645678
-0.110161649286	-0.217080393644
-0.110069352076	-0.217013096665
-0.10998413197	-0.21691443569
-0.109881071527	-0.216865953252
-0.109758725297	-0.216435616205
-0.109647303118	-0.216380375181
-0.109577824958	-0.216335147303
-0.109477222455	-0.216110774887
-0.109408345509	-0.216087602335
-0.109345197714	-0.216021833782
-0.109210097496	-0.215817632676
-0.109142543334	-0.215730530727
-0.109051295285	-0.215703192792
-0.108971881374	-0.215530145145
-0.108869105459	-0.215446076969
-0.108799149555	-0.215433899992
-0.108723591116	-0.215415176788
-0.108638076542	-0.215363629597
-0.10854272645	-0.215221404242
-0.108440786623	-0.215157097131
-0.108341366008	-0.215050835867
-0.108257483519	-0.215031421946
-0.108165955488	-0.214904834357
-0.108076681999	-0.214799165144
-0.108007405977	-0.214625537261
-0.107918009568	-0.214577985399
-0.107807684075	-0.213995839259
-0.107694946384	-0.213957032286
-0.107604581474	-0.21395579107
-0.107539993425	-0.213893892454
-0.107406706278	-0.213837246534
-0.107346697797	-0.213835317642
-0.107272237274	-0.213695421631
-0.10716297385	-0.213645764776
-0.107075231808	-0.213595862159
-0.10696766692	-0.213227519955
-0.106876811705	-0.213212843294
-0.106788511619	-0.213199864926
-0.10672375506	-0.213151306697
-0.10660279361	-0.212927659721
-0.106538198129	-0.212753191954

-0.106470562209	-0.212659277134
-0.106368744233	-0.212428085172
-0.106256070052	-0.212381015482
-0.106166752212	-0.212375503116
-0.106086604868	-0.212264071017
-0.105993203542	-0.212103270056
-0.105913403254	-0.211876181099
-0.105856403509	-0.211874897834
-0.105773310902	-0.21172169692
-0.105710966769	-0.211533721671
-0.105631628471	-0.211399908445
-0.105538609362	-0.211330536836
-0.105439868577	-0.21132616347
-0.105338547791	-0.211069325128
-0.105245256734	-0.211040136532
-0.105155853151	-0.21103010766
-0.105090740782	-0.210961428688
-0.105019699985	-0.210871905361
-0.104925067055	-0.210834082296
-0.104864891469	-0.210813022962
-0.104790506911	-0.210739782424
-0.104721030224	-0.210643623509
-0.104630888631	-0.210348534668
-0.104529746279	-0.209956824672
-0.104453521981	-0.2098379655
-0.10438556461	-0.209783456525
-0.104305828977	-0.209434785231
-0.104198507895	-0.209227247735
-0.10411635807	-0.209203776361
-0.104051586193	-0.208854609049
-0.103975035797	-0.208835115351
-0.103836585454	-0.208241389831
-0.103731361933	-0.208087349151
-0.103650185098	-0.208048900202
-0.103533749073	-0.207587521506
-0.103453504864	-0.207494318256
-0.103350388438	-0.207297109708
-0.103255566313	-0.207167397256
-0.103172275498	-0.206943671811
-0.103087435565	-0.206882133695
-0.103001268291	-0.206880472877
-0.102919768978	-0.20684212374
-0.102833233315	-0.206820104265
-0.10277151418	-0.206812281276
-0.102699041706	-0.206707837058
-0.102586117827	-0.20657014839
-0.102508540266	-0.206507986242
-0.10242180745	-0.206408933941
-0.10233716813	-0.206379554132
-0.102240965894	-0.206317943263
-0.102193860265	-0.2062475187
-0.102118065281	-0.206218075999
-0.102012131414	-0.206203300083
-0.101943522549	-0.205996287807

-0.10186131569	-0.205914445799
-0.101773134269	-0.205851040826
-0.101697087397	-0.205799862391
-0.101603330362	-0.205788810893
-0.10150727711	-0.205635229018
-0.101426752992	-0.205567151348
-0.101354868982	-0.205435216418
-0.101267113485	-0.205327841647
-0.101188433249	-0.205254938859
-0.101109229137	-0.205219844069
-0.101029375775	-0.205132922604
-0.100946657963	-0.205058487327
-0.100835506791	-0.204978834482
-0.100773152191	-0.204954908444
-0.100681615658	-0.204804227655
-0.100578931991	-0.204712363955
-0.100507047968	-0.204582699528
-0.100379369346	-0.204369834347
-0.100306533462	-0.204206993761
-0.100239341287	-0.204112919596
-0.100173676114	-0.204088512303
-0.100093697213	-0.204059125579
-0.100014291193	-0.203954460271
-0.099931740792	-0.203840513435
-0.099834512581	-0.203825346877
-0.099741560948	-0.20376877621
-0.099657491297	-0.203612662852
-0.099572640668	-0.203606513543
-0.099476301152	-0.20344805357
-0.099376198344	-0.203245138164
-0.099297105132	-0.203132230355
-0.09922367729	-0.203131817747
-0.099130952199	-0.20304431684
-0.099050374081	-0.203020970505
-0.098959711365	-0.20280479544
-0.098883747557	-0.202721934452
-0.098769450926	-0.202492958996
-0.09868643124	-0.202114651309
-0.098640341589	-0.202072933892
-0.098541248711	-0.201993021679
-0.098429517249	-0.20174963994
-0.098317410368	-0.201466826652
-0.098224301171	-0.201219398704
-0.098165267683	-0.20120627268
-0.098081871664	-0.20117933237
-0.097952874668	-0.201075065674
-0.097858444834	-0.201074271907
-0.097764255668	-0.200781506594
-0.097671923423	-0.200780709033
-0.097591158854	-0.200483809666
-0.097495041926	-0.200403247483
-0.097395386782	-0.200364723315
-0.097276941654	-0.200254417468
-0.097186073755	-0.200169623369

-0.097124846263	-0.200091145666
-0.097015045704	-0.19990717194
-0.096927656582	-0.199830867041
-0.096857884696	-0.199779402088
-0.096735095861	-0.199534594937
-0.096671474868	-0.199416621156
-0.096551169612	-0.19940871972
-0.096479030835	-0.199399426361
-0.096409160529	-0.199373537221
-0.09633229237	-0.199305520356
-0.096239080547	-0.199297251893
-0.096115882137	-0.199107696103
-0.096040539209	-0.199094834424
-0.095970500232	-0.199013027175
-0.095887039436	-0.198953702846
-0.095789196997	-0.198861820368
-0.095724847278	-0.198837941204
-0.095652788831	-0.198837662326
-0.095565495682	-0.198779711464
-0.095484220154	-0.198677798371
-0.095412655072	-0.198611397834
-0.09533937769	-0.198606894875
-0.095257024988	-0.198551795981
-0.095195599902	-0.198499367105
-0.095102647124	-0.198472205561
-0.094985524259	-0.198069593437
-0.094885924939	-0.198026235361
-0.094823723796	-0.198023635242
-0.094730140334	-0.197630893799
-0.094607624751	-0.197369252176
-0.094543979501	-0.197327553282
-0.094473157328	-0.197258747905
-0.094375724238	-0.197189017007
-0.094292192226	-0.196873960945
-0.094224423671	-0.196866585961
-0.094123676756	-0.196563399568
-0.094021705267	-0.196399186183
-0.093944034503	-0.196396853291
-0.093864130431	-0.196382471627
-0.093788934712	-0.196195059985
-0.093718126889	-0.196136585295
-0.093621553631	-0.195982055503
-0.093543798962	-0.195898089674
-0.093468972928	-0.195885137753
-0.093357131856	-0.195728655035
-0.093245866244	-0.195723110804
-0.093119239991	-0.195704780803
-0.093031306193	-0.195615558923
-0.092963409992	-0.195586922412
-0.092875454496	-0.195399447967
-0.092793004524	-0.195357108746
-0.092709323942	-0.195324358349
-0.092608790475	-0.195314370519
-0.092530569596	-0.195201810419

-0.09244238827	-0.195036343307
-0.092323942221	-0.194970217253
-0.092246907603	-0.194920761965
-0.092123864248	-0.194812368897
-0.091995901165	-0.194767227251
-0.091909550034	-0.194745199101
-0.09181517299	-0.194479222101
-0.091742575535	-0.194458467804
-0.091645959508	-0.194433360063
-0.091544450576	-0.194381686836
-0.091426250061	-0.194169770896
-0.091329775682	-0.193876134036
-0.091234193271	-0.193802255286
-0.091148161614	-0.193494601192
-0.091038598242	-0.193464798858
-0.090946826698	-0.193286000887
-0.090866637412	-0.1932133789
-0.090783497188	-0.193109122034
-0.090684018075	-0.192916724041
-0.09055966008	-0.192833613736
-0.090480731895	-0.19274837572
-0.09040456494	-0.192678991987
-0.09031115357	-0.192259146928
-0.090231583664	-0.192242604796
-0.090170889279	-0.192225013052
-0.090124367204	-0.192208745479
-0.090049272714	-0.192180901937
-0.08994614393	-0.192156344068
-0.089886247402	-0.192126265503
-0.089805298355	-0.192012472385
-0.089711821381	-0.191984558136
-0.089590204281	-0.191730716457
-0.08950680773	-0.191512625195
-0.089435844186	-0.191471419877
-0.089354531237	-0.191456229636
-0.089268448107	-0.191319967279
-0.089195435696	-0.191262850645
-0.089139324469	-0.19124717494
-0.08907690524	-0.191209291571
-0.08898778233	-0.19099062369
-0.088881494087	-0.190912474185
-0.088766250889	-0.190821185072
-0.088636242416	-0.190415764922
-0.088508895268	-0.190379882716
-0.088435818307	-0.190114261511
-0.088356582181	-0.190106485665
-0.088260024789	-0.190086801699
-0.088180909522	-0.190058115243
-0.088097698683	-0.189986959253
-0.087990427707	-0.189959183623
-0.087915322144	-0.189950857159
-0.087837449585	-0.189878477491
-0.087760384445	-0.189734616443
-0.087690382829	-0.189685987541

-0.087604170032	-0.18959940331
-0.087534861426	-0.189547090477
-0.08742765524	-0.189363815561
-0.087353571305	-0.189315265573
-0.087258630196	-0.18909477147
-0.087199502535	-0.189005136946
-0.087109518679	-0.188931547464
-0.087013094203	-0.188647396288
-0.086914082684	-0.188505309159
-0.086837634838	-0.18841326729
-0.086758651002	-0.188363572684
-0.086671162722	-0.188290639146
-0.086598579153	-0.188250627102
-0.086502557502	-0.188015810228
-0.086420510123	-0.187628499282
-0.086315533845	-0.1876209115
-0.086241838667	-0.187569769822
-0.086148807132	-0.187529481296
-0.086074920641	-0.187364271979
-0.08596025686	-0.187051274149
-0.085853068997	-0.186914131917
-0.08576440156	-0.186862306389
-0.085694806916	-0.186846946294
-0.085602378955	-0.18658011292
-0.085525493232	-0.186520405277
-0.085450976536	-0.186353148478
-0.085386917333	-0.186352518281
-0.085312358147	-0.186326597303
-0.085229734903	-0.186257960004
-0.085156103324	-0.186239920141
-0.08506569059	-0.18606724078
-0.084965644291	-0.18601338193
-0.084873031671	-0.185955025147
-0.084794064094	-0.185863314641
-0.084687582077	-0.185599262911
-0.084589395122	-0.185465960276
-0.08448474924	-0.185364557058
-0.084415568075	-0.185236567343
-0.084300169545	-0.184896631247
-0.084224737604	-0.184742877356
-0.084145268656	-0.184707244484
-0.084049491786	-0.184706098567
-0.083975135787	-0.184699270337
-0.083905321824	-0.184608372841
-0.083841916795	-0.184570735576
-0.083724652566	-0.18421120726
-0.08364925054	-0.184145429481
-0.083585358711	-0.184105861902
-0.08350810455	-0.184059483262
-0.08343920948	-0.183996191828
-0.08335285806	-0.183808894335
-0.083243426486	-0.183772250956
-0.083156394833	-0.183691172904
-0.083061283617	-0.1835099236

-0.082956784288	-0.183492592273
-0.082871599107	-0.183465276249
-0.082760137306	-0.183259855499
-0.082647511728	-0.183129924693
-0.082562548025	-0.182959610972
-0.082462344503	-0.182866761181
-0.082393997526	-0.182853669994
-0.082324404016	-0.18284563327
-0.082226165501	-0.182804106037
-0.082128084334	-0.182740943573
-0.082043799498	-0.182653102368
-0.081945155419	-0.182615459023
-0.081857405398	-0.182607399466
-0.081800724413	-0.182539884616
-0.081739387816	-0.182445785488
-0.081643317709	-0.182280157874
-0.081554142149	-0.182245419174
-0.081432193784	-0.181892668856
-0.081345332224	-0.181847491933
-0.081286966092	-0.181755524521
-0.081211842072	-0.181744281008
-0.081103954263	-0.181650005952
-0.08101327099	-0.18155180154
-0.080919164111	-0.181347034632
-0.08084046124	-0.181217243223
-0.080734476275	-0.18116645964
-0.080663214459	-0.181142131971
-0.080576949358	-0.18096802017
-0.080497241973	-0.180967088088
-0.080445227638	-0.18082349907
-0.080356813936	-0.180761234899
-0.0802578791	-0.18075110865
-0.080158104669	-0.180388978768
-0.080055756472	-0.180307714234
-0.079945553713	-0.180306015625
-0.079850718243	-0.180136405518
-0.079764474838	-0.18008829499
-0.07966651956	-0.179958355961
-0.07955345736	-0.179835458843
-0.079449744503	-0.179647102248
-0.079358395909	-0.179177330107
-0.079284490383	-0.179155814198
-0.079180903984	-0.178731566711
-0.07912094496	-0.178662089622
-0.079051732912	-0.178533057713
-0.078945580101	-0.178463487568
-0.078851145264	-0.178082946938
-0.078777021247	-0.17805842662
-0.078678226582	-0.17804451058
-0.078593165167	-0.17779892914
-0.078509226002	-0.177795016658
-0.078424622424	-0.177785876924
-0.078328875498	-0.177739081705
-0.07823915224	-0.177717955776

-0.078150755117	-0.177671851986
-0.078075732204	-0.177546650667
-0.077992338212	-0.177446912676
-0.077916515588	-0.177437360191
-0.077823733922	-0.177237832909
-0.077703452667	-0.176800046934
-0.077627265088	-0.176739686411
-0.077540837252	-0.176726128209
-0.077444397945	-0.176690722942
-0.077347084828	-0.176527748374
-0.077248817772	-0.176444669893
-0.077172139387	-0.176410995395
-0.077105475716	-0.176406724377
-0.077030470109	-0.176385733855
-0.076947649896	-0.17630914664
-0.076855870809	-0.176183434863
-0.076798607153	-0.176158743035
-0.07672188158	-0.176062629649
-0.076647339901	-0.176030885701
-0.076545223366	-0.175782103033
-0.076438297052	-0.17531094663
-0.076386497293	-0.175308899277
-0.07630356462	-0.17506717317
-0.076210135942	-0.174787205385
-0.076153540109	-0.174713811339
-0.07607905454	-0.174656607439
-0.075978985152	-0.174572815982
-0.075920475456	-0.174556325172
-0.075825754805	-0.174519146616
-0.075750299786	-0.174353992288
-0.075686302416	-0.174334748407
-0.075574905824	-0.174329173732
-0.075491575088	-0.174130888839
-0.075405373925	-0.17406726165
-0.075323540442	-0.173864069118
-0.075216822885	-0.173691910076
-0.075099868679	-0.173149977373
-0.075031454124	-0.172882377744
-0.074955496437	-0.172827363101
-0.074874093744	-0.172824399173
-0.074810439622	-0.172800293914
-0.074706854755	-0.172616344905
-0.07458392456	-0.172539794773
-0.074494163458	-0.172472016873
-0.07440670785	-0.172463172932
-0.074335300937	-0.172316653345
-0.074263682333	-0.172302642246
-0.074180956203	-0.172027018268
-0.074109139909	-0.171964970677
-0.074015924434	-0.171891412072
-0.073923213965	-0.171843352853
-0.073837650282	-0.171740949198
-0.073750894704	-0.171690748675
-0.073650349663	-0.171375201905

-0.073561008871	-0.171134314598
-0.07345590079	-0.171057922901
-0.073380857484	-0.171049942531
-0.073294088687	-0.171039384295
-0.073194901463	-0.170894622056
-0.073123627125	-0.170861555232
-0.07303660146	-0.170798095583
-0.072936346812	-0.170617049454
-0.072854555577	-0.170539968653
-0.072759393622	-0.170409236085
-0.072700741603	-0.17032962537
-0.072608269124	-0.170170892131
-0.072513810386	-0.170161082539
-0.072433899833	-0.170098707832
-0.072372994899	-0.17007344556
-0.072299155131	-0.170043207302
-0.072215184658	-0.170025546742
-0.072139933577	-0.169923568188
-0.072062786154	-0.169751491436
-0.071962978182	-0.169674001761
-0.071904971604	-0.169639515358
-0.071822826479	-0.169589224103
-0.071688549866	-0.169022464117
-0.071597854932	-0.168972748032
-0.071525808103	-0.168781421035
-0.071418946382	-0.168775850774
-0.071340405739	-0.168771620061
-0.071271095881	-0.168689435496
-0.071194180015	-0.168577752009
-0.071130632638	-0.168498874692
-0.071045046775	-0.168283545843
-0.070974227682	-0.168193740778
-0.07089757938	-0.168075239204
-0.070821426164	-0.167840615494
-0.0707408969	-0.167554220375
-0.070678459887	-0.167541206925
-0.070589048835	-0.167480725958
-0.070522090598	-0.167399402473
-0.070459220656	-0.167291846388
-0.070354574382	-0.167232154877
-0.070214488487	-0.167025494317
-0.070125736863	-0.166814961997
-0.070057751219	-0.166780978241
-0.069977199135	-0.166744374974
-0.069905099577	-0.166637797784
-0.069817745742	-0.166565306864
-0.0697352474	-0.166559875581
-0.069646855059	-0.166475340679
-0.06955247956	-0.166417685762
-0.069444844598	-0.165925748309
-0.069368225065	-0.165878024985
-0.069269232919	-0.16581755988
-0.069183632989	-0.165581567741
-0.069086517	-0.165541702541

-0.0690020361	-0.165426915542
-0.068912915417	-0.165240954332
-0.068836289457	-0.165182572955
-0.068756813112	-0.16517842567
-0.06863700112	-0.165005887782
-0.068572593656	-0.164986468044
-0.06848158005	-0.164866790486
-0.068384555865	-0.164716517972
-0.06829305732	-0.164628346456
-0.068211818989	-0.164454447562
-0.068155094528	-0.164448169424
-0.068083566241	-0.164222923949
-0.067984526574	-0.164165894176
-0.067886435143	-0.164073713091
-0.06778147938	-0.163970280742
-0.067691320766	-0.163801817407
-0.067593969837	-0.163768940901
-0.067515035524	-0.163732482026
-0.067399173285	-0.163443518099
-0.067278123133	-0.163195476309
-0.067212917766	-0.163020110801
-0.067115780848	-0.162901197382
-0.067045231015	-0.162865270083
-0.066930763205	-0.162807291741
-0.066812891284	-0.162655905266
-0.06672173447	-0.162600511557
-0.066642087579	-0.162331375697
-0.066545048167	-0.162318252366
-0.066464302621	-0.162286402414
-0.066361222893	-0.162225421317
-0.066292812034	-0.162189360126
-0.066219164272	-0.162084539624
-0.066109390216	-0.162060348304
-0.066004998028	-0.161971757526
-0.065894364707	-0.161911835785
-0.06579440933	-0.161787266203
-0.065686974162	-0.161774147281
-0.065609504721	-0.161613302848
-0.06553959616	-0.161610702846
-0.065461231867	-0.161535100378
-0.065353052541	-0.161475641467
-0.065271792008	-0.161288878099
-0.065178363065	-0.16123112488
-0.065072182288	-0.161024786513
-0.064995965041	-0.160968997974
-0.064885469238	-0.160748839998
-0.064799289916	-0.160464828328
-0.064696330036	-0.160362231965
-0.064617878504	-0.160331269975
-0.064529763763	-0.160035030127
-0.064446488182	-0.16002034455
-0.064368306803	-0.159955617914
-0.064300347637	-0.159924397511
-0.064198762011	-0.159848186075

-0.064074214337	-0.159843970101
-0.063956384198	-0.159828544066
-0.063855064323	-0.159744184456
-0.063772821231	-0.159683541636
-0.063685503511	-0.159307077534
-0.063609003528	-0.159297784176
-0.063530166605	-0.159105618522
-0.063443437737	-0.159058167536
-0.063358875937	-0.158973531177
-0.063253001982	-0.158886801872
-0.063132990631	-0.158700650199
-0.063061973765	-0.15854999627
-0.062969031589	-0.158451618658
-0.062884265491	-0.158381562618
-0.06279706138	-0.158380820846
-0.062723319432	-0.158361210874
-0.062641914705	-0.158307289438
-0.062546745925	-0.158263069027
-0.062479870189	-0.158217537932
-0.062388607688	-0.158047245358
-0.062286364362	-0.157890692396
-0.062197944976	-0.157883914279
-0.062141015419	-0.157861219125
-0.06203594136	-0.157694696413
-0.06192894328	-0.157680207699
-0.06181907927	-0.157594193277
-0.061745040013	-0.157579165506
-0.061668520441	-0.157512965008
-0.061584526386	-0.157458440544
-0.061489522339	-0.157410693883
-0.061383488229	-0.157316917711
-0.061303486438	-0.156996241679
-0.061178840431	-0.156455721583
-0.061094388931	-0.15644034587
-0.061014330651	-0.156439747614
-0.060944510013	-0.156429897497
-0.060878783267	-0.156321314048
-0.060762835086	-0.156216389593
-0.060661337177	-0.155969545956
-0.060571816445	-0.155917214533
-0.060464178583	-0.155747503881
-0.060384440987	-0.155745974553
-0.060307404206	-0.155706045027
-0.060236921505	-0.155704395623
-0.060173532804	-0.155626304152
-0.060091024973	-0.155547442269
-0.060006415482	-0.155473532815
-0.059923036801	-0.155362415101
-0.059838543726	-0.155283984431
-0.059761678733	-0.155269060317
-0.059669628316	-0.155120484376
-0.059604057066	-0.155060562947
-0.05950728785	-0.1547985602
-0.059431729957	-0.154753266067

-0.059361442576	-0.154656827841
-0.059292676262	-0.154643649387
-0.059211296686	-0.154615762694
-0.059133534931	-0.154427443341
-0.059036853923	-0.154344614852
-0.058938907921	-0.154095948808
-0.058842878912	-0.153707518389
-0.058751326457	-0.153676437357
-0.058664568762	-0.153509444207
-0.058584439831	-0.153442780699
-0.058475411184	-0.153414730357
-0.058358725198	-0.153233267891
-0.058224058379	-0.153197033096
-0.058140732626	-0.153112916795
-0.058065326257	-0.152919950627
-0.057965336319	-0.152780637834
-0.057868103777	-0.15246954142
-0.057778412401	-0.152422374679
-0.057687371456	-0.152346482739
-0.057587017804	-0.152141865013
-0.05750412336	-0.152015920075
-0.05740799076	-0.151956183286
-0.057335287393	-0.151845039058
-0.05722643397	-0.151821735027
-0.057165068149	-0.15178091477
-0.057101288589	-0.151752644175
-0.057012936946	-0.151603972388
-0.05695049786	-0.151520758797
-0.056815372493	-0.151009144053
-0.056728721245	-0.150847513474
-0.056650339342	-0.150761043558
-0.056537050367	-0.150704971683
-0.056437739384	-0.150632315847
-0.056337209253	-0.15047486032
-0.056249126133	-0.150405843747
-0.056164937508	-0.150316566678
-0.056055404661	-0.1501790126
-0.055966372102	-0.150036266562
-0.055882333032	-0.149941926157
-0.055778577164	-0.149500846619
-0.055720253415	-0.149469426215
-0.055635485999	-0.149338983451
-0.055538633367	-0.149317597
-0.055423089011	-0.149309073648
-0.055347132059	-0.149207379016
-0.055210349986	-0.149026619808
-0.055120696349	-0.148971869503
-0.055065472329	-0.14893313189
-0.054999814467	-0.148912912174
-0.054905710052	-0.14878319505
-0.054792483512	-0.148743543196
-0.054690425851	-0.14848065289
-0.054617668297	-0.148355259175
-0.05453120431	-0.148218086067

-0.054440168674	-0.148200758422
-0.054331985765	-0.148121129481
-0.054239472972	-0.148069121059
-0.054161515253	-0.148040192567
-0.054047341717	-0.147747680832
-0.05395229368	-0.147733953384
-0.053875374262	-0.147561065227
-0.053764481388	-0.147518557551
-0.053669861413	-0.147479815485
-0.053578154764	-0.147359424493
-0.053487113046	-0.147142597653
-0.053397262901	-0.147018309952
-0.053326339383	-0.146948124435
-0.053207680938	-0.146893188609
-0.053118338751	-0.146779767167
-0.053012418801	-0.146652970391
-0.052938949058	-0.146645876253
-0.052867788335	-0.14648800773
-0.052793497372	-0.14645743531
-0.052717041176	-0.146313852414
-0.052602785932	-0.146269256516
-0.052526543238	-0.146169310832
-0.05245555539	-0.146141057883
-0.05237848322	-0.146122595701
-0.052316517147	-0.146112612997
-0.052243859925	-0.146087830658
-0.052136713325	-0.145943577061
-0.052037462237	-0.145703593269
-0.051954959063	-0.145484625362
-0.051848489267	-0.145434500837
-0.051744318346	-0.145357977988
-0.051669251703	-0.145207704807
-0.051565530222	-0.145174840652
-0.05145244173	-0.14491297116
-0.051360554508	-0.144827608212
-0.051278141413	-0.14477079296
-0.051182979924	-0.144689456631
-0.051088580142	-0.144575072364
-0.051015649606	-0.144548679741
-0.050929607186	-0.144433943181
-0.050817181949	-0.144423772347
-0.05073368049	-0.144363272725
-0.05066182309	-0.144360083223
-0.050578579051	-0.144265762903
-0.050509316108	-0.144131724545
-0.050417403107	-0.144043896898
-0.050333176289	-0.143663922711
-0.050227587501	-0.143422414453
-0.05011193284	-0.143311509103
-0.050035361501	-0.1432372745
-0.049954754653	-0.143225921375
-0.049833472083	-0.142594740892
-0.049731839816	-0.142555915886
-0.04966931364	-0.142525333714

-0.04958856885	-0.14252182502
-0.04950960597	-0.142516396031
-0.049407416476	-0.142371252538
-0.049332091716	-0.142241184807
-0.049237678261	-0.142217267821
-0.049148860456	-0.142207405549
-0.049061540631	-0.142004004166
-0.048974936658	-0.141946500623
-0.048877671532	-0.141905499029
-0.048805448847	-0.141905280622
-0.048730155075	-0.141872546785
-0.048634651005	-0.141812392112
-0.048525970649	-0.141810805036
-0.048416882495	-0.141724161066
-0.048322868639	-0.141621234078
-0.048239602122	-0.141386674556
-0.048167202611	-0.141360850171
-0.048075076445	-0.141331776867
-0.047983182655	-0.141325696585
-0.047903483251	-0.141226153449
-0.047779061895	-0.141068087259
-0.047668879342	-0.140898148918
-0.047579488902	-0.140793018168
-0.047505824416	-0.140714463213
-0.047421365012	-0.140594516129
-0.04731300957	-0.140339370883
-0.047185084594	-0.140317882311
-0.047078915504	-0.140236910676
-0.046987477931	-0.140187920422
-0.046909489372	-0.140116179582
-0.046776705985	-0.139774512242
-0.046706541835	-0.139734506078
-0.046607725844	-0.139606420458
-0.046510770145	-0.139522796318
-0.046428243304	-0.139439653608
-0.046377774162	-0.139325638796
-0.046302269805	-0.139256213645
-0.046223540261	-0.139249530531
-0.046102999752	-0.139202020087
-0.046010829374	-0.138900606352
-0.045938679738	-0.138688392453
-0.045868416944	-0.138608977574
-0.045776334731	-0.138506137159
-0.045694071402	-0.138418370137
-0.045598608004	-0.138300477471
-0.045512139531	-0.138082372721
-0.045434445686	-0.138057576031
-0.045343864751	-0.1380370639
-0.045244184613	-0.137948942037
-0.04513904078	-0.137948393164
-0.04502676027	-0.137891250363
-0.044959148197	-0.137833978482
-0.044875222522	-0.137575304661
-0.044768899365	-0.1374880234

-0.044675028611	-0.137271411969
-0.044607150912	-0.137217192131
-0.044526073517	-0.137107242956
-0.044437667279	-0.137013589518
-0.044378071054	-0.13689292299
-0.044276437128	-0.136878152063
-0.044181880348	-0.136831700767
-0.044089927642	-0.136633998386
-0.044010148332	-0.136608795587
-0.043928367495	-0.136584902894
-0.043837576243	-0.136525626915
-0.043750703589	-0.136370614092
-0.043626485291	-0.136356740003
-0.043525583093	-0.136168907423
-0.043434881166	-0.136135248948
-0.043296172393	-0.135564938753
-0.043204013233	-0.135463899756
-0.043098123093	-0.135460697492
-0.043036406393	-0.135401022555
-0.042961677411	-0.135352023974
-0.042826592411	-0.135346347435
-0.042745638858	-0.135300473491
-0.042650577936	-0.135054425219
-0.04255809299	-0.134846362189
-0.042473770813	-0.134696040884
-0.042396741828	-0.134513210125
-0.04225527175	-0.13444750115
-0.042129133549	-0.134337780887
-0.042022094725	-0.134117510303
-0.041929937025	-0.134031063247
-0.041859449543	-0.13388618206
-0.041757559848	-0.133666220023
-0.04165738762	-0.133600753732
-0.041573782471	-0.133586046738
-0.041474667626	-0.133472980777
-0.041380606055	-0.133236535777
-0.041318634212	-0.133228680943
-0.041250977469	-0.133115744887
-0.041175213664	-0.132957256545
-0.041077690686	-0.132769667806
-0.040967265146	-0.13248862456
-0.040889710778	-0.132463451622
-0.040780771842	-0.132185254934
-0.040689189273	-0.131894898759
-0.04057103313	-0.131886473848
-0.04051029233	-0.131857006423
-0.040413901677	-0.131616488737
-0.040331402676	-0.131321750849
-0.040255824284	-0.131272095361
-0.040144695401	-0.131105368467
-0.040082161898	-0.1310669375
-0.039990087362	-0.130916128293
-0.039915493134	-0.130884236494
-0.039827379788	-0.130833901198

-0.039740027783	-0.130802904551
-0.039699264641	-0.130739979385
-0.039637587611	-0.130709082111
-0.039562472165	-0.130707652592
-0.039483550616	-0.130675596364
-0.039385134555	-0.130599668385
-0.039294955898	-0.130255161901
-0.039206916434	-0.129971347321
-0.039129324263	-0.129951745428
-0.039038708199	-0.129869400495
-0.038939279679	-0.129776204495
-0.038866882081	-0.129727065425
-0.038752088373	-0.129492781234
-0.03861498931	-0.128899814154
-0.038476506129	-0.128450396846
-0.038421795555	-0.128433659628
-0.038329752122	-0.128399565322
-0.038241146602	-0.128291458157
-0.038173938146	-0.128107092014
-0.038110889274	-0.128041847537
-0.038046589434	-0.127977979226
-0.03793147115	-0.127957243926
-0.03786058849	-0.127882055792
-0.037791205455	-0.127680820114
-0.037727979528	-0.127650321979
-0.03762495604	-0.127465723915
-0.037541236938	-0.127376493944
-0.037428498713	-0.127182358331
-0.037367999159	-0.127177459526
-0.037284897989	-0.127016146272
-0.037176578481	-0.126854358205
-0.037065057129	-0.126758174185
-0.037001890312	-0.126573632587
-0.036941917382	-0.12653547763
-0.036819018103	-0.126202904722
-0.036734761417	-0.126071690189
-0.036646061028	-0.126006519957
-0.03656318351	-0.125984422795
-0.036473809715	-0.125940011515
-0.036369375068	-0.125911773393
-0.036291430706	-0.125651293372
-0.036220904665	-0.125526522302
-0.036130306911	-0.125518210046
-0.036042099568	-0.125428861141
-0.035934881899	-0.125133785077
-0.035848433144	-0.125130947553
-0.035746821691	-0.125097609815
-0.035655457228	-0.125085713807
-0.035563488026	-0.12502930046
-0.035483529928	-0.124871927626
-0.035374340524	-0.124439794824
-0.035278200804	-0.124427841343
-0.035211047143	-0.124393041519
-0.035127553302	-0.12434682428

-0.035044493446	-0.124300477948
-0.034965859675	-0.123976830378
-0.03489670867	-0.123958780701
-0.034808285191	-0.123818907795
-0.034695550592	-0.123643622816
-0.034589217053	-0.123241330301
-0.034501231595	-0.123231820489
-0.034397373363	-0.123227637746
-0.034298557252	-0.123149886364
-0.034255513525	-0.123106843932
-0.034137914161	-0.12303915638
-0.03404051149	-0.122970729545
-0.033958238673	-0.122856104567
-0.033876070143	-0.122671294742
-0.033813894201	-0.122521945899
-0.033732076107	-0.12243556938
-0.033631479891	-0.122405244458
-0.033531724878	-0.122255412247
-0.033391021182	-0.121658633609
-0.033243562069	-0.120879411626
-0.033167069184	-0.120874100588
-0.033067414881	-0.120637711138
-0.032992834151	-0.120449344709
-0.032884534122	-0.120028621699
-0.032798370668	-0.12001801573
-0.032734141718	-0.119957691788
-0.032651258018	-0.119841121237
-0.032585127518	-0.11983013668
-0.032456864222	-0.119796153401
-0.032368692306	-0.119510283802
-0.03229758927	-0.119501518888
-0.032183356513	-0.119471003347
-0.032087838311	-0.119295703214
-0.032015991792	-0.119030189198
-0.031964276112	-0.11900277551
-0.03189472957	-0.1189682507
-0.031806422863	-0.118753728341
-0.031706641739	-0.118511618255
-0.031624109451	-0.118297726127
-0.031529219543	-0.118085035419
-0.031441270784	-0.118080377609
-0.031338050214	-0.117860988147
-0.031268297403	-0.117849620644
-0.03119240478	-0.117808804042
-0.031127395543	-0.117748847291
-0.031042108432	-0.117736553399
-0.030961754237	-0.117678135323
-0.030878875878	-0.117670380567
-0.030813321703	-0.117578443621
-0.030718157267	-0.117265970345
-0.030649762133	-0.11724409005
-0.030515050884	-0.116974823757
-0.030416781236	-0.116963807466
-0.030308749268	-0.116955109651

-0.030241377563	-0.116846132807
-0.030148996227	-0.116659118358
-0.030064965015	-0.116554473954
-0.029995052231	-0.116503395478
-0.02993179547	-0.116373603553
-0.029811160876	-0.11627123088
-0.029749032994	-0.116215113909
-0.02968056426	-0.116148250343
-0.029605675758	-0.115991028147
-0.029520944026	-0.115903309113
-0.029464293944	-0.115902133325
-0.029374647109	-0.115838883123
-0.029282338309	-0.11579734781
-0.029218036444	-0.115786189527
-0.029161193758	-0.115683554323
-0.029056354524	-0.115457293388
-0.028975358851	-0.115369281266
-0.028884223043	-0.115205631088
-0.028813626761	-0.115174500341
-0.028731339932	-0.11490421114
-0.028656400844	-0.114745756934
-0.028566509488	-0.114631770864
-0.028500233196	-0.114564277327
-0.02841058755	-0.114540078907
-0.028294361386	-0.114222518554
-0.028228786817	-0.114175944953
-0.028122435666	-0.114046687624
-0.028024869381	-0.114029745192
-0.027942451623	-0.113980911229
-0.027862626805	-0.113784090756
-0.027780615373	-0.113720436289
-0.027696155975	-0.113708366188
-0.027608029479	-0.113512323849
-0.027494882131	-0.11345055564
-0.027411713323	-0.113426245542
-0.027327099062	-0.113083658982
-0.027221301833	-0.11280357658
-0.027118159395	-0.112714478623
-0.027046082792	-0.112697616753
-0.026952289843	-0.112508251611
-0.02687046924	-0.112325554868
-0.026760969264	-0.112109119215
-0.026672382404	-0.112045551659
-0.026594082552	-0.112010270441
-0.026521595595	-0.111830484017
-0.026427284022	-0.111668935383
-0.026336911497	-0.111664220821
-0.026254797052	-0.111619884667
-0.026178964022	-0.111382223071
-0.026097873109	-0.111331389093
-0.02597690063	-0.11126139666
-0.025899100555	-0.11114115962
-0.025837966776	-0.110951222416
-0.025751851085	-0.11081711295

-0.025673171656	-0.110773991003
-0.025614454079	-0.110725556511
-0.025547320475	-0.110564653751
-0.025458701384	-0.110452591098
-0.025381503811	-0.110361334728
-0.025286035393	-0.110235500198
-0.025248957574	-0.110188784368
-0.025158088138	-0.110124592435
-0.025078150589	-0.109956281781
-0.024997997026	-0.109938950101
-0.024924958235	-0.109862993107
-0.02484283245	-0.109727047589
-0.024749287943	-0.109635371614
-0.024672166203	-0.109520784622
-0.024599019555	-0.109505878021
-0.024512941948	-0.10948497989
-0.024441000356	-0.109417093414
-0.024352013766	-0.109386066486
-0.024264474828	-0.109347635857
-0.024180942961	-0.109083110573
-0.024096529778	-0.109012889894
-0.023982028002	-0.108655249966
-0.023904648578	-0.108558263762
-0.023802983465	-0.108388044051
-0.023725455857	-0.108298346217
-0.023651965762	-0.108269208128
-0.023556259983	-0.107930201736
-0.023489610502	-0.107895987134
-0.023393305753	-0.107853192283
-0.023296003009	-0.107734616188
-0.023221606201	-0.107720509272
-0.023137296306	-0.107689897914
-0.023056934765	-0.1076596399
-0.022941148985	-0.107186642755
-0.022830215458	-0.107180260037
-0.022751742066	-0.107166171807
-0.022658746208	-0.10693656009
-0.02255929478	-0.106887006564
-0.022480069628	-0.10675072738
-0.02242176853	-0.106665592001
-0.022333481291	-0.106559992523
-0.022266575717	-0.106548959578
-0.022177136204	-0.106383059696
-0.022091732315	-0.106317023027
-0.021969528193	-0.106314081683
-0.021902733654	-0.106310866624
-0.021791495856	-0.106282845307
-0.021662511538	-0.105754831371
-0.02158247865	-0.105754568684
-0.021519621247	-0.105673794631
-0.021455379735	-0.105673263215
-0.021351527083	-0.105416409165
-0.021274378417	-0.105414245618
-0.021195634153	-0.105375014925

-0.021127857094	-0.105237161031
-0.021038551752	-0.105168662158
-0.020962373866	-0.105069668169
-0.020908432767	-0.10498320988
-0.02082538088	-0.104925697856
-0.020759023987	-0.104818861157
-0.020670109043	-0.104817609556
-0.020572250397	-0.104805676183
-0.020487134632	-0.104652654769
-0.020413040656	-0.104471450348
-0.020310737191	-0.104355824755
-0.020229322294	-0.103922855676
-0.020154547334	-0.103884689465
-0.020032942683	-0.103722120624
-0.019965958004	-0.103701736851
-0.019900558554	-0.103655611523
-0.019829164469	-0.103509744141
-0.019766342801	-0.103460125979
-0.019712071952	-0.103459777503
-0.019633520338	-0.103228334408
-0.019564864629	-0.103212299329
-0.019481790939	-0.103161266412
-0.019373706333	-0.102974459058
-0.01930806134	-0.102912455969
-0.019199709097	-0.102770655586
-0.019126127869	-0.102714067618
-0.019039639933	-0.102702225716
-0.018937200813	-0.10268378211
-0.018870462327	-0.102662001325
-0.01877266982	-0.102381649103
-0.018694560191	-0.10217713453
-0.018630625781	-0.101996953083
-0.018521701238	-0.101901868947
-0.018445892269	-0.10173881887
-0.018379897061	-0.10172759158
-0.018303643799	-0.101647403441
-0.018186960678	-0.101456123561
-0.018086831296	-0.10141875108
-0.017999277767	-0.101390782205
-0.017950908039	-0.101319800531
-0.017870430646	-0.101278464802
-0.017777836149	-0.10120085045
-0.017693706044	-0.101080300204
-0.017612262432	-0.100686054098
-0.017534312294	-0.100684236869
-0.017455030491	-0.100556936437
-0.017342972093	-0.100518044298
-0.01727968069	-0.100404328308
-0.017205703616	-0.10020676415
-0.017093315474	-0.100071323264
-0.017034575739	-0.099950326964
-0.016951037223	-0.09994496846
-0.016868723652	-0.099855759363
-0.016780301546	-0.099785777352

-0.016705777619	-0.099649242963
-0.016634190903	-0.099589275228
-0.016557683286	-0.099559000454
-0.016459215726	-0.09913459473
-0.016410380385	-0.099015996032
-0.016313212304	-0.099008393489
-0.016255066509	-0.098967331219
-0.016154071374	-0.098843206959
-0.016072346825	-0.098677664365
-0.015988898327	-0.098555020071
-0.015913578144	-0.09852992246
-0.015817500487	-0.098194095987
-0.015747675494	-0.098010586032
-0.01567056853	-0.097875817939
-0.015586538085	-0.09775577375
-0.015507307017	-0.097654648463
-0.01541178392	-0.097571910477
-0.015325687712	-0.097471321959
-0.015218843131	-0.097261099713
-0.015096391165	-0.097256332236
-0.015003245916	-0.097172174603
-0.014919109642	-0.097146603181
-0.014878147854	-0.097098169301
-0.014750628946	-0.097066439482
-0.0146180885	-0.096718883117
-0.014484074238	-0.096679431455
-0.014418439897	-0.096617686604
-0.014354850972	-0.096615865926
-0.014278028252	-0.096544330776
-0.01418845646	-0.096425391323
-0.014119796962	-0.096327362528
-0.014016037198	-0.096297592128
-0.013932015569	-0.096255647119
-0.013856339417	-0.096183174068
-0.013779431037	-0.096181904362
-0.013703790921	-0.096175281292
-0.013622348825	-0.096168597632
-0.0134999853	-0.096079947349
-0.013417367527	-0.096010197179
-0.013337323545	-0.095949691587
-0.013242844962	-0.095393767015
-0.013129739504	-0.095259810718
-0.013022545786	-0.095102902332
-0.012912672692	-0.094873765445
-0.012840042814	-0.094868280763
-0.012759456487	-0.094678869292
-0.012674383678	-0.09466133784
-0.012584560408	-0.094275784362
-0.012508402172	-0.094112933846
-0.012430859073	-0.09394219205
-0.012334362186	-0.09375462671
-0.012237434006	-0.093683635173
-0.012161099111	-0.093651872327
-0.012083221652	-0.093495105997

-0.012019081326	-0.093370953572
-0.011941103564	-0.093277111712
-0.011865701269	-0.09322951215
-0.011773430673	-0.09319012118
-0.011687511123	-0.09313805463
-0.01157607871	-0.093046423032
-0.011457949045	-0.092985320966
-0.011367801696	-0.092915711324
-0.011283295928	-0.092858682193
-0.011195438821	-0.092852558902
-0.011115270271	-0.092778973755
-0.011041214163	-0.092773831284
-0.010956905098	-0.092719469728
-0.010856062987	-0.09269164749
-0.010768113482	-0.092447381214
-0.010680065015	-0.092239134495
-0.010607830571	-0.092223716869
-0.010533658506	-0.09222016888
-0.010428385879	-0.092171844381
-0.010309385316	-0.092131406063
-0.01022367198	-0.092112616927
-0.010130740438	-0.091891352696
-0.010070198398	-0.09182642099
-0.009966765985	-0.091665944575
-0.00988952571	-0.09164708624
-0.009822811107	-0.091604232536
-0.009751502562	-0.091510292067
-0.009652716992	-0.091166844127
-0.009561538965	-0.090903613739
-0.009490736149	-0.090892989049
-0.009406629818	-0.0903298775
-0.009325283286	-0.090305673065
-0.00923054366	-0.090246348405
-0.009158721342	-0.090013819016
-0.009075877142	-0.089763147919
-0.009006171085	-0.089725513358
-0.008926778356	-0.089686767671
-0.008822498034	-0.089469083284
-0.008710793121	-0.089319619435
-0.008598516442	-0.089263751297
-0.008514459915	-0.089198464882
-0.008442950208	-0.089193054897
-0.008340140274	-0.088973700628
-0.008222142157	-0.08860560303
-0.008155409361	-0.088596693733
-0.008069318578	-0.088376291169
-0.007986840158	-0.088244289892
-0.007905271841	-0.088198498265
-0.007810552409	-0.087978055281
-0.007728016986	-0.087647102313
-0.00764132209	-0.08764138169
-0.007569569431	-0.087605176058
-0.00748274111	-0.087505981921
-0.007389974366	-0.08709216136

-0.007302850746	-0.087081396486
-0.007230155358	-0.086995484663
-0.007144398726	-0.086932131166
-0.007050667237	-0.086877150031
-0.006995241872	-0.08680406465
-0.00686556354	-0.086797496573
-0.006767479439	-0.086675122717
-0.006696136733	-0.086652925771
-0.006584220532	-0.086450498812
-0.00648812168	-0.086383634869
-0.006387073722	-0.086383402794
-0.006300749971	-0.086327968432
-0.006220198913	-0.086297506658
-0.006104254076	-0.086008168012
-0.006012243644	-0.085806999556
-0.00594531792	-0.085781710638
-0.00587519516	-0.085719833166
-0.005774448346	-0.085579165817
-0.005669993445	-0.085428509042
-0.005599393331	-0.085195748156
-0.005536566756	-0.085116975503
-0.005473351059	-0.084960376211
-0.005413852168	-0.084921685513
-0.005308836903	-0.08491667657
-0.005222194474	-0.084901232696
-0.005121361562	-0.084842850446
-0.005017644142	-0.084803507812
-0.004925407417	-0.084802529424
-0.004819326616	-0.084782853112
-0.004737684432	-0.084691687968
-0.004605922504	-0.084690190995
-0.004494566761	-0.084419404613
-0.004421525495	-0.084280481727
-0.004306801506	-0.084167292669
-0.004201959098	-0.084121870471
-0.0041016444	-0.084093397113
-0.004013269919	-0.083952151499
-0.003913386089	-0.083911047553
-0.003806129857	-0.083843808865
-0.00374821939	-0.083830513207
-0.003684030499	-0.083714922157
-0.003568081646	-0.083587686682
-0.003482578677	-0.083500192445
-0.00341308068	-0.083485199104
-0.003316155441	-0.083339995961
-0.003252060938	-0.083335797821
-0.003154764174	-0.08327943785
-0.003080187613	-0.083146054642
-0.002982788597	-0.08270393247
-0.002899845258	-0.082507102822
-0.00281727698	-0.082376930257
-0.002745974446	-0.082371145135
-0.002675587473	-0.082255204869
-0.002570074471	-0.082073544026

-0.002463965955	-0.081882537421
-0.002396485543	-0.081869678554
-0.002282614534	-0.081561550693
-0.002184030734	-0.081508607254
-0.002106133955	-0.081355582745
-0.002015963345	-0.081163009965
-0.001919509407	-0.080999329814
-0.001841176977	-0.080840637298
-0.001763593426	-0.080589253707
-0.001650244623	-0.080430257416
-0.001558046242	-0.080406761314
-0.001458839405	-0.080356175824
-0.001356464008	-0.080255327042
-0.00129011054	-0.080073850289
-0.001221084661	-0.080033905305
-0.001122463346	-0.079906510451
-0.001054476319	-0.079903674142
-0.000982661214	-0.079701181918
-0.000916879425	-0.079698990679
-0.000810719223	-0.079675649732
-0.000719802185	-0.079590547271
-0.000655624913	-0.079492623128
-0.000565882546	-0.079427314535
-0.000505632745	-0.079369382847
-0.000440399434	-0.079300885763
-0.000362366559	-0.079085052978
-0.000273164217	-0.079075270186
-0.000173170285	-0.078987141519
-8.7989276E-05	-0.078858603731
1.101069074E-06	-0.07866652092
0.0001014539284	-0.078177307488
0.0001695815469	-0.078164063862
0.0002632950501	-0.077929258269
0.0003732156569	-0.077206468699
0.0004407018965	-0.077148312667
0.0005408023529	-0.077122413824
0.0006482105725	-0.076863879424
0.0007452893514	-0.076681780605
0.0008293996511	-0.076561661943
0.0009242255381	-0.076477117114
0.0009992919084	-0.076467812618
0.001065230998	-0.076419881679
0.0011472451315	-0.076184247266
0.0012296306555	-0.076138030729
0.0012997025282	-0.075945312297
0.0013635745353	-0.075910699887
0.0014560305862	-0.075714074989
0.0015402385169	-0.07570036116
0.0016225249383	-0.075383793138
0.0017248985057	-0.075379938375
0.0018069420414	-0.07532795409
0.0018996478095	-0.075113986621
0.0019618427515	-0.07496527161
0.002070393753	-0.074779530965

0.0021738343573	-0.074748171226
0.0022694629355	-0.074396137128
0.0023774853473	-0.074255923459
0.0024730785391	-0.074176097823
0.0025436578058	-0.074057120942
0.002616673866	-0.073952291989
0.0027184450701	-0.073728966221
0.0028377705673	-0.073496306587
0.0029134123771	-0.073393400255
0.0030308578953	-0.072818891497
0.0031324575001	-0.072479597061
0.0032323853848	-0.072251491072
0.0032956398288	-0.07220605856
0.0033833505883	-0.072145656993
0.0034679139558	-0.072005277608
0.0035842426559	-0.071655345939
0.0036608410102	-0.071654418232
0.0037362344466	-0.071634702039
0.0038476345835	-0.071454984059
0.0039498491928	-0.07139326716
0.0040386301769	-0.071254570797
0.0041094967246	-0.071237821143
0.0041743406541	-0.07122133377
0.0042398437399	-0.071081463671
0.0043384671654	-0.070851004105
0.0044301190073	-0.070832636999
0.0045067940668	-0.070779416639
0.0046319584802	-0.070696864877
0.0047078767689	-0.070643449067
0.0048070678001	-0.070483204662
0.0048612051239	-0.070478201644
0.0049419195826	-0.070453247343
0.0050381511393	-0.070409229936
0.0051216831275	-0.070404804079
0.0052083922387	-0.070152209135
0.0052960817552	-0.070065867528
0.0053902400081	-0.069900243927
0.0054493196457	-0.069833381729
0.005540582633	-0.069724501911
0.0056331183402	-0.069664132065
0.0057077248813	-0.06958888818
0.005769532186	-0.069566772987
0.0058995120271	-0.069513037235
0.0059880254555	-0.069297912162
0.0060638020053	-0.069282092951
0.0061216728205	-0.06927193255
0.0061965592553	-0.069067722987
0.0063182729234	-0.068988439761
0.0064115245522	-0.068831402946
0.006498177891	-0.068821459267
0.0065659815749	-0.068793592969
0.0066639743335	-0.068531730746
0.0067409934012	-0.068500743013
0.0068362295366	-0.068325123539

0.0069344917262	-0.068251409308
0.0070234102087	-0.068076347552
0.0071481312011	-0.068028879204
0.0072127689531	-0.068007279031
0.0072881324378	-0.067982881682
0.0073944659136	-0.067961903628
0.0074675416785	-0.067917772802
0.0075546937008	-0.067822155279
0.0076460636043	-0.06776109871
0.0077507680714	-0.067700132627
0.0078393415069	-0.067648861911
0.007932041306	-0.067519225419
0.0080249416089	-0.067160207646
0.0081219767654	-0.067041642706
0.0082100700062	-0.066925286683
0.0082899659738	-0.066856524123
0.0084088665203	-0.066661845857
0.0085237817993	-0.06652829241
0.0086525731013	-0.066114613972
0.0087351515802	-0.066066952878
0.0088131901679	-0.065986281865
0.0089081239826	-0.065975947937
0.0089936717878	-0.065933545829
0.0090778650693	-0.065767160697
0.0091494686041	-0.065744774182
0.0092338123887	-0.065649784674
0.0093089803724	-0.065575387061
0.0093587608939	-0.065572414002
0.0094722099014	-0.065480330502
0.0095366310971	-0.065142868742
0.0096115057605	-0.064971228017
0.0096996771587	-0.06479621317
0.0098016149192	-0.064770996536
0.0099081611665	-0.064565476533
0.0099968543297	-0.064452477077
0.010093746551	-0.064014379068
0.0101695115314	-0.063975005973
0.0102593831015	-0.06390842792
0.0103079554703	-0.063830272253
0.0103760601625	-0.063615698692
0.0104516283015	-0.063401617115
0.0105193487862	-0.063368715733
0.0106207570242	-0.063179038829
0.0107089772452	-0.062937928808
0.0108067129258	-0.06263614677
0.0108942325361	-0.062492840004
0.0109663056627	-0.062446582192
0.0110600618181	-0.062201213047
0.0111424219819	-0.062158177636
0.0112338527782	-0.062071340386
0.0112948603947	-0.062061479981
0.0113884860664	-0.062016866833
0.0114914839049	-0.061756017673
0.0115682193082	-0.061687874552

0.0116369507058	-0.06164748094
0.0117266843336	-0.061639900629
0.0117872539881	-0.061423046112
0.0118566837546	-0.061313697544
0.0119150553114	-0.061225128261
0.0120110847181	-0.061172679842
0.0120962428087	-0.061073195461
0.0121917029962	-0.061014354612
0.0122855217327	-0.060550097103
0.0123587028463	-0.060364056898
0.0124514882492	-0.060247668548
0.012535239118	-0.060030639388
0.0126210035946	-0.05970966195
0.0126860366157	-0.059545510924
0.0127612064186	-0.059479036516
0.0128755487849	-0.058843351764
0.0129534165149	-0.058807450655
0.0130276550141	-0.058789280678
0.0131298983052	-0.058739743574
0.0132103132197	-0.058670146644
0.0133272086735	-0.058658387966
0.0133825363649	-0.05863903461
0.013458249034	-0.058601999367
0.0135833372149	-0.058191286196
0.0136799842056	-0.058157486145
0.0137687334227	-0.058105689509
0.0138326938049	-0.058075529202
0.013918251886	-0.057839152054
0.01400239306	-0.057520983798
0.0141015935375	-0.057478377448
0.0141964321567	-0.057407777315
0.0142500718109	-0.057312067179
0.0143443582783	-0.057229775846
0.0144667575108	-0.057057298065
0.0145737659949	-0.056819935744
0.0146544496134	-0.056766808834
0.0147534169513	-0.056496099175
0.014829561067	-0.05648990586
0.0149003210166	-0.05645868452
0.0150094883618	-0.056416028889
0.0150781084126	-0.056378945143
0.0151645953397	-0.056226267102
0.0152545891348	-0.056144502507
0.0153388820245	-0.056140270932
0.01544027003	-0.05589977893
0.015500037931	-0.055767397704
0.0155986026952	-0.055630039587
0.0157033890497	-0.055589384141
0.0157955076001	-0.055564321202
0.0158438754434	-0.055487385077
0.0159263701845	-0.055466979197
0.016017099221	-0.055367566068
0.0160963718877	-0.055343738368
0.0161683934175	-0.055283922289

0.0162310734636	-0.055194573881
0.016316995154	-0.055059747133
0.0164069526365	-0.054786044431
0.0164884254897	-0.054627929398
0.0165779982473	-0.054592533749
0.0166471889919	-0.054503593977
0.0167198552318	-0.054433637853
0.0167711706791	-0.054405254423
0.0168795091779	-0.054238829879
0.0169617325699	-0.054088676834
0.0170473811909	-0.053998214059
0.0171371585816	-0.053891466204
0.0172034127631	-0.053847027155
0.0172879771239	-0.05383677852
0.0173842289518	-0.053659007314
0.0174501548269	-0.053574295598
0.0175317036003	-0.053507504225
0.0176131541951	-0.053484544003
0.0176927538301	-0.053372859848
0.0177767608709	-0.053333554597
0.0178466049108	-0.053322386586
0.0179451138717	-0.053129659185
0.0180404516318	-0.05285287229
0.0181291232052	-0.052702883178
0.0182469536106	-0.052579609241
0.0183522825789	-0.052489581622
0.0184275408412	-0.052236790017
0.0184882601332	-0.052183051885
0.0185688024715	-0.052163459289
0.0186474924807	-0.052037376859
0.0187149069543	-0.05194643455
0.0188062038096	-0.051936448898
0.0188726676066	-0.051897483843
0.0189425904806	-0.051858685071
0.0190356579669	-0.051802920548
0.0191409721113	-0.051695346899
0.0192192752911	-0.051484056316
0.0192878542822	-0.05146105055
0.0193775721221	-0.051235300693
0.0194974936726	-0.050927746226
0.0195826308802	-0.050625638223
0.0196612544789	-0.050464368352
0.0197172365374	-0.050462115065
0.0197904809595	-0.05021856191
0.0198604817063	-0.050146243683
0.0199392820039	-0.050062475781
0.0200387158887	-0.049913008196
0.020113128016	-0.049788776112
0.0202041031662	-0.049622139381
0.0202938223988	-0.049498615375
0.0203798226285	-0.049398238052
0.020462380382	-0.049314454202
0.0205564249842	-0.04913943983
0.0206514473673	-0.048753275746

0.0207439710969	-0.048723440364
0.020828066199	-0.048654506752
0.0209064805136	-0.048277614072
0.0210292134063	-0.047964146448
0.0211115435321	-0.047925873784
0.0211968599001	-0.047891690655
0.0212857787126	-0.047855062182
0.0213816062522	-0.047812672029
0.0214770139718	-0.047437512694
0.02157208162	-0.047384009586
0.0216820139562	-0.047335082739
0.0217826368352	-0.047174675289
0.021853020598	-0.047076640403
0.0219322773028	-0.047062959104
0.0220209080495	-0.046980773261
0.0221255030032	-0.046791338445
0.0221779038849	-0.046715647082
0.0222884171839	-0.04629020005
0.0223733236696	-0.046097690445
0.0224455296848	-0.046096727243
0.0225253095857	-0.046088401183
0.0226226192322	-0.046022479198
0.0227241083125	-0.045772610288
0.0228466305458	-0.045301522162
0.0229365556929	-0.045131631436
0.0230328536549	-0.045117643398
0.0230919529687	-0.045047164596
0.0231622393792	-0.044892331047
0.0232230341189	-0.044835948959
0.0233139051624	-0.044828510064
0.0233831918494	-0.044798861912
0.0234307019735	-0.044790225261
0.0235187344186	-0.044395681756
0.0236345116294	-0.04426941145
0.0237046861503	-0.044188018075
0.0237956439384	-0.044099775584
0.0239129361358	-0.044067527012
0.0239769499322	-0.044044071516
0.0240547180424	-0.043846674531
0.024123082055	-0.043739433163
0.024223185249	-0.043662983897
0.0242948566377	-0.043404222087
0.024382068592	-0.043395870546
0.0244840660709	-0.043228951126
0.0245630820203	-0.043213558932
0.024623992387	-0.043192654125
0.0246882896615	-0.043054745728
0.0248051603254	-0.042983459399
0.0248883177803	-0.042672052479
0.0249552477987	-0.04263201519
0.0250418277134	-0.042574802118
0.0250957036772	-0.042514994199
0.0251605320483	-0.042429210896
0.0252385315662	-0.042330452906

0.0253212763068	-0.042226896773
0.025409318041	-0.042153218394
0.0255060330605	-0.042100728569
0.025617777845	-0.041818671682
0.025727960456	-0.041580031853
0.025819082792	-0.041478349544
0.025910406499	-0.041337438989
0.0259984533389	-0.041266208014
0.0260707211318	-0.041005787517
0.0261498404352	-0.040773771413
0.0262461519693	-0.04062485643
0.0263345433414	-0.040463998641
0.0264312335891	-0.040093732249
0.0265406814155	-0.040088216744
0.026638548946	-0.039948286118
0.0266933089308	-0.039921020802
0.0267782034379	-0.039780515102
0.0268889840197	-0.039569524872
0.0269588709589	-0.039568873212
0.0270086099104	-0.03955994522
0.0270856145777	-0.039229342019
0.027166939206	-0.03916746651
0.027251404802	-0.039147620331
0.0273135724799	-0.03914144316
0.0274127939796	-0.039095487525
0.0274896945362	-0.039047728057
0.0275716453469	-0.039012954464
0.0276439692145	-0.039004458759
0.0277456391471	-0.038941719098
0.0278530232131	-0.038817748752
0.0279623319436	-0.038544995761
0.0280522254597	-0.038333324449
0.0281376460214	-0.038273338238
0.0282082845997	-0.038070613626
0.0282910389742	-0.038026425804
0.028377553754	-0.03796148921
0.0284543946558	-0.037849932045
0.0285341540663	-0.037763802204
0.0285965462075	-0.037727949706
0.0286955394436	-0.037657017397
0.0287577634177	-0.03763761393
0.0288430636439	-0.037457769607
0.028956809095	-0.037247431396
0.0290393944513	-0.037101820675
0.029130875483	-0.037089372694
0.0292368735989	-0.037029841437
0.0293389812088	-0.036987445829
0.0294228579101	-0.036959127142
0.0294911109156	-0.036761804667
0.0295574167213	-0.036754782745
0.0296592756633	-0.036646622854
0.0297548239022	-0.036488582482
0.0298701798128	-0.036472691098
0.0299647212011	-0.03638403304

0.0300477519996	-0.036374380751
0.0301144602167	-0.036266174009
0.0302128999447	-0.036011838255
0.0302920629002	-0.035959243516
0.0303605017178	-0.035855972986
0.0304228413005	-0.035852476612
0.0305205349426	-0.035520134569
0.0306027476485	-0.0354463584
0.0306792415812	-0.035383618358
0.0307906146036	-0.035067317458
0.0308501058851	-0.034954951525
0.0309340297539	-0.034919209522
0.0309969143317	-0.034792433909
0.031057483548	-0.034524490004
0.0311450311146	-0.034192976787
0.031203274458	-0.034135130755
0.0312660930769	-0.034133662142
0.0313515690639	-0.034114163953
0.031432579688	-0.034099674682
0.0314985770291	-0.034048843548
0.0316134301384	-0.034017428312
0.0317145763714	-0.033971302865
0.031808940305	-0.033966351484
0.0318947613458	-0.033930643908
0.0319919038608	-0.033853357838
0.0320813846889	-0.033677056661
0.0321746225697	-0.033557029375
0.032288796949	-0.033360162608
0.0323624572232	-0.033326755272
0.0324331565178	-0.033162783674
0.0325374556848	-0.033067236658
0.0326303714193	-0.032741255944
0.0327179842797	-0.032594987177
0.0328033836514	-0.03222755727
0.0329054133982	-0.031851180797
0.033001363637	-0.031542491431
0.0330824586522	-0.031215663689
0.0331647986447	-0.031016509716
0.0333052813507	-0.030815317903
0.0333963997761	-0.030714320248
0.0335083685685	-0.030618827545
0.033584080185	-0.030548779685
0.0337154749156	-0.030374213413
0.0337939387285	-0.030365477821
0.0338946446034	-0.030263302948
0.0339686755592	-0.029984884148
0.0341127098105	-0.029718293189
0.0341793622193	-0.029635506418
0.034235687669	-0.029538702865
0.0343119577316	-0.029530932214
0.0344491047508	-0.029239002428
0.0345458234444	-0.029072982917
0.0346327127518	-0.028909024265
0.034705300393	-0.028856711541

0.034786681094	-0.028808226634
0.0348660093935	-0.028710330056
0.0349530444851	-0.028278545185
0.0350193400226	-0.0282507868
0.0351105770182	-0.028123649708
0.0351723838078	-0.028100869336
0.0352678676678	-0.028060709887
0.0353557711788	-0.027730230077
0.0354236923806	-0.027723529679
0.0354941244019	-0.027664949452
0.0355919535526	-0.027637820686
0.0356720597469	-0.027516850781
0.0357613794279	-0.027515997355
0.0358534725457	-0.027374566422
0.035942344473	-0.027311996192
0.036027510877	-0.027203620326
0.0361100986136	-0.027078413898
0.0361755344139	-0.027004361261
0.0362651539492	-0.026952361499
0.0363442346203	-0.026809290877
0.0364061020325	-0.026724652618
0.0365010145119	-0.026617987291
0.0365943667526	-0.026290449515
0.0366654517396	-0.026276595198
0.0367587632025	-0.026215828807
0.0368273208241	-0.026118471653
0.0368977102838	-0.026039502363
0.0369824837253	-0.025908678985
0.0370494547194	-0.025905679206
0.0371214009993	-0.025879894217
0.0372142721089	-0.025830837402
0.0373018987807	-0.025730169524
0.0373838787584	-0.025701919923
0.0374571715265	-0.025392767085
0.0375330057594	-0.025222702853
0.0376400257377	-0.025106058077
0.0377241815408	-0.025053092528
0.0378184898327	-0.024945692402
0.0378976838514	-0.024872769881
0.0379686195903	-0.024849888212
0.038075467021	-0.024762542661
0.0381446730731	-0.024587210456
0.0382305013419	-0.024424528206
0.0383106686038	-0.024141692036
0.0383962847447	-0.023955529047
0.0384772350013	-0.02394133889
0.038554003262	-0.023929343501
0.0386567635786	-0.023848451692
0.0387372807755	-0.023700326821
0.0388615033258	-0.023264726532
0.0389341726168	-0.023244090265
0.0390183736277	-0.023166412467
0.0390990061598	-0.023159489472
0.0391793192545	-0.02314127592

0.0392907579476	-0.022993349885
0.0393924376996	-0.022808145002
0.0394810250227	-0.02261414838
0.0395915933711	-0.02185487485
0.039666443596	-0.021799805231
0.0397426323983	-0.021796629339
0.0398482336787	-0.021648740907
0.0399784326738	-0.021431104307
0.0400778604869	-0.021377554261
0.0401527846984	-0.021301571285
0.0402371285056	-0.021297305398
0.0403315345889	-0.021113162459
0.0404064159999	-0.02097910904
0.040477758532	-0.020884505315
0.0405600830086	-0.020864814501
0.0406495260519	-0.020812161662
0.0407332196163	-0.020756097426
0.0408243618835	-0.02073525031
0.0409155094174	-0.020590068717
0.0409936816314	-0.020450967445
0.0410421887719	-0.020438501649
0.0411210788516	-0.020327073802
0.0412074673571	-0.020315794636
0.0413052951102	-0.020247108324
0.0413762933626	-0.02017054554
0.0414577462315	-0.020045687838
0.0415455256676	-0.02001819501
0.0416324700451	-0.019995815857
0.0417641168473	-0.019877186417
0.0418264483379	-0.019736877076
0.0418961825176	-0.019687119353
0.0419715851647	-0.019659787274
0.0420419503028	-0.01965437228
0.0421465699548	-0.019471260336
0.0422377270107	-0.019362253938
0.0423333111749	-0.0190962041
0.0424283234095	-0.018992000896
0.0425076744201	-0.018938482407
0.0426003632321	-0.018861431735
0.0426769411025	-0.018700212771
0.0427570421666	-0.018574271287
0.042824207239	-0.018556324902
0.0429034295838	-0.018548921541
0.0429837954123	-0.018400548377
0.0430836272506	-0.018253650933
0.0431558426061	-0.018207069746
0.0432387847082	-0.018197097551
0.0433194872183	-0.0180298283
0.0433820872993	-0.018000332101
0.0434566352885	-0.017800970608
0.0435215927116	-0.017774084135
0.0436303035714	-0.017656676228
0.0437383299571	-0.017587100841
0.0438228954393	-0.017284097115

0.04393520985	-0.016963106077
0.0440280544148	-0.016866752432
0.0441226925673	-0.016665891365
0.0442267852148	-0.016342215755
0.0443481419982	-0.015968472565
0.044429417328	-0.015939433898
0.0445125880282	-0.015770283493
0.044583309231	-0.015615353225
0.0446784714809	-0.015597196528
0.0447621277945	-0.015534008551
0.0448613976569	-0.015530145104
0.0449470571517	-0.015459079746
0.0450459736951	-0.015404620309
0.045118634403	-0.015298684586
0.0452126495889	-0.015012922429
0.0452937414421	-0.014854943534
0.0453679337638	-0.014609604518
0.0454381735105	-0.014506752559
0.0455162519815	-0.014358716481
0.0455948874789	-0.014183166811
0.0456849856886	-0.014101095963
0.0457708832269	-0.014083900313
0.0458610606797	-0.014000997653
0.045949721837	-0.014000383864
0.0460403642225	-0.013744789424
0.0460977750764	-0.013735987028
0.0461938386001	-0.013409795102
0.0462798201394	-0.01324427546
0.0463554348197	-0.013187959113
0.0464524771226	-0.013063009995
0.0465176606905	-0.013012517537
0.0465807110664	-0.013005103655
0.0466529310709	-0.012979072337
0.0467364806702	-0.012810077684
0.0468551878644	-0.012367437432
0.0469154157296	-0.01233507933
0.0469722649379	-0.012288477168
0.0470278552872	-0.012271073799
0.0470972749741	-0.01226243782
0.0471845018606	-0.012080556129
0.0472652917811	-0.011640579965
0.0473352427528	-0.011366677605
0.0474203718965	-0.011244271866
0.0474953971743	-0.011219087686
0.047603894781	-0.011176032857
0.0476871858898	-0.011150917432
0.0477706020471	-0.011139310719
0.0478697770656	-0.011094936734
0.0479782927048	-0.010690462863
0.0480940807183	-0.0106319425
0.0481753492354	-0.010514092728
0.0482456731515	-0.010452338661
0.048331896929	-0.010384218249
0.0484026498592	-0.010343871029

0.0484773348819	-0.010309422017
0.0485942960853	-0.010092366629
0.0486656492541	-0.009971875747
0.0487424843224	-0.009966185012
0.0488711249834	-0.009836582329
0.048952012255	-0.009712580111
0.049023249941	-0.009620148204
0.0490977652346	-0.009592456842
0.0491871104808	-0.009522260333
0.0492718032437	-0.009472805075
0.0493586413383	-0.009243383161
0.0494477117722	-0.009066328778
0.0495314745312	-0.00902341306
0.0495892047489	-0.008883825003
0.0496670285559	-0.008844528241
0.0497902564702	-0.008781009598
0.0498810023011	-0.008680800644
0.0499322344937	-0.008612036701
0.0500191904813	-0.008584586968
0.0500975720527	-0.008453337901
0.0501829118909	-0.008302354275
0.0502848653891	-0.008152186659
0.0503550963976	-0.008126473826
0.0504076615913	-0.008109631199
0.0504813162312	-0.008023753049
0.0505759158324	-0.007921041261
0.050672823157	-0.00770934311
0.0507803439092	-0.00756304764
0.0508787854485	-0.007305696017
0.0509606767752	-0.007278634187
0.0510446129955	-0.007274092203
0.0511205280177	-0.00696595581
0.0512220850995	-0.006882225203
0.0513170821249	-0.006630842519
0.0513884786972	-0.006620727454
0.0514617818953	-0.006588029303
0.0515429402125	-0.006440058551
0.0516686977894	-0.006120038221
0.0517416188643	-0.006107080972
0.0518118147146	-0.006042949238
0.0519204414632	-0.005667279447
0.0519966394383	-0.005313411738
0.0520852559383	-0.005202241544
0.0521915554233	-0.005127881332
0.0522897213707	-0.005044015827
0.0523937082623	-0.004926562052
0.0524471893351	-0.004879564787
0.0525730514695	-0.004728089442
0.052657520669	-0.004649985705
0.0527623485942	-0.00429110255
0.0528464781551	-0.004288758618
0.0529400860101	-0.004252330021
0.0530332142686	-0.00420351207
0.0531123637381	-0.004172986993

0.0532005521566	-0.00414370021
0.0532856894341	-0.004065685638
0.0533752623	-0.003977154593
0.0534614616819	-0.003580762308
0.053567393541	-0.00354993978
0.0536619771486	-0.0032855694
0.0537582773973	-0.003285480489
0.0538324612637	-0.003093853521
0.0539046862716	-0.003020959165
0.053980733133	-0.003003183059
0.0540996834904	-0.002541661946
0.0541710355701	-0.002498172869
0.054275585141	-0.002198857909
0.054345628203	-0.002145882429
0.0544365862915	-0.002040707275
0.0545157722449	-0.001998749788
0.0545935721811	-0.001997593314
0.0546627488294	-0.001977520303
0.0547357909231	-0.001868373975
0.0548011003318	-0.001803330202
0.0549050131747	-0.001504377683
0.0549908741719	-0.001427957277
0.0550661300381	-0.001387841342
0.0551552560691	-0.001136635382
0.0552274435358	-0.001124354388
0.0553010226607	-0.000957726411
0.0553895810923	-0.000897403631
0.055470207318	-0.000874788306
0.0555753231584	-0.000552596409
0.0556523694876	-0.000549166501
0.0557141902947	-0.000473364345
0.0558032457046	-0.000304191919
0.0559136719974	-0.000292985145
0.0560004619906	-0.000123568846
0.0560953773719	-2.01461788E-16
0.0561725304361	0
0.0562559425213	0
0.0563214321656	0
0.0564161618671	0
0.0565086820265	0.0002895508253
0.056585434642	0.0002986425466
0.0566407653415	0.000392393762
0.056703911832	0.0005000554012
0.0568053763909	0.0005330600162
0.0568708705058	0.000563614783
0.0569410526472	0.0006503913073
0.0570339968515	0.0007301986315
0.057138123036	0.0008153115068
0.0572134543145	0.0008676978337
0.0572944190116	0.0009240875984
0.0573822673155	0.0010739037348
0.057443808833	0.0011306593712
0.0575612604665	0.001785631992
0.0576526960074	0.0018713184379

0.0577497871329	0.0022527738833
0.0578713337457	0.0022538752095
0.0579387116429	0.0022904121244
0.0580353112369	0.0026159465532
0.0581086757544	0.0026784925395
0.0581709630881	0.0026786948073
0.0582528685324	0.0026940400644
0.058359287522	0.0032876986305
0.0584499866874	0.0034192771357
0.0585210207811	0.0036568571581
0.0586094655305	0.0038359845064
0.0586800808773	0.0040817251374
0.0587725191373	0.0041809719522
0.0588386703458	0.004335389446
0.0589572857543	0.0043906874928
0.0590664282511	0.0044652147568
0.0591573420373	0.0044959780841
0.0592318831647	0.004528066214
0.0593454220515	0.0046710708531
0.0594234338067	0.0047459202997
0.0595040480329	0.0047854129629
0.0595663641955	0.0048371091919
0.059642514771	0.0048508017905
0.0596953803364	0.005008741108
0.0598063996871	0.0051732483383
0.0599105288061	0.0052561578396
0.0599952672889	0.005406737885
0.0600670631591	0.0054739273427
0.0601724148246	0.0056196126918
0.0602665311959	0.0056241959586
0.0603605810523	0.0056357270072
0.0604271145055	0.0056711913239
0.060506825475	0.0057807628174
0.0605557210251	0.0058587527419
0.0606148509201	0.0058808655576
0.0607120488162	0.0059248566991
0.0607685187096	0.0059259529659
0.0608682947587	0.0060753688017
0.0609551135552	0.0063885443878
0.061043459928	0.0066709311877
0.0610992399433	0.0067350489995
0.0611918838731	0.0067608545786
0.0612762258583	0.0069543100185
0.0613501669423	0.0070430858518
0.0614354133672	0.0071435704843
0.061521692273	0.0072025137253
0.0615947475663	0.0073319498122
0.061659311112	0.0074930587699
0.0617606662383	0.0076321796972
0.0618445215153	0.0077095718032
0.0619308632414	0.0077557220373
0.0620066927808	0.0078709011303
0.0621047477782	0.0080510961712
0.0621661538464	0.0081703546565

0.0622470727735	0.0083182026108
0.0623366817698	0.0084179242849
0.0624214373962	0.0084787130329
0.0624978879287	0.0085186006642
0.0625733171605	0.008530988823
0.0626594881468	0.0086321442663
0.0627229235256	0.0087879077016
0.0627768122199	0.0088398511246
0.062873028531	0.0088883327102
0.0629834119691	0.0091553014755
0.0630512948311	0.00917275761
0.0631110372548	0.009246260801
0.0632050915521	0.0096019400612
0.0632823282468	0.0097407758316
0.0633760008691	0.0097511922169
0.0634898130221	0.0098654331791
0.0635623760332	0.009972782965
0.0636256273639	0.0100379886664
0.0637620651685	0.010290085322
0.0638575388126	0.0105431590779
0.0639467316308	0.0106145298064
0.0640577205803	0.0106254519897
0.0641546189063	0.0106686241859
0.0642551204738	0.0109297962594
0.0643389529661	0.0111694167958
0.0644119711108	0.0113572905313
0.0644924450797	0.0115262306825
0.0645843429491	0.0115695822847
0.0647013045049	0.0117227149977
0.0648116075948	0.011810781918
0.0648853784087	0.0118737844285
0.0649673553603	0.0119584156584
0.0650746427614	0.012184498284
0.0651363338639	0.0122034849572
0.0652138132261	0.0123490399019
0.0653033878133	0.0124659422588
0.0654087615106	0.0124896759832
0.0654975554093	0.0125871499797
0.0655982701359	0.0126195151397
0.065653452727	0.0126550484375
0.065735510723	0.0126715764579
0.0658104086339	0.0127040581675
0.0658870262388	0.0127470308635
0.0659590344494	0.0127689954118
0.0660148065626	0.0128332401452
0.0661000621195	0.0128462762732
0.0661566013503	0.012875036051
0.0662358283118	0.012944927362
0.0662974473125	0.0130585167478
0.0663599032381	0.0130659467567
0.0664595313447	0.013112387017
0.0665456145266	0.0132195652927
0.0666073301121	0.0133619898095
0.0666923124318	0.0134199707221

0.0667870807513	0.0135390137915
0.0668836281502	0.0135696056181
0.0669585338362	0.013579912512
0.0670181803866	0.013594102484
0.0670855458034	0.0136119106028
0.0671699182282	0.0136200571016
0.0672699351323	0.0136688571618
0.0673527977564	0.0137048230885
0.0674522108311	0.0138983191701
0.0675519396265	0.0141900256924
0.0676201417107	0.0143194405577
0.0676956301537	0.0143530616044
0.067824442989	0.0144411277293
0.0678943542859	0.014478337782
0.0679604699922	0.014516330674
0.068050736087	0.014635758785
0.0681502940774	0.0148096571539
0.0682343247772	0.0149129439324
0.0683476804314	0.0152429663619
0.0684070190773	0.0152491997036
0.0684692396028	0.0152774946949
0.0685571533239	0.0153241374282
0.0686426350671	0.0154156991394
0.0687275868408	0.0154490575563
0.068815123167	0.0154634791531
0.0689009768882	0.0155335850439
0.0689721317719	0.0156182620391
0.0690423184142	0.0156396188985
0.069117977269	0.0156866592807
0.0692275257056	0.0159324570874
0.0693237482732	0.0159968085964
0.069390584725	0.0161623601858
0.0694748075276	0.0161906417573
0.0695394876404	0.0161921119577
0.0696314502695	0.0164188238334
0.0697119297091	0.0166631955592
0.0697837961299	0.0167451359679
0.0698850339832	0.0169095361651
0.0699656105331	0.0170065828999
0.0700732055413	0.0170424081723
0.0701583078301	0.0172220354186
0.0702586632652	0.0172616566128
0.0703229069158	0.0173220712136
0.0704085589685	0.0173988781806
0.0704906315715	0.0175460421236
0.0705922109997	0.0176312571402
0.0706959440924	0.0177552281147
0.0707999866451	0.0178537627253
0.0708694239087	0.0178784787417
0.0709544679167	0.0179641784217
0.0710436196229	0.0179752971425
0.0711177260125	0.0179818396498
0.0712062575708	0.0180731525691
0.0712956579328	0.0183308349307

0.0713651314577	0.0183508114044
0.071448078601	0.0185445421183
0.0715173423644	0.0185577799943
0.0716423697821	0.0185952000995
0.0717387987971	0.0186983842502
0.071816578389	0.0187049392231
0.0718643205951	0.0187642940137
0.0719427965183	0.0187687086437
0.0720288929059	0.0187934070982
0.0721260777013	0.0191254110037
0.0721908308794	0.0191604277975
0.0722714118984	0.0191978147359
0.0723549144665	0.0193214441032
0.0724679352957	0.0194575365453
0.0725318609444	0.0195164206081
0.0726026847463	0.0195530785651
0.0726660116502	0.0197207403691
0.0727579364853	0.0201314849749
0.0728699690257	0.0202622619027
0.0729754281565	0.0204190330111
0.0730665536612	0.0206086935693
0.0731339799645	0.0206282317227
0.0732375669245	0.02073249881
0.0733080259518	0.0207634983009
0.07342676811	0.0207740367237
0.0735028358078	0.0207869598008
0.0735900309981	0.0210956989718
0.0736605129519	0.0211741141924
0.0737689504389	0.0211833760788
0.0738514494659	0.0212932326354
0.0739073708704	0.0212998730002
0.0740017506124	0.0213124865912
0.0740706975164	0.0214692215157
0.0741325185002	0.0215246453523
0.0742000218688	0.0216441035764
0.0742921314433	0.0217577084843
0.0744021527474	0.0218259700784
0.0744784683886	0.0220211339466
0.0745512075	0.0222032468825
0.0746248425121	0.0223525910002
0.0747015297246	0.0223654205259
0.0747636347085	0.0224199105643
0.0748342862856	0.0226324939635
0.0749159047799	0.0226588402674
0.0750385747859	0.0231153435583
0.0751186110993	0.0233270659124
0.0752164703515	0.0234531632353
0.0753130211788	0.0236842611749
0.0754089431713	0.023780067727
0.0754788679031	0.0238203446006
0.0755677643629	0.0239441232257
0.0756719373474	0.0240843543164
0.0757825374342	0.0241231758917
0.0758563423847	0.0242060725647

0.0759416948736	0.0245961874706
0.0760099786233	0.0246702360237
0.0760975691966	0.0246797566492
0.0761762047471	0.0247859735496
0.0762719794611	0.0249293220536
0.0763577549949	0.0249792657737
0.0764083023941	0.0250696212817
0.076524895597	0.0251627490493
0.0766209779531	0.0251802903343
0.0766745949681	0.0252312340244
0.0767605570354	0.0252956922795
0.0768327732292	0.0254668649729
0.0769099230783	0.025551695917
0.0769998133082	0.02576917781
0.0770679257189	0.0258268301631
0.0771735190696	0.0258291444102
0.0772852235566	0.0259937874149
0.0773848622947	0.0260761530171
0.0774592361296	0.0261929939907
0.077525234675	0.0263749828477
0.0775984752334	0.0263843048062
0.077661388246	0.0264470544031
0.0777527450353	0.0265653291045
0.077835430988	0.0266779414766
0.0779223222445	0.026789404085
0.0780159057943	0.0270874507603
0.0780810516183	0.0272116100632
0.078164115434	0.0272886309932
0.0782473463097	0.0273582393916
0.0783426490328	0.0274682337164
0.0784376140009	0.0278925507694
0.0785137548686	0.0279946553025
0.0785780302369	0.0280755992607
0.0786660103742	0.0280768778951
0.0787435502272	0.0282384028809
0.0788117983473	0.0282465900745
0.0788807287546	0.0283905862828
0.0789421716701	0.0284334915143
0.0790149818208	0.0285478981835
0.079110596348	0.028779930178
0.0791893263669	0.0289272887956
0.0792894320061	0.0289700964429
0.0793629135153	0.029126212254
0.0794206583898	0.0292267669371
0.0795142751474	0.0294421110586
0.0796136965351	0.0295310595916
0.0797011824263	0.0295640504659
0.0797761285499	0.0296910142227
0.0798680447264	0.0297529069354
0.0799456457173	0.0298249291493
0.0800249384882	0.0298598152172
0.0800887657833	0.0298654984973
0.0801664965887	0.0299679662756
0.0802325120585	0.0299754609842

0.0803319433093	0.0301122981345
0.080416409833	0.0301329944194
0.0804776372047	0.0301455891543
0.0805497542494	0.0301484546896
0.0806302452376	0.030522782533
0.0807338432089	0.0307744773979
0.0808333059123	0.0310385919773
0.0808948721315	0.0311396757983
0.0809765033416	0.0312161192625
0.0810642592049	0.0313287034281
0.081143667258	0.0313415646455
0.08122879663	0.0315162623423
0.0812889980762	0.0316540119543
0.0814032048625	0.0316858284968
0.0814703800898	0.0317570862557
0.0815855065931	0.0320235945647
0.0816551582603	0.0320439021505
0.0817346547158	0.0322777594894
0.0818157245213	0.0323562731862
0.0818846930195	0.0323916198074
0.0819844426017	0.0326671592118
0.0820739628939	0.0327115229114
0.0821527904721	0.0327790773762
0.082235624407	0.0327853368597
0.0823393202514	0.0329866372948
0.0824674572859	0.0330370752814
0.0825365295785	0.0332561761324
0.0826180689972	0.0334308455994
0.0826929603586	0.0334523517518
0.0827880558908	0.0335777414382
0.0829053663027	0.0338141177617
0.0829674193975	0.0338256143998
0.0830375423706	0.0340083367359
0.0831033278966	0.0341363114341
0.0831682701564	0.0341787191121
0.083276347211	0.0343600919014
0.0833704135043	0.0343699085757
0.0834812803068	0.034547665224
0.0835679938132	0.0346703419386
0.0836981828353	0.0350334679869
0.0837591785428	0.035072170745
0.0837970741045	0.0350789348815
0.0838693599347	0.0352954225314
0.0839290858841	0.0353265306587
0.0840154150478	0.0354456842218
0.0841201624376	0.0355219525211
0.0841823647235	0.0355292152315
0.0842598990408	0.0356920382523
0.0843291607116	0.0357104424646
0.0844094654006	0.0359778264963
0.0845179536135	0.0361272282657
0.0846177355834	0.0363042862664
0.0846790450821	0.0363056355291
0.0847405875062	0.0363645282112

0.0848333096267	0.036828413674
0.0849200465774	0.0370320937886
0.0849826332794	0.037158417912
0.0850793849369	0.0373855916553
0.085190059934	0.0379777618182
0.0852629247134	0.0379795785752
0.0853253141717	0.0380294092975
0.085393794031	0.0381755958786
0.0854912907868	0.0383931620173
0.0855876451369	0.0384964960152
0.0856675802854	0.0385837588321
0.0857527149642	0.0386645748802
0.0858399930476	0.0387228980036
0.0859088188665	0.0388155018232
0.0859727759993	0.0388581042444
0.0860667083942	0.0388850126382
0.0861560756975	0.0389417473411
0.0862412343284	0.038981324391
0.0863514089902	0.0391054353057
0.0864500881356	0.0391772273482
0.0865446078856	0.039210140367
0.0866330725683	0.0395502721857
0.0867191202756	0.0396374677366
0.0867952662029	0.0396822490247
0.086872156747	0.0397311405457
0.0869333472163	0.0397405252891
0.0870326909114	0.0398076159056
0.0871139182889	0.0398746917043
0.0871851603845	0.0401473330158
0.0873062325703	0.0402163811989
0.0874201236289	0.040533722992
0.0874871077182	0.0405538666212
0.0875832946724	0.0406492453397
0.0876541553773	0.0406689212085
0.0877292264393	0.0406777256183
0.0878109202233	0.0408585668134
0.0878970146412	0.0408963400453
0.0879702005035	0.0409803841768
0.0880536413574	0.0409825502952
0.0881309511911	0.0412241599775
0.0882433500905	0.041773864478
0.0883306172613	0.041841012969
0.0884358111305	0.0420969465835
0.0885354020804	0.0422681924558
0.0886347617775	0.0422941979329
0.0887376001756	0.0423289236322
0.0887856897373	0.0423969871731
0.0888515831239	0.0424559046599
0.088950604803	0.0426045519744
0.0890569867065	0.0427394770737
0.089139124835	0.0428559000886
0.0892209476167	0.0429419808339
0.0893256502568	0.0431301529729
0.0893990533228	0.0434249269648

0.0895054666218	0.043555107135
0.0896081978878	0.0436831125232
0.0896827018938	0.0437609122321
0.0897826479254	0.0438380323861
0.0898580442368	0.043903349167
0.0899306130188	0.0440535481835
0.0900160964897	0.0441443374795
0.0900896127807	0.0441716888033
0.0901931397071	0.0444377484241
0.0902633997686	0.0445641586234
0.0903377718116	0.0445814413646
0.0904513590836	0.0449808738992
0.0905125082273	0.0449945440326
0.0906030024378	0.045044924161
0.0906944550956	0.0454023998845
0.090792650213	0.0457964991485
0.0908908430768	0.0458677144727
0.0909598421236	0.0460271888448
0.0910464909575	0.04639761328
0.0911201007786	0.046418797184
0.0912029932105	0.0464435358843
0.0912917936786	0.0465819270651
0.0913739587967	0.0465935023063
0.091432127341	0.0466143120952
0.0915135856662	0.0467505524463
0.0915717093263	0.0468351443405
0.091641728892	0.046882135668
0.0917262165105	0.0470448868304
0.0918395723926	0.0471050349451
0.0919244513458	0.0472063470654
0.0920279726195	0.0473971097294
0.0921120719138	0.0475298450487
0.0922110725734	0.0477126666225
0.0922798991777	0.0478234837567
0.0923689420064	0.0481311690802
0.0924526091704	0.0481643572223
0.0925231243696	0.0483487157035
0.0926199452172	0.0484437258178
0.092686980088	0.04847213453
0.0927545722677	0.0485184101602
0.09282763407	0.0485243839833
0.0929098369176	0.0486919833531
0.0929767279078	0.0487644494668
0.0930644148314	0.0488454106718
0.0931526744139	0.0490474192712
0.0932412079201	0.0491314855943
0.0933084276322	0.0491476310196
0.0933742556243	0.0492842838031
0.0934517035089	0.0493950786371
0.0935387265619	0.0494151513992
0.0936169887037	0.0494722162253
0.0937091268909	0.0495514227825
0.0938034941021	0.0496075827797
0.0939050255369	0.049756585097

0.0940036803837	0.0499620914662
0.0940812103603	0.050066072771
0.0941468842813	0.0500726543696
0.0942220685228	0.0500735232404
0.0942997706744	0.0501905377484
0.0944071766855	0.0503580718661
0.0945096989896	0.0506405828537
0.0946532735972	0.0506701013332
0.0947433404809	0.0508289067527
0.0947990329706	0.050904505996
0.0948616255111	0.0509356481061
0.0949756427714	0.0509597464975
0.095047642361	0.050974056019
0.0951536123445	0.0510021393457
0.0952775663215	0.0510390653628
0.0953760782176	0.0511374883719
0.0954728120184	0.051279217841
0.0955366275127	0.0513020452055
0.0956255384301	0.0513679086381
0.0957032586379	0.0513896076127
0.0957883428926	0.0515251974161
0.0959036614462	0.0516312694405
0.0959639689718	0.0516449075585
0.0960374799798	0.0517832609534
0.09611435379	0.0521435093804
0.096229542063	0.052400198585
0.0963116460839	0.0525848533717
0.0963889924751	0.052598544454
0.0964627149242	0.0526732832368
0.0965434131227	0.052877241397
0.0966448309528	0.052915850047
0.096711185332	0.052948090736
0.0968052219833	0.0530233970864
0.09687861202	0.0530283554229
0.0969594538243	0.0531558851289
0.0970394008583	0.0532301552735
0.0971285317433	0.0535151403508
0.0971989343364	0.0535458352312
0.0972792447373	0.0535855686849
0.0973591407923	0.0535954537337
0.0974423806726	0.0535982274451
0.0975549243409	0.0539201566056
0.0976024676579	0.0540014492825
0.0976886011022	0.0540340256688
0.0977771593568	0.0541251105795
0.0978593929817	0.0541888125673
0.0979301495424	0.0542314087444
0.0980051496254	0.0542384228906
0.0980807773908	0.0543012062072
0.0981777443151	0.0543118841577
0.0982486917419	0.0543778203158
0.0983601367607	0.0546742520127
0.0984461261809	0.0548130894492
0.0985218455128	0.0548635419406

0.0986275915855	0.0551687086852
0.0987077739369	0.0551834796637
0.0988002984682	0.0553259554419
0.0988952072352	0.055342822834
0.0989822387542	0.0554463306226
0.0990710738771	0.0554916216178
0.0991555438449	0.0555080299043
0.0992153226251	0.0555450811861
0.0993002065283	0.0557510550093
0.099368726655	0.0559736326836
0.0994464932357	0.0560385956724
0.0995275611404	0.0561652255647
0.0996159935352	0.0562145395062
0.0996834935169	0.056259697667
0.0997616886735	0.0563896140213
0.0998532145591	0.0565252042649
0.0999646345536	0.0565766007127
0.100067917264	0.0567308382231
0.1001690551176	0.0567876850601
0.1002537860831	0.0568162758379
0.1003416787702	0.057238998648
0.1004552324522	0.0572551826738
0.1005387739925	0.0572904302273
0.1006016054118	0.0573067585371
0.1006703752441	0.0573219166282
0.1007287410455	0.0573579468819
0.1007853706065	0.0574450923441
0.1008581307553	0.057476925123
0.1009536972088	0.0575492592008
0.1010521481154	0.0575529799328
0.1011492670483	0.0577330579114
0.1012578830112	0.0577408052404
0.1013534546793	0.0579743176173
0.1014078620979	0.058041790454
0.1014817372911	0.0580781882242
0.1015618770049	0.0580954882996
0.1016767738389	0.058877834975
0.1017308640349	0.0589211188645
0.1018329716552	0.059158239825
0.1019165607923	0.0591804670272
0.102000279548	0.0592276619269
0.1020694448729	0.0592851456863
0.1022031139275	0.0593197780132
0.1022930896188	0.0593717451222
0.1023679963996	0.0595806759695
0.102477199125	0.0599086761046
0.1025549401343	0.0599105528332
0.1026570050502	0.0599503856354
0.1027398349312	0.0599890665587
0.1028202923577	0.0601531868977
0.1029195816376	0.0601952166108
0.1029816393671	0.0602040036701
0.1030575291629	0.0602098047995
0.1031460961452	0.0605115639249

0.1032303677575	0.0606396383266
0.103319559464	0.0606810951628
0.1034146065903	0.0607435703187
0.1034885708222	0.0607512303556
0.1035662907067	0.0608790968117
0.1036322816304	0.0609781228456
0.1036880160887	0.0609794493906
0.1037736296106	0.0610168067593
0.103854235558	0.0612878603801
0.1039208678674	0.0613040933317
0.1040009675216	0.0613087088126
0.104101518149	0.0613637809413
0.1041877339426	0.0616303227692
0.104273445124	0.061714572178
0.1043588862408	0.0618927283529
0.1044362995652	0.0619486053984
0.1045275924077	0.0622282820061
0.1046108202451	0.0625156461447
0.1046986393584	0.062545612999
0.1047659806702	0.0625931013079
0.1048413747027	0.0626487188012
0.1049667233937	0.0631970311259
0.1050507245355	0.0632870821336
0.1051089989532	0.06335884252
0.105194585982	0.0635052129059
0.1052956423318	0.063573117565
0.1053731510566	0.063801398366
0.1054407847336	0.0638109575539
0.1055127992242	0.0638243868791
0.1055924521057	0.064027357735
0.1057036797322	0.0642290002164
0.105798598647	0.0643405685808
0.1058661172744	0.0644992046488
0.1059399449628	0.0645074427781
0.1060098000316	0.0645112827973
0.1060887963901	0.0645165203723
0.1061753321902	0.0645999404534
0.1063011075305	0.0650876793152
0.1064048012146	0.0651552242812
0.1064669899207	0.0652358245765
0.1065418230418	0.0652482810796
0.1066068769211	0.0652664770125
0.1066745380512	0.0652728889967
0.1067593768906	0.0654955101732
0.1068351527761	0.0656005228942
0.1069166992549	0.0656232821676
0.1069771685169	0.0657558460596
0.1070531629223	0.0658030243154
0.107134845889	0.0658390824343
0.1072385873225	0.0659534255322
0.1073405764751	0.0659555181978
0.1073991458314	0.0660469006012
0.1074763977248	0.0660501524553
0.1075773363999	0.0661691906452

0.1076811588561	0.0661904502664
0.1077930229896	0.0665144778456
0.1078842678851	0.0670033861109
0.1079763831818	0.0671725102467
0.1080538561087	0.0671955135865
0.1081163871702	0.0672323295552
0.1081979526683	0.0672767505132
0.1082783741405	0.0673767694021
0.1083754533364	0.0675438108274
0.1084718599807	0.0676214884977
0.1085396787639	0.0677097210285
0.1086187343079	0.0678627496648
0.1087185367317	0.0679459231469
0.1087785008673	0.0679973515398
0.1088403476412	0.0680222421602
0.1089298874168	0.0680807919043
0.1090352699092	0.0682175400194
0.1091087437685	0.0682500284866
0.1091895578541	0.0686432232004
0.1092516890807	0.0686638654541
0.1093165805899	0.0687669290356
0.1094524460765	0.0691699687886
0.10955333222	0.0692044005224
0.1096264951185	0.0692907439603
0.1096943362835	0.069598373508
0.1098002823339	0.0697605064292
0.1099070773451	0.0698469731935
0.1099939140991	0.0698524252347
0.1100774006341	0.0701570497417
0.1101485131134	0.0701763343752
0.1102189457239	0.0701776037751
0.1102830016264	0.0703338933543
0.1103633735513	0.0704499132703
0.1104463411188	0.070508218855
0.1105407326769	0.0705399929032
0.1105969464489	0.0707601975151
0.1107014846301	0.0709707451507
0.1107711064766	0.0710059843294
0.1108514765784	0.0712381791741
0.1109263374013	0.07125932074
0.1109972013386	0.071283489425
0.1110625820147	0.0714333047926
0.1111651559492	0.0716151351648
0.1112386955564	0.071709953428
0.1112975222713	0.0717538858977
0.1113836675263	0.0718732548466
0.1114600535855	0.0719870262696
0.1115406223503	0.0722286542085
0.1116511153201	0.0723373111963
0.1117443172616	0.0723641782991
0.111813220557	0.0723832433586
0.1118907320298	0.0724068717129
0.111963918265	0.0724404249077
0.1120241807797	0.0726232766321

0.1121291622429	0.0727448033403
0.1121781202939	0.0728006935808
0.1122967080675	0.0730198540399
0.1123826296141	0.0732799117941
0.1125275915157	0.0737149903429
0.1125778795746	0.0738193120152
0.112662446329	0.0738987754885
0.112728372125	0.0739106218457
0.1127961762089	0.0740915996834
0.1128873125611	0.0741270674893
0.112982726793	0.0742146682444
0.1130473179293	0.0742541317525
0.1131386479968	0.0742929877624
0.1132316596279	0.0744454019243
0.1132957449998	0.0745778692049
0.1133812990387	0.0747895005152
0.1134468553844	0.074920241155
0.1135285176589	0.0750879425809
0.1135926042414	0.0751081520499
0.1136808203331	0.0754214471172
0.1137773170304	0.0755931212016
0.1138303816675	0.0758733660368
0.1139044386847	0.0759041397561
0.1139876236222	0.0760146902169
0.1140662533556	0.0760205419113
0.1141468782543	0.0760543654806
0.1142407450331	0.076124106617
0.1143215596303	0.0762199689186
0.1144126629759	0.0763195789853
0.1144961377485	0.0764019954577
0.1145716583788	0.076534850765
0.1146387496313	0.0766198776805
0.1147266459326	0.0767761507998
0.1148322589304	0.0770646383809
0.1149180531172	0.0771105605987
0.114975311009	0.0771127371101
0.1150843638312	0.0773333203859
0.1151578849619	0.077357044774
0.1152445828716	0.0773574894979
0.1153033297564	0.0773653717372
0.1153870140219	0.0773806753003
0.1154476912013	0.0774661697729
0.1155427355731	0.0776903932494
0.1156205777888	0.0777653810996
0.1157201133935	0.077839053469
0.1158006990268	0.0778708284911
0.1158767421204	0.0778755619893
0.1159401881195	0.0779042300387
0.1160214154519	0.0782289671692
0.1160883007789	0.078295366555
0.1161732079743	0.0784571466691
0.1162513543316	0.0784835828033
0.1163279086901	0.0785537652039
0.1164344899297	0.0789889167485

0.1165412929768	0.0790776289456
0.1166112102442	0.0791106903367
0.116689461112	0.0792400825737
0.1168354941642	0.0793173226444
0.1169269277698	0.0794086076021
0.1170122429762	0.0794162086984
0.1170958578019	0.079470448786
0.1171715534971	0.0795210019946
0.1172595536239	0.079678425007
0.1173114302522	0.0797019280139
0.117405504837	0.0797416828679
0.1174822904924	0.0797554401802
0.1175994111647	0.0797714372031
0.1177177129252	0.0798892121595
0.1177972809158	0.0799752247034
0.1178794681545	0.0800098829059
0.1179559664476	0.0800224072726
0.1180629289471	0.0800364448917
0.1181317181102	0.080072314665
0.1182190854669	0.0800785412042
0.1182969224852	0.0802394088115
0.1183835840013	0.0802463594318
0.1184814594965	0.0804426325063
0.1185745563031	0.0804473903143
0.1186528546578	0.0805884889905
0.1187357468247	0.0805942747012
0.1188387743761	0.0808819267851
0.118916352445	0.0809170586652
0.1189836299787	0.0809705007762
0.1191086641177	0.0810482424355
0.1191863136209	0.0812282884428
0.1192867928793	0.0815538697746
0.1193764064037	0.0815956769357
0.1194663155217	0.0816571265711
0.1195555901836	0.0818065118555
0.1196228798867	0.0819817532021
0.1196984908642	0.0820402322527
0.1197646333555	0.0820637709176
0.1198169633745	0.0820798340818
0.1199038385217	0.082232138012
0.1200063865511	0.0823247256575
0.1200854158133	0.082526098098
0.1201834990234	0.0825370411716
0.1202517820798	0.0827158481748
0.1203311522306	0.082806202069
0.1204378374668	0.0829747342877
0.1205452008366	0.0833560617192
0.1206416055508	0.0835498158216
0.1207274843333	0.0836755943093
0.1208218900399	0.0837826552167
0.1209001987506	0.0839103891288
0.1209932286937	0.0840534480527
0.121071917706	0.0840856829514
0.1211517792623	0.0841428356693

0.1212360924535	0.0843521148099
0.1213523637908	0.084393366674
0.1214594459117	0.0846218303028
0.121531371917	0.0846788324728
0.1215960393584	0.08474279545
0.1216596196379	0.0847914407343
0.121736277234	0.0849310416298
0.1218391401342	0.0850783984383
0.1219096161174	0.085083095999
0.1219815584965	0.085133917048
0.1220853108583	0.0855700722641
0.122180739238	0.0859421954071
0.1223048050938	0.0861221559902
0.1223838122189	0.0861505780342
0.1224711927175	0.0864390143151
0.1226057780522	0.086711027657
0.1226833482573	0.0867111094508
0.1227545255743	0.0867358505958
0.1228231045133	0.0869310998387
0.1228854161293	0.0869647273102
0.1229740968719	0.0870864355394
0.1230549857565	0.0873176495355
0.1231456958449	0.0873284788863
0.1232019896656	0.0873479268355
0.1233016998686	0.0876322280003
0.1233945990148	0.0877589207788
0.1235001685468	0.0879502427359
0.1235611984615	0.0880447938405
0.1236641292529	0.0881458485279
0.1237586474313	0.0882709674063
0.1238562813576	0.0883537604802
0.1239412122966	0.0885374834649
0.1240074733639	0.0886699146542
0.1241071083338	0.0887488563107
0.1241623821836	0.0888320666335
0.1242513144656	0.0889368369576
0.1243340237584	0.088957422242
0.1244272330451	0.0889832362982
0.1245207560623	0.0890678851309
0.1246117113014	0.0891504980862
0.1247068060489	0.0892798815883
0.1248134992577	0.0895828798324
0.1248952276522	0.0896179100635
0.1249849243463	0.0896278268319
0.1250561794593	0.0897067469953
0.1251548470359	0.0897855709568
0.1252151003971	0.0899015812462
0.1253326599014	0.0899606548435
0.1254175812753	0.0900544563046
0.1255078046125	0.0901268092903
0.1255636974577	0.0901820359255
0.1256476664231	0.0902566746048
0.1257131906257	0.0902718997421
0.1258017952756	0.0904879485518

0.1259036008306	0.09054677419
0.1259713804666	0.0907251827694
0.1260462612292	0.0907677922664
0.126129123118	0.0908025423214
0.1261959589489	0.0908629978291
0.1262710648348	0.0909070519558
0.1263778355155	0.0909879326581
0.1264444144285	0.0909911237666
0.1264880966766	0.0910109842912
0.1265941815392	0.0911295977261
0.1266796742866	0.0911588725406
0.1267547315788	0.0911760640797
0.1268307780901	0.0913213774249
0.1269203236164	0.0914133571092
0.1270101973141	0.091430160028
0.1270939247838	0.0914350259669
0.1271754930542	0.0914440496497
0.1272593081316	0.0914604898038
0.1273467359775	0.091517217854
0.1274333365631	0.0915245242362
0.1275186664293	0.091581669541
0.1275993587274	0.0917268351328
0.127691089771	0.0917405189875
0.1277841978379	0.0917891866179
0.1278610134494	0.0919539487461
0.1279279605523	0.0921216497939
0.1280006965512	0.0921366679109
0.1280835880724	0.0921455853677
0.1281765950408	0.0922461957108
0.1282726528032	0.0922848031985
0.1283457657958	0.0923662213705
0.128431199271	0.0925861202267
0.1285293693705	0.0927002800525
0.1285948656103	0.0927398881077
0.1286772228331	0.0928761041908
0.1287643974486	0.0929745110514
0.128853267429	0.0930718442268
0.1289170464204	0.0932604372521
0.1290161702114	0.0932783762213
0.1290796838943	0.0934269570005
0.129132215404	0.0934773467274
0.1292370767945	0.0936004893608
0.1293180580908	0.0936200406273
0.1294179700511	0.0936248791795
0.1294880623328	0.0937300934702
0.1295477718709	0.093867261501
0.129622914526	0.0939375058623
0.1297303881367	0.0940078785288
0.1298329940283	0.0942530528651
0.1299060119318	0.0944805735363
0.1299788059539	0.0944830857287
0.130051270523	0.0945043457326
0.1300925726499	0.0945647271244
0.1301559286756	0.094573378369

0.1302388457294	0.0948735686183
0.1303064707744	0.0950100984635
0.1303773471098	0.0950125188392
0.1304544180134	0.0951812123133
0.1305457794451	0.0952790017282
0.1306369121644	0.0953169066676
0.1307043119598	0.0953260471815
0.1307660571236	0.0953301250692
0.1308571023768	0.0954960705178
0.1309269086075	0.09550301431
0.1310090375394	0.0957021504672
0.1310931206854	0.0957360082486
0.1311546301927	0.0957825380844
0.1312306953004	0.095829927723
0.1313286164583	0.0958420479025
0.1314099098432	0.0960338449539
0.1315132757021	0.0964890900587
0.1315885445881	0.0965500356298
0.1316513603373	0.096578639792
0.1317253404513	0.0965938564684
0.1317957890306	0.0967056663385
0.1318619141961	0.0967087596533
0.1319590336235	0.0967785069335
0.1320817531921	0.0968406196937
0.1321510523742	0.0968676031682
0.132235830725	0.09687905422
0.1323081753081	0.0969668461182
0.1323789443891	0.0970002989155
0.1324702105491	0.0972855384982
0.1325555992527	0.0975770077735
0.1326266240785	0.0976974821037
0.1327472094546	0.0977214080421
0.132814180403	0.0977237522832
0.1329245310016	0.0978958256852
0.1329965766972	0.097926506456
0.1330650791942	0.0979563021109
0.1331663445175	0.0981534388577
0.1332710076227	0.0984365046052
0.1333524608427	0.0985650669246
0.1334313418996	0.0985747768028
0.1334926376463	0.0985882676656
0.1335654255085	0.0987189586446
0.1336610548975	0.098748062806
0.1337465271156	0.0988396908243
0.1338331569918	0.098899587071
0.1338991627644	0.0989586481083
0.1339675893799	0.0990920839287
0.13407628815	0.0994510835842
0.1341386206912	0.0994687318426
0.1342121805771	0.0995027509673
0.1342639368856	0.0995209466249
0.1343557016594	0.0995731466548
0.1344441244061	0.0996168053062
0.134523011477	0.0996504127085

0.1346457638351	0.0996702886561
0.1347381306083	0.0997077271065
0.1348055920479	0.0998577046948
0.1348767357518	0.0999657238811
0.134965418622	0.1001124200738
0.1350409344807	0.1001811100653
0.135103610123	0.100242238457
0.1351621229832	0.1003294248586
0.135228715461	0.1003959336928
0.1352976832044	0.1004276437133
0.1353428626495	0.100504769724
0.1354196827196	0.1007243358799
0.135511132095	0.1007801557827
0.1355753332406	0.1008042183199
0.1356706561354	0.1008351396716
0.1357637434427	0.1008888565532
0.1358588093225	0.1008941150389
0.1359359761801	0.101024657655
0.1360476271884	0.1010274592114
0.1361007532222	0.1010288992081
0.1362034022255	0.1013314263658
0.1362797318534	0.1013572506273
0.1364186518281	0.1014075088203
0.1364848208644	0.1015220595148
0.1365612913689	0.101544057048
0.136659776544	0.1015523200776
0.1367390746515	0.1016159181002
0.136867410289	0.1018559011087
0.1369355626118	0.1018780244704
0.137017250918	0.1020907499511
0.1370844112853	0.1022526947773
0.1371817269989	0.102436048593
0.1372795787641	0.1024548662288
0.137388083529	0.1024956471668
0.1374654516697	0.1026807563118
0.1375475817559	0.1026934088621
0.13759194793	0.1027523554974
0.1376574673991	0.1028078835121
0.1377753832522	0.1028220399132
0.137861086135	0.1028751487161
0.1379511129755	0.1029209622907
0.1380134796236	0.1029256555481
0.1381131974602	0.1030036511742
0.1382088234912	0.1030145449099
0.1382901656114	0.1030284844955
0.138384014377	0.1031715651634
0.1384588659132	0.1035847911201
0.1385678188735	0.1036240435434
0.138655284433	0.1036636130514
0.1387475001305	0.1037835961079
0.1388152574642	0.1038494779258
0.1388978290581	0.1038640045458
0.1389660702823	0.1040605356977
0.1390745287561	0.1041396091008

0.1391824837372	0.1044906237465
0.1392620537412	0.1046399164192
0.1393196639317	0.1046769638062
0.1394067511464	0.1047911322111
0.1394972497838	0.1049210544441
0.1395881711465	0.1050305891576
0.1396516225145	0.105041202856
0.1397295943243	0.105161091593
0.1398052628631	0.1051692518204
0.1398859347579	0.1051806500613
0.1399935694172	0.1052908121332
0.1400760345229	0.1052948490811
0.1401529360726	0.1053990119488
0.1402255366521	0.1054594660574
0.1402981050341	0.1054771608867
0.140382338572	0.1058737037198
0.1404557539119	0.1060120629741
0.1405064193399	0.1060201892144
0.1406101570491	0.106216173201
0.1406944591259	0.106238721953
0.1407716930009	0.1062763530259
0.1408317182811	0.1063925433919
0.1409258797416	0.1064825650817
0.1410020807207	0.1066609463875
0.1410860026076	0.1066709852816
0.1411825551212	0.1067044209659
0.1412340401751	0.1067422713456
0.141325684646	0.1067810389545
0.1413940940692	0.1067854933558
0.1414904881479	0.1068261923286
0.1415562929753	0.106944002634
0.1416559003231	0.107202463413
0.1417559832816	0.1073201635879
0.1418186451389	0.1073515222282
0.1418829072682	0.1073958420094
0.1419854121546	0.1076034401278
0.1420795502587	0.1077352389194
0.1421517335275	0.1079489597396
0.1422000819965	0.1079755806097
0.1422797203231	0.1080914467452
0.1423478354941	0.1081198520129
0.1424214483943	0.1081562210826
0.142486962819	0.1081911974013
0.1425568714155	0.108220838645
0.1426405186261	0.1082409231356
0.1427195963311	0.1083833179718
0.1427997308474	0.1084408358169
0.142894262894	0.1085999534373
0.1429970067416	0.1087141566027
0.1430670264442	0.1087714044135
0.1431590158364	0.10883060585
0.1432412558186	0.1090582075198
0.143308572574	0.1091288300834
0.1433782893924	0.1091496441681

0.1434725961852	0.1093233844548
0.1435527297912	0.1097551544754
0.1436384453533	0.1097566975006
0.1437223035502	0.1099951580741
0.1438204190739	0.1100720011332
0.1438967654449	0.1100762204453
0.1439680466628	0.1102378929828
0.144077583466	0.110326772924
0.1441773749029	0.1105004571208
0.1442685684583	0.1107808841088
0.1443889173673	0.1110680127181
0.1444917300459	0.1112571735136
0.1445795843552	0.1113296525615
0.1446560984574	0.1113565788231
0.1447108215319	0.1114089567127
0.1447898503276	0.1114253739573
0.1448668589	0.1115356479224
0.1449477904948	0.1117371845481
0.1450532680324	0.1120074302548
0.1451327152106	0.112096227152
0.1452071425271	0.1121377959501
0.1452743462598	0.1122134385267
0.1453920083634	0.1123926074628
0.1454940283756	0.1123982614496
0.1455943774997	0.1125985262054
0.1456778345701	0.1126199965111
0.1457621370718	0.1129301842442
0.1458301119248	0.1129567891313
0.1459181570939	0.1131445196451
0.1460035044863	0.1131500566904
0.1460653057051	0.1131598698578
0.1461640761871	0.1133683314981
0.1462372424897	0.113464565114
0.1463460354052	0.1140203553143
0.146430989493	0.1141715544218
0.1465091358927	0.1141907331617
0.1465752729183	0.1142032634904
0.1466513602684	0.1142755384609
0.146747893158	0.1143232632095
0.14685070063	0.1145297315616
0.1469316312391	0.1145531371922
0.1470090268634	0.1146462746189
0.1471545794776	0.1146784077076
0.1472356980904	0.1147734849739
0.1473333569521	0.1147911500341
0.1473932468875	0.1148034603214
0.1474716279645	0.1149692934272
0.1475533638599	0.1150841231947
0.1476286659727	0.1152287653851
0.1476842731445	0.115245230526
0.1477835613111	0.1152907258699
0.1478950966652	0.1153101673952
0.1479632003007	0.1155689396147
0.14806149047	0.1156183301283

0.1481763426572	0.1159578627157
0.1482447709844	0.1160159167032
0.1483263344836	0.1160964362169
0.14840934693	0.1161843194982
0.1484936041966	0.1163471074914
0.148570472816	0.1163850729628
0.1486741959354	0.1164121116419
0.148776512114	0.1164565632794
0.1488605227728	0.1164674038428
0.1489191695072	0.1166348012094
0.1490077469252	0.1166467389021
0.1490914133752	0.1166990281079
0.1491914769667	0.1168961936962
0.149275328793	0.1169977247327
0.149335858931	0.1170289230884
0.1494245380454	0.1170558949673
0.1495283593239	0.1172386298118
0.1496042065024	0.1173305105987
0.1496749949477	0.117364111201
0.149778255365	0.117587318247
0.1498543798094	0.1176796772669
0.1499341695328	0.1177561426171
0.1500063366621	0.1178848746296
0.1500790037164	0.1179409791832
0.1501747074744	0.1179682056119
0.1502660949442	0.1179882901151
0.1503401112155	0.1180645705256
0.1504181053175	0.1181107529263
0.1504940871173	0.1181583491234
0.1505907022349	0.1183800467402
0.1506687932355	0.118561820892
0.1507494751409	0.1189314531335
0.150817778673	0.1189858868925
0.1509395398778	0.1190600866247
0.1510122148679	0.1191666491333
0.1511055444453	0.1191750807109
0.1511881497616	0.1193645178686
0.1512637856022	0.119454974412
0.1513482847645	0.1196106435971
0.1513942156213	0.1196264222571
0.1514719482861	0.1196529794907
0.1515413224738	0.1197628092898
0.1516035855561	0.1197683256823
0.1516660539665	0.1198298521794
0.1517161134908	0.1198889383634
0.1517824072933	0.1200441077166
0.1518626456148	0.1201676850227
0.1519315684177	0.1204659402013
0.1520522515233	0.1207760566306
0.1521293570305	0.1209989666439
0.1522073719932	0.121020878091
0.1523141702717	0.1211186369579
0.1523784025617	0.1211861686128
0.1524886025933	0.1213488944559

0.1525868272708	0.1215053504035
0.1526605011245	0.1216882462205
0.1527337936288	0.1217161765855
0.152853133529	0.1219469987639
0.1529347854035	0.1219949202249
0.1530019739781	0.1220735038212
0.1530719686987	0.1222372083892
0.1531510181412	0.122237812685
0.1532180969134	0.1223081840775
0.1532872414576	0.1223319856282
0.1533689588065	0.1223916855985
0.1534525179773	0.1225020901914
0.1535526662963	0.1227262467764
0.1536486670816	0.1227748807079
0.153755890715	0.1229725707371
0.153861106991	0.1230041559397
0.1539520551673	0.1230879975974
0.1540066672162	0.1231259366651
0.1540891964442	0.1231307517807
0.1541747634554	0.1231933231661
0.1542739052377	0.1234770927034
0.1543853360074	0.1239367492596
0.1544576569824	0.1241667743261
0.1545221910949	0.1242324424491
0.1546334092584	0.1246978225698
0.1547138063796	0.1249348786126
0.1548117919095	0.1249555123977
0.1548674228591	0.1250594149257
0.1549376032956	0.1250840974569
0.1549969807513	0.1251810480926
0.1550662888922	0.1252001377216
0.1551543732985	0.1255087543351
0.1552337740339	0.1255128492894
0.1553051934429	0.125531149889
0.155397885679	0.125568488762
0.1555825198255	0.1269246180457
0.1556539841518	0.1270714337164
0.1557388685789	0.1272444370008
0.1558194143911	0.1275116893971
0.1558759103309	0.1276107768611
0.1559428567363	0.1276909815552
0.1560060989396	0.1278151308797
0.1561307257821	0.1279378162572
0.1562110413386	0.1280443974083
0.1562804182629	0.1281132237802
0.1563694270682	0.1282439758713
0.1565014395305	0.1285348189168
0.1565881945423	0.1287407792451
0.156676604879	0.1287988034861
0.1567533133201	0.1289344616352
0.1568473595406	0.1289969398956
0.1569274903605	0.1290915247704
0.1569968167357	0.1291745692371
0.157061410749	0.1292591226715

0.1571398998483	0.1294892932801
0.1572172250167	0.1294991141541
0.157307072101	0.129704054468
0.1574046691135	0.1301135609607
0.1574649849707	0.1301371355226
0.1575660633049	0.1302464096887
0.1576375898836	0.1302503104846
0.1577317536388	0.130372345077
0.157811387167	0.1303839477574
0.1578616360861	0.1304354333004
0.1579465334668	0.1304932575335
0.1580204168012	0.1305582008193
0.1581005590496	0.1306622459236
0.1581635032787	0.1307135074659
0.1582706731051	0.1309000323572
0.158339415178	0.1309095075904
0.1584003932299	0.1311314431302
0.1584664279349	0.131147932235
0.1585206974657	0.1311798029756
0.15858876708	0.13125601098
0.1586621941086	0.1312857205239
0.1587247996482	0.1313581109432
0.1588228099629	0.1316497976707
0.1589021652851	0.1318088480461
0.1589681843753	0.1318872521008
0.1590378729251	0.1319089459806
0.1591011909367	0.1319698611606
0.1591632539731	0.1320145864587
0.159232894652	0.1320248719846
0.1593261341089	0.1320322849678
0.1593997869779	0.1321017619139
0.1594775294672	0.1322894262788
0.1595626886336	0.1323380243548
0.1596461944465	0.1323496421647
0.1597534573562	0.1324703155815
0.159831475187	0.1325173256309
0.1599232018621	0.1325920753333
0.1600277320255	0.1326667624656
0.1601121028301	0.1327121628993
0.1602044551503	0.1327242777744
0.1603124542722	0.1327499378511
0.1604097179298	0.1329342857518
0.1605114661068	0.1331174790384
0.1605919291647	0.1332094510923
0.1606790788504	0.1333254537173
0.1607724370364	0.133370081808
0.1608473671949	0.1336228450653
0.1609617000169	0.1336855842776
0.161036743534	0.1338790842935
0.1611053016445	0.1339996843601
0.1611879066417	0.1341317104904
0.1612660912943	0.1341772147442
0.161353215385	0.1342922185553
0.1614386027295	0.1343413775195

0.1615191527873	0.1343456349975
0.1615897737767	0.1343566376331
0.161670085842	0.1345173670507
0.1617690225723	0.1345468876624
0.1618347953249	0.1345614695023
0.1619180753656	0.1347797888834
0.162002336723	0.13485256717
0.1620759261032	0.1348778716888
0.162158475061	0.1348907832204
0.1622420416231	0.1349102495837
0.1622928403615	0.134921011668
0.1623819133292	0.1351034988823
0.1624609356802	0.135117829141
0.1625305499831	0.1352960511596
0.1626166906445	0.1355098434237
0.1627335571956	0.1357602053625
0.1627986854518	0.1357666220439
0.1628596134204	0.1358980615587
0.1629624126877	0.1360948959174
0.1630472925803	0.1361380798021
0.1631292967457	0.1361806668134
0.1632302544644	0.1362604239411
0.1632828726317	0.1363031251478
0.1633464435391	0.1363166240113
0.1634497505306	0.136513595335
0.163533816532	0.1365586000088
0.1636277755771	0.1365593735957
0.1637290821053	0.1365945861687
0.1638056598529	0.1365993550484
0.1638838940518	0.1366006718399
0.1639610525706	0.1367360911457
0.1640096504857	0.1368712223266
0.1640899924411	0.1368903769808
0.1641596662164	0.1369213237791
0.1642528956441	0.1369905005568
0.1643366872598	0.1370542796158
0.1644101279226	0.137247260554
0.1644916369505	0.1373996424952
0.1645567776613	0.1376759553996
0.1646517428713	0.1377686670369
0.1647459167798	0.1380220028908
0.1648080252051	0.1380825531574
0.1649032489752	0.1381243640197
0.1649995758008	0.1382267679724
0.1650842937781	0.1383479085774
0.1651639333461	0.1384678666111
0.1652394398002	0.1384824886437
0.1653182427804	0.1384830284521
0.1654213831413	0.1388452668594
0.1655063439892	0.138874219245
0.1655651035392	0.1389174097315
0.165685188853	0.1390877304308
0.1657504090979	0.1391861687376
0.1658003842016	0.1392267268016

0.1659002669887	0.1392361911745
0.1659701363921	0.1392803955495
0.1660638650274	0.1393918790921
0.1661480357999	0.1394496777487
0.1662285896623	0.1394525003183
0.1662909621921	0.1394742065388
0.1663532083734	0.1394813754471
0.1664321530965	0.1395054375535
0.1664897155676	0.1395291548461
0.1666009468149	0.1395876122982
0.1666945692196	0.1396343939902
0.1667899229073	0.1398187411825
0.1668854147471	0.1401176118776
0.1669662059472	0.1403557546701
0.1670538890397	0.1403826686066
0.1671483642672	0.1405361192808
0.167199899512	0.1405396692167
0.1672938121795	0.1406053064721
0.167365825734	0.1406076090215
0.1674418917091	0.1408118976249
0.1675125567895	0.1408781624687
0.1675792775748	0.1408884297128
0.1676362434651	0.1409776920386
0.1677260739471	0.1411109069731
0.1677975816874	0.1412187917295
0.1678571808012	0.1414927188754
0.1679448730452	0.1416916833742
0.1680233336738	0.1419231157872
0.1681046817904	0.1419468764116
0.1681929283432	0.1419564073767
0.1682804268169	0.1420195991857
0.1683367165531	0.1421377040739
0.1684542461097	0.1421499540669
0.1685180828435	0.1421622683159
0.1685993669862	0.1421937332029
0.1686809565217	0.1422281925384
0.1687828472909	0.1423741851635
0.1688775144943	0.1424208146832
0.1689557917041	0.1424785271092
0.1690449980216	0.1425341519077
0.1691108361508	0.1425620635584
0.1692002497036	0.1426790346743
0.169280138711	0.1427529682934
0.1693631278203	0.142781813965
0.1694407708351	0.1428561511182
0.1695597055458	0.1428856584721
0.1696554766042	0.1430534002903
0.1697317351583	0.1430640272307
0.1698094949324	0.1431037082195
0.1698952841816	0.1431687966157
0.1699630155724	0.1431829512796
0.1700378497802	0.1432309592411
0.1701032190001	0.1432320458843
0.1701703287531	0.1432736522344

0.1702306430797	0.1432992085497
0.1703511724317	0.1434295751572
0.1704237018033	0.1437663315052
0.1705081739516	0.1438269280077
0.1705910174288	0.1439059546738
0.1706724040906	0.1440680714442
0.1707510808523	0.144068199043
0.1708485911628	0.1442611480688
0.1709024328541	0.144307017289
0.1709962156637	0.144335465924
0.1710731586879	0.1443494303319
0.1711424655843	0.1444771933797
0.1712426892821	0.14457236441
0.1713102062286	0.1446293411303
0.1713893898513	0.1446424442325
0.1714513317853	0.1446496380263
0.1715354491535	0.1448248065704
0.1716267091652	0.1449590177618
0.1716890282539	0.145083602597
0.1717787125753	0.1452018184073
0.1718747659389	0.145288593769
0.1719590083072	0.145417339417
0.1720487140479	0.1455464575612
0.1721601217842	0.1459877580234
0.1722635311311	0.1460589794302
0.1723446392539	0.1460598491638
0.1724102103578	0.1461699891
0.1724764685261	0.1461765895112
0.1725562736676	0.1461915508641
0.1726436176521	0.1463244028479
0.1727155083068	0.1463357855494
0.1727852877287	0.1465427418451
0.1728627169406	0.1465552955186
0.1729522112879	0.1467058608845
0.1730494675002	0.1467587616279
0.1731316555019	0.1467963326025
0.1731997232151	0.1469240433035
0.1732812439564	0.1469301250149
0.1733329608868	0.1470869919654
0.1734063160954	0.1472961625315
0.1734597919903	0.1473041480359
0.1735165719552	0.1473768273302
0.1735991702322	0.1474140462732
0.173686912504	0.1476241891418
0.1738115398506	0.1476388739785
0.1738700099264	0.1476487761247
0.1739462912772	0.1477528710365
0.1740417846629	0.147839954918
0.1741862250177	0.1484065965033
0.1742486936587	0.1484067797523
0.1743519348311	0.1484852503927
0.1744261748448	0.1485520089087
0.1745306547587	0.1486783258143
0.1745830172012	0.14872791916

0.1746495393824	0.1487742609586
0.1747084612315	0.1487866921695
0.1747960517772	0.1489632940912
0.1748644384693	0.1489791258681
0.1749284886448	0.1490038787435
0.1749699901993	0.1490218196502
0.1750357401731	0.1490784753661
0.1751284925019	0.1493438188738
0.1752242306651	0.1497332603819
0.1752978783685	0.1498873408869
0.1753816899477	0.150276842404
0.1754432439292	0.150317312011
0.1755325948907	0.1504239620397
0.1756093796391	0.1504364786348
0.1756837026936	0.150551605302
0.1757399097612	0.1506171358312
0.1758221669287	0.1508516041515
0.1759015894005	0.1509374474185
0.1759846476874	0.1512041152074
0.1760627620038	0.151262939067
0.17613111383	0.1512922763933
0.1761900165042	0.1513298849626
0.1762518479773	0.1513801415495
0.1763461340404	0.1516718081779
0.1764315028177	0.1517079587606
0.1765082287934	0.1518139415889
0.1766278337435	0.1520683774022
0.1766964004804	0.1521166157701
0.1767915932609	0.1521865597528
0.1768701228457	0.1522414717926
0.1769526178489	0.152426110125
0.1770306949347	0.15254990319
0.1771141886644	0.1526536040787
0.1771793377004	0.1526541773955
0.1772664015495	0.1527116638479
0.1773463553809	0.1528228802415
0.1774507953063	0.152854364227
0.1775338398866	0.1530577260778
0.1776552914024	0.153180646575
0.1777439433024	0.1532543736523
0.1778105672701	0.1532671063457
0.1779214082731	0.1533224593836
0.1780133894479	0.1536157358695
0.1781053081396	0.1536888471342
0.1781842303195	0.1537572311841
0.1783014232895	0.1539457139301
0.1783924584964	0.1543092348828
0.1784488911303	0.1543281093552
0.1785437416627	0.1544325732389
0.1786059127024	0.1545846091322
0.1786775623342	0.1547045823577
0.178771272712	0.1548232226737
0.1788403469796	0.1548307715435
0.1789176107681	0.1548732136186

0.1789822876367	0.1549671106314
0.1790743767096	0.1550368516759
0.1791866476834	0.1551365343201
0.1792644751825	0.1551798963613
0.1793251159878	0.1552265170656
0.1793927913012	0.155247426036
0.1794963149312	0.1553751215916
0.1795808176491	0.155662707434
0.179692434013	0.1557213523381
0.1797465374413	0.1557686460277
0.1798146693109	0.1558247598281
0.1799324558908	0.1560570983323
0.1800142146131	0.1565609083905
0.1801014770445	0.1566657259414
0.1801914255782	0.1566828535104
0.1802866230183	0.1567005486393
0.180368765382	0.156753337721
0.1804361280226	0.1567709776552
0.1805303970844	0.1568462274308
0.1806132974208	0.1569520259595
0.1806850920803	0.1569681907393
0.1807653490258	0.1569790005098
0.1808617523945	0.1572963370367
0.1809248070875	0.1573427789745
0.1809899442894	0.1573851563204
0.181070995734	0.1574369569787
0.1811494570391	0.1574429467332
0.1812778797208	0.1574646564923
0.1813344489641	0.1575056529638
0.181390806754	0.157569437093
0.1814773144204	0.1575802448412
0.1815817667343	0.1579634407232
0.1816687941238	0.1580425978025
0.1817538255846	0.1580515426939
0.1818608149544	0.1584023996872
0.1819226753685	0.1584153797738
0.1820072317553	0.1586601508537
0.1820962991006	0.1587813602208
0.1821717706028	0.1588323467851
0.1822528788872	0.158894917358
0.1823211557074	0.1589908772196
0.182434604101	0.1590938965412
0.1825189435326	0.1593239361253
0.1825759966199	0.1593596369029
0.1826576754394	0.1593755362824
0.1827345683427	0.1596088206377
0.1828144716043	0.1597954896139
0.1829303918373	0.1598561683884
0.1829917393747	0.1598800716331
0.1830460959147	0.1598892892884
0.1831268742405	0.1600765829464
0.1832309419931	0.1601459579977
0.1833032165637	0.16016157459
0.1833662182067	0.1602582807489

0.1834280615278	0.1602872481927
0.1835272627925	0.1603140963581
0.1836038379243	0.1603392405104
0.1836697793567	0.1604346913862
0.1837232401608	0.1605583310427
0.1837776218854	0.1606192671738
0.1838718344401	0.1607191738531
0.1839288443489	0.1608679272634
0.1839969163905	0.160904493302
0.184059121003	0.1610055589502
0.1841569896876	0.1612457833308
0.1842696244132	0.1613623456293
0.1843599493964	0.1613817637902
0.1844394467441	0.1614853944379
0.1844851641472	0.1615261867069
0.1845902077976	0.1617556463222
0.1846653929337	0.1618730767759
0.1847667591674	0.1619661183762
0.1848531694051	0.1619953747421
0.1849548444394	0.1621809599853
0.185033827181	0.1622871762809
0.1851284053045	0.1623158273789
0.1852198712278	0.1623235232052
0.1853140014458	0.1625650730697
0.1854080181606	0.1625975485068
0.1855047530719	0.162643082504
0.1855685262691	0.1626520750015
0.1856865814083	0.1626975843899
0.1857558696724	0.1627316294456
0.185850277245	0.1629520583605
0.1859224335387	0.1629708514805
0.1860137505114	0.1631895647224
0.1860877577634	0.1632126280381
0.1861870927841	0.1633682644453
0.1862950085242	0.1633998904846
0.1863976369986	0.1634480543534
0.186475048283	0.1634499762374
0.1865504970244	0.1634589503681
0.1866538973389	0.1635101823309
0.1867077314616	0.1635342864222
0.1867726473641	0.1635677093499
0.1868702488473	0.1636896192702
0.1869469499951	0.1638067372289
0.1870272659603	0.1638321288629
0.1871235723625	0.1638370707979
0.1871929985247	0.1639052643589
0.1872503521739	0.1639741854373
0.1873498373331	0.1639907157217
0.1874414805792	0.1640656609522
0.1875093603799	0.1641374898918
0.187600517517	0.1641456918453
0.1876904016143	0.1643598851129
0.1877678453273	0.1644625654048
0.1878680822988	0.1644855695603

0.1879815907132	0.1646820156242
0.1880489429258	0.1647806223674
0.1881309962854	0.1647864577514
0.188204348273	0.164916596517
0.1882902508803	0.1651129214632
0.1883284278668	0.1651191426592
0.1884040996684	0.165181330861
0.1884821547858	0.1652650181195
0.1885781355821	0.1655738068514
0.1886719116458	0.1656942076021
0.1887425687848	0.1659014175414
0.1888256118092	0.166172333526
0.1889064306916	0.1662629064986
0.1889952706925	0.1663887693792
0.1890798564255	0.1666154040583
0.1891335206481	0.1666308799896
0.1892009728203	0.1666523308656
0.1892484275967	0.1666768255527
0.1893060207215	0.1667579322107
0.1893899380323	0.166774299374
0.1894696486384	0.1668208443387
0.189571311268	0.1668519227584
0.1896463536696	0.1668572052802
0.1897185433795	0.1668671925465
0.189791503448	0.1668818641702
0.1898575017344	0.166962305185
0.1899208379901	0.1670304616229
0.1899908254993	0.1671984722063
0.1900708658203	0.1672195349411
0.1901312528115	0.1673311352747
0.1902534417153	0.1673438449442
0.1903103008591	0.1673635186675
0.1903968186252	0.1673878121632
0.190442112865	0.1673914953096
0.1905172332304	0.1674167571881
0.1906130839403	0.1675216414572
0.190693550725	0.1675636561591
0.1907793504434	0.1679646920319
0.1908953895429	0.168129870619
0.1909577904169	0.1683040876823
0.1910204052463	0.1683762020032
0.19107496759	0.1684024497666
0.1911744124672	0.1684980918417
0.1912330597248	0.1685202093265
0.1913043703025	0.1686410591246
0.1913901814898	0.1686924754363
0.1914719696725	0.1686963655463
0.1915782159778	0.1687177280081
0.1916548586743	0.1687677805791
0.1917401012352	0.1689103625259
0.1918018527401	0.1689589872099
0.1918979252234	0.1690188866971
0.1919950503549	0.1693977110843
0.1920740561389	0.1694967582137

0.192149737692	0.1696329686535
0.1922572775865	0.1698222744449
0.1923302433954	0.1698676644144
0.1923899072027	0.169904478515
0.1924700454798	0.1699160976415
0.1925489155168	0.1700005791475
0.1926611344468	0.1702758177613
0.1927612679255	0.1705004909817
0.1928447609877	0.1705349422893
0.1929126200505	0.1705917282647
0.1930052041343	0.1706382181476
0.1930804123938	0.170666965924
0.1931619807799	0.170725883667
0.1932325770211	0.1708092381225
0.1933116374548	0.1709553155827
0.1933962719208	0.1709684894871
0.1934672394427	0.1710708003723
0.193567100064	0.171098322558
0.193619914452	0.1711112660903
0.1937252365739	0.1714416977064
0.1938078068133	0.1716355205752
0.1938905469145	0.1717191375449
0.1939703434098	0.1719762812221
0.1940473595535	0.1719787407376
0.194136172991	0.1720804675564
0.1942139876121	0.1721243368998
0.1943111805639	0.172164914422
0.1944209429176	0.1723107274118
0.1945053620045	0.1724774595802
0.1945881170175	0.1725830182412
0.1946838765269	0.1726204635284
0.194769191899	0.1726501193684
0.1948500967855	0.1729251177498
0.1949317991254	0.1731000459657
0.1950282804157	0.1733014778572
0.1951209230717	0.1733643021649
0.1952041287698	0.1735443531506
0.1953120425391	0.1739667669534
0.1953804128835	0.1740823396359
0.1954814700092	0.1741356955826
0.1955454900476	0.1744592638841
0.1956252881191	0.1745031863067
0.1957089636066	0.1747685415236
0.195781943097	0.1748045024267
0.1958416934098	0.1748392343501
0.1959005055106	0.1750513954931
0.1959833584391	0.1750930995177
0.1960906602165	0.1751999924489
0.1961785103938	0.1755353541829
0.1962709466363	0.1758698401639
0.1963319764754	0.175872444798
0.1964363347098	0.1758740654213
0.1965839371763	0.1763973048583
0.1966868999442	0.1765649732329

0.1967579492687	0.1765820865578
0.1968633015662	0.1768242428791
0.1969282098799	0.1770861329067
0.1970015681434	0.1771095198066
0.1970868153677	0.1771776964082
0.1971599144649	0.1773056356837
0.1972732357859	0.1773275289947
0.1973600773205	0.1773819274833
0.1974660319328	0.1774512290997
0.1975468194258	0.1775217222063
0.1976246451044	0.1775387033508
0.1977298662737	0.1778328074509
0.1977981904669	0.1778629584251
0.1978856460374	0.1782100166526
0.1979867310593	0.1782660806056
0.1980898933914	0.1785280919605
0.1981380022491	0.178537358262
0.198236364453	0.1786234287825
0.1983111025515	0.1786838456099
0.1983922172225	0.1787671858398
0.1985015397714	0.1787799522113
0.1985648706995	0.1788674882965
0.1986577577626	0.1789433623281
0.1987727302538	0.1792239250678
0.1988413357028	0.1792611146322
0.1989219115388	0.1793902831023
0.1989935952052	0.179536427819
0.1990597860117	0.1796757621084
0.1991601321334	0.1797566360512
0.1992249147882	0.1799288832557
0.1993028107867	0.1799352015364
0.1993694730811	0.1799863466965
0.1994494375154	0.1800016571318
0.1995320108791	0.1800039367844
0.1996057581369	0.1800241388549
0.1996911703485	0.1800596183412
0.1997460180137	0.1802424762883
0.1998092641415	0.1803162641752
0.1999068152816	0.1805021715828
0.1999807672296	0.1806013582519
0.2000556000478	0.180960367578
0.200156921708	0.1810047895704
0.2002345033387	0.1810409947364
0.2003150563348	0.1810681553441
0.2003846366228	0.1811327908332
0.2004673797618	0.1813567531684
0.200540488744	0.1814416039954
0.2006298664884	0.1816033407165
0.200698603591	0.1816551522029
0.2008010963093	0.1818457213456
0.2009004409224	0.181858893543
0.200974327588	0.1818770603503
0.2010742532958	0.1820456421382
0.2011879758617	0.182113337638

0.2012831540592	0.1822425358211
0.2013666326451	0.1822950061642
0.2014550269458	0.18250660748
0.2015397812437	0.1828108466316
0.201629856691	0.1829995663842
0.201703875653	0.1830291078076
0.2017660246007	0.1830571085934
0.2018811932745	0.1831252062814
0.2019750263924	0.1831658894325
0.2020589917311	0.1832191703877
0.2021523549934	0.1832427424474
0.2022435040426	0.1833952333119
0.2023158289143	0.1835952682889
0.2024097148591	0.1836485301889
0.2024876051405	0.1837291789427
0.2025714563863	0.1838324494416
0.2026712946902	0.1838484740162
0.2027537665165	0.1838970601825
0.2028441119615	0.1839477648018
0.2029453946721	0.18428954667
0.203029549538	0.1844767020449
0.2031525817226	0.1846302190516
0.2032252991209	0.1847211612268
0.2032971673621	0.1847391823686
0.2033959312199	0.1847846160808
0.2034638333859	0.1849144725927
0.2035499955672	0.1849147132908
0.2036301584236	0.1849247263323
0.2037402400237	0.1849680185316
0.2038077159734	0.1851010518761
0.2038986294943	0.1852919288239
0.2039811975334	0.1853313729531
0.2040437219479	0.18537175508
0.2041149270301	0.1854940678111
0.2041857081439	0.185539265848
0.2042858380883	0.1856657656473
0.2043627152031	0.1856928136851
0.2044483496393	0.1857975777758
0.204545575346	0.1859995538782
0.204600450421	0.1860040761478
0.2046933554071	0.1860492408687
0.2047739375681	0.1860591120432
0.2048998083539	0.1860684762255
0.2049947940926	0.1862822767131
0.2050552561718	0.1863437944469
0.2051432338926	0.1864897301634
0.2052376768843	0.1866795939561
0.2052956158306	0.1866817845741
0.2053933984639	0.1866884939157
0.2054931734699	0.1866958272622
0.2055684237416	0.1868689206596
0.2056311190685	0.1869305716817
0.2057342022024	0.1869926389812
0.2058177639577	0.1870869400438

0.205913512366	0.1871529497603
0.2060405601949	0.1876019521638
0.2061375505586	0.1879329167476
0.2062537737666	0.1880317754357
0.2063533017813	0.1880341366132
0.2064880789988	0.188349766559
0.2065953585341	0.188697008217
0.2066601592693	0.1888298387456
0.2067406473601	0.1888464737957
0.2068298802373	0.1890048071234
0.2069320451803	0.1891044371653
0.2070342426415	0.1893567427455
0.2071164824673	0.1893738691691
0.2072145306813	0.1896716732394
0.2073191083314	0.1902204830512
0.2074026533306	0.1903032122913
0.2075153683567	0.1906310406564
0.2076304246189	0.190811951233
0.2077283762324	0.1909376022532
0.207798688604	0.1909887424247
0.2078779846934	0.1911856963211
0.2079363777283	0.1911861396323
0.2080687167954	0.1912482213675
0.2081189630646	0.1912588850547
0.2081970446251	0.1914604758409
0.2082688621096	0.1915004813711
0.2083486327335	0.191815417071
0.2084103917023	0.191830489488
0.2085017344155	0.1920875140285
0.2085679205401	0.1921764491497
0.2086352876552	0.1922157393582
0.2086959936887	0.1922382240138
0.2087818874752	0.1924818543357
0.2088606925366	0.1926683095035
0.2089396788153	0.1928741646419
0.2090081631944	0.1928912911017
0.2091138750484	0.1931217134022
0.2092221536845	0.1934779963934
0.2092978639552	0.1935246946479
0.2093529935689	0.1935934620309
0.2094290487082	0.1936802623899
0.2095039819121	0.1940621379208
0.2095697500726	0.1940993871353
0.2096754391966	0.1943489752667
0.2097527020821	0.19435402323
0.2098287907147	0.1944228647186
0.2099239149112	0.1944519332999
0.2100076695899	0.1945473627683
0.2100819586334	0.1945568557732
0.2101643920553	0.1949606736172
0.2102313573775	0.1949861363718
0.2103123322651	0.1950163353146
0.2103735300112	0.195021311276
0.2104574274065	0.1951701168572

0.2105321000153	0.1952138592276
0.2106174997888	0.1952634142994
0.2106976273694	0.1953187238762
0.2107565997019	0.1954202418207
0.2108230158731	0.1955143735901
0.2109283155769	0.1957763719331
0.2109953833786	0.1957882714588
0.2110836677466	0.1958929442707
0.2111604637921	0.195993184137
0.2112504413065	0.1961443792534
0.2113236407912	0.1962009245154
0.2113909108545	0.1962374813583
0.211482295428	0.1962680601235
0.2115777080844	0.1963475065764
0.2116695321532	0.1964752037203
0.2117585065052	0.1966218026348
0.2118093689788	0.1966392156333
0.2118694805796	0.1966488557089
0.2119437068182	0.196663352927
0.2120181916527	0.1968974876826
0.2120663623967	0.1969066790491
0.2121274080115	0.1969373478309
0.2122047928759	0.1969838232668
0.2122780653087	0.1969888531133
0.212353774042	0.1969973234673
0.2124177526492	0.1970115263375
0.2124982068806	0.1970639560208
0.2125668807114	0.197068404747
0.2126614872787	0.1970714153472
0.212728152178	0.1970775698246
0.2128314527569	0.1971816348313
0.2128986031877	0.1973255919193
0.2130000773773	0.1975497879301
0.2130530472666	0.1975876536405
0.2131442643087	0.1976496700263
0.2132420536378	0.1978356886941
0.2133074568852	0.1978685022787
0.2133872938573	0.1978923920012
0.2134701213627	0.1981933291098
0.2135361793753	0.1982874763856
0.213607417953	0.1983296630406
0.2137004350874	0.1984354569458
0.2137964896367	0.1986229501961
0.2139077776343	0.1986298633889
0.2139921238659	0.1986575765988
0.2140789540034	0.1987512243987
0.21416590103	0.1987789567253
0.2142157407805	0.1988084254401
0.2142946091588	0.1989103967579
0.2143642237659	0.1989654205622
0.2144745987283	0.1992081191714
0.214568932352	0.1993214959327
0.2146527725918	0.1994853857524
0.214714801149	0.1996690465739

0.2147638271442	0.1997250540984
0.2148407389763	0.1998152153513
0.2149152401893	0.1998730623226
0.21497507647	0.1998866498988
0.215066612444	0.2000266445443
0.2151597824978	0.2002301743521
0.2152383400233	0.200230524225
0.2153318400469	0.200385226855
0.2154439156464	0.200690119386
0.2155304241731	0.200702013465
0.2156313568526	0.2007206708815
0.2157083707755	0.2009692061516
0.2158137171929	0.2011344858779
0.2159308590711	0.2012437164847
0.216009283699	0.2012449294984
0.2161018829155	0.2014195452529
0.2161840837287	0.2016142978898
0.2163047467508	0.2017759432012
0.2163823992707	0.2019258730418
0.2164677979381	0.2019758236985
0.2165632810167	0.2020174911175
0.216653703984	0.2020590443038
0.2167340796092	0.2021848123964
0.2168113306707	0.2022109705025
0.2168578997937	0.2022328315914
0.2169504514992	0.2022525279208
0.2170702326127	0.202807645509
0.2171446466749	0.2028643736456
0.2172245316142	0.2029820342747
0.2173420882409	0.2031151803065
0.2174206309603	0.2031710909757
0.2174981912653	0.203227376097
0.2175931431553	0.203483238941
0.2177053230017	0.2037372578961
0.2177920857681	0.2037876932914
0.2178681041673	0.2038839090056
0.2179483014737	0.2038870259352
0.2180417558364	0.2040380553209
0.2181108516151	0.2041193690911
0.2181809782135	0.2042963244188
0.2182490107935	0.2045325923585
0.2183424234913	0.2046928603477
0.2184500133435	0.2048843775652
0.2185190953577	0.2051667695023
0.2186089657103	0.2052924759555
0.2187060964134	0.2055051020512
0.218782083087	0.2056249030805
0.2188892711328	0.2058308359474
0.2189816501621	0.2059966624416
0.2190615133051	0.2060329261522
0.2191408632702	0.2062614855022
0.2192362292875	0.2063520547648
0.2193216989564	0.206392570877
0.2194272133857	0.2064802135636

0.2194963485006	0.2066179374384
0.2195773801297	0.2068881041991
0.2196810576476	0.2069189216538
0.2197622408121	0.2069570771426
0.2198614715819	0.2069614810728
0.2199363272912	0.207259787873
0.2200158438845	0.2074768930575
0.2200889561611	0.2077147594176
0.2201671004668	0.2077260949066
0.2202248900919	0.2078638268038
0.2203063760494	0.2080428604664
0.2203683463394	0.2080607309991
0.2204613694263	0.2082560759686
0.220524743477	0.2082925551553
0.2205956398196	0.2083723612585
0.2206994181543	0.208501062109
0.220774335927	0.2085606673516
0.220846106781	0.2086333567424
0.2209204007086	0.2088671877129
0.2209666598322	0.2089083919551
0.2210647198127	0.2089595728875
0.2211281310161	0.2089789689344
0.2212092396722	0.2091083922672
0.2212731485544	0.2092112300749
0.2213746736084	0.2093224693591
0.2214544949048	0.2093234884243
0.221543756138	0.2094043546032
0.2216722120231	0.2094189657019
0.2217432709266	0.2095231640018
0.2218500618438	0.2098847281112
0.2219221611104	0.2100293896734
0.2220401432689	0.2101156322463
0.2221256482704	0.210334472363
0.2221898049746	0.2105134871545
0.2222854457725	0.2106437782388
0.2223871999332	0.2106810035229
0.2224765816237	0.210868778803
0.2225867935503	0.2109181503729
0.2227086797644	0.2112704646722
0.2227885890537	0.2113558522659
0.2228783604467	0.2114037801474
0.2229542977121	0.2115547883245
0.2230289617473	0.2115576337386
0.2231059341365	0.2116287501087
0.223199660182	0.2116485688997
0.2232709734352	0.2117477547933
0.2233524845372	0.2118127942329
0.2234104377045	0.2118439381635
0.2234716109313	0.211924888855
0.2235513836074	0.2121032872498
0.2236196967288	0.2121976126884
0.2237133479695	0.2122614872403
0.2238037086596	0.2122931684313
0.2238802865118	0.2123458246124

0.2239533160663	0.2123754449341
0.2240210292577	0.2124129684841
0.2240935408644	0.2125020065046
0.2241909967672	0.2125943576646
0.2242801633883	0.2128440061261
0.2243979463598	0.2132147796491
0.2244648759464	0.2133215071289
0.2245824645429	0.2142226669193
0.2246701741606	0.2142980625703
0.2247575449426	0.2143480236704
0.2248268566382	0.2143662985114
0.2249021317493	0.214525443917
0.2250056902499	0.2146843209958
0.2250921455568	0.2148266801765
0.2251805489639	0.214951979748
0.2252715656793	0.215015292581
0.2253614077664	0.2150244422789
0.2254483276384	0.2150917814937
0.2255200245155	0.215186334785
0.225602168763	0.2153067349999
0.2256736202767	0.2153321786022
0.2257761694868	0.2159116884695
0.2258533230438	0.2159135746591
0.2259077903967	0.2159843592638
0.2259842861314	0.2159913588042
0.2260633754542	0.2160063031369
0.2261511492033	0.2160705732005
0.2262598731098	0.2162978744957
0.2263595502734	0.2166800024978
0.2264892220713	0.2170599888425
0.2265791943445	0.2172121585217
0.2266365500459	0.2172154285402
0.2267137152745	0.217447841668
0.2268011563215	0.2174952933659
0.2268642185979	0.2175396201094
0.2269472514773	0.2176783389167
0.2270552497286	0.2177716818856
0.2271230372962	0.2177781990401
0.227214484625	0.2179876771338
0.2272885756989	0.2180382110351
0.2273693593246	0.218209471808
0.2274673301887	0.2182487924759
0.2275268658056	0.2183839697965
0.2275886593167	0.2184024685688
0.2276497943944	0.2184214188589
0.2277051246499	0.2185108424538
0.2277917078724	0.2187516577509
0.2278659003605	0.2187925018162
0.2279695467002	0.2188110806434
0.228026752994	0.2188259292839
0.2281006980571	0.2189418255581
0.2281895559675	0.2191951517955
0.2282395496457	0.2193542724341
0.2283092208328	0.2194186589209

0.2284094253118	0.2195085059712
0.2284811657458	0.2196620828262
0.2285421522785	0.2197116596237
0.2286155623252	0.2197123548083
0.2286672551134	0.2197449241088
0.2287614938954	0.2197942645875
0.2288350052481	0.2201597171148
0.2289178212244	0.2202792273109
0.2289805007004	0.2204737717545
0.2290595190769	0.2207115679926
0.2291361107541	0.2207636991876
0.2292179089476	0.2207926466396
0.229301029649	0.2212290261108
0.229387812958	0.221301472596
0.2294980543691	0.2215447416223
0.2295863817694	0.2216828676456
0.2296737515333	0.2217193119227
0.2297342997142	0.2217362365108
0.2298425784149	0.2219655472452
0.2299256800647	0.2219786327454
0.2299994124403	0.2221331328196
0.2300858114782	0.2223298509497
0.2301645329953	0.2223457361433
0.2302542620492	0.222457005422
0.2303355267083	0.2225000467556
0.2304042272085	0.2225394652496
0.2304730708792	0.2228768899939
0.2305785946508	0.2229722773758
0.2306757997879	0.2230412388654
0.2307250807329	0.2230970580962
0.2308088816243	0.2231068027991
0.2308751560412	0.2231997592997
0.230940603974	0.223253907266
0.2310292093268	0.2233471015739
0.2311111121445	0.2233920269676
0.231223412086	0.2237143616287
0.231286770259	0.2237649897495
0.2313593713224	0.2237970084102
0.2314506410948	0.2238839210147
0.231520872715	0.2238966576164
0.2316053626935	0.2239461183847
0.2317084057836	0.2240456294743
0.2318159872295	0.224482423845
0.2319068009137	0.2246304717836
0.2319731984685	0.2246404702418
0.2320544315046	0.22473261086
0.2321366646858	0.2247515463934
0.2322257311038	0.2249726042148
0.2323292293956	0.2250560697665
0.2324015504926	0.2250891121551
0.2324633186186	0.2251091605791
0.2325673327747	0.2251194089088
0.2326269227098	0.2251461426842
0.2327042304359	0.2251600422866

0.2327716867847	0.2252228299154
0.2328932780446	0.2252784369725
0.2329619736585	0.2253111537649
0.2330501087348	0.2253546464939
0.2331378082944	0.225472819604
0.2332205632274	0.2254775885726
0.233276643361	0.2255100786572
0.233339312557	0.2256213291122
0.2334113195853	0.2256748437656
0.2335163208761	0.2258500242025
0.233601905975	0.2258609915732
0.2337037914719	0.2261106224597
0.2337619159796	0.2261917833475
0.2338614527279	0.2262271809776
0.2339374498631	0.2263140163881
0.2340090715048	0.2263151148979
0.2341468874884	0.2264968030606
0.2342147283362	0.2265254498513
0.2342954066343	0.2265884793149
0.2343705644627	0.2266363005245
0.2344324818235	0.2266773467188
0.2345119039607	0.2267473635571
0.234600777136	0.2267730055713
0.2346997777257	0.2268800645474
0.234770208057	0.2270172998256
0.2348616802369	0.2271654413941
0.2349647703106	0.2273921267399
0.2350348443745	0.2274959008849
0.2351059743988	0.2275509201846
0.2351755348565	0.2275803223772
0.2352773333246	0.2276642246926
0.2353581448825	0.2276883419053
0.2354477825209	0.2278555991786
0.2355773251795	0.2281382740303
0.2356498689841	0.228277387741
0.2357455116381	0.2284786770479
0.2358393529532	0.2286748530302
0.2359059020435	0.2287393287425
0.235983443542	0.2287773083038
0.2360825416761	0.22887791902
0.2361711132464	0.2289898055136
0.2362715247399	0.229049701965
0.2363450630371	0.2291602300724
0.2364233527551	0.2292403904889
0.2365178709849	0.2292417268808
0.2366116690028	0.2294152215048
0.2366988514613	0.2294589448002
0.2368186198049	0.2300784990004
0.2369268927758	0.2304279698845
0.2370351371264	0.2304840435509
0.2371088555515	0.2305193661772
0.237215556549	0.2307028751804
0.2372849763553	0.2308396540499
0.2373785892458	0.2310074199638

0.2374464920352	0.2310144075986
0.2375396425003	0.2311564055188
0.2376086854691	0.2314876082516
0.2377019684302	0.2316905318431
0.237783185336	0.2318442056641
0.2378512518638	0.2318798046265
0.2379332124517	0.2319041057767
0.2380223829775	0.2321942846331
0.2381056823222	0.2322356281004
0.2382227508885	0.2326055186538
0.2382898977724	0.2327519069269
0.2383679106704	0.2327762900044
0.2384484675568	0.2329281141092
0.2385704250423	0.2330273502204
0.2386421122928	0.2331397011337
0.2387176135138	0.23326093409
0.2388049672735	0.2332762487229
0.2388987841575	0.2334908360018
0.2389926292584	0.2335378460312
0.2390747297719	0.2338972593741
0.2391358716473	0.2339190644283
0.2392204401468	0.2340379921108
0.2392777963425	0.2341765775752
0.2393460055626	0.2342287694363
0.2394259481255	0.2342688808561
0.2395146997744	0.2343829291835
0.2396030655287	0.2344528957859
0.2397080521922	0.2344976966491
0.2397943062587	0.2345496987242
0.2398479803964	0.2345639419838
0.2399129413358	0.2346062109434
0.2399968326461	0.2346230084692
0.2400720574701	0.2347721209736
0.2401212033628	0.2348566685521
0.2402007148659	0.2348982285722
0.2402915431914	0.2349319279674
0.2403556747431	0.2349841688411
0.2404475064533	0.235067950803
0.2405342002206	0.2353574488249
0.2406136488672	0.2353741208763
0.2407049912908	0.2354718774538
0.2407809446037	0.2355332559067
0.240834497245	0.2356421526637
0.2409211086325	0.2356987736142
0.2409862628839	0.235719030438
0.2410609739485	0.2357414235492
0.2411121724365	0.2358889163855
0.2412036994976	0.2359581919896
0.241294221304	0.2360079038573
0.2413601134986	0.2360796741208
0.2414308967831	0.2361008222451
0.2414976849824	0.2361993938988
0.2415550320795	0.236219541978
0.2416503680579	0.2362719770843

0.2417362666923	0.2363454134088
0.2418192847945	0.2365400805203
0.24190725784	0.2365640895431
0.2419780115947	0.2365740675718
0.2420382865542	0.2367074311471
0.2421085899437	0.2367544578341
0.2421962078154	0.2369111113345
0.2422820427906	0.2369643027581
0.242371516767	0.2371130988319
0.2424619619275	0.2371348266565
0.242562947183	0.2372160684042
0.2426385926195	0.237325601116
0.2427460869616	0.2375005189687
0.2428468473149	0.2376771008986
0.2429127482502	0.2376834307629
0.2429759546694	0.237739954251
0.243054915805	0.2378141534997
0.2431418594191	0.2379456680515
0.243210847591	0.238047062437
0.2433054776548	0.2381635881602
0.243390707889	0.2381801070873
0.2434875897541	0.2383927980583
0.2435689483546	0.2387865742245
0.2436390145801	0.2387895081616
0.2437422164837	0.2389054365894
0.2438136175312	0.2389355182718
0.2438908399727	0.2389580314234
0.2439602908486	0.2390385439708
0.2440324207881	0.2391748249056
0.2441327749949	0.2393395067024
0.2442044417813	0.2394119988779
0.2442952787495	0.2395197237421
0.2443651172567	0.2397479345302
0.2444376207099	0.239804030304
0.2444937277415	0.2398189842444
0.2445519610919	0.239956875516
0.244629403975	0.2400273325232
0.2447048234126	0.2400881871901
0.244788888939	0.2400942404874
0.2448780821799	0.2401134314353
0.2449573346509	0.2401547608178
0.2450459183909	0.2401637252025
0.2451402704203	0.240332724698
0.2452245043862	0.240388687668
0.2452938744142	0.2404024654437
0.2453682385712	0.2404646664235
0.245428559475	0.2405352867401
0.2455059484401	0.2406669893667
0.2455790790811	0.2407197621255
0.2456906611273	0.2409620138273
0.2457717661758	0.240978430852
0.2458549046442	0.2411216365069
0.245931564316	0.2411294708872
0.2460193019715	0.2413128488594

0.2460804654807	0.2413240680111
0.2461291326297	0.2414147967346
0.2462081305703	0.2414327198972
0.2462813144225	0.2414728560692
0.2463528264995	0.2414741846197
0.2464295056553	0.2416466222656
0.2465088808529	0.2416763207587
0.2465810500932	0.2418201861088
0.2466727646492	0.2419147050029
0.2467782863119	0.241973718927
0.2468854362931	0.2422438715962
0.2470152237748	0.2427221431585
0.2470771638847	0.2428281173288
0.2471371256085	0.2429262452632
0.2472179340233	0.2430868441955
0.2473093307382	0.2431824547606
0.2473758267777	0.2431845034757
0.2474550986918	0.243199962068
0.2475320543067	0.2432080245406
0.247625471427	0.2434875725997
0.2477265161345	0.2434985079705
0.2478390324661	0.2438419441506
0.247919568239	0.2439580790651
0.2479840433303	0.2439999793132
0.2480928587429	0.2440360073039
0.2481748240242	0.2440938608121
0.2483140600166	0.2443972682979
0.2483881492246	0.2444691254203
0.2484534931324	0.244495048125
0.2485590379918	0.2446208659543
0.2486441967704	0.2448266106465
0.248714008394	0.2450041947727
0.2488136475743	0.2451234174875
0.2488897153584	0.2451726801128
0.2490157144192	0.2456143165875
0.2490792840561	0.2457547078829
0.2491454870372	0.2458329400066
0.2492072713719	0.245876328936
0.2493150528388	0.2463368772419
0.249400572642	0.2466532512583
0.2494757678501	0.246690410829
0.2495603612647	0.246717947077
0.2496341402398	0.2467445930794
0.2497155115459	0.2468272860393
0.2497889238061	0.2468431153979
0.2498583325053	0.2469132697452
0.2499418277615	0.2471905662405
0.2500320983827	0.2472250886192
0.2501096208365	0.2473174471852
0.2502022911031	0.2473270230974
0.2502970702648	0.2473390173357
0.2503658164664	0.2473770165845
0.2504631385537	0.2473835838882
0.2505168190924	0.2474243791793

0.2505966969399	0.2475588881299
0.250666152111	0.2475766737363
0.2507582922926	0.2476137604483
0.250836135369	0.2476737588185
0.2509055671733	0.2477319205131
0.2509705118807	0.2478378366696
0.2511134946605	0.2480347543407
0.2511979685998	0.2481587739326
0.251287418169	0.2481800736506
0.2513589409392	0.2482199249176
0.2514538823918	0.2484897399911
0.2515261655709	0.2486167381608
0.2516448984492	0.248620762668
0.251723976403	0.2486289176518
0.2517939970687	0.2489714561215
0.2518773373741	0.2490339583207
0.2519940891259	0.2492502008372
0.252054528806	0.2493310712426
0.2521617798004	0.2494707744735
0.2522439949112	0.2496301236893
0.2523188572705	0.249734741579
0.2524019372698	0.2498178213565
0.2524835876942	0.2498403529829
0.2525525753004	0.2498940221782
0.252607192833	0.2499033978475
0.2526971069491	0.2499143613181
0.2527896767818	0.2499954140254
0.2528766554506	0.2500812007232
0.252965284792	0.250405175012
0.2530373714418	0.2504606609561
0.2531375436031	0.2506957977591
0.25318808548	0.2507084450399
0.2532639160873	0.2507454041715
0.25331869696	0.250761496905
0.2533874112213	0.2507624490734
0.2534432171451	0.2507914667242
0.2535295275091	0.2509286373037
0.2536067978293	0.2509361787163
0.2536998376192	0.2509864339188
0.2537744725978	0.2509866136694
0.2538657226784	0.2511757885687
0.253933133293	0.2511866198227
0.2540275300754	0.2512358077318
0.2540990666079	0.2512439769394
0.2541909238926	0.2515966269643
0.2542651316187	0.2516658139197
0.2543443564648	0.2516722693017
0.2544368386889	0.251689923431
0.2545077152721	0.2517048109774
0.2545820139457	0.2518959170714
0.2546237187459	0.2519150340531
0.2546832708911	0.2519268418318
0.2547580831779	0.2520653677415
0.2548445597612	0.2522505420087

0.2549308080508	0.2524054038842
0.2550025957225	0.2524852613279
0.2550778919557	0.2525404787222
0.2551487828344	0.2525985151144
0.2552191659188	0.2526319024832
0.2553315489862	0.2527565558342
0.2554235154382	0.2527891639464
0.2555087479106	0.2528529201821
0.2556046426939	0.2532257109475
0.2556837964252	0.2534010025273
0.2557566224454	0.2534994171731
0.2558358579294	0.2537815884136
0.2559209623612	0.2539713194245
0.2560061070258	0.2540767369275
0.2560814510517	0.2540808678734
0.2561471171122	0.2541129119764
0.256258514734	0.2542631829286
0.2563399474024	0.2544335545929
0.2564364343155	0.2544428965111
0.2565366776942	0.2547248048183
0.2566135011418	0.2547372659426
0.2566777772319	0.254828510532
0.2567374468763	0.2549180150997
0.2568015135729	0.2550073633615
0.2568539226861	0.2550256148097
0.2569398273132	0.2551501814687
0.2570453164433	0.2552243256588
0.2571138490455	0.2552635873471
0.2571873236104	0.2552891393505
0.2573021303273	0.2553566749526
0.2573913445706	0.2555874986733
0.2574738931365	0.2555877829588
0.2575863491854	0.2556960993639
0.2576595651402	0.2558948655294
0.2577495085731	0.2559285518874
0.2578462666738	0.2559403842623
0.2578954540686	0.2559879351361
0.2579958199115	0.2560584264456
0.2580910901873	0.256063401567
0.2581738516773	0.2561332893723
0.2582638863437	0.2561474061699
0.2583304477393	0.2563255847397
0.2584110004684	0.2563590585002
0.258495431194	0.2564127699123
0.2585638102723	0.2564272886208
0.2586431984748	0.2565648463422
0.2587608306234	0.2565711617189
0.2588269491676	0.2566471933233
0.2589077277891	0.2569617152706
0.2589843842208	0.2570964484808
0.2590696449896	0.2571191513414
0.2591483292796	0.2571376273015
0.2592546065467	0.2574259313136
0.259328042946	0.2574294103828

0.2594142977785	0.2575406248996
0.2594636658958	0.2576845925713
0.2595417981081	0.2577125451237
0.2596351044692	0.2579394365804
0.2597022566237	0.2579789259907
0.2597707547098	0.258027342042
0.2598360704307	0.2580373141693
0.2599298697821	0.2582355888343
0.2599872108561	0.258241767412
0.2600824550745	0.2582945295556
0.2601764725457	0.258398304505
0.2602626288058	0.2584716041732
0.2603537621023	0.2586015089224
0.2604428274938	0.258663710153
0.2605125395076	0.2587580878821
0.2606115572285	0.2590092231174
0.2606863590848	0.2590315481772
0.260747495096	0.2590734160205
0.26079954464	0.2591002703146
0.2608977951923	0.2591157038381
0.2609687535265	0.2591912257088
0.2610364985056	0.2592935978584
0.2611222132804	0.2594452688866
0.2612160386913	0.2596679072766
0.2613173775855	0.2597020022439
0.2614184344341	0.2597704726916
0.2615062978547	0.2598319175677
0.2615598967643	0.259843441785
0.2616668955649	0.2600066914886
0.2617420025102	0.2600269018967
0.2618553499082	0.2600574852554
0.2619374327482	0.2601208200583
0.2620273160088	0.2601544095675
0.2620974062468	0.2602710206819
0.2621907102421	0.2602762653566
0.2622636586597	0.2603116506708
0.2623212723991	0.260369143177
0.2623783163715	0.2603810805257
0.2624456837214	0.2605111832256
0.2625567066859	0.260901356065
0.2626268105325	0.2609658224429
0.2626957860351	0.2610296482514
0.2628094701775	0.2612025971936
0.2629171583769	0.2612460927989
0.2629871737067	0.2614055372466
0.2630741205482	0.261436490796
0.2631544057403	0.2615411892444
0.2632597980293	0.261672084356
0.2633240789289	0.2617112884666
0.2634107103951	0.2617712100832
0.2635136323006	0.262261673958
0.2635688331299	0.262297562871
0.2636628557177	0.262393212566
0.2637641452435	0.262577271414

0.2638682978571	0.2630381027325
0.2639584230651	0.2631674963259
0.264039549231	0.263179173665
0.2641073571281	0.2633776654371
0.2642260043859	0.263537081439
0.2642836074769	0.2638151035076
0.2643493251485	0.2638379287467
0.2644548375017	0.263846616724
0.264537466165	0.2640157082182
0.2645896624175	0.2640531014378
0.2646700273464	0.2641812478796
0.2647387675887	0.2641884103986
0.2648236902348	0.2642317555462
0.2649204320762	0.2645416194224
0.2650217573738	0.2646965165516
0.2651016430407	0.2647013186303
0.2651772738011	0.2647199939394
0.265264892036	0.2648535149617
0.2653264741801	0.2649180134111
0.2654089888598	0.2651036983649
0.2654848243281	0.2652452589681
0.2655533878301	0.2654332314455
0.2656154462518	0.2654951867763
0.2656751511772	0.2655344269067
0.2657521521943	0.2656893353957
0.2658629432364	0.265729277487
0.2659449641119	0.265866212836
0.2660127718071	0.2660087439778
0.2660726635808	0.2660376845276
0.2661612193278	0.2660566425264
0.2662216601242	0.2660872586911
0.2663004071306	0.2661519287097
0.2663741642624	0.2662364902572
0.2664565833343	0.2662639922796
0.2665514519499	0.2665124375438
0.2666107415082	0.2665946513595
0.2666701251687	0.2666031877125
0.2667438297868	0.2666843498643
0.2668144349564	0.2668676617288
0.2669147167041	0.2668687472137
0.2669832343856	0.2669220518287
0.2670365642687	0.2671183507594
0.267124980026	0.2672496308914
0.2672085009996	0.2672618665375
0.2673085113439	0.2675242927013
0.2673640011629	0.2676842088877
0.2674343500677	0.2677532561276
0.2675138784605	0.2677919769396
0.2675953266614	0.2679855038893
0.2676707080576	0.2679876972445
0.2677416609601	0.2681218392221
0.2678384344071	0.2682484561592
0.2679009460179	0.2682679937114
0.2679869860621	0.2683965830771

0.2680621471945	0.268429100584
0.2681586879194	0.2684709656661
0.2682644300277	0.2685886766587
0.2683416910095	0.2686657554121
0.2684300401405	0.2686912515118
0.2685127438499	0.2687052993243
0.2685841650308	0.2687622495104
0.2687022111358	0.2689094280234
0.2688001389003	0.2690878475588
0.2688953054686	0.2691385288573
0.2689703993347	0.2691395889004
0.2690290401445	0.2692955479013
0.2691176214619	0.2693981808902
0.2692241970407	0.2695485171349
0.2693116230084	0.2696179259325
0.2694088650649	0.2696967326033
0.2694781294986	0.2697901056743
0.2695467366464	0.2698790188646
0.269628272983	0.2699074730056
0.2697226007888	0.270107629885
0.2697984398474	0.2701127799797
0.2698629430589	0.2701337134788
0.2699311152389	0.2703096391756
0.2700085714682	0.2703405202524
0.2700813422248	0.2703818294972
0.2701789933493	0.2705786307471
0.2702646488458	0.2706791225018
0.2703229792604	0.2707557651043
0.2703968357753	0.2707659203831
0.2704820577293	0.2707937438841
0.2705572287083	0.2708921794846
0.2706387320486	0.2710901936067
0.2707163261732	0.2713401672849
0.2707690626216	0.2714101851121
0.2708654955955	0.2716663306614
0.270923486582	0.2718266139754
0.2710323595905	0.2718664749907
0.2711095157429	0.2719213516182
0.2711748345024	0.2719353744613
0.2712705228526	0.2720457950055
0.2713641856042	0.2721174235525
0.2714462704714	0.2722131432642
0.2715264625329	0.2722819793227
0.2715938504758	0.2723195616412
0.2716972522704	0.2724765184012
0.271754198406	0.272490198105
0.2718345633084	0.2727280129801
0.2719303520099	0.2729762321996
0.2720289220737	0.2731536958061
0.2720977861252	0.2732797926353
0.2721700709603	0.2733753079318
0.2722258264636	0.2733982425052
0.2723105371756	0.2734473586167
0.2723850575549	0.2734707125025

0.272447623346	0.2734914843444
0.2725263731401	0.2739546703636
0.2726318205144	0.2741009635255
0.2727536946762	0.2741466428135
0.2728531584697	0.2742972773104
0.2729298024069	0.2743287046211
0.2730075641057	0.2743469124895
0.2730839960815	0.2744269471102
0.2731926912002	0.2746314652718
0.2732903265018	0.2747702806053
0.2733647532163	0.2749514640233
0.2734369329882	0.2749540060535
0.2734987372921	0.2749832478189
0.2735684960367	0.2750290483412
0.2736469982295	0.2751068627683
0.273732934242	0.2752109529287
0.2737988181895	0.2752189454008
0.2738870486943	0.2753968172182
0.273957537719	0.2754385319195
0.2740254372609	0.2755029316802
0.2741077385256	0.2757216933611
0.2742106797048	0.2759475233369
0.2742821669368	0.2761244587718
0.2743675441774	0.276222661036
0.2744398579818	0.2762645823673
0.2745033480611	0.2763963746223
0.2745990931967	0.2765873691538
0.274704644051	0.2766099616658
0.2747861680815	0.2771166245246
0.2748566675791	0.2771519609957
0.2749102941617	0.2771545959814
0.2749939326156	0.2771880212779
0.2750686228296	0.2774363426991
0.2751767490199	0.2780808303164
0.2752375566632	0.2782209122399
0.2753660174748	0.2786566912416
0.275429387044	0.2786788202551
0.2755183043111	0.2788319585259
0.2755862830582	0.2788392703841
0.2756809867156	0.2788681289253
0.2757792570123	0.2790311560476
0.2758531133127	0.2790706088777
0.2759318685389	0.2790897977423
0.2760101279635	0.2790919130393
0.2760726637254	0.2791549169487
0.2761576243854	0.2792073187315
0.276229255008	0.2792631781376
0.2762969266111	0.2793567297632
0.2763657794285	0.2794452417876
0.276440705609	0.2794997767728
0.2765047092473	0.2796290340447
0.2765862977541	0.2796677645226
0.2766903464301	0.2797048584214
0.276752632655	0.279768596041

0.27680976913	0.2798719492579
0.2768847031456	0.2799330724727
0.2769595275262	0.2799813341564
0.2770267579543	0.2800646434853
0.2771271045634	0.2800669437027
0.2772140039358	0.2800826463484
0.277306205643	0.2801167749999
0.277378239739	0.2801679197859
0.2774304018639	0.28023517223
0.2774965235628	0.2802953072222
0.2776023101924	0.2803215372688
0.2776875198419	0.280394311135
0.2777599786933	0.2804537254087
0.2778408007378	0.2805429658688
0.2778946508204	0.2805689998517
0.2779714474502	0.2806954927703
0.278054549389	0.2807897686765
0.2781498315251	0.2808444906314
0.2782303754561	0.2808667951934
0.2783011033183	0.2808837932084
0.2783889320679	0.2809573204796
0.2784615410577	0.2810933480123
0.2785441167602	0.2812023161495
0.2786296015426	0.2812964510888
0.2787193637283	0.2812971369667
0.2788446799581	0.2815902187341
0.2789385104191	0.2815926668894
0.2790279834258	0.2815990491091
0.2791025858044	0.2816640063995
0.2791532751536	0.2816967837465
0.2792247181438	0.2817047388523
0.2793104269126	0.2817407730414
0.2793833925901	0.2817995843974
0.2794567251578	0.2818266350186
0.2795772933233	0.2821725427181
0.2796575155064	0.2822591790154
0.2797210910564	0.2822602598584
0.2798026856798	0.2822937887883
0.279878397257	0.2824225607132
0.2799717612065	0.2824569506782
0.2800761112101	0.2825229908419
0.2801328969374	0.2825752428457
0.2802472670658	0.2826891974471
0.2803435581678	0.2828463439297
0.2804275008648	0.282896752182
0.2805156097584	0.2830129311853
0.2805954840854	0.283025972899
0.2806506826282	0.2831051081398
0.2807343821688	0.2833900718896
0.2808092593031	0.283430012853
0.2808858049577	0.2836134793933
0.28095603208	0.2836296615075
0.2810560560512	0.2836835048489
0.2811462922274	0.283739702965

0.2812258760209	0.2837664643962
0.281335297889	0.2838002270678
0.2814403340071	0.2838835482861
0.2815157984512	0.2840970710119
0.2816099783873	0.2841877727784
0.2816929715381	0.2842825260557
0.281803140508	0.2844584040414
0.2819055349984	0.2846751387803
0.2819936804309	0.2847168066003
0.282112222137	0.2850510490366
0.282215994992	0.2850693656667
0.2823013964914	0.2850767437673
0.2823694391913	0.2851751194312
0.2824451687389	0.2852396275093
0.2825152758328	0.2852649085084
0.2826373946034	0.285289612633
0.282722420746	0.2852970808149
0.2828438698184	0.2856911965284
0.2828852697689	0.2857980104122
0.2829445135712	0.2859055292287
0.2830564086539	0.2861674392766
0.2831646762816	0.2866889288298
0.2832689954479	0.2868191676732
0.2833620709645	0.2869420115397
0.2834598231017	0.2869668456136
0.283563510405	0.28711541362
0.2836539907419	0.287154635898
0.2837491863781	0.2873104803662
0.2838470539191	0.2874133756257
0.2839306861562	0.2876335201064
0.2840015897589	0.2876736687097
0.2840708742109	0.2877238086017
0.2841559437096	0.2877239781826
0.2842485846082	0.2877654154569
0.2843476112962	0.288063438428
0.2844452627682	0.2880759828834
0.2845267909478	0.2882018310464
0.2846134253586	0.2882325756742
0.2846923982206	0.2882420327818
0.2847613674943	0.288363722365
0.284837970241	0.2884959273064
0.2849185517469	0.2885532155057
0.2849809186852	0.2886269069738
0.2850522767718	0.2887771371006
0.285147548938	0.2894176759766
0.2852217540409	0.289443237247
0.2853040990633	0.2896091635461
0.2853740355036	0.2896360658557
0.2854667381659	0.2896891079192
0.2855284515063	0.2896961103041
0.2855956502364	0.2897697677908
0.2856679008024	0.2899054521629
0.2857628275262	0.2901312941948
0.2858550735676	0.2901623352837

0.2859340109805	0.2902675507234
0.2860243960662	0.2903458980173
0.2860998746634	0.2904927412415
0.2861518476337	0.2905225577768
0.2862486176667	0.2905275409455
0.2863172002502	0.2906259562768
0.2863835917419	0.2906759637368
0.2864989281291	0.2910798674231
0.2865841987283	0.2912111334695
0.2866768288683	0.2912499439142
0.2867364683895	0.291287853995
0.2868176907738	0.2913528243086
0.2868962610232	0.2913539306616
0.2869694241337	0.2913909957362
0.2870454303131	0.2914178262038
0.2871161278616	0.2915105426698
0.2871714365067	0.2916532083579
0.2872482904648	0.2917049123916
0.287327554792	0.2918652897255
0.2874117622047	0.2920985134755
0.2874958883846	0.2922817980637
0.2875869191481	0.2923317427612
0.2876679926272	0.292413858474
0.2877639457645	0.2925718166102
0.2878475193845	0.292689086918
0.287928582148	0.2927377262573
0.2879923702963	0.2927462227831
0.2880551686729	0.2927594500584
0.2881362689889	0.2928581993436
0.2882080186887	0.2929036685118
0.2883078718618	0.2931160171813
0.2883939771416	0.2932664780737
0.2884866029758	0.2933911727625
0.288574703585	0.2934373605994
0.2886432642849	0.2934713819825
0.2887196706816	0.2934804941018
0.2888031160158	0.2937266251998
0.2888644546853	0.2937692755859
0.2889612066688	0.2938108973157
0.2890487680684	0.29385069473
0.2891267430822	0.2939622531088
0.2892089914045	0.2940503691726
0.2892879183167	0.2940719973331
0.2893739648847	0.2941014879098
0.289452963293	0.2942576569808
0.2895170499997	0.2943397539826
0.2896132956355	0.2945999550664
0.2896728788869	0.2946145460182
0.2897732044807	0.2947697177343
0.2898791388801	0.2948746006235
0.2899952178653	0.2951474493888
0.2900795378636	0.2952755587175
0.2901582052683	0.2954589075088
0.2902375152605	0.2958810068048

0.2903321480387	0.2959431293019
0.2904252687445	0.2962604994318
0.2905006480415	0.2963013610621
0.2905574699805	0.2963711021647
0.2906391567702	0.2966060070256
0.2907382989421	0.2967216946231
0.2908456167772	0.2967533065521
0.2909309377737	0.2969609704765
0.2910358513187	0.2972800333477
0.2911165372453	0.2973556188616
0.2912167813312	0.2974588895444
0.2912933585427	0.2974878786276
0.2913519185515	0.2975270577986
0.2914459264953	0.297798562804
0.2915283169669	0.2978485582081
0.2915968454688	0.2980480137119
0.2916599675896	0.2980967248208
0.2917420768529	0.2983658486247
0.2918266112702	0.2985978293082
0.2919107650094	0.2986022487818
0.2919916749155	0.2986882906054
0.2920690665829	0.2987341466966
0.2921557041743	0.298798511106
0.2922632033231	0.299018346137
0.2923103813138	0.299084456891
0.2924056391619	0.2992739233788
0.2924728286593	0.2993448498485
0.2925499478366	0.2993763691152
0.292637265904	0.2995688520555
0.29273086555	0.2996035159233
0.2928251469979	0.2996721547236
0.2929014544399	0.2997429355176
0.2930223106104	0.3004248858409
0.293121309777	0.3007035778275
0.2931920144883	0.3007516273677
0.2932793669136	0.3007866546522
0.2933550230201	0.3008654017677
0.2934370795396	0.3009199182574
0.2935751472759	0.3013268340912
0.293650073734	0.301422363388
0.2937262829666	0.3014973924689
0.2937945343423	0.3016834224993
0.2938707872349	0.301866560483
0.2939456535939	0.3019516018095
0.2940224287194	0.3019520546242
0.2940930428438	0.3020474836249
0.2941520973848	0.3021085306197
0.2942217654195	0.3021109965535
0.294303888784	0.3021444411096
0.2943873644717	0.302265456778
0.2944825346022	0.3022752314078
0.2945733525756	0.3023277717847
0.2946308396318	0.3023412829952
0.2947288113774	0.302447444034

0.2948028572497	0.3025219598282
0.294882398207	0.3025835289418
0.2949762279059	0.3026837752841
0.2950486951614	0.3027083323589
0.2951399280348	0.3027137914263
0.295227723536	0.3027616274313
0.2953164241384	0.3028107518692
0.29540004692	0.3029982171893
0.2954636009792	0.3031522049249
0.295537423993	0.3031691335724
0.2956124447523	0.3033465206831
0.2957362393044	0.3034695042114
0.2958034038902	0.3035256979743
0.295861888565	0.3035368599278
0.2959481114421	0.3036369801437
0.2960466286713	0.3037401073869
0.2961449534853	0.30374311327
0.2962371929556	0.3038872454119
0.2963132205842	0.3039611504366
0.29637739878	0.3040077786454
0.296449410883	0.3040175959297
0.2965413419999	0.3041707937974
0.2966220705634	0.304298937998
0.2967158476098	0.3045855846356
0.2967936140103	0.3046497147976
0.2968770312312	0.304738887039
0.297000041143	0.3049546608618
0.2970767739607	0.3049745729819
0.2971859500763	0.305119646126
0.2972785557884	0.3052084327365
0.2973481414093	0.3052578732642
0.2974639217374	0.3053222505371
0.2975329077157	0.3053357936083
0.29761788364	0.3053626451345
0.2976914509215	0.305441384264
0.2977650210402	0.305544263055
0.2978396349455	0.305841461591
0.2979101204462	0.3059624152144
0.2979686827265	0.3061309442604
0.2980452997415	0.3061687832869
0.2981373036456	0.3063086749898
0.2982213761569	0.3064376687044
0.2982875879309	0.3065490853124
0.2983725090462	0.306719393435
0.2984525461411	0.306824228757
0.2985265786863	0.3068450407843
0.2986255222942	0.3069772425694
0.2987239005869	0.3070727192605
0.2988553756792	0.3074781681541
0.2989257169767	0.3076280730353
0.2990168150386	0.3076983448434
0.2991072327038	0.3079365064112
0.2992064071254	0.3080007251229
0.2992814457212	0.3080653831782

0.2993623565296	0.3082111675626
0.2994567610702	0.3084478759765
0.2995512694762	0.3087153762103
0.2996142022498	0.3087915613277
0.2997202633718	0.3088124476748
0.2997647641072	0.3088480543813
0.2998342664995	0.3088909071111
0.2999060620929	0.3089258987858
0.2999903404652	0.3090362859008
0.3000816508691	0.3090787971194
0.3001667842847	0.3091214418805
0.3002388501239	0.309138278988
0.3003129132346	0.3091998858837
0.3003864162326	0.3093186299795
0.3004621531237	0.3094356078492
0.3005270648287	0.3094489806896
0.3005967459402	0.3094539505211
0.3006766634922	0.3096478290161
0.300767272403	0.3097016666582
0.3008332873867	0.3097298851544
0.3008954036638	0.3097651660559
0.3009759701837	0.3098052082839
0.3010335709127	0.3098546055902
0.3011196734673	0.3099729928466
0.3011936854399	0.3099758608465
0.3012595466316	0.3099856818634
0.3013323935016	0.3102489682727
0.3014354245697	0.3105400424127
0.3015337875903	0.3105614704776
0.3016013226188	0.3106171592586
0.3016881193471	0.3106485413993
0.3017630855527	0.3108318458202
0.3018313927803	0.310837996846
0.301910991879	0.3108463910815
0.301989068209	0.3108728704796
0.3020740570305	0.3111097135088
0.3021388729076	0.3111473979156
0.3022420237691	0.3113352170299
0.3023322601735	0.3113707004441
0.302427683594	0.3114135690613
0.3025129917686	0.3114203633924
0.3025910644972	0.3114551823766
0.302690868794	0.311809084531
0.3027896998597	0.3119040753077
0.3028566408447	0.3119310250589
0.3029622574742	0.311957923604
0.3030685428794	0.3120102383208
0.3031378238766	0.3120163546262
0.3032518005516	0.3121832099788
0.3033283162755	0.3123357609127
0.3033940550418	0.3123571263477
0.3034782838952	0.3126098727158
0.303562290227	0.3127696327894
0.3036586270505	0.3127979342911

0.3037574032908	0.3128643567356
0.3038256191975	0.3128940575936
0.3039185265283	0.3130527995278
0.3040361695306	0.3132420136663
0.3041464781387	0.3133167584664
0.3042331048118	0.3133578778425
0.3043181681792	0.3134642218787
0.3044005807018	0.3135479745715
0.3044763981885	0.3135588262769
0.3045342280596	0.3136564638567
0.3046360114898	0.313770957745
0.3047112649088	0.313892004031
0.3047935533956	0.3139099548805
0.3048648363071	0.3143161904259
0.3049320860968	0.3144269262035
0.3050203515373	0.3145226454654
0.3050918009439	0.3146471198096
0.3051814633559	0.3148044178124
0.3052989986677	0.3150124483939
0.3053647680513	0.3150169060377
0.3054857212293	0.3150785183703
0.3055614030401	0.3151851793852
0.3056377877695	0.3152256038338
0.3057146088735	0.3152457263045
0.3057717766037	0.3153113165258
0.3058696433991	0.3153690981048
0.305928332486	0.3154833838644
0.3059924891221	0.3155071206379
0.3060913114882	0.3157083592064
0.3061398737717	0.3157648721509
0.306210055752	0.315823014484
0.3062870517179	0.3158812398768
0.3063953297226	0.3161680541108
0.3064879051757	0.3164478695375
0.3065512186619	0.316454710117
0.3066404004484	0.3164646806189
0.3067256339927	0.3164747852237
0.3068305490348	0.3167432665795
0.3069500547485	0.3167496622948
0.3070460100064	0.3169887788956
0.3071332777108	0.3170273646581
0.3072116313073	0.3173581177486
0.3072804638442	0.3173589805751
0.3073783422561	0.3173598445456
0.3074493828819	0.317396599993
0.3075264511618	0.3174114945427
0.3075889908215	0.3174185586358
0.3076951964248	0.3177161875881
0.3077599917356	0.3177231417154
0.3078577467103	0.3179466625665
0.3079483975103	0.3180424996553
0.308005400987	0.3180797250169
0.3080724740275	0.3181042578654
0.3081339227893	0.31813001773

0.3082133931598	0.3182396001354
0.3082620176105	0.3182671481101
0.3083627201618	0.318311635534
0.3084442407976	0.3185035201624
0.3085291944672	0.3185189416645
0.3086310520984	0.3186888029561
0.3087061703023	0.3187730863887
0.3087716231662	0.3188020322894
0.3088513794937	0.318896474497
0.3089299273918	0.3189282885283
0.3090225719502	0.3189756825248
0.3091205739152	0.3193437173173
0.309200988207	0.3193734910949
0.3092712039833	0.3194422839643
0.3093381762195	0.319450756178
0.3094217733747	0.3194911545429
0.3094878700373	0.3195187696143
0.3095737238485	0.3197861461154
0.3096446107225	0.3197923326997
0.3096972255564	0.3198635315991
0.3097814924721	0.3199023546997
0.30985205979	0.319928153186
0.3099310916352	0.3200012953672
0.3100269383091	0.3202017404239
0.3100942903786	0.320203904203
0.3101834998365	0.3202539249385
0.3102724025998	0.3203736179227
0.3103804176584	0.3211077310329
0.3104300292267	0.3211277603111
0.3105119720468	0.32113661655
0.3106113409957	0.321259520782
0.3107156849021	0.3212664802162
0.3108134123834	0.3214598115283
0.310896181832	0.3215401820043
0.3109981628324	0.3216142541194
0.311074617535	0.3216803519699
0.311151892469	0.3217595625159
0.3112182763777	0.3218711011327
0.3112908969904	0.3218820339514
0.3114131566303	0.3221120670205
0.3114939672879	0.3221370597652
0.3115790505785	0.322170713766
0.3116540238629	0.3223174870782
0.3117237013602	0.3224401580494
0.3118129252973	0.3226600946399
0.3118983358829	0.3229039384224
0.3119736527442	0.3229834806997
0.3120543049883	0.3232854303874
0.3121668685065	0.3235640567124
0.3122627519383	0.3237975232836
0.312348094246	0.3238607245605
0.3124285181247	0.3239703007618
0.3125003953376	0.3241361487025
0.3125610409021	0.3242198550971

0.3126499359249	0.324235903198
0.3127119799044	0.3242760075227
0.312789903893	0.3245113083283
0.3128627452914	0.3247021190247
0.3129396240315	0.3247037308453
0.3130271143376	0.3248141119121
0.31312145183	0.3248165399565
0.3132082271238	0.3249109019072
0.3132661667451	0.3249420121135
0.3133540301984	0.324961743101
0.3134649646497	0.3251012129387
0.3135430865201	0.3251556917638
0.3136584212702	0.3252740157306
0.3137534809404	0.3253242633508
0.3138348838419	0.3253410459232
0.3139205614422	0.3253702927093
0.3139959452036	0.3256312079327
0.3140632695495	0.3259750751285
0.3141703584378	0.3261113145112
0.3142671188868	0.3261591558449
0.3143683143361	0.326247292223
0.3144405186869	0.326267343823
0.3145115634234	0.3262932069787
0.3145715381943	0.3263090539517
0.3146589298128	0.3264849196962
0.3147400408391	0.3264982034283
0.3148325393978	0.3266083107481
0.3149162643297	0.326756964248
0.3150076055167	0.3268539879286
0.3150943828943	0.3269372146837
0.3151629619693	0.3269486500347
0.3152344925354	0.3270420452731
0.3153140237459	0.3272537176492
0.3153900011702	0.327266253945
0.3154635556081	0.3273284481507
0.3155458321115	0.3273451648723
0.315633222802	0.3273590676907
0.3156917610141	0.3273611197823
0.3157502288665	0.3273809845986
0.3158141711122	0.327431844583
0.3158757056102	0.3274837008476
0.3159587947904	0.3275316954238
0.3160610032898	0.3278185866943
0.3161586234203	0.3278356675197
0.3162361623903	0.3279012094704
0.3163488456631	0.3279565126451
0.3164141344738	0.328046389205
0.3164822646655	0.3281968805384
0.3165728273369	0.3284084129147
0.3166348350886	0.3284322349559
0.3167360298355	0.3284886902288
0.316819221641	0.3286175875784
0.3168835864122	0.3286743676668
0.3169608207056	0.3288101387245

0.3170297984063	0.3288491502555
0.3170875218583	0.3289521474741
0.3171505163259	0.3289948432059
0.3172267098946	0.3290959290259
0.3173024683189	0.3291704887288
0.3173772521549	0.3291889549304
0.3174401603248	0.3293133785412
0.3175058781948	0.3293370837383
0.3175876386632	0.3293388797813
0.3176827196344	0.329496691815
0.3177563580248	0.3295286044089
0.3177992583378	0.3295513338295
0.3178973167256	0.3298365592694
0.3179967609854	0.3298867087846
0.3180717439725	0.3299294525205
0.3181419063956	0.32993036482
0.3182111594018	0.3299332119404
0.3183141360331	0.3300084411836
0.3184019072893	0.3301010100575
0.3184886422988	0.330118578496
0.318562782485	0.3301288603499
0.3186416814271	0.3302506616399
0.3187144385602	0.3302946324313
0.3187827303351	0.3303404924112
0.3188649387515	0.3303800256883
0.3189542240174	0.3305294065031
0.3190214591427	0.330554612315
0.31908901018	0.3306363152646
0.3191770585795	0.3306374602254
0.3192954886888	0.3308971389311
0.3193820152408	0.3309724975629
0.3194678638431	0.3310026739372
0.3195582086307	0.3310224738619
0.3196398412251	0.3310528978146
0.3197066603886	0.3311798259638
0.3197790558511	0.3313768000192
0.3198700840204	0.3314755582478
0.3199355250289	0.3315477653107
0.3200057717643	0.3315556435392
0.3200861260569	0.3315627314627
0.3201809150439	0.3315857867551
0.3202473300842	0.3315939579853
0.3203091286986	0.3316491237812
0.3203759080328	0.3316630404212
0.3204880466039	0.3317873965056
0.3205474667023	0.3317924534729
0.320619115203	0.331797805144
0.3206948364071	0.331874962514
0.3207741239293	0.331896998401
0.320881308019	0.3320459351106
0.3209746678403	0.3320549467684
0.3210398293497	0.3321080987922
0.3211066640812	0.3322113778204
0.3211982847009	0.3323278586499

0.3212702376029	0.3324369259795
0.3213398324666	0.3324427271232
0.3213833373162	0.3325606984782
0.321446909378	0.3326144631944
0.3215137545747	0.3326309972309
0.3215873267248	0.3326646060575
0.3216607036472	0.3326851007375
0.3217645275827	0.3328205962321
0.3218490574536	0.3328522259374
0.3219388124955	0.3329525298847
0.3220185330965	0.3330078204047
0.3221164395403	0.3332504619302
0.322202569871	0.3333647209112
0.3222765070717	0.3334302474503
0.3223864908826	0.3334609841778
0.3224742010348	0.3337383921921
0.322572505878	0.3338686266507
0.3226682835435	0.3339814326596
0.3227274097743	0.3339967593742
0.3227819134852	0.3340056961304
0.3228681687574	0.334077176009
0.3229293441488	0.3343296816517
0.3230235999627	0.3343810975589
0.3231100700659	0.3343844790815
0.3231872236903	0.3344735735253
0.323267994733	0.3344998613906
0.3233556155765	0.3345988280127
0.3234444663394	0.3348354186208
0.3235078236757	0.3348492181393
0.3235589034385	0.3349018946273
0.3236246814198	0.3349103827988
0.323688848188	0.3349121177661
0.323766551301	0.3349516933646
0.3238604184173	0.335261583467
0.3239327254906	0.3352657773374
0.3240159475699	0.3355266114467
0.3240953476196	0.335590974321
0.324170790336	0.3356141818284
0.3242639510464	0.3357365129976
0.3243827152068	0.3357851845069
0.3245029322608	0.33614456879
0.3245978899864	0.3362299061728
0.3246929054754	0.3366838337555
0.3247766654463	0.3367181796006
0.3248537991037	0.3367356148763
0.3249454439472	0.3368414712784
0.3250225991583	0.3368889312015
0.3251160680711	0.3372458171262
0.3252020626195	0.3373557794383
0.3252692545501	0.3374056363626
0.3253539320187	0.3377063613412
0.3254221148876	0.337767437916
0.3255121034921	0.338084203584
0.3255951725971	0.3382097573779

0.3256654978265	0.3382137783453
0.3257719912679	0.3385674148484
0.3258795481397	0.3387113604707
0.3259449916451	0.3387556617017
0.326025135674	0.3389287472281
0.3260847511728	0.3389519698082
0.3261586237779	0.33906557465
0.3262266130146	0.3391789738699
0.3262860991912	0.3391844962618
0.326368988876	0.3391874155228
0.3264466875444	0.3392490480297
0.3265296645699	0.3393418805403
0.3265939168973	0.3393615786756
0.326698021607	0.339460204079
0.3267662116243	0.3396562844695
0.3268609288228	0.3398517051164
0.3269304200239	0.3399890439461
0.3270045661635	0.3400044780866
0.3271224016581	0.3402432007599
0.3272248619731	0.3407265535378
0.3273084974467	0.3407327897177
0.3274140050651	0.3407563626871
0.3275055755357	0.3409585748297
0.3275965263501	0.3409813984338
0.3276375543904	0.340989741856
0.3277371117609	0.3410377909034
0.3278264172703	0.3411506699809
0.3278882079203	0.3413601478495
0.3279847517377	0.3414132547095
0.3280909564876	0.3418891415907
0.3281859900322	0.3419155990508
0.3282355171663	0.3419292113337
0.3282942587694	0.3420156848693
0.3283512122321	0.3420254291859
0.3284254839949	0.34212957123
0.3285006070015	0.3421874767399
0.3285874347291	0.3421973383942
0.328652740718	0.3423681589591
0.3287428331744	0.342533818511
0.3288585759174	0.3426302945981
0.3289439603775	0.342700756715
0.3290090838751	0.3427385339095
0.3291039114261	0.3427617904692
0.3291739734859	0.3427899844098
0.3292662832487	0.3428690942356
0.3293536428971	0.3429160347435
0.3294165430753	0.3429940945621
0.3294834834363	0.3431101987096
0.329579951171	0.343143573668
0.3296737570956	0.343351499961
0.3297397225915	0.3434687989018
0.3298403945592	0.3438669901032
0.329904964896	0.343918753598
0.3299749399628	0.3440246135559

0.3300366150093	0.3441345168639
0.3301204779529	0.3442335671347
0.3301886095925	0.344400880596
0.3302597001017	0.3444942119995
0.3303361014267	0.3447716192022
0.330401046569	0.3448277938789
0.3304683149223	0.3448453111762
0.3305629851595	0.3450790967558
0.3306471733924	0.3452885687426
0.3307183403158	0.3453437660277
0.3307964041913	0.3454799172825
0.3308550014917	0.3454897252857
0.330925181195	0.3455085971403
0.3310216920886	0.3456526178097
0.3310951613886	0.3456733660273
0.3311819790969	0.3456815235999
0.331256512543	0.3457445025339
0.3313340629149	0.3457772758947
0.331382154191	0.3458035228502
0.3314651170759	0.345856231829
0.3315540764259	0.3458806598338
0.3316894926319	0.3460798161432
0.3318046016561	0.3465798392125
0.3319079258982	0.3467379597551
0.3319941919923	0.3468130030447
0.3320679377381	0.3468275653568
0.3321464565168	0.3471518882013
0.332227842698	0.3473397083555
0.3322973883988	0.3474029950661
0.3323699939068	0.3474982364867
0.3324648355412	0.3476797911859
0.3325189043787	0.3477637870752
0.3325791319986	0.347814791
0.3326652125697	0.3479005379471
0.3327505164082	0.3479794989539
0.3328710546917	0.3486021440763
0.3329488350209	0.3487554141083
0.3330464801095	0.3489578477513
0.333122614664	0.3492202780489
0.3331977380889	0.3492365812792
0.3332846738834	0.3492609625609
0.3333501920869	0.3493551267997
0.3334538631153	0.3495168476005
0.3335183449307	0.3495368290153
0.3336045074318	0.3495478887403
0.3337045612385	0.3496433303157
0.3337937127813	0.3499033133839
0.3338744371819	0.3499981728592
0.3339739496605	0.3500542602741
0.3340636358322	0.3501968678062
0.3341599417985	0.3502587615262
0.3342497146359	0.3504236662128
0.3343466681991	0.3508224436938
0.3344702312971	0.351119898779

0.3345476587454	0.3511297856409
0.3346262418785	0.3512075013859
0.3346904801385	0.3512414215586
0.3347946106875	0.35131758275
0.3348889349011	0.35132586845
0.3349662048697	0.3514364047008
0.3350344090306	0.3515700268306
0.3351195965354	0.35159383824
0.3351905634889	0.3516491319806
0.3352649396713	0.351866858222
0.3353259753947	0.351909568154
0.3354260948708	0.3519672249779
0.3354954570658	0.3520508743406
0.3355590056937	0.3520861844143
0.3356147663964	0.352125258624
0.3356779054326	0.3521704647644
0.3357499854485	0.3522537969481
0.3358208997441	0.3523140533689
0.3359073336795	0.3523641969418
0.3359853672058	0.3523930366739
0.3360616316551	0.352397944774
0.3361420755555	0.352424578216
0.3362297035363	0.3524824561739
0.3363162658109	0.3525225300275
0.3363989734544	0.3526063389118
0.3364682668126	0.3526719012593
0.3365620650547	0.3530507550874
0.3366446049314	0.3532265728722
0.3367202394105	0.353407596338
0.3368065974755	0.3535292336058
0.3368697994854	0.353534711528
0.3369538184057	0.3536061760419
0.3370211298363	0.353638349875
0.3371025788097	0.3536402564759
0.3371754252556	0.3536530370685
0.3372645752982	0.3536830538315
0.3373497541004	0.3538321456609
0.3374385676642	0.3539365507556
0.3375318497146	0.3541071260335
0.3376166022339	0.3541129502225
0.3376862512365	0.3541528176582
0.3377743152756	0.3541534882422
0.3378807149645	0.3542242566778
0.3379582495833	0.3542951949302
0.3380202821032	0.3543833122568
0.3380934271359	0.3545648963795
0.3381811372274	0.354773582388
0.338306240311	0.3551970311465
0.3383872639779	0.3552269778039
0.3384574873194	0.3553054347922
0.3385383450975	0.355547868305
0.3386031860357	0.3556982342954
0.3386632429545	0.355900210438
0.3387511815572	0.3562437700502

0.3388385953032	0.3562766530086
0.338930402342	0.3564727424097
0.3390109175918	0.3567974357668
0.3391058516469	0.3570556720797
0.3391909814849	0.3570724810606
0.3392435629829	0.3570786764255
0.3393173947878	0.3572342618213
0.3394465674175	0.3578000168268
0.3395133474555	0.3578363342907
0.3395960044996	0.3578512390228
0.3396825660497	0.3579718094084
0.3397563768926	0.3579847132206
0.3398088919877	0.3580383264798
0.3398770467022	0.3580758363804
0.3399639212015	0.3580897600009
0.340038543217	0.3580943301312
0.3401227304107	0.3580982491053
0.3401971748944	0.358116513115
0.3403176921255	0.358353240804
0.3404161788545	0.3584617440052
0.3404560695495	0.3585664892194
0.3405213988687	0.3588013239796
0.3406221605542	0.3591188224692
0.3406691456318	0.3591959213888
0.3407546840069	0.3594807446029
0.3408435219961	0.359609663588
0.3409089230888	0.3597502908855
0.3409737769134	0.3598170671036
0.3410633390565	0.3598410274947
0.3411372728766	0.359857903455
0.341184020793	0.3599684167006
0.3412698374428	0.3600262197367
0.3413504629158	0.3601920056795
0.3414168806596	0.3602545771259
0.3415357627858	0.3604877713194
0.3416019380279	0.3605400193579
0.3416720454718	0.3606297760915
0.3417557442798	0.3607508457047
0.341820103252	0.3607757827531
0.3418957680691	0.3609433522075
0.3419538560523	0.3609662776194
0.3420370643854	0.3610721966895
0.3421222843413	0.3610749503536
0.3422201274915	0.3611950296945
0.3423051699776	0.361232606028
0.3423721513987	0.3614819246708
0.3424527937265	0.3617239926654
0.3425476737687	0.3617268850813
0.34264134609	0.361759709132
0.3426997118473	0.3617820944012
0.342795901915	0.3618726837403
0.342890695784	0.3619033839991
0.3430121787487	0.3624524350738
0.3430825266186	0.3625412792315

0.3431423217248	0.3625801568057
0.3432143002873	0.3627169747528
0.3433086922192	0.3629755770858
0.3433940150472	0.3631117667109
0.3434555149131	0.3631270377725
0.3435361550634	0.3631552858694
0.3435845691697	0.363160340664
0.3436497983807	0.363221718882
0.3437587372056	0.3634284267723
0.3438303372356	0.3634495707389
0.3439437485971	0.3634517439926
0.3439870480459	0.3634840260042
0.3440700000629	0.3636144178808
0.3441394834001	0.3636160076297
0.3442093780383	0.3636261272302
0.3443130325324	0.3636819497081
0.3444074370403	0.3639723379776
0.3444800181647	0.3640630482817
0.3445579714352	0.364152861267
0.3446237347464	0.3641982560896
0.3446856996715	0.364211040959
0.3447590035694	0.36442047137
0.3448496979705	0.3644524621427
0.3449095554226	0.3644700609703
0.3449643262367	0.3645120363308
0.3450315553849	0.3645990839548
0.3450844292451	0.3646066533785
0.3451468340715	0.3646264497311
0.3452138653257	0.3646699287123
0.3453002904241	0.3648206734719
0.345371573792	0.3648376939645
0.3454654716606	0.3650173556555
0.3455264467088	0.3650228977712
0.3456497051491	0.3652890211445
0.3457703293436	0.3653513490791
0.3458487009187	0.3655570598667
0.3458957419974	0.3656219523536
0.3459800838837	0.3656766899975
0.3460605926415	0.3656825079326
0.3461798134525	0.3661243225315
0.3462494215696	0.3661462614716
0.3463306261121	0.3661898629295
0.3464130287275	0.366195602145
0.3464897907158	0.3663251124753
0.3465530064563	0.3664163261178
0.346631692642	0.3665971582524
0.3466937362854	0.3666057926691
0.3467758699956	0.3666487438525
0.3468400699267	0.3666641528704
0.3469416059276	0.3666801442042
0.347017293996	0.3667524032523
0.3470895648249	0.3668658551642
0.3471529948978	0.3669856206248
0.3472427684386	0.3670793925241

0.3473304756663	0.3671707348449
0.3474153692392	0.3671897583903
0.3474799288272	0.3672090078158
0.3475603491283	0.3675064790873
0.3476418361395	0.3676784903851
0.3477115503349	0.3677730982966
0.3478123345721	0.3679625891521
0.3478924666885	0.3680939648214
0.3479550156632	0.3681446722184
0.3480327239384	0.3682934037732
0.3481060664733	0.3683134404735
0.348193711832	0.3684734457217
0.3482702270555	0.3684900166925
0.3483587634404	0.3687213067127
0.3484265025812	0.3688667576037
0.3485101577511	0.3692621003899
0.3485951381478	0.3693251409037
0.3486752307533	0.3695239636847
0.3487521822949	0.3695281141974
0.3488138101444	0.3696463101029
0.3488801737326	0.3696519593825
0.3489401871388	0.3696700748142
0.3490282688294	0.3697686065262
0.3491075533472	0.3697711101174
0.3492270662482	0.3698136254037
0.3493185146762	0.3698587093787
0.349406053937	0.3701896930168
0.3494973324446	0.3702228823902
0.3495898025862	0.3704247138378
0.3496682521644	0.3704251496478
0.3497531924587	0.3706925134616
0.3498296323319	0.3707277458806
0.3499200064026	0.3707701941206
0.3499723636274	0.3708141025638
0.350036963907	0.370911795134
0.3501120207337	0.3709423077589
0.3501842348212	0.3710618049745
0.3502509551048	0.3712156171089
0.3503290122534	0.3712468643719
0.3504170133489	0.3713029727548
0.3505113373853	0.3713172415466
0.3506244006832	0.3713589971142
0.3507052593681	0.3713786430011
0.3507818344431	0.37147770037
0.3508550175989	0.3714783837349
0.3509590494465	0.3715806368284
0.3510364734196	0.371604140752
0.3511027859757	0.3717220930929
0.3512000592383	0.3718335008945
0.3512687488807	0.3718723658337
0.3513537929382	0.3719811347277
0.3514228236092	0.3722309101894
0.3515038094067	0.3723982801349
0.3515653051	0.3725061704498

0.3516735306743	0.3726147092011
0.3517427729737	0.372625467119
0.3518455214763	0.3728088064222
0.3519175329617	0.3728143615919
0.3519980431142	0.3729442961869
0.3521043402388	0.3731047577557
0.3521764474305	0.3731649208179
0.3522802116438	0.3732866843093
0.3523586922418	0.3733730195329
0.3524315635743	0.3733830132759
0.3524924718771	0.3734802979539
0.3525799565085	0.3736224701874
0.3526459787104	0.3737057476984
0.352719736118	0.3737259880935
0.3528164707574	0.3738185282175
0.3528884652428	0.3738721615514
0.3529785964575	0.3739867649112
0.3530372116416	0.3740918927091
0.3531088028147	0.3741788534768
0.3531619557078	0.374202792638
0.3532744761571	0.3744000903413
0.3533393744859	0.374427729881
0.3534356340221	0.3744452097471
0.3535154795327	0.374488053347
0.3536071441972	0.3745261998475
0.3536973400391	0.3746631966599
0.3537843705017	0.3748326603644
0.3538690466421	0.3749376772056
0.3539431899301	0.3749581805357
0.3540564588254	0.3753190000984
0.3541294752188	0.3754356305762
0.3541939493732	0.3754526291183
0.3542850547203	0.3755854802319
0.3543514137999	0.3756255202897
0.3544054673155	0.375791819778
0.354498256748	0.3762177105866
0.354583142941	0.3762485778544
0.3546640957377	0.3762884235129
0.3547259120827	0.3763414257493
0.3548028329474	0.376356183702
0.3548842722643	0.3763882495646
0.3549778904986	0.3763942091338
0.3550676450335	0.3765824074882
0.3551251846418	0.3766781288307
0.3552278602002	0.3768279614424
0.355303398058	0.3771143200188
0.3553620027735	0.3771292844149
0.3554574753382	0.3772021760223
0.3555401564916	0.3772718076591
0.3556363589337	0.3774080435489
0.3557218240228	0.3775348102036
0.3558189997895	0.3775650963872
0.3558989268661	0.3777172038779
0.3559908223069	0.3778802080525

0.3560510252753	0.3779544007421
0.3561017117893	0.3779548086767
0.3561796626984	0.3780339270559
0.3562696425421	0.3782195217426
0.3563704420478	0.3785141094508
0.3564458384172	0.3786120914903
0.3565154471267	0.3786403341899
0.3566047720071	0.3786751512057
0.3567411812617	0.3790653589901
0.3568500410421	0.3795022712015
0.3569606219346	0.3796692867303
0.3570305515071	0.37971112703
0.3571175841139	0.3797801617646
0.3572079321108	0.3797949290963
0.3572794293212	0.3798805997597
0.3573637853529	0.3799737001672
0.3574675310671	0.3800457824237
0.3575399706989	0.3801634518951
0.3576162476794	0.3802410069923
0.3576826081424	0.3803255764611
0.3577653424065	0.3805410549823
0.3578872922236	0.3810565332412
0.357961261777	0.3810997270676
0.3580331583636	0.3811905737285
0.3580876976918	0.3812539320614
0.358179686155	0.3814638108682
0.3582703665602	0.3815032464119
0.3583708486753	0.3816609766099
0.3584680815633	0.3818257780023
0.358565118335	0.3821848003333
0.3586601760287	0.3825171461766
0.3587413386479	0.3825844728332
0.358813120789	0.3826212518208
0.3589019386988	0.3829992871417
0.3589617722607	0.3830931518419
0.3590377404366	0.383168446827
0.359100466154	0.3832785957283
0.3591933247808	0.3834235703031
0.3592726409299	0.3834449032234
0.3593372979861	0.3836067100232
0.3594167822716	0.3836281823571
0.3594955953657	0.3837551778623
0.3595690953094	0.3837635438529
0.3596196057093	0.3837953766735
0.3596986802775	0.3840697181787
0.3597477818188	0.3840752148011
0.3598256833536	0.3842281967657
0.3599370112538	0.3843722652084
0.3600132737498	0.384418718059
0.3600911016449	0.3844391692699
0.3601464888615	0.3845202771537
0.3602237805467	0.3846058108383
0.3603387029309	0.3846130315659
0.3604098042477	0.3846186405579

0.360492954522	0.3846632164078
0.3605665402227	0.3846795028264
0.3606782711155	0.3848769878839
0.3607668686911	0.3848811305567
0.3608238675964	0.3849605534428
0.3608682166452	0.3849607717847
0.3609276867115	0.3849618017724
0.3609990814491	0.3851606116832
0.3610891443134	0.3852968890565
0.3611593993077	0.3853837467717
0.3612473653135	0.3854186738765
0.3613225505791	0.385553860091
0.3614146699013	0.3856665352136
0.3614630588324	0.3857403768678
0.3615428611904	0.3858148809801
0.3616096508026	0.3858297013438
0.3616784243997	0.3859668378192
0.3617555942492	0.3860664637923
0.3618701587978	0.3861804097177
0.3619784663122	0.3862593428862
0.3620710681154	0.386689130253
0.3621454014812	0.3867733679305
0.3621937827817	0.3867741884471
0.3622773713789	0.386895096805
0.3623444411811	0.386921523821
0.3624094411669	0.3869550673961
0.3624947788447	0.387120706775
0.3625723130275	0.3872415540793
0.3626458402741	0.3872760652407
0.3627171882365	0.3874701886935
0.3627786268637	0.387510478569
0.3628666684783	0.3876657508279
0.3629693986765	0.3876909683858
0.3630733886142	0.3877290822984
0.3631563377969	0.3878953360573
0.3632310941063	0.3879934618347
0.3633194979606	0.3880369126318
0.3634022652638	0.3880959104802
0.3634976812533	0.3883455627027
0.3635924313651	0.3884483419385
0.3636693339638	0.3885136578857
0.3637601417513	0.388528603802
0.3638276240033	0.3885700809645
0.3638888173507	0.3886839348475
0.3640078585162	0.3887013979176
0.3640793073998	0.3887640434203
0.364187166656	0.3888834115749
0.3642477705747	0.3889168107166
0.3643280345588	0.3890580222464
0.3644141827612	0.3890731272395
0.3644964277928	0.3891456314097
0.3645747823308	0.3892850241165
0.3646347997429	0.3894014339136
0.364716005077	0.3898773584577

0.3648155208441	0.3899935638903
0.364880463302	0.3901933156812
0.3649677087894	0.3902083899692
0.3650482974293	0.3903388216849
0.3651186367918	0.3904932055711
0.3652262683509	0.3905212027033
0.3652852888899	0.3905493297439
0.3653881462229	0.3906162111667
0.3654531043371	0.3906386784809
0.3655186062089	0.3906744474435
0.3656173590795	0.3907435259748
0.3656964543297	0.3907505994333
0.3657764516249	0.3909683410286
0.3658322542735	0.3910050573813
0.3658964637243	0.3910354959906
0.3659728497373	0.3910381676231
0.3660791742682	0.3911098854345
0.366171016189	0.3911767108251
0.3662469702179	0.3912948263196
0.3663423743975	0.3914716387404
0.3664348836579	0.3914961298779
0.3665391055144	0.3917543409846
0.3666302702115	0.3921090983236
0.3666985416602	0.3922019906841
0.3667823811986	0.3922528248159
0.3668359002626	0.3923196087019
0.3669214706009	0.3923751949717
0.3669874110298	0.392473444461
0.3670539704332	0.3925331934174
0.3671496326889	0.3927255694294
0.3672054209568	0.3927451934738
0.367312160729	0.3927692533962
0.3673763112097	0.3927783784562
0.3674849699249	0.3929322516516
0.367574758463	0.393015209117
0.3676852388351	0.3931924992538
0.3677661893356	0.3932267779661
0.3678436544164	0.3933821100787
0.3679488349831	0.3935119954674
0.3680219624807	0.3936238053437
0.3681076817211	0.3937834358561
0.3681754258166	0.3939080266922
0.3682787248142	0.3941144811641
0.3683713887642	0.3944773364682
0.3684583759714	0.3944964538681
0.3685455911222	0.3945508527544
0.3686292331797	0.3945528659585
0.3687258209293	0.3946771195724
0.3687721258057	0.3946999420291
0.368866025935	0.3947697426777
0.3689575236475	0.3948731700687
0.3690774019965	0.3948961378828
0.3691768352128	0.3949327455154
0.3692485412576	0.3950080276257

0.3693237491934	0.3951154703321
0.3693939860289	0.3951978596329
0.3695059409688	0.3953508940368
0.3695773962172	0.3953787192403
0.36964292805	0.3954414887485
0.3697239521031	0.3957280039394
0.3698316754345	0.3958389631685
0.369899881716	0.3960765402606
0.3699492167674	0.396102262054
0.3699970932211	0.3961550136228
0.370096414641	0.3962405704651
0.370159519126	0.3962701624405
0.3702588917938	0.3965056774199
0.3703327028595	0.3965215597849
0.3704109479374	0.3965509117719
0.3704739152205	0.3966897903312
0.3705352405611	0.3968522651788
0.3706039712012	0.3968906633286
0.3706825427385	0.3970036747306
0.3707938536312	0.3974341702261
0.3708882994047	0.3976002896301
0.370962534325	0.3977124758269
0.3710232478975	0.397735716877
0.3711044102934	0.3978089954736
0.3711897810596	0.3979239961718
0.3712818426001	0.3979344017257
0.3713897243167	0.3980485337854
0.3714642024693	0.3982127229658
0.3715503977493	0.3983163757397
0.3716429102223	0.3983257724743
0.3717086100375	0.3986822598509
0.371774947874	0.3987293981304
0.3718264128358	0.3987919045101
0.3719031071728	0.3990844146429
0.3719917106667	0.3992399820614
0.3720834361123	0.3992453799696
0.3721710560908	0.3992562401553
0.3722408894613	0.3993122918067
0.3723183372913	0.3994015886114
0.372410501603	0.3995370139783
0.3724718802229	0.3996954200208
0.3725378480359	0.3996986662329
0.3726003469097	0.3997908186503
0.3726602924657	0.3998067467619
0.3727438059517	0.3998317653305
0.3728177578813	0.3999260351313
0.3729008660834	0.3999909235551
0.3730013584334	0.4000282955925
0.3730598417334	0.4001102072217
0.3731325536368	0.4001716599761
0.3732101174761	0.4003651549773
0.3732910902011	0.4003675875126
0.3733662632099	0.4003789753965
0.3734323492248	0.4003865114717

0.3734953652163	0.400411737641
0.3735909969403	0.4005725594417
0.3736646292848	0.4006390794187
0.3737243338616	0.4007347690496
0.3737956137978	0.4007680246264
0.3738596460274	0.4008010450121
0.3739281410811	0.4008013888575
0.3740514829992	0.401424977417
0.3741377584333	0.4016384185415
0.3742007783554	0.401649598964
0.374260150801	0.4016860360472
0.3743834175794	0.4018120668907
0.3744744782066	0.4018677480988
0.3745540812234	0.4019612485505
0.3746685026474	0.4019930502278
0.374762194467	0.4021605636238
0.3748335825986	0.4022124899903
0.3749101499965	0.4022768707003
0.3750031418446	0.4023485815357
0.375070802107	0.4023997276506
0.3751466654833	0.4025693138911
0.3751931195572	0.4025829366348
0.3753324398905	0.4031120409702
0.3754095980174	0.4031230873458
0.3754730798989	0.4031372008764
0.3755584300017	0.4031552470656
0.3756457402843	0.4033724025933
0.3757092617333	0.4034841112057
0.3757777688045	0.4035186849603
0.3758515644606	0.403599017819
0.3759278576372	0.4036067959625
0.3760350926455	0.4036628192109
0.3761192100386	0.4038701671638
0.3762139663251	0.4039477570827
0.3762895360489	0.4040206597844
0.3763875092773	0.4040544238833
0.3764564580866	0.4040604446519
0.3765114010771	0.4041583679629
0.3766077629725	0.4041747030249
0.3767038198624	0.4043271346884
0.37677829777	0.4044284948143
0.3768346644879	0.4045720467406
0.3769072791673	0.4046352082228
0.3769871195064	0.4047212740273
0.3770725396663	0.4047367938464
0.3771624916971	0.4047688122186
0.3772485046434	0.4048083537414
0.3773331945525	0.4048575357303
0.3773922073376	0.4048625323455
0.3774621200789	0.4050033167579
0.3775446069474	0.4051709814082
0.3776452168703	0.4052802705706
0.3777196908324	0.4053386551641
0.3778045644749	0.4054214576748

0.3778719463232	0.405601952128
0.3779324872396	0.4056475364144
0.3779933718629	0.4056592032127
0.3780906623983	0.4057542123723
0.3782027204902	0.406192917783
0.3782903698065	0.406275330692
0.3783507398718	0.4063883242313
0.3784193690546	0.4064576285387
0.3784964124935	0.406471245264
0.3785646256979	0.4064936088859
0.3786359220981	0.4066086507955
0.3787340220366	0.4066519620542
0.378823145116	0.4067107468446
0.3789045344673	0.4069609107894
0.3789885819451	0.4073376141006
0.3790520286626	0.4073898681889
0.3791254339489	0.407415555079
0.3791968454975	0.4077328765852
0.3792644218212	0.4077454033375
0.3793236339481	0.4077963092034
0.3794241335029	0.4080007529662
0.3794928687871	0.408071245018
0.3795736886144	0.4080859105888
0.3796257576995	0.4081087124209
0.3797133445706	0.4081301966641
0.3797802969045	0.4081379286388
0.3798608809844	0.4081519913882
0.3799178629442	0.408185489563
0.3799810449363	0.4081901661506
0.380058621381	0.4084746563014
0.3801475644395	0.4086145542178
0.3802094936837	0.4086600435571
0.3802829076249	0.4087433428617
0.3803821818932	0.4088092516011
0.3804538648126	0.4089695028718
0.3805424751735	0.409058450036
0.3806148452965	0.4090610099016
0.3807140938171	0.4095555500987
0.3807787175308	0.4096586921275
0.3808200827824	0.4096615344607
0.3809247809713	0.409822887877
0.3809882522508	0.4098470629645
0.3810719038254	0.4099712285245
0.3811744021994	0.410025678668
0.3812550854876	0.4102176700664
0.3813337828124	0.4102220837467
0.3814291630018	0.410270101104
0.3815008430434	0.410407283441
0.3816076831808	0.4105439294554
0.3816977502514	0.4108987058267
0.3818017714426	0.4112726629881
0.3818847479027	0.411363116431
0.3819832402437	0.411584436968
0.3820593606542	0.4116306471501

0.3821481769003	0.4116441343706
0.3822085527514	0.4116868585589
0.3822917708843	0.4120362215626
0.3823525355194	0.4120560937774
0.3824144465938	0.4120824607262
0.3824975495124	0.4121851830253
0.3825640971968	0.412323577152
0.3826559435288	0.412325583014
0.3827608542474	0.4124035281525
0.3828476082075	0.4124189015128
0.3829034607006	0.4125473556099
0.3829799773163	0.4126702717515
0.3830557368203	0.4126831633936
0.383153869018	0.4128148284252
0.3832086250186	0.4128652271186
0.3832785725545	0.4129341347921
0.3833598754473	0.4129773133454
0.3834448426338	0.4130142048589
0.3835284699233	0.4131276111655
0.3836086968277	0.4131616929603
0.3837021509531	0.4133293854932
0.383765205012	0.4133303014737
0.3838349202135	0.4133514281557
0.3839145435988	0.4134062471691
0.3840082255659	0.413417029193
0.3841117239809	0.4137444031797
0.3841928885268	0.4138047271398
0.3843100231955	0.4141680140776
0.3844018157513	0.4142723067665
0.3844780658937	0.414276640653
0.384545896343	0.4143676698489
0.3846442000881	0.4145290908775
0.3847128381921	0.4145426421216
0.384806332096	0.4146611089859
0.3848789881475	0.4148222068063
0.3849678039965	0.4148758094275
0.385073297604	0.4149303623464
0.3851651606982	0.4150719086326
0.3852654149948	0.415309184508
0.3853794124636	0.4153268475464
0.3854900591733	0.4153747512295
0.3855635145456	0.4154652620373
0.3856481767718	0.4156372538998
0.3857259545772	0.4158808912412
0.3858024563197	0.4160196199296
0.3858920016034	0.4162961211913
0.3859589150587	0.4163182422403
0.3860510162264	0.4163470896209
0.3861627830153	0.4167060523943
0.386233139049	0.4169049694067
0.3863440113764	0.4171780756279
0.3864004471937	0.4171942322716
0.3864707195028	0.4171993331118
0.3865829583454	0.4174808050044

0.3866624946133	0.417514717953
0.3867456377682	0.4175495778984
0.386861252106	0.4176624430473
0.3869522729917	0.4177483866402
0.3870639577798	0.4179351356969
0.3871799570383	0.4181400008761
0.3872598982549	0.4181963153212
0.3873573456187	0.4185314012554
0.3874673627078	0.4190582872365
0.3875661901142	0.4192953651627
0.3876543637357	0.419360781302
0.3877365634615	0.4196348115626
0.3878103718891	0.4196876108786
0.3878693824286	0.4197591128873
0.3879423334667	0.4198671738748
0.3880176419746	0.4198881657389
0.3881027700601	0.4199717019551
0.3881573185845	0.4200087156974
0.3882220196334	0.4200238685661
0.3882912011263	0.4200447264255
0.3883794900632	0.4201555011411
0.3884815350427	0.4203056294173
0.3885513898368	0.4204527563126
0.3886607784237	0.4210446335676
0.388725453638	0.4210930840999
0.3888136630511	0.4212043763351
0.3889300332667	0.4214896072796
0.3890314459861	0.4215345109659
0.3891006023944	0.4216761539089
0.3891662946696	0.4217969630347
0.3892730072066	0.4220450447992
0.3893335174932	0.4220576816818
0.3893843212502	0.4220787448994
0.3894608110741	0.4222635794012
0.3895325451073	0.4222745034683
0.3896346468274	0.4223555472282
0.3897037621136	0.4224742526333
0.3897747460498	0.4224820179835
0.3898510667662	0.4224875142661
0.3899508006425	0.4225799077061
0.3900823467546	0.4227354692855
0.3901495239494	0.4228244188911
0.3902371772468	0.4228688851376
0.3903273583826	0.4229478476232
0.3903997680251	0.4230668237971
0.3904535090002	0.4231458637819
0.3905526010577	0.4237125147473
0.3906236154898	0.4237860117388
0.3907080525122	0.4237975838394
0.3907825658438	0.4238901422358
0.3908700011655	0.4240143012469
0.3909687778188	0.4240635827221
0.3910492387736	0.4240990885533
0.3911219068634	0.4241079684236

0.3911885661236	0.4241377390003
0.3912594978601	0.4241425455093
0.3913208675884	0.4242154310211
0.3914076133416	0.4243543763628
0.3914648759501	0.4244890971071
0.3915406994403	0.4245264069426
0.3916142908789	0.424580721607
0.3916994815964	0.4245818159885
0.3917732816727	0.4245873369326
0.3918569783262	0.4245937589501
0.3919254506382	0.4245957012206
0.3920301543037	0.4248218596962
0.3921050907767	0.4250039568494
0.3921979858896	0.4250643592195
0.3923176289107	0.4251876053845
0.3923611570909	0.425204253278
0.3924330925082	0.4252193921551
0.3925034738496	0.4254169182312
0.3925853658801	0.4255923752402
0.3926817648211	0.4259740345074
0.3927420930927	0.4259944996147
0.3928071489748	0.4260408122686
0.3928973073816	0.426301913497
0.3929604105327	0.4263075931983
0.3930698474966	0.4263198171868
0.3931530704833	0.4263432918464
0.393254458997	0.4263712688473
0.3933403075784	0.4264600125347
0.3934283678737	0.4265725048546
0.3935104595869	0.4266684087029
0.3936184048098	0.4269193662592
0.3936936703106	0.4269790118069
0.3938001884364	0.4272271194702
0.39389354221	0.4273531709569
0.3939866979514	0.4274883086502
0.3940567777213	0.4275261614914
0.3941532503872	0.4277225060288
0.394243329849	0.4278200008098
0.3943601778082	0.427904520359
0.3944270038265	0.4280095432028
0.3944877504848	0.4280340873427
0.3945341638373	0.4281733694815
0.3945898231148	0.4281892845648
0.394663300176	0.4282715735318
0.3947433765084	0.4283532862944
0.3948200252953	0.4284224841265
0.3948881773601	0.4284431415523
0.3950024869531	0.4285074669089
0.3950814303495	0.4285076354979
0.3951557154837	0.4285281403407
0.3952521316285	0.4286160484613
0.395323478611	0.4287395336896
0.3954029056189	0.4289184707736
0.3954849997714	0.4289345466886

0.3955595869048	0.4289953373441
0.3956574482575	0.4291028037025
0.3957423665016	0.4293025707885
0.3958082616793	0.429355156291
0.3959349687817	0.4295243074858
0.3960188173485	0.4295390850119
0.3961037057551	0.4296580766299
0.3961834957549	0.4296719546584
0.3962501172909	0.4297468428607
0.396333703333	0.4297721849457
0.3964154550344	0.4297994616615
0.3965114460536	0.4298571922743
0.3965867321901	0.4299263038039
0.3967010561607	0.4303282957052
0.396803328761	0.4303468698263
0.3968839232031	0.4304157626957
0.3969521183047	0.4304504802132
0.3970360819803	0.4304551118205
0.3971175541931	0.4304949169538
0.3972380269754	0.430875108516
0.3973285900785	0.4310261364293
0.3974184611521	0.4310862472763
0.3975108196093	0.4311605961908
0.397590415802	0.4311615149265
0.3976801295634	0.4312026523101
0.3977633866723	0.4312545240739
0.3978682070692	0.4312580647522
0.3979882860347	0.4314458522815
0.3980799271386	0.431709831129
0.3981644892405	0.4317436196722
0.3982212918067	0.431815955937
0.3982744183243	0.4318708636527
0.3983382818932	0.4318735926697
0.3984165798196	0.43206518632
0.398499880021	0.4322094729099
0.3985837856333	0.432282333485
0.3986696264992	0.4323264814854
0.3987587102196	0.4323288967573
0.3988648493241	0.4325137394802
0.3989511772143	0.4325380474045
0.3990278376438	0.432648137496
0.3990801250583	0.4326752388544
0.3991817906069	0.432970284439
0.3992865393799	0.4330819488411
0.3993476735925	0.433131050593
0.399425140575	0.4331382489879
0.3995212599611	0.4335339168378
0.399594049863	0.4335987165768
0.3996787126467	0.4337443730066
0.3997524141765	0.4339436211865
0.3998396255804	0.4339552570701
0.3999221790391	0.4340549255252
0.399982110393	0.4341020199785
0.4000610764982	0.4341070760256

0.4001268408261	0.4342478786942
0.400210199269	0.4343243879107
0.4002886018385	0.4346233173324
0.4003591254379	0.4347901689716
0.4004466640422	0.4348595426022
0.4005181779539	0.434937015755
0.4005977770779	0.4350749653381
0.4006993814869	0.4356203639522
0.4007775673009	0.4356411073413
0.4008681022487	0.4357865494739
0.40094365821	0.435950166249
0.4010221437862	0.4361424982528
0.4010784082161	0.4361794093257
0.401181994962	0.4361952003915
0.4012761864149	0.4364060521475
0.4013582501875	0.4364359889768
0.4014533956246	0.4364679584383
0.4015389361837	0.4364847559224
0.4016309613229	0.4365760981906
0.4017289076398	0.4367289320655
0.4018010570515	0.4368655272937
0.4018743301316	0.437003786612
0.401947639825	0.4371182910469
0.4020419300851	0.4371779663447
0.4021448370018	0.4372115447732
0.4022352064487	0.4372120376265
0.4023083162277	0.437226356412
0.4023872163768	0.4372350737065
0.4024581475233	0.4373576882107
0.4025132397884	0.4373871849414
0.4025835251782	0.4374279748188
0.4026590879647	0.4374444745638
0.4027246504285	0.4374506524057
0.4028097715629	0.4375006312184
0.4028781415447	0.4375039168698
0.4029641004138	0.4376837909485
0.4030460063097	0.4377861216505
0.4031354690771	0.4381759204429
0.4032205736023	0.4382529217536
0.4033258066114	0.4383537372521
0.4034262327876	0.4384813142393
0.40348586389	0.4385414848606
0.4035502664238	0.4386381102255
0.4036220624913	0.4386622349477
0.4036914278744	0.4387060540532
0.4037956302335	0.4388877848775
0.4038925469299	0.4389053109993
0.4039763287577	0.4389382489499
0.4040532611911	0.4390003263976
0.4041414096797	0.43917836956
0.4042417593301	0.4394694949565
0.4043106767939	0.4397075984546
0.4043950647096	0.4398608855753
0.4044725686419	0.4400944768805

0.4045621425295	0.4401521847102
0.4046220273735	0.4401591166147
0.4046943415743	0.4401699169176
0.4047520646568	0.4401812421943
0.4048289181452	0.4402362351587
0.4048847348659	0.4403229630324
0.4049545857855	0.440347762557
0.4050378353013	0.4403605488651
0.4050979522267	0.4404567766223
0.4051980773508	0.4405943008826
0.4052631050134	0.4408165271695
0.4053414522676	0.440864363242
0.4054223538745	0.4408680826859
0.4055012521377	0.4409553109856
0.4055490729323	0.4409771203594
0.4056344873369	0.4410498561825
0.4057001742769	0.4410963437626
0.4057660626355	0.4411087707072
0.4058223546492	0.4411598883086
0.4059203412097	0.4412945419526
0.4059869712952	0.4413705390133
0.4060440328897	0.4413762076274
0.4061183589657	0.4413860210293
0.406207048559	0.4416530036835
0.4062724080711	0.4417616852479
0.40634578269	0.441809070539
0.4063977640736	0.4418318380666
0.4064872634128	0.4418557159606
0.4065529604149	0.4418754891584
0.4066311519312	0.4418903227391
0.4067008774381	0.4419183024575
0.4068209889992	0.4419738311672
0.4068794126434	0.4420446074082
0.4069681776398	0.4422068821441
0.4070586731007	0.4422204388851
0.4071350028604	0.4422623414337
0.4071878680092	0.4423567759517
0.4072878699049	0.442380501348
0.4073500244575	0.4424009834078
0.4074118982223	0.4424097528646
0.4075270982478	0.4424601108126
0.4075871080448	0.4424633681349
0.407649657422	0.4424713639295
0.4077321347723	0.4427096795227
0.407837761182	0.4427296643435
0.407917003486	0.4429932678046
0.4079976010339	0.4430163994185
0.4080903747556	0.4430902102345
0.4081872726725	0.4430925039167
0.4082620965478	0.4431844417883
0.4083713958919	0.4431920728121
0.4084285235675	0.4432488879695
0.4085144314697	0.4435575564739
0.4085695337112	0.4435951173136

0.4086571765641	0.443634236933
0.4087452983415	0.4436342626393
0.4088158879218	0.4436669137928
0.4088752101448	0.443713868596
0.408943122556	0.4437426903669
0.4090045043737	0.4437636042962
0.4091169590902	0.4437949111435
0.4091801444006	0.4438293028764
0.40927191085	0.4438775585638
0.4093658953012	0.444169264822
0.4094345291532	0.4442615639382
0.4095335267639	0.4443430224872
0.4095966736484	0.4443631117426
0.4096545861942	0.4444000267805
0.4097024203865	0.4444142243552
0.4097741529352	0.4444245069041
0.4098593281704	0.4444795851956
0.409919766187	0.4445307852137
0.4099998589246	0.444667987846
0.4100717115934	0.4448791332798
0.4101380340018	0.4450031778281
0.4102109658333	0.4450396699111
0.4103056184479	0.4450902728217
0.4103888489349	0.4451536640101
0.4104766769282	0.4451845222175
0.4105593030999	0.4452827971802
0.4106474753124	0.4453364933019
0.4107306033171	0.4453836464939
0.4108181972219	0.4456584255325
0.4108683379577	0.4456813197582
0.4109483045549	0.4457048248697
0.4110421368203	0.4459418934353
0.4111394591446	0.445978842938
0.4111841853838	0.4460117575131
0.4112671999657	0.4461086131067
0.411383206234	0.4464080999776
0.4114744985154	0.4466773380324
0.4115301734071	0.4467092313793
0.4116062549491	0.4467261046694
0.4116971933941	0.4468377339008
0.4117652748798	0.4468761386005
0.4118468539434	0.4468845778303
0.4119049261424	0.4468873540009
0.4119997681609	0.4469395868279
0.41211094684	0.4472040303875
0.412208246008	0.4474775054757
0.4122761773735	0.4474834772597
0.4123702986074	0.4475518172215
0.412460372386	0.4476055664107
0.4125317618128	0.4476794608701
0.4125958912621	0.4476953986421
0.4126670623417	0.4477048287251
0.4127336499906	0.4478382773076
0.4128592616049	0.4481324490518

0.4129194879579	0.4481621717071
0.4129861152389	0.4482505739943
0.4130589946511	0.4482606084765
0.4131675854536	0.4484075596186
0.4132358257823	0.4484335286728
0.4133108442095	0.4486018402621
0.4133836924876	0.4486056122611
0.4134877641048	0.4486211304966
0.4135903260937	0.4486236433668
0.4136626884185	0.4487987168752
0.41373567029	0.4488600862504
0.413788467293	0.4488948557762
0.4138457358173	0.449094820984
0.4139035296195	0.449105898305
0.4139802164733	0.4492905279211
0.414039587843	0.449395727467
0.4141148185197	0.4495745507341
0.4142024759368	0.4496447277612
0.4143380344574	0.449909809888
0.4144293308974	0.4499497249382
0.4145125909004	0.4499540985704
0.4145642336205	0.4499909934067
0.4146451940567	0.4501184336336
0.4147436522772	0.4501865782472
0.4148235321722	0.4502098146992
0.4149106967047	0.4506094729353
0.4149659169601	0.4506478957694
0.415049177849	0.4506508654465
0.4151209686631	0.4506692735387
0.4152249286744	0.4507748342213
0.4152926682718	0.4508264977193
0.4153639767754	0.4509244358966
0.4154440678983	0.4509295485313
0.4155241792343	0.4510483522473
0.4156125497064	0.4511231816467
0.4157120579263	0.4514216339449
0.4157835859839	0.4514258735681
0.4158772254715	0.4515064045127
0.4159353986688	0.4515301814607
0.4160237145715	0.4515581230944
0.4160879959236	0.4515933357351
0.4161783747246	0.4516839600127
0.4162574137715	0.4516937090177
0.416336811175	0.4517042540787
0.4164112223854	0.4517110736652
0.4165179976194	0.4519098904556
0.416593082145	0.4520768411906
0.4166764996468	0.4520926044998
0.4167474439908	0.4521815294228
0.4168245322954	0.4522294416591
0.4168780629619	0.4522546954336
0.4169478760048	0.4523826947811
0.4170232038844	0.4524081004154
0.4170990229109	0.4525081838889

0.4172150197011	0.4525091948025
0.4172964593681	0.4529093238833
0.4173837539426	0.4530030350579
0.4174560300012	0.4531321936867
0.4175378989404	0.4531680267716
0.4175998738802	0.4532731258462
0.41768445322	0.4535258950969
0.4177360336431	0.4535275157653
0.4178268850049	0.4536942724651
0.4179064894557	0.4537161061951
0.4179854353395	0.4537929818827
0.4180742676105	0.4537981254508
0.4181246895966	0.4538006016455
0.4182190755733	0.4542404809929
0.4182873419101	0.4544045620269
0.4183842752961	0.4544527559334
0.4184520439068	0.4544837046797
0.4185388776789	0.4545089403525
0.4185953864854	0.4545329537208
0.4186824967657	0.4546307123991
0.418765611776	0.4547339570431
0.4188365041147	0.4549036719355
0.4189082449357	0.4549944464477
0.4189878129334	0.4550921304343
0.4190504370642	0.455142612691
0.4191186592056	0.4551622798775
0.419196352344	0.455346197229
0.4192764882504	0.4556150229619
0.419350994779	0.4556496962795
0.4194311556426	0.4557971310059
0.4195744607859	0.4565581339638
0.4196466332775	0.4565618149978
0.4197161136468	0.4565669644578
0.419789365654	0.4567510208246
0.4198848242882	0.4570114890798
0.4199261676271	0.4570130780341
0.4199811073607	0.4570420019115
0.4200426013284	0.4571083061869
0.4201356565104	0.4572061204423
0.4202328554886	0.4572404427644
0.4203114081647	0.4572569012907
0.4203862594679	0.4573456850512
0.4204850872637	0.4574118831635
0.4205770668076	0.45747795271
0.4206307172888	0.4575211376508
0.4207422827241	0.4577351634027
0.4208407051558	0.4577768764976
0.4209473168041	0.457825535018
0.4210304779618	0.4578429219834
0.4211285638787	0.457868019915
0.4212063726492	0.4579682602673
0.421287476702	0.4579765129479
0.421379887165	0.4580673851287
0.421436094899	0.4580701096509

0.4215068463637	0.4581832796684
0.4215657398294	0.4581897884953
0.4216659427744	0.4582690295358
0.4217272343571	0.4583737657706
0.4218008112967	0.4585329897686
0.4218773280378	0.4586368772512
0.4219358006941	0.4586834164679
0.4220299751198	0.4587853911083
0.4221267027574	0.4587885660868
0.4221909920757	0.4589568426063
0.4222968281535	0.4589586338544
0.422401215863	0.4590443202736
0.4224688707751	0.4591627040667
0.4225778516549	0.4592348838605
0.422648286315	0.4592406694561
0.4227267476287	0.459311918008
0.4227960886204	0.4593661865102
0.4228926371079	0.4595616213942
0.4229759461058	0.4597918865907
0.4230342945481	0.4598358425684
0.4231097924003	0.4599533713436
0.4231837476317	0.4600583635054
0.423269095275	0.4600894842871
0.4233713966696	0.4601787287296
0.4234544645851	0.46027127468
0.4235281271249	0.4602809123894
0.4236074173842	0.4602950059651
0.4236790538067	0.4603480896819
0.4237709596577	0.4604849280537
0.4238293448398	0.4605579654525
0.4238955248661	0.4605684695428
0.4239678274704	0.4606180903694
0.4240501453087	0.4608882111043
0.424140237442	0.4610090702426
0.4241884397363	0.4611409363575
0.4242806842343	0.4611887864793
0.4243535575895	0.4613975860719
0.4244164276806	0.4614324781984
0.4245076830742	0.4614845045702
0.4245865315224	0.4615403739539
0.4246782148814	0.4615441289377
0.4247536592499	0.4615773908983
0.4248946968001	0.4619046122827
0.4249461641466	0.4619685602555
0.4250402988732	0.4620428814962
0.4250990484745	0.4620755152986
0.4251590842309	0.462200312639
0.4252313217642	0.4622314177386
0.4253405320742	0.462587448353
0.4254285085037	0.4627038598758
0.4255303217586	0.4628328026139
0.4256530922431	0.4632570058035
0.4257324104925	0.4632928809513
0.4258076555999	0.4635103071483

0.4258782023334	0.4636889966749
0.425939739788	0.4637033662214
0.4260356707019	0.4639060087398
0.4261272705505	0.4639274930233
0.42621452303	0.4640781475777
0.4262838589452	0.4643240326657
0.4263642340802	0.4644697623622
0.426440431974	0.4645348188007
0.4265290930375	0.4647080288165
0.4266166001282	0.4647242604536
0.4267071342316	0.4648484245008
0.4267856174167	0.4649238111258
0.4268587119872	0.4650984300108
0.4269656534599	0.4654849849302
0.4270483335638	0.46551171186
0.4271305946216	0.4657933243415
0.4271972794065	0.4658087865661
0.4272618118684	0.4658111387761
0.4273452574887	0.4660009017034
0.4274173802696	0.4660479253461
0.427475484821	0.4661141236878
0.4275716410755	0.4663379013535
0.4276432637129	0.4665089854077
0.4277288543656	0.4667239911982
0.427807564417	0.4667761765312
0.4279092574764	0.4667806460188
0.4279789560829	0.4668436932577
0.4280744264553	0.4668874650496
0.4281592183392	0.4669342229069
0.4282657276353	0.4672578263206
0.4283467356753	0.4673857714041
0.4284189247109	0.4675484032343
0.428523267455	0.4677528261971
0.4286263880174	0.4677561148862
0.4286856956074	0.4678090051947
0.4287640993481	0.467927433676
0.4288300262443	0.4680990118295
0.4289267380486	0.4681983186919
0.4290022759265	0.4683319216863
0.4290794080777	0.4683358867005
0.4291826236702	0.4684962378453
0.4292735437924	0.4685309502989
0.4293642529432	0.4685577536253
0.4294106584323	0.4685902236236
0.4295448807341	0.4691035001551
0.4296298577296	0.4691414058508
0.4297167459684	0.4691617517087
0.4297935673906	0.4692133178958
0.4298757000829	0.4692323255146
0.4299253493254	0.4692466679625
0.430009482848	0.4694969789542
0.4300839063963	0.4695705753475
0.4301588224444	0.4696138900526
0.430235088045	0.4697140208016

0.4302934485198	0.4697163785822
0.4303920072071	0.4698867798673
0.4304885399516	0.4699157983469
0.4305526427159	0.4700676175043
0.4306344186369	0.4701476470322
0.4307253853385	0.4702109391353
0.4308160084198	0.4702641924256
0.4308926333697	0.470384680875
0.4309640956253	0.4705457236448
0.4310384736106	0.4705677915994
0.4311247646025	0.4706810188446
0.4311951721482	0.4707537530645
0.4312971352641	0.4710567864019
0.4313738381726	0.4711476677955
0.4314503571353	0.4712289931745
0.4315166828389	0.4713370617127
0.4316186269463	0.471686157394
0.4317058355025	0.4718372871232
0.4317796530859	0.4719344838531
0.4318622316758	0.4719929989599
0.43194035696	0.4720143279281
0.4319892873498	0.4720216564964
0.4320736472324	0.4721409626546
0.4321416288143	0.4722073898091
0.4322335003592	0.4722967983371
0.4323227560136	0.4725087904999
0.4323915492357	0.4725254401821
0.4324545457485	0.4725483640898
0.4325353794547	0.4725508543996
0.4326006544332	0.4726139492137
0.4326833915267	0.4727946809408
0.432769177452	0.4728272410725
0.4328834118311	0.4731535011771
0.4329471775264	0.4732089356002
0.4330239556099	0.4734367922376
0.4331214130456	0.473475177024
0.433185545108	0.473489459957
0.4332538672061	0.4735121417334
0.4333437598891	0.4735702503038
0.4334571219612	0.4736308744693
0.433542316113	0.4736631065083
0.4336088908809	0.4736942482239
0.4336993535709	0.4738329362855
0.4337821849756	0.473869145849
0.4338535693628	0.4742063009768
0.4339450005942	0.4742220180988
0.4340440173171	0.4744620981477
0.4341114650199	0.4744821088581
0.4341974308006	0.4744999475269
0.4342613716049	0.4745108184961
0.434372565862	0.4745140373502
0.4344533751929	0.4745518001383
0.4345152282893	0.4746062549044
0.4345860797968	0.4746101048531

0.4346466120181	0.4746930242676
0.4347196894579	0.4748466937739
0.4348522348829	0.4749877116607
0.4349364950659	0.475370423698
0.4350185883882	0.4753739667432
0.4351212295187	0.4754467763673
0.4352051473964	0.4754531898724
0.4352911081904	0.475482557537
0.4353539007243	0.4755149502523
0.435425186915	0.4758379875401
0.4354998180938	0.4758573802254
0.4355744380195	0.4759136552636
0.4356390484471	0.4759370055583
0.4357247146038	0.4759399112276
0.4358483608071	0.47619523476
0.4359459348713	0.4762485862369
0.4360218898821	0.4764214717177
0.4360994599831	0.4764668670285
0.4361613295741	0.4765846959321
0.4362221345091	0.4766946783932
0.436286758594	0.4767325725634
0.4363638806578	0.4768074636182
0.4364331085188	0.4768977780896
0.4365117826533	0.4769314300959
0.4365682595121	0.4770380950764
0.4366408366563	0.4770434655898
0.4367286232373	0.4771842773887
0.4368168961429	0.4771921666424
0.4368982386597	0.4774199777774
0.4369649048002	0.4774769080483
0.4370270534777	0.4774991445611
0.4370920377625	0.4775494296751
0.4371687080306	0.4775915022472
0.4372791732814	0.4779960663827
0.4373610805134	0.4781762047512
0.4374347235216	0.4782609899018
0.4375364819269	0.4785959694916
0.4376089647138	0.478662274475
0.4376883494	0.4787184636725
0.43774446835	0.478732723368
0.4378232033881	0.4789553976379
0.4378954054074	0.4789970099024
0.4379417802357	0.4790038789099
0.4380365163325	0.479018586346
0.4380991861719	0.479045339639
0.4381683028752	0.4790595888991
0.4382457694527	0.4793342523739
0.438310909493	0.4793798827581
0.4383686074268	0.4794451121985
0.4384331093323	0.4794462639534
0.438503853555	0.479459945015
0.4385734314547	0.4795950692383
0.4386661485684	0.4796385638568
0.4387479146286	0.4797245303834

0.4388148072786	0.4798473063497
0.4388756725256	0.4798501329118
0.438941960963	0.4798851932543
0.4390406088849	0.4799799428308
0.4391178925277	0.4800592812098
0.4391902791248	0.4800861485216
0.4392569882368	0.4802956094461
0.4393253378261	0.4803028791491
0.4394381608484	0.4803303972325
0.4395131606926	0.4803648947129
0.4396010887554	0.4805863760366
0.4397047502126	0.4806763330534
0.4398043008516	0.4807364731786
0.4399069279795	0.481021472035
0.4399683570331	0.4811072468374
0.4400846235413	0.4811074477811
0.4401550289234	0.4811531979512
0.4402015510273	0.4811966083713
0.4402856813848	0.4812473205571
0.4403820642247	0.4813995550224
0.4404683419712	0.4814480786748
0.4405367693385	0.4816692866405
0.4406260104184	0.4816805209145
0.4407085941462	0.4817923409051
0.4408095348796	0.4818900978488
0.4408577082426	0.4819898951356
0.4409283431918	0.4821353947077
0.4410283613918	0.4821895082556
0.4411079296182	0.4822129871606
0.441188909442	0.4822195749131
0.4412911444254	0.4824063923299
0.4413826152437	0.4826863256909
0.4414655325287	0.4828293430101
0.4415562399829	0.4828589379424
0.44165124067	0.4828658156488
0.4417168339813	0.482887095606
0.4417741317981	0.4828951126964
0.4418490242387	0.482941584932
0.4419296912682	0.483094986047
0.4420197282963	0.4832216130654
0.4420819988195	0.4833030744067
0.4421815762927	0.4834510683638
0.4422594940721	0.483546475485
0.4423357096489	0.4836752508033
0.4424023191348	0.4836842308773
0.4424676283314	0.4837143643094
0.4425471905778	0.4837955826251
0.4426313924913	0.4840327530681
0.4426928954126	0.4840417114583
0.4427800314221	0.4840604441742
0.4428586539033	0.4840997198199
0.442913842471	0.484121516953
0.4429947735388	0.4841379313372
0.443073051214	0.484228137532

0.4431464131646	0.4843195224166
0.443248924562	0.4844327677532
0.4433212027043	0.4845591903977
0.4433892911941	0.4846248275817
0.44347666265	0.4846475429624
0.4435465992418	0.4846523681514
0.4436153329885	0.4847533915433
0.443689257842	0.484755239999
0.4437486208528	0.4848102078008
0.4438100002194	0.4848338349218
0.443904290667	0.484843794315
0.4439904373802	0.4850021851055
0.4440748800223	0.4851472773759
0.4441464068413	0.4852013630233
0.4442527787588	0.4856976698157
0.4443392300612	0.4858502647358
0.4444112230391	0.4859392206664
0.4444917228231	0.4864170702273
0.4445583021041	0.4864478749092
0.444633301847	0.4864670499823
0.4447109658567	0.4865198634067
0.4448431035296	0.4866277537766
0.4449329403886	0.4866813535741
0.4450018481008	0.4868037659488
0.4451085465341	0.4868785867174
0.4451687613982	0.4869063915943
0.4452252065186	0.4869261604925
0.4453206890207	0.4871417045289
0.4453907345557	0.4871591242289
0.4454760345872	0.4873136803183
0.4455425315219	0.487468999767
0.4456332954371	0.4878322893373
0.4456860579784	0.4878462871998
0.4457552750483	0.4878849199249
0.4458287433468	0.4879007362566
0.4459322233059	0.4879676316093
0.4460269792163	0.4880430680852
0.4461307059709	0.4881102420649
0.4462059322363	0.4882834239252
0.4462832047057	0.4883430176816
0.4463712981886	0.4883607714736
0.4464447220028	0.4883960160498
0.4464939451638	0.4884123046583
0.4465728309557	0.4885105752391
0.4466701575363	0.4886839979083
0.4467539059543	0.4888606371506
0.4468295285086	0.4888663565078
0.4468945238597	0.4888737759353
0.4469859066042	0.4890136433862
0.4470922079442	0.4891053227887
0.4471449138601	0.4891570136457
0.4472239935364	0.4892451602251
0.4472981721307	0.4893198582122
0.4473657920627	0.4894720478872

0.4474298345232	0.4895636502855
0.4475141308517	0.4899235591588
0.4476201152661	0.490220784463
0.4477125038568	0.4904205221349
0.4477819956925	0.4904759701873
0.4478450373712	0.490630958889
0.4479177309015	0.49063535635
0.4480066889219	0.4906493456829
0.4480919907081	0.4907529959057
0.4481587377413	0.4909463815289
0.448254926946	0.4911426415147
0.4483205742644	0.4911925511108
0.4483893695736	0.4912735390071
0.4484659284538	0.4913621887961
0.4484982995292	0.4914073533194
0.4485580869132	0.4915066297384
0.4486212097003	0.4916656797199
0.448725816359	0.4917313359564
0.4487966282951	0.4917975729656
0.4489003650315	0.492300065626
0.4489711576888	0.4923577499297
0.4490495944957	0.4923710287756
0.449122930575	0.4925192563227
0.4492205761496	0.4926625113128
0.4493138330568	0.492731045741
0.4493904489769	0.4927416589636
0.4494645866622	0.4929484568216
0.4495377893002	0.4931046417154
0.4496099139079	0.4931637755674
0.4496774034937	0.4932501569541
0.4497733980027	0.4935020784509
0.4498533829105	0.4936290546954
0.4499335665876	0.493736891096
0.450046032149	0.4938663820563
0.450118627034	0.4939356593365
0.4502020096883	0.494107446141
0.450272441179	0.4941688421735
0.4503491213657	0.4941695989893
0.4504051304199	0.4941802366353
0.4504804282988	0.4942876055568
0.4505336836851	0.4943283154671
0.4506075558851	0.4943928964615
0.4506789451255	0.4943942445375
0.4507980860155	0.4947710934097
0.4508700728745	0.4948754896784
0.4509309527717	0.4949469225839
0.451008557614	0.4949550698118
0.4510853321217	0.4950120635855
0.4511565566269	0.4951023156284
0.4512192705752	0.4952157401497
0.4512997148589	0.4952164493706
0.4513635446373	0.4952466016436
0.4514248720845	0.4952919313889
0.4514725327736	0.495356716536

0.4515144242258	0.4953719141194
0.4515726968295	0.4953815597949
0.4516387304208	0.4954171124855
0.4517388602912	0.4956178038675
0.4518262287766	0.4956237163752
0.4519095945565	0.4956372273032
0.4520092097232	0.495832980847
0.4520923606427	0.495834287511
0.4521850984714	0.4960819309752
0.452259770119	0.4960844423848
0.4523340610108	0.4961466493886
0.4524070316297	0.4966589263219
0.4524985889066	0.4967376667667
0.4525419118467	0.4967651720021
0.4526517498675	0.4973264332155
0.4527197856413	0.4973289385986
0.452783878499	0.4973351320502
0.4528381033618	0.4973519220174
0.4529077139373	0.4975198021363
0.4529851869386	0.4975454948498
0.4530695676553	0.4975580420264
0.4531278748688	0.4975585711643
0.4531895674793	0.4977304148517
0.453259940623	0.4978971509876
0.4533129401268	0.4979407122333
0.4533924796916	0.4979999363277
0.4534763260259	0.4981314561769
0.4535545254798	0.4981385585927
0.4536215008659	0.4981577456489
0.4537050230743	0.4983234206083
0.4537962719295	0.4983247000181
0.4538615275564	0.4983996138384
0.4539441950103	0.4984304235683
0.4540221570125	0.4984405464043
0.4540901006251	0.4987048640858
0.4541564238931	0.4987897311374
0.4542074450553	0.49891920573
0.4542695488801	0.4990479237493
0.4543482596743	0.499108505302
0.4544202329587	0.4991992426228
0.4545007089767	0.4992108036981
0.4545563688744	0.4992593909023
0.4546277263298	0.4993083408111
0.4547008413527	0.499340488081
0.4547869079778	0.4995610836918
0.4548499021114	0.4996975211109
0.4549374580452	0.4998361887841
0.4550188651345	0.4998369021489
0.4551150449757	0.4998962932565
0.4552161573547	0.4999265858848
0.4553070102143	0.500114816294
0.4553551978576	0.5002455366335
0.4554502287668	0.500266477496
0.4555217737619	0.5002705078046

0.4556018655475	0.5005405902295
0.4556802553572	0.5005895652212
0.455765955416	0.5006732316854
0.4558494618431	0.5006971692928
0.4559522727135	0.5011216616718
0.4560450191503	0.501274944326
0.456143862827	0.5013028256551
0.4562506534017	0.5014013857063
0.4563371144894	0.5014129407283
0.4564056833283	0.5014494352439
0.4565041351065	0.5014665191463
0.4565987545193	0.5015121619884
0.4566697922866	0.5015595090374
0.4567260037244	0.5016073884218
0.4568260742602	0.5016225662081
0.4568906624056	0.5016793137378
0.4569625099005	0.501694787418
0.4570493838201	0.5018388497485
0.4571118006165	0.5019028849593
0.4571906924212	0.5019333256892
0.4572710839538	0.5019411400146
0.4573923004513	0.5020219574839
0.4574935310102	0.5026962576506
0.4575894457094	0.5027607222221
0.4576635638527	0.5027992904868
0.4577531235781	0.5032157625589
0.4578394061724	0.5032950586518
0.457928489339	0.5033471741307
0.4579954263136	0.5033655054229
0.458058942321	0.5034199830509
0.4581272715336	0.5035001124995
0.4582422215637	0.5038949585817
0.4583347613579	0.503949944151
0.4584045999348	0.5039829285804
0.4585035971343	0.5042865848016
0.4586108924404	0.5045737934559
0.4587113836995	0.5046290095899
0.4587949994726	0.5046365429282
0.4588637653912	0.5046639209379
0.4589307397156	0.5046807320706
0.4589911612488	0.5046995583325
0.4590823784402	0.5047728554097
0.4591618057847	0.504906204586
0.4592622466275	0.5049219771952
0.4593463374552	0.5049418812612
0.4594225661086	0.5051454590329
0.4594993104103	0.5051698965847
0.4595948021919	0.5051760093852
0.459702124505	0.5053367219271
0.4597750219219	0.505476727727
0.4598498449843	0.5055858840867
0.4599181700574	0.5056193531368
0.4600015767893	0.5058697034889
0.4600538935381	0.505910939839

0.4601255118685	0.5059118727797
0.4602014242975	0.5061215379922
0.4602953379859	0.5061296931604
0.4603846371604	0.5064758062953
0.4604681080353	0.5065943222336
0.4605721705272	0.5071034728417
0.4606461993065	0.5072874529055
0.4607167379509	0.5074202538561
0.4608297245156	0.5079239135735
0.460955790857	0.5079631886305
0.4610472763017	0.5080499317673
0.4611379813451	0.5084999945482
0.4612162716825	0.508631462284
0.4613213597395	0.5087114942943
0.4613694717265	0.5088334064864
0.4614298796187	0.5088690963827
0.4615066550557	0.5088917350466
0.4615701581878	0.5089385030772
0.4616321240226	0.5089787231089
0.4617139230308	0.5090389732068
0.461812094959	0.5091840784262
0.4619022223105	0.5094505439092
0.4620005436594	0.5095086689709
0.462054637606	0.5095951403343
0.4621132012304	0.5096016736922
0.4622020038108	0.5096782160455
0.4622872496006	0.5097647062817
0.4623664437484	0.5099590134208
0.4624222416482	0.5099887592493
0.462524958436	0.5102451105413
0.4626027357083	0.5103190525179
0.4626600711969	0.5103245842804
0.4627193471415	0.5103501528739
0.462808624126	0.5104659057388
0.4629019913808	0.5106024113788
0.4629894338288	0.5106732804369
0.463059551045	0.5107472218457
0.4631513828995	0.5107735361404
0.4632122290937	0.5108627343309
0.4633014669487	0.5110370454424
0.4633576803321	0.5110799959467
0.4634150932932	0.5111916132376
0.4635139248087	0.5113682844013
0.4635780640379	0.5113842016558
0.4636499964393	0.511611695121
0.4637501735847	0.5116481924636
0.4637973189142	0.5116507793367
0.4638897852446	0.5117848976647
0.4639857763676	0.5119435905007
0.464034344798	0.5119469637811
0.4641325856956	0.512179324989
0.4642236339582	0.5122689390113
0.4643102475151	0.5125078469222
0.4644303681257	0.512514068871

0.4645272196528	0.5125539469855
0.4646056118184	0.5125809694587
0.4646935506915	0.5126683681295
0.4647642571544	0.5126728134611
0.4648306409207	0.5127429226676
0.4649082885024	0.5127548026274
0.4650094622196	0.5127882443477
0.4651230239738	0.5133584971812
0.4651903679359	0.5134708418698
0.4652686375019	0.5134889667038
0.4653449870767	0.5135173521291
0.4654100441405	0.5135502107448
0.4654940558722	0.5136543637034
0.4655739522856	0.5138271841464
0.4656468067949	0.5139024147782
0.4657403353766	0.5139329008818
0.4658263835414	0.5141495063805
0.4659140741065	0.51419847676
0.4659856904268	0.5142660222343
0.4660558348956	0.5142728076476
0.4661230786963	0.5143103767253
0.4661773739815	0.5143494453728
0.4662725740566	0.514507797556
0.466357244261	0.5145698013674
0.4664630556254	0.5145720011256
0.4665415229179	0.5148140178045
0.4666571490937	0.5148835857146
0.4667114303586	0.5149150751109
0.466791475308	0.5150672868284
0.4668550116201	0.5151343441597
0.4669303830104	0.5151874201412
0.4670213860875	0.5153938555047
0.4671128561717	0.5155225970665
0.4671848841033	0.5155960659517
0.4672599796016	0.5157145145829
0.4673359532228	0.5157262197204
0.4673988102802	0.5157487668945
0.4674804204317	0.5158551595975
0.4675518694586	0.5159023723048
0.4676162555016	0.5161014965814
0.4677094063412	0.5161325412949
0.4677884059141	0.5163000499459
0.4678573081149	0.5163499729999
0.4679335878767	0.5164456114858
0.4680041774853	0.5166239001844
0.4681423608331	0.5169098745704
0.4682139081769	0.5172053679672
0.4682965486482	0.5173745690655
0.4683806240726	0.5174017597074
0.4684801436004	0.5174139979954
0.4685804184858	0.5178700726751
0.4686637983685	0.5181521744638
0.4687811834231	0.5182476278161
0.4688678136531	0.5182931289048

0.4689504639961	0.5184222202956
0.4690391698502	0.5184836797262
0.4691691984028	0.5186852301289
0.4692387478297	0.518945774452
0.469310215655	0.5190304118603
0.4693881228504	0.519080914996
0.4694618762686	0.5191195198022
0.4695344091533	0.51942878462
0.4696063636565	0.5195686415343
0.4697002532629	0.5195932411515
0.4697922212602	0.5196010866188
0.4698868467628	0.5199782144976
0.4699466103468	0.520120056267
0.4700343424118	0.5203034015134
0.47011227536	0.5203044072995
0.4701894485552	0.5203747049648
0.4702868067777	0.5204761613476
0.4703741339205	0.5208415274814
0.4704454203737	0.5208473987785
0.4705559165066	0.5210116615781
0.4706254876518	0.5210521771209
0.4707129716882	0.5212711103651
0.4707709773206	0.5213123071672
0.4708329561397	0.5213211408925
0.4708911222802	0.5213991868067
0.4709744908048	0.5214254791611
0.4710411221704	0.5215784413052
0.471132809843	0.5216066960643
0.4711970394384	0.5216476096195
0.4712728404116	0.5216622288615
0.4713874412786	0.5217956072854
0.4714555150287	0.5219177349309
0.4715103200404	0.5219608337854
0.4715791752135	0.5219618368566
0.4716683852884	0.5221183174792
0.4717326416483	0.5221432409008
0.4718311406898	0.5223290535593
0.4718833168645	0.5223394870297
0.4719467986514	0.5224119766797
0.4720232425689	0.5224612306642
0.4721226573945	0.5226292204329
0.472202856623	0.5226366543926
0.4722846396487	0.5227259213381
0.4723770730533	0.5232044831372
0.4725116375523	0.523540947933
0.4726082130236	0.5237806051602
0.4726807723572	0.5238216647916
0.47278104607	0.5238494119171
0.4728427629086	0.5238717172924
0.4729193196971	0.5239265641204
0.472983946507	0.5239985915633
0.4730503121812	0.5240854091612
0.473146472113	0.5241109505563
0.4732352856307	0.5241515262699

0.4733253859886	0.5241541437169
0.4734099832671	0.5242208705244
0.4734826525692	0.5242530007033
0.4735412219532	0.5243173349997
0.473653758474	0.5244058412934
0.4737347019973	0.5244371731583
0.473783810833	0.5245483700922
0.4738950540973	0.5246181131421
0.4739717718942	0.5246968486156
0.4740464536505	0.5247587512882
0.4741325375833	0.5251690785518
0.4742116272364	0.525206746069
0.4742692207573	0.5252815553403
0.4743686812159	0.5253890552869
0.4744560625517	0.5258076579186
0.4745511294578	0.5260075232044
0.4746278872246	0.5260273613704
0.4747103331059	0.5260779408784
0.4747909482838	0.5261610820027
0.4748806523096	0.5263654566972
0.4749665670289	0.5264096705953
0.4750489110912	0.5265267639356
0.4751270764309	0.5266415928602
0.4752140174861	0.5267414128594
0.4752869962267	0.5267435694031
0.4753925402081	0.5268950166295
0.4754678217341	0.5269164416663
0.4755326937988	0.5269832031605
0.4756089079377	0.526986456325
0.4757047132489	0.5270526541703
0.4757842339764	0.5273009043438
0.4758433060003	0.5274175020544
0.4759095693318	0.5274732682321
0.4760141057809	0.5275213896953
0.4760968086386	0.5276094962168
0.4761637787927	0.5276801647398
0.4762410272972	0.5277166734476
0.4763218846805	0.5279508149517
0.4764077393212	0.5282295978074
0.4764764282405	0.5282828851975
0.4765302772742	0.5283104311466
0.4766118477029	0.5283292603045
0.4766757804613	0.5284669387169
0.4767465599374	0.5285984612107
0.4768453095787	0.528727880553
0.4769051206583	0.5288041009265
0.4769525788203	0.5288555575819
0.4769967496451	0.5289018296252
0.477091721976	0.5291135873436
0.4771765744189	0.5293794313476
0.477246730866	0.5294931945896
0.4773421304715	0.5295055232704
0.477424601158	0.5295874906439
0.4775209007366	0.5296628038982

0.4776161697283	0.5296695502107
0.4776854778619	0.5297560605074
0.4777841838592	0.5297665110027
0.4778611845057	0.5297863146451
0.4779440978427	0.5299093474894
0.4780522468348	0.5300471422812
0.4781453924311	0.5301287442324
0.4782297395616	0.5301790309613
0.4783023706724	0.5302173383277
0.478367418847	0.5302877572389
0.4784599283565	0.5304503997738
0.4785487491858	0.5304949495569
0.4786423107111	0.5305990062805
0.4787362767017	0.5306450176378
0.4787961011244	0.5306675638021
0.4788970950548	0.5307991411224
0.478972688911	0.5309972956733
0.479032845984	0.5310286007569
0.4791164838722	0.5311752966367
0.4791925857775	0.5312592295746
0.4792454588222	0.5312864032301
0.4793292339674	0.5314009970784
0.4794094518396	0.5314189050117
0.4794944845424	0.5315170848862
0.4795366711531	0.5315212967029
0.4796290991	0.5315883845843
0.4797097496342	0.5319589896142
0.479777785227	0.5321108905241
0.4798536364898	0.5322217520101
0.4799308113819	0.5322225452736
0.4800608550757	0.5322498764481
0.4801431833058	0.5323038436911
0.480207370551	0.532415377987
0.4802784517044	0.5326358541417
0.4803746453887	0.5327861662838
0.4804616697797	0.5330337858741
0.4805368915863	0.5331038913014
0.4805945206602	0.5331395302457
0.480670438991	0.5331543100429
0.4807657020736	0.5331888882138
0.4808600133214	0.5333213307474
0.4809513348887	0.5333457445873
0.481038828168	0.5336216643866
0.4811043815695	0.5336710008259
0.4811801136151	0.5336747736794
0.4812526246601	0.5336874690449
0.4813535967062	0.53373237274
0.4814440861888	0.5337768299038
0.4815293663788	0.5338588646488
0.4816243389369	0.5341621012218
0.4817062769138	0.5341745428167
0.4817658901055	0.5342409787806
0.4818635647047	0.5342615097562
0.4819273289896	0.5343040159902

0.4819982048188	0.5343618010128
0.4820762189358	0.53444060145
0.4821410030261	0.5345325495482
0.4822401754374	0.5346333892661
0.4823341314496	0.5346957639924
0.4824104738343	0.534699574493
0.4825003715285	0.5349595143029
0.4825798984704	0.5350021749938
0.4826525005755	0.5351524636073
0.4827176884953	0.5353273253587
0.4827881809996	0.5353937928804
0.4828515314121	0.5354262041722
0.4829398109639	0.5354270314019
0.4830047794657	0.535452679311
0.4830759936068	0.5356623655398
0.4831536863926	0.5358766294319
0.4832436561666	0.5359545107336
0.4833297389401	0.5360953946682
0.4834274285586	0.5361063600716
0.4834866343139	0.5361713436314
0.4835745968996	0.5362279283364
0.4836474044693	0.5362406087351
0.483723932808	0.5364962469984
0.4838128422454	0.5366013464075
0.4839115069496	0.5366946617015
0.48399366313	0.5369369671834
0.4840578548834	0.5369706013244
0.4841244164468	0.5371981088801
0.4841974696904	0.5374550698324
0.4842661912192	0.5375352464844
0.4843347568355	0.5375623403444
0.4843944617701	0.5376417760555
0.4844652102565	0.5376754124975
0.4845266345039	0.5376988513018
0.4846203702694	0.5378791382598
0.4846980434858	0.5382142650835
0.4848219712178	0.538251111198
0.4849170814659	0.5383221804474
0.484977060537	0.5383941694865
0.4850593158673	0.5386001134091
0.4851354601318	0.5386385082958
0.4852162539284	0.5386884900688
0.4852894088108	0.5387522912211
0.48536380797	0.5387710422458
0.4854331258002	0.5387776387104
0.4855093282362	0.5387919519003
0.4856095893334	0.539065186181
0.4856761829027	0.5390729363996
0.4857427490723	0.5390788216847
0.4858335676015	0.5393014385366
0.4859171747077	0.5395195298721
0.4859817344547	0.5395774639265
0.4860492787965	0.5397609066033
0.4861385777197	0.5397609621578

0.4862000750165	0.539785880338
0.4863146999959	0.5398627561839
0.4863645042745	0.5398750307074
0.4864416904017	0.5398781389587
0.4865222741126	0.5399180536646
0.4866023819426	0.5399502059604
0.4866736972217	0.540069094144
0.4867592772707	0.5404982671374
0.4868170698302	0.5405045460196
0.4869057519886	0.5406342046035
0.4869564853717	0.5407267809032
0.4870554843072	0.5408109116086
0.4871408054204	0.5408137884932
0.4872313945255	0.5409484457451
0.4873126237217	0.5411092414888
0.4873654503553	0.5411145958929
0.4874223062419	0.5411536154125
0.4875086975717	0.5412802219177
0.4876107887556	0.5413748644341
0.4876605639967	0.5413947891612
0.4877308931727	0.5414393987412
0.4878107313233	0.5414724179963
0.4878848759252	0.5415818345399
0.488004249433	0.5417384239771
0.4880566042669	0.5417429258427
0.4881127972816	0.5418283142045
0.488190390451	0.5420440592579
0.4882999737331	0.5420741422963
0.4884118731677	0.5422452970847
0.4884781097213	0.5422658468568
0.4885474509284	0.5423126471969
0.4886345342966	0.5424054430806
0.4887021372813	0.5425598786064
0.4888034554829	0.5426362277215
0.4888948156537	0.542859773479
0.4889872261794	0.5429318061506
0.4890658371614	0.5429750493458
0.4891361978966	0.5429987953912
0.4892287622358	0.5431641015358
0.4893033155038	0.5432266893989
0.4893793462827	0.5433093436305
0.4894361902655	0.5433605127357
0.4895212727518	0.5435818884823
0.4896057161979	0.5436702969084
0.4896762292383	0.5437000329238
0.4897637688914	0.5438593067404
0.4898558838482	0.543870412368
0.4899486423989	0.5440254728457
0.4900301698111	0.544162818361
0.4901198177678	0.5442850684717
0.4902060487292	0.5443277877303
0.4902958839233	0.5444266194136
0.4903744140574	0.5444956867821
0.4904603066177	0.5446373069979

0.4905438368423	0.5447466311765
0.4906058433447	0.544962833281
0.4906901710547	0.5450742819551
0.4907523115566	0.5451232411115
0.4908371279296	0.5451421463885
0.4909515209258	0.5455374097107
0.491013759284	0.5455913190931
0.4911011746402	0.5456275180816
0.4911805929199	0.5456870011686
0.4912601134622	0.5458013271079
0.4913399569717	0.5458331516522
0.4913944315612	0.5458730552144
0.4915289061571	0.5459800128025
0.4916038848898	0.5460239659719
0.4916698077631	0.5461381812371
0.4917494398587	0.5462422088282
0.4918482962998	0.5462726392277
0.4919357970375	0.5462800933701
0.4920007605026	0.5462924823256
0.4920742416939	0.5463507918396
0.4922101269411	0.5470015725097
0.4923310814829	0.5471446879347
0.4924182752983	0.5472463094714
0.4925070445152	0.5472639454913
0.492575109525	0.5473121588731
0.4926521989253	0.5473144668801
0.4927238675059	0.5475159355708
0.4928100069275	0.5477105996908
0.4928545533163	0.5477567490807
0.4929404056844	0.5479728364357
0.4930260414863	0.5479786614389
0.4930969773321	0.548151698908
0.4932058566126	0.5482128349173
0.4933001618971	0.5483114340961
0.4933681406754	0.5483981391738
0.4934544373144	0.5485528957519
0.4935315519294	0.5485681605021
0.4936330129237	0.5488687328958
0.4937350491528	0.5491567479704
0.4938370373369	0.5492354964565
0.4939279625624	0.5493679434594
0.494015688411	0.5494038896464
0.4940888244258	0.5494567288528
0.4941664707711	0.549581185666
0.4942328562843	0.5496222956278
0.4943396546642	0.5496383894187
0.4944123238624	0.5496936340636
0.4944814403634	0.5497226573985
0.4945444203066	0.5498192310028
0.4946290871612	0.5498824854503
0.4947094448562	0.5498931291162
0.4947852462935	0.5499074798266
0.4948738805586	0.549911821462
0.4949838415487	0.5499298640273

0.4950523479662	0.5500319184409
0.4951259204516	0.5500922584783
0.4951792210985	0.5501541093773
0.4952691459578	0.5502630022153
0.4953427073504	0.5503426219567
0.4954362332851	0.5504218845685
0.4955093786343	0.5504381364588
0.4955846725809	0.5506831937414
0.4956833295285	0.5507473213747
0.4957751038694	0.5507957658928
0.495852758961	0.5508460915429
0.4959604924555	0.5510380838659
0.4960486743048	0.5510811976506
0.4961184312192	0.5511971332859
0.4962310176985	0.5514525145536
0.4962890069567	0.5514993450414
0.4963597721565	0.5515063152507
0.4964064273559	0.551567531912
0.4964761689023	0.5517310564051
0.496558438597	0.5517463856777
0.4966291756852	0.5517801137558
0.4967219057746	0.5520071438157
0.4967932627278	0.552020747645
0.4969184240033	0.5522758658074
0.496975684859	0.5523923192633
0.4971091196428	0.5524593964491
0.4972000495568	0.5525038149733
0.497283399397	0.5525706040291
0.4973604082413	0.5526125381762
0.4974015227981	0.5527142264659
0.497479244098	0.5527767986314
0.4975625586786	0.5527817507065
0.4976488356284	0.5529254825371
0.4977504203045	0.5532265791198
0.4978265739185	0.553271864991
0.4979080660451	0.5534341878587
0.4979718335519	0.553455743333
0.4980530037552	0.5536547815621
0.4981781600058	0.5538334365702
0.4982528626791	0.553841893056
0.4983944136659	0.554040812154
0.4984567925342	0.5541476885092
0.4985501961593	0.5542392153712
0.4986250527738	0.5543789102241
0.4986988251765	0.5544476108792
0.4987725624463	0.5544799283416
0.4988598975108	0.554612004361
0.4989534236256	0.5549483540481
0.4990352220267	0.5551488366893
0.4991191599206	0.5553740666465
0.4992064950961	0.5553916883081
0.4992691568399	0.5554249503108
0.4993637582846	0.5554867142262
0.4994368805544	0.5555218608924

0.4995064499541	0.5555524819712
0.4995913741803	0.5558149734873
0.4996570312884	0.5558331274439
0.4997290283309	0.5559780477117
0.4998020848377	0.5562753348904
0.4998690352301	0.5565032194497
0.4999458020675	0.5565637832379
0.5000195328469	0.5566122808385
0.5001250854347	0.5566341091878
0.5002193962663	0.5566885427953
0.5003221256244	0.5567955570333
0.5004088717673	0.5568150281963
0.5004946157724	0.5569095161677
0.5005675337709	0.5569173093434
0.5006536693136	0.5570241418362
0.5007542127236	0.5573097329569
0.5008325014573	0.5574190434362
0.5009139398771	0.5574262638553
0.5009908867973	0.5574968232499
0.5010729231963	0.5575538241737
0.5011678056955	0.5577943169097
0.5012813501168	0.5584559135329
0.5013508719075	0.5584871258143
0.5014258903174	0.5584930826917
0.5015236849298	0.5585472473448
0.501600273316	0.5586059405398
0.5017157988299	0.5588554916325
0.5017923723874	0.5589495084393
0.5019168706349	0.5592959403545
0.5019955499249	0.5594139593626
0.5020516309763	0.5594520665887
0.5021341039817	0.5596706597317
0.5022448738497	0.5597719137363
0.5023296608782	0.5599276183874
0.5024209542497	0.5602923297688
0.5024912996853	0.560333334455
0.502544559621	0.5603885510185
0.5026256124021	0.5604600910847
0.5026920432727	0.5606086015827
0.5027970911051	0.5608269714474
0.502899125758	0.560894884466
0.502980432164	0.5609119848685
0.5030526553513	0.56100924824
0.5031334837959	0.5610119587032
0.503214970344	0.5610300827156
0.5033093484694	0.5611855488799
0.5034295975249	0.5612089864198
0.5035139510774	0.5615644388853
0.5035966847957	0.5615834310038
0.5036831682577	0.5616007994126
0.5037524581206	0.5616039897072
0.5038679664184	0.5617200986438
0.5039699805694	0.561921414844
0.5040580124608	0.5620581980339

0.5041400370258	0.5621354340068
0.504219225574	0.5622634085439
0.5042969907442	0.5622793153202
0.5043994605324	0.5623553608782
0.5044823947332	0.5623722790901
0.50455869587	0.5625568969502
0.5046287194041	0.5626780056954
0.504696642895	0.5627283792695
0.5048124067338	0.5630452642721
0.504885080499	0.5630641834815
0.5049651208893	0.5632058765832
0.5050460163806	0.5632073071389
0.5051233332705	0.5634417583696
0.5051765650036	0.5634520608153
0.5052637246491	0.5634678552726
0.5053372347615	0.5635977218254
0.5054197062288	0.5637377828655
0.5055042304404	0.5638076693543
0.5055586796632	0.5640984876816
0.5056313156982	0.5641745904372
0.5057029786863	0.5642016308578
0.5057964867063	0.5642510064611
0.5058749515128	0.564368032573
0.5059350043348	0.5644813973661
0.5060001904283	0.5644843109574
0.506071678821	0.5645412737731
0.5061559250766	0.5645820033084
0.5062507178093	0.5647534107382
0.5063371624297	0.5647843801136
0.5064065105248	0.5647858879764
0.5064919812147	0.564869574915
0.5065570885204	0.565014328551
0.5066295142913	0.565095473387
0.5067473409893	0.5651681574167
0.5068227461907	0.5652180942622
0.5068789862403	0.5653459112022
0.5069723177621	0.5654789265332
0.507049556369	0.565479220127
0.5071568407255	0.5656227126747
0.5072336044513	0.5658347004171
0.5073140497069	0.5658476479944
0.5073843722894	0.5659913919558
0.507431750782	0.5660126147257
0.5075014017268	0.5660956627734
0.5075835257293	0.5661943971282
0.5077142065177	0.5661983350069
0.5077886199799	0.5662689193358
0.5078850235083	0.5665706719893
0.5079589770898	0.566667372863
0.5080308157129	0.5666690774321
0.5081337906008	0.5667465107808
0.5082191564697	0.5669133696058
0.5082961128006	0.5670273820316
0.5083473992323	0.5670807490586

0.508433456556	0.5671082580869
0.5085030532295	0.5671680272595
0.508593653663	0.5673300732395
0.5086746218976	0.5673733041277
0.5087617010358	0.5674823263759
0.5088366207777	0.5675757447911
0.5089084694753	0.5676970003779
0.5089707351976	0.5678102032075
0.5090677047498	0.567819218192
0.5091154136765	0.5680070914222
0.5092022150305	0.5680990967473
0.5092841673847	0.5681161798336
0.5094012138655	0.5681621457422
0.5094670426509	0.5683396643291
0.5095353482357	0.5685089707515
0.5096229810925	0.568533272014
0.5097025680951	0.5685943588774
0.5097770500335	0.5687928348187
0.5098761665917	0.5688437215508
0.5099493472941	0.5689733784968
0.5100388675192	0.5689847722078
0.5101276109241	0.5689953125589
0.5102226635961	0.569387782361
0.5103083320706	0.5695831378919
0.5103798528781	0.5695877052641
0.5104506341043	0.569649775377
0.510521339319	0.5697917695389
0.5105770839717	0.5697934951118
0.5106592828681	0.5699073141942
0.5107497925627	0.5699355845197
0.5108053111197	0.5699436526545
0.5108902590641	0.5702763311757
0.5109889600508	0.5703281794141
0.5110571683625	0.5704285510281
0.5111312560714	0.5704438659397
0.511221790642	0.5707933900494
0.5113230410146	0.5712361498902
0.5114063987836	0.5712588644188
0.511493857192	0.5713123614646
0.5115840808214	0.5713399125847
0.511688792074	0.5714768400293
0.5117542459686	0.5715040203713
0.5118510313623	0.571518919563
0.5119545388947	0.5715314349091
0.5120658864343	0.571759892716
0.512130689618	0.5717880824058
0.5121967611482	0.5720088292349
0.5122857697907	0.5720627166142
0.512369569	0.5721578855017
0.5124650455365	0.5722166883414
0.5125649855873	0.5724717618478
0.5126675433365	0.5724837413525
0.5127786469043	0.5725623733098
0.5128443186051	0.5726900859574

0.5129207881529	0.5726991853825
0.5129787509861	0.5727639040552
0.5130540419181	0.5727892586686
0.5131394079444	0.5728446356267
0.5132094638922	0.5728991116328
0.5132746772155	0.5729921336354
0.5133425835627	0.5731194425773
0.5134112647065	0.573176880426
0.513479186036	0.5734807865064
0.5135776445368	0.5735743519086
0.5136669599671	0.5737338674223
0.5137705062404	0.5739040761367
0.5138599069982	0.5739761360254
0.5139359914335	0.574195875134
0.5139921255467	0.5742967431592
0.5140649286276	0.5743428272169
0.5141585975538	0.5743479777289
0.5142355142649	0.5743507091761
0.5143012375804	0.5744787552414
0.5143888065799	0.5745831044872
0.5144536109451	0.5745915172351
0.5145530992081	0.5746808204267
0.5146765456311	0.57490484359
0.5147599706635	0.5749123609999
0.5148325671874	0.57504112171
0.5149348811851	0.5753711725492
0.5150128527289	0.5754203253869
0.5150816517366	0.575437348826
0.5151406293018	0.5754491210034
0.5152538756273	0.575896089223
0.5153527453673	0.5762075243025
0.515449479022	0.5762379734178
0.5155624689792	0.5765963176918
0.5156568142375	0.5766394470106
0.5157244122243	0.5767544281093
0.5158255614964	0.5768392808745
0.5159017602217	0.5769515022
0.5159603114174	0.5769596430165
0.5160145817902	0.576984437651
0.5161117217593	0.5770049295585
0.5161888851992	0.5770284630787
0.5163251681396	0.5772189605746
0.5164116502013	0.5773164494321
0.5164772847376	0.5775680399852
0.516568239767	0.5775906276495
0.5166427800844	0.5776025686656
0.5167294648236	0.5777262257378
0.5168143445438	0.5780558288862
0.5168844252432	0.578120966733
0.5169594805459	0.5781995760272
0.5170381878	0.5782028439758
0.5171044287806	0.5782105715553
0.5172012667316	0.5783224236886
0.5172715884933	0.5783366800265

0.5173748713299	0.5785146568867
0.5174362628717	0.5785352312863
0.5175207674854	0.5786532530838
0.5175831156739	0.5787585772419
0.5176683957902	0.5787925939219
0.5177423710514	0.5789106370145
0.517826169878	0.5789170702817
0.5179214002161	0.5790413293409
0.5179861401127	0.5790697981881
0.5180972879676	0.5790757103889
0.5181954325374	0.5790807941382
0.5183259238139	0.5793780749928
0.5183981273435	0.5793884830843
0.5184513566465	0.5794306543146
0.5185663222732	0.5796388633949
0.5186616778635	0.5796632980459
0.5187526534439	0.5796663251046
0.5188760017675	0.5796738714619
0.5189529082494	0.5797517312605
0.519036480366	0.5800727551519
0.5191251351859	0.5801868457936
0.5192150873054	0.5801914381233
0.5192787012605	0.5802225601897
0.5193652726883	0.5802290091888
0.5194685476996	0.5802808191607
0.5195460219401	0.5805490767039
0.519625377755	0.5806226634024
0.5197288396151	0.5806408269833
0.5197961464186	0.5807005164271
0.5198694390484	0.5808462862063
0.5199679723847	0.5808530365558
0.5200346582193	0.5808871295744
0.5201287956372	0.5811325733331
0.5202049631727	0.5813766654597
0.5202764426906	0.5815383361392
0.5203348524586	0.5815792816785
0.5204031807456	0.5816216174374
0.5204896672615	0.5817921129925
0.520573014223	0.5818367564769
0.5206442576748	0.5818535320182
0.5207178722518	0.581878806354
0.5208125616582	0.5819626593233
0.5209037578052	0.5823163276093
0.5209577673534	0.5823679281418
0.5210283391391	0.5824384684513
0.5210987569063	0.5825207988149
0.5211891010828	0.5825704425598
0.521281119553	0.5827577665488
0.5213668761362	0.5828371056985
0.5214578607173	0.5834962310151
0.5215164760111	0.5834981132364
0.5215722218369	0.5835809758405
0.5216309954879	0.5836362391325
0.5217041713521	0.5836425439725

0.5217780169447	0.5836715811565
0.5218603044238	0.5839042683535
0.5219166010193	0.583910409592
0.5219851632918	0.5839663960399
0.5220576272835	0.5840023722444
0.5221459366871	0.5840347659236
0.5222173394077	0.5840352466327
0.5223349668777	0.5842279862106
0.5224345464752	0.5842798241316
0.5225153167292	0.5843427361601
0.5225948022689	0.5844058493637
0.5226733866614	0.5844315555985
0.5227407931557	0.5846965527974
0.5228013462754	0.5847178318011
0.5228850200567	0.5848094704448
0.5229409657099	0.5848474406237
0.5230012315062	0.5849206025265
0.5230698415471	0.5849815627645
0.5231568464342	0.5850709207247
0.523257765729	0.5852459527739
0.5233128590519	0.5852609680379
0.5234111567422	0.5852772044767
0.5235022248046	0.5854653749749
0.5235787071157	0.5854849664811
0.523650223486	0.5854943589126
0.5237149980533	0.5856426579787
0.5238276949103	0.585667060205
0.5238810804804	0.5858422529271
0.5239553827713	0.585896352048
0.5240088568335	0.5859886384688
0.5241021301861	0.5863989239641
0.5241992807064	0.5864796816395
0.5242568437915	0.5864856136472
0.5243094508455	0.5865111306313
0.5243978467051	0.5865181440835
0.5245025122359	0.5866827059217
0.5245938773282	0.5866981426054
0.5246704128439	0.5868651173802
0.524781045624	0.58703268064
0.5248441902495	0.5870955802821
0.5249127632192	0.5871488777952
0.5249923523632	0.5871918076259
0.5250790109947	0.5872221121026
0.5251481777318	0.5873625802216
0.525210198037	0.5874190673982
0.5252714228411	0.5874421636189
0.5253439768248	0.587682619003
0.52540995023	0.5877340873527
0.5255113693995	0.5879791975924
0.5256093325305	0.5881281017522
0.5256844127635	0.588134974412
0.5257392325502	0.5881749183249
0.5258081139301	0.5881866142908
0.5259137648899	0.5884574163671

0.5259728054921	0.5885057770603
0.5260467861588	0.5885298057075
0.5261414684569	0.5886681807542
0.5262288541424	0.5886875310627
0.5263118595092	0.5887108940723
0.5263800459023	0.5887346611515
0.5264670949764	0.588840511794
0.5265542726212	0.5889615249847
0.5266218884601	0.5889722274817
0.526688132263	0.5890866538276
0.526791260375	0.589192985226
0.5268728842984	0.5892141252264
0.5269778589128	0.5893928439759
0.5270370538355	0.5893951858603
0.5271059916837	0.5894286893031
0.5271681023431	0.5896995884366
0.5272537231981	0.5898661732968
0.527351969157	0.5899024159915
0.5274741727661	0.5903756978163
0.5275413822502	0.5903971611162
0.5276139240121	0.5904443017566
0.5276839995598	0.5906004169409
0.5277745274698	0.5906504521251
0.5278572317394	0.590681168015
0.5279366630363	0.590723208156
0.5280005586572	0.5907694564392
0.5280745747246	0.5909250732101
0.5281484771916	0.5909860008715
0.5282486057411	0.5911896567752
0.5283202796215	0.5912141969841
0.5283743786192	0.5912465372332
0.5284516122193	0.5913955364568
0.5285522036431	0.5914088931456
0.5286157653354	0.5914458015446
0.5286784653548	0.5914880324168
0.5287519629861	0.5915181514551
0.5288205439701	0.5915702608662
0.5289444804779	0.5918516048408
0.5290157337702	0.5918641176719
0.5290981648694	0.5919053414854
0.5291999952443	0.5920314713233
0.5292701464955	0.5921824548756
0.5293407473786	0.5923054282918
0.5294491599838	0.5925450319187
0.5295373761612	0.5926693982011
0.5296188656071	0.5926779215047
0.5297220405053	0.5926979849007
0.5298033066366	0.5928392362406
0.5298802429931	0.5929386179399
0.5300031505052	0.5930279874888
0.5300819462871	0.5931017029495
0.5301780623159	0.5933704663818
0.5302701755612	0.5933800245524
0.5303401077294	0.5934181961755

0.5304039367565	0.593536227045
0.5304676344509	0.5935486999763
0.5305752459127	0.5939080349352
0.5306590882724	0.5939528111312
0.5307821089672	0.5940011672809
0.5308637383914	0.5940326865831
0.5309385892658	0.5941483690154
0.5310202389605	0.594170515377
0.5311431836719	0.5943812786099
0.5312008745424	0.5944417693312
0.5312690747929	0.5945639407083
0.5313430329263	0.594642257276
0.5314223932058	0.5947385594559
0.5314866206185	0.5947643368149
0.5315610229447	0.595077841391
0.5316483665987	0.5951579872538
0.5317358753624	0.5951665412222
0.5317968954165	0.5952206416804
0.5318880604813	0.5952385130937
0.5319767402689	0.5953070207062
0.5320553128301	0.5953568448553
0.5321155694915	0.595364856659
0.5321925864041	0.5954252786119
0.5322490457939	0.5954321551507
0.5323364986761	0.5954513329805
0.5324450455956	0.5955379168102
0.5325179277641	0.595570905007
0.5325816409785	0.5957007042786
0.5326510909795	0.5958193035798
0.5327428760417	0.5959315003077
0.5328101030095	0.595964776018
0.5328812432006	0.596091539172
0.5329752599536	0.5961492427457
0.5330636944188	0.5961903008253
0.5331572750922	0.5962234097157
0.5332304852971	0.5963170124567
0.5332979050554	0.5964021461917
0.5333860467085	0.5964701075336
0.5334492494072	0.5966783460329
0.533545461923	0.5967826408056
0.5336198773628	0.5969675160526
0.5336943008701	0.5970454589151
0.5337651144223	0.5970909299079
0.5338415709053	0.5971398501061
0.5339223378824	0.5972705273735
0.5340097861081	0.5973522761639
0.5341025714578	0.5973809503016
0.5341922677709	0.5975993457228
0.5342493583011	0.5976418733412
0.5343295314181	0.5977593155707
0.5344023088361	0.5977719147126
0.534461853276	0.597775894436
0.5345623412765	0.5978646299757
0.5346747689189	0.5978761058716

0.5347429271319	0.5979701500187
0.5348241147885	0.5979936071207
0.5349014449342	0.5980673407519
0.5349680112038	0.5980692832193
0.5350453237596	0.5980694230124
0.5351350478608	0.5981139330143
0.5351984761543	0.5981441220329
0.5352801124517	0.5983537635975
0.535407883721	0.5987868815785
0.5355000606086	0.5988110029295
0.5355473093288	0.5988152757382
0.5356045261531	0.5988365204623
0.5357041789864	0.5990503982898
0.5357778281498	0.5991384107515
0.5358334110311	0.5991836763755
0.5359160862549	0.5992526308384
0.5359597266671	0.5992627285934
0.5360354747871	0.5993306302842
0.5361138618042	0.5994250946772
0.536208550492	0.5995755955191
0.5362664587341	0.5996141848561
0.5363294373439	0.5997016447124
0.5364587220853	0.5999547886534
0.5365481333127	0.5999627150758
0.5366221024787	0.6000044560648
0.5367376955861	0.600172793615
0.5368080450638	0.6002453556024
0.5368962608884	0.6003631259439
0.536964519219	0.6004311427982
0.5370279950684	0.6005204806089
0.5371063360514	0.6005441271719
0.5371915497651	0.6007546811358
0.5372816825937	0.6007650669704
0.5373478647695	0.6008449824662
0.5374364630226	0.6011571963825
0.5375412897341	0.6013837614714
0.5376283127937	0.6014152967421
0.5377170280038	0.6014233434767
0.5377942685223	0.6014494352594
0.5378916527245	0.6014848423376
0.5379644047509	0.6015416563467
0.5380280005423	0.6015613214932
0.5381157132658	0.6015998450417
0.5381874763171	0.6018186148026
0.5382637938142	0.6019156995634
0.5383366963262	0.6019304395435
0.5384161664076	0.6019341957104
0.5385004361267	0.6020603470685
0.5385747444866	0.6021202201728
0.5386430939868	0.6021821962963
0.5387041644166	0.6022200018768
0.5387888001857	0.6022808100245
0.5388929336388	0.6028054023533
0.5389679506084	0.6028413613974

0.5390463300847	0.6030680637328
0.5391144464254	0.6032251633485
0.5391845264864	0.6035101414132
0.5392635247409	0.6035337063787
0.5393447631973	0.6035472125569
0.5394256736517	0.6036109206495
0.5395070249992	0.6036125880845
0.5395901855942	0.6037048837392
0.5396800116719	0.6037059471298
0.5397514402271	0.6037409797356
0.5398051724102	0.6037539051136
0.5398536157483	0.6037637441242
0.5399394418543	0.6037902556637
0.540018598287	0.6039496136153
0.5401324169865	0.6041076957419
0.5402169919123	0.6042439725423
0.5402866451681	0.6042692916102
0.5403899453171	0.6043290240917
0.5404463824158	0.6043947366036
0.5405187225636	0.6044989240816
0.5405948536678	0.6047362821067
0.5406533440697	0.6047892550333
0.5407541657842	0.6048662526265
0.5408461379402	0.6049777900125
0.5409181312617	0.6049956081199
0.5409827173686	0.6050607053567
0.5410557367831	0.6051262195482
0.5411348076167	0.6052178700956
0.5412060197743	0.6052919654141
0.541275913539	0.6054545180603
0.541353113911	0.6054712650979
0.5414168377171	0.6055179479975
0.5415177019492	0.6056246472304
0.541559150437	0.605637848744
0.5416199947543	0.6058003546241
0.5416830675789	0.6058031613438
0.5417383232428	0.6059322303185
0.5417857954558	0.6060468118017
0.5418526335804	0.606099606006
0.5419614118607	0.6063549268972
0.5420212000647	0.6063929930164
0.5420990500885	0.606468978661
0.5421777727617	0.6064979320648
0.5422881826299	0.6070047811897
0.5423662245747	0.6071453578032
0.542462220843	0.6071563533284
0.5425571726648	0.6073169233732
0.5426249308	0.6073917917213
0.5426964221688	0.607490647066
0.5427713613512	0.6075180697929
0.5428445622312	0.6075921013728
0.5429191521364	0.6076219128114
0.5429963095064	0.6076335894779
0.5430785797728	0.607796988079

0.5431522791945	0.6079671885518
0.5432457101451	0.6081524392226
0.5433534040267	0.6082376575327
0.5434174330207	0.6082521057869
0.5435030038198	0.6083059429246
0.5435931729357	0.608567287214
0.5436509188117	0.6086137480612
0.5437387014861	0.6086452317871
0.5438417606242	0.6086626009183
0.5439301649078	0.6090223754573
0.5440090417372	0.6090286960753
0.5440794975318	0.6090451224595
0.5441653884859	0.6091659868466
0.5442395680961	0.6091753180458
0.5443416140607	0.609272640039
0.544424805528	0.609358962363
0.5445132251248	0.609386346297
0.5445682022083	0.6094065805524
0.5446732619073	0.6096090640095
0.5447468372892	0.6096413210311
0.5448480902846	0.6097934361253
0.5449119528131	0.6098662862189
0.5449956310078	0.6099035482022
0.5450833122661	0.6100828173221
0.5451574597561	0.6101028721851
0.5452167180163	0.610224635324
0.545285600199	0.610267749553
0.5453529299989	0.6103269638687
0.5454257373467	0.6104502195186
0.5455122239588	0.6104715250419
0.5455625636884	0.6104719440392
0.5456399885592	0.6107397033396
0.5457129379829	0.6107977578746
0.545805719473	0.6109113711841
0.5459036750918	0.6112281346589
0.5459781765127	0.6113690915687
0.5460651639436	0.6114875345835
0.5461500370795	0.611549516642
0.5462208977529	0.6115729542922
0.5463227581404	0.6117220129548
0.5464114337897	0.6119215947077
0.5464791002353	0.6121068869198
0.5465482945054	0.6121194034775
0.5466298403679	0.6121470126716
0.5466999283387	0.6122133658735
0.5467803873532	0.612275092876
0.5468648462442	0.6124133937512
0.5469584962547	0.6125537397806
0.547037969984	0.6127963555848
0.5471249319038	0.6128517597047
0.5471858185489	0.6128986514883
0.5472488431767	0.612985473016
0.5473513278945	0.6130952531736
0.5474369844434	0.6132840854162

0.5475458430444	0.6135737195297
0.5476173513025	0.6135980955368
0.5476930559762	0.6136053857684
0.5477610111962	0.613639020898
0.5478399021426	0.6136571101094
0.547917297397	0.6136747935386
0.5479888648106	0.6136992402914
0.5480612747549	0.6137244614599
0.5481291773814	0.6137847592203
0.5482026085879	0.613804072904
0.5482794971824	0.6138865764747
0.5483655669191	0.6141617554198
0.5484262076813	0.6142654973256
0.548500375924	0.6143575237891
0.5485985865119	0.6144979092312
0.548688533231	0.6147432543762
0.5487602500683	0.614855371889
0.5488334172616	0.6149000093888
0.5489003960632	0.6149173480769
0.5489654925039	0.6149717230557
0.5490745606532	0.6150016263608
0.5491334352268	0.6150658062579
0.549210720586	0.6151324873363
0.54929551252	0.6151394331463
0.5493729103331	0.6151456581634
0.5494678005356	0.6151789596113
0.5495503409282	0.6153995148712
0.5496257829888	0.6154478556502
0.5497209626852	0.6155478646464
0.5497981082642	0.6157064340718
0.5498735108473	0.6158860812875
0.549943035385	0.6159677668702
0.5500479091437	0.6160009666084
0.5501405620204	0.6160786908555
0.5502173624372	0.6160840338451
0.5503026646463	0.6161156421682
0.550403731268	0.6164026202343
0.5505081631945	0.6164376624461
0.5505897981341	0.6164419086161
0.5506766494165	0.6165509453888
0.5507464415376	0.6166289907805
0.5508076186112	0.616716631299
0.550885531733	0.616719122776
0.5509537349603	0.6168188436786
0.5510299028908	0.6169743071296
0.5511320632682	0.6171564730812
0.5512118643275	0.6172151467767
0.5513074147551	0.617221803601
0.5513932837454	0.6175941422419
0.5514462724817	0.6176061149439
0.5515267127263	0.6176605013368
0.5515912009289	0.6177054319048
0.5516727843184	0.6179318531689
0.5517714348258	0.6179480669848

0.5518290849463	0.6180238296197
0.5519166624654	0.6180483289943
0.5519927627241	0.6181930759416
0.5520790517232	0.6184234418656
0.5521516988022	0.6184559526397
0.552225980981	0.6185447735447
0.5522894165808	0.6186824197576
0.5523474922088	0.6187192686405
0.5524338329263	0.6190871420781
0.5525262641316	0.6190911302123
0.552603397089	0.6190936620985
0.5527007252026	0.6191088211503
0.5527871569321	0.6192508053133
0.5528600670329	0.6192711683816
0.5529302134976	0.6192743179735
0.5530055860779	0.6194482438453
0.5530986189778	0.6195529511871
0.5531743419559	0.619660634694
0.5532640374728	0.6199513271968
0.5533445307989	0.6200149853899
0.5534158888222	0.6200229339009
0.5534852199589	0.6201155379259
0.5535683580163	0.6203014394967
0.5536296371797	0.620334775518
0.5536849100113	0.6204450793494
0.5537719190426	0.6204706982096
0.5538526470674	0.6205373387526
0.5539365192365	0.6205616542831
0.5540343537101	0.6206285639628
0.5541129453377	0.6206312827187
0.5542197164629	0.6207277352221
0.554309904252	0.6207615833259
0.554405422417	0.6208114338185
0.5544802292171	0.620836037258
0.5545375731816	0.6208926707089
0.5546240006828	0.6209286352059
0.5547370634031	0.6210261738151
0.5548014541049	0.6210969814214
0.5549030052341	0.6212803805756
0.5549928283686	0.6213692520138
0.5550555827268	0.6213736015299
0.5551452163176	0.6214101726797
0.5552499742155	0.6215647328637
0.5553300788419	0.6216275751394
0.5554062896163	0.621709463639
0.5554678229276	0.6217250429807
0.55557539811	0.6218421319231
0.5556536357974	0.6219819826547
0.5557519439673	0.622264831973
0.5558294120772	0.6223235663275
0.5559006428963	0.622488710407
0.5559754537881	0.6224983356375
0.5560244847241	0.6225258109485
0.5561085490804	0.6226278415165

0.5561875660431	0.6226665128892
0.5562528224895	0.6227936713858
0.5563130934953	0.6229740979937
0.5563864079773	0.6230084316595
0.5564756562585	0.6232421083658
0.5565679826262	0.6233225823303
0.556622928336	0.6233352494643
0.5566897491202	0.6233840863649
0.5568051308101	0.6239336789398
0.5568692887674	0.6239408165654
0.5569436750522	0.6239694645205
0.5570221980848	0.6240137473631
0.557092319427	0.6241945026984
0.557150408544	0.6242343445922
0.5572299294022	0.6244405097703
0.5573272230056	0.6246046120021
0.5573926050262	0.624787823996
0.5574577210873	0.6248259501306
0.5575312576714	0.6248355017013
0.557617662481	0.6248357431307
0.5576910959028	0.6250847587482
0.5577854892122	0.6251458961353
0.5578829071259	0.6254453112025
0.5579665974594	0.6255170212801
0.5580455181959	0.6256237558082
0.5581103159715	0.625706227647
0.5582128662871	0.6259181111947
0.5582741504658	0.626127625869
0.5583332986942	0.6262089520733
0.5584594927778	0.6265832928792
0.5585619531186	0.6268161322456
0.5586189788948	0.6268255989673
0.5587018544445	0.6269079171321
0.5587950546743	0.6271152987175
0.5588554713155	0.6271543473993
0.5589378953867	0.6271946614248
0.559009628011	0.6272992049595
0.559069989932	0.6273094721724
0.5591487597358	0.6274215209758
0.5592097762575	0.6274807873045
0.5592814846951	0.6274849638912
0.5593687554578	0.6275554777319
0.559469331727	0.6276572414758
0.559564627936	0.6277284203977
0.5596310443722	0.6277293178554
0.5597096153239	0.6278560283934
0.559801371938	0.628102082504
0.5598912058276	0.6281606145421
0.559959752196	0.6282434542188
0.5600350095457	0.6283292618951
0.5601164752786	0.6283833759729
0.5602013747157	0.6284677572197
0.5602802167676	0.6284909552078
0.56035449151	0.6284921141815

0.5604718840888	0.6290732825261
0.5605922753863	0.6293571974212
0.5606909893347	0.6296394427833
0.5607730218664	0.6296964113535
0.5608884735181	0.6298802800325
0.5609520788436	0.629885025466
0.5610295589577	0.6299913630629
0.5611117516121	0.6300946809285
0.5611914672179	0.6300949056105
0.5612884407081	0.6301813778742
0.5613921996882	0.6302725546644
0.5614687272725	0.6303072254281
0.5615549409358	0.6303922314512
0.5616546675825	0.6305029609275
0.5617425875126	0.6306247880767
0.5618094957531	0.63066925146
0.5619013447539	0.6307310778851
0.561978476104	0.6307627170766
0.5620487883589	0.6308131486027
0.5621212226133	0.6308260173983
0.5622121558486	0.6309664475814
0.562293470402	0.6309894989503
0.562387521945	0.6310806787154
0.5624916474528	0.6310814967651
0.5625795008867	0.6311738261724
0.5626818564077	0.6313772671334
0.5627516249859	0.6315196191424
0.5628286632648	0.6315206975957
0.562930418757	0.6317135386994
0.5629948759944	0.6317788108748
0.5630747454837	0.631870386892
0.5631505493872	0.6321622327254
0.5632237000967	0.632220104516
0.5633288882724	0.6325823907546
0.5634257020764	0.6327183559024
0.5635062458123	0.6327256233331
0.5636088069439	0.6328453356726
0.5636769830743	0.6329625321206
0.563790066703	0.6330329020062
0.563869436627	0.6330809003311
0.5639604567059	0.6333076825054
0.5640409865689	0.6333121514574
0.5640996480814	0.6333730879551
0.5641753794843	0.6336417862869
0.5642763715159	0.6338079919781
0.5643286553889	0.6338452718343
0.5643835710116	0.6339490580547
0.5644716174489	0.6340335851313
0.5645591021225	0.6341321216686
0.5646401686823	0.6341733625593
0.5646956106335	0.6343014114861
0.5648013157595	0.6345030855518
0.5648717090294	0.6345161978192
0.5649617839038	0.6346771102133

0.565021525614	0.6347870091191
0.5650973276519	0.6348037802187
0.5651853075622	0.6349659012791
0.5652671916323	0.6349782272738
0.5653357636573	0.635022653917
0.5654152960646	0.6350901852413
0.565489943312	0.6351039494895
0.5655297751067	0.6351341000852
0.5655967731892	0.6352593815913
0.5656749565917	0.6353224110171
0.5657825416359	0.6354723381948
0.5658561076544	0.6355883553176
0.5659207172051	0.6356783540583
0.5659891101179	0.6357566652292
0.5660969168257	0.6361652627177
0.566187179195	0.6364150437242
0.5663013476769	0.6364310955383
0.5663793552761	0.6364970891745
0.5664429557021	0.636559416902
0.5665150009501	0.6367227957613
0.5665976434246	0.6370957602529
0.5666783841593	0.6371842702904
0.566782020785	0.6373491317538
0.5669046425365	0.6375406672832
0.5669877692578	0.6375549562506
0.5670395588824	0.6375596097227
0.5671027853155	0.6376012640561
0.5671918069851	0.6376876763111
0.5672730716249	0.6377480282518
0.5673409030053	0.6377873054318
0.5674107727043	0.637988485485
0.5674794792336	0.637996484541
0.5675786675876	0.6380837836648
0.5676599386881	0.6380902995971
0.5677393239208	0.6380969876449
0.5678212941442	0.6382940676349
0.5678930836124	0.6383046085863
0.5679632709529	0.6383046408886
0.5680779037047	0.6383227159334
0.5681658728403	0.6383933842986
0.5682133306462	0.6386433836588
0.5683474311138	0.6390336085043
0.5684211272614	0.6390987375856
0.5685192658102	0.6391047778447
0.5685824598428	0.639133577877
0.5686709186122	0.6395010135749
0.5687684028575	0.6397411162303
0.5688963844639	0.6397858409586
0.5689755299465	0.6399840164359
0.5690546732812	0.6400548458005
0.5691600543529	0.6403946146793
0.5692514032864	0.6405811478904
0.5693115093964	0.6405914616908
0.5693743202708	0.6406421105464

0.5694611152428	0.6406842917526
0.5695264196605	0.6407065173175
0.5695961410327	0.6408336435141
0.5696878554031	0.6408914920582
0.5697929983792	0.640971980967
0.5698524689171	0.6410018446245
0.5699627254911	0.6412026964616
0.5700497290355	0.6412571403711
0.5701485231665	0.6412966513558
0.5702171668818	0.6413539623419
0.5703323300117	0.6414435576422
0.5704021828967	0.641483322077
0.5704932373949	0.6415029536044
0.5705836049938	0.6417502859107
0.5706415243909	0.6418805252755
0.5707346609723	0.6420060304329
0.5708073114754	0.6420101502335
0.5709137066113	0.6421129543895
0.5709740857798	0.642138317614
0.5710746624091	0.6421928294321
0.5711590219067	0.6422587488994
0.5712251567798	0.6423360821676
0.5713030468361	0.642465672012
0.5713754575727	0.6424664531764
0.5714363621478	0.6424670986642
0.5715590802594	0.6426036129605
0.5716701975095	0.64273993793
0.5717553626795	0.6427911449785
0.5718507950124	0.6428628697708
0.5719446008824	0.642890630976
0.5720098273365	0.6429019746242
0.5720815192531	0.6429113398625
0.572168313453	0.6429405237736
0.5722343221914	0.6429904480855
0.5723038832921	0.6431126428583
0.5723629335567	0.6431428659456
0.5724479695644	0.6431833878603
0.5725348143982	0.6433624336422
0.5725909833696	0.6434798383804
0.5726861723856	0.6434802055758
0.5727732572389	0.6435621956483
0.5728424757235	0.6435903671078
0.5729375798023	0.6436430023217
0.573007594825	0.6436561176534
0.5730938349197	0.6438353733467
0.57316346715	0.6438744965464
0.57325547752	0.6440316987205
0.5733407140152	0.6442920470799
0.5734384284082	0.6443754419929
0.5735301339842	0.6443807386384
0.5736092982055	0.644727635869
0.5737029078503	0.6451110832309
0.5737825786476	0.6451657413995
0.573854698033	0.6452269179653

0.5739348755844	0.645350049869
0.5740344091449	0.645436927752
0.5741130002923	0.6455783459229
0.5742102740413	0.645684349026
0.5743105994154	0.6457586770083
0.5743811464108	0.6458280054001
0.5744643961267	0.6459865481092
0.5745467603401	0.6461284285558
0.5746315098911	0.6461872088204
0.574700979678	0.6462064399534
0.5748033898105	0.6463862134121
0.5748713763409	0.6465146674089
0.5749381961953	0.6465464275663
0.5750016932764	0.6466028198704
0.5750864510208	0.6466830757252
0.5751699687849	0.646702156463
0.5752499495306	0.646716549491
0.5753611789289	0.6469556001768
0.5754311731707	0.6471245131581
0.5755057983496	0.6473742492617
0.5755874747297	0.6473843939769
0.575681793443	0.6474300223156
0.5758002347189	0.6476921704419
0.5758704625549	0.6476924933935
0.5759680856306	0.6477343745546
0.5760348064535	0.6477615994518
0.5761303498893	0.6479733802436
0.5762213344053	0.6480554229082
0.5762773824086	0.6481256267789
0.5763217133103	0.6481568205594
0.5764273382196	0.6481678146464
0.5764992062123	0.6482434486644
0.5765762584988	0.6484304139364
0.5766738356882	0.648544598502
0.5767370065524	0.6485891212475
0.576891065773	0.6488310276755
0.5769586701772	0.6488395601044
0.5770537697053	0.6489676988
0.5771312021089	0.6490266489639
0.5772353636495	0.6490310915063
0.5773197215441	0.649047702007
0.5774123347363	0.6492418962066
0.577491737142	0.6492943522738
0.5775573216523	0.6492985478438
0.5776478055123	0.6493015079908
0.5777293209363	0.6495962530598
0.5777860641712	0.649603992863
0.5778635846919	0.6498075761851
0.577944546667	0.649812185759
0.5780305688718	0.6498810655149
0.5781151346816	0.6499439684215
0.5781967478569	0.6501824768634
0.5782648730724	0.6506284054444
0.5783406988952	0.6506483195449

0.5784143201083	0.6507398845407
0.5784802818116	0.6507675413016
0.5785441224097	0.6509718142467
0.578651005145	0.651052762319
0.5787392556694	0.6510980448468
0.5788549401007	0.6514340766963
0.578948920577	0.6515628413104
0.5790085274438	0.6515706076824
0.5791339080525	0.6519406650079
0.5791960649833	0.6519931270322
0.5792534551886	0.6520075823608
0.5793517316305	0.6523052088517
0.5794219071661	0.6523750148822
0.5795167859045	0.652379593267
0.5795917582616	0.6526869895845
0.579675423243	0.6529285446683
0.5797396857309	0.6529974198667
0.5798521342456	0.6530521816276
0.5799274526539	0.6530856820784
0.5800052504644	0.6533234045294
0.5800894551322	0.6533308460793
0.5801702386325	0.6534253247429
0.5802744416579	0.6534771387446
0.5803751209201	0.6534931082853
0.5804600857388	0.6535435457549
0.5805483468101	0.6536716063306
0.5806249185839	0.6537095395846
0.5807397859547	0.653741175589
0.5808114171921	0.6537953994466
0.5809137783398	0.6539054711562
0.5809915767524	0.6539982900254
0.5810747193726	0.6540769350605
0.5811368782588	0.6541536629386
0.5812163794641	0.6541677912739
0.5813034541944	0.6547089935464
0.581388815333	0.6547338345338
0.5814754552707	0.6548135453692
0.5815480931232	0.6549956335005
0.5816245503189	0.6553781324457
0.5816830374027	0.6554396784573
0.581757295009	0.6555080219295
0.5818369404903	0.6555230499124
0.5819461200002	0.6555368879853
0.5820291698334	0.6555975401546
0.5821046968573	0.6556536387847
0.5822056604041	0.6557195631298
0.5822778594714	0.6557698467851
0.5823639492332	0.6558624603241
0.5824382695965	0.655878789559
0.582549232703	0.656005014898
0.5826184557118	0.6560771978431
0.5826825204632	0.6561580189218
0.5827699017427	0.6561738362393
0.5828708397287	0.6564747528808

0.5829381646705	0.6567200302848
0.5830207663608	0.6568447196428
0.5830773091093	0.6568521074581
0.5831606720902	0.6569556098777
0.5832220845936	0.6569962743104
0.583316886886	0.6570442142072
0.5833832748336	0.6571158738749
0.5834611618385	0.6571767441726
0.5835204148877	0.6572366740355
0.583621447529	0.6577222734904
0.5836889362488	0.6578830133009
0.5837774339441	0.6579842595571
0.5838788689185	0.6580623134295
0.5839726284394	0.6580652775113
0.5840551678711	0.6581529297642
0.5841385950364	0.6582205695037
0.5842218932425	0.6582682430578
0.584331827051	0.6583668476043
0.5843993005372	0.6584314005754
0.5844765003656	0.6585137500067
0.5845663586823	0.6586487566427
0.5846655061095	0.658729424761
0.5847639194715	0.6587466513833
0.5848481725195	0.6587495506433
0.584953048234	0.6588117627826
0.5850511964368	0.6588817905334
0.5851418338223	0.6590818778412
0.5852315290545	0.659106546048
0.5853533971735	0.6594385222978
0.5854290585781	0.6595038198861
0.5855298466635	0.6595139731096
0.5856304783571	0.6595540369384
0.5856788393987	0.6595636805001
0.5858031030256	0.6596369011028
0.5858738850562	0.6596462760827
0.5859666831667	0.6597216439621
0.5860528629279	0.659792534839
0.5861103568035	0.6599532026157
0.5861548940692	0.6599934480183
0.586197127084	0.6599980729157
0.5863025303964	0.6604272369981
0.5863827439176	0.660485819101
0.5864508666216	0.6605986947218
0.5865262986141	0.6606150493141
0.5865998592228	0.6607172363653
0.5866990218058	0.6608377142648
0.5867861871284	0.6608699170983
0.5868687276674	0.6608704343839
0.5869461574698	0.6608944417423
0.5870180983755	0.6609938640757
0.5870855319096	0.661033931048
0.5871718778289	0.6612230412059
0.5872600197323	0.6612333825535
0.5873446527852	0.6612382766212

0.5874256998355	0.6613112380356
0.5875411002874	0.6615368741037
0.5875903829305	0.6615377229397
0.5876923776926	0.6618002924946
0.5877800460704	0.661837076358
0.5878667840149	0.6619044442225
0.5879593118634	0.6620586771573
0.5880440082796	0.6621742940661
0.5881286197821	0.6622694456182
0.5882443119956	0.662580370017
0.5883371869594	0.6626762073369
0.5884346479126	0.6629156389241
0.5885044676771	0.6629562776506
0.5885787620148	0.6629775343751
0.5886329582047	0.6629802778572
0.588719837142	0.6631361475378
0.5887967743278	0.6631808166615
0.5889119010999	0.6635408582989
0.5890021128173	0.6635643256555
0.5890999824743	0.6637082934517
0.5891818843509	0.6638057643035
0.5892651077665	0.66394531628
0.5893247819109	0.6640005548562
0.5893993863545	0.6640599717812
0.5895054430827	0.6643783354397
0.5895983839579	0.6646506090283
0.5896839270343	0.6647258742326
0.5897523427023	0.6648629648735
0.5898041956788	0.6649189477491
0.5898684530324	0.6649653357367
0.5899341097842	0.6650119760229
0.5899868509516	0.6650892084284
0.5900527155954	0.6652859980831
0.5901261655178	0.6655066057161
0.5902137795038	0.6655131952204
0.590298008163	0.6656306249491
0.5903508541544	0.6656395695213
0.5904438112688	0.6658179079261
0.5905185190136	0.6658564250956
0.5905807155943	0.6658600723574
0.5906621186357	0.6658605709852
0.5907915311811	0.6664164672879
0.5908501830454	0.6664890634955
0.5909449474823	0.6667275601552
0.5910249567511	0.6667747335798
0.5911047416118	0.6668295505179
0.5911831777499	0.6668317187441
0.5912608979121	0.6669039704262
0.5913290331094	0.6669325798001
0.5914036482697	0.6669701611985
0.5914763888686	0.6669803343581
0.5915610755816	0.666997624035
0.5916346341745	0.6670077930408
0.591748256639	0.6672747392941

0.5918316445346	0.6674104419667
0.5919041349271	0.6674127281818
0.591980762323	0.6674253723715
0.5920549989924	0.6674450781499
0.5921380175226	0.6676470336514
0.592227760822	0.6677133991281
0.5923107780683	0.6677974595248
0.5923772646848	0.6678180563405
0.5924543553631	0.6679143044756
0.5925497101128	0.6682081486221
0.5926246609594	0.6682397577796
0.5927137471834	0.6683451386897
0.5927859240107	0.6684034164893
0.5929045448262	0.669059968684
0.5930055533315	0.6693524205759
0.5930775982215	0.6693632605775
0.59316073256	0.6694032616714
0.5932262634069	0.6694938865196
0.5932962231497	0.6694992102425
0.5933802475415	0.6695741879821
0.5934590138718	0.6696238046207
0.5935419335021	0.6696368754566
0.5936263583974	0.6696770446841
0.5936850388869	0.6696786400892
0.593796544435	0.6697953856729
0.593882260665	0.6699059799575
0.5939616035821	0.6701472841646
0.5940728519834	0.6703340676238
0.5941640880995	0.6706788846669
0.5942631511519	0.6710072673726
0.5943291917888	0.6712159359584
0.5943951329607	0.6712573729463
0.5944586305917	0.6712973241541
0.5945259164044	0.6713792285
0.594609989098	0.6714817493619
0.5946841910136	0.6714828950111
0.5947549358762	0.6714839868782
0.5948236167038	0.6715089554162
0.5948945663044	0.6715173917336
0.594967478373	0.6715189590825
0.5950341511133	0.6715256086293
0.5951301726369	0.6715653840096
0.5951970742638	0.6716129083397
0.5952767986505	0.6717769169498
0.5953661355979	0.6720501027746
0.5954300246999	0.6720840830685
0.5955268668636	0.6722419106054
0.5956101925525	0.6722802159588
0.5956934911561	0.6724369713995
0.5957710441849	0.6724428521811
0.5958451551483	0.6725394771805
0.5959147337634	0.6727249182956
0.5959788485353	0.6729641499582
0.596033200906	0.6729836823245

0.5961430538347	0.6729946605284
0.5962426477169	0.6730256577805
0.5963019231418	0.6730518078863
0.5963836869721	0.6732550709482
0.5964978655993	0.6733654464294
0.5965582027584	0.6734066090233
0.5966333046475	0.6734635243197
0.5967063267498	0.6735159042608
0.5967987798115	0.6736246735452
0.5968735063211	0.6736840092563
0.596937919438	0.6737029493828
0.5970172889252	0.6737506974896
0.5971055555698	0.6738058575657
0.5971885419576	0.6738775884206
0.5972883023886	0.6741346215729
0.5973837500339	0.6742153551159
0.597485506707	0.6743494868926
0.5975869114465	0.6744827624578
0.5976570896926	0.6745101603721
0.5977496331289	0.674657060083
0.5978036351097	0.6747235556923
0.5978935974112	0.6747722502839
0.5979650801855	0.6747926989695
0.5980248941401	0.6747976354548
0.5980922257953	0.6748401173899
0.5982086776085	0.6748751829325
0.5983234441675	0.6752428579131
0.598416847659	0.6752657511806
0.5984927688472	0.6753162792818
0.5985426200113	0.6753195281109
0.5986076278654	0.6753433550912
0.5987141551417	0.6753755382256
0.5988175538729	0.6755026239791
0.5988941951447	0.6755870342396
0.5989563807137	0.6755900903938
0.5990708806993	0.6758743321468
0.5991443883198	0.6761945430998
0.5992437993241	0.6762451769857
0.5993154752373	0.6762454774091
0.599416734587	0.6762496517424
0.5994663753464	0.6762541181151
0.5995337410069	0.676269587964
0.5996703603161	0.6768986011056
0.5998210094208	0.6769670374407
0.5999290300673	0.6769849336282
0.5999761889516	0.6770648762549
0.6000529460163	0.677141748799
0.6001263883622	0.6771815270782
0.6001688456753	0.6772462921388
0.6002481826277	0.6772541262574
0.6003101363861	0.6772911624751
0.6004254116307	0.6773217059176
0.6005080206187	0.677585931368
0.6006012077796	0.6778099381558

0.6007048203273	0.6778234373984
0.6007654843563	0.6778505152419
0.6008538727542	0.6778635999045
0.6009375722198	0.6779115320826
0.6010216783876	0.6780793279141
0.6011098631275	0.6784887638959
0.6012519001718	0.6786575434475
0.6013068364382	0.6786606121362
0.601387471621	0.6787010961591
0.6015019304523	0.6787565424515
0.6015760626181	0.6787597778749
0.6016739167007	0.6790569902208
0.6017319015848	0.6792270308218
0.6018103105981	0.6793280764419
0.6018740173433	0.6794583155546
0.6019627653565	0.679797157211
0.6020448262789	0.6799792952415
0.6021431527997	0.6800310371474
0.6022208465123	0.6800470236598
0.6023163951719	0.6801986406731
0.6024116437357	0.6804135763547
0.6024816036201	0.6804472130301
0.6025830025658	0.6805736424295
0.6026466295721	0.680626290953
0.6027368677003	0.6809125348661
0.6028151711369	0.680933750285
0.6029115077606	0.6811266674761
0.6029766018284	0.6811328346607
0.6030760772368	0.6812012404156
0.6031939534486	0.6815813160566
0.6032885498827	0.6817877640037
0.6033806524874	0.6818472451878
0.6034405357087	0.6819508593817
0.6035401255981	0.6820594851323
0.603619803274	0.6820980530781
0.6036931384601	0.6821121979317
0.6037741676409	0.6821734887637
0.6038459207428	0.6822025969932
0.6039385802724	0.6822202331287
0.6040379553541	0.6824660927286
0.6041170097472	0.682604903348
0.6041808032061	0.6827112539591
0.6042719178322	0.6827176687663
0.6043526708299	0.6827326556673
0.6044353737902	0.6828438908941
0.6045081432585	0.682937120599
0.6045821299118	0.6830893223552
0.6046668912887	0.6833174590715
0.6047466774956	0.6833563977304
0.6048604779176	0.6837240967414
0.6049311366209	0.6838658433376
0.6050080301549	0.6840687633569
0.6051225391581	0.6847096119866
0.6052046116251	0.6847822630416

0.6053058212217	0.6849781317907
0.6053762216242	0.6849888880747
0.605473360331	0.6851378144913
0.6055873133944	0.685157115627
0.6056780523765	0.6851716102424
0.6057410427651	0.6853157114166
0.6058383843052	0.6855415453264
0.6059240364174	0.6855945306433
0.6060168011101	0.685621396498
0.6061129406984	0.6859436288333
0.6061723033791	0.6859992954003
0.6062512415608	0.6860212153557
0.6063249062518	0.6860225920638
0.6063841031866	0.6860358635987
0.6064905614785	0.686217286947
0.6065961962525	0.6862963492443
0.6066691324418	0.6862996314021
0.6067455279757	0.6863473164938
0.6068292835527	0.6863515237388
0.606902455739	0.6864587032651
0.6069852481935	0.6865048152862
0.6070857492591	0.6865284752825
0.607167717257	0.6866564467106
0.6073017888141	0.6869003867644
0.607399773636	0.6871279403126
0.6074843792728	0.687290862877
0.6075703757925	0.6873737385
0.6076661991052	0.6875062905338
0.6077450953755	0.6876276219675
0.6078159538773	0.687679654896
0.6078872288784	0.6876825227249
0.6079899100065	0.6879754644091
0.6080527241118	0.6880132070431
0.6081478523295	0.6880681628207
0.6082126674408	0.6882171753658
0.6082983297358	0.6883321417155
0.6083957939728	0.6884547362311
0.6085067794866	0.6886767295074
0.6085705705798	0.6887961699976
0.6086547470156	0.6887982705753
0.6087182991202	0.6889165681819
0.6088061060263	0.6889553121493
0.6088793261699	0.6892381095923
0.6089730588598	0.6893149891037
0.6090357284093	0.6893607316889
0.6090913834553	0.6893677270792
0.6091663912425	0.6893943454868
0.6092364797956	0.6894300523031
0.6093301578664	0.6894328313767
0.6093967030882	0.6894918797262
0.6094835013691	0.6898925689588
0.6095672796235	0.6899152185802
0.6096489149376	0.6899231853309
0.6097326128465	0.6899699563411

0.6097896194701	0.6900774659837
0.6098569275252	0.6901008901196
0.6099190144812	0.6901072724506
0.6099914814812	0.6901634724276
0.6100876864117	0.6902472319535
0.6101661508721	0.690253016136
0.6102421356481	0.6903298374307
0.6103159406291	0.6904696227416
0.6103876672345	0.6905273593931
0.6104579489637	0.6906558419249
0.6105537173955	0.6909170159265
0.6106442285807	0.6909343012117
0.6107355780267	0.6909644406179
0.6108367683076	0.6909871807195
0.6109274864923	0.69099930949
0.6110026163815	0.6910913237803
0.61109362956	0.6911247748253
0.6111729225965	0.6913671760044
0.6112633640068	0.6914171429354
0.611361088503	0.6914398233936
0.6114545723731	0.6915418066744
0.6115381977972	0.6915740413329
0.6116198437363	0.6917734285386
0.6117072135493	0.6919199606163
0.611792173542	0.6919221239731
0.611887954698	0.6919448390761
0.6119787937107	0.6919610153977
0.6120437714409	0.6921295540674
0.6121381084946	0.6922036010986
0.6122272678123	0.6923081180324
0.6123065971657	0.6923445276383
0.6123970142747	0.6924291023593
0.6125318728732	0.6926078942483
0.6126443462837	0.692721429337
0.612699491695	0.692862259038
0.6127743074364	0.6929566072185
0.612892572332	0.6932700260886
0.612997452882	0.6934299215408
0.6130841799522	0.693495861656
0.6131665953396	0.6935640601826
0.6132762547824	0.6936518761344
0.6133557940327	0.6936705681421
0.6134570667221	0.6937077947354
0.6135420664054	0.6937950977354
0.6136379161145	0.6938703758191
0.6136962353825	0.6938757941187
0.613772513704	0.6939307238894
0.6139040314666	0.6942329154256
0.6139671343002	0.6943065351214
0.6140473888069	0.6943677036121
0.6141346606929	0.6944624403595
0.6142133518849	0.6945353636429
0.6142889409975	0.6946162845088
0.6143730116732	0.6949694463693

0.6144408253243	0.6949754409758
0.6145070491532	0.6950232376404
0.6145848936434	0.695331840833
0.6146663840039	0.6955967955458
0.6147530486155	0.6958012096341
0.6148198489484	0.6958477809819
0.6148901170399	0.6958792217427
0.6149659279365	0.6960279478415
0.6150658149477	0.6960281652275
0.6151463805379	0.696145715256
0.6152199992565	0.6961468883374
0.6153058224691	0.6963030100175
0.6154000926114	0.6963893801229
0.6154797449958	0.6964011632276
0.6155638658692	0.696405466778
0.6156966431652	0.696459678158
0.6157788992246	0.6966277868904
0.6158685708003	0.6967191550732
0.615981229484	0.6967597591864
0.6160848879946	0.6968629216953
0.6161797698054	0.6969784723015
0.6163106317153	0.6976909689615
0.6163898084994	0.6977571152693
0.6165104702016	0.6979353843149
0.6165627382974	0.6979589399811
0.6166488473275	0.6979929012462
0.6167137849515	0.6979968722971
0.6168032823129	0.6980855717409
0.6168768468883	0.6980960445923
0.6169796937368	0.6983097975914
0.6170676413386	0.6983380946263
0.6171412839411	0.6984014219135
0.6172660422058	0.6986019790822
0.617362730191	0.6986215127525
0.6174809230306	0.6986707513687
0.617574504764	0.698732143466
0.6176650259786	0.6987837262726
0.6177239164415	0.6988075321285
0.617783226526	0.6990117662718
0.6178714617679	0.6990348184396
0.6179431047675	0.6991055019169
0.6180234316868	0.6993858176271
0.6180899554503	0.699478134933
0.6181709480576	0.6996571813046
0.6182379648222	0.6996687307359
0.6183433782204	0.6998249997122
0.6184089252815	0.6998449583906
0.6185023439047	0.6999595036344
0.6185851155973	0.7000874637209
0.6186739410495	0.7002129788488
0.6187713486112	0.7005060242025
0.6188345839634	0.7005241545368
0.6188997934242	0.7006351892777
0.6189880217395	0.7007495647735

0.6190687732973	0.7008208937716
0.6191446499107	0.7009101653446
0.6192457644554	0.7011548757488
0.6193094882188	0.7012271243931
0.6193948707526	0.7013346647917
0.6194593424145	0.701373812214
0.6195178359775	0.7014307924934
0.6196005071396	0.7015134201425
0.6196798494892	0.7015663484766
0.6197475417363	0.7015823712052
0.6198054108419	0.7015992004301
0.6198911788273	0.7016672906133
0.6199743175987	0.7017801914285
0.6200792581068	0.701862363807
0.6201646147378	0.7021580529702
0.6202315174173	0.7021711401019
0.6203173861294	0.7023576636101
0.6203923126832	0.7023598648317
0.6204703215275	0.7024644545303
0.62054444799	0.7025425598206
0.6206348843453	0.7026578307747
0.6207269437009	0.7028790625493
0.6208168543286	0.7029450171667
0.6209020362728	0.7030056944446
0.6209752378833	0.7030124906819
0.6210773659512	0.7032132060758
0.6211328131322	0.7033926601241
0.6211877458112	0.7034080401852
0.6212737206335	0.7035694711218
0.6213688945629	0.7036425158377
0.6214287545016	0.703739997768
0.6215093667432	0.7037895768718
0.6215797979328	0.7038048220483
0.6216688995847	0.7041074434821
0.621770522068	0.7042241442411
0.6218394936368	0.7043935516463
0.6219260277799	0.7044078470845
0.6220029731566	0.7044129556474
0.6220938462361	0.7044984083096
0.6221701904449	0.7044990722687
0.6222518463233	0.7046698691132
0.6223377700302	0.7048788723276
0.6224211435183	0.704925427326
0.6225141120983	0.7049314087085
0.6226118409875	0.7049852446479
0.6227209438909	0.7050733948163
0.6227890975401	0.7051450953788
0.6228601929023	0.7052480722528
0.6229388371707	0.7052946902756
0.6230600785058	0.7053846765928
0.6231637067588	0.7055452155868
0.6232503900837	0.7056115393647
0.6233410074859	0.7057163473954
0.6234092901251	0.7057822354071

0.6234914742342	0.7058279814461
0.6235464227184	0.7058820375371
0.6236067697973	0.7059773694953
0.623675626389	0.7061427864643
0.6237694585083	0.7062523077929
0.6238537567219	0.7062829679402
0.6239353903362	0.7063225205761
0.6240339645437	0.7064050534428
0.6241219879467	0.7065296495916
0.6241806842691	0.7065941223186
0.6242723227479	0.7066405737559
0.6243620460659	0.7067413771763
0.6244598913322	0.7067756334381
0.6245584656478	0.7069986298154
0.6246287115707	0.7072170982662
0.6247241199304	0.707302641051
0.6248214678105	0.7073165482009
0.6249096874885	0.7074071440246
0.6249893983201	0.7074776698695
0.625040176106	0.7075329903478
0.6251256279133	0.7075521202567
0.625193473596	0.7075628782712
0.6252609030023	0.7076679137512
0.625339454936	0.7078386941039
0.6254088992529	0.7079366323702
0.6255167274977	0.7079773841646
0.6256211166362	0.7081201960936
0.6257285023457	0.7081720888504
0.6258012961386	0.7081877740835
0.6258865870683	0.708198792841
0.6259573910944	0.7082976712981
0.626065237294	0.7084483920037
0.6261329836183	0.7084676937178
0.6261981592943	0.708596098287
0.6262680218345	0.7086202322025
0.6263525139955	0.7087484943358
0.626438982973	0.7088601047255
0.6265231596846	0.7094396271312
0.6265828118379	0.7094442212401
0.6266716047268	0.7095104245257
0.6267583785715	0.7095199563471
0.6268730670843	0.7096376829183
0.6269373687641	0.7096635307571
0.6270234720869	0.709716621083
0.6270965101822	0.7097441864667
0.6271768081368	0.7099109918018
0.6272505584358	0.7100363056002
0.6273247578947	0.7103689830407
0.627386942807	0.7103716441422
0.6274589445697	0.7103807596851
0.6275172006433	0.7103879783625
0.6276305458658	0.7105229208004
0.6277047734021	0.7106022448093
0.6277711501182	0.710681997657

0.6278541143853	0.7107814388894
0.6279480505698	0.7111942329339
0.6279979354854	0.711261324718
0.6280914526314	0.7115373218779
0.6281895068387	0.7115469537071
0.6282785055767	0.7117151918559
0.6283741214295	0.7120198146853
0.6284690893803	0.7122665110441
0.6285769916417	0.7124387891545
0.6286784983594	0.7124388765271
0.6287479770706	0.7125315436939
0.6288095917112	0.7125789501343
0.6289088623746	0.7126246801809
0.6289931124692	0.7127494858813
0.6290962909707	0.7129544202066
0.6291885212288	0.7131212541951
0.6292375538284	0.7131271953985
0.6292931836312	0.7132390957475
0.6293760662622	0.7132953221561
0.629465221171	0.7134247544043
0.6295414706865	0.7135218687212
0.6296542562528	0.7135222258591
0.6297230211766	0.7137727984686
0.6297654929714	0.713795730882
0.6298368662741	0.7138539212609
0.6298958011255	0.7139159777656
0.6299747265395	0.7139404519791
0.6300984115593	0.7140476988968
0.6301623360952	0.714312631194
0.6302393087203	0.7143815178661
0.630335260976	0.714422279279
0.63040055915	0.7144435450722
0.6304887414234	0.7145595204059
0.6305705561861	0.7145717708817
0.6306564993958	0.7146134804068
0.6307535871216	0.714707261416
0.6308124195297	0.7147483243409
0.6309048984802	0.7147608024355
0.6310053745551	0.7149261756299
0.6310757662159	0.7151518600971
0.6311454140014	0.7151582838032
0.6312353172124	0.7152131276457
0.6313582481879	0.7154468180796
0.6314414320479	0.715465024978
0.6315253291567	0.7157016745399
0.6316010325251	0.7159732951583
0.6317077699667	0.7160072089308
0.6317706844351	0.7161396525886
0.6318487032091	0.7161461804355
0.6319522465159	0.7164690839711
0.6320382093753	0.7165266415756
0.6321199518797	0.7166749975457
0.6322057111669	0.7166779327469
0.6322641291077	0.7167266273993

0.6323587718615	0.7171861929399
0.6324671860292	0.7174448393082
0.6325387553908	0.7174885411764
0.6326677645924	0.7179574449687
0.6327679588069	0.7180742573906
0.6328896723694	0.7182002541328
0.6329717129657	0.7182796918856
0.6330488731854	0.7183415687261
0.6331205539728	0.7183448219409
0.6331862429541	0.7183483543024
0.6332808707235	0.7183760516297
0.6333714150675	0.7184412905724
0.6334553440878	0.7185638450869
0.633557225702	0.718734170155
0.6336295739114	0.7188963735851
0.6337190082225	0.7189580365983
0.6337813327251	0.719050457037
0.6338891387191	0.7191146941716
0.6339506728094	0.7191608184511
0.6340315764066	0.7191860304076
0.6341279694306	0.7192760976755
0.6341979810821	0.7193723791327
0.6342876652518	0.7195965803918
0.6343778218681	0.7196079195094
0.6344491109755	0.7196456817255
0.6345648695634	0.7200423013975
0.6346350630763	0.7200940032823
0.6347178636638	0.7202943681252
0.6348321028955	0.7204529744055
0.6349235928817	0.7205857700049
0.6350102129722	0.7207517527785
0.6350748025543	0.7208685045431
0.6351564958329	0.7209390850898
0.6352196641537	0.7209461679356
0.6353116254783	0.7209963704671
0.6353947434547	0.7211307033116
0.6354486920542	0.7211862914145
0.6355217050325	0.7212705673333
0.6356320640658	0.721423140678
0.635722525386	0.7214420190989
0.6358093965406	0.7214521993785
0.6358999293682	0.7214723967461
0.6359853072393	0.7215733421407
0.6360541630967	0.721594583855
0.6361846005333	0.7216168992015
0.6362801309773	0.7216245274929
0.6363817847083	0.7216745353068
0.6364828897966	0.7218346621098
0.636580522164	0.7218358767366
0.6366457704986	0.7218740241385
0.6367133038735	0.7218838067124
0.6367984577901	0.7219048143914
0.6368731218965	0.722049992383
0.6369554570678	0.7220604527752

0.6370252656955	0.7220699862053
0.6371174798841	0.7225401733112
0.6371728171359	0.7225404702324
0.6372833281964	0.7226480409826
0.6373736520974	0.722877511385
0.6374662115551	0.7229226141729
0.6375663769282	0.7229245182802
0.6376609806977	0.7229585902764
0.6377484257484	0.7229996022681
0.6378332069027	0.7233145732425
0.6379120544706	0.723378538378
0.6379887992745	0.7234540778979
0.6380803839705	0.723496072841
0.6381632881654	0.723636882572
0.6382731443869	0.723732456747
0.6383427986829	0.7238531070493
0.6384047902945	0.7238704185929
0.6384414205589	0.7238801249587
0.6385151137247	0.7239289013097
0.6386195167399	0.7241000975204
0.6386822887917	0.7243575464231
0.638797744474	0.7244063522719
0.6388855758019	0.7244379682895
0.6389791666871	0.7244564489512
0.6390530841283	0.7246588470963
0.6391553580861	0.7247430371694
0.6392415269146	0.7249941563418
0.6393232701802	0.725004952594
0.6394108705225	0.7251063613185
0.6395147018365	0.725143578391
0.6396210869858	0.7252513514172
0.6397047982697	0.7253990020193
0.6397934084067	0.7255819694623
0.6398761836483	0.7257820261155
0.6399797348939	0.7258060090709
0.6400555863001	0.7258854899603
0.6401484552007	0.7260387747439
0.6402582401486	0.7263600919252
0.6403524124831	0.7265587637909
0.6404293490705	0.7265741998867
0.6405097537698	0.7266169059855
0.6406060683748	0.7267051689412
0.640685573982	0.7268564997991
0.6407534042739	0.7268624027326
0.6408136993372	0.7269239500907
0.6408780902088	0.7269994898801
0.6409511330862	0.7271456725766
0.6410347900642	0.727184446891
0.6411184816056	0.7272691502399
0.6411914222911	0.7274886281262
0.6412791582142	0.7277553698591
0.6413641562233	0.7277602222679
0.6414460250356	0.7278588876766
0.6415615898719	0.7282282395967

0.6416199278537	0.7283929135567
0.641719581068	0.7284646308686
0.6418185977339	0.7284705403358
0.6419307818518	0.7285754923829
0.6420309223311	0.7288364935145
0.6421327666346	0.72896028124
0.6421943913779	0.7289811106623
0.6422954018896	0.7291603830744
0.6423972562834	0.7292169665326
0.6424623847446	0.7292195028701
0.6425270723055	0.7293908629872
0.6425951780114	0.7294375162962
0.6426489089316	0.7295591356673
0.6427113106743	0.7295873280055
0.6428260881502	0.7300896581203
0.6429080118642	0.7301028051022
0.6429740779837	0.7301941302389
0.6430860825812	0.7302657121345
0.6431870337879	0.7304337423755
0.6432424856539	0.7304404069173
0.6433053084215	0.7304407908238
0.6433754479975	0.7305283256005
0.6434685771821	0.7307636517339
0.6435219091358	0.7307654242723
0.6436336929575	0.7308185735585
0.643691604157	0.7309098823798
0.6437810101688	0.7309993332261
0.643874469238	0.7310289919423
0.643963113829	0.7312094446415
0.6440822614991	0.7314600369553
0.6441756059594	0.7319213848056
0.6442761482686	0.7321533536234
0.6443494321893	0.7322242468604
0.6444552411881	0.7324168766596
0.6445177492227	0.73247296999
0.6445819115993	0.732590689917
0.6446682773302	0.7326433403581
0.6447620038637	0.7327795688683
0.6448670518407	0.7330430187864
0.6449505717723	0.7333242039511
0.6450279476784	0.7334055256397
0.6450846104635	0.7336077682225
0.645175134762	0.7336099398225
0.6452480522155	0.7336285995889
0.645314846444	0.7337619620707
0.6454005949649	0.7339661619119
0.6454965303903	0.7340570867167
0.6455860452356	0.7340817980246
0.6456800467372	0.7341434115719
0.6457667165017	0.7341864938724
0.6458562533381	0.734457364589
0.6459273276694	0.7345413995351
0.6460082581895	0.734743084848
0.6460867231034	0.7348942800751

0.6461421441384	0.7349076932031
0.6462372706706	0.735184757837
0.6463161053146	0.7352004666388
0.6464257490291	0.7353037872953
0.6464969603552	0.735327537732
0.6466142238056	0.7354318835098
0.6466937660492	0.7354908091203
0.6467593520118	0.7355304673029
0.6468335403537	0.73556999319
0.6469257012504	0.7356436307415
0.6469968075187	0.7357111138277
0.6470562242932	0.7357349843666
0.6471418627594	0.7357368344679
0.6472387516549	0.7359824716208
0.6473106452689	0.736090743105
0.6473980696633	0.7361210069546
0.6474877606431	0.7361877345259
0.6475586849125	0.7363128908772
0.6476462249181	0.7363334114128
0.6477149953746	0.736382954003
0.6477872109106	0.7364509725197
0.6478548528929	0.736466798865
0.6479413738118	0.7365306409714
0.6480098866521	0.7366736371142
0.6481235380108	0.7367026657272
0.6481782001219	0.7367636251087
0.6482502169664	0.7368157624831
0.6483415513237	0.737078036569
0.6484314742395	0.7370959016869
0.6485041298784	0.7371580150258
0.6486216474542	0.7371873981636
0.6487042005696	0.7372251932345
0.6488066386579	0.7372473941432
0.648893104916	0.7373445411845
0.6489870889554	0.7375524508517
0.6491115711603	0.7377807262676
0.6491805594362	0.7377855776977
0.6492515480053	0.7377989689515
0.6493377255671	0.7378879371835
0.649432812562	0.738058987932
0.6495154766961	0.7380709924591
0.6496136023292	0.7382303527856
0.6497109516615	0.7382433042047
0.6497595224383	0.7384034594578
0.6498494985481	0.7385886087406
0.6499189622537	0.738648470711
0.6499880242639	0.7387012500466
0.6500613105703	0.7387076208871
0.6501310951069	0.738767621043
0.6502469873127	0.7390113716021
0.6503322350384	0.7391831457638
0.6503889067531	0.7392109280241
0.6504851483387	0.7392939093367
0.6505944078423	0.7393676170166

0.6506825666784	0.7395743287647
0.6507698404653	0.7397777843396
0.6508410755414	0.7397905321188
0.6509147030343	0.7398517376066
0.6510145503585	0.7400907970757
0.6511135758513	0.7402670527063
0.6511943596823	0.7402721158173
0.6512713702238	0.7403678943436
0.6513652054099	0.7406173680712
0.6514291347508	0.7407937569526
0.6515183549983	0.7408169258694
0.6516034647994	0.7409117641835
0.6517053514993	0.7409317534126
0.651776032909	0.741028996869
0.651872310507	0.7410393187454
0.6519568347066	0.7412304201904
0.6520519664118	0.7417440323876
0.6521448142188	0.7419229752182
0.6522139193091	0.7421068762675
0.6523069323256	0.7422707555947
0.6524019137635	0.7424753806947
0.6524713700251	0.7425072386741
0.652547626516	0.7426031806727
0.65265443716	0.7427042936186
0.6527235468581	0.7428436310145
0.6528017491743	0.7428838446449
0.6528924888522	0.7430763855052
0.6530395070952	0.7431153028077
0.6531149103723	0.7435599913376
0.6531996520024	0.743616412728
0.6532824917041	0.7436925751626
0.6533668283736	0.7437161727705
0.6534546323446	0.7437624137907
0.6535401644148	0.7437873655546
0.6536159722996	0.7440272440414
0.6536887560265	0.7440372147488
0.6537722111988	0.7441042307532
0.6538632362812	0.7441587195409
0.6539327867214	0.7442576167915
0.6540351216821	0.7442947922269
0.6541546963354	0.7447165068453
0.6542648002896	0.7448197928595
0.6543509433192	0.7449346900315
0.6544605341402	0.7450218083842
0.6545378262366	0.7451410359368
0.6546104461561	0.7452116759097
0.6547022371083	0.7452343608088
0.6547681971712	0.7452689862286
0.6548583747233	0.7452724015743
0.6549275889124	0.7453238747811
0.6550166169721	0.7455027360984
0.6550914998745	0.7456544411429
0.655197655193	0.7456576359795
0.655271336166	0.7457096243388

0.6553330840876	0.7457098487277
0.6553960495592	0.7457419060333
0.6554547347022	0.7458147301654
0.655542073778	0.7460611048747
0.655649296385	0.746246421009
0.6557304941171	0.7462660035979
0.6558363449218	0.7468079816649
0.6559343059695	0.7468323431635
0.6560457899177	0.7471223673377
0.6561105015761	0.7471688791957
0.6561854811601	0.7472116458567
0.656240178845	0.7472207982156
0.6563208850237	0.7474152136718
0.6564010604106	0.7474388997384
0.6564705144716	0.7475337918577
0.6565328748693	0.7475466510107
0.6565875878529	0.747577904409
0.6566568722994	0.7476341407337
0.6567637016302	0.7476593813602
0.656836763599	0.747783702226
0.6568957662043	0.7478232220571
0.6570251214304	0.7481185390392
0.6571045155085	0.7481616695301
0.657175131343	0.7481630565557
0.6572550306146	0.7481794511641
0.65735654715	0.7483851763026
0.6574309633563	0.7484224794669
0.6575482884329	0.7486566766485
0.6576539568067	0.7488607601892
0.6577306086535	0.7489897424256
0.6578247143392	0.7493799841117
0.6579038640496	0.7494826117398
0.6580015578555	0.7495622126515
0.6580954422871	0.7497056648375
0.6581935087242	0.7498715759013
0.6583039777901	0.749893499115
0.6583922068556	0.7500165885596
0.6585131596062	0.7501138355705
0.6585873774246	0.7502710417136
0.6586733094285	0.7504052536244
0.6587825505962	0.7504290364361
0.6588725812641	0.7504913360166
0.6589628300187	0.7505068221869
0.6590474823043	0.750741386368
0.6591486088587	0.7508695988294
0.6592313870567	0.7509237083252
0.6593112454342	0.7510165697024
0.6594062262058	0.7510755995663
0.6595193720086	0.7512570363438
0.6596117507015	0.7513263429646
0.659678397592	0.7513450785259
0.6597596734801	0.7513916578111
0.6598510828119	0.7516015331353
0.6599403382226	0.7518913607221

0.6600036818807	0.7519359061076
0.6600875019229	0.7521822989818
0.6601536229251	0.7521957672288
0.6602655295894	0.7523906553626
0.6603476137913	0.7525812531387
0.6604467636965	0.7526312107483
0.6605301882965	0.7526874584978
0.6606010869469	0.7527062992785
0.6606898735926	0.7528946848197
0.6607775377356	0.7529236834726
0.6608392130343	0.7529977364707
0.6609247310746	0.7531022420346
0.6610021950345	0.7531162154593
0.6610857096297	0.7532588661632
0.6611542353038	0.7533166530554
0.6612358852038	0.7533786861611
0.6613529284102	0.7535628715264
0.6614418959824	0.7535874575081
0.6615150559811	0.7536340610011
0.6616092299424	0.7537348723708
0.6616890587326	0.753815822788
0.6617395138827	0.7538583291791
0.6618184458609	0.7540131667499
0.6619172013053	0.7542649056006
0.6619941241281	0.7542746626714
0.6620706707607	0.7543910064639
0.6621510390943	0.754540654471
0.6622303767621	0.7546428024609
0.6623306874326	0.7546991183486
0.6624040379333	0.7547492068951
0.6624579072677	0.7548046965001
0.6625313626162	0.754812082827
0.6626277733505	0.7551633750471
0.6627176357254	0.7554597079746
0.6627941174348	0.7554995752958
0.6628598989605	0.755503478975
0.6629627012371	0.7555204333614
0.6630619876256	0.7556474188667
0.6631535587229	0.755963604234
0.6632430981955	0.7560473248056
0.6633257762931	0.7560650034805
0.6633962844881	0.7563694074398
0.6634559863014	0.7563728616748
0.6635378348155	0.75640167494
0.6636189526004	0.7565690507843
0.6636994681324	0.7566821098359
0.6637918797325	0.7568879968983
0.6639025360806	0.7570235426287
0.6639750036939	0.7571602755314
0.6640476425124	0.7572731317378
0.6641282148854	0.7572829002698
0.6642145687599	0.7573646931061
0.6643074120665	0.7573796904008
0.6643875542017	0.7575387793929

0.6645286165433	0.7575498299993
0.6646321446726	0.7576756163528
0.6647102991711	0.7577315454325
0.6648046107958	0.7578933465518
0.6648936255235	0.7579490417312
0.6649699688839	0.758093028419
0.6650411281484	0.7581184105431
0.6651233389899	0.7581447951889
0.6651933938549	0.7581599488817
0.6652645701384	0.758249500541
0.665372022221	0.758544553545
0.6654354623312	0.7585463897507
0.6654808967213	0.7585618243199
0.6655606621774	0.7586363871052
0.6656518786351	0.7587520353926
0.6657479065048	0.7587840161944
0.6658589638055	0.7588348829654
0.6659671836579	0.75886444966
0.666046982978	0.7588695440346
0.6661315420464	0.7588850926299
0.6661962739933	0.7588871024277
0.6662949587879	0.7590414826341
0.6663836369918	0.7590933586194
0.6664781833122	0.759183524734
0.6665947920353	0.7593964606726
0.6666638161381	0.7595582055445
0.666765401368	0.7596956954551
0.6668466268629	0.7597511519308
0.6669515988191	0.7599150671244
0.6670169863046	0.7600477964643
0.6671530241446	0.7604535245822
0.6672471602521	0.7606214702096
0.6673474864076	0.7606980937346
0.6674380074901	0.7608401826641
0.6675015595015	0.7609317415674
0.6675749302141	0.7609418410712
0.6676802011707	0.7610408556899
0.6677701826664	0.7610630410287
0.6678168566833	0.7610688087684
0.667911219193	0.7612357089039
0.6679764192132	0.7613511554765
0.6680752395333	0.7614121336478
0.6681406530885	0.7615322491815
0.6682475986883	0.7615773496482
0.6683262506735	0.7615904466734
0.6684180692048	0.7616783779022
0.6685279454676	0.7618514744203
0.6686054226193	0.7619730919742
0.6686574458512	0.7620571447472
0.6687585402139	0.7622571403768
0.6688588336715	0.7622819962486
0.6689298307572	0.7623071950685
0.6690233738991	0.7624241450688
0.6691028726831	0.7626706684217

0.6691771843509	0.7628920540992
0.669244471246	0.7628922706529
0.6693541823394	0.762921265841
0.6694267315065	0.762969097723
0.6694913228667	0.762971765325
0.6695943532542	0.7630255019857
0.6696455145768	0.7630525774212
0.6697200964961	0.7632164183675
0.6697774743647	0.7632447108018
0.6698678396936	0.7634272798569
0.6699662544122	0.7634604273125
0.6700888294254	0.763819074795
0.6701555565242	0.7638602373962
0.670237409258	0.7638823495681
0.6703083144687	0.7640061816762
0.6703920787803	0.7642426601612
0.6704663791192	0.7643187135818
0.6705515722967	0.7643350901747
0.6706283936339	0.7644183882361
0.6707073440135	0.7645056703938
0.6707899825225	0.7645589377889
0.6708456915252	0.7645607430762
0.6709132749747	0.764565534447
0.6709971112545	0.7646822838711
0.6710745268788	0.7647435240122
0.6711792274122	0.7651950092294
0.6712385844964	0.7652952029642
0.6713272380934	0.7653411726431
0.6714190522514	0.7654224062163
0.6715183815151	0.7657692619532
0.6716421574016	0.7662424716826
0.6717477631806	0.7662611818133
0.6718472782848	0.7662740743639
0.6719686770959	0.7667777974231
0.6720508421709	0.7668249778879
0.6721397574505	0.766881936763
0.6722370687104	0.7668967879644
0.6723161521531	0.7669687220578
0.6724074440673	0.7670452145436
0.6724622812793	0.7670758516774
0.6725414549671	0.7670787426614
0.6726189249983	0.7672156398158
0.6727250254796	0.7673417136379
0.6728151863646	0.7674607094544
0.6728710457194	0.7674951338511
0.6730148430902	0.7678566620146
0.6731036975322	0.7678757568271
0.6732212892093	0.7679705739847
0.6733143934439	0.7681559728298
0.6733999284813	0.7682494961419
0.6734726245078	0.7682598647185
0.6735420185509	0.7683482130598
0.6736341058772	0.7684288153052
0.6737093363549	0.7684352725512

0.6738026526886	0.7685268237528
0.673891996566	0.7686150867019
0.6740012756935	0.7687428372317
0.6741007974874	0.7688848620841
0.6741871081995	0.7689145085995
0.6742631098558	0.768948531134
0.6743430488467	0.7689984066346
0.6744319075761	0.7690035537518
0.674546431324	0.7692035497313
0.6746277558063	0.7692281427608
0.6747214656638	0.769429907929
0.6748032439258	0.7695739489234
0.6748861837188	0.7698320374262
0.6749740687217	0.7698609458799
0.6750407914687	0.7699304286738
0.6750970641807	0.769941766683
0.6751837225686	0.7699697655822
0.6752531899934	0.7699729958087
0.6753682709815	0.7700871079515
0.6754350407877	0.7703214304325
0.6754987709968	0.7703791253792
0.6755971473645	0.7706676640292
0.675702402268	0.7707461910242
0.675779708682	0.7707492261061
0.675883815987	0.7709789796539
0.6759963246095	0.771036994606
0.6760655558536	0.7710485394808
0.6761662724635	0.7711276505784
0.6762823298924	0.7711335118225
0.6763751055053	0.7712028313987
0.6764409759615	0.7713488156701
0.6765621467608	0.7714069049546
0.6766436607108	0.7714499711593
0.6767247794247	0.7714855120208
0.6768273621176	0.771669318582
0.6768962212931	0.7717349931636
0.6770063354328	0.7720407429977
0.6771042596321	0.7720570087053
0.6771867923436	0.7720957534099
0.6772765353624	0.7724784164365
0.6773536493842	0.7724997942732
0.6774236918806	0.7725195055095
0.6774916625992	0.7726552023204
0.6775703030887	0.7729559533379
0.6777082457794	0.7730558389705
0.6777909224491	0.7732554757751
0.6778567061694	0.7733870285174
0.6779602775694	0.7734701785934
0.6780618631896	0.7734922421166
0.6781737408488	0.7739305298316
0.6782357316219	0.7739714768517
0.6783243224534	0.7739802663649
0.6784158173073	0.7741403897498
0.678496199084	0.7742908180974

0.6785900057691	0.7744161720062
0.6786914547543	0.7746578417045
0.6788026385032	0.7747456576208
0.6788885320083	0.7747592804416
0.6789567838886	0.774794529067
0.6790445150146	0.774862762124
0.6791274074433	0.7749757725584
0.6792150556566	0.7752413750984
0.6792930047612	0.7752742556356
0.6793876109706	0.7753855894361
0.6794652564384	0.7755272521688
0.6795947415684	0.7759153753134
0.6796849228221	0.7760895460377
0.6797576271577	0.7760921610173
0.6798681885542	0.7762220919636
0.6799636957312	0.7763357932977
0.6800541226013	0.7764560499644
0.6801341004253	0.776632845439
0.6802086164324	0.7766438966327
0.6803379246148	0.7766847090819
0.6803925838862	0.7766917858804
0.6804729607731	0.7767639685417
0.6805472057181	0.776877448453
0.6806389232645	0.7769015890676
0.6807008412287	0.7769071509498
0.6807805857397	0.7769767733948
0.6808731166107	0.7772232738095
0.6809590946103	0.777423172833
0.6810615833867	0.7777496841896
0.6811461625641	0.7777546354209
0.6812439295797	0.7778888186203
0.6813398627707	0.7780708915834
0.6814446778409	0.7782588424176
0.6815250581704	0.7784177620403
0.6816036127337	0.7785510133463
0.6816862208025	0.7786015256694
0.6817584884659	0.778655165555
0.6818477818829	0.7787914058124
0.6819161172851	0.7788249213461
0.6819844116631	0.7788960007307
0.6820611165503	0.7792480882101
0.6821604011795	0.7792991323838
0.6822278331126	0.7793143607585
0.6823296638781	0.779415022017
0.6824143779665	0.7794707625542
0.6824879257508	0.7795867727458
0.6825565992168	0.779655035269
0.6826494703148	0.7796602403783
0.6827241728803	0.7797588654408
0.6828243425633	0.7798789483898
0.6829276973153	0.7800186935725
0.6830134992747	0.7800289428453
0.6830770812809	0.7800310357462
0.6831735000479	0.7800923935929

0.6832706593023	0.7800960124237
0.6833733033494	0.780273882264
0.6834640071889	0.7804245686575
0.683559144362	0.7804733459248
0.6836683133732	0.780602568383
0.6837494735153	0.7806107902284
0.6838334207495	0.7807250893178
0.6839033732212	0.7807396314877
0.6839970659061	0.7807494757957
0.6840982079052	0.7807547469877
0.6841981986355	0.780844233681
0.6842940528731	0.7809049198368
0.6843698597979	0.7810476400629
0.6844408554693	0.7810609327886
0.6845140916617	0.7811104628877
0.6846420174042	0.7814273764378
0.6847000031298	0.7814561743162
0.6847607801029	0.7814598859442
0.6848455924257	0.7814727753064
0.6849377349628	0.7815192567075
0.6850282714823	0.7815490114412
0.685110228774	0.7815910513607
0.6851865648576	0.781606926122
0.6852523515341	0.7816757671052
0.6853254014554	0.7817394130013
0.6854318306354	0.7820345582383
0.6855314619509	0.7820771660431
0.6856028022067	0.7821898174883
0.6856822376894	0.7822166995553
0.6857711385109	0.7822634145048
0.6858525148291	0.7824041461494
0.685970328028	0.7825203923136
0.6860583024781	0.7825868781626
0.6861612995766	0.7826490057424
0.6862484141336	0.7826593521133
0.6863277725527	0.7827452331624
0.6864437697894	0.7827848733351
0.6865582005784	0.7830139041883
0.6866368740545	0.7830349616601
0.6867158919018	0.7832006391581
0.6868015676005	0.7832611551938
0.6869066098074	0.7834895085961
0.6869833419531	0.7835677528809
0.6870533447161	0.7835818578922
0.6871508019116	0.7836310612716
0.6872482389563	0.7836842725506
0.687349221776	0.7837268551601
0.6874222351939	0.783731150358
0.6875098039799	0.7838209715238
0.6875951197535	0.7839597607647
0.6876908665962	0.7840035837551
0.6877730428172	0.7842265289387
0.687828737632	0.7842295556462
0.6879326948082	0.7842550924475

0.6880122299042	0.7843063206336
0.688076409114	0.7843721966324
0.6881599078885	0.78443763517
0.6882514306018	0.7847538665347
0.6883208292841	0.784890837205
0.6883789868752	0.7848945333629
0.6884501784208	0.7850014377587
0.6885414961644	0.7852539561026
0.6886257763012	0.785278667375
0.6886797006136	0.7853176353763
0.6887423064891	0.7853478417214
0.6888260092533	0.7854502390057
0.6889156711395	0.7854526578696
0.6889952559012	0.7855594242419
0.6890939058309	0.7855669065803
0.689160658379	0.78557700557
0.6892317516918	0.785599211633
0.6893142478222	0.7856021964396
0.6893894991445	0.7856765441005
0.6894833327285	0.7857211720356
0.6895750530487	0.7858230248302
0.6896759598869	0.7858405332039
0.6897455906231	0.7858534940656
0.689829403661	0.7859296199427
0.6899291419461	0.7859647106612
0.6900501737834	0.7859715385357
0.6901262126111	0.7860381308812
0.6902257467861	0.7862224013849
0.6903205673688	0.7862522285527
0.690382178323	0.7863271119605
0.6905070202123	0.7864641671728
0.6905768334384	0.7865728847486
0.6906546979031	0.7866273747206
0.6907554154027	0.7869873526052
0.6908358857759	0.7870148835654
0.690949549424	0.7874471413167
0.6910368371169	0.7874972577639
0.6911201507097	0.7875651816095
0.6911861169614	0.7875997311917
0.6912975107511	0.7879314397595
0.6914225775862	0.788020913481
0.6915214975701	0.7882788681604
0.6916106302566	0.7883263384374
0.6917164660891	0.7883712328963
0.6918249216063	0.7885869119314
0.691905346889	0.7886084369539
0.6919817936439	0.7886345591573
0.6920524049394	0.7889826816098
0.6921474228946	0.7891327233024
0.6922203835468	0.7891662889953
0.6923152106443	0.7892442136183
0.6924200744595	0.789290407226
0.6925082583594	0.7893750814213
0.6925949236238	0.7893812926354

0.6926903705763	0.7896475571536
0.6927680267755	0.7896685133186
0.6928556221196	0.7897040454365
0.6929659910673	0.7897422341206
0.693041699568	0.7898211030627
0.6931489246566	0.7900212725838
0.6932439102016	0.7903581912371
0.6933698967529	0.7906773961524
0.6934637962738	0.7907373828038
0.6935701550302	0.7907632686652
0.6936512745148	0.7908736607939
0.6937547588192	0.7909520321732
0.6938504102472	0.7911388176701
0.6939597430382	0.7911411632457
0.6940326439671	0.7912130884058
0.6941149599991	0.7912171892332
0.6942067128003	0.7912460389816
0.6942910356043	0.7912872139358
0.6943895762977	0.7912955723301
0.6945037950152	0.7913307624093
0.6945771481952	0.7915554353291
0.694647552344	0.7915656052325
0.6946971371309	0.7916048880836
0.6947839598382	0.7916903258975
0.6948718579327	0.7917307174189
0.6949701638318	0.7917581505438
0.695036991708	0.7917604613767
0.6951252589931	0.7917616653727
0.6951982403758	0.7917712719587
0.6952678315591	0.791819773297
0.6953560739164	0.7919101334275
0.6954325936637	0.7919612101435
0.6954979458469	0.7920066618503
0.6955716606414	0.7921180426487
0.6956494617006	0.7921681906919
0.695737979087	0.7922594113054
0.6958394204361	0.7923275537718
0.6959082887352	0.7923336783182
0.6959902517076	0.7923733218775
0.6960708724643	0.7923764692374
0.6961705213377	0.7925621750052
0.6962437208533	0.792605568152
0.6963258465913	0.7927906615501
0.6963838455829	0.7929600323641
0.6964655813093	0.7929908081711
0.6965479414108	0.7931247460033
0.6966260631542	0.793157661227
0.696742702776	0.7932671044547
0.696880321851	0.7936276405176
0.6969780057967	0.793732211529
0.6970549773352	0.793903242447
0.6971425641463	0.79392241179
0.6972348271125	0.7939808909665
0.6973560305623	0.7940095620077

0.6974176225629	0.7940561330653
0.6975014007787	0.7941335810641
0.6975805197522	0.7942316074761
0.6976551584524	0.7942767284994
0.6977390019092	0.7943589411628
0.6978127797051	0.7943741054238
0.6979046563516	0.7944420764994
0.6980283057239	0.7948594216254
0.6980961625368	0.7948782882541
0.698170426835	0.7948842605998
0.6983002668484	0.7950297988237
0.6983971091376	0.7950730635272
0.6984881023886	0.795215456605
0.6985535061216	0.795258338002
0.6986528039257	0.7953167951431
0.6987633333544	0.7954465891714
0.6988220584859	0.7954676516133
0.6988711085378	0.7955246540252
0.6989910694604	0.7957311048458
0.6990943433341	0.7959179212741
0.6991473977786	0.7959307469116
0.6992531981303	0.7959456733042
0.6993206408876	0.7959516156093
0.6993687126652	0.7959744338214
0.6994458756122	0.7961287129631
0.6995271495386	0.7962653567469
0.6995917738221	0.7963138455158
0.699650233798	0.7963166971795
0.6997316799147	0.7963869222957
0.6998064659447	0.7965752574091
0.6999206105919	0.7966092185146
0.6999887531565	0.7966764837098
0.7000699892399	0.7968928063747
0.7001403940785	0.7969823633614
0.7002229506498	0.7971346411027
0.70031683421	0.7972163164602
0.7004140294644	0.797284992529
0.7005289956869	0.7974996997909
0.700613875345	0.79772105122
0.7007105357902	0.7977457992544
0.7008116579545	0.7978530667813
0.7008865678928	0.7978647885528
0.7009391785048	0.7979467910291
0.7010241928571	0.7981970962071
0.7011146854837	0.7983304043932
0.7011748760014	0.7984293350357
0.7012607780806	0.7985297603711
0.7013616016936	0.7985891621367
0.701432798885	0.7987051338211
0.7015137058005	0.7987442727882
0.7016204268799	0.7988612605891
0.70173255264	0.7990004872203
0.7018165154994	0.7990384176161
0.7018894580488	0.7991427662109

0.701973493873	0.7991756045597
0.7020745674483	0.7995123554189
0.7021572069546	0.7995303412606
0.7022265189562	0.799611088731
0.7023220653865	0.7996725741117
0.7023933147446	0.7996792651019
0.7024801246699	0.7997824972535
0.7025674424922	0.8000141291954
0.7026774980784	0.8002372072697
0.7027854355996	0.8004760504397
0.7028655533091	0.8004848740259
0.7029480944421	0.8005236063904
0.7030604508121	0.8007604848285
0.7031434583808	0.8009568071464
0.7032402233747	0.8010787450372
0.7033100555903	0.8011735562025
0.7033852705354	0.8013124102132
0.7034648790926	0.8015615571262
0.7035314395759	0.8015975520254
0.7036246181465	0.8017005405541
0.7036870572284	0.8017032951881
0.7037590903835	0.8018181074453
0.7038281452218	0.8019490985183
0.7039298942486	0.8020705026303
0.7040073092757	0.802348451074
0.7040732863762	0.8023784529416
0.7041747889954	0.8024229938372
0.7042586254025	0.8024447648797
0.7043312241854	0.8024634712583
0.7043976783969	0.8024941116902
0.7045022957384	0.8027857689284
0.7045741876838	0.8027927289074
0.7046236742691	0.8028749720581
0.704716594973	0.803032014272
0.7047900689624	0.8031695825719
0.7049059449681	0.8033887527353
0.7049776402993	0.8034146981512
0.7050513963066	0.8035198407331
0.7051423630871	0.8036047341087
0.7052432958032	0.8036739327287
0.7053391322985	0.8039126893899
0.7054144641155	0.803961994559
0.7054864862393	0.8039727902136
0.7055673056129	0.8040656002724
0.705683723035	0.8042883976813
0.705763399738	0.8043322517944
0.7058061337789	0.8044025976909
0.7058704800312	0.8044403389662
0.7059726162882	0.8045042436729
0.706065424582	0.804565347403
0.7061720097089	0.8046173302723
0.7062479134894	0.8046221192467
0.7063375092727	0.8047599229478
0.7064213492104	0.805028035831

0.7065115152984	0.8050780161661
0.7066030201721	0.805086198303
0.7067006259016	0.8051416783562
0.7067907769607	0.8052008620491
0.7068988156024	0.8053119830032
0.7070140283766	0.8053700715824
0.7071376184855	0.8058175248256
0.707223012174	0.8059194503348
0.7073055749362	0.805954649626
0.7073736519315	0.8062248243846
0.7074690626008	0.8064626410984
0.7075441009949	0.8065064636724
0.7076887190827	0.8068799471175
0.7078098364075	0.8069444183276
0.7079000774202	0.8069828906247
0.7079967974761	0.807125555867
0.7080834728766	0.8071319543943
0.7081774105821	0.8072609460077
0.7082708512752	0.807284040147
0.7083514845327	0.8073136428057
0.7084408010946	0.8073896213208
0.7085094367812	0.8074074692612
0.7085658183058	0.8074171432507
0.7086870045611	0.8078442908639
0.7087805140448	0.8080122269759
0.7088729753192	0.8082338587545
0.708968470629	0.8083832004848
0.7090661077998	0.8085226585586
0.7091513996	0.8085582610852
0.7092240627898	0.80864133133
0.7093033184268	0.8086611946964
0.7093592517554	0.8088030008274
0.7094433863819	0.8088131521031
0.7095488545216	0.8088205604642
0.7096182244199	0.8088705685108
0.709663633009	0.8088971569742
0.7097486661468	0.8090671549958
0.7098001284775	0.8090728277715
0.7098721406172	0.8090760161651
0.7099442378433	0.8092981495772
0.7100260276621	0.8094694336053
0.7101162874147	0.8095002267664
0.7101700160634	0.8095178735735
0.7102672883876	0.8095628408979
0.710330089503	0.8096060527924
0.7104431890971	0.8096740361689
0.7105329071846	0.8098382167554
0.7106448206268	0.8099126836276
0.710750701097	0.8099301268192
0.7108297315659	0.8101391045098
0.7109171691393	0.8103079087454
0.711006534279	0.81058517697
0.7110972533517	0.8106486709353
0.7112042905384	0.8106671123341

0.7112776607375	0.8107925309243
0.7113753302223	0.8108586085178
0.7114323568631	0.8109751816063
0.7115035268101	0.8110499188353
0.711591949457	0.811160541292
0.7116940904991	0.8111640239072
0.7117786873647	0.8111900359753
0.7118452516803	0.8112262763571
0.7119343338779	0.8112317476535
0.7120278134518	0.8112431438202
0.7121160610015	0.811263323768
0.7122099416967	0.8116515595833
0.7122947924656	0.8117547176225
0.7123806135307	0.8117745415245
0.7124932607207	0.8119385131221
0.7125618927047	0.8119560753545
0.7126822918289	0.8120391062264
0.7127533392126	0.8121667897789
0.7128273490646	0.8122341858266
0.7129328319066	0.8122898322234
0.7129933752848	0.8123300431659
0.7130862426593	0.8123415793324
0.7131780050198	0.8124825773476
0.7132529760728	0.8125468615806
0.7133543942807	0.8127026059293
0.7134462227335	0.812744943502
0.7135228090669	0.8127820976879
0.7136173656286	0.8128704831424
0.7137233698796	0.8130350350449
0.713778549954	0.8130443760502
0.7138714129201	0.8131746558952
0.7139729421174	0.8132122327528
0.7140297600294	0.8133188815485
0.7141203887067	0.8133193151373
0.7141743517885	0.8133545193694
0.7142853468788	0.8135962527769
0.7143824767186	0.8137328818118
0.714458829795	0.8138217200672
0.7145457842374	0.8138324383383
0.7146475235688	0.8138845729086
0.7147210987034	0.8139202739058
0.7147904720755	0.8139631611275
0.7148914447955	0.8140057407134
0.7150157421051	0.8140951183847
0.7151002340921	0.8141388097825
0.7152023924282	0.8142711888877
0.7152823685241	0.8143062227026
0.7153621624104	0.8146829497741
0.7154507012891	0.8147428057419
0.7155358522277	0.8148372915522
0.7156290478061	0.8151482222129
0.7157341163502	0.8152111859153
0.7157911083893	0.815362747431
0.7158798757097	0.8153744290834

0.7159407006747	0.8155747873322
0.7160079127601	0.8155908438896
0.7160820594078	0.8157075530128
0.7161739939717	0.8157854840736
0.7162495778769	0.8158060720192
0.7163554521337	0.8161772233655
0.7164129132574	0.8162109290041
0.7164702098753	0.8162311268449
0.7165576739211	0.8163372397522
0.7166251761247	0.8163647255721
0.7167214546631	0.8164631973656
0.7167760100081	0.8164674639022
0.7168563364108	0.8165050330814
0.7169584865332	0.8166790168997
0.7170665704138	0.816755888065
0.7171583588264	0.8170892696483
0.7172498432656	0.8172058573719
0.7173083988708	0.8172157086607
0.7173824588789	0.8174154666185
0.7174617078935	0.8175550914085
0.7175645773584	0.8176539704597
0.7176401967883	0.8176811746483
0.7177110548003	0.8177451928676
0.7178258111913	0.8179034112571
0.7179137519463	0.8179227852658
0.7180207160588	0.8179429801173
0.7181308927141	0.8182372082402
0.7182124713259	0.8184045136001
0.7183250268074	0.8187979178236
0.7184261364335	0.8189053631003
0.7185025483642	0.8190436334778
0.7185543596406	0.8190826960617
0.7186448672658	0.81912978188
0.7187322064256	0.8192351912808
0.7188150334205	0.819397981317
0.7189320206283	0.8196086566947
0.7190238075711	0.8196223031422
0.7191058567251	0.8197301833729
0.7191808193564	0.8198246344474
0.7192740762757	0.819840895544
0.719368338214	0.8198545063453
0.7194662882224	0.8198985900205
0.7195487881402	0.8200287298407
0.7196190402499	0.8200541431682
0.7196990944542	0.8203868700126
0.7197713796937	0.8204883512361
0.7198729480201	0.8207646839191
0.7199235755601	0.8207961598371
0.7200299054107	0.8208114174121
0.7201231250868	0.820848625029
0.7202214794685	0.8209020077267
0.7202911639157	0.8211922216287
0.7203848385497	0.8212170909204
0.7204868693233	0.8215270400177

0.7205622208599	0.8215527779247
0.7206280415777	0.8215705750507
0.7207228871878	0.8216676133936
0.7208255624218	0.8219500604545
0.7208956389201	0.8219568527849
0.7209893627757	0.8220734799917
0.7211022029547	0.8221262464745
0.7212293428564	0.8221430657112
0.7213066943929	0.8222815771232
0.7213901960105	0.8223262661412
0.721487040266	0.8223557367558
0.7215832796646	0.822501344322
0.721702360919	0.8225639768005
0.7218073008629	0.8225670292386
0.7218851023431	0.8226071515456
0.7219713811538	0.8226212499933
0.7220438366442	0.8226600265013
0.7221327742731	0.8227563722127
0.7222378146165	0.8228821274911
0.7223234830006	0.8230432781536
0.7224177451065	0.823086751528
0.7225185153173	0.8232193509833
0.7225749948365	0.8232296992293
0.7226762101468	0.8233624816102
0.7227379514552	0.8234727812359
0.7228104459147	0.823622199726
0.7228910166662	0.8237831629546
0.7229824177032	0.8237859116808
0.7230326452409	0.8237932166326
0.7230980866825	0.8238510877502
0.7231996266786	0.8240380763691
0.7232857811081	0.8240554192557
0.723362044959	0.8241443018989
0.7234267925658	0.8241915321339
0.7234969448209	0.8242895412859
0.7235613243351	0.8243667185397
0.723629179621	0.8244037547568
0.7237242205467	0.8244525620774
0.7237946038102	0.82447148561
0.7238636388734	0.8245072569928
0.7239328509411	0.8245104106068
0.7240042175476	0.824540648859
0.724077743228	0.824660725388
0.724140176849	0.8246658338008
0.7242347182373	0.8246853918863
0.7243191273989	0.8247493038505
0.7244058168513	0.8248003698779
0.7245140589405	0.8248307596688
0.7245761521862	0.8248722599626
0.7246842901629	0.8251613128266
0.7247748163762	0.8253321703558
0.7248555238735	0.8256226085958
0.7249374207193	0.8257586642726
0.725035465407	0.8257849765937

0.7251068846568	0.8257898941323
0.7252128200855	0.8260410040201
0.7252904770977	0.8261232810747
0.7253600804345	0.8262856860857
0.7254423192813	0.8264474144096
0.7255413705321	0.826470001805
0.7255847364152	0.8264825121319
0.7256582411396	0.8266033156632
0.7257435882863	0.8267331255711
0.7258445677425	0.8267789098088
0.7259605782106	0.8268358760501
0.7260593557611	0.826928721927
0.7261410169114	0.8270349140529
0.7262318636917	0.8271435315285
0.7263307393628	0.8272716611085
0.7264209392425	0.8273748705117
0.7264891762483	0.8274638992984
0.7265514631963	0.8274978468355
0.7266408214797	0.8275486094087
0.7267101273962	0.8275577868758
0.726794702413	0.8276367210342
0.7268780706159	0.8276889378741
0.7269607414671	0.8276934471026
0.7270507561958	0.8279554368689
0.7271253792893	0.8279671627459
0.7272365500789	0.8280760164582
0.7272998559318	0.8280931431673
0.7273777389421	0.8280943302977
0.7274454149435	0.8281943976413
0.7275597971155	0.8282612826189
0.7276309444155	0.828403988007
0.7276825229393	0.828517963462
0.727765657855	0.8285822143694
0.7278336611008	0.8286391060145
0.7279135918743	0.8287115598951
0.7280023705335	0.8289389546954
0.7280654872678	0.8290308238276
0.7281803933404	0.8292803089904
0.7282672037115	0.8294014817314
0.7283232551359	0.829420269561
0.7284276575409	0.8294685391398
0.7285297513314	0.8298254121049
0.7285953225225	0.8298475095174
0.7286759069041	0.8299722447957
0.7287608946735	0.8300363847997
0.7288330514041	0.8300666715613
0.7289190251756	0.8301024020423
0.7290173776843	0.8302268787604
0.7290999050465	0.8303678802673
0.7292112845554	0.8305692936963
0.7293447549919	0.8307858188862
0.7294314406423	0.8310085744833
0.7295274763609	0.8310497400667
0.7296341869975	0.8311256169411

0.7297172962298	0.8313380366427
0.7298007827985	0.8313432603987
0.7298717166962	0.8314151223412
0.729968882565	0.8315598474406
0.7300340497951	0.8316351651079
0.7300979585004	0.831701617417
0.7301675801584	0.8317211575654
0.730276179437	0.8318670531955
0.7303473153603	0.8319087773918
0.7304298031316	0.831964390922
0.7305146179635	0.8319747214866
0.7305839070199	0.8320174669205
0.730652017893	0.8322243020179
0.7307243757201	0.8322776716239
0.7308418676484	0.8324852988966
0.7309431969528	0.8325366129708
0.7310244292015	0.8327237056457
0.7311089058025	0.8327747586419
0.7311947965388	0.8327822318039
0.731268880644	0.832807091518
0.7313274430481	0.8328307751845
0.7314084614933	0.8331122545193
0.7314738098489	0.8331879676676
0.7315715177354	0.8332456945511
0.73164668187	0.833358932312
0.7317503494426	0.8334315101558
0.7318235443845	0.8335040156335
0.7318721129887	0.8335228554598
0.7319642592229	0.8335538628279
0.732065520613	0.8337094369475
0.732133776412	0.8338744923741
0.7322396249244	0.8339194822486
0.7323087649214	0.8339986137661
0.7323768390612	0.8340647092781
0.732452922422	0.8341434103967
0.7325229830421	0.834158071674
0.7325910188273	0.8341635437541
0.7327028761185	0.834186376155
0.7327870514948	0.8341888675988
0.7328835546703	0.8343214619818
0.7329715525381	0.8344441089834
0.7330835174898	0.8347062384207
0.7331939136644	0.8350015509794
0.7332702236513	0.8352536137379
0.7333576218997	0.8355033928916
0.7334470380272	0.8356582658424
0.733513271147	0.8357997666036
0.733585374984	0.8358429159253
0.7336753170136	0.8359997102001
0.733739643264	0.8360274238556
0.7338459512742	0.8363534009207
0.7339201950129	0.8363583685242
0.7340252492042	0.8363984426491
0.7341165322388	0.836576948766

0.7342025186296	0.8367331535052
0.7343050694323	0.8367881235295
0.7343748430297	0.8369298230085
0.7344906502	0.8369841058873
0.7345624233692	0.8369869482896
0.7346541288694	0.837046700421
0.7347338539936	0.8371185914455
0.734812657818	0.8372076587748
0.7348972796598	0.8373037416789
0.734970797187	0.8375498261092
0.7350753705033	0.8375844955454
0.735187439275	0.8378073061898
0.7352609932631	0.8378164717724
0.7353346932172	0.8379657072026
0.7354502610542	0.8380688913283
0.7355621837446	0.8382690068391
0.7356596141107	0.8382706776653
0.7357734631574	0.838325104025
0.7358695923581	0.8385685414785
0.73594334953	0.8386028977379
0.7360534668214	0.8389453690655
0.7361686313709	0.839068995436
0.7362481555363	0.8391047334679
0.7363858344197	0.8391504268683
0.7365042462761	0.8396847237482
0.7365680894797	0.8397336050444
0.7366382031722	0.8399917842817
0.736730610172	0.8400528117297
0.7367946182202	0.8400983279354
0.7368712100376	0.8401629615703
0.7369353767294	0.8402153197708
0.7370259077941	0.8405004631351
0.7370808523941	0.840508703112
0.7371703598662	0.8405443675572
0.7372356810941	0.8405511521083
0.7373025640731	0.8405772354952
0.737378924631	0.8406380210024
0.7374486945528	0.8409066672668
0.7375766747478	0.8409334732738
0.737646108662	0.8409457718014
0.7377678114475	0.8412548106878
0.7378843848654	0.841274894052
0.7379528421652	0.8413198768133
0.7380206244975	0.8413735896572
0.738097237428	0.8414336563146
0.7381914961462	0.8416276149986
0.7383170991579	0.8416750589782
0.7384225992776	0.8417233244979
0.7384954729675	0.8417704762902
0.7385985969689	0.8420933115096
0.7386719107978	0.8421466888514
0.7387559412182	0.8421478350917
0.7387985683954	0.8421662122901
0.7388945371462	0.8422670337905

0.7389954984725	0.8423177369738
0.7390844650978	0.8423445718942
0.739204053057	0.8424803809311
0.7393334041268	0.8429771029647
0.7394094239213	0.8430432025846
0.7394937067362	0.8430777042737
0.7395747655164	0.84310335212
0.7396752047466	0.8431301338419
0.7397887244949	0.8432122744932
0.7398810248619	0.8433657751707
0.7399479301822	0.8434909815658
0.7400403215849	0.8435177368779
0.7401390351531	0.8435751047931
0.7402340939226	0.8436878492606
0.7403080910666	0.8436897724
0.7404095790307	0.8437204257855
0.7405135899213	0.8438097360042
0.7405918169254	0.8438596828484
0.7407365550405	0.8444506550905
0.7408298169287	0.844591223446
0.7408980140969	0.8445996160482
0.7409720180016	0.8446197435598
0.7410769268776	0.8446510858298
0.7411928324192	0.8447881259105
0.7412760838963	0.8448091635549
0.7413460364353	0.8448430978484
0.7414641872187	0.8450822336102
0.7415519023234	0.8451463319084
0.741658300146	0.8451645379376
0.741740383193	0.8452425939389
0.7418005552005	0.8452851486123
0.7418760118473	0.8453413993279
0.741980130444	0.8456614333419
0.7421050869619	0.8458176256808
0.7421732168387	0.845876493246
0.7422598700382	0.8459526578493
0.7423601498185	0.8459657239318
0.7424339027959	0.84606915838
0.7425516004534	0.8462063648429
0.7426566682677	0.8462343390868
0.7427626676268	0.8463289167021
0.7428216915435	0.8463304078332
0.7429141613075	0.8464031371022
0.7430084164993	0.8465549290227
0.7431024010827	0.8466398980817
0.7431894467414	0.8468278352321
0.7432794945213	0.8469427886349
0.7433512568271	0.8470247728269
0.7434326626788	0.8470766300162
0.7434989183852	0.8473159120477
0.7435904833361	0.8473837576879
0.7437001793078	0.8473962279661
0.7437808799415	0.8474129013125
0.7439065873195	0.8475564791754

0.7440066117462	0.8478779793878
0.7440881613566	0.8479552408994
0.7441968434907	0.8480298104157
0.7442745587728	0.8480873857852
0.7443788052329	0.8481157516956
0.7445072897491	0.8481517303428
0.7445955795023	0.8482517828823
0.7446967441206	0.8483127121644
0.7447861472002	0.8486147313065
0.7448912833868	0.8487300325823
0.745012530404	0.8490102634766
0.7450981517696	0.8491600111414
0.7451838358791	0.849297380956
0.7453006772416	0.8494339024918
0.7453846438257	0.8495504235784
0.7454939988534	0.8498413193931
0.7455927161046	0.8500352135911
0.7456664160861	0.8501921530075
0.7457502843173	0.8502618574215
0.7458519564909	0.8503257005588
0.7459347546014	0.8507011299649
0.7460038281477	0.850739127649
0.7460998033952	0.85082863486
0.7461682310635	0.850876624744
0.7462372265598	0.851031088424
0.7463436907639	0.8511736269815
0.7464584144999	0.8518061310239
0.7465611788424	0.8523371459685
0.746646968881	0.8523765738702
0.7467610901761	0.8525820725685
0.7468635492863	0.8526328818934
0.7469467305283	0.8528523366351
0.7470595054174	0.8529611576008
0.7471431515967	0.8530395173428
0.747235217688	0.8530978837833
0.747316775429	0.8531155327632
0.7473975877244	0.8531765346193
0.7474913782197	0.8537936435427
0.7476013451508	0.853935166747
0.7476797537491	0.854172566748
0.7477601559219	0.8541837361215
0.7478986663507	0.8543288856095
0.7480178810946	0.8544576187786
0.7480865388099	0.8545413308799
0.7481686125881	0.854637823209
0.7482407745601	0.8547200065731
0.7483519083897	0.8547569103888
0.7484176475909	0.8547801545384
0.7484951429075	0.8548895454755
0.7485716895673	0.8550087382333
0.7486721748753	0.8550388140707
0.7487793403392	0.8551245628065
0.7488654942723	0.8552862266324
0.7489681998139	0.8553130265988

0.7490862146425	0.8554652082196
0.7491359018013	0.8554708079889
0.7492153383576	0.855486444623
0.7493003499526	0.8557473812712
0.7493643248672	0.8557568566358
0.7494354486993	0.8557847928501
0.7495092799987	0.8558385379592
0.7495905655695	0.8559395896529
0.7497234898447	0.856667630716
0.7498133235408	0.8567982186434
0.74989011046	0.8568846252612
0.749965546556	0.856889402506
0.7500646571634	0.8569913102709
0.7501761016652	0.8570378792119
0.750260173626	0.8573199694385
0.7503647679835	0.8577208108479
0.7504866479471	0.8584250217956
0.7505742925533	0.8585284942985
0.750667220526	0.8586735515753
0.750784948639	0.8588422537983
0.7508356678903	0.8588428086858
0.7509073220944	0.8588715734762
0.7509767889013	0.8590438933484
0.7510484713936	0.8591520654299
0.7511087999066	0.8591620942224
0.7512420894303	0.8593668880695
0.7513059277223	0.8593761436379
0.7513961823908	0.8593880406995
0.7515443733863	0.8594354477536
0.7516481592729	0.8594727065254
0.7517204565868	0.8595188598761
0.7518171211473	0.8596846634038
0.7518903390343	0.859689778835
0.7519721947062	0.8597709429581
0.7520491748582	0.8597887277493
0.7521304903117	0.859881670917
0.7522172698408	0.8599517120468
0.7523202971176	0.8601090316776
0.7524234899069	0.8602674168293
0.7525262915824	0.8603543919887
0.7526017969192	0.8604166502121
0.752665982501	0.8604428477087
0.7527619227629	0.8605813953203
0.7528596374956	0.8606272002182
0.7529444239378	0.8606373885447
0.7530321412552	0.8606396945804
0.7531154543445	0.8607497093541
0.7532032193616	0.8608506151606
0.7533415705842	0.8610975833289
0.7534222708246	0.8613388314669
0.7535327473848	0.8614278049919
0.753625815045	0.8618915763985
0.7536979936465	0.8619464410722
0.7538102427121	0.8620245636532

0.7538974935977	0.8621014491337
0.7539823809539	0.8621720560986
0.7540835063235	0.8622434238773
0.7541677696379	0.8624267540572
0.7542719058474	0.8626114883319
0.7543399911478	0.8627127121503
0.7544398755196	0.8627671557807
0.7545176614093	0.862843558836
0.7545945344926	0.8629269221163
0.7546668755292	0.8630318789802
0.7547381592524	0.8630777641098
0.7548523024354	0.8630889256999
0.7549443740597	0.8631164352071
0.7550233632655	0.8632550964716
0.755115199199	0.8635484415996
0.7552108575014	0.8635595448945
0.7552867059024	0.8636856204925
0.7553753003683	0.8637708995457
0.7554966228556	0.8641462748542
0.7555690512532	0.8641935303229
0.7556551837166	0.8642687932075
0.7557511211158	0.8642801718504
0.7558172663624	0.8642873254193
0.755916064212	0.8644361670845
0.7560136931785	0.8645545369845
0.7561135114265	0.8647097768699
0.7561958630627	0.8647885363462
0.7562549263349	0.8647959722933
0.7563404512354	0.8648430406091
0.7564279272309	0.864964930935
0.7565019601274	0.8649711137802
0.7566062485122	0.8651016059791
0.7566876285075	0.8651117765865
0.7567861066944	0.8654603798615
0.7568963262003	0.8656832039729
0.7569695661665	0.8657479351112
0.7570517413815	0.8657521675672
0.7571435898163	0.8657850498028
0.7572581859719	0.8658150407106
0.7573428960311	0.8658420363235
0.7574641784853	0.8662901654786
0.7575362321078	0.8663699264561
0.7576209154245	0.8668434258475
0.7577244634881	0.8668726115585
0.757820414434	0.8671656979913
0.7579006618962	0.8672026803232
0.7580123181548	0.8672541580457
0.7580770953391	0.8672798150105
0.7581782831377	0.8673595742981
0.7582548482654	0.8673630489398
0.7583375496271	0.8674074286046
0.7584432921059	0.8674706878739
0.758511426204	0.8674803045668
0.7585792285213	0.8675667699784

0.7586740998139	0.8677240084203
0.758778700838	0.8678648049805
0.7588539338371	0.8679025232482
0.7589339674973	0.8679142205324
0.7590074019199	0.867950973221
0.759111080899	0.8680114130758
0.7592029838009	0.8680677237881
0.7593251205957	0.8681789496916
0.7594387465527	0.8682352791261
0.7594948932622	0.8682969152314
0.7595612663663	0.8683189343037
0.759633251601	0.8683488614648
0.7597445650582	0.8683680623692
0.7598253019364	0.8685948104367
0.7599209864455	0.8686810660585
0.7600071346214	0.8688757038811
0.7601190421097	0.868986950061
0.7601932552754	0.8691274631853
0.7602986073739	0.869178901348
0.7603674399413	0.8692111779083
0.7604535587422	0.8693743039799
0.7605624571451	0.8694028242448
0.760673285753	0.869512407534
0.7607872909451	0.8695476219352
0.7608996175533	0.8698118755246
0.7609884533972	0.8698569742144
0.7610913453509	0.8699464216292
0.7611592169266	0.8700207257946
0.7612504797281	0.8700898559446
0.7613538627639	0.8701535812048
0.7614327947475	0.8702667177225
0.7615256240888	0.870282895423
0.7616233539122	0.8703563806193
0.7617081866532	0.8703627831865
0.7618105115916	0.8704365370265
0.7619000584973	0.8704853454091
0.7619856666704	0.8705313657032
0.7620805142743	0.8705317521558
0.7621945411976	0.8706408153512
0.762280615824	0.8706802543016
0.7623649549192	0.8706969769459
0.7624452775412	0.8707388645207
0.7625321955177	0.8708015813734
0.7626087400333	0.8708154573593
0.7627218814734	0.8708742302378
0.7627807903092	0.8709871587775
0.7628704316548	0.8711263648592
0.7629710992041	0.8711808118963
0.7631178731851	0.8715429321701
0.7631876581558	0.8716863413006
0.7632645842974	0.8717441553547
0.7633355661972	0.8717775899827
0.7634363053323	0.8717900811217
0.7635212356788	0.8718778442448

0.7636427300122	0.871907671242
0.7637386710419	0.8720044332582
0.7638435339351	0.8720375354948
0.7639223113049	0.8720676120951
0.7640036711733	0.8721127772705
0.7641040698423	0.8721845763551
0.7641908673177	0.8721870676996
0.7642656201486	0.8722278454462
0.7643492934562	0.8722647752818
0.7644514614956	0.8723464183239
0.7645490068131	0.8723742342225
0.7646938847266	0.8725227773611
0.7647723701695	0.8725681714972
0.7648444895148	0.8725752401362
0.764899787719	0.8725914996425
0.7649957010902	0.8726449909182
0.7651000711212	0.8726508614477
0.7651631493884	0.8727763422297
0.7652589733985	0.8728664708382
0.7653435157157	0.8729135317888
0.7654512273832	0.8731472074557
0.7655576397402	0.8734987864473
0.765633754508	0.8735708823853
0.7657159615782	0.8736067001982
0.7658063611023	0.8739554256857
0.7658865350719	0.8739917466133
0.7659774387034	0.8740446341583
0.7660696835759	0.8741261731093
0.766168781653	0.8742400625936
0.7662820776454	0.8742861853971
0.766388554512	0.8744127021917
0.7664725721746	0.8744423061542
0.7665529837682	0.8744632784727
0.7666335240993	0.8745937414596
0.766714052515	0.8747390219558
0.7667732715025	0.8747557732493
0.7668876581075	0.8748295581569
0.7669760624573	0.8748386103448
0.7670464009449	0.8748463062555
0.7671062946479	0.8748510836765
0.7672105406792	0.8752668418549
0.7673105643391	0.8752949215855
0.7674029720294	0.8756088100145
0.7674585921168	0.8756218377586
0.767567027523	0.8757248442334
0.7676804912726	0.8759454369652
0.7677476693787	0.8760105026794
0.7678517073842	0.8761064646569
0.7679546843026	0.8762340253865
0.7680476533015	0.8762704205646
0.7681276979791	0.8763620753801
0.7682138075429	0.8764292508236
0.7683096568899	0.8764513024097
0.7684119099586	0.8765209103797

0.7685200902891	0.8766170792642
0.76861028589	0.8766786411477
0.7687338955302	0.8767774811919
0.768845456892	0.8769361457689
0.7689638318202	0.8770914642574
0.769025421772	0.8771335206869
0.76912826058	0.8772015015214
0.7692593106955	0.8772604178372
0.7693839014955	0.8773230084222
0.7694831258513	0.8773817360002
0.7695809236007	0.8774182194956
0.7696549711592	0.8775178539476
0.7697390392189	0.8775219183062
0.7698395731118	0.8775901810812
0.7699186900212	0.8776025029348
0.7700044102303	0.8776251054044
0.7700856818795	0.877683802857
0.7701766944756	0.8777365960686
0.7702695859734	0.8778706841491
0.7703636486588	0.8779130810865
0.7704404475093	0.8780522434617
0.7705051274019	0.8781023171649
0.7705837675249	0.8781233699959
0.7706964119064	0.878174462055
0.7707695902334	0.8781900602236
0.7708712937157	0.8785679509126
0.7709627683072	0.8787293478872
0.7710610914251	0.8787944909923
0.7711513830883	0.8789270778849
0.771256072503	0.8789875379041
0.7713225804943	0.8792002396609
0.7714214362461	0.8795018509052
0.7715095688451	0.8796601098259
0.7715939890011	0.8797737430162
0.771703263196	0.8799115618518
0.7718068043347	0.880033795979
0.7719040821489	0.8800439699744
0.771981323905	0.8800483626799
0.7720738487765	0.880104955467
0.7721392691822	0.8801112389954
0.7722061876172	0.8801150246467
0.7723252003173	0.8801720140984
0.772383536603	0.8802219359253
0.7724684135481	0.8802497382738
0.7725517868229	0.8804127930427
0.7726411527356	0.8806139802455
0.7727122308309	0.8806688676545
0.7728281488817	0.8807225621193
0.7729290555097	0.8811253194634
0.7730465390878	0.8816010598367
0.7731500401395	0.881601829
0.7732554084607	0.8816030944648
0.7733345673715	0.8818035224828
0.773405967721	0.881928417935

0.773506615224	0.8820245824592
0.773587291578	0.8820314398179
0.7736742596179	0.8820643522978
0.7737921860749	0.8821761633808
0.7738933235642	0.8823600105478
0.7739727945398	0.8824386017669
0.7740681525156	0.8824808046268
0.7741174923213	0.8825100897965
0.7742070947541	0.8825655018979
0.7742904465322	0.8826483358394
0.774381499457	0.8827025243238
0.7744623886858	0.8827878621025
0.7745366003174	0.8828551101096
0.7746445290319	0.8828928841524
0.774731869347	0.8829276475513
0.7748346232505	0.8834715821821
0.7749403786818	0.8835674265975
0.7750416346407	0.8838664339099
0.7751257728877	0.8839051138704
0.7751884361979	0.8840014055763
0.7752688271431	0.8840074349583
0.7753628725468	0.88415461671
0.7754667746199	0.8843035219501
0.7755475643266	0.8843250627409
0.7756250189605	0.8843307680605
0.7756916088377	0.8843826589439
0.7758045488357	0.8848898324979
0.7758793870136	0.8849196526625
0.7759751282745	0.884937108636
0.7760590936958	0.8849610747536
0.7761463014076	0.8849930010881
0.7762262756658	0.8850658526143
0.7763545158038	0.8850840938317
0.7764250758751	0.8851524387575
0.7765100906966	0.8852522463758
0.7766234662855	0.8852894992807
0.7767000605709	0.8853111721413
0.7767657967749	0.8853627328546
0.7768479552142	0.8854454692358
0.7769855197184	0.8859059207011
0.7770974305918	0.8859351148419
0.7771753305485	0.8860235748021
0.7772604824302	0.8860263911049
0.7773595680349	0.8861299390888
0.7774483673761	0.886284975152
0.7775307307868	0.886474055043
0.7776269708917	0.8865186635271
0.7777272348588	0.8866039897376
0.7778043889433	0.8866987117268
0.7779059173817	0.8867128179157
0.7779938973923	0.8868276978981
0.7780752948308	0.8868368924924
0.7781488129931	0.8869907981108
0.7782687734579	0.8870060269145

0.7783689534833	0.8871437608075
0.77847344058	0.8872235794861
0.7785583361243	0.8872800967986
0.7786605742973	0.8872818740802
0.7787245779536	0.8873275431724
0.7788429027888	0.8873919114186
0.7789346764251	0.8873977077611
0.7790206304177	0.8874994690017
0.7791612505784	0.887709106781
0.7792308435546	0.8877116272252
0.7793125017388	0.8877220980461
0.7794121477739	0.887998296137
0.7795477774501	0.8880135502517
0.779640664945	0.8880418287193
0.7797545372244	0.8880581786331
0.7798502952584	0.8882465467097
0.7799280830006	0.8882905741997
0.7800232656615	0.8882938823975
0.7801252993566	0.8883249981564
0.7802162214215	0.8884143510812
0.7803042062846	0.8885222379139
0.7803772036592	0.8886114741344
0.7804405189792	0.8886274925227
0.7805117768362	0.8890266773349
0.7805874312921	0.889037777044
0.7806932707633	0.8891399499695
0.7807662593283	0.8892107341982
0.7808611105927	0.8893762292085
0.7809285401723	0.8893867045085
0.781021619072	0.8894904787552
0.7811048293578	0.8895033400045
0.7812294970016	0.8897510740726
0.7812995270224	0.8897910977623
0.781410965862	0.8898182223995
0.7815617637352	0.8898857859497
0.7816969338825	0.8900531679448
0.7817876872513	0.8901832028214
0.7818737400647	0.8902203987076
0.7819673441718	0.890342868313
0.7820603977843	0.8905493385388
0.7821542201769	0.8906206127898
0.7822806368942	0.890723919591
0.7823588059333	0.8908928664053
0.7824551412171	0.8909449223887
0.7825365163399	0.8912902258471
0.7826354792408	0.8913157401162
0.7827129822679	0.8913792199325
0.7827920874636	0.8914762216155
0.7829101128807	0.8918390691106
0.7829718782759	0.8920537693584
0.7830692737563	0.8923119332869
0.7831574190194	0.8923903633024
0.7832407967837	0.8925509747781
0.7833110937315	0.8926727004799

0.7833997056062	0.8927494378723
0.7834889194102	0.8927691256679
0.783591553602	0.8931247823229
0.783665255492	0.8932305569101
0.783744562508	0.8933093112254
0.78381293621	0.8934281624921
0.7839027119498	0.8934613767848
0.7840370882889	0.8939466700121
0.7841167455	0.8939556885701
0.7841911339296	0.8940540435392
0.7842994677546	0.8941799055714
0.7844031492814	0.8943307500842
0.7844722067141	0.8943868293925
0.784573714	0.8944398838355
0.7847219619468	0.8945200055055
0.7848163836483	0.8945223873507
0.7848892319566	0.8945240546008
0.7850023884707	0.894873369745
0.7850911511355	0.8950215039212
0.7851963678978	0.8951068689233
0.7852955310799	0.8951356421992
0.7853989964116	0.8952447233252
0.7855007502131	0.8952572748897
0.7855914403368	0.8953060495156
0.7856429931439	0.8953612778009
0.7857144340635	0.8953949703914
0.7858006902187	0.8954166009536
0.785901933324	0.8954978034083
0.7860238229023	0.8956853586169
0.786091979202	0.8957681813846
0.7861746277411	0.8957771276931
0.786281088428	0.8958226405564
0.7863793544558	0.8958393047398
0.7864381978138	0.8958734142955
0.786523147936	0.8958778024927
0.7866339468957	0.8959261065422
0.7867276452505	0.8962519955068
0.7868093552295	0.8963424152354
0.7868904876808	0.8963468955689
0.7869572766237	0.8963709173991
0.7870782914224	0.8966098174825
0.7871680894139	0.8966130886434
0.7872918179738	0.8966407623748
0.7873905617268	0.8970632784858
0.7874915354874	0.8971367266474
0.7875806433759	0.8972119437694
0.7876813001462	0.8974506336508
0.7877867907888	0.8975091894136
0.7879171944453	0.8981159953004
0.7879898519184	0.8981197983956
0.7880846608245	0.8982412457307
0.7881564073133	0.8982564405966
0.7882532343546	0.8984706396984
0.7883933139296	0.8988725135487

0.7884688506676	0.8989547286378
0.7885315974295	0.8989897499834
0.7886269865107	0.8991278662345
0.7886977407564	0.8991779513914
0.788798108018	0.8993467344585
0.7888817023495	0.899453943743
0.788965893333	0.8997090853769
0.7890869456511	0.8997805763235
0.7891675963415	0.8998107978079
0.7892718310592	0.899888031338
0.7893758724749	0.9000298219558
0.7894836654375	0.9000324534719
0.789565261176	0.9001109688809
0.7896536813012	0.9001649184528
0.7897105109412	0.9002548364181
0.7898130942075	0.9003727484129
0.7898833114746	0.9004474010501
0.7899584959784	0.9004910650362
0.7900655298959	0.9005378115734
0.7901230243195	0.9005426879678
0.7902618850551	0.9005467732685
0.7903386833467	0.9005873287311
0.7904369054834	0.9006113016785
0.7905412943852	0.900642402734
0.7906375943299	0.9009237201431
0.7907436175539	0.9010025912361
0.7908303875125	0.9010637983788
0.7909044217121	0.901146351133
0.7909902391728	0.9011733637567
0.7911039350428	0.9013039224261
0.7911904006182	0.9013369101243
0.7913360406568	0.9023096746373
0.7914221579722	0.9023696847364
0.7915058265032	0.9024500520622
0.7916216417367	0.9026283807991
0.7917195032787	0.9026717237616
0.7917998293371	0.902699034057
0.7918992948885	0.9029305175199
0.7919932341791	0.9029760016408
0.7920767568712	0.903072334386
0.7921441755643	0.9031564819718
0.7922256239424	0.9032961412863
0.792316870465	0.9034046722344
0.7923787906658	0.9034989724595
0.7924557147151	0.903539085665
0.7925883143075	0.903549434461
0.7926922299126	0.9036105752433
0.7928017191747	0.9036964895292
0.7929413686845	0.9039971885087
0.7930246785678	0.9040742946955
0.7931083801952	0.9040839434288
0.7932323190633	0.9041332310645
0.793303980294	0.9042002581489
0.7933869462333	0.9043281396988

0.7934863156231	0.9043570698643
0.7935849449078	0.9044001461073
0.7936760904492	0.9045208695689
0.7937480620799	0.9045863881413
0.7938170465581	0.9046084592076
0.7939418207454	0.9048177373029
0.7940422549581	0.9048352270778
0.7941554265282	0.9051642469878
0.7942532078714	0.9053401494849
0.7943826052007	0.9056032248862
0.7944745916508	0.9056513915888
0.7946071834036	0.9056624650644
0.7947048577704	0.9057980507833
0.7948035559125	0.905846975625
0.7949072337602	0.9061642539867
0.7950052731127	0.90630214605
0.7950912100706	0.9063598387699
0.7951817415624	0.9064257295592
0.7952677312093	0.9065473635004
0.7953516705731	0.9066047168801
0.7954487803073	0.906629625952
0.7955210140806	0.9066516114359
0.7956310488225	0.9067247606738
0.7957151408499	0.9067702380057
0.7958174893372	0.9070069596591
0.7959207231976	0.9070226710133
0.7960225535172	0.9074232308252
0.7961163272821	0.9074523115426
0.7961795292314	0.9075265461901
0.7962671965184	0.9076458472587
0.7963574401306	0.9076565958797
0.7964475747931	0.9077897352172
0.7965398952754	0.9081097318938
0.7965927274424	0.9081343650125
0.7966677112831	0.9082475610366
0.7967651899205	0.9082802622029
0.7968416226717	0.9083059986314
0.7969268952981	0.9083808220936
0.7970198372335	0.9084299811624
0.7971124576281	0.9084393539051
0.7971855549261	0.9085875607191
0.7973205702353	0.9087833812395
0.797436045534	0.9089811738592
0.7975385318325	0.9090316250885
0.7976539755912	0.9092053372948
0.7977392509106	0.9094846768833
0.7978254802303	0.9095126462527
0.7978916256471	0.9095403279346
0.7979724280834	0.9096694989871
0.7980795831278	0.9098903304523
0.7981591975369	0.9099130041612
0.7982814132957	0.9099911384462
0.7983764282584	0.9100384453904
0.7984682938877	0.9100464426821

0.7986021507356	0.9103148242418
0.7986746503097	0.9104545798547
0.7987546981069	0.9106017782298
0.7988162010974	0.9106819633213
0.7989202265991	0.9109368905695
0.7990302495308	0.9109425262006
0.799116277692	0.911032391207
0.7992143371756	0.9112284081123
0.7993390702853	0.9112392383548
0.7994293474637	0.9112707399447
0.7995040465944	0.911561747548
0.7995823267825	0.9117626609305
0.7996712675981	0.911882620747
0.7998293170731	0.9121734494172
0.7999180737226	0.9122019029121
0.8000202892449	0.9122263709575
0.8001077328113	0.9122817339733
0.8001953301463	0.9123625585099
0.8002733347901	0.9126148149968
0.8003558572467	0.9127250452927
0.8004358152992	0.9128066847467
0.800514317887	0.9128239522796
0.8006488552686	0.912829602761
0.8007384845169	0.9131411377314
0.8008249389287	0.9131673835867
0.8008901090202	0.9131708612042
0.8009839135378	0.9132647998336
0.8010695712851	0.9133700596673
0.8011597987599	0.9136371713074
0.8012559444443	0.9136765717261
0.8013543181427	0.9137015107275
0.801443077016	0.9137718389621
0.8015263113489	0.9138565613216
0.8016022895328	0.9138618157063
0.8017015942341	0.913894095418
0.8017900919912	0.913908081232
0.8018635168433	0.9139644898689
0.8019578858397	0.913992761736
0.8020690672095	0.9141560498334
0.8021289178678	0.9141627827176
0.8022281278993	0.9141818414849
0.8022864004824	0.9141906594535
0.8023773282205	0.914209818251
0.8024788985177	0.9142989620854
0.8025856123783	0.9143151149275
0.8026562269328	0.9144385093446
0.8027164094381	0.9144762744591
0.8028104558489	0.9145188468908
0.8028821756571	0.9146462364231
0.8030353571167	0.9148815384215
0.8031559681688	0.9150392907052
0.8032435614303	0.915051727132
0.8033379261278	0.9150774348832
0.8034236261078	0.9150879076687

0.8035261378651	0.9151434545422
0.8036207379445	0.9153374007367
0.8037190709058	0.9154282715682
0.8038026981467	0.9154942359615
0.8039168223986	0.9157203362996
0.8040055563251	0.9159634944589
0.8041285324645	0.916307265417
0.8042259732712	0.9166067452041
0.8042936653664	0.9166225759531
0.804401508958	0.9166748600772
0.8044625929593	0.9166757565093
0.804540927126	0.916837568985
0.8046216340134	0.9169547880397
0.8047116256675	0.9170198704341
0.8048255709724	0.9170683978389
0.8049100279034	0.9171639387669
0.8049906736137	0.9172203502258
0.8050829475508	0.9172477765933
0.8051543225118	0.917265388833
0.8052982709554	0.9174757612933
0.8054224366139	0.9185035779806
0.8054925269498	0.9185879050371
0.8056033537818	0.9186788455422
0.805712131843	0.9190111928771
0.8057744710423	0.9190574471994
0.8058762958498	0.919072186956
0.8060038083589	0.9193802791169
0.8061031450077	0.919624134466
0.8062184038652	0.9196464421109
0.8062911992949	0.9196586271343
0.8063971874182	0.919772907519
0.8065002769481	0.9198703424081
0.8065658351585	0.919876384125
0.8066377462104	0.9199006906207
0.8067081959074	0.9201054515136
0.8068408195384	0.9201404500294
0.8069026538762	0.920245327623
0.8069947263549	0.9203969406967
0.8070899649169	0.9204284831681
0.807224035492	0.9205246666106
0.8073226610923	0.9206279087972
0.8074703200673	0.9212848426945
0.8075622810091	0.9213036838226
0.8076453683376	0.9214636467981
0.8077942980437	0.9215312322672
0.8078776896191	0.9215471842926
0.8079554744379	0.921569435438
0.8080518884845	0.9218595394953
0.8081390627667	0.9221565120621
0.8082487198671	0.9223841707755
0.8083926366335	0.9229239767601
0.8084945578274	0.9232205109482
0.80859312182	0.9234935237101
0.8086628683777	0.9237413761931

0.8087868825097	0.92408655073
0.8089504938178	0.9241744837857
0.8090213483392	0.9242445654088
0.8091233883807	0.9247533917095
0.8092033421644	0.924808757041
0.8092940730859	0.9248237574497
0.8093606589531	0.9248458560434
0.8094318249726	0.9248465125755
0.8095384740451	0.9248930319037
0.8096086854797	0.9249477884781
0.8096753015294	0.9250166788494
0.8097928738545	0.9250279742883
0.8098903638777	0.9253669781681
0.8099971081972	0.9254034158403
0.8100674047499	0.9256139052407
0.810152912395	0.9258406595098
0.8102467717769	0.9260944905172
0.8103794831707	0.9262067208917
0.8104968906061	0.9268112290844
0.8105794676092	0.926856038175
0.8106700902192	0.9268621834823
0.8107667102586	0.9268992377559
0.8108342537675	0.9269898328817
0.81093658602	0.9270825960301
0.8110177834905	0.9271354524356
0.8111323237341	0.9273546103202
0.8112358903743	0.9273801041749
0.8113335322967	0.9274598817269
0.8114336975951	0.9274660823184
0.8115541235039	0.9278439429234
0.8116816527985	0.9280095065646
0.8117745320215	0.9282671680161
0.8118881237506	0.9282835190478
0.8119592319545	0.9284272225045
0.8120510680516	0.9284852666378
0.812157865171	0.9285414346628
0.8122208665321	0.9285596288456
0.8123004130896	0.9285620170384
0.8123822029039	0.928597386525
0.8124654928225	0.9286081109121
0.8125586145086	0.9286215857889
0.8126420639853	0.9286502032285
0.8127578951666	0.9288572572751
0.8128535060515	0.9289670448391
0.8129662288646	0.9290391262376
0.8131302686392	0.9295377866908
0.8132250793292	0.9295911755284
0.813360366795	0.9296450476885
0.8134683345224	0.9297969549128
0.8135731993136	0.9298630253511
0.8136842011561	0.9301274544199
0.8137827270934	0.930136603409
0.8139002033939	0.9302218891502
0.8140075264451	0.9302348218241

0.8141341078073	0.9302973428264
0.8142118334223	0.9304475285279
0.8143136513338	0.9305743761445
0.8144069559922	0.9305979380941
0.8144811660039	0.9306031056498
0.814598451769	0.9306366612054
0.8146839333054	0.9306370113145
0.8147738968703	0.9307358870846
0.8148575265056	0.9307555547846
0.8149423886037	0.9308690831924
0.815030589248	0.9309292360355
0.8151129471815	0.9309978292298
0.8152559240799	0.9310910146901
0.8153887186109	0.9311431380649
0.8154641231589	0.9311675123187
0.8155981447711	0.9313939525555
0.8156913124755	0.9315656950322
0.815784996148	0.9315665080854
0.8158744749551	0.9315904961193
0.8159643939719	0.9316373772663
0.816056280672	0.9316952940383
0.816156677398	0.9317148756101
0.8162747937254	0.9317275366537
0.8163631012571	0.9317616425702
0.8164743049401	0.9318533127616
0.8165952746108	0.931872568843
0.8167037422598	0.9320092763259
0.8168072469523	0.9320473864891
0.8168937150908	0.9323661402183
0.8169762454938	0.9324849481537
0.8170737809059	0.9325615221381
0.8172134286762	0.9325637600309
0.8173238423655	0.9325670599898
0.8174024570939	0.9326499414269
0.8174986893698	0.9328663683497
0.8175622173415	0.9329364635529
0.8176418860371	0.9329460882371
0.817727951961	0.9331970890325
0.8178604574188	0.93343491608
0.8179460588877	0.9334727902607
0.8180301914739	0.933535904231
0.8181453206249	0.9337075870031
0.8182411914522	0.9338266004189
0.8183138197422	0.9339559498126
0.818426555281	0.9340229257815
0.8185136812494	0.9340247650127
0.8186054749173	0.9341870520722
0.8186839543488	0.9342257830905
0.8187838604549	0.9342648284975
0.8188569251108	0.9342891136394
0.8189623597309	0.9345433088558
0.8190756851993	0.9346070594096
0.8192002919699	0.9348086842222
0.8192911533462	0.9348246942927

0.8193777034506	0.9348293473183
0.8194753963978	0.9348921623839
0.8195661779425	0.9349438731494
0.8196673310161	0.9349534114643
0.8197705885417	0.9351414616833
0.8198476173363	0.9352947931852
0.8199464734103	0.9355445189948
0.8200333834489	0.9355681883902
0.8201183962261	0.9357188316808
0.8202271795193	0.9361628628185
0.820330698424	0.9364789786006
0.8204211237893	0.9365038690447
0.8205136264258	0.936574140772
0.8206471720582	0.9366683311037
0.8207649930466	0.9367059648495
0.8208690214941	0.9367874560613
0.8209599698523	0.9368525076071
0.8210574868205	0.9368959589934
0.821128732265	0.9370163025851
0.8212173901206	0.9370831912665
0.8213265751121	0.9370895296174
0.821442815493	0.9371024459164
0.8215293769673	0.9372884733236
0.8216398144915	0.9374092850818
0.8217006807876	0.9375237921492
0.8218030481395	0.9378242117569
0.8219153892376	0.9378295447661
0.8219983112832	0.937846260523
0.8220838592047	0.9378567376236
0.8221618478082	0.9378821910891
0.8222495416306	0.9380079557461
0.8223692929026	0.9381216465579
0.8224695174612	0.938154331237
0.8225612409539	0.9382458474165
0.8226644688519	0.9384359304454
0.8227594783908	0.9385576683645
0.8228822993794	0.938673010995
0.8229919430674	0.938890031021
0.8230887515581	0.939199450003
0.8231712155828	0.9392308494126
0.8233015846166	0.9394027572565
0.8234354120521	0.9395265150651
0.8235184417623	0.9395374989664
0.8236318154532	0.9400383620707
0.8237224446688	0.9400598156413
0.8238299128161	0.9401175875114
0.8238963249176	0.9403464625933
0.8239797622602	0.9403807562015
0.8241148405046	0.940447083769
0.8242597150841	0.9407700718587
0.8243420009012	0.9407752794008
0.824426288379	0.9409210863912
0.8245149419278	0.9410816691079
0.824636432074	0.9415041866957

0.8247297628462	0.9415236407118
0.8248466311939	0.9417422665119
0.8249286274925	0.9418031114478
0.8250280932661	0.9422009018559
0.8251291001378	0.9422252350956
0.825251623684	0.9423470795838
0.825376160097	0.9423683213704
0.8254471790583	0.9424342072692
0.8255306422307	0.9425026417184
0.8256278141322	0.9426685317515
0.8257299108071	0.9427355185608
0.8258109360332	0.942853589553
0.8259304208244	0.9429130705077
0.8260138357967	0.9430344387781
0.8260945140087	0.9430359655169
0.826186143477	0.9430991291694
0.826257511844	0.9431919693285
0.8263698235595	0.9432066343627
0.8264692776433	0.9433337040395
0.8265620924235	0.9433746581186
0.8266340931356	0.9433971287448
0.8267120156493	0.9434213174703
0.8267910151805	0.9434672585793
0.8269284586729	0.9436307320107
0.8270154941337	0.9436989338327
0.8271145885895	0.9439304035613
0.8272049008077	0.9440845378648
0.8273212693637	0.9444175443642
0.827419407064	0.9445203103347
0.8275255812161	0.9446040645166
0.8276345963826	0.9446573377781
0.8277070575268	0.9447755921235
0.8278263771613	0.9449777942072
0.8278980744101	0.9450193975763
0.8279853585532	0.9451604907198
0.8280662944018	0.9452025713777
0.8281870033699	0.9452554794802
0.8282622938158	0.9452650192645
0.8283579104346	0.945407278143
0.8284546074053	0.9454238227877
0.8285141897244	0.945426382703
0.8285990039975	0.9454663637214
0.8286902298283	0.9454712696155
0.8287981629882	0.9455411689733
0.8289125451005	0.9456061658319
0.8290418392462	0.9458561858913
0.8291046719049	0.9458749188698
0.8291771246912	0.9461544569273
0.8292568119742	0.946301142509
0.8293553130933	0.9464208250628
0.8294542377235	0.9465638004478
0.8295550116175	0.9466981427279
0.8296601908274	0.9469036119633
0.829765078103	0.9469127525466

0.8298594835784	0.94692189053
0.8299916163179	0.9470499232249
0.8300851442911	0.9471382147045
0.8301795697315	0.9472085189628
0.8302908228963	0.9472739332051
0.8303958435131	0.9472911680249
0.8304986536417	0.9472995466076
0.8306096242554	0.9473506555669
0.8307067992964	0.9475712465773
0.8307905084229	0.9476298693597
0.8309107764782	0.9476738464069
0.8310138916057	0.9478328018419
0.8311126226306	0.9478929727998
0.8311959675894	0.9479247985926
0.8313458385654	0.9480078256371
0.8314297911693	0.9482390433736
0.8315323278124	0.9485685025888
0.8316297969899	0.9486460290767
0.8317394182178	0.9489082081596
0.8318426139045	0.9491294054174
0.8319386638819	0.9491323360603
0.8320100980984	0.9493326828948
0.8320849057289	0.9493648699554
0.8322405839203	0.9494847087843
0.8323277944423	0.9495488075609
0.8324167297049	0.9495948957466
0.8325139562199	0.9500246286149
0.8325919796209	0.9500391834843
0.8327067558494	0.9501630878972
0.832778093541	0.9501642476436
0.8328807464074	0.9502634036234
0.8329746728281	0.9503195033757
0.8331005415206	0.9504122258839
0.8331680338535	0.9504279055083
0.8332532612871	0.9505240980709
0.8333322213073	0.9506351417417
0.8334476199273	0.9507896352449
0.8335704151337	0.9510017611849
0.8337424119996	0.9510635204516
0.8338155483332	0.9511723856483
0.8338970225705	0.9512174178092
0.8340460873556	0.9516895212933
0.8341736927473	0.9518944948095
0.8342896242467	0.9519649706384
0.8343818602334	0.9520509267809
0.834466566972	0.9521444653227
0.8345676258506	0.9523872893045
0.8346657642604	0.9525286245154
0.8347321897168	0.9525345344233
0.8348194627281	0.952558265027
0.8349168915471	0.9525962615428
0.8350528801173	0.9529917735909
0.835169009279	0.9531211892835
0.8352445058585	0.95316629671

0.8353241521826	0.9532423055004
0.8354103068284	0.9534827082735
0.8354925421728	0.9536152068658
0.8356163518441	0.9537915702247
0.835724257266	0.9538377169354
0.8358425325715	0.9541322449758
0.8359416763839	0.9541614864464
0.8360319431697	0.9542610958207
0.8361255979214	0.9543210971747
0.8362228693951	0.9544589759897
0.8363276879169	0.9545320192215
0.8364070005184	0.9545743450462
0.836511486433	0.9546562619864
0.8366040679849	0.9548067315756
0.8367098706962	0.9548417895735
0.8368644515202	0.9550186568506
0.836989013473	0.9550701426724
0.8370789587739	0.9551091573928
0.8371712314839	0.9551171471414
0.8372673407047	0.9552919654732
0.8373774416143	0.9554730961467
0.8374902749818	0.9555077718169
0.837603212209	0.9556806335133
0.837710547308	0.9556839551599
0.8378375419219	0.9560571910957
0.8379185496916	0.9561245093288
0.8379972943994	0.9561607905265
0.8381363140533	0.9563001075271
0.838241618717	0.9565636364203
0.8383627105386	0.9566672892527
0.83844581728	0.9567642437005
0.8385104408742	0.956875928198
0.8386026660326	0.9569512669707
0.8386834875313	0.9570259243036
0.838737372387	0.9570324611794
0.8388072124745	0.9571494632024
0.8389174184788	0.9571997059988
0.8389852380935	0.9572848469906
0.8390575839173	0.957316758395
0.8391254099316	0.9574022379544
0.8392353406768	0.9578080733733
0.8393441280585	0.9579626325592
0.8394628951013	0.958099873828
0.8395484340468	0.9583976090042
0.8396423526439	0.9585127662481
0.8397159594953	0.9585403212047
0.8398117439233	0.9586446594892
0.8398901761133	0.9586793736988
0.840020364315	0.9590979151483
0.8401327855871	0.9591007101665
0.8402579778801	0.959291680691
0.8403436274966	0.9594823036073
0.8404148342462	0.959515075321
0.840498010935	0.9595644278895

0.8406440338011	0.9596128424609
0.8407376909817	0.9596827719228
0.8408271668353	0.9598124723877
0.8408949695607	0.959873032293
0.8409846009247	0.9599284954431
0.8410906904876	0.9600045560302
0.8411712063396	0.9600766319213
0.8413090450251	0.9600999516315
0.8414098645119	0.9601925108194
0.8415114105547	0.9602421777025
0.8416124015051	0.9605667759109
0.8417288016973	0.9606736569845
0.8418378440461	0.9608673184829
0.8419365694993	0.9609039671944
0.8420431373007	0.9612058676534
0.8421529453651	0.9614636525732
0.8422600517839	0.9615368850963
0.8424255085814	0.9621841475519
0.8425130011175	0.9621866538303
0.8426329784397	0.9623460240172
0.8427288666613	0.9625108550168
0.8428260267618	0.9625282719106
0.842936477944	0.9625431949955
0.8430473731136	0.9629847975398
0.8431524950303	0.9633334629577
0.8432153253743	0.9633510217134
0.8433315227525	0.9633549209088
0.8434165507492	0.9634277128539
0.843532014082	0.9637126689906
0.8436094437517	0.9637985366387
0.8437035232611	0.9639901812956
0.8438146346978	0.9640128835465
0.8439157294473	0.9640784142763
0.8440193210678	0.9640868262701
0.8441090296269	0.9641410443202
0.8442325347608	0.9644555755386
0.8443395872188	0.964625684832
0.8444037819073	0.9646993701336
0.8445111743592	0.9647028034287
0.8446152926652	0.9647767285027
0.8447101857274	0.9648362294249
0.8447941218877	0.9649112051218
0.8449241220928	0.9652350005283
0.8450387486	0.9657381580979
0.8451334039118	0.9657422063836
0.8452461732136	0.9657531785448
0.8453929044747	0.9658043973405
0.8454900925331	0.9661581531174
0.8455624707367	0.9662949317474
0.8456687090015	0.9663840212217
0.8457460868662	0.9664062834074
0.8458315359485	0.9664314279717
0.8459222911047	0.9664450520749
0.8460137016976	0.9666612375239

0.8461185530775	0.9667306998958
0.8462303084591	0.9667950757541
0.8462944587152	0.966808300953
0.8464234616208	0.9668825891285
0.84655024383	0.9672948967422
0.8466176964955	0.9673947073088
0.8467091186213	0.9675393782037
0.846842981958	0.9676382029653
0.8469290409163	0.9676611567495
0.8470305382812	0.9676916677526
0.8471060024055	0.9678254438658
0.8471941801721	0.9679389429096
0.8472939352553	0.9680150260507
0.8474541274498	0.9686864565717
0.8475379547764	0.9687049190787
0.84763101853	0.968752684706
0.8477429956284	0.9687727538431
0.8478834273485	0.9690523545721
0.8480159225208	0.9693337381475
0.8481046654015	0.9693972610487
0.8481871864273	0.9695665685745
0.848283135839	0.9696457649035
0.8484195905112	0.9696872488739
0.8485483896403	0.9699009212121
0.8486543781893	0.9700156242798
0.848750815342	0.9704417205553
0.8488071427149	0.9705050178982
0.8489125437184	0.9705380756435
0.8490079591904	0.9706256367205
0.8491147747236	0.9706777202261
0.8492205542605	0.9707532310355
0.849328697844	0.9707850831438
0.8494370233163	0.9710045280639
0.8495232449146	0.9712272795342
0.8496074919816	0.9712629272345
0.8497014106246	0.9712995981684
0.8498538944072	0.9720978723748
0.8499332637817	0.9721753663117
0.8500452520899	0.972183390364
0.8501579256471	0.972242393066
0.8502941205313	0.9722776119899
0.8503808042581	0.9722790195187
0.8504821381578	0.9726958650389
0.8505908558163	0.9727358666299
0.8506737250346	0.9727619320484
0.8507684282209	0.9729942763496
0.8508773992739	0.973038849351
0.8509775080238	0.9731828977433
0.8510464735532	0.9732552508461
0.8511282649004	0.9733026737587
0.8512014409083	0.9733168972782
0.8513178659646	0.9733837209935
0.8514255011443	0.9734248299019
0.8515091236094	0.9734766736035

0.8516262136034	0.9734893626605
0.8517320075175	0.973647660521
0.8518236207235	0.9737377015585
0.8518821384022	0.973830079825
0.8519583071908	0.9739779916761
0.8520578873412	0.9739929826911
0.8521603401337	0.97451942449
0.8522684887914	0.9745965313615
0.8523937066617	0.9752205028107
0.8524910308824	0.9752889042389
0.8525877510015	0.9754164295453
0.8526588432254	0.9754182358579
0.8527320073232	0.9754828404108
0.8528327811373	0.9755881240307
0.8529306749718	0.9756733997601
0.8530070778258	0.9757098762279
0.8531140645351	0.9758452774002
0.8532575911592	0.9759605685359
0.8533654366487	0.9760331055244
0.8534785268616	0.9763080148773
0.8535688474869	0.9763208995792
0.8536521347474	0.9763704254356
0.8537800475712	0.9765141571688
0.8538639376653	0.9765642409664
0.8539605027973	0.9766172472872
0.8541019332586	0.9768973022007
0.8541917373084	0.9770204771296
0.8542676599268	0.9771196177752
0.8543633366234	0.9772949689471
0.8544422309289	0.9773277501817
0.8545507070339	0.977344777713
0.8546859904204	0.9773646374173
0.8547573954531	0.9774234042719
0.8548488142299	0.9776287072388
0.8549813828278	0.9778589981405
0.8550549415508	0.9778725146313
0.8551496366677	0.9780106686003
0.855274614422	0.9781581819361
0.8553756761958	0.9782467974212
0.8554599968009	0.9782813707495
0.8555994266835	0.9783254146949
0.8556721321463	0.9783435594672
0.8557550724639	0.9783581864903
0.8558833656398	0.9784710766225
0.8560233494206	0.9791664771499
0.8561065999239	0.9792066264123
0.8561839389448	0.9793061253748
0.8562926720595	0.9796645706616
0.8563975648661	0.9797481941877
0.856514559259	0.9802325050207
0.8565749436253	0.9802353182004
0.8566801696041	0.9803372140421
0.8567902284996	0.9804891749554
0.8568836670387	0.9804939275691

0.8569673963178	0.9807680343
0.8570738404546	0.9808291437528
0.8571421642315	0.9808307411547
0.857226862431	0.9808991722846
0.8573114800871	0.9809202376402
0.8574204748893	0.9812719605776
0.8574940539295	0.9814646506241
0.8575853076001	0.9816373471814
0.8576791647611	0.9816384569126
0.857783951366	0.9816460080948
0.8578725045868	0.981717968316
0.858003101527	0.9819584324693
0.8580902217146	0.9819645058667
0.8581797369803	0.9821027970083
0.8582807836644	0.9821516636891
0.8583873132697	0.9821546096834
0.8584768160888	0.9822350271119
0.8585829935104	0.9826082546957
0.8586619151032	0.9827124265558
0.858728983092	0.982776626351
0.858828496416	0.982788485401
0.858931182051	0.9829384659934
0.8590316761426	0.9829679347362
0.8591324423492	0.9829884997364
0.8592307288447	0.9830245514136
0.8593245635494	0.9830739808246
0.8594263102791	0.983088747729
0.8595062199114	0.9831998150859
0.8595985309588	0.9833696346184
0.8597332716825	0.9833706831486
0.8598173607754	0.9833775556711
0.8598957175103	0.9834077713037
0.8599632184274	0.9834761421424
0.8600725807087	0.9835637684514
0.8601725273463	0.9841285772548
0.860324477264	0.9848456952277
0.8604127999168	0.9848932994076
0.8605416563758	0.9854731984176
0.8606600856861	0.9856380430225
0.8607709978456	0.9858865980119
0.8608961073557	0.9860250100271
0.8610115175391	0.9861085864195
0.8611142345013	0.9861385642052
0.8612019873177	0.9862384121023
0.861330608567	0.9863721554789
0.8614240985365	0.9864973538653
0.8615213304439	0.9865535939748
0.8616059438369	0.9866184564619
0.8616843977805	0.9866513123632
0.8618053441761	0.9869476202436
0.8619260286903	0.9869788135177
0.862040611395	0.9870617654261
0.8621295473187	0.9871226900331
0.8622154952249	0.9871426239767

0.8623309705231	0.9875218727633
0.8624133427439	0.9875543689763
0.8625045534515	0.9875798140528
0.8625839230777	0.9876098678203
0.8626898652394	0.9878323988261
0.8627865148549	0.9879132958817
0.8628726221131	0.9879158931376
0.862970447543	0.987986236757
0.8631100428767	0.9880319761574
0.8631975873885	0.9883054830986
0.8632709441255	0.9883463263126
0.8633917511041	0.9887035436805
0.8634889867053	0.9887373282999
0.8636090511973	0.9889681598345
0.863762251826	0.9890200826804
0.8638839296381	0.9892026502077
0.8639747971247	0.9892375167569
0.8640464854498	0.9893584345116
0.8641719918951	0.9894563855103
0.8642657921355	0.9894959579851
0.8643665388346	0.9895536108537
0.8644624216318	0.9895737837239
0.8645411383736	0.9896756994146
0.8646468809414	0.99004252289
0.8647474745584	0.9901037926631
0.8648318709148	0.9901417860591
0.8649203974119	0.9903201217973
0.8650535742342	0.9903623122311
0.8651629066952	0.9903627058946
0.8652606934863	0.9904950896012
0.8653792828979	0.990559523521
0.8654741671168	0.9905758248622
0.8655681807006	0.9906116318867
0.8656573751792	0.9906427458544
0.865747282264	0.9909533397994
0.8658499030385	0.9909928780071
0.8659625033112	0.9910031886034
0.866067532337	0.9910585378417
0.8661580019043	0.9913547921027
0.8662736175601	0.9915410724171
0.8663783358912	0.991582794487
0.8664478708074	0.9916587610801
0.8665202501294	0.9917405586736
0.8666175837396	0.9918034012941
0.8666844401023	0.9918592225413
0.8667843484528	0.9919321345701
0.8669138927283	0.9919371144359
0.8669940131083	0.9919406873859
0.8670756149911	0.9921331045716
0.867183432416	0.9921585722412
0.8672874801841	0.9923679147232
0.8673804047924	0.992442489076
0.8674750873405	0.9924483579799
0.867583272646	0.9926331824593

0.8677489923916	0.9927763280555
0.8678604911432	0.9930191372009
0.8679360320272	0.9930850668286
0.8680276572154	0.9930879463347
0.868093862335	0.9931243184369
0.86819655227	0.99316427998
0.8683357447567	0.9936457400767
0.8684414224929	0.9936970177786
0.8685168477271	0.9938554657845
0.8686084176877	0.9941408226211
0.8686872995404	0.9942557417359
0.868766231769	0.994349844957
0.8688378118098	0.9943642004907
0.8689453892586	0.9945979127874
0.8690634023537	0.9948088348084
0.8691313585872	0.9949329758093
0.8692115355491	0.9949740797784
0.8693038140851	0.9949768925391
0.8693921409283	0.9950530469647
0.8695023149434	0.9950779408871
0.8696418924959	0.9954737495091
0.8697510161281	0.9954751999841
0.8698516648151	0.9955659093291
0.8700033660126	0.9957325168397
0.8700848548188	0.9957456041761
0.8701588390363	0.9960021968088
0.8702464633173	0.9961105319821
0.8703546444588	0.9963408427764
0.8704629218801	0.9967243865889
0.8705648356544	0.9968765980207
0.8706857652996	0.9969130903235
0.8707632866868	0.9970265919433
0.8708786378142	0.9971030090765
0.8709946254857	0.9971822313424
0.8710543735914	0.997213444697
0.8711583415372	0.9974112675803
0.871264220649	0.997629100565
0.871370158572	0.9977525456856
0.8714505990195	0.9977968776569
0.8715486194181	0.9978245114106
0.8716714546843	0.9981432044902
0.8717777960398	0.9981934490193
0.871880813004	0.9983617440616
0.8719613018989	0.9985564679652
0.8720394082615	0.9985901598176
0.872127767458	0.9986228790331
0.8722270475316	0.9989280891041
0.8723261448871	0.9990055691563
0.8724319506166	0.9993474526767
0.8725780986546	0.9997130610023
0.872684360895	0.9997935599793
0.8727630838657	0.9998086328824
0.872871993514	0.9998790374601
0.87302237132	0.9999531171294

0.8731529223295	0.9999843453304
0.873227834141	1.0000701235691
0.8733280806825	1.0003611993721
0.87341464866	1.0003626202112
0.8735172292106	1.000525521202
0.8736032537863	1.0005497640365
0.8736628981183	1.0006344514934
0.8737655017169	1.0007466987408
0.8738747539495	1.0008005457982
0.8739649527276	1.0008136111003
0.8740711688351	1.0008136341264
0.8741668673218	1.0008162662294
0.8742745946887	1.000828540353
0.8743776315054	1.0008683555099
0.87446529274	1.0009476004626
0.8745569870924	1.0011176113386
0.8747100393725	1.0011469545418
0.8748065593827	1.0011577505571
0.87490426031	1.0012280747262
0.8750441690433	1.0012633413121
0.875137582776	1.0012638330635
0.8752484835771	1.0013196681184
0.8753238581915	1.0014182712315
0.8754059851297	1.0014185266797
0.8755086208588	1.0014253061789
0.8756254288701	1.0017783321895
0.8757328450689	1.0018298604645
0.875856243754	1.0022700068775
0.8759547311455	1.0023346064536
0.8760352520186	1.0023403308288
0.8761595777925	1.0024545179626
0.87626955033	1.0027000798699
0.8763791022652	1.0029402405338
0.8764844412322	1.0029969845252
0.8765961529651	1.0030230615719
0.8767118798109	1.0030588659834
0.876799211077	1.0030861904697
0.8769290438007	1.0032128091976
0.8770080170255	1.0032812171531
0.8771238138956	1.0035445689051
0.8772638371844	1.0039000961818
0.8774017638256	1.0040734820503
0.8775092106592	1.0040894397095
0.8776274919602	1.0042620495419
0.8777631686896	1.004343454695
0.8779045240308	1.0043678150459
0.8780195028435	1.0043885649975
0.8781366914339	1.004647669643
0.878242577449	1.0047032229792
0.8783148161054	1.0048318883347
0.8784385296064	1.0051763527415
0.8785676534482	1.0053582594951
0.8786724039221	1.0054414683593
0.8787387526075	1.005482929769

0.878833232265	1.0056197636241
0.878942302392	1.0056501760185
0.8790663173428	1.0058648870215
0.8791964712399	1.006019552509
0.8792986937572	1.0060396925973
0.8794192914954	1.0061122283714
0.87949872231	1.0061358750516
0.8795881372726	1.0062248569389
0.8797302703541	1.0064285910253
0.8798042788709	1.0064306828752
0.8799009780107	1.0064501546659
0.8799777802463	1.0065509170601
0.880077254834	1.0065718373089
0.8802456751109	1.0067691169711
0.8803486721681	1.0068108500354
0.8804195307587	1.006901173289
0.8805226885629	1.0070481025322
0.8806588772786	1.0070831911277
0.8807569138815	1.0071132507132
0.8808357377181	1.0071250924791
0.8809424450843	1.0073831662553
0.8810333653218	1.0073987773605
0.8811340626024	1.0074805404231
0.8812239040645	1.0075035838938
0.8813312686108	1.007652341028
0.8813963367519	1.007716967552
0.8814907045305	1.0079754232029
0.8815944321697	1.0081738351108
0.8816662394051	1.0082150799484
0.8817271503411	1.0082519612535
0.8818012754568	1.0083091603632
0.8819128695215	1.0084987917767
0.8819988806785	1.0085754772154
0.8820846703211	1.0086973084318
0.8821989709295	1.0089349236136
0.8823100383705	1.0092219998162
0.882370773222	1.009322691893
0.8824419796629	1.0093694773969
0.8825671593455	1.0097937788916
0.8826562268266	1.0098643481438
0.8827520198272	1.0100744640006
0.8828302186577	1.0100951159657
0.8829229624196	1.0105399036172
0.8830229000269	1.0107357823607
0.8831058175754	1.0107502289205
0.8832167076077	1.0107516851028
0.8833307205515	1.0108343467828
0.8834348685377	1.0108982641002
0.8835744373593	1.0109285930912
0.8836917025297	1.0112114803251
0.8837843188506	1.0113365661261
0.8839104244379	1.0116143276969
0.8840121676589	1.0117349889399
0.8841123471279	1.0119783825972

0.8842046142275	1.0121023446297
0.8842969892699	1.0122967394183
0.8844079864752	1.0124493812016
0.8845379172424	1.0125592641609
0.8846149460089	1.0126146393166
0.8847463217226	1.012627775098
0.8848263428042	1.0128769953065
0.8849512672903	1.0128981311504
0.8850691942919	1.0129457782957
0.8851645136547	1.0130563611765
0.8852499351009	1.0131175004762
0.8853370074775	1.0132145465
0.8854241909653	1.0132221036092
0.8855515316485	1.0132485651098
0.8856655635838	1.0135394151117
0.885781689963	1.0136973267089
0.8858879267269	1.0139478190386
0.8859806747738	1.0139558995047
0.8860660864735	1.0139789013032
0.8861562454761	1.0139802912089
0.8862665932764	1.0140787065473
0.8863552416738	1.0142267200833
0.8864814572476	1.0146984198999
0.8866127882213	1.0150537614733
0.8866924638339	1.0151857563406
0.8867595348439	1.0153676704031
0.8868677639246	1.015416480547
0.886998233958	1.0154250409683
0.8870858163706	1.0155461327383
0.8872117952577	1.0155759410357
0.8873313262327	1.0159239734626
0.8874307175703	1.0159431607107
0.8875159713041	1.0160055244911
0.8876150926429	1.0161350556817
0.8877088839262	1.0161513208947
0.8877901901066	1.016167734239
0.8878764677834	1.0161683291082
0.8879722559725	1.016449283052
0.8880907560168	1.0167963075169
0.8881767740092	1.0169084588299
0.8882808525883	1.0171784140549
0.8884036490167	1.0173478106046
0.888502463296	1.0175074592036
0.8886058809028	1.0176110217342
0.8887189375772	1.0176810983261
0.8888309967492	1.017743394606
0.8889102530901	1.0178507780341
0.8889966554443	1.0178739735149
0.8890957050263	1.017949292951
0.8892184617631	1.0179876085332
0.8893040461919	1.0183081503817
0.889394566428	1.0183567676221
0.8894782341484	1.018379351561
0.8895769256598	1.0186208453249

0.8896789248802	1.0189403399804
0.8898201312105	1.0189988978517
0.8899690376403	1.0192503516338
0.8900894821165	1.0192638837724
0.8902248864637	1.0194592052347
0.8903574715947	1.0198609749833
0.8904705553753	1.0199663533148
0.8905764234806	1.0200301720677
0.8906908576985	1.0201593280687
0.8907962067594	1.0201915398992
0.8908878178174	1.0202369840189
0.8909827062456	1.0203085190448
0.891079137008	1.0203496293933
0.8912208261874	1.0205355775448
0.8913266554931	1.0207749541011
0.8914079482559	1.0208979045746
0.891495890747	1.0209602128373
0.891585421354	1.0210172094138
0.8917684876527	1.0214766316262
0.891906341925	1.0214810416125
0.8920040560192	1.0215493687448
0.8921367471235	1.021564550924
0.8922658281232	1.0215916508766
0.8923252032422	1.0216387064808
0.892413327743	1.0217533799943
0.8925174790534	1.0218232054446
0.8926106671457	1.0218587645457
0.8927221240417	1.0219660210065
0.8928250668701	1.022138740902
0.8929534015378	1.0221945019513
0.8930338722504	1.0221969423627
0.8930905415749	1.0223448302006
0.893174478183	1.0225015952348
0.8932574408175	1.0225295408408
0.8933607498328	1.0227432858858
0.8934886614564	1.0230566763965
0.8935957733655	1.0231791750701
0.8936958466445	1.0232714640123
0.8938114781458	1.0233052025336
0.8938994385826	1.0233556074722
0.8940033867161	1.0233561683647
0.8941069314678	1.0237067993617
0.8941798105345	1.0237099468116
0.8942943305924	1.0242875880744
0.8943852600739	1.0243591358869
0.8944615904299	1.0243980645083
0.8945476721592	1.0244250408659
0.8946401334147	1.0248058907699
0.8947532554697	1.0248674884243
0.8948708524204	1.0248936079553
0.8949865095083	1.0249076244979
0.8950746261074	1.0249250039859
0.8951794232461	1.0250888166088
0.8952923550152	1.0251229560013

0.8953610157103	1.0254875070444
0.8954427397578	1.0255705466231
0.8955652007913	1.0256046722096
0.8956631593442	1.0257701979194
0.8957837330983	1.0257958242187
0.8959103342275	1.0262000129614
0.896041377197	1.0262810424411
0.8961573076945	1.0265248395623
0.8962831849631	1.0269568881763
0.896346245231	1.0269608053465
0.8964525963064	1.0271118439021
0.8965368505797	1.0272284639179
0.896660219614	1.0272893245363
0.8967537704428	1.0273975960149
0.8969042872417	1.0275359247262
0.8970230508459	1.0277700219533
0.8971565827181	1.0279250897384
0.8972517982828	1.0280373280729
0.8973674433901	1.0282025046053
0.8974745977145	1.0283236212972
0.8976006214212	1.0283795576712
0.8977073765547	1.0284703368638
0.897786497591	1.0285172968006
0.897897588472	1.0285422277323
0.8980069297917	1.0286520017407
0.8980875491357	1.0287255088695
0.8981965724593	1.0289746959907
0.8982666255882	1.0289963109509
0.8983470234747	1.0290954242868
0.8984726735282	1.0293486640517
0.898543668035	1.029446531605
0.8986469236431	1.0295247283574
0.8987309519142	1.0299019661598
0.8988218659559	1.0302516108174
0.8989064227506	1.0302882562191
0.8989959216153	1.0305266624428
0.8991085870363	1.0306084617864
0.8992062704771	1.0306810528856
0.8992747229069	1.0307243815196
0.8993665701468	1.030890642457
0.8994840019867	1.0309048415885
0.8996116183806	1.031319198585
0.8997210253403	1.0313397933339
0.8998520840333	1.031586520054
0.8999570131114	1.0315952973381
0.9000569541211	1.0316627649411
0.9001779875426	1.0317196866244
0.9002535084732	1.0317890408859
0.9003855328743	1.0318662330263
0.9004929867321	1.0319991581503
0.9005742441692	1.0320913512476
0.9006693067573	1.0321030769094
0.9007478028261	1.0321660265294
0.9008447450802	1.0323771729984

0.9009451043361	1.032552464561
0.9010682546119	1.0327041694551
0.9011737583444	1.0328172076217
0.9012564758271	1.0328453659706
0.9013780487868	1.0330225817048
0.9015096016371	1.0332485804137
0.9016601805042	1.0333008008853
0.9018347885934	1.0337033494495
0.9019237704305	1.0337855492679
0.9020692070041	1.0340898131085
0.9021693648847	1.0342572869368
0.9022792188538	1.0342617067711
0.9023770720303	1.0342770879705
0.9024752764914	1.0343012461158
0.9026082712526	1.0347147163746
0.9027146468761	1.0348504276926
0.9028298045415	1.035076972366
0.9029214847484	1.0352716312309
0.903013210195	1.0354529736928
0.9031452624216	1.0356854380308
0.9032542168905	1.0357471113625
0.903352767743	1.0357703650534
0.9034378705962	1.0360277116698
0.9035747544	1.0362137770849
0.9036571108559	1.0362209011469
0.9037824820458	1.0363063640109
0.9038840121677	1.0363819087537
0.9039810282823	1.0364257853921
0.9040740495973	1.0367570422042
0.9042071673396	1.0369180971393
0.9043048570351	1.0371368671342
0.9044008236898	1.0373108675435
0.9045236232109	1.0375287342835
0.9046232014657	1.0375676915674
0.9047299697123	1.0375955423912
0.9048481956235	1.037650153843
0.9049277301895	1.0376677145856
0.9050146781258	1.0377233387599
0.9051078432703	1.037836585923
0.9052011196376	1.0379333102559
0.9053296189736	1.0381386476173
0.9054511364352	1.0381724182768
0.9055621906278	1.0388747612982
0.9056742158734	1.0388895180433
0.9058319077395	1.0390153265712
0.9059108873559	1.0390740851911
0.9060340180301	1.0392258753058
0.9061464873073	1.0393055363182
0.9063085596	1.0395355563383
0.9064183834199	1.0397820111434
0.9065519495359	1.0401555727467
0.9066298697617	1.0403141203071
0.906731085362	1.0403501951258
0.9068242006665	1.0404121012788

0.906933955945	1.04073769262
0.9070293548569	1.040987251473
0.9071459933352	1.0415944145897
0.9072833243076	1.0418626180709
0.9073656662388	1.0421242853979
0.9074562079949	1.0421449855433
0.9075720625607	1.0422534703263
0.9077082087845	1.0422604126883
0.9078379132261	1.042349414754
0.9079554454835	1.0423957978067
0.9080318798694	1.0423971225602
0.9081334844847	1.0424448317287
0.9082215218151	1.0425220939333
0.9083182128598	1.0425309171517
0.9084126424697	1.0426980934675
0.9085517702525	1.043021498879
0.9086722101445	1.0435198063346
0.9087498227258	1.0435277314509
0.908871925353	1.0437408357973
0.9089789586203	1.0437496215605
0.9090732303716	1.0438477163041
0.9091638656507	1.0438821710756
0.9093502809795	1.0439089320064
0.909463752549	1.0440092934411
0.9095566388476	1.0440898912151
0.9096694595685	1.0441448639051
0.9097452480945	1.0442346129984
0.9098687800051	1.044511345162
0.9099441078256	1.0445714166534
0.9100316026359	1.04464056576
0.9101310733381	1.0447872576444
0.910247209909	1.0450361616265
0.910354430808	1.0450694328927
0.9104606628294	1.0452098460114
0.9105708332016	1.0452098937253
0.9106727945408	1.045308943001
0.9107520950429	1.0455354609469
0.9108601298966	1.0457309281629
0.9109874041803	1.0457650364572
0.9111309631272	1.04592741209
0.9112197269294	1.0461584626303
0.9113875859549	1.0470727256999
0.9114905677479	1.0471193279245
0.9116376598763	1.0473639530169
0.9117575807871	1.0474195715894
0.9118912005963	1.0476395101111
0.9120234444908	1.0477456346712
0.9121599357553	1.0479977575786
0.912304773661	1.0481699915327
0.9124334582075	1.0485573854998
0.9125239315102	1.0485835298079
0.9126459738659	1.0488227726274
0.9127435050826	1.0489011999843
0.91286422981	1.0494065110621

0.9130170753025	1.0495359159442
0.9131244339813	1.0495481997964
0.9132329176652	1.0496881204337
0.9133345106365	1.0497054347077
0.9134428834742	1.0498013707789
0.9135570435868	1.0499100170717
0.9137003800463	1.0500011903604
0.9138229577895	1.0500456830591
0.9139307473941	1.0501611053616
0.914036538786	1.0501777965302
0.9141404342352	1.0502384486307
0.9142467936844	1.050518067041
0.9143478110051	1.0505531147651
0.9144493422467	1.0508473184194
0.9145677299047	1.0508966733743
0.9146735811768	1.0509389392445
0.9147933502308	1.0513072632
0.9149139931914	1.051517060522
0.9150113398455	1.0516593280757
0.9150859276547	1.0516649221278
0.9152022998482	1.0516727596737
0.9152967393269	1.0516886998629
0.9153814735654	1.0517620815155
0.9154965299772	1.051798742958
0.9156071423497	1.0518017956338
0.9157238598388	1.0519391652385
0.9158244478676	1.0521384688607
0.9159427352793	1.0521588424157
0.9160463578625	1.05225272921
0.9161595483068	1.0522746847781
0.916235842939	1.052590152323
0.916379335906	1.0527024005506
0.9164877071215	1.052789447199
0.9165663843103	1.0528458722028
0.916663364639	1.0530415607307
0.9167627584702	1.0530818493403
0.9168791279228	1.053225616781
0.9169915182493	1.0534018575943
0.9170696639418	1.0534045558465
0.9171583727258	1.0536204066523
0.9172991990424	1.0537944337405
0.9174113212823	1.0541689124939
0.9174947991446	1.0541779824037
0.9176409880175	1.0543718330009
0.9177273228236	1.0544330055024
0.917821285041	1.0545232232562
0.9178957089862	1.0545488174918
0.9180299194099	1.0552298490803
0.9181230694354	1.0553119125591
0.9182395732089	1.0554030109469
0.9183275950529	1.0555079915095
0.9184091631347	1.0555533905086
0.918533940733	1.0557227786164
0.9186419340458	1.0559260135598

0.9187471095376	1.0559761920282
0.9188460894085	1.0562242126342
0.9189699386854	1.0564407104025
0.9190588785556	1.0564699493783
0.9191516976351	1.0564890495621
0.9192701333441	1.0565675799194
0.9194299886456	1.0567377984388
0.9195566303197	1.057027314504
0.9196772060379	1.0570660677554
0.919768548291	1.0571187988572
0.9198547469795	1.0572840396992
0.9199887072275	1.0574106291404
0.9201136479121	1.0576505829439
0.920231847496	1.0577510357159
0.9203312469248	1.0579813333736
0.9204100109843	1.0580638848602
0.9205141691611	1.0583366886344
0.9206554241707	1.0590005085208
0.9207418342791	1.0590681355975
0.9208309163623	1.0591185873759
0.9209301971469	1.0591403690408
0.9210118221364	1.0591581132352
0.9210922167932	1.0593958980482
0.921189897644	1.059594225059
0.9213198375363	1.0598400231033
0.9214126408511	1.059890850241
0.9215212449041	1.0599475914888
0.9216177778319	1.0600211498435
0.9217174390582	1.0600380420395
0.9218158872268	1.0601324772796
0.9219340295633	1.0604220312077
0.9220372586778	1.0606081542113
0.9221536764479	1.0606971785066
0.9222596900171	1.0608089500534
0.9223624410498	1.0608433199174
0.9224878774989	1.0611093628403
0.9225805846914	1.0611692792772
0.9227043351957	1.0612208990964
0.9228049549284	1.0612579881947
0.9229400053258	1.061896189979
0.9231201947299	1.0626233662407
0.9232055991752	1.0626412202737
0.923314092957	1.0626754510261
0.9234366703055	1.0626871111257
0.9235631322731	1.0629116144159
0.9236662904024	1.063028414084
0.923752258405	1.0630332104884
0.9238536333627	1.0630880077226
0.9239627470087	1.0633972967769
0.9240701076111	1.0634619273399
0.9241689770857	1.0634908099819
0.9243005945424	1.0636514570957
0.9243935599404	1.0637299193183
0.9245173044593	1.0638386697017

0.9246098804596	1.0638921634575
0.9247044960662	1.0639272546437
0.9248823335812	1.0640667034416
0.9249514370989	1.0640895504018
0.925057855697	1.06421813873
0.9251620147012	1.0644073308272
0.9252587254171	1.0644690675829
0.9254005474813	1.0647424899273
0.9255361654745	1.0647733297315
0.9255982440461	1.0647827410708
0.9256808173471	1.0648894671472
0.9257944628158	1.0648902821314
0.9259097418778	1.0649108772098
0.9260264196233	1.0651381385334
0.926234952087	1.0655456749512
0.9263258154799	1.0656357766037
0.9264308052942	1.0656621507435
0.9265297253988	1.065961126314
0.9266267865508	1.0660118938003
0.9267616603206	1.0660441370036
0.9268734150518	1.0660736327425
0.9269624051097	1.066149219643
0.9270748137744	1.066663053922
0.9271790915928	1.0667594378223
0.927366010814	1.0668217690809
0.9274832461269	1.0671480432736
0.9275891271316	1.0675286210994
0.927675580047	1.0675325609107
0.9277821105517	1.0676832072594
0.9278835289381	1.0677106800598
0.9279865204763	1.0678342425109
0.9281234333848	1.0682608476425
0.9282275533318	1.0682841379097
0.9282986804812	1.0683424290427
0.9284191235605	1.0684150948093
0.9284983999588	1.0684162259238
0.928612996018	1.0684282624014
0.9287002649088	1.0685781970559
0.9287758034539	1.0685784869744
0.9288905462287	1.0685901253402
0.9289617161623	1.0686299646334
0.929098355696	1.068934070682
0.9292147445391	1.069212651915
0.9293122864218	1.0693976142881
0.9294218899395	1.0695247946271
0.92950132346	1.0696908688138
0.9295689887427	1.0697793900619
0.9296632082673	1.0698009758743
0.9297667529884	1.0698018400848
0.9298843178979	1.0699306209507
0.9300510702326	1.0703732245949
0.9301492636347	1.0704184562511
0.9302421121755	1.0706276597807
0.9303534758214	1.0707608556699

0.9304484148291	1.0708782218575
0.9305795884395	1.0709092186324
0.9306929225381	1.0713583426334
0.9307864616922	1.0714047214117
0.9308933325525	1.0714369907716
0.9309995894744	1.0715742883854
0.9311210556791	1.0716505391893
0.9312261844254	1.0716771994634
0.9313373865844	1.0720978441392
0.9314165784551	1.0721657032422
0.9315689587529	1.0722348675446
0.9317009939691	1.0725343317025
0.9317870988602	1.0726029387229
0.9318782281173	1.0728432920132
0.9320300588471	1.0730490195882
0.9321691206044	1.0732115079886
0.9322944448128	1.073341717692
0.9324254898731	1.073435070458
0.9325913717126	1.0737650999024
0.9326984012021	1.0738376979602
0.9328219196334	1.0739754767805
0.9329135407034	1.074082435026
0.9330513656848	1.0742656388651
0.9331663577809	1.074287455452
0.9333169445821	1.0744549183009
0.9334645119119	1.0745974785899
0.9335845789772	1.07503197186
0.9337061573416	1.075094020698
0.9338465229668	1.0754973800373
0.9339368605266	1.0755088486474
0.9340784137393	1.0755638217143
0.9341982177092	1.0755808497306
0.9342801852517	1.0756989742212
0.9344179815956	1.0757398250764
0.934503504411	1.0757954232646
0.9346158567032	1.0758796641707
0.9347564137813	1.0760966087705
0.9348681269673	1.0763031108061
0.9350007380867	1.0763311054533
0.9350814785827	1.0763528104723
0.9352023220148	1.0763950922528
0.9353171909155	1.0764957185
0.9354475585275	1.0765040634001
0.9355760234516	1.0767832862039
0.9357093183121	1.077213972548
0.9358774552174	1.0775097119256
0.9359813734477	1.0775491506894
0.9360704781443	1.0775845874034
0.9362152454074	1.0781756635168
0.9363459290959	1.0782581673021
0.9364471202898	1.0783789484895
0.9365598422537	1.078576835569
0.9366809923026	1.078819600886
0.9367787511413	1.0788910591489

0.9368877724884	1.0789980424937
0.9369824204315	1.0790566418524
0.9370681699767	1.0791138803406
0.93717522124	1.0791277616551
0.9373012365159	1.0792477330952
0.9374260850868	1.079281367108
0.9375201557322	1.0795883765818
0.9376319117159	1.0798254395836
0.9377421878848	1.079864279753
0.937870190682	1.0800144654633
0.9379795668529	1.0800880030775
0.9380551386859	1.0801437382389
0.9381591740251	1.080182530065
0.9382493655209	1.0802395941117
0.9383532662953	1.0802670507261
0.9384672694796	1.0803903847707
0.9385801561727	1.0804329616745
0.9386787098762	1.0805762474383
0.9387789449342	1.0805828566556
0.9388808127831	1.08061236594
0.9389984990314	1.0808694901081
0.9391174243791	1.0810624736276
0.9392900766639	1.081139843257
0.9393967075907	1.0812902339799
0.9394885287502	1.0814712257765
0.9396393731154	1.0815172920846
0.9397464255632	1.0815194875759
0.9398583484416	1.0816268854672
0.939948002195	1.0816950048312
0.9400043982012	1.0816951289138
0.9401522949984	1.081906576615
0.9402526067265	1.0819197230252
0.940363114196	1.082214148863
0.9404689998424	1.0822343838487
0.9406114095396	1.0823657919332
0.9407284459479	1.0824176618793
0.9408299431453	1.0824555833266
0.940911965684	1.0826328670592
0.9410063476572	1.0826362234751
0.9411493569341	1.0829858009026
0.9412761785771	1.0831532973957
0.9413656966325	1.0831772254099
0.9414941627654	1.083308556744
0.9415628597393	1.0833875667929
0.9416435600274	1.0834774410638
0.9417686129201	1.0835283188929
0.9419001675804	1.0837810605792
0.9420247306945	1.0838647944073
0.9421334577365	1.0839697286803
0.9422670722147	1.0841421856232
0.9423571241036	1.0842898261111
0.9424361909766	1.0843124024917
0.9425711691324	1.0843538366531
0.9427082443299	1.084541259199

0.9428161481688	1.0847207584419
0.9429138027259	1.0848295145108
0.943020089857	1.0849619175962
0.9431480755642	1.0850415925284
0.9432592345611	1.0850533158334
0.943334968778	1.0850942573067
0.9434431692854	1.085257186346
0.9435578102653	1.0854264502811
0.9437046445878	1.0856163814711
0.9438250527966	1.0856951178005
0.943961200629	1.0859043479992
0.9440599540651	1.0859153630668
0.9441306136682	1.0859580155608
0.9442470199283	1.085996717963
0.9443610640449	1.0860762613875
0.9444608614651	1.0861011519999
0.9445520520778	1.0862055743119
0.9446557132575	1.0862235394954
0.9447740591014	1.0862497826984
0.9448639286563	1.0863258609764
0.9449624328217	1.0863616856665
0.9450889472019	1.086733097591
0.9451666679531	1.0867442094332
0.945284705009	1.0868541279106
0.9454189790924	1.0871937703098
0.945523818844	1.0872792205812
0.9456249684524	1.0875131050237
0.9457832255745	1.0876555409416
0.9458628120287	1.0876718504277
0.9459794838965	1.0880722524742
0.9461304151151	1.0884613598398
0.9462634559444	1.0884733332732
0.9463796183218	1.0885923775989
0.9464612937293	1.0886031432402
0.9465690202164	1.0887354597574
0.9466553006115	1.0887890824067
0.9467357756258	1.0889128735207
0.9468474683471	1.0889247653454
0.9469797169774	1.0892023457439
0.9471131116623	1.0893383298233
0.9472428460095	1.0894260126658
0.9473316687454	1.0896843965492
0.9474167024569	1.0897561006501
0.947532975656	1.0899699253692
0.9476430806396	1.0901665245243
0.9477732860607	1.0904072673849
0.9478587754582	1.0904763750742
0.9479574148206	1.0906227813107
0.9480559287242	1.0907035016121
0.9481488940869	1.0907926467643
0.9482484932662	1.0907932058972
0.9483551655668	1.0908444994568
0.9484742038136	1.0911089698881
0.9485961842063	1.0912922629173

0.9486880700608	1.0917169283331
0.9487727785695	1.0919606553814
0.9488987094043	1.0920871626088
0.9490065220416	1.0922904261571
0.949102732599	1.0923149300337
0.949207397334	1.0923237581883
0.9493246897019	1.0923939775186
0.9494442850023	1.0924574556534
0.9495851652098	1.0925378174394
0.9496894768335	1.0927604784221
0.94980990809	1.0928201642762
0.9499198354428	1.0928477898539
0.9500763691979	1.0932199955056
0.950138445532	1.0932309430315
0.9502088131114	1.0932988254036
0.950291162659	1.0933297872795
0.9504208420935	1.0934329871416
0.950514891651	1.0934750030401
0.9506033285692	1.0935092514371
0.9507350740654	1.0936738934884
0.950863281609	1.0940857958311
0.950956239418	1.094338256491
0.9510861450566	1.0943488312495
0.9512017808983	1.0943630916544
0.9513263332899	1.0946430227104
0.9515120459597	1.0947437478457
0.9516667578498	1.0947469617391
0.951780201518	1.0949759764797
0.9518743299128	1.0951269490525
0.9519776699154	1.0954213468593
0.9521322768101	1.0962419578071
0.9522451874832	1.0963401937732
0.9523802654457	1.0964152381724
0.9524908227824	1.0965153991584
0.9525865518114	1.0965288556047
0.9527120919747	1.0965471428305
0.9528365952093	1.0966372001406
0.9529340778177	1.096653638466
0.953031554445	1.096751851329
0.95313906949	1.0970015229383
0.9532440568688	1.097159177674
0.9533716218584	1.0971750383317
0.9534898363019	1.0972832610838
0.9536047933602	1.0975030878791
0.953700882888	1.0976646693274
0.9538161236778	1.0978547025946
0.9539712014552	1.0981191782974
0.9540909233844	1.0981741913067
0.9541870489085	1.0981883742922
0.9543772869081	1.0984175075523
0.9544611109659	1.0984905225728
0.954565695932	1.0987308066883
0.9546906477042	1.0988326402244
0.9547708792494	1.0989942991678

0.9548655786351	1.0990327825406
0.9549881372026	1.0990520148469
0.9551054768508	1.0992873041794
0.9552080383071	1.099420865026
0.9553264776402	1.0996679728338
0.9554215209672	1.0997912027927
0.9555274391366	1.0998067533059
0.9556329219044	1.0998959702021
0.9557588836606	1.1000213435192
0.9558820796619	1.1000811515648
0.9559771777266	1.100195584823
0.956071098627	1.1002030536047
0.9562079118914	1.1002056941824
0.9563594503878	1.1003318435785
0.9564845816216	1.100332433044
0.9565801874619	1.1003895979318
0.9567344443247	1.1005752722137
0.9568494930861	1.1006115231345
0.9569805533812	1.1006132414207
0.957088722373	1.1007726399828
0.9571834550786	1.1008377912145
0.9572660635135	1.1009509877192
0.9573651651538	1.1011590819372
0.9575119041311	1.1013474830862
0.9576376227058	1.1015949337848
0.9577301698365	1.1017524332805
0.9578340037675	1.101899333036
0.9579335023487	1.101909164169
0.9580428070738	1.1019192869369
0.9581759695087	1.1023190249406
0.9582952783806	1.1025832713382
0.9583985538215	1.1026426307058
0.9585399815104	1.1026433614557
0.9586194499296	1.1026963695671
0.9587154984911	1.1027714481515
0.9588589046636	1.1030311786566
0.9589912147985	1.1032975820519
0.9590804540435	1.1033438882722
0.9592077368285	1.1034177564784
0.9593307023823	1.1034764914434
0.9594394072192	1.103610301758
0.959521164418	1.1037073162331
0.9596018144818	1.103712579909
0.959715011183	1.1040988556477
0.9598166073405	1.1041504910353
0.9599122268331	1.104175581965
0.960005748943	1.1042916607478
0.960138088089	1.1050741968575
0.9602716530636	1.1054223760589
0.9604113988342	1.1054331340313
0.9605099991475	1.1054519481016
0.9606412415195	1.1056324181218
0.960742128777	1.1060363558021
0.9608282655186	1.1060910714577

0.9609245531832	1.1063358719571
0.9610807604632	1.1065179585977
0.9612104241716	1.1065569638241
0.9613407872139	1.1070775444377
0.9614345638138	1.1071231950779
0.961557718169	1.1072608027192
0.961673431493	1.1073346826602
0.9617955977181	1.1073450221545
0.9618835170134	1.1075754129087
0.9620065366173	1.1076347252011
0.9621225956431	1.1077445477791
0.9622129950801	1.1077775698908
0.9623025250523	1.1078235429133
0.962460468659	1.1082430058347
0.9625833225168	1.1083862254106
0.9626778034055	1.108668946735
0.9628025822195	1.1088365774413
0.9628945748361	1.1089115791199
0.9630048410195	1.1090949831188
0.9631053296933	1.1093105491162
0.9632199954127	1.1095368893219
0.9633467426304	1.1097575250024
0.9635003150539	1.1103771279319
0.9636091369125	1.1104729535189
0.9637027336943	1.1105340609688
0.9638484895207	1.1105824643754
0.9640058515235	1.1108259823563
0.9640968639832	1.1110022648944
0.9641904496829	1.1111207227439
0.9642780032853	1.1112412963526
0.9644095008039	1.1114796958551
0.9644819534658	1.1114881233674
0.9646181346586	1.1115451477469
0.9647560789312	1.1117110131481
0.9648649673333	1.1117790010661
0.9649651578858	1.1120157266037
0.9650689545253	1.1120835428323
0.9651578982143	1.1121517393455
0.9652463690722	1.1123618545721
0.965372618389	1.11265998111
0.9654694186122	1.1127379239917
0.9656104954263	1.1128801198468
0.9657371319408	1.1129009240033
0.9658326838104	1.1131696280846
0.9659472439565	1.1134407691008
0.9661589173353	1.1141791043474
0.966258889061	1.1142882477211
0.9663813187117	1.1144851133816
0.9664545416346	1.1144961927514
0.9665341841179	1.1145721958795
0.9666564816165	1.1149157251413
0.9667191917335	1.1150341915493
0.9668321418716	1.1152506531085
0.9669247177641	1.1152637514867

0.9670462324211	1.115521140433
0.9672114469343	1.1157789770211
0.9673208644387	1.1158584300122
0.967411389001	1.1158668858021
0.9675516034045	1.1162796818506
0.9676821666618	1.1165977871178
0.9678086754122	1.1166540174392
0.9679085587513	1.1169087553838
0.9680115927278	1.1169580708191
0.968111401394	1.1170443170114
0.968231401474	1.1173159426068
0.9683302827663	1.117317151269
0.968426212223	1.1173637497313
0.9685290352188	1.1174544245007
0.9686062259515	1.1175828673292
0.96868821655	1.1176279316353
0.9687929115238	1.117775325603
0.9689574747105	1.1179193421415
0.9691566764758	1.1188070507071
0.9692593479752	1.119048747996
0.9693761051313	1.1192459625686
0.9695189707159	1.119433275698
0.969642957036	1.1194474381598
0.9697858330178	1.119914517439
0.9698842828419	1.1199152361968
0.9700143333134	1.1200903908875
0.9701301553937	1.120253191619
0.9702419193015	1.120368178874
0.9703372827951	1.1204402802719
0.9704674881667	1.120535607536
0.9705774815293	1.1206765509465
0.9706875471896	1.1208084087311
0.9707910163839	1.1209204480669
0.9708779436225	1.1210832073147
0.9709888094419	1.1212910322776
0.9711368709077	1.1216843545522
0.9712639672473	1.1218806803921
0.9714050253557	1.1221593628199
0.9715526387103	1.1223223406985
0.971662224521	1.1223893932191
0.9717831197066	1.1225072376174
0.9719062639211	1.1226843995752
0.9720101560652	1.1227512586703
0.9721250027636	1.1227737633698
0.9722477392719	1.1229581237189
0.9723570756618	1.1231083132201
0.9724952320656	1.123132602836
0.9726199565365	1.1232076420413
0.9727059199665	1.1232340833063
0.9728435765178	1.1233613801046
0.9729447313829	1.1233629670262
0.9730315120147	1.123414747359
0.9731290892624	1.1237497209467
0.9732210090904	1.1238181611968

0.9733374337023	1.1240184598804
0.9734868557554	1.1245002055686
0.9735734013141	1.1247407769989
0.9736961045931	1.1247834152489
0.9738281775709	1.1250802763155
0.9739659768007	1.1250852543673
0.9740783032804	1.1250865311663
0.9742012418147	1.125163756518
0.9743154218395	1.1253787482716
0.97443997452	1.125579835056
0.9745274146415	1.1255963538649
0.9746754194188	1.1259614953948
0.9747808135581	1.1259905331627
0.9748800296836	1.1260064068516
0.9750284848666	1.1264185616234
0.9751405479391	1.1265546647421
0.9752675092282	1.1265614846627
0.9753833263572	1.1266008528467
0.9754688574268	1.1267748789984
0.9755894530253	1.1269331823529
0.9756944803252	1.127148610686
0.9758017389334	1.127395446214
0.9759144688535	1.1274321134842
0.9760063293572	1.1275067954043
0.9761217021484	1.1275740550531
0.9762231376821	1.1276054132635
0.9763389656973	1.1276748428404
0.9764232674042	1.1277466094554
0.9765364750602	1.1277474424504
0.976605012358	1.1278082942037
0.976703820904	1.1278158587144
0.9768382549403	1.1278855342216
0.9769185882011	1.1279626771417
0.9770107003227	1.1280914753957
0.9771373464566	1.1281522305409
0.9772408891068	1.1281525802934
0.9773222503606	1.1282299584155
0.9774517959698	1.1282739237379
0.9775350376861	1.1283144809411
0.9776225453881	1.1283179624704
0.9777374736722	1.1283333015777
0.9778925638403	1.128431547744
0.9780812890451	1.1287756487451
0.9781624713163	1.1288247448928
0.9782601912416	1.128861879492
0.9783438341574	1.1288929032293
0.978437433608	1.1289407229341
0.9785335725102	1.1290539189034
0.9786554797155	1.1292341879467
0.9787862450145	1.1292355266894
0.9789080044558	1.1292416146463
0.9790154015949	1.1296942201986
0.9791215844025	1.1298619522268
0.9792209692021	1.1299391945543

0.9793898280347	1.1300308739207
0.9795542483492	1.1300920715672
0.9796418014803	1.1302243051887
0.9797556537568	1.1303935983984
0.9798879294287	1.1307353387048
0.9800517671872	1.1309414463895
0.9801643668267	1.1310168927452
0.9802555309218	1.1310797738698
0.980366344177	1.1310928833404
0.9804816906859	1.1311212799845
0.9805500008805	1.1312037198011
0.9806441084416	1.1313265066163
0.9807433770297	1.1313735455782
0.9808537814812	1.1314607824102
0.9809713761039	1.1315949789232
0.9810943780458	1.1316379032474
0.9812229523573	1.1318150398
0.9813301206231	1.1318642796752
0.9814111335696	1.1320595564388
0.981516795075	1.1322111220609
0.9816273828642	1.1323054573171
0.981765000593	1.1323842303676
0.9818867515659	1.1325999474671
0.9819470815557	1.1326139810135
0.9820638380835	1.1326148553226
0.9821830703826	1.1329242108771
0.9823021387761	1.1329330405138
0.9823965112163	1.1330243371231
0.9825394951107	1.1330823769711
0.9826958170622	1.1331377685045
0.9828381886933	1.1333729700709
0.9829697126973	1.1336673504763
0.98311426156	1.1337366674203
0.9832557630425	1.1339113635007
0.983388289814	1.1340614928953
0.9835077932964	1.1342070342419
0.9836149028221	1.134452606133
0.9837270820108	1.1345966580081
0.9838335038497	1.1348538494982
0.9839558001972	1.134917454492
0.9841036911949	1.1351580672291
0.9841834995604	1.1351628692498
0.984304828292	1.1352909558836
0.9843868658276	1.1353689002292
0.9844899256182	1.1353989213987
0.9846014623527	1.1356157232329
0.9847398292445	1.1357835945308
0.9848438610711	1.1359353045906
0.9849481699936	1.1360176487252
0.9850526081674	1.1360532544734
0.9851824541198	1.1361042116931
0.9853010497293	1.1361124331141
0.9854412779348	1.1362940522328
0.9855837904241	1.1363249191557

0.9856932647326	1.1364549730615
0.9858201881925	1.1365604265321
0.9859477926003	1.1368787887095
0.986042675507	1.1369951295937
0.9861391668343	1.1370679970243
0.9862373721261	1.1371237997321
0.9863195383128	1.1371377398033
0.9864502213278	1.1371870445795
0.9865079113206	1.1372129175137
0.9866271740154	1.1372169044846
0.9867666290548	1.1373707363476
0.9869031079022	1.1375385878022
0.9870195424788	1.1375873602569
0.9871664660275	1.1377319832043
0.9872634374332	1.137794176968
0.9873403429381	1.1378060744015
0.9874691826365	1.1381984734593
0.9875712234783	1.1384516149625
0.9877155708542	1.1391608652136
0.9878176219624	1.1392366544928
0.9879568065078	1.1394721204117
0.9881006551085	1.1396938967973
0.9882143736015	1.1397730755553
0.988376680886	1.1399094353413
0.9885103230351	1.1400395511856
0.9886255445793	1.1401875907233
0.9887900553803	1.1411376460296
0.9889096034477	1.1413274479378
0.9890372243694	1.141637750824
0.9891494418501	1.1416661697939
0.9892550347482	1.1417378601147
0.9893803743766	1.141854076212
0.9894989651438	1.1418731263661
0.9896372362622	1.1419455107153
0.9897898417069	1.1421722820798
0.9899215564207	1.1422549202265
0.9900462116384	1.1424824509082
0.9901342153981	1.1425149106803
0.9902787875123	1.1425700640122
0.9904789488967	1.1425755449458
0.9905991642305	1.1425874438559
0.9907091580811	1.14279284222
0.9907831620104	1.1429070635269
0.9909402824784	1.1429786186034
0.9910415375946	1.1430067754258
0.9911300300256	1.1431318249572
0.9912731605856	1.1431762749824
0.99140044973	1.1432028075516
0.9914808174608	1.1435362325823
0.9916274811413	1.1435414745852
0.9917618212058	1.1435416066758
0.9918589017868	1.1435830360236
0.9919616095303	1.1435968454848
0.9920652458391	1.1436585444867

0.9921702104461	1.1438065610712
0.9923482754555	1.1442441676156
0.9924591622408	1.1443575175455
0.9925962916466	1.1445568799472
0.992695572003	1.1449347204485
0.9928031184773	1.144975682159
0.9928909399173	1.1450029169983
0.9930312571619	1.1450776985345
0.9931343097595	1.1451316783227
0.9932738959288	1.145242151237
0.9934057872546	1.1456017402196
0.9935342378121	1.1457053699812
0.9936626021868	1.1458498789246
0.9937793530799	1.1458509960232
0.9938909884246	1.1458952470639
0.9939815769036	1.1459450630062
0.9940893442155	1.1459521324853
0.9941943046029	1.1460103552087
0.9942792864944	1.1460816980411
0.9944129190673	1.1461295481385
0.9945064754265	1.1462746331742
0.9946216428336	1.1464427960947
0.9947179607509	1.1465410813056
0.9948201743336	1.1465972643638
0.9949251629753	1.1466183277704
0.9950142618838	1.1466575890075
0.9951264974161	1.146663221289
0.9952676660801	1.1466634103256
0.9953499831685	1.1466835891545
0.9955013143056	1.1469834621813
0.9956084388177	1.147048264359
0.9957090982561	1.1470641015493
0.9958240636671	1.1471278852854
0.995945655898	1.1471730656561
0.9960409099224	1.1472664932023
0.9961370634367	1.1474428919615
0.9962802353651	1.1475440405008
0.9964098352194	1.1477994783143
0.9965861018615	1.1487774250485
0.9966786254447	1.1491230293732
0.9967655258231	1.1492694104971
0.9968902039768	1.1493723999244
0.9970224210511	1.1495707423064
0.9971772867804	1.1496896336919
0.9972851443547	1.149942851166
0.9974103102374	1.1503103628332
0.9974962280862	1.1503905262558
0.9976078483435	1.1506341392821
0.997716412449	1.1507005528361
0.9978315108783	1.1510896948912
0.9980150682829	1.1512942258949
0.9981096865488	1.1512986050938
0.9982411202794	1.1513029417509
0.9983699058597	1.1514622941117

0.9984983719636	1.1514838979021
0.9986084347073	1.1515421568241
0.9986701632093	1.1515539346077
0.9987899135876	1.1516999121508
0.9989354396378	1.1517412979049
0.9990726723518	1.1521802750946
0.9991647530901	1.1523491676684
0.9992446649089	1.1524973873364
0.9993411358812	1.1526177213067
0.9994500872015	1.1529483283113
0.9995573529631	1.1530059533203
0.9996847333186	1.153054413135
0.9998200245886	1.1531169723016
1.0000037853454	1.1536620421005
1.0001111111737	1.1536842159263
1.0002379821638	1.1540794751778
1.0003395783394	1.1541388928554
1.0004657729165	1.1542374124074
1.0006005283103	1.1543717525429
1.0007375563045	1.1548397282813
1.0008723004643	1.1549196985492
1.00097039553	1.1550857793557
1.0010944929938	1.1551075164507
1.0011990840217	1.155116989109
1.001290987201	1.1551604716248
1.0013863929305	1.1551993503409
1.001522162229	1.1553132438073
1.0016263930229	1.1555155338802
1.0017541090949	1.1555982602678
1.0018695216653	1.1557337661282
1.0020050058788	1.1557558514542
1.0020860159615	1.1558945656058
1.0021832675372	1.1559333624419
1.0022954337164	1.1561020784447
1.0023974282975	1.156119800813
1.0025077274064	1.1561280749054
1.0026273424618	1.1561732070646
1.0027579699256	1.1561819748173
1.0028948891276	1.1564453110113
1.002969051222	1.1564934699621
1.0030687317863	1.1565014715399
1.0031817149427	1.1565104797947
1.0033065782568	1.156744663914
1.0034345363846	1.1567482313638
1.0035509768625	1.1568207651739
1.0036360390249	1.1568446929379
1.003744865143	1.1569971478487
1.0038618172024	1.1570600818383
1.0039542867737	1.1573206268478
1.0040426178556	1.1574919880746
1.0041315780389	1.1576224890336
1.0042419448794	1.1576464513995
1.0043791532283	1.1579179274879
1.0044702560879	1.1581043977456

1.0045797549312	1.1582791478986
1.0046847415571	1.1584197304939
1.0047856045693	1.1585010661905
1.0049314362137	1.1591719757962
1.0050400872728	1.1594085207586
1.0051989197543	1.160602826341
1.0053259744013	1.1606970615623
1.0054908191484	1.1610204690764
1.0056026370874	1.1610988354544
1.0057588790212	1.1611372572031
1.0059014198034	1.161151603709
1.0060264486551	1.1613231120259
1.0061593409547	1.1616143370699
1.0062696003362	1.1616972087642
1.0064158904531	1.1619508871342
1.0065646557386	1.1621968294806
1.0066964830605	1.162326495814
1.0068024016931	1.162450488344
1.0068905631008	1.162526313554
1.0070370769084	1.1625920034063
1.007144789697	1.162769904698
1.0072681513381	1.1630790541198
1.0074051395668	1.1631102034143
1.0075118121627	1.163252264219
1.0076754963223	1.1633336340914
1.0077971570703	1.1634274510711
1.0078847624324	1.163630255564
1.0079968904451	1.1637212322001
1.0081177287228	1.1638617660833
1.0082807702493	1.1643925061053
1.008408684991	1.1646868193459
1.0085395996935	1.1647188259706
1.0086859187291	1.1648375863707
1.0088088081637	1.1649359141183
1.0089378654543	1.164966090032
1.0090214930065	1.1650281760231
1.0091029382585	1.1651948448536
1.0092083629155	1.1653194921769
1.0093278005005	1.1653875971319
1.0094562811511	1.1658249552965
1.0095742516759	1.1659675542432
1.0096440120946	1.1660044496531
1.0098215805898	1.166009139671
1.0099362581111	1.1661842997674
1.0101113082145	1.1666134011354
1.0102241024313	1.1670896028364
1.0103569881952	1.1671115610643
1.0104964324107	1.1673097210356
1.0106402352564	1.1675462540572
1.0107981080616	1.167566452741
1.0109753053969	1.1676110083369
1.0111077008497	1.1676139114782
1.0112360996143	1.1676184977771
1.0113119568984	1.1677280119688

1.0114175725306	1.1677568258908
1.0115622685999	1.1683266319038
1.0116636565595	1.1683657391464
1.0117755320489	1.1687537449274
1.0118630399063	1.1687871622042
1.0120056345816	1.1689409790473
1.0121356560027	1.1689571291338
1.0122586490594	1.1690234334441
1.012361984889	1.1691958640207
1.0124581429112	1.1692334362455
1.0125944616619	1.1693752173761
1.0127354402898	1.1695344879644
1.012845306691	1.1699426764611
1.0129168720606	1.1701482436315
1.0130008198426	1.1702089972557
1.0131183822608	1.1702280471661
1.0132341377532	1.1704135254713
1.0133511322343	1.1705347636556
1.0134384150661	1.1706008362784
1.0135884567565	1.1713951554106
1.0136809496526	1.1714754601629
1.0138297438645	1.1717568338658
1.0140062387107	1.1718664427443
1.0141503503399	1.1719295107678
1.0143164975921	1.1720475587447
1.0144352074503	1.1720851331763
1.0146021470183	1.1722287637986
1.0147320889511	1.1722519144091
1.0148114610315	1.1724126659583
1.0149079439935	1.1724131930689
1.0150029828494	1.1724588427783
1.0151550903545	1.1725122420076
1.0152535872651	1.1726039175885
1.0153819899277	1.1726258053274
1.0154667954107	1.172643147706
1.0156214122785	1.1728410987099
1.0157467507537	1.1730767466112
1.0159072838296	1.1735083654432
1.0160148886869	1.1736026535321
1.0161491303957	1.1738055257669
1.0162872489671	1.1738917886729
1.0164043673377	1.174106443369
1.0165516609587	1.1742458677083
1.0166747338251	1.1743653398125
1.0167782355297	1.1744362843359
1.0169229213494	1.1745707976848
1.0170335590356	1.1746302227294
1.0171640963544	1.1746473045727
1.0173184005605	1.1747871011511
1.0174345367363	1.1749195557724
1.0175437697009	1.1753252091842
1.017659129632	1.1753489237827
1.0177897949181	1.1754621460181
1.0179229237908	1.175678548956

1.0180470629168	1.1756860278696
1.0182002050425	1.1761662411053
1.0183540088036	1.1767278903321
1.0184778969982	1.1768965790091
1.0186154740338	1.1771650991692
1.0187170635934	1.1772638021055
1.0188084856308	1.1773250216878
1.0189649467682	1.1773266243072
1.0191149856407	1.1773923513006
1.0192075537827	1.1776410999737
1.0193124860618	1.1777621633276
1.019452577394	1.1780330570121
1.0196245131872	1.1780885060601
1.0197323383789	1.1782663362335
1.0198383159567	1.1784636023173
1.0199385673093	1.1785311381715
1.0200362052662	1.178579979092
1.0201355951948	1.1785972310851
1.0202787532372	1.1790383658885
1.0203835335898	1.1790579354116
1.0204821337157	1.1791390847483
1.0206025060779	1.1792820051625
1.020787402754	1.179485592552
1.0209303469438	1.1796496531976
1.0210469506445	1.1797653557117
1.021196799863	1.1798529196083
1.0213358771279	1.1798844678246
1.0214898001103	1.1802417272681
1.0215982101067	1.1802432301506
1.0217638610389	1.1810959539162
1.0219006722299	1.1812815085815
1.0220495849473	1.1813339086169
1.0222286649176	1.1819488112807
1.0223523928966	1.1821999534847
1.0224291966485	1.1822452522367
1.0225492407773	1.1823177753414
1.0226802030423	1.182365601255
1.0227990919717	1.1824122100194
1.0229147962329	1.1824240968972
1.0230821650379	1.182451655118
1.0231647366845	1.1824653710439
1.0232865792727	1.1826389924337
1.0233664817022	1.1827526151273
1.0235170404048	1.1830649628146
1.0236631886552	1.1832500434306
1.0237599830261	1.1833201594769
1.0239260665581	1.1833968932793
1.0240439728841	1.1835493382912
1.0241581287535	1.1836110831078
1.0242340901295	1.1836930983638
1.024314235999	1.1837451984325
1.024453807476	1.1837702002457
1.0245886694887	1.1840446913399
1.0247437215176	1.1841117604493

1.0248604418983	1.1841786406993
1.0250249677879	1.184192480521
1.0251552951313	1.1843935499861
1.0252584396782	1.1847373087092
1.025363939474	1.1847776095024
1.0254827622425	1.1849702804002
1.0256427134965	1.1851610249323
1.0257496812888	1.1851973088572
1.0258468571398	1.1852787395325
1.0259541591214	1.1853300238928
1.0260876040384	1.1854219823106
1.026228379821	1.1854381274337
1.0263470725297	1.1854607438211
1.0264727955078	1.1855479608153
1.0265922470632	1.1860408373682
1.0267072336657	1.1860734307457
1.0268077907408	1.1862305487964
1.0269251022916	1.1863139056259
1.0270349781053	1.1864469627917
1.0271499983491	1.1865983448424
1.0272569904141	1.186704749051
1.0273641496313	1.1867106315321
1.0275171449002	1.1868023700683
1.0276437942097	1.1871018455027
1.0277516681514	1.1871241313043
1.0278561269865	1.1871764399302
1.0280057418749	1.1873401801874
1.0281470981079	1.1873450307132
1.028274163735	1.1873726152883
1.0283881034158	1.1873825079058
1.0285254561731	1.1875730256163
1.028671438585	1.1877987647917
1.0287916322304	1.1878405517119
1.0289007211295	1.1879576296647
1.0290240657506	1.1881499402439
1.0291076055746	1.1881661937121
1.0292453875227	1.1884017856898
1.0293922801749	1.1886185127719
1.0294928784931	1.1886299048196
1.0296407199304	1.1886405682189
1.0297457886973	1.188675877844
1.0298453794847	1.1888123746458
1.0299856631053	1.1888682291933
1.0301311233251	1.1889021181943
1.0303016993511	1.1890552229488
1.0304014576372	1.1891276722991
1.0305513304154	1.1892648498127
1.0306919913683	1.1893785590531
1.0308166791788	1.189389715162
1.0309546280916	1.1894012299713
1.0310887292786	1.1894849735711
1.0312092228774	1.1897616856801
1.0313272127188	1.1898149421656
1.0314092257632	1.1899251214141

1.031509982756	1.1899435983731
1.0315838733697	1.1901792521899
1.0317248247078	1.1902360826659
1.0318522468744	1.1902413280617
1.0319320045574	1.1903779078989
1.0320343967453	1.1903812818212
1.0322002314043	1.1908081700919
1.0323904546743	1.1910195773086
1.0325071628969	1.1911442442323
1.0326348354307	1.191823165118
1.0327139488503	1.1918385953472
1.0328445776807	1.1918565948837
1.032965441623	1.1919290557177
1.0330682129344	1.1919944298724
1.0331904303734	1.1921480961355
1.0333195657728	1.1921813260147
1.0334333443295	1.192232555776
1.0335673807579	1.1923497217889
1.0337181697528	1.1925437281819
1.0338413120256	1.192718092116
1.0339941731725	1.1928498988315
1.034108629774	1.1929750468849
1.0342534139122	1.1930556393257
1.0344251613802	1.1931358313782
1.0345746351361	1.1931782264451
1.0347304152959	1.1935641558745
1.0349001966785	1.1937908520143
1.0350222767504	1.193947335328
1.0351319409433	1.1940626698399
1.0352410958073	1.1943162363635
1.0353699378385	1.1948825142361
1.0354848386127	1.1950275621956
1.0356239064086	1.1951731487515
1.0357433533205	1.1955041847059
1.0358350404133	1.1955316238867
1.0359796997678	1.1956352466718
1.0361118815199	1.1956917511203
1.0362384137541	1.1958142254686
1.0363290672401	1.1958461916696
1.0364409715593	1.1961784968963
1.0365852722751	1.1962327144776
1.0367366401953	1.1963597275037
1.0368409234949	1.1966370135731
1.0369687475709	1.1969612062326
1.0370834230757	1.1971513733952
1.0372240625214	1.1972572825382
1.0373757744627	1.1973364528804
1.0374884503155	1.197400097138
1.0375934285174	1.1974084261308
1.0377108604362	1.1979630421528
1.0378477483341	1.1981455389431
1.0379521664065	1.198267430348
1.0380469403407	1.1983407115748
1.0381465645506	1.1984531684564

1.0382524018378	1.1987489516897
1.0383439302527	1.1987716184121
1.0384494664308	1.1988589050142
1.038549854419	1.1989394488958
1.0386907230728	1.1990495858445
1.0388629209463	1.1990654660921
1.0390338142166	1.1996210873707
1.0391313920539	1.199641895074
1.0392267036821	1.1997980599725
1.0393348661197	1.1998654014092
1.039471236943	1.199890893001
1.0395865180548	1.1999747639498
1.0396852395512	1.1999989557324
1.0398468293554	1.2007612409714
1.0399573136809	1.2008086598698
1.040080116517	1.2008146298087
1.040199370995	1.2009920629003
1.0403132727637	1.2011034103678
1.0404057849794	1.2011671088868
1.0405487074574	1.201174805228
1.0407003947853	1.2014229800841
1.0408410290777	1.2015842862451
1.0409827587789	1.2017994399484
1.0411040994188	1.2018861758369
1.0412305821241	1.2021221969143
1.0413307554642	1.2022508008353
1.0414630441126	1.2024309524577
1.0416060754409	1.2025199979023
1.0417770517909	1.2025860213616
1.0418736764389	1.202764487747
1.0420084107704	1.2028136627175
1.0421505756894	1.2028556751543
1.0422499208609	1.2028674123426
1.0423806750945	1.2030169678306
1.0424576200082	1.2030291204842
1.0426089317842	1.2030373049607
1.0427084290051	1.2030575936997
1.0428439539681	1.2031307474118
1.0429578041112	1.2031990730962
1.043095096936	1.2033335533716
1.0432206013071	1.2034992185573
1.0433340590324	1.2036562852787
1.0434879785874	1.2037264825792
1.0436380569592	1.2037319233032
1.0437667797685	1.2038638773805
1.0439110431198	1.204167003349
1.0440053725295	1.2042002824805
1.0441645786295	1.2042124293967
1.0442926900184	1.204293820197
1.0444209778374	1.2043926941227
1.0445248368392	1.204395557471
1.0446296676841	1.204695022133
1.0447951361013	1.2047797080654
1.0449546426834	1.2052107450558

1.0450554357496	1.2054131705035
1.045179924293	1.205479721195
1.0452926839659	1.2058208524858
1.0454183472227	1.2058724796626
1.0455645661962	1.2059089603355
1.0456600201077	1.20601013269
1.045749223738	1.2060802812841
1.045935899432	1.2070207227201
1.0460349741909	1.2071103014334
1.0461800701065	1.2072209371651
1.0463173223171	1.2072680625868
1.0464556037936	1.2072716968421
1.0465801064841	1.2073069225747
1.0467311606855	1.207517401384
1.0468924016786	1.2076321586746
1.0470183840794	1.2076412916285
1.0471260807768	1.2077523771021
1.0472360364897	1.2078162752022
1.0474076831653	1.2080912545981
1.0475639097099	1.2087270082305
1.0476843218158	1.208765635283
1.047818837017	1.2088342360853
1.0479356025792	1.2089538056027
1.0480549141376	1.2090988965106
1.0481478635209	1.2091187474878
1.048271600236	1.2091305588493
1.0484199176669	1.2092773110213
1.0485192111492	1.2095593622552
1.0486424437012	1.2096107126784
1.0487966647326	1.2102693109514
1.0489549608623	1.2102696837379
1.0490945178812	1.2104582058661
1.0492442688909	1.2104873377587
1.0493787058631	1.2105045002824
1.0495299727396	1.2106452686183
1.0496122605801	1.2107231996082
1.0497342139235	1.2107785663058
1.0498769259396	1.2109218463079
1.0500387971959	1.2109787682598
1.050150541595	1.2111150658022
1.0502522590123	1.2112408285612
1.0503641289546	1.2114250150123
1.0505142763692	1.2115163356823
1.0506003457097	1.2115309056772
1.0507209072573	1.2117186554272
1.050893619241	1.2121780743123
1.0510287266731	1.2123951478109
1.051175440206	1.2125645790414
1.0512975399242	1.2127869862475
1.051447694692	1.213009537228
1.051570798621	1.2131667998064
1.0517005058271	1.21318225709
1.051816356621	1.2132002283088
1.0519307751026	1.213301044547

1.0520494931878	1.2133286629169
1.0521995946635	1.2135301594323
1.0523269578637	1.2136160322482
1.0524529784266	1.2138841233199
1.0525689703403	1.2139815855839
1.0527042767626	1.2140248776084
1.0528386189723	1.2140552691816
1.0529614127968	1.2141400014384
1.0530968674303	1.2143216151249
1.0531668182892	1.2144392360061
1.053305039817	1.2145371538055
1.053416108113	1.2146016957892
1.0535789540402	1.2146415294324
1.0537079311318	1.2147693706047
1.0538709802353	1.2153142574274
1.0540237688579	1.2153416279435
1.0541529189126	1.2155637378885
1.0542827008249	1.2158464754675
1.0544007111681	1.2159767524565
1.0545121822562	1.2161067408961
1.0545897631992	1.2161609087861
1.0546884319161	1.2162324813791
1.0548354073729	1.2164381624626
1.0549242205002	1.216501003537
1.0550871937725	1.2167844430435
1.0552366239546	1.2167858430634
1.0553919335291	1.216925990311
1.0555694672875	1.2170011223007
1.0557591231327	1.2170138143704
1.0558579675045	1.2170217389768
1.0559578615479	1.2170447846316
1.0561178248648	1.2171774113544
1.0562291651151	1.2172170156539
1.0563533662655	1.217526153134
1.0564779301618	1.217574573664
1.0566581415184	1.2176242810668
1.0567655176154	1.2176512405632
1.0569352429104	1.2177279003861
1.0570247719436	1.2177796610293
1.0571728670595	1.2181182410626
1.0572645598337	1.2181203163914
1.0574014056022	1.2182264933854
1.0575305679328	1.2183586785228
1.0576386468665	1.2184437269421
1.0577536234276	1.2185762996416
1.0578828497796	1.2187130850819
1.0580314120265	1.2190356246007
1.0581249804313	1.2191234598838
1.0582187000861	1.2191995828107
1.0583723198069	1.2193506934583
1.0585540442408	1.2196702952473
1.0587444494485	1.2203886777841
1.0588959807096	1.2205587045904
1.0590229128134	1.2206513446258

1.0591685311367	1.221605091197
1.059251851286	1.2217332242978
1.059394307436	1.2219234235953
1.0595729461208	1.2227419296599
1.0596980347264	1.2228849717964
1.0598406915718	1.2229345684366
1.059929786749	1.2233664142277
1.0600259656501	1.2235037773199
1.0601703570591	1.2235501064496
1.0603006192485	1.223582721539
1.0604096771679	1.2237952186861
1.0605661689601	1.2239049331276
1.0606723757956	1.2239330863305
1.0608061026776	1.2242864738469
1.0609436200575	1.2243121328352
1.061036598045	1.2243590468281
1.0611900878336	1.2245399822924
1.0613408827172	1.2246596863867
1.0614767136414	1.2249220773967
1.0616030978303	1.2250854375676
1.0617099386727	1.2251576136116
1.0618493848058	1.2255441136215
1.0620031539891	1.2255629782645
1.0621367948947	1.2257244213327
1.0622609651464	1.2259095848142
1.0624533806443	1.2266695820432
1.0625806194963	1.2268041361338
1.0627323831523	1.2273808156374
1.0629284630414	1.2274051546355
1.0630677270257	1.227504659824
1.0632241986961	1.2275856313645
1.063378377566	1.2279118518468
1.0635568480121	1.2285207030459
1.0636432389713	1.2285933291778
1.0637462356952	1.2286384758411
1.063865515992	1.2288046114055
1.0640113998155	1.2292383838546
1.0641364517738	1.2293391004781
1.0642597738007	1.2294007903289
1.0643963259287	1.2294105717901
1.0645320355888	1.229484363251
1.0646568719029	1.2295024354969
1.0648401751412	1.2300669420237
1.0649963913716	1.2301780847928
1.0651355468993	1.2301932378493
1.0652954113823	1.2309271543201
1.0653999854955	1.2309933052721
1.0655871900437	1.2311298134714
1.0657268057449	1.231145681661
1.0658567116778	1.2311926459528
1.0659695347462	1.2312894134664
1.0660915204487	1.2316496425266
1.0662211818695	1.2317524760533
1.0663935966289	1.2319363331219

1.0665264387828	1.2320920303913
1.0666562324642	1.2322834897081
1.0668693660379	1.2326607394014
1.0670543970504	1.2327210137309
1.0671668597975	1.2327341798856
1.0673333481105	1.2329334298352
1.0674487120366	1.2329493598997
1.0675562751963	1.2330271248883
1.0676951654534	1.2330932283777
1.0677946374319	1.2331613195538
1.0679338644895	1.2334406855382
1.0680644931306	1.2337127423721
1.0681891063328	1.233845816095
1.0683222043771	1.2338587141084
1.0684392646227	1.2339200213645
1.0685180466017	1.2340001438772
1.0686685003547	1.2342302485511
1.0687720445553	1.2342463571853
1.0688864396232	1.2342680520151
1.0690310577655	1.2344220494119
1.0691589685209	1.2349452701611
1.0693532287871	1.235035604016
1.0694737339419	1.2350443229859
1.0696119024097	1.2352325867581
1.069771997303	1.2353754718474
1.0698881304136	1.2354738303
1.0700628631872	1.2357803399956
1.0701561423511	1.235936738296
1.0703374174659	1.235993336197
1.0704714834919	1.236047082666
1.0706166915741	1.2360716346603
1.0707432398368	1.2363539924106
1.0709293483634	1.2364369360277
1.0710838314485	1.2368591413833
1.0712365175653	1.2370028546177
1.0713677252955	1.2372053741723
1.0714979577194	1.2373750627883
1.0716124371515	1.2375194963614
1.071736625692	1.2376685470247
1.0718708597697	1.2379660886157
1.0720560927954	1.2380635116906
1.0721619178301	1.2381696212666
1.0722652523838	1.2383316249514
1.0723934595236	1.2386638246722
1.0725209743614	1.2389276597514
1.0726678172656	1.2393101438345
1.0728495207663	1.2395478974956
1.072994453781	1.2397164846602
1.0730948091194	1.2397229260306
1.0731954003402	1.2398526653191
1.0733364234281	1.2400305788288
1.0734688646875	1.2400876506826
1.073601169495	1.2403127671812
1.0736981939148	1.240413346836

1.0738405311123	1.2405643748749
1.0739138254914	1.2407225971783
1.0740528820827	1.2408921532814
1.0741801593892	1.2412176130083
1.0743469363808	1.2412294432042
1.0744888299002	1.2414095215941
1.0746382078445	1.2417127674813
1.074751483937	1.2418025818773
1.0748425133199	1.2419440586533
1.0750003639595	1.2422954501265
1.0751821205014	1.2425545333637
1.0752868435325	1.2426430692936
1.0753986902751	1.2428359944094
1.0755217551551	1.2430259180442
1.0756794162803	1.2430325243497
1.0758421101572	1.2432127542854
1.075990462451	1.2434204298214
1.0761211968853	1.2438160553135
1.0762093824076	1.2438276703151
1.0763619066396	1.244403176258
1.0764986568789	1.2445631769899
1.0766131499268	1.2448199844084
1.0767367820042	1.2449181617599
1.0768545627302	1.2449507102941
1.0770394556356	1.2449829305904
1.0771283753164	1.2449888393162
1.0772438398634	1.2454611527016
1.0773720522507	1.2455049111327
1.0775139096404	1.2458538195804
1.0776515114005	1.246036434171
1.0777921389058	1.2462675887448
1.077901619708	1.2463658895242
1.0779985778123	1.2465436039255
1.0781132442115	1.2465672189595
1.0782792288317	1.246862349573
1.0784119460304	1.2471578934046
1.078605164691	1.2474184956351
1.0787395807347	1.2475132198173
1.0788576482128	1.2476458775973
1.0789591716815	1.2476570394889
1.0790795623092	1.2477487440912
1.0791681959704	1.2477821140695
1.0793185991729	1.2479011235765
1.0794683512293	1.2481309065444
1.0795838463282	1.2482572945479
1.0797021258795	1.2482995063552
1.079855389179	1.2484404143387
1.0799752970679	1.2484875927943
1.0800917736099	1.2486336917919
1.0802183711125	1.2487205910157
1.0803104127568	1.2487277166086
1.0804957076883	1.2489473741893
1.0805893846205	1.2490588750414
1.0807174301597	1.2491180957201

1.080845488022	1.2494302440349
1.0809767012492	1.2494709092474
1.0810997752495	1.2495221410658
1.0812248530726	1.2495514732322
1.0813609573571	1.2496820066476
1.08151656432	1.2500111746235
1.0816502573854	1.2501122792291
1.0817776687689	1.250242097835
1.0819265714909	1.2503916958205
1.082074816017	1.2506095445038
1.0822734374108	1.2510002466679
1.0824207591843	1.2510106099438
1.0826306764905	1.2516500730786
1.0828525880769	1.2517307894312
1.082982995772	1.2518629591142
1.0831297581879	1.2519775592622
1.0832509601221	1.2521023426726
1.0833592907194	1.2522608209191
1.0835389490109	1.2530573035084
1.08367277351	1.2534247725839
1.0837960828723	1.2534454887389
1.0839069673655	1.2537302048844
1.0840301677704	1.2540601661038
1.0841639101245	1.2541495223854
1.0843400634997	1.2543615508839
1.0844322306654	1.2545505394048
1.0845369308062	1.2545819406362
1.08469977128	1.2548635089967
1.0848224966643	1.2550231143794
1.0849559918393	1.2550878245469
1.0851489357491	1.2553223031743
1.0853053026232	1.2553410687467
1.0854382695588	1.2553608933361
1.0856112551273	1.2557868173801
1.0857226897852	1.2559607335699
1.0858406774493	1.2563541751496
1.0859784656732	1.2565151220058
1.0861254318514	1.2566010720897
1.0862306504049	1.2567310091707
1.0864205471398	1.2569683513363
1.0866027641702	1.2573707618039
1.0867145351451	1.2579154227844
1.0868794172674	1.25845768282
1.0870949020034	1.258566025001
1.0871947183081	1.2586744558266
1.0872971931794	1.2589461646847
1.0874123861496	1.2591891041298
1.0875707511131	1.2596332049412
1.0877051030852	1.2597384837783
1.0879001195041	1.2598209457398
1.0880196917274	1.2598315874032
1.0881583676399	1.2598933135062
1.0883038655755	1.260052984549
1.0884171188662	1.2601705982474

1.0885661368226	1.2605131037862
1.0887009098087	1.2605923576679
1.0887994407339	1.260754575305
1.0889671099502	1.2608603124015
1.0891095439399	1.2608801767819
1.0892174054731	1.2609361303936
1.0893866006122	1.2611283284082
1.089546230698	1.2612178395574
1.0896303722546	1.2613388161176
1.0897599599418	1.2614371436889
1.0898574703882	1.2616693253937
1.0899736857063	1.2617136957768
1.0901114317867	1.2617935359659
1.0902431672594	1.2620123132869
1.0904704595915	1.2622605599674
1.0905788063379	1.2627057118952
1.090729926065	1.2628333626413
1.0908993878543	1.2630130079367
1.0910219323243	1.2631369324494
1.0911602247811	1.2631929381145
1.091305760057	1.2632510276111
1.0914221135391	1.2633590332933
1.0915457818018	1.2633978411414
1.091718407033	1.2634872784051
1.0918351003733	1.2635776464893
1.0919645979726	1.2636042799467
1.0920817929513	1.263615424873
1.0922092567613	1.2636265054738
1.09235526584	1.2637879715367
1.0924724086887	1.2638308777911
1.0926346352622	1.2641289800345
1.0927527448078	1.2642687933142
1.0929015041922	1.2644841960909
1.0930154688003	1.2647360865707
1.0931428231212	1.2649554419316
1.0933047332863	1.2651254258191
1.0934695586784	1.2660308165029
1.0936499238748	1.2660919447884
1.0937772037446	1.2664059825511
1.0939284003029	1.2664764701755
1.0940553745701	1.2664807641302
1.0941665942133	1.2668438701102
1.0943043543414	1.2668993431557
1.0944244780524	1.2669642205113
1.0945713478843	1.2669949730426
1.094722652095	1.2671213913864
1.0948309562153	1.2671840195182
1.094941822617	1.2671887910627
1.0950541009176	1.2677367366078
1.0952427392988	1.2678228746172
1.0954069715905	1.2678800289591
1.0955787119614	1.2680532009953
1.0957146112991	1.2681992114109
1.0958810623248	1.2682676185222

1.0960087696467	1.2682866079713
1.0961927719037	1.2684859630129
1.0963386101277	1.2685068701474
1.0964778040605	1.268508607508
1.0966116860255	1.2685094933866
1.0967532812161	1.2686644062561
1.0968871249629	1.2686814458122
1.0970795626505	1.2690962642872
1.0972164178336	1.2692807571421
1.0973703042812	1.2693573972875
1.0974680824012	1.2694287222972
1.0976449631512	1.2695191385774
1.0977850285547	1.2696179088935
1.0978787725591	1.2696263412734
1.0980453280243	1.2697454284693
1.0981684761225	1.269776183488
1.098319140745	1.2701609560311
1.0984162580866	1.2704638810491
1.0985667405599	1.2705492570232
1.0987107048659	1.2706112452258
1.0988567764298	1.270892274922
1.0989580297908	1.2709126022155
1.0990765512678	1.2710811320172
1.0992013739519	1.2711317156471
1.0993429066871	1.2714614554546
1.0995122528263	1.2717201679348
1.0996697296695	1.2720352949652
1.0998615954469	1.2721194432309
1.0999884056641	1.2721564401526
1.1000783814783	1.2723795222766
1.1002237332155	1.2723988769336
1.1003411720108	1.2729299984265
1.1004606202141	1.2732225615565
1.1006374959437	1.2737784307294
1.1007921800044	1.2742221577973
1.1009460694947	1.2742688202775
1.1010875610908	1.2744165116322
1.1012609724658	1.2747735344201
1.1014099463197	1.2747739538231
1.1015236725641	1.2747750612106
1.1016671485783	1.2748232781179
1.1017770512396	1.2748317203596
1.1019187934449	1.2750712369623
1.1020427575936	1.2753436767191
1.1021574483737	1.2753508587788
1.1023178473413	1.2757907668972
1.1024354773885	1.2758372518476
1.102606596951	1.2762231571383
1.1027728337192	1.2767967818347
1.1028989205633	1.2769790487608
1.10299393461	1.276998800406
1.1031162175121	1.2772796342888
1.1032532136134	1.2773392342873
1.1033579543525	1.2774970582248

1.1034737418203	1.2775059525732
1.1036209929136	1.2780565637439
1.1037777343772	1.2780898248375
1.1039023582077	1.2782894120866
1.1040142838086	1.2783683311244
1.1042010846373	1.2787671111143
1.1043578095342	1.2787812026492
1.1044771368019	1.2787960636743
1.1046001963287	1.2788977142581
1.1047144435689	1.2790337935176
1.1048660563094	1.279180180385
1.1050022374485	1.2791832508331
1.1051413175458	1.2794907177309
1.1052798665568	1.2795452452476
1.1054244112065	1.2798637964302
1.1055688515244	1.2799133097835
1.1056773323137	1.2800338027639
1.1058407197312	1.2805138478203
1.1059886411671	1.2806897573809
1.1061464043085	1.280870920978
1.1063091810932	1.2810302378373
1.1064110779669	1.2811700222349
1.1065687021135	1.2813806043835
1.1066942683532	1.2814717687565
1.106868914855	1.2815055035956
1.1069866942442	1.2815525039685
1.1071513555802	1.2818431213626
1.1073259314279	1.2820369122569
1.1075021598209	1.2820467533667
1.1077302664729	1.2831294974102
1.1078577146231	1.2831515535715
1.1079990600457	1.2834944326788
1.1081542421915	1.2836683856783
1.1082932128768	1.283941625379
1.1084602938277	1.2841203111779
1.1085652586109	1.2844267920246
1.1087049034245	1.2846586117652
1.1088575486031	1.2847849864668
1.1090459828637	1.2849584707865
1.1091878699639	1.2851825133647
1.109308690124	1.2853650651659
1.10946692912	1.2856359117501
1.1095659005125	1.2858366953495
1.1096950412309	1.2859611452769
1.109810019764	1.2859649163096
1.1098780875075	1.2859739177415
1.1100027528906	1.2861304850276
1.1101386792161	1.2863382065616
1.1102523409706	1.2866066250362
1.1104200097627	1.2868068672406
1.1106039788144	1.2869640338158
1.1107567847335	1.2872889727249
1.1109582450147	1.2873108229035
1.1111080853932	1.2873150207434

1.1112940200423	1.2875109449972
1.1114195116427	1.287570913446
1.1115317265411	1.2877013864153
1.1116627674027	1.2878313520608
1.1118365941791	1.2879084059794
1.1119726037514	1.288002327095
1.1120962673484	1.2881866134803
1.1122396456237	1.2882080465696
1.1123430106774	1.288257445696
1.1124672308823	1.2885696943415
1.1126380302076	1.2886930650063
1.1128012395912	1.2887528346913
1.1129068648415	1.2887592435218
1.1130808764072	1.288802605167
1.1131823269459	1.2889214794783
1.1132926561934	1.2889262004841
1.1134288448944	1.2892475488041
1.1135553302168	1.2894559904202
1.1136542768928	1.2896051371112
1.113792253877	1.2900761214128
1.113953196577	1.2902054149066
1.1140660754602	1.290320711434
1.1142015006206	1.2905059441825
1.11430580736	1.290529947971
1.1144330956252	1.2907800647528
1.1145528191797	1.2910494609872
1.1147120741185	1.2910760322647
1.1148827897351	1.2912721460947
1.1150576816544	1.2916685227469
1.1151729921757	1.2917764238481
1.1152808353614	1.2919956110003
1.1154439887607	1.2921246429468
1.1156065097642	1.2923043200225
1.1157552649444	1.2926093606406
1.1159352840143	1.2929643030879
1.1160852913863	1.2933112206585
1.1162126107241	1.2934559986574
1.1163317787071	1.2935211111533
1.1164557693732	1.2936663140167
1.1166157221292	1.2936761693612
1.116813005534	1.2938043442451
1.1169428212232	1.2939613870199
1.117076681042	1.2941843835308
1.1172378570919	1.2945662135529
1.1174622179784	1.2945911781918
1.1176139424452	1.2947973270254
1.1176999746748	1.2948828515649
1.1178351274084	1.2949156135804
1.1180219042686	1.2949777705636
1.1181633322147	1.2950464611081
1.1182786974681	1.2953430581901
1.1184122174459	1.2954088073435
1.1185428512441	1.2955502547955
1.1186789790162	1.2956878310235

1.1188053708799	1.2959072523738
1.1189814675284	1.2961497780351
1.1191177230736	1.2962246408173
1.1192385919913	1.2964217362798
1.1193714213054	1.2967531129761
1.1195529179844	1.2968935700848
1.1197042875637	1.2970181471606
1.1198283814237	1.297122722605
1.1199476956855	1.2972292036618
1.1201220669842	1.2973753991544
1.12028981071	1.2974823274319
1.1204362725571	1.2977918655837
1.1206155695396	1.298079314103
1.1207520530027	1.2981097292169
1.12090306105	1.2981406832942
1.1210334924637	1.2982228670447
1.1211963326257	1.2982699669035
1.1213900465982	1.2984348214777
1.1215263593311	1.2984908653734
1.1217267718716	1.2986651017084
1.1218102068217	1.298678818753
1.1219366564111	1.2988986991676
1.122101951066	1.2991063940956
1.1222560866211	1.2994050168842
1.1224146473566	1.2996817332545
1.1225389049623	1.2996888657166
1.1226518464951	1.2997992912864
1.1227503053699	1.2998895238477
1.1228894499667	1.3000792859403
1.1230637750253	1.3000950022964
1.1232425793151	1.3012443048717
1.1234135451558	1.3013556182675
1.1235826384399	1.3013653977618
1.1237387687462	1.3013993294989
1.1238726271531	1.3014978607457
1.1240124516574	1.301901434154
1.1241633331493	1.3019563631894
1.1242979309463	1.3021876273831
1.1244287313322	1.3023841597679
1.124602596397	1.3028019075368
1.1247589218633	1.3029075362178
1.1248711264758	1.3029246582905
1.1249797292842	1.3029773008071
1.1251945336178	1.3030236880652
1.1253066968169	1.3031189052455
1.125456347815	1.3032830173305
1.1255661090627	1.3033588391895
1.1256645812134	1.3034058062277
1.125805083581	1.3034565656308
1.1259501497783	1.3036512465726
1.1260955262122	1.3036966485062
1.1262415979113	1.3039970571485
1.1263960816807	1.3043075921255
1.1265317564645	1.304334547019

1.1266764659555	1.3043511355442
1.1267925795373	1.3043627565943
1.126909150637	1.3044572724939
1.1270934736215	1.3045554338503
1.1272883527756	1.3051337307652
1.1274102658401	1.3052794024031
1.1275366732643	1.3055907870744
1.1276584442394	1.3056008019547
1.127813247844	1.3060076127456
1.1279584421185	1.3060545588096
1.1280760722228	1.3066228266495
1.1281830764875	1.3067828602313
1.1283098052455	1.3067991857344
1.1284697992097	1.3071389620143
1.1285953441008	1.3072329283081
1.1287096958465	1.3073576468434
1.1288644558145	1.3073717846787
1.128977276218	1.3075763303562
1.1291140589893	1.3078213229142
1.1292234811373	1.3080449330725
1.1294238970059	1.309078544185
1.1295641730134	1.3091789162013
1.1297231543929	1.3095964918481
1.1298394986739	1.309658400561
1.1299975000635	1.3097481771918
1.130145145544	1.310141592194
1.1303285501059	1.3101994622557
1.1304571982309	1.3102922052577
1.1306237593626	1.3104714036087
1.1307882428389	1.310748910516
1.1309124708122	1.3109700156219
1.1310293349079	1.3110154342848
1.1311699422971	1.3117018730934
1.13128405786	1.3117510059704
1.1314356096767	1.3118720767411
1.1315714905081	1.3118816002284
1.1317499763254	1.3120404319779
1.1319852942561	1.312041375601
1.1321250320317	1.3124270791422
1.132248688336	1.3125008958047
1.1323683327456	1.3128180115613
1.1325197006921	1.3134708431117
1.1326453803788	1.3137359912277
1.1328149261253	1.3142323728278
1.1329350956056	1.3142826734091
1.1330310463445	1.3142864186308
1.1331734095153	1.3143952916107
1.1333041250531	1.3145890582159
1.1334236815644	1.3148616131943
1.1335493872601	1.3150050994719
1.1336929647923	1.3150136786355
1.133832940132	1.3152883554876
1.1340180837059	1.3154302274295
1.1341629314342	1.3155475674151

1.1343365758637	1.3160798796334
1.1344778490415	1.3162163805008
1.1345725378962	1.316268717154
1.134711578825	1.3162924335175
1.1349085843856	1.3164028768805
1.1350044999751	1.3164368808681
1.1350828360102	1.3164423598594
1.1352339275335	1.3164455917682
1.1353856758177	1.3168092478187
1.1355028902979	1.3169766797105
1.1356461492162	1.3171952386125
1.1357399218218	1.317198441992
1.1359156822419	1.3174788981672
1.136077258028	1.3176108754446
1.136245423242	1.3180903405064
1.1363901483369	1.3183796942476
1.1365691747375	1.3184815039232
1.136728508461	1.3186343500634
1.1368735413842	1.3188949358201
1.1370454575053	1.3190131603802
1.1372238184999	1.3194235713414
1.1374026967983	1.31942516266
1.1375637342073	1.319833695756
1.1377357656831	1.3200589640392
1.1378761385091	1.3204791113142
1.138047045618	1.3205771628147
1.1381678327841	1.3207644681662
1.1383137371322	1.3209511447276
1.1384444530237	1.3210353839063
1.1385448095473	1.3211376850507
1.1387027039132	1.3212822457674
1.1388931460148	1.3213633017519
1.1390355621545	1.3215445068535
1.1391674975199	1.3215912477027
1.1393362015971	1.3220557077198
1.1394641543587	1.3220741036489
1.139612859075	1.3222909868466
1.1397526568289	1.3224179368759
1.1399193983996	1.3228697794674
1.1400837341968	1.3230148590847
1.1401929192584	1.3230249437915
1.1403523338608	1.3234868164418
1.1404576913166	1.3234905893471
1.1405926835163	1.323504914775
1.1407405097612	1.3236715422181
1.1408918091847	1.3237622694435
1.1410304354908	1.3241956568133
1.1411057535067	1.3242141525742
1.1412837708681	1.3243250210374
1.1414400993552	1.3243738749287
1.1415246650499	1.3243772918913
1.1417002639859	1.3244091523653
1.1418342565371	1.324422677994
1.141958579814	1.3246370298426

1.1420729724505	1.3247279001231
1.1422178992101	1.3249315100438
1.1423392540784	1.3251138314709
1.1424808846606	1.3251367742
1.1426261847171	1.3253766716691
1.1428531813449	1.3262067739013
1.1429815525048	1.3262152744798
1.1432016300309	1.3262656849802
1.1433610245282	1.3264796789472
1.1434678187282	1.3265752143238
1.1436143599627	1.3267354614836
1.1436979134006	1.3268161758653
1.1439429239359	1.3277497841559
1.1440888259657	1.3277665291898
1.144241397421	1.3280783938032
1.1443834494585	1.328099085832
1.1445063065492	1.3281447105855
1.1445889218812	1.328145537334
1.1447248231126	1.328361426299
1.1448396448214	1.3284250702613
1.1449435437306	1.3285514282703
1.1450923656152	1.328664828267
1.1452804067992	1.3290250175185
1.1454186524421	1.329159275469
1.1455764357775	1.3291991230203
1.1457124263084	1.3293160649621
1.1458373029615	1.3295064157668
1.1459543498378	1.3295767169907
1.1460824636936	1.329578179262
1.1462334138068	1.3297868037727
1.1463498475661	1.3297902781606
1.14651288915	1.329914573805
1.1467075050937	1.3300501634196
1.1468672942686	1.3302080707455
1.1470360371972	1.3304453531039
1.147203487893	1.3307369385051
1.1473364105612	1.3311430519537
1.1474743256576	1.3313980254329
1.1476331468618	1.3316954873643
1.1477393278824	1.3317760776396
1.1478383959857	1.331847337842
1.148009505564	1.3318776316518
1.1480981998086	1.3322491267519
1.1482566739092	1.3330435060274
1.1483945696211	1.3331366943815
1.1486300299406	1.3333644876856
1.1487635419205	1.3335739069933
1.1489007551938	1.3336370477122
1.149011244863	1.3340948449576
1.149092100875	1.3340953311435
1.1492639200542	1.3342615242629
1.149364015695	1.3343099839285
1.1494928756478	1.3345658693844
1.149640493536	1.3347600743856

1.1498432621887	1.335252363609
1.1499683697713	1.3352909616302
1.1500822127393	1.335331196141
1.1502150359914	1.3355707543296
1.1503514696627	1.3356748540902
1.1505789049621	1.3366728147317
1.150773841005	1.336839569378
1.1509270400935	1.336839879023
1.15106796012	1.3370528963843
1.1511946479732	1.3371552492522
1.1513053801704	1.3373235529427
1.1514260332241	1.3375351816304
1.1515884099274	1.3376759183356
1.1517033360468	1.3377195660666
1.1518390616608	1.3377274448673
1.1520202290414	1.3378248917785
1.152200555006	1.3378825817338
1.1523237078537	1.3379958542259
1.1525034528388	1.3380758599188
1.1526983927811	1.3382017279107
1.1528430963085	1.3385170558407
1.153003881257	1.3387166714284
1.1531231167517	1.3388171703195
1.1533569843308	1.339883684918
1.153467801718	1.3401045362997
1.1536072766502	1.3403006906438
1.1537770501465	1.3406020262925
1.1539083085461	1.3407496048462
1.1540581646059	1.3407957755676
1.1542332476566	1.3409324094115
1.1543465867899	1.3410801647289
1.1544434122279	1.3415050661764
1.1545966889906	1.3416337399041
1.1547383972715	1.3416491619474
1.1548902274822	1.3419789721116
1.1550153510999	1.3420601692901
1.1551797076929	1.3421188916932
1.1553527773882	1.3422531039458
1.155550132455	1.3423981477169
1.1557144060737	1.3424842706354
1.1558308662511	1.3426640030403
1.1559834928822	1.3427323652269
1.156151340707	1.3428498421726
1.1563127901085	1.3428842981343
1.1564273639791	1.3429416545168
1.1565349168038	1.3431945230504
1.1567308800145	1.3436244240975
1.1568482384632	1.3438602336649
1.1569669254305	1.3438721851351
1.1570621968466	1.3439121492314
1.1572393901776	1.3440568692032
1.1573828685031	1.3442362918276
1.1575501179058	1.34451593556
1.1577054354815	1.3445911808412

1.1578829756298	1.3451806947553
1.1580052634556	1.3455959201759
1.1581596135618	1.3456014346992
1.1582788589388	1.3456110285147
1.1584195070336	1.3458261700989
1.1585592021648	1.3460314193361
1.1587332348049	1.3463952126416
1.1588422379531	1.3464043016791
1.1589925655509	1.3464388461662
1.1591054926127	1.3467986302614
1.1592330050613	1.3468757045102
1.1593849811999	1.34700595916
1.1595247893612	1.3470591525028
1.1596583052738	1.3471312954023
1.1598056799355	1.3473055734213
1.159927824019	1.3473421753891
1.1600787215086	1.3476557226049
1.1602524475025	1.3477132594571
1.1603799265691	1.348080085355
1.1605286059497	1.3481169565088
1.1606733787865	1.3482582959063
1.1608493853076	1.3485157058036
1.1610400163343	1.3487800544439
1.1611992684793	1.3490779220794
1.1613908520433	1.3497064075637
1.1615912351897	1.3505610505439
1.1617243220056	1.350750942835
1.1619170919689	1.3517875994412
1.162095688498	1.3520280518662
1.1622981744004	1.3520516985224
1.1624419687622	1.3520807712656
1.1625658825736	1.3521865304274
1.162704929576	1.3522498242962
1.1628111416319	1.3526279505623
1.162921710546	1.3526859116802
1.1630908205345	1.3529020755144
1.1632681428797	1.352989489982
1.1634359594776	1.3533497309821
1.1635521080624	1.3535009287866
1.1637082120765	1.353504465161
1.1638984297473	1.3536001954038
1.1640622743408	1.3536215134403
1.1642107468962	1.3536269206097
1.1643753808519	1.3537565156864
1.1645078867679	1.3539757463388
1.1647207428018	1.3540775678487
1.1648414914518	1.3542334828958
1.1650206343775	1.3543543984556
1.1651869601249	1.3549132041931
1.1653534741009	1.3551015553853
1.165517236953	1.3555032839582
1.1656822422384	1.3557706371233
1.1658972923598	1.3561630688979
1.1660299967648	1.3563201949675

1.1661327135115	1.3563238031038
1.1662870653236	1.356715797405
1.1665076674172	1.357746649379
1.166659464311	1.3580535383723
1.1667954883466	1.3581311279141
1.1669024073687	1.3582715686102
1.16705314587	1.3583653754378
1.1672377192104	1.3586399233026
1.1673633401158	1.3588212305544
1.1675573556786	1.3593024983219
1.1677013108737	1.3594513573329
1.1678266489777	1.3594843115081
1.1680058176222	1.359673205745
1.1681382013085	1.3598587338871
1.1683020736998	1.3602087593962
1.1684451451149	1.3602587977964
1.1686224830797	1.3603149639458
1.168765870571	1.3605463391298
1.1689015612923	1.3606345538255
1.1690129779549	1.3606570725181
1.1691565503122	1.3608706990314
1.1692450626961	1.3610848213608
1.1693991090905	1.3611875182233
1.1695557037999	1.3612086042607
1.1697517197065	1.3615736277656
1.1698936282243	1.3615785141806
1.1700350444768	1.3621554771523
1.170220931722	1.3621780836534
1.1704121861273	1.3622325178162
1.1705927307871	1.3626693678876
1.1707286163669	1.3627748082196
1.1709287954263	1.3637082211445
1.1711182700093	1.3641070046473
1.1712599108092	1.3641593058466
1.1714725203403	1.3644840772545
1.1715950197244	1.3644981433389
1.1718027438682	1.364546591323
1.1719625637623	1.3647987616748
1.1720922059333	1.3648682896567
1.1722265708649	1.3650359510991
1.1723562790753	1.36540052922
1.1725694073034	1.3659405204262
1.1726859290074	1.3659539185585
1.1728392953656	1.3659856453188
1.1729882497235	1.3662474748553
1.1732689247262	1.3670771633342
1.1733999290588	1.3670971636246
1.1735642747463	1.367464559631
1.1737837690078	1.368613486341
1.1739465609922	1.3687788331087
1.1741085067238	1.3690984500668
1.1742967746499	1.3700190376956
1.1744380376444	1.3702896937429
1.174586861236	1.3705786516138

1.174723000114	1.3712897067151
1.174892803454	1.3712928959542
1.1750931781089	1.3715464852572
1.1752419476142	1.3716768423452
1.1754197458478	1.3717508629258
1.1755944415652	1.3718231897895
1.1757384909137	1.3719059398865
1.1758961808952	1.3720245982539
1.1761161781822	1.3722177947195
1.1762380151119	1.3722437986357
1.1764260011535	1.3723576627435
1.1765231061336	1.3723811661292
1.176683722659	1.3725475843288
1.1768623759683	1.372648259564
1.1769782537125	1.3728388028852
1.1771211963112	1.3728762767998
1.1772885713912	1.3729514013644
1.1774991597221	1.3734142192613
1.1776167355146	1.3735110987733
1.1777438426353	1.3735655532231
1.1778625535455	1.3737681872908
1.1780430997652	1.373933113386
1.1781731235411	1.3744647112166
1.1783304151211	1.3745071962124
1.178529609501	1.3751299221791
1.1787326337368	1.375717336841
1.1788896385151	1.3758563858627
1.1790370480526	1.3759042862727
1.179252048122	1.3764332563635
1.1793992565288	1.3764734718311
1.1794863385742	1.376685964773
1.179618159182	1.3767413970591
1.1798168650226	1.3768944190387
1.1799606569017	1.3769714537404
1.1801121446324	1.3771250963209
1.1802658181095	1.3772080486911
1.1803992854205	1.3775920942961
1.1805829129687	1.3776956672769
1.1808129235644	1.3778228880041
1.1809773754148	1.3779995283527
1.1811352517891	1.3780131112035
1.1812916523752	1.3780215355069
1.181443291689	1.3784059651159
1.1815868645524	1.3784930983038
1.181778923573	1.3785741787919
1.1819385539833	1.3786321102893
1.1821481821365	1.3787832340753
1.1822592453287	1.3788324623826
1.1824058907399	1.3788665222606
1.182538612983	1.3789904380536
1.1826936547921	1.3790306722901
1.1828840501838	1.379404679368
1.183057853131	1.3796328855192
1.1832046325132	1.3797853525872

1.1833724228087	1.3800502575211
1.1835168235233	1.3800503514567
1.1836697263706	1.380154161772
1.1838344896496	1.3802464093534
1.1839746226932	1.3804303396108
1.1842080449641	1.3813324470786
1.1843906760239	1.3813712844319
1.1845283997134	1.3816523367638
1.1846693612431	1.3816634027057
1.1848435829092	1.3819545035439
1.1849601621604	1.3820945861489
1.1851251137691	1.3820957322102
1.185273208912	1.3821295158915
1.1853911441879	1.3821636379652
1.1855947721837	1.382286881611
1.1857805737039	1.3825817820192
1.1859640334225	1.3829710591339
1.186141330081	1.3831390482474
1.1862825745592	1.383317141274
1.1864443937144	1.3837320676244
1.1866410342635	1.3838444027714
1.1867762505905	1.3838503151227
1.1869204140164	1.3840323801287
1.1870334493033	1.3841594226352
1.1872336372318	1.3841922386876
1.1874236474986	1.3843474957486
1.1875546238686	1.3843494462312
1.1877670752273	1.3843701871401
1.1879556137219	1.3844385789583
1.1880963578203	1.384601446446
1.1882411996442	1.3848873611145
1.1884402692232	1.3849395344944
1.1886205740675	1.3851788537691
1.1887954409425	1.3859063425761
1.188994678401	1.3860679442429
1.1891128964231	1.3860720160189
1.1892627698763	1.3861258049549
1.1893547739923	1.386326356142
1.1895242212227	1.3863532874481
1.189695928615	1.3865815430734
1.1898074691092	1.3865916517488
1.1899578365345	1.3867254528456
1.1900938806032	1.386741166274
1.1902313779244	1.3868074337295
1.1903554050999	1.3869447654125
1.1905019891188	1.3872570787352
1.1906240649573	1.3873817550431
1.1907981067057	1.3879389290697
1.1909794363761	1.3882257651152
1.1910527211494	1.3883269702567
1.1912544682703	1.3888415993808
1.1914192990686	1.3888568780321
1.1915608624758	1.3888743056012
1.1917578733867	1.3889900536855

1.1918965460617	1.3892616126856
1.192109286104	1.3896588335047
1.1923028011212	1.3899767796571
1.192429868567	1.390061010387
1.1925820570332	1.3902129909156
1.1927478294469	1.3902492525688
1.1928915237879	1.39034587586
1.1930759576692	1.3904709624749
1.1932069364241	1.3907562444913
1.1933809975172	1.3909257174624
1.1935181475375	1.3910638731226
1.1937251115242	1.3914766055599
1.1938200963831	1.3916573318383
1.1939726198567	1.3917600290007
1.1941343523494	1.39185119718
1.1943066029694	1.391928250888
1.1944027529545	1.3921287979224
1.1946621315648	1.3924482632506
1.1949023842291	1.3925541744687
1.1950633970278	1.3927080007226
1.1952651756584	1.3928106975122
1.1954146843124	1.3929370775294
1.1955523560285	1.3931471147203
1.1957540725395	1.3932595843583
1.1959012159226	1.3934391597168
1.1959846129317	1.3936617386805
1.1961127464914	1.3937688077163
1.1963089818686	1.3939375768323
1.1964483839239	1.3939940531666
1.1965633542066	1.3940323102578
1.1967530199143	1.3940325973106
1.1968858381315	1.3941287170899
1.1970891118915	1.3947607777116
1.1973184107602	1.3952984041306
1.1975289156517	1.3953129953456
1.1976634804336	1.3954892098927
1.197801693982	1.3956556109026
1.1979755444689	1.3958389113057
1.1981435859796	1.3959107611004
1.198313526511	1.3966338330619
1.198467795783	1.3967552292011
1.1986394969486	1.3967586262268
1.198830207287	1.3970794184095
1.1989422720336	1.3972463504007
1.1990823181633	1.3972926408007
1.1992424966149	1.3974144408349
1.1993540325989	1.3976957884217
1.1994883540147	1.397872557103
1.1996242889442	1.397907494989
1.1997948771114	1.3980941713139
1.199936846861	1.3981100585944
1.2000419749063	1.3981651971673
1.200185784299	1.398269122008
1.2003412045678	1.3983097606772

1.2004838763348	1.3983332649879
1.2006011585632	1.3989388852355
1.2007760429704	1.3989633605145
1.2009122320374	1.3990570244407
1.2010986454716	1.3994175025227
1.2012782814289	1.3995697912792
1.2014691137156	1.3997215729567
1.2016461175808	1.3997290089495
1.2017832012764	1.3997379207745
1.2019591179479	1.3998430197095
1.2020847548331	1.3999542226273
1.202248501962	1.3999780421552
1.2024794148766	1.4001685135294
1.2027301667569	1.4008976151658
1.2028747456542	1.401029072012
1.2030406085316	1.4015136363197
1.2031763664264	1.4015831424293
1.2032826604978	1.4016132342185
1.2034298102291	1.4016263395111
1.203548884972	1.4016720157819
1.2036897120391	1.4017333863768
1.203876289972	1.4020271350967
1.203978099206	1.4021935736709
1.204132021619	1.4022440235133
1.2042978549891	1.4023474120585
1.2044453456352	1.4024232622254
1.2045819936598	1.4025821158809
1.2047230290986	1.4027473371542
1.20486253569	1.4027473829216
1.2050106412014	1.4035106102595
1.2051166176684	1.4035461577977
1.2052446087808	1.4035818498383
1.2054457500954	1.4038887824729
1.2056747888232	1.4043383289171
1.205844226595	1.4046762831973
1.2060389527784	1.4047991247573
1.2062100965596	1.4049639439972
1.2064014937176	1.4059828219569
1.206515066435	1.4061096204267
1.206653190695	1.4061194187846
1.2067496041166	1.4061341698819
1.2069335918392	1.4063915332622
1.2071142008398	1.4071157378443
1.207282960123	1.4074267921151
1.2073929843651	1.4074291685092
1.2075290963084	1.4075459905647
1.2077923816169	1.4093192370578
1.207943896028	1.4093946467603
1.2081360575389	1.4099114241064
1.208340551338	1.4103578570221
1.2084666948165	1.4103967987827
1.2086315834805	1.4109797647452
1.2088176341958	1.4111561895319
1.2089963854119	1.4112753418958

1.2091967060142	1.4113702781055
1.2093637236423	1.4119167907461
1.2095333689869	1.4120046195192
1.2096952823932	1.412211287908
1.2098689669903	1.4122856867202
1.2100598529058	1.4123166125189
1.2102256747641	1.4123532809447
1.210374956953	1.4123655326923
1.2105508948222	1.4123863538088
1.2107174802755	1.4125449584003
1.2108474715716	1.412580381089
1.2110501688453	1.4130325503063
1.2111887098935	1.4130637228118
1.2113670731394	1.4131806902878
1.2115694441006	1.4135073994353
1.2117626894021	1.4138517784073
1.2119122713838	1.4139836370228
1.2120708934521	1.4139951071313
1.2122152184971	1.4141522319714
1.2123460638582	1.4145473519094
1.2124885392129	1.4146687163385
1.2127369392364	1.4148540770828
1.2128941152887	1.4149757972373
1.2131007528576	1.4149883004962
1.2132893217239	1.4151701965884
1.2134033378381	1.4151709942951
1.2135677289463	1.4153986761638
1.2137443443916	1.4154533082785
1.2139080992483	1.4155097656354
1.2140455655011	1.4158969374371
1.2142286980462	1.4159021733124
1.2143951644293	1.4160296379638
1.2145980162105	1.4160799537793
1.2147754081206	1.4164038309794
1.2149250914052	1.4165942153832
1.2151477139617	1.4169584475946
1.215296547574	1.4169783926289
1.2154965122475	1.4174629659183
1.215664207721	1.4177643434865
1.2158116380966	1.4178208781238
1.2160628947708	1.4182461075623
1.2162666677052	1.4185939258274
1.2164262902705	1.4187306267595
1.2165912504475	1.4188115975491
1.2167699584821	1.4190799381611
1.2169329625234	1.4193436227987
1.2171188284242	1.4196653825743
1.2173110755316	1.419737110248
1.2175082687762	1.4200847329965
1.2176241274481	1.4203024065477
1.2177354986277	1.4203491509726
1.2179139415642	1.4212728916051
1.2180809356193	1.4217200037415
1.2182346647621	1.4217800040532

1.2183854525124	1.4218394304715
1.2185831513883	1.4223957198549
1.2186821417135	1.4224613271598
1.2188203899592	1.4228558498974
1.2190357914638	1.4237771598861
1.2192010340248	1.4238905166773
1.2193395181317	1.4239776133715
1.2194461273952	1.4240054247581
1.2196186574723	1.4242997874359
1.219785228262	1.4246342929655
1.2199711467813	1.4251565465964
1.2201170055149	1.425539448154
1.2203063117882	1.4258318927293
1.2204804990769	1.4258701257856
1.2207366930381	1.4267583740691
1.2208844018814	1.4268598092882
1.2210386794645	1.4270756484097
1.2212449578985	1.4272087013448
1.2214826296981	1.4272298814287
1.2216110397672	1.4273038340357
1.221764381699	1.4273298975103
1.2219156128596	1.4275242184703
1.2220772372297	1.4275938320471
1.2222464652568	1.4276812289036
1.2223947568049	1.4282817034478
1.2225746261619	1.4288317225578
1.2227657712811	1.4293030339166
1.2229554303601	1.4294832839786
1.2231447954398	1.4295698359252
1.2232908816015	1.4297863024448
1.2233851962423	1.4299326235919
1.2236107470902	1.4299443801415
1.2237963609003	1.4305051165816
1.2239584844711	1.4305996341948
1.2240932029739	1.4307552555317
1.2242889205844	1.4309826678199
1.2244883387553	1.4315109295734
1.2246167563649	1.4317791165706
1.2247590249904	1.432000216432
1.2249231460442	1.4326349958314
1.2251269356837	1.4327785436641
1.2252835454565	1.4328097350407
1.2254527263635	1.4329850669204
1.2256041063287	1.4335753257439
1.2257328646604	1.4336188092561
1.2258896445726	1.433646051764
1.2260627607499	1.4338665699634
1.2262554422162	1.4340721397122
1.2263987961674	1.4341966437957
1.2266106540357	1.4345665479498
1.22669870975	1.4345759625785
1.2268483475969	1.4349691451973
1.2269995131885	1.4349972320596
1.2270965320231	1.4351076638001

1.2272498428167	1.4351835366731
1.2274258257732	1.4353829504516
1.227598261961	1.4357152757205
1.227703104471	1.435774840174
1.2278267217087	1.4358062027546
1.2280236880954	1.4358675032751
1.2281882601037	1.4360752359888
1.2283327685305	1.436126393749
1.2285204591569	1.436237339555
1.2286917629028	1.4365088991016
1.2288457536335	1.4368081026403
1.2289673379359	1.4369349465438
1.2290845451271	1.4369722397622
1.2292231762711	1.4370818366819
1.2294149667705	1.4371305651552
1.229585505498	1.4372673157241
1.2296920871268	1.4374505846267
1.2298525216754	1.4376250769096
1.2300419802724	1.4380512130507
1.2302026092427	1.4381354859607
1.230334393905	1.4387164651819
1.2305168772321	1.4390548299215
1.2307202410829	1.4391200137844
1.2308592626397	1.4394748937952
1.2310856207185	1.4395910545791
1.2312500351818	1.439669926604
1.2314200852108	1.4399513746767
1.2316058798945	1.4400466405261
1.2317471708031	1.4403339356044
1.2318981427498	1.4403824966919
1.2320662318125	1.4407643574255
1.2323518701316	1.4408924878106
1.2324846174687	1.4409093707136
1.2326934011418	1.4411237377397
1.2328715852351	1.441325768717
1.2330474145604	1.441358424687
1.2332357335023	1.4414286631282
1.2334131161649	1.4416792907959
1.2335346194241	1.4418518896077
1.2337072307374	1.4419333858969
1.2338158778678	1.4419867121621
1.2340256601068	1.442000429354
1.2341623147429	1.4420507959201
1.2343316587079	1.4420578210707
1.2344913588826	1.4422623176021
1.2346755404151	1.4425101710734
1.234859111363	1.4427112257252
1.2350209332725	1.4432560564981
1.2351984312921	1.4435741159046
1.2353666107836	1.4437541997229
1.2355420587003	1.4438153607338
1.2357103014464	1.4439603831611
1.235875365353	1.4440571530141
1.2360081052886	1.4441557321627

1.2361961549978	1.444195098542
1.2363614043843	1.4449983926543
1.2365098032286	1.4452100350453
1.2366891432292	1.445415963591
1.2368482230529	1.4456696094963
1.2370862146582	1.4457494203924
1.2372333327484	1.4457940608939
1.2374030575716	1.4460625158147
1.2376004041086	1.4460822510294
1.2377792369922	1.4461615019938
1.2379187544039	1.4463125115029
1.2380453872035	1.4464415524488
1.2382143656261	1.4465384003034
1.2383743639243	1.4466194996132
1.2386034990908	1.4467144769899
1.2387772554265	1.4467907108722
1.2388963240658	1.4471204186715
1.2391487025973	1.4472930362443
1.2393914311495	1.4475580094861
1.2395553374893	1.447615916496
1.239733278798	1.4477482493581
1.2399305093288	1.4483004689515
1.2400707627393	1.4484028355819
1.2402620844066	1.4486594388675
1.2405203579721	1.4487993025659
1.2406852307285	1.4489100777363
1.2408829602501	1.4493056781968
1.2409859689564	1.4493665305869
1.2412374018323	1.4493938156006
1.2414416133286	1.4494483101201
1.2416264132208	1.4499904568827
1.241786846787	1.4506423113971
1.2419209672525	1.4507055941529
1.2420888314167	1.4507671365722
1.2423279594357	1.4508216599581
1.2424990811502	1.451689038616
1.2427018061914	1.4519931874923
1.2428823099636	1.4520453976445
1.2430517713221	1.4523075508412
1.2431740598379	1.4524971026366
1.2433180515309	1.4526084441394
1.2435085696642	1.4526142934076
1.2436708898316	1.4527948531609
1.2438486949895	1.4530301630581
1.2440210364936	1.4530892183804
1.2441457820568	1.4533258867902
1.2443531492108	1.4534995176128
1.2445181355901	1.4535673607063
1.2447163087203	1.4538807437099
1.2448434250396	1.4540810887872
1.2450684566482	1.4542853682786
1.2452584441692	1.4544446254621
1.2454407648797	1.4545413336369
1.2456322434323	1.4545914800565

1.2458021431633	1.4552338353982
1.245956167324	1.4553533937694
1.2461703097079	1.4563595583025
1.2463305677217	1.4563998348393
1.2465874172326	1.4568266059898
1.2468060343973	1.4572311049909
1.2470914398203	1.4574959553722
1.2473082038233	1.4583057110541
1.2474622175957	1.4583248654156
1.2476121443758	1.4588582017335
1.2478178851073	1.4589879694345
1.2481105922994	1.4613009577133
1.2482932230984	1.4617589913734
1.248476787361	1.4617941502211
1.2486589707292	1.4618002721567
1.2489255331474	1.4621842059505
1.249120179911	1.4628831947272
1.2492716954864	1.4630422337211
1.249439267123	1.4634349207741
1.2495939906449	1.4636061996084
1.2498097062892	1.4640548013252
1.250048639278	1.4645486620899
1.250262847073	1.464851261622
1.2504177089867	1.4650269127317
1.2505940343608	1.4654407496558
1.2507785092055	1.4656058614964
1.250955852674	1.4656567599552
1.2511347167131	1.4657928037242
1.2513773676312	1.4658424055358
1.2515883352962	1.4668904943352
1.2517966915631	1.4669663193996
1.2519604126483	1.4674336713739
1.2521301074828	1.4676923344652
1.2523444591567	1.4677464592017
1.2524760688924	1.4678504989841
1.2526937869898	1.4681685957309
1.2528965007197	1.4681788517607
1.2530962825523	1.4689653138736
1.2532888490753	1.46904283246
1.2534178075745	1.4691407056932
1.253585746817	1.4696260688586
1.2537357770364	1.4699256328814
1.2538463527841	1.4703587708494
1.2540495241532	1.4703769935347
1.2541790083258	1.4704862507905
1.2543562603794	1.4707846236359
1.2545461560454	1.4709018923568
1.2546788144305	1.4709792394416
1.2548613289513	1.4712464352056
1.2550092013503	1.4717251827367
1.2551888216175	1.4721701009336
1.2553537679145	1.4722751806699
1.2555305989508	1.4722860988837
1.2556863669877	1.4725290526121

1.2558579212049	1.4726158608453
1.2560490188716	1.4732518771261
1.2562226478438	1.4736651920916
1.2563994627841	1.4737660297558
1.25654437694	1.4739017839524
1.256689261802	1.4742789693983
1.256956986865	1.4744577615751
1.2571518756867	1.4747691463765
1.2573368104909	1.4748502623758
1.257518211449	1.474935307584
1.2577104169429	1.4751117470493
1.2579079656832	1.4752303169575
1.2580534183985	1.4753584131272
1.2581936549444	1.4753666629159
1.2583314855179	1.4753866576984
1.2584836931881	1.4754703004641
1.25861031196	1.4755226229499
1.2587919337924	1.4758883568632
1.2589496423423	1.4761433547919
1.2591934602983	1.4766758949202
1.2593327478987	1.4767078874862
1.2595459700691	1.4772402997101
1.2597178542393	1.4774373466838
1.2599790997525	1.4775168135063
1.2601042189006	1.4776018355734
1.2602488011488	1.4777511730263
1.2603477951577	1.4778400770587
1.2605506532986	1.4778458932206
1.2607326306936	1.4782656373425
1.2609265174083	1.4785506392012
1.2610718609324	1.4787864439529
1.2612427710158	1.4790519618565
1.2614112349504	1.479297695414
1.2616638220137	1.479760423497
1.2617961371278	1.4798820593859
1.261951266529	1.4798856659997
1.2621667902922	1.4799778550366
1.2623878792574	1.4800044964125
1.2625766296251	1.4803922401402
1.2627524963331	1.4804006278646
1.2628743397816	1.4806177844478
1.2630510288605	1.4807039444052
1.2632649408667	1.4809282400512
1.2635166642253	1.4810966104819
1.2636776435175	1.4813473066038
1.263925509923	1.4814554602258
1.2640959129296	1.4815117289493
1.264273712877	1.4819936905793
1.2644553199647	1.4821619417209
1.2646045240669	1.4822761235059
1.2647702018148	1.4825141217885
1.2649090667866	1.482874329116
1.2650671869541	1.4828913834469
1.2652333370563	1.4831550551221

1.265423589136	1.4833852021559
1.265585595261	1.4834167043295
1.2657670683698	1.4837321785269
1.2659026781283	1.4839212346636
1.2661134552865	1.4847967222883
1.2662556636771	1.485121355176
1.2664018440237	1.4853327997627
1.2665880752504	1.4855171582872
1.2667730861775	1.4856443711203
1.2669131223071	1.485906628191
1.2670895989175	1.4859391377428
1.2672269997219	1.4863048923427
1.2674616704196	1.4873265679296
1.2676196195794	1.4877811854289
1.2678544054962	1.4879105481043
1.2680835671908	1.4885576330927
1.2682799778965	1.4886968888079
1.2684981851871	1.4887468051012
1.2686916444189	1.4888767913551
1.2688819062056	1.4890692616229
1.2691452845507	1.4902285720475
1.2693120687859	1.4905551428953
1.2695154585547	1.4907975074827
1.2697491548556	1.4911539825591
1.269868348347	1.491180812408
1.2700437519474	1.4914177666342
1.2701766638544	1.491706119077
1.2703761886238	1.4920543640904
1.2705903103725	1.4920991801077
1.270780433535	1.4923005609083
1.2709306458894	1.4924510136049
1.2710956306853	1.4926399255083
1.2712643046396	1.4930067350548
1.2714453615149	1.4932887889715
1.2716604353155	1.493386817376
1.2718148047161	1.4937841329383
1.2720610293232	1.4944199655294
1.2722446949494	1.4945817432305
1.2723952901043	1.4950907758464
1.2726914371199	1.4953618023202
1.2728984375073	1.4955104618337
1.2731081817392	1.4959338248019
1.2732848323702	1.4963196677606
1.2734599435404	1.4967042207825
1.273651205321	1.4975772472266
1.2737651159364	1.4976516182255
1.2739477427672	1.4977417495347
1.2741221266909	1.4979868829838
1.2744073538537	1.4989494306637
1.2745144084196	1.4990277872355
1.2747569166812	1.499341156206
1.274919666176	1.4993715148513
1.275145980648	1.4996653953331
1.2753358438642	1.4997160876326

1.2754790640289	1.4997343127229
1.2756980185482	1.5005693227862
1.2758494347085	1.5006253833979
1.2760175756559	1.5006289615616
1.2761563741504	1.5007876869072
1.2762957258745	1.5010839232258
1.2765019591714	1.5012667164037
1.276654499942	1.5015048326123
1.2768476350941	1.5016724337446
1.2771085454268	1.5021242153394
1.2773042214857	1.5022546343919
1.2774896265287	1.5023423065388
1.2776285242439	1.5023728252652
1.2778522492389	1.5030670166264
1.2780534039982	1.5035278816951
1.2782361550393	1.5036510184204
1.2784521802587	1.5039207553984
1.2786019789439	1.5041073090922
1.278858726445	1.5046212023456
1.2790895363922	1.5052200908371
1.2792560385822	1.5052575999281
1.2794273218437	1.5052618015974
1.2795635038168	1.5054246491736
1.2797365349406	1.5054581509445
1.2799077011131	1.5055629809483
1.2801188427306	1.5060962677904
1.280286598837	1.5061298287859
1.2804496729635	1.5061823612571
1.2805920751773	1.5062774881682
1.2807888632034	1.5070713898779
1.2808874663839	1.507249756382
1.2811897826789	1.5075535166455
1.2813662562961	1.507558409563
1.2816299241045	1.5077624376572
1.2818453897745	1.5078399487014
1.2820012964069	1.5082145500953
1.2821707787748	1.5086955262869
1.2823733209502	1.5093537103964
1.2825696976628	1.5094034011831
1.2828446313113	1.5098108054083
1.2830091594242	1.5099079814008
1.2832036720331	1.510246920767
1.283398944414	1.5109514901384
1.2835813858149	1.5111959923665
1.2837647562893	1.5112177227878
1.2839462694441	1.5114303771335
1.2841073575867	1.5119683036487
1.2842796941929	1.512160687821
1.2844637750762	1.5123990434528
1.2846895016218	1.5131718044563
1.284868534292	1.5133405796045
1.2850239956972	1.5136472158237
1.2852627973706	1.5136524838098
1.2854194605637	1.5136616682687

1.2856326932048	1.5143495470317
1.2858140787897	1.5145366261797
1.2860036259371	1.5148124842116
1.2861962328451	1.514823135049
1.2863850769681	1.5149986202853
1.2865861565328	1.5153156521455
1.2867615017989	1.5154541312127
1.2869991995749	1.5156331206354
1.2872041330671	1.5163252596213
1.2874074473356	1.5167211071593
1.287566134789	1.5168641727367
1.2877542847761	1.5172345144437
1.2879879365718	1.5173802302881
1.2881966444017	1.5174524047337
1.2884150784805	1.5174913585342
1.2886058300001	1.5175738061227
1.2887847130999	1.5178939196998
1.2890114266208	1.5186707267999
1.2891337573882	1.5189516015453
1.2893238604928	1.5191331541945
1.2895059470021	1.519206205537
1.2896979391537	1.5192129464118
1.2898764197726	1.5192783013053
1.2899992775625	1.5193196335176
1.2902761025161	1.5201872364768
1.2905303754138	1.5207607552977
1.2906588170647	1.5212313835457
1.2908450850935	1.5213019558415
1.291075923201	1.5225044909758
1.2912598781217	1.5225925424846
1.2914862587736	1.5238392355906
1.2917114431344	1.5240109522491
1.2918673769864	1.524083736399
1.2921069875151	1.5244895927204
1.2923560748014	1.5247012799576
1.2925073046997	1.5247064790376
1.2926383090829	1.5247133729416
1.2929405139336	1.5253510233541
1.2930889646333	1.5253971411672
1.2932716932134	1.5255148549372
1.2934648896155	1.5255340377236
1.2935902616128	1.5257375926658
1.2936798728002	1.525932988606
1.2938335941486	1.5260185218347
1.29402135152	1.5260686631835
1.2942155713401	1.5263566732395
1.2943847292827	1.5264197416141
1.2946100376323	1.5273760630989
1.2948520335893	1.5274899974819
1.2949781436149	1.52779011284
1.2951225142945	1.5278367508651
1.2953176949649	1.527954026589
1.2954941964473	1.5281150684476
1.2956606544258	1.5282887633448

1.2958575136806	1.5284186938964
1.2960545395466	1.5285647962848
1.2962380035664	1.5289730555655
1.2964126790889	1.5290795501763
1.2966344782598	1.5296863374694
1.2968884703988	1.5300412112403
1.2970582973916	1.5302916920541
1.2972438915278	1.5304497486747
1.2974322073467	1.5305240683998
1.2976476641384	1.5305907917818
1.297800290622	1.5309579846372
1.2979629875359	1.5312239360161
1.2981685610167	1.5312500338123
1.2983312245297	1.5312718155926
1.2985512096565	1.5313463870075
1.2987408833425	1.5317621109803
1.2989086278159	1.531867375824
1.2991279055092	1.5319674190168
1.2993508446534	1.5328199210449
1.2995132383235	1.5328554612057
1.2997385323417	1.5332165401403
1.2998818460064	1.5333431279369
1.3000645757206	1.5334997093336
1.3002254394085	1.5335070649488
1.3003983007204	1.5340487782352
1.3006191163166	1.5341481607053
1.3008247199445	1.5343086000378
1.3009725775007	1.5346795740014
1.3012268247825	1.5346899062832
1.301436316386	1.5350752532706
1.301641241631	1.5355099480809
1.3018624112013	1.5358692590281
1.30204342039	1.535978977592
1.3023027062093	1.5359825084101
1.3025105706496	1.5361475985789
1.3026614416619	1.5364754179667
1.3027780075884	1.5364774402552
1.3030387712814	1.5366946255898
1.3032190062263	1.5368371922787
1.3033796282272	1.5369214134142
1.3035605278656	1.5370432994008
1.303750308456	1.5370738187106
1.303945870302	1.537116737249
1.304117571355	1.5375617932656
1.3042438453979	1.5376370709357
1.3044371424032	1.537748890678
1.3046171657055	1.5377985601482
1.3048178269141	1.5378017249963
1.3049876957259	1.5384596552679
1.3051566178631	1.5388374893712
1.3053888332819	1.5397215855904
1.3055643227947	1.5399869546438
1.3057810851412	1.5406201096019
1.3059859706846	1.5407415118411

1.3062102678264	1.5408145476955
1.3064620601657	1.54160458677
1.3066825780862	1.5420360676397
1.3068495437263	1.5426697008835
1.30705855895	1.5433621838271
1.3072055171514	1.5434633631831
1.3074343683497	1.543506143739
1.3076195692018	1.5436283206651
1.3078558707197	1.5436517871308
1.3080906175975	1.543909714453
1.3083424212932	1.5445772603466
1.3085453968208	1.5449357833744
1.3087808855438	1.5455144069118
1.3089963767341	1.5457109337966
1.3092110319505	1.5461097291857
1.3094258397558	1.5462325604262
1.3095999197297	1.5463869490873
1.3098416530702	1.5470118863325
1.3100178994938	1.5471553977602
1.3101594874443	1.5473336139864
1.3103436592469	1.5479019875553
1.3106089370478	1.548491130448
1.3107897766221	1.5484927414237
1.3109770263085	1.5486019369802
1.3112551501822	1.5492486940888
1.3114181652179	1.5493045962189
1.3115680070795	1.5494876066494
1.3118392493433	1.549720732088
1.3120543221125	1.5501055810259
1.3122317577451	1.5503249667825
1.3124372388629	1.550878724491
1.3126641687169	1.550913674359
1.312789996146	1.551006561656
1.3129656759079	1.5511853211184
1.3130870103366	1.5513030335878
1.3132686684626	1.5513171758974
1.3135552726539	1.5525495629195
1.3137185080429	1.5526370263759
1.3139219049402	1.552949708345
1.3140856601786	1.5530324257337
1.3143280717466	1.5530360757408
1.3145014486003	1.5530667336219
1.3147430969159	1.5535743701821
1.3149017483021	1.5539903323033
1.3150696912043	1.5541465655011
1.3152125332633	1.5543115999233
1.3153525869254	1.5543286733015
1.3155154346899	1.5545027020062
1.315746103884	1.5546839764774
1.315985909293	1.5552566978932
1.3161843455922	1.5555079240308
1.3163509289438	1.5558880897244
1.3165693398128	1.5559028953664
1.3167663213433	1.5562429120201

1.3169618611762	1.5565264484068
1.3171607072475	1.5567627158919
1.3174046900852	1.5572750742655
1.3176354406422	1.557363878133
1.3177940670472	1.5573761732013
1.3179815602542	1.5574580370974
1.318170530576	1.5577613121287
1.3183154873531	1.5579405085178
1.3185352463957	1.558101180973
1.3187434091005	1.558191788254
1.3189297805412	1.5582502723571
1.3191568384562	1.5584471987593
1.3193288083223	1.5584684629726
1.3195828013992	1.5587233133682
1.3198287217356	1.5593069656035
1.3199868213173	1.5596275625795
1.3201436321354	1.5601121207539
1.3203356084749	1.5607775812123
1.3204978092765	1.5608860699881
1.3207921907333	1.5616338929427
1.3210525381965	1.5616960180248
1.3212924238822	1.5621695253812
1.3215185855019	1.5626586298232
1.3216759502248	1.5627572390521
1.3218487571025	1.5630276146046
1.3220928680502	1.563279740022
1.3222895806386	1.5633801370098
1.32246811862	1.5635438040837
1.3226948106634	1.5635672511583
1.3229205386143	1.5639405507958
1.3231025853124	1.5641667677677
1.3232994956004	1.5648109505064
1.3235076661578	1.565139560063
1.3236696252346	1.5652573793186
1.3239541834182	1.5656373871766
1.3241359645037	1.5657497997018
1.3243606111072	1.5663224369711
1.3245914325512	1.5665887191222
1.3247784409851	1.5665991043679
1.3249595155377	1.5666820701614
1.3251337004824	1.566966071779
1.3253603473353	1.5669790181861
1.3255592308925	1.5670476544081
1.325760466726	1.5670800618503
1.3259620336956	1.5671152190417
1.3261414704289	1.5672818457807
1.3263441905606	1.5673165180869
1.3265692021669	1.567561122962
1.3268314544553	1.5677483256212
1.3270693765305	1.5683926508421
1.3272381856574	1.568584485624
1.3274554263461	1.5687316786518
1.3276437165659	1.5689027786032
1.3278953819928	1.5690667186317

1.3281389845725	1.5691360479272
1.3283142301299	1.5695457416904
1.3284974696848	1.569769503661
1.3286788837492	1.5701159235344
1.3289198724721	1.570198264945
1.3291239662533	1.5703634767967
1.3292691039275	1.570820286348
1.3294151629498	1.5709481880182
1.3295588090995	1.5710037583551
1.3296842906321	1.5712779456727
1.3299915140815	1.5718346139251
1.3302348692067	1.5721492925861
1.3304639156287	1.5722045030434
1.3306533292834	1.572258524708
1.3308177377851	1.5722689943544
1.3309977535834	1.5722863732814
1.3312724741081	1.5725677611033
1.3314986587785	1.5730370657301
1.3316406786159	1.573112218627
1.331788897301	1.5731211137987
1.3319631739532	1.5731566662882
1.3321534398622	1.5731946811616
1.3322994844487	1.5733888980152
1.3325369942718	1.5742704373942
1.3327599900683	1.5746946183196
1.3330060944555	1.574805727811
1.3331800711396	1.5750303525632
1.3333761071295	1.575213509926
1.3336606018306	1.5759038935133
1.3338098051424	1.5760060691406
1.3340413905941	1.5760307218842
1.3342542752154	1.5765419539787
1.3344653350563	1.576883103913
1.3346357876015	1.5769542155786
1.3348046596089	1.5770865256728
1.3349987665798	1.5776206918497
1.3352145449165	1.5780573982263
1.335447873572	1.5782640459488
1.335634049189	1.5783933754309
1.3358387810003	1.5784128903926
1.3360001088577	1.5787765655365
1.3361467279827	1.5788512654573
1.3363289295549	1.5789240426991
1.3365217713713	1.5789313148309
1.3368808517472	1.5790909632179
1.3370926171101	1.579420807127
1.3373265406433	1.5799459808504
1.3375294459293	1.5802141519712
1.3377677787565	1.5805208157264
1.3379464610369	1.5805801007519
1.338084148347	1.5809594143605
1.3382302552443	1.5809660227231
1.3384226183191	1.581038441097
1.3386105356838	1.5814823144812

1.3388600098499	1.5815833630761
1.339111053072	1.5815916812926
1.339375259862	1.5816133048654
1.3395621044297	1.5817532788523
1.3397266560042	1.5817984823927
1.3399470986355	1.5819029635667
1.3401747532822	1.5819461792921
1.340473440216	1.5822875453565
1.3407330869307	1.5827978391587
1.3409019694258	1.5828573252531
1.3411694919393	1.583457596271
1.3413587078248	1.5834984205982
1.3415741569898	1.5839151544649
1.3417215824522	1.5840329956499
1.3419461326979	1.584336171037
1.3421098617028	1.5848440177406
1.3423005731762	1.5850401974098
1.3424972079209	1.5855759991287
1.3427453665697	1.5857756668776
1.3430095298201	1.5862722743044
1.343170513668	1.5864323038727
1.3433457537357	1.5865328340636
1.3435673865586	1.5865740110215
1.3437608112086	1.5869217414265
1.3438844272612	1.5870982716795
1.3440732576667	1.5871851893446
1.344265092992	1.5871919499303
1.3444385050122	1.5875369965416
1.3446561630618	1.5876165402438
1.3449267707615	1.5877445275606
1.3451598201668	1.5889068281607
1.3453949688731	1.5894916135982
1.3455709287044	1.5897872318227
1.3458700173449	1.5912947569992
1.346189986641	1.5916618447728
1.3464154447572	1.5920359703911
1.346590775829	1.5920531963481
1.3467622495191	1.5921087956721
1.3470163157562	1.5926290690514
1.3472409200741	1.593347432834
1.3474178643696	1.5934017939711
1.3476533173771	1.5934203563149
1.3478085118562	1.5936447386746
1.3479636357835	1.594064329079
1.3481812435358	1.5941487426185
1.3484321113348	1.5943771430928
1.3486753622411	1.5950009331285
1.3488924211725	1.5952050822493
1.3491266571694	1.5958021282989
1.3493756071785	1.5959821884871
1.3495631239209	1.5960304384521
1.3497932985396	1.5965110974754
1.3499538010849	1.5966939589388
1.3501531998528	1.5969798958918

1.3504057757653	1.5972184040706
1.3506075382714	1.5977267173043
1.3508222931366	1.5978386468953
1.3509854241172	1.5979251989549
1.3511547588142	1.598027052604
1.3513251015088	1.5981308005471
1.3514904806508	1.5983121992786
1.3517987889673	1.5985457178429
1.3519783708413	1.5985787155147
1.3522022874026	1.5985903079885
1.3523934271702	1.5990113444187
1.3525957008837	1.5990622152584
1.3528976168707	1.6000724231517
1.3531368048286	1.6001243509972
1.3533935136419	1.6003587980135
1.3536006958565	1.6003691392483
1.3538061414332	1.6004523717411
1.3540253703455	1.6005008209943
1.3541647988298	1.6005962149997
1.3543785207936	1.6005995087516
1.3545713774243	1.6007015286839
1.3548221524506	1.6012562702648
1.355095039447	1.6013205105934
1.3552789789051	1.6013290598397
1.3554779702815	1.601853663686
1.3556355057958	1.6019637718177
1.3558024262147	1.6019808980195
1.3560859496517	1.6022769464132
1.3562910311144	1.6022954450418
1.3564900195019	1.6024656062134
1.3566675805246	1.6024838770757
1.3569430950562	1.6028209626875
1.357142724844	1.6031803536062
1.3573763707953	1.6037469252727
1.3575983334934	1.6042301238298
1.3577501685922	1.6043656946037
1.3579806924389	1.6046911058315
1.3581803283605	1.6047891155835
1.3583970167682	1.6049729801092
1.3585921780428	1.6052083503509
1.3588587036947	1.6055412948941
1.3590558342752	1.6056450342722
1.359337256262	1.605830287144
1.3595214279501	1.6062363152291
1.359858966394	1.6070593342244
1.360060040995	1.6072544891557
1.3602435411564	1.6073151492425
1.360422316031	1.6076752385337
1.360684482935	1.6078238210066
1.3608778500552	1.6082952897377
1.3610826110779	1.6084887901481
1.3613035523726	1.6089423129506
1.3615061791306	1.6091524274099
1.3617298320147	1.6091920243886

1.361973255459	1.6104730214964
1.3622740087501	1.6109332976506
1.3624647193642	1.611138525225
1.3627014363693	1.6113723114364
1.3629344848426	1.6119931157169
1.3631903028206	1.6128360961842
1.3633943893345	1.6129760176681
1.3635719840037	1.612980497802
1.3637063045275	1.6129972273948
1.3638504177949	1.6132651109952
1.3640660671123	1.6136152580067
1.364261677536	1.613642731535
1.3644613648433	1.6138251809203
1.3646539556969	1.6141178233395
1.3649234787809	1.6144944458594
1.3652199502111	1.6151659882359
1.3653674019997	1.6152120641491
1.3655828156644	1.6154234843
1.3658481845966	1.6173777503973
1.3660641763221	1.6175118140787
1.366290896458	1.6180668741328
1.3665366543444	1.6184074149361
1.366770219241	1.6184559924719
1.3670579581837	1.6186092582359
1.3672346799472	1.6189088373302
1.3676272697151	1.6198709266217
1.3678351200437	1.6204751416493
1.3680424154913	1.6206537788461
1.3682684983233	1.6207131572589
1.3684823009357	1.6207199408623
1.3687409005304	1.6213223752214
1.368966040413	1.6215833360412
1.3691205005992	1.6217892343626
1.3692905492699	1.621792548367
1.3694969836415	1.6222510856294
1.3696743749963	1.622619559994
1.3698547350185	1.6226428734167
1.3701341936334	1.6229891437411
1.3703460079094	1.6231210000102
1.3704844220434	1.6232680662042
1.3707448918447	1.6233985107328
1.3708724444305	1.6234817276009
1.3711715772926	1.6236777695827
1.3713284788996	1.6241422555445
1.371511976877	1.6241931927663
1.3716899300904	1.6242284222797
1.3718579129759	1.6243520524477
1.372108555297	1.6246418235171
1.3724358662551	1.6252444420587
1.3725931316068	1.6254640307878
1.3728372766763	1.6255578677671
1.3730402497926	1.6256943562505
1.3732531099476	1.6260011895488
1.3735187043013	1.6267036613137

1.3738296426301	1.6274654351317
1.374068394985	1.6276144881756
1.3742485806316	1.6276172088242
1.3744762236078	1.6278940654418
1.3746593177216	1.6283335532011
1.3747856983164	1.6283931533356
1.3750186970204	1.6287647753724
1.3751754317446	1.6288074680594
1.3753550656779	1.628979752148
1.3755678516762	1.6291348628282
1.3758859353744	1.6297184420548
1.3760317603147	1.6300128646298
1.3763402674333	1.6302023497515
1.3765840060401	1.6310695971118
1.3768045646369	1.6312341246853
1.3770208634673	1.6313961255959
1.3772913518088	1.631578246765
1.3775555282133	1.631813329346
1.3777568775126	1.6321313675848
1.3780106218892	1.633580757327
1.3781931795167	1.6338251115992
1.378536294227	1.6339577869633
1.3787912045509	1.6351461870718
1.3790199254543	1.63580238189
1.3792886725097	1.6360531581962
1.3794959132514	1.6360869958112
1.3796763992123	1.6361469145853
1.3799146066828	1.6367498712734
1.3801688511653	1.6368307139116
1.3803389096728	1.6368955930484
1.3805154386466	1.6372268432643
1.3807482607605	1.6373365359889
1.3809405734849	1.6375778731148
1.3811598173597	1.6380143555038
1.3814528296145	1.6385827369235
1.381661664761	1.6386293669759
1.3819019978393	1.6388257949131
1.3821094904151	1.6389639615016
1.3823934966842	1.639183095949
1.382642061725	1.6392975038473
1.3828806398222	1.6396049852989
1.3830979303523	1.6403720372821
1.3832749906998	1.6403909218275
1.3834892825439	1.6410827247528
1.3837968858009	1.6411609665029
1.3839892792739	1.6414761880679
1.384205568622	1.6417911363529
1.3844607437407	1.6420447260958
1.3846277591839	1.6420811714988
1.3848740212784	1.6421691140676
1.385037816131	1.6425136543895
1.3852362334199	1.6427032331024
1.3855910493372	1.643356578647
1.3857640244332	1.6434413120982

1.3859988192035	1.6434759979684
1.386345651455	1.6441759038113
1.3865590359513	1.6444638420417
1.3868245729243	1.6446113814163
1.3871225413544	1.6449594258074
1.3874788427067	1.645672432374
1.3877148342098	1.6462540379455
1.3880469158275	1.6469891331566
1.3882203418188	1.647230228553
1.3883449180421	1.6472360554747
1.3884861857798	1.6475981492635
1.3886446980294	1.6477659849874
1.3888120112901	1.6478105098087
1.3890291259603	1.6478800315581
1.3892261477033	1.6483889526335
1.3894152652312	1.6485755588328
1.3895927520481	1.6486791304456
1.3898673487414	1.649332001421
1.3900740626537	1.6494702261823
1.3902977480174	1.6497381108966
1.3905214899872	1.6499591457615
1.3907954867696	1.6499960501007
1.3909939172054	1.6503352978879
1.3912014963786	1.6503515924537
1.3914510483706	1.6508480625969
1.3916361287267	1.6513961573448
1.3919196682484	1.6521508634798
1.3921832226581	1.6528803034599
1.3923969394856	1.6528936313512
1.3925563695206	1.6529861694502
1.3928702486531	1.6536439628377
1.3931228394218	1.6539180778866
1.3933038770083	1.654081428363
1.3935238523054	1.6541686754525
1.3937223292891	1.6543067809601
1.3939354637179	1.6543983379423
1.394155615593	1.6546865918496
1.3944304847053	1.6549471636628
1.3947196853978	1.6552082383731
1.3948879990708	1.65521153502
1.3950974712712	1.6553805872432
1.3953705198623	1.6554762987869
1.3955716356222	1.6556426092173
1.3957819305729	1.65573499091
1.3959922275555	1.655814110661
1.3962683433372	1.6561178274385
1.396500687538	1.6561847326842
1.3967388478051	1.6562227832451
1.3969493081836	1.656436036443
1.3972306243989	1.6566019240495
1.3974920883888	1.6566718781472
1.3977424366955	1.6569419675483
1.397987768851	1.6572997863169
1.3982379004514	1.657782958112

1.3984337460701	1.658110764138
1.3986641545788	1.6581792336097
1.3989025479861	1.6582739804417
1.3990821424729	1.6583559771972
1.3992822124754	1.6589184018572
1.3995143275884	1.6593995419895
1.3997118186601	1.6599360651296
1.3998399993701	1.6601122735086
1.4000242611365	1.660241988737
1.400141322095	1.6604242332175
1.4003258158231	1.6605513447925
1.4005864839886	1.6606896992222
1.4008144978746	1.6607389671228
1.4011176536616	1.6616502311236
1.4014114788875	1.6627207812467
1.4017027440202	1.6630307953661
1.401972189871	1.6634574034448
1.402124787074	1.6636712315225
1.402296088552	1.6638880671634
1.4026032954463	1.6648060747674
1.402885805459	1.6658666623663
1.403087331532	1.66624838709
1.4032712429858	1.6664704329083
1.4035521886809	1.6672379287632
1.403752166766	1.6673016893972
1.4040341921212	1.6678897083218
1.404214741787	1.667969507754
1.4043979276655	1.6681933249492
1.4046468866092	1.6682067829933
1.4048525554665	1.6683444130079
1.4052082049056	1.6686646925391
1.4054449995706	1.668864090934
1.4056423051054	1.6689055396833
1.4058939517576	1.669569168204
1.4061548451061	1.6707715141751
1.4064332890615	1.6716533427914
1.406658350479	1.6719357697913
1.4068444890442	1.672211628722
1.4070019753567	1.6722163215689
1.4072421924492	1.6728671338392
1.4074935512666	1.6729017264903
1.4076890121329	1.6729137004538
1.4080141955556	1.6732236108882
1.408248113851	1.6737104524716
1.4085371185149	1.6740858927591
1.4088161207491	1.6746184771282
1.409003710779	1.6748564121119
1.4092213287116	1.6748981161731
1.409457473303	1.6753081278429
1.4097680105785	1.6753490469876
1.4099672583621	1.6754597637459
1.4101580203069	1.675600827606
1.4104363887232	1.6769166028956
1.4105993597816	1.6770895383316

1.4107874470004	1.6771288414508
1.4109542763036	1.6771901913206
1.4112753022389	1.6779838690456
1.4115627031914	1.6783160979682
1.4117529222619	1.6783940796246
1.4119325953832	1.6788329169589
1.412134211398	1.679222075661
1.4123439007279	1.6797675644532
1.4125098164124	1.6798065186587
1.4127146727816	1.6798584999184
1.4128789602481	1.6800518699918
1.4130909565249	1.6801867830481
1.4132630895424	1.6802469655184
1.4135021426162	1.6802711831508
1.4137019867886	1.6802810922727
1.4139591317314	1.6812031400928
1.4142151539697	1.6816633208152
1.414419722539	1.6817194588717
1.4146166709993	1.6819713791785
1.4148537251049	1.6820816769226
1.4150703932737	1.6821073354774
1.415385386079	1.6826535896941
1.4156742375729	1.6833744987859
1.4158858833703	1.6839141672937
1.4162019228991	1.6845430173376
1.4164212319518	1.6845651649568
1.4166039272946	1.6846512797942
1.4168887860341	1.6852228056865
1.4171304746337	1.68546255249
1.4174178027754	1.685735213475
1.4175694681424	1.6860301988748
1.417855713978	1.6861133532327
1.418170039492	1.6861298056832
1.4183811756574	1.6862101265592
1.4186339472807	1.686571177852
1.4188381768324	1.6867761151268
1.4190189977951	1.6869173804553
1.4192970976533	1.6875850215145
1.4195510585522	1.6877251127159
1.4197730495253	1.6877830689661
1.4199878472569	1.6878050218649
1.4202634593695	1.6879880909857
1.4204689879928	1.6886151945356
1.4207145093585	1.6887504814385
1.4209025512106	1.6888758896824
1.4211194405119	1.6889332927552
1.4214956090763	1.689390385565
1.4216961212587	1.6894471614517
1.4219856740345	1.6895757093078
1.4222757141429	1.6896694249054
1.422505381177	1.6897719118285
1.4227686398742	1.6900195838916
1.4230195448868	1.6901991682408
1.4233374857544	1.6902327998764

1.4236778055322	1.6912519775604
1.4238825908337	1.6913006451042
1.4241503129212	1.692031297726
1.4243139088901	1.692031979043
1.4245499578818	1.6921687848923
1.4248208130967	1.6928941776874
1.4250864524437	1.6934884929464
1.4252128084308	1.6935496665535
1.4254493206893	1.6936528165652
1.4256962660192	1.6936798719977
1.4259265191868	1.6940876910256
1.4261333929517	1.694126847678
1.4264237649165	1.694794586255
1.4266764946666	1.6951651582071
1.4269324674491	1.6957581650843
1.4271672436644	1.6962584803302
1.4273739997887	1.6967034439759
1.4274918689867	1.6971408685123
1.4277611115869	1.6976786670689
1.4280826532397	1.6989810438367
1.4283340918179	1.6989842763189
1.4286195775292	1.6997637432903
1.4288569682854	1.7004491686535
1.4290298165347	1.7009895604219
1.4293235047357	1.7011615858612
1.4295170093009	1.7012348219572
1.4297723517733	1.7016953580167
1.4299827839849	1.701699700922
1.4302153078397	1.7018438424466
1.4304410577432	1.7019215432278
1.4307286269642	1.7019877778331
1.4310126019269	1.7033556343912
1.4312976827492	1.7035942079374
1.4315314830316	1.7037848090318
1.4318537811887	1.7045479971711
1.4320305710442	1.7049740534998
1.4322388854329	1.7050551414007
1.432512876023	1.7055339954211
1.4327394956436	1.7055680419274
1.4330578961179	1.706050173066
1.4332151535788	1.7066108744418
1.4334385288744	1.7066344284189
1.4336865158724	1.7073846799183
1.4338525507323	1.7077233353528
1.4341072325431	1.7077994938534
1.4343819837939	1.7084641751305
1.434557819512	1.7085370258265
1.434788932597	1.7087176131239
1.4350514887174	1.7087733908886
1.4353021882719	1.7089965998096
1.4355711730112	1.709345609859
1.4357889684177	1.7094142863741
1.4360475011836	1.7100639636692
1.4361996498155	1.7103416223884

1.4364565007364	1.7106068847601
1.4367532230621	1.710752701606
1.4369462249474	1.7108196714739
1.4371444564457	1.7108565704562
1.4373684480519	1.7109702781668
1.4376931384223	1.7112402787101
1.4379354863439	1.7119561530558
1.4382522436653	1.7122924564463
1.4385206546509	1.7129884229732
1.4387616852322	1.7139053328376
1.4390847332804	1.7141301165389
1.439286546997	1.7143766254913
1.4396108888321	1.7146047815273
1.4397813074253	1.7146463287833
1.4400975733601	1.7151344259993
1.440282503787	1.7152609862292
1.4404989101924	1.7158287399148
1.4407058791834	1.7160097292973
1.4409113800204	1.7161165915001
1.4411674279689	1.7161717176924
1.4414026263212	1.7162382347925
1.4416444773373	1.7164076876071
1.4419500048794	1.7165612417075
1.4422389220896	1.7168657870133
1.4425133506622	1.7173487534549
1.4427598649442	1.7175464288921
1.442926540252	1.7179012218269
1.4431040502734	1.7180241458207
1.4434213456195	1.7181449877185
1.4437156435257	1.7186157440977
1.4440773457125	1.7196246844281
1.444363382741	1.7197943238463
1.4445930526483	1.7203534619317
1.444796649834	1.7203585432724
1.4450135130345	1.7204043805733
1.445275342681	1.7212003429017
1.4455412954746	1.7220654857447
1.4458169463429	1.7227025986386
1.4461603914512	1.7227517893948
1.4463974223706	1.72285354285
1.4467704180002	1.723860400213
1.4470516142768	1.7244622639661
1.4473174661607	1.7251813779144
1.4475482448592	1.7260658219216
1.4479331551068	1.7271994428725
1.4480900584727	1.7272619882379
1.4482844476457	1.7273633431765
1.4486024420716	1.728097512129
1.4488538122508	1.7286757122851
1.4491533479664	1.7289180384528
1.4494365049704	1.7294546272918
1.4496999623382	1.7296802904716
1.4499555324039	1.7297744848965
1.4502110707526	1.72996024217

1.4503879532804	1.7299782056271
1.4506250451566	1.7308766695197
1.4508472060401	1.731008234599
1.4510629133517	1.7310584610076
1.4512928072604	1.7311892072429
1.4515620302731	1.7315867803741
1.4518146909673	1.7316812075945
1.4520348211084	1.7317146330096
1.4523294035531	1.7325355863518
1.4525133261067	1.7325958283291
1.4527604241956	1.7327020533964
1.4530332838688	1.7327975490848
1.4533179057699	1.7330868682015
1.4536721485853	1.7336781350688
1.4539146343696	1.7341532835549
1.4541966667969	1.7343389623664
1.4544261433653	1.7344058212742
1.4547156820902	1.734670470574
1.454926654469	1.7351774914582
1.4551552367682	1.7352247696067
1.455417400556	1.7357874753922
1.4556578259867	1.7358367447447
1.4558543460886	1.7359371214994
1.4561057047327	1.7362743557021
1.4564297207331	1.736771341369
1.4566554640542	1.7368939415116
1.456952812267	1.7375793049277
1.4571016571184	1.7377971037765
1.4573722218564	1.7379596126741
1.4575707139601	1.738267054144
1.4578384015407	1.7389799973971
1.4581268510352	1.7396556612338
1.4583915862071	1.739803114566
1.4586629187053	1.7400270747384
1.4588286330175	1.7401311973294
1.4590324631615	1.7401570555567
1.4594031491167	1.7414434259655
1.4596349850958	1.7415137673545
1.4600087145672	1.7418077666785
1.4601685229256	1.7418254805854
1.4604686753395	1.7419517980134
1.4607610705438	1.7422032711345
1.4610715295557	1.7424245074612
1.46131093978	1.742434258232
1.4615073712709	1.74326677206
1.4618317654918	1.7432697511495
1.4621033410428	1.7433472644207
1.4624915132057	1.7443614402146
1.4626932194903	1.7444037693628
1.4630374786226	1.7450660178448
1.4633118076164	1.7452728312912
1.463521769204	1.7454893476831
1.4637873510944	1.7454899383343
1.4641130290358	1.7458730100263

1.4643808124877	1.7459022280814
1.4646924073175	1.7465238944441
1.4649462907814	1.7469082145686
1.4651101208786	1.7469957687923
1.4653878234907	1.7475862958867
1.465679073984	1.7488222758902
1.4658786335931	1.7490592055282
1.4661161102908	1.749175998361
1.466385864501	1.7493333369088
1.4666684916787	1.7495827830472
1.4669323494443	1.7498092317099
1.4672387084988	1.7505748641718
1.4674320299278	1.750599673388
1.467612500585	1.7507169988919
1.467900372733	1.751290684181
1.4682401702325	1.7514234199588
1.4685148741059	1.7518757337502
1.468858101327	1.7524032544098
1.4690491515528	1.7524491641305
1.4693648479458	1.7525042492058
1.4696388486559	1.7528763627131
1.4699369837141	1.753121039455
1.4701794668496	1.7533097411448
1.4704504159185	1.7533684506266
1.4707100124625	1.7534453701003
1.470980316516	1.7537740674453
1.4711104733648	1.7538263161386
1.471266321097	1.7538879675219
1.4715296155815	1.7539316386008
1.4717696754639	1.7546471962665
1.4720478164083	1.7553690733767
1.4723075366971	1.7555118211818
1.4725097128778	1.7555933746605
1.4726793461089	1.7558034308953
1.4728579907509	1.755956050839
1.4732029545615	1.7582706207397
1.4734441093325	1.7583575747184
1.4737140289314	1.758365791343
1.4739896158178	1.7586820893774
1.4742261769086	1.7593677973761
1.4745516319804	1.7595228900675
1.4748422229847	1.7599038363838
1.4750021437199	1.7599971694308
1.4752347643019	1.7600847063501
1.4754398560402	1.7603201382131
1.4756794316338	1.7609822753611
1.4759473568427	1.761191526738
1.4761187189587	1.7612096546379
1.476373288851	1.7612557628825
1.4766797792971	1.761817522186
1.4770474928007	1.7629027817774
1.4773463349798	1.7629391581373
1.4776249981464	1.7632372571337
1.4779048766915	1.7635660067371

1.4781661019338	1.7639697731075
1.4783936097399	1.7644422388458
1.4786594579335	1.7650583892059
1.4790158713408	1.7656167117156
1.4791701830684	1.7656816522597
1.4794580163019	1.7660800342168
1.4797577717915	1.7669084812342
1.480027599814	1.7678298369572
1.4803071721828	1.7681002815046
1.4805673261286	1.7688698828947
1.4808273006745	1.7689917512927
1.4810693342451	1.7694457657853
1.4813320913598	1.7698521564564
1.4815390757508	1.7705520303162
1.4818934205657	1.7706765443917
1.4821236619971	1.771714859781
1.4824047151199	1.7720591478657
1.4827024454472	1.7722154995816
1.4830467929571	1.7727266588834
1.4833279068602	1.7729723216512
1.4835427894922	1.7734227103499
1.4837019415475	1.7736248988797
1.4839462306819	1.7736695381594
1.4842056669077	1.7737559820636
1.4844205775408	1.7738190732323
1.4846256427045	1.7738995442687
1.4849790627909	1.7741117831267
1.4852241690432	1.7748631316076
1.4855419010849	1.7750928272979
1.4858210376581	1.7761827353752
1.486190296494	1.7774690179781
1.4864099877312	1.7775621906813
1.4866909972594	1.778007036801
1.4868997040778	1.7780721311119
1.4873385875679	1.7801824849105
1.4876148683701	1.7804071405093
1.4878770804344	1.7809198240618
1.4881144941857	1.7809638837515
1.488397078828	1.78096447267
1.4887522628399	1.7812202451712
1.4890240759666	1.7813500790171
1.4892583188413	1.7817595663323
1.4895847224792	1.7820949176562
1.4898146949328	1.7821729494361
1.490074789593	1.7822727336248
1.4903933497886	1.782667788525
1.4906724728199	1.7827254885388
1.4909467071496	1.7828616740531
1.4912118917281	1.783284940895
1.4915115688378	1.7835196121942
1.4917744057127	1.7838204560938
1.4920353383531	1.7844684859672
1.4923707514967	1.7845148667052
1.4926462338986	1.7847521961895

1.4929276831609	1.7852040111705
1.4932278277008	1.7852098423084
1.4934738768441	1.7852931671364
1.493829562899	1.7856501913235
1.4940345396276	1.785723748878
1.4942962006679	1.786039367149
1.4945634170859	1.7866633525606
1.4949547927736	1.7872832702936
1.4952637089432	1.7881193062089
1.4955208367396	1.7881642226321
1.4957234612999	1.7886634099204
1.4959656479091	1.7887604999158
1.4962015379816	1.7887801475387
1.4964064172967	1.7887848105967
1.4966874048758	1.7888166903567
1.4969925605312	1.7892349444487
1.4972785369071	1.7895127288163
1.4974740388444	1.7900213989872
1.4978037830335	1.7903835753683
1.4981997671561	1.7907013050333
1.4986401791791	1.7920078688693
1.4989298387242	1.792679158881
1.4992554338582	1.7926927536822
1.4994817607868	1.7926967049498
1.4997582649484	1.793713118987
1.4999514734802	1.7937456907117
1.5002717475081	1.7937879109028
1.5005675197835	1.7942799344247
1.5008471220032	1.7945598190187
1.5010993013488	1.7945908984088
1.5014020673111	1.7949344115669
1.5016728120818	1.7950914946284
1.5018767015945	1.7954291729116
1.5022781243521	1.7955272767277
1.5025378127178	1.7958096404496
1.502824503804	1.7958813725517
1.5031647462453	1.7961708806835
1.5034791758954	1.796303620717
1.5037123478181	1.7967870994295
1.5039572876482	1.7968665122105
1.5044806915015	1.7976504959651
1.5047860824904	1.797689640978
1.5051353238508	1.7985507566252
1.5054335390577	1.7994440415119
1.5056341052534	1.7996726676904
1.5058704872355	1.8003554314844
1.5061356650305	1.800431772948
1.5064112869029	1.8006295163951
1.5067376326758	1.8007198406898
1.507125271194	1.8025789738302
1.5074311751649	1.8026460074695
1.507819104261	1.8038088144862
1.5080358467499	1.8038659824265
1.5084089415303	1.8040762474526

1.5086632767718	1.8042621205754
1.5088829380198	1.8043484319363
1.509167119807	1.8046741049237
1.5095239446905	1.8048251583149
1.5098097809483	1.805225523506
1.5100910401174	1.8052634626891
1.5104231753088	1.8069745563862
1.5106069775108	1.8071833278688
1.5109830209245	1.8074309129549
1.5112583724025	1.8080585197775
1.5114769712917	1.8083878603822
1.5117309323561	1.8086095524931
1.5121577396059	1.8099376786807
1.5124738555426	1.8100641068118
1.5127527439067	1.8103357068423
1.5130637122412	1.810583620199
1.5134001132829	1.8106135456627
1.5136072103178	1.8108943083988
1.5138200305668	1.8112937491412
1.514209955609	1.8116812981868
1.514425307546	1.811866932549
1.5146462013705	1.8120217230079
1.5148829593751	1.8121282942458
1.5151215919528	1.8122603669386
1.5155009813836	1.8129053841062
1.5157783121995	1.813067473295
1.5160143229568	1.8132413469788
1.5162309823253	1.8135451422903
1.5166266400533	1.8137990260313
1.5170176658631	1.8146563034826
1.5173658732197	1.8153244803022
1.5176634606855	1.8167535115731
1.5178861461962	1.8172626765449
1.5182402519619	1.8179982406103
1.5185049903756	1.8182420374995
1.5187528183625	1.8183202404192
1.5190664543989	1.8185577718713
1.519295159801	1.8188376800471
1.5194870750462	1.8189056423542
1.5198147058637	1.8203647654094
1.5201524078721	1.8208823916029
1.5205743983552	1.8214868425816
1.5209404483019	1.8217767857784
1.5210913802689	1.8218942929619
1.5213000040666	1.8220926703893
1.5216230222398	1.8229190671287
1.5219306262639	1.8237500171227
1.5222779019631	1.8237805708515
1.5225469361758	1.8237952887182
1.5228313054456	1.8239727273953
1.5230729315634	1.8240952102968
1.523424054927	1.8244494813169
1.5236517650937	1.8245728203791
1.523993368171	1.8256485356341

1.5242087358677	1.8256567530683
1.5245433125941	1.8260844531955
1.5248129100418	1.8263904411843
1.5250372116872	1.826668079611
1.5253735998908	1.8267582414056
1.5256856821181	1.8268687443296
1.5260199277747	1.8271536744892
1.5263330418696	1.8276496510566
1.5266297796897	1.8284519870739
1.5269889536696	1.828456008864
1.5272366793126	1.8289012410735
1.5274914047507	1.8292623893263
1.5276877667036	1.8293361574501
1.5279728455523	1.8298920023037
1.5282009534074	1.8302171702583
1.5284194897322	1.8303600021289
1.5286975741636	1.8305018552758
1.5290327041132	1.8308007979673
1.5292924509078	1.8311220465234
1.5295690975459	1.8317912297762
1.5298807344779	1.8320279412955
1.5301246194786	1.8323597347905
1.5303538230253	1.833334516134
1.5306895071715	1.8333403661961
1.5309792216656	1.8335718693342
1.5313592102475	1.8348539068798
1.5316118795298	1.8349068344585
1.5319267008248	1.8356477585169
1.5321943497684	1.8363906225863
1.5325400815799	1.8369666566201
1.5327677947277	1.837017003988
1.5330021227577	1.8371588198577
1.5332839723317	1.8371660982421
1.5335303515397	1.8375718584093
1.5338904292301	1.8378130643682
1.5340960625859	1.8380734564075
1.5344900249013	1.8381632453249
1.5347756018494	1.8386109568615
1.5350808161904	1.8388218888075
1.5354871987405	1.8390601484857
1.5357454810502	1.8391448060209
1.5360904713185	1.8393254052134
1.5364514995197	1.8398559065813
1.5369439385826	1.8402618867444
1.5371785213361	1.8402956698549
1.5374901101088	1.8404192377421
1.537838474217	1.8404820083901
1.5381968912866	1.8405493949492
1.5384700144671	1.8408478272717
1.5387682669154	1.8408858675665
1.5390105341624	1.8413017436145
1.539231879774	1.8413149419053
1.5396104687142	1.8417625694242
1.5399197486497	1.842721284921

1.54019782687	1.8430820122016
1.5404185257599	1.8433275301935
1.5407641202411	1.843714378228
1.541085297994	1.8443171906845
1.5413635347048	1.8446684347095
1.5418092391816	1.8471570932206
1.5421709744748	1.8472800882341
1.5425211823282	1.8480191571263
1.5428116706428	1.8480639258892
1.5430339995103	1.8486872064172
1.543327453541	1.8489561630262
1.5435286245593	1.8495614959008
1.5437655977873	1.8496366888627
1.5441162361797	1.8498213690931
1.54453102097	1.8512635237353
1.5447687829799	1.8513732537873
1.54500656143	1.8519884017868
1.5453151570522	1.8527395623643
1.5456063521953	1.8533251437445
1.5459287314955	1.8540690215373
1.5461941011924	1.8549577991405
1.5466122671788	1.8559194592941
1.5469045166126	1.8560073011
1.5472617690511	1.8571489517854
1.5476249132854	1.8575580102922
1.5480318598978	1.8576126173714
1.5482956052175	1.8577605483608
1.5487016981354	1.858141194404
1.5488928794572	1.8581498709925
1.549242325801	1.8583540711755
1.5494757858719	1.8584275025602
1.5498039571572	1.8591449511103
1.5500994165916	1.8592782854308
1.5503603019449	1.8593167095191
1.5506685304712	1.859642787566
1.5508658823046	1.8598222695063
1.5511991466927	1.8607070466703
1.5515358376765	1.8609808395934
1.551839424037	1.8612582964658
1.5521556433252	1.8612879632917
1.5525732127936	1.8623585925678
1.5530175566666	1.8636834170021
1.5532611409293	1.8638524375658
1.5536159912949	1.8640739305033
1.5538540450821	1.8642109477264
1.5541570456173	1.8645295299846
1.5545287128084	1.8645630512464
1.5547804108874	1.8650790953304
1.5550214439995	1.8655864662837
1.5552578536511	1.8659901166691
1.5555655202403	1.8668644861729
1.5558626598651	1.8672447520895
1.5561142678896	1.8677086653096
1.5564575415839	1.8681798308435

1.5569045003888	1.8682302414064
1.557175068542	1.8682604935702
1.5575011516446	1.8683968471515
1.5578543272636	1.8685846994711
1.5581638155752	1.8691978209375
1.558472906988	1.8695241723873
1.5587901837458	1.8696305008641
1.5590042915201	1.8697360844026
1.5593140169388	1.8701610505247
1.559541736298	1.8707279620215
1.5597825355218	1.8707765733829
1.5601592283765	1.8712046671611
1.5604561545135	1.8713496149342
1.560727023866	1.8723175495328
1.5610041248825	1.8723892897514
1.5612895701664	1.8726109629738
1.5616264687553	1.8737173490529
1.5618987424289	1.873902271822
1.5622459274402	1.8742545300219
1.5626422426913	1.8752213076634
1.5630215373839	1.8760387316344
1.5634162052795	1.8765208411458
1.563716236991	1.8768845957024
1.5640725339814	1.8781830854357
1.5643838246585	1.8786226776934
1.5646795724885	1.8788142879496
1.5650048780636	1.8794182251803
1.5652626773999	1.8797211866051
1.5656999897401	1.8800486121363
1.5659749418678	1.8803101129114
1.5661839540272	1.8803827147332
1.5666475262234	1.8818293684742
1.5669748325531	1.881958885144
1.5674100376015	1.883960448955
1.5677944770241	1.8841064200536
1.5680729712486	1.884949472678
1.5685227379509	1.8851436358203
1.5688498467268	1.8852113151997
1.5692233619895	1.8858716655813
1.5696311623607	1.8865515466192
1.5699513559855	1.8870179284011
1.5702313222064	1.887303465857
1.5705757374147	1.8877520679965
1.5709148605146	1.8877803320888
1.5713034438985	1.8882870038287
1.5717257297885	1.8883751514173
1.5720653082356	1.8885332294925
1.5725354120506	1.8894055126592
1.5728535805136	1.8905032799283
1.5731756102092	1.8907596574966
1.5734941198051	1.8910623268358
1.5738591357422	1.8915950522977
1.5743404626701	1.8934035011452
1.574671344692	1.8945760818336

1.5750988007088	1.8949836240256
1.575429760601	1.8952045761305
1.5759068895503	1.8953646405862
1.5762107958402	1.8958074240161
1.5765528278082	1.8959200247861
1.5767823352642	1.8959815300667
1.5771341031552	1.8961180011824
1.5774100408243	1.896233258927
1.5776544066254	1.8968534886062
1.577900159954	1.8973408024261
1.5781666626676	1.8973570361814
1.5784619639452	1.898194009699
1.5787515321213	1.8986248092569
1.5791309461807	1.8996636903458
1.5794331466916	1.8997056835236
1.5798171386032	1.9006321357085
1.5801843709037	1.9009385982204
1.5804532256311	1.9017424766273
1.580757719859	1.901794621567
1.5810354146838	1.9019542764222
1.5813544192312	1.902124687456
1.5816988184818	1.9027041647044
1.582068315208	1.9029234925114
1.582486804317	1.9047252759467
1.5828356875885	1.9053534941731
1.5831589064023	1.9057170260366
1.5834118583232	1.9058538320782
1.583720969781	1.9063602901134
1.5840179685041	1.9064743310012
1.5843621161447	1.907437680374
1.584798423947	1.9087343534118
1.5851907644366	1.9094076658351
1.5854331841206	1.9097596431588
1.5856267510741	1.9099537630176
1.5860726764337	1.9115989479659
1.5863467785895	1.9117594368525
1.5866868964898	1.9123008522976
1.5870006061361	1.9124661256043
1.5872988262264	1.9126578087475
1.5876394882118	1.9127545214263
1.5880643314734	1.9129575824924
1.5882590568228	1.9133759287781
1.5886683687608	1.9139290180646
1.5889134645738	1.9142280615163
1.5891351733722	1.9145024629551
1.5896359056391	1.9162987289362
1.5899736069456	1.9164730660185
1.5904217596766	1.9170078282553
1.5907168977865	1.9170206386695
1.5910500956432	1.9178672660659
1.5913770523473	1.9182539944416
1.5916141365629	1.9184035561259
1.5918633843685	1.9186431953321
1.5923272706861	1.9193552123158

1.5926358245812	1.9196280729754
1.5930120863426	1.9205030213623
1.5933866643275	1.9206817998082
1.5937731487386	1.9216092746913
1.5941327094064	1.9217961415881
1.5944939513159	1.9222269833973
1.5948381866628	1.9222544562339
1.5952373570015	1.9225173838383
1.5956090563228	1.9228983139871
1.5959062888936	1.9235065780965
1.5963100983673	1.924288989405
1.5967754169179	1.9254144676826
1.5972132530194	1.9254756885026
1.597689029341	1.9270796479832
1.5980830813306	1.9274995743888
1.5984102797355	1.9278195610089
1.5987510116049	1.9278608892405
1.599091756929	1.9279342096868
1.5994288036357	1.9283479331543
1.5997921474431	1.9294360086714
1.6000225953514	1.9295505579058
1.6002491526852	1.9296262086876
1.6006059910993	1.9299543147113
1.600844084703	1.9301217856486
1.6011611689766	1.9302126222975
1.6015114586019	1.9315526458992
1.6018795624275	1.9324021303528
1.6022603099559	1.9329915116185
1.6025303637852	1.9338400128199
1.6028465597493	1.9340620604544
1.6032196829621	1.9348041075763
1.6035866167102	1.9355086774331
1.6039066061392	1.9367220711436
1.6042339788204	1.9377752110435
1.6045343934042	1.9378426581446
1.6049240874859	1.9381247194293
1.6051935250188	1.9386000511011
1.6055438392151	1.9387590215705
1.6058470594201	1.9389491082459
1.606126593097	1.9392076430312
1.6065540771346	1.9393222971762
1.6069665122656	1.9397424115273
1.6074175134123	1.9400073249209
1.6077404501099	1.9407356542422
1.6080709069491	1.9409918767332
1.608432676848	1.9412754441076
1.6089151391544	1.9417060234853
1.6091972058383	1.941962953525
1.6094503629662	1.9421415416418
1.6098023763901	1.9425084592525
1.6101674650423	1.9426646407981
1.6104188387783	1.9427836901412
1.6107512670646	1.9433418140923
1.6111407392966	1.9441402971109

1.6113661283607	1.9441537541028
1.6116617104909	1.9442823802964
1.6120545966182	1.9443997013576
1.6123929469995	1.9444950126905
1.612833751924	1.9453709997186
1.61314166899	1.9453769290279
1.6135758224057	1.9455323708847
1.6138740999307	1.9457881596914
1.6142012916273	1.9457953116852
1.6145500835773	1.9459106191166
1.6150175534818	1.9472556820675
1.6154495672681	1.9472705898528
1.6158108856533	1.9479351739724
1.6160266972855	1.9482141065932
1.6162638215784	1.9487397519022
1.6165045438028	1.9488067168831
1.6169277548902	1.950369512453
1.6173341804577	1.9513348244384
1.6176219462812	1.9514383876696
1.6179617756987	1.9515797636141
1.6182988292611	1.9528333746141
1.6187358195069	1.9529806622355
1.6191151842654	1.9534178857746
1.6194265227137	1.9537967344351
1.6197561183709	1.9539481577215
1.6201434460169	1.9558476525805
1.6204593750645	1.9561937932227
1.620734435471	1.9562319456505
1.6209203927862	1.9564751318453
1.6212571661681	1.9564925708166
1.6217168514831	1.9571963770196
1.6221101297889	1.9575488616345
1.622341214568	1.9575687070715
1.6227357154804	1.9581035247602
1.6230567248665	1.9582343419055
1.6236060595082	1.9590243427613
1.6238708697536	1.9591637491155
1.6243437245694	1.9594067466875
1.6245695875024	1.9595118119279
1.6249202789859	1.9595709802019
1.6253155237515	1.9605249126884
1.6256950745833	1.961275734639
1.6261266474085	1.9628286727268
1.6266041138461	1.9637332430387
1.6270446397652	1.9642612312272
1.6274520276689	1.9644092810176
1.6279187098141	1.964699575005
1.6283967352784	1.9650534413516
1.6286740321394	1.9655096156852
1.6290311696335	1.9659007699078
1.6293428831303	1.9659182970648
1.6297605955115	1.9674655896531
1.6302093710206	1.9681051364585
1.6306325285198	1.9681925031686

1.6311635272451	1.9687245461096
1.63139247612	1.9687276001049
1.6316992439212	1.969309373755
1.6321198220368	1.9693789826697
1.6323680347912	1.9695950719374
1.6327491281002	1.9699504523982
1.63305899326	1.9704312506299
1.6334839176194	1.9704705097841
1.6338533329082	1.9712119306956
1.6343559376318	1.9721419044631
1.6347560363114	1.9722269021782
1.63510681899	1.9724475491278
1.6354507192164	1.9737295992396
1.6357880817764	1.9737442338922
1.6362133060782	1.9739729649337
1.6365432149399	1.9743050632221
1.637016047082	1.9751385589526
1.6373655159478	1.9762453024922
1.6377265176716	1.9779423713757
1.6380775514672	1.9782758913845
1.6384609903886	1.9791268554876
1.6388953815988	1.9795832397391
1.6393047349536	1.9796013510991
1.6396553103163	1.9797145692371
1.6400767324324	1.9799509359597
1.6404111819837	1.9803639060851
1.6406721793597	1.9805598178528
1.6408888255736	1.9806430995689
1.6411615344126	1.9808486951564
1.641586987818	1.9816120422125
1.6418956662412	1.9816466887102
1.642305031901	1.9829285334818
1.6425989244216	1.9835310309373
1.6430006969883	1.9837361619006
1.6434761897603	1.9838341623317
1.6438358010534	1.9850396385016
1.6443187371904	1.9853719420689
1.6446658277458	1.9853823877935
1.644977001312	1.9856872135958
1.6452648726236	1.9858857336319
1.6455070289761	1.9858959435788
1.6458812123924	1.9868897521394
1.6462045556518	1.987040309722
1.6465931403126	1.987306470112
1.6469782606778	1.987833881617
1.6473489220578	1.9882047009737
1.6476681575234	1.988516196398
1.6480227825248	1.9886607177169
1.6484607206243	1.9889597340011
1.6488261513073	1.9890911699559
1.6491766570677	1.9893206828727
1.6495267495557	1.9893898006586
1.6499208580998	1.9900241027131
1.6504847936928	1.9915270548925

1.6508007207924	1.9915476518604
1.6512615793732	1.9942320403817
1.6516797161811	1.9959084644507
1.6520411695568	1.9967147845552
1.65242094049	1.9977042420687
1.6528195685842	1.9983475668609
1.6532526426205	1.998577702001
1.6536680726324	1.9999568690154
1.6539855803327	2.0002170826888
1.6545223333083	2.0014289674352
1.6549101594526	2.0020091578449
1.6553707364887	2.0026927562851
1.6557558490854	2.0037019578885
1.6561220158757	2.0049233637642
1.6564227499484	2.004957889327
1.6568440338388	2.0054049792569
1.6572376429587	2.0063275365206
1.6575959513005	2.006776429105
1.6580356139012	2.0071039543809
1.6584363615419	2.0073841820473
1.6586277121968	2.0074142905755
1.6589432500705	2.0076186794223
1.6591838409033	2.008213927079
1.6596751119726	2.0105368663171
1.6599335022	2.0106386698881
1.6602549352081	2.0115960656069
1.660649175007	2.011717765542
1.6610288921875	2.0119825045392
1.6615022158754	2.0137510117923
1.6618395935795	2.0139466024062
1.662136200382	2.0141145594012
1.6625508490698	2.016096866548
1.6628410882346	2.0162778770887
1.6632568159968	2.0163567634767
1.6637499911839	2.0163775294158
1.6640852648167	2.016523075868
1.6644896196615	2.0170713583804
1.6648908624504	2.0175627835355
1.6651772849525	2.0183743208213
1.6656269972984	2.0186019236493
1.6659638823267	2.0187992122248
1.6663818528231	2.0188735402933
1.666736649723	2.0199689728204
1.6671093410738	2.0205212236449
1.6675468558343	2.0208391338616
1.6681050464879	2.0224867791127
1.6683583007616	2.0228542592821
1.6686853647893	2.0229036933927
1.6691377847996	2.0230930258949
1.6694317700955	2.0231186592676
1.6697734483118	2.0232941483531
1.6702098808205	2.02341340855
1.6706596508756	2.0246911476947
1.6712650949925	2.0248554157274

1.6715362436405	2.0249433898305
1.6719101448926	2.0250847659109
1.6722036142801	2.0252109373407
1.6724987942162	2.0252290692363
1.6730019641026	2.0265657385606
1.6734646956739	2.0266981805391
1.6738775013175	2.0280144304277
1.6742035559991	2.028280532455
1.6746122808439	2.0283103770542
1.6749594701833	2.0285898070134
1.6753760355768	2.0286237685549
1.6758539992502	2.0292704071997
1.6762751920877	2.031518252237
1.6766490877884	2.0317153607456
1.676990282106	2.0318643784919
1.67752052795	2.0329448236805
1.6781105490211	2.0346133016346
1.6785553538105	2.0358128457423
1.6789698411907	2.0358324675046
1.6794473959682	2.0363704207154
1.6798445481622	2.0376812727894
1.6803252776093	2.0378412299399
1.6807233164579	2.0392235214481
1.6812409911832	2.0397430030238
1.6815238016162	2.040284759168
1.6819285348695	2.0405388455599
1.6823726813229	2.0409530622421
1.6828160532292	2.0432477474112
1.6831101394469	2.0435740300813
1.6835576636691	2.0438147675433
1.683908554074	2.043909819214
1.6842475520522	2.0440235061508
1.6845725891108	2.0444871021062
1.6850478629854	2.0454574800626
1.6855479383897	2.0462461466876
1.6859367953462	2.0463374523108
1.686452361589	2.04645419126
1.6867645237319	2.046731092609
1.6871696314254	2.0471859920822
1.6875175510261	2.0476056397883
1.687938311202	2.0478385993149
1.6884069160626	2.0482172360374
1.6888843292762	2.0501357819187
1.6893300461097	2.0508829022369
1.6896319854594	2.0512764113569
1.6900467682005	2.0517000959782
1.6905569214035	2.0543270119039
1.6907898353196	2.0544252823013
1.6912185050258	2.0566246851136
1.6916124579533	2.0566385678544
1.6920390923672	2.0569118213055
1.6925551815532	2.0571037779982
1.6928901493715	2.0577741418433
1.6933053098224	2.0579638105816

1.6936436911974	2.0583283240774
1.6940957967568	2.0583588392446
1.6944132603067	2.0592004770235
1.6948808024326	2.0604432774001
1.6952754750461	2.0605455637942
1.6955702522876	2.0615480313138
1.6960988060804	2.0615547320347
1.6965285148907	2.0629143534326
1.696915806684	2.0636606627337
1.6972340192253	2.0637600733416
1.6975915913081	2.063861247767
1.6981235634535	2.0665686299957
1.6986694962289	2.0685411519933
1.6991691248498	2.0709862473045
1.6995500397692	2.0710787458214
1.6999166376649	2.071167356055
1.7003616924001	2.0714226359396
1.7007901738216	2.0714600185905
1.7011871526023	2.0724351692717
1.7015585825262	2.0726106123466
1.7019331681723	2.073413462665
1.7022127181628	2.073863663875
1.7026311690262	2.0740430575642
1.70302308297	2.0743525528844
1.7034221696345	2.0746764965057
1.7037657487181	2.0749063226079
1.704083121408	2.074980574409
1.7045096170065	2.075680526475
1.7049580440922	2.0758305634361
1.7054042357928	2.0773754206908
1.7058080444482	2.0775181130064
1.7062316470168	2.0784809399794
1.706695987725	2.0791101890776
1.7071070371654	2.0801056252014
1.7075461199514	2.0803332053701
1.7078677216558	2.0808184699589
1.7082289593461	2.0810403998682
1.708686589736	2.0824163592346
1.7092244779849	2.0828118053952
1.709549200695	2.0829977307793
1.7100723541039	2.0849623006403
1.7106465820594	2.0851677798228
1.7110885682142	2.0863022861525
1.7114764767545	2.0864914037431
1.7118569509015	2.086499196507
1.7122042804103	2.0865298424984
1.7125621238879	2.086923780059
1.7129460991625	2.0873367440148
1.713422792136	2.0875747066898
1.7138587096146	2.0877305931186
1.7143029217151	2.0877578237645
1.7148992111879	2.0903957914509
1.7154052774377	2.0915846964086
1.716045845949	2.0917270086786

1.7164058548962	2.0932299882981
1.7167565232154	2.0935673390952
1.7172870664556	2.094781759342
1.7177172255012	2.0948277862035
1.7181496571476	2.0952901857932
1.7184109890133	2.0956145759452
1.71901349113	2.095685670268
1.7195294463144	2.0959091583192
1.7197866894983	2.095934928879
1.7202925766968	2.0963714828648
1.7207375287509	2.0970339230128
1.7212998780003	2.097241451765
1.7217086173703	2.0981800794518
1.7220642364486	2.0983229987561
1.7224268533625	2.0984031655457
1.7229727829795	2.098963256317
1.7234472062405	2.0991529380359
1.7238112365665	2.1001172302116
1.7241303189422	2.1001706223785
1.7247571065418	2.1012085216099
1.7251328467217	2.1015151420146
1.7256139160415	2.1015967691504
1.7259712619971	2.1028739581876
1.7265684170519	2.1035749981363
1.7270192926017	2.1038216722701
1.7274906673861	2.104082039063
1.7278580173559	2.104329569956
1.7283366566503	2.1048122371859
1.7288762198293	2.1053464968353
1.7292825054743	2.105468141802
1.7298287150635	2.1054768517185
1.7302321900151	2.1059226903581
1.7306237388015	2.1063891688108
1.7312038840661	2.1078420375295
1.7316234058642	2.1078905705525
1.7321371618346	2.1078965085788
1.7326209796497	2.109246754191
1.7331826592313	2.1094749893301
1.7335149967399	2.1099551813938
1.7338517684389	2.1100177370407
1.7342812615065	2.1103615749619
1.7346939592551	2.1112138420544
1.7351666585803	2.1143810300371
1.7356849250055	2.1144737950823
1.736181754279	2.1145402751077
1.7364970685952	2.1147408208787
1.736902764062	2.1155386418597
1.7374153953379	2.1157143873738
1.7379851779469	2.1160888409888
1.7384293019193	2.1161670277392
1.7389703733317	2.117414836136
1.7393984508946	2.1185632578976
1.7398630659402	2.119043934874
1.740398700926	2.1197184511779

1.7408882164896	2.1199648348489
1.7414258913027	2.1203983427124
1.7419004555378	2.1205159553038
1.7423023346532	2.1210416665097
1.7426421457349	2.1214682330149
1.7432006043906	2.1225683637581
1.7437692712537	2.12312212242
1.7442751431772	2.1231446709556
1.7447690034351	2.1235244560758
1.7452186736507	2.124051693209
1.7456380478939	2.124705978824
1.7462822961544	2.1279620142729
1.7466668402043	2.1284357615735
1.7471546462104	2.1290831169256
1.7475863335962	2.1291602755278
1.7479827722186	2.1293540932377
1.748455798658	2.1299434948166
1.7490197816907	2.1299472828832
1.7495198238027	2.1302364999266
1.7503548233237	2.1355618472725
1.7508968846469	2.1365717065393
1.7512542454407	2.1365992049116
1.7517221060919	2.1367715401045
1.7521478483953	2.1369026734158
1.7525361917616	2.1381409982075
1.7529833404546	2.1383966162324
1.7535894831076	2.1393792313231
1.753894491462	2.1394231870108
1.7542245101666	2.139507439834
1.7547459661948	2.139720300893
1.7552344912677	2.1412989131258
1.7556454988766	2.1414515350338
1.7561838352989	2.142811550137
1.7566694023683	2.1428727668027
1.7572434002078	2.1430002589062
1.757695776949	2.1433807208613
1.7580991737692	2.1442461339239
1.7585789353072	2.1444943332257
1.7590952187698	2.1466147105778
1.7595994655956	2.1494202563361
1.7601862820612	2.1512667973067
1.7605229337532	2.1513413083791
1.7610509495786	2.1516805601476
1.7614496560787	2.1536359798852
1.7622521155381	2.1547246459247
1.7626749913068	2.1551158769609
1.7630951608289	2.1566162015906
1.7635522396834	2.1576520390924
1.7640737957265	2.1598829950183
1.7644248655652	2.1599020506249
1.7649685415844	2.1609775669064
1.7653819439241	2.1611099281079
1.76578609005	2.1616437808773
1.7663814721396	2.1619351092839

1.7668075294342	2.1621435185922
1.7671680090875	2.1626609919501
1.7676365154527	2.1633467567714
1.7680083162333	2.1636650337984
1.7687459516894	2.1650612378668
1.7692164097622	2.165325201475
1.7697051857373	2.1655608403731
1.7701410042667	2.1666791002301
1.7706296055919	2.168236283829
1.7712096422948	2.1683306675405
1.7716959693099	2.1712160977456
1.772125798216	2.1718942011379
1.772629809491	2.1722781049002
1.7730886475294	2.1723390650599
1.7734936331955	2.1731151976164
1.7738478643212	2.1731467027741
1.7743651764772	2.1745684429115
1.7747291062951	2.1748740608569
1.7750561779606	2.174972973474
1.7755004136042	2.1750751259496
1.7760019177291	2.1759227387818
1.7765570171115	2.1768496439092
1.7770542303096	2.1772570155988
1.7775722063551	2.1784179892303
1.7781229859482	2.1792891796648
1.7785572040559	2.1805407165415
1.7789823597708	2.1805623178561
1.7793613717811	2.1814063030596
1.7798470326832	2.1821946097133
1.7802782591446	2.1822671030147
1.7806600263611	2.1824261902645
1.7813790842091	2.183147499698
1.7817791404506	2.1832636603686
1.782469260492	2.183545392751
1.7829526755333	2.1844307149711
1.7836844083452	2.1855723737336
1.7843104234527	2.1856553902503
1.7846610000711	2.18584680674
1.7851424990721	2.1864340232621
1.7856902478673	2.1879026049466
1.7862469380803	2.188085490945
1.7868172459507	2.188440825335
1.7873230828897	2.1892465341107
1.7879400128521	2.1893880384113
1.788548078414	2.1898101113781
1.7890115143724	2.1899295111947
1.7897296982311	2.1906174279984
1.7903023933498	2.1907148609782
1.7907724533956	2.1914390572721
1.7913315627486	2.1916112892881
1.7918013825696	2.1926605231751
1.7924866657328	2.1946925173797
1.7930883868376	2.1950245727816
1.793388316013	2.1954637859543

1.7939989935532	2.1961087268347
1.794559706388	2.1961148037784
1.7950456213555	2.1973953391899
1.7954933552604	2.1975444338022
1.795965997109	2.1977976130498
1.7965607798872	2.1979143645156
1.7971318640173	2.1982615542422
1.7975502187532	2.1983081339949
1.798110052517	2.1985276539383
1.7985787231469	2.1990989221834
1.7991724880816	2.1992915880671
1.7996003357598	2.1999258939908
1.8000295357291	2.2007053853694
1.8006277939625	2.2009804281866
1.8010016131177	2.2012192027364
1.801558081618	2.2015192894252
1.8019912162527	2.2015446153025
1.8024289643735	2.2022754617005
1.8031469808347	2.2039050640543
1.804037167568	2.2069474127279
1.804691589672	2.2101362150975
1.8052294090821	2.2110323731401
1.8057941854281	2.2121881977609
1.8065332514965	2.2125988386403
1.8068614496234	2.2133158499283
1.8073513066889	2.2133266374649
1.8080254365328	2.2151977258898
1.8085491909671	2.2159330171077
1.8089713497785	2.2161925011641
1.8093922951537	2.2164253996073
1.8098921137686	2.216837057769
1.810384389513	2.2175620858985
1.8110159760256	2.2199278491311
1.8114991593389	2.2205602928102
1.8119896637076	2.22223732489
1.8125547272771	2.2228950145569
1.8131299792051	2.2233424731163
1.813606755394	2.2245238998147
1.8142765220337	2.225351090979
1.814626450393	2.2255121613669
1.815077427781	2.2256168904649
1.8154312441928	2.2256242860023
1.8158455283791	2.2260049683249
1.8163707786862	2.2260196484799
1.8169061885481	2.2277562084637
1.8175578970908	2.2283799055012
1.8180724298911	2.2295839701676
1.818800748318	2.2296027459774
1.8192497506598	2.2298626405882
1.8198395596265	2.2317193516534
1.8204204770268	2.2328940141307
1.8209317725998	2.2329310329789
1.8215604808749	2.2346078333746
1.822140675525	2.2349809078291

1.822576529872	2.2351572823578
1.8232090551647	2.2354539414867
1.82371196417	2.236702973338
1.8241966061773	2.2369872571255
1.8248148252786	2.2373497904124
1.8254178648238	2.2385441247504
1.8258972694716	2.2392170000402
1.8263169980938	2.2392374597022
1.8268359933185	2.2396451799048
1.8274091733767	2.2410709798885
1.8278702088775	2.2426867299191
1.828670960571	2.2427209972392
1.8292037014468	2.2427576079463
1.8296932841319	2.2428164836761
1.8303561241609	2.2436533553124
1.8309583974072	2.2446261248333
1.8315661043013	2.245330593435
1.8322174699062	2.2455871835259
1.8328404452689	2.2463598262145
1.8332752414896	2.2463845854082
1.8337856135366	2.2473368019527
1.8344319566526	2.2482506724404
1.8352739073536	2.2483123110332
1.8361109237925	2.2525632831565
1.8366306285681	2.2533799209842
1.8371003890712	2.2535606616676
1.8376673059759	2.2549015263281
1.838229648626	2.2560469982569
1.8388332541114	2.2595774334285
1.839450704446	2.260023742779
1.8399692381197	2.2600833919213
1.840406855766	2.2621284658008
1.8408331264554	2.2624990166067
1.8415513708599	2.2650431216522
1.8422316906116	2.2658370468341
1.8426496032474	2.2669073220678
1.8432151171882	2.2672128358143
1.8437710579251	2.2677247861991
1.8445495209099	2.267843301855
1.8448576101323	2.2681909612002
1.8453791128582	2.2697576323761
1.8461869106272	2.2723144293414
1.8466648573285	2.2730374202502
1.847167997632	2.2750390595862
1.8477428520749	2.275285301848
1.84830919171	2.2763007199586
1.8488547835015	2.2774787003476
1.8493463457918	2.2787984445092
1.8498803571142	2.2790505256157
1.8503395651259	2.2790692621534
1.8506908335059	2.2791395950155
1.8511929631612	2.2794451010862
1.8517331841163	2.2799874135546
1.8522557418818	2.2815834395772

1.8526594316888	2.2817035494149
1.853288141669	2.2828782444007
1.853923975204	2.2843435119906
1.8546903710893	2.2847017584594
1.8551684166886	2.2858045323426
1.8557320336444	2.2867729008621
1.8561306002397	2.2868265056262
1.8567577538645	2.2877622441199
1.8574693450652	2.2890184728215
1.8581166775977	2.2897728213156
1.8587865022373	2.2915471651468
1.8595590394874	2.2927446507719
1.8601929587497	2.293513146493
1.8609304628067	2.2942580139216
1.8615697722675	2.2947856496651
1.8625568271495	2.2960563827493
1.8631184801162	2.2975205778153
1.8637405709119	2.2979470725103
1.864196258696	2.2983918374277
1.8648230333952	2.2986340124164
1.8653177658782	2.2988807311605
1.8657701767504	2.2996513293133
1.8664263131703	2.2998045060916
1.8669656957643	2.3002964711564
1.8676619501614	2.3016195969233
1.868264604546	2.3027955051709
1.8688715506973	2.3043008175974
1.8694078283326	2.304448418992
1.8703873207418	2.309621959834
1.8710506796284	2.3111407995889
1.8716349139109	2.3128272737555
1.8721813547406	2.3132769256538
1.8728643066847	2.3135031779122
1.8734705816794	2.3142168625284
1.8741430383598	2.3166795745694
1.8746504145512	2.3172043026241
1.8751675885137	2.317239707529
1.8758099458126	2.3175670100991
1.8763949644892	2.3176022225782
1.8769882091122	2.3179066573641
1.8775203044389	2.3199320913514
1.8781620872534	2.3202268584289
1.8788573025412	2.3207342980442
1.8799619961287	2.3223443730839
1.8803863494248	2.3232915055646
1.8810462889096	2.3249130332309
1.8815469663878	2.325501296763
1.8823122534639	2.3273241917101
1.8830616328197	2.327447497278
1.8836278443236	2.3277738711142
1.8842546498119	2.329662743942
1.8848040009702	2.3306584157873
1.8852404413014	2.3320869421836
1.8858579551269	2.3336914053348

1.8864083614314	2.3337024427721
1.8873431004976	2.3346886801718
1.8880143645145	2.3355593752107
1.888678687246	2.3361147228883
1.8892516329627	2.33670492261
1.8897720297501	2.3369389858961
1.8902733815751	2.3371451918339
1.8909286130175	2.3388801399375
1.8917706435355	2.3404573923983
1.8924619639325	2.3416099404536
1.8930791524299	2.3416724965001
1.8936693030787	2.3417290958105
1.8943560439469	2.342064094036
1.8951272043313	2.3429544269573
1.8959145368463	2.3451596753823
1.8966971083974	2.345170197002
1.8973157987894	2.3476690564519
1.8979224228561	2.3490456333814
1.8985422465419	2.3498453136981
1.899182071092	2.3517093399594
1.899779180039	2.3526408581696
1.9004507437698	2.353342250684
1.9010792571816	2.3550867342334
1.9018197830469	2.3576647972723
1.9022245890692	2.3581093678937
1.9028109443749	2.3584276906694
1.9033219658313	2.359715516793
1.9039634069044	2.3619482214344
1.9046372941772	2.3638611063633
1.9051703281174	2.3641295547668
1.9057358455494	2.3645748258083
1.9064613581299	2.3647445566385
1.9071842098078	2.3667659794213
1.9077523891258	2.3673050362302
1.9082189347984	2.3674686944092
1.9090804709931	2.3678988950944
1.9097669274666	2.3685382411986
1.9103407886452	2.3694310994139
1.9108478779687	2.3696397987761
1.9115340887573	2.3699096879453
1.9120387130869	2.3700233716084
1.9127187043197	2.370475576771
1.9134208015099	2.3713925993891
1.9140615555247	2.3719032558929
1.9148644057716	2.3757473472333
1.9155677593591	2.3762758443693
1.9161183673503	2.3775592076187
1.9165885507046	2.3794022676503
1.9172174077894	2.3803461235364
1.9179697153476	2.3827116364773
1.9184926547508	2.3838154896286
1.9190891456324	2.3839799067503
1.9197808871908	2.3853787993954
1.9205422646102	2.3863326993674

1.9211193953449	2.3864004056286
1.9217412242559	2.3865143470831
1.9222997418387	2.3872618918261
1.9230153074789	2.3888583909501
1.9236641519559	2.3889538416442
1.9243320942083	2.3889822871767
1.9248848449962	2.3894643755737
1.9256063263704	2.3909556006208
1.9261652005885	2.3912864838564
1.9271768748462	2.3931443996763
1.9275790751386	2.3932148285422
1.9284470578094	2.3964139732411
1.9289722052289	2.3972259680262
1.9297056996432	2.3975491707406
1.9303697078294	2.3986689761468
1.9309694520874	2.399365077629
1.9318871287551	2.3999373772807
1.9324684073658	2.4005795941352
1.933128140494	2.4016588597037
1.9339480903099	2.4032923911147
1.9347719854998	2.4050546364199
1.9353892336529	2.4062846054877
1.9360218849679	2.40660628279
1.9366906293564	2.4086444210423
1.937294897003	2.4098252146418
1.9378367273077	2.4100828703831
1.9384270191535	2.4106620817434
1.9391529095096	2.4121125924513
1.9397640796102	2.4123029360961
1.9403954690423	2.4141774509341
1.941172051663	2.4155859582539
1.9416673777051	2.4156036216227
1.9425749820352	2.4165287376262
1.9431996910034	2.4167037971279
1.9440205714744	2.4183832958199
1.944904341787	2.4187399064531
1.9458313538144	2.4220537309549
1.9466000848703	2.422699869565
1.9472779824485	2.4234487821643
1.9477347815748	2.4235510721731
1.9486697430927	2.4241371341142
1.9492330123023	2.4249813767841
1.9498915502278	2.4253136171826
1.9504616756632	2.4260679777019
1.951228026811	2.426691311178
1.9518409454318	2.4280575747495
1.9524721324639	2.4282221073991
1.9530495029381	2.4296590367586
1.9539708525902	2.4326387703918
1.9546555906555	2.4332178466885
1.9553966471443	2.433323517368
1.9561798552283	2.4364129065047
1.956989348767	2.4373778673402
1.9576542826274	2.4374708544746

1.9585642743985	2.4383923481626
1.959478101944	2.4391015755866
1.9601617712408	2.4404472500167
1.9611976008653	2.4405142672635
1.9621101496678	2.4415573770189
1.9625597962293	2.4416255948309
1.9632922372604	2.4430216145259
1.9639171741828	2.4432883125535
1.964665448193	2.444117832646
1.9657605218694	2.4473597094714
1.9666956565141	2.4488636597141
1.9674825821387	2.4494791172056
1.9682684750905	2.449527757692
1.9688368915696	2.4518432265472
1.9694129785077	2.4522084135807
1.9700176049112	2.4535926114855
1.9706537373814	2.4538184938781
1.9714220362745	2.4543976210916
1.9721166827134	2.4558504052658
1.972701157063	2.4567887872713
1.9736188179386	2.4588636267196
1.9746106869347	2.4602194280923
1.975260642058	2.4609272535863
1.9760288722875	2.4615722415352
1.9767762406929	2.4621549036117
1.9772738300747	2.4624442438344
1.9780000415051	2.463000674898
1.9785444880238	2.4641160730267
1.9792560140383	2.464572244521
1.9797640317136	2.4650000036537
1.9804958522833	2.4650170585437
1.9813460694597	2.4661845914181
1.9819710694662	2.4663866193803
1.9829913328442	2.4675053693916
1.983861547085	2.4712219556494
1.984544705915	2.4722480858785
1.985213286128	2.4726749475955
1.9859006383354	2.4728358705751
1.9866176548554	2.472866154575
1.9874804041288	2.4748580987849
1.9882887067678	2.4794018757593
1.9892185843475	2.4806445277555
1.9901020756963	2.4815770274282
1.9909200241749	2.4834755122573
1.9915486530201	2.4835731993598
1.9922432865943	2.4836917220831
1.9928010425235	2.4846738224836
1.9936778930911	2.485959204208
1.9946620127422	2.4868237238509
1.9955751388044	2.489200024109
1.9965167538801	2.4897090223985
1.9975828041376	2.4905850392814
1.9986507567076	2.4930194786826
1.9996596443133	2.4936385816591

2.0005407028536	2.4956064740452
2.0012585863415	2.4956549308986
2.001731726768	2.4960438883308
2.0025604077316	2.4968737036734
2.0034041655183	2.4982434811083
2.0041089984	2.4995120036863
2.004955126349	2.5010394696128
2.0059079274865	2.5011420412037
2.0067621008408	2.501199646672
2.0079524653972	2.5012776803424
2.0087180916162	2.5020372289567
2.0099624697643	2.5022404455097
2.0108112022639	2.5036355646492
2.0115678644453	2.5037395863171
2.0124231933007	2.5049584791331
2.013280261868	2.5065320069584
2.0139661247393	2.5066112142532
2.0150477964023	2.5075050749439
2.0155630474127	2.5080727438802
2.0161897013504	2.5093568139047
2.0167363182923	2.5097518467961
2.0175571116868	2.5109028479654
2.0182716488366	2.5112797887976
2.0190494656537	2.5122783951991
2.0197540546579	2.5122885962181
2.0206696161924	2.5137640263593
2.0215985549973	2.5151315915145
2.022427528066	2.5169038237185
2.0231820263229	2.5182833589043
2.0239548613378	2.5187384711367
2.0243825140643	2.5187449075029
2.0250060306585	2.5204731384642
2.0256363923866	2.521072245953
2.0264055740534	2.5243283558284
2.0273304451071	2.5273299928436
2.0279598415004	2.5280571449172
2.0287916829937	2.5307001875873
2.0297379195986	2.5312991171729
2.0306402005941	2.53144210341
2.0315035208594	2.5317842213866
2.0326472616309	2.5332066266655
2.033571449788	2.5334986962253
2.0343355927636	2.5356297692821
2.0354531321721	2.5361045858145
2.0364617679402	2.5362059389115
2.0376487667133	2.5374587176695
2.0384911252958	2.5390581403687
2.0391487553745	2.5400461155987
2.0400161255919	2.5413892456527
2.0407993865611	2.5418896308185
2.0418680428926	2.5421507221397
2.0430835330095	2.5478862922289
2.0440048608084	2.5491641016273
2.0452618974115	2.5545663150815

2.045963928164	2.5551582349478
2.0470439848707	2.555640435903
2.0479767662699	2.5563807067475
2.0488353185817	2.5592860569655
2.0496537016573	2.5603752388131
2.0506025230328	2.560976198592
2.0511738784298	2.5614163959766
2.052116220277	2.5618738884354
2.052916778683	2.5646795536133
2.0535795751593	2.565132491623
2.0544322047819	2.5651457329461
2.0555523514442	2.5658463023261
2.05671539768	2.5669639630598
2.0573419385239	2.5682264980433
2.0582640272452	2.5696346050513
2.0588705793951	2.5722886318925
2.0597459945699	2.5732480059489
2.0606698691948	2.5764315134128
2.0622374071105	2.581768962212
2.0633936964737	2.5818728365469
2.0641182461571	2.5823395866584
2.0653401899389	2.5832255935087
2.0661809182493	2.5837785449645
2.0670720226816	2.5856458161885
2.0680651514609	2.5859459196954
2.0688933754453	2.5861627051175
2.0697818798118	2.5866644713582
2.0709709992827	2.5887606340886
2.0718007142778	2.5901287794733
2.0728871739232	2.5904487390853
2.0737033361077	2.5906468630035
2.0745905287654	2.5930222481892
2.0756045866354	2.594591917298
2.0763514988416	2.5951658865427
2.0771888864257	2.5952278666737
2.0780877865118	2.5961836127545
2.0788447854168	2.5967577236081
2.07974487539	2.5998941473534
2.0805588745977	2.6000014298444
2.0814634054436	2.6005435875873
2.0822498317308	2.6008726064207
2.0830993446468	2.6012410162212
2.0840014263243	2.6021023824212
2.0852918413155	2.6062897585678
2.0865988615434	2.6094894415141
2.0879582757672	2.6106075894861
2.0889699851432	2.6121096853317
2.0901787524873	2.6122874178344
2.0909639173849	2.6149346437396
2.0918556025803	2.6154079417123
2.092609215836	2.6155083345725
2.0933341447321	2.6168909216826
2.0940718456527	2.6174907817382
2.0948439887052	2.6179396900725

2.0957389346814	2.6190289652652
2.0965382634016	2.6193498411826
2.0974775493082	2.6206806913071
2.0984316217357	2.6221757704373
2.0992991784621	2.6228019279888
2.100321612105	2.6234929453387
2.1011026973936	2.6237936038702
2.1019009260061	2.6240997028174
2.102605815709	2.6242510176682
2.1038995860784	2.6246523159228
2.1048232024284	2.6280404384171
2.1057392194157	2.6282846228991
2.1067351204453	2.630208795727
2.1077232612162	2.6314486170607
2.1088394579503	2.6340390769179
2.1095603282151	2.6351034534022
2.1105086786727	2.6355474693484
2.1114619970757	2.636440809849
2.1124333332053	2.6370677099378
2.113522801792	2.6399859796782
2.1145853340922	2.64027952108
2.1153358790361	2.641402413921
2.1165934841306	2.6419293302527
2.117432585562	2.6420719664035
2.1185800567179	2.6470620648658
2.1201174054586	2.6535366616455
2.1212016199931	2.6566929315071
2.1223131755702	2.6569979003685
2.1231788348063	2.6593196414424
2.1243159910416	2.6601652788022
2.1251480346632	2.6603409193814
2.1265521630921	2.6648676941535
2.1281587593582	2.6711332367807
2.1292045734065	2.6714937271035
2.1302407287328	2.6780292273173
2.1312858914228	2.6786181950136
2.1324126532528	2.6793694178361
2.1334988655287	2.681193853015
2.1340569618586	2.6813663778853
2.1351423615217	2.6824687580277
2.1358957824696	2.6830995484881
2.1366484948073	2.6841491129976
2.1378514718444	2.6841648631562
2.1386586826237	2.6851678327857
2.1395291016127	2.6851885875079
2.1405935782835	2.6855580249021
2.1416069458085	2.6870313367365
2.1431050782689	2.6879438877026
2.1442318530571	2.6914066977722
2.1448796375709	2.6914680978939
2.1457865561061	2.6926679674251
2.1466034845014	2.6934166405638
2.1476020004956	2.6945832900947
2.1484679802849	2.6957805796736

2.1493628947308	2.6977722398792
2.1502213567336	2.6991169008085
2.1513006623925	2.7007639508004
2.1522067202219	2.7014581077332
2.1533683121264	2.702231962749
2.1547602296034	2.7038752877024
2.1555607418134	2.7049231412277
2.156723855933	2.7074582422492
2.1577261343782	2.7088098215511
2.1589059069657	2.7102216796262
2.1598526652623	2.7105505149658
2.1609115832861	2.7107284433862
2.162064540859	2.7120876623745
2.1635580366531	2.7127545222814
2.1646585898231	2.7158123318102
2.1658071666571	2.7178458598692
2.1671673585693	2.7202507921173
2.1683021506231	2.7233262970672
2.1691696564746	2.7237096353102
2.1700856275459	2.7242457100714
2.1711383466062	2.7272537817085
2.1722122919618	2.7299576441539
2.1734869930729	2.7307303252436
2.1746287260248	2.7313305225086
2.1760754532323	2.7315871576879
2.1774296388867	2.7342715157261
2.1785139962281	2.7346379638159
2.1792922141751	2.73502557795
2.1806419188966	2.7368800253385
2.1815572266902	2.7394947916436
2.1826296150538	2.7417870742583
2.1840543386738	2.7465605960685
2.1856818909334	2.7492680245936
2.1868784167401	2.7509481722549
2.187930045161	2.7533817014314
2.1896618700479	2.7565819860886
2.190482465518	2.7569000227855
2.1915214693521	2.757725717636
2.1926158152851	2.7589493721055
2.1935294482432	2.7602314929299
2.1945646230982	2.7603597829171
2.1956353747525	2.7642756431615
2.1967570269199	2.7644317117141
2.1980929845386	2.7656128406518
2.1988912358733	2.7658654527899
2.2003711924635	2.7668493461865
2.2014824870763	2.7674019794265
2.2026190941368	2.7705814922213
2.2041738320978	2.7740153724238
2.2052230563847	2.7758656784635
2.2065278009197	2.779685586049
2.2074556542307	2.7803610942748
2.2089303677497	2.7843275904679
2.2103447591367	2.794462963632

2.2111580961539	2.7950858449995
2.2124922996243	2.7956981119587
2.2137208086735	2.7980918809041
2.2150300325312	2.8011033973511
2.2164426236707	2.8029709175003
2.2179307295792	2.8053190909461
2.2194627209918	2.8085751700923
2.2202800697529	2.8101604923118
2.2212500474582	2.8113614180432
2.2226786037568	2.8126920899416
2.2238696503517	2.8156961791292
2.2252507000504	2.8178642065516
2.2266952833719	2.8191380660785
2.2278759173887	2.8197633867855
2.2291498421307	2.8202070752175
2.2304760759499	2.8257741434262
2.2315678823973	2.8258522821131
2.2328329236559	2.8281399930746
2.2338188420863	2.8300029167843
2.2351317071402	2.8331940098201
2.2359356928582	2.8346985787301
2.2375584723679	2.8377443177177
2.2388147780005	2.8381820711607
2.2397905086027	2.838712035233
2.2416104170879	2.8440403139384
2.2429935770237	2.8456312908897
2.2440099857306	2.8459527730565
2.2449182517411	2.8465767963422
2.2466555760729	2.8487554050201
2.2480050699398	2.852148551798
2.2488714530821	2.852620338201
2.2499371975178	2.8532452142453
2.2509419785593	2.8538466370181
2.2523826531459	2.8585387758659
2.2538795386918	2.8596644915252
2.2554924947145	2.8634353880255
2.2568699800026	2.8688609151128
2.2583315435918	2.8707679229687
2.2594185384875	2.8710569902213
2.2604671215235	2.8734437550437
2.2616841013596	2.875789680223
2.2628146583226	2.8763383416659
2.2645858245829	2.8765382737518
2.2658341476162	2.8782259535501
2.2669281697579	2.8790976262478
2.2683049038719	2.8803161220619
2.2694718415652	2.8821480798107
2.2703757797481	2.8821881275854
2.2717727997483	2.886330463398
2.2727833552117	2.8882151300773
2.2741000923021	2.8892123202115
2.2755350896903	2.892480325509
2.2767397210584	2.8930055887337
2.2780446866924	2.893456270681

2.2791930193465	2.8968043911621
2.2808374021675	2.8989662426865
2.2821557176419	2.9024908012741
2.2833544347111	2.9035597446582
2.2845668425039	2.9046859591896
2.2855642944568	2.9064817895654
2.2871218133608	2.9081543329648
2.288567698995	2.9088936868806
2.2897020256642	2.9097495878348
2.2909131928012	2.9125878818083
2.2919427370256	2.9129560826011
2.2937838250044	2.9156753969286
2.2951914559789	2.9189839955432
2.2967090143628	2.9230385627987
2.2978295490128	2.9240237354602
2.298928955912	2.9242105498396
2.300315664167	2.9244881702181
2.3017299649487	2.9257390126073
2.3034260208217	2.9346016889026
2.3044557473049	2.9352888268407
2.305924948377	2.9373631569361
2.3078822742582	2.9398964087414
2.3093905756418	2.9402029309575
2.3112317447157	2.944057803674
2.3127993496834	2.9500087409153
2.3149683362878	2.9503189062132
2.3164832845109	2.9517940449196
2.3186491063232	2.951853192682
2.3203821958931	2.9553719239362
2.3214950844085	2.9570202443219
2.3231755573932	2.9594608990783
2.3248313543151	2.961051190246
2.3264722846892	2.9623280891179
2.3276436652478	2.9633077672445
2.3294440310565	2.9639828663308
2.3308640593781	2.9653574333566
2.3321863849649	2.9658083459319
2.3333531089132	2.9706261164295
2.3350076176121	2.9781789792986
2.3364758598703	2.9781943707785
2.3379514457999	2.981446006772
2.3391954447826	2.9817740303776
2.3414926100757	2.9893511895124
2.3428704673131	2.9916630761499
2.3443146492826	2.9932123801057
2.3461128608331	2.9939506690423
2.3476244841186	2.99447601722
2.3489169098137	2.9959242250512
2.3501786222163	2.9980975908426
2.3512724857102	2.9998874012603
2.3527054584855	3.0019232688802
2.3548464754443	3.0055740481809
2.3561756081324	3.0060935490253
2.35765778672	3.0061230263625

2.3591859007927	3.0061603988181
2.3609611392747	3.0157773993121
2.3624470891781	3.0164556070062
2.3642745231795	3.017087573745
2.3658989293281	3.0186863266649
2.3676925538538	3.0206656520508
2.3694666707049	3.0208346404956
2.3708804414565	3.0212562758971
2.3723876739994	3.0220242273081
2.3736639517274	3.0238570703174
2.3750951404052	3.0264440288934
2.3768199713216	3.0276166305376
2.3792426071044	3.0376480708587
2.3805697970047	3.0377748814701
2.3826251349049	3.0396992629488
2.38434111274	3.0426336989666
2.3854753429958	3.0464373023341
2.3874199113645	3.0482287137705
2.3889617470682	3.0484922959225
2.3906115869552	3.0500609705721
2.3923319105749	3.0520352325958
2.3941425550182	3.0531673182969
2.3963481170629	3.0597837435504
2.3980812435332	3.0628797291684
2.3992519961459	3.0659061983982
2.4008167912288	3.0695284157178
2.4024654856907	3.0701867302053
2.4038233857361	3.0721063868296
2.4059719650455	3.0751644664179
2.4076784025621	3.0776749091576
2.4099089503399	3.0784318509919
2.412067465226	3.0813909600946
2.4138777361215	3.0850621210842
2.415501003269	3.0852542460942
2.4173661080525	3.0862584625454
2.4200616863291	3.0866344304587
2.4215588544263	3.0908554877603
2.4234108320555	3.091950220162
2.4257619445839	3.0954596618244
2.4275169045471	3.0957757294649
2.429446811242	3.1024155186731
2.4319713436107	3.1061192701604
2.4335822776402	3.1067932011285
2.4349461235448	3.1080671945411
2.4365539878124	3.1097698767475
2.4385636194587	3.1111973684878
2.4399914271929	3.1115686189273
2.441666030839	3.1147474841066
2.442959563727	3.1158096810824
2.4450317081988	3.1202590392591
2.4472599910039	3.1271095845076
2.4489593273379	3.1271162318376
2.4507129624699	3.1289819315022
2.4518074757639	3.1299625963349

2.4537668144189	3.1395092809479
2.4560001392674	3.1422760044476
2.458031706019	3.1470527341628
2.4598075983607	3.1505178709752
2.4613366583731	3.153436787086
2.4640506004294	3.1700373379238
2.4658136288789	3.1749403719333
2.46838446907	3.1750912453108
2.4717829994925	3.1782064326107
2.4743347854445	3.1819890135852
2.476951512117	3.189626344693
2.4793490911763	3.1913825875321
2.480682082974	3.193133202694
2.4826480849211	3.1931844712521
2.4845762888362	3.1985855004941
2.4865819347395	3.2006413372186
2.4881282496117	3.2011927260307
2.4898290446639	3.2021988063014
2.4915852121738	3.2045573503858
2.4935247532991	3.2064349411407
2.4954630475354	3.2077659308708
2.4981089050374	3.2079724243538
2.500216187215	3.2114710402055
2.5020661942932	3.2152896397375
2.5040412949175	3.2164717194372
2.5059833326843	3.2179164390904
2.5073254113232	3.2183045372588
2.5101677348339	3.2268716106213
2.5119445129236	3.2293230330278
2.5148768189268	3.2344648250803
2.5171262898101	3.2351849326387
2.5203302478629	3.2395523874192
2.5227651556195	3.2419174597666
2.5253336710676	3.2519752888139
2.5273310419801	3.2536117289529
2.5301796369984	3.2651128785832
2.5319497024145	3.2659837781542
2.5337718556426	3.271986774934
2.5360601249846	3.2743977566965
2.5381382990028	3.2809152021748
2.5413098402222	3.2882996420549
2.5429613783284	3.2886308139638
2.5458744508668	3.2923300950559
2.5492528346521	3.3023784136836
2.5520915181513	3.3111900349062
2.5544920806403	3.3158569249752
2.5565378322619	3.3159774793673
2.5586125045059	3.3183859426853
2.56105075959	3.3207844534603
2.5645346050129	3.3392635357475
2.5669125595246	3.3417835332319
2.5693291476929	3.3473246070368
2.5735142725937	3.3642035141695
2.5756539630218	3.3644645747192

2.5788662364329	3.3733288153462
2.5817696292109	3.3772556872575
2.5844775436739	3.3804790278834
2.5877058951532	3.3834212252111
2.5906117987548	3.3849441513512
2.5929088586109	3.3881994526465
2.5953752397356	3.3885899205457
2.5974343350902	3.392049105833
2.6005390523493	3.3949626094574
2.6040799100511	3.4032504522624
2.6067992852868	3.41178206129
2.6088256425268	3.4128390239391
2.611488225468	3.4138406935002
2.613293081125	3.4153854664826
2.615544875236	3.4159266880727
2.618576868391	3.4321617286251
2.6217686232844	3.4326672072367
2.6244464145371	3.4338493359546
2.6268996984692	3.4400715169817
2.6300487481591	3.4414359500188
2.6323644056936	3.4485279246263
2.6358627702549	3.4644559279762
2.6400141523924	3.4840079432873
2.6421778449721	3.4875986460609
2.6449400176849	3.4922833770216
2.6472474464503	3.4926711894962
2.6499660174985	3.4971554600948
2.6530238061721	3.5048002197502
2.6564874785148	3.5110392956089
2.6589823184834	3.5147018636327
2.6614250895478	3.5147669019026
2.6641231972333	3.5155284130104
2.6679333874994	3.5183979497213
2.6701816909252	3.518587377256
2.673075386507	3.5186320622914
2.6766046242641	3.5219654991469
2.6792452419955	3.5227622590084
2.6817486645049	3.5277784416385
2.6861571909218	3.5596906878353
2.689690518787	3.5660912166782
2.6922288546633	3.5669689577606
2.6951960166646	3.5683604354186
2.6978625872715	3.5854294004974
2.700908810318	3.5954336027104
2.7034910266674	3.5961109744457
2.7057653022088	3.5971302860805
2.7093830265843	3.5987024283461
2.7127504158442	3.6069476161964
2.7161787804999	3.6142982131611
2.7188861966019	3.6148358670856
2.7217234191033	3.618862591899
2.7250992923542	3.6258381503087
2.7286382289422	3.6386701422001
2.7319957978578	3.642963385447

2.7354966481316	3.6583322218477
2.7380079397662	3.6591095959581
2.7415056698072	3.6686978950073
2.7449955934273	3.6729437182584
2.7478681842818	3.6746082391219
2.7519287388456	3.6830869080118
2.7548109363691	3.6840987448457
2.7576937781219	3.6924912609302
2.7612938364473	3.6964029133904
2.7651791581733	3.70062587701
2.7678943441163	3.7008722472434
2.7720106750947	3.701872462313
2.7755610435342	3.7024962006911
2.7796892916343	3.7069733358662
2.7836705797488	3.7141769810225
2.7874045827173	3.7148977686958
2.7910891385768	3.7278224650082
2.7945865208519	3.7382327348327
2.7987920375439	3.7495493409489
2.802761529743	3.7544904530484
2.8072285746271	3.7618935156563
2.8111239398237	3.7625046034846
2.817715751233	3.7918134295995
2.8220454716205	3.7922968757841
2.8260847644225	3.7992410661674
2.8324824223941	3.8050878117844
2.8375720410188	3.8163666144881
2.8409543695927	3.822866187676
2.8450507915197	3.8230094241006
2.8486545694666	3.8368114370508
2.8521191257062	3.8394473768374
2.8578664043134	3.8590053812524
2.8617938402762	3.8648556700744
2.8655317656896	3.8825695959766
2.8705348150781	3.8964027305531
2.8757635420586	3.8975639243311
2.8807381544387	3.8990692610258
2.8857165507858	3.9062815377074
2.8901849113423	3.9072513022225
2.8942009571395	3.9103764159506
2.8999434077286	3.912977577851
2.9029971679128	3.9155130471012
2.9073888076443	3.9225818898391
2.9118057948155	3.9258995568613
2.916293374115	3.9352573871554
2.9236030700736	3.9545235776815
2.9286273506005	3.9562101228682
2.93380771992	3.9655308321937
2.9389267115729	3.9673741839315
2.9428769326521	3.9808913663054
2.9473803939383	3.9855658006802
2.9525574087615	4.0025732268063
2.9581204315548	4.0030540042757
2.9671570961764	4.0067206104484

2.9712897957958	4.0215127753714
2.9777710910289	4.0329175897574
2.9817384572803	4.0358079113403
2.9865347284078	4.0448240199965
2.9933102075159	4.0634829760891
2.9970900273121	4.0646724395698
3.0016969506956	4.0672575046509
3.0077626603384	4.071855953165
3.014822189094	4.0853571967656
3.0202885127696	4.0889799848505
3.02561868397	4.1017564674188
3.0332613577068	4.1058800481958
3.0386254024335	4.1086844411361
3.0491244762213	4.1311499389874
3.0550957464513	4.1568570090098
3.0596413808488	4.1597172175172
3.0653913863637	4.1712529128573
3.0712707784381	4.1851736309338
3.0774453529229	4.1875422472201
3.0852117745473	4.1902944745793
3.0901862843351	4.1930635037285
3.0994131204038	4.2217790186933
3.104899424315	4.2256281200708
3.1097850481127	4.2304226172763
3.1158809856374	4.2334003200351
3.1248628528753	4.2610954923405
3.1333112788991	4.2751628029098
3.1422690613468	4.2774267274695
3.1496392534391	4.2782631460187
3.1570782399023	4.2788875344285
3.1637966986876	4.2810089568884
3.1702469707125	4.3025746951172
3.1798688810221	4.3372607297797
3.1878107841214	4.3400316984188
3.1955577879827	4.345484307211
3.2036475029131	4.3724023214766
3.2134672175068	4.3833456547724
3.2199349299739	4.3862728949094
3.2321191419931	4.4021005743015
3.2414725180586	4.4047956478528
3.251172051679	4.4257155190848
3.2595617268579	4.4530942355377
3.2732599196671	4.4533220297268
3.2828331889767	4.4618124671492
3.2906705362737	4.4698596363921
3.3002605175021	4.4780672768697
3.3154194343369	4.4861516290159
3.32682784207	4.4996206890082
3.3378639176178	4.5447282454071
3.3492629588725	4.5704374506297
3.3653632656521	4.5887803265135
3.3771456068208	4.589367397308
3.3859727102538	4.6093993150274
3.3986790067399	4.6246434210889

3.4123647207673	4.6532712570723
3.4258943379315	4.7042041743655
3.439770677265	4.7663908277393
3.4513352011381	4.7740063961262
3.467464464007	4.8586835422163
3.4818000727262	4.8671509983422
3.4937907816237	4.8742135968858
3.510134122658	4.8868163167522
3.5269286962789	4.9352036903424
3.5373171310112	4.941710397781
3.5517512097937	4.9831663483244
3.564383293215	4.9842455254048
3.5807172921985	5.0111963394744
3.6030248677903	5.0295039556553
3.6199175417252	5.0896947089337
3.638791811106	5.1012896308268
3.6551516686288	5.120931856528
3.671900313493	5.1455576366215
3.6907606398511	5.1872108599212
3.7138945428206	5.2271046377767
3.7478761926972	5.2944952980874
3.7695722739471	5.3149775164112
3.791299645747	5.3215790258197
3.8207808082085	5.3457624307662
3.8399101274759	5.3999970666799
3.8682162449477	5.4979018118935
3.8874860197989	5.5221557002384
3.9106925180889	5.5346703071327
3.936213006012	5.5507038905598
3.9630218884979	5.5547191184969
3.988008199607	5.618292509152
4.0205650856143	5.6489643296406
4.0547974222411	5.7614267398701
4.1117043913481	5.8049035126514
4.1503070593919	5.8248953396951
4.2010140712542	5.9168792844749
4.2326381552317	5.9222569958652
4.2948043099862	5.9494153510988
4.341084622786	5.9927024190744
4.3946736675822	5.9968704742949
4.4631149629619	6.0504706943288
4.5455424080099	6.126164437204
4.6324080203177	6.1493358659265
4.7399538729956	6.2322628786161
4.8517162541974	6.2876686641129
4.9690404122631	6.4540562551545
5.0706054340835	6.6049041346878
5.2565160526521	6.628892111355
5.5028815361964	7.0514238112646
5.7791129153431	7.4212558670415
6.3053528555025	8.3783790062219
7.6154205238114	10.239791950658
